# Nationwide trends and features of human salmonellosis outbreaks in China

**DOI:** 10.1080/22221751.2024.2372364

**Published:** 2024-06-26

**Authors:** Zining Wang, Haiyang Zhou, Yuhao Liu, Chenghu Huang, Jiaqi Chen, Abubakar Siddique, Rui Yin, Chenghao Jia, Yan Li, Guoping Zhao, Min Yue

**Affiliations:** a Key Laboratory of Systems Health Science of Zhejiang Province, School of Life Science, Hangzhou Institute for Advanced Study, University of Chinese Academy of Sciences, Hangzhou, People’s Republic of China; b Hainan Institute of Zhejiang University, Sanya, People’s Republic of China; c Department of Veterinary Medicine, Zhejiang University College of Animal Sciences, Hangzhou, People’s Republic of China; d CAS Key Laboratory of Synthetic Biology, Institute of Plant Physiology and Ecology, Shanghai Institutes for Biological Sciences, Chinese Academy of Sciences, Shanghai, People’s Republic of China; e Department of Microbiology and Microbial Engineering, School of Life Sciences, Fudan University, Shanghai, People’s Republic of China; f State Key Laboratory for Diagnosis and Treatment of Infectious Diseases, National Clinical Research Center for Infectious Diseases, National Medical Center for Infectious Diseases, The First Affiliated Hospital, College of Medicine, Zhejiang University, Hangzhou, People’s Republic of China

**Keywords:** *Salmonella* infection, salmonellosis, outbreak, systematic review, meta-analysis

## Abstract

Salmonellosis is one of the most common causes of diarrhea, affecting 1/10 of the global population. Salmonellosis outbreaks (SO) pose a severe threat to the healthcare systems of developing regions. To elucidate the patterns of SO in China, we conducted a systematic review and meta-analysis encompassing 1,134 reports across 74 years, involving 89,050 patients and 270 deaths. A rising trend of SO reports has been observed since the 1970s, with most outbreaks occurring east of the Hu line, especially in coastal and populated regions. It is estimated to have an overall attack rate of 36.66% (95% CI, 33.88-39.45%), and antimicrobial resistance towards quinolone (49.51%) and beta-lactam (73.76%) remains high. Furthermore, we developed an online website, the Chinese Salmonellosis Outbreak Database (CSOD), for visual presentation and data-sharing purposes. This study indicated that healthcare-associated SO required further attention, and our study served as a foundational step in pursuing outbreak intervention and prediction.

## Introduction

The salmonellosis outbreak (SO), due to chocolate contamination spanning 17 countries across Europe and North America, has evoked continued profound global vigilance [1–3]. Salmonellosis, which typically presents with symptoms of diarrhea, abdominal pain, fever, and vomiting, is a collective term for illnesses infected by bacteria of the genus *Salmonella* [4]. Non-typhoidal salmonelloses are primarily associated with gastroenteritis and extra-intestinal infections, whereas typhoidal salmonellosis not only leads to enteric fever (EF) but also causes symptoms such as rose spots and splenomegaly [5, 6]. Given the differing characteristics of the pathogens, these two types of salmonellosis require disease-specific treatment options [7–13.] As the most common foodborne pathogen, *Salmonella* is prone to outbreaks in various settings, including communities, schools, and hospitals, adversely affecting on public health and economic development [14, 15].

As a bacterial pathogen responsible for causing more than one million disability-adjusted life years (DALY) annually, *Salmonella* is widely distributed throughout the intricate and dynamic food web of the natural environment [16]. *Salmonella* infections contribute to approximately 131 million cases worldwide annually, resulting in at least 370,000 fatalities [17]. Non-typhoidal *Salmonella* (NTS) primarily disseminates through various food processing routes, with a substantial portion of historical reports on *Salmonella* infections originating from food poisoning incidents [18]. On the other hand, pathogens causing typhoid and paratyphoid fevers can initiate widespread outbreaks by contaminating water sources, posing a formidable challenge in terms of disaster control [19].

While outbreaks are promptly monitored and reported in developed regions, such as North America and Western Europe, characterized by high incomes and well-established healthcare systems [20–22], the frequency and severity of SO are notably higher in low – and middle-income developing countries, necessitating comprehensive public health attention. Global Burden of Disease (GBD) studies show that *Salmonella* infections in Sub-Saharan Africa and South Asia are more severe [17]. In China, Salmonellosis accounts for 70–80% of bacterial foodborne diseases, establishing it as the primary cause of gastrointestinal infections [23, 24]. Historically, research on the prevalence of SOs has predominantly concentrated on sporadic cases, with limitations arising from the absence of medical records for self-limiting symptomatic cases [25]. Here, we leverage the comprehensive collection of SO records in a single developing country. Alongside systematic analysis, we calculated epidemiological indicators such as attack rates (used to measure the number of new cases occurring in a specific population within a certain period) in order to facilitate an understanding of the dynamics of the disease’s spread and to assess the effectiveness of disease prevention and control measures. Investigating SO characteristics in China will support epidemic prediction and policy making.

## Methods

### Search strategy and inclusion criteria

According to the Preferred Reporting Items for Systematic Reviews and Meta-Analyses (PRISMA) guidelines [26], we conducted a systematic review and meta-analysis on the SO in China. The study has been registered online with PROSPERO (ID: CRD42023458488). The search terms (“*Salmonella*” OR “Typhi”) AND “China” were employed for literature screening in six databases, including Web of Science (WOS, https://www.webofscience.com), PubMed (https://pubmed.ncbi.nlm.nih.gov), China National Knowledge Infrastructure (CNKI, https://www.cnki.net), Wanfang Data (WFD, https://www.wanfangdata.com.cn), Airiti Library (https://www.airitilibrary.com), and China Science and Technology Journal Database (VIP, https://qikan.cqvip.com). All pertinent literature published in Chinese or English up to March 31, 2023, was included in the initial screening process through Rayyan and manual proofreading (**Table S1**). A two-step screening process was employed to acquire the final set of studies included in the analysis [27, 28].

### Data extraction and analysis

In addition to publication details, four categories of data were comprehensively extracted from the full texts (**Table S2**): (1) Temporal and spatial information, including the onset year and month, duration, provinces, prefectures, and settings of the outbreaks; (2) Patient information, encompassing the number of new cases (NC) and people exposed (PE, the group of individuals who have been in contact with the source of the infection during the period of risk and are therefore at potential risk of developing the disease), Age range and average, as well as the count of male (M) and female (F) patients; (3) Medical diagnostics, covering food sources, serogroups, serovars, sequence types, profiles of antimicrobial resistance, and identification methods; (4) Clinical management, detailing antimicrobial treatment regimens and prognosis. Additionally, the relevant extracted data can be used to calculate hysteresis bias (HB, time lag between publication year and outbreak year), attack rates (AR, the proportion of new cases to the exposed population), gender ratios (GR, the proportion of male patients among the new cases), and incidence rates (IR) of designated symptoms such as diarrhea, fever, stomach cramps, nausea, vomiting, and headache. We assessed the quality and risk of bias in outbreak studies included in the systematic review using the Epidemiological Data Checklist adapted from the Joanna Briggs Institute (with appropriate modifications) (**Table S3**). Each study was scored based on the checklist, and only those achieving a high-quality score with a low risk of bias (total score = 10) were included in the meta-analysis [29].

Relevant information from eligible literature meeting the inclusion criteria was extracted and summarized in a unified table on an event-based level. Furthermore, additional public data has been collected for correlation analysis (**Table S4**). To further investigate the epidemiological patterns and prevention priorities of the SO in China, the included outbreaks were divided into multiple research matrices within the systematic review based on differences in data integrity. One-way analysis of variance, Pearson correlation analysis, sixth-order polynomial fitting, and Max-Min standardization were employed for the systematic review. Meta-analysis was performed using a binary random-effects model (SJ: Sidik-Jonkman) with a 95% confidence level on Open Meta-Analyst [30]. Microsoft Office LTSC 2021 was used for result compilation, while the Standard Map Services System, GraphPad Prism 9, Origin 2022, ChiPlot, Diagrams.net, and Adobe Illustrator 2023 were utilized for visualization and graphical projection.

## Results

### Data integration and summary

**Figure 1.**
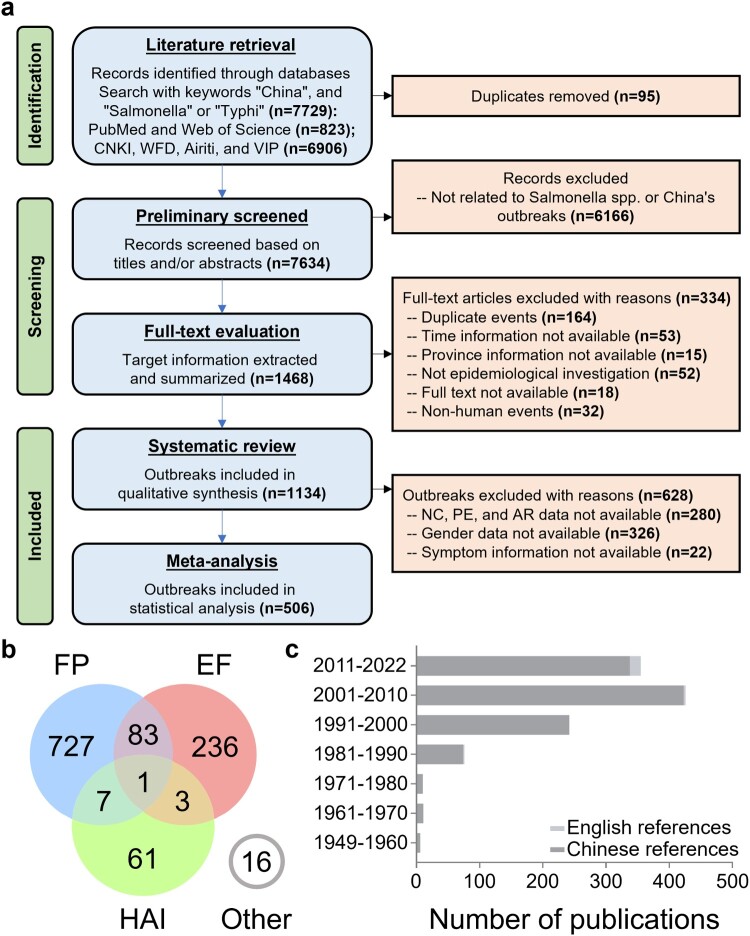
**Analysis process and dataset characteristics of a systematic review and meta-analysis of *Salmonella* outbreaks in China, 1949–2022**. **a.** Preferred Reporting Items for Systematic Reviews and Meta-Analyses (PRISMA) flow diagram of search strategy and selection of articles. **b.** The 1134 outbreaks included in the systematic review were clustered according to three main features: food poisoning (FP), enteric fever (EF), and healthcare-associated infection (HAI). **c.** The number of Chinese and English publications included in the systematic review by year period.

Through a rapid review of titles and abstracts conducted on the initial 7,634 articles included in the preliminary screening, only original reports explicitly related to SO within China were incorporated in the second step of screening, which involved a full-text assessment. Out of the 1,468 articles included in the second-stage screening, 334 articles were excluded during the full-text evaluation for various reasons, including duplicate events, missing temporal or geographical information, non-epidemiological research reports, non-human outbreak events, or the unavailability of the full text. A total of 1,134 outbreak reports, with 89,050 patients, were ultimately included for information extraction and systematic review. Among these, 506 high-quality reports were incorporated into the meta-analysis (Figure 1a). To further elucidate the patterns of SO from the perspectives of transmission routes, pathogenic characteristics, and susceptible populations, we identified three clusters chosen for their abundance in the literature, similarity in approach, and alignment with our aims. Specifically, they comprised 818 outbreaks of food poisoning (FP), 323 outbreaks of EF caused by *Salmonella* Typhi (*S.* Typhi), *Salmonella* Paratyphi A/B/C (*S.* Paratyphi A/B/C), and notably, 72 outbreaks associated with healthcare-associated infections (HAI) reported in healthcare-related settings, including hospitals and nursing homes (Figure 1b). The included literature consisted of 1,112 Chinese publications and 22 English publications, primarily published between 1991 and 2022 (Figure 1c). The collected data and subsequent analysis results were integrated into the Chinese Salmonellosis outbreak Database (CSOD v1.2) at http://139.9.85.208:81/#/index (**Figure S1a**).

### Spatiotemporal trend

**Figure 2.**
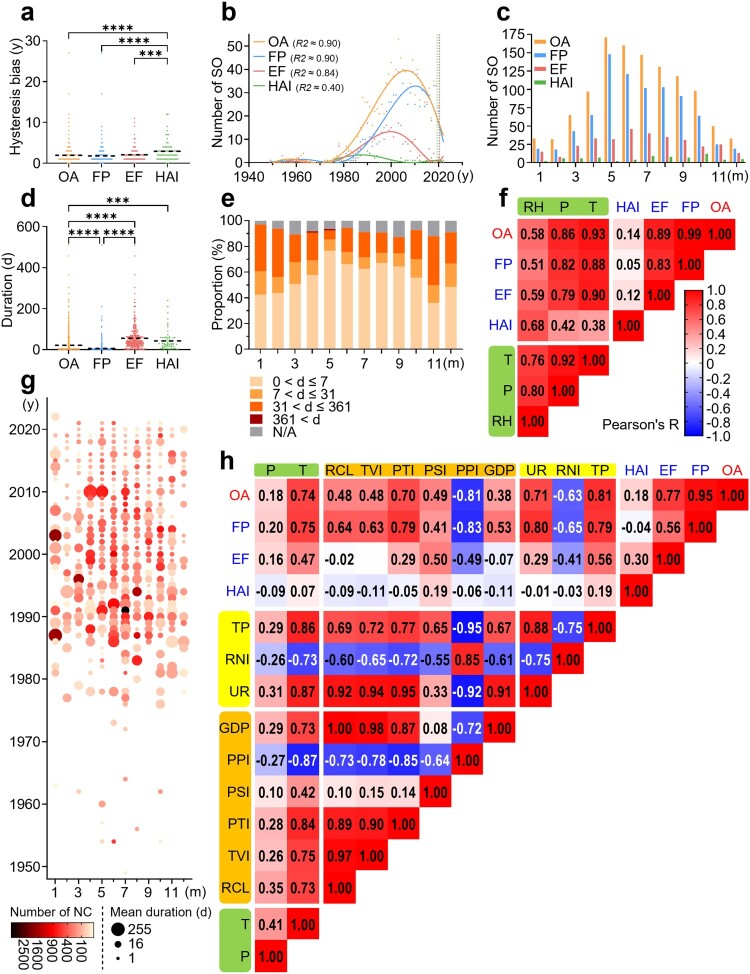
**Temporal variation and correlation analysis of salmonellosis outbreaks**. **a.** Hysteresis bias in the overall or three event clusters of SO reported in China between 1949 and 2022. **b.** Yearly variation in the number of outbreaks (the curves are sixth-order polynomial fit regressions, and the vertical line to the left is the confidence region after correction for hysteresis bias). **c.** Monthly variation in the number of outbreaks. **d.** Duration of outbreaks. **e.** Alterations in the monthly distribution of outbreaks of various durations. **f.** Correlation analysis of the potential factors (climatic in green) affecting the monthly outbreaks. **g.** Two-dimensional visualization of outbreak size in months through the number of new cases (NC) and mean duration. **h.** Correlation analysis of the potential factors (economic in yellow, demographic in orange, and climatic in green) affecting the yearly outbreaks. The abbreviations in f and h are listed in **Table S6**.

The spatiotemporal parameters represent the essential basis for epidemiological investigations of outbreaks. The overall HB was estimated to be 1.9 years, significantly lower than 2.9 years for HAI, lending credibility to the annual number of SO before April 2022 (Figure 2a). The fitted curve suggested a low frequency or under-reported SO prior to 1970, followed by a marked increase in the documentation of such events during the 1970s (Figure 2b). Apart from HAI, mainly observed in indoor environments, summer was identified as the peak season for SO, especially reaching its highest point in May (Figure 2c). The monthly reported SO number exhibited a high correlation with temperature, a significant correlation with precipitation, and a moderate correlation with relative humidity (Figure 2f). The overall average duration of SO was 21.23 days, substantially higher than 6.10 days for FP, but significantly lower than 55.71 days for EF and 43.06 days for HAI (Figure 2d). Interestingly, although summer outbreaks were more frequent, they tended to have shorter durations, while winter outbreaks were more likely to persist for an extended period (Figure 2e), with a higher proportion resulting in large-scale events (Figure 2g). The comprehensive results of the correlation analysis demonstrated a strong association between the annual number of SO and total population, temperature, urbanization rate, and the proportion of tertiary industry (Figure 2h).

**Figure 3.**
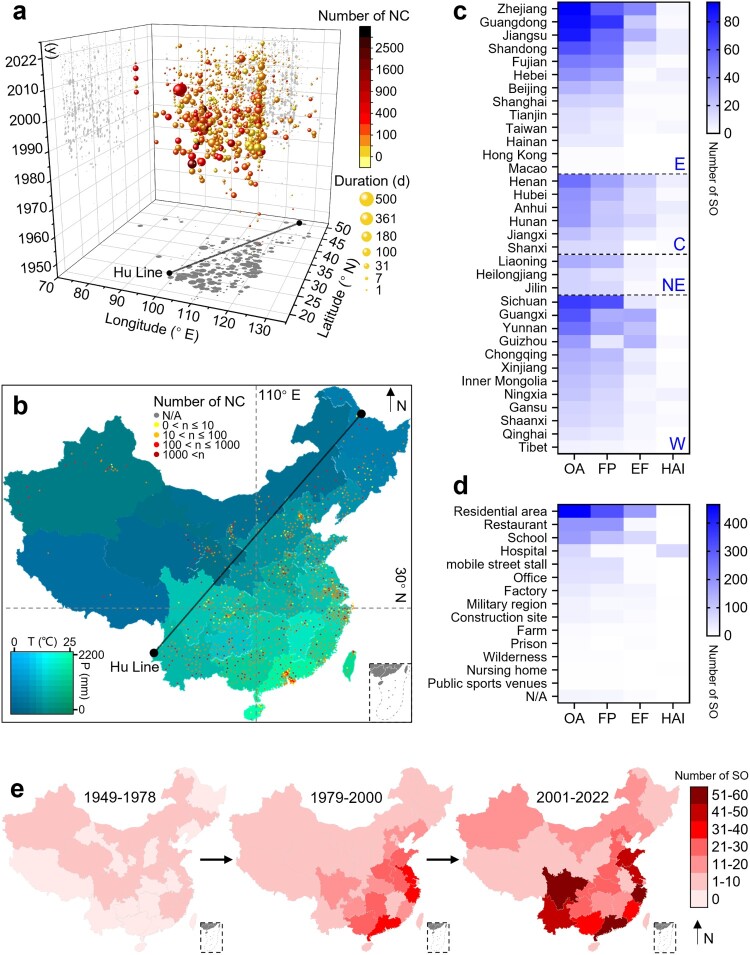
**Spatial distribution of salmonellosis outbreaks**. **a.** Three-dimensional visualization of outbreak size based on individual events. **b.** Geographic distribution of SO in China based on individual events between 1949 and 2022 (the colour changes of the map and the dots correspond to the climate and the number of new cases, respectively). **c.** The number of outbreaks in each province, ranked from highest to lowest within the same economic region. **d.** The number of outbreaks in different settings. **e.** Trends in the geographic distribution of outbreaks in three periods of similar length.

A three-dimensional bubble chart shows that the distribution of SO is mainly concentrated in the eastern region of the Hu Line, which divides the area of China into two parts with contrasting population densities, with large-scale outbreaks primarily reported in Guangxi, Yunnan and Guizhou (Figure 3a). The frequency of SO in densely populated areas, such as the Yangtze and Pearl River Deltas, is relatively high (Figure 3b). Although climate change maps suggest that the geographic distribution of SO may be related to temperature and humidity, a correlation analysis indicates that the number of SO in administrative regions is highly correlated with total population and GDP (**Figure S1c**). Accordingly, based on the four major economic regions defined by the National Bureau of Statistics, we find that the eastern region has the highest number of SO, followed by the western and northeast regions. Among these, the provinces of Zhejiang, Guangdong, and Jiangsu have reported the most outbreaks, while Sichuan and Guangdong have the highest number of FP (Figure 3c). The western regions of Guangxi, Guizhou, and Yunnan also have an increased risk of EF (**Figure S2**). Regarding the specific settings, residential areas are indicated as the main sites of SO, followed by restaurants and schools, with residential areas and schools having the highest number of EF (Figure 3d). FP in mobile street stalls presents a substantial public health concern due to their significant impact on the average number of individual outbreaks (**Figure S1b**). Although the number of T/TP in factories seems relatively low, their large-scale impact emphasizes the importance of addressing cross-contamination. Notably, military hospitals, apart from regular hospitals, experience the most significant scale of HAI. By integrating the spatiotemporal aspects, the geographical distribution of SO in China has undergone a dramatic transformation since the implementation of the opening-up policy in 1978, shifting from sporadic nationwide outbreaks to frequent occurrences in coastal provinces and their hinterlands (Figure 3e). Furthermore, since 2000, in addition to the persistently high incidence in southeast coastal areas, Yunnan and Sichuan provinces- have witnessed a relatively elevated number of SO.

### Etiology of salmonellosis outbreaks

**Figure 4.**
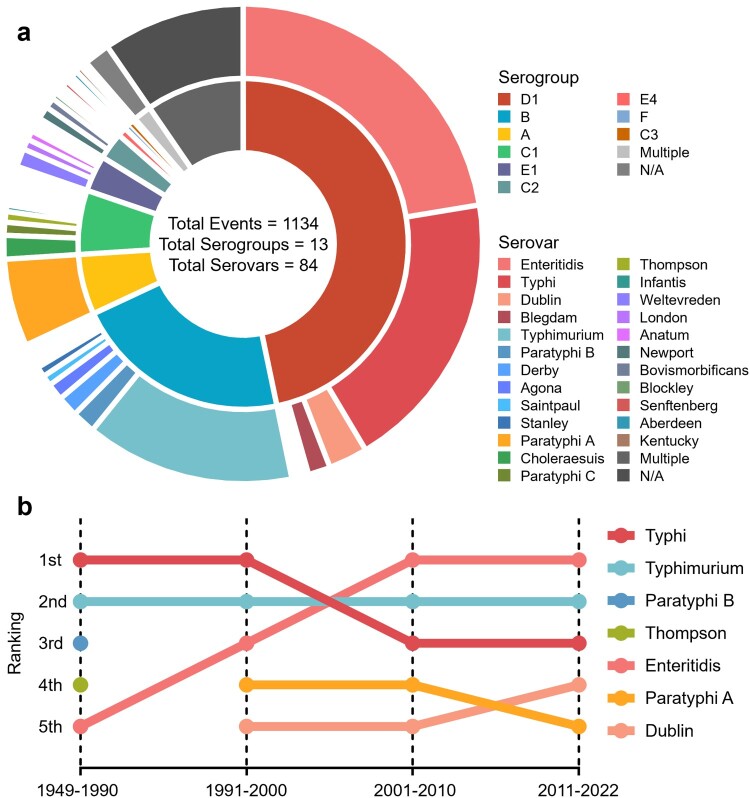
**Diversity of serovars identified in salmonellosis outbreaks**. **a.** The composition of serogroups (inner ring) and serovars (outer ring) that cause *SO*, as well as different serogroups or serovars within the same serogroup, are ranked clockwise from highest to lowest. **b.** Ranking the historical trends of the main *Salmonella* serovars responsible for outbreaks.

Laboratory diagnosis and clinical investigations are two crucial steps in managing SO. In China, selective bacterial culture, immunological tests, and biochemical identification are the three most commonly used methods for pathogen detection during SO. PFGE, PCR techniques, and WGS are gradually used for source tracing (**Figure S3**). Due to their limited applicability and the aim of the investigation, which led to a scarcity of genotyping description reports (8/1134), serotyping has become the primary focus of the current study. Among the 84 serovars of the 13 serogroups associated with SO in China, *Salmonella* Enteritidis (*S.* Enteritidis), *S.* Typhi, and *Salmonella* Typhimurium (*S.* Typhimurium) were the three major serovars (Figure 4a). In recent years, there has been a gradual shift in the predominant serovar, with *S.* Enteritidis becoming the most prevalent, while *S.* Typhimurium has consistently posed a high risk of outbreaks (Figure 4b). Outbreaks caused by *S.* Typhi have been widely distributed across eastern, central, and western regions of China (**Figure S4**). The spatiotemporal changes have shifted from a predominantly northern distribution to nationwide expansion and eventually to a mostly southern-prone endemic (**Figure S5**).

According to the reference Chinese Food Production Licensing Classification Catalog, meat products, pastries, and egg products are the most common types of food associated with *Salmonella* FP (**Figure S6**). Notably, pastries, mainly those served in school settings, rank second in causing *Salmonella* (mainly *S.* Enteritidis) FP, following meat products. *S.* Enteritidis has the broadest geographical distribution and is among the food categories implicated in FP. Residential areas and restaurants are the most frequent settings for FP, with meat products (the food category with the highest diversity of serovars) being the primary contaminated food source. The antimicrobial treatment for SO in China mainly relies on quinolone (QNs, 49.51%), with chloramphenicol (CPs, 56.10%) being predominantly used for EF and aminoglycoside (AGs, 71.05%) being used mainly for HAI (**Figure S7a**). Additional antimicrobial resistance analysis suggests that resistance to beta-lactams (BLs, 73.76%; BLs excluding AMP, 58.82%) is the most severe and has remained high over the years (**Figure S7b**). Alarmingly, HAI strains have the highest potential for antimicrobial resistance (**Figure S7c**). Antimicrobial resistance analysis of the four main serovars in SO shows that *S.* Typhimurium has the highest rate of antimicrobial resistance, with minimal changes observed before and after the year 2000, while the resistance spectra of *S.* Enteritidis and *S.* Typhi show significant variations, and the *S.* Typhi resistance rate in *S.* Paratyphi A has shown some alleviation (**Figure S7d-h**). From 270 death cases (0.30%) out of 96 outbreaks (8.47%), *S.* Typhimurium was the primary serovar-causing fatal outbreak, followed by *S.* Typhi (**Figure S8a-d**). Although the number of HAI is relatively low, the percentage of deadly events (49.30%) and the average number of deaths per catastrophic event (3.83) are substantially higher than the overall levels (8.47%, 2.81) (**Figure S8e**). Considering the higher lag deviation and antimicrobial resistance risk associated with nosocomial infections, these SO in healthcare settings should require particular attention.

### Meta-analysis of patient information

**Figure 5.**
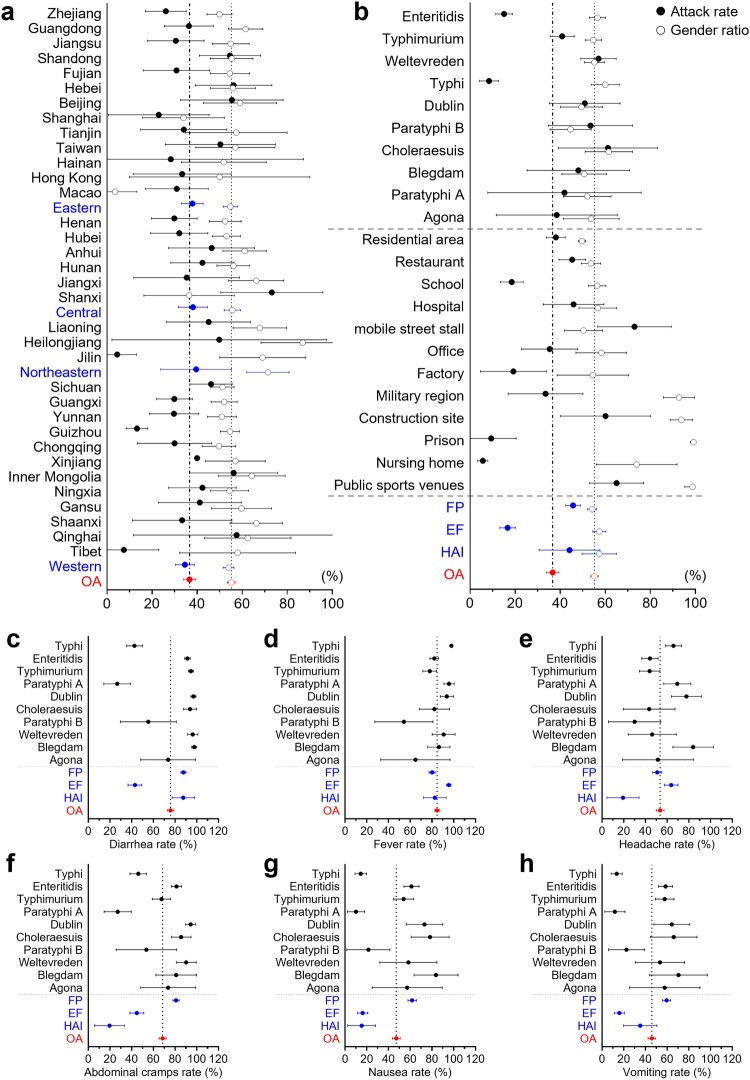
**Forest plot for the subgroup meta-analysis: attack rate, gender ratio, and incidence rate of designated symptoms**. The overall proportions are shown in red. **a** and **b** are attack rate (solid circles) and gender ratio (hollow circles) with a 95% confidence interval. **a.** Subgroup analysis based on provinces and economic regions (in blue). **b.** Subgroup analyses based on settings, event clustering types (in blue), and the top ten serovars for the number of events in the 506 outbreaks included in the meta-analysis. Subgroup analysis of the top ten serovars and event clustering types (in blue) on incidence rates of designated symptoms regarding **c.** diarrhea, **d.** fever, **e.** headache, **f.** abdominal cramps, **g.** nausea, **h.** vomiting with a 95% confidence interval.

Information on the attack rate (AR), gender ratio (GR), and incidence rate (IR) of designated symptoms extracted from 506 high-quality reports of SO in China was used for meta-analysis and the following subgroup analysis (**Table S5**). The overall AR was 36.66% (95% CI, 33.88-39.45%), and the gender ratio was calculated as 55.19% (95% CI, 53.47-56.90%) (Figure 5). Subgroup analyses of prevalence rate and gender ratio were done based on administrative divisions, economic regions, the top ten serovars, outbreak settings, and event clusters. Referring to the clinical diagnostic guidelines for *Salmonella* infection provided by the U.S. Centers for Disease Control and Prevention, the IR of six designated symptoms was determined and calculated as follows: diarrhea 75.81% (95% CI, 72.78-78.85%), fever 84.67% (95% CI, 82.53-86.80%), headache 53.53% (95% CI, 49.91-57.14%), abdominal cramps 68.51% (95% CI, 65.28-71.74%), nausea 47.11% (95% CI, 43.50-50.72%), and vomiting 45.91% (95% CI, 42.43-49.39%). Subgroup analyses of the prevalence rates of these six designated symptoms were also done based on the top ten serovars and event clusters.

## Discussion

This study provides the most extensive and up-to-date datasets, employing various statistical approaches and visualizations to project epidemiological patterns. This effort ultimately contributes to improving control strategies for salmonellosis in China [31]. Spatiotemporal analysis revealed that China experienced a gradual increase in SO since the economic reform and opening-up policy were implemented in 1978, which led to significant developments in import-export trade [32]. Meanwhile, we also obtained consistent results from the regional distribution, supported by the fact that coastal provinces with prosperous foreign trade had more SO. Based on the correlation analyses, it can be speculated that globalization and trade are essential factors influencing SO in China. Summer, especially between May and September, is the period of a high incidence of SO, coinciding with the optimal growth temperature of *Salmonella*, and such a clear seasonal tendency is the critical point for infectious disease interventions. Surprisingly, identifying outbreaks of HAI can be challenging due to prolonged exposure in indoor temperature-controlled environments and the complexity of clinical settings. Considering the higher HB in reporting and increased fatality risk (due to the high susceptibility to salmonellosis among sub-healthy individuals and pathogens with a broader spectrum of antimicrobial resistance), the mitigation strategies for HAI caused by *Salmonella* are worthy of further attention [33, 34]. While climate factors significantly influence the temporal dimension of SO, in the spatial distribution dimension, human social behaviours, including the aggregation and growth of urban populations, enhanced epidemiological investigations, and reporting awareness in economically developed regions, as well as the unique dietary structure and lifestyle habits in the southwest region, exert a more significant impact on the reported number of SO.

Based on the fact that serovars *S.* Enteritidis, *S.* Typhi, and *S.* Typhimurium caused 55.56% (630/1134) of the SO in China, and as the dominant serovars continue to change, *S.* Enteritidis and *S.* Typhimurium remain two major ones [35]. The issue of *Salmonella* resistance to antimicrobials is becoming increasingly prominent in clinical practice [35, 36]. In particular, *S.* Typhimurium, which leads at high levels and carries the broadest antimicrobial resistance spectrum, is the major serovar responsible for fatal SO. In recent years, a monophasic variant of *S.* Typhimurium has emerged, and indeed, only two outbreaks have been reported in western China, showing a heightened risk for the potential emergence of more related outbreaks [37]. Overall, we should be alert to the healthcare burden of *S.* Enteritidis FP (especially in residential areas and restaurants) and the antimicrobial resistance crisis associated with *S.* Typhimurium outbreaks. Another matter of concern is the emergence of four outbreak isolates with carbapenem-resistant phenotypes in recent years, occurring in 2007, 2012, 2018 (imipenem-resistant), and 2020 (ertapenem – resistant) [38]. Although the mortality of SO in China is relatively low, the severity of deaths among patients in HAI-related settings indicates the importance of timely medical assistance for sub-healthy populations affected by *Salmonella* infections, especially supportive therapy for those infected with *S.* Typhimurium or *S.* Typhi.

Subgroup analyses suggested high homogeneity in AR and GR across economic regions, with significant differences in only a few provinces. The AR of EF was significantly lower than the overall population at 16.67% (95% CI, 13.17-20.17%) due to the large exposed population, which influenced the lower AR in school, factory and military regions. In addition, the lower AR in prisons and nursing homes may be due to the restricted crowd mobility, which reduces the frequency of contact between the exposed population and the pathogen. In contrast, mobile street stalls, construction sites, and public sports venues are settings with high-level crowd mobility, where the higher frequency of contact between exposed populations and pathogens increases the AR of epidemic outbreaks, and environmental hygiene and routine disinfection must be strictly enforced. Affected mainly by construction sites, prisons, and military regions, the overall gender ratio of SO in China is higher than the sex ratio of the Chinese population, with men having a higher risk of disease [39].

Discussing the incidence of designated symptoms would benefit the clinical diagnosis and symptomatic-based treatment [40]. We found that fever was the most common and consistently present symptom in SO, followed by diarrhea (with the highest IR in FP and HAI), while the four gastrointestinal symptoms of diarrhea, stomach cramps, nausea and vomiting showed significant pathogenic variability and differences in event or episode clusters, particularly in EF where the IR of gastrointestinal symptoms was significantly lower than the overall rate. It is worth noting that the lack of subjective symptom recording in acute deaths, probably due to the poor expression of critically ill patients or infants in healthcare-related settings, results in a lower IR of the three symptoms of headache, abdominal pain and nausea in HAI, but does not selectively ignore analgesic or antiemetic treatment [41].

The present study is subject to the following limitations: (1) The lack of open access to official Chinese monitoring systems databases precludes access and comparison with datasets from other countries. (2) The presentation of early Chinese data is passive, with significant outbreaks being the main focus for recording and reporting. This approach may result in errors in statistical interpretation and heterogeneity between reality and analytical results. (3) The veracity of the data is contingent upon the distribution of local technological and human resources, which may not fully align with the local disease burden. Consequently, further investigation is warranted, particularly in less developed regions. (4) Given the distinctive national circumstances in China, a sensitivity towards outbreaks that could cause societal panic may result in related events being treated as confidential and inaccessible for public disclosure. After a comprehensive examination of the achievements and shortcomings of our research, we propose the following recommendations for public health initiatives regarding SO in China and other developing countries: (1) To ensure the protection of individual privacy, it is essential to promptly share outbreak data resources with research institutions. This will enhance the accuracy of epidemic prediction and the professionalism of prevention and intervention. (2) In light of the impact of global warming, it is recommended that the key monitoring periods of national CDCs be extended during epidemic seasons, particularly in spring and autumn. (3) It is recommended that food safety be given a high priority, with long-term monitoring of China’s unique fermented food products. (4) The implementation of updated detection technologies for *Salmonella* pathogens is a crucial step in the prevention of outbreaks.

Foodborne pathogens are ubiquitous, and due to the complex and expanding farm-to-table production chain, a transmission chain spanning the environment, animals, food, and humans highlights the contemporary value of the One Health concept [42]. *Salmonella*, a common and widely studied pathogen, has gradually transcended ecological boundaries, exploiting diverse host niches and multidimensional transmission pathways [43, 44]. *Salmonella* represents an undeniable threat to the lives and health of the Chinese population, particularly during SO events, which always deal a substantial blow (economic losses and human casualties) to the relaxed infectious disease prevention and control systems [45, 46]. In conclusion, this study unveils the epidemiological trends in China attributed to SO, providing a foundation for developing countries and the global community to comprehend the burden of Salmonellosis and to make informed predictions about outbreaks.

## Supplementary Material

Supplemental Material

Supplemental Material

Supplemental Material

Supplemental Material

Supplemental Material

Supplemental Material

Supplemental Material

## Data sharing

Data collected for the study will be made available from the FIgshare platform upon reasonable request with CC-BY license (https://doi.org/10.6084/m9.figshare.24033690).
