# Supplementary material for: Nationwide trends and features of human salmonellosis outbreaks in China
Source: Emerg Microbes Infect. 2024 Jun 26;13(1):2372364. doi: 10.1080/22221751.2024.2372364 (PMC11259058; doi:10.1080/22221751.2024.2372364)
Supplement: Supplemental Material [file TEMI_A_2372364_SM2464.pdf]

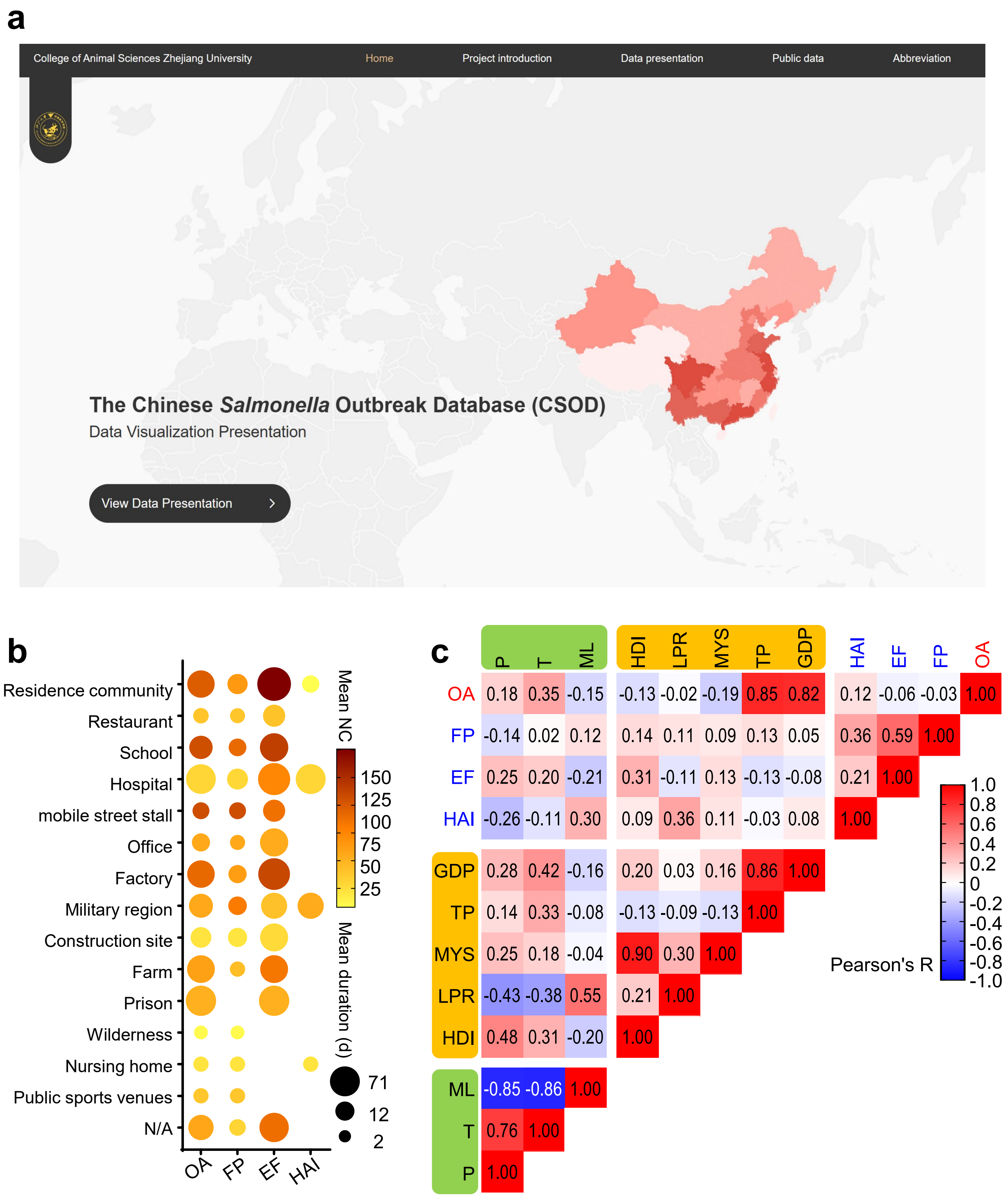

Figure S1. Visualization of the CSOD v1.2, setting distribution, and correlation analysis based on outbreak provinces

a. Homepage of the CSOD v1.2 visualization database website. **b.** The size of outbreaks in different settings is indicated by the mean number of new cases (NC) and the mean duration. **c.** Correlation analysis of potential factors (social-economic in orange and climatic in green) affecting the number of outbreaks per province.

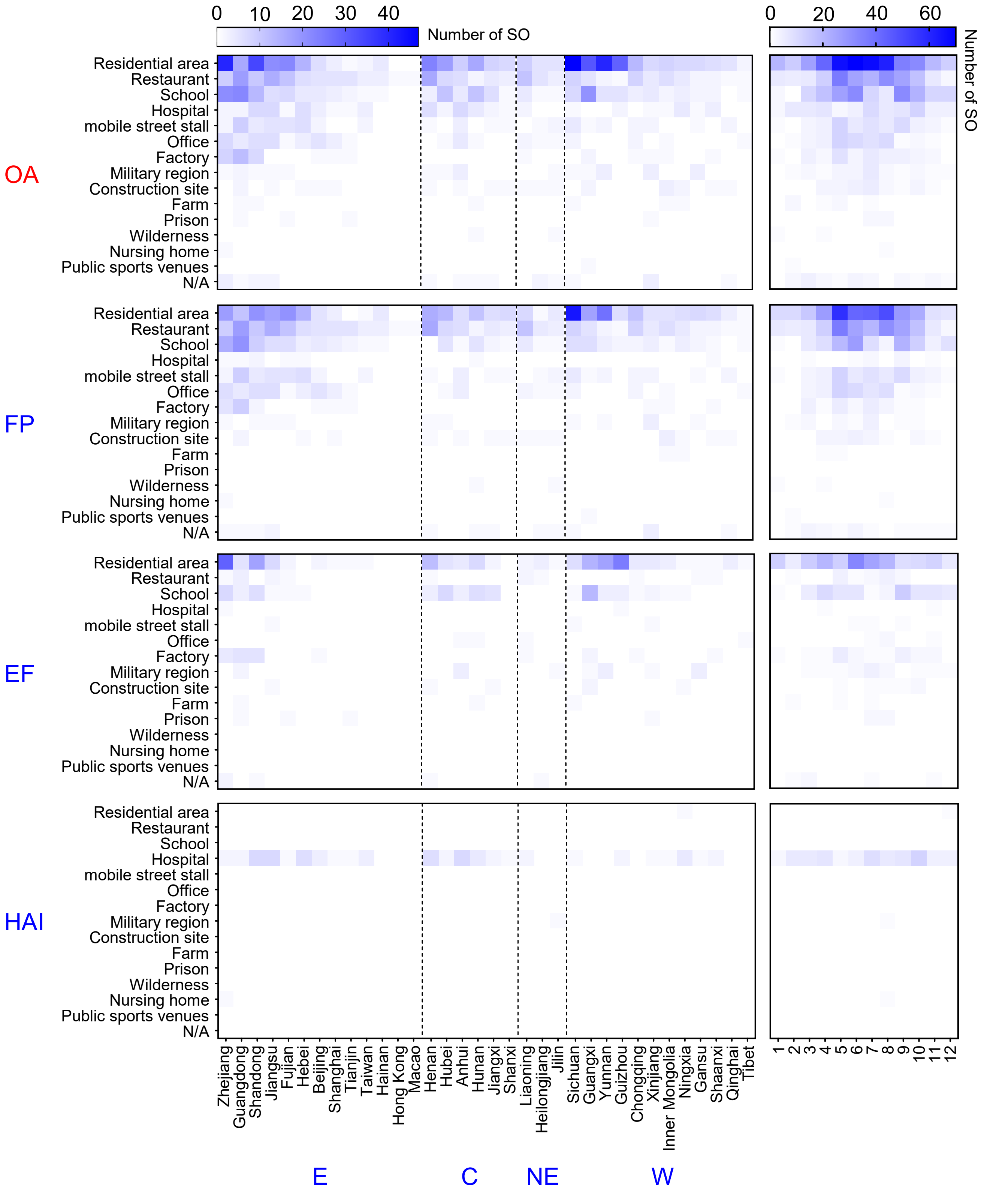

Figure S2. Additional details on spatial distribution of outbreaks

Based on the overall and three event clusters, the number of outbreaks at each type of setting within various provinces (and economic regions, four heat maps on the left) or various months (four heat maps on the right) is shown.

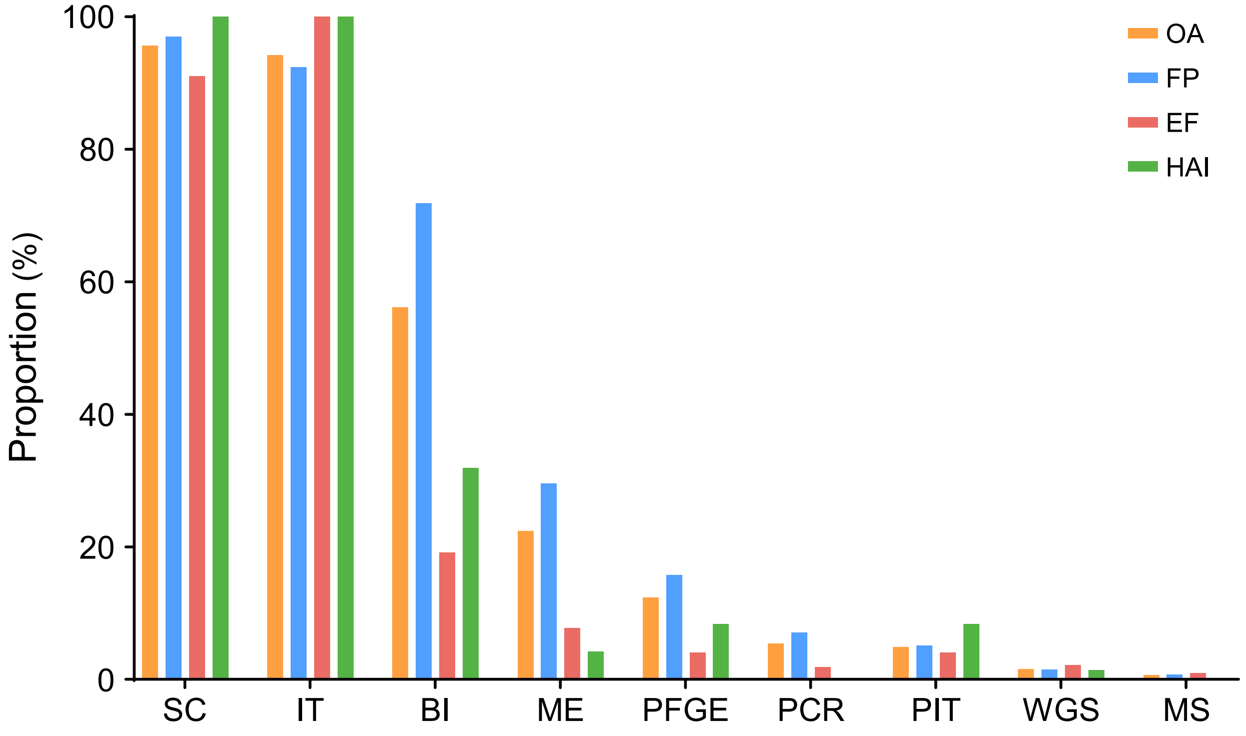

Figure S3. Statistical analysis of laboratory diagnostic techniques

The proportion of laboratory pathogen diagnostic techniques occurring overall and in the three event clusters. The abbreviations are explained in the Table S6.

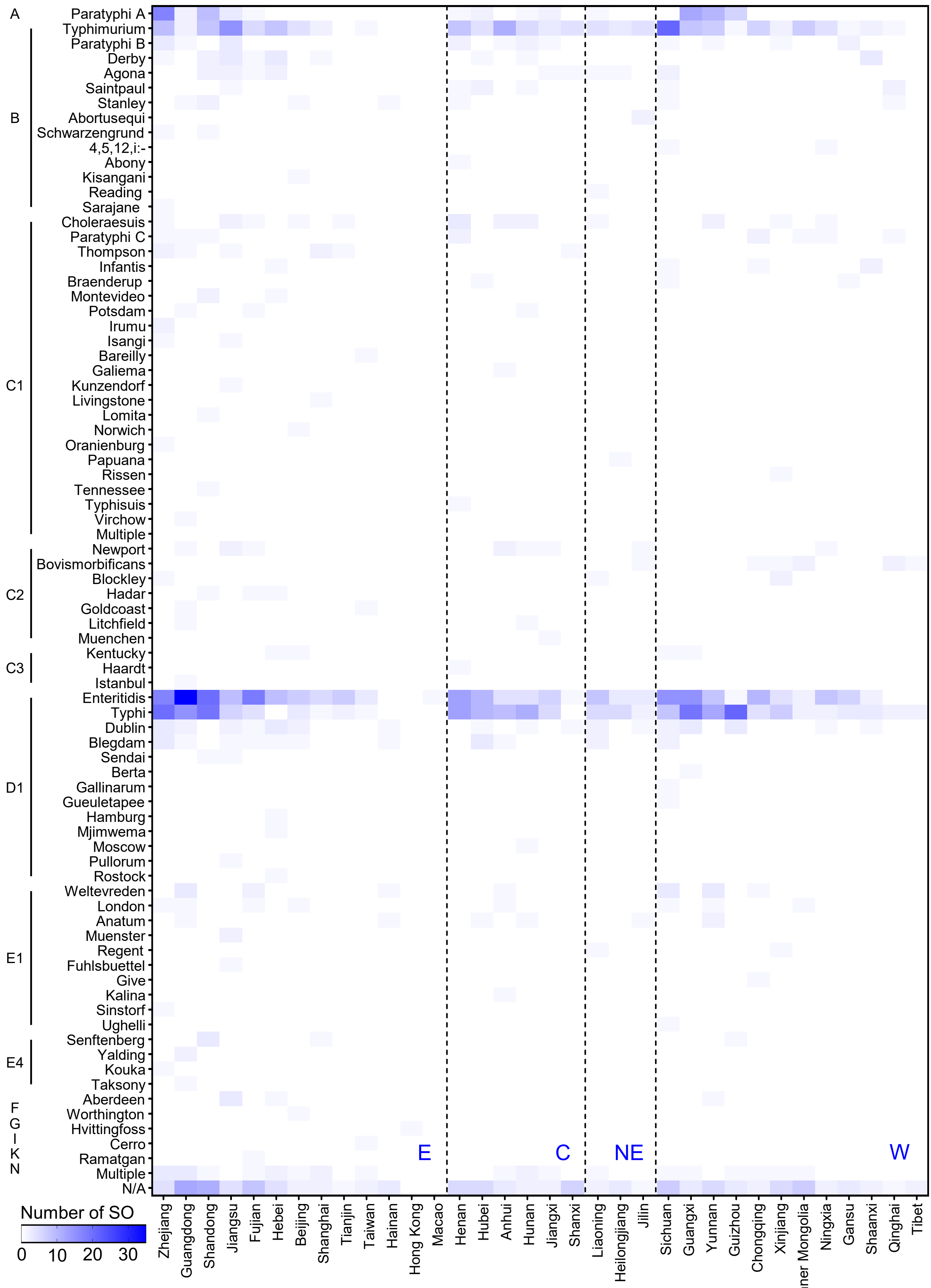

Figure S4. Distribution of Salmonella serovars in outbreaks by province

The number of outbreaks caused by different *Salmonella* serovars in each province, ranked from highest to lowest within the same serogroup.

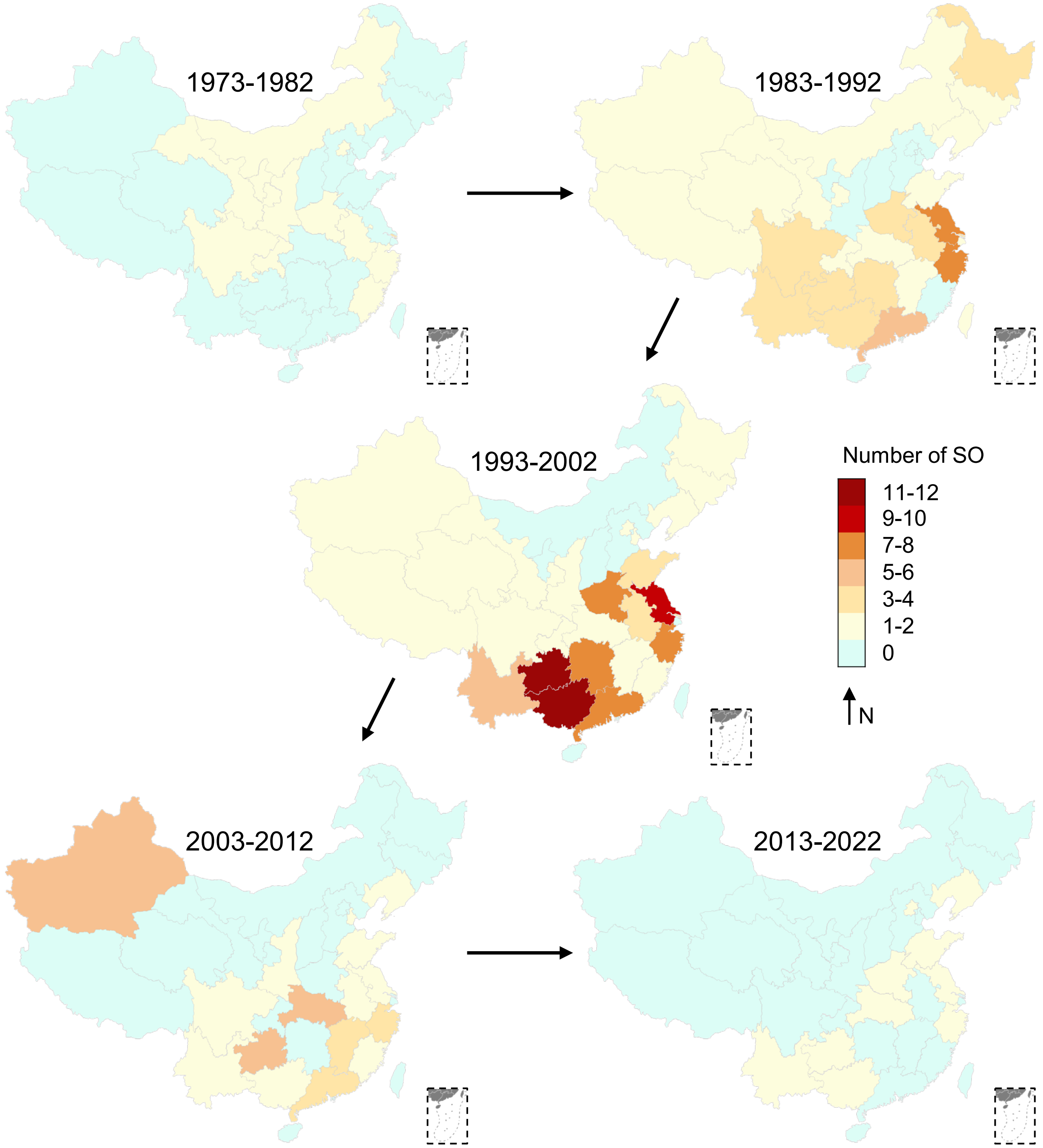

Figure S5. Trends in the dissemination of typhoid fever outbreaks in China

Spanning the documented period of 1973-2022, depicted in ten-year intervals, showcasing the number of typhoid fever outbreaks across provinces.

	Meat products	Pastries	Egg products	Vegetable products	Aquatic products	Legume products	Processed grain products	Nut products	Seasonings	Non-frozen beverages	Starch products	Fruits	Dairy products	Infant formula	Potatoes	Frozen food	Frozen beverages	Others	N/A
OA	386	67	52	20	19	15	12	8	8	5	5	4	3	3	2	2	1	41	165
Enteritidis	95	49	16	3	5	4	8	1	5	1	0	1	1	0	1	0	1	20	43
Typhimurium	66	3	15	2	2	2	0	0	0	0	1	0	1	0	0	1	0	1	19
Typhi	7	1	1	2	1	0	1	0	0	0	3	1	0	0	0	0	0	1	24
Dublin	13	2	3	2	1	0	0	0	1	0	1	0	0	0	0	0	0	2	5
Blegdam	7	3	2	0	0	0	0	0	1	0	0	0	0	0	0	0	0	1	3
Paratyphi B	10	0	1	1	0	0	0	0	0	0	0	0	0	1	0	0	0	0	2
Choleraesuis	12	0	0	0	0	1	0	0	0	0	0	0	0	0	0	0	0	0	2
Paratyphi A	0	0	0	2	0	0	0	0	0	0	0	0	0	0	0	0	0	0	12
Weltevreden	6	0	0	1	0	0	0	0	0	0	0	2	0	0	0	0	0	1	4
Derby	11	0	0	0	1	0	0	0	0	0	0	0	0	0	0	0	0	0	1
Residential area	173	8	22	8	4	6	3	2	1	1	2	2	0	0	1	1	0	18	67
Restaurant	93	8	9	3	12	1	6	4	3	2	0	0	0	0	0	1	0	12	38
School	36	30	7	5	3	0	2	0	1	0	1	0	2	0	0	0	1	7	25
mobile street stall	28	15	2	0	0	3	0	1	2	1	1	2	1	0	0	0	0	1	1
Office	18	4	7	2	0	2	1	1	0	1	0	0	0	0	0	0	0	1	12
Factory	7	1	2	2	0	0	0	0	1	0	1	0	0	0	0	0	0	0	8
Construction site	10	0	1	0	0	0	0	0	0	0	0	0	0	0	1	0	0	0	5
Military region	9	0	1	0	0	0	0	0	0	0	0	0	0	0	0	0	0	0	2
Hospital	1	1	0	0	0	1	0	0	0	0	0	0	0	3	0	0	0	1	1
Wilderness	2	0	0	0	0	0	0	0	0	0	0	0	0	0	0	0	0	0	0
Farm	1	0	1	0	0	0	0	0	0	0	0	0	0	0	0	0	0	0	0
Nursing home	0	0	0	0	0	0	0	0	0	0	0	0	0	0	0	0	0	0	1
Public sports venue	1	0	0	0	0	0	0	0	0	0	0	0	0	0	0	0	0	0	0

Figure S6. Categories and characteristics of Salmonella sources in food poisoning

The number of *Salmonella* foodborne outbreaks by food categories, differentiated by overall (in red), top ten serovars (in blue), and various settings (in green).

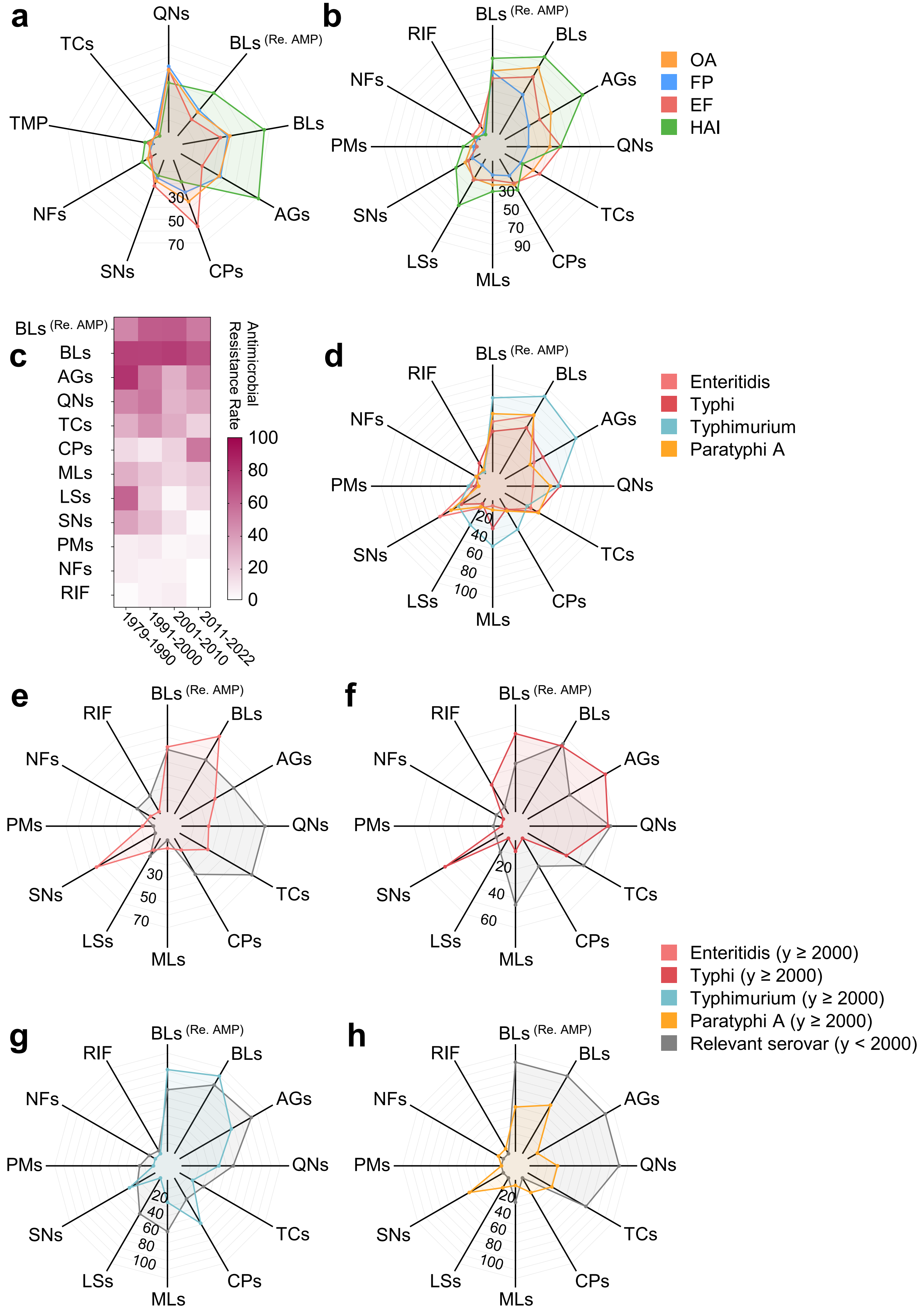

Figure S7. Treatment options and antimicrobial resistance

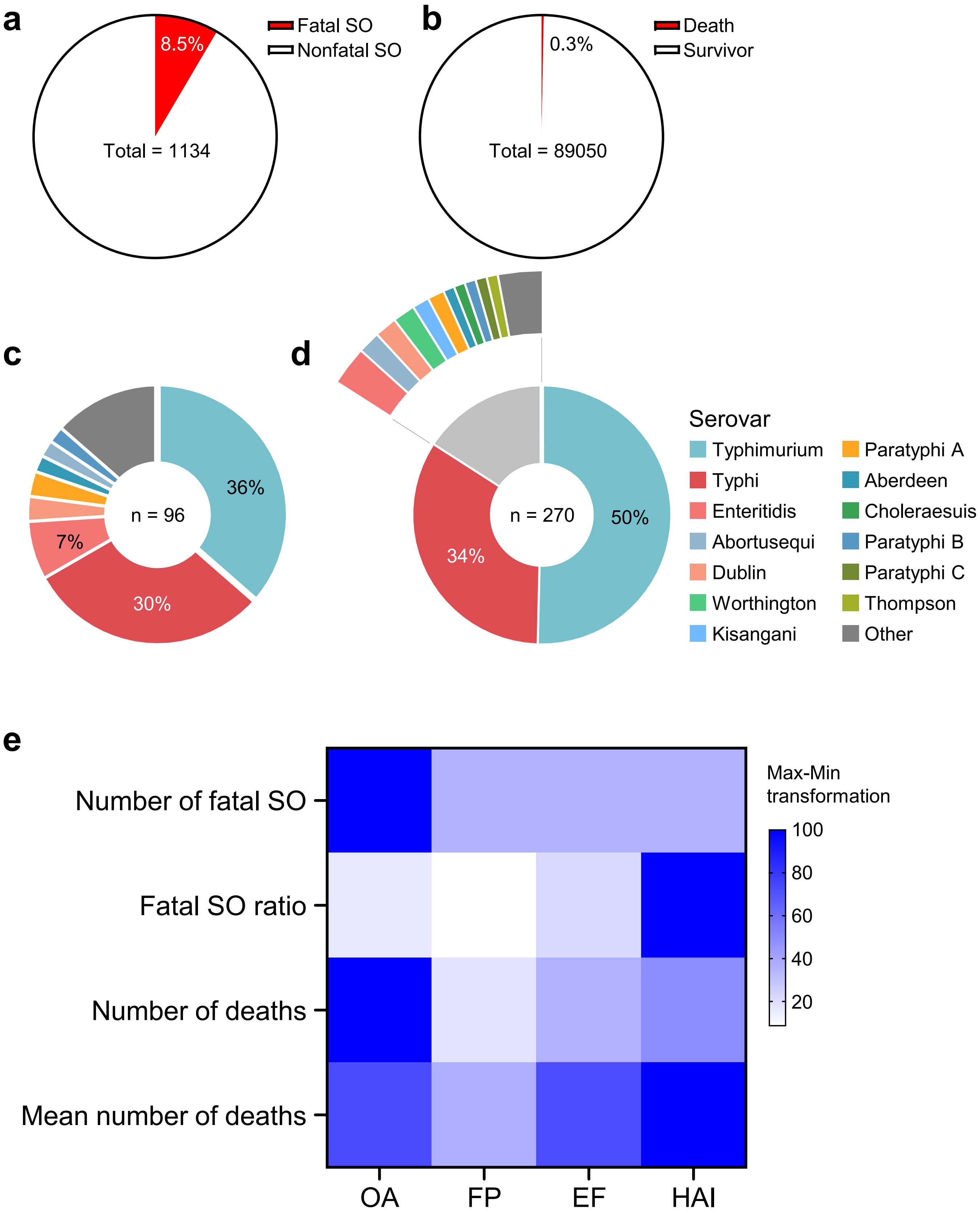

Figure S8. Overview of fatalities in Salmonella outbreaks

a. The proportion of outbreaks with recorded patient fatalities. **b.** The proportion of recorded deceased patients. **c.** The composition of *Salmonella* serovars causes fatal outbreaks. **d.** The composition of *Salmonella* serovars causes patient fatalities. Shared legend for c and d positioned on the right. **e.** Comparison of fatality outcomes across the overall and three event clusters (converted via max-min transformation).
