# Supplementary material for: Nationwide trends and features of human salmonellosis outbreaks in China
Source: Emerg Microbes Infect. 2024 Jun 26;13(1):2372364. doi: 10.1080/22221751.2024.2372364 (PMC11259058; doi:10.1080/22221751.2024.2372364)
Supplement: Supplemental Material [file TEMI_A_2372364_SM2461.pdf]

Table S1: Preliminary bibliography of literature obtained from database searches that have not yet been assessed against inclusion criteria
Sheet1: Chinese publication
Sheet2: English publication

Sheet1: Chinese publication											
No.	Title	Title_E.T.	Authors	Authors_E.T.	Journal	Journal_E.T.	Volume	Issue	Page	Publication Year	DOI
1	感染：肠炎杆菌败血病十	Study of Seventeen Cases o	黄祯祥,张孝骞,刘维德.	henxiang,Zhang Xiaoqian,Li	中华医学杂志	Chinese Medical Journal	23	5	668-669	1937	-
2	中央医院内肠炎沙门氏菌	Acute Gastroenteritis in Na	徐采,阎林肯,黎磊石.	Xu Cai,Yan Lincoln,Li Leishi	内科学报	ournal of Internal Medicine	1	1	57-58	1949	-
3	门氏菌属猪霍乱感染两例报	cases of Salmonella cholera	任芳璧,潘玉堂.	Ren Fangbi,Pan Yutang.	中华内科学报	ese Journal of Internal Med	3	6	446-448	1955	-
4	引起的细菌性心内膜炎（	litis caused by Salmonella s	胡远峰.	Hu Yuanfeng.	中华内科杂志	ese Journal of Internal Med	3	12	922-924	1955	-
5	起的二十九例沙门氏菌急	s of acute Salmonella infec	纲,黄大有,郝士海,林旭东	Huang Dayou,Hao Shihai,Li	中华卫生杂志	Chinese Health Journal	3	3	212-219	1955	-
6	氏菌属猪霍乱菌感染临床	of Salmonella choleraesuis	顾寄真,李彦三,张瑞祥.	izhen,Li Yansan,Zhang Ruix	中华内科杂志	ese Journal of Internal Med	3	12	918-921	1955	-
7	氏菌属引起的细菌性心内	ndocarditis caused by Salm	胡远峰,李邦琦（摘）.	Yuanfeng,Li Bangqi (excer	中华医学杂志	Chinese Medical Journal	42	2	188-189	1956	-
8	氏菌属细菌性心内膜炎一	cterial endocarditis caused	任宏造,金春和.	Ren Hongzao,Jin Chunhe.	中华内科杂志	ese Journal of Internal Med	5	8	622-624	1957	-
9	猪霍乱沙门氏菌113株的初	in Guiyang City113Prelimi	廖子哲.	Liao Zizhe.	中华医学杂志	Chinese Medical Journal	43	6	431-434	1957	-
10	市屠宰猪沙门氏菌感染的	monella infection in slaugh	郝士海,戴寅,周桂莲.	o Shihai,Dai Yin,Zhou Guili	中华卫生杂志	Chinese Health Journal	5	2	104-105	1957	-
11	杆菌败血症并发病理性	by pathological femur fract	汉勋,冯汉魂,谢沸迅,邓存	,Feng Hanhun,Xie Feixun,D	中华医学杂志	Chinese Medical Journal	43	1	44-45	1957	-
12	发沙门氏猪霍乱杆菌感染	monella Choleraesuis-infe	刘荣耀,陈树德.	Liu Rongrong,Chen Shude.	中华外科杂志	Chinese Journal of Surgery	5	6	517	1957	-
13	上海市沙门氏菌族感染临	is Report on Salmonella Gr	文寿,吴明漪,陆钟贞,陈乃	,Wu Mingyi,Lu Zhongzhen,	中华医学杂志	Chinese Medical Journal	43	7	497-504	1957	-
14	霍乱沙门氏菌感染问题的	n choleraesuis Salmonella	志靖,周邦靖,陈永龄,平镜	u Bangjing,Chen Yongling,	中华内科杂志	ese Journal of Internal Med	5	8	618-621	1957	-
15	氏菌属猪霍乱菌感染的临	ysis of Salmonella cholerae	许行健,张铮.	Xu Xingjian,Zhang Zheng.	中华内科杂志	ese Journal of Internal Med	5	8	614-617	1957	-
16	氏菌食物传染一起的卫生	vestigation of food-infected	陈邦宪,叶自儁.	Chen Bangxian,Ye Zijun.	中华卫生杂志	Chinese Health Journal	5	2	96-100	1957	-
17	菌（S. kisangani）引起食	kisangani) Diagnosis of path	郝士海,周桂莲,戴寅.	o Shihai,Zhou Guilian,Dai Y	中华卫生杂志	Chinese Health Journal	6	3	162-164	1958	-
18	海市沙门氏菌属的类型分	ution of Salmonella genus	,叶自俊,刘雪园,尤锡根,	Zijun,Liu Xueyuan,You Sig	中华卫生杂志	Chinese Health Journal	6	3	179-185	1958	-
19	菌属的类型分布及流行病	ution and epidemiological i	邦靖,张永华,李光富,林志	g,Zhang Yonghua,Li Guang	中华卫生杂志	Chinese Health Journal	6	3	185-188	1958	-
20	氏菌引起的一次食物中毒	oisoning infection caused b	王月华,刘以贤,杨传华.	Yuehua,Liu Yixian,Yang Chu	中华卫生杂志	Chinese Health Journal	6	3	167-169	1958	-
21	菌（S. Newport）引起甲	(S. Newport) caused a cas	丘文宣,王粹然,陈长贵.	nxuan,Wang Cuiran,Chen C	中华医学杂志	Chinese Medical Journal	44	3	297-298	1958	-
22	沙门氏菌急性感染122例调	n acute infection122Exan	孔庆长.	Kong Qingchang.	中华卫生杂志	Chinese Health Journal	6	3	164-167	1958	-
23	分离的118株沙门氏菌初	City118Preliminary analys	曾凡济.	Zeng Fanji.	中华医学杂志	Chinese Medical Journal	46	2	143-146	1960	-
24	某村乙型副伤寒暴发流行	break of paratyphoid feve	-	-	山东医刊	Shandong Medical Journa	-	5	16+15	1961	-
25	少见的马流产沙门氏菌食	lmonella food poisoning in	姜克.	Jiang Ke.	中华医学杂志	Chinese Medical Journal	48	5	331	1962	-
26	猪霍乱亚种沙门氏菌骨髓	choleraesuis subspecies os	王承武,王澍寰.	ang Chengwu,Wang Shuhu	中华外科杂志	Chinese Journal of Surgery	11	6	451-452	1963	-
27	猪霍乱感染并发溶血性贫	holeraesuis infection compl	潘玉堂.	Pan Yutang.	中华内科杂志	ese Journal of Internal Med	11	12	981	1963	-
28	地区沙门氏菌属的类型分	on of Salmonella genus in Z	张嵩山.	Zhang Songshan.	中华卫生杂志	Chinese Health Journal	9	3	181-183	1964	-
29	（S. boris morbeficans）引	e (S. boris morbeficans) fo	本枝,袁文起,瞿民辉,邓君	i,Yuan Wenqi,Qu Minhui,D	中华卫生杂志	Chinese Health Journal	9	2	129	1964	-
30	霍乱沙门氏菌感染72例临	n in adults in Guiyang City	杨勤槐,张国培.	Yang Qinhuai,Zhang Guopei	中华内科杂志	ese Journal of Internal Med	12	9	849	1964	-
31	副伤寒沙门氏菌引起的食	ning caused by Salmonella	苗五阳.	Miao Wuyang.	中华卫生杂志	Chinese Health Journal	9	3	170	1964	-
32	对沙门氏菌属诊断价值的	alue of blood and bone ma	-	-	中华内科杂志	ese Journal of Internal Med	12	10	994	1964	-
33	门氏菌引起的食物中毒调	food poisoning caused by	,苗克远,曾惠春,于哲生,	eyuan,Zeng Huichun,Yu Zh	中华卫生杂志	Chinese Health Journal	9	3	143	1964	-
34	炎沙门氏菌食物中毒调查	eritidis food poisoning inve	任耀先,罗兴祖.	Ren Yaoxian,Luo Xingzu.	中华卫生杂志	Chinese Health Journal	9	5	287	1964	-
35	的丁沙门氏菌引起的食物	ning caused by Salmonella	李兰田,封培信.	Li Lantian,Feng Peixin.	中华卫生杂志	Chinese Health Journal	9	3	154	1964	-

36	乱沙门氏菌食物中毒调查	rt on Choleraesuis Salmone	王声涌,余长兴.	ang Shengyong,Yu Changxi	中华卫生杂志	Chinese Health Journal	9	5	294	1964	-
37	值、活菌数与沙门氏菌检	ue, viable bacterial count a	-	-	中华卫生杂志	Chinese Health Journal	9	2	126	1964	-
38	寒沙门氏菌食物中毒调查	rt on Salmonella Typhimuri	元芳,陶显亮,戴光世,张洪	ao Xianliang,Dai Guangshi,	中华卫生杂志	Chinese Health Journal	9	5	323	1964	-
39	乩沙门氏菌上颌窦感染一	y sinus infection caused by	-	-	中华耳鼻咽喉科杂志	nese Journal of Otolaryngol	11	2	131	1965	-
40	可密沙门氏菌败血症一例	eport of salmonella sepsis i	-	-	中华内科杂志	ese Journal of Internal Med	13	6	571	1965	-
41	菌所致之胰腺脓肿、肺脓	cess and lung abscess caus	杨勤槐,钱大康.	Yang Qinhuai,Qian Dakang.	中华内科杂志	ese Journal of Internal Med	13	3	280-281	1965	-
42	氏菌食物中毒调查报告的	Salmonella food poisoning	-	-	中华卫生杂志	Chinese Health Journal	10	5	322-323	1965	-
43	门氏菌引起两次食物中毒	cases of food poisoning ca	,余长兴,杨宁远,钱祖根,	hangxing,Yang Ningyuan,	中华卫生杂志	Chinese Health Journal	10	5	298-299	1965	-
44	沙门氏菌所致的一次食物	a food poisoning caused by	姜必成.	Jiang Bicheng.	中华卫生杂志	Chinese Health Journal	10	5	296-297	1965	-
45	六四年八月中华医学会安	han Infectious Disease Aca	-	-	中华内科杂志	ese Journal of Internal Med	13	4	393-394	1965	-
46	口葡萄球菌食物中毒调查	monella and staphylococcal	-	-	中华卫生杂志	Chinese Health Journal	11	3	208-209	1966	-
47	水型伤寒暴发流行的流行	of an outbreak of waterb	-	-	新医学	new medicine	-	10	1	1974	-
48	物型伤寒暴发流行49例的	borne typhoid fever49clin	乔富渠	Qiao Fuqu	陕西新医药	Shaanxi New Medicine	-	3	17-18+20	1975	-
49	市××厂伤寒暴发的调查	tion report on typhoid fe	-	-	重庆医药	Chongqing Medicine	-	5	8-10+20	1977	-
50	起海岱堡沙门氏菌食物中	by eating homemade tem	葛素琴.	Ge Suqin.	铁道医学	Railway Medicine	-	5	277	1978	-
51	乩沙门氏菌食物中毒调查	nalysis of Salmonella Food	局中心卫生防疫站,牛兰	h and Epidemic Prevention	中华预防医学杂志	se Journal of Preventive Me	12	2	114-115	1978	-
52	菌感染并发血小板减少性	raesuis infection complicat	张麟.	Zhang Lin.	中华内科杂志	ese Journal of Internal Med	17	6	422	1978	-
53	沙门氏菌肠炎并发多尿症	ella typhimurium enteritis	王友真,黄才华.	ang Youzhen,Huang Caihu	中华内科杂志	ese Journal of Internal Med	18	6	418	1979	-
54	伤寒沙门氏菌感染50例报	yphimurium Infection50Ex	),周渝膺①,李磐玲①,王	,Zhou Yuying①,Li Panling	中华儿科杂志	Chinese Journal of Pediatric	17	2	80-82	1979	sn.0578-1310.1979.02.110
55	寒沙门氏菌感染156例临床	urium Infection156Case c	李凤英,拉莫,李传祜,樊生	,Ramo,Li Chuanhu,Fan She	中华儿科杂志	Chinese Journal of Pediatric	17	2	77-79	1979	sn.0578-1310.1979.02.108
56	门氏菌引起食物中毒暴发	poisoning outbreak caused	张桂宁.	Zhang Guining.	中华预防医学杂志	se Journal of Preventive Me	13	2	90	1979	-
57	部队农场伤寒暴发流行的	utbreak of typhoid fever	郭安荣;李志成;	Guo Anrong;Li Zhicheng	人民军医	people's military medicine	-	7	14-16	1979	-
58	村104例伤寒暴发的调查报	nvestigation report on ty	刘立峰;	Liu Lifeng;	河南预防医学杂志	Journal of Preventive M	-	3	61-64	1979	-
59	中的一次鼠伤寒沙门氏	almonella typhimurium in	郑传经.	Zheng Chuanjing.	宁夏医科大学学报	al of Ningxia Medical Univ	-	2	-	1980	-
60	伤寒沙门氏菌变异研究	n the variation of Salmonel	吉瑞庭,刘维先.	Ji Ruiting,Liu Weixian.	微生物学通报	Bulletin of Microbiology	7	5	-	1980	-
61	寒暴发43例临床及流行病	reak43Clinical and epid	沙介荣;顾益明;	Sha Jierong;Gu Yiming;	浙江医学	Zhejiang Medicine	-	4	25-26+14	1980	-
62	食源型伤寒暴发流行的调	t on an outbreak of foodb	宋清林;	Song Qinglin;	人民军医	people's military medicine	-	7	34-36	1980	-
63	叉感染引起的一次家庭中	a family caused by cross	顾清礼;	Gu Qingli;	宁夏医学院学报	al of Ningxia Medical Co	-	2	6-7	1980	-
64	起慕尼黑沙门氏菌食物中	salmonella food poisoning	蔡恒星,胡英俊.	Cai Xingxing,Hu Yingjun.	中华预防医学杂志	se Journal of Preventive Me	15	4	243-244	1981	-
65	自贡市沙门氏菌菌型分布	distribution and drug susc	梁正新.	Liang Zhengxin.	中华预防医学杂志	se Journal of Preventive Me	15	6	372	1981	-
66	鼠伤寒沙门氏菌耐药性的	drug resistance of Salmon	家璈,雍立英,俞惠敏,陈家	,Yong Liying,Yu Huimin,Ch	中华流行病学杂志	inese Journal of Epidemiol	2	4	257-259	1981	sn.0254-6450.1981.04.122
67	加内伦敦沙门氏菌的细菌	gnosis of Salmonella Lond	罗仲伯,程佩芸.	Luo Zhongbo,Cheng Peiyun	中华医学检验杂志	urnal of Medical Laborator	4	2	75	1981	-
68	达盟人群沙门氏菌菌型	onella strains in Zhaowu	玉龙,王国珍,王亚辉,付佩	Wang Guozhen,Wang Yahu	中华流行病学杂志	inese Journal of Epidemiol	2	4	278	1981	sn.0254-6450.1981.04.133
69	菌引起的结肠炎40例临床	lla typhimurium40Clinical a	李义方,杨畅.	Li Yifang,Yang Chang.	中华儿科杂志	Chinese Journal of Pediatric	19	2	107	1981	sn.0578-1310.1981.02.120
70	伤寒暴发流行的菌型调查	cterial types prevalent in	叶云凌;李明;	Ye Yunling;Li Ming;	浙江医学	Zhejiang Medicine	-	5	41-43	1981	-
71	山县沃角伤寒暴发流行的	reak of typhoid fever in V	世泉;王建利;黄再添;施丽	uan;Wang Jianli;Huang	福建医药杂志	Fujian Medical Journal	-	2	30-32	1981	-
72	食从业人员沙门氏菌带菌	ype distribution among cat	黎佩芳.	Lai Pei Fong.	铁道医学	Railway Medicine	-	2	99	1982	-

73	型分布及其对环境污染与	strains and their relationsh	罗兴祖,万绿波.	Luo Xingzu,Wanlvbo.	中华流行病学杂志	inese Journal of Epidemiolc	3	5	291-293	1982	sn.0254-6450.1982.05.118
74	区沙门氏菌菌型调查及药	ation and drug susceptibili	王启顺,张大兰,黄荣真.	shun,Zhang Dalan,Huang R	中华预防医学杂志	se Journal of Preventive Me	16	2	118-119	1982	-
75	菌柏林变种引起的医院内	hea in hospital caused by S	胡恩来,祖照基,陈林平.	Enlai,Zu Zhaoji,Chen Linpi	中华流行病学杂志	inese Journal of Epidemiolc	3	4	238	1982	sn.0254-6450.1982.04.126
76	鼠伤寒沙门氏菌引致爆发	ated Salmonella Typhimuri	钱本耀,庄志成.	ian Benyao,Zhuang Zhicher	中华流行病学杂志	inese Journal of Epidemiolc	3	5	296	1982	sn.0254-6450.1982.05.121
77	株伤寒沙门氏菌药物敏感	tibility testing of Salmonel	张敏,刘四华,白静琪.	hang Min,Liu Sihua,Bai Jing	中华预防医学杂志	se Journal of Preventive Me	16	2	128	1982	-
78	伤寒沙门氏菌菌血症一例	of Salmonella typhimurium	白玉龙,赵清志,张华.	Yulong,Zhao Qingzhi,Zhang	中华医学杂志	Chinese Medical Journal	62	6	370	1982	-
79	河北省沙门氏菌调查报告	investigation report in Heb	省沙门氏菌调查协作组,侯	a Investigation Collaboratic	中华预防医学杂志	se Journal of Preventive Me	16	3	176	1982	-
80	急性感染性腹泻的致病菌	ic bacteria in acute infectic	友红,徐麦玲,谈康英,徐肇	,Xu Mailing,Talk about Kan	中华内科杂志	ese Journal of Internal Med	21	9	500-542	1982	-
81	群沙门氏菌菌型分布、耐药	haracteristics of Salmonell	昌良,叶友松,朱时春,叶玉	ng,Ye Yousong,Zhu Shichur	中华流行病学杂志	inese Journal of Epidemiolc	3	2	69-73	1982	sn.0254-6450.1982.02.103
82	较研究：Ⅱ．从市售蔬菜	ods: Ⅱ. Isolation of contan	IREF="IMAGES/0253-962	HREF="IMAGES/0253-96	中华预防医学杂志	se Journal of Preventive Me	16	4	219-221	1982	-
83	沙门氏菌脑膜炎	salmonella meningitis	-	-	国际儿科学杂志	rnational journal of paedia	9	5	235-238	1982	sn.1673-4408.1982.05.104
84	霉素伤寒沙门氏菌的生物学	ics of chloramphenicol-resi	张玉萍,韩秀芝,贾明功,翟	ing,Han Xiuzhi,Jia Minggon	微生物学通报	Bulletin of Microbiology	10	1	-	1983	-
85	伤寒沙门氏菌引起的院内	comial infection caused by	郑迅.	Zheng Xun.	中华流行病学杂志	inese Journal of Epidemiolc	4	2	112	1983	sn.0254-6450.1983.02.124
86	伤寒沙门氏菌感染——附3	tion in Lanzhou City——at	刘建斗,李前俊.	Liu Jiandou,Li Qianjun.	中华传染病杂志	se Journal of Infectious Dis	1	3	147-150	1983	sn.1000-6680.1983.03.110
87	伤寒沙门氏菌感染66例报	yphimurium Infection66Ex	裔令,夏建锋,谭惠玉,张乃	Xia Jianfeng,Tan Huiyu,Zhar	中华传染病杂志	se Journal of Infectious Dis	1	3	151-153	1983	sn.1000-6680.1983.03.111
88	猪和鸡蛋带染沙门氏菌的	nella contamination in slau	庆万森,孙兆敏,康美.	wansen,Sun Zhaomin,Kang	中华预防医学杂志	se Journal of Preventive Me	17	5	269	1983	-
89	伤寒沙门氏菌脑膜炎治愈一	ry from Salmonella typhim	骆宗琼.	Luo Zongqiong.	中华儿科杂志	Chinese Journal of Pediatric	21	6	361-362	1983	sn.0578-1310.1983.06.132
90	穿刺液分离出一株猪伤寒	Salmonella typhisuis from	潘玉兰,殷峰.	Pan Yulan,Yin Feng.	中华流行病学杂志	inese Journal of Epidemiolc	4	4	210	1983	sn.0254-6450.1983.04.107
91	肠杆菌和沙门氏菌感染流行	athogenic E. coli and salmo	大麟,施云程,张乃本,邬慧	i Yuncheng,Zhang Naiben,	中华儿科杂志	Chinese Journal of Pediatric	21	3	145	1983	sn.0578-1310.1983.03.111
92	鼠伤寒沙门氏菌食物中毒	ases of Salmonella Typhim	河北省石家庄市卫生防疫	and Epidemic Prevention	中华流行病学杂志	inese Journal of Epidemiolc	4	3	191	1983	sn.0254-6450.1983.03.139
93	酸联合治疗2例顽固性鼠伤	herapy2A case report of re	沈耕荣,潘钰卿.	Shen Gengrong,Pan Yuqing	中华传染病杂志	se Journal of Infectious Dis	1	3	185-186	1983	sn.1000-6680.1983.03.126
94	利桑那沙门氏菌食物中毒	monella Food Poisoning In	珍,张永祥,王中民,姚正霖	ongxiang,Wang Zhongmin,	中华预防医学杂志	se Journal of Preventive Me	17	6	352-354	1983	-
95	食物型伤寒爆发流行的调	t on an outbreak of food-b	甘肃省高台县卫生防疫站	and Epidemic Prevention St	中华流行病学杂志	inese Journal of Epidemiolc	4	2	108-109	1983	sn.0254-6450.1983.02.121
96	婴儿室鼠伤寒暴发流行的调	on typhoid fever outbrea	苏静君;蒋国伟;陈鹏;	gjun;Jiang Guowei;Chen	贵州医药	Guizhou Medicine	-	1	2	1983	-
97	鼠伤寒沙门氏菌败血症一	onatal sepsis due to Salmo	刘玉林.	Liu Yulin.	铁道医学	Railway Medicine	-	3	143	1984	-
98	区沙门氏菌株对氯霉素耐	trains to chloramphenicol i	李明法,郭符则,李向荣.	Mingfa,Guo Fuze,Li Xiangro	中华传染病杂志	se Journal of Infectious Dis	2	2	136	1984	-
99	伤寒沙门氏菌脑膜炎一例	t of Salmonella typhimuriu	侣安,周和祥,张一平,许克	Zhou Hexiang,Zhang Yiping	中华传染病杂志	se Journal of Infectious Dis	2	4	287251	1984	-
100	沙门氏菌感染37例临床分	infection37Case clinical ar	梦湘 沈松柏 狄兆玉 徐美	xiangShen SongbaiDi Zhao	中华儿科杂志	Chinese Journal of Pediatric	22	6	343	1984	-
101	腹泻细菌性病原学及某些流	ome epidemiological chara	宗林,郭则宇,宋桂芝,孙立	en Zonglin,Guo Zeyu,Song	中华流行病学杂志	inese Journal of Epidemiolc	5	1	47-49	1984	sn.0254-6450.1984.01.128
102	起纽波特沙门氏菌食物中	salmonella Newport food	江西省上高县防疫站检验科	mic Prevention Station, Sha	中华流行病学杂志	inese Journal of Epidemiolc	5	1	8	1984	sn.0254-6450.1984.01.104
103	州地区沙门氏菌菌型分布	stribution of Salmonella st	,韩惠娟,郦馨若,杨佩江,	n Huijuan,Li Xinruo,Yang P	中华流行病学杂志	inese Journal of Epidemiolc	5	3	160-163	1984	sn.0254-6450.1984.03.117
104	鼠伤寒沙门氏菌鉴定及药	susceptibility test of Salmo	李国珍,胡文彬.	Li Guozhen,Hu Wenbin.	中华预防医学杂志	se Journal of Preventive Me	18	3	150	1984	-
105	78～80年沙门氏菌菌型鉴	tification and distribution	型调查科研组,卫生部成	esearch Group,Chengdu In	中华流行病学杂志	inese Journal of Epidemiolc	5	1	4	1984	sn.0254-6450.1984.01.102
106	门氏菌引起院内感染的细	nosocomial infections caus	李广恩,苏胜久,杨凡格.	uangen,Su Shengjiu,Yang Fa	中华流行病学杂志	inese Journal of Epidemiolc	5	3	175	1984	sn.0254-6450.1984.03.127
107	菌感染311例流行病学及临	n311Case epidemiology a	姜素椿 余平 黄玉兰.	ang SuchunYu PingMagnol	中华儿科杂志	Chinese Journal of Pediatric	22	3	129	1984	-
108	一起伤寒病爆发流行的调	ysis of an outbreak of typh	安徽省六安地区卫生防疫	and Epidemic Prevention,	中华流行病学杂志	inese Journal of Epidemiolc	5	3	157-159	1984	sn.0254-6450.1984.03.116
109	氏菌感染临床分析及防治	yphimurium infection in in	储宗瀛 综合.	hu Zongyingcomprehensiv	中华儿科杂志	Chinese Journal of Pediatric	22	3	136	1984	-

110	猪带沙门氏菌的调查	estigation of Salmonella in	刘民庆.	Liu Minqing.	中华流行病学杂志	inese Journal of Epidemiolo	5	3	164-166	1984	sn.0254-6450.1984.03.119
111	门氏菌院内交叉感染的流行	f nosocomial cross-infectio	区人民医院,山东省德州地	ce,Shandong Province Dezh	中华流行病学杂志	inese Journal of Epidemiolo	5	3	154-156	1984	sn.0254-6450.1984.03.114
112	暴发流行624例临床分析(	break624Case clinical ar	贾荣盛,董春红	Rongsheng,Dong Chunh	山东医药	Shandong Medicine	-	7	3	1984	-
113	卢庄村伤寒暴发流行的调	eak of typhoid fever in Lu	;扬龙献;赵九现;曲春叶;	ngxian;Zhao Jiuxian;Qu	河南预防医学杂志	Journal of Preventive M	-	3	21-24	1984	-
114	冬季水型伤寒暴发流行	n of a winter waterborne	丁世昌;鲍明荣;周天仇;	hang;Bao Mingrong;Zho	人民军医	people's military medicine	-	2	29-30	1984	-
115	流行的疫水中分离出几株	a isolated from epidemic v	王广和.	Wang Guanghe.	微生物学通报	Bulletin of Microbiology	12	3	-	1985	-
116	门氏菌食物中毒(附348例	oisoning(attached348Comp	宋路明.	Song Luming.	兰后卫生	Hygiene after orchid	-	4	332	1985	-
117	儿鼠伤寒沙门氏菌感染17	lla Typhimurium Infection1	赛珠,倪一尹,葛瑞祥,辛维	izhu,Ni Yiyin,Ge Ruixiang,X	中华传染病杂志	se Journal of Infectious Dis	3	4	249-251	1985	-
118	省泰安地区沙门氏菌菌型	onella strains in Tai'an, Sha	刘建华,赵建华.	Liu Jianhua,Zhao Jianhua.	中华预防医学杂志	se Journal of Preventive Me	19	6	377	1985	-
119	性腹泻患者的病原菌分析	eria and investigation of dr	胡超文,姬云,张为民,邱勤	owen,Ji Yun,Zhang Weimin,	中华流行病学杂志	inese Journal of Epidemiolo	6	6	336-338	1985	sn.0254-6450.1985.06.106
120	门氏菌胃肠炎医院内爆发	comial outbreak of Salmon	祖述宪,文师吾.	Zu Shuxian,Wen Shiwu.	中华流行病学杂志	inese Journal of Epidemiolo	6	4	219-222	1985	sn.0254-6450.1985.04.119
121	厂和禽类加工厂沙门氏菌	n in meat processing plant	项莉,李一军.	Xiang Li,Li Yijun.	中华预防医学杂志	se Journal of Preventive Me	19	2	88-89	1985	-
122	非伤寒沙门氏菌感染24例	almonella infectiontwenty	,余声华,欧阳玲,宗乐伟,	nghua,Ouyang Ling,Zong Le	中华儿科杂志	Chinese Journal of Pediatric	23	1	15-17	1985	sn.0578-1310.1985.01.109
123	型伤寒暴发的流行病学调	n report on an outbreak	黄俊俏;林永健;罗平金;韦	Lin Yongjian;Luo Pingjin;	右江医学	Youjiang Medicine	-	1	1-4	1985	-
124	氏菌对抗菌药物的体外敏	eptibility of Salmonella to a	金永寿.	Kim Yong-soo.	湖北医药导报	Hubei Medical Herald	-	3	18	1986	-
125	伤寒沙门氏菌败血症30例	emia in Infants and Young	王振营,徐恩洪.	Wang Zhenying,Xu Enhong	中华传染病杂志	se Journal of Infectious Dis	4	3	165-166	1986	-
126	生沙门氏菌中毒的大爆发	ilk-borne salmonella poiso	刘凤洁.	Liu Fengjie.	畜禽疾病	vestock and poultry disease	-	5	11	1986	-
127	对鼠伤寒沙门氏菌体外抗	ects of several antibacteria	德,顾亚明,张梅芳,刘裕	,Gu Yaming,Zhang Meifang	中华传染病杂志	se Journal of Infectious Dis	4	4	205-208，插页	1986	-
128	发鼠伤寒沙门氏菌感染二	ns complicated by Salmon	王仁昌.	Wang Renchang.	兰后卫生	Hygiene after orchid	-	4	90	1986	-
129	菌肠炎并发休克急性肾功	urium enteritis complicat	昶,贾氢,阿哈特,王云,何	ng,Jia Hydro,Ahat,Wang Yu	中华传染病杂志	se Journal of Infectious Dis	4	4	243	1986	-
130	炎沙门氏菌感染误诊2例报	lmonella Enteritidis infecti	,邢士儒,严小兴,刘卫林,	Shiru,Yan Xiaoxing,Liu Wei	中华传染病杂志	se Journal of Infectious Dis	4	1	57-58	1986	-
131	鼠伤寒沙门氏菌的药物敏	st of Salmonella typhimuri	朱庆义,黄元桐.	Zhu Qingyi,Huang Yuantong	中华流行病学杂志	inese Journal of Epidemiolo	7	1	25	1986	sn.0254-6450.1986.01.117
132	敦型，脑膜炎一例及其	case of meningitis and its	林怀彦.	Lin Huaiyan.	中华医学检验杂志	urnal of Medical Laborator	9	3	181	1986	-
133	沙门氏菌引起的败血症	Salmonella Stanleyville and	梅杰.	major.	中华医学检验杂志	urnal of Medical Laborator	9	4	208	1986	-
134	液便中检出一株纽波特	Newport was detected in t	王志敏,钱秀荣.	Wang Zhimin,Qian Xiurong	中华医学检验杂志	urnal of Medical Laborator	9	3	149	1986	-
135	沙门氏菌感染爆发流行	on an outbreak of Salmon	幼保健院,南昌市卫生防疫	pital,Nanchang Health and	中华流行病学杂志	inese Journal of Epidemiolo	7	4	221	1986	sn.0254-6450.1986.04.116
136	儿德尔卑沙门氏菌败血症	lmonella Delphi Septicemi	湘 *吴民忠 *王野坪 **尚	*Wu Minzhong *wangyepi	中华儿科杂志	Chinese Journal of Pediatric	24	1	47	1986	-
137	科一次鼠伤寒暴发流行调	hoid fever outbreak in th	仪贞;王彦厚;郑钧;郭广	Wang Yanhou;Zheng Jun	临沂医专学报	rnal of Linyi Medical Coll	-	Z1	61-63	1986	-
138	伤寒沙门氏菌属感染75例	Typhimurium Infection75	江瑞峰,鲁真焕,王淑春.	ifeng,Noh Jin Hwan,Wang S	新生儿科杂志	Journal of Neonatology	2	2	54	1987	-
139	菌食物中毒的初步调查—	nella food poisoning in sic	余绍珍.	Yu Shaozhen.	中华传染病杂志	se Journal of Infectious Dis	5	4	244	1987	-
140	斯坦利沙门氏菌感染20例	infection in neonates20Ca	李影,穆伟晶.	Li Ying,Mu Weijing.	新生儿科杂志	Journal of Neonatology	2	2	55,62	1987	-
141	伤寒沙门氏菌引起食物中	f food poisoning caused by	殷华,谭秀华,魏淑荣,况兴	an Xiuhua,Wei Shurong,Ku	中国农村卫生事业管理	na's rural health managem	-	9	28-	1987	-
142	河兰州段水中沙门氏菌的	the Water of the Lanzhou	孙荣高.	Sun Ronggao.	微生物学通报	Bulletin of Microbiology	14	5	-	1987	-
143	寒沙门氏菌医院内感染48	rium Nosocomial Infection	张松林.	Zhang Songlin.	新乡医学院学报	nal of Xinxiang Medical Co	-	4	-	1987	-
144	路局地区204株沙门氏菌	Bureau area204Salmonella	孟瑞芳.	Meng Ruifang.	铁道医学	Railway Medicine	-	5	303	1987	-
145	德尔俾沙门氏菌食物中毒	a case of Salmonella delp	刘凤鸣,李增海.	Liu Fengming,Li Zenghai.	中华预防医学杂志	se Journal of Preventive Me	21	4	253-254	1987	-
146	沙门氏菌败血症（附二	by Salmonella sepsis (with	赵春安.	Zhao Chunan.	中华整形烧伤外科杂志	rnal of Plastic Surgery and	3	4	281	1987	sn.1000-7806.1987.04.120

147	鼠伤寒沙门氏菌败血症	urns complicated by Salmo	王仁昌,崔光辉.	Yurenchang,Cui Guanghui.	中华整形烧伤外科杂志	nal of Plastic Surgery and	3	4	250	1987	sn.1000-7806.1987.04.105
148	阿哥纳沙门氏菌肺炎一例	of Salmonella agona pneu	徐美荣,吴民忠.	Xu Meirong,Wu Minzhong.	中华儿科杂志	Chinese Journal of Pediatric	25	1	42	1987	sn.0578-1310.1987.01.134
149	腹泻患者粪便检出旺兹沃	was detected in the stool	铁甫,邵义圭,胡锦元,顾沫	,Shao Yigui,Hu Jinyuan,Gu	中华医学检验杂志	urnal of Medical Laborator	10	3	155	1987	-
150	沙门氏菌引起肺炎并发	icated by empyema caused	王新桃,孟庆光.	ang Xintao,Meng Qingguan	中华医学检验杂志	urnal of Medical Laborator	10	3	153	1987	-
151	脑脊液中检出一株纽波特	port was detected in the c	张纪领,李卫军.	Zhang Jiling,Li Weijun.	中华医学检验杂志	urnal of Medical Laborator	10	4	204	1987	-
152	的旺兹沃思沙门氏菌感	case of Salmonella infectio	,邵义圭,胡锦元,钟海明,	ui,Hu Jinyuan,Zhong Haimi	中华流行病学杂志	inese Journal of Epidemiol	8	1	8	1987	sn.0254-6450.1987.01.104
153	冻猪肉沙门氏菌带菌调查	on salmonella infection in	,唐蕊妍,张丽蓉,夏铁恒,	iyan,Zhang Lirong,Xia Tiehe	中华流行病学杂志	inese Journal of Epidemiol	8	4	196	1987	sn.0254-6450.1987.04.102
154	沙门氏菌感染100例临床	n in newborns100Clinical	,魏书珍,赵复东,邹巧云,	Shuzhen,Zhao Fudong,Zou	中华儿科杂志	Chinese Journal of Pediatric	25	1	9	1987	sn.0578-1310.1987.01.107
155	病房用品检出鼠伤寒沙门	imurium detected in hospit	郑国雄,张玉英,贾勇.	Guoxiong,Zhang Yuying,Jia	中华预防医学杂志	e Journal of Preventive Me	21	4	245	1987	-
156	伤寒暴发所涉及的经济费	mic costs involved in a t	Shandera WX;陈顺弟;	handera WX;Chen Shund	国外医学(卫生经济分册)	edicine(Health Economic	-	4	35-36+32	1987	-
157	鼠型斑疹伤寒暴发流行	tigation of an outbreak o	东渊民,杨兰萍,罗银,陶其	in,Yang Lanping,Luo Yin	中国人兽共患病杂志	hinese Journal of Zoonos	-	6	34-36	1987	-
158	一起伤寒暴发的流行病	gation of a typhoid fever	;潘志明;曾财娣;刘树国;	himing;Zeng Caidi;Liu Sh	广州医药	Guangzhou Medicine	-	6	2-4+6	1987	-
159	伤寒暴发流行临床特征的	al characteristics of an o	顾清礼;	Gu Qingli;	宁夏医学院学报	nal of Ningxia Medical Co	-	2	28+30	1987	-
160	水型伤寒暴发流行的调查	rt on an outbreak of wat	杨劼;杨金龙;米芳苏;吕梅;	Yang Jinlong;Mi Fang S	河南预防医学杂志	Journal of Preventive M	-	4	34-36	1987	-
161	一九八六年伤寒暴发流	oid fever outbreak in Na	杨聚宝;	Yang Jubao;	河南预防医学杂志	Journal of Preventive M	-	4	37-39+43	1987	-
162	一起水型肠伤寒暴发流行	reak of water-type enteri	维民;付卓杰;吴凤义;李志	min;Fu Zhuojie;Wu Feng	黑龙江医药科学	ilongjiang Medical Scien	-	2	116-118	1987	-
163	沙门氏菌感染新生儿11例	infection in newborns11E	书明,郭桂荣,常东,许书	g,Guo Guirong,Changdong	新生儿科杂志	Journal of Neonatology	3	5	203-204	1988	-
164	鼠伤寒沙门氏菌感染(附19	yphimurium Infection(attac	梁广信.	Liang Guangxin.	新乡医学院学报	nal of Xinxiang Medical Co	-	2	-	1988	-
165	C1群婴儿沙门氏菌肠炎30	onella enteritis in infants30	马敏珊,张坤生,张业权.	han,Zhang Kunsheng,Zhang	新生儿科杂志	Journal of Neonatology	3	5	201-202	1988	-
166	门氏菌感染致急性胃肠炎	troenteritis caused by Salm	刘婕.	Liu Jie.	铁道医学	Railway Medicine	-	2	89	1988	-
167	鼠伤寒沙门氏菌感染的实	n of neonatal Salmonella t	周惠平,戴淑凤,宋琳琳.	Huiping,Dai Shufeng,Song	新生儿科杂志	Journal of Neonatology	3	5	225-227	1988	-
168	儿鼠伤寒沙门氏菌感染临	f neonatal Salmonella typh	艳缇,钱培德,贾中芬,郎振	Qian Peide,Jia Zhongfen,La	新生儿科杂志	Journal of Neonatology	3	2	53-54	1988	-
169	鼠伤寒沙门氏菌食物中毒	ase of Salmonella Typhimu	普明,高小宁,胡大荣,余	,Gao Xiaoning,Hu Darong,	中国农村卫生事业管理	na's rural health managem	-	7	28-29	1988	-
170	2年来鼠伤寒沙门氏菌病	prevalence of Salmonellosis	王锦德.	Wang Jinde.	兰后卫生	Hygiene after orchid	-	2	9	1988	-
171	研究-从一次鼠伤寒暴发	engthened-Let's start with	李力,于清,于虹.	Li Li,Yu Qing,Yu Hong.	中国卫生经济	China's health economy	-	11	34-36,1	1988	-
172	重症鼠伤寒6例临床分析	typhoid fever6Case clinical	田秀慧,鄢裕光.	Tian Xiuhui,Yan Yuguang.	中华传染病杂志	se Journal of Infectious Dis	6	1	59	1988	-
173	741株沙门氏菌的菌型调查	n of bacterial types of Sal	沙门氏菌菌型调查协作组	Type Investigation Collabo	中华预防医学杂志	e Journal of Preventive Me	22	3	186-187	1988	-
174	沙门氏菌株对14种抗生素	Luodian area14antibiotic	吴光财,毛成华.	u Guangcai,Mao Chenghu	中华流行病学杂志	inese Journal of Epidemiol	9	4	256	1988	sn.0254-6450.1988.04.131
175	门氏菌莫斯科血清型致菌	aused by Salmonella Enteri	贾昉,王顺维.	Jia Fang,Wang Shunwei.	中华医学检验杂志	urnal of Medical Laborator	11	2	101	1988	-
176	性感染性腹泻的临床与细	re analysis of acute infecti	郑德联,徐肇玥,戴自英.	g Delian,Xu Zhaoyue,Dai Zi	中华老年医学杂志	ese Journal of Geriatric Me	7	4	196-199	1988	sn.0254-9026.1988.04.102
177	伤寒沙门氏菌属的医院内	ections with Salmonella typ	李英菊,许芝兰,刘玉江.	Yingju,Xu Zhilan,Liu Yujian	中华医院管理杂志	se Hospital Management J	4	6	355-356	1988	sn.1000-6672.1988.06.116
178	门氏菌败血症误诊为大叶	murium septicemia misdiag	王志刚.	Wang Zhigang.	中华内科杂志	ese Journal of Internal Me	27	8	491	1988	-
179	伤寒沙门氏菌脑膜炎五例	ses of Salmonella typhimu	马堃.	Ma Kun.	中华儿科杂志	Chinese Journal of Pediatric	26	6	368-369	1988	sn.0578-1310.1988.06.140
180	培养检出沙门氏菌梦得维	idia serotype detected in b	梁卓智,郭秀筠.	eung Cheuk Chi,Guo Xiujun	中华医学检验杂志	urnal of Medical Laborator	11	5	290	1988	-
181	污水中分离到姆班达卡沙	monella Mbandaka from h	钱小毛,陈永金.	Qian Xiaomao,Chen Yongjin	中华预防医学杂志	e Journal of Preventive Me	22	3	172	1988	-
182	腹泻暴发流行中分离出41	reak of neonatal diarrhea	占水,岳云升,姚家莹,邹伟	ui,Yue Yunsheng,Yao Jiayin	中华医学杂志	Chinese Medical Journal	68	6	341	1988	-
183	寒沙门氏菌引起的医院内	-infection caused by Salm	文,田珍广,张绪团,郭桂荣,	enguang,Zhang Xutuan,Gu	中华儿科杂志	Chinese Journal of Pediatric	26	6	331-332,插9	1988	sn.0578-1310.1988.06.108

184	氏菌引起的败血症及菌株	nleyville and its isolation a	王考风.	Wang Kaofeng.	中华医学检验杂志	urnal of Medical Laborator	11	1	43	1988	-
185	蒙得维多沙门氏菌肠炎45	is in neonates Montevideo	殷之琳,张克智.	Yin Zhilin,Zhang Kezhi.	中华流行病学杂志	inese Journal of Epidemiolo	9	4	252	1988	sn.0254-6450.1988.04.126
186	鼠伤寒沙门氏菌药物敏感	lity testing of Salmonella t	裴秀藏,张敏,刘爱芬,王燕	ang,Zhang Min,Liu Aifen,W	中华预防医学杂志	se Journal of Preventive Me	22	1	47	1988	-
187	沙门氏菌引起的败血症及	Salmonella reading and its	秀英,王云英,李健开,陆天	,Wang Yunying,Li Jiankai,L	中华医学检验杂志	urnal of Medical Laborator	11	5	293	1988	-
188	氏菌引起新生儿腹泻爆发	outbreak of neonatal diarr	K,岳云升,姚家莹,邹伟民,文	Yunsheng,Yao Jiaying,Zou V	中华医学检验杂志	urnal of Medical Laborator	11	6	356-357	1988	-
189	研究——从一次鼠伤寒暴发	gthened——Let's start w	李力;于清;于虹;	Li Li;Yu Qing;Yu Hong;	中国卫生经济	China's health economy	-	11	34-36+1	1988	-
190	以学龄儿童为主的伤寒暴发	oid fever mainly affected	郭瑞元;	Guo Ruiyuan;	福建医药杂志	Fujian Medical Journal	-	4	54	1988	-
191	流行性斑疹伤寒暴发流行的	into an outbreak of epic	,陈民新,吴桂莲,赵国龙,	inxin,Wu Guilian,Zhao G	第四军医大学学报	f Fourth Military Medical	-	3	205-206	1988	-
192	一次水型伤寒暴发的调查	an outbreak of waterbo	家炽;王颖晰;白志洁;张荣	Vang Yingxi;Bai Zhijie;Zh	内蒙古医学杂志	er Mongolia Medical Jou	-	1	36-37	1988	-
193	都柏林沙门菌败血症1例	Salmonella Septicemia1ex	张云斌,周业宽,牟淑琴.	Yunbin,Zhou Yekuan,Mou S	人民军医	people's military medicine	-	11	56	1989	-
194	门氏菌败血症的临床特点	rium sepsis in infants and y	阮大宝.	Ruan Dabao.	天津医学院学报	rnal of Tianjin Medical Coll	13	1	62-65	1989	-
195	检出沙门氏菌等多种肠道	inal pathogenic bacteria su	秦贞奎.	Qin Zhenkui.	动物检疫	animal quarantine	-	1	10-12	1989	-
196	寒沙门氏菌流行菌株的药	ysis of prevalent strains of r	仁,马琳,苟普仁,李隆昌,肖	a Lin,Gou Puren,Li Longcha	白求恩医科大学学报	al of Bethune Medical Univ	-	4	354-356	1989	-
197	业人员中肠道带菌及乙肝	estinal bacteria and hepatit	陈西员.	Chen Xiyuan.	疾病监测	disease surveillance	4	6	88-89	1989	-
198	氏菌院内交叉感染的流行	f nosocomial cross-infectio	周崇连,陈红莲.	ou Chonglian,Chen Honglia	中国疾病监测	China disease surveillance	4	5	-	1989	-
199	中沙门氏菌污染的主要因素	sures for Salmonella conta	吴炳章.	Wu Bingzhang.	肉类研究	meat research	-	1	40-41	1989	-
200	寒沙门氏菌肠炎致肠穿孔2	d by Salmonella typhimuriu	孙黎明,张宗华,史翠英.	ming,Zhang Zonghua,Shi Cu	青海医学院学报	rnal of Qinghai Medical Col	-	2	116-117	1989	-
201	寒沙门氏菌的药物敏感试	susceptibility test of Salmo	熊敏.	Xiong Min.	贵阳医学院学报	nal of Guiyang Medical Col	-	3	227-228	1989	-
202	丹沙门菌食物中毒的调查	port on Salmonella bulidan	郭诚.	Guo Cheng.	人民军医	people's military medicine	-	9	14-15	1989	-
203	暴发型鼠伤寒沙门菌感染	natal fulminant Salmonella	向辉.	Xiang Hui.	湖南医学	Hunan Medicine	-	4	201-202	1989	-
204	临床初诊为急性菌痢的病原	a case initially diagnosed	范家珍,戴立人,李少华.	n Jiazhen,Dai Liren,Li Shaoh	武汉医学杂志	Wuhan Medical Journal	-	Z2	81-82	1989	-
205	门氏菌同时引起院内交叉	infection caused by two ki	敬,王东济,王会清,徐京	s,Wang Dongji,Wang Huiq	医学理论与实践	Medical theory and practice	-	4	23	1989	-
206	筵席造成食物型伤寒暴发	break of food-borne typhoi	冯继良,贾惠英.	Feng Jiliang,Jia Huiying.	中国食品卫生杂志	hinese Food Hygiene Journ	-	2	64	1989	-
207	门氏菌是医院交叉感染的主	the main pathogen causing	俊,李东阳,王云,周文华,王	ngyang,Wang Yun,Zhou W	河南预防医学杂志	n Journal of Preventive Me	-	3	67-68	1989	-
208	肉引起食物中毒的细菌检验	acteria causing food poiso	许龙福.	Xu Longfu.	肉品卫生	Meat hygiene	-	12	-	1989	-
209	例小儿夏季腹泻病原学分	he etiology of summer dia	培芳,连玉英,吕秀文,吕文	ng,Lian Yuying,Lu Xiuwen,L	青岛大学医学院学报	f Qingdao University Medic	-	4	-	1989	-
210	门氏菌院内交叉感染的流行	f nosocomial cross-infectio	崇连,陈红莲,伍运生,孙莲	Chen Honglian,Wu Yunshe	中国疾病监测	China disease surveillance	4	5	74-76	1989	-
211	里利氏沙门氏菌脑膜炎误诊	gnosis of neonatal Salmon	朱菊芳,李荷斯,刘玲.	hu Jufang,Lee Hoss,Liu Ling	蚌埠医药	Bengbu Medicine	-	1	75	1989	-
212	区沙门氏菌药变迁及临床	cal efficacy of Salmonella d	杜继昭,王其南.	Du Jizhao,Wang Qinan.	重庆医科大学学报	l of Chongqing Medical Uni	14	1	20-33	1989	-
213	犊牛致死性腹泻的实验室	gnosis of fatal diarrhea in r	葛君慧,左玉婷,靳诚.	Junhui,Zuo Yuting,Jin Chen	甘肃畜牧兽医	al Husbandry and Veterina	-	5	13-14	1989	-
214	暴发鼠伤寒沙门氏菌肠炎1	almonella Typhimurium En	李祖荣,梁绮霞,应宽.	rong,Liang Qixia,should be	江西医学院学报	rnal of Jiangxi Medical Coll	-	3	67-69	1989	-
215	寒沙门氏菌82株药敏试验	urium82Strain drug suscep	王玉山,刘文军.	Wang Yushan,Liu Wenjun.	临沂医专学报	urnal of Linyi Medical Colle	-	2	170-171	1989	-
216	新生儿感染性腹泻原因分析	causes of neonatal infectio	俞文娟,张贵江,周文杰.	njuan,Zhang Guijiang,Zhou	南通医学院学报	nal of Nantong Medical Co	9	4	310-311	1989	-
217	伤寒沙门氏菌食物中毒的	e of food poisoning caused	陈亢川,邓家焕.	hen Kangchuan,Tang Jiahua	中华流行病学杂志	inese Journal of Epidemiolo	10	1	34-36	1989	-
218	购销环节中沙门氏菌污染	amination in the purchase	高淑华,葛梅.	Gao Shuhua,Kamei.	肉品卫生	Meat hygiene	-	8	-	1989	-
219	伤寒沙门氏菌对12种抗生	infants and young children	萍,林红,袁雄伟,王莹,检验	Lin Hong,Yuan Hongwei,W	中级医刊	termediate medical journa	-	12	22-23	1989	-
220	菌用荧光抗体法与常规法	dy method and convention	,刘淑梅,李钢锋,杨超英,	umei,Li Gangfeng,Yang Cha	动物检疫	animal quarantine	-	2	15-16	1989	-

221	猪白条肉的细菌学检验	al inspection report on por	张美兰,岳玉娥.	Zhang Meilan,Yue Yu'e.	牧业工程高等专科学校	zhou Animal Husbandry En	-	Z1	44-45	1989	-
222	某奶牛场一起沙门氏菌病	salmonellosis in a dairy farr	董国雄,李俊宝,孙茂芝.	Guoxiong,Li Junbao,Sun M	中国畜禽传染病	stock and poultry infectiou	-	Z1	23-24	1989	-
223	国小儿感染性腹泻病的现	infectious diarrhea in child	方鹤松.	Fang Hesong.	实用儿科杂志	ournal of Practical Pediatri	-	2	89-90	1989	-
224	门氏菌院内交叉感染的流	f nosocomial cross-infectio	崇连,伍运生,陈红莲,孙莲	Wu Yunsheng,Chen Hongli	衡阳医学院学报	al of Hengyang Medical Co	-	4	347-349	1989	-
225	的哈达尔沙门氏菌引起的	od poisoning caused by Sa	夷,陈霞,梁进兴,吴采菲,李	en Xia,Liang Jinxing,Wu Ca	中国人兽共患病杂志	Chinese Journal of Zoonose	-	2	36-38	1989	-
226	镇西岸村伤寒水型暴发	typhoid fever outbreak in	云生,汪妙素,王忠意,袁	,Wang Miaosu,Wang Zhon	浙江疾病监测	hejiang disease surveillanc	-	S2	21-23	1989	-
227	场肉品沙门氏菌污染情	nella contamination of me	夷,陈毓玲,邓燕,徐昌志,	n Yuling,Deng Yan,Xu Chan	中国食品卫生杂志	hinese Food Hygiene Journ	-	4	18-19	1989	-
228	儿非伤寒沙门氏菌感染细	ysis of non-typhoid salmon	张宝昌.	Zhang Baochang.	天津医药	Tianjin Medicine	-	11	647-649	1989	-
229	卑沙门氏菌中毒38例调查	delphi poisoning38Case stu	赵清占,夏淑英,焦荣华.	ingzhan,Xia Shuying,Jiao Ro	医学理论与实践	Medical theory and practice	-	2	10-11	1989	-
230	地区死亡畜禽的病原分	ns from dead livestock and	隆华.	longhua.	贵州畜牧兽医	mal Husbandry and Veterin	-	2	1-3	1989	-
231	鸡沙门氏菌病病原分离与	fication of the pathogen of	文其乙,张如宽,朱雁.	n Qiyi,Zhang Rukuan,Zhu Y	中国家禽	Chinese poultry	-	1	30-31	1989	-
232	菌感染在新生儿病室的流	ium infection in the neona	艳芝,罗克娴,李国荣,魏秀	Luo Kexian,Li Guorong,Wei	河北医学院学报	urnal of Hebei Medical Colle	-	S1	38-39	1989	-
233	细菌性食物中毒	bacterial food poisoning	任立.	Ren Li.	中国公共卫生	Chinese public health	-	1	6-10	1989	-
234	鼠伤寒沙门氏菌感染22例	himurium Infectiontwenty	秉岳,汪惠芳,汤辅娣,王志	Wang Huifang,Tang Fudi,W	蚌埠医学院学报	rnal of Bengbu Medical Col	-	2	121-122	1989	-
235	鼠伤寒沙门氏菌的药敏试	ty testing of Salmonella ty	崇连,陈红莲,伍运生,孙莲	Chen Honglian,Wu Yunshe	衡阳医学院学报	al of Hengyang Medical Co	-	2	147-148	1989	-
236	出口分割猪肉生产的污	nination of export cut pork	黄万国,张俊勇.	uang Wanguo,Zhang Junyo	肉品卫生	Meat hygiene	-	1	-	1989	-
237	睢县城乡腹泻病原学的	arrhea in urban and rural a	钢建,陈振东,靳福成,吴建	uction,Chen Zhendong,Jin F	河南预防医学杂志	n Journal of Preventive Me	-	2	4-9	1989	-
238	膜炎血中分离出猪霍乱沙	lood in subacute bacterial	杜连荣.	Du Lianrong.	海军总医院院刊	rnal of Naval General Hosp	2	4	276-277	1989	-
239	鼠伤寒医院内感染31例临	n of infant typhoid fever31	石杰,SHANDONG.	Shi Jie,SHANDONG.	临沂医专学报	urnal of Linyi Medical Colle	-	1	37-39	1989	-
240	菌感染引起婴儿食物中毒	t food poisoning caused b	,谭丽梅,李锦光,杨芳娣,	n Limei,Li Jinguang,Yang Fa	中国食品卫生杂志	hinese Food Hygiene Journ	-	4	27-30	1989	-
241	市婴幼儿腹泻的病原菌	genic bacteria of infant di	裕,林红,梁成龙,唐振柱,	g,Liang Chenglong,Tang Zh	广西医学	Guangxi Medicine	-	3	158-159	1989	-
242	区猪胴体沙门氏菌检出率	Salmonella detection rates	赵显文.	Zhao Xianwen.	肉品卫生	Meat hygiene	-	8	-	1989	-
243	2,729例血培养及药敏分析	d culture and drug sensitiv	秀英,王云英,李健开,孙志	g,Wang Yunying,Li Jiankai,	青海医药杂志	Qinghai Medical Journal	-	5	42-44	1989	-
244	地区生活污水中沙门氏	ella in Domestic Sewage in	李杏爱,张平,陈健雄.	ngai,Zhang Ping,Chen Jianx	中国国境卫生检疫杂志	ntier Health and Quarantin	-	1	42-43	1989	-
245	寒沙门氏菌感染流行与控	e and control of Salmonella	嘉格,杨淑明,王陵玲,李明	Yang Shuming,Wang Lingli	中国公共卫生	Chinese public health	-	2	7-8,6	1989	-
246	乙沙门菌引起食物中毒	ak of food poisoning cause	徐金旗.	Xu Jinqi.	人民军医	people's military medicine	-	8	15-16	1989	-
247	服务从业人员29,549人沙	gbu City29,549Investigatio	赵和璧.	Zhao Hebi.	蚌埠医药	Bengbu Medicine	-	1	64-67	1989	-
248	寒沙门氏杆菌性心内膜炎	of Salmonella typhi endoc	杨立础,戴经华.	Yang Lichu,Dai Jinghua.	中华儿科杂志	Chinese Journal of Pediatric	27	2	101	1989	sn.0578-1310.1989.02.124
249	鼓县河水污染沙门氏菌调	ella contamination of river	尹子达.	Yin Zida.	中华预防医学杂志	se Journal of Preventive Me	23	3	177	1989	-
250	部渗出物中分离五株姆	nella Mbandaka from infa	吕力伟.	Lu Liwei.	中华医学检验杂志	urnal of Medical Laborator	12	4	244	1989	-
251	伤寒沙门氏菌颅内感染一	anial infection with Salmon	永贵,胡志宏,郭月珠,毕会	i,Hu Zhihong,Guo Yuezhu,	中华医学检验杂志	urnal of Medical Laborator	12	6	356	1989	-
252	起的颈部淋巴结化脓性	caused by Salmonella in a	卢连法,刘朋香.	Lu Lianfa,Liu Pengxiang.	中华医学检验杂志	urnal of Medical Laborator	12	3	185	1989	-
253	沙门氏菌引起婴幼儿腹	by Salmonella Shanftonbe	裴秀藏,刘爱芬,张敏,王燕	ang,Liu Aifen,Zhang Min,W	中华预防医学杂志	se Journal of Preventive Me	23	1	57	1989	-
254	毒分离出汤卜逊沙门氏菌	olated from a case of food	陆建荣.	Lu Jianrong.	中华医学检验杂志	urnal of Medical Laborator	12	5	291	1989	-
255	物中毒标本中检出圣保罗	Paul detected in a food po	马登辰,张青.	Ma Dengchen,Zhang Qing.	中华医学检验杂志	urnal of Medical Laborator	12	3	150	1989	-
256	炎血清型致耳后脓肿一	used by Salmonella Enterit	王定佳.	Wang Dingjia.	中华医学检验杂志	urnal of Medical Laborator	12	1	53	1989	-
257	腹泻大便中同时分离出4	ions and diarrheal stools si	张平美,杨淑元,吕力伟.	Pingmei,Yang Shuyuan,Lu	中华医学检验杂志	urnal of Medical Laborator	12	1	41	1989	-

258	饮食服务人员沙门氏菌属带	carriers among catering se	蚌埠市卫生防疫站,赵和	alth and Epidemic Preventi	中华预防医学杂志	se Journal of Preventive Me	23	3	182	1989	-
259	伤寒沙门氏菌食物中毒的讠	e of food poisoning caused	家焕,吴宗诚,陈延柱,郑智	ngcheng,Chen Yanzhu,Zhen	中华流行病学杂志	inese Journal of Epidemiolo	10	1	34-36	1989	sn.0254-6450.1989.01.113
260	染患者尿中检出一株阿纳	a Ana from the urine of a p	郭秀琴,赵萍,周国华.	Xiuqin,Zhao Ping,Zhou Guo	中华医学检验杂志	urnal of Medical Laborator	12	1	51	1989	-
261	沙门氏菌引起新生儿败血症	natal sepsis caused by Saln	王新桃,邵白雪,李雪波.	g Xintao,Shao Baixue,Li Xu	中华医学检验杂志	urnal of Medical Laborator	12	4	248	1989	-
262	中三例鼠伤寒沙门氏菌分离	solation of Salmonella typh	怀玲,史金宝,张福礼,刘俊	ling,Shi Jinbao,Zhang Fuli,L	中华医学检验杂志	urnal of Medical Laborator	12	2	123	1989	-
263	门氏菌属C肠炎46例临床分	spp.Centeritis46Case clini	王荣之,莫剑青,刘金贵.	g Rongzhi,Mo Jianqing,Liu J	中华传染病杂志	se Journal of Infectious Dis	7	1	59	1989	sn.1000-6680.1989.01.126
264	菌引起食物中毒一例及菌株	lmonella St. Paul and repor	吴庆周.	Wu Qingzhou.	中华医学检验杂志	urnal of Medical Laborator	12	2	103	1989	-
265	一起中学伤寒暴发流行调	hoid fever outbreak in m	蒋兴发;	Jiang Xingfa;	学校卫生	school health	-	5	28-29	1989	-
266	田师范一起水型伤寒暴发流	typhoid fever occurs in C	;马风生;马龙宝;赵重生;江	ng;Malong Bao;Zhao Ch	学校卫生	school health	-	5	29	1989	-
267	伤寒暴发流行的经济效益	ic benefits of waterborne	武秀玉;阚冬梅;	Wu Xiuyu;Kan Dongmei;	中国卫生经济	China's health economy	-	10	32-34	1989	-
268	起水源性伤寒暴发的调查报	on an outbreak of water	王玉霞	Wang Yuxia	环境与健康杂志	nal of Environment and H	-	2	38	1989	ki.1001-5914.1989.02.028
269	起伤寒暴发流行的调查报	report on an outbreak of	王复国;	Wang Fuguo;	铁道医学	Railway Medicine	-	2	98	1989	-
270	沙门氏菌感染暴发流行(附	tion outbreak in nursery(a	丁文静,王玉,朱玲.	g Wenjing,Wang Yu,Zhu Li	安徽医学	Anhui Medicine	-	6	32-33	1990	-
271	寒沙门氏菌病医院内感染	socomial infections of saln	史金宝,张福礼.	Shi Jinbao,Zhang Fuli.	中华流行病学杂志	inese Journal of Epidemiolo	11	5	284-287	1990	-
272	生肉引起沙门氏菌中毒的报	nella poisoning caused by	邓青莲.	Deng Qinglian.	云南畜牧兽医	nal Husbandry and Veterina	-	4	35	1990	-
273	鼠伤寒沙门氏菌感染点状	utbreak of Salmonella typh	楚建华,何维霞.	Chu Jianhua,He Weixia.	中国疾病监测	China disease surveillance	5	1	-	1990	-
274	鼠伤寒沙门氏菌感染40例临	a Typhimurium Infection40	-	-	宜春医药	Yichun Medicine	-	20	15-16	1990	-
275	年上虞县健康人群中沙门氏	nella strains among health	贾杰.	Jia Jie.	浙江预防医学与疾病监测	entive Medicine and Diseas	-	4	30-31	1990	-
276	寒沙门氏菌的生物学性状	stics and drug resistance of	惠云,袁祖行,干梦九,陆红	uan Zuxing,Qian Mengjiu,Lu	浙江医科大学学报	al of Zhejiang Medical Univ	-	1	18-20	1990	-
277	伤寒沙门氏菌感染41例疗	m infection in children41Ca	王军仓,傅满霖,王淑惠.	Juncang,Fu Manlin,Wang S	中医杂志	l of Traditional Chinese Me	-	9	33-34	1990	-
278	氏菌院内感染18例流行病学	ial infection18Case epiden	翟奇,陈茂余.	Zhai Qi,Chen Maoyu.	华西医讯	West China Medical News	-	3	261-262	1990	-
279	鼓县沙门氏菌菌型分布调	stribution of Salmonella str	尹子达.	Yin Zida.	中国人兽共患病杂志	Chinese Journal of Zoonose	-	2	48-50	1990	-
280	霉素伤寒沙门氏菌生物学	ics of chloramphenicol-resi	村全美,王瑞华,杨昌伦,刘大	mei,Wang Ruihua,Yang Ch	贵州医药	Guizhou Medicine	-	2	115-117	1990	-
281	伤寒沙门氏菌感染21例临	infection in childrentwenty	陈刚.	Chen Gang.	江西医药	Jiangxi Medicine	-	6	345-346	1990	-
282	市肉类沙门氏菌污染动态调	n Salmonella contaminatio	马彬,孙立琴.	Ma Bin,Sun Liqin.	新疆畜牧业	Xinjiang animal husbandry	-	3	29-30,32	1990	-
283	伤寒沙门氏菌食物中毒调	a case of Salmonella typhi	德伟,张留子,范钦敬,张春兰	hang Liuzi,Fan Qinjing,Zha	中国食品卫生杂志	hinese Food Hygiene Journ	-	2	62-63	1990	-
284	的亚沙门氏菌感染73例临	ction in Montevideo73Case	芳,黄佳滋,蒋道荣,王凌,许	uang Jiazi,Jiang Daorong,Wa	上海医学	Shanghai Medicine	-	7	407-409	1990	-
285	伤寒沙门氏菌分型及药敏结	drug susceptibility results	水,杨美云,斯志萍,卓平定,鲍	Meiyun,Si Zhiping,Zhuo Pin	浙江预防医学与疾病监测	entive Medicine and Diseas	-	6	7-8	1990	-
286	寒沙门氏菌感染400例临床	imurium Infection400Case	李前俊.	Li Qianjun.	甘肃医药	Gansu Medicine	9	1	24-25	1990	-
287	寒沙门氏菌感染并发症193	onella Typhimurium Infecti	王振营,王文联.	ang Zhenying,Wang Wenlia	医师进修杂志	ian Continuing Education J	-	11	12-13	1990	-
288	市场鲜猪肉污染沙门氏菌情	la contamination of fresh p	秀,尉亚范,米钧,魏建平,梁	Yafan,Mi Jun,Wei Jianping	动物检疫	animal quarantine	-	2	20-22	1990	-
289	起严重的沙门氏菌食物中	s case of salmonella food p	常天宜,蔡佩钦.	Chang Tianyi,Cai Peiqin.	新疆医学院学报	nal of Xinjiang Medical Col	13	4	260-261	1990	-
290	肉引起的43人汤卜逊沙门	eef43Salmonella Thompson	金献,吴建中.	Jin Xian,Wu Jianzhong.	中国食品卫生杂志	hinese Food Hygiene Journ	-	2	60-61	1990	-
291	生儿非伤寒沙门氏菌感染	of neonatal non-typhoid sa	赵亚平.	Zhao Yaping.	新生儿科杂志	Journal of Neonatology	5	5	207-208	1990	-
292	门氏菌感染在新生儿室的	onella typhimurium infecti	罗凤珍,王太梅.	Luo Fengzhen,Wang Taimei	新生儿科杂志	Journal of Neonatology	5	2	75-76,86	1990	-
293	沙门氏菌感染的诊断和治疗	a typhimurium infection in	雷崇辉,马伏龙.	Lei Chonghui,Ma Fulong.	新生儿科杂志	Journal of Neonatology	5	2	84-86	1990	-
294	儿室山夫登堡沙门氏菌肠	lla enteritis in obstetrics an	陆进,李庆麟,陈岩.	Lu Jin,Li Qinglin,Chen Yan.	新生儿科杂志	Journal of Neonatology	5	5	201-202	1990	-

295	儿鼠伤寒沙门氏菌脑膜炎	onella Typhimurium Menir	陈淑风,胡伟明.	Chen Shufeng,Hu Weiming	新生儿科杂志	Journal of Neonatology	5	5	235-236	1990	-
296	氏菌引起的慢性骨髓炎及	elitis caused by Salmonella	兰英,翟启武,马黎明,刘亚	ng,Zhai Qiwu,Ma Liming,Li	中华医学检验杂志	urnal of Medical Laborator	13	5	293	1990	-
297	沙门氏菌引起的新生儿	nplicated by sepsis caused	立宪,胡建华,关冰,李景	xian,Hu Jianhua,Guan Bing	中华预防医学杂志	se Journal of Preventive Me	24	4	256	1990	-
298	贡纳沙门氏菌食物中毒	case of Salmonella Agonna	陈建军,魏平.	Chen Jianjun,Wei Ping.	中华预防医学杂志	se Journal of Preventive Me	24	2	69	1990	-
299	粪便中检出塔克松尼沙门	emolyticus detected in the	褚国方.	Chu Guofang.	中华医学检验杂志	urnal of Medical Laborator	13	2	70	1990	-
300	1962～1989年沙门氏菌	1989Distribution of Salmon	简永新.	Jian Yongxin.	中华预防医学杂志	se Journal of Preventive Me	24	5	267	1990	-
301	寒沙门氏菌引起的成人	ng in an adult caused by Sa	荣西,张远炎,杨金玉,罗	xi,Zhang Yuanyan,Yang Jin	中华传染病杂志	se Journal of Infectious Dis	8	4	225	1990	sn.1000-6680.1990.04.110
302	门氏菌引起的胆囊炎及菌	lmonella Herrenii and isola	钱小毛.	Qian Xiaomao.	中华医学检验杂志	urnal of Medical Laborator	13	5	262	1990	-
303	内鼠伤寒沙门氏菌感染的	lmonella typhimurium infe	楚建华,何维霞.	Chu Jianhua,He Weixia.	中华预防医学杂志	se Journal of Preventive Me	24	4	237	1990	-
304	副伤寒沙门氏菌院内感	socomial Infection with Sa	荣,孙书明,常冬,许书言,彭	huming,Changdong,Xu Shu	中华传染病杂志	se Journal of Infectious Dis	8	2	118-119	1990	sn.1000-6680.1990.02.125
305	伤寒沙门氏菌感染的病原	alysis of Salmonella typhin	-	-	内蒙古医科大学学报	f Inner Mongolia Medical U	-	2	122	1990	-
306	寒沙门氏菌肠炎157例临	enteritis in children157Ex	张如弼,时淑宁,田月英.	g Rubi,Shi Shuning,Tian Yue	宁夏医科大学学报	al of Ningxia Medical Univ	-	3	-	1990	-
307	儿腹泻的病原学及临床	d clinical analysis of neona	祥,徐景蓁,倪安平,谢秀丽,	Jingzhen,Ni Anping,Xie Xiul	中华儿科杂志	Chinese Journal of Pediatric	28	6	338-340	1990	sn.0578-1310.1990.06.111
308	吃皮蛋中毒10例报告	n eating preserved eggs10E	郑炳雄.	Zheng Bingxiong.	广州医药	Guangzhou Medicine	-	4	37,40	1990	-
309	医院污水的肠道致病菌	hogenic bacteria in sewage	书,梅玲,钟晓英,周亚南,	Ling,Zhong Xiaoying,Zhou	环境与健康杂志	nal of Environment and He	-	3	135	1990	-
310	市食品行业从业人员健康	for employees in the food	兰荪,房玉华,金伟民,林	sun,Fang Yuhua,Jin Weimin	浙江预防医学与疾病监测	entive Medicine and Diseas	-	4	20	1990	-
311	例急性感染性腹泻患者的	on the etiology of patient	宋佩辉,黄元成,雷汉题,陈	uang Yuancheng,Lei Han's	武汉医学杂志	Wuhan Medical Journal	-	2	97-98	1990	-
312	自来水管破漏引起的伤寒	r outbreak caused by a bro	王昌信.	Wang Changxin.	浙江预防医学与疾病监测	entive Medicine and Diseas	-	4	22-23	1990	-
313	寒临床防治体会-附65例	treatment of murine typho	肖家惠.	Xiao Jiahui.	中国农村医学	Chinese rural medicine	-	7	33-34	1990	-
314	分割车间工用具中五种	enic bacteria in slaughterin	志辉,田光琼,曾襟碧,白廷	i,Tian Guangqiong,Zeng Jin	中国食品卫生杂志	hinese Food Hygiene Journ	-	1	63-64	1990	-
315	感染性腹泻病原组成及其	on pattern of infectious dia	,奉兆明,甘普祥,余珍英,	oming,Gan Puxiang,Yu Zhe	武汉医学杂志	Wuhan Medical Journal	-	4	213	1990	-
316	区急性腹泻患者病原学	gy of patients with acute	全忠,王世意,程维粹,俞忠	,Wang Shiyi,Cheng Weicui	浙江预防医学与疾病监测	entive Medicine and Diseas	-	4	15-16	1990	-
317	食物中毒162人的调查报告	oning162human investigat	,徐金旗,费宝双,王廷伟,	ei Baoshuang,Wang Tingw	人民军医	people's military medicine	-	6	10-11	1990	-
318	起水传伤寒暴发流行的调	an outbreak of waterbo	赵常喜,董有诚,李惠福	hangxi,Dong Youcheng,	环境与健康杂志	al of Environment and H	-	5	237-238	1990	ki.1001-5914.1990.05.019
319	市水型伤寒暴发的流行病	tion of an urban waterbo	顺先;王福泉;宋金林;胡	n;Wang Fuquan;Song J	中国公共卫生学报	nese Journal of Public He	-	5	306	1990	-
320	伤寒暴发调查及流行因素	of typhoid fever and dis	钱仁兴;马华卿;朱剑融;张	Huaqing;Zhu Jianrong;	南京医学院学报	al of Nanjing Medical Co	-	3	239-240	1990	-
321	季枯水型伤寒暴发流行	outbreak of low-water typ	陈祖府,吴光财	Chen Zufu,Wu Guangcai	解放军预防医学杂志	ve Medicine of the Peopl	-	4	397-398	1990	-
322	村伤寒暴发流行对其小	er outbreak in a natural	江世平;	Jiang Shiping;	中国校医	Chinese school doctor	-	3	45-46	1990	-
323	起水型伤寒暴发的调查报	t on an outbreak of water	炽霞;张淑颖;奈淑娟;赵汉	Shuying;Nai Shujuan;Z	中国公共卫生	Chinese public health	-	5	221-222	1990	-
324	接触传播为主的伤寒暴	outbreak of typhoid feve	丽将;雷动;姚廷桓;韦应德;	under;Yao Tinghuan;Wei	广西医学	Guangxi Medicine	-	4	276+281	1990	-
325	伤寒暴发流行100例分析	ever outbreak100Exampl	邵良学;任连勤;	hao Liangxue;Ren Lianqi	辽宁医学杂志	Liaoning Medical Journa	-	2	112	1990	-
326	乡骆西村伤寒暴发的流	fever outbreak in Luoxi	王莉敏;	Wang Limin;	皖南医学院学报	al of Wannan Medical C	-	4	75-76	1990	-
327	市一起食物型伤寒暴发	ood-borne typhoid fever	姜美君;李红兵;	Jiang Meijun;Li Hongbing	西藏医药杂志	Tibetan Medical Journal	-	1	32-34	1990	-
328	源型肠伤寒暴发的流行病	gation of a waterborne e	李照坤	Li Zhaokun	解放军预防医学杂志	ve Medicine of the Peopl	-	1	59-61	1990	-
329	寒沙门氏菌感染分子流行	emiology of neonatal Salm	吴仕孝,唐毅.	Wu Shixiao,Tang Yi.	中华传染病杂志	se Journal of Infectious Dis	9	2	75-78,C1	1991	sn.1000-6680.1991.02.105
330	04株鼠伤寒沙门氏菌耐	eillance of drug resistance	童青青,王水湘.	ng Qingqing,Wang Shuixian	江西医药	Jiangxi Medicine	-	5	266-269	1991	-
331	门氏菌肠炎的特点(附17	a Typhimurium Enteritis(at	张开瑞,辛绍杰.	Zhang Kairui,Xin Shaojie.	北京医学	Beijing Medicine	-	6	339-340	1991	-

332	起沙门氏菌食物中毒调查报	port on a case of salmonella	黄彪.	Huang Biao.	广东卫生防疫	ng Health and Epidemic Pr	-	4	83-84	1991	-
333	引起鼠伤寒沙门氏菌食物中	urium Food Poisoning Cau	端,侯美英,柴崇山,张增龙,左	Chai Chongshan,Zhang Zen	中国食品卫生杂志	hinese Food Hygiene Journ	-	3	37-38	1991	-
334	生儿鼠伤寒沙门氏菌感染6	onella Typhimurium Infect	张玉华,秦璞.	Zhang Yuhua,Qin Pu.	新生儿科杂志	Journal of Neonatology	6	5	222-223	1991	-
335	寒沙门氏菌感染95例临床	himurium Infection95Case	翟琦,张文倩.	Zhai Qi,Zhang Wenqian.	中华传染病杂志	se Journal of Infectious Dis	9	2	111-112	1991	-
336	沙门氏菌VI噬菌体分型及	ou areaVIPhage typing an	建华,孟冬梅,余文炳,杨佩	Meng Dongmei,Yu Wenbing	浙江预防医学与疾病监测	entive Medicine and Diseas	-	1	10-12	1991	-
337	鸡蛋传递沙门氏菌的情况	ransmission of Salmonella f	傅启勇,林安仔.	Fu Qiyong,Lin Anzi.	肉品卫生	Meat hygiene	-	1	7-8	1991	-
338	伤寒沙门氏菌的耐药监测报	urveillance report of Salmo	邓符则,郭恩琪,林宏,平云旺	e,Guo Enqi,Lin Hong,Pingy	蚌埠医药	Bengbu Medicine	-	3	47-48	1991	-
339	动物药材沙门氏菌的污染	ntamination of animal medi	金秋颖,郑钩镛,陈光华.	ng,Zheng Gouyong,Chen Gu	中成药	Chinese patent medicine	-	8	9-10	1991	-
340	尔沙门菌食物中毒的流行	ation of a case of Salmonel	为成,黄平益,王士明,张长	ang Pingyi,Wang Shiming,Z	人民军医	people's military medicine	-	10	10-11	1991	-
341	件感染病人胆汁中检出纽波	rt from the Bile of Patients	吴方斋.	Wu Fangzhai.	中国微生态学杂志	inese Journal of Microecolo	-	2	81	1991	-
342	伤寒沙门氏菌引起的食物中	ing caused by Salmonella T	刘亚男,闫琳.	Liu Yanan,Yan Lin.	中国卫生检验杂志	ese Journal of Health Inspe	-	4	252-253	1991	-
343	个旧市沙门氏菌分布调查	the distribution of Salmon	李家云,王维英,蒋远萍,倪文	ang Weiying,Jiang Yuanpin	疾病监测	disease surveillance	6	8	118-120	1991	-
344	—1990年检出107株沙门氏	l inspection107Laboratory	时耀光,佟亚杰.	Shi Yaoguang,Tong Yajie.	中华医学检验杂志	urnal of Medical Laborator	14	2	114-115	1991	-
345	沙门氏菌对16种抗生素敏感	um16Analysis of antibiotic	黄文智,李淑英.	Huang Wenzhi,Li Shuying.	江西医学检验	Jiangxi Medical Laboratory	9	4	28	1991	-
346	耐药鼠伤寒沙门氏菌败血症	idrug-resistant Salmonella	佘祥奎,马崇德.	She Xiangkui,Ma Chongde.	实用医学杂志	ournal of Practical Medicin	-	3	124-125	1991	-
347	、副伤寒沙门氏菌耐药性	f Salmonella typhoid and p	石优章,黄勇,徐霞芬,邱华士	ng,Huang Yong,Xu Xiafen,Q	浙江预防医学与疾病监测	entive Medicine and Diseas	-	4	13-14,17	1991	-
348	卯肉中沙门氏菌定型试验的	he typing test of Salmonella	顾金泉.	Gu Jinquan.	肉品卫生	Meat hygiene	-	10	3-4,2	1991	-
349	鼠伤寒沙门氏菌感染49例	murium Infection in Adults4	肖忠,陈媛.	Xiao Zhong,Chen Yuan.	青海医学院学报	rnal of Qinghai Medical Col	-	1	70-72	1991	-
350	沙门氏菌食物中毒的实验	ntal report on salmonella fo	郑丁华.	Zheng Dinghua.	广东卫生防疫	ng Health and Epidemic Pr	-	4	85-86	1991	-
351	沙门氏菌感染-流行病学相	ultry and humans-Epidemic	S.I.奥伯格布伦,杜爱芳.	SIObergbrunn,Du Aifang.	浙江畜牧兽医	mal Husbandry and Veterin	-	3	47-48	1991	-
352	鼠伤寒沙门菌感染:附40例	in infants and young childr	何毓琮,陈申义.	He Yucong,Chen Shenyi.	重庆医药	Chongqing Medicine	21	1	10-11	1991	-
353	氏菌引起的新生儿败血症	by Salmonella enjili and is	姚为宁,邓国良.	Yao Weining,Deng Guoliang	中华医学检验杂志	urnal of Medical Laborator	14	4	227	1991	-
354	原发性伤寒沙门氏菌脑膜炎	ary Salmonella typhi menin	郭良君,方永善,左鹏鸥.	ngjun,Fang Yongshan,Zuo P	中华传染病杂志	se Journal of Infectious Dis	9	2	121	1991	sn.1000-6680.1991.02.134
355	寒沙门氏菌感染95例临床	himurium Infection95Case	翟琦,张文倩,尤都斯,滕美珍	hang Wenqian,Yudus,Teng	中华传染病杂志	se Journal of Infectious Dis	9	2	111-112	1991	sn.1000-6680.1991.02.122
356	伤寒沙门氏菌肺脓肿一例报	ng abscess caused by Salmo	刘增荣.	Liu Zengrong.	中华放射学杂志	Chinese Journal of Radiolog	25	2	76	1991	-
357	伤寒沙门氏菌感染40例临	urium Infection in Adults40	绍珍,武力力,孔素平,张爽	n,force,Kong Suping,Zhang	中华传染病杂志	se Journal of Infectious Dis	9	1	45-46	1991	sn.1000-6680.1991.01.122
358	~1990年检出107株沙门氏	l inspection107Laboratory	耀光,佟亚杰,骆剑秋,王振	,Tong Yajie,Luo Jianqiu,Wa	中华医学检验杂志	urnal of Medical Laborator	14	2	114-115	1991	-
359	菌引起新生儿腹泻医院内	omial outbreak of neonata	昌言,周正亮,杨桂文,王素	hou Zhengliang,Yang Guiw	中华流行病学杂志	inese Journal of Epidemiolo	12	5	301	1991	sn.0254-6450.1991.05.118
360	生沙门氏菌引起食物中毒	f food poisoning caused by	孙保良,阎启明.	Sun Baoliang,Yan Qiming.	中华医学检验杂志	urnal of Medical Laborator	14	4	223	1991	-
361	门氏菌败血症合并脑膜炎	almonella typhimurium se	英,郑锡铭,谭巨莲,见金陵,潘	Ximing,Tan Julian,See Jinl	中华医学检验杂志	urnal of Medical Laborator	14	2	95	1991	-
362	脑脊液中分离出鼠伤寒沙	lla typhimurium from infan	李巍,于秀恩.	Li Wei,Yu Xiuen.	中华医学检验杂志	urnal of Medical Laborator	14	6	391	1991	-
363	菌及福氏痢疾菌混合性脑膜	itis caused by Salmonella t	董天德,张吉平,李致斌.	g Tiande,Zhang Jiping,Li Zh	中华传染病杂志	se Journal of Infectious Dis	9	2	119-120	1991	sn.1000-6680.1991.02.131
364	寒沙门氏菌感染17例临床	himurium Infection17Case	张永祥.	Zhang Yongxiang.	铁道医学	Railway Medicine	-	6	365	1991	-
365	患者血液中分离出雷丁沙	nella Redding isolated from	李广英,李迎霞,李珍大.	Guangying,Li Yingxia,Li Zhen	金陵医院学报	Journal of Jinling Hospital	-	3	252	1991	-
366	伤寒沙门氏菌感染(附9例临	infection in children(attach	柳龙根.	Liu Longgen.	实用医学杂志	ournal of Practical Medicin	-	5	242-243	1991	-
367	鼠伤寒沙门菌败血症65例	Typhimurium Septicemia6	云,张俊,申保生,钱惠菌,郑	ng Jun,Applicant,Qian Huij	新乡医学院学报	nal of Xinxiang Medical Col	-	3	-	1991	-
368	菌流行菌株耐药谱及R质粒	valent strains of typhoid b	,龚正,马亦林,杨浓花,傅	Zheng,Ma Yilin,Yang Nong	浙江医科大学学报	al of Zhejiang Medical Univ	-	4	166-169	1991	-

369	一起伤寒流行的病原学诊断	diagnosis of a typhoid feve	陈大波.	Chen Dabo.	中国卫生检验杂志	ese Journal of Health Inspe	-	4	252	1991	-
370	新生儿化脓性脑膜炎临床	ysis of neonatal purulent m	张仁德,王群思,吴慧,吴雪银	de,Wang Qunsi,Wu Hui,Wu	中原医刊	entral Plains Medical Journ	-	3	14-15	1991	-
371	与新生儿肠道传染病-附2	atal intestinal infectious dis	周冬菊,王岩,张晞.	u Dongju,Wang Yan,Zhang	河南预防医学杂志	n Journal of Preventive Me	-	5	841-842	1991	-
372	市南岗区食品从业人员带	ission among food worker	李桂贤,李岩松,王珍,杨立秋	n,Li Yansong,Wang Zhen,Ya	中国卫生检验杂志	ese Journal of Health Inspe	-	3	176-177	1991	-
373	江省伤寒的监测及防制对	ion strategies for typhoid f	建,莫顺堂,黄景苹,陈恩富,张	Tang,Huang Jingping,Chen	浙江预防医学与疾病监测	entive Medicine and Diseas	-	3	13-15	1991	-
374	感染性腹泻病原菌分布的	tion of pathogenic bacteria	战师珍,张安玉,沈宗林.	hizhen,Zhang Anyu,Shen Z	天津医药	Tianjin Medicine	-	5	301-303	1991	-
375	市医院污水肠道致病菌的	athogenic bacteria in hospi	淑滨,杨建平,王新清,刘尊	Yang Jianping,Wang Xinqing	环境与健康杂志	rnal of Environment and He	-	5	230	1991	-
376	起伤寒暴发的经济费用分	nomic costs of a typhoid	素洁;陈士保;惠建华;杨亚	en Shibao;Hui Jianhua;Y	中国公共卫生管理杂志	ournal of Public Health M	-	S1	278-280	1991	.cnki.23-1318.1991.s1.196
377	一起伤寒暴发的调查	on into an outbreak of typ	吕红云;	Lu Hongyun;	中国公共卫生学报	hese Journal of Public He	-	5	274	1991	-
378	镇一起水传伤寒暴发流行	eak of water-borne typho	中辉,刘成龙,赵友诚,谢丛生	englong,Zhao Youcheng	环境与健康杂志	nal of Environment and H	-	4	186	1991	ki.1001-5914.1991.04.023
379	有关肠伤寒暴发流行的报道	ts on enteric typhoid outb	-	-	广东卫生防疫	ng Health and Epidemic F	-	4	24-26	1991	-
380	某部队医院伤寒暴发调查	yphoid fever outbreak in	田;田树林;陈武泽;王忠灿;朱	n;Chen Wuze;Wang Zho	人民军医	people's military medicine	-	7	19-20	1991	-
381	部家属区伤寒暴发流行的调	k of typhoid fever in a far	启荣;管懋钧;刘惠敏;彭湘	uan Maojun;Liu Huimin;F	解放军预防医学杂志	ve Medicine of the Peopl	-	3	220	1991	-
382	一起水型伤寒暴发调查报告	on an outbreak of water	单卫,杨祚弥,唐文廷	uard,Yang Zuomi,Tang V	重庆医药	Chongqing Medicine	-	3	186	1991	-
383	起学校副伤寒暴发流行的调	n outbreak of paratyphoi	谢锦梅;刘文生;	Xie Jinmei;Liu Wensheng	中国学校卫生	Chinese school health	-	3	164-165	1991	-
384	暴发流行的血清肥达氏反应	l Reaction Results in a T	莫美仪;黄乐佳;	Mo Meiyi;Huang Lejia;	广东卫生防疫	ng Health and Epidemic F	-	3	9-12	1991	-
385	感染在新生儿病室和婴儿	Monteverde infection in ne	邵钰华,单丽玲,汪明.	o Yuhua,Shan Liling,Wang M	新生儿科杂志	Journal of Neonatology	7	2	49-50	1992	-
386	氏菌引起36例食物中毒的	B36Etiology identification	华,李金学,邓秀琴,于静华,刘	hua,Li Jinxue,Deng Xiuqin,	中国卫生检验杂志	ese Journal of Health Inspe	-	5	317-318	1992	-
387	离134株沙门氏菌的鉴定及	tification and drug suscept	刘内生,袁圣蓉.	u Neisheng,Yuan Shengron	民族学院学报（畜牧兽医	ationalities (Animal Husbar	18	1	98-100	1992	-
388	林沙门氏菌引起的82人食	monella Dublin82Human fo	张桂珍,李影林.	Zhang Guizhen,Li Yinglin.	中华医学检验杂志	urnal of Medical Laborator	15	3	163-164	1992	-
389	株沙门氏菌属菌型鉴定报	onella strain identification	凡,张英华,闫文海,赵玲红,于	Zhang Yinghua,Yan Wenha	中国卫生检验杂志	ese Journal of Health Inspe	-	4	246	1992	-
390	伤寒沙门氏菌所致爆发性食	ak of food poisoning cause	王淑敏.	Wang Shumin.	中国食品卫生杂志	hinese Food Hygiene Journ	-	3	36	1992	-
391	贸市场鲜销猪肉沙门氏菌污	of fresh pork sold in farme	张彦明,贾靖国.	Zhang Yanming,Jia Jingguo.	肉品卫生	Meat hygiene	-	12	8-11	1992	-
392	农贸市场鲜猪肉沙门氏菌污	contamination of fresh po	成,王晓玲,李承钰,韩玉兰,赵	Xiaoling,Li Chengyu,Han Y	青海畜牧兽医杂志	mal Husbandry and Veterin	-	1	29-30	1992	-
393	寒沙门氏菌软组织脓肿2例	murium soft tissue abscess	李昆,熊英.	Li Kun,Xiong Ying.	江西医学院学报	rnal of Jiangxi Medical Coll	-	2	177186	1992	-
394	起沙门氏菌食物中毒调查报	port on a case of salmonella	冉丽莎.	Ran Lisa.	昌都科技	Qamdo Technology	-	1	68-69	1992	-
395	克兰沙门菌食物中毒63例的	ckland food poisoning63ca	王建瑞.	Wang Jianrui.	人民军医	people's military medicine	-	10	18-19	1992	-
396	血液中分离出22例鼠伤寒沙	oodtwenty twoReport of a	林惠英,黄炳勇.	Lin Huiying,Huang Bingyong	福建医药杂志	Fujian Medical Journal	-	2	40	1992	-
397	市洛克兰沙门氏菌引起的食	poisoning caused by Salmo	朱健铭,吴晋兰.	Zhu Jianming,Wu Jinlan.	浙江预防医学与疾病监测	entive Medicine and Diseas	-	4	24-25	1992	-
398	婴幼儿暴发鼠伤寒沙门氏菌	ea outbreak in hospitalized	阁,李统义,孙毅,许小棉,王	i Tongyi,Sun Yi,Xu Xiaomia	中国医院感染学杂志	ournal of Hospital Infectiou	-	4	220-222	1992	-
399	食行业人员沙门氏菌带菌情	e among personnel in the	李星.	Li Xing.	泰山医学院学报	rnal of Taishan Medical Col	-	1	75-77	1992	-
400	地区鼠伤寒沙门氏菌噬菌	monella typhimurium in sc	维萍,周用直,张鹏云,张锦	Zhou Yongzhi,Zhang Pengyu	河南预防医学杂志	n Journal of Preventive Me	-	3	141-142	1992	-
401	沙门氏菌污染病牛肉中毒6	ed by salmonella typhimur	张龙庭,王方元.	ang Longting,Wang Fangyu	青海医药杂志	Qinghai Medical Journal	-	4	41	1992	-
402	株沙门氏菌生化反应结果	chemical reaction results of	段韵玉,宗泓.	Duan Yunyu,Zong Hong.	陕西医学检验	haanxi Medical Examinatio	7	3	176-177	1992	-
403	沙门氏菌食物中毒后追踪调	od poisoning caused by Sal	王玉兰,高杰谦,王燕.	ng Yulan,Gao Jieqian,Wang	河南预防医学杂志	n Journal of Preventive Me	-	2	67-70	1992	-
404	噬菌体分型在鼠伤寒沙门	ermination and phage typi	谢一俊,陈亢川.	Xie Yijun,Chen Kangchuan.	中国人兽共患病杂志	Chinese Journal of Zoonose	-	1	38-40	1992	-
405	阳县215株沙门氏菌的菌型	5Type determination of Sa	邓国良.	Deng Guoliang.	中国卫生检验杂志	ese Journal of Health Inspe	-	2	110	1992	-

406	入抑菌剂直接分离沙门氏菌	isolating Salmonella by ad	张传新,冯玉平,周观涛,孟	uanxin,Feng Yuping,Zhou G	中国卫生检验杂志	ese Journal of Health Inspe	-	2	93-95	1992	-
407	院鼠伤寒沙门氏菌致医院	by Salmonella typhimuriu	单景生,陈月华,沈燕.	ingsheng,Chen Yuehua,She	中国医院感染学杂志	ournal of Hospital Infectiou	-	2	86-88	1992	-
408	沙门氏菌院内感染的分子流	y of a nosocomial infectio	康,单兆南,邵亚萍,李秀荣,孙	Zhaonan,Shao Yaping,Li Xi	中国卫生检验杂志	ese Journal of Health Inspe	-	1	62-63	1992	-
409	寒沙门氏菌食物中毒临床	od poisoning caused by Sal	王新忠,宋继成.	Vang Xinzhong,Song Jichen	吉林医学情报	Jilin Medical Information	-	7	55-56	1992	-
410	寒沙门氏菌感染41例临床	himurium Infection41Case	刘昱,陈奇.	Liu Yu,Chen Qi.	临床医学	clinical medicine	-	6	246-247	1992	-
411	污水中沙门氏菌动态观察	observation of Salmonella	覃方珍,罗光荣.	in Fangzhen,Luo Guangron	环境与健康杂志	nal of Environment and He	-	2	70-72	1992	-
412	门氏菌引起食物中毒的病原	gy of food poisoning cause	章,向一元,王淑芳,刘黎,张	one yuan,Wang Shufang,Li	中国卫生检验杂志	ese Journal of Health Inspe	-	3	180-181	1992	-
413	沙门菌感染790例的流行病	phi infection790Epidemiol	尹澎,王晓峰,姜素椿.	eng,Wang Xiaofeng,Jiang Su	人民军医	people's military medicine	-	8	32-36	1992	-
414	鼠伤寒沙门氏菌食物中毒	lmonella Typhimurium Foo	刘凤云.	Liu Fengyun.	河南预防医学杂志	n Journal of Preventive Me	-	6	375	1992	-
415	鼠伤寒沙门菌感染-附40例	in infants and young childr	何毓琮,陈申义.	He Yucong,Chen Shenyi.	重庆医药	Chongqing Medicine	-	1	10-11	1992	-
416	地区745株沙门氏菌菌型	rea745Salmonella strain di	简永新.	Jian Yongxin.	中国食品卫生杂志	hinese Food Hygiene Journ	-	4	56	1992	-
417	山夫登堡沙门氏菌食物中毒	a cross-regional Salmonella	陈煜,黄晓炜,张宝马.	Yu,Huang Xiaowei,Zhang B	食品科学	food science	-	9	47-50	1992	-
418	儿童沙门氏菌感染临床研究	dy of salmonella infection	王晓峰,尹澎,罗进华,姜素椿	eng,Yin Peng,Luo Jinhua,Ji	实用儿科临床杂志	ctical Pediatric Clinical Jou	-	3	115-117	1992	-
419	内鼠伤寒沙门氏菌污染情况	onella Typhimurium Conta	李玉荣,关德显,李春琳.	urong,Guan Dexian,Li Chur	中国卫生检验杂志	ese Journal of Health Inspe	-	1	50-52	1992	-
420	伤寒沙门氏菌的病原学鉴	ion and analysis of Salmon	张敏,张秀云.	Zhang Min,Zhang Xiuyun.	中华预防医学杂志	se Journal of Preventive Me	26	1	51	1992	-
421	猪体内沙门氏菌的分离与	tification of Salmonella fro	谈亚英,谢宗清.	Tan Yaying,Xie Zongqing.	肉品卫生	Meat hygiene	-	2	7-8	1992	-
422	伤寒沙门氏菌校内感染的	hool-wide infection with Sa	吴首敬,黄传滋,丁季(目亘)	jing,Huang Chuanzi,Ding Ji	上海医学	Shanghai Medicine	-	5	295	1992	-
423	同体猪霍乱沙门氏菌的分离	Salmonella Choleraesuis fr	李槿年,潘玲.	Li Jinnian,Pan Ling.	肉禽蛋	Meat, poultry and eggs	-	6	16-17	1992	-
424	儿沙门氏菌脑膜炎五例临床	five cases of neonatal saln	李初阳,詹美玲,肖英,邹音	ang,Zhan Meiling,Xiao Ying,	新生儿科杂志	Journal of Neonatology	7	5	219-220	1992	-
425	内里丁沙门氏菌食物传播的	tbreak of Salmonella Ridin	康涅狄格州,何娟.	Connecticut,He Juan.	疾病监测	disease surveillance	7	5	139	1992	-
426	寒沙门氏菌致化脓性关节炎	arthritis caused by Salmone	贾昉.	Jia Fang.	中华医学检验杂志	urnal of Medical Laborator	15	3	179	1992	-
427	兰沙门氏菌引起家族性食	rockland causes familial fo	占伟,刘淑纯,张立然,苗春	Liu Shuchun,Zhang Liran,M	中华医学检验杂志	urnal of Medical Laborator	15	3	166	1992	-
428	伤寒沙门氏菌的病原学鉴	ion and analysis of Salmon	王燕,王桂芹,邓正,张冬民,张	ng Guiqin,Deng Zheng,Zha	中华预防医学杂志	se Journal of Preventive Me	26	1	51	1992	-
429	耳道分泌物中分离出婴儿	ella infantis from ear canal	燕萍,田桂荣,张军民,程彦	an Guirong,Zhang Junmin,	中华医学检验杂志	urnal of Medical Laborator	15	4	247	1992	-
430	液中分离到一株胥伐成格	ngstrom was isolated from	龚伯玲,宋霞,苏浩,张亚民	oling,Song Xia,Su Hao,Zhang	中华医学检验杂志	urnal of Medical Laborator	15	4	224	1992	-
431	引起鼠伤寒沙门氏菌食物中	hurium Food Poisoning Cau	素云,魏秀瑞,柴崇山,侯美英	i,Chai Chongshan,Hou Mei	中华流行病学杂志	inese Journal of Epidemiolo	13	2	109	1992	sn.0254-6450.1992.02.120
432	环境传染物中同时分离出	d from the blood, feces an	邓君宽,韩桂香.	Deng Junkuan,Han Guixiang	中华医学检验杂志	urnal of Medical Laborator	15	2	98	1992	-
433	脓疡检出一株无动力B群沙	detected from an infant ab	相法,王新桃,刘运弟,李相	a,Wang Xintao,Liu Yundi,Li	中华医学检验杂志	urnal of Medical Laborator	15	3	162	1992	-
434	婴儿沙门氏菌脑膜炎一例	f salmonella meningitis in a	刘茂贤,王茂贵,汪定成.	ian,Wang Maogui,Wang Di	中华传染病杂志	se Journal of Infectious Dis	10	1	13-13,32	1992	sn.1000-6680.1992.01.105
435	林沙门氏菌引起的82人食	monella Dublin82Human fo	英淑,徐秀杰,李影林,王艾	-sook,Xu Xiujie,Li Yinglin,W	中华医学检验杂志	urnal of Medical Laborator	15	3	163-164	1992	-
436	液中分离出甲型副伤寒沙门	onella paratyphi A from ce	蔡润芳,许萍,关廷枢.	Runfang,Xu Ping,Guan Ting	中华医学检验杂志	urnal of Medical Laborator	15	5	287	1992	-
437	引起鼠伤寒沙门氏菌食物中	n caused by Salmonella typ	柴崇山,唐恩辉,徐素云.	Chongshan,Tang Enhui,Xu S	中华流行病学杂志	inese Journal of Epidemiolo	13	2	122	1992	sn.0254-6450.1992.02.124
438	霍乱沙门氏菌肠炎12例报	s Salmonella enteritis12Exa	许五云.	Xu Wuyun.	铁道医学	Railway Medicine	-	4	0	1992	-
439	儿颅内鼠伤寒沙门氏菌感染	racranial infection with Sal	张占平.	Zhang Zhanping.	兰后卫生	Hygiene after orchid	-	1	64	1992	-
440	散发性鼠伤寒沙门氏菌医	n of Salmonella typhimuriu	沈春明,HEFEI,ANHUI PR	en Chunming,HEFEI,ANH	中国医院感染学杂志	ournal of Hospital Infectiou	-	3	138-140	1992	-
441	1990~1991年食品从业人员	0~1991Annual survey on b	张新群.	Zhang Xinqun.	广东卫生防疫	ong Health and Epidemic Pr	-	4	89-90	1992	-
442	腹泻病患者细菌病原学调	s of bacterial etiology in pa	施培福,吴立珍,秋宝明,洪峰	,Wu Lizhen,Qiu Baoming,H	安徽医学	Anhui Medicine	-	6	44	1992	-

443	儿室鼠伤寒医院感染暴发	of nosocomial infection wi	尹瑞明,林燕英.	Yin Ruiming,Lin Yanying.	泸州医学院学报	rnal of Luzhou Medical Coll	-	3	211-212	1992	-
444	副伤寒的耐药变化及临床	nce and clinical analysis of	陈亮.	Chen Liang.	养猪	pig raising	-	2	32-33	1992	-
445	914名"健康人"肠道致病菌	lthy person"Investigation c	陆建荣,何玉祥.	Lu Jianrong,He Yuxiang.	浙江预防医学与疾病监测	entive Medicine and Diseas	-	3	15-17	1992	-
446	性感染性腹泻病原流行病	the pathogens of acute infe	赛明,阎立群,吴素珍,刘加	aiming,Yan Liqun,Wu Suzh	中华流行病学杂志	inese Journal of Epidemiolo	13	2	85-88	1992	sn.0254-6450.1992.02.112
447	染监控系统监测报表中常	g reports of the National H	秋丽,李六亿,巩志业,王枢	Li Liuyi,Gong Zhiye,Wang S	中国医院感染学杂志	ournal of Hospital Infectiou	-	1	64	1992	-
448	顺义县1974-1990年食物中	g City1974-1990annual foo	郝兰英,赵文华,孟凡超.	ying,Zhao Wenhua,Meng F	中国食品卫生杂志	hinese Food Hygiene Journ	-	3	31	1992	-
449	院水型伤寒暴发及人群带	tain hospital and investig	自力,孟庆泗,田树林,陈武	leng Qingsi,Tian Shulin,(	解放军预防医学杂志	ve Medicine of the Peopl	-	6	2	1992	-
450	起毛蚶引起副伤寒暴发的调	ak of paratyphoid fever c	生,丁恩琴,袁平,王忠意,汪	Enqin,Yuan Ping,Wang Z	中国公共卫生	Chinese public health	-	12	533	1992	-
451	一起冬季伤寒暴发的调查	to an outbreak of typhoid	松荣,李辉平	Song Rong,Li Huiping	中国公共卫生	Chinese public health	-	12	558	1992	-
452	县么索村1990年伤寒暴发	ounty1990Survey on annu	李谷保;	Li Gubao;	疾病监测	disease surveillance	-	6	161	1992	-
453	起伤寒暴发流行的临床分	alysis of an outbreak of ty	邓以秀,高永忠	eng Yixiu,Gao Yongzhon	湖南医学	Hunan Medicine	-	6	368-369	1992	-
454	起水媒伤寒暴发流行的调	o an outbreak of waterbo	东力,刘希真,周菊林,刘国	Liu Xizhen,Zhou Julin,Liu	解放军预防医学杂志	ve Medicine of the Peopl	-	5	2	1992	-
455	宗伤寒暴发流行的临床报	eport on an outbreak of ty	罗淦祥;	Luo Ganxiang;	广州医药	Guangzhou Medicine	-	4	42	1992	-
456	水源性伤寒暴发性流行的	xplosive Epidemic of Wat	邱福军;齐飞;	Qiu Fujun;Fly together;	人民军医	people's military medicine	-	7	12-13	1992	-
457	来水引起伤寒暴发流行的	yphoid outbreak caused	陆致齐,李义平,谭速建,	Zhiqi,Li Yiping,Tan Sujia	浙江预防医学与疾病监测	ntive Medicine and Disea	-	3	22-23	1992	sn1007-0931.1992.03.013
458	起水型伤寒暴发流行的调	o an outbreak of waterbo	雷补团,王义,耿逢成	Tuan,Wang Yi,Geng Fen	解放军预防医学杂志	ve Medicine of the Peopl	-	2	3	1992	-
459	寒恢复期带菌者引起的伤寒	hoid fever caused by a c	赵善强,姜器	hao Shanqiang,ginger wa	浙江预防医学与疾病监测	ntive Medicine and Disea	-	2	36	1992	sn1007-0931.1992.02.021
460	起伤寒暴发流行菌型分布调	n of epidemic bacterial ty	普布,拉吉	Pubu,raj	西藏医药杂志	Tibetan Medical Journal	-	1	39-40	1992	-
461	感染引起仔猪副伤寒暴发	hoid outbreak in piglets c	山;高云霞;张云鹏;冯殿军	unxia;Zhang Yunpeng;F	辽宁畜牧兽医	nal Husbandry and Veteri	-	1	13-14	1992	-
462	局部地区一起伤寒暴发的	yphoid fever outbreak in	升;力志峰;蒋震羚;黄忠耀;	ifeng;Jiang Zhenling;Hua	广西医学	Guangxi Medicine	-	1	69-70	1992	-
463	儿沙门菌感染临床流行病	ogical analysis of salmonell	吴实,樊绍曾.	Wu Shi,Fan Shaozeng.	中华医学杂志	Chinese Medical Journal	73	1	14-16	1993	-
464	达尔沙门菌引起的食物中	soning caused by Salmone	李洪宝,颜夏梅.	Li Hongbao,Yan Xiamei.	中华预防医学杂志	se Journal of Preventive Me	27	3	139-140	1993	-
465	门氏菌感染(附60例新生儿	nfection(attached60Clinical	曹友文,杭桂香.	Cao Youwen,Hang Guixiang	江苏医药	Jiangsu Medicine	-	3	145-146	1993	-
466	人员携带沙门氏菌调查及	eptibility testing of Salmon	黄健,封丽芝,简风兰.	ng Jian,Feng Lizhi,Jian Feng	中国卫生检验杂志	ese Journal of Health Inspe	-	6	361-362	1993	-
467	鹅肉而暴发纽波特沙门氏	isoning outbreak caused by	潘玲,姚敬业.	Pan Ling,Yao Jingye.	肉品卫生	Meat hygiene	-	11	F003-F003,28	1993	-
468	鼠伤寒沙门氏菌食物中毒	lmonella Typhimurium Foo	商晓春.	Shang Xiaochun.	浙江预防医学与疾病监测	entive Medicine and Diseas	-	2	13	1993	-
469	型副伤寒沙门氏菌院内感	ial infection of neonates w	戴丽珍,雷后兴,王伟.	Lizhen,Lei Houxing,Wang W	温州医学院学报	nal of Wenzhou Medical Co	-	2	115-116,104	1993	-
470	科病房鼠伤寒沙门氏菌感	phimurium Infection in a P	冯振勃,安晓华,马宏.	g Zhenbo,An Xiaohua,Mah	中国医院感染学杂志	ournal of Hospital Infectiou	-	2	89	1993	-
471	地区蛇类检出沙门氏菌的	ella detected in snakes in t	尹子达,吴采菲.	Yin Zida,Wu Caifei.	中国人兽共患病杂志	Chinese Journal of Zoonose	-	5	18-22	1993	-
472	寒沙门氏菌的耐药性变迁	drug resistance of Salmone	谢一俊,陈亢川,林成水.	n,Chen Kangchuan,Lin Che	中国人兽共患病杂志	Chinese Journal of Zoonose	-	3	13-16	1993	-
473	鼠伤寒沙门氏菌院内感染	omial infection with Salmo	张文浩,张爱荣.	hang Wenhao,Zhang Airon	河南预防医学杂志	n Journal of Preventive Me	4	6	331-332	1993	-
474	南部分地区猪沙门菌菌型	monella swine strains in so	李学良,李炳云.	Li Xueliang,Li Bingyun.	云南畜牧兽医	nal Husbandry and Veterina	-	4	9-10	1993	-
475	分离一株发酵乳糖的鼠伤寒	ermenting Salmonella typh	张乐海,傅云霜.	Zhang Lehai,Fu Yunshuang	中国微生态学杂志	inese Journal of Microecolo	-	1	67	1993	-
476	伤寒沙门菌病暴发流行菌	s of Salmonella typhimuriu	吴汉勇,林发榕.	Wu Hanyong,Lin Farong.	中国人兽共患病杂志	Chinese Journal of Zoonose	-	4	8-10,2	1993	-
477	伤寒沙门氏菌外科感染:	ith Salmonella typhimurium	刘宝萍,李达泉.	Liu Baoping,Li Daquan.	武汉职工医学院学报	al of Wuhan Staff Medical C	-	1	29-30	1993	-
478	株伤寒沙门氏菌药敏结果	susceptibility results of Sal	雷华.	Lei Hua.	江西医学检验	Jiangxi Medical Laboratory	11	1	27	1993	-
479	西省654株沙门氏菌菌型	ince654Salmonella strain i	,宋元鍉,樊招凤,蔡定妍,	n Dynasties,Fan Zhaofeng,(	中国人兽共患病杂志	Chinese Journal of Zoonose	-	4	42-43	1993	-

480	售蔬菜表面沙门氏菌污	on on the surface of comm	小燕,郭成宏,吴道忠,陶芝	uo Chenghong,Wu Daozho	昆明医学院学报	nal of Kunming Medical Co	-	4	47-50,64	1993	-
481	寒沙门氏菌感染的临床特	agnosis and treatment of	白兆淑,方金龙.	Bai Zhaoshu,Fang Jinlong.	长治医学院学报	nal of Changzhi Medical Co	7	4	428-429	1993	-
482	猪体内沙门氏菌的分离与	tification of Salmonella fro	谈亚英.	Tan Yaying.	肉品卫生	Meat hygiene	-	2	7-8	1993	-
483	林巴结带沙门氏菌情况调	ion of Salmonella in lymph	顾金泉.	Gu Jinquan.	肉品卫生	Meat hygiene	-	5	16-17	1993	-
484	寒沙门氏菌所致医院内交	ocomial cross-infection cau	,方国辉,赵小娟,钱小英,	ohui,Zhao Xiaojuan,Qian X	浙江预防医学与疾病监测	entive Medicine and Diseas	-	2	19-20	1993	-
485	沙门菌感染在我料暴发流	nella Typhimurium infectio	李甘美,程志刚.	Li Ganmei,Cheng Zhigang.	浙江医学	Zhejiang Medicine	15	6	369	1993	-
486	沙门氏菌药物敏感性分	tibility analysis of Salmon	俊成,郭秋成,刘学贤,张如	Guo Qiucheng,Liu Xuexian,	中国畜禽传染病	stock and poultry infectiou	-	4	54-55,53	1993	-
487	伤寒沙门菌感染95例临床	himurium infection95Case	曹友文,杭桂香.	Cao Youwen,Hang Guixiang	南京医学院学报	rnal of Nanjing Medical Col	-	3	265-266	1993	-
488	区2095株伤寒沙门氏菌噬	Monitoring and research	马桂芳,裴秀藏.	Ma Guifang,Pei Xiuzang.	河南预防医学杂志	Journal of Preventive Me	4	3	163-166	1993	-
489	1991年健康人群沙门氏菌	e distribution of Salmonell	何水渊.	He Shuiyuan.	浙江预防医学与疾病监测	entive Medicine and Diseas	-	4	14-15	1993	-
490	液中检出一株鼠伤寒沙	Salmonella typhimurium f	董桂荣,赵丽君,郭秀琴.	Guirong,Zhao Lijun,Guo Xi	大连大学学报	Journal of Dalian University	-	3	149-150	1993	-
491	1989年伤寒沙门氏菌噬菌	Annual survey on phage ty	张凤琴,刘桂荣,曹润九,王	gqin,Liu Guirong,Cao Runji	中国公共卫生	Chinese public health	-	6	247-248	1993	-
492	猪鸭沙门氏菌带菌调查	on Salmonella carriage in p	任锦玉,程苏云,沈向红.	yu,Cheng Suyun,Shen Xian	中国公共卫生	Chinese public health	-	S2	62-63	1993	-
493	伤寒沙门氏菌医院内感染	ocomial infections caused b	颖,上官玉梅,张风莲,徐	g,Shangguan Yumei,Zhang	中国微生态学杂志	inese Journal of Microecolo	-	1	35	1993	-
494	伤寒沙门氏菌噬菌体分型、	detection of Salmonella typ	广业,贺延平,李菊鸣,孙殿	gye,He Yanping,Li Juming,S	中国人兽共患病杂志	Chinese Journal of Zoonose	-	4	44-45	1993	-
495	鼠伤寒沙门氏菌鉴定及药	susceptibility test of Salm	葛德林.	Gedelin.	冶金医药情报	ical and pharmaceutical in	-	1	48-49	1993	-
496	体猪霍乱沙门氏菌的分离	on of Salmonella cholerae	李槿年,潘玲.	Li Jinnian,Pan Ling.	肉品卫生	Meat hygiene	-	6	18-19	1993	-
497	鼠伤寒沙门氏菌感染(附12	himurium Infection(attach	黄耀堂,刘晓红,赵凤梅.	aotang,Liu Xiaohong,Zhao	实用医学杂志	ournal of Practical Medicin	-	6	1-2	1993	-
498	沙门氏菌(S.haardt)引起食	rtella(S.haardt)Reports of f	陈玉荣,李永强.	Chen Yurong,Li Yongqiang.	河南预防医学杂志	Journal of Preventive Me	4	4	214-215	1993	-
499	建康体检检出的382株沙	Tianhe District, Guangzhou	田执东.	Tian Zhidong.	广东卫生防疫	ng Health and Epidemic Pr	-	1	43-45	1993	-
500	2株沙门氏茁的噬菌体分	age typing of Salmonella β	小玲,张梦萍,陆佩华,樊招	pling,Zhang Mengping,Lu P	江西医学检验	Jiangxi Medical Laboratory	11	2	1-4	1993	-
501	合并鼠伤寒沙门氏菌脓毒	combined with Salmonell	杨发莲,娄洲明,黄晓娣.	alian,Lou Zhouming,Huang	青海医药杂志	Qinghai Medical Journal	-	5	18	1993	-
502	腹主动脉沙门氏菌感染	minal aorta salmonella infe	康进.	Kang Jin.	国外医学.外科学分册	eign medicine.Surgery Bra	-	1	61	1993	-
503	中分离的90株鼠伤寒沙门	with diarrhea90Analysis of	世瑜,卢亚林,谭红,黄亚	yu,Lu Yalin,Tan Hong,Huang	实用医药杂志	ournal of Practical Medicin	-	1	26-27	1993	-
504	区1979~1992年沙门氏菌	e1979~1992Annual Salmo	其宇,方维才,李志悦,董桂	iyu,Fang Weicai,Li Zhiyue,	中国公共卫生	Chinese public health	-	S2	61-62	1993	-
505	沙门氏菌引起食物中毒	ase of food poisoning caus	爱玲,孙锦山,张爱国,郭宪	g,Sun Jinshan,Zhang Aiguo,	中华流行病学杂志	inese Journal of Epidemiolo	14	1	44	1993	sn.0254-6450.1993.01.120
506	鼠伤寒沙门菌病皮肤感	ction associated with Salm	冬平,陈铁英,白奎龙,葛继	g,Chen Tieying,Bai Kuilong,	中华皮肤科杂志	inese Journal of Dermatolo	26	5	316	1993	-
507	门氏菌引起食物中毒调查	on food poisoning caused	蔡正选.	Cai Zhengxuan.	中华预防医学杂志	se Journal of Preventive Me	27	2	128	1993	-
508	者大便培养中检出一株当	ancaster from the stool cul	王英珍,张路,赵宏涛.	Yingzhen,Zhang Lu,Zhao Ho	中华医学检验杂志	urnal of Medical Laborator	16	4	198	1993	-
509	达尔沙门菌引起的食物中	soning caused by Salmone	夏梅,田为成,田珍广,于增	an Weicheng,Tian Zhengua	中华预防医学杂志	se Journal of Preventive Me	27	3	139-140	1993	-
510	儿沙门菌感染临床流行病	ogical analysis of salmonel	,樊绍曾,林凤,王顺林,段	eng,Lin Feng,Wang Shunlin	中华医学杂志	Chinese Medical Journal	73	1	14-16	1993	-
511	沙门菌引起败血症一例及	ed by Salmonella Concord	杨成方.	Yang Chengfang.	中华医学检验杂志	urnal of Medical Laborator	16	4	225	1993	-
512	鼠伤寒沙门氏菌引起的食	isoning caused by Salmone	吴佩琳.	Wu Peilin.	河南医科大学学报	nal of Henan Medical Unive	-	1	64	1993	-
513	和鼠伤寒型沙门氏菌感染	urium Infections in Neona	方淑霞,张玉华,秦(王卜).	huxia,Zhang Yuhua,Qin(Wa	青岛大学医学院学报	Qingdao University Medic	-	2	-	1993	-
514	伤寒沙门氏菌败血症15例	Typhimurium Septicemia1	吕菁,陆彪,郭若冰.	u Jing,Lu Biao,Guo Ruobing	宁夏医科大学学报	nal of Ningxia Medical Univ	-	1	-	1993	-
515	染性腹泻病原调查及耐药	diarrhea pathogens and dr	,郭宗琪,尹致英,徐跃芳,	gqi,Yin Zhiying,Xu Yuefang,	预防医学情报杂志	of Preventive Medicine Inf	-	S1	115	1993	-
516	蒙古西部90株鼠伤寒菌	ongolia90Phage typing res	涛,张利平,郭秀英,金格,王	ng Liping,Guo Xiuying,Ging	内蒙古医学杂志	ner Mongolia Medical Jour	-	4	37	1993	-

517	对虾细菌污染及污染源调	contamination and pollutio	李泽瑶.	Li Zeyao.	现代商检科技	commodity inspection tec	-	6	10-12,19	1993	-
518	镇一次细菌性食物中毒	bacterial food poisoning in	张国嵘.	Zhang Guorong.	预防医学情报杂志	of Preventive Medicine Info	-	S1	117	1993	-
519	区农村腹泻病病原谱及流	miology of diarrheal disea	建,陈振东,沈祥生,白艳	on,Chen Zhendong,Shen Xi	中级医刊	termediate medical journa	-	11	25-27	1993	-
520	新生儿败血症(附82例报告	l sepsis(attached82Exampl	琪,张玉琢,杨锡英,金静仪,	ang Yuzhuo,Yang Xiying,Kir	新生儿科杂志	Journal of Neonatology	8	5	211-212	1993	-
521	流行菌株的耐药谱及质粒	d plasmid map of epidemi	,杜继昭,王其南,郑行苹,李	Jizhao,Wang Qinan,Zheng	中国抗生素杂志	hinese Journal of Antibioti	-	3	196-201	1993	-
522	西伤寒杆菌VI噬菌体分型	bacilliVIPhage typing and d	唐振柱,方志峰,林红,周艳	,Fang Zhifeng,Lin Hong,Zh	广西医学	Guangxi Medicine	-	6	571-573	1993	-
523	牛乳中的病原细菌	Pathogenic bacteria in milk	房兴利.	Fang Xingli.	黄牛杂志	scalper magazine	-	3	80-82	1993	-
524	儿感染性腹泻常见病原菌	athogenic bacteria of infect	,詹利生,吕建华,贾银芝,	Lisheng,Lu Jianhua,Jia Yin	衡阳医学院学报	al of Hengyang Medical Co	-	2	150-154,149	1993	-
525	源性伤寒局部暴发流行调	port on a local outbreak of	胡小铭,张晓莉.	Hu Xiaoming,Zhang Xiaoli.	预防医学情报杂志	of Preventive Medicine Info	-	S1	130	1993	-
526	鸡源肠道杆菌的分离与鉴	tion of enterobacteriaceae	陈金顶,罗德英,吴筱华,刘	ding,Luo Deying,Wu Xiaoh	甘肃农业大学学报	l of Gansu Agricultural Uni	-	3	272-275	1993	-
527	凉拌猪响皮引起的食物中	poisoning caused by cold p	喻胜万.	Yu Shengwan.	预防医学情报杂志	of Preventive Medicine Info	-	S1	117	1993	-
528	寒流行菌株噬菌体型别的	hage types of epidemic typ	方志峰,梁玉裕,林红,林玫	eng,Liang Yuyu,Lin Hong,Li	广西医学	Guangxi Medicine	-	1	59-61	1993	-
529	感染性腹泻34例临床分析	us diarrhea34Case clinical	陈帼英.	Chen Guoying.	张家口医学院学报	al of Zhangjiakou Medical C	-	2	70,80	1993	-
530	伤寒病人院内感染麻疹一	measles infection in a patie	廖定.	Liao Ding.	中国医院感染学杂志	ournal of Hospital Infectiou	-	2	117	1993	-
531	儿细菌性腹泻271例病原分	ea in children271Case pat	卢知吾,张开瑞,张晓峰.	wu,Zhang Kairui,Zhang Xia	人民军医	people's military medicine	-	7	55-56	1993	-
532	鹤峰县一起水源型伤寒暴	hoid fever occurred in H	李端方;李慧荣;王喜富;	anfang;Li Huirong;Wang	湖北预防医学杂志	Journal of Preventive Me	-	4	47	1993	-
533	起水型伤寒暴发的调查报	t on an outbreak of wate	善根;吴济生;常隆述;杜光	Jisheng;Chang Longshu	数理医药学杂志	nal of Mathematical Med	-	4	89	1993	-
534	南县伤寒暴发后慢性带菌	l carriers after typhoid fe	卓晓琴;吕春枝;陈祖建;张	oqin;Lu Chunzhi;Chen Z	疾病监测	disease surveillance	-	6	159-160	1993	-
535	起水型伤寒暴发流行调查	analysis of an outbreak	树贤;梁琴秋;范建英;张藻	ang Qinqiu;Fan Jianying	云南医药	Yunnan Medicine	-	5	320-321	1993	-
536	伤寒暴发流行的调查	on on the outbreak of typ	,郭启高,周庆礼,刘电臣,	igao,Zhou Qingli,Liu Dia	蚌埠医学院学报	al of Bengbu Medical Co	-	3	216	1993	-
537	卫生 家庭自办酒席 食源	d hygieneFamily banquetf	朱宝华;	Zhu Baohua;	上海预防医学杂志	ai Journal of Preventive M	-	9	46	1993	8/j.cnki.sjpm.1993.09.044
538	寒暴发流行调查及预防效	oid fever outbreak and e	来法,傅岳山,郑雪兰,洪云	ueshan,Zheng Xuelan,H	浙江预防医学与疾病监测	tive Medicine and Disea	-	4	15-17	1993	sn1007-0931.1993.04.011
539	伤寒暴发的特征及流行	mic factors of typhoid fev	灿义,廖群力,温汀昭,刘旭	Qunli,Wen Tingzhao,Liu	中国公共卫生	Chinese public health	-	8	353	1993	-
540	水传播一起伤寒暴发流行	ak of typhoid fever trans	李水芸	Li Shuiyun	中国学校卫生	Chinese school health	-	4	222	1993	-
541	起鼠伤寒暴发流行病学调	tigation of an outbreak o	;郭秀英;徐家玲;马文涛;	Xiuying;Xu Jialing;Ma W	中国医院感染学杂志	urnal of Hospital Infectio	-	2	85-86	1993	-
542	雍县伤寒暴发流行中78例	Nayong County, Guizho	何志旭;	He Zhixu;	贵阳医学院学报	al of Guiyang Medical C	-	2	147-149	1993	ki.1000-2707.1993.02.019
543	惠远乡某部伤寒暴发的调	yphoid fever in a certain	林学忠;唐振华;	Xuezhong;Tang Zhenh	人民军医	people's military medicine	-	6	14-15	1993	-
544	站辖区内地方性斑疹伤寒	demic typhus occurred i	张庆奎,张新生,张潮	ngkui,Zhang Xinsheng,Zh	中国人兽共患病杂志	inese Journal of Zoonos	-	2	37	1993	-
545	某部水型伤寒暴发调查	ne typhoid fever outbrea	刘德兴;	Liu Dexing;	解放军预防医学杂志	ve Medicine of the Peopl	-	2	132-133	1993	-
546	疆伤寒沙门氏菌分类和群	sis in classification and pop	文斌,刘敏生,侯惠珍,刘远	Minsheng,Hou Huizhen,Li	中华流行病学杂志	inese Journal of Epidemiol	15	1	42-45	1994	sn.0254-6450.1994.01.117
547	新生儿斯坦利沙门氏菌感	ella Stanley infection in ne	青云,贡桂清,张志华,夏国	Gong Guiqing,Zhang Zhihua	新生儿科杂志	Journal of Neonatology	9	2	64-66	1994	-
548	学特征I.我国部分地区伤	ountryI.Multi-site enzyme	徐文斌,祁国明,刘延清.	Venbin,Qi Guoming,Liu Yan	中华流行病学杂志	inese Journal of Epidemiol	15	4	218-222	1994	sn.0254-6450.1994.04.112
549	特(ASSIUT)地区屠宰动物	SIUT)Salmonella in region	EIN YOUSSEF,林培榆,杨	N YOUSSEF,Lin Peiyu,Yar	肉品卫生	Meat hygiene	-	1	29-30	1994	-
550	2市五种肉品沙门氏菌检	ction status of five types o	任家琰,耿丹,张明行.	iayan,Geng Dan,Zhang Min	动物检疫	animal quarantine	-	2	36-39	1994	-
551	小儿鼠伤寒沙门氏菌感染	ella typhimurium infection	龙庆敏.	Long Qingmin.	传染病信息	fectious disease informati	-	1	20	1994	-
552	污染引起鼠伤寒沙门氏菌	od poisoning caused by co	朱小洁.	Zhu Xiaojie.	上海预防医学杂志	ai Journal of Preventive M	-	7	4	1994	-
553	分离出乙型副伤寒沙门氏	isolated from peritoneal di	郑小明.	Zheng Xiaoming.	江西医学检验	Jiangxi Medical Laboratory	-	4	13	1994	-

554	少门氏菌菌型和志贺氏菌	pes discovered in Henan Pr	周用直.	Zhou Yongzhi.	河南预防医学杂志	n Journal of Preventive Me	-	3	151-154	1994	-
555	22株乙型副伤寒沙门氏菌	n, identification and drug su	郝育英,陈本华.	Hao Yuying,Chen Benhua.	现代中西医结合杂志	grated Traditional Chinese	-	2	19	1994	-
556	占洛克兰沙门氏菌食物中毒	salmonella Brockland food	叶永青,张瑛,倪昔州.	Yongqing,Zhang Ying,Ni Xizh	石河子科技	Shihezi Technology	-	5	55-56,52	1994	-
557	伤寒沙门氏菌食物中毒的调	e of food poisoning caused	李金虎,李平农.	Li Jinhu,Li Pingnong.	安庆医学	Anqing Medicine	15	4	21	1994	-
558	门氏菌引起踝关节感染的	kle joint infection caused b	郭秀琴,刘有莲.	Guo Xiuqin,Liu Youlian.	中国微生态学杂志	inese Journal of Microecolo	-	6	49	1994	-
559	少门氏菌噬菌体分型、质粒	ction and drug resistance o	王世瑜.	Wang Shiyu.	传染病信息	fectious disease informatio	-	2	67	1994	-
560	17株沙门氏菌菌型分布调	on the type distribution of S	李槿年,郭符则.	Li Jinnian,Guo Fuze.	肉品卫生	Meat hygiene	-	10	9-11	1994	-
561	康人群携带沙门氏菌的菌	Salmonella carried by heal	陈培光,金利胜,王炳青.	eiguang,Jin Lisheng,Wang B	浙江预防医学与疾病监测	entive Medicine and Diseas	-	2	5-6	1994	-
562	氏菌属的PCR检测及病原	tPCRDetection and pathog	F MILITARY MEDICINES	TITUTE OF MILITARY M	西南国防医药	west National Defense Me	-	3	146-147,192	1994	-
563	儿鼠伤寒沙门氏菌败血症	atal sepsis caused by Salmo	陈康伟,彭健,赵桂兰,黄荣宁	i,Peng Jian,Zhao Guilan,Hu	新生儿科杂志	Journal of Neonatology	9	2	89	1994	-
564	省87株人源沙门氏菌群型的	tribution of swarm types of	莫美仪,谭海玲.	Mo Meiyi,Tan Hailing.	广东卫生防疫	ong Health and Epidemic Pr	-	3	55-56	1994	-
565	氏菌感染性腹泻5例临床分	neonates5Clinical analysis	七开,黄日瑶,冯瑛,刘正玉,林	eye,Huang Riyao,Feng Ying	泸州医学院学报	rnal of Luzhou Medical Coll	-	3	221-222	1994	-
566	鼠伤寒沙门氏菌食物中毒的	of food poisoning caused by	王智林,蔺娜.	Wang Zhilin,Lin Na.	吉林医学信息	Jilin Medical Information	-	1	-	1994	-
567	鼠伤寒沙门菌感染45例临	a Typhimurium Infection45	徐光铸,宋家芹.	Xu Guangzhu,Song Jiaqin.	蚌埠医学院学报	rnal of Bengbu Medical Col	-	4	317-318	1994	-
568	室鼠伤寒沙门氏菌病暴发	outbreak of Salmonella typ	简鹏,关敬业.	Jian Peng,Guan Jingye.	中华医院感染学杂志	ournal of Hospital Infectiou	-	2	81-82	1994	-
569	沙门氏菌感染22例临床观	Infectiontwenty twoCase cl	杨德芬.	Yang Defen.	石河子医学院学报	rnal of Shihezi Medical Coll	-	1	46-48	1994	-
570	猪霍乱沙门氏菌肠炎12例	suis Salmonella enteritis12	许五云.	Xu Wuyun.	传染病信息	fectious disease informatio	-	1	19-20	1994	-
571	52株沙门氏菌的噬菌体分	hage typing of Salmonella s	小玲,张梦萍,陆佩华,樊招凤	oling,Zhang Mengping,Lu P	中国人兽共患病杂志	Chinese Journal of Zoonose	-	6	19-22	1994	-
572	9~1993年伤寒沙门氏菌的	1993Surveillance of drug re	谭海玲,莫美仪.	Tan Hailing,Mo Meiyi.	广东卫生防疫	ong Health and Epidemic Pr	-	2	72-74	1994	-
573	幼儿鼠伤寒沙门氏菌肠炎9	n enteritis in infants and yo	李敦辉,窦纪霞.	Li Dunhui,Dou Jixia.	开封医专学报	rnal of Kaifeng Medical Col	-	3	216-217	1994	-
574	寒沙门氏菌引起医院感染6	um causes nosocomial infec	孙贵,徐夕兰.	Sun Gui,Xu Xilan.	中华医院感染学杂志	ournal of Hospital Infectiou	-	2	89	1994	-
575	少门氏菌感染86例流行病学	tion in children86Case epid	韩艳荣,麻多,贾亚薇.	an Yanrong,Mado,Jia Yawe	山西妇幼卫生	anxi Maternal and Child Hea	5	2	-	1994	-
576	人业人员的粪便中检出5株	he feces of catering worker	白建棣,刘峥嵘.	Bai Jiandi,Liu Zhengrong.	中国国境卫生检疫杂志	ontier Health and Quarantin	-	S5	156-157	1994	-
577	沙门氏菌的分离鉴定及药	drug susceptibility test rep	徐红军,刘秀杰.	Xu Hongjun,Liu Xiujie.	职业与健康	Occupation and health	-	6	19	1994	-
578	纽波特沙门菌所致鞘膜积	used by endogenous infect	武素梅.	Wu Sumei.	临床检验杂志	journal of clinical testing	-	S1	155-156	1994	-
579	从两例骨髓标本中分离出	two bone marrow specime	桂炳东,贾坤如,徐建民.	Bingdong,Jia Kunru,Xu Jian	江西医学院学报	rnal of Jiangxi Medical Coll	-	4	80	1994	-
580	例鼠伤寒沙门菌感染临床分	of cases of Salmonella typh	跃敏,刘晓东,AFFILIATED	emin,Liu Xiaodong,AFFILI	承德医学院学报	nal of Chengde Medical Co	-	1	14-16	1994	-
581	学特征Ⅰ.我国部分地区伤	untry Ⅰ.Multi-site enzyme	ESE ACADEMY OF PREV	CHINESE ACADEMY OF	中华流行病学杂志	inese Journal of Epidemiolo	-	4	218-222	1994	-
582	门氏菌在新生儿中的一次	lmonella Worthington infe	江,吴坚,文载律,罗燕萍,归	,Wu Jian,Wen Zai Lv,Luo Ya	新生儿科杂志	Journal of Neonatology	9	5	224	1994	-
583	寒沙门菌引起败血症1例及	rd causes sepsis1Examples	胡秀菊,王粉云.	Hu Xiuju,Wang Fenyun.	邯郸医专学报	rnal of Handan Medical Col	-	4	341-342	1994	-
584	市区鼠伤寒沙门氏菌噬菌	lmonella typhimurium in e	志,张梦萍,张淑贞,樊招凤,章	hang Shuzhen,Fan Zhaofen	中华预防医学杂志	se Journal of Preventive Me	-	3	136-139	1994	-
585	肉沙门氏菌污染的探讨及	ention of salmonella contar	夏颖.	Xia Ying.	山东肉类科技	Shandong Meat Technology	-	6	17-18	1994	-
586	年北京伤寒沙门氏菌噬菌	cation of Salmonella Typho	张凤琴,王劲,赵贵苏.	g Fengqin,Wang Jin,Zhao G	中国公共卫生学报	inese Journal of Public Hea	-	4	258	1994	-
587	寒沙门氏菌噬菌体型别及耐	d drug resistance of Salmon	李世竣,田克诚,张王琼.	n,Tian Kecheng,Zhang Wan	贵州医药	Guizhou Medicine	-	2	114-117	1994	-
588	196人变形杆菌与沙门氏菌	196Mixed food poisoning b	张淑华.	Zhang Shuhua.	江苏预防医学	iangsu Preventive Medicine	-	2	42-43	1994	-
589	门氏菌耐药性及其耐药质粒	nce of Salmonella typhi and	黄瑞,穆荣谱.	Huang Rui,Mu Rongpu.	中华传染病杂志	se Journal of Infectious Dis	12	4	202-205,C1	1994	sn.1000-6680.1994.04.105
590	寒沙门氏菌肠炎56例临床	himurium Enteritis56Case	杜曾庆.	Du Zengqing.	医学信息（云南）	Medical Information (Yunnan	-	11	4-6	1994	-

591	少见的哈达尔沙门菌所致	poisoning caused by rare Sa	刘振武,赵瑞霞.	Liu Zhenwu,Zhao Ruixia.	承德医学院学报	nal of Chengde Medical Co	-	1	52-53	1994	-
592	中同时分离出伤寒沙门菌和	were simultaneously isola	马翔,刘汉红.	Ma Xiang,Liu Hanhong.	中华医学检验杂志	urnal of Medical Laborator	17	6	364	1994	-
593	者粪便中分离出一株拉巴	i was isolated from the fec	尹富华,卢彩霞,姜桂香.	uhua,Lu Caixia,ginger cinna	中华医学检验杂志	urnal of Medical Laborator	17	2	123	1994	-
594	菌感染合并中心性浆液性	urium infection complicate	武英,任书云,文克显,洪波	,Ren Shuyun,Wen Kexian,H	中华传染病杂志	se Journal of Infectious Dis	12	2	124	1994	sn.1000-6680.1994.02.134
595	江省沙门氏菌菌型分布与	fication of Salmonella strai	李慧琳,张文娟,孙洁.	Huilin,Zhang Wenjuan,Sun	中华流行病学杂志	inese Journal of Epidemiol	15	1	30	1994	sn.0254-6450.1994.01.112
596	胆汁中分离到布利丹沙门菌	onella bulidan isolated fror	闫淑芬,牛锦华,董良.	Shufen,Niu Jinhua,Dong Li	中华医学检验杂志	urnal of Medical Laborator	17	1	39	1994	-
597	便中同时分离出雷丁沙门菌	um were simultaneously is	利云,何家超,赵翠兰,王兰	,He Jiachao,Zhao Cuilan,W	中华医学检验杂志	urnal of Medical Laborator	17	4	237	1994	-
598	中毒患者粪便中检出雷根	detected in feces of food	郑立,付玉坤,张素艳.	eng Li,Fu Yukun,Zhang Suy	中华医学检验杂志	urnal of Medical Laborator	17	4	239	1994	-
599	败血症型布鲁克沙门氏菌	tal sepsis-type Salmonella	白夷,贾桂清.	Baiyi,Jia Guiqing.	新生儿科杂志	Journal of Neonatology	9	2	87	1994	-
600	寒沙门氏菌院内感染及药	drug susceptibility testing c	张新玲.	Zhang Xinling.	兰后卫生	Hygiene after orchid	-	1	30	1994	-
601	起圣保罗沙门氏菌食物中	Salmonella St. Paul food p	彭亚立.	Peng Yali.	铁道医学	Railway Medicine	-	5	302	1994	-
602	院内鼠伤寒沙门氏菌感染	of Salmonella typhimuriu	李长玉.	Li Changyu.	宁夏医科大学学报	nal of Ningxia Medical Univ	-	4	-	1994	-
603	门氏菌耐药性及其耐药质	ce of Salmonella typhi and	谱,SUZHOU MEDICAL C	Rongpu,SUZHOU MEDIC	中华传染病杂志	se Journal of Infectious Dis	-	4	202-205	1994	-
604	携带肠道致病菌及HBSA	stinal pathogenic bacteria a	子亭,刘振秀,陈志超,杨	g,Liu Zhenxiu,Chen Zhicha	沈阳部队医药	Shenyang Army Medicine	-	1	59-60	1994	-
605	例腹泻患者病原学监测结	urveillance results of patie	英琪,王永全,张宇,罗兴	Wang Yongquan,Zhang Yu,	中国卫生检验杂志	ese Journal of Health Inspe	-	3	172-173	1994	-
606	地区农户鸡病调查及其防	armers in Nanchang area a	张渊魁.	Zhang Yunkui.	江西农业科技	gricultural Science and Te	-	4	46-47	1994	-
607	水源地含水层存活时间的	val time of pathogenic bact	丝梅,唐允吉.	Simei,Tang Yunji.	中国公共卫生	Chinese public health	10	8	342-343	1994	-
608	儿童医院内胃肠道感染临	gastrointestinal infections	吴丽,贾淑泉.	Wu Li,Jia Shuquan.	中原医刊	entral Plains Medical Journ	-	5	4-5	1994	-
609	的鼠伤寒沙门氏菌食物中	typhimurium food poiso	彭,梅景杰,曹承来,朱	Jingjie, Cao Chenglai, Z	中国公共卫生	Chinese public health	-	7	302	1994	-
610	一起伤寒暴发流行的报告	of an outbreak of typhoi	马龙祥	Ma Longxiang	浙江医学	Zhejiang Medicine	-	S1	1	1994	-
611	寒暴发与散发病例菌株药	of typhoid fever outbrea	罗建军,唐文庭,黄明英	un,Tang Wenting,Huang	重庆医学	Chongqing Medicine	-	6	366-367	1994	-
612	伤寒暴发流行事件的调查	nd reflection on a typhoid	陈正清;马武;林霞;	n Zhengqing;Ma Wu;Lin	广西医学	Guangxi Medicine	-	6	530-531	1994	-
613	伤寒暴发的流行病学调查	stigation report on an out	饶浩然,李仁	Rao Haoran, Li Ren	湖北预防医学杂志	Journal of Preventive Me	-	3	1	1994	-
614	德县一次伤寒暴发的调	n outbreak of typhoid fev	王世华,杨秀芸	Vang Shihua, Yang Xiuyu	川省卫生管理干部学院学	uan Health Management	-	3	3	1994	-
615	来女工发生伤寒暴发的调	d fever outbreak among	夏家骠	Xia Jiabiao	上海预防医学杂志	ai Journal of Preventive M	-	8	1	1994	8/j.cnki.sjpm.1994.08.010
616	起水传性伤寒暴发的调查	sis of an outbreak of wa	虹,李慧玲,王新华,	ng, Li Huiling, Wang Xin	中国公共卫生	Chinese public health	-	8	1	1994	-
617	年新余市渝水区伤寒暴发	hoid fever outbreak in Y	刘平华,彭增源,蒋晓	ua, Peng Zengyuan, Jian	疾病监测	disease surveillance	-	6	1	1994	-
618	一起食物型丙型副伤寒暴	eak of food-borne paraty	玉,刘小平,赵荣艳,杨	Xiaoping, Zhao Rongya	疾病监测	disease surveillance	-	6	1	1994	-
619	伤寒暴发后带菌情况调查	cterial carriage after typ	邱闾如,陆致齐,谭速	Jinru, Lu Zhiqi, Tan Suj	疾病监测	disease surveillance	-	6	1	1994	-
620	一起伤寒水型暴发的调查	an outbreak of water-ty	华明贵	Hua Minggui	疾病监测	disease surveillance	-	6	165+168	1994	-
621	起缫丝工人伤寒暴发流行	demic of typhoid fever am	良,陈国忠,吴志明,王	en Guozhong, Wu Zhim	苏州医学院学报	nal of Suzhou Medical Co	-	2	2	1994	-
622	水污染引起伤寒暴发的流	hoid outbreak caused by	智英,刘丹,董众,于志	g, Liu Dan, Dong Zhong	中国公共卫生	Chinese public health	-	4	1	1994	-
623	病学特征:Ⅱ.部分伤寒	a typhi in my country: Ⅱ. S	徐文斌,祁国明,刘延清.	Venbin,Qi Guoming,Liu Yan	中华流行病学杂志	inese Journal of Epidemiol	16	1	29-35	1995	sn.0254-6450.1995.01.114
624	省1554株沙门氏菌菌型鉴	1554Salmonella strain ide	李慧琳,张文娟.	Li Huilin,Zhang Wenjuan.	疾病监测	disease surveillance	10	3	76-80	1995	-
625	门氏菌肠道感染59例临床	nal infection59Example cli	向骏,陈炳锋,苏智军.	g Jun,Chen Bingfeng,Su Zh	中国人兽共患病杂志	Chinese Journal of Zoonose	11	1	61-62	1995	-
626	年深圳市748株沙门氏菌	city748Type distribution	周萍.	Zhou Ping.	中国人兽共患病杂志	Chinese Journal of Zoonose	11	4	56-57	1995	-
627	伤寒沙门氏菌有关生物学	characteristics of Salmonel	任锦玉,李慧琳,程苏云.	n Jinyu,Li Huilin,Cheng Suy	中国卫生检验杂志	ese Journal of Health Inspe	-	2	96-99	1995	-

628	个月婴儿的伤寒沙门氏菌	a typhi brain abscess in a s	邵清.	Shao Qing.	传染病信息	fectious disease informatic	-	1	34-35	1995	-
629	非伤寒沙门氏菌脑膜炎	-typhoid salmonella menin	赵蕊.	Zhao Rui.	传染病信息	fectious disease informatic	-	2	67-68	1995	-
630	病学特征Ⅱ.部分伤寒沙门	a typhi in my country Ⅱ.S	LOGY AND,MICROBIOL	EMIOLOGY AND,MICRO	中华流行病学杂志	inese Journal of Epidemiolo	-	1	29-35	1995	-
631	奇(NOWICH)沙门氏菌引起	NOWICH)food poisoning	任慧敏,王英珍,刘芙玉.	Huimin,Wang Yingzhen,Liu	航空航天医药	Aerospace medicine	-	3	175-176	1995	-
632	鲜牛肉的沙门氏菌检验	lmonella testing of fresh be	钟奇兴.	Zhong Qixing.	肉品卫生	Meat hygiene	-	3	22-23,25,24	1995	-
633	寒沙门氏菌引起的一起食物	isoning caused by Salmone	黄淑常.	Huang Shuchang.	中国卫生检验杂志	ese Journal of Health Inspe	-	4	243-244	1995	-
634	鼠伤寒沙门氏菌感染的粪便	ces of neonates infected w	酆玉瑛.	Feng Yuying.	天津护理	Tianjin Nursing	-	2	67-68	1995	-
635	伤寒流行中分离到不发酵	rbitol was isolated from a t	赵国胜,张德轩.	hao Guosheng,Zhang Dexua	预防医学文献信息	ive medicine literature info	-	2	479-480	1995	-
636	门氏菌属感染75例治疗体	fection75Example treatme	张凡鲜,樊中瑜,张忠居.	anxian,Fan Zhongyu,Zhang	湖南中医杂志	urnal of Traditional Chinese	-	4	31-32	1995	-
637	门氏菌致新生儿腹泻暴发	natal diarrhea outbreaks ca	贾娅.	Jaya.	陕西医学杂志	Shaanxi Medical Journal	-	9	543-544	1995	-
638	顺市区沙门氏菌环境污染调	monella Environmental Poll	李湖录.	Li Hulu.	环境与健康杂志	rnal of Environment and He	-	2	74-76	1995	-
639	门氏菌医院内婴幼儿腹泻	ella Delphi Outbreak of Inf	恩,严铭,刘民,马剑畅,何	ling,Liu Min,Ma Jianchang,	中华医院感染学杂志	ournal of Hospital Infectiou	-	3	163	1995	-
640	布洛克兰沙门氏菌引起的	d poisoning caused by Saln	倪昔洲,叶永青.	Ni Xizhou,Ye Yongqing.	石河子医学院学报	rnal of Shihezi Medical Coll	-	1	40-41	1995	-
641	门氏菌感染并发急性肠穿	ntestinal perforation cause	王万库.	Wang Wanku.	张家口医学院学报	al of Zhangjiakou Medical C	-	2	35-36	1995	-
642	17株沙门氏菌菌型分布调	on the type distribution of	李槿年,郭符则.	Li Jinnian,Guo Fuze.	畜牧与兽医	Husbandry and Veterinary M	-	6	251-252	1995	-
643	康体检粪便中检出504株沙	s during physical examinat	孙凤琪,曾爱芳,李焕枝.	Fengqi,Zeng Aifang,Li Huan	广东卫生防疫	ong Health and Epidemic Pr	-	1	56-58	1995	-
644	伤寒沙门氏菌败血症17例	Typhimurium Septicemia1	季钢,程功,王玉红,万声贤	ng Gong,Wang Yuhong,Wa	临床医学	clinical medicine	-	4	13-14	1995	-
645	阿贡纳沙门氏菌引起的食	poisoning caused by Salmo	石力.	Shi Li.	预防医学文献信息	ive medicine literature info	-	2	315-316	1995	-
646	氏杆菌肠炎并发中毒性脑	itis complicated by toxic en	陈帼英.	Chen Guoying.	张家口医学院学报	al of Zhangjiakou Medical C	-	2	62	1995	-
647	沙门氏菌和空肠弯菌带菌	e of Salmonella and Campy	李槿年.	Li Jinnian.	中国家禽	Chinese poultry	-	4	32	1995	-
648	158株沙门氏菌菌型分布调	igation on the type distribu	,邹桂玲,王秀华,王立新,	iling,Wang Xiuhua,Wang Li	蚌埠医药	Bengbu Medicine	-	1	34-36	1995	-
649	起沙门氏菌食物中毒调查分	nalysis of a case of salmon	张希圣,张凯,李波.	hang Xisheng,Zhang Kai,Li B	实用预防医学	ractical preventive medicin	-	4	235	1995	-
650	伤寒沙门氏菌病922例临床	s Typhimurium922Case clin	周文华,魏艳,吴惠.	ou Wenhua,Wei Yan,Wu H	河南医药信息	Henan Medical Information	-	8	1-3	1995	-
651	纳沙门氏菌食物中毒23例	food poisoningtwenty thre	徐玉良,刘方,季丽艳.	Xu Yuliang,Liu Fang,Ji Liyan	牡丹江医学院学报	al of Mudanjiang Medical C	-	1	57-58	1995	-
652	次从痰液中分离出俾斯倍	ated from sputum for the fi	光续,李伟,郭静珍,姬惠	ngxu,Li Wei,Guo Jingzhen,	安交通大学学报（医学版	an Jiaotong University (Me	-	2	149-150	1995	-
653	生猪宰后沙门氏菌的调查	ion of Salmonella after pig	贾延明,张俊育,林洪强.	ming,Zhang Junyu,Lin Hong	黑龙江畜牧兽医	nimal Husbandry and Veter	-	9	34	1995	-
654	连锁店发生肠炎沙门氏菌	ritidis outbreak occurs at a	芮洪福.	Rui Hongfu.	江苏预防医学	iangsu Preventive Medicin	-	1	32	1995	-
655	(S.LONDON)所致的新生	LONDON)Outbreak of ne	秀兰,高丽娅,韩吉玉,马德	ulan,Gao Liya,Han Jiyu,Ma	安徽预防医学杂志	i Journal of Preventive Me	-	1	36	1995	-
656	寒沙门氏菌引起的食物中毒	e of food poisoning caused	韩成英,余翠玲,董桂英.	hengying,Yu Cuiling,Dong G	安徽预防医学杂志	i Journal of Preventive Me	-	1	81	1995	-
657	诺维奇沙门氏菌引起的食	poisoning caused by Salm	任慧敏,王英珍.	Ren Huimin,Wang Yingzhen	航空航天医药	Aerospace medicine	6	3	175-176	1995	-
658	区1158株沙门氏菌菌型分	igation on the type distribu	瑞萍,王克和,王启顺,龚传	,Wang Kehe,Wang Qishun,	疾病监测	disease surveillance	10	3	80-81	1995	-
659	寒沙门氏菌感染134例临床	imurium Infection134Case	谢玲,余文鑫.	Xie Ling,Yu Wenxin.	南京铁道医学院学报	of Nanjing Railway Medical	-	4	256	1995	-
660	沙门氏菌后在孵化期和出	ing stages after hatching e	-	-	畜禽疾病	vestock and poultry disease	-	1	32	1995	-
661	贤县感染性腹泻细菌谱分	ectrum of infectious diarrh	谢晓红.	Xie Xiaohong.	上海预防医学杂志	hai Journal of Preventive M	-	4	168-169	1995	-
662	安市常见蝇类带菌调查报	on common flies carrying b	刘建华,董淑琴,史玲.	Jianhua,Dong Shuqin,Shi Li	中国卫生检验杂志	ese Journal of Health Inspe	-	6	352-354	1995	-
663	类等海产品的微生物学检	al examination of fish and	书琨,钟耿,何云生,李观	n,Zhong Geng,He Yunshen	中国动物检疫	China Animal Quarantine	-	5	12-13	1995	-
664	一起新生儿医院感染的调查	ion into a neonatal hospita	孟蕾,张广业.	Meng Lei,Zhang Guangye.	疾病监测	disease surveillance	10	2	49	1995	-

665	一起院内感染的调查分析	and analysis of a nosocom	宋振如,陈文萍.	ong Zhenru,Chen Wenping	蚌埠医药	Bengbu Medicine	-	2	35	1995	-
666	医院内感染64例临床分析	ial infection64Case clinical	何书.	He Shu.	开封医专学报	rnal of Kaifeng Medical Col	-	2	82-83	1995	-
667	体加工肉制品细菌卫生学i	estigation of Individual Pro	任家琰,王建寿,苏华,耿丹	n,Wang Jianshou,Su Hua,G	肉品卫生	Meat hygiene	-	5	6-8	1995	-
668	菌性食物中毒的危害度分	the hazards of bacterial foo	吴涌兴,孙瑞兴.	Wu Yongxing,Sun Ruixing.	江苏预防医学	iangsu Preventive Medicin	-	4	27-28	1995	-
669	鲜鸡蛋细菌污染情况的调	terial contamination of co	彦明,贾靖国,阎祖书,孙冰	ming,Jia Jingguo,Yan Zushu,	肉品卫生	Meat hygiene	-	9	9-11	1995	-
670	血培养83例阳性分析	d culture83case positive an	黄莺.	Oriole.	广后医学	Hiroshi Medicine	-	4	349	1995	-
671	食卤虾引起的食物中毒的i	ase of food poisoning cause	杨会芹.	Yang Huiqin.	职业与健康	Occupation and health	-	3	38-39	1995	-
672	北京地区的细菌性腹泻	cterial diarrhea in Beijing a	虞爱华.	Yu Aihua.	传染病信息	fectious disease informati	-	3	108	1995	-
673	起因误诊引起伤寒暴发的	phoid outbreak caused b	杨仕励，罗小华	Yang Shili, Luo Xiaohua	新医学	new medicine	-	S1	1	1995	-
674	留水村水型伤寒暴发流行i	rne typhoid fever outbre	戴和波	Dai Hebo	实用预防医学	actical preventive medici	-	4	2	1995	-
675	食物型伤寒暴发流行调查	eport on an outbreak of f	李中华，龙碧霞	Li Zhonghua, Long Bixia	实用预防医学	actical preventive medici	-	4	1	1995	-
676	甲型副伤寒暴发流行的调	s of outbreaks of typhoid	刘巧突，沈喆，王炳武	aotu, Shen Zhe, Wang B	解放军预防医学杂志	ve Medicine of the Peopl	-	6	3	1995	-
677	井水污染引起伤寒暴发流	eak of typhoid fever cau	潮,徐建人,项惠,虞昌根,颜	anren,Xiang Hui,Yu Char	浙江预防医学	ejiang Preventive Medici	-	5	6-7	1995	sn1007-0931.1995.05.006
678	一起水型伤寒暴发调查	an outbreak of waterbo	袁兆虎	Yuan Zhaohu	江苏预防医学	angsu Preventive Medici	-	3	2	1995	-
679	S-PHA在伤寒暴发现场的	ion at the scene of typho	张恩惠,王国荣	hang Enhui,Wang Guoro	浙江预防医学	ejiang Preventive Medici	-	4	56-57	1995	sn1007-0931.1995.04.039
680	蒙古族自治县伤寒暴发流	typhoid fever in Henan	李承宁，仁青多杰，梁晓	ing, Renqing Duojie, Lia	青海医药杂志	Qinghai Medical Journal	-	7	2	1995	-
681	合型斑疹伤寒暴发流行的i	report on an outbreak of	贞莉，韩红，白淑美，梅	Han Hong, Bai Shumei	山西医学院学报	nal of Shanxi Medical Co	-	2	2	1995	-
682	沙垦制造厂伤寒暴发流行	yphoid fever in Shabu Ma	骆云德，谢显请	nde, thank you for your ir	实用预防医学	actical preventive medici	-	2	1	1995	-
683	民族地区中小学伤寒暴发	mary and secondary sch	,杨刚元,杨胜富,覃章世,李	uan,Yang Shengfu,Qin Z	中国学校卫生	Chinese school health	-	1	36	1995	-
684	管理所伤寒暴发的流行病	typhoid fever outbreak i	明，彭志强，吴辉绪，李	Peng Zhiqiang, Wu Huix	解放军预防医学杂志	ve Medicine of the Peopl	-	1	2	1995	-
685	起水型伤寒暴发流行的调	an outbreak of waterbo	李兴无，焦金鸿，郑国贤	u, Jiao Jinhong, Zheng	解放军预防医学杂志	ve Medicine of the Peopl	-	1	2	1995	-
686	鼠伤寒沙门菌感染现状及	ituation of Salmonella Typ	周刚,茹维萍,张锦,傅炳南	u,Ru Weiping,Zhang Jin,Fu	中华流行病学杂志	inese Journal of Epidemiol	17	5	268-271	1996	sn.0254-6450.1996.05.106
687	伤寒沙门菌噬菌体型及耐	g Resistance of Salmonella	陈亢川,谢一俊,郑国魁.	angchuan,Xie Yijun,Zheng	中华流行病学杂志	inese Journal of Epidemiol	17	5	261-263	1996	sn.0254-6450.1996.05.103
688	岸进口冻禽肉沙门氏菌监	lmonella in imported froze	张东亮,吴国华.	hang Dongliang,Wu Guohu	旅行医学科学	travel medical science	2	1	31-32	1996	-
689	起鼠伤寒沙门氏菌食物中	lmonella typhimurium foo	朱争鸣.	Zhu Zhengming.	安徽预防医学杂志	i Journal of Preventive Me	-	3	72	1996	-
690	1982~1991年沙门氏菌菌	91Distribution of Salmone	素梅,刘庆文,刘善萍,贾云	iu Qingwen,Liu Shanping,Ji	临沂医专学报	urnal of Linyi Medical Colle	-	1	19-22	1996	-
691	伤寒沙门氏菌感染91例临	ium infection in children91	林秋君,程贤芬,林丽珍.	iujun,Cheng Xianfen,Lin Li	福建医药杂志	Fujian Medical Journal	-	5	27-28	1996	-
692	寒沙门氏菌所致食物中毒	e of food poisoning caused	玉泉,王振军,武福周,郭宝	Wang Zhenjun,Wu Fuzhou	中国卫生检验杂志	ese Journal of Health Inspe	-	2	86-87	1996	-
693	物中毒菌株的质粒图谱及	ce spectrum analysis of Sal	叶梅君,余倩,陈冬青,韩宇	eijun,Yu Qian,Chen Dongqi	现代预防医学	modern preventive medicin	-	2	19-21,34	1996	-
694	氏菌肠炎一次暴发流行(	enteritis outbreak(attache	黄凯风,赵焕喜.	Huang Kaifeng,Zhao Huanxi	实用医学杂志	ournal of Practical Medicin	-	1	38	1996	-
695	业人员中检出一株威斯顿	n was detected among wo	蔡卫红.	Cai Weihong.	广东卫生防疫	ong Health and Epidemic Pr	-	1	71	1996	-
696	鼠伤寒沙门氏菌感染30例	Typhimurium Infection30	王泝,王玉国.	Wang Su,Wang Yuguo.	临沂医专学报	urnal of Linyi Medical Colle	-	3	265-266	1996	-
697	伤寒沙门氏菌感染44例临	ium infection in children44	王晶.	Wang Jing.	临床医学	clinical medicine	-	8	20-21	1996	-
698	哥纳沙门氏菌引起的食物	soning Caused by Salmone	高志慧.	Gao Zhihui.	江苏预防医学	iangsu Preventive Medicin	-	3	40	1996	-
699	非伤寒沙门氏菌属感染16	al Salmonella spp. infectio	朱亚和,秦亚娟.	Zhu Yahe,Qin Yajuan.	交通医学	traffic medicine	-	2	107-108	1996	-
700	沙门氏菌68株药敏的	salmonella68drug-sensitive	倪丽萍.	Ni Liping.	南通医学院学报	nal of Nantong Medical Co	16	1	115	1996	-
701	鸡沙门氏菌引起一起食物	lla in turkey causes food p	饶道平,卢德俊.	Rao Daoping,Lu Dejun.	安徽预防医学杂志	i Journal of Preventive Me	-	2	55-56	1996	-

702	床鼠伤寒沙门氏菌药敏试验	ty test report of Salmonella	王进,周文华.	Wang Jin,Zhou Wenhua.	河南医药信息	Henan Medical Information	-	8	9-10	1996	-
703	科住院病员的鼠伤寒沙门	phimurium infection in a pe	胡波,朱守法,聂燕子.	Hu Bo,Zhu Shoufa,Nie Yanzi	安徽医科大学学报	nal of Anhui Medical Unive	-	2	123-125	1996	-
704	出一株阿哥纳沙门氏菌及药	a Agona from ascites fluid a	郭厚慧.	Guo Houhui.	江西医学检验	Jiangxi Medical Laboratory	-	4	24	1996	-
705	病牛沙门氏菌食物中毒调查	a case of salmonella food	朱芳麟,冯忠生,赵邦斌,赵	n,Feng Zhongsheng,Zhao B	青海医药杂志	Qinghai Medical Journal	-	9	58	1996	-
706	鼠伤寒沙门氏菌感染临床	of cases of Salmonella typ	里景维,杨亚萍.	Li Jingwei,Yang Yaping.	甘肃科技	ansu Science and Technolo	-	S1	121	1996	-
707	进境动物产品检出沙门氏菌	ins detected in imported a	超,沈孝明,陈枝华,程彬,黄	Xiaoming,Chen Zhihua,Ch	中国兽医杂志	Chinese Veterinary Journal	-	6	54-55	1996	-
708	沙门菌Vi-Ⅱ噬菌体型及其a	Vi-ⅡBacteriophage type	,邹志英,屠静,刘桂荣,张庆	Zhiying,Tu Jing,Liu Guirong	中华流行病学杂志	inese Journal of Epidemiolo	17	5	289-291	1996	sn.0254-6450.1996.05.118
709	鼠伤寒沙门氏菌噬菌体分型	of Salmonella typhimurium	姚文清,卢春明.	Yao Wenqing,Lu Chunming	中国微生态学杂志	inese Journal of Microecolo	8	1	36-39	1996	-
710	伤寒沙门氏菌污染造成的	caused by Salmonella Typh	刘俊荣.	Liu Junrong.	医学动物防制	medical animal control	-	3	57-58	1996	-
711	阿哥纳沙门氏菌引起329人	nd Salmonella Agona329R	永才,肖桂芳,马忠礼,郭素	i,Xiao Guifang,Ma Zhongli,	现代中西医结合杂志	grated Traditional Chinese	-	1	24-25	1996	-
712	株沙门氏菌菌型分布情况	of the distribution of Salm	黄美婵,林世光,曾财娣.	Meichan,Lin Shiguang,Zen	中国卫生检验杂志	ese Journal of Health Inspe	-	6	337-340	1996	-
713	儿鼠伤寒沙门氏菌败血症4	lla Typhimurium Septicem	李先蓉.	Li Xianrong.	实用医学进修杂志	Practical Medical Continuin	24	1	48-49	1996	-
714	起布利丹沙门菌食物中毒	f Salmonella bulidan food	潘怀欢,黄兴,梁恩炽,杨淮海	n,Huang Xing,Liang Enchi,Y	广东卫生防疫	ong Health and Epidemic Pr	22	2	72-73	1996	-
715	儿沙门菌感染58例临床分	fection in children58Case c	董丽,张元明,何涓.	g Li,Zhang Yuanming,He Ju	贵阳医学院学报	nal of Guiyang Medical Col	-	2	72-73	1996	-
716	罗沙门氏菌引起食物中毒	case of food poisoning caus	刘义先.	Liu Yixian.	青海医药杂志	Qinghai Medical Journal	26	9	54-55	1996	-
717	伤寒沙门氏菌感染25例临	Typhimurium Infection25C	张可霞.	Zhang Kexia.	中国冶金工业医学杂志	rnal of Metallurgical Indust	-	3	162	1996	-
718	儿鼠伤寒沙门氏菌肠炎11	n enteritis in children in wi	史靖邦,王子林.	Shi Jingbang,Wang Zilin.	适宜诊疗技术	e diagnostic and treatment	14	3	7-8	1996	-
719	起鼠伤寒沙门氏菌食物中	lmonella typhimurium foo	黄仁兴.	Huang Renxing.	宜春医药	Yichun Medicine	-	27	32-33	1996	-
720	寒沙门菌食物中毒180例临	urium food poisoning180C	兰宁,杨萍,黄邦和,黄德珑	ang Ping,Huang Banghe,Hu	中国农村医学	Chinese rural medicine	-	7	23-24	1996	-
721	人鼠伤寒沙门菌败血症一	onella typhimurium septice	程德云,陈文彬.	Cheng Deyun,Chen Wenbin	中华内科杂志	ese Journal of Internal Med	35	7	500	1996	-
722	用隔夜饭引起的沙门氏菌	oning caused by eating ove	邓明,朱逸明,陈志礼.	g Ming,Zhu Yiming,Chen Z	广东卫生防疫	ong Health and Epidemic Pr	-	3	49-50	1996	-
723	氏菌感染引起新生儿腹泻的	f neonatal diarrhea caused	佟彤,张子惠.	Tong Tong,Zhang Zihui.	天津医药	Tianjin Medicine	-	8	460-463	1996	-
724	儿鼠伤寒沙门氏菌感染的	onella typhimurium infecti	申田,段满乐,杨晋榕,张新兰	uan Manle,Yang Jinrong,Zh	长治医学院学报	nal of Changzhi Medical Co	-	1	42	1996	-
725	肥西县沙门氏菌型分布调查	distribution of Salmonella	周心民,李钰.	Zhou Xinmin,Li Yu.	安徽预防医学杂志	i Journal of Preventive Med	-	4	55-56	1996	-
726	门氏菌引起的暴发感染和	by Salmonella Worthingto	民,吴坚,张有江,罗燕萍,谢	u Jian,Zhang Youjiang,Luo Y	传染病信息	fectious disease informatio	-	2	68-69	1996	-
727	纳沙门氏菌感染32例临床	agona infection32Case clin	姜天俊,虞爱华.	Jiang Tianjun,Yu Aihua.	传染病临床与研究	ous Disease Clinical and Re	9	2	19-20	1996	-
728	沙门氏菌引起的食物中毒	case of food poisoning cau	金玲,卢延玲,高建民,马作	g,Lu Yanling,Gao Jianmin,M	中国卫生检验杂志	ese Journal of Health Inspe	-	5	290-293	1996	-
729	州市159株沙门氏菌菌型	Investigation on the type d	徐勉华,刘周.	Xu Mianhua,Liu Zhou.	工企医刊	ial and Enterprise Medical	-	1	149-150	1996	-
730	伤寒沙门氏菌食物中毒的	is of Cases of Salmonella T	李馨,吴庆恩.	Li Xin,Wu Qingen.	吉林医学信息	Jilin Medical Information	-	11	49-50	1996	-
731	血(骨髓)沙门氏菌培养结果	(marrow)Salmonella cultur	刘志斌.	Liu Zhibin.	青海医药杂志	Qinghai Medical Journal	-	12	51	1996	-
732	寒沙门氏菌噬菌体分型和	nce detection of Salmonel	刘兰珍,关冰,赵克义.	Lanzhen,Guan Bing,Zhao K	预防医学文献信息	ive medicine literature info	-	1	57	1996	-
733	沙门菌性腹泻菌型分布和药	acterial type distribution a	王继远.	Wang Jiyuan.	中华医学检验杂志	urnal of Medical Laborator	-	4	51	1996	-
734	内鼠伤寒沙门氏菌肠炎暴	nurium enteritis outbreak i	曾白莹.	Zeng Baiying.	厂矿医药卫生	ry and mine medicine and	-	1	22	1996	-
735	鼠伤寒沙门菌感染现状及	ituation of Salmonella Typ	ALHYGIENEEPIDEMICP	OUINCIALHYGIENEEPI	中华流行病学杂志	inese Journal of Epidemiolo	-	5	13-16	1996	-
736	刺液中分离出一株猪霍乱沙	oleraesuis was isolated fro	彭卫华.	Peng Weihua.	江西医学检验	Jiangxi Medical Laboratory	-	1	32	1996	-
737	株沙门氏菌药敏试验结果	susceptibility test results o	蒋卫平,马志俊,陈晓红.	Veiping,Ma Zhijun,Chen Xia	江苏预防医学	iangsu Preventive Medicin	-	3	38-40	1996	-
738	儿童鼠伤寒沙门菌感染14	nfection in children in the h	徐艳云,毛海洲.	Xu Yanyun,Mao Haizhou.	人民军医	people's military medicine	-	9	36-37	1996	-

739	患者粪便中检出布伦登芦	donloop from the feces of	周爱荣,张安军.	Zhou Airong,Zhang Anjun.	中华流行病学杂志	inese Journal of Epidemiolo	17	2	98	1996	sn.0254-6450.1996.02.116
740	儿脓血便中检出圣胡安沙	la San Juan in infants' puru	姜秀云,计国欣.	Jiang Xiuyun,Ji Guoxin.	中华医学检验杂志	urnal of Medical Laborator	19	3	144	1996	-
741	门菌性腹泻菌型分布和药	acterial type distribution a	王继远.	Wang Jiyuan.	中华医学检验杂志	urnal of Medical Laborator	19	4	246	1996	-
742	91～1995年）的分离鉴定	Year) isolation, identificati	屠金娟.	Tu Jinjuan.	中华传染病杂志	se Journal of Infectious Dis	14	3	187-188	1996	sn.1000-6680.1996.03.132
743	沙门菌致腹腔多发性脓肿	in the abdominal cavity ca	淑秀,金友进,王学斑,关沙	iu,Jin Youjin,Wang Xueban	中华医学检验杂志	urnal of Medical Laborator	19	1	27	1996	-
744	马流产沙门菌引起食物中	oning caused by salmonella	董爱萍,李作华.	Dong Aiping,Li Zuohua.	中华医学检验杂志	urnal of Medical Laborator	19	2	102	1996	-
745	门氏菌所致新生儿流行性	demic diarrhea caused by S	时淑宁,靳国强.	Shi Shuning,Jin Guoqiang.	宁夏医科大学学报	al of Ningxia Medical Univ	-	1	-	1996	-
746	伤寒沙门氏菌属的医院内感	ections with Salmonella typ	刘新贵,刘立.	Liu Xingui,Liu Li.	中国公共卫生管理	ese public health managen	-	2	147-148	1996	-
747	性食物中毒137例临床分	d poisoning137Case clinica	段光琦,李建东.	Duan Guangqi,Li Jiandong.	张家口医学院学报	al of Zhangjiakou Medical C	-	2	38	1996	-
748	市市区松花江水细菌污染	ntamination of Songhua R	景伟力,张季娟,张亚男.	Weili,Zhang Jijuan,Zhang Ya	中国卫生检验杂志	ese Journal of Health Inspe	-	6	372	1996	-
749	行业从业人员肠道致病菌	bacteria among employees	葛健民.	Ge Jianmin.	中国卫生检验杂志	ese Journal of Health Inspe	-	1	42-43	1996	-
750	流行菌株的耐药谱、噬菌体	d analysis of epidemic stra	川,王南志,余倩,叶梅君,周	Vang Nanzhi,Yu Qian,Ye Me	中国卫生检验杂志	ese Journal of Health Inspe	-	6	333-334	1996	-
751	92-1995年鸡病流行特点和	racteristics and prevention	根成,杜元钊,朱万光,刘佩	,Du Yuanzhao,Zhu Wangua	山东畜牧兽医	mal Husbandry and Veteri	-	1	14-16	1996	-
752	见的致病菌对药物的敏感性	common chicken pathoge	徐仕忠.	Xu Shizhong.	中国家禽	Chinese poultry	-	9	14	1996	-
753	性食物中毒137例临床分	d poisoning137Case clinica	段光琦,李建东.	Duan Guangqi,Li Jiandong.	张家口医学院学报	al of Zhangjiakou Medical C	-	1	38	1996	-
754	起伤寒爆发流行的病原学	n of the etiology of a typho	甘莉萍,陈应坚,江锐曙.	iping,Chen Yingjian,Jiang R	广东卫生防疫	ng Health and Epidemic Pr	-	2	41-42	1996	-
755	一起伤寒暴发流行	An outbreak of typhoid feve	张成寿,巫后松.	ang Chengshou,Wu Houso	安徽预防医学杂志	i Journal of Preventive Me	-	3	69	1996	-
756	建筑工地伤寒暴发流行病学	ion of a typhoid fever outb	开国,储全胜,陶秀莲,张西	u Quansheng,Tao Xiulian,Z	预防医学文献信息	ive medicine literature info	-	2	113-114	1996	-
757	内熟肉食品受致病菌污染	eat products by pathogenic	郝长利.	Hao Changli.	实用预防医学	ractical preventive medicin	-	2	93	1996	-
758	腹泻患者病原菌调查	pathogenic bacteria in patie	孙家立,吴纯信.	Sun Jiali,Wu Chunxin.	安徽预防医学杂志	i Journal of Preventive Me	-	3	99-100	1996	-
759	我国腹泻病调查研究现况	ions and research on diarr	王进平,王志友.	Wang Jinping,Wang Zhiyou	预防医学文献信息	ive medicine literature info	-	3	221-222	1996	-
760	例腹泻病粪便培养调查分	alysis of Stool Culture in Ca	马江红.	Ma Jianghong.	实用医技杂志	l of Practical Medical Tech	-	7	489-490	1996	-
761	一起食物中毒调查报告	tigation report on food pois	李伟,谢杰,马兵.	Li Wei,Xie Jie,Horse soldiers	安徽预防医学杂志	i Journal of Preventive Me	-	3	67-68	1996	-
762	分饮食业从业人员健康带菌	atus of some catering indu	卓玛,于德江,尼玛彭多,陈	a,Yu Dejiang,nima pendo,	西藏医药杂志	Tibetan Medical Journal	-	S1	50-51	1996	-
763	县住院腹泻患者致泻病原	hospitalized patients with	海,王洪如,蒋成峰,沈钢建	Hongru,Jiang Chengfeng,S	中华流行病学杂志	inese Journal of Epidemiolo	17	5	272-274	1996	sn.0254-6450.1996.05.108
764	胚细菌分离及消毒剂抑菌	ad duck embryos and antib	俊,王琴,周廷宣,沙莎,徐兴	Vang Qin,Zhou Tingxuan,Sh	教师教育学报	ournal of Teacher Educatio	-	3	26-28	1996	-
765	起食物型伤寒暴发流行的调	an outbreak of food-bor	林凤池	Lin Fengchi	上海预防医学杂志	ai Journal of Preventive M	-	11	2	1996	8/j.cnki.sjpm.1996.11.035
766	匀洒洋村伤寒暴发流行分	yphoid fever outbreak in	莫洪群	mohongqun	黔南民族医专学报	Qiannan National Medic	-	4	38-39	1996	-
767	古市第四中学伤寒暴发调查	ever outbreak in No. 4 M	莉，王二梅，王新生，徐	ng Ermei, Wang Xinshen	疾病监测	disease surveillance	-	10	2	1996	-
768	一例伤寒暴发流行调查报	n report on 11 cases of t	朱丽芳	Zhu Lifang	疾病监测	disease surveillance	-	10	1	1996	-
769	古市第四中学伤寒暴发调查	id fever outbreak in No. 4	莉，苏桂枝，王二梅，王	Su Guizhi, Wang Ermei,	河南预防医学杂志	Journal of Preventive M	-	5	2	1996	-
770	三江县伤寒暴发的调查	outbreak of typhoid feve	宋作新,吴自林,杨林清,胡起	oxin,Wu Zilin,Yang Linqi	广西预防医学	uangxi Preventive Medici	-	5	1	1996	-
771	入性乙型副伤寒暴发的调	n an outbreak of incomin	平，王获章，王章云，胡	Huozhang, Wang Zhang	中国公共卫生	Chinese public health	-	9	1	1996	-
772	县看守所伤寒暴发调查报	phoid fever outbreak in Ji	孙志忠，于凤太，贾艳合	Zhizhong, Yu Fengtai, J	疾病监测	disease surveillance	-	6	2	1996	-
773	里河局伤寒暴发流行的调	e outbreak of typhoid fev	陈晓波	Chen Xiaobo	内蒙古医学杂志	er Mongolia Medical Jou	-	3	1	1996	-
774	大学发生一起伤寒暴发流行	break of typhoid fever in	世雄，夏伟，陈坚，黄鸿	, Xia Wei, Chen Jian, Hu	实用预防医学	actical preventive medici	-	2	2	1996	-
775	一起由洪灾引起的伤寒暴发	k of typhoid fever cause	令,古绍文,梁建中,石新玉,王	haowen,Liang Jianzhong	广西预防医学	uangxi Preventive Medici	-	3	2	1996	-

776	起食物型伤寒暴发流行调	on of an outbreak of food	李中华，潘沫清	Li Zhonghua, Pan Moqing	疾病监测	disease surveillance	-	4	1	1996	-
777	县地方性斑疹伤寒暴发流行	mic typhus outbreak in H	刘有盛，陈红梅	Yousheng, Chen Hongm	山西医药杂志	Shanxi Medical Journal	-	2	2	1996	-
778	起溪水污染引起的副伤寒暴	ratyphoid caused by stre	杨林清，吴自林，荣作新	Linqing, Wu Zilin, Rong Z	广西预防医学	uangxi Preventive Medici	-	2	2	1996	-
779	和日常生活型伤寒暴发流	n outbreak of fly-type and	赵世年，郭夏亭	Zhao Shinian, Guo Xiating	河南预防医学杂志	Journal of Preventive M	-	1	1	1996	-
780	行的经济损失及预防保健	reventive health care ser	傅若宇,傅飞,邓庆昌	uoyu,Fu Fei,Deng Qingc	中国基层医学	primary medicine in china	-	1	1	1996	-
781	~1995年伤寒沙门氏菌病	urveillance study on the pat	张政,梅玲玲.	Zhang Zheng,Mei Lingling.	疾病监测	disease surveillance	12	5	165-167	1997	-
782	岸出境水产品检出沙门氏	nonella in outbound aquati	周向阳,沈彪.	Zhou Xiangyang,Shen Biao.	旅行医学科学	travel medical science	3	1	40,46	1997	-
783	伤寒沙门氏菌食物中毒调	a case of Salmonella typhi	金志善,梁玉梅.	Kim Ji Sun,Liang Yumei.	山东预防医学	handong Preventive Medici	17	1	34	1997	-
784	一起沙门氏菌致食物中毒	food poisoning caused by S	吴松涛.	Wu Songtao.	安徽预防医学杂志	i Journal of Preventive Med	-	4	60	1997	-
785	株鼠伤寒沙门氏菌药物敏	ceptibility test of Salmonel	纪惠玲,何丹华.	Ji Huiling,He Danhua.	广东卫生防疫	ong Health and Epidemic Pr	-	4	12-13	1997	-
786	门氏菌属和志贺氏菌属带	nella and Shigella carriage	谢晓红.	Xie Xiaohong.	上海预防医学杂志	hai Journal of Preventive M	-	5	210-211	1997	-
787	江市甘棠湖沙门菌污染调	nella contamination in Gan	邵丽君,龚天恩.	Shao Lijun,Gong Tianen.	九江医学	Jiujiang Medical	-	4	55-56	1997	-
788	伤寒沙门氏菌食物中毒的调	e of food poisoning caused	石改乔,靳增民.	Shi Gaiqiao,Jin Zengmin.	职业与健康	Occupation and health	-	2	21-22	1997	-
789	沙门氏菌引起院内感染调	osocomial infections cause	成可经,陶源勇,苏秀霞.	g Kejing,Tao Yuanyong,Su X	潍坊医学院学报	nal of Weifang Medical Col	-	1	51-52	1997	-
790	血液中检出都柏林沙门氏菌	olin detected in infant bloo	周小平.	Zhou Xiaoping.	厂矿医药卫生	ry and mine medicine and h	-	1	49	1997	-
791	伤寒沙门氏菌感染36例临	a Typhimurium Infection36	袁向尚,李丽.	Yuan Xiangshang,Li Li.	开封医专学报	rnal of Kaifeng Medical Col	-	1	13-15	1997	-
792	沙门氏菌属食物中毒70例	onella food poisoning70exa	朱文崇.	Zhu Wenchong.	广东医学	Guangdong Medicine	18	2	135	1997	-
793	寒沙门氏菌引起的食物中	case of food poisoning caus	张玉妹,刘德宗,曾照常,王	Dezong,It was business as	齐齐哈尔医学院学报	rnal of Qiqihar Medical Coll	-	2	144	1997	-
794	吃马齿苋引起的沙门氏菌食	onella food poisoning caus	郝安勤,武红学.	Hao Anqin,Wu Hongxue.	实用医技杂志	l of Practical Medical Tech	4	2	159-160	1997	-
795	起沙门氏菌食物中毒的调	into a case of salmonella fo	姜树强,张建玲.	ang Shuqiang,Zhang Jianlin	航空军医	aviation medic	-	2	115	1997	-
796	株伤寒沙门氏菌药物敏感	drug susceptibility test res	蔡卫平,刘小伟,唐小平.	eiping,Liu Xiaowei,Tang Xia	广东医学	Guangdong Medicine	-	3	201-202	1997	-
797	沙门氏感染肺炎型10例临	n Infection Pneumonia Typ	会福,高位年,宋美娟,马建	h position year,Song Meiju	医学动物防制	medical animal control	-	4	232-233	1997	-
798	起鼠伤寒沙门氏菌致食物中	oisoning caused by Salmone	李金虎,张萍.	Li Jinhu,Zhang Ping.	安徽预防医学杂志	i Journal of Preventive Med	-	1	52	1997	-
799	菌感染暴发流行与院内感	outbreak and nosocomial in	朱家龙,刘竹君.	Zhu Jialong,Liu Zhujun.	安徽预防医学杂志	i Journal of Preventive Med	-	2	101-102	1997	-
800	沙门氏菌(S.auona)食物中	gether(S.auona)food poiso	肖绍明,林剑琴.	Xiao Shaoming,Lin Jianqin.	福建医药杂志	Fujian Medical Journal	19	1	73-74	1997	-
801	1975~1994年沙门氏菌监	5~1994Annual Salmonella	曹国林,李克光.	Cao Guolin,Li Keguang.	中国公共卫生	Chinese public health	-	7	16	1997	-
802	门氏菌引起的食物中毒63	caused by Salmonella spp.6	张小明.	Zhang Xiaoming.	实践医学杂志	ournal of Practical Medicin	10	2	-	1997	-
803	品从业人员沙门氏菌带菌	lla carrier status among foo	周支军.	Zhou Zhijun.	江苏预防医学	iangsu Preventive Medicine	-	4	37-38	1997	-
804	食品引起的沙门氏菌食物	ella food poisoning caused	熊谷进,路新国.	Xiong Gujin,Lu Xinguo.	中国烹饪	Chinese cooking	-	7	23	1997	-
805	氏菌肠炎败血症伴化脓性	ed by purulent meningitis c	钟绍文.	Zhong Shaowen.	桂林医学杂志	Guilin Medical Journal	13	3	256	1997	-
806	门氏鼠伤寒杆菌食物中毒	a case of salmonella typhi	毛秀芳,董红英.	Mao Xiufang,Dong Hongying	山东预防医学	handong Preventive Medici	17	3	143-144	1997	-
807	D群引起食物中毒爆发的	gical analysis of food poiso	华,何志辉,彭义森,韩金	ua,He Zhihui,Peng Yisen,H	广东卫生防疫	ong Health and Epidemic Pr	-	1	28-29	1997	-
808	年嘉定区从业人员沙门氏	tection analysis among em	章齐平.	Zhang Qiping.	现代预防医学	modern preventive medicin	-	2	78-79,70	1997	-
809	沙门氏菌引起食物中毒的	ion of food poisoning caus	肖世泽.	Xiao Shize.	中国卫生检验杂志	ese Journal of Health Inspe	-	4	249	1997	-
810	贝塔沙门菌败血症一例	f septicemia due to Salmon	解晓珍,潘绍武,张中奎.	zhen,Pan Shaowu,Zhang Zh	中华医学检验杂志	urnal of Medical Laborator	20	4	251	1997	-
811	肠炎沙门菌肝脓肿一例	abscess caused by Salmon	义,郝淑媛,王万俊,袁玲,杜	uyuan,Wang Wanjun,Yuan	中华传染病杂志	se Journal of Infectious Dis	15	1	2	1997	sn.1000-6680.1997.01.102
812	脓肿中检出切斯特沙门菌	on of Salmonella chester fr	朱晓辉,王德春,朱忠勇.	hui,Wang Dechun,Zhu Zho	中华医学检验杂志	urnal of Medical Laborator	20	6	360	1997	-

813	门菌致糖尿病患者肌肉脓	ss in a diabetic patient cau	陈道雄,高勇义,贾杰.	n Daoxiong,Gao Yongyi,Jia	中华传染病杂志	se Journal of Infectious Dis	15	1	19	1997	sn.1000-6680.1997.01.109
814	便和血液中同时检出新港	on of Salmonella Newport	何竞,牟兆钦.	He Jing,Mou Zhaoqin.	中华医学检验杂志	urnal of Medical Laborator	20	6	369	1997	-
815	双城市鸡病流行调查报告	n chicken disease epidemi	余水秋,张春贷,温元臣,刘	Zhang Chundai,Wen Yuanc	黑龙江畜牧兽医	nimal Husbandry and Veter	-	6	28-29	1997	-
816	合型致病菌感染引起食物	ing caused by mixed patho	黄平,何光立,陈修兰.	ng Ping,He Guangli,Chen Xi	广东卫生防疫	ong Health and Epidemic Pr	-	4	56-57	1997	-
817	及近郊三条主要河流细菌	on in three major rivers in	李永念,左丽,唐俊杰,佘晓	ian,Zuo Li,Tang Junjie,She	贵阳医学院学报	nal of Guiyang Medical Col	-	3	36-38	1997	-
818	性腹泻患者病原学监测结	veillance results of patient	绿,张永刚,黄青青,汪萍,冯	onggang,Huang Qingqing,W	中国卫生检验杂志	ese Journal of Health Inspe	-	3	188-189	1997	-
819	996年部分肠杆科菌耐药	esistance spectrum of som	骆明波,刘晓白,林智.	o Mingbo,Liu Xiaobai,Lin Z	湖南医学	Hunan Medicine	-	S1	92	1997	-
820	03例鼠伤寒及医院感染的	s of cases of typhoid fever	马景淑,王文珍,贺美兰.	gshu,Wang Wenzhen,He M	河南医药信息	Henan Medical Information	-	6	9-10	1997	-
821	细菌病的流行特点和控制	control methods of bacte	,朱万光,范根成,刘佩兰,	nguang,Fan Gencheng,Liu	禽业科技	Poultry Technology	-	1	5-7	1997	-
822	起食物中毒病原菌的检验	sis of pathogenic bacteria	程苏云,任锦玉,杨德珍.	g Suyun,Ren Jinyu,Yang De	浙江预防医学	hejiang Preventive Medicin	-	S1	43-44	1997	-
823	校伤寒病流行病学调查分	tigation and analysis of typ	邓泽红.	Deng Zehong.	中国现代医学杂志	ese Journal of Modern Med	-	9	68	1997	-
824	一起伤寒暴发调查	ion into a Typhoid Fever	金岿立	Jin Kuili	浙江预防医学	ejiang Preventive Medici	-	6	62-63	1997	sn1007-0931.1997.06.045
825	门氏菌污染井水引起的伤	aused by salmonella con	项慧	Xiang Hui	浙江预防医学	ejiang Preventive Medici	-	6	63	1997	sn1007-0931.1997.06.046
826	一起伤寒暴发流行临床报	port of an outbreak of typ	徐善,叶玉兰	Xu Shan,Ye Yulan	河南医药信息	Henan Medical Informatio	-	12	1	1997	-
827	一起乙型副伤寒暴发流行	an outbreak of paratyph	王尚斌,殷祖胜	ang Shangbin,Yin Zushe	安徽预防医学杂志	Journal of Preventive Me	-	4	1	1997	-
828	菌者引起伤寒暴发流行的	an outbreak of typhoid fe	陈海涛	Chen Haitao	中国国境卫生检疫杂志	ntier Health and Quaranti	-	5	267-268	1997	3/j.1004-9770.1997.05.008
829	一起山区伤寒暴发的调查	utbreak of typhoid fever	黄益民	Huang Yimin	疾病监测	disease surveillance	-	9	2	1997	-
830	起伤寒暴发的疫区处理报	f an outbreak of typhoid	余录根	Yu Lugen	疾病监测	disease surveillance	-	7	2	1997	-
831	起伤寒暴发流行的调查分	d analysis of an outbreak	詹根龙	Zhan Genlong	疾病控制杂志	Journal of disease contro	-	2	145	1997	-
832	一起食物型伤寒暴发的调	an outbreak of food-bo	明,尹桂成,严加和,蔡金桃,	Guicheng,Yan Jiahe,Ca	江苏预防医学	angsu Preventive Medici	-	2	1	1997	-
833	一起水型伤寒暴发调查	an outbreak of waterbo	江建华,陈公超	ng Jianhua,Chen Gongc	江苏预防医学	angsu Preventive Medici	-	2	3	1997	-
834	城两个单位伤寒暴发流行	eak of typhoid fever in tv	曾纯青,谢元甫	Zeng Chunqing,Xie Yuan	实用预防医学	actical preventive medici	-	2	102	1997	-
835	专科学校水型伤寒暴发的	water-type typhoid fever	铁生,付盛元,刘建湘,周永	u Shengyuan,Liu Jianxia	实用预防医学	actical preventive medici	-	1	14	1997	-
836	泽库县伤寒暴发流行分析	s of typhoid fever outbrea	王玉萍,白永华	Wang Yuping,Bai Yonghu	疾病监测	disease surveillance	-	2	2	1997	-
837	341例伤寒暴发的调查分析	and analysis of typhoid	柳,李翠云,林玫,董柏青,唐	iyun,Lin Mei,Dong Baiqi	广西预防医学	uangxi Preventive Medici	-	1	23-24	1997	-
838	228株伤寒沙门氏菌噬菌	228Study on phage typing	周宝珍,茹维萍,王建阳,王	hen,Ru Weiping,Wang Jiany	疾病监测	disease surveillance	13	5	169-173	1998	-
839	标本中检出鼠伤寒沙门菌	urium Copenhagen variant	李柏桂,王章云.	Li Baigui,Wang Zhangyun.	中华医学检验杂志	urnal of Medical Laborator	-	4	0	1998	sn:1009-9158.1998.04.036
840	丁沙门菌致化脓性脑膜炎	t meningitis caused by Sal	王苏建,吴健娟.	Wang Sujian,Wu Jianjuan.	中华医学检验杂志	urnal of Medical Laborator	-	3	0	1998	sn:1009-9158.1998.03.024
841	毒患者粪便中检出韦太夫	tterfrieden in the feces of	李长庆,胡艳冰,李以贵.	Changqing,Hu Yanbing,Li Yi	中华医学检验杂志	urnal of Medical Laborator	21	2	95	1998	sn:1009-9158.1998.02.031
842	血培养分离出11株雏沙门	m blood culture11Salmon	贾晻,叶宏.	Jia An,Ye Hong.	中华医学检验杂志	urnal of Medical Laborator	21	2	111	1998	sn:1009-9158.1998.02.027
843	株伤寒沙门氏菌耐药性分	rug resistance of Salmone	邵本令,张淑荣.	hao Benling,Zhang Shuron	医师进修杂志	ian Continuing Education J	-	3	45	1998	-
844	门氏菌质粒与耐药性的关	ween Salmonella Enteritidi	英伟,孙萌,林静,赵鑫,李	ei,Sun Meng,Lin Jing,Zhao	中国卫生检验杂志	ese Journal of Health Inspe	-	3	11-13	1998	-
845	痰和血标本中检出都柏林	tected in sputum and bloo	李仕英,张宏,谢小民,候天	hang Hong,Xie Xiaomin,Ho	北京军区医药	ijing Military Region Medic	-	4	307	1998	-
846	的种属分布对10种抗生素	of Salmonella strains10Ant	蒋火刚,张莉.	Jiang Huogang,Zhang Li.	遵义医学院学报	urnal of Zunyi Medical Colle	21	4	20-21	1998	-
847	伤寒沙门氏菌引起的食物	poisoning caused by Salm	李振元,英若娴.	Li Zhenyuan,Ying Ruoxian.	广东卫生防疫	ong Health and Epidemic Pr	-	1	82	1998	-
848	市越秀区食品从业人员沙门	carriage among food work	丁珊,何翠霞.	Ding Shan,He Cuixia.	广东卫生防疫	ong Health and Epidemic Pr	-	3	23-24	1998	-
849	菌致小儿败血症并化脓性	ent meningitis in children	吴庆周,谷赛华,袁伯宏.	ingzhou,Gu Saihua,Yuan Bo	现代中西医结合杂志	grated Traditional Chinese	-	4	489-490	1998	-

850	43人沙门氏菌检出及分布	he detection and distributi	汪萍,冯玉华.	Wang Ping,Feng Yuhua.	安徽预防医学杂志	i Journal of Preventive Med	-	4	-	1998	-
851	鼠伤寒沙门氏菌肠炎44例	a Typhimurium Enteritis44	尹国燕,马素芬,郭瑞彬.	Guoyan,Ma Sufen,Guo Rui	长治医学院学报	nal of Changzhi Medical Co	-	1	42-44	1998	-
852	994~1996年志贺菌,沙门	-1996shigella,Salmonella ty	姜秀云,王丽娟,李芳.	g Xiuyun,Wang Lijuan,Li Fa	苏州医学院学报	rnal of Suzhou Medical Coll	-	10	0	1998	-
853	寒沙门氏菌医院内感染的	nosocomial infection caus	赵晓娟.	Zhao Xiaojuan.	山西预防医学	Shanxi Preventive Medicine	7	4	308-309	1998	-
854	寒沙门菌的生物学性状和	and drug susceptibility testi	陈土良,张翊.	Chen Tuliang,Zhang Yi.	上海医学检验杂志	ournal of Medical Laborator	-	2	96-97	1998	-
855	汤卜逊沙门氏菌病流行病	al investigation of Salmon	郭秀翠,付鹤迎.	Guo Xiucui,Fu Heying.	山东预防医学	handong Preventive Medici	18	1	23-23,22	1998	-
856	培养伤寒沙门氏菌的鉴定	nalysis of Salmonella Typhi	田淑美,孙秀华.	Tian Shumei,Sun Xiuhua.	青岛医药卫生	ingdao Medicine and Healt	-	1	41-42	1998	-
857	安徽省沙门氏菌血清型分	f Salmonella serotypes in A	张慧玲,韦秀英.	Zhang Huiling,Wei Xiuying.	安徽预防医学杂志	i Journal of Preventive Med	-	2	54-56	1998	-
858	门氏菌引起食物中毒的细	tion of food poisoning caus	王章云,李柏桂,褚宝林.	g Zhangyun,Li Baigui,Chu B	肉品卫生	Meat hygiene	-	7	6-7	1998	-
859	菌属的耐药性变异和抗生	nella spp. in children and r	学,吴建良,顾金华,ZHEJIA	Wu Jianliang,Gu Jinhua,ZH	天津医药	Tianjin Medicine	-	10	609-611	1998	-
860	株伤寒沙门氏菌药敏结果	susceptibility results of Sal	金法祥.	Jin Faxiang.	河北医学	Hebei Medicine	-	2	71-72	1998	-
861	夫登堡沙门氏菌所致食物	oisoning caused by Salmon	宋玉兰.	Song Yulan.	上海预防医学杂志	hai Journal of Preventive M	-	7	312-313	1998	-
862	霍乱沙门氏菌食物中毒的	case of food poisoning cau	孙敬中,尹志晶.	Sun Jingzhong,Yin Zhijing.	山东预防医学	handong Preventive Medici	18	3	176	1998	-
863	鼠伤寒沙门氏菌引起的食	isoning caused by Salmon	延松,褚保林,胡志林,李中	g,Chu Baolin,Hu Zhilin,Li Z	现代预防医学	modern preventive medicin	-	1	84-85	1998	-
864	沙门氏菌分离株的血清型	ility of Salmonella isolates f	周接水.	Zhou Jieshui.	中国禽业导刊	China Poultry Industry Guid	-	4	34-35	1998	-
865	伤寒沙门氏菌食物中毒的	e of food poisoning caused	阮晓玲.	Ruan Xiaoling.	安徽预防医学杂志	i Journal of Preventive Med	-	2	73	1998	-
866	口冻鸡副产品沙门氏菌污染	a contamination of import	王志强,黄美婵.	ang Zhiqiang,Huang Meicha	肉品卫生	Meat hygiene	-	3	7-8	1998	-
867	鼠伤寒沙门氏菌感染及其	nella Typhimurium Infectio	王青玲.	Wang Qingling.	青海医药杂志	Qinghai Medical Journal	-	3	40-41	1998	-
868	圣保罗沙门氏菌引起的食	poisoning caused by Salm	段安彬,周凤琴.	Duan Anbin,Zhou Fengqin.	湖北预防医学杂志	i Journal of Preventive Med	-	3	45	1998	-
869	肠炎沙门氏菌所致食物中	poisoning caused by Salm	张继荣,程永森,梁华栋,多	g,Cheng Yongsen,Liang Hu	安徽预防医学杂志	i Journal of Preventive Med	-	1	46	1998	-
870	96年血培养沙门菌检出情	lture detection status of Sa	吴麖.	Wu Qi.	同济医科大学学报	nal of Tongji Medical Unive	-	5	406	1998	-
871	96株沙门氏菌菌型分布情	Distribution of Salmonella s	汪萍,冯玉华.	Wang Ping,Feng Yuhua.	现代预防医学	modern preventive medicin	-	4	509-510	1998	-
872	治疗小儿重症鼠伤寒沙门菌	almonella typhimurium infe	杨君,刘志峰,卫东.	ang Jun,Liu Zhifeng,weidon	医学理论与实践	Medical theory and practice	-	10	470-471	1998	-
873	分离出39株沙门氏菌的菌	body39Type identification	甘小琳.	Gan Xiaolin.	江西医学检验	Jiangxi Medical Laboratory	-	3	140-141	1998	-
874	门氏菌引起食物中毒的细	tion of food poisoning caus	王章云,李柏桂.	Wang Zhangyun,Li Baigui.	医学检验进修杂志	dvanced Medical Laborator	5	1	30-30,39	1998	-
875	市656株伤寒沙门氏菌对	ties656Salmonella typhi str	刘爱芬,宁凤莲.	Liu Aifen,Ning Fenglian.	中国卫生检验杂志	ese Journal of Health Inspe	8	6	381-382	1998	-
876	起沙门氏菌引起的食物中	on food poisoning caused	赵淑兰,林化同.	Zhao Shulan,Lin Huatong.	现代中西医结合杂志	grated Traditional Chinese	-	11	0	1998	-
877	学分型方法在流行病学调	lmonella Typhimurium in e	周刚,茹维萍,廖予妹.	g Zhou,Ru Weiping,Liao Yu	中华流行病学杂志	inese Journal of Epidemiolo	-	4	221	1998	sn:0254-6450.1998.04.008
878	区鼠伤寒沙门菌的质粒谱	monella typhimurium in so	潘若男,魏旭兰,罗明.	Ruonan,Wei Xulan,Luo M	中华预防医学杂志	se Journal of Preventive Me	32	4	-	1998	sn:0253-9624.1998.04.007
879	韦太夫雷登沙门菌引起的	poisoning caused by Salmon	李长庆,胡艳冰,李以贵.	Changqing,Hu Yanbing,Li Yi	中华流行病学杂志	inese Journal of Epidemiolo	-	4	247	1998	sn:0254-6450.1998.04.020
880	汁中分离出纽兰芝沙门菌	monella Newrange isolated	赵建平,周秀岚,李占海.	Jianping,Zhou Xiulan,Li Zha	中华传染病杂志	se Journal of Infectious Dis	16	1	-	1998	sn:1000-6680.1998.01.033
881	鼠伤寒沙门氏菌感染27例	a Typhimurium Infection27	王晓,吴步芳.	Wang Xiao,Wu Bufang.	新生儿科杂志	Journal of Neonatology	13	2	80	1998	-
882	用汤团引起食物中毒的病	od poisoning caused by ea	卓晓琴,金仁块,陈笑南.	oqin,Jin Ren Nugget,Chen	中国卫生检验杂志	ese Journal of Health Inspe	-	6	49-51	1998	-
883	餐饮业的肉品质量调查与	ation and monitoring in a c	赵春林.	Zhao Chunlin.	中国兽医科技	Veterinary Science and Tech	-	7	44-45	1998	-
884	伤寒并鼻衄2例报告	fever and epistaxis2Examp	马桂英.	Ma Guiying.	江西医药	Jiangxi Medicine	-	4	63-64	1998	-
885	病菌对19种抗菌药物的敏	ic bacteria19Antimicrobial	眷衡,尹烨华,陈天杰,王秉	g,Yin Yehua,Chen Tianjie,W	广东畜牧兽医科技	mal Husbandry and Veterin	-	3	14-15	1998	-
886	性疾病的流行菌株及流行	ains and trends of foodbor	李景云.	Li Jingyun.	传染病信息	fectious disease informatio	-	3	135-136	1998	-

887	寒并发心肌炎的观察与护	g care of typhoid fever com	陈文英.	Chen Wenying.	黑龙江护理杂志	eilongjiang Nursing Magazi	-	7	79-80	1998	-
888	市一起食物中毒病原学	gy of a case of food poison	唐建红,陈志好.	Tang Jianhong,Chen Zhihao	广东卫生防疫	ng Health and Epidemic Pr	-	1	70-71	1998	-
889	急性腹泻细菌病原学检	erial etiology detection of	陈多炎,周素芳,刘素云.	Duoyan,Zhou Sufang,Liu S	工企医刊	ial and Enterprise Medical	-	3	15-16	1998	-
890	1990~1995年食物中毒情	0~1995Annual food poison	夏克红,张淑娟.	Xia Kehong,Zhang Shujuan.	青海医药杂志	Qinghai Medical Journal	-	3	52-53	1998	-
891	感染病原体的耐药谱及感	spectrum and infection site	任玲,叶和清,李若兰.	en Ling,Ye Heqing,Li Ruolar	徐州医学院学报	rnal of Xuzhou Medical Coll	-	6	82-83	1998	-
892	牛副伤寒流行病学调查	observation on paratypho	詹发茂.	Zhan Famao.	中国兽医科技	eterinary Science and Tech	-	5	33-34	1998	-
893	用烧鸭引起中毒的调查分	alysis of poisoning caused	于芳.	Yu Fang.	广东卫生防疫	ng Health and Epidemic Pr	-	2	68-69	1998	-
894	乳牛乳腺炎病原调查	the pathogenic factors of d	体康,文耀南,邓光明,施君	en Yaonan,Deng Guangmin	中国兽医科技	eterinary Science and Tech	-	9	31-33	1998	-
895	东地区腹泻病原菌监测报	n diarrhea pathogenic bact	冰,翟文济,孟庆增,胡建华	hai Wenji,Meng Qingzeng,	中国卫生检验杂志	ese Journal of Health Inspe	-	4	42-44	1998	-
896	食物中毒看现今流通领域	es in the circulation field fr	李魁伟.	Li Kuiwei.	肉品卫生	Meat hygiene	-	12	6-7,16	1998	-
897	凉拌粉引起食物中毒的检	od poisoning caused by ea	唐燕飞.	Tang Yanfei.	桂林医学杂志	Guilin Medical Journal	14	3	181	1998	-
898	境中的人畜共患致病性细	pathogenic bacteria in the	孙桐,李忠.	Sun Tong,Li Zhong.	环境与健康杂志	rnal of Environment and He	-	5	45-47	1998	-
899	处理粪便用于农业和渔业	manure used in agriculture	宏建,钟志雄,林锦炎,丘志	Zhong Zhixiong,Lin Jinyan,	广东卫生防疫	ng Health and Epidemic Pr	-	4	60-62	1998	-
900	菌者引起的伤寒暴发流行	n typhoid outbreaks caus	王立富,严先民	Wang Lifu,Yan Xianmin	疾病监测	disease surveillance	-	12	1	1998	-
901	婚宴引起伤寒暴发流行调	n typhoid fever outbreak	王孝儒	Wang Xiaoru	遵义医学院学报	rnal of Zunyi Medical Col	-	S1	109-110	1998	-
902	校内甲型副伤寒暴发流行	n outbreak of paratyphoi	马小垒,唐昌新,唐华安	blei,Tang Changxin,Tang	广西预防医学	uangxi Preventive Medici	-	6	1	1998	-
903	流行性斑疹伤寒暴发流行	outbreak in Burundi linke	汪谋岳	Wang Mouyue	中华医学信息导报	se Medical Information H	-	22	1	1998	-
904	暴发流行菌株对药物的敏	ty of epidemic strains of	,曹俊峰,王玉凤,史晓琳,	Junfeng,Wang Yufeng,S	河南预防医学杂志	Journal of Preventive M	-	5	1	1998	-
905	伤寒暴发的流行病学调查	estigation and analysis o	桂玲,李航文,张栓虎,吕明	Li Hangwen,Zhang Shua	河南预防医学杂志	Journal of Preventive M	-	5	1	1998	-
906	铁路地区伤寒暴发流行的	tbreak of typhoid fever i	杨军刚	Yang Jungang	现代预防医学	odern preventive medici	-	3	268	1998	-
907	一起水型伤寒暴发调查	an outbreak of waterbo	,张瑞民,崔顺领,郭登陆,	Ruimin,Cui Soonling,Gu	河南预防医学杂志	Journal of Preventive M	-	4	1	1998	-
908	县九八年伤寒暴发流行调	typhoid fever outbreak in	赵清风,王立春	hao Qingfeng,Wang Lich	黑河科技	Heihe Technology	-	3	29-30	1998	-
909	起水型伤寒暴发流行的调	an outbreak of waterbo	张子平	Zhang Ziping	疾病监测	disease surveillance	-	6	3	1998	-
910	村一起经婚宴引起的伤寒	fever caused by a weddi	祥,陆安祥,林永宏,许云峰,	Lu Anxiang,Lin Yonghor	疾病监测	disease surveillance	-	6	1	1998	-
911	料引发伤寒暴发流行的调	rt on a typhoid outbreak	金良华,徐子伟,李卓	Lianghua,Xu Ziwei,Li Zh	江苏预防医学	angsu Preventive Medici	-	2	2	1998	-
912	例伤寒暴发流行的调查分	and analysis of typhoid	陈建华	Chen Jianhua	厂矿医药卫生	y and mine medicine and	-	2	132-133	1998	-
913	伤寒暴发流行特征分析及	haracteristics of typhoid	刘波,陈从新,郭顺明,吴建	Congxin,Guo Shunming	人民军医	people's military medicine	-	5	286	1998	-
914	县库区伤寒暴发、流行	alence of typhoid fever i	盛其伟,刘子杰,李景先	ng Qiwei,Liu Zijie,Li Jing	预防医学文献信息	ve medicine literature inf	-	2	138-139	1998	sn.1672-9153.1998.02.035
915	潼中学伤寒暴发流行的调	break of typhoid fever in	王宏军,王龙海	ang Hongjun,Wang Long	实用预防医学	actical preventive medici	-	2	1	1998	-
916	被病原学证实的伤寒暴发	of typhoid fever confirme	隋隽殊,侯建林,胡立新	Junshu,Hou Jianlin,Hu L	中国公共卫生管理	ese public health manage	-	2	135-136	1998	cnki.23-1318.1998.02.033
917	部一起水型伤寒暴发的调	k of water-type typhoid f	张军,欧阳卫华	hang Jun,Ouyang Weihu	解放军预防医学杂志	ve Medicine of the Peopl	-	1	1	1998	-
918	饮、服行业人员沙门菌	riage among personnel in f	陈义忠,梁暄,陈清,林智.	hong,Liang Xuan,Chen Qin	疾病控制杂志	journal of disease control	3	2	136	1999	sn.1674-3679.1999.02.033
919	型伤寒沙门氏菌分子流行	lecular epidemiological st	,俞晓进,刘光中,杨华富,	Xiaojin,Liu Guangzhong,Ya	疾病监测	disease surveillance	14	9	325-327	1999	-
920	门氏菌引起食物中毒的细	n of food poisoning cause	,滕焕昭,李柏桂,林青成,	g Huanzhao,Li Baigui,Lin Q	中国人兽共患病杂志	Chinese Journal of Zoonose	9	3	115	1999	sn.1002-2694.1999.03.040
921	罗沙门茵菌引起一起食物	poisoning caused by Salm	李金学,郭晓玲.	Li Jinxue,Guo Xiaoling.	河南预防医学杂志	n Journal of Preventive Me	10	5	308	1999	-
922	从冻干鲜贝中检出沙门氏	almonella from freeze-dri	王少玲,温瑞荣,符振华.	haoling,Wen Ruirong,Fu Zh	中国卫生检验杂志	ese Journal of Health Inspe	-	6	476-477	1999	-
923	都柏林沙门氏菌阿克拉变	monella dublin Accra varian	王章云,李柏桂,朱晓兰.	Zhangyun,Li Baigui,Zhu Xi	现代预防医学	modern preventive medicin	-	1	104	1999	-

924	市区鼠伤寒沙门菌生物学	lmonella typhimurium in s	茹维萍,潘若男,张新增,王	eiping,Pan Ruonan,Zhang X	中国卫生检验杂志	ese Journal of Health Inspe	-	2	11-13	1999	-
925	株沙门氏菌菌型鉴定结果	of Salmonella strain identif	悦,郭玲,李素华,杨玉芝,李	uo Ling,Li Suhua,Yang Yuzh	安徽预防医学杂志	i Journal of Preventive Me	-	1	48-49	1999	-
926	鼠伤寒沙门氏菌败血症16	la Typhimurium Septicemi	周媛.	Zhou Yuan.	中国厂矿医学	a factory and mining medi	-	5	50-51	1999	-
927	业人员1121株沙门氏菌菌	kers1121Salmonella strain	蔡卫红.	Cai Weihong.	广东卫生防疫	ng Health and Epidemic Pr	-	3	36	1999	-
928	-1995年伤寒沙门氏噬菌体	nnual type distribution and	张婵,张爱玲.	Zhang Chan,Zhang Ailing.	中国公共卫生	Chinese public health	-	2	45	1999	-
929	沙门菌引起家庭暴发食物	reaks of food poisoning ca	李长庆,胡艳冰.	Li Changqing,Hu Yanbing.	华南国防医学杂志	Journal of National Defen	-	2	64	1999	-
930	寒沙门氏菌食物中毒的	case of food poisoning caus	刘杰,于永萍,郭宪彩.	Jie,Yu Yongping,Guo Xian	中国卫生检验杂志	ese Journal of Health Inspe	-	3	67	1999	-
931	公共场所从业人员沙门氏	mong Workers in Food and	谢平.	Xie Ping.	安徽预防医学杂志	i Journal of Preventive Me	-	2	34-35	1999	-
932	猪带染沙门氏菌调查分析	lysis of Salmonella infectio	君,王文周,张正尧,丁力,张	Venzhou,Zhang Zhengyao,D	中国卫生检验杂志	ese Journal of Health Inspe	-	4	64-65	1999	-
933	制品同时检出两株沙门氏	onella detected in cooked r	蔡海珊,林朝,叶众.	i Haishan,Lin Chao,Ye Zhor	中国卫生监督杂志	se Journal of Health Super	-	5	220-221	1999	-
934	门氏菌污染的牛板筋引起	ed by eating beef tendons	松,董晓平,李玮,赵宝玉,張	ng Xiaoping,Li Wei,Zhao Ba	吉林医学院学报	urnal of Jilin Medical Colle	-	4	45	1999	-
935	液中检出一株纽兰芝沙门	onella Newrange was dete	张玲,黄青青,王川安.	ng,Huang Qingqing,Wang C	淮海医药	Huaihai Pharmaceutical	-	1	10	1999	-
936	肠道沙门氏菌属与志贺氏	rriage of Salmonella and Sh	玉,叶林,杨梅,朱成武,王	in,bayberry,Zhu Chengwu,	现代预防医学	modern preventive medicin	-	3	317-319	1999	-
937	伦登卢普沙门氏菌食物中	ase of Salmonella Brendon	柱,周亚平,杨春秀,郑玲,刘	Yaping,Yang Chunxiu,Zhe	预防医学文献信息	ive medicine literature info	-	1	14-15,17	1999	-
938	门菌330株药物敏感试验结	0Analysis of strain drug se	赵宏,马志俊.	Zhao Hong,Ma Zhijun.	上海医学检验杂志	urnal of Medical Laborato	-	2	12	1999	-
939	寒沙门氏菌肠炎420例临床	imurium Enteritis420Case	启坤,刘秋莹,邵雪英,欧内	n,Liu Qiuying,Shao Xueyin	黔南民族医专学报	f Qiannan National Medica	-	1	39-40	1999	-
940	保罗沙门氏菌引起的食物	e of food poisoning caused	王襄,郑向梅,李泽霞.	Xiang,Zheng Xiangmei,Li Z	预防医学文献信息	ive medicine literature info	-	1	39	1999	-
941	市沙门氏菌菌型分布情况	tribution of Salmonella str	李星,史玲.	Li Xing,Shi Ling.	泰山卫生	Taishan Health	23	3	-	1999	-
942	病牛沙门氏菌引起食物中	oisoning caused by Salmo	郭林春,曹克新.	Guo Linchun,Cao Kexin.	内蒙古预防医学	Mongolia Preventive Med	-	1	14	1999	-
943	引起败血症的沙门氏菌菌	s of Salmonella strains cau	李红玉,梁卓智,林君仪.	ngyu,Leung Cheuk Chi,Lin J	实用预防医学	ractical preventive medicin	-	5	392	1999	-
944	都柏林沙门氏菌阿克拉变	monella dublin Accra varia	王章云,李柏桂.	Wang Zhangyun,Li Baigui.	中国卫生检验杂志	ese Journal of Health Inspe	-	2	49-50	1999	-
945	为沙门氏菌引起食物中毒	food poisoning caused by	王捷.	Wang Jie.	肉品卫生	Meat hygiene	-	4	19-19,11	1999	-
946	区饮食从业人员沙门氏菌	ers among catering worke	霞,颜湘,陈小丽,吴金宏,曹	n Xiang,Chen Xiaoli,Wu Jin	预防医学文献信息	ive medicine literature info	-	1	54	1999	-
947	鸡肉中沙门氏菌的分离和	tion of Salmonella from fr	金,朱士盛,王新,张肖正,刘	sheng,Wang Xin,Zhang Xia	中国动物检疫	China Animal Quarantine	-	6	10-11	1999	-
948	柏林沙门菌引起脑膜炎一	eningitis caused by Salmo	卢翠珍,张云生,李连敬.	zhen,Zhang Yunsheng,Li Li	中华医学检验杂志	urnal of Medical Laborato	-	1	32	1999	sn:1009-9158.1999.01.024
949	中分离出1株山夫顿堡沙	ted from blood1Salmonella	韩善桥.	Han Shanqiao.	临床检验杂志	journal of clinical testing	-	3	171	1999	n.1001-764X.1999.03.031
950	液中检出1株圣保罗沙门	ed in sputum1Salmonella s	朱燕英.	Zhu Yanying.	临床检验杂志	journal of clinical testing	-	5	265	1999	n.1001-764X.1999.05.026
951	0株沙门氏菌药敏试验分	rug susceptibility test of Sa	马志俊,蒯迎平.	Ma Zhijun,Kuai Yingping.	右江民族医学院学报	ujiang Medical College for	-	1	65	1999	sn.1001-5817.1999.01.052
952	痢沙门氏菌耐药性的变化	ug resistance of Salmonell	学贤,倪振亚,刘文博,高	n,Ni Zhenya,Liu Wenbo,Ga	中国预防兽医学报	rnal of Preventive Veterina	21	4	305-307	1999	-
953	儿院内鼠伤寒沙门氏菌感	f nosocomial Salmonella ty	李汉萍.	Li Hanping.	中国优生与遗传杂志	Journal of Eugenics and G	-	4	114116	1999	-
954	门氏菌Ⅲ型引起食物中毒	case of food poisoning cau	魏洪启,吕保新,石素珍.	i Hongqi,Lu Baoxin,Shi Suz	河北职工医学院学报	of Hebei Workers' Medical	-	4	32	1999	-
955	山夫顿堡少门氏菌食物中	d poisoning caused by Sha	张谷亮,刘继共.	Zhang Guliang,Liu Jigong.	肉品卫生	Meat hygiene	-	6	19	1999	-
956	84~1996年微生物性食物	1996Annual analysis of mi	胡小红,胡淑梅,朱水芝.	aohong,Hu Shumei,Zhu Sh	实用预防医学	ractical preventive medicin	-	1	55-56	1999	-
957	市饮食服务人员肠道带菌	ria among catering service	陈玉华,马龙宝,顾斌.	en Yuhua,Malong Bao,Gu B	中国厂矿医学	a factory and mining medi	-	1	77-78	1999	-
958	香鸡加工过程中微生物污	ination during processing	晓勤,吴建聪,周冰莹,谢新	Wu Jiancong,Zhou Bingying,	现代商检科技	commodity inspection tec	-	1	44-45,47	1999	-
959	96~1997年伤寒、副伤寒	Annual detection of typhoi	朱水荣,张政,莫顺堂.	irong,Zhang Zheng,Mo Shu	浙江预防医学	hejiang Preventive Medicin	-	2	1-2	1999	-
960	微生物污染情况调查及卫	evaluation of imported foo	王志强,黄美婵.	ang Zhiqiang,Huang Meich	肉品卫生	Meat hygiene	-	1	11-12	1999	-

961	肺癌118例误诊分析	er118Analysis of misdiagn	王中平.	Wang Zhongping.	镇江医学院学报	al of Zhenjiang Medical Co	-	2	222	1999	-
962	共场所从业人员肠道致病	intestinal pathogenic bacte	唐晓磊,席培华.	Tang Xiaolei,Xi Peihua.	青海医药杂志	Qinghai Medical Journal	-	1	40-41	1999	-
963	液细菌L型培养及药敏试	Type culture and drug susc	潘虹,江冬梅.	Pan Hong,Jiang Dongmei.	青海医药杂志	Qinghai Medical Journal	-	9	44-45	1999	-
964	鲁西南及临沂地区常见鸡	eases in Xuzhou, southwes	喻涛,王斌.	Yu Tao,Wang Bin.	畜牧与兽医	Husbandry and Veterinary M	-	S1	40	1999	-
965	市生活垃圾的生物性污染	gical pollution of domestic	连建华,伍碧雯,陈思东.	Jianhua,Wu Biwen,Chen Si	中国卫生检验杂志	ese Journal of Health Inspe	-	5	371-372	1999	-
966	某大酒楼食客细菌性食物	poisoning among diners in	刘巨多.	Liu Juduo.	广东卫生防疫	ng Health and Epidemic Pr	-	2	64	1999	-
967	婚宴引起的食物中毒致病	ctors of food poisoning cau	李春声.	Li Chunsheng.	广东卫生防疫	ng Health and Epidemic Pr	-	1	52-53	1999	-
968	市医院污水消毒监测现状	Wastewater Disinfection M	张凡非,贾瑛,冯斌,郭小燕	ei,Jia Ying,Feng Bin,Guo Xia	中国卫生检验杂志	ese Journal of Health Inspe	-	1	45-46	1999	-
969	市感染性腹泻病原学变迁	in etiology of infectious di	郭登陆.	Guo Landing.	中国卫生检验杂志	ese Journal of Health Inspe	-	5	364-365	1999	-
970	腹泻的病原学分析及289名	nalysis of cases of traveler	李慧琳,马斑,颜小军.	Huilin,horse zebra,Yan Xiaoj	旅行医学科学	travel medical science	5	4	40	1999	sn.1006-7159.1999.04.018
971	市个体医疗单位细菌污染	ntamination in individual r	何丽慧,纪晓芳,林克平.	e Lihui,Ji Xiaofang,Lin Kepin	中国消毒学杂志	hinese Journal of Disinfecti	16	4	250-251	1999	sn.1001-7658.1999.04.035
972	出420株菌的菌谱分布及	trum distribution and drug	建美,张式鸿,廖康,陈菊香,	Zhang Shihong,Liao Kang,C	中国微生态学杂志	inese Journal of Microecolo	11	1	38-40	1999	n.1005-376X.1999.01.013
973	起伤寒暴发流行的调查分	d analysis of an outbreak	汪维	Wang Wei	广西预防医学	uangxi Preventive Medici	-	S1	22	1999	-
974	两起介水伤寒暴发疫情调	on into two hydrotyphoid	贺小良	He Xiaoliang	江苏预防医学	angsu Preventive Medici	-	4	35-36	1999	-
975	经水传播甲型副伤寒暴发	outbreak of water-borne	琥,孙波,朱汝涌,朱凤东,潘	Bo,Zhu Ruyong,Zhu Fen	江苏预防医学	angsu Preventive Medici	-	4	40	1999	-
976	、狗场、小新寨伤寒暴发	ks in Panjiazhuang Tow	夏德茂	Xia Demao	数理医药学杂志	nal of Mathematical Med	-	4	339-340	1999	-
977	来水引起伤寒暴发流行调	prevalence of typhoid fe	许秀勇,张嘉祥	u Xiuyong,Zhang Jiaxian	海峡预防医学杂志	Journal of Preventive M	-	4	29-13	1999	-
978	一起甲型副伤寒暴发的调	nto an outbreak of paraty	朱磷扬,徐曼如,营亮	Foyang,Xu Manru,Yingli	预防医学文献信息	ve medicine literature inf	-	4	330-331	1999	sn.1672-9153.1999.04.020
979	省三起甲型副伤寒暴发调	e paratyphoid fever outb	汪华,施平,庄菱	ng Hua,Shi Ping,Zhuang	中国公共卫生	Chinese public health	-	10	1	1999	-
980	起伤寒暴发的流行病学调	vestigation of an outbrea	王同来,梁永春,吉玉国	onglai,Liang Yongchun,J	江苏预防医学	angsu Preventive Medici	-	3	77-78	1999	-
981	于武鸣县双桥镇第一中学	Region on the outbreak	-	-	广西政报	Guangxi Political News	-	25	24-25	1999	-
982	教学校一起伤寒暴发流行	of typhoid fever in a juve	邹晓忠	Zou Xiaozhong	疾病监测	disease surveillance	-	6	2	1999	-
983	一起伤寒暴发流行调查	investigation of a typhoid	彭维斌,纪有根	Peng Weibin,Ji Yougen	江苏预防医学	angsu Preventive Medici	-	2	2	1999	-
984	学校幼儿园内伤寒暴发流	ce of typhoid fever outbr	王同来,梁永春,吉玉国	onglai,Liang Yongchun,J	江苏预防医学	angsu Preventive Medici	-	2	1	1999	-
985	1986～1995年伤寒暴发疫	6~1995Analysis of annua	刘于飞,任蕴慧,刘树国	Yufei,Ren Yunhui,Liu Shu	广东卫生防疫	g Health and Epidemic P	-	1	4	1999	-
986	起伤寒暴发疫情的调查报	report on an outbreak of	熊建华,杜其筠,欧智勇	Jianhua,Du Qijun,Ou Zh	现代预防医学	odern preventive medici	-	1	1	1999	-
987	13起外源性伤寒暴发的调	ation and analysis of exo	郑云	Zheng Yun	中国公共卫生学报	nese Journal of Public He	-	1	1	1999	-
988	人员肠道沙门氏菌带菌调	od workers in Tianhe Dist	梁,蔡卫红,黄志标,张惠文	Weihong,Huang Zhibiao,Z	现代预防医学	modern preventive medicin	27	3	335-336	2000	sn.1003-8507.2000.03.025
989	肠炎沙门菌可致脓肿二例	abscess caused by Salmone	徐瑞龙.	Xu Ruilong.	上海医学检验杂志	ournal of Medical Laborato	15	5	272-272	2000	sn.1673-8640.2000.05.027
990	、鱼、鸭、兔等带染沙门	infection in pigs, fish, duck	林玲,崔敏莉.	Lin Ling,Cui Minli.	现代预防医学	modern preventive medicin	27	2	244	2000	sn.1003-8507.2000.02.071
991	寒沙门氏菌噬菌体分型及	trum monitoring of Salmon	白羽,姚文清.	white feather,Yao Wenqing	中国公共卫生	Chinese public health	16	6	532-533	2000	sn:1001-0580.2000.06.030
992	门氏菌污染熟肉丸引起的	used by salmonella contam	宋整风,黄崇艾.	ng Zhengfeng,Huang Chong	解放军预防医学杂志	tive Medicine of the People	18	1	69	2000	sn.1001-5248.2000.01.030
993	162例沙门氏菌感染分析	2Salmonella infection analy	赵忠,宋立晶,沈亚玉.	o Zhong,Song Lijing,Shen Y	宁夏医学院学报	rnal of Ningxia Medical Coll	22	6	437-438	2000	sn.1674-6309.2000.06.028
994	市鼠类沙门氏菌感染状况	nella Infection Status of Ra	陆,宗自库,付辉,许银怀,何	ding,Zongziku,Fu Hui,Xu Yi	河南预防医学杂志	n Journal of Preventive Me	11	1	25-26	2000	sn.1006-8414.2000.01.010
995	寒沙门氏菌对13种抗生素敏	a typhi13Antibiotic suscept	王丽华.	Wang Lihua.	华夏医学	Chinese Medicine	13	1	62-63	2000	sn.1008-2409.2000.01.040
996	菌体型伤寒沙门氏菌的分	on common phage-type Sa	俞晓进,刘光中,汪华,杨华富	n,Liu Guangzhong,Wang H	中国公共卫生	Chinese public health	16	5	465-467	2000	sn:1001-0580.2000.05.045
997	型伤寒沙门氏菌的分子流	lar epidemiological charac	李琦,高瞻,俞晓进,刘光中	h vision,Yu Xiaojin,Liu Guar	中国公共卫生	Chinese public health	16	11	985-987	2000	sn:1001-0580.2000.11.009

998	地区鸡沙门氏菌病流行病	gation of chicken salmonel	庆华,亢文华,李平安,关平	ng Wenhua,Li Pingan,Guan	中国家禽	Chinese poultry	22	10	33-34	2000	sn.1004-6364.2000.10.021
999	l起的食物中毒及患者带	d by Salmonella Riding and	郭振坤,张晓冰,贺侠,王芳	un,Zhang Xiaobing,He Xia,\	中国卫生检验杂志	ese Journal of Health Inspe	10	6	747-748	2000	sn.1004-8685.2000.06.078
1000	可区1989年--1998年沙门	u1989Year--1998Salmonell	李爱莲,黄燕,张惠文.	ilian,Huang Yan,Zhang Huiv	中国卫生检验杂志	ese Journal of Health Inspe	10	2	194-195	2000	sn.1004-8685.2000.02.038
1001	伤寒沙门菌致下肢感染一	b infection caused by Salm	陈丽,周文娟.	Chen Li,Zhou Wenjuan.	上海医学检验杂志	ournal of Medical Laborato	15	1	37	2000	sn.1673-8640.2000.01.048
1002	寒沙门氏菌骨关节炎1例	phimurium Osteoarthritis1	张合先,周亚鹏.	Zhang Hexian,Zhou Yapeng	实用放射学杂志	ournal of Practical Radiolog	16	7	445-445	2000	sn.1002-1671.2000.07.030
1003	1125株伤寒沙门菌噬菌体	1125Phage typing and rese	闯,张婵,刘杰伟,刘爱芬,宁	ng Chan,Liu Jiewei,Liu Aifer	中国卫生检验杂志	ese Journal of Health Inspe	10	4	410-411	2000	sn.1004-8685.2000.04.012
1004	肠炎沙门氏菌引起的食	poisoning caused by Salmo	学,卜宪岭,徐保红,王海燕,	u Xianling,Xu Baohong,Wa	中华流行病学杂志	inese Journal of Epidemiolo	21	1	63	2000	sn:0254-6450.2000.01.036
1005	液和血液中同时检出都柏	ously detected in cerebros	吕华,孙继梅.	Lu Hua,Sun Jimei.	中国医科大学学报	nal of China Medical Unive	29	6	478	2000	sn.0258-4646.2000.06.035
1006	泌物中分离出都伯林沙门	Dublin isolated from subm	红,陈双峰,哈桂珍,许文莲	n Shuangfeng,Ha Guizhen,	上海医学检验杂志	ournal of Medical Laborato	15	2	111	2000	sn.1673-8640.2000.02.045
1007	毒尸检标本中检出鼠伤寒	phimurium from Autopsy S	玉环,赵丽君,张明华,曾祥	hao Lijun,Zhang Minghua,Z	中国卫生检验杂志	ese Journal of Health Inspe	10	1	78-78	2000	sn.1004-8685.2000.01.046
1008	伤寒沙门氏菌感染32例临	ium infection in children32	邹晓红,项曙光,韩红芬.	hong,Xiang Shuguang,Han	临床儿科杂志	Journal of Clinical Pediatrics	18	3	191	2000	sn.1000-3606.2000.03.047
1009	夫雷登沙门氏菌引起的食	of food poisoning caused by	周江,高兵.	Zhou Jiang,Gao Bing.	川省卫生管理干部学院学	huan Health Management	-	2	141	2000	-
1010	门氏菌血清分群及药敏试	serogrouping and drug susc	刘敏鸿,张红梅.	iu Minhong,Zhang Hongme	安徽预防医学杂志	i Journal of Preventive Med	-	3	195	2000	-
1011	寒沙门氏菌污染牛肉引起	used by beef contaminated	肖绍昌.	Xiao Shaochang.	职业与健康	Occupation and health	-	9	25-26	2000	-
1012	门氏菌引起的一次雏鸭副	tion in Ducklings Caused by	曹蔚,文明.	Cao Wei,civilization.	贵州畜牧兽医	mal Husbandry and Veterin	-	2	25-26	2000	-
1013	起沙门氏菌致食物中毒调	a case of food poisoning ca	王道成.	Wang Daocheng.	安徽预防医学杂志	i Journal of Preventive Med	-	6	423	2000	-
1014	鸡肠炎沙门氏菌感染监测	Salmonella Enteritidis infec	史同瑞.	Shi Tongrui.	畜牧兽医科技信息	d Veterinary Science and Te	-	9	11	2000	-
1015	脊液中检出鼠伤寒沙门氏	a typhimurium from cereb	贾伟,肖月琴.	Jia Wei,Xiao Yueqin.	宁夏医学院学报	rnal of Ningxia Medical Coll	22	6	406-406	2000	sn.1674-6309.2000.06.050
1016	肠炎沙门氏菌食物中毒调	n a case of Salmonella Ente	彭旺初.	Peng Wangchu.	广东卫生防疫	ng Health and Epidemic Pr	-	3	79-80	2000	-
1017	大便标本分离出圣保罗沙	from stool specimens of foo	杨兴惠,姚从美.	Yang Xinghui,Yao Congmei.	成都医药	Chengdu Medicine	-	3	191	2000	-
1018	门氏菌属的分型及药敏试	susceptibility test results o	岳凤.	Yue Feng.	嘉兴医学	Jiaxing Medicine	-	4	-	2000	-
1019	食物中毒34例中西医诊断	of diagnosis and treatment	潘虹霞,陈志明.	Pan Hongxia,Chen Zhiming	中国中医急症	traditional medicine emer	-	S1	23-24	2000	-
1020	脾囊肿穿刺液分离沙门菌	on results from renal cyst a	胡莉萍,许美荣,邓丽华.	Liping,Xu Meirong,Teresa T	徐州医学院学报	rnal of Xuzhou Medical Coll	-	1	70-72	2000	-
1021	株沙门氏菌型分布及药敏	in type distribution and dru	张慧,杨先安,韩世兰.	ng Hui,Yang Xianan,Han Sh	武汉市职工医学院学报	of Wuhan Workers' Medica	-	1	25-26	2000	-
1022	重沙门氏菌食物中毒的调	on a case of severe salmo	,杨雄健,尉向海,王化珍,李	ngjian,Wei Xianghai,Wang	南大学学报（医学科学版	nan University (Medical Sci	-	1	73-76	2000	-
1023	寒沙门氏菌所致的食物中	d poisoning caused by Salr	鞠勇.	Ju Yong.	预防医学情报杂志	of Preventive Medicine Info	-	2	162	2000	-
1024	沙门氏菌引起的食物中	d poisoning caused by Sal	兴宇,桂立新,孙玉方,桂林	ngyu,Gui Lixin,Sun Yufang,	安徽预防医学杂志	i Journal of Preventive Med	-	2	159	2000	-
1025	体检人员便中检出1株阿	cal examiners in the caterin	张艳亭,陆燕萍.	Zhang Yanting,Lu Yanping.	山西预防医学杂志	i Journal of Preventive Me	9	4	336-337	2000	-
1026	肠炎沙门氏菌致脓肿二例	abscess caused by Salmone	徐瑞龙.	Xu Ruilong.	浙江临床医学	Zhejiang Clinical Medicine	12	2	794	2000	sn.1008-7664.2000.02.059
1027	人员沙门氏菌菌型分布及	nd drug resistance of Salm	陈建辉,谢一俊.	Chen Jianhui,Xie Yijun.	海峡预防医学杂志	s Journal of Preventive Med	6	2	43-44	2000	-
1028	安徽省沙门氏菌血清型分	f Salmonella serotypes in A	张慧玲,韦秀英.	Zhang Huiling,Wei Xiuying.	上海预防医学杂志	ai Journal of Preventive M	-	6	275-276	2000	-
1029	沙门氏菌菌型分布和药敏	in distribution and drug sus	付竹霓.	Fu Zhuni.	邯郸医学高等专科学校学	nal of Handan Medical Col	-	4	297-298	2000	-
1030	伤寒沙门菌感染30例临床	himurium infection30Case	李金柱,刘磊,常建平.	Jinzhu,Liu Lei,Chang Jianpin	中国综合临床	hina Comprehensive Clinica	16	10	764	2000	sn.1008-6315.2000.10.033
1031	门氏菌引起暴发性食物中	outbreak of food poisonin	包建华,周斌,朱建华,郭芳,	anhua,Zhou Bin,Zhu Jianhu	湖北预防医学杂志	i Journal of Preventive Med	-	3	30-31	2000	-
1032	肠炎沙门氏菌致食物中	of food poisoning caused	徐赟,胡松花,章荣花.	un,Hu Songhua,Zhang Rong	安徽预防医学杂志	i Journal of Preventive Med	-	2	98-99	2000	-
1033	体检粪便中检出查理沙门	was detected in stool during	胡锦元,余铁甫,陈芬珍.	inyuan,Yu Tiefu,Chen Fenz	上海预防医学杂志	ai Journal of Preventive M	-	6	276-277	2000	-
1034	寒沙门氏菌的鉴定和抗生素	sceptibility determination	郭锐,卢玉娥.	Guo Rui,Lu Yu'e.	现代中西医结合杂志	grated Traditional Chinese	-	19	1909-1910	2000	-

1035	霍乱沙门氏菌引起的食物	of food poisoning caused b	冬琴,谢红辉,罗亮宇,宋立	,Xie Honghui,Luo Liangyu,S	实用预防医学	ractical preventive medicin	7	3	212	2000	sn.1006-3110.2000.03.035
1036	雷登沙门氏菌引起的食物	on food poisoning caused	杨海宁,张晓峰.	ang Haining,Zhang Xiaofen	安徽预防医学杂志	i Journal of Preventive Me	-	2	98	2000	-
1037	伤寒沙门氏菌食物中毒的	e of food poisoning caused	孟金香,刘志霞.	Meng Jinxiang,Liu Zhixia.	山东食品科技	Shandong Food Technology	-	3	19	2000	-
1038	胸壁脓肿检出伤寒沙门氏菌	etected in anterior chest wa	蒋伟,闫玉萍,王靖.	ng Wei,Yan Yuping,Wang Ji	预防医学文献信息	ive medicine literature info	6	2	179	2000	sn.1672-9153.2000.02.077
1039	鼠伤寒沙门氏菌引起的食物	isoning caused by Salmone	任玉团,赵瑛,杨嵩.	Yutuan,Zhao Ying,Yang So	包头医学院学报	rnal of Baotou Medical Coll	16	4	275-276	2000	n.1006-740X.2000.04.010
1040	沙门氏菌引起的食物中毒的	case of food poisoning ca	,曾凡伟,邓富玉,李玉珍,关	eng Fanwei,Deng Fuyu,Li Yu	预防医学文献信息	ive medicine literature info	6	4	374-375	2000	sn.1672-9153.2000.04.062
1041	液和大便中同时检出鲁克	etected simultaneously in t	蒲文华,孟梅.	Pu Wenhua,Meng Mei.	齐鲁医学检验	Qilu Medical Testing	11	2	49	2000	sn.1673-5013.2000.02.037
1042	菌肠炎引起的细菌性颈动脉	arotid aneurysm caused by	金龙俊,陈福真.	Jin Yongjun,Chen Fuzhen.	中华普通外科杂志	nese Journal of General Sur	15	4	212	2000	n:1007-631X.2000.04.027
1043	都柏林沙门菌败血症一例	e of salmonella sepsis in D	高明亮,席维岳,张吉平.	Mingliang,Xi Weiyue,Zhang	中华传染病杂志	se Journal of Infectious Dis	18	3	165	2000	-
1044	沙门菌血清型肠炎88例临	idis serovar Enteritidis88Ca	赵敏,曲芬,姜素椿.	ao Min,Qu Fen,Jiang Suchu	中华内科杂志	ese Journal of Internal Med	39	6	412	2000	sn:0578-1426.2000.06.021
1045	中伤寒沙门氏菌抗生素药	ceptibility results of Salmo	刘锦光,许岸高.	Liu Jinguang,Xu Angao.	实用医学杂志	ournal of Practical Medicin	16	2	162-163	2000	sn.1006-5725.2000.02.042
1046	沙门菌感染并发肠出血1例	n complicated by intestina	,庞桂芬,刘晓艳,张小平,马	ang Guifen,Liu Xiaoyan,Zha	临床荟萃	clinical collection	15	4	186	2000	n.1004-583X.2000.04.045
1047	肠炎沙门氏菌致脓肿二例	abscess caused by Salmone	徐瑞龙.	Xu Ruilong.	浙江临床医学	Zhejiang Clinical Medicine	2	12	794	2000	sn.1008-7664.2000.12.059
1048	甲型副伤寒沙门氏菌药敏	ibility test of Salmonella pa	傅松维.	Fu Songwei.	浙江预防医学	hejiang Preventive Medicin	12	10	64	2000	sn.1007-0931.2000.10.052
1049	伤寒沙门氏菌的分离鉴定	Drug Susceptibility Test of	陈益明.	Chen Yiming.	浙江预防医学	hejiang Preventive Medicin	12	7	封三	2000	sn.1007-0931.2000.07.052
1050	起沙门氏菌食物中毒的细	n of Salmonella Salmonella	金晓萍,江雪清.	Jin Xiaoping,Jiang Xueqing.	浙江预防医学	hejiang Preventive Medicin	12	3	27	2000	sn.1007-0931.2000.03.021
1051	西副伤寒沙门氏菌药物敏	lity analysis of Salmonella	冬梅,王鸣柳,李翠云,唐振	,Wang Mingliu,Li Cuiyun,T	广西预防医学	Guangxi Preventive Medicin	6	5	277-278	2000	n.1673-758X.2000.05.008
1052	霍乱沙门氏菌食物中毒调	case of choleraesuis and s	王垂佑,颜礼芬,马瑞华.	g Chuiyou,Yan Lifen,Ma Ru	河南预防医学杂志	n Journal of Preventive Me	-	3	188	2000	sn.1006-8414.2000.03.039
1053	由拉马特根沙门氏菌引起	nella Ramatogen was disc	谢红英,钟凌,蔡宏.	Hongying,Zhong Ling,Cai H	福建医药杂志	Fujian Medical Journal	22	Z1	189	2000	sn.1002-2600.2000.z1.054
1054	年新检出的沙门氏菌型别	y detected Salmonella type	谢一俊,陈亢川,陈建辉.	un,Chen Kangchuan,Chen J	海峡预防医学杂志	s Journal of Preventive Me	6	2	42	2000	sn.1007-2705.2000.02.024
1055	炎沙门氏菌引起的食物中毒	of food poisoning caused	赵宏胜,李现军,郭素敏.	Hongsheng,Li Xianjun,Guo S	预防医学文献信息	ive medicine literature info	6	2	139	2000	sn.1672-9153.2000.02.070
1056	人员沙门氏菌菌型分布及	bution and drug resistance	陈建辉,谢一俊,郭维植.	n Jianhui,Xie Yijun,Guo We	海峡预防医学杂志	s Journal of Preventive Me	6	2	43	2000	sn.1007-2705.2000.02.025
1057	门氏菌药敏试验及噬菌体	m drug susceptibility test a	李萍.	Li Ping.	河南预防医学杂志	n Journal of Preventive Me	-	4	246	2000	sn.1006-8414.2000.04.035
1058	特沙门氏菌致化脓性脑膜	nt meningitis caused by Sal	卢德国,朱德全.	Lu De,Zhu Dequan.	齐鲁医学检验	Qilu Medical Testing	11	4	53	2000	sn.1673-5013.2000.04.043
1059	鼠伤寒沙门氏菌病流行病学	analysis of typhoid salmone	周文华,吴惠,李永杰.	ou Wenhua,Wu Hui,Li Yong	河南预防医学杂志	n Journal of Preventive Me	-	2	85-86	2000	sn.1006-8414.2000.02.008
1060	病性大肠杆菌、沙门氏菌	pathogenic Escherichia co	宁,刘书亮,陶勇,吴琦,黄勇	Shuliang,Tao Yong,Wu Qi,H	西南农业学报	urnal of Southwest Agricult	13	Z1	84-90	2000	sn.1001-4829.2000.z1.016
1061	一起食物中毒的调查与分析	and analysis of a case of fo	肖安保.	Xiao Anbao.	疾病控制杂志	journal of disease control	4	3	278	2000	sn.1674-3679.2000.03.038
1062	儿结核性脑膜炎16例误诊分	itis in children16Analysis o	王建怡.	Wang Jianyi.	临床误诊误治	l misdiagnosis and mistrea	13	6	434	2000	sn.1002-3429.2000.06.033
1063	一起食物中毒的病原学分析	analysis of a case of food	翠琴,吴惠琴,吴学琴,高志	,Wu Huiqin,Wu Xueqin,Ga	中国卫生检验杂志	ese Journal of Health Inspe	10	6	746-747	2000	sn.1004-8685.2000.06.077
1064	克苏地区鸡病流行与防治	revention of chicken diseas	李云侠,邱之华,邓红江,何	unxia,Qiu Zhihua,Deng Hor	中国兽医杂志	Chinese Veterinary Journal	26	8	40-41	2000	sn.0529-6005.2000.08.039
1065	素对4666株医院致病菌的	66Analysis of drug resistar	庆兴,潘发愤,王邦松,周铁	an is angry,Wang Bangson	浙江临床医学	Zhejiang Clinical Medicine	2	5	297-299	2000	sn.1008-7664.2000.05.005
1066	泻病源菌分型及药敏试验	pathogenic bacterial typing	李景云,黄上媛,汪力亚,宋敏	uang Shangyuan,Wang Liy	传染病信息	fectious disease informatio	-	3	135-136	2000	-
1067	产妇肠道致病菌特征与院	ic bacteria and nosocomial	辛辛,云雅丽,孙丽萍,梁建	n,Yun Yali,Sun Liping,Liang	中国微生态学杂志	inese Journal of Microecolo	12	1	28-29	2000	n.1005-376X.2000.01.012
1068	排污废水和附近海水的细菌	ushan City's Sewage Wast	黄备,唐静亮.	Huang Bei,Tang Jingliang.	海洋环境科学	Marine Environmental Scien	19	1	29-34	2000	sn.1007-6336.2000.01.007
1069	败血症430例病原菌及药	athogen and drug susceptib	郭云霞,王美巧,马绿珍.	unxia,Wang Meiqiao,Ma Lu	陕西医学杂志	Shaanxi Medical Journal	29	7	387-388	2000	sn.1000-7377.2000.07.001
1070	伤寒少门氏菌骨关节炎1例	phimurium osteoarthritis1	张合先,周亚鹏.	Zhang Hexian,Zhou Yapeng	实用放射学杂志	ournal of Practical Radiolog	-	7	-	2000	-
1071	化中引起胚胎死亡原因的	e causes of embryonic dea	梁俊明,托尔更,袁立岗.	g Junming,Torgen,Yuan Lig	中国禽业导刊	China Poultry Industry Guid	-	1	26	2000	-

1072	骨关节感染	Bone and joint infection	王云钊.	Wang Yunzhao.	实用医学影像杂志	nal of Practical Medical Ima	-	1	-	2000	-
1073	宾馆、饭店酱卤肉卫生合格	f Braised Pork with Sauce i	陈学威,姜松法.	Chen Xuewei,Jiang Songfa.	浙江预防医学	hejiang Preventive Medicin	12	4	39	2000	sn.1007-0931.2000.04.028
1074	并溶血性尿毒综合征一例	l fever combined with hem	张力,安华松.	tension,Anhua Song.	贵州医药	Guizhou Medicine	24	6	342	2000	n.1000-744X.2000.06.046
1075	地区蛋鸡疾病流行情况及	evention of laying hen dise	磊,刘思当,叶来敏,谢连成,王	Sidang,Ye Laimin,Xie Lianc	山东家禽	Shandong poultry	-	6	22-23	2000	sn.1673-1085.2000.06.013
1076	起中小学伤寒暴发的调查分	yphoid fever outbreak in	宋鲁平,桑耀峰,周建平,裴丽	ping,Sang Yaofeng,Zhou	疾病监测	disease surveillance	-	11	413-414	2000	-
1077	武鸣县一起伤寒暴发流行的	k of typhoid fever in Wu	李保才	Li Baocai	右江民族医学院学报	ujiang Medical College fo	-	5	752	2000	-
1078	副伤寒暴发流行的病原学	etiology of an outbreak	傅武良,张政	Fu Wuliang,Zhang Zheng	中华流行病学杂志	nese Journal of Epidemic	-	4	1	2000	-
1079	起伤寒暴发流行的调查报	report on an outbreak of	民,王鲜梅,杨俊杰,罗磊,杨	anmei,Yang Junjie,Luo L	河南预防医学杂志	Journal of Preventive M	-	4	217	2000	/j.cnki.hnjpm.2000.04.026
1080	起水型伤寒暴发的调查分	ysis of an outbreak of wa	华军,梁宇群,龙海泉,余永	g Yuqun,longhaiquan,Yu	解放军预防医学杂志	ve Medicine of the Peopl	-	4	300	2000	04/j.cnki.jyyx.2000.04.030
1081	仔猪副伤寒暴发	ratyphoid outbreak in pig	董恒勋,赵斌	Dong Hengxun,Zhao Bin	中国兽医杂志	Chinese Veterinary Journa	-	7	37	2000	-
1082	水县伤寒暴发流行的调查报	the outbreak of typhoid f	拜有庆	Bai Youqing	疾病监测	disease surveillance	-	5	188	2000	-
1083	下陈镇伤寒暴发流行病学调	ysis of typhoid fever outb	杨年忠,赵善强	g Ninzhong,Zhao Shanq	职业与健康	Occupation and health	-	5	77-78	2000	9/j.cnki.zyyjk.2000.05.074
1084	关镇一起伤寒暴发的流行病	n outbreak of typhoid fev	陈文林,娄山,钟俊伦	Wenlin,Loushan,Zhong J	中华流行病学杂志	nese Journal of Epidemic	-	2	1	2000	-
1085	县一起伤寒暴发流行的调查	of an outbreak of typhoid	刘问	Liu asked	疾病监测	disease surveillance	-	4	134	2000	-
1086	食物引起的甲型副伤寒暴发	outbreak of paratyphoid	张国兴	Zhang Guoxing	浙江预防医学	ejiang Preventive Medici	-	2	23-24	2000	sn1007-0931.2000.02.012
1087	伤寒暴发流行93例报告	fever outbreak93Examp	莫爱颜	Mo Aiyan	实用医学杂志	urnal of Practical Medici	-	2	132-133	2000	-
1088	起某部水型伤寒暴发流行调	ater-borne typhoid fever i	E,陈泽建,李建明,胥明玉,对	n Zejian,Li Jianming,Xu M	西南国防医药	west National Defense M	-	1	62-63	2000	-
1089	山区农村伤寒暴发流行的	break of typhoid fever in a	黄廷松	Huang Tingsong	中国城乡企业卫生	giene in urban and rural a	-	1	1	2000	5/j.1003-5052.2000.01.023
1090	和副溶血性弧菌共同引起的	ng caused by Salmonella de	刘红,刘秀锋,陈伟,潘珍瑜	g,Liu Xiufeng,Chen Wei,Pan	中国卫生检验杂志	ese Journal of Health Inspe	11	2	209	2001	sn.1004-8685.2001.02.058
1091	患者的脑脊液分离出1株肠	of patients with traumatic b	晓群,李珍大,王卫萍,邵海	Li Zhenda,Wang Weiping,S	临床检验杂志	journal of clinical testing	19	4	245-245	2001	n.1001-764X.2001.04.045
1092	炎型沙门菌食物中毒的调	n a case of Salmonella Ente	史旭明,宁鸣.	Shi Xuming,Ning Ming.	预防医学情报杂志	of Preventive Medicine Info	17	3	216-216	2001	sn.1006-4028.2001.03.069
1093	从业人员沙门氏菌带菌状况	s of Salmonella carrier statu	方,颜湘,陈小丽,王海燕,梁	ang,Chen Xiaoli,Wang Haiya	中国卫生监督杂志	ese Journal of Health Superv	8	3	105-106	2001	sn.1077-6131.2001.03.005
1094	甲型副伤寒杆菌药敏试验	susceptibility test of strain	华,赵婴,李晓英,王丽扬,刘	ao Ying,Li Xiaoying,Wang Li	临床荟萃	clinical collection	16	14	654-655	2001	n.1004-583X.2001.14.028
1095	地区鸡沙门氏菌病流行病	gation of chicken salmonel	庆华,亢文华,李平安,关平	ng Wenhua,Li Pingan,Guan	中国兽医杂志	Chinese Veterinary Journal	37	9	22-23	2001	sn.0529-6005.2001.09.011
1096	鼠林沙门氏菌引起的食物中	food poisoning caused by S	斯友全,周天平.	Si Youquan,Zhou Tianping.	职业与健康	Occupation and health	17	2	50-51	2001	sn.1004-1257.2001.02.047
1097	伤寒沙门氏菌肠炎并发败	onella typhimurium enterit	周文华,吴惠.	Zhou Wenhua,Wu Hui.	河南预防医学杂志	n Journal of Preventive Me	12	6	323	2001	sn.1006-8414.2001.06.033
1098	沙门氏菌引起的新生儿室院	in neonatal rooms caused	姚英民,陈红武,庄小青.	min,Chen Hongwu,Zhuang	新生儿科杂志	Journal of Neonatology	16	2	88-89	2001	sn.1673-6710.2001.02.028
1099	尿液和血液中分离出甲	s isolated from nephrosis,	熊桃菊.	Xiong Taoju.	临床检验杂志	journal of clinical testing	19	1	50	2001	n.1001-764X.2001.01.034
1100	中穿刺液分离出肠炎沙门氏	from blood and abscess as	郑新兰.	Zheng Xinlan.	职业与健康	Occupation and health	17	9	116-116	2001	sn.1004-1257.2001.09.113
1101	伤寒并发噬血细胞综合征1	licated by hemophagocytic	王又和,雷幸荣,蔡子光.	Youhe,Lei Xingrong,Cai Zig	内科急危重症杂志	f Internal Medicine and Cri	7	4	183	2001	sn.1007-1024.2001.04.028
1102	菌致腰椎化脓性骨髓炎并	complicated by sepsis caus	包贻顺,张晓峰,王惠敏.	hun,Zhang Xiaofeng,Wang	第四军医大学学报	of Fourth Military Medical U	22	10	867	2001	sn:1000-2790.2001.10.028
1103	古地区鸡沙门氏菌流行病学	gation of Salmonella in chic	,张庆华,亢文华,李平安,关	hua,Kang Wenhua,Li Pinga	内蒙古兽医	r Mongolia Veterinary Med	-	1	16-17	2001	-
1104	白林沙门氏菌食物中毒的调	on a case of salmonella foo	国,朱玲,翁小林,樊莉玲,唐	Ling,Weng Xiaolin,Fan Lili	遵义医学院学报	urnal of Zunyi Medical Colle	24	4	362366	2001	sn.1000-2715.2001.04.034
1105	化后期死亡鸡胚的细菌分	en embryos that died at th	刚,张再清,刘根强,马勋,付	ng Zaiqing,Yan Genqiang,M	石河子大学学报（自然科学版	hezi University (Natural Sci	5	2	143-144	2001	sn.1007-7383.2001.02.016
1106	起沙门氏菌食物中毒的调	into a case of salmonella fo	大培,周维山,苏良放,陈志	Weishan,Su Liangfang,Che	预防医学情报杂志	of Preventive Medicine Info	17	2	125-125	2001	sn.1006-4028.2001.02.051
1107	初诊时误诊血液病38例临床	ring initial diagnosis of typ	徐聪琴,谢文远,张宣义.	ngqin,Xie Wenyuan,Zhang X	浙江实用医学	Zhejiang Practical Medicine	6	2	49-50	2001	sn.1007-3299.2001.02.034
1108	区小儿沙门菌感染菌株群	lla infection strains in child	周文华,冯喜全,吴惠.	u Wenhua,Feng Xiquan,Wu	河南预防医学杂志	n Journal of Preventive Me	12	3	129-130	2001	sn.1006-8414.2001.03.001

1109	地区1990～1999年腹泻病	1990~1999Annual surveilla	牛桓彩,王连秀,赵维勇.	ancai,Wang Lianxiu,Zhao W	中华流行病学杂志	inese Journal of Epidemiolo	22	1	70-71	2001	sn:0254-6450.2001.01.024
1110	门菌污染辣子鸡所致食物	used by spicy chicken conta	祝洪山,李汉芳,陈淑英,杨	ngshan,Li Hanfang,Chen Sh	预防医学文献信息	ive medicine literature info	7	5	550-550	2001	sn.1672-9153.2001.05.052
1111	-2000年伤寒噬菌体分型与	ual Typhoid Phage Typing a	施平,庄菱.	Shi Ping,Zhuang Ling.	疾病监测	disease surveillance	16	10	365-367	2001	sn.1003-9961.2001.10.005
1112	甲型副伤寒病人的临床观察	n and nursing care of patie	袁月莲.	Yuan Yuelian.	当代护士（学术版）	nporary Nurses (Academic I	-	10	18	2001	sn.1006-6411.2001.10.012
1113	邓柏林沙门氏菌食物中毒	a case of salmonella food p	刘和丽,郗可怡.	Liu Heli,Xi Keyi.	山西预防医学杂志	xi Journal of Preventive Me	10	1	33	2001	-
1114	寒沙门氏菌引起足部感染	fection caused by Salmone	白洛琴,雷海飞.	Bai Luoqin,Lei Haifei.	中华流行病学杂志	inese Journal of Epidemiolo	22	2	95	2001	sn:0254-6450.2001.02.028
1115	菌血症成年患者的脉管感	lar infection in adult patien	-	-	国外医药（抗生素分册）	Medicine (Antibiotics Part '	22	5	238	2001	-
1116	都柏林沙门氏菌食物中毒	f salmonella food poisoning	李莉,马秀英.	Li Li,Ma Xiuying.	预防医学情报杂志	of Preventive Medicine Info	17	1	59-60	2001	sn.1006-4028.2001.01.049
1117	由肠炎沙门氏菌引起的食物	poisoning caused by Salmo	张群,李晓霞,辛颖.	hang Qun,Li Xiaoxia,Xin Yin	中国公共卫生	Chinese public health	17	2	183	2001	-
1118	培养中检出莫斯科沙门菌	oscow detected in blood cu	关茹,庞登文,徐斌.	uan Ru,Pang Dengwen,Xu B	临床检验杂志	journal of clinical testing	19	1	52	2001	n.1001-764X.2001.01.035
1119	由肠炎沙门菌引起的食物	poisoning caused by Salmo	王琳娜.	Wang Linna.	海峡预防医学杂志	s Journal of Preventive Me	7	3	31	2001	sn.1007-2705.2001.03.014
1120	氏菌产超广谱β-内酰胺酶	uces extended spectrumβ-	李双林,赵常芬.	Li Shuanglin,Zhao Changfen	齐鲁医学检验	Qilu Medical Testing	12	3	36-37	2001	sn.1673-5013.2001.03.023
1121	伤感染猪霍乱沙门氏菌死	n choleraesuis salmonella in	岑丹辉,黄卫.	Cen Danhui,Huang Wei.	广西预防医学	Guangxi Preventive Medicin	7	3	170	2001	n.1673-758X.2001.03.037
1122	菌感染致不全性肠梗阻的	complete intestinal obstruc	王琼英.	Wang Qiongying.	现代医药卫生	modern medicine and healt	17	1	68	2001	sn.1009-5519.2001.01.085
1123	鼠伤寒沙门菌化脓性脑膜	lent meningitis caused by S	仇爱明.	Qiu Aiming.	中华传染病杂志	se Journal of Infectious Dis	19	4	228	2001	sn:1000-6680.2001.04.026
1124	肠炎沙门菌引起的食物中	poisoning caused by Salm	,朴斗铉,陈龙男,朱明姬,金	oo-hyun,Chen Longnan,Zh	中国卫生检验杂志	ese Journal of Health Inspe	11	1	114	2001	sn.1004-8685.2001.01.070
1125	外环境水样中沙门氏菌检测	lmonella in environmental	吴厚营.	Wu Houying.	实用新医学	Practical new medicine	3	10	-	2001	-
1126	性脑膜炎脑脊液酶学检测	d enzyme test results in ch	显芬,徐光芝,郝轶梅,王静	en,Xu Guangzhi,Hao Yimei,V	上海医学检验杂志	ournal of Medical Laborator	16	3	170-171	2001	sn.1673-8640.2001.03.021
1127	58例急性腹泻流行病学分	iological analysis of acute d	陆红,从纬,金国娴,孙方.	From latitude,Jin Guoxian,S	辽宁医学杂志	Liaoning Medical Journal	15	1	26-27	2001	sn.1001-1722.2001.01.016
1128	糖的鼠伤寒沙门菌医院感	onella typhimurium nosoco	段英梅,张京文,李显波,邓	ngmei,Zhang Jingwen,Li Xia	上海医学检验杂志	ournal of Medical Laborator	16	3	152	2001	sn.1673-8640.2001.03.032
1129	寒沙门氏菌引起的食物中	of food poisoning caused l	王晶玲,姜海燕.	Wang Jingling,Jiang Haiyan	中华医学写作杂志	nese Journal of Medical Wr	-	8	-	2001	-
1130	伤寒合并右髋关节脓肿1例	combined with right hip ab	刘成林,崔仲华,王菲.	englin,Cui Zhonghua,Faye '	哈尔滨医科大学学报	nal of Harbin Medical Unive	35	2	88	2001	sn.1000-1905.2001.02.033
1131	6例化脓性脑膜炎临床分析	analysis of purulent menin	志平,易益芳,胡金平,卢志	ing,Yi Yifang,Hu Jinping,Lu	国际医药卫生导报	ational Medical and Health	-	8	50，52	2001	sn.1007-1245.2001.08.041
1132	起食物中毒分离出二种沙门	monella isolated from food	钱瑶,严涛.	Qian Yao,Yan Tao.	中国卫生检验杂志	ese Journal of Health Inspe	11	6	760	2001	sn.1004-8685.2001.06.090
1133	宁地区部分省级医院污水	ection situation of some pr	毅中,李溥仁,郭鹏,马忠文	ng,Li Puren,Guo Peng,Ma Z	医学动物防制	medical animal control	17	2	39-40	2001	sn.1003-6245.2001.02.022
1134	甲型副伤寒沙门菌耐药性检	of drug resistance of Salm	陶晓东,金法祥.	Tao Xiaodong,Jin Faxiang.	现代中西医结合杂志	grated Traditional Chinese	10	10	975	2001	sn.1008-8849.2001.10.083
1135	菌产生超广菌β—内酰胺酶	roduces hyperbacteriaβ—	王倩,年华.	Wang Qian,years.	临床检验杂志	journal of clinical testing	19	2	104	2001	-
1136	髓细胞白血病合并伤寒感	emia combined with typho	张勇,张一军.	Zhang Yong,Zhang Yijun.	第三军医大学学报	the Third Military Medical	23	3	345	2001	sn:1000-5404.2001.03.053
1137	液中同时检出纽波特沙门	ted in both cerebrospinal f	孙学春,朱德全.	Sun Xuechun,Zhu Dequan.	临床检验杂志	journal of clinical testing	19	1	38	2001	n.1001-764X.2001.01.033
1138	一起沙门氏菌食物中毒调查	into a case of salmonella fo	张继荣,王翠霞.	Zhang Jirong,Wang Cuixia.	安徽预防医学杂志	i Journal of Preventive Med	-	1	33-34	2001	-
1139	89年—1998年沙门氏菌型	r—1998Salmonella type di	贾云.	Jia Yun.	实用新医学	Practical new medicine	3	4	-	2001	-
1140	伤寒380例临床分析	oid fever380Case clinical an	陈南晖.	Chen Nanhui.	遵义医学院学报	urnal of Zunyi Medical Colle	24	6	536538	2001	sn.1000-2715.2001.06.032
1141	物饲料中细菌污染状况调	on bacterial contaminatio	史思,应郁敏,沙洁,杨淑青	Ying Yumin,Sha Jie,Yang Sh	动物学杂志	journal of zoology	36	6	38-39	2001	sn.0250-3263.2001.06.009
1142	市服务行业人员沙门氏菌携	rriage among service indus	黄烈泓,韩世兰.	Huang Liehong,Han Shilan.	湖北预防医学杂志	i Journal of Preventive Med	12	1	34	2001	sn.1006-2483.2001.01.037
1143	沙门氏菌肠炎重症失水输	ere dehydration in neonat	苏雅荣,张俐.	Su Yarong,Zhang Li.	黑龙江医学	Heilongjiang Medicine	25	7	502-502	2001	sn.1004-5775.2001.07.018
1144	副伤寒沙门氏菌感染159例	A infection159A prelimina	蒲海.	Puhai.	中华医学写作杂志	nese Journal of Medical Wr	-	19	-	2001	-
1145	市甲型副伤寒沙门菌耐药性	ction of Salmonella Paratyp	金法祥,李水法.	Jin Faxiang,Li Shuifa.	医学文选	Selected Medical Essays	20	5	687	2001	sn.1673-6575.2001.05.093

1146	甲型副伤寒沙门氏菌的鉴	ptibility testing of Salmone	顾雪影.	Gu Xueying.	预防医学文献信息	ive medicine literature info	7	3	268-269	2001	sn.1672-9153.2001.03.031
1147	寒沙门氏菌对23例抗生素的	phitwenty threeAntibiotic s	王云芳.	Wang Yunfang.	桂林医学	Guilin Medicine	-	3	63	2001	-
1148	腹泻粪便中检出病牛沙门	e Salmonella from infant d	尚亚琳,周文华.	Shang Yalin,Zhou Wenhua.	河南职工医学院学报	of Henan Workers Medical	13	4	376	2001	sn.1008-9276.2001.04.075
1149	一起食物中毒报告	A report of food poisoning	王俊明.	Wang Junming.	航空军医	aviation medic	29	4	163-164	2001	-
1150	氏菌引起院内交叉感染的	of a nosocomial cross-infe	陈辉.	Chen Hui.	湖北预防医学杂志	i Journal of Preventive Me	12	6	37	2001	sn.1006-2483.2001.06.031
1151	新港沙门氏菌食物中毒调	n a case of salmonella food	李冬琴.	Li Dongqin.	实用预防医学	ractical preventive medicin	8	1	47-47	2001	sn.1006-3110.2001.01.036
1152	起由沙门氏菌引起的食物中	food poisoning caused by s	白水仙,付志娟,张美珍.	arcissus,Fu Zhijuan,Zhang M	内蒙古科技与经济	ia Science and Technology	-	6	144-144	2001	sn.1007-6921.2001.06.095
1153	肠炎沙门引起的食物中毒	poisoning caused by Salm	朴松子,朴斗铉.	Park Songja,Park Doo-hyun	中国卫生检验杂志	ese Journal of Health Inspe	11	1	114	2001	-
1154	首发TTV感染致急性瘀胆	TVAcute biliary stasis hepat	张世萍,贺远,黄致模.	g Shiping,He Yuan,Huang Z	中华肝脏病杂志	hinese Journal of Hepatolo	9	1	15	2001	sn:1007-3418.2001.01.030
1155	夏秋季腹泻病原与药敏情	rs and drug susceptibility o	敏,夏光明,李景云,崔恩博	Xia Guangming,Li Jingyun,	肝脏	liver	-	S1	92-93	2001	-
1156	上海市普陀区食品微生物	microbial inspection result	罗颖.	Luo Ying.	广西预防医学	Guangxi Preventive Medicin	7	1	46-47	2001	n.1673-758X.2001.01.016
1157	副伤寒甲150例临床分析	yphoid A150Case clinical a	张艳梅,杨微波,骈淮燕.	anmei,Yang Weibo,Parallel	中国综合临床	hina Comprehensive Clinic	17	4	280-281	2001	sn.1008-6315.2001.04.022
1158	儿沙门菌感染35例临床分	fection in children35Case c	黄璟,严波,郭乃宏.	ang Jing,Yan Bo,Guo Naiho	贵阳医学院学报	nal of Guiyang Medical Co	26	6	519-520	2001	sn.1000-2707.2001.06.020
1159	型副伤寒沙门菌分离株16	Sonella paratyphi A isolates	诚,阚飙,吕太富,游旅,张	Kan Biao,Lu Taifu,Travel,Z	疾病监测	disease surveillance	16	12	445-447	2001	sn.1003-9961.2001.12.005
1160	和县一起沙门氏菌中毒事	la poisoning incident in Mi	来身德.	Come to body virtue.	医学动物防制	medical animal control	17	10	524-525	2001	sn.1003-6245.2001.10.011
1161	寒沙门氏菌感染爆发流行	rns from outbreaks of Salm	刘智慧,邱向莉,何荣海.	Zhihui,Qiu Xiangli,He Rong	承德医学院学报	nal of Chengde Medical Co	18	2	117-118	2001	sn.1004-6879.2001.02.017
1162	门氏菌污染牛肉引起食物	od poisoning caused by Sal	郭新春,苗志红.	Guo Xinchun,Miao Zhihong	实用医技	Practical medical skills	8	8	622	2001	sn.1671-5098.2001.08.080
1163	起沙门氏菌食物中毒的调	into a case of salmonella f	加恒,刘喜梅,杨春梅,刘小	Liu Ximei,Yang Chunmei,Li	医学动物防制	medical animal control	17	12	659-660	2001	sn.1003-6245.2001.12.022
1164	甲型副伤寒沙门菌的培养	eptibility results of Salmon	储从家,孔繁林,吴惠玲.	Congjia,Kong Fanlin,Wu Hu	云南医药	Yunnan Medicine	-	6	450-451	2001	-
1165	骨髓细菌培养阳性病人NA	and bone marrow bacteria	王永伦,谈介凡,骆科允.	g Yonglun,Tan Jiefan,Luo K	遵义医学院学报	urnal of Zunyi Medical Coll	24	1	82-83	2001	sn.1000-2715.2001.01.051
1166	血培养中沙门菌的检测及	ty analysis of Salmonella in	沈忠海.	Shen Zhonghai.	上海医学检验杂志	ournal of Medical Laborato	16	5	295-295	2001	sn.1673-8640.2001.05.034
1167	腹泻检出产碱普罗菲登斯	genes detected in acute di	陈自武.	Chen Ziwu.	蚌埠医学院学报	rnal of Bengbu Medical Col	26	2	160	2001	sn.1000-2200.2001.02.041
1168	门菌两个血清型混合感染	used by mixed infection of	,林凤梅,邹立镇,黄金萍,	ngmei,Zouli Town,Huang J	中国卫生检验杂志	ese Journal of Health Inspe	11	5	633-634	2001	sn.1004-8685.2001.05.086
1169	养殖动物鲜肉中分离耐抗	nonella from retail fresh m	纪谷.	Jigu.	中华医学信息导报	ese Medical Information H	-	23	21	2001	-
1170	败血症病原菌分析与抗生	cteria and selection of ant	吕菁,赵芳,张学红.	ing,Zhao Fang,Zhang Xueh	宁夏医学院学报	rnal of Ningxia Medical Col	23	2	96-98	2001	sn.1674-6309.2001.02.008
1171	儿非伤寒沙门菌感染的检	s of neonatal non-typhoid S	胡莉萍,汪丽玉,刘连生.	Liping,Wang Liyu,Liu Liansh	徐州医学院学报	rnal of Xuzhou Medical Col	21	6	465-466	2001	sn.1000-2065.2001.06.018
1172	物穿刺液中分离出肠炎沙	is isolated from chest wall	冯刚,郝凤艳.	Feng Gang,Hao Fengyan.	化工劳动保护	Chemical labor protection	-	8	291	2001	-
1173	地区环境中细菌分布的	acterial distribution in the e	曾国宇,李明远,张苏.	g Guoyu,Li Mingyuan,Zhang	预防医学情报杂志	of Preventive Medicine Inf	17	3	141-141,143	2001	sn.1006-4028.2001.03.007
1174	1起食物中毒的调查分析	ation and analysis of food	沈波.	Shen Bo.	预防医学情报杂志	of Preventive Medicine Inf	17	6	466-466	2001	sn.1006-4028.2001.06.026
1175	沙门氏菌引起食物中毒的	case of food poisoning ca	黄小明.	Huang Xiaoming.	宜春学院学报	Journal of Yichun University	-	S1	-	2001	-
1176	伤寒沙门菌食物中毒的实	a case of Salmonella typhi	李铁墙,李秀昵,李胜春.	Tieqiang,Li Xiuni,Li Shengch	中国卫生检验杂志	ese Journal of Health Inspe	11	5	637	2001	sn.1004-8685.2001.05.089
1177	寒沙门氏菌引起食物中毒	e of food poisoning caused	陈少刚,董磊.	Chen Shaogang,Dong Lei.	宁夏医学杂志	Ningxia Medical Journal	23	11	691-691	2001	sn.1001-5949.2001.11.032
1178	布利丹沙门菌引起的食物	poisoning caused by Salm	芳,靳云清,李高波,彭斌,蒋	Yunqing,Li Gaobo,Peng Bi	中华预防医学杂志	e Journal of Preventive Me	35	4	224	2001	sn:0253-9624.2001.04.038
1179	年人沙门菌肠炎32例临床	eritis in the elderly32Case	王晓峰,张文瑾,李景云.	Xiaofeng,Zhang Wenjin,Li J	中国人兽共患病杂志	Chinese Journal of Zoonose	17	6	129-129	2001	sn.1002-2694.2001.06.044
1180	由沙门氏菌所致食物中毒	food poisoning caused by	鲍庆玲,和顺,黄成.	ngling,Harmonious,Huang	职业与健康	Occupation and health	17	11	56-56	2001	sn.1004-1257.2001.11.052
1181	都柏林沙门菌感染1例	nella infection in Dublin1ex	于农,许勇臣,崔金凤.	Nong,Xu Yongchen,Cui Jinf	沈阳部队医药	Shenyang Army Medicine	-	4	367	2001	-
1182	新生儿败血症107例临床分	tal sepsis107Case clinical a	张学红,闫宝兰,吕菁.	ng Xuehong,Yan Baolan,Lu	宁夏医学院学报	rnal of Ningxia Medical Col	23	5	321-323	2001	sn.1674-6309.2001.05.004

1183	康人群携带沙门菌的调查	eport on Salmonella carriag	,陈智,齐小保,徐小菊,龙一	en Zhi,Qi Xiaobao,Xu Xiaoj	中国卫生检验杂志	ese Journal of Health Inspe	11	6	713	2001	sn.1004-8685.2001.06.047
1184	已肠炎沙门菌引起的食物中	poisoning caused by Salmo	许少洪,孙凤琪.	Xu Shaohong,Sun Fengqi.	中国卫生检验杂志	ese Journal of Health Inspe	11	3	381-381	2001	sn.1004-8685.2001.03.087
1185	食品中几种微生物污染监测	microbial contaminations i	三艳秋,赵共和,杨修军,杨红	Zhao Gonghe,Yang Xiujun,Y	中国卫生检验杂志	ese Journal of Health Inspe	11	1	69-70	2001	sn.1004-8685.2001.01.036
1186	昆合性食物中毒的流行病学	analysis of a case of mixed	汤勇广.	Tang Yongguang.	代临床医学生物工程学杂	Modern Clinical Medical Bio	7	2	137	2001	sn.1674-1927.2001.02.036
1187	乙鼠伤寒沙门氏菌感染的防	eatment of Salmonella typ	卜兆玮,封民平.	Bu Zhaowei,Feng Minping.	中国医学理论与实践	ese Medical Theory and Pra	-	8	-	2001	-
1188	斤、饮食服务业从业人员沙	employees in public place	谭海玲,柯碧霞,黎薇,钟豪杰	iling,Ke Bixia,Li Wei,Zhong	中国卫生检验杂志	ese Journal of Health Inspe	11	4	444-444	2001	sn.1004-8685.2001.04.037
1189	儿鼠伤寒沙门氏菌感染的	of neonatal Salmonella typ	周媛.	Zhou Yuan.	现代医药卫生	modern medicine and healt	17	8	641	2001	sn.1009-5519.2001.08.055
1190	菌感染乳及乳制品引起的食	oning caused by Salmonell	万生,谭彩霞,罗厚洪,梁平生	,Tan Caixia,Luo Houhong,L	广州医药	Guangzhou Medicine	32	5	61	2001	sn.1000-8535.2001.05.050
1191	夫雷登沙门氏菌引起的食	of food poisoning caused b	杜昌海,杨周祥,段继芳.	nghai,Yang Zhouxiang,Duan	中国卫生监督杂志	ese Journal of Health Super	8	1	8-9	2001	sn.1077-6131.2001.01.006
1192	已沙门氏菌食物中毒调查报	ort on a case of salmonella	欧阳仁贵,罗建平,李硕.	ang Rengui,Luo Jianping,Li S	实用预防医学	ractical preventive medicin	8	4	298-298	2001	sn.1006-3110.2001.04.037
1193	沙门氏菌食物中毒的调查	nalysis of a case of salmon	刘添发,王淑文,周联.	Tianfa,Wang Shuwen,Zhou	中国公共卫生管理	ese public health managem	17	2	155-156	2001	sn.1001-9561.2001.02.064
1194	门诊腹泻患者粪便病原菌检	ria examination results of p	罗小铭,冯雪琴.	Luo Xiaoming,Feng Xueqin.	广东医学	Guangdong Medicine	22	8	697-698	2001	sn.1001-9448.2001.08.020
1195	伤寒沙门氏菌病80例临床观	salmonellosis80Case clinic	党胡琼.	Dang Hu Qiong.	遵义医学院学报	urnal of Zunyi Medical Colle	24	3	249-249	2001	sn.1000-2715.2001.03.041
1196	院近4年沙门菌耐药变迁分	nalysis on Salmonella Resis	戴自强.	Dai Ziqiang.	贵阳医学院学报	nal of Guiyang Medical Col	26	2	138-139	2001	sn.1000-2707.2001.02.018
1197	起沙门氏菌引起的食物中	food poisoning caused by s	文杰,吴建明,马占礼,高志	Wu Jianming,Ma Zhanli,Ga	中国学校卫生	Chinese school health	-	4	306	2001	sn.1000-9817.2001.04.052
1198	县输入性伤寒暴发流行的	eak of imported typhoid	蓝周灵	Lan Zhouling	广西预防医学	uangxi Preventive Medici	-	S1	6-8	2001	-
1199	起甲型副伤寒暴发流行调	ation of an outbreak of pa	蒋晓英	Jiang Xiaoying	广西预防医学	uangxi Preventive Medici	-	S1	21-22	2001	-
1200	丹县伤寒暴发流行的调查报	the outbreak of typhoid f	田兴寿,邹双燕	n Xingshou,Zou Shuang	广西预防医学	uangxi Preventive Medici	-	S1	29-31	2001	-
1201	水型伤寒暴发的流行病学	ation of an outbreak of w	黄世金	Huang Shijin	广西预防医学	uangxi Preventive Medici	-	S1	45-46	2001	-
1202	学甲型副伤寒暴发流行的	utbreak of paratyphoid fe	刘利华,谭德林	Liu Lihua,Tan Delin	广西预防医学	uangxi Preventive Medici	-	S1	64	2001	-
1203	伤寒暴发的流行病学调查	stigation report on an out	肖良	Xiao Liang	疾病监测	disease surveillance	-	12	473-474	2001	-
1204	族中学甲型副伤寒暴发流	atyphoid fever A in Ningy	何凡,吴春生,尹红,阙水祥	Chunsheng,Yin Hong,Qu	实用预防医学	actical preventive medici	-	6	424	2001	-
1205	工地伤寒暴发的相关因素	typhoid fever outbreak a	农智,高健华,江超穗	Zhi,Gao Jianhua,Jiang C	右江民族医学院学报	ujiang Medical College fo	-	6	911-912	2001	-
1206	伤寒暴发流行3年的临床研	break3Years of clinical re	周建芳;	Zhou Jianfang;	传染病信息	ectious disease informat	-	4	184-185	2001	-
1207	地发生伤寒暴发的流行病	typhoid fever outbreak a	杨勤保,江超穗,高健华,梁	,Jiang Chaosui,Gao Jian	广西预防医学	uangxi Preventive Medici	-	5	287-289	2001	-
1208	一起伤寒暴发流行病学调查	cal investigation of a typh	杨平生	Yang Pingsheng	镇江医学院学报	al of Zhenjiang Medical C	-	5	2	2001	sn.1671-7783.2001.05.106
1209	动血凝法在一起伤寒暴发	ion of passive hemagglu	罗学辉,张怡明	Luo Xuehui,Zhang Yiming	浙江预防医学	ejiang Preventive Medici	-	9	21	2001	ssn1007-0931.2001.09.015
1210	型副伤寒暴发疫情的控制	ts of an outbreak of para	王智琼	Wang Zhiqiong	职业与健康	Occupation and health	-	9	114	2001	-
1211	被动血凝试验在一起伤寒	on of passive hemagglut	建民,王文英,金莞尔,叶承	Vang Wenying,Jin Wan'e	中国卫生检验杂志	se Journal of Health Insp	-	4	469-470	2001	-
1212	型副伤寒暴发流行20例临	f paratyphoid A20Case c	吴芝兰	Wu Zhilan	临床儿科杂志	ournal of Clinical Pediatri	-	3	146-147	2001	-
1213	寒暴发的卫生流行病学分	logical analysis of typho	张萍;赵开胜;毕洪芹;	Ping;Zhao Kaisheng;Bi H	黑龙江医药	Heilongjiang Medicine	-	3	244-245	2001	-
1214	起伤寒暴发的流行病学分	al analysis of a typhoid f	张昆照,龚俊平,袁辉,吴树	,Gong Junping,Yuan Hu	疾病监测	disease surveillance	-	5	190-191	2001	-
1215	99年伤寒暴发疫情分析及	al typhoid fever outbreak	沁,何平,潘家秀,聂伟,姚光	ing,Pan Jiaxiu,Nie Wei,Y	中国预防医学杂志	e Journal of Preventive M	-	2	2	2001	-
1216	伤寒暴发的疫区处理	yphoid fever outbreak in	明姬,李秋荣,曲冬娟,王丽	i,Li Qiurong,Qu Dongjuan	中国卫生工程学	hinese sanitary engineeri	-	2	1	2001	sn.1671-4199.2001.02.029
1217	一起食源性伤寒暴发的调查	o an outbreak of foodbor	张敏娟	Zhang Minjuan	浙江预防医学	ejiang Preventive Medici	-	4	18	2001	ssn1007-0931.2001.04.011
1218	伤寒暴发的流行病学调查	estigation and analysis o	黄大成,田翠英,杨胜堂	heng,Tian Cuiying,Yang	疾病监测	disease surveillance	-	4	140-141	2001	-
1219	起甲型副伤寒暴发流行的	f an outbreak of paratyph	黄晓春,全文郎,吴国祯	ochun,Quan Wenlang,W	疾病监测	disease surveillance	-	4	141-142	2001	-

1220	5 1999年伤寒暴发流行的调	ation report on the outbr	周红,袁子耀,朱仁珍,石敏	uan Ziyao,Zhu Renzhen,	疾病监测	disease surveillance	-	3	109	2001	-
1221	型副伤寒暴发流行35例临	f paratyphoid A35Case c	吴芝兰	Wu Zhilan	浙江临床医学	Zhejiang Clinical Medicin	-	3	191-192	2001	-
1222	州省1999年伤寒暴发疫情	1999Analysis of annual	沁,何平,潘家秀,聂伟,姚光	ing,Pan Jiaxiu,Nie Wei,Y	疾病监测	disease surveillance	-	2	49-50	2001	-
1223	起输入性伤寒暴发的流行	ristics of an imported typ	冯永才	Feng Yongcai	预防医学情报杂志	f Preventive Medicine In	-	1	35	2001	-
1224	一起伤寒暴发的调查分析	d analysis of an outbreak	工伟,韩灿宇,郭影,薛浩,韩	an Canyu,Guo Ying,Xue	疾病监测	disease surveillance	-	1	16-17	2001	-
1225	区农村伤寒暴发流行的调	eak of typhoid fever in ru	每,何柱强,冉启鸿,彭平惠,	uqiang,Ran Qihong,Pen	疾病监测	disease surveillance	-	1	33	2001	-
1226	沙门菌引起婴幼儿迁延性	auses persistent diarrhea i	建辉,李海丹,谢一俊,吴珍	hui,Li Haidan,Xie Yijun,Wu	海峡预防医学杂志	s Journal of Preventive Me	8	2	78-78	2002	sn.1007-2705.2002.02.054
1227	年常熟市从业人员肠道沙	ric Salmonella testing amo	庆华,张缪伟,常伟冰,吴国	ng Miaowei,Chang Weibin	中国卫生检验杂志	ese Journal of Health Inspe	12	6	708-708	2002	sn.1004-8685.2002.06.055
1228	东农村腹泻病原菌的调查	ogenic bacteria of diarrhea	树生,林柳英,林成水,陈亢,	Lin Liuying,Lin Chengshui,C	现代预防医学	modern preventive medicin	29	6	782-783	2002	sn.1003-8507.2002.06.018
1229	症血培养菌株近十年的变	of neonatal septicemia blo	小梅,徐位仁,何林,李志光	aomei,Xu Weiren,He Lin,Li	临床儿科杂志	Journal of Clinical Pediatric	20	5	266-267	2002	sn.1000-3606.2002.05.004
1230	沙门氏菌引起集体食物中	of collective food poisoning	杨钦,刘晓辉.	Yang Qin,Liu Xiaohui.	公共卫生与预防医学	Health and Preventive Me	13	4	25	2002	sn.1006-2483.2002.04.016
1231	病患者血液中分离出都柏	lla Dublin from the blood	韩善桥,马聪,郝秀红,蒋学	ao,Ma Cong,Hao Xiuhong,Ji	海军总医院学报	rnal of Naval General Hosp	15	1	43-43	2002	sn.1009-3427.2002.01.013
1232	菌血症成年患者的脉管感	ular infection in adult patie	-	-	国外医药（抗生素分册）	Medicine (Antibiotics Part	23	2	封三-封四	2002	-
1233	症状的沙门氏菌食物中毒	lmonella food poisoning w	方向明,赵俊铁.	Clear direction,Zhao Juntie	公共卫生与预防医学	Health and Preventive Me	13	3	11	2002	sn.1006-2483.2002.03.013
1234	生肉沙门菌污染状况调查	n salmonella contaminatio	胡慧玲,陈伟伟.	Hu Huiling,Chen Weiwei.	海峡预防医学杂志	s Journal of Preventive Me	8	6	52-53	2002	sn.1007-2705.2002.06.029
1235	尔市1985～1999年食物中	985~1999annual food pois	卢顺德,王照明,强宏伟.	nde,Wang lighting,Qiang H	中国卫生监督杂志	ese Journal of Health Super	9	1	24-25	2002	sn.1077-6131.2002.01.018
1236	性腹泻病原菌调查及流行	of pathogenic bacteria of a	王吉英,魏可林.	Wang Jiying,Wei Kelin.	预防医学情报杂志	of Preventive Medicine Info	18	2	161-161	2002	sn.1006-4028.2002.02.037
1237	门氏菌引起食物中毒的调	n a case of food poisoning	肖荀.	Xiao Xun.	职业与健康	Occupation and health	18	9	60-61	2002	sn.1004-1257.2002.09.053
1238	田区污水生物性污染状况	pollution status of sewage	莫浩联,曾敏,朱浴辉,杨柳青	an,Zeng Min,Zhu Yuhui,Ya	实用预防医学	ractical preventive medicin	9	6	583-585	2002	sn.1006-3110.2002.06.003
1239	少见菌所致化脓性脑膜炎	ent meningitis caused by ra	易维芬,曹淑琴.	Yiweifen,Cao Shuqin.	中华儿科杂志	Chinese Journal of Pediatric	40	5	311	2002	-
1240	及公共场所服务行业人员	n catering and public servi	陈义忠,胡丽萍,高永堂,林智	ong,Hu Liping,Gao Yongtar	华南预防医学	uth China Preventive Medic	28	3	31-32	2002	sn.1671-5039.2002.03.013
1241	业人员沙门氏菌携带情况	nonella carriage among wo	邓丽娟,张卉,刘琦.	Deng Lijuan,Zhang Hui,Liu Q	职业与健康	Occupation and health	18	7	45-46	2002	sn.1004-1257.2002.07.044
1242	年细菌性食物中毒分析与	e Analysis and Control of B	刘中富.	Liu Zhongfu.	中国卫生监督杂志	ese Journal of Health Super	9	1	44-46	2002	sn.1077-6131.2002.01.028
1243	房两次肠道鼠伤寒暴发流	nteric murine typhoid feve	李英,宋江美.	Li Ying,Song Jiangmei.	中华医院感染学杂志	ournal of Hospital Infectiou	12	10	754756	2002	sn:1005-4529.2002.10.016
1244	寒沙门氏菌致感染性心内	endocarditis caused by Sal	富,陈增杰,李新祥,颜道茹,	hen Zengjie,Li Xinxiang,Yan	中华心血管病杂志	Journal of Cardiovascular	30	11	701	2002	sn:0253-3758.2002.11.019
1245	门氏菌污染牛肉引起的食	ood poisoning caused by Sa	刘文光,郝树芬,薛爱梅.	Wenguang,Hao Shufen,Xue A	山西预防医学杂志	xi Journal of Preventive Me	11	2	140	2002	-
1246	柏林沙门氏菌所致食物中	case of food poisoning cau	王先松,何建军,徐文杰.	g Xiansong,He Jianjun,Xu W	职业与健康	Occupation and health	18	4	55-56	2002	sn.1004-1257.2002.04.052
1247	抗某部炊事人员健康状况	cooking staff in a certain	金保中,吴景清,沈瑛,沈丽敏	,Wu Jingqing,Shen Ying,Sh	解放军预防医学杂志	tive Medicine of the People	20	2	142-142	2002	sn.1001-5248.2002.02.028
1248	沙门菌引起食物中毒的调	is of a case of food poisoni	孙东胜,杨自成.	Sun Dongsheng,Yang Zichen	中国乡村医药	Chinese rural medicine	9	3	52-53	2002	sn.1006-5180.2002.03.057
1249	鸡场鸡胚与种蛋的细菌分	from chicken embryos and	刘,刘根强,张再清,李泽宇,王	enqiang,Zhang Zaiqing,Li Z	畜禽业	vestock and poultry indust	-	9	6-7	2002	sn.1008-0414.2002.09.003
1250	热患者血液标本菌群及血	ological analysis of blood sa	韦家秀,杨俊,王义光.	Jiaxiu,Yang Jun,Wang Yigu	中国微生态学杂志	inese Journal of Microecolo	14	5	290-291	2002	n.1005-376X.2002.05.019
1251	感染合并异型淋巴细胞增多	ined with atypical lympho	,苏先狮,龚国忠,赖力英,陈	anshi,Gong Guozhong,Lai L	中日友好医院学报	of China-Japan Friendship	16	1	32-33	2002	sn.1001-0025.2002.01.010
1252	由德尔卑沙门菌引起的食	d poisoning caused by Salm	蒋秀芳.	Jiang Xiufang.	江苏预防医学	Jiangsu Preventive Medicin	13	1	41-42	2002	sn.1006-9070.2002.01.025
1253	沙门氏菌引起的食物中毒	case of food poisoning ca	聂伟光,张灵珠.	Nie Weiguang,Zhang Lingzhu	中国热带医学	Chinese tropical medicine	-	S1	48	2002	-
1254	名饮食和公共场所从业人	n the carriage of pathogeni	林剑琴.	Lin Jianqin.	福建医药杂志	Fujian Medical Journal	24	6	162-162	2002	sn.1002-2600.2002.06.135
1255	寒沙门氏菌所致食物中毒	od poisoning caused by Sal	李莉,王晓,姜学刚.	Li,Wang Xiao,Jiang Xuegan	山东食品科技	Shandong Food Technology	-	10	25	2002	-
1256	病患者血液中分离出者柏	ella isolated from blood of	韩善桥,马聪.	Han Shanqiao,Ma Cong.	海军总医院学报	rnal of Naval General Hosp	15	1	43	2002	-

1257	上城区沙门菌菌型分布动	tion of Salmonella strains i	何连生.	He Liansheng.	中国卫生检验杂志	ese Journal of Health Inspe	12	2	205-205	2002	sn.1004-8685.2002.02.049
1258	型副伤寒沙门菌药敏试	sceptibility test of Salmone	谢林红.	Xie Linhong.	贵阳医学院学报	nal of Guiyang Medical Co	27	2	153-154	2002	sn.1000-2707.2002.02.025
1259	岸沙门菌引起食物中毒	poisoning caused by Salmo	孙凤琪,计国欣.	Sun Fengqi,Ji Guoxin.	中华流行病学杂志	inese Journal of Epidemiol	23	6	472	2002	sn:0254-6450.2002.06.035
1260	鼠伤寒沙门菌病原学特	acteristics of Salmonella ty	谢一俊,陈亢川,林成水.	n,Chen Kangchuan,Lin Che	海峡预防医学杂志	s Journal of Preventive Me	8	3	1-4	2002	sn.1007-2705.2002.03.001
1261	出产超光谱β-内酰胺酶的	m bone marrow fluidβ-La	王叶芳.	Wang Yefang.	实用医技杂志	l of Practical Medical Tech	9	6	475-475	2002	sn.1671-5098.2002.06.076
1262	沙门菌噬菌体分型与耐药	resistance monitoring of Sa	施平,庄菱.	Shi Ping,Zhuang Ling.	中国公共卫生	Chinese public health	18	7	843-843	2002	sn:1001-0580.2002.07.035
1263	门氏菌腹泻暴发的病原学	on results of an outbreak o	家豪,许永炎,王炎业,陈	Xu Yongyan,Wang Yanye,S	中国热带医学	Chinese tropical medicine	2	4	550-550	2002	sn.1009-9727.2002.04.058
1264	寒、副伤寒沙门氏菌的	analysis of Salmonella typh	鏖明霞.	Yan Mingxia.	中国医学理论与实践	ese Medical Theory and Pr	-	7	-	2002	-
1265	副伤寒、伤寒菌株的药	ity analysis of paratyphoid	男,张云,王玉春,李林,文	ng Yun,Wang Yuchun,Li Li	华夏医学	Chinese Medicine	15	3	288-290	2002	sn.1008-2409.2002.03.004
1266	型副伤寒暴发流行的调	ort on an outbreak of para	车昆屏,孔祥斌.	Che Kunping,Kong Xiangbin	疾病监测	disease surveillance	17	2	73	2002	sn.1003-9961.2002.02.026
1267	鼠伤寒沙门氏菌感染的药	es in Salmonella typhimuri	李丽娟,谢俐萍,晏明佑.	ijuan,Xie Liping,Yan Mingy	内蒙古医学杂志	ner Mongolia Medical Jour	34	2	128-129	2002	sn.1004-0951.2002.02.015
1268	雷登沙门菌引起食物中	a case of food poisoning c	张国江,罗兰妹,蔡苏薇.	Guojiang,Luo Lanmei,Cai	中国卫生检验杂志	ese Journal of Health Inspe	12	4	495-495	2002	sn.1004-8685.2002.04.078
1269	炎沙门菌引起的食物中	of food poisoning caused	桂香,谭伟芬,陈卫民,周云	,Tan Weifen,Chen Weimin	现代预防医学	modern preventive medicin	29	4	585-585,589	2002	sn.1003-8507.2002.04.072
1270	业人员沙门氏菌携情况调	nella carriage among work	邓丽娟,张卉.	Deng Lijuan,Zhang Hui.	职业与健康	Occupation and health	18	7	45-46	2002	-
1271	市213株伤寒沙门菌耐药	f drug resistance spectrum	胡琳.	Hu Lin.	预防医学文献信息	ive medicine literature info	8	1	89-90	2002	sn.1672-9153.2002.01.089
1272	寒沙门菌感染121例临床	imurium infection121Case	李波,冯慕仁.	Li Bo,Feng Muren.	中国煤炭工业医学杂志	Journal of Coal Industry M	5	7	694-695	2002	sn.1007-9564.2002.07.048
1273	急性腹泻患者致病菌调	nic bacteria in patients wit	邢素琴,金惠艳,吴剑波.	g Suqin,Jin Huiyan,Wu Jian	内蒙古医学杂志	ner Mongolia Medical Jour	34	5	429-430	2002	sn.1004-0951.2002.05.026
1274	应性关节炎误诊20例分	hritis misdiagnosis20Exam	亚丽,贾振和,王霄扬,薛芙	Jia Zhenhe,Wang Xiaoyang	中国误诊学杂志	inese Journal of Misdiagno	2	9	1384-1385	2002	sn.1009-6647.2002.09.092
1275	苏州河水中沙门菌调查	n Salmonella in Suzhou Riv	昕,刘忠明,张磊,祁雄,黄宗	ngming,Zhang Lei,Qi Xiong	中国卫生检验杂志	ese Journal of Health Inspe	12	2	206-207	2002	sn.1004-8685.2002.02.047
1276	沙门菌致臀部脓肿及其药	almonella Typhimurium ar	贾川.	Jia Chuan.	上海医学检验杂志	ournal of Medical Laborato	17	2	89-89	2002	sn.1673-8640.2002.02.034
1277	巴布亚纳沙门菌引起的食	poisoning caused by Salmo	李华英,韩秀琴,王悦友.	aying,Han Xiuqin,Wang Yu	中华检验医学杂志	e Journal of Laboratory Me	25	6	344	2002	sn:1009-9158.2002.06.018
1278	导致79人食物中毒的调查	ation and analysis of huma	曾庆平,谭丹雪.	Zeng Qingping,Tan Danxue	预防医学情报杂志	of Preventive Medicine Inf	18	5	478-478	2002	sn.1006-4028.2002.05.060
1279	沙门菌引起的食物中毒	se of food poisoning cause	巩涛,吴卫东,赵心泉.	Tao,Wu Weidong,Zhao Xin	预防医学文献信息	ive medicine literature info	8	4	456-457	2002	sn.1672-9153.2002.04.058
1280	型副伤寒沙门菌引起的食	ning caused by Salmonella	雷鸣,吴文岸,皮治文.	der,Wu Wen'an,George Pi	预防医学情报杂志	of Preventive Medicine Inf	18	3	210-210	2002	sn.1006-4028.2002.03.071
1281	包积液中分离出1株肠炎	ericardial effusion1Salmo	高东佳,梁玉兰,李子文.	Dongjia,Liang Yulan,Li Ziw	预防医学文献信息	ive medicine literature info	8	3	320-320	2002	sn.1672-9153.2002.03.046
1282	生和重新出现的一些细菌	nd re-emerging bacterial p	李增光,张侃吉,李洪芳.	gguang,Zhang Kanji,Li Hon	中国禽业导刊	China Poultry Industry Guid	-	6	6-9	2002	-
1283	140例沙门氏菌食物中毒	xperience of patients with	林岭,官红梅.	Linling,Guan Hongmei.	西部医学	western medicine	14	2	26-27	2002	sn.1672-3511.2002.02.012
1284	沙门氏菌食物中毒调查	ort on a case of salmonell	钟逶迤,邱德星.	Zhong Yiyuan,Qiu Dexing.	实用预防医学	ractical preventive medicin	9	5	575-575	2002	sn.1006-3110.2002.05.096
1285	门氏菌食物中毒事件的调	alysis of a salmonella food	陈卫东,刘先明,方向明.	eidong,Liu Xianming,Clear	公共卫生与预防医学	Health and Preventive Me	13	6	45	2002	sn.1006-2483.2002.06.035
1286	94～2001细菌性食物中毒	01Dynamic analysis of bact	谢红英.	Xie Hongying.	职业与健康	Occupation and health	18	10	61-62	2002	sn.1004-1257.2002.10.053
1287	150例副伤寒甲临床分析	ical Analysis of Paratyphoi	段玲,章银娣.	Duan Ling,Zhang Yindi.	大理学院学报（医学版）	of Dali University (Medical	11	2	67-68	2002	-
1288	林市甲型副伤寒菌药物敏	g susceptibility test results	邓曼玲,郭炳才,张振开.	anling,Guo Bingcai,Zhang	广西预防医学	uangxi Preventive Medicin	8	3	164-166	2002	n.1673-758X.2002.03.015
1289	沙门菌引起食物中毒的	food poisoning caused by	陆幸儿.	Lu Xinger.	中国卫生检验杂志	ese Journal of Health Inspe	12	1	121-121	2002	sn.1004-8685.2002.01.081
1290	床分离菌的种类分布及耐	n and drug resistance analy	唐孝亮,刘筱玲,雷兰芳.	iaoliang,Liu Xiaoling,Lei La	中国现代医学杂志	ese Journal of Modern Me	12	13	96-97	2002	sn.1005-8982.2002.13.045
1291	市618例腹泻病原菌分布与	drug susceptibility of path	丽,方川,吴艳萍,徐百成,刘	huan,Wu Yanping,Xu Baich	河北职工医学院学报	of Hebei Workers' Medical	19	2	6-8	2002	n.1674-490X.2002.02.003
1292	某部生猪肉沙门菌污染情	ation of raw pork in a certa	庭海,徐英杰,张健,韩兴	i,Xu Yingjie,Zhang Jian,Han	预防医学文献信息	ive medicine literature info	8	2	228-228	2002	sn.1672-9153.2002.02.092
1293	1～2000年儿童败血症病	of changes and drug resist	徐亚萍,邵洁.	Xu Yaping,Shao Jie.	中华医学杂志	Chinese Medical Journal	82	14	945-948	2002	sn:0376-2491.2002.14.004

1294	甲型副伤寒62例临床分析	yphoid A62Case clinical an	郑惠珠.	Zheng Huizhu.	中国基层医药	China primary medicine	9	9	830	2002	sn.1008-6706.2002.09.037
1295	伤寒沙门氏菌致菌血症 1	d by Salmonella paratyphi	磨现斌,陈全根,黄翠柳.	nbin,Chen Quangen,Huang	广西预防医学	uangxi Preventive Medicin	8	1	37	2002	n.1673-758X.2002.01.032
1296	利丹沙门氏菌食物中毒	a case of Salmonella bulid	骆小君,朱淑英.	Luo Xiaojun,Zhu Shuying.	浙江预防医学	hejiang Preventive Medicin	14	4	34-34	2002	sn.1007-0931.2002.04.022
1297	省食品中致病菌污染现状	pathogenic bacteria conta	晓敏,马弋,李宇红,谢茂	omin,Ma Yi,Li Yuhong,Xie	中国食品卫生杂志	hinese Food Hygiene Journ	14	6	24-26	2002	sn.1004-8456.2002.06.009
1298	腹泻688例病原菌及耐药	sis of pathogenic bacteria	杨卫军.	Yang Weijun.	陕西医学杂志	Shaanxi Medical Journal	31	9	809-810	2002	sn.1000-7377.2002.09.022
1299	林沙门氏菌引起食物中	se of food poisoning cause	王永勤,刘新芳,徐美芹.	Yongqin,Liu Xinfang,Xu M	职业与健康	Occupation and health	18	5	58-58	2002	sn.1004-1257.2002.05.056
1300	鸭沙门氏菌引起的食物中	se of food poisoning cause	戴俊,文亚林,张晓英.	Jun,Wen Yalin,Zhang Xiaoy	实用预防医学	ractical preventive medicin	9	3	241-241	2002	sn.1006-3110.2002.03.028
1301	省5类食品中沙门菌污染的	ation of Salmonella contar	戴建华,陈华.	Dai Jianhua,Chen Hua.	江苏预防医学	iangsu Preventive Medicin	13	3	32-33	2002	sn.1006-9070.2002.03.019
1302	常见蝇类携带病原菌情	c bacteria carried by comm	陈秀锦,袁高林,叶恩禧.	en Xiujin,Yuan Gaolin,Ye Er	现代预防医学	nodern preventive medicin	29	5	639-640	2002	sn.1003-8507.2002.05.019
1303	门菌感染649例临床流行	hildren649Clinical epidemi	冯慕仁,李波.	Feng Muren,Li Bo.	中国当代儿科杂志	Journal of Contemporary P	4	6	519-520,522	2002	sn.1008-8830.2002.06.034
1304	儿甲型副伤寒26例临床分	d A in children26Case clini	杨莲芳,吕凤菊.	Yang Lianfang,Lu Fengju.	浙江临床医学	Zhejiang Clinical Medicine	4	9	697	2002	sn.1008-7664.2002.09.047
1305	肠炎沙门菌引起的食物中	of food poisoning caused	白宏伶.	Bai Hongling.	预防医学文献信息	ive medicine literature info	8	1	59-59	2002	sn.1672-9153.2002.01.052
1306	操作人员带沙门氏菌引起	aused by salmonella introd	朱玉静.	Zhu Yujing.	上海预防医学	hanghai Preventive Medicin	14	3	126-127	2002	sn.1004-9231.2002.03.012
1307	寒沙门氏菌食物中毒的	e of food poisoning caused	玉裕,陈兴乐,陈杰,黄林,郭	hen Xingle,Chen Jie,Huang	中国卫生监督杂志	se Journal of Health Super	9	6	342-343	2002	sn.1077-6131.2002.06.008
1308	宴引起的肠炎沙门氏菌食	s food poisoning caused by	翁良,焦振山.	Weng Liang,Jiao Zhenshan.	中国城乡企业卫生	ygiene in urban and rural a	-	3	45-46	2002	sn.1003-5052.2002.03.030
1309	000年食品污染状况监测	food contamination monit	茂起,王竹天,包大跃,冉	oqi,Wang Zhutian,Bao Day	中国食品卫生杂志	hinese Food Hygiene Journ	14	2	3-8	2002	sn.1004-8456.2002.02.001
1310	生肉携带沙门氏菌的调查	s of Salmonella in commer	杨洪波,张宇琴,曹家清.	Hongbo,Zhang Yuqin,Cao Ji	职业卫生与应急救援	ional health and emergenc	20	3	133-133	2002	sn.1007-1326.2002.03.010
1311	村婚宴感染性腹泻病爆	eak of infectious diarrhea a	车代铭,刘刚,宋红.	Daiming,Liu Gang,Song Ho	职业与健康	Occupation and health	18	8	58-58	2002	sn.1004-1257.2002.08.054
1312	物性饲料中沙门氏菌的分	fication of Salmonella in im	陈沁,李健,杨英.	Chen Qin,Li Jian,Yang Ying.	中国动物检疫	China Animal Quarantine	19	1	24-26	2002	n.1005-944X.2002.01.021
1313	症患者致病菌及其耐药	nd their drug resistance ch	余雪涛,方伟强.	Yu Xuetao,Fang Weiqiang.	中国微生态学杂志	inese Journal of Microecol	14	4	227-228	2002	n.1005-376X.2002.04.019
1314	混合型细菌性食物中毒	t of mixed bacterial food p	林鹭芳,黄健利,钟凌.	Lufang,Huang Jianli,Zhong L	海峡预防医学杂志	s Journal of Preventive Me	8	2	10-10	2002	sn.1007-2705.2002.02.061
1315	例医院感染致病菌耐药率	ance rates of pathogenic ba	侯爱红.	Hou Aihong.	海峡药学	Straits Pharmacy	14	6	88-89	2002	sn.1006-3765.2002.06.052
1316	农村婚宴引起食物中毒的	oisoning caused by a rural	张妙珍.	Zhang Miaozhen.	浙江预防医学	hejiang Preventive Medicin	14	8	42-42	2002	sn.1007-0931.2002.08.027
1317	鼠伤寒沙门菌噬菌体135	Salmonella typhimurium ph	周祖木.	Zhou Zumu.	学（流行病学.传染病学	(Epidemiology.Infectious	-	6	380	2002	-
1318	00-2001年度食源性致病菌	1Annual Foodborne Patho	升清,马群飞,陈伟伟,杨毓	g,Ma Qunfei,Chen Weiwei,	海峡预防医学杂志	s Journal of Preventive Me	8	6	66-67	2002	sn.1007-2705.2002.06.041
1319	鼠伤寒沙门菌医院感染调	ocomial infection of neona	陈建蕊,谢多希.	Chen Jianrui,Xie Duoxi.	中华医院感染学杂志	ournal of Hospital Infectiou	12	1	51-51	2002	sn:1005-4529.2002.01.025
1320	阿哥纳沙门菌引起的食物	d poisoning caused by Salm	崔萍.	Cui Ping.	中国学校卫生	Chinese school health	23	4	383-384	2002	sn.1000-9817.2002.04.060
1321	沙门氏菌感染暴发流行的	into an outbreak of salmon	华,范学杰,王冬梅,李群,	uejie,Wang Dongmei,Li Qu	解放军预防医学杂志	tive Medicine of the People	20	3	224-224	2002	sn.1001-5248.2002.03.031
1322	型副伤寒沙门菌169株分	ella Paratyphi A169strain a	齐友萍.	Qi Youping.	世界今日医学杂志	world medical journal today	3	4	348-349	2002	-
1323	8-2000年沙门菌属耐药性	nges in drug resistance of S	波,李景云,姚蕾,张力,胡昌	Jingyun,Yao Lei,tension,Hu	中国抗感染化疗杂志	urnal of Anti-Infectious Che	2	2	84-86	2002	sn:1009-7708.2002.02.007
1324	沙门氏菌食物中毒调查	nalysis of a case of salmon	兵,谢坤清,林立新,陈小	g,Xie Kunqing,Lin Lixin,Che	华南预防医学	uth China Preventive Medic	28	5	52-52	2002	sn.1671-5039.2002.05.023
1325	感染鼠伤寒沙门菌耐药性	of Salmonella typhimuriu	楚世尊,何子毅,孙俊.	Chu Shizun,He Ziyi,Sun Jun	中国抗感染化疗杂志	urnal of Anti-Infectious Che	2	4	234-235	2002	sn:1009-7708.2002.04.014
1326	儿甲型副伤寒26例临床分	d A in children26Case clini	杨莲芳.	Yang Lianfang.	临床误诊误治	l misdiagnosis and mistrea	15	1	21-21	2002	sn.1002-3429.2002.01.013
1327	酶甲型副伤寒沙门菌的检	drug susceptibility test of S	陈峥,王晓彬.	Chen Zheng,Wang Xiaobin.	贵州医药	Guizhou Medicine	26	4	326-327	2002	n.1000-744X.2002.04.016
1328	中毒患者粪便中检出仙台	detected in feces of food	侯明英,齐延臻,齐涛.	u Mingying,Qi Yanzhen,Qi T	中华实用医学	Chinese Practical Medicine	4	14	96-96	2002	-
1329	康人群携带沙门氏菌、志	d Shigella carried by health	宁德清,房翠平,卞爱红.	Deqing,Fang Cuiping,Bian A	微生物学杂志	journal of microbiology	22	6	60-60,62	2002	sn.1005-7021.2002.06.021
1330	法检出健康人群肠道沙门	ods for detecting enteric Sa	贾迎辉.	Jia Yinghui.	洛阳农业高等专科学校学	l of Luoyang Agricultural C	22	3	184-184	2002	-

1331	2株沙门氏菌培养结果分	of culture results of Salmo	张绍银.	Zhang Shaoyin.	实用医技杂志	l of Practical Medical Tech	9	7	521-522	2002	sn.1671-5098.2002.07.038
1332	伤寒病房交叉感染11例报	n in typhoid fever ward11E	邓小田.	Deng Xiaotian.	青海医药杂志	Qinghai Medical Journal	32	10	31-32	2002	sn.1007-3795.2002.10.021
1333	镇海区2000年伤寒发病情	y2000Analysis of the annua	张亚芬.	Zhang Yafen.	浙江预防医学	hejiang Preventive Medicin	14	1	28-28	2002	sn.1007-0931.2002.01.017
1334	斯特沙门菌致泌尿感染一	ry infection caused by Salm	胡建军,周峰.	Hu Jianjun,Zhou Feng.	汾煤科技	Fenmei Technology	-	3	46	2002	-
1335	2000年夏秋季腹泻病原菌	ogenic bacteria spectrum	春花,郗园林,张梅喜,段广	a,Xi Garden,Zhang Meixi,D	郑州大学学报（医学版）	hengzhou University (Med	37	6	840-843	2002	sn.1671-6825.2002.06.043
1336	标本细菌培养菌株的分	bacterial culture strains in	黄建香,余崇镐,林伟珍.	anxiang,Yu Chonghao,Lin V	齐鲁医学检验	Qilu Medical Testing	13	3	26-27	2002	sn.1673-5013.2002.03.012
1337	染性腹泻病2 489例病因分	rheal disease2 489Cause ar	杨梦雨,张杰,常荣芬.	Mengyu,Zhang Jie,Chang Ro	陕西医学杂志	Shaanxi Medical Journal	31	3	197-198	2002	sn.1000-7377.2002.03.002
1338	48例细菌性食物中毒的	utbreak of bacterial food p	李桂.	Li Gui.	传染病信息	fectious disease informatio	-	3	139	2002	-
1339	汉污染引起的沙门菌食物	lmonella food poisoning ca	贺国强,庞爱君.	He Guoqiang,Pang Aijun.	预防医学文献信息	ive medicine literature info	8	4	451-451	2002	sn.1672-9153.2002.04.050
1340	沙门菌引起食物中毒的病	etiology of food poisoning	玉敏,高玉荣,孙素梅,赵	hin,Gao Yurong,Sun Sumei,	中国卫生检验杂志	ese Journal of Health Inspe	12	1	120-120	2002	sn.1004-8685.2002.01.080
1341	泻病人致病菌血清分群及	ensitivity test of pathogeni	丁绮容.	Ding Qirong.	安徽预防医学	Anhui Preventive Medicine	8	1	40	2002	-
1342	大陈镇一起伤寒暴发疫情	reak of typhoid fever in	,骆淑英,陶洪文,叶晓军,	ying,Tao Hongwen,Ye X	中国公共卫生管理	ese public health manage	-	6	523	2002	cnki.23-1318.2002.06.038
1343	2001年一起伤寒暴发的调	tion and analysis of the	提,尔西丁,艾尔肯,阿孜古	siddin,Elken,Aziguli,Jiang	疾病监测	disease surveillance	-	10	377-379	2002	-
1344	中学生发生伤寒暴发流	d fever among students	,陈金霞,万明忠,曹世明,	a,Wan Mingzhong,Cao S	疾病监测	disease surveillance	-	10	397	2002	-
1345	伤寒暴发的流行病学调	nd prevention of an outbr	力旦·尤努斯,苏文霞,阿斯	idan·Yunus,Su Wenxia,A	地方病通报	ndemic disease notificatio	-	3	99	2002	cnki.jbyfkztb.2002.03.056
1346	甲型副伤寒暴发20例分析	id A outbreak20Example	吴飞仙	Wu Feixian	小儿急救医学	diatric Emergency Medic	-	3	172	2002	-
1347	起伤寒暴发流行的调查报	report on an outbreak of	王良周	Wang Liangzhou	实用乡村医生杂志	nal of Practical Rural Do	-	3	48	2002	-
1348	一起乙型副伤寒暴发的调	nto an outbreak of paraty	佼,夏怀林,陈媛,李平,石	Huailin,Chen Yuan,Li Pin	实用预防医学	actical preventive medici	-	3	230	2002	-
1349	4起伤寒暴发流行特征分	mic characteristics of typ	梅华,覃章世,石金泉	ua,Qin Zhangshi,Shi Jin	疾病监测	disease surveillance	-	5	184-185	2002	-
1350	克乡伤寒暴发流行调查分	sis of typhoid fever outbr	尔,阿布都热希提,雷忠江,	,abdurehiti,Lei Zhongjian	疾病监测	disease surveillance	-	4	131-132	2002	-
1351	一起水源污染的伤寒暴	outbreak caused by wate	杨静淑,张云生,李继华	ngshu,Zhang Yunsheng,	疾病监测	disease surveillance	-	4	132-133	2002	-
1352	伤寒暴发的流行病学调查	tigation report on an out	彭文通,汤慧荣,罗利华	Ventong,Tang Huirong,Lu	疾病监测	disease surveillance	-	4	151-152	2002	-
1353	食物型伤寒暴发的流行病	ation of an outbreak of f	陈学勇,梁学福	Chen Xueyong,Liang Xue	解放军预防医学杂志	ve Medicine of the Peopl	-	2	146	2002	-
1354	径引起甲型副伤寒暴发流	reak of paratyphoid A ca	胡迎春,黄旦云,周元娣	chun,Huang Danyun,Zho	疾病监测	disease surveillance	-	2	52-53	2002	-
1355	市场食品被污染引起的	utbreak caused by conta	黄文国,咸兴敏,姜文国	enguo,Ham Xingmin,Jian	预防医学文献信息	ve medicine literature inf	-	1	43-108	2002	-
1356	监狱甲型副伤寒病原学的	e etiology of paratyphoid	张国江,罗兰妹,蔡苏薇.	Guojiang,Luo Lanmei,Cai S	中国卫生检验杂志	ese Journal of Health Inspe	13	3	318-318	2003	sn.1004-8685.2003.03.036
1357	沙门氏菌食物中毒的调查	ort on a case of salmonella	兰长金,何荣双.	an Changjin,He Rongshuan	现代预防医学	modern preventive medicin	30	6	836-836	2003	sn.1003-8507.2003.06.041
1358	道区主要猪病的监测调查	n report on major swine di	儒,王华,赵周全,班建全,蒲	g Hua,Zhao Zhouquan,Ban	甘肃畜牧兽医	al Husbandry and Veterina	33	1	18-20	2003	n.1006-799X.2003.01.008
1359	期大批死亡病例中的细	ng in early mass mortality	卢受昇,许楚杰.	Lu Shoucheng,Xu Chujie.	广东畜牧兽医科技	mal Husbandry and Veterin	28	6	26-29	2003	sn.1005-8567.2003.06.010
1360	粪便中病原体的检出与	analysis of pathogens in f	陆其兵,徐耀武.	Lu Qibing,Xu Yaowu.	上海医学检验杂志	ournal of Medical Laborato	18	6	369-371	2003	sn.1673-8640.2003.06.007
1361	腹泻患者中检出山夫登堡	rdenberg detected in a pati	郭勇峰,李惠芬,王洁.	Yongfeng,Li Huifen,Wang	中华流行病学杂志	inese Journal of Epidemiol	24	12	1111-1111	2003	sn:0254-6450.2003.12.031
1362	分离培养出肠杆菌科细菌	robacteriaceae isolated an	程烜,马小明,梁勤.	g Xuan,Ma Xiaoming,Liang	中国实验诊断学	nese experimental diagnos	7	4	350-351	2003	sn.1007-4287.2003.04.031
1363	时分离出都柏林沙门菌	lated from blood and cere	王家斌,余桂香.	Wang Jiabin,Yu Guixiang.	蚌埠医学院学报	rnal of Bengbu Medical Col	28	5	454-454	2003	sn.1000-2200.2003.05.041
1364	甲型副伤寒沙门氏菌耐	nce of Salmonella typhoid	水荣,张政,莫顺堂,陈秀	Zhang Zheng,Mo Shun Tang	疾病监测	disease surveillance	18	4	139-141	2003	sn.1003-9961.2003.04.012
1365	庭聚餐食物中毒事故的调	sis of a food poisoning acc	朱明福,杨惠玲.	Zhu Mingfu,Yang Huiling.	中国卫生监督杂志	se Journal of Health Super	10	2	79-80	2003	sn.1077-6131.2003.02.009
1366	992～2001年沙门菌菌型	01Annual distribution of S	贾云,范玉波.	Jia Yun,Fan Yubo.	职业与健康	Occupation and health	19	7	41-42	2003	sn.1004-1257.2003.07.032
1367	菌污染水源引起的肠道传	ctious diseases caused by	智,熊燕,龙一兵,朱焰,付	Xiong Yan,Long Yibing,Zhu	中国人兽共患病杂志	Chinese Journal of Zoonose	19	4	127-127,126	2003	sn.1002-2694.2003.04.045

1368	伤寒副伤寒暴发的调查分	ysis of outbreaks of typhoi	兴建,肖邦忠,邓学英,张建	opin Chung,Deng Xueying,	预防医学情报杂志	of Preventive Medicine Info	19	1	12-14	2003	sn.1006-4028.2003.01.005
1369	门氏菌引起食物中毒事故	food poisoning incident ca	黄德海,陈祈斌,彭岳松.	Dehai,Chen Qibin,Peng Yu	中国卫生监督杂志	ese Journal of Health Super	10	1	24-25	2003	sn.1077-6131.2003.01.012
1370	沙门氏菌属食物中毒调查	food poisoning caused by	赵耀林,史可梅.	Zhao Yaolin,Shi Kemei.	长治医学院学报	nal of Changzhi Medical Co	17	1	20-21	2003	sn.1006-0588.2003.01.009
1371	永兴岛水域海水细菌学	n of seawater in the water	周一平,赖福才.	Zhou Ping,Lai Fucai.	感染、炎症、修复	fection, inflammation, repa	4	2	106-108	2003	sn.1672-8521.2003.02.012
1372	小水产品中肠道致病菌污	estinal pathogenic bacteria	晶,王衡,姚怀芳,孙昼,汪皓	g Heng,Yao Huaifang,Sun Z	预防医学文献信息	ive medicine literature info	9	6	657-658	2003	sn.1672-9153.2003.06.012
1373	炎沙门菌食物中毒的检	a case of Salmonella Enter	莉,刘形,李秀彬,杨东霞,杨	u Xing,Li Xiubin,Yang Dong	中国卫生检验杂志	ese Journal of Health Inspe	13	6	779-779	2003	sn.1004-8685.2003.06.071
1374	沙门菌的菌型分布及对抗	antibiotic resistance of clir	胡四海,周祖岳,李吾雄.	Sihai,Zhou Zuyue,Li Wuxio	中国微生态学杂志	inese Journal of Microecolo	15	5	287-288	2003	n.1005-376X.2003.05.025
1375	西伤寒、副伤寒流行特征	revention and control stra	柏青,曾竣,王鸣柳,李翠云	ing,Zeng Jun,Wang Mingliu	华南预防医学	th China Preventive Medic	29	4	10-12	2003	sn.1671-5039.2003.04.004
1376	感染性腹泻病原菌及耐药	cteria and drug resistance i	杨智哲,杨卫军.	Yang Zhizhe,Yang Weijun.	中华现代临床医药杂志	Journal of Modern Clinical	4	8	12-14	2003	-
1377	氏菌引起婚宴食物中毒的	of food poisoning at a wed	谢峰,纪红.	Xie Feng,Ji Hong.	中国卫生监督杂志	ese Journal of Health Super	10	2	70-71	2003	sn.1077-6131.2003.02.004
1378	肠炎沙门菌引起的食物	oisoning caused by Salmo	赵鸣镝,张旭.	Zhao Mingdi,Zhang Xu.	预防医学文献信息	ive medicine literature info	9	6	709-709	2003	sn.1672-9153.2003.06.052
1379	寒沙门氏菌188株培养药敏	8Analysis of strain culture	张淑琼,杨映平,李琼芬,马	huqiong,Yang Yingping,Li	中华现代临床医药杂志	Journal of Modern Clinical	4	3	35-36	2003	-
1380	成都市青羊区从业人员健	cteria carried by employee	孟琳.	Meng Lin.	预防医学情报杂志	of Preventive Medicine Info	19	6	549-550	2003	sn.1006-4028.2003.06.036
1381	门氏菌食物中毒事故的调	ndling of a salmonella foo	朱小燕,黄杰.	Zhu Xiaoyan,Huang Jie.	中华卫生监督与健康	se Health Supervision and	2	6	61-62	2003	-
1382	伤寒沙门菌对抗菌药物的	tance of Salmonella typhi s	柱,董柏青,谭冬梅,权怡,杨	ong Baiqing,Tan Dongmei,	中国预防医学杂志	se Journal of Preventive Me	4	4	289-291	2003	sn.1009-6639.2003.04.022
1383	贤县两起卤菜食物中毒的	ases of braised vegetable f	余友德,李小斌.	Yu Youde,Li Xiaobin.	中华实用医药杂志	ese Journal of Practical Me	3	24	2313-2314	2003	-
1384	茨坦沙门菌引起的食物	od poisoning case caused b	李秀兰,孙凤琪.	Li Xiulan,Sun Fengqi.	中华流行病学杂志	inese Journal of Epidemiolo	24	12	1085-1085	2003	sn:0254-6450.2003.12.028
1385	港沙门菌食物中毒的实验	a case of Salmonella Newp	郑历,张淑滨,刘尊玉.	ng Li,Zhang Shubin,Liu Zun	预防医学文献信息	ive medicine literature info	9	5	592-592	2003	sn.1672-9153.2003.05.064
1386	康人群沙门菌属和志贺菌	a carriage among healthy p	潘江,陈悦,童锐,沈健民.	g,Chen Yue,Tong Rui,Shen	上海预防医学	hanghai Preventive Medicin	15	9	457-458	2003	sn.1004-9231.2003.09.015
1387	)排放污水卫生细菌和沙门	estigation of sanitation bac	魏丽红.	Wei Lihong.	肉品卫生	Meat hygiene	-	3	9-10	2003	-
1388	床分离菌的菌群分布和耐	d drug resistance of clinical	繁林,储从家,管新龙,罗次	u Congjia,Guan Xinlong,Ra	玉溪师范学院学报	rnal of Yuxi Normal Univer	19	z1	76-79	2003	sn.1009-9506.2003.z1.020
1389	合并甲型副伤寒沙门氏	E complicated with Salmo	彭湘明.	Peng Xiangming.	广西医学	Guangxi Medicine	25	9	1749-1749	2003	sn.0253-4304.2003.09.086
1390	门氏菌早期感染45例的	um in children45Analysis o	萍,汪晓炜,成胜权,许东亮	ang Xiaowei,Right to win,	中国儿童保健杂志	ese Journal of Child Health	11	1	69-69	2003	sn.1008-6579.2003.01.041
1391	泻病403例病原学检测与	403Etiology detection and	杨丽芳,陈亚莉,李月华,焦	g Lifang,Chen Yali,Li Yuehu	实用儿科临床杂志	ctical Pediatric Clinical Jou	18	7	528-529	2003	n.1003-515X.2003.07.014
1392	伤寒沙门氏菌肠炎24例分	murium Enteritistwenty fou	志伟,陈益平,卢朝升,汪洪	en Yiping,Lu Chaosheng,W	浙江临床医学	Zhejiang Clinical Medicine	5	11	827-827	2003	sn.1008-7664.2003.11.017
1393	都柏林沙门氏菌食物中毒	of Salmonella Dublin foo	凤兰,王安姝,崔代华,白敏	lan,Wang Anshu,Cui Daihu	贵州医药	Guizhou Medicine	27	1	91-93	2003	n.1000-744X.2003.01.051
1394	型副伤寒沙门菌鉴定及药	susceptibility results of Sal	俞连琴,骆勇.	Yu Lianqin,Luo Yong.	中华医院感染学杂志	ournal of Hospital Infectiou	13	1	6-6	2003	sn:1005-4529.2003.01.042
1395	一起感染性腹泻暴发调查	n into an infectious diarrhe	丕建,邓化君,曾子同,金则	Deng Huajun,Zeng Zitong,	实用预防医学	ractical preventive medicin	10	4	584-584	2003	sn.1006-3110.2003.04.078
1396	本中分离出都柏林沙门菌	of Salmonella Dublin from	张建敏,任秀云.	Zhang Jianmin,Ren Xiuyun.	江西医学检验	Jiangxi Medical Laboratory	21	3	220	2003	sn.1674-1129.2003.03.054
1397	自动培养临床分离菌细菌	of clinical isolates and bac	繁林,储从家,罗次节,管新	u Congjia,Rattsuga Festival	中国微生态学杂志	inese Journal of Microecolo	15	5	295-296	2003	n.1005-376X.2003.05.029
1398	01年广西感染性腹泻流行	logical analysis of infectiou	翠云,唐振柱,龚健,王鸣柳	ng Zhenzhu,Gong Jian,War	广西预防医学	uangxi Preventive Medicin	9	1	18-21	2003	n.1673-758X.2003.01.006
1399	氏菌致骨髓炎1例并文献	myelitis1Review of examp	荣,梁雨田,董纪元,缪力,崔	ang Yutian,Dong Jiyuan,Mi	中国医学影像学杂志	ese Journal of Medical Ima	11	2	152-153	2003	sn.1005-5185.2003.02.031
1400	门氏菌感染引起硬膜下积	nfection causing subdural	张文旻,柳宏波.	Zhang Wenmin,Liu Hongbo	中国社区医师（医学专业）	munity physician (medical	5	6	46	2003	-
1401	性致病菌监测及其耐药性	c bacteria in Jiangsu and a	戚建华,袁宝君,陈太基,乔明	ua,Yuan Baojun,Chen Taiji,	中国卫生检验杂志	ese Journal of Health Inspe	13	3	330-331	2003	sn.1004-8685.2003.03.046
1402	一起家宴引起的食物中毒	oisoning caused by a family	热合木.	thermolaminated wood.	中国自然医学杂志	ese Journal of Natural Med	5	3	封底	2003	-
1403	柏林沙门氏菌败血症4例报	monella Septicemia4Exam	苏桂同,宋玉兰,刘金侠.	Guitong,Song Yulan,Liu Jin	承德医学院学报	nal of Chengde Medical Co	20	1	75-76	2003	sn.1004-6879.2003.01.046
1404	寒、甲型副伤寒沙门菌耐	istance of Salmonella typhi	水荣,张政,莫顺堂,陈秀英	hang Zheng,Mo Shun Tang	浙江预防医学	hejiang Preventive Medicin	15	11	5-6,9	2003	sn.1007-0931.2003.11.003

1405	院内鼠伤寒沙门菌交叉感染	ocomial cross-infection of S	魏曰兴,张树锡.	Wei Yuexing,Zhang Shuxi.	中国乡村医药	Chinese rural medicine	10	4	54	2003	sn.1006-5180.2003.04.066
1406	肠炎沙门氏菌食物中毒的	a case of Salmonella Enterit	廖达升.	Liao Dasheng.	广西预防医学	Guangxi Preventive Medicin	-	S1	41-42	2003	-
1407	感染性腹泻病原及流行病学	gy and epidemiology of ac	任风珠,顾淑玲.	Ren Fengzhu,Gu Shuling.	中华卫生监督与健康	se Health Supervision and H	2	6	88-89	2003	-
1408	猪分割肉沙门氏菌的菌型	cterial types of Salmonella i	黄晔.	Huang Ye.	肉品卫生	Meat hygiene	-	3	16-18	2003	-
1409	一起沙门菌食物中毒的调查	into a case of salmonella fo	王新村,李建芳.	Wang Xincun,Li Jianfang.	职业与健康	Occupation and health	19	5	50-50	2003	sn.1004-1257.2003.05.043
1410	菌食物中毒致心律失常33例	hing causes cardiac arrhyth	孙中波,覃江,伍广伟,张琴	bo,Qin Jiang,Wu Guangwei,	中国实用内科杂志	ournal of Practical Internal	23	12	707	2003	sn.1005-2194.2003.12.034
1411	门菌污染真空软包装扒鸡引	onella bulidan contaminate	夏长勇,夏淑青,黄玉新.	angyong,Xia Shuqing,Huang	预防医学文献信息	ive medicine literature info	9	2	189-189	2003	sn.1672-9153.2003.02.052
1412	沙门菌引起食物中毒暴发的	outbreak of food poisoning	凌,赵君,周媛媛,马明娜,胡小	Jun,Zhou Yuanyuan,Ma Mi	第三军医大学学报	the Third Military Medical	25	22	2014-2014	2003	sn:1000-5404.2003.22.037
1413	胰脓液中检出1株甲型副伤寒	ncreatic cyst pus1Salmone	褚从家,潘丽,吴惠玲,孔繁林	gjia,Pan Li,Wu Huiling,Kon	临床检验杂志	journal of clinical testing	21	6	362-362	2003	n.1001-764X.2003.06.041
1414	从业人员带菌引起的食物	oning caused by bacteria ca	董纪明,支亚渊.	Dong Jiming,Zhi Yayuan.	中华卫生监督与健康	se Health Supervision and H	2	1	36	2003	-
1415	液中分离出一株山夫顿堡沙	ain of Salmonella Shanfton	张广玲,王开升,孙连芹.	angling,Wang Kaisheng,Su	中华微生物学和免疫学杂志	rnal of Microbiology and Im	23	6	435	2003	sn:0254-5101.2003.06.011
1416	康人群沙门菌带菌及菌型分	carriers and bacterial type	姜钧惠.	Jiang Junhui.	疾病监测	disease surveillance	18	10	376-377	2003	sn.1003-9961.2003.10.009
1417	寒沙门氏菌所致食物中毒的	od poisoning caused by Salr	张中昌,聂鸿雁.	ang Zhongchang,Nie Hongy	宜春学院学报	Journal of Yichun University	25	4	103-103	2003	n.1671-380X.2003.04.046
1418	州市蛇类沙门氏菌带菌调	Salmonella carriers in snak	肖杨,刘建平,梁立文.	Yang,Liu Jianping,Liang Liv	中国人兽共患病杂志	Chinese Journal of Zoonose	19	6	115-116	2003	sn.1002-2694.2003.06.037
1419	阳市崇安区从业人员肠道沙	estinal testing results amo	李春毅,葛丽燕,何迎峰.	Chunyi,Ge Liyan,He Yingfer	中国卫生检验杂志	ese Journal of Health Inspe	13	6	740-740	2003	sn.1004-8685.2003.06.038
1420	门氏菌引起食物中毒34例调	is causes food poisoning34	陈诗安,刘云金.	Chen Shi'an,Liu Yunjin.	中华医学实践杂志	ese Journal of Medical Pra	2	4	372-373	2003	-
1421	沙门氏菌病和巴氏杆菌病	avian salmonellosis and pa	春,黄志秋,刘勇,张明国,王	Zhiqiu,Liu Yong,Zhang Ming	西昌农业高等专科学校学报	al of Xichang Agricultural C	-	3	3-4	2003	-
1422	引起山夫顿堡沙门氏菌感染	a infection from Fort Shan	石观平,陈永金,梅建华.	anping,Chen Yongjin,Mei Ji	中国人兽共患病杂志	Chinese Journal of Zoonose	19	6	112-112	2003	sn.1002-2694.2003.06.041
1423	地区食源性疾病致病菌污染	tus of foodborne disease p	蒋震羚,吕素玲,王红,车光	ling,Lu Suling,Wang Hong,c	广西预防医学	Guangxi Preventive Medicin	9	4	222-224	2003	n.1673-758X.2003.04.009
1424	腔积液中分离出肠炎沙门	ted from hepatogenic pleur	吴立奇,王春萍,郑新世.	qi,Wang Chunping,Zheng X	浙江预防医学	Zhejiang Preventive Medicin	15	8	65-65	2003	sn.1007-0931.2003.08.045
1425	由沙门菌引起食物中毒调查	n a case of food poisoning	海燕,严纪文,黄吉城,宋曼	Yan Jiwen,Huang Jicheng,S	华南预防医学	uth China Preventive Medic	29	5	52-52	2003	sn.1671-5039.2003.05.022
1426	株甲型副伤寒沙门氏菌耐药	nce of Salmonella paratyph	狄云湘.	Di Yunxiang.	河北医药	Hebei Medicine	25	6	474	2003	sn.1002-7386.2003.06.034
1427	沙门杆菌性肠炎院内感染	osocomial infection of Salm	于美兰.	Yu Meilan.	华北煤炭医学院学报	of North China Coal Medica	5	5	595-596	2003	sn.1008-6633.2003.05.039
1428	甲型副伤寒沙门菌药物敏感	bility testing of Salmonella p	周敏华,金法祥,李水法.	u Minhua,Jin Faxiang,Li Shu	预防医学文献信息	ive medicine literature info	9	4	457-457	2003	sn.1672-9153.2003.04.067
1429	鼠伤寒沙门菌肠炎40例	lla Typhimurium Enteritis40	郑之卿,孙明华.	Zheng Zhiqing,Sun Minghua	实用儿科临床杂志	ctical Pediatric Clinical Jour	18	9	751-751	2003	n.1003-515X.2003.09.056
1430	1株伤寒沙门菌药敏试验结	rug susceptibility test resul	昔,刘和录,徐志康,华建江,董	Helu,Xu Zhikang,Hua Jianjia	中华检验医学杂志	se Journal of Laboratory Me	26	4	206-206	2003	sn:1009-9158.2003.04.022
1431	儿猪霍乱沙门菌感染15例	ection in infants and young	陶云珍,诸丽娟,丁云芳.	unzhen,Zhu Lijuan,Ding Yu	苏州大学学报（医学版）	Soochow University (Medic	23	2	248-249	2003	sn.1673-0399.2003.02.051
1432	蛋引起的食物中毒调查及检	isoning caused by salted du	卢翠珍,王国志.	Lu Cuizhen,Wang Guozhi.	中华卫生监督与健康	se Health Supervision and H	2	3	45	2003	-
1433	海吉威玛沙门氏菌引起的食	poisoning caused by Salmon	杨桂凤.	Yang Guifeng.	中国现代医药科技	ese modern medical techno	3	5	83	2003	-
1434	市从业人员肠道带菌调查	of intestinal bacteria amo	杨晓玲,杨道遇.	Yang Xiaoling,Yang Daoyu.	现代预防医学	modern preventive medicin	30	4	527-528	2003	sn.1003-8507.2003.04.028
1435	染引起急性细菌性肠炎暴发	k of acute bacterial enteriti	张秀霞.	Zhang Xiuxia.	中华卫生监督与健康	se Health Supervision and H	2	6	95-96	2003	-
1436	人员沙门菌带菌菌型分布及	tion and drug susceptibility	李毅,章乐怡.	Li Yi,Zhang Leyi.	蚌埠医学院学报	rnal of Bengbu Medical Col	28	4	362-363	2003	sn.1000-2200.2003.04.044
1437	海市普陀区肉制品中的致	bacteria in meat products	张磊,陈昕.	Zhang Lei,Chen Xin.	中国卫生检验杂志	ese Journal of Health Inspe	13	3	319-319	2003	sn.1004-8685.2003.03.037
1438	型副伤寒沙门菌引起的食物	of food poisoning caused b	张仁柱,张维栋.	ang Renzhu,Zhang Weidon	预防医学文献信息	ive medicine literature info	9	4	449-449	2003	sn.1672-9153.2003.04.054
1439	873株鼠伤寒菌药敏分析	eptibility analysis of Typhin	吴孝田.	Wu Xiaotian.	中国误诊学杂志	inese Journal of Misdiagno	3	3	424-425	2003	sn.1009-6647.2003.03.085
1440	斯坦利沙门氏菌引起肠炎1	lla Stanley causes enteritis1	李桂梅,黄宏章.	i Guimei,Huang Hongzhang	中华临床医药杂志	ese Journal of Clinical Med	4	21	24-24	2003	-
1441	业人员携带的沙门菌血清学	ting of Salmonella carried	熊国强,杨红,应艳,余希.	uoqiang,Yang Hong,Ying Ya	中国卫生检验杂志	ese Journal of Health Inspe	13	4	414-415	2003	sn.1004-8685.2003.04.006

1442	～2002年食品中沙门氏菌	~2002Monitoring and anal	升清,马群飞,杨毓环,陈建	ing,Ma Qunfei,Yang Yuhua	中国食品卫生杂志	hinese Food Hygiene Journ	15	5	406-409	2003	sn.1004-8456.2003.05.005
1443	省272份食品沙门氏菌检测	ovince272Food Salmonella	童哲,程苏云,梅玲玲.	Zhe,Cheng Suyun,Mei Ling	浙江预防医学	hejiang Preventive Medicin	15	4	33-34	2003	sn.1007-0931.2003.04.023
1444	97～2001年186株沙门菌的	1Year186Type identificatio	徐铁民.	Xu Tiemin.	职业与健康	Occupation and health	19	1	38-39	2003	sn.1004-1257.2003.01.027
1445	夫地区奶牛隐性乳房炎的	tent mastitis in dairy cows	魏学良,张家骅,袁登秀.	eliang,Zhang Jiahua,Yuan D	黄牛杂志	scalper magazine	29	6	16-19,23	2003	sn.1001-9111.2003.06.006
1446	儿鼠伤寒沙门菌感染26例	urium infection in children	余吉仙,岑立冲.	Yu Jixian,Cen Lichong.	浙江预防医学	hejiang Preventive Medicin	15	7	47-48	2003	sn.1007-0931.2003.07.033
1447	炎沙门菌引起的食物中毒	ase of Food Poisoning Cau	李少彤,孙凤琪,曾德荣.	otong,Sun Fengqi,Tsang De	中国医学检验杂志	urnal of Medical Laborator	4	3	204-205	2003	-
1448	一起鼠伤寒沙门菌食物中	almonella typhimurium foo	陈敏,彭继旭.	Chen Min,Peng Jixu.	实用预防医学	ractical preventive medicin	10	6	1034-1034	2003	sn.1006-3110.2003.06.081
1449	菌感染致新生儿肾上腺广	renal hemorrhage caused b	韦,赖剑蒲,赵宁,黄晓虹,吕	ianpu,Zhao Ning,Huang Xia	中华围产医学杂志	inese Journal of Perinatolo	6	4	255	2003	sn.1007-9408.2003.04.026
1450	有关从业人员中检出沙门	ected among relevant em	智,陈义忠,胡丽萍,高永	en Yizhong,Hu Liping,Gao	中国热带医学	Chinese tropical medicine	3	4	521-521,520	2003	sn.1009-9727.2003.04.060
1451	由肠炎沙门氏菌引起的食	poisoning caused by Salmo	王禹国,吴才仰,潘群慧.	Yuguo,Wu Caiyang,Pan Qu	现代预防医学	nodern preventive medicin	30	1	28-28	2003	sn.1003-8507.2003.01.041
1452	年来健康人群沙门菌带菌	atus among healthy people	虹,吴薇,程刚平,邓晓丽,赵	Vei,Cheng Gangping,Deng	中国卫生检验杂志	ese Journal of Health Inspe	13	6	749-749	2003	sn.1004-8685.2003.06.045
1453	菌性腹泻病原菌分析及耐	d drug resistance changes	李毅,张美和,王艳.	i Yi,Zhang Meihe,Wang Yar	实用医技杂志	l of Practical Medical Tech	10	3	193-195	2003	sn.1671-5098.2003.03.023
1454	菌肠炎76例临床特点及耐	linical characteristics and	李波,洪纬,曲芬.	Li Bo,Hong Wei,Qu Fen.	中国抗生素杂志	hinese Journal of Antibioti	28	9	552-554	2003	sn.1001-8689.2003.09.013
1455	沙门菌食物中毒流行病学	stigation of a case of salm	杨年忠,梁祚法.	Yang Ninzhong,Liang Zuofa	现代预防医学	nodern preventive medicin	30	6	846-846	2003	sn.1003-8507.2003.06.047
1456	坦利沙门氏菌引起肠炎一	nteritis caused by Salmonel	黄宏章,李桂梅.	Huang Hongzhang,Li Guime	内蒙古医学杂志	ner Mongolia Medical Jour	35	6	507	2003	sn.1004-0951.2003.06.072
1457	肠道致病菌的分类及耐药	g resistance analysis of inte	锋芝,马全萍,刘安荣,苑广	a Quanping,Liu Anrong,Yu	中国微生态学杂志	inese Journal of Microecol	15	3	163-164	2003	n.1005-376X.2003.03.020
1458	沙门氏菌引起的食物中毒	case of food poisoning ca	李新平.	Li Xinping.	中国社区医师（综合版）	nunity Physician (Compreh	5	8	66	2003	-
1459	区沙门氏菌感染30例临床	lmonella infection30Case	王若初.	Wang Ruochu.	中华现代临床医药杂志	Journal of Modern Clinical	4	8	43-43	2003	-
1460	儿感染性腹泻病685例病因	rhea in children685Cause a	东一,曹联宏,拜康利,王秀	ao Lianhong,Bai Conley,Wa	中国校医	Chinese school doctor	17	4	357-358	2003	sn.1001-7062.2003.04.047
1461	儿制品引起食物中毒致白	od poisoning caused by Sa	赖伟坚,龚智峰,雷厉风.	Weijian,Gong Zhifeng,Lei Li	广西医科大学学报	al of Guangxi Medical Univ	20	6	935	2003	n.1005-930X.2003.06.050
1462	陕西省食品中食源性致病	orne pathogenic bacteria i	王安礼,刘长宏,连西兰,潘	nli,Liu Changhong,Lian Xila	中国食品卫生杂志	hinese Food Hygiene Journ	15	6	489-491	2003	sn.1004-8456.2003.06.003
1463	中五种食源性致病菌污染	ion status of five foodborn	赵春玲,张文力,韩喜荣,张	Zhang Wenli,Han Xirong,Zh	中国卫生检验杂志	ese Journal of Health Inspe	13	5	570-571	2003	sn.1004-8685.2003.05.014
1464	伤寒沙门菌心肌炎16例临	phimurium Myocarditis16	赵云霞.	Zhao Yunxia.	中华实用医药卫生杂志	urnal of Practical Medicine	1	8	35-36	2003	-
1465	中学伤寒暴发流行的调查	sis of typhoid fever outb	仰霖,谢登煌,包伟民,温远	e Denghuang,Bao Weim	海峡预防医学杂志	Journal of Preventive M	-	5	42	2003	-
1466	对一起伤寒暴发流行的病	h outbreak of typhoid fev	李桂荣;李志廷;	Li Guirong;Li Zhiting;	基层医学论坛	Primary Medicine Forum	-	9	859	2003	-
1467	伤寒暴发的流行病学调查	tigation report on an out	段兴云	Duan Xingyun	疾病监测	disease surveillance	-	6	233	2003	-
1468	3例副伤寒暴发疫情的启示	nment from paratyphoid	周庚荣	Zhou Gengrong	江苏预防医学	angsu Preventive Medici	-	2	84-85	2003	-
1469	起伤寒暴发的流行病学调	nvestigation of an outbre	邓晶,俞月芬,徐进,张渝,崔	ng,Yu Yuefen,Xu Jin,Zha	浙江预防医学	ejiang Preventive Medici	-	5	24-25	2003	sn1007-0931.2003.05.012
1470	起伤寒暴发流行病学调查	investigation report on a	保洪伟	Bao Hongwei	疾病监测	disease surveillance	-	2	74-75	2003	-
1471	门氏菌食物中毒事故的调	nalysis of a Salmonella Foo	许代英,马建国.	Xu Daiying,Ma Jianguo.	中华卫生监督与健康	se Health Supervision and	3	4	351	2004	-
1472	鼠伤寒沙门菌所致的食物	hing Caused by Salmonella	沈来红,余刚.	Shen Laihong,Yu Gang.	预防医学情报杂志	of Preventive Medicine Info	20	2	196-197	2004	sn.1006-4028.2004.02.050
1473	沙门氏菌爆发流行的病原	d analysis of an outbreak	成,闫鹏,胡逢蛟,徐景野,应	eng,Hu Fengjiao,Xu Jingye,	现代预防医学	nodern preventive medicin	31	5	752-753	2004	sn.1003-8507.2004.05.054
1474	中沙门氏菌的检出及药敏	blood culture and analysis	徐海棠,杜娟.	Xu Haitang,Du Juan.	齐鲁医学检验	Qilu Medical Testing	15	1	41-42	2004	sn.1673-5013.2004.01.021
1475	污染头照蛋引起食物中毒	caused by Salmonella-con	孔繁才.	Kong Fancai.	江苏预防医学	iangsu Preventive Medicin	15	1	40-41	2004	sn.1006-9070.2004.01.025
1476	公共场所从业人员沙门菌	ng employees in individual	曹春红.	Cao Chunhong.	预防医学情报杂志	of Preventive Medicine Info	20	4	451-453	2004	sn.1006-4028.2004.04.043
1477	、公共场所从业人员沙门	among workers in individu	曹春红,刘红梅.	Cao Chunhong,Liu Hongme	中华卫生监督与健康	se Health Supervision and	3	2	147144	2004	-
1478	装豆制品熟食引起食物中	caused by illegal sales of	薛志娟.	Xue Zhijuan.	中华医学实践杂志	nese Journal of Medical Pra	3	2	180-181	2004	-

1479	肠炎沙门菌引起的食物	poisoning caused by Salmo	周乃根,徐景野,杨芝厦.	u Naigen,Xu Jingye,Yang Zh	中国预防医学杂志	se Journal of Preventive Me	5	3	204-205	2004	sn.1009-6639.2004.03.016
1480	门氏菌引起食物中毒的调	is of a case of food poisoni	淑荣,杨凤艳,王静,任福	g,Yang Fengyan,Wang Jing	中华卫生监督与健康	se Health Supervision and	3	6	535-535	2004	-
1481	少门氏菌污染状况调查及	identification of Salmonel	焦彦朝,钱莘莘,蔡秋,余萍	nchao,Qian Xinxin,Cai Qiu,	贵州农业科学	uizhou Agricultural Science	32	4	59-59	2004	sn.1001-3601.2004.04.021
1482	公共场所从业人员沙门	workers in catering and pu	杨丽华,陈悦,沈健民.	g Lihua,Chen Yue,Shen Jian	上海预防医学	hanghai Preventive Medicir	16	9	435-435	2004	sn.1004-9231.2004.09.009
1483	02年龙岩市食源性致病菌	nalysis of foodborne patho	丹茅,邱卿如,金建潮,刘素	mao,Qiu Qingru,Jin Jiancha	实用预防医学	ractical preventive medicin	11	5	979-980	2004	sn.1006-3110.2004.05.059
1484	少门菌感染及严重并发症	serious complications in n	王秀兰.	Wang Xiulan.	首都医科大学学报	hal of Capital Medical Unive	25	1	126-127	2004	sn.1006-7795.2004.01.040
1485	船舶食品和垃圾中的细菌	ination in food and garbag	吉生,黄立业,张常印,陈国	uang Liye,Zhang Changyin,	口岸卫生控制	Port health control	9	6	15-16	2004	sn.1008-5777.2004.06.006
1486	餐导致沙门氏菌食物中	monella food poisoning cau	于浩,李炜,赵旭东,奥福田	ao,Li Wei,Zhao Xudong,ofu	中国饮食卫生与健康	inese food hygiene and hea	2	5	37-38	2004	-
1487	型布利丹沙门菌引起的食	ing caused by Salmonella b	杜丽霞,刘烜,姜宏伟.	Lixia,Liu Xuan,Jiang Hongw	预防医学文献信息	ive medicine literature info	10	1	85-86	2004	sn.1672-9153.2004.01.064
1488	少门菌的鉴定及病原学特	ogenic characteristics analy	田维珍,邓光荣,寇艳玲.	izhen,Deng Guangrong,Kou	中国实验诊断学	nese experimental diagnos	8	4	413-414	2004	sn.1007-4287.2004.04.045
1489	沙门氏菌败血症顽固性发	methacin in the treatment	武英,郑锦辉,高茹,路立业	ing,Zheng Jinhui,Gao Ru,Lu	中国医学研究与临床	ese medical research and cl	2	15	-	2004	-
1490	食用的生葱中检出沙门氏	of Salmonella from raw o	焰,孙敏,方敏,陈汛芳,李秋	Min,Fang Min,Chen Xunfa	中国食品卫生杂志	hinese Food Hygiene Journ	16	3	248-249	2004	sn.1004-8456.2004.03.016
1491	间手动开关微生物污染调	icrobial Contamination of	,马荣华,李昆,张凤荣,宗	hua,Li Kun,Zhang Fengron	中国感染控制杂志	ese Journal of Infection Co	3	1	50-51	2004	sn.1671-9638.2004.01.021
1492	血液中分离一株D群伤寒	d a strain from bloodDSal	张晓宇,张碧莹.	Zhang Xiaoyu,Zhang Biying	实用医技杂志	l of Practical Medical Tech	11	9	662-662	2004	sn.1671-5098.2004.09.125
1493	少门氏菌感染病人临床特	are of clinical characteristi	申益萍.	Shen Yiping.	江西医药	Jiangxi Medicine	39	5	379-380	2004	sn.1006-2238.2004.05.047
1494	门氏菌及副溶血性弧菌的	monella and Vibrio parahae	王志强,李志勇,凌莉.	ng Zhiqiang,Li Zhiyong,Ling	食品科学	food science	25	11	252-255	2004	sn:1002-6630.2004.11.066
1495	仁中亚利桑那菌的检测及	analysis of Arizona bacteri	晖,胡浩纹,徐家文,郭明	Hu Haowen,Xu Jiawen,Guo	化学与生物工程	mical and Biological Engine	21	1	54-55	2004	sn.1672-5425.2004.01.020
1496	所致硬脑膜下积水积脓并	perforation caused by Sal	王小苏.	Wang Xiaosu.	中国社区医师	hinese community physicia	20	23	47	2004	-
1497	β-内酰胺酶的鼠伤寒沙门	trumβ-lactamase of Salmo	刘强.	Liu Qiang.	中国实验诊断学	nese experimental diagnos	8	5	552	2004	sn.1007-4287.2004.05.046
1498	引起急性细菌性肠炎爆	k of acute bacterial enteriti	许英路,张秀霞,刘丹彤.	inglu,Zhang Xiuxia,Liu Dant	中国公共卫生管理	ese public health managen	20	3	243-243	2004	sn.1001-9561.2004.03.038
1499	洛克兰沙门氏菌引起的食	poisoning caused by Salmo	瑞怀,张铁刚,李长龙,马洪	,Zhang Tiegang,Li Changlor	医学动物防制	medical animal control	20	3	152-153	2004	sn.1003-6245.2004.03.013
1500	市海淀区食品致病菌监测	f food pathogenic bacteria	伟,关晶玉,马平焕,毕欣,翟	Jingyu,Ma Pinghuan,Bi Xin	中国食品卫生杂志	hinese Food Hygiene Journ	16	6	516-518	2004	sn.1004-8456.2004.06.011
1501	门菌骨髓炎的临床特点和	and treatment of Salmone	守荣,梁雨田,董纪元,缪	ong,Liang Yutian,Dong Jiyu	中国医师杂志	Chinese Physician Journal	6	1	58-60	2004	sn.1008-1372.2004.01.024
1502	03年伤寒、副伤寒沙门菌	e study on the pathogenicit	鲍倡俊,庄菱.	Bao Changjun,Zhuang Ling.	疾病控制杂志	journal of disease control	8	5	402-404	2004	sn.1674-3679.2004.05.006
1503	细菌感染所致原发性败血	bacterial infection in AIDS	吴立奇.	Wu Liqi.	上海预防医学	hanghai Preventive Medicir	16	10	478-479	2004	sn.1004-9231.2004.10.005
1504	售牛乳及乳制品卫生情况	ene status of commercial m	彦明,郭抗抗,张国军,谢宝	Guo Kangkang,Zhang Guoj	中国动物检疫	China Animal Quarantine	21	11	29-31	2004	n.1005-944X.2004.11.019
1505	售鲜鸡蛋和生鸡肉的沙门	ination of fresh eggs and r	董忠,陈倩,骆海鹏.	Zhong,Chen Qian,Luo Hai	中国食品卫生杂志	hinese Food Hygiene Journ	16	6	514-516	2004	sn.1004-8456.2004.06.010
1506	少门菌感染及严重并发症	serious complications in n	医科大学附属北京友谊	ospital Affiliated to Capital	首都医科大学学报	hal of Capital Medical Unive	-	1	-	2004	-
1507	利丹沙门氏菌引起的食	poisoning caused by Salm	芦晓光,王岩,刘汉伟.	aoguang,Wang Yan,Liu Har	中国食品卫生杂志	hinese Food Hygiene Journ	16	5	454-455	2004	sn.1004-8456.2004.05.023
1508	源性致病菌及其耐药性	foodborne pathogenic bac	天,李志刚,姚景惠,付萍,杨	,Li Zhigang,Yao Jinghui,Fu	卫生研究	health research	33	1	49-54	2004	sn.1000-8020.2004.01.013
1509	2002-2003食源性致病菌监	03Foodborne Pathogen Sur	巢国祥,徐勤,朱炳炎,马锋	xiang,Xu Qin,Zhu Bingyan,	中国卫生检验杂志	ese Journal of Health Inspe	14	4	484-486	2004	sn.1004-8685.2004.04.055
1510	细菌性食物中毒流行病学	tion and analysis of househ	陈兴乐,黄林,黄兆勇,方志	,Huang Lin,Huang Zhaoyor	广西预防医学	uangxi Preventive Medicin	10	4	197-199	2004	n.1673-758X.2004.04.002
1511	门氏菌引起食物中毒的调	n a case of food poisoning	明杰,白俊涛,孙冬梅,孟宪	,Bai Juntao,Sun Dongmei,	中国饮食卫生与健康	inese food hygiene and hea	2	3	64	2004	-
1512	西沙某岛水域海水细菌调	er bacteria in waters of a c	伟,周一平,古妙宁,赖福	ei,Zhou Ping,Gu Miaoning,L	解放军医学杂志	PLA Medical Journal	29	5	463-464	2004	sn:0577-7402.2004.05.032
1513	致病菌的分离及其阳性结	ria from blood culture and	曹锡标.	Cao Xibiao.	中国自然医学杂志	ese Journal of Natural Med	6	2	65-68	2004	-
1514	林沙门菌引起食物中毒的	d poisoning caused by Sal	连秀,彭智会,赵维勇,张文	Peng Zhihui,Zhao Weiyong	中国卫生检验杂志	ese Journal of Health Inspe	14	3	386-386	2004	sn.1004-8685.2004.03.079
1515	区鼠伤寒沙门菌病流行动	amics of salmonellosis typ	周文华,康文清,宋继军.	Venhua,Kang Wenqing,Son	河南预防医学杂志	Journal of Preventive Me	15	6	339-341	2004	sn.1006-8414.2004.06.007

1516	型副伤寒并发胸腹水1例报	licated by thoracic and asc	陈胜利.	Chen Shengli.	中国航天医药杂志	ha Aerospace Medicine Jou	6	2	74	2004	sn.1672-9463.2004.02.043
1517	性腹泻病原菌的分布及其	spital infectious diarrhea a	华,刘华,张春平,乔宁,黄丽	,Zhang Chunping,Qiao Ning	世界感染杂志	world journal of infections	4	5	468-470	2004	-
1518	氏菌引起胆管炎合并胆石症	emia caused by cholelithias	李明,利娟.	Li Ming,Lijuan.	中国厂矿医学	ha factory and mining medi	17	1	6	2004	sn.1674-8182.2004.01.078
1519	1975-2003年沙门菌监测结	3Analysis of annual Salmo	国樾,陈建辉,李海丹,罗朝	en Jianhui,Li Haidan,Luo Cl	海峡预防医学杂志	s Journal of Preventive Me	10	6	7-10	2004	sn.1007-2705.2004.06.003
1520	居民主要腹泻病原菌现状	of main diarrhea pathogeni	,梅玲玲,朱敏,张俊彦,徐黎	Lingling,Zhu Min,Zhang Ju	中国卫生检验杂志	ese Journal of Health Inspe	14	4	415-416	2004	sn.1004-8685.2004.04.012
1521	炎合并肠炎沙门菌败血症一	A complicated with Salmon	张金辉,陈志辉.	Zhang Jinhui,Chen Zhihui.	第二军医大学学报	f Second Military Medical U	25	2	191-191	2004	n:0258-879X.2004.02.040
1522	伤寒沙门菌食物中毒的调	od poisoning case caused	朱玉兰,沈广顺,邵爱和.	ulan,Shen Guangshun,Shao	职业与健康	Occupation and health	20	8	43-44	2004	sn.1004-1257.2004.08.042
1523	年生鲜鸡肉中食源性致病	ontamination of foodborn	冰,金化瑞,张燕,王利平,韩	n Huarui,Zhang Yan,Wang	中华卫生监督与健康	se Health Supervision and H	3	9	769-770	2004	-
1524	路地区1974年～1996年食	a1974year~1996annual foo	晁蕊,迪丽拜.	Chao Rui,dilibai.	中国食品卫生杂志	hinese Food Hygiene Journ	16	1	54-56	2004	sn.1004-8456.2004.01.019
1525	炎沙门菌引起的食物中毒	ood poisoning caused by Sa	沈翠梅.	Shen Cuimei.	预防医学文献信息	ive medicine literature info	10	1	82-83	2004	sn.1672-9153.2004.01.061
1526	区败血症菌种变化及其特	racteristics of septicemia b	王鸿程,李艳萍,李万成,陈洁	heng,Li Yanping,Li Wanche	泸州医学院学报	rnal of Luzhou Medical Coll	27	1	28-30	2004	sn.1000-2669.2004.01.010
1527	枣庄市沙门菌菌型调查分析	nalysis of Salmonella strain	孙延銮,王介友.	Sun Yanfu,Wang Jieyou.	中国预防医学杂志	se Journal of Preventive Me	5	2	151	2004	sn.1009-6639.2004.02.037
1528	食物中毒流行病学评价与	isoning epidemiological ev	振柱,黄林,杨文敏,彭琪元,林	uang Lin,Yang Wenmin,Per	广西预防医学	Guangxi Preventive Medicin	10	4	200-204	2004	n.1673-758X.2004.04.003
1529	定量危险性评估的初步研究	lmonella in shell eggs in Ch	赵志晶,刘秀梅.	Zhao Zhijing,Liu Xiumei.	中国食品卫生杂志	hinese Food Hygiene Journ	16	3	201-206	2004	sn.1004-8456.2004.03.002
1530	患者血液一株猪霍乱沙门菌	as obtained from the blood	俞莲花,马琴飞,竺佩燕.	Lianhua,Ma Qinfei,Zhu Peiy	江西医学检验	Jiangxi Medical Laboratory	22	1	21-22,4	2004	sn.1674-1129.2004.01.009
1531	雷利沙门菌食物中毒的调查	rol of a case of Salmonella	靖峰,王和生,刘江波,刘思美	,Wang Hesheng,Liu Jiangb	中华国际医学杂志	se International Medical Jo	4	5	250-253	2004	-
1532	5株甲型副伤寒杆菌药敏分	tibility analysis of strains o	孟曙芳,谢珏.	Meng Shufang,Xie Jue.	浙江预防医学	hejiang Preventive Medicin	16	12	24,36	2004	sn.1007-0931.2004.12.017
1533	01年广西伤寒监测结果分	typhoid fever surveillance r	杰,唐振柱,梁大斌,王鸣柳	g Zhenzhu,Liang Dabin,Wa	广西预防医学	Guangxi Preventive Medicin	10	3	142-144	2004	n.1673-758X.2004.03.005
1534	28例儿童甲型副伤寒分析	f a case of paratyphoid feve	黄梦.	Huang Meng.	中华当代医药	inese contemporary medic	-	11	-	2004	-
1535	德尔卑沙门菌引起的食物中	se of food poisoning caused	石亚素,童国忠.	Shi Yasu,Tong Guozhong.	疾病监测	disease surveillance	19	4	137-138	2004	sn.1003-9961.2004.04.010
1536	生儿病区发生医院感染6例	occurred in the neonatal w	刘斌哲.	Liu Binzhe.	现代中西医结合杂志	grated Traditional Chinese	13	21	2889-2889	2004	sn.1008-8849.2004.21.076
1537	起流感与伤寒同时爆发的调	ultaneous outbreak of influ	发,高景枝,郑向梅,崔龙,李	ingzhi,Zheng Xiangmei,Cui	现代预防医学	modern preventive medicin	31	5	749-750	2004	sn.1003-8507.2004.05.051
1538	180起细菌性食物中毒病因	nalysis of causes of bacter	陶非,赵文彬.	Tao Fei,Zhao Wenbin.	现代预防医学	modern preventive medicin	31	4	575-576	2004	sn.1003-8507.2004.04.055
1539	杭州市细菌性食物中毒病	s of pathogenic bacteria of	静,吴奇志,韦东芳,王一泓,	zhi,Wei Dongfang,Wang Yi	中国卫生检验杂志	ese Journal of Health Inspe	14	3	320-320	2004	sn.1004-8685.2004.03.030
1540	沙门菌致食物中毒的细菌	tion on food poisoning caus	石亚素,童国忠.	Shi Yasu,Tong Guozhong.	中国卫生检验杂志	ese Journal of Health Inspe	14	6	784	2004	sn.1004-8685.2004.06.083
1541	2003年食品微生物监测结	sis of annual food microbio	汪涛,谈晔.	Wang Tao,Tan Ye.	预防医学论坛	Preventive Medicine Forum	10	5	622-623	2004	sn.1672-9153.2004.05.084
1542	沙门菌食物中毒的实验室	lysis of food poisoning caus	张满珍,于旭东,龚宇君.	Manzhen,Yu Xudong,Gong	预防医学文献信息	ive medicine literature info	10	2	205-205	2004	sn.1672-9153.2004.02.049
1543	肠炎沙门菌引起的食物中毒	of food poisoning caused	潘亚光,徐维纲,张静.	Yaguang,Xu Weigang,Zhang	上海预防医学	hanghai Preventive Medicin	16	11	555-555	2004	sn.1004-9231.2004.11.025
1544	殖场主要病原菌耐药性及	hology of main pathogenic	王全亮,李勤建,李忠建.	Quanliang,Li Qinjian,Li Zho	湖北畜牧兽医	al Husbandry and Veterina	-	3	39-40	2004	n.1007-273X.2004.03.013
1545	引起学生腹泻流行的调查	valence of diarrhea among	详,王树坤,温润生,杨学文,王	un,Wen Runsheng,Yang Xu	中国热带医学	Chinese tropical medicine	4	1	117-118	2004	sn.1009-9727.2004.01.060
1546	血液及粪便中同时分离出	as isolated from the patien	毓安,李珍大,王卫萍,邵海	Zhenda,Wang Weiping,Sha	临床检验杂志	journal of clinical testing	22	5	363	2004	n.1001-764X.2004.05.044
1547	05株腹泻病原菌耐药性监	resistance monitoring resu	王红旗,徐军,夏光明.	g Hongqi,Xu Jun,Xia Guangm	中华实用医学	Chinese Practical Medicine	6	9	45-46	2004	-
1548	起沙门菌引起食物中毒的调	into food poisoning caused	周于祥.	Zhou Yuxiang.	预防医学情报杂志	of Preventive Medicine Info	20	3	287	2004	sn.1006-4028.2004.03.063
1549	肠炎沙门菌引起食物中毒	poisoning caused by Salmo	秦毅,迂君.	Qin Yi,Yu Jun.	中国公共卫生	Chinese public health	20	9	1060-1060	2004	sn:1001-0580.2004.09.069
1550	寒沙门氏菌致化脓性关节	aused by Salmonella typhin	李燕,庄学伟,夏西燕.	Yan,Zhuang Xuewei,Xia Xiya	中华医药杂志	Chinese Medical Journal	4	2	175-175	2004	-
1551	县1起肠炎沙门菌食物中毒	alysis of Salmonella Enterit	李友书.	Li Youshu.	预防医学情报杂志	of Preventive Medicine Info	20	2	198-198	2004	sn.1006-4028.2004.02.052
1552	门氏菌引起食物中毒的调	is of a case of food poisoni	徐开玲,关春煦,王婕,王玲	g,Guan Chunxu,Wang Jie,W	中国城乡企业卫生	ygiene in urban and rural ar	-	2	12	2004	sn.1003-5052.2004.02.007

1553	谱β-内酰胺酶的沙门菌感染	Salmonella infection missed	刘强,张红梅,赵靓.	iang,Zhang Hongmei,Zhao	第四军医大学学报	f Fourth Military Medical U	25	11	997-997	2004	sn:1000-2790.2004.11.036
1554	腹泻病人志贺氏菌药敏结	eptibility results of Shigella	阴爱珍,郭旭昌,孙明德.	izhen,Guo Xuchang,Sun Mi	中国实用医学研究杂志	ournal of Practical Medical	3	3	225-226	2004	-
1555	痛为首发症状的沙门菌属	er occipital neuralgia as the	杨享元,陈贵丽.	Yang Xiangyuan,Chen Guili	中华当代医药	inese contemporary medic	-	9	-	2004	-
1556	织病并沙门氏菌脓肿及巨	vith salmonella abscess and	颜美秋.	Yan Meiqiu.	浙江临床医学	Zhejiang Clinical Medicine	6	7	550	2004	sn.1008-7664.2004.07.059
1557	患者两次并发感染猪霍乱	patient infected with chol	贾冀川,刘钉宾.	Jia Jichuan,Liu Dingbin.	中华检验医学杂志	e Journal of Laboratory Me	27	10	659	2004	sn:1009-9158.2004.10.030
1558	院鼠伤寒沙门菌感染流行	actors of Salmonella typhir	申田,叶作栋.	Shen Tian,Ye Zuodong.	中国感染控制杂志	ese Journal of Infection Co	3	2	146-147	2004	sn.1671-9638.2004.02.019
1559	东省市售食品中沙门菌污	ella contamination in comr	,严纪文,王海燕,宋曼丹,黄	ven,Wang Haiyan,Song Mar	华南预防医学	uth China Preventive Medic	30	5	50-51	2004	sn.1671-5039.2004.05.024
1560	甲病例临床分析及药物疗	atyphoid A cases and comp	朱先女.	Zhu Xiannu.	中国感染控制杂志	ese Journal of Infection Co	3	3	234-235	2004	sn.1671-9638.2004.03.016
1561	细菌性食物中毒事故分析及	poisoning accidents and dis	张嵘,张健,谷政,龚玲.	ong,Zhang Jian,Gu Zheng,G	中华卫生监督与健康	se Health Supervision and H	3	7	617-619	2004	-
1562	瓯海区1998～2002年伤寒疫	y1998~2002Analysis of anr	诸田胜.	Moroda Katsu.	浙江预防医学	hejiang Preventive Medicin	16	1	38-38	2004	sn.1007-0931.2004.01.023
1563	肠炎沙门菌引起的食物中毒	ood poisoning caused by Sa	文中芹,郑玉柱,余枫华.	hongqin,Zheng Yuzhu,Yu Fe	预防医学论坛	Preventive Medicine Forum	10	4	425-426	2004	sn.1672-9153.2004.04.028
1564	2001年猕猴微生物携带状	n microbial carrying status	杨敬,隋丽华,田浩,郑振峰,孙	ui Lihua,Tian Hao,Zheng Zhe	实验动物科学与管理	ry Animal Science and Man	21	1	47-50	2004	sn.1006-6179.2004.01.014
1565	中沙门氏菌和志贺氏菌的	onella and Shigella among	吴杰.	Wu Jie.	现代预防医学	modern preventive medicin	31	1	131-132	2004	sn.1003-8507.2004.01.070
1566	品中沙门氏菌的耐药性研	n drug resistance of Salmor	新川,李灶平,谢劲心,林斯星	,Li Zaoping,Xie Jinxin,Lin Si	现代预防医学	modern preventive medicin	31	3	330-332	2004	sn.1003-8507.2004.03.007
1567	超广谱β-内酰胺酶菌的检测	spectrumβ-Detection and	何理,吴松远,王晓红,郭照静	ngyuan,Wang Xiaohong,Gu	实用医技杂志	l of Practical Medical Tech	11	16	1583-1584	2004	sn.1671-5098.2004.16.043
1568	林沙门氏菌引起的食物中	case of food poisoning cau	张俊义,栗勇坤,王燕.	ng Junyi,Li Yongkun,Wang	中华卫生监督与健康	se Health Supervision and H	3	9	819-820	2004	-
1569	2003年食源性致病菌污染状	nnual foodborne pathogeni	胡婕,石韬.	Hu Jie,Shi Tao.	公共卫生与预防医学	Health and Preventive Me	15	2	33-34	2004	sn.1006-2483.2004.02.022
1570	5岁以下儿童细菌性脑膜炎	etiology of bacterial menin	台,梁大斌,廖和壮,徐志一,	song,Quan Yi,Liang Dabin,L	中国热带医学	Chinese tropical medicine	4	3	333-336	2004	sn.1009-9727.2004.03.010
1571	鼠伤寒沙门菌所致的食物	ning Caused by Salmonella	沈来红,余刚.	Shen Laihong,Yu Gang.	攀枝花医药	Panzhihua Pharmaceutical	26	1	113-114	2004	-
1572	肠炎沙门菌引起的食物中毒	ood poisoning caused by Sa	陈荣华,陈冬林.	Chen Ronghua,Chen Donglin	预防医学论坛	Preventive Medicine Forum	10	4	427-428	2004	sn.1672-9153.2004.04.030
1573	0株伤寒沙门菌药敏试验结	rug susceptibility test resul	董明.	Dong Ming.	江西医学检验	Jiangxi Medical Laboratory	22	5	477-477	2004	sn.1674-1129.2004.05.051
1574	2000～2002年食源性疾病监	2002Annual Foodborne Di	邵绶衡,李原浩,陈焱,段丽玚	ng,Li Yuanhao,Chen Yan,D	中国热带医学	Chinese tropical medicine	4	4	632-635,637	2004	sn.1009-9727.2004.04.075
1575	区伤寒耐药情况及临床疗	and clinical efficacy of typh	曾明明,曾传生.	g Mingming,Zeng Chuansh	中华现代中西医杂志	of Modern Chinese and W	2	9	835-836	2004	-
1576	起沙门菌引起的食物中毒调	case of food poisoning ca	姜金胜.	Jiang Jinsheng.	海峡预防医学杂志	s Journal of Preventive Me	10	4	26-26	2004	sn.1007-2705.2004.04.024
1577	肉品常见中素养性细菌污染	ltured bacterial contamina	黄祖华,彭梅仙.	Huang Zuhua,Peng Meixian	肉品卫生	Meat hygiene	-	6	31-32	2004	-
1578	革兰阴性杆菌的鉴定及药敏	cilli in blood samples and a	周忠华,刘华,杨静,喻华.	honghua,Liu Hua,Yang Jing,	实用医院临床杂志	ctical Hospital Clinical Jour	1	4	93-94	2004	sn.1672-6170.2004.04.054
1579	起沙门氏菌食物中毒的调	into a case of salmonella fo	付永清,于丽红,苏黎,李文军	gqing,Yu Lihong,Zurich,Li W	新疆预防医学（汉文版）	reventive Medicine (Chines	22	1	4-5	2004	-
1580	菌引起的食物中毒调查及相	poisoning caused by Salmo	姜涛,许正,齐惠荣.	ng Tao,Xu Zheng,Qi Huiron	中华卫生监督与健康	se Health Supervision and H	3	2	149-150	2004	-
1581	伯林沙门菌致小儿败血症	lin causes septicemia in ch	杨燕,邓三季.	Yang Yan,Deng Sanji.	中国感染控制杂志	ese Journal of Infection Co	3	3	281-281	2004	sn.1671-9638.2004.03.042
1582	起沙门氏菌食物中毒的调	into a case of salmonella fo	王永辉,刘红晶.	Wang Yonghui,Liu Hongjing	中国饮食卫生与健康	inese food hygiene and hea	2	3	62	2004	-
1583	副伤寒沙门氏菌流行株的	prevalent strains of Salmon	游旅,张玉琼.	Travel,Zhang Yuqiong.	贵州医药	Guizhou Medicine	28	4	371-372	2004	n.1000-744X.2004.04.048
1584	株伤寒沙门氏菌药敏试验	g susceptibility test results	任结梅,张庆业,蔡晓虹,罗	ei,Zhang Qingye,Cai Xiaoh	海峡药学	Straits Pharmacy	16	6	58-60	2004	sn.1006-3765.2004.06.031
1585	伤寒沙门氏菌对20种抗生素	hi in a patient20Antibiotic	吕承旻.	Lu Chengmin.	华夏医学	Chinese Medicine	17	4	526-527	2004	sn.1008-2409.2004.04.027
1586	皮肤黏膜淋巴综合征1例	neous lymphatic syndrome	刘晓曲.	Liu Xiaoqu.	黔南民族医专学报	of Qiannan National Medica	17	4	245	2004	sn.1008-4983.2004.04.043
1587	腹泻病原菌的分布及耐药	and drug resistance of dia	陈慧玉,何加宁,吴茜.	en Huiyu,He Jianing,Wu Qi	华西医学	West China Medicine	19	3	419-420	2004	sn.1002-0179.2004.03.038
1588	共场所从业人员携带沙门	rried by employees in publ	明霞,马雪兰,彭广萍,黄玉	a Xuelan,Peng Guangping,	江西医学检验	Jiangxi Medical Laboratory	22	3	279-279,238	2004	sn.1674-1129.2004.03.053
1589	副伤寒沙门菌的鉴定及药敏	susceptibility analysis of Sa	刘小芹,赵洁.	Liu Xiaoqin,Zhao Jie.	齐鲁医学检验	Qilu Medical Testing	15	2	47,65	2004	sn.1673-5013.2004.02.030

1590	一起沙门氏菌肠炎暴发调	n into a Salmonella Enterit	徐建荣,叶众,杨存军.	ianrong,Ye Zhong,Yang Cur	疾病监测	disease surveillance	19	10	378-379	2004	sn.1003-9961.2004.10.009
1591	儿急性腹泻粪便中检出病	le detected in feces of an i	周文华,石红娜.	Zhou Wenhua,Shi Hongna.	河南预防医学杂志	n Journal of Preventive Me	15	2	100	2004	sn.1006-8414.2004.02.034
1592	那沙门菌所致食物中毒的	case of food poisoning cau	杨美琴,王凤云,王锐.	Meiqin,Wang Fengyun,Wa	职业与健康	Occupation and health	20	5	62-62	2004	sn.1004-1257.2004.05.050
1593	伤寒沙门菌食物中毒的临	od poisoning caused by Sal	彭俊,李宝芬,白鹏飞.	ng Jun,Li Baofen,Bai Pengf	中国预防医学杂志	se Journal of Preventive Me	5	5	383	2004	sn.1009-6639.2004.05.040
1594	因素引起的食物中毒爆发	n a food poisoning outbre	昌芳,陈炳耀,李涛,刘素	gfang,Chen Bingyao,Li Tao,	国际医药卫生导报	ational Medical and Health	-	17	11-12	2004	sn.1007-1245.2004.17.004
1595	业人员沙门菌属和志贺菌	igella carriage among emp	李爱英,刘红.	Li Aiying,Liu Hong.	中国卫生检验杂志	ese Journal of Health Inspe	14	2	218-219	2004	sn.1004-8685.2004.02.048
1596	氏菌引起食物中毒的调查	ort on food poisoning caus	周华英,张永英.	hou Huaying,Zhang Yongyi	中国饮食卫生与健康	inese food hygiene and hea	2	6	44	2004	-
1597	福氏志贺氏菌药敏试验与	l drug efficacy analysis of	郑建林,袁萍.	Zheng Jianlin,Yuan Ping.	中华医学写作杂志	hese Journal of Medical Wr	11	6	457-459	2004	-
1598	寒沙门氏菌引起腹膜后脓	bscess caused by Salmone	周燕,主劲,查筑红.	u Yan,main force,Cha Zhuh	检验医学与临床	boratory medicine and clini	1	3	140	2004	sn.1672-9455.2004.03.030
1599	新港沙门菌引起的食物中	food poisoning caused by S	刘旭光,王亚东,宋文君.	uang,Wang Yadong,Song W	预防医学论坛	Preventive Medicine Forum	10	4	434-434	2004	sn.1672-9153.2004.04.037
1600	业人员和外环境沙门菌调	ella among service industr	陈伟文,冯文悦.	Chen Weiwen,Feng Wenyue	职业与健康	Occupation and health	20	4	4-7	2004	sn.1004-1257.2004.04.002
1601	康人群沙门菌属带菌情况	Salmonella carriage in hea	周茜.	Zhou Qian.	江苏预防医学	iangsu Preventive Medicin	15	3	82-82	2004	sn.1006-9070.2004.03.050
1602	炎沙门氏菌食物中毒的调	is of a case of Salmonella E	鲁溪,沙飞,陈冬梅,杨韶	i,Shafei,Chen Dongmei,Yan	中华卫生监督与健康	se Health Supervision and	3	7	627-628	2004	-
1603	2002年食品从业人员健康	sis of annual health exami	杨慧芳.	Yang Huifang.	中华卫生监督与健康	se Health Supervision and	3	2	159-159	2004	-
1604	大肠杆菌、沙门氏菌分离	cation of Escherichia coli a	,王琴,赵耘,冯忠武,徐士	g Qin,Zhao Yun,Feng Zhong	中国预防兽医学报	rnal of Preventive Veterina	26	2	142-145	2004	sn.1008-0589.2004.02.016
1605	例伊斯坦堡沙门菌所致食	oisoning caused by Salmo	何东娥.	He Dong'e.	中华综合临床医学杂志	nal of Comprehensive Clini	6	9	61	2004	-
1606	南省红河地区副伤寒甲杆	of A. paratyphoid in Hongh	陈贵辽,李国伦,张兵.	n Guiliao,Li Guolun,Zhang	云南医药	Yunnan Medicine	25	6	492-493	2004	-
1607	急性细菌性肠炎暴发来	eristics by handling an acu	张秀霞.	Zhang Xiuxia.	中华卫生监督与健康	se Health Supervision and	3	1	84-85	2004	-
1608	沙门菌耐药性与菌株质粒	stance and strain plasmids	运能,郭炳才,邓曼玲,袁荣	,Guo Bingcai,Deng Manlin	疾病控制杂志	journal of disease control	8	5	405-407	2004	sn.1674-3679.2004.05.007
1609	株沙门菌分型鉴定及药敏	ping, identification and dru	黄思红,林峰.	Huang Sihong,Lin Feng.	浙江预防医学	hejiang Preventive Medicin	16	3	32-32	2004	sn.1007-0931.2004.03.020
1610	寒沙门菌生化和血清学特	aracteristics and analysis o	张伯兰.	Chamberlain.	中国卫生检验杂志	ese Journal of Health Inspe	14	6	739	2004	sn.1004-8685.2004.06.047
1611	地区92株伤寒沙门菌耐药	sis of drug resistance of Sal	宗良,吴有全,邹敏超,钟锐	Vu Youquan,Zou Minchao,	现代医药卫生	modern medicine and healt	20	19	2011-2012	2004	sn.1009-5519.2004.19.047
1612	血培养中常见病原菌及耐	ic bacteria and drug resista	迟富丽,王惠云,贡桂清.	uli,Wang Huiyun,Gong Gui	海南医学	Hainan Medicine	15	8	125-126	2004	sn.1003-6350.2004.08.084
1613	血培养病原菌种类及耐药	bacteria species and drug	王超,曾其莉,艾彪,段六生	ao,Zeng Qili,Ai Biao,Duan	北省卫生职工医学院学	Provincial Medical College	17	1	42-43	2004	-
1614	鸡沙门氏菌引起食物中毒	ella chicken causes food p	孟琳.	Meng Lin.	现代预防医学	modern preventive medicin	31	1	30-30	2004	sn.1003-8507.2004.01.046
1615	甲型副伤寒暴发流行的调	ort on an outbreak of pa	黎明强,殷凯,刘金纪	ming Qiang,Yin Kai,Liu J	广西预防医学	uangxi Preventive Medici	-	5	317	2004	-
1616	1起甲型副伤寒暴发的调	tigation and Analysis of	庆福,王树坤,钦绍德,马国	Wang Shukun,chinsaud,	预防医学情报杂志	f Preventive Medicine In	-	5	557-558	2004	-
1617	寒暴发疫情流行因素的病	the epidemic factors of	振开,黄运能,黄少新,袁荣	ang Yunneng,Huang Sha	中国自然医学杂志	se Journal of Natural Me	-	3	129-130	2004	-
1618	起伤寒暴发流行疫区消毒	demic area where a typh	莫遵华,李秀连	Mo Zunhua,Li Xiulian	中国消毒学杂志	nese Journal of Disinfec	-	3	1	2004	-
1619	源污染引起的甲型副伤寒	A outbreak caused by w	,田克城,潘忠伦,欧建中,	City,Pan Zhonglun,Ou Jia	预防医学情报杂志	f Preventive Medicine In	-	4	455-456	2004	-
1620	接触型伤寒暴发的流行病	ation report on a living	,周国甫,李华民,谢尚兰,陈	ofu,Li Huamin,Xie Shang	湖北预防医学杂志	Journal of Preventive Me	-	4	34-35	2004	-
1621	菌者引起的伤寒暴发流行	typhoid outbreaks caus	焦芳桓	Jiao Fanghuan	医学动物防制	medical animal control	-	7	428-429	2004	-
1622	麦盖提县一起学校伤寒暴	oid fever outbreak in a s	,胡吉力提甫,阿地力,艾合	g,Hu Jili Tipu,Adili,Aihem	疾病监测	disease surveillance	-	6	235	2004	-
1623	起甲型副伤寒暴发的调查	into the outbreak of para	林向利,李瑞英,张成翠,李	,Li Ruiying,Zhang Cheng	预防医学文献信息	ve medicine literature inf	-	3	278-279	2004	-
1624	异食癖引发副伤寒暴发的	phoid outbreak caused	朱海明,韩建忠	hu Haiming,Han Jianzho	中国动物保健	China animal health	-	4	1	2004	-
1625	学校甲型副伤寒暴发的流	paratyphoid A outbreak i	信,张进,戴健尔,易槐明,段	ng Jin,Dai Jian'er,Yi Hua	中国公共卫生	Chinese public health	-	4	1	2004	-
1626	伤寒暴发的流行病学调查	estigation and analysis o	恒,杨春梅,王福元,张红霞	unmei,Wang Fuyuan,Zh	解放军预防医学杂志	ve Medicine of the Peopl	-	1	69	2004	-

1627	寒暴发地区住院伤寒病例	ver cases in typhoid feve	袁萍,李诗佩	Yuan Ping,Li Shipei	预防医学文献信息	ve medicine literature inf	-	1	115-116	2004	-
1628	州市婴幼儿腹泻的病原谱	m and drug resistance spec	植,徐海滨,李海丹,林震宇	Xu Haibin,Li Haidan,Lin Zhe	中国自然医学杂志	ese Journal of Natural Med	7	1	13-15	2005	-
1629	镇海区1985～2003年伤寒	y1985~2003Analysis of ann	-	-	中国公共卫生管理	ese public health managen	21	5	406-407	2005	-
1630	糕点沙门菌污染食物中毒	isoning contaminated by S	耀启,林立新,王东勉,陈志	in Lixin,Wang Dongmian,Ch	华南预防医学	uth China Preventive Medic	31	5	71-71	2005	sn.1671-5039.2005.05.034
1631	京地区1 542株腹泻病原药	of drug susceptibility test re	,鲍春梅,崔恩博,徐军,李清	unmei,Cui Eunbo,Xu Jun,L	中华检验医学杂志	se Journal of Laboratory Me	28	4	384-386	2005	sn:1009-9158.2005.04.014
1632	年10年间沙门菌感染的临	amination and analysis of S	-	-	学（临床生物化学与检验	ical Biochemistry and Labo	26	4	封底	2005	-
1633	染患者246例血培养阳性结	ients246Analysis of positiv	,徐安平,李建武,吴志成,李	Anping,Li Jianwu,Wu Zhic	中华检验医学杂志	se Journal of Laboratory Me	28	2	178-180	2005	sn:1009-9158.2005.02.017
1634	沙门氏菌的分离鉴定与药	g susceptibility test of Salr	,马红霞,于守平,单晓枫,胡	ngxia,Yu Shouping,Shan Xia	经济动物学报	ournal of Economic Zoolog	9	4	231-234	2005	sn.1007-7448.2005.04.015
1635	血培养病原菌分布和耐药	acteria distribution and dr	祝进,徐礼锋,陆军,陆顺建	Jin,Xu Lifeng,army,Lu Shun	检验医学	laboratory medicine	20	4	378-381	2005	sn.1673-8640.2005.04.008
1636	2003年食物中毒病原菌检	od poisoning pathogenic b	许磊,邓辉,刘孝严.	u Lei,Deng Hui,Liu Xiaoyan	中国预防医学杂志	se Journal of Preventive Me	6	4	385-386	2005	sn.1009-6639.2005.04.049
1637	社区及医院败血症病原菌	and prognosis in communit	燕萍,林萍萍,王宗明,谢国	Pingping,Wang Zongming,	临床检验杂志	journal of clinical testing	23	6	466-467	2005	n.1001-764X.2005.06.038
1638	人员沙门氏菌和志贺氏菌	monella and Shigella strains	一曼,邱亚群,张倩,黄新	Qiu Yaqun,Zhang Qian,Hua	中华医学与健康	Chinese medicine and healt	2	4	36-38	2005	-
1639	区沙门菌流行特点及耐药	teristics and drug resistanc	-	-	中国预防医学杂志	se Journal of Preventive Me	6	5	462-463	2005	-
1640	中食源性致病菌污染状况	n status of foodborne path	华,吴平芳,刘涛,王瑞端,陈	ngfang,Liu Tao,Wang Ruidu	中国热带医学	Chinese tropical medicine	5	2	357-358	2005	sn.1009-9727.2005.02.086
1641	医院感染调查与危险因素	k factor analysis of pediatr	许素珍,王小永.	Xu Suzhen,Wang Xiaoyong	中国感染控制杂志	ese Journal of Infection Co	4	3	238-239	2005	sn.1671-9638.2005.03.016
1642	分地区食品中致病菌污染	ria contamination in food i	周惠新,杨庆文,李晓莉.	Huixin,Yang Qingwen,Li X	职业与健康	Occupation and health	21	8	1153	2005	sn.1004-1257.2005.08.041
1643	市生鸡肉中沙门菌的耐药	ance of Salmonella in raw	,刘渠,刘衡川,李灶平,白	Qu,Liu Hengchuan,Li Zaopi	现代预防医学	modern preventive medicin	32	7	732753	2005	sn.1003-8507.2005.07.011
1644	兰阴性菌耐药性连续5年监	continues in Kunming5An	王雪娟,杜廷义,张宏波,刘	uejuan,Du Tingyi,Zhang Ho	中国呼吸与危重监护杂志	urnal of Respiratory and Cr	4	4	298-302	2005	sn.1671-6205.2005.04.017
1645	流行小儿甲型副伤寒216	children spreads in Yuxi Ci	马永寿,马树芬.	Ma Yongshou,Ma Shufen.	现代诊断与治疗	dern Diagnosis and Treatm	16	2	81-82	2005	sn.1001-8174.2005.02.008
1646	炎粪培养病原学分布及耐	g resistance analysis of fec	蒋煜,都国基.	Jiang Yu,Du Guoji.	海南医学	Hainan Medicine	16	6	147-148	2005	sn.1003-6350.2005.06.099
1647	售3类食品肠道致病菌污染	on the contamination stat	伟文,冯文悦,崔楚平,莫群	,Feng Wenyue,Cui Chupin	职业与健康	Occupation and health	21	12	1881-1883	2005	sn.1004-1257.2005.12.003
1648	区部分医院血培养分离菌	changes of blood culture is	光辉,朱德妹,张婴元,汪	,Zhu Demei,Zhang Yingyua	中华医院感染学杂志	ournal of Hospital Infectiou	15	6	691-696	2005	sn:1005-4529.2005.06.033
1649	菌所致硬膜下积水积脓并	brane perforation caused	王小苏.	Wang Xiaosu.	中国乡村医药	Chinese rural medicine	12	9	38	2005	sn.1006-5180.2005.09.049
1650	寒沙门菌药敏试验和临床	d clinical efficacy analysis	杨莉萍.	Yang Liping.	寄生虫病与感染性疾病	ic diseases and infectious d	3	4	179-180	2005	sn.1672-2116.2005.04.012
1651	4年深圳市食品污染状况	y on food contamination in	浩,李慧,黄薇,王舟,吴丽	Hui,Huang Wei,Wang Zhou	职业与健康	Occupation and health	21	9	1334-1336	2005	sn.1004-1257.2005.09.039
1652	利丹沙门菌食物中毒的	first case of Salmonella bul	袁珣,李铁墙,冯梅,李依建	Kun,Li Tieqiang,Feng Mei,Li	中国卫生检验杂志	ese Journal of Health Inspe	15	7	873-873	2005	sn.1004-8685.2005.07.053
1653	列腹泻患者粪便培养结果	cal culture results of patie	黄谦吉,上官敏.	uang Qianji,Shangguan Mi	井冈山医专学报	l of Jinggangshan Medical	12	2	55-55	2005	-
1654	氏菌食物中毒致多器官功	ning causes changes in mu	崔洁,祁兰,马英.	Cui Jie,Qilan,Ma Ying.	新疆医学	Xinjiang Medicine	35	6	114-115	2005	sn.1001-5183.2005.06.077
1655	浮市城市污水中沙门菌调	almonella in Urban Wastew	黄明安,冯文悦,陈伟文.	ing'an,Feng Wenyue,Chen	环境与健康杂志	nal of Environment and He	22	3	194-195	2005	sn.1001-5914.2005.03.012
1656	城区居民主要腹泻病原菌	iarrhea pathogenic bacteria	谭海芳,钟健辉,梁国明.	fang,Zhong Jianhui,Liang G	中华临床医学研究杂志	Journal of Clinical Medical	11	14	-	2005	-
1657	株丙型副伤寒杆菌耐药性	of drug resistance of Paraty	李烽,李虹,梁静.	Li Feng,Li Hong,Liang Jing.	现代医药卫生	modern medicine and healt	21	11	1340	2005	sn.1009-5519.2005.11.016
1658	沙门氏菌感染血培养耐药	ection in blood culture of S	-	-	遵义医学院学报	urnal of Zunyi Medical Colle	28	4	365-365	2005	-
1659	沙门菌食物中毒的微生	tion of a case of Salmonell	林凤,谭海芳.	Lin Feng,Tan Haifang.	中国卫生检验杂志	ese Journal of Health Inspe	15	12	1512-1513	2005	sn.1004-8685.2005.12.053
1660	传染性疾病爆发的流行	nfectious disease outbreak	巴剑波.	Ba Jianbo.	疾病控制杂志	journal of disease control	9	5	380-383	2005	sn.1674-3679.2005.05.004
1661	寒沙门氏菌引起食物中毒	e of food poisoning caused	贺为民.	He Weimin.	中华卫生监督与健康	se Health Supervision and	4	10	53-54	2005	-
1662	～2004年细菌性食物中毒	f pathogenic bacteria of b	群,林一曼,张倩,朱高云,黄	nan,Zhang Qian,Zhu Gaoyu	中国热带医学	Chinese tropical medicine	5	3	569-570	2005	sn.1009-9727.2005.03.093
1663	沙门菌株的脉冲电场凝胶	el electrophoresis typing o	春光,王琳娜,何宏明,刘	uang,Wang Linna,He Hong	学（流行病学.传染病学	(Epidemiology.Infectious	32	3	140-142	2005	sn.1673-4149.2005.03.004

1664	省食品中食源性致病菌污染	ogenic bacteria contamina	珽,罗山,杨庆文,周慧新,吕	an,Yang Qingwen,Zhou Hu	中国卫生检验杂志	ese Journal of Health Inspe	15	7	846-848	2005	sn.1004-8685.2005.07.038
1665	年伤寒沙门菌噬菌体分型	yping and drug resistance a	吾福尔,刘清,马建民,刘敏	Liu Qing,Ma Jianmin,Liu Mi	地方病通报	ndemic disease notificatio	20	4	58-59	2005	sn.1000-3711.2005.04.027
1666	教育学院食堂食物中毒调	poisoning in the cafeteria c	陈杰,谢会业.	Chen Jie,Xie Huiye.	淮海医药	Huaihai Pharmaceutical	23	4	287	2005	sn.1008-7044.2005.04.018
1667	分离菌株及其抗生素敏感性	rom pediatric blood and th	赖晓全,王洪波.	ai Xiaoquan,Wang Hongbo	中国妇幼保健	ina maternal and child hea	20	20	2711-2713	2005	sn.1001-4411.2005.20.070
1668	高校大学生伤寒发病情况分	phoid fever among college	梁晓红.	Liang Xiaohong.	山西医药杂志	Shanxi Medical Journal	34	12	1085-1086	2005	sn.0253-9926.2005.12.064
1669	床常见沙门菌及其耐药性分	n clinical Salmonella and th	毛文祥,刘玲珑.	Mao Wenxiang,Liu Linglong	海峡预防医学杂志	s Journal of Preventive Me	11	3	77-78	2005	sn.1007-2705.2005.03.054
1670	饮用水污染引起食物中毒的	food poisoning caused by c	黎明强,秦景新,周磊.	ing Qiang,Qin Jingxin,Lei Zh	现代预防医学	modern preventive medicin	32	3	277-277	2005	sn.1003-8507.2005.03.044
1671	对六种氟喹诺酮类药物的敏	sting of Salmonella to six fl	魏秀丽,陈杖榴.	Wei Xiuli,Chen Zhangliu.	中国兽药杂志	ese Journal of Veterinary D	39	9	4-8	2005	sn.1002-1280.2005.09.002
1672	氏菌中超广谱β-内酰胺酶的	n-ultra-broad spectrumβ-La	曹春红.	Cao Chunhong.	中国药师	Chinese pharmacist	8	1	39-40	2005	n.1008-049X.2005.01.016
1673	州区7类食品中致病菌污染	the contamination status	兰荣,常欣,刘桂玲,迟复平,	rong,Chang Xin,Liu Guiling,	中国公共卫生管理	ese public health managem	21	6	508-510	2005	sn.1001-9561.2005.06.041
1674	系对肉鸡生产中沙门氏菌的	ct of the system on Salmon	张苏华,王建,齐新勇,沈莉	hua,Wang Jian,Qi Xinyong,	动物医学进展	vances in Veterinary Medic	26	4	61-63	2005	sn.1007-5038.2005.04.018
1675	门菌污染卤制品引起的食物	isoning caused by salmone	张以春,唐娟,朱伟光.	Yichun,Tang Juan,Zhu Wei,	江苏预防医学	iangsu Preventive Medicin	16	4	84-84	2005	sn.1006-9070.2005.04.051
1676	沙门菌食物中毒的调查与	eatment of a case of salmo	黄峰,刘思美,曹举生.	ng Feng,Liu Simei,Cao Jush	中国预防医学杂志	se Journal of Preventive Me	6	6	548	2005	sn.1009-6639.2005.06.035
1677	门菌分离培养基的应用和	comparison of Salmonella	河,杨兰萍,陈纯,席曼芳,胡	Lanping,Chen Chun,Xi Ma	中国卫生检验杂志	ese Journal of Health Inspe	15	7	773-775,778	2005	sn.1004-8685.2005.07.002
1678	伤寒沙门氏菌食物中毒的调	e of food poisoning caused	秀春,张建军,刘立元,程国	Zhang Jianjun,Liu Liyuan,C	中国城乡企业卫生	ygiene in urban and rural a	-	6	17	2005	sn.1003-5052.2005.06.010
1679	门氏菌引起的食物中毒调	n a case of food poisoning	余新天,张素丽,张保云.	ntian,Zhang Suli,Zhang Bao	中国现代临床医学	nese modern clinical medi	4	12	-	2005	-
1680	山区蛇类沙门菌菌型分布	Salmonella strains in snake	隋达伟,王春生,辛生.	awei,Wang Chunsheng,Xins	中国医学检验杂志	urnal of Medical Laborator	6	2	137-137	2005	-
1681	寒误诊为急性阑尾炎11例	agnosed as acute appendici	邓志海.	Deng Zhihai.	临床误诊误治	l misdiagnosis and mistrea	18	7	511-511	2005	sn.1002-3429.2005.07.059
1682	时由两种病原菌引起的食	ed by two pathogenic bact	程慰先.	Cheng Weixian.	河南预防医学杂志	n Journal of Preventive Me	16	1	51-51	2005	sn.1006-8414.2005.01.023
1683	生尿中分离出一株粘液型沙	ucoid Salmonella strain fro	何祖光.	He Zuguang.	江西医学检验	Jiangxi Medical Laboratory	23	2	116-116	2005	sn.1674-1129.2005.02.058
1684	分泌物中分离出基桑加尼沙	sangani isolated from wou	周美容,李慧冰.	Zhou Meimei,Li Huibing.	中华检验医学杂志	e Journal of Laboratory Me	28	8	773	2005	sn:1009-9158.2005.08.037
1685	猪霍乱沙门菌败血症误诊1	holeraesuis Salmonella Sep	王波,董明.	Wang Bo,Dong Ming.	中国医药卫生	hinese medicine and healt	6	14	-	2005	-
1686	奶牛乳腺炎调查及病原菌分	nd isolation and identificat	玉君,周建胜,马慧玲,王安	ou Jiansheng,Ma Huiling,W	食品与药品A	Food and DrugsA	7	9	53-55	2005	n.1672-979X.2005.09.020
1687	部分食品中3种致病菌污染	ion into the contamination	长青风,杜俊甫,李林奇,张豪	ingfeng,Du Junfu,Li Lynch,	中国食品卫生杂志	hinese Food Hygiene Journ	17	1	54-56	2005	sn.1004-8456.2005.01.019
1688	用皮蛋引起的食物中毒事	soning incident caused by e	俞曼华,毛寅森.	Yu Manhua,Mao Yinsen.	中国农村卫生事业管理	na's rural health managem	25	4	56-57	2005	sn.1005-5916.2005.04.026
1689	血症病原菌的类型及其耐药	g resistance of pathogenic	刘华,喻华,周中华,黄湘宁	Hua,Zhou Zhonghua,Huang	世界感染杂志	world journal of infections	5	3	233-234,237	2005	-
1690	市一起学生食源性疾病调查	of foodborne diseases amo	何洁仪,马林,蔡衍珊,刘维斯	eyi,Marin,Cai Yanshan,Lew	热带医学杂志	journal of tropical medicine	5	3	348-349	2005	sn.1672-3619.2005.03.033
1691	检出乙型副伤寒沙门氏杆	e b detected in cerebrospi	徐翠英,王汉青.	Xu Cuiying,Wang Hanqing.	泰山卫生	Taishan Health	29	1	46	2005	-
1692	鼠伤寒沙门菌耐药株引起医	murium-resistant strains ca	段英梅.	Duan Yingmei.	中华医院感染学杂志	ournal of Hospital Infectiou	15	7	750-750	2005	sn:1005-4529.2005.07.051
1693	沙门氏菌性食源性疾病情	monella foodborne disease	彩虹,林波,李罗少,莫韵韶,陈	g,Limbo,Li Luoshao,Mo Yu	实用预防医学	ractical preventive medicin	12	4	855-856	2005	sn.1006-3110.2005.04.057
1694	鼠伤寒沙门氏菌引起的食	isoning caused by Salmone	杨小玲,郝建荣.	Yang Xiaoling,Hao Jianrong	中华卫生监督与健康	se Health Supervision and	4	3	47-47	2005	-
1695	镇海区1985~2003年伤寒疫	y1985~2003Analysis of an	胡依红,范善康,董选军.	ong,Fan Shankang,Dong Xu	中国公共卫生管理	ese public health managem	-	5	406-407	2005	-
1696	肠炎沙门菌引起食物中毒	food poisoning caused by	振宏,张震文,肖惠贞,谭雪	hang Zhenwen,Xiao Huizhe	中国热带医学	Chinese tropical medicine	5	4	823-824	2005	sn.1009-9727.2005.04.092
1697	都柏林沙门菌引起的食物	isoning caused by Salmone	刘钢.	Liu Gang.	预防医学论坛	Preventive Medicine Forum	11	4	487-488	2005	sn.1672-9153.2005.04.074
1698	区服务业从业人员传染病	ous diseases among servic	,孟瑞琳,罗玉兰,谢雪妹,许	g Ruilin,Luo Yulan,Xie Xuer	华南预防医学	uth China Preventive Medi	31	3	22-24	2005	sn.1671-5039.2005.03.008
1699	门菌引起食物中毒的实验室	ysis of a case of food poiso	,吴振宏,谭雪芳,廖鹏飞,肖	Zhenhong,Tan Xuefang,Lia	中国热带医学	Chinese tropical medicine	5	3	602-602	2005	sn.1009-9727.2005.03.113
1700	福州市儿童腹泻病原菌分布	ic bacteria of children's dia	富,郭维植,黄心宏,徐海滨,	uo Weizhi,Huang Xinhong,	海南医学	Hainan Medicine	16	11	120-121	2005	sn.1003-6350.2005.11.082

1701	产品及肉制品中沙门菌的	nonella in Imported Aquati	张宏伟,赵宏,姚霞,侯丽萍	ngwei,Zhao Hong,Yao Xia,H	中国食品卫生杂志	hinese Food Hygiene Journ	17	2	121-122	2005	sn.1004-8456.2005.02.009
1702	食源性疾病微生物学检验	crobiological Test Results C	盛维立,汪道发,秦阿敏.	g Weili,Wang Daofa,Qin A	疾病监测	disease surveillance	20	4	187-188	2005	sn.1003-9961.2005.04.009
1703	份血液细菌培养及耐药性	terial culture and drug resi	李小平,谢建新,毛福青.	aoping,Xie Jianxin,Mao Fu	医学临床研究	medical clinical research	22	1	80-82	2005	sn.1671-7171.2005.01.031
1704	区血样70株甲型副伤寒沙	in Shandong70Analysis of	武文青,厉伟彩,肖霞,马振勇	ing,Li Weicai,Xiao Xia,Ma Z	实用医药杂志	ournal of Practical Medicin	22	4	348-349	2005	sn.1671-4008.2005.04.049
1705	床标本的细菌培养结果和	s and drug resistance anal	繁林,储从家,管新龙,罗次	u Congjia,Guan Xinlong,Ra	中国微生态学杂志	inese Journal of Microecolo	17	1	61-62,64	2005	n.1005-376X.2005.01.025
1706	垦区奶牛乳房炎致病菌的	eria of mastitis in dairy cov	进,陈创夫,滕文军,吴洁,任	Chuangfu,Teng Wenjun,W	中国草食动物	Chinese herbivores	25	2	49-52	2005	sn.2095-3887.2005.02.022
1707	株甲型副伤寒沙门菌的分	on molecular typing of Sal	菊莲,程苏云,许锋华,陆群	eng Suyun,Xu Fenghua,Lu	疾病监测	disease surveillance	20	11	565-567	2005	sn.1003-9961.2005.11.004
1708	起沙门菌引起的食物中毒	case of food poisoning ca	李昕.	Li Xin.	中国公共卫生	Chinese public health	21	5	586-586	2005	sn:1001-0580.2005.05.081
1709	门菌感染致婴幼儿腹泻、	s diarrhea and convulsions	刘敏,李腾海.	Liu Min,Li Tenghai.	中华现代中西医杂志	of Modern Chinese and W	3	4	-	2005	-
1710	物携带沙门菌监测结果分	oring results of Salmonella	李红星,王晓梅,马莉.	Hongxing,Wang Xiaomei,Ma	中国卫生检验杂志	ese Journal of Health Inspe	15	5	581-582	2005	sn.1004-8685.2005.05.037
1711	丙型副伤寒的分离与鉴定	and identification of parat	王丽芳,陈飞,姚永华,戴先祥	ang,Chen Fei,Yao Yonghua,	畜牧兽医科技信息	d Veterinary Science and Te	-	8	64-65	2005	sn.1671-6027.2005.08.045
1712	学甲型副伤寒暴发的流行	ses of paratyphoid A outbr	云,廖和壮,张咸奇,玉明科	ezhuang,Zhang Xianqi,Yu M	实用预防医学	ractical preventive medicin	12	1	98-100	2005	sn.1006-3110.2005.01.037
1713	寒沙门菌的扩增片段长度	ength polymorphism of Sal	坚,刘丹红,王敏,万强,王	hhong,Wang Min,Wan Qia	中国卫生检验杂志	ese Journal of Health Inspe	15	10	1194-1196	2005	sn.1004-8685.2005.10.017
1714	科医院感染鼠伤寒沙门菌	Typhimurium in Pediatric	韩学贞.	Han Xuezhen.	中国医药卫生	Chinese medicine and healt	6	5	-	2005	-
1715	屠宰后沙门氏菌的实验室	esting of Salmonella after	陶思源,徐昕.	Tao Siyuan,Xu Xin.	畜牧与兽医	Husbandry and Veterinary	37	5	34-36	2005	sn.0529-5130.2005.05.015
1716	源性沙门氏菌药敏试验结	ptibility test results of food	-	-	广西预防医学	Guangxi Preventive Medicin	11	5	319-319	2005	-
1717	起沙门氏菌食物中毒的调	into a case of salmonella fo	祖洪明.	Zu Hongming.	中华卫生监督与健康	se Health Supervision and	4	4	53-53	2005	-
1718	峰县都柏林沙门氏菌引起	y Salmonella Dublin in Xife	松,陈梅,白敏,曹薇,王天	n Mei,Bai Min,Cao Wei,Wa	中国西部科技	rn China Science and Tech	-	9	48	2005	sn.1671-6396.2005.09.033
1719	西食物中毒流行病学分析	ysis and preventive measu	-	-	广西预防医学	Guangxi Preventive Medicin	11	5	284-286	2005	-
1720	例健康体检大便培养结果	ol Culture Results of Healt	许素芬,励余康.	Xu Sufen,Li Yukang.	中国卫生检验杂志	ese Journal of Health Inspe	15	1	120-120	2005	sn.1004-8685.2005.01.070
1721	05年首例副伤寒患者流行	vestigation of the first pati	孟慧,何茂祥.	Meng Hui,He Maoxiang.	中华医学与健康	Chinese medicine and healt	2	4	110	2005	-
1722	型副伤寒沙门菌暴发的实	tion of an outbreak of Saln	缪蔚蔚.	Miao Weiwei.	中国卫生检验杂志	ese Journal of Health Inspe	15	2	234-234	2005	sn.1004-8685.2005.02.059
1723	000～2004年沙门氏菌菌	~2004Distribution of Salm	余玮.	Yu Wei.	现代预防医学	modern preventive medicin	32	11	1487-1488	2005	sn.1003-8507.2005.11.032
1724	共场所从业人员肠道致病	genic bacteria among work	曼,邱亚群,张倩,黄新凤	aqun,Zhang Qian,Huang Xir	中国热带医学	Chinese tropical medicine	5	1	124-125	2005	sn.1009-9727.2005.01.065
1725	中粪大肠菌群和肠道致病	ns and enteric pathogenic	杨赟,封少龙,陈淑兰.	Yun,Feng Shaolong,Chen S	美国中华临床医学杂志	Journal of Chinese Clinical	7	4	327-327,330	2005	-
1726	猪仔猪腹泻的诊断及药敏	g sensitivity test of diarrhe	红艳,刘培琼,张启林,周思	n,Liu Peiqiong,Zhang Qilin	中国畜牧兽医	nal husbandry and veterina	32	3	48-50	2005	sn.1671-7236.2005.03.017
1727	售肉类大肠埃希菌与沙门	nd Salmonella contaminati	汤雪梅,丛柏林,魏锋,周	uemei,Cong Berlin,Wei Fen	中国公共卫生	Chinese public health	21	10	1233-1234	2005	sn:1001-0580.2005.10.045
1728	例患儿血中分离出肠炎沙	itidis was isolated from the	沈菁.	Shen Jing.	广西预防医学	Guangxi Preventive Medicin	11	3	174	2005	n.1673-758X.2005.03.031
1729	5种食源性致病菌监测结	nitoring results of foodbor	政安,顾宝柯,许学斌,何	eng'an,Gu Baoke,Xu Xuebi	上海预防医学	hanghai Preventive Medicir	17	6	278-279	2005	sn.1004-9231.2005.06.016
1730	垦区奶牛乳房炎致病菌的	eria of mastitis in dairy cov	进,陈创夫,滕文军,吴洁,任	Chuangfu,Teng Wenjun,W	家畜生态学报	Journal of Livestock Ecology	26	4	91-95	2005	sn.1673-1182.2005.04.023
1731	沙门菌引起食物中毒的检	f a case of food poisoning	郑敏,武晓军,周雪妹.	g Min,Wu Xiaojun,Zhou Xu	职业与健康	Occupation and health	21	2	235-236	2005	sn.1004-1257.2005.02.053
1732	与MPN法定量检测禽肉和	parison of quantitative dete	王晓英,余东敏,刘秀梅.	Xiaoying,Yu Dongmin,Liu X	中国食品卫生杂志	hinese Food Hygiene Journ	17	2	106-108	2005	sn.1004-8456.2005.02.004
1733	及脑脊液中同时分离出伤	sly isolated from the blood	刘彩霞,杨锦红,李方去.	aixia,Yang Jinhong,Li Fang	中华检验医学杂志	e Journal of Laboratory Me	28	7	709	2005	sn:1009-9158.2005.07.035
1734	特殊群体沙门菌调查分析	nd analysis of special group	志红,张文俊,钟淙,彭国华	Zhang Wenjun,Zhong Cong	江西医学检验	Jiangxi Medical Laboratory	23	6	535-536	2005	sn.1674-1129.2005.06.017
1735	饮用水源污染引起腹泻爆	diarrhea caused by contan	叶宝英,曾惠芳,张然,扈庆	Zeng Huifang,Zhang Ran,H	中国热带医学	Chinese tropical medicine	5	2	368-369	2005	sn.1009-9727.2005.02.092
1736	4株沙门氏菌药敏试验分析	ug susceptibility test of Sa	黎雪英,陈伟文.	Li Xueying,Chen Weiwen.	现代医药卫生	modern medicine and healt	21	16	2221-2222	2005	sn.1009-5519.2005.16.141
1737	细菌性痢疾暴发的流行病	estigation of an outbreak o	-	-	宁夏医学院学报	rnal of Ningxia Medical Coll	27	5	411-412	2005	-

1738	区近十年检出的沙门菌型	ypes detected in Haizhu Dis	孙凤琪.	Sun Fengqi.	中华医学与健康	Chinese medicine and healt	2	4	73-74	2005	-
1739	3年广西细菌性食物中毒	ogical analysis of bacterial	乐,黄林,黄兆勇,方志峰,	ng Lin,Huang Zhaoyong,Fa	中国食品卫生杂志	hinese Food Hygiene Journ	17	3	224-227	2005	sn.1004-8456.2005.03.005
1740	中单核细胞增生李斯特菌	atus of Listeria monocytoge	沈晓盛,蔡友琼.	hen Xiaosheng,Cai Youqion	中国卫生检验杂志	ese Journal of Health Inspe	15	1	85-86	2005	sn.1004-8685.2005.01.045
1741	生微生物检验质量控制考	iene microbiological inspec	秀丽,廖兴广,张蒙,高葆真	ao Xingguang,Zhang Meng,	中国卫生检验杂志	ese Journal of Health Inspe	15	7	856-857	2005	sn.1004-8685.2005.07.043
1742	夏地区71例伤寒临床分	71Clinical analysis of typho	张栩,石云玲.	Zhang Xu,Shi Yunling.	临床荟萃	clinical collection	20	2	87-88	2005	n.1004-583X.2005.02.015
1743	2003年食品公共场所从	nnual physical examinatio	曾维,田华伟.	Zeng Wei,Tian Huawei.	职业与健康	Occupation and health	21	2	228-229	2005	sn.1004-1257.2005.02.046
1744	菌、26株志贺菌的检出	,26Detection results and a	吕芝平,彭小峻,王艳.	hiping,Peng Xiaojun,Wang	中国卫生检验杂志	ese Journal of Health Inspe	15	12	1491	2005	sn.1004-8685.2005.12.040
1745	都柏林沙门菌引起的食物	d poisoning caused by Salm	建群,罗学辉,张怡明,何水	,Luo Xuehui,Zhang Yiming	中国卫生检验杂志	ese Journal of Health Inspe	15	9	1122-1122	2005	sn.1004-8685.2005.09.050
1746	菌引起新生儿病房感染的	atal ward infections cause	安礼,马国柱,连西兰,刘	Anli,Ma Guozhu,Lian Xilan,	中国卫生检验杂志	ese Journal of Health Inspe	15	7	879-879	2005	sn.1004-8685.2005.07.058
1747	省286起沙门菌食物中毒	ce286Analysis of salmonel	呈珠,薛良辉,张红,王晓	Xue Lianghui,Zhang Hong,W	中国食品卫生杂志	hinese Food Hygiene Journ	17	1	20-22	2005	sn.1004-8456.2005.01.006
1748	农贸市场鲜猪肉沙门氏菌	f fresh pork in farmers' ma	窦春香,李国福.	Dou Chunxiang,Li Guofu.	畜牧与饲料科学	Livestock and feed science	26	3	51	2005	sn.1672-5190.2005.03.023
1749	海洋贝类微生物调查及其	n of marine shellfish micro	盛,顾润润,于慧娟,李庆,黄	u Runrun,Yu Huijuan,Li Qin	海洋渔业	Marine fisheries	27	1	64-67	2005	sn.1004-2490.2005.01.012
1750	晚饮食摊点抹布的致病菌	n in rags from morning and	李剑,刘力健,毕延光.	i Jian,Liu Lijian,Bi Yanguang	医学动物防制	medical animal control	21	5	331-332	2005	sn.1003-6245.2005.05.010
1751	54株沙门菌菌型鉴定报告	monella strain identificatio	石惠清,曾宪启.	Shi Huiqing,Zeng Xianqi.	中国预防医学杂志	se Journal of Preventive Me	6	6	539-540	2005	sn.1009-6639.2005.06.029
1752	寒并发急性肾功能衰竭7例	cated by acute renal failure	蔡丽敏,颜鸣鹤.	Cai Limin,Yan Minghe.	实用医学杂志	ournal of Practical Medicin	21	19	2176-2178	2005	sn.1006-5725.2005.19.032
1753	区2003年第四季度食品卫	alysis of food hygiene testi	凤良,刘红英,蒋臣英,李双	u Hongying,Jiang Chenying,	中华卫生监督与健康	se Health Supervision and	4	12	47-48	2005	-
1754	鼠伤寒沙门氏菌的分离与	ification of Salmonella typh	新岗,黄银云,郭广富,金彩	Huang Yinyun,Guo Guangf	畜牧与兽医	Husbandry and Veterinary	37	11	44-45	2005	sn.0529-5130.2005.11.020
1755	炎沙门菌致伤口感染一例	infection caused by Salmo	陈燕,刘云庆,张云根.	Yan,Liu Yunqing,Zhang Yu	检验医学	laboratory medicine	20	4	312316	2005	sn.1673-8640.2005.04.039
1756	D群沙门菌引起的食物中	ion into food poisoning cau	赵哲,冒小鸥,凌剑.	Zhao Zhe,Mao Ou,Ling Jian	江苏预防医学	iangsu Preventive Medicin	16	3	39	2005	sn.1006-9070.2005.03.022
1757	8份血培养检出病原菌的	sults of pathogenic bacteri	陈群英.	Chen Qunying.	中国热带医学	Chinese tropical medicine	5	3	577-578	2005	sn.1009-9727.2005.03.098
1758	亚利桑那沙门菌腹膜炎1例	nella arizona peritonitis1ex	李显蓉,邓存良.	Li Xianrong,Deng Cunliang.	寄生虫病与感染性疾病	ic diseases and infectious d	3	2	82-82	2005	sn.1672-2116.2005.02.023
1759	门氏菌引起食物中毒的调	n a case of food poisoning	孙云峰.	Sun Yunfeng.	中华卫生监督与健康	se Health Supervision and	4	5	44-44	2005	-
1760	004年广西食源性致病菌	study on foodborne pathog	蒋震羚,吕素玲,黄兆勇,方	g,Lu Suling,Huang Zhaoyon	实用预防医学	ractical preventive medicin	12	6	1268-1270	2005	sn.1006-3110.2005.06.005
1761	门菌致老年患者急性泌尿	uses acute urinary tract inf	武国威,贾丽,李平.	Wu Guowei,Jia Li,Li Ping.	齐鲁医学检验	Qilu Medical Testing	16	1	61	2005	sn.1673-5013.2005.01.035
1762	妊娠合并伤寒35例临床分	cated by typhoid fever35Ca	秦秀妹.	Qin Xiumei.	华夏医学	Chinese Medicine	18	6	915-915	2005	sn.1008-2409.2005.06.071
1763	患病的现状与防治--沙门	prevention of zoonotic dise	李巨银,傅宏庆.	Li Juyin,Fu Hongqing.	吉林畜牧兽医	l Husbandry and Veterinar	-	8	54-55	2005	sn.1672-2078.2005.08.031
1764	481例腹泻患者病原学检测	en detection in patients wi	刘晓云,汤冬静,肖辉川.	yun,Tang Dongjing,Xiao Hu	预防医学论坛	Preventive Medicine Forum	11	4	469-470	2005	sn.1672-9153.2005.04.059
1765	食物中毒的流行病学调查	tiological analysis of colle	素娥,王冯彬,陈家福,余凤	Wang Fengbin,Chen Jiafu,Y	中国感染控制杂志	ese Journal of Infection Co	4	3	230-231	2005	sn.1671-9638.2005.03.012
1766	分泌物中检出一株肠炎沙	of Salmonella Enteritidis fr	张建英,汪定成.	ang Jianying,Wang Dingche	第四军医大学学报	f Fourth Military Medical U	26	12	1136-1136	2005	sn:1000-2790.2005.12.037
1767	儿沙门菌感染68例临床分	fection in children68Case c	叶飞跃,张莹莹.	Ye Feiyue,Zhang Yingying.	中国社区医师（综合版）	munity Physician (Compreh	-	12	47-48	2005	-
1768	门氏菌引起肠炎暴发流行	od poisoning caused by sa	石新伦.	Shi Xinlun.	中国现代临床医学	nese modern clinical medic	4	12	-	2005	-
1769	城区居民主要腹泻病原菌	arrhea pathogenic bacteria	谭海芳,钟健辉,梁国明.	fang,Zhong Jianhui,Liang G	国际医药卫生导报	ational Medical and Health	11	24	123-124	2005	sn.1007-1245.2005.24.070
1770	染性肠炎的病原菌分布及	pathogenic bacteria in inf	包东武,丁可,范泽旭.	o Dongwu,Ding Ke,Fan Zex	现代预防医学	modern preventive medicin	32	7	847-848	2005	sn.1003-8507.2005.07.077
1771	肿脓液中检出1株甲型副伤	ovarian cyst pus1Salmone	从家,吴惠玲,高晓玲,孔繁	,Wu Huiling,Gao Xiaoling,K	临床检验杂志	journal of clinical testing	23	5	346	2005	n.1001-764X.2005.05.050
1772	品沙门氏菌污染状况及防	mmercially available meat	洪文展.	Hong Wen Zhan.	肉类工业	meat industry	-	10	43-44	2005	sn.1008-5467.2005.10.019
1773	12年临床分离病原菌情况	e clinical isolation of path	巫远忠,陈国强,曹海燕.	nzhong,Chen Guoqiang,Cao	河北医药	Hebei Medicine	27	4	310-311	2005	sn.1002-7386.2005.04.049
1774	者骨髓和血液同时分离出	ne marrow and blood of p	桂云,多丽波,闫立昕,孙	uiyun,dolipo,Yan Lixin,Sun	世界华人消化杂志	ld Chinese Journal of Diges	13	18	2287	2005	sn.1009-3079.2005.18.029

1775	民甲型副伤寒沙门菌耐药	nce of Salmonella paratyph	应英.	Ying Ying.	江西医学检验	Jiangxi Medical Laboratory	23	2	179-179	2005	sn.1674-1129.2005.02.049
1776	一起沙门菌食物中毒的报	ort of salmonella food pois	姜良成.	Jiang Liangcheng.	中国社区医师（综合版）	unity Physician (Compreh	-	23	70	2005	-
1777	型副伤寒沙门菌抗药性调	drug resistance of Salmon	史煜波,楼燕如,董燕,李光	bo,Lou Yanru,Dong Yan,Li	浙江实用医学	Zhejiang Practical Medicine	10	5	361-362	2005	sn.1007-3299.2005.05.036
1778	巴氏杆菌和沙门氏菌的分	cation of Pasteurella multo	罗凤珍,张衍俊.	uo Fengzhen,Zhang Yanjun	山东畜牧兽医	mal Husbandry and Veteri	-	4	1-2	2005	sn.1007-1733.2005.04.001
1779	牛头肉引起沙门氏菌中毒	ella poisoning caused by e	吴有英.	Wu Youying.	青海医药杂志	Qinghai Medical Journal	35	10	14	2005	sn.1007-3795.2005.10.047
1780	计同时检出鼠伤寒沙门氏	eous detection of Salmonel	郑瑞侠.	Zheng Ruixia.	实用医技杂志	l of Practical Medical Tech	12	16	2296-2297	2005	sn.1671-5098.2005.16.143
1781	～2004年甲副伤寒菌株质	Analysis of plasmid map o	水荣,陈恩富,张政,叶菊	g,Chen Enfu,Zhang Zheng,	中国卫生检验杂志	ese Journal of Health Inspe	15	2	138-139	2005	sn.1004-8685.2005.02.004
1782	型副伤寒沙门氏菌的耐药	rug resistance of Salmonell	温小凤,马顺平,陈海莹.	aofeng,Ma Shunping,Chen	中日友好医院学报	of China-Japan Friendship	19	2	114-115	2005	sn.1001-0025.2005.02.019
1783	和沙门氏菌病病原的分离	y test of Escherichia coli ar	刘萍,胡世君,周秀富.	u Ping,Hu Shijun,Zhou Xiuf	畜禽业	vestock and poultry indust	-	7	40-42	2005	sn.1008-0414.2005.07.019
1784	脓液中分离1株猪霍乱沙	upper arm pus1Salmonella	王俊智,仲冬梅.	Vang Junzhi,midwinter plun	工企医刊	ial and Enterprise Medical	18	3	48	2005	n.1001-814X.2005.03.060
1785	氏菌的鉴定和分群及抗感	Salmonella in Wuhan and s	李奇志,孙自镛,叶嗣颖.	i Qizhi,Sun Ziyong,Ye Siying	热带医学杂志	ournal of tropical medicine	5	4	434-437	2005	sn.1672-3619.2005.04.007
1786	脓肿病人围手术期的观察	on and nursing care of a pa	罗艳丽,邬涛.	Luo Yanli,Wu Tao.	华西医学	West China Medicine	20	4	755-755	2005	sn.1002-0179.2005.04.100
1787	菌性食物中毒病原菌分布	ristics of pathogenic bacter	李少彤,蒋卓勤,曾德荣.	tong,Jiang Zhuoqin,Tsang	华南预防医学	uth China Preventive Medic	31	5	46-47,49	2005	sn.1671-5039.2005.05.017
1788	肠炎沙门菌所致原发性败	y Salmonella Enteritidis in	鲁姣英,吴立奇.	Lu Jiaoying,Wu Liqi.	中国预防医学杂志	se Journal of Preventive Me	6	3	261-262	2005	sn.1009-6639.2005.03.041
1789	人员沙门菌耐药性及质粒	characteristics of Salmonel	陈义忠,楚心唯,吴敏,吴爱	hong,Chu Xinwei,Wu Min,	中国公共卫生	Chinese public health	21	2	205-206	2005	sn:1001-0580.2005.02.042
1790	菌86例药敏结果及临床治	tivity results and clinical tre	陈秀苹,任苇.	Chen Xiuping,Ren Wei.	重庆医学	Chongqing Medicine	34	11	1711-1712	2005	sn.1671-8348.2005.11.062
1791	沙门氏菌病的分离及病菌	icks in Yili area and investi	苏战强,单文鲁,王治才.	nqiang,Shan Wenlu,Wang	畜禽业	vestock and poultry indust	-	12	14-15	2005	sn.1008-0414.2005.12.005
1792	型副伤寒沙门菌鉴定及药	susceptibility analysis of Sa	邹均.	Zou Jun.	黔南民族医专学报	f Qiannan National Medica	18	1	17-18	2005	sn.1008-4983.2005.01.006
1793	鸭沙门氏菌的分离与鉴定	and identification of Salmo	芳,王定发,阮征,宫时玉,	,Wang Dingfa,Ruan Zheng	上海畜牧兽医通讯	hal Husbandry and Veterina	-	6	20	2005	sn.1000-7725.2005.06.011
1794	由波茨坦沙门菌引起的食	poisoning caused by Salmo	文革,张雪白,林建宗,蔡顺	hang Xuebai,Lin Jianzong,	海峡预防医学杂志	s Journal of Preventive Me	11	6	45-46	2005	sn.1007-2705.2005.06.027
1795	霍乱沙门氏菌食物中毒调	case of choleraesuis and s	郎福伟.	Lang Fuwei.	中国饮食卫生与健康	inese food hygiene and hea	3	5	46-47	2005	-
1796	菌污染状况及金黄色葡萄	nic bacteria in raw milk and	晓勤,胡建平,周丽萍,马	in,Hu Jianping,Zhou Liping	江苏预防医学	iangsu Preventive Medicin	16	3	7-9	2005	sn.1006-9070.2005.03.003
1797	肠炎沙门氏菌引起的食物	poisoning caused by Salmo	蔡秀英,聂秀敏,杨守丽.	Xiuying,Nie Xiumin,Yang Sh	中华卫生监督与健康	se Health Supervision and	4	11	46-47	2005	-
1798	群沙门氏菌食物中毒的调	d analysis of food poisoning	-	-	中国公共卫生管理	ese public health managen	21	5	433	2005	-
1799	食堂供餐引发食物中毒事	d by food served in a kinde	林晔.	Lin Ye.	职业与健康	Occupation and health	21	9	1341-1342	2005	sn.1004-1257.2005.09.046
1800	中分离出1例鼠伤寒沙门	ts1Investigation report on	启,黄萍,代敏,黄成辉,郑	Ping,Dai Min,Huang Cheng	中国卫生检验杂志	ese Journal of Health Inspe	15	1	89-89	2005	sn.1004-8685.2005.01.048
1801	居民饮水污染现状及预防	asures of drinking water p	坚,蒲朝文,王兴明,何军,	haowen,Wang Xingming,H	职业与健康	Occupation and health	21	1	85-87	2005	sn.1004-1257.2005.01.069
1802	城区蜚蠊密度及其带菌状	es and Their Bacteria Carry	琴,汪宏卫,高亚玲,陈秋梅	g Hongwei,Gao Yaling,Che	中国媒介生物学及控制杂	ournal of Vector Biology ar	16	6	465-466	2005	sn.1003-4692.2005.06.021
1803	氏菌引起的食物中毒调查	ort on food poisoning caus	黄昌胜,赵鸣镝.	ang Changsheng,Zhao Ming	中国饮食卫生与健康	inese food hygiene and hea	3	2	43-44	2005	-
1804	～2004年食物中毒特征分	ual food poisoning charac	何慧芬,刘海东,高群威.	uifen,Liu Haidong,Gao Qur	实用预防医学	ractical preventive medicin	12	3	634-636	2005	sn.1006-3110.2005.03.089
1805	家庭聚餐引起食物中毒的	alysis of food poisoning cau	陈丽萍.	Chen Liping.	包头医学院学报	rnal of Baotou Medical Coll	21	1	24-25	2005	n.1006-740X.2005.01.012
1806	肉中沙门菌分离株多重耐	nce spectrum of Salmonell	郭云昌,刘秀梅.	Guo Yunchang,Liu Xiumei.	中国食品卫生杂志	hinese Food Hygiene Journ	17	2	100-103	2005	sn.1004-8456.2005.02.002
1807	门氏菌脉冲场凝胶电泳分	el electrophoresis typing o	赵熙,杨宝兰,李志刚,刘秀	i,Yang Baolan,Li Zhigang,Li	卫生研究	health research	34	3	338-340	2005	sn.1000-8020.2005.03.024
1808	起沙门氏菌食物中毒的调	into a case of salmonella f	张丽霞,马莉,雷莉.	Zhang Lixia,Ma Li,Raleigh.	中华卫生监督与健康	se Health Supervision and	4	7	51	2005	-
1809	部脓汁中分离1株猪霍乱沙	rom leg pus1Salmonella ch	杨丽丽.	Yang Lili.	临床检验杂志	journal of clinical testing	23	1	47-47	2005	n.1001-764X.2005.01.037
1810	儿童感染性腹泻病原学研	etiology of infectious diar	少平,刘学菊,全博,张海	ing,Liu Xueju,Full blog,Zhar	中国微生态学杂志	inese Journal of Microecol	17	5	352-353	2005	n.1005-376X.2005.05.017
1811	起细菌性食物中毒的调查	port on a case of bacterial	黄星文.	Huang Xingwen.	中国临床医药研究杂志	Journal of Clinical Medical	-	142	-	2005	-

1812	伤寒沙门氏菌引起的食物	f food poisoning caused by	李益斌,谭甲忠.	Li Yibin,Tan Jiazhong.	中国饮食卫生与健康	inese food hygiene and hea	3	4	52-53	2005	-
1813	群沙门氏菌食物中毒的调	analysis of food poisoning	王鲁溪,宋树川,杨韶宇.	Luxi,Song Shuchuan,Yang S	中国公共卫生管理	ese public health managen	-	5	433-433	2005	-
1814	养病原菌菌群分布及耐药	acterial flora distribution a	,陈超群,吴移谋,杨祚升,陈	aoqun,Wu Yimou,Yang Zuo	中国抗生素杂志	hinese Journal of Antibioti	30	7	407-411	2005	sn.1001-8689.2005.07.008
1815	熟肉食品中检出沙门菌的	tion of Salmonella in cooke	方敏,张春梅,周军波,朱焰	Zhang Chunmei,Zhou Junb	中国卫生检验杂志	ese Journal of Health Inspe	15	12	1523	2005	sn.1004-8685.2005.12.061
1816	门氏菌引起食物中毒的病	of food poisoning caused	苏华瑜,庞琼英.	Su Huayu,Pang Qiongying.	广东医学院学报	al of Guangdong Medical C	23	5	627-628	2005	sn.1005-4057.2005.05.079
1817	地区甲型副伤寒沙门菌的	tance of Salmonella paraty	繁林,储从家,管新龙,罗次	u Congjia,Guan Xinlong,Ra	中国临床药学杂志	ese Journal of Clinical Phar	14	3	182-184	2005	sn.1007-4406.2005.03.017
1818	食品沙门氏菌污染状况及	status and drug resistance	勤,李禾,胡建平,周丽萍,马	i He,Hu Jianping,Zhou Lipin	世界感染杂志	world journal of infections	5	2	102-104	2005	-
1819	环境及半成品中沙门氏菌	he environment of broiler	赵瑞兰,张培正,李远钊.	uilan,Zhang Peizheng,Li Yu	中国食物与营养	Chinese food and nutrition	-	9	32-34	2005	sn.1006-9577.2005.09.010
1820	沙门菌食源性疾病的调查	f Salmonella Enteritidis fo	李孝权,刘衡川,张健,李钏华	Liu Hengchuan,Zhang Jian,	现代预防医学	modern preventive medicin	32	7	751-753	2005	sn.1003-8507.2005.07.023
1821	品沙门氏菌污染状况及防	mmercially available meat	洪文展.	Hong Wen Zhan.	肉品卫生	Meat hygiene	-	4	25-26	2005	-
1822	门氏菌引起食物中毒的调	nt of a case of food poison	曾建军,郑要红,辛家辉.	ianjun,Zheng Yaohong,Xin	中华卫生监督与健康	se Health Supervision and	4	10	42-43	2005	-
1823	起多菌性食物中毒调查分	alysis of a case of multibac	刘登权,刘自远.	Liu Dengquan,Liu Ziyuan.	地方病通报	endemic disease notificatio	20	1	85-86	2005	sn.1000-3711.2005.01.033
1824	BLs细菌及其耐药性检测室	oratory quality evaluation	魏莲花,张俭,邹凤梅,刘刚	ua,Zhang Jian,Zou Fengmei	中国感染控制杂志	ese Journal of Infection Co	4	4	356-357,359	2005	sn.1671-9638.2005.04.022
1825	3年饮食服务行业从业人员	fectious disease indicators	,孟虹,张泓,张俟清,彭鹏,刘	g,Zhang Hong,Zhang Qiqin	中国健康教育	health education in china	21	1	28-30	2005	sn.1002-9982.2005.01.010
1826	次发现蟑螂及其孳生带菌	e in Shangqiu City and inve	锋,马巧云,杨红兵,陈忠超	aoyun,Yang Hongbing,Che	河南预防医学杂志	n Journal of Preventive Me	16	6	359-360	2005	sn.1006-8414.2005.06.020
1827	衢州市食品中食源性致病	dborne pathogenic bacteri	陈卫国,王文英,叶承华.	eiguo,Wang Wenying,Ye Ch	中国卫生检验杂志	ese Journal of Health Inspe	15	6	736-736,758	2005	sn.1004-8685.2005.06.048
1828	年桂林市区伤寒监测结果	oid fever surveillance result	斌,王鸣柳,赵日秀,李翠云	Wang Mingliu,Zhao Rixiu,Li	广西医科大学学报	al of Guangxi Medical Univ	22	5	808-810	2005	n.1005-930X.2005.05.096
1829	海珠区网吧微生物污染状	ation status of Internet caf	华,胡文静,梁玉潮,罗娜,许	u Wenjing,Liang Yuchao,Lo	中国卫生检验杂志	ese Journal of Health Inspe	15	7	844-845	2005	sn.1004-8685.2005.07.037
1830	甲型副伤寒沙门菌药敏试	susceptibility test results c	-	-	医学文选	Selected Medical Essays	24	5	697-698	2005	-
1831	一起甲型副伤寒暴发的调查	nto an outbreak of paraty	叶方友;林兴崇;林哲宾;	gyou;Lin Xingchong;Lin	预防医学情报杂志	f Preventive Medicine In	-	6	776	2005	-
1832	起学校伤寒暴发的调查报	n report on a school typh	秦丽忠;	Qin Lizhong;	疾病监测	disease surveillance	-	10	531+544	2005	-
1833	四中甲型副伤寒暴发调查	ratyphoid A Outbreak in	蓝岳云,张进	Lan Yueyun,Zhang Jin	疾病监测	disease surveillance	-	9	1	2005	-
1834	县岩瑞中学副伤寒暴发因素	atyphoid outbreak in Yan	林上丽,李少华,祝庆伟	angli,Li Shaohua,Zhu Qi	中国自然医学杂志	se Journal of Natural Me	-	3	2	2005	-
1835	一起甲型副伤寒暴发疫情	outbreak of paratyphoid	树坤,李顺祥,刘红雁,姚云	n,Li Shunxiang,Liu Hong	预防医学情报杂志	f Preventive Medicine In	-	4	478	2005	-
1836	农村中学水型伤寒暴发的	ak of waterborne typhoid	徐志成,谢起理,罗卫民	Zhicheng,Xie Qili,Luo We	预防医学论坛	reventive Medicine Foru	-	3	359-360	2005	-
1837	市一起甲型副伤寒暴发流	paratyphoid fever A in T	胡富宇,彭道法	Hu Fuyu,Peng Daofa	中国热带医学	Chinese tropical medicine	-	5	1005-1006	2005	-
1838	伤寒暴发流行病例筛查方法	thods for epidemic case	章跃炎	Zhang Yueyan	中国卫生检验杂志	se Journal of Health Insp	-	5	614-615	2005	-
1839	起伤寒暴发的流行病学调	vestigation of an outbre	罗建军,陈平,于顺明,任明i	Chen Ping,Yu Shunming,	预防医学情报杂志	f Preventive Medicine In	-	2	188-189	2005	-
1840	一起水型伤寒暴发调查	an outbreak of waterbo	陶旦	Tao Dan	预防医学情报杂志	f Preventive Medicine In	-	2	191-192	2005	-
1841	甲型副伤寒暴发流行调查	nd analysis of an outbre	庆福,王树坤,曾丽萍,钦绍	Wang Shukun,Zeng Lipi	中国热带医学	Chinese tropical medicine	-	1	77-133	2005	-
1842	某中学289例感染性腹泻暴	ar289Investigation into inf	宁,苏萍,龙兮,赵丽娜,王	u Ping,Long Xi,Zhao Lina,W	广西预防医学	Guangxi Preventive Medicin	12	2	89-90	2006	n.1673-758X.2006.02.009
1843	沙门氏菌引起食物中毒的	ase of food poisoning cause	潘榕,秦景新,罗东,覃进山	g,Qin Jingxin,Luodong,Qin	中国医学文摘（内科学）	Medical Abstracts (Internal	-	2	164-165	2006	-
1844	食品中沙门菌污染状况调	f Salmonella contaminatio	,王英豪,张淑红,关文英,E	ghao,Zhang Shuhong,Guar	中国卫生检验杂志	ese Journal of Health Inspe	16	3	332-332,375	2006	sn.1004-8685.2006.03.040
1845	都柏林沙门菌引起的食物	poisoning caused by Salm	万茂传.	Biography of Wan Mao.	浙江预防医学	hejiang Preventive Medicin	18	3	41-41	2006	sn.1007-0931.2006.03.026
1846	沙门氏菌引起的食物中毒	is of a case of food poisoni	廖少江,李红,陈美玲,米庆秀	ang,Li Hong,Chen Meiling,	基层医学论坛	Primary Medicine Forum	10	16	750-751	2006	sn.1672-1721.2006.16.065
1847	1～2005年食物中毒特点与	Characteristics and Count	宁,迟晓文,李建新,陈兴乐	Chi Xiaowen,Li Jianxin,Che	公共卫生与预防医学	Health and Preventive Me	17	4	30-33	2006	sn.1006-2483.2006.04.011
1848	学生胃肠传染病爆发的流行	reak of gastrointestinal inf	,肖奎光,黄建平,陈复才,黄	uang,Huang Jianping,Chen	中国热带医学	Chinese tropical medicine	6	11	1979-1980,1986	2006	sn.1009-9727.2006.11.027

1849	寒血培养取血量与阳性率之	volume and positive rate	强,李顺祥,姚云波,刘红雁	,Li Shunxiang,Yao Yunbo,L	中华检验医学杂志	e Journal of Laboratory Me	29	7	664	2006	sn:1009-9158.2006.07.034
1850	致病性沙门氏菌耐药基因的	genes of pathogenic Salmo	宁,余勇,李成忠,张东,羊云	u Yong,Li Chengzhong,Zha	畜牧兽医学报	mal Husbandry and Veterin	37	1	65-70	2006	sn:0366-6964.2006.01.013
1851	一起都柏林沙门菌食物中毒	almonella food poisoning i	万茂传.	Biography of Wan Mao.	中华预防医学杂志	e Journal of Preventive Me	40	2	100	2006	sn:0253-9624.2006.02.027
1852	市饮食服务从业人员携带沙	salmonella carried by cater	黄烈泓,韩世兰.	Huang Liehong,Han Shilan.	华中医学杂志	Huazhong Medical Journal	30	5	415417	2006	-
1853	寒、甲型副伤寒224例耐药	, paratyphoid A224Example	罗友昌.	Luo Youchang.	安徽医药	Anhui Medicine	10	5	367	2006	sn.1009-6469.2006.05.032
1854	非伤寒沙门氏菌的耐药性	investigation of non-typho	镛,申正义,陈如,朱旭慧,李	Shin Zhengyi,Chen Ru,Zhu	中国人兽共患病学报	Chinese Journal of Zoonose	22	1	92-93,91	2006	sn.1002-2694.2006.01.027
1855	加工流通环节沙门氏菌的	nella in pig slaughtering, pr	冬冬,黄金林,陶善华,焦新	g,Golden Forest,Tao Shanh	猪业科学	Swine Science	23	2	63-64	2006	sn.1673-5358.2006.02.023
1856	婴儿非伤寒沙门菌感染38例	almonella infection in infar	刘运启,吴振松.	Liu Yunqi,Wu Zhensong.	实用医药杂志	ournal of Practical Medicin	23	10	1160	2006	sn.1671-4008.2006.10.042
1857	临床分离菌菌群分布和耐药	istribution and drug resista	繁林,储从家,管新龙,罗次	u Congjia,Guan Xinlong,Ra	中国临床医学	Chinese clinical medicine	13	1	156-158	2006	sn.1008-6358.2006.01.068
1858	培养阳性结果分析和病原	d culture results and distrib	闫利娟.	Yan Lijuan.	南科技大学学报（医学版	rsity of Science and Techn	24	4	265-266	2006	n.1672-688X.2006.04.012
1859	区甲型副伤寒沙门菌的耐药	ance of Salmonella paraty	义,刘海云,袁嘉丽,钏艾莉,	Haiyun,Yuan Jiali,Ellie,Zhe	中国药物与临床	Chinese medicine and clinica	6	2	92-93	2006	sn.1671-2560.2006.02.003
1860	中沙门菌污染状况及耐药性	contamination status and	,梅玲玲,张严峻,张俊彦,汪	ing,Zhang Junjun,Zhang Ju	中国卫生检验杂志	ese Journal of Health Inspe	16	9	1103-1104	2006	sn.1004-8685.2006.09.039
1861	寒沙门氏菌致股骨颈骨髓炎	omyelitis caused by Salmon	欣志,范天凤,康少英,方绍	Tianfeng,Kang Shaoying,F	中国矫形外科杂志	hinese Journal of Orthopedi	14	11	879-880	2006	sn.1005-8478.2006.11.025
1862	食品5种致病菌污染状况的	monitoring of contaminatio	沈伟伟,阮琦,王敬,葛素君	eiwei,Ruan Qi,Wang Jing,G	中国卫生检验杂志	ese Journal of Health Inspe	16	12	1513-1514,1519	2006	sn.1004-8685.2006.12.053
1863	脉冲场凝胶电泳在肠炎沙门	pulsed field gel electrophor	,李燕俊,王玉平,崔生辉,汪	un,Wang Yuping,Cui Sheng	中华流行病学杂志	inese Journal of Epidemiolo	27	12	1065-1068	2006	sn:0254-6450.2006.12.015
1864	术检测熟肉制品中沙门氏菌	al detection of Salmonella	荣兰,杨玉英,潘树德,苏振	an,Yang Yuying,Pan Shude,	安徽农业科学	Anhui Agricultural Sciences	34	2	222226	2006	sn.0517-6611.2006.02.009
1865	229例血培养阳性结果分析	ysis of positive blood cultur	云祥,吴娅娅,黄友泽,金镒	iang,Wu Yaya,Huang Youze	中国微生态学杂志	inese Journal of Microecolo	18	2	135-136	2006	n.1005-376X.2006.02.023
1866	见的沙门菌所致食物中毒报	eport of food poisoning cau	王晓红,张素燕,潘黎正.	iaohong,Zhang Suyan,Pan	上海预防医学	hanghai Preventive Medicin	18	3	140-141	2006	sn.1004-9231.2006.03.020
1867	副伤寒沙门菌致脊柱骨髓炎	teomyelitis caused by Salm	薛文,刘林,王和平.	ue Wen,Liu Lin,Wang Hepin	中华创伤杂志	Chinese Journal of Trauma	22	8	640	2006	sn:1001-8050.2006.08.029
1868	一起食物中毒的调查报告	on report on a case of food	王宪章.	Wang Xianzhang.	现代预防医学	modern preventive medicin	33	4	585	2006	sn.1003-8507.2006.04.062
1869	人员带菌致学生肠道传染	diseases among students ca	黄纯建,何冰.	Huang Chunjian,He Bing.	中国学校卫生	Chinese school health	27	4	351-351	2006	sn.1000-9817.2006.04.045
1870	年广西食品中沙门氏菌的	ng and analysis of Salmone	吕素玲,唐振柱,李秀桂,王	ng,Tang Zhenzhu,Li Xiugui,	实用预防医学	ractical preventive medicin	13	5	1262-1264	2006	sn.1006-3110.2006.05.070
1871	流通过程中主要食源性病原	pathogenic bacteria during	何蕊,许海燕,苏洁,潘志明,	Rui,Xu Haiyan,Su Jie,Pan Z	中国食品学报	urnal of Chinese Food Scien	6	6	121-125	2006	sn.1009-7848.2006.06.025
1872	品中食源性致病菌污染状况	orne pathogenic bacteria co	沈月华,吴晓芳,程平庆.	ehua,Wu Xiaofang,Cheng P	实用预防医学	ractical preventive medicin	13	1	146-147	2006	sn.1006-3110.2006.01.068
1873	脓液中检出甲型副伤寒沙	yphi A detected in ovarian	赵德军,张碧霞.	Zhao Dejun,Zhang Bixia.	西南军医	outhwest Military Medicin	8	5	43	2006	sn.1672-7193.2006.05.078
1874	455例儿童血培养结果分析	sis of blood culture results	刘文娟,曹春梅,周樱,钱厚明	Cao Chunmei,Zhou Ying,Qi	海南医学	Hainan Medicine	17	10	142-143	2006	sn.1003-6350.2006.10.095
1875	伤寒、副伤寒79例临床分析	paratyphoid fever79Case c	陈茂伟,吴健林,江建宁.	Maowei,Wu Jianlin,Jiang Jia	热带医学杂志	Journal of tropical medicine	6	5	570-572	2006	sn.1672-3619.2006.05.033
1876	利丹沙门菌引起食物中毒	e of food poisoning caused	刘汉伟.	Liu Hanwei.	职业与健康	Occupation and health	22	2	83-83	2006	sn.1004-1257.2006.02.020
1877	阳市伤寒流行因素调查及控	of epidemic factors of typh	国,石作宏,陈梅,罗月雪,蒋	uohong,Chen Mei,Luo Yuex	现代预防医学	modern preventive medicin	33	5	814-816	2006	sn.1003-8507.2006.05.068
1878	市食品病原微生物污染的	thogenic microbial contam	芳,刘涛,贺连华,石晓路,王	iu Tao,He Lianhua,Shi Xiao	中国热带医学	Chinese tropical medicine	6	3	525-526	2006	sn.1009-9727.2006.03.084
1879	都柏林沙门菌引起的食物	d poisoning caused by Salm	万茂传.	Biography of Wan Mao.	海峡预防医学杂志	s Journal of Preventive Me	12	1	42-43	2006	sn.1007-2705.2006.01.023
1880	哥纳沙门氏菌引起食物中毒	se of food poisoning cause	韩丽英.	Han Liying.	现代预防医学	modern preventive medicin	33	1	98-98	2006	sn.1003-8507.2006.01.046
1881	山市春季婴幼儿腹泻病因分	among infants and young	,裴银辉,陈艳,俞合韦,武英	inhui,Chen Yan,Yu Hewei,W	山西医药杂志	Shanxi Medical Journal	35	4	312-313	2006	sn.0253-9926.2006.04.012
1882	衢州市市售食品食源性致	he pathogenic bacteria in co	金莞尔,陈卫国,张建民.	n'er,Chen Weiguo,Zhang Ji	中国卫生检验杂志	ese Journal of Health Inspe	16	3	335-336	2006	sn.1004-8685.2006.03.042
1883	疾病的病原学研究及主要	s in broiler breeders and th	秋艳,胡晓娜,毕建敏,刘红	yan,Hu Xiaona,Bi Jianmin,Li	农业生物技术学报	nal of Agricultural Biotechn	14	5	646-651	2006	sn.1674-7968.2006.05.002
1884	伤寒沙门菌肠炎临床及耐药	ug resistance status of non	赵二强,胡景玉,赵红丽.	Erqiang,Hu Jingyu,Zhao Ho	医疗装备	medical equipment	19	1	42-43	2006	sn.1002-2376.2006.01.022
1885	伤寒性胆囊炎合并胆囊结石	itis complicated with gallbl	群龙,叶建宇,刘晓平,蔡志	ng,Ye Jianyu,Liu Xiaoping,Ca	罕少疾病杂志	Journal of Rare Diseases	13	5	52-53	2006	sn.1009-3257.2006.05.025

1886	门氏菌引起食物中毒的调	is of a case of food poisoni	郑伟,曹志强.	Zheng Wei,Cao Zhiqiang.	右江民族医学院学报	ujiang Medical College for	28	3	433-433	2006	sn.1001-5817.2006.03.067
1887	割产品生产过程中的沙门	e Production of Fresh Froz	李焰,许正金.	Li Yan,Xu Zhengjin.	养禽与禽病防治	and poultry disease preven	-	3	39-40	2006	-
1888	电泳技术应用于沙门菌鉴	gel electrophoresis techn	曹春红,刘维华.	Cao Chunhong,Liu Weihua.	现代检验医学杂志	l of Modern Laboratory Me	21	3	40-42	2006	sn.1671-7414.2006.03.014
1889	2005年市售食品中食源性	orne pathogenic bacteria c	黄家兵,汪艳平,司徒谊萍,	Jiabing,Wang Yanping,Situ	中国卫生检验杂志	ese Journal of Health Inspe	16	8	964-965	2006	sn.1004-8685.2006.08.034
1890	肠炎沙门菌引起的食物中毒	ood poisoning caused by S	周红芳,徐枫,郦佳莹.	u Hongfang,Xu Feng,Li Jiay	上海预防医学	hanghai Preventive Medici	18	11	548-549	2006	sn.1004-9231.2006.11.008
1891	炎沙门氏菌食物中毒的检	of a Case of Salmonella En	唐石伏,张海英,潘群慧,邹碧	u,Zhang Haiying,Pan Qunh	广西预防医学	Guangxi Preventive Medicin	12	2	124-124	2006	n.1673-758X.2006.02.027
1892	炎沙门菌致化脓性关节炎	caused by Salmonella Ente	利宁,邵海枫,王卫萍,张小	o Haifeng,Wang Weiping,Z	中国感染与化疗杂志	urnal of Infection and Che	6	5	344-344	2006	sn:1009-7708.2006.05.015
1893	猪霍乱沙门菌引起的食物	oisoning caused by Salmon	胡景生,李玉珍.	Hu Jingsheng,Li Yuzhen.	海峡预防医学杂志	s Journal of Preventive Me	12	6	9,17	2006	sn.1007-2705.2006.06.032
1894	鼠伤寒沙门菌食物中毒调	a case of Salmonella typhi	李继业.	Li Jiye.	中国卫生检验杂志	ese Journal of Health Inspe	16	6	757-757	2006	sn.1004-8685.2006.06.065
1895	河粉引起的沙门菌食物中	of salmonella food poisoni	文,麦充志,许桂锋,孙立梅,	Mai Chongzhi,Xu Guifeng,S	中国热带医学	Chinese tropical medicine	6	12	2252-2253	2006	sn.1009-9727.2006.12.087
1896	2004年衢州市食源性致病	of foodborne pathogenic	建民,金莞尔,王文英,于丽	anmin,Jin Wan'er,Wang W	卫生研究	health research	35	5	627-628	2006	sn.1000-8020.2006.05.038
1897	工食品-鹅肝中沙门氏菌分	Isolation and identification	盘宝进,黄利平.	Pan Baojin,Huang Liping.	现代食品科技	Modern food technology	22	2	223-224	2006	sn.1673-9078.2006.02.078
1898	工地沙门氏菌食物中毒的	s of salmonella food poiso	李吉兰.	Li Jilan.	中国卫生监督杂志	se Journal of Health Super	13	5	354-355	2006	sn.1077-6131.2006.05.010
1899	胆汁中检出阿哥纳沙门菌	f Salmonella agona from bi	-	-	医学理论与实践	Medical theory and practice	19	7	769-769	2006	-
1900	～2004年医疗机构消毒监	annual disinfection monit	王洁,陈嘉琳,何健.	Wang Jie,Chen Jialin,He Jian	中国热带医学	Chinese tropical medicine	6	4	695-696	2006	sn.1009-9727.2006.04.082
1901	4年食物中毒流行病学分析	analysis of food poisoning	蒋传舟.	Jiang Chuanzhou.	海峡预防医学杂志	s Journal of Preventive Me	12	3	37-38	2006	sn.1007-2705.2006.03.018
1902	市城区卤菜卫生状况的调	e hygienic status of stewed	钱惠芬.	Qian Huifen.	职业与健康	Occupation and health	22	3	201-202	2006	sn.1004-1257.2006.03.026
1903	气单胞与肠炎沙门菌致腹膜	monas hydrophila and Salm	颜伟文,王红梅.	an Weiwen,Wang Hongme	中华现代医学与临床	ern Chinese Medicine and	-	7	-	2006	-
1904	并发肠道鼠伤寒沙门氏菌	testinal Salmonella typhim	李小侠,张晓霞,梁远娣.	oxia,Zhang Xiaoxia,Liang Yu	护理学杂志	Journal of Nursing	21	18	72-73	2006	sn.1001-4152.2006.18.041
1905	门菌食物中毒31例临床分	food poisoning31Case clini	阮,金萍,陆必森,许锦姬,罗	Jin Ping,Lu Bisen,Xu Jinji,L	中国儿童保健杂志	ese Journal of Child Health	14	6	642-643	2006	sn.1008-6579.2006.06.047
1906	省食品中沙门氏菌的耐药	istance of Salmonella in fo	志,申志新,张淑红,王英豪,俨	hixin,Zhang Shuhong,Wang	现代预防医学	modern preventive medici	33	10	1761-1763	2006	sn.1003-8507.2006.10.007
1907	甲鲁木沙门菌食物中毒的	first case of Salmonella Iru	许素芬,周海慧.	Xu Sufen,Zhou Haihui.	中国卫生检验杂志	ese Journal of Health Inspe	16	1	116-116	2006	sn.1004-8685.2006.01.062
1908	对氟喹诺酮类药物治疗耐	ance to Fluoroquinolone Tr	李明,彭树道.	Li Ming,Peng Shu Road.	中国民康医学（下半月）	ealth medicine (second half	18	11	854862	2006	-
1909	4年服务行业从业人员沙门	onella Detection and Analy	罗美华,龙碧霞.	Luo Meihua,Long Bixia.	医学动物防制	medical animal control	22	6	455-457	2006	sn.1003-6245.2006.06.040
1910	农村食源性中毒调查及其	in rural areas of Fuyang Ci	群,吴江春,王荣妹,钱小平,	gchun,Wang Rongmei,Qia	中国卫生检验杂志	ese Journal of Health Inspe	16	8	982-984	2006	sn.1004-8685.2006.08.044
1911	大东地区1004株腹泻病原	Analysis of drug susceptibi	郑力.	Zheng Li.	实用药物与临床	ractical medicine and clini	9	1	48-49	2006	sn.1673-0070.2006.01.032
1912	004年儿科败血症病原菌	and drug resistance of pa	李秀云,王宏伟,孙自镛,杜	un,Wang Hongwei,Sun Ziy	医药导报	Medical Herald	25	12	1323-1325	2006	sn.1004-0781.2006.12.041
1913	4-2005年部分市售食品病	pathogenic bacteria in som	刘素意,李土荣,袁丹茅.	Suyi,Li Turong,Yuan Danm	职业与健康	Occupation and health	22	21	1827-1828	2006	sn.1004-1257.2006.21.038
1914	04年新疆阜康市餐具消毒	infection Monitoring Repo	姜涛,沙依拉,蔡凤,李晓琴	g Tao,Shayla,Cai Feng,Li Xia	地方病通报	endemic disease notificatio	21	5	79,82	2006	sn.1000-3711.2006.05.033
1915	小儿鼠伤寒沙门菌感染	la typhimurium infection i	张建梅,吴长春.	hang Jianmei,Wu Changchu	中外健康文摘·医药学刊	Foreign Health Digest·Me	3	2	66-67	2006	-
1916	酒席中食物中毒发生的调	t of food poisoning at self-	廖碧琴,张友强.	Liao Biqin,Zhang Youqiang.	中华实用中西医杂志	actical Traditional Chinese a	19	4	488-488	2006	-
1917	中分离出甲型副伤寒沙门	monella paratyphi A from	黄爱芳.	Huang Aifang.	中国医药论坛	China Pharmaceutical Foru	-	11	-	2006	-
1918	门菌污染水源所致腹泻暴	iarrhea caused by Salmon	杨,刘金芳,许静静,郭惠,童	u Jinfang,Xu Jingjing,Guo H	疾病监测	disease surveillance	21	9	463-466	2006	sn.1003-9961.2006.09.008
1919	广东省食品中食源性致病	f foodborne pathogenic ba	冰,宋曼丹,赖蔚苳,王建,马	Mandan,Lai Weiruan,Wang	中国食品卫生杂志	hinese Food Hygiene Journ	18	6	528-531	2006	sn.1004-8456.2006.06.009
1920	植患者尿中检出一株德比	was detected in the urine	王赛芳,周铁丽,李超.	ng Saifang,Zhou Tielia,Li C	江西医学检验	Jiangxi Medical Laboratory	-	S1	648	2006	-
1921	测食品中的沙门菌及分离	resistance of isolated stra	学斌,顾宝柯,金汇明,胡培	in,Gu Baoke,Jin Huiming,H	中国食品卫生杂志	hinese Food Hygiene Journ	18	3	202-204	2006	sn.1004-8456.2006.03.003
1922	服务从业人员携带沙门氏	monella carried by caterin	黄烈泓.	Huang Liehong.	公共卫生与预防医学	Health and Preventive Me	17	2	68	2006	sn.1006-2483.2006.02.031

1923	起沙门菌食物中毒检验报	onella food poisoning test r	李常教.	Li Changjiao.	中国卫生检验杂志	ese Journal of Health Inspe	16	7	869-870	2006	sn.1004-8685.2006.07.056
1924	城区学校食堂卫生现状调	giene status of school cant	長,俞惠飞,徐孝波,陈圣安,张	Huifei,Xu Xiaobo,Chen Shen	中国预防医学杂志	se Journal of Preventive Me	7	5	418-420	2006	sn.1009-6639.2006.05.014
1925	列沙门菌感染患者的护理	rience of patients with salr	邦翠,吴世萍,李艳春,史明	i,Wu Shiping,Li Yanchun,Sh	贵州医药	Guizhou Medicine	30	2	189-189	2006	n.1000-744X.2006.02.047
1926	起沙门菌引起食物中毒的	case of food poisoning ca	吴卫东,臧燕,彭武梅.	Weidong,Zang Yan,Peng Wu	职业与健康	Occupation and health	22	4	267	2006	sn.1004-1257.2006.04.021
1927	1年～2005年食源性致病	nnual surveillance analysis	戴建华,乔昕,袁宝君,沈赟	ua,Qiao Xin,Yuan Baojun,S	江苏预防医学	iangsu Preventive Medicin	17	3	50-52	2006	sn.1006-9070.2006.03.029
1928	疆巴州374份夏季腹泻病	74Reference test analysis o	晖,王海燕,陈文隽,吴海燕,	Haiyan,Chen Wenjun,Wu	地方病通报	endemic disease notificatio	21	3	93	2006	sn.1000-3711.2006.03.050
1929	幼儿腹泻病原微生物感染	and control strategies for	张春平,晏江丽,周忠华,江	g,Yan Jiangli,Zhou Zhonghu	江西医学检验	Jiangxi Medical Laboratory	24	2	113-115	2006	sn.1674-1129.2006.02.008
1930	业从业人员肠道沙门氏菌	c Salmonella Test Results a	徐胜玲,蔡师志,汤国球.	hengling,Cai Shizhi,Tang Gu	热带医学杂志	ournal of tropical medicine	6	6	712-713	2006	sn.1672-3619.2006.06.033
1931	160份食品致病菌检测	60Food pathogen detectio	蒋兴祥,何婷婷,沈红.	Xingxiang,He Tingting,Shen	浙江预防医学	hejiang Preventive Medicin	18	12	36,42	2006	sn.1007-0931.2006.12.024
1932	K产品中沙门菌的流行病	cteristics of Salmonella in a	陈春梅,陈宗辉,王斌.	Chunmei,Chen Zonghui,Wa	中国国境卫生检疫杂志	ontier Health and Quarantin	29	3	175-176	2006	sn.1004-9770.2006.03.018
1933	俄界河(瑚布图河)卫生监	ary River(hubutu river)hea	王波,周庆华,张爱英,马微,张	u Qinghua,Zhang Aiying,Ma	检验检疫科学	pection and Quarantine Scie	16	5	5-6	2006	sn.1674-5354.2006.05.002
1934	起突发伤寒疫情的应急处	ponse to a sudden typhoid	承德,朱臣凯,谷永香,扬翠	Zhu Chenkai,Gu Yongxiang	现代预防医学	modern preventive medicin	33	3	331-332,334	2006	sn.1003-8507.2006.03.037
1935	例高热患者血液中检出甲	blood of a patient with hig	泉水,王杰,周向国,李作生,	ang Jie,Zhou Xiangguo,Li Z	中国卫生检验杂志	ese Journal of Health Inspe	16	4	490-490	2006	sn.1004-8685.2006.04.056
1936	鸡肉中沙门菌的耐药性研究	drug resistance of Salmone	林启辉.	Lin Qihui.	实用预防医学	ractical preventive medicin	13	1	175-176	2006	sn.1006-3110.2006.01.087
1937	～2004年广西伤寒副伤寒	ce of typhoid and paratyp	振柱,龚健,李翠云,王鸣柳,曾	Gong Jian,Li Cuiyun,Wang M	现代预防医学	modern preventive medicin	33	10	1834-1837	2006	sn.1003-8507.2006.10.038
1938	型副伤寒沙门菌104株药敏	ratyphi A104strain drug sus	尹建雯,高援,徐闻,杨建斌	wen,Gao Yuan,Xu Wen,Yang	预防医学情报杂志	of Preventive Medicine Info	22	3	351-352	2006	sn.1006-4028.2006.03.037
1939	起甲型副伤寒爆发调查分	d Analysis of an Outbreak c	邹毅,胡国超.	Zou Yi,Hu Guochao.	热带医学杂志	ournal of tropical medicine	6	6	726-727	2006	sn.1672-3619.2006.06.039
1940	伤寒沙门菌检出TEM-1耐药	typhi was detectedTEM-1	安,徐景野,石优章,金春光,杨	e,Shi Youzhang,Jin Chungu	中国卫生检验杂志	ese Journal of Health Inspe	16	4	403-404	2006	sn.1004-8685.2006.04.008
1941	食源性致病菌污染状况监	odborne pathogenic bacter	赵江义.	Zhao Jiangyi.	中国卫生检验杂志	ese Journal of Health Inspe	16	5	587-587	2006	sn.1004-8685.2006.05.038
1942	南阳市犬病流行现状调查	pidemic status of canine dis	飞,边东生,赵月宾,张同信,	Dongsheng,Zhao Yuebin,Zh	医学动物防制	medical animal control	22	5	341-342	2006	sn.1003-6245.2006.05.015
1943	伤寒的肠炎沙门菌食物中毒	almonella Enteritidis food p	梁衍珊,王玉林,谭铭雄,何浩	anshan,Wang Yulin,Tan Mi	疾病监测	disease surveillance	21	9	467-469	2006	sn.1003-9961.2006.09.009
1944	区食品从业人员沙门菌带	tatus among food workers	张秋丽,黄嘉盈,杨房友.	iuli,Huang Jiaying,Roomma	华南预防医学	uth China Preventive Medic	32	1	78-79	2006	sn.1671-5039.2006.01.035
1945	测出甲型副伤寒沙门氏菌的	detection of Salmonella Pa	景野,章丹阳,金春光,郑剑	g Danyang,Jin Chunguang,Z	卫生研究	health research	35	2	204-205	2006	sn.1000-8020.2006.02.033
1946	血培养病原菌分布及耐药	acteria distribution and dru	姜友珍.	Jiang Youzhen.	南华大学学报（医学版）	iversity of South China (Me	34	3	414-416	2006	sn.2095-1116.2006.03.032
1947	伯林沙门氏菌引起食物中毒	d poisoning caused by Salr	李常教,刘慧民,温正恒.	ngjiao,Liu Huimin,Wen Zhen	现代预防医学	modern preventive medicin	33	11	21192121	2006	sn.1003-8507.2006.11.045
1948	道感染的致病菌及其耐药	a and their drug resistance	彦香,赵舒斌,谢振华,孙霆	g,Zhao Shubin,Xie Zhenhua	宁夏医学杂志	Ningxia Medical Journal	28	10	781-783	2006	sn.1001-5949.2006.10.033
1949	型副伤寒沙门菌48例临床分	a Paratyphi A48Case clinic	-	-	实用全科医学	Practical General Medicine	4	5	544-545	2006	-
1950	西安市细菌性腹泻病监测结	acterial diarrhea surveillan	张晓宇,蔡正华,李一航.	g Xiaoyu,Cai Zhenghua,Li Yi	疾病监测	disease surveillance	21	9	470-471	2006	sn.1003-9961.2006.09.010
1951	霍乱病人标本中检出沙门	cted in specimens from fou	刘红丽.	Liu Hongli.	中国公共卫生管理	nese public health manager	22	6	532-533	2006	sn.1001-9561.2006.06.050
1952	广州市市区食品污染状况	sis on Food Pollution Situat	蒋卓勤,李迎月,毛新武,黄聪	n,Li Yingyue,Mao Xinwu,Hu	实用预防医学	ractical preventive medicin	13	4	956-958	2006	sn.1006-3110.2006.04.068
1953	菌性食物中毒病原菌的调	on of bacterial food poison	路,扈庆华,王冰,贺连华,文	Qinghua,Wang Bing,He Lian	职业与健康	Occupation and health	22	19	1563-1564	2006	sn.1004-1257.2006.19.024
1954	岛甲型副伤寒沙门菌耐药性	esistance of Salmonella pa	狄云湘,应英.	Di Yunxiang,Ying Ying.	上海预防医学	hanghai Preventive Medicin	18	10	494-494	2006	sn.1004-9231.2006.10.006
1955	患者粪便中分离出斯坦利沙	isolated from feces of AID	徐振明,刘峰,张丽君,叶兴德	ning,Liu Feng,Zhang Lijun,Y	山东医药	Shandong Medicine	46	25	50	2006	n.1002-266X.2006.25.087
1956	一起易误诊的食物中毒	misdiagnosed case of food	石,郑辉,周少聪,石艳杰,马艳	Hui,Zhou Shaocong,Shi Yan	武警医学	Armed Police Medicine	17	1	62-63	2006	sn.1004-3594.2006.01.032
1957	中沙门菌和单增李斯特菌	monocytogenes in food in	刘桂华,杨红,龚云伟,黄鑫,孙	ang Hong,Gong Yunwei,Hua	中国卫生检验杂志	ese Journal of Health Inspe	16	10	1227-1228	2006	sn.1004-8685.2006.10.040
1958	地血标本中革兰阴性杆菌	m-negative bacilli in blood	王瑶,李菁菁,徐英春,谢秀丽	o,Li Jingjing,Xu Yingchun,X	中国感染与化疗杂志	urnal of Infection and Che	6	4	251-254	2006	sn:1009-7708.2006.04.009
1959	市市售小水产品污染状况	atus of small aquatic produ	奋,裘立晓,陈美芬,金晓霞,	Lixiao,Chen Meifen,Jin Xiao	中国卫生检验杂志	ese Journal of Health Inspe	16	7	839	2006	sn.1004-8685.2006.07.039

1960	幼儿乳粉和米粉微生物污	commercially available infa	亭,李玉伟,李凤琴,计融,张立	Yuwei,Li Fengqin,Ji Rong,Z	现代预防医学	nodern preventive medicin	33	7	1065-1067	2006	sn.1003-8507.2006.07.004
1961	关节腔积液内分离的肠炎	monella Enteritidis isolated	-	-	中国临床药理学杂志	e Journal of Clinical Pharma	22	4	302	2006	-
1962	～2005年饮食服务者病原	surveillance of pathogenic	忠,胡丽萍,舒桂梅,陈淑云,	Liping,Shu Guimei,Chen Sh	中国热带医学	Chinese tropical medicine	6	11	2045-2046	2006	sn.1009-9727.2006.11.070
1963	中食源性致病菌污染状况调	ation status of various foo	谭海芳,丁丽娜,陈华.	n Haifang,Ding Lina,Chen H	热带医学杂志	ournal of tropical medicine	6	1	63-64	2006	sn.1672-3619.2006.01.022
1964	近海牡蛎污染状况调查与	ation of oyster pollution st	蔡雷鸣,肖莹.	Cai Leiming,Xiao Ying.	海洋环境科学	arine Environmental Scien	25	1	80-83	2006	sn.1007-6336.2006.01.022
1965	患者粪便中分离出1例斯坦	e feces of AIDS patients1Sa	余振明,刘峰,张丽君,叶兴德	ning,Liu Feng,Zhang Lijun,Y	中华临床医学研究杂志	Journal of Clinical Medical	12	12	-	2006	-
1966	感染性腹泻病原菌分布及耐	ance of pathogenic bacteri	-	-	人民军医	people's military medicine	49	7	386-387	2006	-
1967	沙门氏菌化脓性脑膜炎1例	phi purulent meningitis1Ex	覃敏.	Qin Min.	临床儿科杂志	Journal of Clinical Pediatrics	24	10	789796	2006	sn.1000-3606.2006.10.023
1968	儿粪便中检出鼠伤寒沙门	n detected in feces of child	朱元祺,秦萍,黄伟丽,任丽娟	qi,Qin Ping,Huang Weili,Re	世界华人消化杂志	rld Chinese Journal of Diges	14	12	1233-1234	2006	sn.1009-3079.2006.12.022
1969	起由沙门菌引起的食物中	food poisoning caused by s	汪琦,李国梁.	Wang Qi,Li Guoliang.	中国卫生检验杂志	ese Journal of Health Inspe	16	6	744-744	2006	sn.1004-8685.2006.06.060
1970	成一起较大食物中毒事件的	or food poisoning incident	智聪,毛新武,景钦隆,林晓	Mao Xinwu,Jing Qinlong,Lin	中国热带医学	Chinese tropical medicine	6	12	2268-2269,2281	2006	sn.1009-9727.2006.12.096
1971	州市城区污水微生物学调	nvestigation of urban sewa	卢嘉明,许雅.	Lu Jiaming,Xu Ya.	华南预防医学	uth China Preventive Medic	32	4	68-69	2006	sn.1671-5039.2006.04.027
1972	氏菌食物中毒并发血尿的	of salmonella food poisonin	鄢艳,周静,陈钦开.	n Yan,Zhou Jing,Chen Qink	实用临床医学	practical clinical medicine	7	12	60,62	2006	sn.1009-8194.2006.12.019
1973	门氏菌污染熟牛肉引起的食	caused by salmonella conta	朱映丽.	Zhu Yingli.	中国医学研究与临床	ese medical research and cl	4	2	-	2006	-
1974	食源性致病菌污染状况调	borne pathogenic bacteria	茂慧,马弋,杨晓敏,付洁,李	ui,Ma Yi,Yang Xiaomin,Fu Ji	公共卫生与预防医学	Health and Preventive Me	17	2	31-33	2006	sn.1006-2483.2006.02.010
1975	丹沙门菌引起化脓性脑膜	ngitis caused by Salmonella	江东,徐新波,苏兆兰,吴金	ong,Xu Xinbo,Su Zhaolan,W	中华医院感染学杂志	ournal of Hospital Infectiou	16	7	813-813	2006	sn:1005-4529.2006.07.058
1976	甲型副伤寒214例临床分析	phoid A214Case clinical an	狄君斐.	Di Junfei.	中国医师杂志	Chinese Physician Journal	8	1	85-86	2006	sn.1008-1372.2006.01.038
1977	引起幼儿群体性食物中毒	od poisoning in young child	朱水龙.	Zhu Shuilong.	实用医技杂志	l of Practical Medical Tech	13	17	3096-3097	2006	sn.1671-5098.2006.17.112
1978	离菌株及其抗生素敏感性	om blood and changes in th	赖晓全,王洪波.	Lai Xiaoquan,Wang Hongbo	中国公共卫生	Chinese public health	22	1	66-67	2006	sn:1001-0580.2006.01.031
1979	年中江县细菌性食物中毒	alysis of Bacterial Food Poi	赖霖.	Lai Lin.	职业卫生与病伤	ccupational health and illne	21	1	61-62	2006	n.1006-172X.2006.01.036
1980	河南省食源性致病菌和耐药	pathogenic bacteria and dr	张丁,胡巅,高葆真,炊慧霞,	,Hu Dian,Gao Baozhen,Cui	中国卫生检验杂志	ese Journal of Health Inspe	16	7	842-844	2006	sn.1004-8685.2006.07.042
1981	变异伤寒沙门氏菌致败血症	used by Salmonella typhi v	孙胜利,陈虹,张媛媛.	engli,Chen Hong,Zhang Yua	西北国防医学杂志	west Journal of Defense Me	27	2	149	2006	sn.1007-8622.2006.02.050
1982	3年我国766起细菌性食物	untry766Analysis of bacteri	杜杰,杨杰,谌志强,张伟,李君	g Jie,Chen Zhiqiang,Zhang	中国预防医学杂志	se Journal of Preventive Me	7	3	180-184	2006	sn.1009-6639.2006.03.006
1983	沙门菌食物中毒的快速诊	bility of a case of Salmonell	王冰,林一曼,刘小立,林和顺	g,Lin Yiman,Liu Xiaoli,Lin H	中华检验医学杂志	se Journal of Laboratory Me	29	7	623	2006	sn:1009-9158.2006.07.017
1984	对氟喹诺酮类药物治疗耐	ance to Fluoroquinolone Tr	李明,彭树道.	Li Ming,Peng Shu Road.	中国民康医学	nese People's Health Medic	18	22	854862	2006	sn.1672-0369.2006.22.011
1985	分离株脉冲场凝胶电泳分	d A isolates from four provi	田克诚,钟豪杰,朱凤才,梁	cheng,Zhong Haojie,Zhu Fe	中华流行病学杂志	inese Journal of Epidemiolo	27	10	871-874	2006	sn:0254-6450.2006.10.011
1986	副伤寒病原学调查及耐药	aratyphoid fever and moni	真,杨元斌,金春光,徐景野,徐	Yuanbin,Jin Chunguang,Xu	疾病监测	disease surveillance	21	4	176-177	2006	sn.1003-9961.2006.04.005
1987	7种食源性致病菌污染状况	ation status and drug resist	晓勤,徐勤,何日,周丽萍,周	u Qin,What day,Zhou Lipin	中国食品卫生杂志	hinese Food Hygiene Journ	18	1	23-25	2006	sn.1004-8456.2006.01.006
1988	郡柏林沙门菌食物中毒病原	thogen of Salmonella food	金建洪,祝凤英,耿卫红.	hong,Zhu Fengying,Geng W	浙江预防医学	hejiang Preventive Medicin	18	11	39-39	2006	sn.1007-0931.2006.11.026
1989	鼠伤寒沙门氏菌混合感染引	caused by mixed infection	韩丽英.	Han Liying.	现代预防医学	nodern preventive medicin	33	6	1017-1017,1019	2006	sn.1003-8507.2006.06.081
1990	株甲型副伤寒沙门菌药敏	ility analysis of Salmonella	军,张碧霞,张彤,曹雁,毛跃	Bixia,Zhang Tong,Cao Yan,	实用医技杂志	l of Practical Medical Tech	13	22	3977-3978	2006	sn.1671-5098.2006.22.051
1991	肃省特禽疫病的流行病学调	ation of special poultry dis	詹发茂.	Zhan Famao.	中国畜牧兽医	mal husbandry and veterina	33	7	54-56	2006	sn.1671-7236.2006.07.018
1992	加工流通环节沙门氏菌的	nella in pig slaughtering, pr	冬冬,黄金林,陶善华,焦新	g,Golden Forest,Tao Shanh	畜禽业	vestock and poultry indust	-	3	44-45	2006	sn.1008-0414.2006.03.017
1993	人血流感染预后相关因素分	ed to prognosis of bloodstr	丁续红,胡苏萍,吴小军.	Xuhong,Hu Suping,Wu Xia	中华全科医师杂志	e Journal of General Practi	5	10	599-602	2006	sn.1671-7368.2006.10.008
1994	膜炎致病菌的分离与鉴定及	alysis of pathogenic bacte	宏坤,杨少华,王长法,高运	Yang Shaohua,Wang Chang	中国畜牧兽医	mal husbandry and veterina	33	11	28-31	2006	sn.1671-7236.2006.11.009
1995	儿甲型副伤寒53例临床分	d A in children53Case clini	滕承志.	Teng Chengzhi.	浙江预防医学	hejiang Preventive Medicin	18	1	44-45	2006	sn.1007-0931.2006.01.032
1996	地区甲型副伤寒沙门菌基因	of Salmonella paratyphi A in	泽,金春光,章丹阳,杨元斌,沈	ang,Zhang Danyang,Yang Y	中国公共卫生	Chinese public health	22	11	1373-1374	2006	sn:1001-0580.2006.11.053

1997	寒甲并发中毒性肝炎的临床	of paratyphoid A complica	可飞,杨守平,陶维玉,黄志	ang Shouping,Tao Weiyu,H	中华传染病杂志	se Journal of Infectious Dis	24	1	62-63	2006	sn:1000-6680.2006.01.020
1998	甲型副伤寒临床特征及药	and drug sensitivity analysi	孔庆芬,缪新权.	Kong Qingfen,Miao Xinquan	中国现代医学杂志	ese Journal of Modern Med	16	9	1407-1408,1411	2006	sn.1005-8982.2006.09.035
1999	铁路辖区部分从业人员沙	tuation among Some Empl	梁春霞.	Liang Chunxia.	预防医学论坛	Preventive Medicine Forum	12	1	94-95	2006	sn.1672-9153.2006.01.047
2000	44株革兰阴性杆菌的耐药	ance distribution and chang	繁林,储从家,管新龙,罗次	u Congjia,Guan Xinlong,Ra	中国微生态学杂志	inese Journal of Microecolo	18	3	229-231	2006	n.1005-376X.2006.03.029
2001	髓培养伤寒沙门菌3例报	culture Salmonella typhi3E	邵宏伟.	Shao Hongwei.	中国冶金工业医学杂志	rnal of Metallurgical Indust	23	1	100	2006	sn.1005-5495.2006.01.083
2002	生食蔬菜胡萝卜中检出沙门	cted in commercially availa	邓华英,郭爱玲,蒋涛,余军,王	ing,Guo Ailing,Jiang Tao,Yu	中国卫生检验杂志	ese Journal of Health Inspe	16	12	1537-1537	2006	sn.1004-8685.2006.12.067
2003	婴儿沙门菌杆菌引起的食	poisoning caused by Salm	陈国忠,王红.	Chen Guozhong,Wang Hong	中国卫生检验杂志	ese Journal of Health Inspe	16	4	476-476	2006	sn.1004-8685.2006.04.045
2004	沙门氏菌引起食物中毒的调	of food poisoning caused b	刘文.	Liu Wen.	安徽卫生职业技术学院学报	ui Health Vocational and Te	5	4	93-94,88	2006	sn.1671-8054.2006.04.056
2005	菌对喹诺酮类耐药机制研究	m and genotyping of Salmo	陈晶,孙自镛,张振林.	n Jing,Sun Ziyong,Zhang Zhe	中华检验医学杂志	se Journal of Laboratory Me	29	8	730-732	2006	sn:1009-9158.2006.08.020
2006	耐药甲型副伤寒430例临床	tant paratyphoid A430Case	高峰,杨朝晖,张培会.	ak,Yang Zhaohui,Zhang Peih	实用医学杂志	ournal of Practical Medicin	22	8	949-950	2006	sn.1006-5725.2006.08.042
2007	区奶牛乳腺炎调查及病原菌	ws and isolation test of pat	明举,郑志新,罗永华,王若	heng Zhixin,Luo Yonghua,W	北方学院学报（自然科学	Northern University (Natura	22	3	54-57	2006	sn.1673-1492.2006.03.017
2008	腹泻病中沙门氏菌的分离与	ntification of Salmonella fr	俞华英,胡文兵,贾桂珍,蒋涛	g,Hu Wenbing,Jia Guizhen,	畜牧与兽医	Husbandry and Veterinary M	38	2	41-42	2006	sn.0529-5130.2006.02.017
2009	染性腹泻452例临床和病原	hildren452Clinical and etio	马建中,刘庆日.	Ma Jianzhong,Liu Qingri.	基层医学论坛	Primary Medicine Forum	10	8	312-313	2006	sn.1672-1721.2006.08.017
2010	腹泻病中沙门氏菌的分离与	ntification of Salmonella fr	俞华英,胡文兵,贾桂珍,蒋涛	g,Hu Wenbing,Jia Guizhen,	塔里木大学学报	Journal of Tarim University	18	1	10-12,15	2006	sn.1009-0568.2006.01.003
2011	融合术后都柏林沙门菌感	n infection after hip fusion	姜丽萍,于桂英.	Jiang Liping,Yu Guiying.	沈阳部队医药	Shenyang Army Medicine	-	5	348	2006	-
2012	库尔勒市夏季肠道致病菌	results of intestinal pathog	海珍,牛向辉,尹艳荣,赵立	en,Niu Xianghui,Yin Yanrong	地方病通报	endemic disease notificatio	21	2	77-78	2006	sn.1000-3711.2006.02.031
2013	扬州市食源性致病菌污染状	atus of foodborne pathogen	高玉春,巢国祥.	Gao Yuchun,Chao Guoxiang	江苏预防医学	Jiangsu Preventive Medicine	17	4	34-36	2006	sn.1006-9070.2006.04.017
2014	人猪霍乱沙门菌败血症1例	a Swine Salmonella Septice	开绿,李文军,郑晓凤,刘艳	Li Wenjun,Zheng Xiaofeng,	中国感染与化疗杂志	urnal of Infection and Chem	6	1	61-61	2006	sn:1009-7708.2006.01.018
2015	72例伤寒暴发流行分析	c analysis of typhoid feve	范旭;	Fan Xu;	现代医药卫生	odern medicine and heal	-	23	3493	2006	-
2016	开发区一起水型副伤寒暴	water-type paratyphoid fe	毅;王建民;龚朝阳;陶宏;	nmin;Gong Chaoyang;Ta	现代预防医学	odern preventive medicin	-	10	1977-1978	2006	-
2017	起发生在中学学校的伤寒暴	r outbreak occurred in a	刘文生;吴中发;许先来;	nsheng;Wu Zhongfa;Xu	预防医学论坛	reventive Medicine Foru	-	4	499-500	2006	-
2018	对地方性斑疹伤寒暴发的调	outbreak of endemic typh	王庆新;李育民;刘继政;	Qingxin;Li Yumin;Liu Jiz	现代预防医学	odern preventive medicin	-	6	1010	2006	-
2019	起中学生伤寒暴发的疫情分	of typhoid fever among	杨建刚;张宏民;罗祥莉;	gang;Zhang Hongmin;Lu	黔南民族医专学报	Qiannan National Medic	-	2	93+95	2006	-
2020	生活饮用水污染与伤寒暴发	elf-prepared drinking wat	翁晓林;吕彪;施聃;	ng Xiaolin;Lu Biao;Shi D	现代预防医学	odern preventive medicin	-	4	555	2006	-
2021	副伤寒暴发疫情的病原学	diagnosis of paratyphoid	马海玲;陈媛;	Ma Hailing;Chen Yuan;	实用预防医学	actical preventive medici	-	2	352-353	2006	-
2022	城社区甲型副伤寒暴发流行	typhoid A outbreak in Ha	张宗祥;柴程良;陈辉华;	xiang;Chai Chengliang;C	中国预防医学杂志	e Journal of Preventive M	-	2	135-136	2006	-
2023	流行时病人的心理状况及	ng intervention of patient	永红;罗显荣;王惠珍;曾国	uo Xianrong;Wang Huizh	护理研究(中旬版)	ng research(mid-term ed	-	11	954-956	2006	-
2024	副伤寒暴发疫情的现场流行	l study on an outbreak of	;杨涛;张颖;龚健;黄运能;	ng Ying;Gong Jian;Huan	广西预防医学	uangxi Preventive Medici	-	1	16-18	2006	-
2025	污染引起的甲型副伤寒暴发	of Paratyphoid A Cause	晶;黄诚孝;郑琳;考庆君;王	engxiao;Zheng Lin;Kao C	疾病监测	disease surveillance	-	1	16-17+34	2006	-
2026	胶电泳分型在伤寒暴发中	electrophoresis typing in	萍;刘渠;陈应坚;杨慧;石	Qu;Chen Yingjian;Yang	现代预防医学	odern preventive medicin	-	1	63-65	2006	-
2027	寒沙门菌污染哈密瓜引起的	by salmonella typhi conta	邓丽芳,王雪梅,许立新.	g Lifang,Wang Xuemei,Xu L	预防医学论坛	Preventive Medicine Forum	13	3	236-237	2007	sn.1672-9153.2007.03.044
2028	门菌及致病性大肠埃希菌	g caused by Salmonella typ	周龙成,廖安波,颜雯雯,谌	eng,Liao Anbo,Yan Wenwe	预防医学情报杂志	of Preventive Medicine Info	23	1	82-83	2007	sn.1006-4028.2007.01.025
2029	务业人员携带沙门菌菌型分	tance of Salmonella carried	钟学庆.	Zhong Xueqing.	职业与健康	Occupation and health	23	21	1955-1956	2007	sn.1004-1257.2007.21.035
2030	沙门菌引发食物中毒的调	n a case of food poisoning	刘兆宇,白化民,刘铭.	Zhaoyu,albino people,Liu M	中华现代内科学杂志	ournal of Modern Internal	4	12	-	2007	-
2031	斯托夫沙门菌引起食物中	poisoning caused by Salm	建华,兰增权,金丽萍,雷永	,Lan Zengquan,Jin Liping,Le	中国食品卫生杂志	hinese Food Hygiene Journ	19	4	356-357	2007	sn.1004-8456.2007.04.025
2032	州市熟肉制品微生物污染	ntamination of cooked me	陈秀恋,杨育红,刘建忠.	iulian,Yang Yuhong,Liu Jian	海峡预防医学杂志	s Journal of Preventive Me	13	5	62	2007	sn.1007-2705.2007.05.043
2033	杨杆菌及沙门菌中磺胺类	Escherichia coli and Salmo	安云,吴琦,黄勇,柳萍,田	nyun,Wu Qi,Huang Yong,Li	中国兽医科学	Chinese Veterinary Science	37	4	287-290	2007	sn.1673-4696.2007.04.005

2034	沙门氏菌食物中毒的微生	etection of Salmonella kia	彩云,廖国东,邓绍甫,罗雪	Liao Guodong,Deng Shaofu	国际医药卫生导报	ational Medical and Health	13	8	123-124	2007	sn.1007-1245.2007.08.047
2035	伤寒沙门菌的临床分布及	usceptibility results report	吴颖,曹伟.	Wu Ying,Cao Wei.	实用预防医学	ractical preventive medicin	14	2	552-553	2007	sn.1006-3110.2007.02.140
2036	要食源性致病菌的耐药状	pectrum of several importa	波,朱海明,马聪,何冬梅,杨	ng,Ma Cong,He Dongmei,Y	中国卫生检验杂志	ese Journal of Health Inspe	17	11	2030-2032,2077	2007	sn.1004-8685.2007.11.046
2037	急性腹泻病原菌及药敏分	bacteria and drug suscepti	徐少保,吴晓玲,沈强.	haobao,Wu Xiaoling,Shen Q	安徽医药	Anhui Medicine	11	8	719-720	2007	sn.1009-6469.2007.08.028
2038	村自办婚宴引发食物中毒	ng caused by a self-hosted	朱星财.	Zhu Xingcai.	海峡预防医学杂志	s Journal of Preventive Me	13	5	56	2007	sn.1007-2705.2007.05.034
2039	伤寒爆发疫情的流行病学	on and analysis of a typhoi	瑾,马汉武,王敬忠,张小岚	Hanwu,Wang Jingzhong,Zha	热带医学杂志	ournal of tropical medicine	7	8	821-823,836	2007	sn.1672-3619.2007.08.037
2040	胶电泳分型技术对江西省	rovince using pulsed field	张京云,李育强,刘晓青,崔	Jingyun,Li Yuqiang,Liu Xia	疾病监测	disease surveillance	22	8	514-515	2007	sn.1003-9961.2007.08.005
2041	宰生猪肉样沙门菌的PCR	in slaughtered raw pork in	新安,魏建忠,黄金林,李超	Jianzhong,Golden Forest,L	中国卫生检验杂志	ese Journal of Health Inspe	17	11	2018-2019	2007	sn.1004-8685.2007.11.040
2042	005年进口海产品中3种致	seafood per year3Analysis	于兵,王殿夫,麻丽丹.	u Bing,Wang Dianfu,Malida	中国国境卫生检疫杂志	ntier Health and Quarantin	30	2	104-105	2007	sn.1004-9770.2007.02.013
2043	鼠伤寒沙门菌株耐药谱及P	lmonella typhimurium stra	周正,刘秀梅.	Zhou Zheng,Liu Xiumei.	中国食品卫生杂志	hinese Food Hygiene Journ	19	3	221-224	2007	sn.1004-8456.2007.03.008
2044	~2006年食品中食源性致	nitoring and analysis of foo	,金莞尔,张建民,王文英,	Van'er,Zhang Jianmin,Wang	中国卫生检验杂志	ese Journal of Health Inspe	17	4	680-681,706	2007	sn.1004-8685.2007.04.050
2045	猪肉中沙门菌分离株多重	e spectrum of Salmonella	新安,魏建忠,王强,王伟,	Jianzhong,Wang Qiang,Wa	中华流行病学杂志	inese Journal of Epidemiol	28	12	1246	2007	sn:0254-6450.2007.12.032
2046	伤寒性髋关节炎	Typhoid hip arthritis	辉,林淦松,廖穗祥,赵秉航,	ansong,Liao Suixiang,Zhao	广西医学	Guangxi Medicine	29	7	1075-1076	2007	sn.0253-4304.2007.07.065
2047	儿腹泻暴发病原学调查和	a outbreak in a hospital ar	王靖虹.	Wang Jinghong.	职业与健康	Occupation and health	23	23	2183-2185	2007	sn.1004-1257.2007.23.037
2048	品和公共场所从业人员健	amination results for empl	阳,麦世樑,谢春霖,潘顺金	,Mai Shiliang,Xie Chunlin,P	中国公共卫生管理	ese public health managen	23	4	343-345	2007	sn.1001-9561.2007.04.015
2049	省食品中沙门菌污染状况	onella contamination in foo	,马国柱,张芳,李雪梅,潘	a Guozhu,Zhang Fang,Li Xu	中华预防医学杂志	se Journal of Preventive Me	41	6	510-511	2007	sn:0253-9624.2007.06.022
2050	菌和沙门氏菌的发病趋势	nce changes of E. coli and	陈书民,兰邹然,马慧玲,孙	hen Shumin,Lan Zouran,Ma	畜牧兽医科技信息	d Veterinary Science and Te	-	6	28-31	2007	sn.1671-6027.2007.06.017
2051	沙门菌株的脉冲场凝胶电	oresis typing of Salmonella	,钟豪杰,柯昌文,邓小玲,	ie,Ke Changwen,Deng Xiao	中国热带医学	Chinese tropical medicine	7	1	12-13,23	2007	sn.1009-9727.2007.01.006
2052	门菌引起食物中毒事件的	n a food poisoning incident	王英彬,陈玉芳,陈思伟.	Yingbin,Chen Yufang,Chen	中国新医学论坛	China New Medicine Forum	7	12	-	2007	-
2053	引起学生肠炎沙门茵食物	ng caused by salmonella en	陈志铨.	Chen Zhiquan.	中国学校卫生	Chinese school health	28	11	1041	2007	-
2054	005年饮食服务从业人员	urvey on Salmonella carria	伟文,叶朝红,钟红苗,岑玫	Ye Chaohong,Zhong Hongn	中国热带医学	Chinese tropical medicine	7	4	634651	2007	sn.1009-9727.2007.04.077
2055	副伤寒分离菌株脉冲场凝	oresis typing of paratyphoi	秋,潘劲草,孟冬梅,张蔚,	Jincao,Meng Dongmei,Zha	中国公共卫生	Chinese public health	23	3	369-370	2007	sn:1001-0580.2007.03.058
2056	分省市猪肉产品细菌学指	rs of pork products in som	,郑增忍,王晶钰,张衍海,	engren,Wang Jingyu,Zhang	安徽农业科学	Anhui Agricultural Sciences	35	9	2620-2621	2007	sn.0517-6611.2007.09.057
2057	杆菌垂直感染造成雏鸡大	monella in breeders causes	管辉,王永生,扬魁,农莉.	i,Wang Yongsheng,Yangkui	新疆畜牧业	Xinjiang animal husbandry	-	1	60-61	2007	sn.1003-4889.2007.01.031
2058	模养鸡场沙门氏菌流行病	sis of Salmonella in small-s	兰邹然,陈书民,马慧玲,孙	n Zouran,Chen Shumin,Ma	家禽科学	poultry science	-	5	11-13	2007	sn.1673-1085.2007.05.005
2059	本中分离出一株布里丹沙	train of Salmonella bridan f	王芳,潘慧英,张宏宾.	Fang,Pan Huiying,Zhang H	中华现代医学与临床	ern Chinese Medicine and	-	2	-	2007	-
2060	菌血培养分离株中各型分	ceptibility analysis of Salm	罗成富,杜燕.	Luo Chengfu,Du Yan.	西南军医	outhwest Military Medicin	9	4	50-51	2007	sn.1672-7193.2007.04.030
2061	关市区食品污染物监测研	rch on food contaminants	陈志好,苏少芳,招莉,陈志	hao,Su Shaofang,Zhaoli,Ch	中国食品卫生杂志	hinese Food Hygiene Journ	19	4	322-324	2007	sn.1004-8456.2007.04.012
2062	温州市食品中食源性致病	ne pathogenic bacteria in f	李小春.	Li Xiaochun.	中国卫生检验杂志	ese Journal of Health Inspe	17	10	1843-1845	2007	sn.1004-8685.2007.10.052
2063	品中沙门菌污染状况及其	ation status and drug resis	,梁柏年,尹本康,陈子慧,	Bainian,Yin Benkang,Chen Z	中国热带医学	Chinese tropical medicine	7	2	264-265	2007	sn.1009-9727.2007.02.049
2064	食源性和人源沙门氏菌的	od-borne and human-deriv	泉,焦新安,刘晓文,陈祥,	o Xinan,Liu Xiaowen,Chen	微生物学报	Acta Microbiologica Sinica	47	2	221-227	2007	sn:0001-6209.2007.02.008
2065	由阿贡纳沙门菌引起的食	d poisoning caused by Salm	刘娅兰.	Liu Yalan.	现代预防医学	modern preventive medicin	34	10	1907	2007	sn.1003-8507.2007.10.042
2066	利丹沙门菌引起食物中毒	bulidan causes food poison	,刘华,韩秀荣,刘显军,辛	ua,Han Xiurong,Liu Xianju	检验医学与临床	boratory medicine and clini	4	1	54-54	2007	sn.1672-9455.2007.01.041
2067	伤寒沙门菌的耐药现状与	s and clinical use of Salmo	孔繁林,储从家.	Kong Fanlin,Chu Congjia.	中国微生态学杂志	inese Journal of Microecol	19	1	116-117	2007	n.1005-376X.2007.01.054
2068	业职工食堂食物中毒的调	n on a case of food poison	陈顺仙.	Chen Shunxian.	职业与健康	Occupation and health	23	23	2171-2172	2007	sn.1004-1257.2007.23.028
2069	伦敦沙门氏菌引起的食物	poisoning caused by Salmo	秀峰,李玉堂,梁和平,甄国	,Li Yutang,Liang Heping,Zh	首都公共卫生	capital public health	1	6	264-266	2007	sn.1673-7830.2007.06.012
2070	活动中ICU空气细菌污染状	ctivitiesICUSurvey on air b	陈璐,杨彬,赵克义,李爱萍	hen Lu,Yang Bin,Zhao Keyi,	预防医学论坛	Preventive Medicine Forum	13	8	707-709	2007	sn.1672-9153.2007.08.015

2071	门菌与大肠杆菌耐药性的	rug resistance of Salmonel	韩伟,顾鸣,肖云民,郑永捷	Gu Ming,Xiao Yunmin,Zhen	兽药与饲料添加剂	rinary drugs and feed addi	12	1	4-7	2007	-
2072	空气中人类主要致病细菌	in human pathogenic bacte	慧,戚继忠,由士江,李华娟	,Qi Jizhong,Yu Shijiang,Li H	城市环境与城市生态	environment and urban e	20	4	42-43	2007	-
2073	沙门氏菌污染监测及其	ination in farmers' market	惠琼,李妮,曾光厅,寇莹,无	,Li Ni,Zeng Guangting,Kou	云南畜牧兽医	nal Husbandry and Veterin	-	2	38-39	2007	sn.1005-1341.2007.02.024
2074	州市上城区食物中毒事件	ens in food poisoning incid	生,张蔚,吴爱萍,杨素青,杨	g Wei,Wu Aiping,Yang Su	中国卫生检验杂志	ese Journal of Health Inspe	17	8	1478-1480	2007	sn.1004-8685.2007.08.060
2075	传染病疫情的沙门菌群分	almonella species causing	刘渠,甘莉萍,陈应坚,杨慧	Gan Liping,Chen Yingjian,Y	现代预防医学	nodern preventive medicin	34	24	4654-4656	2007	sn.1003-8507.2007.24.023
2076	检测鸡胴体中沙门菌结果	different methods for dete	安,段莹,刘颜泓,孙海欧,崔	n Ying,Liu Yanhong,Sun Ha	中国微生态学杂志	inese Journal of Microecol	19	3	259-260,262	2007	n.1005-376X.2007.03.008
2077	童甲型副伤寒78例临床分	d A in children78Case clini	马文娜.	Ma Wenna.	山东医学高等专科学校学	nal of Shandong Medical C	29	4	285-286	2007	sn.1674-0947.2007.04.019
2078	2005年曲靖市食物中毒资	of food poisoning data in	福华,李继华,罗荆荣,唐金	a,Li Jihua,Luo Jingrong,Ta	预防医学论坛	Preventive Medicine Forum	13	3	255-257	2007	sn.1672-9153.2007.03.035
2079	中沙门菌等6种食源性致	gfang City6Surveillance of	民,刘体霞,李玉婵,邱颖,肖	Liu Tixia,Li Yuchan,Qiu Ying	中国卫生检验杂志	ese Journal of Health Inspe	17	12	2283-2284	2007	sn.1004-8685.2007.12.065
2080	寒沙门菌致腮腺脓肿的临	gland abscess caused by S	张立江.	Zhang Lijiang.	检验医学与临床	boratory medicine and clin	4	3	封4	2007	sn.1672-9455.2007.03.065
2081	病人粪便中致病菌的构成	pathogenic bacteria in the	莉,裘宇蓉,张少芬,童晓文	Yurong,Zhang Shaofen,Ton	热带医学杂志	journal of tropical medicine	7	4	326-329	2007	sn.1672-3619.2007.04.008
2082	2004年食物中毒实验室检	nual food poisoning labora	邓辉.	Deng Hui.	中国自然医学杂志	ese Journal of Natural Med	9	1	69-70	2007	-
2083	场断奶腹泻仔猪致病菌的	bacteria in weaned piglets	宗,祝海宝,吴庆儒,李文波	,Zhu Haibao,Wu Qingru,Li	中国畜牧兽医	nal husbandry and veterin	34	8	114-116	2007	sn.1671-7236.2007.08.039
2084	炎沙门菌引起食物中毒的	e of food poisoning caused	张冬民,丁新民,贾永强.	ongmin,Ding Xinmin,Jia Yo	中国微生态学杂志	inese Journal of Microecol	19	1	98	2007	n.1005-376X.2007.01.046
2085	融合术后都柏林沙门菌	infection after hip fusion	姜丽萍,于桂英.	Jiang Liping,Yu Guiying.	临床检验杂志	journal of clinical testing	25	2	95	2007	n.1001-764X.2007.02.043
2086	年湖州市食源性致病菌污	orne pathogenic bacteria c	月华,徐雪明,程平庆,查云	Xu Xueming,Cheng Pingqin	中国卫生检验杂志	ese Journal of Health Inspe	17	3	520538	2007	sn.1004-8685.2007.03.065
2087	况调查及VITEK微生物分	on in foodVITEKEvaluatio	黄雪珍,谭海芳,陈华.	Xuezhen,Tan Haifang,Che	热带医学杂志	journal of tropical medicine	7	3	263-265	2007	sn.1672-3619.2007.03.023
2088	00～2006年食源性致病菌	surveillance analysis of fo	友,李玉芳,崔龙,董芳,王滨	Yufang,Cui Long,Dong Fang	中国卫生检验杂志	ese Journal of Health Inspe	17	7	1255-1257	2007	sn.1004-8685.2007.07.046
2089	例血培养病原菌阳性结果	e results of pathogenic ba	郑利平,黎冬梅,甘泳江.	Liping,Li Dongmei,Gan Yon	应用预防医学	applied preventive medicin	13	1	53-54	2007	n.1673-758X.2007.01.019
2090	沙门菌感染180例诊治体会	180Experience in case diag	海波,黄莉梅,马真银,王冬	ang Limei,Ma Zhenyin,Wa	人民军医	people's military medicine	50	4	231-232	2007	sn.1000-9736.2007.04.028
2091	斑狼疮合并猪霍乱沙门菌	matosus combined with ch	谢君辉,雷小妹,胡绍先.	unhui,Lei Xiaomei,Hu Shao	中华风湿病学杂志	nese Journal of Rheumatol	11	11	702-704	2007	sn:1007-7480.2007.11.021
2092	农村D群沙门菌食物中毒	nvestigation and analysis o	肖汉平,杨浩,张菊华.	Hanping,Yang Hao,Zhang J	预防医学情报杂志	of Preventive Medicine Inf	23	1	85-86	2007	sn.1006-4028.2007.01.027
2093	艾滋病机会性感染临床特	aracteristics of AIDS oppor	松,吴欣华,陆铭,蒙艳,李	u Xinhua,Lu Ming,Meng Ya	卫生软科学	health soft sciences	21	6	513-516	2007	sn.1003-2800.2007.06.029
2094	由喜宴污染引起的沙门菌	caused by contamination	张国祥.	Zhang Guoxiang.	山西医药杂志（下半月版）	cal Journal (Second Half M	36	20	760	2007	-
2095	-2005年进口海产品中3种	orted seafood per year3Te	麻丽丹,王殿夫,金东权.	an,Wang Dianfu,Kim Dong	中国食品卫生杂志	hinese Food Hygiene Jour	19	3	254-255	2007	sn.1004-8456.2007.03.017
2096	宰生猪主要微生物学指标	ain microbiological indicat	魏建忠,王桂军,李春芬,张	ng,Wang Guijun,Li Chunfe	中国卫生检验杂志	ese Journal of Health Inspe	17	7	1191-1192,1221	2007	sn.1004-8685.2007.07.016
2097	2例血培养结果及耐药性	re results and drug resista	益明,黄孟启,卓晓娅,许少	Huang Mengqi,Zhuo Xiaoy	中国微生态学杂志	inese Journal of Microecol	19	2	223-224	2007	n.1005-376X.2007.02.045
2098	中学生肠道致病菌携带现	of intestinal pathogenic ba	,白丽,王晶,武有聪,申元	ng Jing,Wu Youcong,Shin Y	中国校医	Chinese school doctor	21	2	152-153	2007	sn.1001-7062.2007.02.010
2099	血培养病原菌分布及其耐	and drug resistance of pat	方梅,谷成祥,戴春梅.	Mei,Gu Chengxiang,Dai Ch	实用预防医学	ractical preventive medici	14	2	377-379	2007	sn.1006-3110.2007.02.043
2100	006年食品中沙门菌污染	monitoring of Salmonella c	均,周军波,熊燕,李佳,张	ou Junbo,Xiong Yan,Li Jia,Z	中国卫生检验杂志	ese Journal of Health Inspe	17	12	2266-2267	2007	sn.1004-8685.2007.12.058
2101	门菌病原学诊断及药物敏	diagnosis and drug sensit	萍,杨绍军,马真银,许爱国	ng Shaojun,Ma Zhenyin,Xu	中华传染病杂志	se Journal of Infectious Dis	25	6	374-376	2007	sn:1000-6680.2007.06.015
2102	感染甲型副伤寒沙门菌败	phi A sepsis after tooth ex	敖道畅.	Ao Daochang.	广州医学院学报	al of Guangzhou Medical C	35	5	62-62	2007	sn.1008-1836.2007.05.017
2103	脑脊液中检出都柏林沙门	ed in cerebrospinal fluid f	胡素侠,李艳玲.	Hu Suxia,Li Yanling.	蚌埠医学院学报	rnal of Bengbu Medical Co	32	3	352	2007	sn.1000-2200.2007.03.046
2104	门氏菌食物中毒27例临床	food poisoning27Case clin	徐丽坤,姜帆,康卫红.	Likun,Jiang Fan,Kang Weih	中国当代医学	inese contemporary medic	-	12	-	2007	-
2105	中毒样品中肠炎沙门菌分	of Salmonella Enteritidis fr	孙宏,关菲,李玉.	Sun Hong,Guan Fei,Li Yu.	中国公共卫生	Chinese public health	23	1	42	2007	sn:1001-0580.2007.01.072
2106	区食品中食源性致病菌及	bacteria and drug resista	,梁柏年,李占裕,陈子慧,	g Bainian,Li Zhanyu,Chen Z	疾病监测	disease surveillance	22	10	680-682	2007	sn.1003-9961.2007.10.012
2107	江北区食源性致病菌监测	odborne pathogenic bacte	金晓霞,费素定,裘立晓.	Xiaoxia,Fei Suding,Qiu Lix	中国卫生检验杂志	ese Journal of Health Inspe	17	9	1672-1673	2007	sn.1004-8685.2007.09.060

2108	型副伤寒68例临床流行病	dren68Clinical epidemiolog	周虹,周艳.	Zhou Hong,Zhou Yan.	遵义医学院学报	urnal of Zunyi Medical Colle	30	2	178-179	2007	sn.1000-2715.2007.02.032
2109	中重要食源性病原菌污染	foodborne pathogenic cont	,兰真,赵晋,薛晴,辛又川,	en,Zhao Jin,Xue Qing,Xin Y	现代预防医学	modern preventive medicin	34	20	3810-3812	2007	sn.1003-8507.2007.20.004
2110	甲型副伤寒沙门菌临床药	eptibility analysis of Salmon	段兴钧,章银娣,曹市成.	Xingjun,Zhang Yindi,Cao Shi	大理学院学报	Journal of Dali University	6	z1	238240	2007	sn.1672-2345.2007.z1.113
2111	分引起的沙门氏菌食物中	of salmonella food poisoni	柏峰,林坤立,黄志,梁德	g,Lin Kunli,Huang Zhi,Liang	实用预防医学	ractical preventive medicin	14	2	443-443	2007	sn.1006-3110.2007.02.076
2112	菌、志贺菌的血清学分型	sistance analysis of Salmon	蒋火刚,李树仁.	Jiang Huogang,Li Shuren.	贵州医药	Guizhou Medicine	31	6	561-562	2007	n.1000-744X.2007.06.055
2113	年临床分离病原菌谱及耐	inically isolated pathogenic	吴昌猛,陈泽慧,肖代敏,徐	ngmeng,Chen Zehui,Xiao D	遵义医学院学报	urnal of Zunyi Medical Colle	30	3	297-300	2007	sn.1000-2715.2007.03.026
2114	术后并发鼠伤寒沙门氏菌	by Salmonella typhimurium	刘倩,张淑凤,杨小红.	an,Zhang Shufeng,Yang Xia	中华护理杂志	Chinese Nursing Journal	42	9	798-799	2007	-
2115	门菌和志贺菌的菌型分布及	eptibility analysis of Salmo	何梅芳,钟学庆.	He Meifang,Zhong Xueqing	职业与健康	Occupation and health	23	23	2180-2181	2007	sn.1004-1257.2007.23.035
2116	基在口岸从业人群沙门菌	hromogenic culture media	,章琪,张晓航,方筠,沈小明	Zhang Xiaohang,Fang Jun,S	现代预防医学	modern preventive medicin	34	24	4648-4649	2007	sn.1003-8507.2007.24.020
2117	中肠炎沙门菌分子生物学	cal characteristics of Salmo	俊英,付有荣,郭清莲,齐小	g,Fu Yourong,Guo Qinglian	中国公共卫生	Chinese public health	23	10	1227-1228	2007	sn:1001-0580.2007.10.030
2118	型副伤寒沙门菌致菌血症	used by Salmonella Paratyp	马昌义,杨祖蓉.	Ma Changyi,Yang Zurong.	实用医学杂志	ournal of Practical Medicin	23	9	1394	2007	sn.1006-5725.2007.09.093
2119	患者血液中病原菌分布及	g resistance of pathogenic	陆文香,徐卫东,吴培南.	enxiang,Xu Weidong,Wu P	江苏大学学报（医学版）	f Jiangsu University (Medica	17	1	71-73	2007	sn.1671-7783.2007.01.020
2120	沙门菌的鉴定及耐药分析	nd drug resistance analysis	黄明海,陈群英.	uang Minghai,Chen Qunyin	实用药物与临床	ractical medicine and clinic	10	6	380	2007	sn.1673-0070.2007.06.026
2121	州市非发酵性豆制品卫生	atus of non-fermented soy	秀丽,廖兴广,胡巅,高葆真	o Xingguang,Hu Dian,Gao B	河南预防医学杂志	n Journal of Preventive Me	18	2	136-137	2007	sn.1006-8414.2007.02.028
2122	型伤寒并发阑尾穿孔1例	nplicated by appendiceal pe	陈明春.	Chen Mingchun.	中国校医	Chinese school doctor	21	1	6,15	2007	sn.1001-7062.2007.01.066
2123	学校食堂卫生学调查与	Research on Hygiene in Ca	沈永康,俞惠飞.	Shen Yongkang,Yu Huifei.	中国公共卫生管理	ese public health managem	23	1	84-86	2007	sn.1001-9561.2007.01.055
2124	沙门菌和奇异变形杆菌引起	aused by Salmonella Enter	群文,李维克,李博,曾小思	unwen,Li Weike,Li Bo,Zeng	现代预防医学	modern preventive medicin	34	12	2299-2300	2007	sn.1003-8507.2007.12.042
2125	群携带沙门菌菌型分布及	sceptibility test of Salmone	邹跃玲.	Zou Yueling.	检验医学与临床	boratory medicine and clini	4	10	954-955	2007	sn.1672-9455.2007.10.022
2126	物中毒事件的快速诊断与	pathogen analysis of a foo	林一曼,石晓路,王冰.	Yiman,Shi Xiaolu,Wang Bi	中国卫生检验杂志	ese Journal of Health Inspe	17	5	919-920	2007	sn.1004-8685.2007.05.070
2127	肉类大肠埃希氏菌与沙门	oli and Salmonella contami	句立言,黄明越,孙宏.	statement,Huang Mingyue,	中国保健	China health care	15	11	9-10	2007	-
2128	的病原菌监测及致泻性大	ty test of diarrheagenic Esc	顾松叶,石亚素,龚红霞.	Songye,Shi Yasu,Gong Hong	中国卫生检验杂志	ese Journal of Health Inspe	17	9	1670-1671	2007	sn.1004-8685.2007.09.059
2129	部分腹泻患者沙门氏菌	tion in some diarrhea patie	佳莹,徐枫,杨兰萍,刘淮	g,Xu Feng,Yang Lanping,Liu	社区卫生保健	community health care	6	6	425-426	2007	-
2130	食品从业人员肠道沙门菌	Salmonella carriage among	张成河.	Zhang Chenghe.	海峡预防医学杂志	s Journal of Preventive Me	13	6	46	2007	sn.1007-2705.2007.06.025
2131	泳在沙门菌食物中毒溯源	eld gel electrophoresis in t	黄薇,王冰,林一曼,贺连华	Wang Bing,Lin Yiman,He Li	疾病控制杂志	journal of disease control	11	5	502-504	2007	sn.1674-3679.2007.05.018
2132	阳性87例副伤寒(乙)的临	e87Paratyphoid fever(Seco	汤银江,张春梅,陈春.	njiang,Zhang Chunmei,Che	医学新知杂志	Medical News Magazine	17	5	306-306	2007	sn.1004-5511.2007.05.025
2133	肠炎沙门氏菌食物中毒调	is of a case of Salmonella E	,李莺,马骏,张卉,袁宏祥,	Ma Jun,Zhang Hui,Yuan Ho	公共卫生与预防医学	Health and Preventive Me	18	3	65-65	2007	sn.1006-2483.2007.03.026
2134	市感染性腹泻病原菌调查	pathogenic bacteria of inf	朱晓云.	Zhu Xiaoyun.	职业与健康	Occupation and health	23	15	1321-1323	2007	sn.1004-1257.2007.15.025
2135	门氏茵的血清型鉴定和耐	g resistance study of salm	富华,宋立,赵晖,芦秀梅,宁	ua,Song Li,Zhao Hui,Lu Xiu	中国家禽	Chinese poultry	29	1	18-19,36	2007	sn.1004-6364.2007.01.005
2136	伤寒沙门菌的鉴定及药敏	g susceptibility analysis of S	许磊,李红.	Xu Lei,Li Hong.	医学检验与临床	edical Laboratory and Clini	18	2	98	2007	sn.1673-5013.2007.02.061
2137	年北京市顺义区食品致病	lysis of food pathogenic ba	秀峰,李玉堂,梁和平,甄国	,Li Yutang,Liang Heping,Zh	中国食品卫生杂志	hinese Food Hygiene Journ	19	6	534-536	2007	sn.1004-8456.2007.06.016
2138	务人员大便培养伤寒沙门	e staff member was positiv	刘兰花.	Liu Lanhua.	总装备部医学学报	cine of the General Armam	-	2	123	2007	-
2139	秦皇岛市食源性疾病病原	nation status of foodborne	树民,代静林,冯艳洁,刘兰	min,Dai Jinglin,Feng Yanjie,	中国卫生检验杂志	ese Journal of Health Inspe	17	6	1090-1091	2007	sn.1004-8685.2007.06.062
2140	鼠伤寒沙门菌引起的食物	oisoning caused by Salmon	王润叶,王艳霞,韩君.	g Runye,Wang Yanxia,Han	中国煤炭工业医学杂志	Journal of Coal Industry M	10	4	463-463	2007	sn.1007-9564.2007.04.086
2141	伦敦沙门菌引起食物中毒	e of food poisoning caused	容,蒋德媚,董雪,詹珍洁,金	g Demei,Dong Xue,Zhan Zh	中国热带医学	Chinese tropical medicine	7	11	2170-2170	2007	sn.1009-9727.2007.11.113
2142	村自办婚宴食物中毒的调	on food poisoning at a rur	刘永建,张奇,张英.	Yongjian,Zhang Qi,Zhang Y	海峡预防医学杂志	s Journal of Preventive Me	13	6	61	2007	sn.1007-2705.2007.06.037
2143	患儿血中分离出1株伦敦	blood of children with seps	照,郭思建,李宪,张晋湘,李	o Sijian,Li Xian,Zhang Jinxia	中华医院感染学杂志	ournal of Hospital Infectiou	17	8	1023-1023	2007	sn:1005-4529.2007.08.073
2144	食物中毒实验室病原体查	s of pathogens in a food po	,吴振宏,谭雪芳,廖鹏飞,	Zhenhong,Tan Xuefang,Lia	中国热带医学	Chinese tropical medicine	7	2	307320	2007	sn.1009-9727.2007.02.073

2145	门氏菌噬菌体的分型及耐	ance analysis of Salmonella	王鹤林,赵琼,赵宗越.	Helin,Zhao Qiong,Zhao Zor	现代实用医学	modern practical medicine	19	12	937-938	2007	sn.1671-0800.2007.12.004
2146	甲型副伤寒132例临床分析	phoid A132Case clinical ar	沈显元,喻春红,陈金香.	nyuan,Yu Chunhong,Chen	临床内科杂志	nal of Clinical Internal Med	24	10	714-715	2007	sn.1001-9057.2007.10.024
2147	/体/沙/门/氏/菌/污/染/的/	/Door/clan/bacteria/Sewag	郑克新.	Zheng Kexin.	中国兽医杂志	Chinese Veterinary Journal	43	1	51-51	2007	sn.0529-6005.2007.01.026
2148	布利丹沙门菌引起的食物	soning caused by Salmonel	向小兵,杜枝国,郑玲.	Xiaobing,Du Zhiguo,Zheng	预防医学论坛	Preventive Medicine Forum	13	8	封3	2007	sn.1672-9153.2007.08.045
2149	发酵豆制品微生物污染调	amination of commercially	振柱,黄兆勇,蒋震羚,方志	haoyong,Jiang Zhenling,Fan	实用预防医学	ractical preventive medicin	14	4	1130-1131	2007	sn.1006-3110.2007.04.067
2150	面米食品中微生物污染状	ontamination in quick-froz	建潮,袁丹茅,何云,曹春	,Yuan Danmao,He Yun,Cao	河南预防医学杂志	n Journal of Preventive Me	18	4	305320	2007	sn.1006-8414.2007.04.034
2151	沙门菌感染的临床变化研	inical changes of Salmonel	汪相武.	Wang Xiangwu.	中国微生态学杂志	inese Journal of Microecolo	19	2	206	2007	n.1005-376X.2007.02.047
2152	属对常用抗菌药物敏感性	of Salmonella spp. to comr	张静萍,褚云卓,陈佰义.	Jingping,Chu Yunzhuo,Cher	中华医院感染学杂志	ournal of Hospital Infectiou	17	10	1296-1298	2007	sn:1005-4529.2007.10.043
2153	由2种病原菌引起的食物	poisoning caused by patho	刘玉兰.	Liu Yulan.	预防医学论坛	Preventive Medicine Forum	13	11	1042-1043	2007	sn.1672-9153.2007.11.043
2154	沙门菌引发食物中毒的	ase of food poisoning caus	王琳娜.	Wang Linna.	中国卫生检验杂志	ese Journal of Health Inspe	17	7	1299-1299	2007	sn.1004-8685.2007.07.068
2155	病猪肉引起的细菌性食物	cterial food poisoning caus	冉桂芬,郭庆安,王琳,钟涛	en,Guo Qingan,Wang Lin,Zh	职业与健康	Occupation and health	23	23	2170-2171	2007	sn.1004-1257.2007.23.027
2156	寒沙门菌引起化脓性胆管	ent cholangitis caused by S	王明兴,唐松军.	Vang Mingxing,Tang Songju	医学检验与临床	edical Laboratory and Clini	18	2	100	2007	sn.1673-5013.2007.02.063
2157	坦利沙门菌引起食物中毒	e of food poisoning caused	骆坤.	Luo Kun.	职业与健康	Occupation and health	23	16	1416	2007	sn.1004-1257.2007.16.022
2158	伤寒沙门氏菌引起的食物	f food poisoning caused by	任智慧,温真.	Ren Zhizhi,Wen Zhen.	地方病通报	endemic disease notificatio	22	3	51	2007	sn.1000-3711.2007.03.082
2159	沙门氏菌引起的食物中毒	case of food poisoning ca	赵丽娜,梁惠宁,沈爱军.	Lina,Liang Huining,Shen A	应用预防医学	applied preventive medicin	13	4	235	2007	n.1673-758X.2007.04.030
2160	所及食品卫生从业人员健	lts of public places and foo	朱晓云.	Zhu Xiaoyun.	社区医学杂志	urnal of Community Medici	5	3	7-9	2007	sn.1672-4208.2007.03.004
2161	门氏菌群体性食物中毒的	t on a case of salmonella m	俊才,陈琦元,李观前,陈	i,Chen Qiyuan,Li Guanqian	现代保健·医学创新研究	alth care·Medical innovati	4	26	162	2007	-
2162	吉林省食品污染物监测结	contaminant monitoring r	桂华,龚云伟,杨红,孔祥云	ong Yunwei,Yang Hong,Kon	中国卫生检验杂志	ese Journal of Health Inspe	17	2	309-310,324	2007	sn.1004-8685.2007.02.050
2163	5～2006年食品致病菌污	nual survey on food pathog	杨娟,周静,杨海玉,蔡震.	an,Zhou Jing,Yang Haiyu,C	中国热带医学	Chinese tropical medicine	7	8	1482-1482,1497	2007	sn.1009-9727.2007.08.112
2164	市食源性致病菌污染状况	on status of foodborne pat	许滋宁,周雪萍.	Xu Zining,Zhou Xueping.	职业与健康	Occupation and health	23	1	24-24	2007	sn.1004-1257.2007.01.021
2165	居民小区、高校中水沙门	water in some residential ar	史燕萍,钱城,狄飞.	hi Yanping,money city,Di Fe	现代预防医学	modern preventive medicin	34	3	558-559,569	2007	sn.1003-8507.2007.03.061
2166	学校水源污染事件的调查	analysis of a school water	柳彦峰.	Liu Yanfeng.	医学动物防制	medical animal control	23	6	452-453	2007	sn.1003-6245.2007.06.029
2167	幼儿春季腹泻的病原学探	of spring diarrhea in infan	永松,刘权胜,张立新,官	ng,Liu Quansheng,Zhang L	中国卫生检验杂志	ese Journal of Health Inspe	17	3	483-484	2007	sn.1004-8685.2007.03.046
2168	近年食品行业从业人员传	among food industry emplo	王秀兰.	Wang Xiulan.	职业与健康	Occupation and health	23	15	1326-1327	2007	sn.1004-1257.2007.15.028
2169	05年食品中食源性疾病致	survey of foodborne diseas	,张治芳,陈克功,李建坡,	g Zhifang,Chen Kegong,Li J	现代预防医学	modern preventive medicin	34	6	1090-1091,1097	2007	sn.1003-8507.2007.06.040
2170	发生肠炎沙门菌食物中毒	almonella Enteritidis food	,景钦隆,何洁仪,毛新武,	Qinlong,He Jieyi,Mao Xinwu	中国食品卫生杂志	hinese Food Hygiene Journ	19	4	354-356	2007	sn.1004-8456.2007.04.024
2171	售食品食源性致病菌污染	borne pathogenic bacteria	,李钏华,张欣强,庞杏林,	nhua,Zhang Xinqiang,Pang	热带医学杂志	journal of tropical medicine	7	8	804-806	2007	sn.1672-3619.2007.08.031
2172	液中分离出1株甲型副伤	om chest pus1Salmonella p	卫萍,邵海枫,李珍大,张小	g,Shao Haifeng,Li Zhenda,Z	医学研究生学报	l of Medical Postgraduate S	20	9	1006	2007	sn.1008-8199.2007.09.032
2173	S和EMB类群细菌动态监	CBSandEMBDynamic mo	德胜,潘子强,曾延辉,朱义	eng,Pan Ziqiang,Zeng Yanh	海洋学报（中文版）	of Oceanography (Chinese	29	1	105-110	2007	sn:0253-4193.2007.01.015
2174	伤寒沙门菌对抗菌药物的	nalysis of antimicrobial resi	顾本思,李方,木塔力甫·托	r,Gu Bensi,Li Fang,Mutalifu	地方病通报	endemic disease notificatio	22	6	16-18,21	2007	sn.1000-3711.2007.06.006
2175	城乡自备井水质微生物污	ation of water quality in urb	吴建根,岑迪,马旭亮.	Vu Jiangen,zendi,Ma Xulian	中国卫生检验杂志	ese Journal of Health Inspe	17	7	1273-1274	2007	sn.1004-8685.2007.07.055
2176	食服务人员沙门菌携带率	ella carrying rate among ca	牟成惠,陈淑云.	Mou Chenghui,Chen Shuyun	中国热带医学	Chinese tropical medicine	7	7	12311241	2007	sn.1009-9727.2007.07.089
2177	3年间市售熟肉制品卫生	atus of cooked meat produ	曾晓犁,左金容,何君茹,傅	Xiaoli,Zuo Jinrong,He Junru,	现代预防医学	modern preventive medicin	34	23	4502-4502	2007	sn.1003-8507.2007.23.048
2178	霍乱沙门氏菌的快速分离	nd identification of Salmon	,郭爱珍,贾爱卿,刘维红,	hen,Jia Aiqing,Liu Weihong,	畜牧与兽医	Husbandry and Veterinary M	39	2	8-11	2007	sn.0529-5130.2007.02.003
2179	由喜宴污染引起的沙门菌	caused by contamination	张国祥.	Zhang Guoxiang.	山西医药杂志（下半月版）	cal Journal (Second Half Mo	36	10	760	2007	0253-9926-B.2007.10.075
2180	康从业人员沙门菌感染菌	drug resistance among hea	薏妲,姚宗蓓,吴凯,宋黎	da,Yao Zongbei,Wu Kai,Son	上海预防医学	hanghai Preventive Medicir	19	5	222-223	2007	sn.1004-9231.2007.05.008
2181	株甲型副伤寒沙门氏菌的	t from a strain of Salmonel	弓耀忠,赵海霞.	Gong Yaozhong,Zhao Haixia	大众标准化	mass standardization	-	S2	48-49	2007	-

2182	沙门氏菌临床分离株的耐药	linical isolates of Salmonel	恒敏,李昌文,陈洪岩,刘怀	Li Changwen,Chen Hongya	中国兽医杂志	Chinese Veterinary Journal	43	1	31-32	2007	sn.0529-6005.2007.01.015
2183	6年汕头市食源性致病菌	dy on foodborne pathoger	,林洁敏,蔡亚军,吴耀波,美	emin,Cai Yajun,Wu Yaobo,	疾病监测	disease surveillance	22	5	302-304	2007	sn.1003-9961.2007.05.007
2184	基因在鸡源沙门氏菌中的	line resistance genes in Sa	彦红,吴双,焦新安,潘志明	hhong,Wu Shuang,Jiao Xina	中国家禽	Chinese poultry	29	9	10-12,18	2007	sn.1004-6364.2007.09.003
2185	地牧草病原性微生物污染	status of pasture pathogen	玲,胡晓宁,苏诚玉,余群秀	,Hu Xiaoning,Su Chengyu,	中国卫生检验杂志	ese Journal of Health Inspe	17	11	2074-2075	2007	sn.1004-8685.2007.11.066
2186	-2006年细菌性食物中毒流	demiological analysis of ba	符立龙.	Fu Lilong.	海峡预防医学杂志	s Journal of Preventive Me	13	3	41-42	2007	sn.1007-2705.2007.03.018
2187	引起学生肠炎沙门菌食物中	ng caused by Salmonella E	陈志铨.	Chen Zhiquan.	中国学校卫生	Chinese school health	28	11	1041-1041	2007	sn.1000-9817.2007.11.045
2188	办丧事聚餐引起沙门菌食物	ella food poisoning caused	李永宏,赵有林.	Li Yonghong,Zhao Youlin.	中国预防医学杂志	se Journal of Preventive Me	8	2	155-156	2007	sn.1009-6639.2007.02.034
2189	仑区1985-2005年伤寒流行	05Analysis of annual epide	肖伟,贺佩.	Xiao Wei,He Pei.	中国预防医学杂志	se Journal of Preventive Me	8	1	24-26	2007	sn.1009-6639.2007.01.007
2190	儿猪霍乱沙门菌感染临床	lla choleraesuis infection ir	朱余德.	Zhu Yude.	基层医学论坛	Primary Medicine Forum	11	18	781	2007	sn.1672-1721.2007.18.012
2191	年8426株腹泻病原菌的分	ion and drug resistance of	,崔恩博,郭桐生,鲍春梅,对	nbo,Guo Tongsheng,Bao Ch	解放军检验医学杂志	ory Medicine of the People	3	1	52-55	2007	-
2192	鼠伤寒沙门氏菌食物中毒	ase of Salmonella Typhimu	,莫若琦,王孔前,施向东,阝	oqi,Wang Kongqian,Shi Xia	应用预防医学	applied preventive medicin	13	4	封3	2007	n.1673-758X.2007.04.033
2193	晾晒过程中几种常见致病	athogenic bacteria during t	孟岳成,任人栋.	eng Yuecheng,Ren Rendon	食品工业科技	Food Industry Technology	-	1	82-83,86	2007	sn.1002-0306.2007.01.022
2194	细胞增生李斯特菌及金黄	lla, Listeria monocytogene	周丽萍,巢国祥.	Zhou Liping,Chao Guoxiang	中国卫生检验杂志	ese Journal of Health Inspe	17	3	505-507	2007	sn.1004-8685.2007.03.057
2195	常见病原菌分布及耐药性	drug resistance of commor	施巧霞,李晓非,杨惠仙.	Qiaoxia,Li Xiaofei,Yang Hui	预防医学情报杂志	of Preventive Medicine Info	23	2	196-199	2007	sn.1006-4028.2007.02.022
2196	农业冷荤间熟肉类制品卫生	at products in cold meat ro	孙伟,刘国蓉,闫革彬.	n Wei,Liu Guorong,Yan Geb	中国卫生检验杂志	ese Journal of Health Inspe	17	11	2059-2060	2007	sn.1004-8685.2007.11.059
2197	巴山村甲型副伤寒的暴发	utbreak of paratyphoid fev	永泰,牛冬花,他平芬,常月	tai,Niu Donghua,tapfen,Ch	现代预防医学	modern preventive medicin	34	23	4574-4575	2007	sn.1003-8507.2007.23.087
2198	门氏菌引起群体食物中毒	of mass food poisoning ca	紫杭,邓志爱,李孝权,梁晅,	eng Zhiai,Li Xiaoquan,Liang	现代医学仪器与应用	medical instruments and ap	-	6	58-60	2007	-
2199	肠炎沙门菌引起的食物中	poisoning caused by Salmo	魏红琴.	Wei Hongqin.	江苏预防医学	Jiangsu Preventive Medicin	18	1	86	2007	sn.1006-9070.2007.01.056
2200	菌的分离与鉴定以及PCR	nella choleraesuis andPCR	诗,郭万柱,徐志文,李雯,王	Wanzhu,Xu Zhiwen,Li We	安徽农业科学	Anhui Agricultural Sciences	35	20	6020-6023	2007	sn.0517-6611.2007.20.063
2201	检测海产品中甲型副伤寒	simultaneous detection o	,金春光,沈玄艺,章丹阳,天	guang,Shen Xuanyi,Zhang D	中国卫生检验杂志	ese Journal of Health Inspe	17	8	1464-1465	2007	sn.1004-8685.2007.08.051
2202	沙门菌污染酱牛肉引起的食	used by salmonella contam	张其成.	Zhang Qicheng.	预防医学论坛	Preventive Medicine Forum	13	4	382-382	2007	sn.1672-9153.2007.04.048
2203	沙门菌F群交叉凝集的阴沟	ross-agglutinating Enterob	徐建设,吴海,蒋万扬,邱永	Hai,Jiang Wanyang,Qiu Yon	中国卫生检验杂志	ese Journal of Health Inspe	17	8	1492-1493	2007	sn.1004-8685.2007.08.065
2204	省龙岩市食源性致病菌监	foodborne pathogenic bac	前进,张月花,何云,廖琳虹	jin,Zhang Yuehua,He Yun,L	疾病监测	disease surveillance	22	7	447-448	2007	sn.1003-9961.2007.07.007
2205	城区4类食品食源性致病	ontamination status of foo	许晓玲,赵春玲,吴伟.	iaoling,Zhao Chunling,Wu	现代预防医学	modern preventive medicin	34	9	1670-1671,1675	2007	sn.1003-8507.2007.09.024
2206	由圣保罗沙门菌引起的食物	poisoning caused by Salm	王章云,朱晓兰,万锦云,褚	ngyun,Zhu Xiaolan,Wan Jir	中国卫生检验杂志	ese Journal of Health Inspe	17	11	20832100	2007	sn.1004-8685.2007.11.071
2207	液培养病原菌的分布及耐药	esistance analysis of blood	,伍严安,陈发林,胡辛兰,傅	Wu Yanan,Chen Falin,Hu Xi	福建医药杂志	Fujian Medical Journal	29	4	19-20	2007	sn.1002-2600.2007.04.010
2208	源性致病菌及食源性疾病	ne pathogenic bacteria and	亚军,吴耀波,林奕华,林洁	un,Wu Yaobo,Lin Yihua,Lin	中国热带医学	Chinese tropical medicine	7	5	804-805	2007	sn.1009-9727.2007.05.076
2209	疆麦盖提县伤寒暴发疫情	of typhoid fever outbrea	冯玉萍;阿地江·买买提;	g Yuping;Adijiang·Maim	地方病通报	ndemic disease notificati	-	6	107	2007	cnki.jbyfkztb.2007.06.046
2210	校水型伤寒暴发的调查报	waterborne typhoid feve	王卫民;孙朝辉;	ang Weimin;Sun Chaoh	咸宁学院学报(医学版)	Xianning University(medi	-	6	537-538	2007	ki.2095-4646.2007.06.004
2211	暴发疫情的消毒处理措施	fectiveness report on an	陆备芳;马焰;	Lu Beifang;Ma Yan;	医学动物防制	medical animal control	-	10	741-742	2007	-
2212	学校伤寒暴发的流行病学	nalysis of typhoid fever o	殷凯;黎明强;刘金纪;	Kai;Liming Qiang;Liu Ji	现代预防医学	modern preventive medici	-	13	2559-2560	2007	-
2213	型副伤寒暴发疫情流行因	ic factors of an outbreak	叶晓军;	Ye Xiaojun;	中国预防医学杂志	e Journal of Preventive M	-	3	257-259	2007	-
2214	寺区甲型副伤寒暴发流行	yphoid A outbreak in Liuz	胡春晔;扬春莲;	u Chunye;Yang Chunlian	现代医药卫生	modern medicine and heal	-	9	1365-1366	2007	-
2215	学校甲型副伤寒暴发的调	n outbreak of paratypho	东;刘兴富;廖泽胜;黄燕;缪	ngfu;Liao Zesheng;Huan	海峡预防医学杂志	Journal of Preventive M	-	2	49-50	2007	-
2216	市感染性腹泻细菌病原学	results of bacterial etiolog	张晓宇,吴守芝,李焕枝.	Xiaoyu,Wu Shouzhi,Li Hu	职业与健康	Occupation and health	24	4	352-353	2008	sn.1004-1257.2008.04.029
2217	006南京市食品致病菌污	ood pathogenic bacteria c	江晓,丁洁,陈晓蔚.	g Xiao,Ding Jie,Chen Xiaov	预防医学情报杂志	of Preventive Medicine Info	24	1	16-18	2008	sn.1006-4028.2008.01.006
2218	区61株鼠伤寒沙门菌的耐药	f drug resistance of Salmo	自镛,李丽,朱旭慧,张蓓,林	ong,Li Li,Zhu Xuhui,Zhang	中国感染与化疗杂志	urnal of Infection and Che	8	1	47-49	2008	sn:1009-7708.2008.01.011

2219	食品化学污染物和食源性	llutants and foodborne pat	志强,王大华,张颖,朱风	iang,Wang Dahua,Zhang Y	首都公共卫生	capital public health	2	5	211-214	2008	sn.1673-7830.2008.05.004
2220	年广州市108起食物中毒	8Analysis of pathogenic ba	陈妤,蒋卓勤.	Chen Yu,Jiang Zhuoqin.	中国卫生检验杂志	ese Journal of Health Inspe	18	3	485-487	2008	sn.1004-8685.2008.03.041
2221	食品污染物监测网络实验	tection Situation in Food C	王桂侠,温静,吴占国.	uixia,Gentle and quiet,Wu	中国卫生检验杂志	ese Journal of Health Inspe	18	3	564-564,569	2008	sn.1004-8685.2008.03.081
2222	食品中食源性致病菌污染	tus of foodborne pathoge	吕斌,李启泉.	Lu Bin,Li Qiquan.	中国卫生检验杂志	ese Journal of Health Inspe	18	1	135-137	2008	sn.1004-8685.2008.01.061
2223	蛋内外沙门氏菌和大肠杆	lla and E. coli inside and o	凯,曲鲁江,许月英,潘孝	qulujiang,Xu Yueying,Pan X	中国动物检疫	China Animal Quarantine	25	7	41-41	2008	n.1005-944X.2008.07.019
2224	炎沙门菌分离株的PFGE	und in some foods in Chin	,刘秀梅,赵熙,吴婷,崔志	umei,Zhao Xi,Wu Ting,Cui	中国食品卫生杂志	hinese Food Hygiene Journ	20	5	385-388	2008	sn.1004-8456.2008.05.001
2225	包头市感染性腹泻细菌病	of bacterial etiology of inf	杨永华,李秋梅,常勇.	Yonghua,Li Qiumei,Chang	职业与健康	Occupation and health	24	12	1177-1178	2008	sn.1004-1257.2008.12.026
2226	泻病原菌的分离鉴定及药	g susceptibility test of path	安益龙,王返里.	An Yilong,Wang Huili.	中国兽医杂志	Chinese Veterinary Journal	44	10	45-46	2008	sn.0529-6005.2008.10.023
2227	伤寒沙门菌62株耐药性临	yphi A62Clinical analysis of	朱火明.	Zhu Huoming.	中国乡村医药	Chinese rural medicine	15	11	49	2008	sn.1006-5180.2008.11.043
2228	度非伤寒沙门菌监测及病	e of non-typhoid Salmone	陈嘉琳,柯昌文,郭川,曹海	n,Ke Changwen,Guo Chua	中华流行病学杂志	inese Journal of Epidemiol	29	12	1199-1203	2008	sn:0254-6450.2008.12.007
2229	株圣地亚哥沙门氏菌(Sa	an Diego isolated from blo	国君,童武林,吴晓华,张	n,Tong Wulin,Wu Xiaohua	医学检验与临床	edical Laboratory and Clini	19	2	114	2008	sn.1673-5013.2008.02.061
2230	办喜宴引起沙门氏菌食物	a food poisoning caused b	黄继贵,章光斌.	uang Jigui,Zhang Guangbi	现代预防医学	nodern preventive medicin	35	9	17271730	2008	sn.1003-8507.2008.09.055
2231	感染性腹泻病原菌监测及	of pathogenic bacteria in i	凤琴,王勇,孙新婷,王贵	Wang Yong,Sun Xinting,W	中国临床药理学杂志	e Journal of Clinical Pharm	24	4	303-306	2008	sn.1001-6821.2008.04.004
2232	公共场所服务行业从业	ees in catering and public s	陆龙,陈若恒,周晓敏.	ng,Chen Ruoheng,Zhou Xia	华南预防医学	th China Preventive Medi	34	6	64-65	2008	sn.1671-5039.2008.06.025
2233	鼠伤寒沙门氏菌食物中	a typhimurium food poiso	王兵.	Wang Bing.	泸州医学院学报	rnal of Luzhou Medical Col	31	6	656-657	2008	sn.1000-2669.2008.06.021
2234	007年沙门菌主动监测及	Salmonella active surveilla	,韩艳青,申玉学,王英豪,	ing,Shen Yuxue,Wang Ying	实用预防医学	ractical preventive medicin	15	5	1363-1368	2008	sn.1006-3110.2008.05.016
2235	共场所从业人员沙门菌携	e among employees in pub	徐梦媚,吴鹏,浦敏杰.	Mengmei,Wu Peng,Pu Min	中华现代临床医学杂志	Journal of Modern Clinical	6	8	-	2008	-
2236	2005-2007年食源性致病	07Annual Foodborne Path	黄谷孙,黄忠生.	ng Gusun,Huang Zhongsh	中国食品卫生杂志	hinese Food Hygiene Journ	20	4	301-303	2008	sn.1004-8456.2008.04.005
2237	食物中毒典型案例分析	ort on a typical case of foo	木哈什·木哈提.	muhash·muhati.	中外健康文摘·医药月刊	Foreign Health Digest·Med	5	7	609	2008	-
2238	平顶山市7家大酒店卫生	7Survey and Analysis on Hy	王轶,罗学东,席慧霞.	ang Yi,Luo Xuedong,Xi Huix	河南预防医学杂志	n Journal of Preventive Me	19	2	135-135	2008	sn.1006-8414.2008.02.029
2239	刺液中分离出肠炎沙门	nteritidis isolated from kn	王少增,侯俊环.	ang Shaozeng,Hou Junhua	医学检验与临床	edical Laboratory and Clini	19	3	132	2008	sn.1673-5013.2008.03.065
2240	类沙门氏菌菌型分布情	tion of Salmonella strains i	董芳,姜义,苗雨.	Dong Fang,Jiang Yi,Miao Yu	中华中西医学杂志	f Traditional Chinese and	6	6	-	2008	-
2241	食源性沙门菌菌型分布及	and drug susceptibility res	,陈燕梅,王红,张正东,许	i,Wang Hong,Zhang Zheng	检验医学与临床	boratory medicine and clin	5	23	1434-1434,1436	2008	sn.1672-9455.2008.23.014
2242	菌的耐药表型分析及其耐	f pathogenic Salmonella a	魏建忠,张玮,王强,谢玉洁	g,Zhang Wei,Wang Qiang,	中国家禽	Chinese poultry	30	14	26-28	2008	-
2243	北京市西城区食品致病菌	ogenic bacteria contamina	王永全,张晶波,王利萍,崔	quan,Zhang Jingbo,Wang L	中国自然医学杂志	ese Journal of Natural Med	10	5	337-340	2008	-
2244	门菌致慢性骨髓炎反复发	nic osteomyelitis caused by	邵海枫,王卫萍,史利宁,张	feng,Wang Weiping,Shi Lin	中华传染病杂志	se Journal of Infectious Dis	26	5	312	2008	sn:1000-6680.2008.05.016
2245	从业人员肠道致病菌检测	acteria detection results fo	帅慧群,赵雪琴,商晓春.	uiqun,Zhao Xueqin,Shang X	中国卫生检验杂志	ese Journal of Health Inspe	18	4	688-689	2008	sn.1004-8685.2008.04.048
2246	童血培养的病原菌分布及	e of pathogenic bacteria in	,马东礼,赵瑞珍,王红梅,	ngli,Zhao Ruizhen,Wang H	中国热带医学	Chinese tropical medicine	8	8	1335-1336,1347	2008	sn.1009-9727.2008.08.025
2247	伤寒、副伤寒暴发的流	outbreak of foodborne ty	章,徐景野,毛国华,董红军,	ingye,Mao Guohua,Dong H	疾病监测	disease surveillance	23	7	420-421,441	2008	sn.1003-9961.2008.07.009
2248	市豆芽行业卫生质量现状	s of health quality of bean	胡君平,郑天柱,李新庆.	nping,Zheng Tianzhu,Li Xin	现代预防医学	nodern preventive medicin	35	21	4250-4252	2008	sn.1003-8507.2008.21.063
2249	铁路局站车供应食品食源	athogenic bacteria in food	李廷栋.	Li Tingdong.	中国预防医学杂志	se Journal of Preventive Me	9	8	742-744	2008	sn.1009-6639.2008.08.013
2250	沙门氏菌肠炎调查报告	gation reports on cases of s	滕盘棋,李玉厚.	Tengpanqi,Li Yuhou.	中国初级卫生保健	primary health care in chin	22	7	59	2008	n.1001-568X.2008.07.032
2251	沙门菌污染引起的食物	isoning caused by Salmone	何政.	He Zheng.	职业与健康	Occupation and health	24	3	233-233	2008	sn.1004-1257.2008.03.019
2252	区128例细菌性痢疾的病	128A study on the etiolog	G JIAN-PING,田国保,胥	AN-PING,Tian Guobao,Xu	中国民康医学（下半月）	ealth medicine (second hal	20	7	1537-1538	2008	-
2253	区奥运相关餐饮业食源	ria in Olympic-related cate	玲,苏君,伊璇,赵伟,孙卫,	,Su Jun,Yixuan,Zhao Wei,Su	首都公共卫生	capital public health	2	6	252-255	2008	sn.1673-7830.2008.06.002
2254	京市食品中食源性致病菌	on status of foodborne pat	陈洋,江晓,马连凯.	en Yang,Jiang Xiao,Ma Lian	现代预防医学	nodern preventive medicin	35	19	3811-3813,3815	2008	sn.1003-8507.2008.19.059
2255	沙门氏菌引起食物中毒	e of food poisoning cause	李怀玉.	Li Huaiyu.	中国医药指南	Chinese Medicine Guide	6	10	86-87	2008	sn.1671-8194.2008.10.057

2256	公共场所从业人员肠道沙	onella among workers in ca	抗,田桢干,方筠,阎俊,章琪,	enqian,Fang Jun,Yan Jun,Z	中国病原生物学杂志	ese Journal of Pathogen Bi	3	1	80-81	2008	sn.1673-5234.2008.01.027
2257	氏菌骨髓炎并巨大腰大肌	e salmonella osteomyelitis	军,王健,周传利,陈晓亮,冯	Jian,Zhou Chuanli,Chen Xia	中华骨科杂志	hinese Journal of Orthopedi	28	12	1056-1057	2008	sn:0253-2352.2008.12.021
2258	岗地区沙门菌的菌群分布	e of Salmonella bacteria in	陈应坚,甘莉萍,杨慧.	n Yingjian,Gan Liping,Yang	职业与健康	Occupation and health	24	5	412-415	2008	sn.1004-1257.2008.05.002
2259	沙门氏菌的检疫风险评估	tine risk assessment of Sal	黄燕琼.	Huang Yanqiong.	广东农业科学	angdong Agricultural Scien	-	7	109-111	2008	n.1004-874X.2008.07.041
2260	区部分社区老年活动场所	activity places in some cor	张欣,沈永,许德荣.	ang Xin,Shen Yong,Xu Dero	预防医学论坛	Preventive Medicine Forum	14	6	500-502	2008	sn.1672-9153.2008.06.007
2261	腹泻病人中发现苏贝鲁血	discovered in Xinjiang for t	理,顾本思,木合亚提,刘清,	,Gu Bensi,muheyati,Liu Qin	地方病通报	endemic disease notificatio	23	6	20-21,23	2008	sn.1000-3711.2008.06.006
2262	株药物敏感试验和抗生素	nd antibiotic resistance an	琼,孔繁德,彭海滨,徐淑	g,Kong Fande,Peng Haibin,	中国抗生素杂志	hinese Journal of Antibioti	33	1	S3	2008	-
2263	从业人员肠道沙门菌和志	and Shigella among emplo	红,王惠峰,沈春莉,谢晓红,	g Huifeng,Shen Chunli,Xie	上海预防医学	hanghai Preventive Medici	20	3	119-120	2008	sn.1004-9231.2008.03.004
2264	区医院常见病原菌分布及	f common pathogenic bac	陈超群,张秋桂,刘双全,王	n,Zhang Qiugui,Liu Shuang	中国抗生素杂志	hinese Journal of Antibioti	33	5	303-306,316	2008	-
2265	堂发生肠炎沙门氏菌的食	ing caused by Salmonella E	赵丽华,刘加学,王晓东.	Lihua,Liu Jiaxue,Wang Xiao	宁夏医学院学报	rnal of Ningxia Medical Col	30	2	235-236	2008	sn.1674-6309.2008.02.048
2266	料中沙门氏菌的分离与鉴	nd identification of Salmon	王彪.	Wang Biao.	中国牧业通讯	a Animal Husbandry Newsl	-	7	40-41	2008	sn.2095-2473.2008.07.019
2267	血培养分离出肠炎沙门菌	isolated from blood cultur	于慧敏,徐波,张光芯.	Huimin,Xu Bo,Zhang Guang	检验医学与临床	boratory medicine and clini	5	1	52	2008	sn.1672-9455.2008.01.038
2268	伤寒沙门菌致小儿颅内感染	hildren caused by Salmone	黄斌,樊孝春.	Huang Bin,Fan Xiaochun.	检验医学与临床	boratory medicine and clini	5	17	1075-1075	2008	sn.1672-9455.2008.17.035
2269	童肠道感染细菌及耐药性	nd drug resistance in childr	杨洁.	Yang Jie.	实用预防医学	ractical preventive medicin	15	3	894-895	2008	sn.1006-3110.2008.03.125
2270	感染性腹泻暴发疫情调查	analysis of an infectious d	秦忠营,孔庆麟,王宇峰,黄	ing,Kong Qinglin,Wang Yu	应用预防医学	pplied preventive medicin	14	2	102-103	2008	n.1673-758X.2008.02.015
2271	06年熟肉制品微生物污染	ial contamination status o	管峰,应微微,裘丹红.	Feng,Ying Weiwei,Qiu Dan	中国卫生检验杂志	ese Journal of Health Inspe	18	6	1141-1142	2008	sn.1004-8685.2008.06.073
2272	汉市食品中沙门菌耐药性	esistance of Salmonella in	邓华英,谭贝妮,熊燕,曾莹春	ng,Tan Beini,Xiong Yan,Ze	中国医学检验杂志	urnal of Medical Laborator	9	6	-	2008	-
2273	市售食品中微生物污染监	bial contamination in com	孙燕萍,葛敏英,遥远,周帅	ping,Ge Minying,distant,Zh	现代预防医学	modern preventive medicin	35	16	3073-3074,3081	2008	sn.1003-8507.2008.16.014
2274	7年熟肉制品微生物学检	ection and monitoring ana	军,金莞尔,于丽莉,叶承华,	un,Jin Wan'er,Yu Lili,Ye Ch	中国卫生检验杂志	ese Journal of Health Inspe	18	8	1593-1594	2008	sn.1004-8685.2008.08.059
2275	地区零售肉中沙门菌耐药	istance of Salmonella in re	伟,只帅,崔生辉,席美丽,杨	ndsome,Cui Shenghui,Xi M	中华预防医学杂志	e Journal of Preventive Me	42	10	758-761	2008	sn:0253-9624.2008.10.013
2276	门菌暴发的脉冲场凝胶电	g and drug resistance of an	伟伟,陈建辉,李闽真,欧剑	i,Chen Jianhui,Li Minzhen,	中国预防医学杂志	e Journal of Preventive Me	9	7	671-673	2008	sn.1009-6639.2008.07.028
2277	髋关节沙门菌感染两例报	s of salmonella infection in	黄志峰,邱贵兴,翁习生,赵	g,Qiu Guixing,Weng Xishe	中国骨与关节外科	inese Bone and Joint Surge	1	1	90-92	2008	sn.1674-1439.2008.01.020
2278	业人员肠道病原菌携带情	tinal pathogenic bacteria a	马志强.	Ma Zhiqiang.	中国卫生检验杂志	ese Journal of Health Inspe	18	3	567-567	2008	sn.1004-8685.2008.03.084
2279	肠炎沙门菌食物中毒检测	of a Case of Salmonella En	青,谭海芳,丁丽娜,林风,程	Haifang,Ding Lina,Lin Feng,	中国卫生检验杂志	ese Journal of Health Inspe	18	8	1650-1651	2008	sn.1004-8685.2008.08.086
2280	西安市细菌性腹泻病监测	acterial diarrhea surveillan	王春娟.	Wang Chunjuan.	职业与健康	Occupation and health	24	20	2174-2175	2008	sn.1004-1257.2008.20.035
2281	病合并猪霍乱沙门菌败血	n choleraesuis salmonella s	张志民,左毅,曾红莲.	g Zhimin,Zuo Yi,Zeng Hong	四川医学	Sichuan Medicine	29	11	1578	2008	sn.1004-0501.2008.11.066
2282	品中沙门菌的检测及定量	tative analysis of Salmonel	王殿夫.	Wang Dianfu.	中国酿造	China Brewing	-	16	89-90	2008	-
2283	一起慕尼黑沙门菌食源性	gel electrophoresis in the	扈庆华,兰全学,张齐良,	ghua,Lan Quanxue,Zhang	中华预防医学杂志	e Journal of Preventive Me	42	11	827-830	2008	sn:0253-9624.2008.11.013
2284	染沙门菌和志贺菌血清型	ance of Salmonella and Shi	陈建英,何梅芳,何燕,潘寒	ianying,He Meifang,He Ya	中国卫生检验杂志	ese Journal of Health Inspe	18	12	2612-2615	2008	sn.1004-8685.2008.12.064
2285	郴州地区猪高热病的病因	of pig hyperthermia in Che	邓国荣.	Deng Guorong.	中国兽医杂志	Chinese Veterinary Journal	44	9	40-41	2008	sn.0529-6005.2008.09.020
2286	门氏菌的分离、鉴定以及	and drug susceptibility test	孙彬,徐益斌,徐连均.	Sun Bin,Xu Yibin,Xu Lianjun	北京农业	Beijing Agriculture	-	30	32-34	2008	sn.1000-6966.2008.30.012
2287	市西城区食品中主要致病	alysis of main pathogenic s	崔京辉,李达,王丽萍,王永	i,Li Da,Wang Liping,Wang	中国卫生检验杂志	ese Journal of Health Inspe	18	6	1163-1165	2008	sn.1004-8685.2008.06.083
2288	丁引起鼠伤寒沙门菌食物	caused by Salmonella typh	石峻.	Shi Jun.	现代预防医学	modern preventive medicin	35	2	250252	2008	sn.1003-8507.2008.02.019
2289	为主要症状的疾病300例临	as the main symptom300	陈南晖,李莉.	Chen Nanhui,Li Li.	中国医疗前沿（上半月）	lical Frontier (First Half of t	3	7	60-61	2008	-
2290	发生沙门氏菌和梭菌混合	of Salmonella and Clostridi	宫国令.	Palace Order.	中国草食动物	Chinese herbivores	28	3	70-71	2008	sn.2095-3887.2008.03.028
2291	模化鸡场病原菌及耐药性	drug resistance trends in la	李淑梅,杨帆,刘兴友.	Shumei,Yang Fan,Liu Xingy	养禽与禽病防治	and poultry disease preven	-	9	7-9,4	2008	-
2292	吉林省食品中食源性致病	esults of foodborne pathog	军,刘桂华,孔祥云,黄鑫,	uihua,Kong Xiangyun,Hua	中国卫生检验杂志	ese Journal of Health Inspe	18	7	1400-1402	2008	sn.1004-8685.2008.07.073

2293	苏州市吴中区细菌性食物	food poisoning incidents in	杨翠英.	Yang Cuiying.	职业与健康	Occupation and health	24	21	2290-2291	2008	sn.1004-1257.2008.21.027
2294	菌引起新生儿血液、脑脊	d and cerebrospinal fluid in	任萍,周文.	Ren Ping,Zhou Wen.	中华医院感染学杂志	ournal of Hospital Infectiou	18	2	223-223	2008	sn:1005-4529.2008.02.068
2295	由病牛沙门菌引起的食物	isoning caused by salmone	波,李海燕,周宗俞,周石芝,	Haiyan,Zhou Zongyu,Zhou S	现代预防医学	modern preventive medicin	-	S1	22-23	2008	-
2296	人猪霍乱沙门菌败血症一	n-swine cholera and salmo	华,姜立胜,张莹,李秀兰,李	ng Lisheng,Zhang Ying,Li Xiu	中华临床感染病杂志	ournal of Clinical Infectious	1	1	60	2008	sn.1674-2397.2008.01.019
2297	甲型副伤寒沙门菌隐蔽质	smid of a strain of Salmone	李军,陶建蜀,刘杨,黄海.	,Tao Jianshu,Liu Yang,yellow	医学检验与临床	edical Laboratory and Clinic	19	6	16-19	2008	sn.1673-5013.2008.06.005
2298	院2003～2005年沙门菌检	~2005Analysis of annual Sa	陈黔.	Chen Qian.	实用医药杂志	ournal of Practical Medicin	25	3	299	2008	sn.1671-4008.2008.03.026
2299	汤卜逊沙门氏菌的检验	report of Salmonella Thom	刘涛,闫善信.	Liu Tao,Yan Shanxin.	中华医学实践杂志	ese Journal of Medical Prac	7	4	-	2008	-
2300	售食品食源性致病菌监测	pathogenic bacteria in co	谢明英,闫立群,张月娥,秦	Mingying,Yan Liqun,Zhang Y	宁夏医学杂志	Ningxia Medical Journal	30	3	273-274	2008	sn.1001-5949.2008.03.046
2301	广西食源性沙门氏菌污染	sis of Foodborne Salmonell	,蒋震羚,黄彦,王红,车光,	henling,Huang Yan,Wang H	应用预防医学	applied preventive medicine	14	1	4-7	2008	n.1673-758X.2008.01.002
2302	沙门氏菌集体食物中毒临	ctive food poisoning by Sa	余惠琴,王晓艳.	Yu Huiqin,Wang Xiaoyan.	大理学院学报	Journal of Dali University	7	2	49-50,53	2008	sn.1672-2345.2008.02.023
2303	市生食蔬菜中病原菌污染	cteria contamination in raw	柱,黄兆勇,王红,吕素玲,蒋	Zhaoyong,Wang Hong,Lu S	应用预防医学	applied preventive medicine	14	6	361-363	2008	n.1673-758X.2008.06.017
2304	服务行业从业人员健康	al health examination resul	陈建晟,潘辉,许旭艳.	n Jiansheng,Pan Hui,Xu Xu	海峡预防医学杂志	s Journal of Preventive Me	14	6	42-44	2008	sn.1007-2705.2008.06.024
2305	性致病菌定量风险评估的	ative risk assessment for fo	陈艳,刘秀梅.	Chen Yan,Liu Xiumei.	中国食品卫生杂志	hinese Food Hygiene Journ	20	4	336-340	2008	sn.1004-8456.2008.04.018
2306	狱伤寒爆发疫情分子流行	estigation of typhoid fever	依旦·吾福尔,李方,木和亚	·Wufu,Li Fang,wood and a	中国热带医学	Chinese tropical medicine	8	10	1699-1700,1703	2008	sn.1009-9727.2008.10.011
2307	炎沙门氏菌引起食物中毒	of food poisoning caused	夏玛丽,王复元,孟炜,胡俊	a,Wang Fuyuan,Meng Wei	中国公共卫生管理	ese public health managen	24	4	443-444	2008	sn.1001-9561.2008.04.059
2308	性主要致病菌分布及耐	sistance of major pathogen	强忠,谢展雄,李文郎,张肄	g,Xie Zhanxiong,Li Wenlan	中国实用医药	Chinese Practical Medicine	3	19	16-18	2008	sn.1673-7555.2008.19.009
2309	寒沙门菌药物敏感试验及	nd drug resistance change	,屠传建,单平囡,陈英,寿	jian,Shan Pingnan,Chen Yin	现代实用医学	modern practical medicine	20	1	42-43	2008	sn.1671-0800.2008.01.023
2310	沙门氏菌引起的食物中毒	food poisoning caused by S	余惠琴,严文芳.	Yu Huiqin,Yan Wenfang.	云南预防医学杂志	n Journal of Preventive Me	13	2	65	2008	-
2311	7～2006年伤寒副伤寒流	annual epidemic characte	淑云,柴程良,余昭,莫顺堂	hai Chengliang,Yu Zhao,Mo	浙江预防医学	hejiang Preventive Medicin	20	5	1-2,5	2008	sn.1007-0931.2008.05.001
2312	水源污染引起腹泻爆发的	an outbreak of diarrhea ca	李华玲,郑国庆,田野.	Hualing,Zheng Guoqing,fie	中国校医	Chinese school doctor	22	1	56,58	2008	sn.1001-7062.2008.01.024
2313	在急性腹泻患者沙门氏	bacterial culture method	小丽,郑晓燕,栗绍刚,齐志	ng Xiaoyan,Li Shaogang,Qi	中国病原生物学杂志	ese Journal of Pathogen Bio	3	10	735-737	2008	sn.1673-5234.2008.10.005
2314	9～2006年细菌性食物中	nalysis of annual bacterial	刘秀峰,刘红.	Liu Xiufeng,Liu Hong.	中国卫生检验杂志	ese Journal of Health Inspe	18	2	327-328	2008	sn.1004-8685.2008.02.057
2315	利丹沙门菌引起的食源性	a bridan in Changping Distr	张金菊,彭华,郝鹤军.	ang Jinju,Peng Hua,Hao Hej	疾病监测	disease surveillance	23	12	803-804	2008	sn.1003-9961.2008.12.026
2316	国家监测点2005～2006年	Monitoring Point2005~2006	余东,叶菊莲,秦淑文,朱水	ong,Ye Julian,Qin Shuwen,	浙江预防医学	hejiang Preventive Medicin	20	6	1-2,17	2008	sn.1007-0931.2008.06.001
2317	型包装熟肉制品中微生	n non-standard packaged	,黄兆勇,黄彦,王红,车光,	oyong,Huang Yan,Wang Ho	实用预防医学	ractical preventive medicin	15	5	1462-1463	2008	sn.1006-3110.2008.05.055
2318	市南山区细菌性食物中	od poisoning results in Nan	锐敏,陈辉,温群文,俞慕华	hin,Chen Hui,Wen Qunwen	河南预防医学杂志	n Journal of Preventive Me	19	5	375-376,392	2008	sn.1006-8414.2008.05.030
2319	食源性致病菌污染状况及	itoring results of foodborn	秋云,杜光文,曾树权,尹映	u Guangwen,Zeng Shuquan	中国热带医学	Chinese tropical medicine	8	6	1015-1017	2008	sn.1009-9727.2008.06.065
2320	02～2006年食源性致病菌	06Annual foodborne path	立,刘长宏,王安礼,李雪梅	Li,Liu Changhong,Wang An	中国公共卫生	Chinese public health	24	2	222-224	2008	sn:1001-0580.2008.02.047
2321	炎沙门菌引起食物中毒报	poisoning caused by Salmo	秀,于帆,杨媛魁,贾雪芝,王	Fan,Yang Yuankui,Jia Xuezh	检验医学与临床	boratory medicine and clini	5	14	896-封3	2008	sn.1672-9455.2008.14.045
2322	4～2007年分离沙门菌的	Resistance analysis of annu	学,林一曼,扈庆华,石晓路,	Lin Yiman,Hu Qinghua,Shi X	中国卫生检验杂志	ese Journal of Health Inspe	18	8	1579-1580	2008	sn.1004-8685.2008.08.052
2323	致病菌监测网的建立及主	oring network in Hebei Prov	,申玉学,韩艳青,王英豪,	ue,Han Yanqing,Wang Ying	中国卫生检验杂志	ese Journal of Health Inspe	18	2	225-228,247	2008	sn.1004-8685.2008.02.012
2324	境中流通食品的食源性致	ic bacteria in food circulat	仕瑛,杨赟,邓仲良,刘碧源	,Yang Yun,Deng Zhongliang	中国现代医生	modern chinese doctor	46	29	114-116	2008	sn.1673-9701.2008.29.060
2325	副伤寒沙门菌药敏结果及	ts and clinical analysis of Sa	媛,朱姝媛,张彦,李云,余	,Zhu Shuyuan,Zhang Yan,Li	西南国防医药	west National Defense Me	18	2	244-245	2008	sn.1004-0188.2008.02.037
2326	安市感染性腹泻细菌病原	nce results of bacterial etio	晓宇,吴守芝,李一航,王春	,Wu Shouzhi,Li Yihang,Wa	疾病监测	disease surveillance	23	1	34-36	2008	sn.1003-9961.2008.01.013
2327	疗患者并发猪霍乱沙门菌	rapy patient complicated w	唐美玲,张午声.	ang Meiling,Zhang Wushen	中华护理杂志	Chinese Nursing Journal	43	6	522-523	2008	sn.0254-1769.2008.06.017
2328	氏菌对四种抗菌药物敏感	ella susceptibility to four a	胡文杰,马进,万静忠.	Wenjie,Ma Jin,Wan Jingzho	中外医疗	Chinese and foreign medica	27	10	67	2008	sn.1674-0742.2008.10.052
2329	市江干区食源性致病菌污	oodborne pathogenic bact	,徐丹戈,包芳珍,高海明,	ge,Bao Fangzhen,Gao Haim	中国卫生检验杂志	ese Journal of Health Inspe	18	3	488-490	2008	sn.1004-8685.2008.03.042

2330	民医院沙门菌对抗菌药物敏	bility to antibacterial drugs	坚,吴劲松,卢月梅,李红林	Wu Jinsong,Lu Yuemei,Li Ho	中国感染与化疗杂志	urnal of Infection and Cher	8	6	459-462	2008	sn:1009-7708.2008.06.014
2331	鸭沙门氏菌的分离与鉴定	and identification of Salmo	路振香,曾华东,吴菊莲.	enxiang,Zeng Huadong,Wu	畜牧兽医科技信息	d Veterinary Science and Te	-	10	28	2008	sn.1671-6027.2008.10.014
2332	炎的检测及病原菌的分离鉴	olation, identification and d	拉达来,刘晓松,朱宪光,赵	lai,Liu Xiaosong,Zhu Xiangu	畜牧与饲料科学	Livestock and feed science	29	6	74-76	2008	sn.1672-5190.2008.06.030
2333	门氏菌的分离鉴定及生长	nd growth model analysis o	刘晓川,殷文政,黄丹.	ochuan,Yin Wenzheng,Huar	农产品加工·学刊	ing of agricultural products	-	1	86-88	2008	1671-9646-B.2008.01.028
2334	重感染败血症1例的分析论	sis1Analysis and argument	香志远.	Xiang Zhiyuan.	中华临床医学研究杂志	Journal of Clinical Medical	14	10	-	2008	-
2335	～2007年食源性致病菌监	alysis of annual foodborne	丽荣,刘小嘉,李小艳,陈允	Liu Xiaojia,Li Xiaoyan,Chen	中国热带医学	Chinese tropical medicine	8	7	1258-1259	2008	sn.1009-9727.2008.07.097
2336	台州市市售食品中食源性致	odborne pathogenic bacter	伟伟,葛素君,包文君,徐佳	eiwei,Ge Sujun,Bao Wenjur	中国卫生检验杂志	ese Journal of Health Inspe	18	12	2730-2731,2762	2008	sn.1004-8685.2008.12.117
2337	07年无锡市食源性致病菌	tudy on foodborne pathog	周伟杰,诸芸,艾永才.	ou Weijie,Zhu Yun,Ai Yong	现代预防医学	modern preventive medicin	35	19	3704-3705,3707	2008	sn.1003-8507.2008.19.010
2338	寒沙门氏菌质粒谱分析及	phage typing of Salmonella	龚艳妮,孙殿斌.	Gong Yanni,Sun Dianbin.	中国人兽共患病学报	Chinese Journal of Zoonose	24	7	683-683,685	2008	sn.1002-2694.2008.07.024
2339	济宁市市售奶粉卫生检测结	esults of commercially avai	边贻海.	Bian Yihai.	济宁学院学报	Journal of Jining University	29	3	44-45	2008	sn.1004-1877.2008.03.017
2340	沙门氏菌感染蛋鸭的鉴定及	ty test of a special case of S	峰,赤群群,顾春阳,王文倢	un Qun,Gu Chunyang,Wan	水禽世界	waterfowl world	-	3	35-36	2008	-
2341	菌分离株耐药表型与耐药	otype and drug resistance	,李郁,张玮,王桂军,孙裴,秦	u,Zhang Wei,Wang Guijun	中国卫生检验杂志	ese Journal of Health Inspe	18	12	2488-2490	2008	sn.1004-8685.2008.12.013
2342	致病性大肠杆菌和沙门氏	nic Escherichia coli and Sal	李刚.	Li Gang.	中国动物检疫	China Animal Quarantine	25	10	33-34	2008	n.1005-944X.2008.10.018
2343	儿童腹泻病原菌分布及耐药	ance monitoring of childho	许建成,周琪,郑雁.	Jiancheng,Zhou Qi,Zheng Y	中国妇幼保健	ina maternal and child hea	23	6	863-864	2008	sn.1001-4411.2008.06.069
2344	寒A沙门菌耐药谱的连续	ous monitoring of Salmonel	董虹,杨善浦.	Dong Hong,Yang Shanpu.	江西医药	Jiangxi Medicine	43	5	475-476,472	2008	sn.1006-2238.2008.05.047
2345	6-2007年食源性致病菌污	al survey on foodborne pa	东,卢俊荣,李顺利,王静,步	,Lu Junrong,Li Shunli,Wang	职业与健康	Occupation and health	24	20	2160-2161	2008	sn.1004-1257.2008.20.026
2346	品行业从业人员沙门菌检出	tatus and drug resistance a	齐文岚,江建真,张琼,庄玲	ang Jianzhen,Zhang Qiong,	职业与健康	Occupation and health	24	10	961-962	2008	sn.1004-1257.2008.10.029
2347	感染性腹泻病原学和耐药	sistance of severe infectiou	晶,李全瑞,王久伶,刑建刚	Quanrui,Wang Jiuling,Xing	世界华人消化杂志	ld Chinese Journal of Diges	16	10	1137-1139	2008	sn.1009-3079.2008.10.022
2348	分出口食品微生物检验结	nd analysis of some exporte	丽颖,苗丽,王亚宾,金钺,郭	Miao Li,Wang Yabin,Golden	中国食品卫生杂志	hinese Food Hygiene Journ	20	5	431-433	2008	sn.1004-8456.2008.05.013
2349	性肺炎为首发表现的伤寒	s the first manifestation of	邹峻.	Zou Jun.	中国感染与化疗杂志	urnal of Infection and Cher	8	3	228-229	2008	sn:1009-7708.2008.03.017
2350	甲型副伤寒沙门菌复合PC	monella paratyphi A compl	王斌,梁昊宇,计国欣,曾明	in,Liang Haoyu,Ji Guoxin,Ze	中国公共卫生	Chinese public health	24	2	238-239	2008	sn:1001-0580.2008.02.042
2351	人因感染沙门氏菌致死的	eath from salmonella infec	-	-	基础医学与临床	Basic medicine and clinical	28	6	632	2008	-
2352	市市售食品中食源性致病	e pathogenic bacteria in co	司徒谊萍,余枫华,吴谨呈	Yiping,Yu Fenghua,Wu Jincl	中国卫生检验杂志	ese Journal of Health Inspe	18	1	126-128	2008	sn.1004-8685.2008.01.057
2353	无名高热病的鉴别诊断及治	and treatment of unknown	方玉田,孙政慧,姜松华.	utian,Sun Zhenghui,Jiang So	畜牧市场	livestock market	-	7	20-21	2008	-
2354	原地区儿童细菌性腹泻病	ug resistance of pathogeni	原慧云,张镁硒,马荣伟.	Huiyun,Zhang MeiSe,Ma Ro	中国微生态学杂志	inese Journal of Microecolo	20	6	589-590,593	2008	n.1005-376X.2008.06.026
2355	从业人员大便培养现状分析	nt Situation of Stool Cultur	曹占敏,马洪棉.	Cao Zhanmin,Ma Hongmian	现代预防医学	modern preventive medicin	35	22	4543	2008	sn.1003-8507.2008.22.065
2356	市中心城区餐饮业卫生状况	h status of catering industr	正华,林党柒,陈娟,王铁程	ua,Lin Dangqi,Chen Juan,W	职业与健康	Occupation and health	24	8	749-750	2008	sn.1004-1257.2008.08.021
2357	003-2006 年食源性致病菌	06Analysis of annual foodb	刘国蓉,王燕,孙伟.	Guorong,Wang Yan,Sun W	中国预防医学杂志	se Journal of Preventive Me	9	6	533-535	2008	sn.1009-6639.2008.06.024
2358	中分离出一株圣地亚哥沙门	lmonella San Diego isolate	邱燕芬,赵德扬.	Qiu Yanfen,Zhao Deyang.	浙江检验医学	hejiang Laboratory Medicin	-	2	44	2008	-
2359	沙湾县两起突发公共卫生事	two public health emerge	木哈什·木哈提.	muhash·muhati.	地方病通报	endemic disease notificatio	23	4	71-72	2008	sn.1000-3711.2008.04.033
2360	顺德区一起食物中毒的实验	ase of food poisoning in Sh	,巫结冰,陈德云,张慧红,胡	bing,Chen Deyun,Zhang H	华南预防医学	uth China Preventive Medic	34	2	78-79	2008	sn.1671-5039.2008.02.032
2361	儿沙门菌引起食物中毒的实	ase of food poisoning caus	邹静波,程跃,郑显奇.	Jingbo,Cheng Yue,Zheng Xi	检验医学与临床	boratory medicine and clini	5	1	62-63	2008	sn.1672-9455.2008.01.048
2362	腹泻病人致病菌的培养结	sults of pathogenic bacteria	陈文,周永红.	Chen Wen,Zhou Yonghong	福建医药杂志	Fujian Medical Journal	30	4	104-105	2008	sn.1002-2600.2008.04.059
2363	沙门菌引起的食物中毒的	se of food poisoning cause	全玉平,贾永强,贾桂华.	Yuping,Jia Yongqiang,Jia Gu	中国卫生检验杂志	ese Journal of Health Inspe	18	5	918-919	2008	sn.1004-8685.2008.05.067
2364	为主要症状的疾病300例临	as the main symptom300C	陈南晖,李莉.	Chen Nanhui,Li Li.	中国医疗前沿（上半月）	lical Frontier (First Half of t	3	13	60-61	2008	sn.1673-5552.2008.13.037
2365	肉与肉制品微生物污染状况	mination of meat and meat	晓林,许敬东,马军武,高天	Xu Jingdong,Ma Junwu,Ga	中兽医医药杂志	raditional Chinese Veterina	27	3	22-25	2008	sn.1000-6354.2008.03.008
2366	沙门氏菌及大肠杆菌对16种	hia coli from a pig farm in	傅力,任娟,李瑾瑜,牛淑萍	i,Ren Juan,Li Jinyu,Niu Shup	新疆农业科学	Xinjiang Agricultural Science	45	4	754-757	2008	-

2367	者喹诺酮耐药鼠伤寒沙门	nolone-resistant Salmonell	李景云,马越,胡昌勤,张力	yun,Ma Yue,Hu Changqin,te	中华检验医学杂志	e Journal of Laboratory Me	31	11	1253-1258	2008	sn:1009-9158.2008.11.013
2368	标本细菌培养结果与药敏	e results and drug suscepti	刘海燕.	Liu Haiyan.	现代医药卫生	nodern medicine and healt	24	17	2631-2632	2008	-
2369	肉类、海产品中6种食源	and Seafood Center6Conta	学辉,张建群,梅允淼,张怡	Zhang Jianqun,Mei Yunmia	疾病监测	disease surveillance	23	7	427-429	2008	sn.1003-9961.2008.07.012
2370	1株沙门氏菌的耐药性分析	sistance analysis of Salmon	东琼,孔繁德,彭海滨,徐淑菲	g,Kong Fande,Peng Haibin,	畜牧与兽医	Husbandry and Veterinary M	40	8	77-79	2008	-
2371	猪霍乱沙门氏菌败血症1	a Swine Salmonella Septic	平,张大东,陈跃光,冯小弟	ang Dadong,Chen Yueguan,	中国疗养医学	hinese convalescent medici	17	4	244-245	2008	n.1005-619X.2008.04.044
2372	炎沙门菌所致食物中毒的	ase of food poisoning cause	何攀.	He Pan.	职业卫生与病伤	ccupational health and illne	23	5	320-321	2008	n.1006-172X.2008.05.025
2373	原位心脏移植术后肠道感	after orthotopic heart trans	师桃,吕毅,李明,耿希刚.	Tao,Lu Yi,Li Ming,Geng Xiga	心肺血管病杂志	ardiopulmonary and Vascu	27	2	暗4	2008	sn.1007-5062.2008.02.018
2374	少门氏菌感染105例药敏结	nd young children105Exam	万蟾,李继军,孙希平,李百	Vanchan,Li Jijun,Sun Xiping,	中国现代医生	modern chinese doctor	46	18	235238	2008	sn.1673-9701.2008.18.133
2375	肠炎沙门菌引起的食物中	poisoning caused by Salmo	钟凌,黄健利,赖林鹏.	g Ling,Huang Jianli,Lai Linp	中国卫生检验杂志	ese Journal of Health Inspe	18	8	1652	2008	sn.1004-8685.2008.08.087
2376	利丹沙门菌引起腹壁脓肿	scess caused by Salmonella	张昊.	Zhang Hao.	中国实验诊断学	nese experimental diagnos	12	5	688	2008	sn.1007-4287.2008.05.046
2377	门菌食物中毒的病原学调	etiology of Salmonella Ente	李文明,樊磊,李宏斌.	Wenming,Fan Lei,Li Hongb	医药论坛杂志	Medical Forum Magazine	29	7	67-67,71	2008	sn.1672-3422.2008.07.026
2378	副伤寒沙门氏菌引起的食	s of food poisoning caused	刘秋菊.	Liu Qiuju.	医学动物防制	medical animal control	24	4	295-296	2008	sn.1003-6245.2008.04.025
2379	炎,祸起肉骨粉——奶牛肠	meal——Research on prev	张剑柄.	Zhang hilt.	中国动物保健	China animal health	-	5	29-30,32	2008	sn.1008-4754.2008.05.005
2380	分地区鸡源沙门菌流行株的	of Salmonella originating fr	库华,周守长,张晓剑,刘文	ou Shouchang,Zhang Xiaoji	中国家禽	Chinese poultry	30	22	50-51	2008	-
2381	门菌多位点序列分型技术	sequence typing technology	计融,王玉平,江涛,崔生辉,	ang Yuping,Jiang Tao,Cui S	卫生研究	health research	37	1	46-49	2008	sn.1000-8020.2008.01.017
2382	模化猪场病原菌的分离及	scale pig farms in northern	李淑梅,杨帆,刘兴友.	Shumei,Yang Fan,Liu Xingyo	畜牧与兽医	Husbandry and Veterinary M	40	6	82-84	2008	-
2383	市食源性致病菌污染状况	status of foodborne patho	,刘建琪,张红,湛志飞,张	nqi,Zhang Hong,Zhan Zhife	中国热带医学	Chinese tropical medicine	8	9	16631665	2008	sn.1009-9727.2008.09.106
2384	扇贝中沙门氏菌检测方法	n method of Salmonella in	妍,马琳,许岩,桂英爱,王慧	Ma Lin,Xu Yan,Gui Yingai,W	口岸卫生控制	Port health control	13	5	31-32	2008	sn.1008-5777.2008.05.011
2385	菌属引起的食物中毒事件	of food poisoning incidents	李光晖,李泽波.	Li Guanghui,Li Zebo.	云南预防医学杂志	n Journal of Preventive Me	13	2	64-65	2008	-
2386	袭性都柏林沙门菌感染一	invasive Salmonella Dublin	杨璐,孙若鹏.	Yang Lu,Sun Ruopeng.	中华儿科杂志	Chinese Journal of Pediatric	46	9	720	2008	sn:0578-1310.2008.09.026
2387	阳市食品致病菌污染状况	Food Pathogenic Bacteria	包云娟,韦俊超.	Bao Yunjuan,Wei Junchao.	中国食品卫生杂志	hinese Food Hygiene Journ	20	3	252-254	2008	sn.1004-8456.2008.03.017
2388	致病菌耐药性分析及临床	tion of drug resistance of in	袁志军,颜秉兴.	Yuan Zhijun,Yan Bingxing.	医药论坛杂志	Medical Forum Magazine	29	5	50-51	2008	sn.1672-3422.2008.05.023
2389	海市沙门菌病流行特征分	ogical characteristics of sal	安,金汇明,许学斌,林亚萍,	Huiming,Xu Xuebin,Lin Ya	环境与职业医学	mental and Occupational M	25	3	245-247,251	2008	sn.1006-3617.2008.03.006
2390	炎沙门菌引起的食源性疾	borne illness caused by Sal	越江,胡英,汤丽,汪建秀,韩	ng,Hu Ying,Tang Li,Wang Ji	中国预防医学杂志	e Journal of Preventive Me	9	6	563-564	2008	sn.1009-6639.2008.06.044
2391	细菌性食物中毒病原菌	detection results of bacter	孙健.	Sun Jian.	现代预防医学	nodern preventive medicin	35	19	3702-3703	2008	sn.1003-8507.2008.19.009
2392	雷登沙门菌引起食物中毒	se of food poisoning cause	何攀.	He Pan.	中国卫生检验杂志	ese Journal of Health Inspe	18	9	1905-1905,1909	2008	sn.1004-8685.2008.09.090
2393	自贡市食源性疾病病原菌	ination status of foodborne	崔敏莉,陈燕梅,许烈英,何	Chen Yanmei,Xu Lieying,H	中国卫生检验杂志	ese Journal of Health Inspe	18	6	1171-1173	2008	sn.1004-8685.2008.06.086
2394	门菌PFGE分子分型及耐药	FGEMolecular typing and d	,段永翔,鞠长燕,石晓路,	Yongxiang,Ju Changyan,Shi	中国卫生检验杂志	ese Journal of Health Inspe	18	2	214-215,254	2008	sn.1004-8685.2008.02.008
2395	省沿海地区伤寒副伤寒疫	idemic situation and survei	云,柴程良,莫顺堂,韦余东,	Chengliang,Mo Shun Tang,V	中国预防医学杂志	e Journal of Preventive Me	9	4	284-286	2008	sn.1009-6639.2008.04.011
2396	表现开始的鼠伤寒沙门氏	imurium that began with r	王明华,孙萍.	Wang Minghua,Sun Ping.	实用临床医学	practical clinical medicine	9	3	125	2008	sn.1009-8194.2008.03.048
2397	中沙门氏菌的分离与血清	identification of Salmonel	华,龙丽坤,蔡先全,林娜,伍	ng Likun,Cai Xianquan,Lin	检验检疫科学	pection and Quarantine Scie	18	2	42-44	2008	sn.1674-5354.2008.02.015
2398	氏菌分离株的血清型和药	lity analysis of Salmonella i	安,魏建忠,王伟,王强,李春	ong,Wang Wei,Wang Qian,	中国人兽共患病学报	Chinese Journal of Zoonose	24	1	67-70	2008	sn.1002-2694.2008.01.017
2399	氨基糖苷类耐药基因	aphella swine in Tai'an, Shando	钟相,张文娟,李晓静,张永	g,Zhang Wenjuan,Li Xiaojin	中国抗生素杂志	hinese Journal of Antibiotic	33	9	557-560	2008	-
2400	5-2007年出口水产品卫生	on the sanitary quality of e	,纪强,肖德雄,高丽芳,黄	,Xiao Dexiong,Gao Lifang,H	中国国境卫生检疫杂志	ontier Health and Quarantin	31	5	329-332	2008	sn.1004-9770.2008.05.014
2401	沙门菌所致感染性动脉瘤	ious aneurysm caused by S	叶益聪,李剑,周宝桐.	Yicong,Li Jian,Zhou Baoton	中华医学杂志	Chinese Medical Journal	88	27	1944	2008	sn:0376-2491.2008.27.022
2402	市2004～2007年食物中毒	004~2007annual food pois	周有森.	Zhou Yousen.	右江民族医学院学报	ujiang Medical College for	30	6	986-988	2008	sn.1001-5817.2008.06.037
2403	级酒店凉菜间自制熟肉制品	cooked meat products in th	张磊,田晓燕.	Zhang Lei,Tian Xiaoyan.	职业与健康	Occupation and health	24	2	137-138	2008	sn.1004-1257.2008.02.023

2404	杯中沙门氏菌的分离及血	entification of Salmonella	范玉霞.	Fan Yuxia.	四川畜牧兽医	nal Husbandry and Veterin	35	8	27-28	2008	sn.1001-8964.2008.08.012
2405	从业人员肠道致病菌检测	acteria detection results fo	许素芬,周海慧.	Xu Sufen,Zhou Haihui.	中国卫生检验杂志	ese Journal of Health Inspe	18	12	2725-2726	2008	sn.1004-8685.2008.12.115
2406	98-2007年伤寒和甲型副伤	lysis of annual epidemic tr	强,王树坤,王艳芳,李六九,	ang Shukun,Wang Yanfang	中国自然医学杂志	ese Journal of Natural Med	10	6	401-405	2008	-
2407	首次检出格罗斯出浦沙门	ve analysis of Salmonella g	王殿夫.	Wang Dianfu.	中国酿造	China Brewing	-	20	86-87	2008	-
2408	大兴区食品污染物监测结	contaminant monitoring re	王桂侠,吴占国.	Wang Guixia,Wu Zhanguo.	中国卫生检验杂志	ese Journal of Health Inspe	18	11	2356-2357,2377	2008	sn.1004-8685.2008.11.077
2409	市感染性腹泻监测点细菌	ce results at the Xi'an infec	王春娟,张晓宇,吴守芝,李	unjuan,Zhang Xiaoyu,Wu S	职业与健康	Occupation and health	24	15	1543-1544	2008	sn.1004-1257.2008.15.036
2410	性感染性腹泻病原菌的耐	of pathogenic bacteria ca	李建华,包东武.	Li Jianhua,Bao Dongwu.	现代预防医学	modern preventive medicin	35	23	4758-4759	2008	sn.1003-8507.2008.23.093
2411	沙门菌引起食物中毒的实	of a case of food poisoning	燕,李毅,章乐怡,吴跃进,洪	Li Yi,Zhang Leyi,Wu Yuejin,	中国热带医学	Chinese tropical medicine	8	7	1223-1224	2008	sn.1009-9727.2008.07.080
2412	无名高热病的鉴别诊断及	s and Treatment of Pig Yua	方玉田,别、政慧,姜松华.	n,Farewell, Zheng Hui,Jiang	畜牧市场	livestock market	-	7	20-21	2008	-
2413	伤寒沙门菌对常用抗菌药	aratyphi A strains to comm	,蒋贤高,陈秀平,邵朝朝,	iangao,Chen Xiuping,Shao	中国感染与化疗杂志	urnal of Infection and Che	8	5	377-378	2008	sn:1009-7708.2008.05.013
2414	浙江省玉环县伤寒疫情及	tuation and surveillance re	,应旭华,刘灿磊,施海云,	uhua,Liu Canlei,Shi Haiyun	疾病监测	disease surveillance	23	6	355-357	2008	sn.1003-9961.2008.06.010
2415	公共场所从业人员沙门菌	ong workers in food and p	李贻汉,蔡妙森,吴为应,龙	an,Cai Miaosen,Wu Weiyi	职业与健康	Occupation and health	24	6	534-535	2008	sn.1004-1257.2008.06.017
2416	性腹泻主要病原菌调查研	ia of bacterial infectious di	,周晓红,吴海平,陈鸣芳,	hong,Wu Haiping,Chen Mi	中国卫生检验杂志	ese Journal of Health Inspe	18	6	1187-1188,1218	2008	sn.1004-8685.2008.06.092
2417	区儿童细菌性腹泻病原菌	tibility of pathogenic bacte	赵兰兰,朱朝敏,张爱华.	anlan,Zhu Chaomin,Zhang	中国实用儿科杂志	ese Journal of Practical Pedi	23	1	45-49	2008	sn.1005-2224.2008.01.015
2418	鸡沙门氏菌的分离与鉴定	dentification of Salmonella	郑启宏,朱兴贵,王红戟.	Qihong,Zhu Xinggui,Wang	中国畜禽种业	estock and poultry breeding	4	10	66-69	2008	sn.1673-4556.2008.10.041
2419	面及蛋内容物的微生物污	ntamination on egg shell s	美玉,王永霞,孔德江,任发	ang Yongxia,Kong Dejiang,	肉类研究	meat research	-	3	62-65	2008	sn.1001-8123.2008.03.018
2420	07年夏秋季感染性腹泻病	eillance results of infectio	红星,赵东敏,刘利容,杜浩	hao Dongmin,Liu Lirong,D	中国卫生检验杂志	ese Journal of Health Inspe	18	8	1601-1602	2008	sn.1004-8685.2008.08.063
2421	重感染败血症1例的分析论	sis1Analysis and argument	香志远.	Xiang Zhiyuan.	中国临床医药研究杂志	Journal of Clinical Medical	-	16	-	2008	-
2422	8例甲型副伤寒流行病学	ogical investigation of para	从家,孔繁林,吴惠玲,李杰	jia,Kong Fanlin,Wu Huiling	云南医药	Yunnan Medicine	29	1	57	2008	-
2423	粉微生物学品质安全特	uality and safety character	马超,夏成文.	Ma Chao,Xia Chengwen.	安徽农业科学	Anhui Agricultural Sciences	36	24	10476-10479	2008	sn.0517-6611.2008.24.084
2424	通细菌培养的病原菌分布	tion and drug resistance in	延根,石燕,陈拥军,邓为之	en,Shi Yan,Chen Yongjun,D	实用预防医学	ractical preventive medicin	15	6	1950-1953	2008	sn.1006-3110.2008.06.114
2425	感染性腹泻监测点细菌病	eillance results at infectio	王春娟,张晓宇,吴守芝,李	unjuan,Zhang Xiaoyu,Wu S	实用预防医学	ractical preventive medicin	15	5	1590-1591	2008	sn.1006-3110.2008.05.120
2426	革兰氏阴性杆菌的分布及	ance analysis of Gram-neg	王潭枫,符永玫,滕奔琦.	Tanfeng,Fu Yongmei,Teng	国际医药卫生导报	ational Medical and Health	14	6	76-79	2008	sn.1007-1245.2008.06.028
2427	耐药沙门菌I类整合子与耐	ghtered pigsIDetection of	焦新安,魏建忠,王桂军,张	i Jianzhong,Wang Guijun,Z	中国人兽共患病学报	Chinese Journal of Zoonose	24	4	327-330	2008	sn.1002-2694.2008.04.011
2428	海岛文蛤模拟加工的细菌	ulated processing of clams	雷晓凌,吴红棉,吴晓萍,叶茂	,Wu Hongmian,Wu Xiaopi	现代食品科技	Modern food technology	24	5	483-486	2008	sn.1673-9078.2008.05.024
2429	病"病猪细菌性病原的分离	d identification of bacteria	海君,崔金生,朱瑞良,王汝	Cui Jinsheng,Zhu Ruiliang,W	动物医学进展	vances in Veterinary Medic	29	9	30-34	2008	sn.1007-5038.2008.09.009
2430	-2005年腹泻病原菌的分布	n of diarrheal pathogenic	恩博,郭桐生,鲍春梅,刘立	Guo Tongsheng,Bao Chunn	中华内科杂志	ese Journal of Internal Med	47	4	304-307	2008	sn:0578-1426.2008.04.013
2431	沙门氏菌引起食物中毒的	case of food poisoning ca	李怀玉.	Li Huaiyu.	中国医药指南	Chinese Medicine Guide	6	10	56-57	2008	sn.1671-8194.2008.10.035
2432	年上海市市售食品污染物	ults of contaminants in co	,陈蓉芳,赵宇翔,吴卫平,	ongfang,Zhao Yuxiang,Wu	环境与职业医学	mental and Occupational M	25	4	337-341	2008	sn.1006-3617.2008.04.003
2433	市餐饮业食源性致病菌的	odborne pathogenic bacte	阮明捷,巴蕾,单志兰,刘胡	Minh Chiet,Bale,Shan Zhila	中国预防医学杂志	se Journal of Preventive Me	9	5	390-392	2008	sn.1009-6639.2008.05.018
2434	市从业人员沙门菌带菌状	carriage status among em	简小珊,何艳.	Jian Xiaoshan,He Yan.	中国预防医学杂志	se Journal of Preventive Me	9	11	998	2008	sn.1009-6639.2008.11.025
2435	狗感染沙门氏杆菌病的诊	nt report on salmonellosis	建新,朱进江,闫江林,余银	n,Zhu Jinjiang,Yan Jianglin,	湖北畜牧兽医	al Husbandry and Veterina	-	10	25	2008	n.1007-273X.2008.10.011
2436	并发院外获得性败血症2	y out-of-hospital acquired	冬梅,李倩,张颖,王英,马建	Qian,Zhang Ying,Wang Yin	医学研究生学报	l of Medical Postgraduate	21	9	966-968	2008	sn.1008-8199.2008.09.019
2437	猪霍乱沙门菌医院感染调	omial infection of neonate	明娟,石太新,钱惠茵,唐成	uan,Shi Taixin,Qian Huiyin,	中国实用医药	Chinese Practical Medicine	3	31	26-27	2008	sn.1673-7555.2008.31.014
2438	者感染阿拉莫沙门氏菌引	sed by Salmonella alamo in	,范振平,张文瑾,罗生强,	ng,Zhang Wenjin,Luo Shen	肝脏	liver	13	6	534	2008	sn.1008-1704.2008.06.037
2439	006年食品中沙门菌检测	ysis of Salmonella detectio	王晓松.	Wang Xiaosong.	职业与健康	Occupation and health	24	10	944-945	2008	sn.1004-1257.2008.10.018
2440	型副伤寒沙门菌耐药监测	ysis of drug resistance of S	谢伟乾,梁汉钦,杨其.	Weiqian,Liang Hanqin,Yang	广西医学	Guangxi Medicine	30	2	234-235	2008	sn.0253-4304.2008.02.043

2441	县日哈乡伤寒暴发的流	phoid fever outbreak in	袁文福;许军红;	Yuan Wenfu;Xu Junhong	寄生虫病与感染性疾病	diseases and infectious	-	3	146-147	2008	-
2442	型副伤寒暴发疫情的病	use of an outbreak of pa	孙昼;谢立;丁华;许向军	Zhou;Xie Li;Ding Hua;Xu	中国预防医学杂志	Journal of Preventive M	9	9	798-802	2008	-
2443	甲型副伤寒暴发疫情流行	factors of a paratyphoid	金忠平;王平;潘金仁;	ngping;Wang Ping;Pan	疾病监测	disease surveillance	-	7	417-419	2008	-
2444	型副伤寒暴发疫情的调	alysis of an outbreak of	方勇;龙江;	Fang Yong;longjiang;	疾病监测	disease surveillance	-	6	353-354	2008	-
2445	甲型副伤寒暴发的调查	alysis of an outbreak of	新梅;周运孙;徐连妹;蒋紫	Zhou Yunsun;Xu Lianme	中国热带医学	Chinese tropical medicine	-	6	1024	2008	-
2446	起的学校甲型副伤寒暴发	typhoid A Outbreaks in S	杨涛;张颖;麦浩;邓玄;	;Zhang Ying;Mai Hao;De	社区医学杂志	rnal of Community Medi	-	11	37-38	2008	-
2447	1起甲型副伤寒暴发调查	ation into Paratyphoid A	张晓丽;雷杰;	Zhang Xiaoli;Lei Jie;	预防医学论坛	reventive Medicine Foru	-	3	233-235	2008	-
2448	学一起甲型副伤寒暴发的	ak of paratyphoid fever	杨春婷;	Yang Chunting;	中国预防医学杂志	Journal of Preventive M	-	2	90	2008	-
2449	务工人员的食源性伤寒	reak of foodborne typhoi	俞新莲;苏成豪;陈景坪;	an;Su Chenghao;Chen J	现代预防医学	odern preventive medici	-	3	571-572	2008	-
2450	发流行的现场流行病学	vestigation and treatme	韦显威;	Wei Xianwei;	广西医学	Guangxi Medicine	-	1	77-78	2008	-
2451	海珠区罕见沙门菌的发现	ion of rare salmonella in Ha	许少洪,孙凤琪,计国欣.	haohong,Sun Fengqi,Ji Gu	中华流行病学杂志	inese Journal of Epidemiol	30	12	1316	2009	sn.0254-6450.2009.12.027
2452	腹泻病原菌的分离与鉴定	ntification of diarrhea pat	文智,李淑英,张敏,朱小	zhi,Li Shuying,Zhang Min,Z	中华医院感染学杂志	ournal of Hospital Infectiou	19	21	2995-2996	2009	sn:1005-4529.2009.21.072
2453	~2006年细菌性腹泻病监	ysis of annual bacterial dia	李家云,张腾允.	Li Jiayun,Zhang Tengyun.	现代预防医学	modern preventive medicin	36	6	1152-1153	2009	-
2454	霍乱沙门氏菌败血症1例报	Salmonella septicemia1Ex	琼,邓丹琪,郭芸,周晓鸿,	ng Danqi,Guo Yun,Zhou Xi	皮肤病与性病	ses and sexually transmitte	31	2	51	2009	sn.1002-1310.2009.02.037
2455	起食物中毒的调查分析	Poisoning Caused by Salm	-	-	中国保健	China health care	-	19	-	2009	-
2456	罕见沙门菌型腹泻的溯	bility of rare salmonella-ty	冉陆,刁保卫,陈敏,金汇明	Baobao,Chen Min,Jin Hui	上海预防医学	hanghai Preventive Medici	21	1	7-10	2009	sn.1004-9231.2009.01.003
2457	氏伤寒菌感染致肝脓肿1例	by salmonella typhi infect	宋囡.	Song Nan.	辽宁中医药大学学报	g University of Traditional	11	5	122-123	2009	-
2458	-2008年食源性致病菌污	f annual foodborne pathog	薛超波,王萍亚,孙瑛.	Chaobo,Wang Pingya,Sun	中国食品卫生杂志	hinese Food Hygiene Journ	21	6	533-536	2009	-
2459	35例细菌性食物中毒分析	s of cases of bacterial food	周有森.	Zhou Yousen.	临床医学工程	clinical medical engineering	16	9	95-96	2009	sn.1674-4659.2009.09.053
2460	儿血培养检出病原菌的分	stance of pathogenic bacte	黄明海.	Huang Minghai.	中国卫生检验杂志	ese Journal of Health Inspe	19	12	2894-2895,2962	2009	-
2461	门氏菌的血清型分布与耐	resistance analysis of Salm	建舫,何诚,查振林,杨汉	g,He Cheng,Zha Zhenlin,Ya	养禽与禽病防治	and poultry disease preve	-	8	9-11	2009	-
2462	山市食源性致病菌监测与	of foodborne pathogenic b	燕,陶勇,石志峰,王利,汪永	Yong,Shi Zhifeng,Wang Li,W	现代预防医学	modern preventive medicin	36	16	3126-3128,3135	2009	-
2463	门氏菌引起的软组织感染	infection and leakage caus	李伟青,汲泓.	Li Weiqing,Ji Hong.	辽宁中医药大学学报	g University of Traditional	11	1	119-120	2009	-
2464	患者血液、骨髓中分离出	ood and bone marrow of p	李慧平.	Li Huiping.	中华医院感染学杂志	ournal of Hospital Infectiou	19	11	1452	2009	sn:1005-4529.2009.11.066
2465	家家宴沙门菌食物中毒调	ysis of salmonella food poi	成锡山,付玉亮.	Cheng Xishan,Fu Yuliang.	西医药杂志（下半月版）	cal Journal (Second Half M	38	8	381	2009	-
2466	鸡沙门氏菌分离株的生化	stics of Salmonella chicken	武志强.	Wu Zhiqiang.	中国预防兽医学报	rnal of Preventive Veterina	31	5	406-408	2009	-
2467	门氏菌引起食物中毒的调	n a case of food poisoning	陈普成,叶恒平,赵淑军.	ucheng,Ye Hengping,Zhao	公共卫生与预防医学	Health and Preventive Me	20	5	76-77	2009	-
2468	学水源污染致腹泻暴发的	tbreak caused by water so	魏强,田野.	Wei Qiang,field.	预防医学论坛	Preventive Medicine Forum	15	3	287-288	2009	-
2469	合并血液感染的病原菌分	acteria in chronic obstructi	洪昆,侯蓓蕾,马丽梅,潘柳	gkun,Hou Beilei,Ma Limei,	现代预防医学	modern preventive medicin	36	21	4150-4152	2009	-
2470	多例急性感染性肠炎原	ltiple Cases of Acute Infect	陈出新,刘素球,张嘉.	en Chuxin,Liu Suqiu,Zhang	中国内镜杂志	hinese Journal of Endoscop	15	5	551-552	2009	-
2471	株血培养沙门菌的药物敏	ty of Salmonella strains cul	周文,杨肇立,陈旭,任萍.	Wen,Yang Zhaoli,Chen Xu,Re	检验医学与临床	boratory medicine and clini	6	2	112-112,114	2009	sn.1672-9455.2009.02.019
2472	-2008年甲型副伤寒沙门菌	ysis of drug resistance of S	陈彬彬,孙昼,汪皓秋.	Binbin,Sun Zhou,Wang Ha	浙江预防医学	hejiang Preventive Medici	21	12	35,37	2009	sn.1007-0931.2009.12.017
2473	季北京市沙门菌腹泻监测	rhea Surveillance Results i	高志勇,严寒秋,刘园,黄芳	vere cold autumn,Liu Yuan	疾病监测	disease surveillance	24	5	352-353	2009	sn.1003-9961.2009.05.014
2474	壁市食源性致病菌检测报	orne pathogenic bacteria	李素娥,杨丽,裴保河.	Li Su'e,Yang Li,Pei Baohe.	中国卫生检验杂志	ese Journal of Health Inspe	19	11	2643-2644	2009	-
2475	杆菌的分离鉴定及双重	Riemerella anatipestifer in	兴晓,姜世金,孙亚妮,刘美	xiao,Jiang Shijin,Sun Yani,Li	中国兽医学报	l of Chinese Veterinary Me	29	6	721-725	2009	-
2476	移植物感染的诊治体会(	nent of aortic stent graft in	符伟国,王玉琦,郭大乔,徐	Weiguo,Wang Yuqi,Guo Da	中国普外基础与临床杂志	nal of Basic and Clinical Ge	16	6	429-432	2009	-
2477	建省沙门菌监测菌株血	bility analysis of Salmonell	谢一俊,陈彩粼,陈武,杨劲	n,Chen Cailing,Chen Wu,Y	中国卫生检验杂志	ese Journal of Health Inspe	19	10	2376-2379	2009	-

2478	02～2006年食源性致病菌	ual surveillance analysis of	韩喜荣,谢利军,韩晔,汤丽	Xirong,Xie Lijun,Han Ye,Tar	现代预防医学	nodern preventive medicin	36	2	333-334	2009	-
2479	培养病原菌分布及耐药性	tance analysis of pathogen	琼,姜良飞,胡永林,陈泽慧	g,Jiang Liangfei,Hu Yonglin,	检验医学与临床	boratory medicine and clini	6	21	1841-1842	2009	sn.1672-9455.2009.21.026
2480	污染"水果刨冰"引起食物	ion"Fruit shaved ice"Analy	赛琴,吴少群,翁奕敏,倪肖	qin,Wu Shaoqun,Weng Yim	海峡预防医学杂志	s Journal of Preventive Me	15	3	48-49	2009	-
2481	患者病原体的分离鉴定与	ug resistance analysis of pa	陆其兵,李强,徐跃武.	u Qibing,Li Qiang,Xu Yuewu	检验医学与临床	boratory medicine and clini	6	20	1736-1737	2009	sn.1672-9455.2009.20.024
2482	型超市及酒店熟肉制品微	cooked meat products in la	志超,林梅艳,卢彩兰,曹春	,Lin Meiyan,Lu Cailan,Cao	职业与健康	Occupation and health	25	6	605-606	2009	-
2483	菌感染血清型及药物敏感性	ction serotype and drug ser	罗湘蓉,唐祥蜀,陆春凤.	ngrong,Tang Xiangshu,Lu Ch	检验医学与临床	boratory medicine and clini	6	24	2106-2107	2009	sn.1672-9455.2009.24.014
2484	省滨州市商品鸡传染病现状	ious diseases in commercia	马景霞.	Ma Jingxia.	畜牧与兽医	Husbandry and Veterinary M	41	7	82-84	2009	-
2485	市从业人员肠道沙门氏菌检	onella detection results am	,袁兰琼,冼桂江,李卓鹏,宋	Lanqiong,Xian Guijiang,Li Z	应用预防医学	applied preventive medicine	15	1	35-36	2009	n.1673-758X.2009.01.014
2486	多药耐药株的整合子及抗	ance genes of multidrug-re	黄海,李军,黄健,陶建蜀.	sea,Li Jun,Huang Jian,Tao J	中国实验诊断学	nese experimental diagnos	13	4	466-468	2009	sn.1007-4287.2009.04.015
2487	肝炎合并猪霍乱沙门氏菌	d with choleraesuis and sa	王晓花,张照华,边鹏飞.	aohua,Zhang Zhaohua,Bian	实用肝脏病杂志	urnal of Practical Hepatolo	12	2	103	2009	sn.1672-5069.2009.02.031
2488	者并发败血症感染菌及其	g resistance in patients wit	李皇,卢平宣.	Li Huang,Lu Pingxuan.	广西医学	Guangxi Medicine	31	4	567-569	2009	sn.0253-4304.2009.04.064
2489	市儿童社区获得性腹泻致	m of community-acquired	传清,何磊艳,王爱敏,张文	g,He Leiyan,Wang Aimin,Z	微生物与感染	Microbes and infections	4	2	97-100	2009	sn.1673-6184.2009.02.008
2490	菌性食物中毒的病原学检	of the etiology of a case of	建华,胡君平,岳改英,于旭	ua,Hu Junping,Yue Gaiying,	公共卫生与预防医学	Health and Preventive Me	20	5	82-83	2009	-
2491	8年梧州市糕点微生物检	crobial inspection results o	李梦映,莫桂姮,陈莉,李勇	gying,Mo Guiheng,Chen Li,	应用预防医学	applied preventive medicine	15	6	355-356	2009	n.1673-758X.2009.06.014
2492	炎沙门氏菌引起食物中毒	f food poisoning caused by	毅,石志翼,李军义,马立志	ng Yi,Shi Zhiyi,Li Junyi,Ma L	中国农村卫生事业管理	na's rural health managem	29	1	61-62	2009	-
2493	柏林沙门菌引起食物中毒	ous Occurrences of Food P	丽艳,王惠君,宋会荣,吴科	Vang Huijun,Song Huirong,	中国保健	China health care	-	19	-	2009	-
2494	新疆沙湾县一起食物中毒	ort on food poisoning in Sh	·阿衣铺,于美,麦丽开·阿	l·Ayipu,Yu Mei,Mai Likai·A	地方病通报	endemic disease notificatio	-	3	66	2009	-
2495	门县从业人员粪便沙门菌	lmonella testing results of	章跃炎.	Zhang Yueyan.	中国卫生检验杂志	ese Journal of Health Inspe	19	1	231-231	2009	-
2496	猪霍乱沙门菌菌血症二例	of choleraesuis Salmonella	,薛佩莲,贾桂芝,谭国英,陈	eilian,Jia Guizhi,Tan Guoyin	中华传染病杂志	se Journal of Infectious Dis	27	2	123	2009	sn.1000-6680.2009.02.017
2497	致病菌的动态监测及预警	warning and control of foo	吴达莘,荣飚.	Wu Dalshen,Rong Biao.	海峡预防医学杂志	s Journal of Preventive Me	15	1	60-62	2009	-
2498	例从业人员沙门和志贺菌	ion of Salmonella and Shige	杨桂芝,段飞云,王加银.	Guizhi,Duan Feiyun,Wang J	中国卫生检验杂志	ese Journal of Health Inspe	19	10	23902394	2009	-
2499	品中食源性致病菌污染状	s of foodborne pathogenic	王殿夫.	Wang Dianfu.	微生物学杂志	journal of microbiology	29	3	105-106	2009	sn.1005-7021.2009.03.022
2500	甲型副伤寒病例血培养研究	blood culture of paratypho	家,山德生,孔繁林,刘红雁,	Desheng,Kong Fanlin,Liu H	中华检验医学杂志	se Journal of Laboratory Me	32	5	543-546	2009	sn.1009-9158.2009.05.013
2501	药情况看喹诺酮类药物的	quinolones based on bact	叶秀娜.	Ye Xiuna.	临床医学工程	clinical medical engineering	16	8	129-130	2009	sn.1674-4659.2009.08.066
2502	国食源性鼠伤寒沙门菌病	ak of foodborne salmonella	徐进.	Xu Jin.	中国食品卫生杂志	hinese Food Hygiene Journ	21	2	144-146	2009	-
2503	伤寒89例临床表现与药敏	manifestations and drug se	李文全,康涛.	Li Wenquan,Kang Tao.	中国医药导报	China Medical Herald	6	22	36-37	2009	sn.1673-7210.2009.22.018
2504	沙门菌引起食物中毒的流行	a case of food poisoning	刘宏声.	Liu Hongsheng.	海峡预防医学杂志	s Journal of Preventive Me	-	1	48-49	2009	-
2505	用餐引起沙门氏菌食物中	monella food poisoning ca	英,黄运红,陈应平,朱晓琳	ang Yunhong,Chen Yingpin	现代预防医学	nodern preventive medicin	36	20	38593862	2009	-
2506	7年广西感染性腹泻流行	prevalence of infectious d	柳,周凌云,方锦嵩,李永红	ou Lingyun,Fang Jinsong,L	疾病监测	disease surveillance	24	7	507-510	2009	sn.1003-9961.2009.07.012
2507	07年重庆市沙门菌监测分	la surveillance and analysis	勤,赵寒,李志峰,王红,漆莉	ao Han,Li Zhifeng,Wang Ho	疾病监测	disease surveillance	24	9	694-696	2009	-
2508	道病原菌的分离鉴定及药	espiratory tract pathogenic	王业民.	Wang Yemin.	亚太传统医药	ia Pacific Traditional Medic	5	10	58-59	2009	-
2509	床分离79株沙门菌耐药性	aration79Salmonella drug r	勤,廖春艳,段刚,赵波,何源	o Chunyan,Duan Gang,Zha	中国卫生检验杂志	ese Journal of Health Inspe	19	1	182-184	2009	-
2510	寒沙门菌致病性及耐药机制	and drug resistance mecha	潘荞,邓治邦.	Pan Qiao,Deng Zhibang.	动物医学进展	vances in Veterinary Medic	30	2	93-97	2009	sn.1007-5038.2009.02.025
2511	川省鼠伤寒沙门菌PFGE分	in Sichuan ProvincePFGE	晋,杨小蓉,徐耀方,何树森	ang Xiaorong,Xu Yaofang,H	预防医学情报杂志	of Preventive Medicine Info	25	12	987-989	2009	-
2512	1998-2007年伤寒流行趋势	07Analysis on annual epide	红强,王艳芳,李六九,吴强	iang,Wang Yanfang,Li Liuji	职业与健康	Occupation and health	25	2	170-172	2009	-
2513	伤寒沙门菌引起食源性疾	dborne illness caused by Sa	李静媚,甘莉萍.	Li Jingmei,Gan Liping.	职业与健康	Occupation and health	25	4	382-383	2009	-
2514	沙门菌PFGE分子分型及耐	PFGEMolecular typing an	海芳,谭翰清,丁丽娜,林凤	ang,Tan Hanqing,Ding Lina,	中国卫生检验杂志	ese Journal of Health Inspe	19	9	2039-2041	2009	-

2515	禽沙门氏菌的分离和鉴定	nd Identification of Avian S	王利明,刘宏艳.	Wang Liming,Liu Hongyan.	畜牧兽医科技信息	d Veterinary Science and Te	-	2	27	2009	sn.1671-6027.2009.02.015
2516	自办宴席引起的肠炎沙门	d poisoning caused by a se	素艳,王静辉,史永刚,张国	Wang Jinghui,Shi Yonggang,	中国卫生检验杂志	ese Journal of Health Inspe	19	12	29853015	2009	-
2517	童细菌性腹泻病原菌分布	ogenic bacteria causing ba	龙,夏邦世,陈芬,陈琼娜,郑	onne,Chen Fen,Joanna Che	中国卫生检验杂志	ese Journal of Health Inspe	19	8	1853-1854	2009	-
2518	沙门菌引起食物中毒的流行	a case of food poisoning c	刘宏声.	Liu Hongsheng.	海峡预防医学杂志	s Journal of Preventive Me	15	1	48-49	2009	-
2519	一起甲型副伤寒暴发流行	an outbreak of paratyphoid	义春,李艳华,张树兰,邓	Yanhua,Zhang Shulan,Deng	中国学校卫生	Chinese school health	30	5	475-476	2009	-
2520	炎沙门氏菌引起的食物中	ood poisoning caused by Sa	曲桂娟,邢素琴,于慧霞.	Guijuan,Xing Suqin,Yu Hui	预防医学论坛	Preventive Medicine Forum	15	4	封3	2009	-
2521	腹泻病416例病原学检测与	416Etiology detection and	富勇,康华,张逊凯,郝如	g,Kanghua,Zhang Xunkai,H	中国妇幼健康研究	maternal and child health re	20	2	175-177	2009	sn.1673-5293.2009.02.023
2522	30份血液培养阳性结果分	ysis of positive blood cultur	陈刚,王玉春,高玲.	n Gang,Wang Yuchun,Gao	华夏医学	Chinese Medicine	22	1	62-63	2009	sn.1008-2409.2009.01.027
2523	儿童血培养病原菌分布及	nce of pathogenic bacteria	慧燕,杨锦江,舒旷怡,余玲	n,Yang Jinjiang,Shu Kuangy	中国实用儿科杂志	se Journal of Practical Pedi	24	12	954-956	2009	-
2524	重点餐饮单位凉拌菜食品	of cold dishes in key cateri	峰,陈东宛,张彦春,甄国新	ngwan,Zhang Yanchun,Zhe	微量元素与健康研究	e elements and health rese	26	3	35-36	2009	-
2525	染性腹泻病原学分类与临	n and clinical analysis of ac	茂茂,于红霞,吴金英,包	mao,Yu Hongxia,Wu Jinyin	中国临床医生	Chinese clinician	37	8	35-37	2009	sn.1008-1089.2009.08.015
2526	宝鸡市食源性致病菌污染	of foodborne pathogenic b	党春霞,高艳芹,薛彩娥.	Chunxia,Gao Yanqin,Xue C	中国食品卫生杂志	hinese Food Hygiene Journ	21	2	140-141	2009	-
2527	008年人源性沙门菌监测	annual human-derived Sal	刚,王红,王文斟,何源,李志	g Hong,Wang Wenzhen,He	中国卫生检验杂志	ese Journal of Health Inspe	19	5	11771192	2009	-
2528	区藏族儿童感染性腹泻病	fectious diarrhea among Ti	李荣.	Li Rong.	中外医疗	Chinese and foreign medica	27	12	131-131	2009	sn.1674-0742.2009.12.116
2529	及其养殖环境中食源性致	enic bacteria in tilapia and	燕燕,李凤霞,杨贤庆,刁石	Fengxia,Yang Xianqing,Diao	水产学报	Journal of Fisheries	33	5	823-831	2009	sn:1000-0615.2009.05.015
2530	门菌在宁波市售牡蛎中存	monella Paratyphi A in oys	许国章,周爱民,金春光,	hang,Zhou Aimin,Jin Chung	中国预防医学杂志	se Journal of Preventive Me	10	10	920-922	2009	-
2531	快速检测动物食品中沙门	etection of Salmonella cont	吕玉红,刘坤祥,陈泽慧,白	Yuhong,Liu Kunxiang,Chen	中国农学通报	na Agricultural Science Bull	25	20	95-99	2009	-
2532	食品中沙门菌的主动监测	w meat foods in Henan Pro	兴广,郝宗宇,胡巅,炊慧	ng,Hao Zongyu,Hu Dian,Cui	中国卫生检验杂志	ese Journal of Health Inspe	19	7	1545-1548	2009	-
2533	肇庆市食品中食源性致病	is of foodborne pathogenic	谭海芳,丁丽娜.	Tan Haifang,Ding Lina.	中国食品卫生杂志	hinese Food Hygiene Journ	21	3	267-270	2009	-
2534	鼠伤寒沙门氏菌引起的食	isoning caused by Salmone	苏明彦,张丽.	Su Mingyan,Zhang Li.	中国实用医药	Chinese Practical Medicine	4	9	247	2009	sn.1673-7555.2009.09.206
2535	餐饮业生食类水产蓄养箱	uaculture tank culture wate	王立斌,朱海明,辜少虹,曲	g Libin,Zhu Haiming,Gu Sha	预防医学论坛	Preventive Medicine Forum	15	12	1211-1212,1215	2009	-
2536	场所从业人员肠道沙门菌	c Salmonella among worke	春,帅慧群,赵雪琴,张睿,胡	uai Huiqun,Zhao Xueqin,Zh	中国卫生检验杂志	ese Journal of Health Inspe	19	8	1895-1898	2009	-
2537	干炎合并猪霍乱沙门氏菌	c Hepatitis B Complicated w	刘光美.	Liu Guangmei.	护理研究	nursing research	23	14	1312-1312	2009	sn.1009-6493.2009.14.058
2538	用冰块微生物污染状况调	microbial contamination of	宋晓荷.	Song Xiaohe.	浙江预防医学	hejiang Preventive Medicin	21	8	45-46	2009	sn.1007-0931.2009.08.025
2539	甲副伤寒沙门菌PFGE数据	Yunnan ProvincePFGEData	坤,阚飙,刁保卫,崔志刚,	Biao,Diao Baobao,Cui Zhig	疾病监测	disease surveillance	24	10	770-773	2009	-
2540	共场所从业人员沙门菌感	ella infection among work	金利胜.	Jin Lisheng.	浙江预防医学	hejiang Preventive Medicin	21	9	25,28	2009	sn.1007-0931.2009.09.011
2541	品中食源性病原菌污染状	odborne pathogenic conta	胡俊,斯国静,俞骅,王一泓	,Si Guojing,Yu Hua,Wang Y	中国卫生检验杂志	ese Journal of Health Inspe	19	9	2152-2153,2156	2009	-
2542	7～2008食源性致病菌污	of contamination status of	梅建华,兰进权,柳付明,陈	i Jianhua,Lan Jinquan,Liu F	中国卫生检验杂志	ese Journal of Health Inspe	19	7	1598-1599	2009	-
2543	甲型副伤寒沙门菌耐药基	enes of multidrug-resistan	李军,陈桂林,黄海,陶建蜀	hen Guilin,yellow sea,Tao J	济宁医学院学报	rnal of Jining Medical Colle	32	4	233-235	2009	sn.1000-9760.2009.04.002
2544	家宴沙门菌食物中毒调	ysis of salmonella food poi	成锡山,付玉亮.	Cheng Xishan,Fu Yuliang.	山西医药杂志（下半月版）	cal Journal (Second Half M	38	4	381	2009	0253-9926-B.2009.04.069
2545	08南京市食品污染物污染	ood contaminant contami	晓,陈晓蔚,叶艳华,曾理,王	en Xiaowei,Ye Yanhua,Zeng	预防医学情报杂志	of Preventive Medicine Info	25	6	399-401	2009	-
2546	沙门菌的分离鉴定及耐药	and Drug Resistance Study	刘立志,吴锦如,游鹏.	Liu Lizhi,Wu Jinru,You Peng	实用预防医学	ractical preventive medicin	16	5	1599-1600	2009	sn.1006-3110.2009.05.117
2547	08年奥运相关餐饮业食源	esults of foodborne pathog	-	-	微量元素与健康研究	e elements and health rese	26	4	33-34	2009	-
2548	05-2008年细菌性食物中毒	Analysis of the etiological	苏碧华.	Su Bihua.	华南预防医学	uth China Preventive Medic	35	5	61-63	2009	-
2549	奥运会期间肠道传染病病	tinal infectious diseases du	秋,高志勇,窦相峰,张海	,Gao Zhiyong,Dou Xiangfer	中华预防医学杂志	se Journal of Preventive Me	43	9	789-792	2009	sn.0253-9624.2009.09.010
2550	肠杆菌和沙门氏菌耐药	nterobacteriaceae and Sal	王玉东,郑增忍,赵思俊,黄	dong,Zheng Zengren,Zhao	家禽科学	poultry science	-	6	8-10	2009	sn.1673-1085.2009.06.003
2551	为主要临床表现的艾滋病	he main clinical manifestat	彭继承,罗宜辉,李运泽.	ng inherits,Luo Yihui,Li Yun	中国实用医药	Chinese Practical Medicine	4	31	66-67	2009	sn.1673-7555.2009.31.046

2552	大兴区食品污染物监测结	contaminant monitoring re	付士武,王桂侠.	Fu Shiwu,Wang Guixia.	中国卫生检验杂志	ese Journal of Health Inspe	19	10	2388-2389	2009	-
2553	门氏菌引起的群体性食物	mass food poisoning cause	郭欣,张中伟,杨辉,孙玲.	Zhang Zhongwei,Yang Hui,	国外医学（医学地理分册）	Medicine (Medical Geograp	30	4	185-186	2009	sn.1001-8883.2009.04.009
2554	霍乱沙门菌引起心内膜感染	aesuis causing endocardial	蒋利君,李钰,韦柳华.	iang Lijun,Li Yu,Wei Liuhua	中华医院感染学杂志	ournal of Hospital Infectiou	19	5	513-513	2009	sn:1005-4529.2009.05.048
2555	物中毒的病原菌的快速诊断	ceability of pathogenic bac	曼,李远奇,兰全学,刘涛,林	Yuanqi,Lan Quanxue,Liu Ta	中国热带医学	Chinese tropical medicine	9	6	1144-1145,1005	2009	-
2556	社区环境中沙门菌特性研究	eristics of Salmonella in con	孙吉能,周艳.	Sun Jineng,Zhou Yan.	社区医学杂志	urnal of Community Medici	7	11	27-28	2009	-
2557	星级酒店高风险食品连续监	analysis of high-risk foods i	巴蕾.	Bale.	职业与健康	Occupation and health	25	21	2285-2287	2009	-
2558	年重庆市人群沙门菌监测	sis of Salmonella in Chongq	刚,廖春艳,何源,赵波,王	Liao Chunyan,He Yuan,Zha	疾病监测	disease surveillance	24	5	325-328	2009	sn.1003-9961.2009.05.006
2559	体检肠道致病菌检测方法	intestinal pathogenic bact	昆蓉,杨梅,黄剑屏,周渝蓉	,bayberry,Huang Jianping,	预防医学情报杂志	of Preventive Medicine Info	25	5	435-436	2009	-
2560	2007年各酒店冷食肉制品	giene quality monitoring of	孙芳,张一飞,葛素君.	un Fang,Zhang Yifei,Ge Suju	中国卫生检验杂志	ese Journal of Health Inspe	19	4	910-911	2009	-
2561	菌爆发的菌株鉴定和分子	molecular traceability study	斌,刁保卫,王红霞,陈军,周	aobao,Wang Hongxia,Chen	上海预防医学	hanghai Preventive Medicin	21	12	593-596	2009	sn.1004-9231.2009.12.003
2562	科患者血培养阳性病原菌	bacteria with positive bloo	自镛,李丽,张蓓,陈中举,王	Li Li,Zhang Bei,Chen Zhongj	中国感染与化疗杂志	ournal of Infection and Che	9	4	276-279	2009	sn:1009-7708.2009.04.010
2563	利丹沙门氏菌致食物中毒	d poisoning incident cause	芳,潘京海,余晓辉,许晓	g,Pan Jinghai,Yu Xiaohui,Xu	首都公共卫生	capital public health	3	5	219-221	2009	sn.1673-7830.2009.05.011
2564	泳在沙门菌食物中毒调查	phoresis in investigation an	陈伟伟,钟凌,叶玲清,陈建	ei,Zhong Ling,Ye Lingqing,C	海峡预防医学杂志	s Journal of Preventive Me	15	3	54-55	2009	-
2565	感染性腹泻病原菌分布及耐	nce status of pathogenic ba	陈洁,阮冰.	Chen Jie,Ruan Bing.	浙江预防医学	hejiang Preventive Medicin	21	1	1-3,51	2009	sn.1007-0931.2009.01.001
2566	离病原菌的菌群分布及耐	in drug resistance of patho	程黎明,简翠,孙自镛.	eng Liming,Jian Cui,Sun Ziyo	内科急危重症杂志	f Internal Medicine and Cri	15	4	210-212	2009	sn.1007-1024.2009.04.016
2567	猪肉中沙门氏菌的检测与分	nd analysis of Salmonella in	冬梅,董开忠,雒晓芳,程燕	i,Dong Kaizhong,Luo Xiaofa	民族大学学报（自然科学	niversity for Nationalities (	30	2	63-65,90	2009	sn.1009-2102.2009.02.014
2568	逊沙门菌分离株的分子	sis of Salmonella Thompson	学斌,李勤,张代涛,陈春霞	in,Li Qin,Zhang Daitao,Che	疾病监测	disease surveillance	24	6	422-425	2009	-
2569	年动物源性航空食品微生	al testing and analysis of a	刘艳华,高璟瑜,李梅,汪明	hua,Gao Jingyu,Li Mei,Wan	中国动物检疫	China Animal Quarantine	26	6	50-51	2009	n.1005-944X.2009.06.023
2570	沙门氏菌食物中毒的病原学	ysis of a case of salmonella	史煜曼,刘赛琴,翁奕敏.	Yuman,Liu Saiqin,Weng Yin	国际检验医学杂志	onal journal of laboratory	30	8	799-800	2009	sn.1673-4130.2009.08.035
2571	盐田区食品中常见食源性	mmon foodborne pathogen	蔡俊源.	Cai Junyuan.	华南预防医学	uth China Preventive Medic	35	3	60-61,65	2009	-
2572	004～2009年食源性致病	2009Annual Foodborne Pa	,黄善盛,梁均和,陈子慧,	nsheng,Liang Junhe,Chen	中国热带医学	Chinese tropical medicine	9	12	2225-2226	2009	-
2573	细菌性食物中毒的调查与	analysis of a case of bacter	杨玉英,陈志华.	Yang Yuying,Chen Zhihua.	中外医疗	Chinese and foreign medica	28	32	117	2009	sn.1674-0742.2009.32.098
2574	起沙门氏菌导致的食物中	food poisoning caused by s	尉建林,郭定成,牛金虎.	ianlin,Guo Dingcheng,Niu J	医药与保健（下旬版）	ine and Health Care (late e	17	8	48-49	2009	-
2575	贝类养殖区菲律宾蛤仔	hilippine clams in the main	郑燕,蔡雷鸣.	Zheng Yan,Cai Leiming.	福建水产	Fujian aquatic products	-	4	62-66	2009	sn.1006-5601.2009.04.013
2576	物源大肠杆菌、沙门杆菌耐	animal-derived Escherichi	述永,舒娅,李蕊艳,王琦,万	ng,Shuya,Li Ruiyan,Wang Q	黑龙江畜牧兽医	nimal Husbandry and Veter	-	4	93-94	2009	sn.1004-7034.2009.04.042
2577	区儿童感染性腹泻的病原	of infectious diarrhea in ch	永强,邓秋连,虢艳,万根平	ng,Deng Qiulian,Guo Yan,W	中国当代儿科杂志	Journal of Contemporary P	11	2	107-109	2009	-
2578	国其他感染性腹泻报告病例	reported cases of infectiou	,高永军,冯子健,王子军,	Yongjun,Feng Zijian,Wang	世界华人消化杂志	rld Chinese Journal of Diges	17	32	3370-3375	2009	sn.1009-3079.2009.32.019
2579	转为食物中毒突发公共卫	ease epidemic that turned	刘国蓉,左晨.	Liu Guorong,Zuo Chen.	中国预防医学杂志	se Journal of Preventive Me	10	2	160-161	2009	-
2580	急性感染性腹泻病原菌监	f pathogenic bacteria in acu	于国慧,宋文琪,甄景慧.	uohui,Song Wenqi,Zhen Jin	中国妇幼保健	ina maternal and child hea	24	18	2506-2508	2009	-
2581	源性沙门菌的分布菌型及耐	mination test of foodborne	周艳,杨赟,张晓亮	Yang Yun,Zhang Xiaoliang,	中国现代医生	modern chinese doctor	47	15	191-192,242	2009	sn.1673-9701.2009.15.102
2582	一起甲型副伤寒暴发流行	break of paratyphoid fever	泽,马世武,何海英,禹征,	a Shiwu,He Haiying,Yu Zhe	中华临床感染病杂志	ournal of Clinical Infectious	2	5	314-315	2009	sn.1674-2397.2009.05.016
2583	饮业直接入口食品食源性	ic bacteria in directly impo	余红,封会茹,赵伟,董晓根	Feng Huiru,Zhao Wei,Dong	现代预防医学	modern preventive medicin	36	21	4038-4040	2009	-
2584	鼠伤寒沙门菌性心肌炎	acute typhoid salmonella	赵云霞,吉世军,刘倩,白静	Yunxia,Ji Shijun,Liu Qian,Ba	医药论坛杂志	Medical Forum Magazine	30	14	29-30	2009	-
2585	深圳市某区禽肉微生物检	testing results of poultry m	严燕,史卫军.	Yan Yan,Shi Weijun.	华南预防医学	uth China Preventive Medic	35	5	37,41	2009	-
2586	床型奶牛乳腺炎病原菌的	pathogenic bacteria of clini	李桐,刘鑫,张裕琪,杨鸣琦	g,Liu Xin,Zhang Yuqi,Yang M	动物医学进展	vances in Veterinary Medic	30	7	115-119	2009	sn.1007-5038.2009.07.031
2587	江苏省食源性致病菌监测	is of foodborne pathogenic	袁宝君,戴建华,乔昕,沈赟	bjun,Dai Jianhua,Qiao Xin,S	中国食品卫生杂志	hinese Food Hygiene Journ	21	2	114-116	2009	-
2588	品食源性致病菌污染状况	ic bacteria contamination i	,刘长宏,马国柱,石一,张	ghong,Ma Guozhu,Shi Yi,Z	中国卫生检验杂志	ese Journal of Health Inspe	19	9	2111-2112	2009	-

2589	饲料中沙门氏菌的分离鉴定	tion and analysis of Salmor	何继军,王彪.	He Jijun,Wang Biao.	中国农业科技导报	ultural Science and Techno	11	z1	56-58	2009	sn.1008-0864.2009.z1.018
2590	起鼠伤寒沙门氏菌食物中	nology in traceability of Sal	,黄纯健,唐振柱,王红,李秀	g Chunjian,Tang Zhenzhu,W	应用预防医学	pplied preventive medicin	15	5	259-261	2009	n.1673-758X.2009.05.002
2591	患者颈部甲型副伤寒沙门菌	ss in the neck of a patient	王晓洲,栾晓军.	Vang Xiaozhou,Luan Xiaoju	内科急危重症杂志	f Internal Medicine and Cri	15	1	52-53	2009	sn.1007-1024.2009.01.023
2592	售食品中食源性致病菌检	hogenic bacteria in comme	丽萍,徐岚,何晓砚,李歆	ing,Xu Lan,He Xiaoyan,Li Xi	实用预防医学	ractical preventive medicin	16	6	1835-1837	2009	sn.1006-3110.2009.06.056
2593	沙门菌暴发疫情病原体检验	drug susceptibility test of a	磊,邓辉,秦启忠,魏娅,庞明	Hui,Qin Qizhong,Wei Ya,Pa	中国预防医学杂志	se Journal of Preventive Me	10	1	68-69	2009	-
2594	海市沙门菌血清型流行特	teristics of Salmonella serot	金汇明,冉陆,刁保卫,肖文	,Ran Lu,Diao Baobao,Xiao	中国人兽共患病学报	Chinese Journal of Zoonose	25	2	156-158	2009	sn.1002-2694.2009.02.017
2595	分离株的鉴定及与丙型副	tbreak isolates and their di	,许学斌,李勤,王晓梅,崔志	uebin,Li Qin,Wang Xiaome	中国人兽共患病学报	Chinese Journal of Zoonose	25	4	348-351	2009	sn.1002-2694.2009.04.013
2596	合医院住院病案细菌污染调	mination of inpatient med	张保刚,于文红,赵文申.	ogang,Yu Wenhong,Zhao V	中国消毒学杂志	hinese Journal of Disinfecti	26	2	191-192	2009	-
2597	疆沙湾县一起食物中毒调	ort on food poisoning in Sh	·阿衣铺,于美,麦丽开·阿	l·Ayipu,Yu Mei,Mai Likai·A	地方病通报	ndemic disease notificatio	24	3	66-66	2009	-
2598	共场所从业人员沙门菌带	carriers among workers in	冼桂江,劳希,林宇,盘珍梅	n Guijiang,Rausch,Lin Yu,Pa	中国卫生检验杂志	ese Journal of Health Inspe	19	8	1865-1866,1869	2009	-
2599	型副伤寒沙门菌致肝脓肿	aused by Salmonella Paraty	林秉滔,阮宏标,姚秀卿.	gtao,Ruan Hongbiao,Yao X	现代中西医结合杂志	grated Traditional Chinese	18	3	305-306	2009	sn.1008-8849.2009.03.065
2600	沙门氏菌引起食物中毒的	s of a case of food poisonir	许华.	Xu Hua.	医学信息（医药版）	nformation (Pharmaceutica	-	11	-	2009	-
2601	菌引起细菌性食物中毒的	n bacterial food poisoning	-	-	中国中医药咨讯	litional Chinese Medicine In	-	4	-	2009	-
2602	猪沙门氏菌病的流行与防制	nd prevention of swine sal	潘培生.	Pan Peisheng.	中国猪业	China pig industry	4	12	36-37	2009	-
2603	登堡沙门菌流行特征和分	s and molecular typing of S	文佳,顾宝柯,陈敏,冉陆,刁	a,Gu Baoke,Chen Min,Ran L	中华流行病学杂志	inese Journal of Epidemiolo	30	9	933-937	2009	sn.0254-6450.2009.09.018
2604	2008年儿童感染性腹泻病	is of the etiology of infecti	董方,刘锡清,许宏,宋文琪	ng,Liu Xiqing,Xu Hong,Song	中国医刊	Chinese Medical Journal	44	9	28-30	2009	sn.1008-1070.2009.09.012
2605	南省食源性致病菌的监测	sults of foodborne pathoge	秀丽,廖兴广,张丁,胡巅,张	guang,Zhang Ding,Hu Dian	中国卫生检验杂志	ese Journal of Health Inspe	19	1	173-175,192	2009	-
2606	护病房阿伯丁沙门菌医院	lla Aberdeen nosocomial in	李卫光,朱其凤.	Li Weiguang,Zhu Qifeng.	中华医院感染学杂志	ournal of Hospital Infectiou	19	15	1954-1955	2009	sn:1005-4529.2009.15.011
2607	院血培养中病原菌的分布及	pathogenic bacteria in bloo	平,杨启红,唐爱国,姜叶灵	ang Qihong,Tang Aiguo,Jian	中国抗生素杂志	hinese Journal of Antibioti	-	1	39	2009	-
2608	肠杆菌和沙门菌对喹诺酮	tance of Escherichia coli an	,王晓泉,丛秋霞,潘志明,高	Xiaoquan,Cong Qiuxia,Par	中国人兽共患病学报	Chinese Journal of Zoonose	25	7	630-635	2009	sn.1002-2694.2009.07.005
2609	氏菌对抗菌药物的耐药性	esistance of Salmonella to a	刘素梅.	Liu Sumei.	安徽农业科学	Anhui Agricultural Sciences	37	23	11023-11024,11031	2009	sn.0517-6611.2009.23.084
2610	症患者甲型副伤寒沙门菌	nce of Salmonella paratyph	秀芬,刘晓莉,杨旭,胡芸,吴	n,Liu Xiaoli,Yang Xu,Hu Yun	检验医学	laboratory medicine	24	4	259263	2009	sn.1673-8640.2009.04.028
2611	08年荆州市食源性致病菌	nalysis of foodborne patho	胡国才,益琼,石韬.	Hu Guocai,Yi Qiong,Shi Tao	中国病原生物学杂志	ese Journal of Pathogen Bio	4	10	742-743,762	2009	-
2612	年钦州市伤寒、副伤寒86	paratyphoid fever in Qinzh	陈焯彬,马卫国,姚钦江.	Zhuobin,Ma Weiguo,Yao Qi	内科	Internal Medicine	4	1	5-7	2009	sn.1673-7768.2009.01.003
2613	术在3起沙门菌食物中毒调	in3Application in Salmonel	,罗芸,叶菊莲,朱敏,潘雪霞	o Yun,Ye Julian,Zhu Min,Pa	中国卫生检验杂志	ese Journal of Health Inspe	19	12	2954-2956	2009	-
2614	霍乱沙门菌左膝局部脓肿	eft knee due to salmonella	兵,杨建华,刘军,张国英,戴	g Jianhua,Liu Jun,Zhang Gu	中国感染与化疗杂志	urnal of Infection and Che	9	6	469	2009	sn:1009-7708.2009.06.015
2615	~2008年伤寒沙门菌脉冲场	dy on molecular typing of S	金玉娟,甘莉萍,杨慧,杨坤祥	,Gan Liping,Yang Hui,Yang	中国卫生检验杂志	ese Journal of Health Inspe	19	7	1540-1541,1577	2009	-
2616	巴结中沙门菌的分离及血	ification of Salmonella from	李刚.	Li Gang.	中国兽医杂志	Chinese Veterinary Journal	45	10	79-80	2009	-
2617	床标本细菌培养结果与药敏	results and drug susceptib	劳炳焕,林岗,朱庆明.	Binghuan,lingang,Zhu Qingr	检验医学与临床	boratory medicine and clini	6	5	361-362	2009	sn.1672-9455.2009.05.022
2618	沙门氏菌污染状况调查及抗	f livestock products in supe	平,胡慧,李苗云,赵改名,黄	Miaoyun,Zhao changed his	中国病原生物学杂志	ese Journal of Pathogen Bio	4	6	475-475,477	2009	-
2619	菌性腹泻病原菌分布及耐	g resistance of pathogenic	李文郎,陈爱华.	Li Wenlang,Chen Aihua.	中国医药导刊	China Medical Guide	11	3	478-479	2009	sn.1009-0959.2009.03.069
2620	鼠伤寒沙门氏菌的分离和鉴	ification of Salmonella typh	国栋,张文东,黄文忠,马坤	Zhang Wendong,Huang We	现代畜牧兽医	mal husbandry and veterina	-	10	51-52	2009	sn.1672-9692.2009.10.029
2621	切斯特沙门菌致肺脓肿1例	ella chester lung abscess1e	李光才.	Li Guangcai.	中外医学研究	se and foreign medical res	7	11	191	2009	sn.1674-6805.2009.11.168
2622	门氏菌的分离与鉴定及生	acteristics observation of S	建,高继业,唐妤,汪敏,李维	n,Gao Jiye,Tang Yu,Wang M	中国畜牧兽医	mal husbandry and veterina	36	5	203-205	2009	-
2623	养中分离出肠沙门菌维尔	a enterica serotype Virchov	新元,姜艳梅,韩丽霞,王贞	n,Jiang Yanmei,Han Lixia,W	中华检验医学杂志	e Journal of Laboratory Me	32	3	351	2009	sn.1009-9158.2009.03.031
2624	起细菌性食物中毒调查报	eport on a case of bacterial	张玉波,刘艳波,王双军.	Yubo,Liu Yanbo,Wang Shua	中国保健	China health care	-	32	-	2009	-
2625	动脉瓣赘生物及瓣周脓肿	ombined with aortic valve	燕杭,何炼图,叶素敏,许峭	ang,He Liantu,Ye Sumin,Xu	中华超声影像学杂志	se Journal of Ultrasound Im	18	3	193	2009	sn.1004-4477.2009.03.003

2626	门菌PFGE分型分析及数据	tyPFGETyping analysis and	,汤敏,张宏萍,张伟,赵哲,	,Zhang Hongping,Zhang We	交通医学	traffic medicine	23	5	461-464	2009	-
2627	大肠杆菌和沙门氏菌耐药	f Escherichia coli and Salm	娜,赵思俊,黄秀梅,龚振	Sijun,Huang Xiumei,Gong	中国动物检疫	China Animal Quarantine	26	5	64-65	2009	n.1005-944X.2009.05.029
2628	检出肠炎沙门菌误诊为伤	cerebrospinal fluid of one	张清杰,王爱梅.	Zhang Qingjie,Wang Aimei.	中国卫生检验杂志	ese Journal of Health Inspe	19	8	19181923	2009	-
2629	霉素治疗小儿沙门氏菌感	treatment of salmonella i	何涓.	He Juan.	社区医师（医学专业半月	ty Physician (Medical Profe	11	19	95	2009	sn.1007-614x.2009.19.123
2630	一起由猪霍乱沙门氏菌引	soning caused by Salmonel	尹薛荣,王群.	Yin Xuerong,Wang Qun.	地方病通报	endemic disease notificatio	24	1	63-63	2009	-
2631	氏菌O9引起细菌性食物中	igation and analysis of bac	青,吴吟涓,唐飞,吴维权,香	Yinjuan,Tang Fei,Wu Weid	中国基层医药	China primary medicine	16	7	1294-1295	2009	sn.1008-6706.2009.07.105
2632	寒沙门氏菌血液感染的临	ntyphoidal Salmonella bloo	素明,杨静,崔恩博,鲍春梅	ming,Yang Jing,Cui Eunbo,E	肝脏	liver	14	4	281-283	2009	sn.1008-1704.2009.04.005
2633	209株沙门菌PFGE指纹图	ce209SalmonellaPFGEFing	菊莲,朱敏,张俊彦,潘军航	hu Min,Zhang Junyan,Pan J	中国卫生检验杂志	ese Journal of Health Inspe	19	11	2478-2481,2492	2009	-
2634	食品中沙门菌污染情况调	s of Salmonella contamina	进权,雷永良,陈沙彬,李永	,Lei Yongliang,Chen Shabi	中国卫生检验杂志	ese Journal of Health Inspe	19	10	2392-2394	2009	-
2635	徐州市食源性致病菌污染	ation status of foodborne	,杨晋川,张惠,张雷,郭惠,	chuan,Zhang Hui,Zhang Le	中国卫生检验杂志	ese Journal of Health Inspe	19	1	189-190	2009	-
2636	市区2004-2008年食源性致	nce2004-2008Annual surv	均和,黄善盛,吴瑞英,陈子	ang Shansheng,Wu Ruiying	中国食品卫生杂志	hinese Food Hygiene Journ	21	6	536-538	2009	-
2637	中同时检出肠炎沙门菌和	ureus were simultaneously	王锦彤,田建伟,熊定凯.	intong,Tian Jianwei,Xiong D	中国国境卫生检疫杂志	ntier Health and Quarantin	32	1	43-44	2009	-
2638	性腹泻病原菌10年变迁与	children10Annual changes	纪文静,董方,徐樨巍.	Venjing,Dong Fang,Xu Weiv	中国实用儿科杂志	se Journal of Practical Pedi	24	12	934-936	2009	-
2639	城市留鸟携带人体病原微	microorganisms carried by r	金志民,杨春文,刘铸.	Zhimin,Yang Chunwen,Liu Z	安徽农业科学	Anhui Agricultural Sciences	37	28	13641-13642	2009	sn.0517-6611.2009.28.079
2640	及其养殖环境中食源性致	c bacteria in tilapia and its	,吴燕燕,李来好,杨贤庆,	nyan,Li Laihao,Yang Xianqi	安徽农业科学	Anhui Agricultural Sciences	37	21	10026-10028,10031	2009	sn.0517-6611.2009.21.082
2641	务行业从业人员肠道致病	on results among service i	卫红,王惠峰,沈春莉,谢晓	Wang Huifeng,Shen Chunli,	职业与健康	Occupation and health	25	17	1860-1861	2009	-
2642	沙门菌耐药性及PFGE分	i drug resistance andPFGE	,李孝权,邓志爱,陈守义,	quan,Deng Zhiai,Chen Shou	中国卫生检验杂志	ese Journal of Health Inspe	19	6	1222-1224,1339	2009	-
2643	脑脊液中检出肠炎沙门菌	monella Enteritidis from the	王爱梅.	Wang Aimei.	中国城乡企业卫生	ygiene in urban and rural a	-	4	92-93	2009	-
2644	～2007年食源性致病菌监	Analysis of annual foodborn	,侯秀丽,李文,关冰,邵坤,	u Xiuli,Li Wen,Guan Bing,S	中国公共卫生管理	ese public health manager	25	1	98-99	2009	-
2645	99-2007年伤寒副伤寒流行	annual epidemic characte	克诚,游旅,马青,韦小俞,	g,Travel,Ma Qing,Wei Xiao	医学动物防制	medical animal control	25	3	161-163,165	2009	ssn.1003-6245.2009.03.01
2646	业人员沙门菌携带状况流	monella carrying status am	宇岚,何德辉,麦婉湘,覃昆	e Dehui,Mai Wanxiang,Qin	中国热带医学	Chinese tropical medicine	9	5	911-912,914	2009	-
2647	炎沙门菌流行特征和分子	ics and molecular typing o	明,肖文佳,顾宝柯,陈敏,	Xiao Wenjia,Gu Baoke,Che	上海预防医学	hanghai Preventive Medici	21	4	149-152,195	2009	sn.1004-9231.2009.04.001
2648	疫情中病例的耐药特征	eatment efficacy evaluatio	杨进,全文郎,王鸣柳,张杰	uan Wenlang,Wang Mingliu	中国热带医学	Chinese tropical medicine	9	10	1970-1971	2009	-
2649	沙门菌引起的食物中毒调	into food poisoning caused	子媛媛,朱海兰.	Zi Yuanyuan,Zhu Hailan.	预防医学论坛	Preventive Medicine Forum	15	5	478-479	2009	-
2650	2007年不同来源沙门菌的	study on drug resistance of	红丽,赵建宏,马玉国,宋红	Zhao Jianhong,Ma Yuguo,S	临床误诊误治	al misdiagnosis and mistrea	22	9	14-16	2009	sn.1002-3429.2009.09.011
2651	禽沙门氏菌的分离和鉴定	nd identification of avian S	王利明,刘宏艳.	Wang Liming,Liu Hongyan.	畜牧兽医科技信息	Veterinary Science and Te	-	2	27	2009	-
2652	菌引起细菌性食物中毒的	n bacterial food poisoning	丁学霞,陈永俊.	Ding Xuexia,Chen Yongjun.	中国中医药咨讯	itional Chinese Medicine I	-	4	-	2009	-
2653	染物和食源性致病菌监测	contaminants and foodbo	修军,王岙,刘桂华,刘思洁	Xiujun,Wang Ao,Liu Guihua	中国卫生检验杂志	ese Journal of Health Inspe	19	9	2115-2117	2009	-
2654	04-2006年甲型副伤寒流行	n the epidemic characterist	明,许国章,徐景野,毛国华,	Guozhang,Xu Jingye,Mao G	中国预防医学杂志	se Journal of Preventive Me	10	10	928-931	2009	-
2655	人血培养病原菌分布及耐	tance of pathogenic bacter	张金锋,方晔.	Zhang Jinfeng,Fang Ye.	中国卫生检验杂志	ese Journal of Health Inspe	19	11	2632-2634	2009	-
2656	沙门菌引起的食物中毒及	isoning and drug resistance	周玖英.	Zhou Jiuying.	医学信息	medical information	22	12	2718-2719	2009	sn.1006-1959.2009.12.052
2657	工肉鸡中沙门氏菌风险评	assessment in processed	玲,许喜林,周彦良,芮汉明	Xu Xilin,Zhou Yanliang,Rui	现代食品科技	Modern food technology	25	7	825-829	2009	sn.1673-9078.2009.07.031
2658	院艾滋病患者肺部病变26	tients in general hospitals	杰,周兴荣,张雪漫,李红兵,	ingrong,Zhang Xueman,Li	四川医学	Sichuan Medicine	30	2	205-207	2009	sn.1004-0501.2009.02.024
2659	门菌血清学鉴定和药敏试	and drug susceptibility test	岳凤,杨正林.	Yue Feng,Yang Zhenglin.	浙江预防医学	hejiang Preventive Medici	21	10	38-39	2009	sn.1007-0931.2009.10.017
2660	寒沙门菌感染42例临床及	tion in children42Clinical a	叶茂.	Ye Mao.	儿科药学杂志	ournal of Pediatric Pharma	15	4	33-35	2009	-
2661	生畜禽肉沙门菌污染监测	lla Contamination in Livest	张梦寒.	Zhang Menghan.	江苏预防医学	iangsu Preventive Medicin	20	4	41-42	2009	sn.1006-9070.2009.04.023
2662	散装熟肉制品微生物时空	of microorganisms in bulk	韦镇萍,韩方岸,柳艳.	Zhenping,Han Fang'an,Liu	职业与健康	Occupation and health	25	20	2171-2173	2009	-

2663	盐县市售食品中食源性致	teria contamination in com	,陆文彪,徐佩华,周晓红,	biao,Xu Peihua,Zhou Xiao	疾病监测	disease surveillance	24	8	573-575	2009	sn.1003-9961.2009.08.006
2664	07年伤寒副伤寒流行病学	l and etiological studies of	爱明,金春光,毛国华,石优	hunguang,Mao Guohua,Sh	中华流行病学杂志	inese Journal of Epidemiolo	30	3	252-256	2009	sn.0254-6450.2009.03.013
2665	市伤寒沙门菌多位点序列	ence typing of Salmonella t	,潘劲草,张蔚,郑伟,俞骅,	ao,Zhang Wei,Zheng Wei,Y	中国卫生检验杂志	ese Journal of Health Inspe	19	8	1751-1753	2009	-
2666	对从业人员肠道致病菌筛	tion of enteric pathogen sc	汪武新,谢远辉,张倩,黄新,	in,Xie Yuanhui,Zhang Qian,	中国热带医学	Chinese tropical medicine	9	1	12-13	2009	-
2667	仔猪腹泻流行病学调查及	and prevention research or	许开云.	Xu Kaiyun.	中国动物检疫	China Animal Quarantine	26	11	56-57	2009	n.1005-944X.2009.11.029
2668	沙门氏菌引起食物中毒	food poisoning caused by S	琼,高翠文,朱怀甫,李世	Cuiwen,Zhu Huaifu,Li Shi D	中外健康文摘	hese and Foreign Health Di	6	33	18-19	2009	sn.1672-5085.2009.33.010
2669	寒沙门菌引起食物中毒的	e of food poisoning caused	庞惠勇,田锦萍,孙波.	g Huiyong,Tian Jinping,Sun	中国卫生检验杂志	ese Journal of Health Inspe	19	12	2980-2981	2009	-
2670	4-2007年旺苍县食物中毒	ysis of food poisoning in W	肖汉平.	Xiao Hanping.	预防医学情报杂志	of Preventive Medicine Info	25	6	424-426	2009	-
2671	炎沙门氏菌引起食物中毒	of food poisoning caused	刘万郁.	Liu Wanyu.	现代预防医学	modern preventive medicin	36	24	4675-4675,4683	2009	-
2672	分离出甲型副伤寒沙门菌	nella paratyphi A from bile	宋玉英,朱亚梅,王丽霞.	g Yuying,Zhu Yamei,Wang L	检验医学与临床	boratory medicine and clini	6	7	544-544	2009	sn.1672-9455.2009.07.042
2673	昌市食品食源性致病菌检	rne pathogenic bacteria in	仝玉平.	Tong Yuping.	中国卫生检验杂志	ese Journal of Health Inspe	19	12	2938-2939	2009	-
2674	伤感染阿伯丁沙门菌一	monella aberdeen infectio	李梅芳,张凤杰,王建国.	ang,Zhang Fengjie,Wang Ji	江西医学院学报	rnal of Jiangxi Medical Coll	49	5	14	2009	sn.1000-2294.2009.05.038
2675	门菌所致人感染性动脉瘤	an infectious aneurysms ca	,符伟国,蒋俊豪,陈斌,史振	Weiguo,Jiang Junhao,Chen	中华普通外科杂志	hese Journal of General Sur	24	9	715-717	2009	n.1007-631X.2009.09.009
2676	南部干紫菜几种致病菌	everal pathogenic bacteria	夕和,王李宝,胡传明,凌云	ang Libao,Hu Chuanming,Li	中国卫生检验杂志	ese Journal of Health Inspe	19	9	2142-2143	2009	-
2677	食品相关致病菌的污染机	of pathogenic bacteria rel	,王靖飞,付朝阳,郭莹莹,	Jingfei,Fu Chaoyang,Guo Y	畜牧兽医科技信息	d Veterinary Science and Te	-	12	4-5	2009	N.1671-6027.2009.12.004
2678	上海市闵行区腹泻病病原	of diarrheal pathogenic ba	徐彩萍,汪萍,陈秀华,刘芸,	Caiping,Wang Ping,Chen X	疾病监测	disease surveillance	24	12	920-923	2009	sn.1003-9961.2009.12.007
2679	霍乱沙门氏菌感染21例临	infection in childrentwenty	,赵建美,徐美玉,褚少朋,李	Jianmei,Xu Meiyu,Chu Sh	中国当代儿科杂志	Journal of Contemporary P	11	12	1012-1013	2009	-
2680	源性致病菌的污染现状	tance status of major food	容,李小春,董雪,陈佩,金	Xiaochun,Dong Xue,Chen	中国预防医学杂志	e Journal of Preventive Me	10	4	290-292	2009	-
2681	综合征合并伤寒、脓肿、	ne combined with typhoid	周蕾,郭宁茹.	Zhou Lei,Guo Ningru.	天津医药	Tianjin Medicine	37	2	160,插3	2009	sn.0253-9896.2009.02.036
2682	间隙猪霍乱沙门菌感染一	nella choleraesuis infectio	龚旭,周强.	Gong Xu,Zhou Qiang.	中华骨科杂志	hinese Journal of Orthoped	29	6	597	2009	sn.0253-2352.2009.06.020
2683	肠炎沙门菌引起的食物中	food poisoning caused by	正波,甘启芦,吴景文,蔡	gbo,Gan Qilu,Wu Jingwen,	海峡预防医学杂志	s Journal of Preventive Me	15	4	44-45	2009	-
2684	沙门氏菌引起的食物中毒	is of a case of food poisoni	苗晓红.	Miao Xiaohong.	中国现代药物应用	dern drug application in Ch	3	14	193	2009	sn.1673-9523.2009.14.162
2685	寒沙门菌扩增片段长度多	h polymorphism typing of S	,薄志坚,洪梅,万强,王敏,	ian,Hong Mei,Wan Qiang,W	中国公共卫生	Chinese public health	25	1	113-114	2009	sn:1001-0580.2009.01.058
2686	门菌毒力基因分布特征的	n characteristics of Salmon	,汤敏,张宏萍,张伟,赵哲,	Zhang Hongping,Zhang We	齐齐哈尔医学院学报	rnal of Qiqihar Medical Coll	30	19	2355-2357	2009	sn.1002-1256.2009.19.002
2687	76株沙门菌属菌群菌型分	cterial type distribution an	,贾红兵,李春英,王靖,李	,Li Chunying,Wang Jing,Li	中日友好医院学报	of China-Japan Friendship	23	4	213-215	2009	sn.1001-0025.2009.04.007
2688	2005年伤寒暴发流行特	e epidemic characteristi	吴曙光;	Wu Shuguang;	安徽预防医学杂志	Journal of Preventive Me	15	5	336-337+348	2009	-
2689	沿海地区一起伤寒暴发疫	eak of typhoid fever in a	富;林海江;常玥;杨艺;王	jiang;Chang Yue;Yang Y	中国预防医学杂志	e Journal of Preventive M	10	8	743-745	2009	/j.1009-6639.2009.08.032
2690	1起伤寒暴发的调查	ation into Typhoid Fever	刘琳;蒋有琴;	Liu Lin;Jiang Youqin;	预防医学论坛	reventive Medicine Foru	15	7	671-672	2009	sn.1672-9153.2009.07.031
2691	煤矿甲型副伤寒暴发的	outbreak of paratyphoid	国林;张华林;金长云;李	;Zhang Hualin;Jin Chan	中国热带医学	Chinese tropical medicine	9	3	533	2009	-
2692	州市越秀区食品质量监测	g and analysis in Yuexiu D	黄亮宇,朱紫杭.	Huang Liangyu,Zhu Zihang	中国社区医师（医学专业）	munity physician (medical	12	23	250252	2010	sn.1007-614x.2010.23.271
2693	市售鲜鸡肉微生物污染状	l contamination of fresh c	素红,郭善广,符小燕,蒋爱	uo Shanguang,Fu Xiaoyan,	现代食品科技	Modern food technology	26	7	746-749	2010	sn.1673-9078.2010.07.026
2694	2005-2007年食物中毒情	007Annual food poisoning	萍,李红,韩淑杰,邓建和,成	ong,Han Shujie,Deng Jianh	华南预防医学	uth China Preventive Medic	36	3	55-57	2010	-
2695	餐饮业食品安全风险性调	food safety risks in caterin	峰,何伦发,林海,黄国贤,曹	e Lunfa,Lin Hai,Huang Guo	中国卫生检验杂志	ese Journal of Health Inspe	20	5	1212-1214	2010	-
2696	寒沙门氏菌引起食物中毒	case of food poisoning caus	王金锋.	Wang Jinfeng.	医学动物防制	medical animal control	26	6	556	2010	sn.1003-6245.2010.06.032
2697	08年食源性疾病监测中沙	drug resistance of Salmone	李丽婕,李秀娟,赵冬.	i Lijie,Li Xiujuan,Zhao Dong	中国卫生检验杂志	ese Journal of Health Inspe	20	9	2262-2263	2010	-
2698	寒沙门菌败血症并发脾脓	himurium septicemia comp	,朱碧红,张慧芳,李庆兴,王	hong,Zhang Huifang,Li Qing	中华传染病杂志	se Journal of Infectious Dis	28	7	397	2010	sn.1000-6680.2010.07.004
2699	009年食品食源性致病菌	urvey on foodborne patho	瑞玲,岳红卫,刘小娟,陈克	,Yue Hongwei,Liu Xiaojuan	职业与健康	Occupation and health	26	8	877-879	2010	-

2700	沙门菌所致食物中毒的	case of food poisoning cau	,陈真,姚红,王姗,唐漪灵,	en,Yao Hong,Wang Shan,T	医学信息（下旬刊）	edical information (late issu	23	2	280-280	2010	-
2701	见传染性胚胎性疾病发病	on infectious embryonic di	智,祁学军,訾金良,邢莉,马	i,Qi Xuejun,Zi Jinliang,Xing	养禽与禽病防治	and poultry disease preven	-	7	35-37	2010	-
2702	沙门杆菌致化脓性关节	c arthritis caused by Salmo	张艳丽,陈坚,杨建华.	g Yanli,Chen Jian,Yang Jian	中华传染病杂志	se Journal of Infectious Dis	28	9	576	2010	sn.1000-6680.2010.09.020
2703	公共卫生事件网络报告食	reported by the National P	张昕,王子军,冉陆.	hang Xin,Wang Zijun,Ran L	疾病监测	disease surveillance	25	5	406-409	2010	sn.1003-9961.2010.05.020
2704	雅安市食源性病原菌监	lysis of foodborne pathog	永权,刘思寨,王俊琳,魏洪	n,Liu Sizhai,Wang Junlin,W	中国微生态学杂志	inese Journal of Microecol	22	4	337-338	2010	-
2705	者阴道分泌物中分离出	aginal secretions of patien	玉红,佟忠山,姜月红,遇平	Tong Zhongshan,Jiang Yue	中华医院感染学杂志	ournal of Hospital Infectiou	20	23	3728	2010	-
2706	患者细菌分离及药敏480例	ensitivity in patients with	,谢昌霞,程龙强,张伦军,	xia,Cheng Longqiang,Zhan	中华全科医学	Chinese general medicine	8	4	495-496	2010	-
2707	食品中食源性致病菌监测	of foodborne pathogenic b	燕,陈峰,马俊锋,沈辉,谭	n Feng,Ma Junfeng,Shen H	中国热带医学	Chinese tropical medicine	10	10	1205-1206	2010	-
2708	沙门菌引起百余人食物	than 100 people caused by	程徽.	Cheng Hui.	中国卫生检验杂志	ese Journal of Health Inspe	20	11	3036	2010	-
2709	起沙门菌肠炎暴发疫情溯	s on the source of Salmone	川,赵晋,徐耀方,罗隆泽,杨	n,Zhao Jin,Yaofang,Luo Lor	预防医学情报杂志	of Preventive Medicine Info	26	11	855-857	2010	-
2710	大中型宾馆饭店自制熟肉	ducts in large and medium	岳红,郭振平.	Yue Hong,Guo Zhenping.	河南预防医学杂志	n Journal of Preventive Me	21	4	318-319	2010	-
2711	细菌感染性腹泻的病原菌	eptibility test of bacterial i	颜善活,卓永光.	n Shanhuo,Zhuo Yongguan	检验医学与临床	boratory medicine and clini	7	9	848-850	2010	sn.1672-9455.2010.09.036
2712	共场所从业人员肠道沙门	esults among Workers in F	邓国,刘岚,五建军.	uo,Liu Lan,The fifth foundin	旅行医学科学	travel medical science	16	3	71-72	2010	sn.1006-7159.2010.03.029
2713	年衢州市食品中食源性致	oodborne pathogenic bact	尔,张建民,于丽莉,叶承华,	hang Jianmin,Yu Lili,Ye Che	中国预防医学杂志	se Journal of Preventive Me	11	6	588-590	2010	-
2714	堡沙门菌引起的婴幼儿腹	and young children caused	王爱英.	Wang Aiying.	现代中西医结合杂志	grated Traditional Chinese	19	16	2043-2043	2010	sn.1008-8849.2010.16.074
2715	90～2008年痢疾流行病学	of epidemiological charac	王戬,刘辉.	Wang Jian,Liu Hui.	现代预防医学	modern preventive medicin	37	3	427-428	2010	-
2716	8-2009年沙门菌感染性腹	current status of Salmone	桂荣,曲梅,高志勇,严寒秋,	Mei,Gao Zhiyong,severe c	中华流行病学杂志	inese Journal of Epidemiolo	31	12	1438-1439	2010	sn.0254-6450.2010.12.029
2717	省食品中食源性致病菌监	borne pathogenic bacteri	,张芳,潘立,马国柱,刘长	ng Fang,Pan Li,Ma Guozhu,	中国卫生检验杂志	ese Journal of Health Inspe	20	12	3439-3440,3442	2010	-
2718	原料奶中微生物污染程度	microbial contamination le	,陈庆森,阎亚丽,庞广昌,	Qingsen,Yan Yali,Pang Gua	食品科学	food science	31	17	351-354	2010	-
2719	包装小食品质量安全分析	ged snacks imported from	凌经昌,蓝翊文,谭灵,袁俊,	chang,Lan Yiwen,Tan Ling,	中国食品卫生杂志	hinese Food Hygiene Journ	22	6	540-541	2010	-
2720	嘉兴市食源性病原菌监	of foodborne pathogenic	黎霞,燕勇,高雯洁,沈志	a,Yan Yong,Gao Wenjie,Sh	中国食品卫生杂志	hinese Food Hygiene Journ	22	5	438-439	2010	-
2721	产加工过程中沙门菌的	during the production and	文,孙裴,魏建忠,王桂军,	Sun Pei,Wei Jianzhong,War	动物医学进展	vances in Veterinary Medic	31	z1	225-227	2010	sn.1007-5038.2010.z1.059
2722	伤寒沙门菌100株药敏结	A100Analysis of strain drug	张日妹,彭常军.	Zhang Rimei,Peng Changjun	临床合理用药杂志	l of Rational Clinical Use of	3	13	22-23	2010	sn.1674-3296.2010.13.017
2723	0-2009年食源性致病菌污	annual foodborne pathog	军,刘桂华,孔祥云,黄鑫,	uihua,Kong Xiangyun,Huar	实用预防医学	ractical preventive medicin	17	10	2001-2003	2010	sn.1006-3110.2010.10.028
2724	生鸡肉肠炎沙门氏菌的污	almonella Enteritidis in ra	祥,高继业,唐妤,丁孟建,朱	Jiye,Tang Yu,Ding Mengjia	食品科学	food science	31	15	208-211	2010	-
2725	～2008年细菌性食物中毒	nalysis of annual bacterial	王清,张勇.	Wang Qing,Zhang Yong.	现代预防医学	modern preventive medicin	37	5	921-923	2010	-
2726	登封市伤寒副伤寒疫情控	typhoid and paratyphoid	德祥,李洪民,张胜勇,白建	Li Hongmin,Zhang Shengyo	地方病通报	endemic disease notificatio	25	5	67,69	2010	-
2727	区2008年食品细菌性污	Annual food bacterial conta	金晓霞,费素定,裘立晓.	Xiaoxia,Fei Suding,Qiu Lixi	中国公共卫生管理	ese public health managen	26	4	362-363	2010	-
2728	区饮食和服务行业从业人	employees in catering and	叶玉龙,钟海明,乔国良.	ng,Zhong Haiming,Qiao Gu	职业与健康	Occupation and health	26	23	2793-2794	2010	-
2729	副伤寒沙门菌基因分型与	drug resistance of Salmon	光,章丹阳,沈玄艺,石优章,	ng Danyang,Shen Xuanyi,Sh	中国卫生检验杂志	ese Journal of Health Inspe	20	12	3145-3151	2010	-
2730	年黑龙江省食品中沙门菌	is of Salmonella Contamin	成玉,遇晓杰,谢萍会,郑晓	,Meet Xiaojie,Xie Pinghui,Z	中国卫生检验杂志	ese Journal of Health Inspe	20	7	1730-1732	2010	-
2731	猪场健康猪沙门菌带菌情	ella carriage in healthy pigs	玮,魏建忠,詹松鹤,谢倩,李	ei Jianzhong,Zhan Songhe,	中国人兽共患病学报	Chinese Journal of Zoonose	26	9	888-890	2010	sn.1002-2694.2010.09.026
2732	毒中三种食源性致病菌的	e foodborne pathogenic ba	刘军,王勤,王延东,袁新华	ang Qin,Wang Yandong,Yu	中国卫生检验杂志	ese Journal of Health Inspe	20	10	2609-2610	2010	-
2733	感染性腹泻97例病原学	rrhea97Analysis of etiolog	赵茂茂,蒲增惠,于晓.	o Maomao,Pu Zenghui,Yu X	中国医师杂志	Chinese Physician Journal	12	6	831-832	2010	sn.1008-1372.2010.06.044
2734	染性腹泻1 080例病原体检	children1 080Case pathog	君,霍晶,魏佳琦,陈玉梅,陈	o Jing,Wei Jiaqi,Chen Yume	中国误诊学杂志	inese Journal of Misdiagno	10	4	880-880	2010	-
2735	品中沙门菌污染状况及耐	nination status and drug re	明华,徐佩华,徐静妤,倪春	ua,Xu Peihua,Xu Jingyu,Ni	中国卫生检验杂志	ese Journal of Health Inspe	20	12	3425-3427	2010	-
2736	部分零售畜禽肉沙门氏菌	genotypes in some retail l	,席美丽,崔生辉,申进玲,	Cui Shenghui,Shen Jinling,Z	微生物学报	Acta Microbiologica Sinica	50	5	654-660	2010	-

2737	伤寒沙门菌食物中毒的调	of food poisoning caused b	舒开奎.	Shu Kaikui.	中国药业	hina Pharmaceutical Indust	19	12	6-6	2010	sn.1006-4931.2010.12.005
2738	门氏菌属食物中毒的流行病	ysis of food poisoning caus	刘英民.	Liu Yingmin.	临床合理用药杂志	l of Rational Clinical Use of	3	15	28	2010	sn.1674-3296.2010.15.020
2739	市市场销售食品致病菌污	eria contamination of food	徐岚,刘丽萍.	Xu Lan,Liu Liping.	预防医学论坛	Preventive Medicine Forum	16	5	432-434	2010	-
2740	性腹泻细菌病原学监测结果	tiology surveillance results	束理,刘栓奎,吴静怡,李明,王	gli,Liu Shunkui,Wu Jingyi,Li	实用预防医学	ractical preventive medicin	17	12	2389-2390	2010	sn.1006-3110.2010.12.016
2741	脊液中分离出埃森沙门菌	of Salmonella Essen from	玲玲,王慧燕,杨锦红,杨海	Vang Huiyan,Yang Jinhong,	检验医学	laboratory medicine	25	2	154-155	2010	sn.1673-8640.2010.02.024
2742	伤寒沙门菌感染24例临床分	rium infectiontwenty fourC	何聪.	He Cong.	内蒙古中医药	ngolia Traditional Chinese	29	21	16-17	2010	sn.1006-0979.2010.21.014
2743	马盖利沙门菌引起的食物中	se of food poisoning cause	郭皓,陈松.	Guo Hao,Chen Song.	预防医学情报杂志	of Preventive Medicine Info	26	3	230-230	2010	-
2744	水污染引发学生腹泻流行	mong students caused by w	张云霞,田力,李婷婷.	ang Yunxia,Tian Li,Li Tingti	中国医药指南	Chinese Medicine Guide	8	3	75-76	2010	sn.1671-8194.2010.03.052
2745	010年暑期食品微生物污染	icrobial contamination res	刘兰吉,袁玉荣,戴静林.	Lanji,Yuan Yurong,Dai Jing	中国卫生检验杂志	ese Journal of Health Inspe	20	11	2958-2960	2010	-
2746	9年梧州市细菌性食物中毒	Analysis of Bacterial Food	光,梁炳健,冼桂江,林宇,盘	ang Bingjian,Xian Guijiang,L	应用预防医学	applied preventive medicin	16	4	218-220	2010	n.1673-758X.2010.04.012
2747	鼠伤寒沙门菌感染26例临床	ium infection in children26	宋淑萍,李润霞.	Song Shuping,Li Runxia.	临床荟萃	clinical collection	25	19	1708-1709	2010	-
2748	地区食物中毒中检出肯塔基	y detected in food poisoni	王清,代彦,张勇.	ang Qing,Dai Yan,Zhang Yo	现代预防医学	modern preventive medicin	37	20	3914-3916	2010	-
2749	东省流通领域食品沙门氏	ntamination of food in circ	玉贞,邵坤,关冰,胡彬,董海	Shao Kun,Guan Bing,Hu Bin	中国公共卫生管理	ese public health managem	26	2	163-165	2010	-
2750	副伤寒沙门菌脉冲场凝胶	horesis and multi-locus sec	军,周海健,崔志刚,杜鹏程,阚	aijian,Cui Zhigang,Du Peng	中华预防医学杂志	se Journal of Preventive Me	44	9	810-814	2010	sn.0253-9624.2010.09.010
2751	析华东地区动物源产品中肠	monella enterica isolates f	强,陈扬,薛峰,徐飞,蒋原,陆	Yang,Xue Feng,Xu Fei,Jian	大学学报（农业与生命科学	University (Agriculture and	31	3	13-16	2010	-
2752	年小儿腹泻病的病原体调查	alysis of pathogens of diar	朱利华,陈群英,黄明海.	ua,Chen Qunying,Huang M	中华医院感染学杂志	ournal of Hospital Infectiou	20	22	3606-3607	2010	-
2753	丹沙门菌食物中毒实验室	ion and analysis of Salmon	李岳良.	Li Yueliang.	中国卫生检验杂志	ese Journal of Health Inspe	20	4	908945	2010	-
2754	检查中沙门菌分离方法的	onella isolation methods du	文良,杨兰萍,胡雪明,许学	ng,Yang Lanping,Hu Xuemin	环境与职业医学	mental and Occupational M	27	12	739-741,744	2010	-
2755	鸡制品中食源性致病菌污染	pathogenic contamination	王华洪,吴日明.	Wang Huahong,Wu Riming	实用预防医学	ractical preventive medicin	17	7	1314-1315	2010	sn.1006-3110.2010.07.024
2756	沙门菌食物中毒的实验室	ction of a case of salmonell	陈松,郭皓.	Chen Song,Guo Hao.	预防医学情报杂志	of Preventive Medicine Info	26	3	222-223	2010	-
2757	沙门菌主动监测网建立及	Salmonella Active Surveill	振柱,黄兆勇,黄彦,诸葛石	ang Zhaoyong,Huang Yan,Z	中国卫生检验杂志	ese Journal of Health Inspe	20	7	1765-1767	2010	-
2758	门氏菌的分离及其ERIC-P	rom seafood and itsERIC-	佳,邱玉玉,张海涛,于广福,陈	hang Haitao,Yu Guangfu,Ch	中国病原生物学杂志	ese Journal of Pathogen Bio	5	3	164-167	2010	-
2759	6年血培养病原菌分布及	nic bacteria and changes in	以军,徐瑞龙,应华永,邵丽	Ruilong,Ying Huayong,Sha	中华医院感染学杂志	ournal of Hospital Infectiou	20	14	2137-2140	2010	-
2760	副伤寒PulseNet数据库的	APulseNetDatabase establi	王冰,林一曼,程锦泉,马汉	g,Lin Yiman,Cheng Jinquan	中国热带医学	Chinese tropical medicine	10	8	936-938	2010	-
2761	芝地区鸡沙门氏菌的分离	tion of Salmonella chicken	海潮,次仁多吉,曾江勇,拉巴	hao,Tsering Dorje,Zeng Jian	浙江畜牧兽医	mal Husbandry and Veterin	35	4	4-6	2010	sn.1005-7307.2010.04.002
2762	州地区食源性致病菌污染	odborne pathogenic bacte	春,李毅,陈慧燕,章乐怡,洪	Chen Huiyan,Zhang Leyi,Ho	中国食品卫生杂志	hinese Food Hygiene Journ	22	2	168-170	2010	-
2763	感染情况及药敏动态观察	nalysis of Salmonella infec	陈卫群.	Chen Weiqun.	检验医学与临床	oratory medicine and clini	7	14	1486-1488	2010	sn.1672-9455.2010.14.040
2764	脑膜炎病原菌的分布和耐	tance of pathogenic bacter	蓉,武素梅,李连青,朱镭,崔	u Sumei,Li Lianqing,Zhu Lei	实用检验医师杂志	of Practical Laboratory Phy	2	4	205-208	2010	sn.1674-7151.2010.04.004
2765	菌新近流行株的分离鉴定	new epidemic strains of Sa	胡,吴庭才,张春杰,李银聚,王	Ngo Dinh Chai,Zhang Chun	中国家禽	Chinese poultry	32	17	31-35	2010	-
2766	杨杆菌和沙门菌的分离鉴定	ility test of Escherichia coli	光伟,杨晓伟,郑琳红,张成	ang Xiaowei,Zheng Linhong	黑龙江畜牧兽医（下半月）	dry and Veterinary Medicin	-	8	73-74	2010	-
2767	致沙门菌食物中毒的微生物	almonella food poisoning	雅琴,叶菊莲,韦俊超,李月	qin,Ye Julian,Wei Junchao,L	中国卫生检验杂志	ese Journal of Health Inspe	20	10	2555-2557	2010	-
2768	道门诊腹泻患者肛拭病原	s in anal swabs of patients	绪红霞,刘芸,骆玲飞,陈国强	xia,Liu Yun,Luo Lingfei,Che	中国实用医药	Chinese Practical Medicine	5	36	141-142	2010	sn.1673-7555.2010.36.111
2769	基地斑头雁沙门菌的耐药性	of Salmonella in bar-head	朱丽娜,罗薇,刘内生,刘群	na,Luo Wei,Liu Neisheng,Li	中国家禽	Chinese poultry	32	15	65-67	2010	-
2770	人口食品大肠菌群和致病	Food Coliform and Pathog	光,屈飞飞,孙晓华,杨扬,刘	u Feifei,Sun Xiaohua,Yang Y	现代预防医学	modern preventive medicin	37	3	564-566	2010	-
2771	株的分布及金黄色葡萄球菌	d culture and analysis of dr	石祖亮,简翠.	Shi Zuliang,Jian Cui.	热带医学杂志	journal of tropical medicine	10	2	177-180	2010	-
2772	~2008年食源性沙门菌耐药	n annual changes in drug r	秀丽,廖兴广,银恭举,胡巅,张	guang,Yin Gongju,Hu Dian	现代预防医学	modern preventive medicin	37	18	3542-3543,3547	2010	-
2773	川地区鸡源沙门菌耐药性	of Salmonella originating f	红,林居纯,陈雅莉,马驰,张	Lin Juchun,Chen Yali,Ma Ch	中国家禽	Chinese poultry	32	4	55-56	2010	-

2774	伤寒沙门菌流行特征及分子	s and molecular typing of S	,肖文佳,陈敏,冉陆,刁保卫	enjia,Chen Min,Ran Lu,Diao	上海预防医学	hanghai Preventive Medicin	22	2	57-61,封3	2010	sn.1004-9231.2010.02.001
2775	食品中食源性致病菌污染	foodborne pathogenic ba	询,聂菱,邓淑芬,胡凰仙,李	e Ling,Deng Shufen,Hu Hua	中国卫生检验杂志	ese Journal of Health Inspe	20	7	1778-1779	2010	-
2776	副伤寒沙门菌对抗生素的	tivity of Salmonella Paratyp	王玉春,杨峻,高玲.	ng Yuchun,Yang Jun,Gao Li	应用预防医学	pplied preventive medicin	16	2	108-109	2010	n.1673-758X.2010.02.021
2777	由沙门菌引起的食物中毒	se of food poisoning cause	王正兴,代传焕,孟海群.	ngxing,Dai Chuanhuan,Me	职业与健康	Occupation and health	26	5	517-518	2010	-
2778	株沙门菌的菌型分布及耐	l type distribution and dru	林一曼,扈庆华,石晓路,李迎	han,Hu Qinghua,Shi Xiaolu,	实用预防医学	ractical preventive medicin	17	8	1644-1646	2010	sn.1006-3110.2010.08.073
2779	008年甲型副伤寒沙门菌	on the molecular typing of	伟,潘劲草,鲍千红,张蔚,孟	cao,Bao Qianhong,Zhang W	中国预防医学杂志	se Journal of Preventive Me	11	9	926-929	2010	-
2780	边贫山区儿童集体食物中	among children in poor mo	黄泽萍.	Huang Zeping.	中国医药指南	Chinese Medicine Guide	8	32	234-235	2010	sn.1671-8194.2010.32.181
2781	物产品沙门氏菌污染情况	Salmonella contamination	德胜,张险朋,黄炳炽,洪伟	hang Xianpeng,Huang Bing	中国畜牧兽医	mal husbandry and veterina	37	10	202-203	2010	-
2782	炎沙门氏菌引起食物中毒	e of food poisoning caused	刘惠虹.	Liu Huihong.	河南预防医学杂志	n Journal of Preventive Me	21	3	封3	2010	-
2783	2004-2008年传染病疫情	004-2008Annual trends in i	曙光,牟霞,付青梅,王海琴,	Mou Xia,Fu Qingmei,Wang	解放军预防医学杂志	tive Medicine of the People	28	5	353-354	2010	-
2784	009年食源性致病菌污染	survey on foodborne patho	春东,卢俊荣,庞振清,祁业	ong,Lu Junrong,Pang Zhenq	医学动物防制	medical animal control	26	10	940-941	2010	sn.1003-6245.2010.10.024
2785	市江北区婴幼儿腹泻病原	rrhea in infants and young	徐景野,裘立晓,陈美芬,金晓	e,Qiu Lixiao,Chen Meifen,J	中国卫生检验杂志	ese Journal of Health Inspe	20	1	181-182,195	2010	-
2786	中毒的剩余食物中检出伤寒	phi detected in food poiso	刘惠虹.	Liu Huihong.	中国卫生检验杂志	ese Journal of Health Inspe	20	4	918	2010	-
2787	-2008年临床分离细菌种群	bution and changes of clini	,储从家,管新龙,李杰芬,杨	u Congjia,Guan Xinlong,Li Ji	中国感染控制杂志	ese Journal of Infection Co	9	3	196-199	2010	sn.1671-9638.2010.03.015
2788	门菌中沙门菌基因组岛1的	species carried by healthy	徐保红,闫鹤,赵红丽,李继	Baohong,Yan He,Zhao Hong	分子诊断与治疗杂志	Molecular Diagnosis and Th	2	4	231-236	2010	sn.1674-6929.2010.04.004
2789	年江苏省食源性致病菌监	is of foodborne pathogeni	,乔昕,袁宝君,戴月,符晓梅	Xin,Yuan Baojun,Dai Yue,Fu	中国卫生检验杂志	ese Journal of Health Inspe	20	8	2011-2013	2010	-
2790	市餐饮业凉菜间卫生状况	s of cold dish rooms in the	王亚丽.	Wang Yali.	现代预防医学	modern preventive medicin	37	1	173-174	2010	-
2791	北京市大兴区食源性致病	borne pathogenic bacteria	王桂侠,温静.	Wang Guixia,Gentle and quie	首都公共卫生	capital public health	4	3	130-131	2010	sn.1673-7830.2010.03.012
2792	年登封市肠道传染病监测	is of intestinal infectious d	德祥,李洪民,张建萍,张翠	ng,Li Hongmin,Zhang Jianp	中国病原生物学杂志	ese Journal of Pathogen Bi	5	7	558-559	2010	-
2793	门菌血清学鉴定和药敏试	and drug susceptibility test	邓家全.	Deng Jiaquan.	中外医疗	Chinese and foreign medica	29	23	15-16	2010	sn.1674-0742.2010.23.008
2794	2006-2009年食品污染物	09Analysis of annual food	赵惠玲,孟柯,王小英,潘峰,孙	,Meng Ke,Wang Xiaoying,	华南预防医学	uth China Preventive Medic	36	5	71-73	2010	-
2795	性疾病哨点医院主动监测	lts of foodborne disease se	戴月,袁宝君.	Dai Yue,Yuan Baojun.	中国实用医药	Chinese Practical Medicine	5	1	266-267	2010	sn.1673-7555.2010.01.232
2796	沙门菌食物中毒事件调查	nalysis of a Salmonella Foo	林华,余杭民,郭大为,王玲虹	Hangmin,Guo Dawei,Wang	海峡预防医学杂志	s Journal of Preventive Me	16	1	61	2010	-
2797	腹泻病人粪便中检出里森	a Risen in the feces of patie	芦晓光,杜丽霞.	Lu Xiaoguang,Du Lixia.	中国卫生检验杂志	ese Journal of Health Inspe	20	12	3441-3442	2010	-
2798	市场鲜鸡蛋黄中沙门氏菌	onella in fresh egg yolks fro	赵从凯,王洪波,丁雪珍,房	kai,Wang Hongbo,Ding Xue	畜禽业	vestock and poultry indust	-	10	50-51	2010	sn.1008-0414.2010.10.031
2799	工厂沙门菌感染暴发危险	for Salmonella infection ou	,黄锡青,张雪宝,萧松建,陈	g Xiqing,Zhang Xuebao,Xia	实用预防医学	ractical preventive medicin	17	1	54-56	2010	sn.1006-3110.2010.01.020
2800	08年分离60株伤寒沙门菌	ated60Drug sensitivity ana	邱亚群,兰全学,石晓路,刘沫	n,Lan Quanxue,Shi Xiaolu,	实用预防医学	ractical preventive medicin	17	1	138-139	2010	sn.1006-3110.2010.01.059
2801	分地区牛奶中几种微生物	ial indicators in milk in som	李桂兰.	Li Guilan.	中国动物检疫	China Animal Quarantine	27	6	47-49	2010	n.1005-944X.2010.06.026
2802	程中沙门氏菌的监控方法	a during broiler slaughteri	广全,张昕,汪琦,张捷,畅	an,Zhang Xin,Wang Qi,Zha	肉类工业	meat industry	-	3	4-7	2010	sn.1008-5467.2010.03.002
2803	市食源性和禽源性沙门氏	borne and poultry-borne Sa	,杨保伟,廖兴广,郭晓玲,	aowei,Liao Xingguang,Guo	中国人兽共患病学报	Chinese Journal of Zoonose	26	12	1134-1136	2010	sn.1002-2694.2010.12.013
2804	列细菌性腹泻病原菌分布	nd drug resistance of path	仙,焦建中,朱平,胡利斌,张	anzhong,Zhu Ping,Hu Libin,	武警医学	Armed Police Medicine	21	6	488-490	2010	sn.1004-3594.2010.06.009
2805	6-2008年食源性致病菌监	lysis of annual foodborne p	翔,李蕾,谢明英,闫立群,郝	Lei,Xie Mingying,Yan Liqun	宁夏医学杂志	Ningxia Medical Journal	32	4	371-372	2010	sn.1001-5949.2010.04.038
2806	7年志贺菌、沙门菌菌型	of type distribution and dr	,徐佩华,陈鸣芳,吴海平,倪	eihua,Chen Mingfang,Wu H	中国卫生检验杂志	ese Journal of Health Inspe	20	2	404-407	2010	-
2807	省宁波市食源性疾病暴发	on foodborne disease outb	明,徐景野,许国章,方挺,毛	ingye,Xu Guozhang,Fang T	疾病监测	disease surveillance	25	8	623-626	2010	sn.1003-9961.2010.08.011
2808	市汤卜逊沙门菌流行特征	characteristics of Salmone	,张前龙,许学斌,王洪霞,	ianlong,Xu Xuebin,Wang H	中国人兽共患病学报	Chinese Journal of Zoonose	26	4	394-396	2010	sn.1002-2694.2010.04.024
2809	副伤寒疫情暴发的实验室	ry testing of a paratyphoid	斌,李怡,王春丽,侯敏,刘贵	Yi,Wang Chunli,Hou Min,Li	中国卫生检验杂志	ese Journal of Health Inspe	20	9	23332335	2010	-
2810	超广谱β-内酰胺酶和喹诺	pectrumβ-Lactamase and q	陈希莲,朱德全,季海生.	Xilian,Zhu Dequan,Ji Haish	中华医院感染学杂志	ournal of Hospital Infectiou	20	18	2749-2751	2010	-

2811	寒副伤寒沙门菌耐药及	typing of Salmonella typhi	莲,徐宝祥,张政,金大智,占	oxiang,Zhang Zheng,Jin Da	中国公共卫生	Chinese public health	26	3	328-330	2010	-
2812	寒沙门菌耐药性及脉冲	olecular typing of Salmon	应坚,甘莉萍,杨慧,金玉	jian,Gan Liping,Yang Hui,Ji	职业与健康	Occupation and health	26	16	1805-1808	2010	-
2813	诊断芯片的研制及在一起	ment of diagnostic chips an	承宁,叶立青,刘志明,杜	,Ye Liqing,Liu Zhiming,Du J	南方医科大学学报	al of Southern Medical Univ	30	3	417-421	2010	sn.1673-4254.2010.03.001
2814	8年荆门市食源性致病菌	ults of foodborne pathoge	,陈天林,曾祥梅,马晓丽,	n Tianlin,Zeng Xiangmei,M	职业与健康	Occupation and health	26	18	2080-2081	2010	-
2815	门氏菌引起的食物中毒	case of food poisoning ca	宋冬梅.	Song Dongmei.	中外健康文摘	hese and Foreign Health Di	7	33	24	2010	sn.1672-5085.2010.33.018
2816	品中食源性致病菌污染	tus of foodborne pathogen	涛,谢翊,陈淑玲,林秋芬,卢	Xie Yi,Chen Shuling,Lin Qiuf	中国热带医学	Chinese tropical medicine	10	6	707-708	2010	-
2817	发酵棉粕饲料喂养蛋鸡	ing hens with microbial fe	,孙国林,李爱巧,王六和,	Sun Guolin,Li Aiqiao,Wang	新疆畜牧业	Xinjiang animal husbandry	-	3	32-33	2010	sn.1003-4889.2010.03.016
2818	市售食品食源性致病菌污	thogenic bacteria contami	黄健利,钟凌,姚海燕.	g Jianli,Zhong Ling,Yao Ha	预防医学论坛	Preventive Medicine Forum	16	9	832-834	2010	-
2819	医疗机构室内空气和物	rface disinfection monitor	波,张化江,孙琦,丛庆美,肖	Huajiang,Sun Qi,Cong Qing	预防医学论坛	Preventive Medicine Forum	16	12	1145-1146	2010	-
2820	群沙门菌暴发菌株鉴别	ifferential diagnosis of Sal	王红霞,冉陆,金汇明,陈敏,	gxia,Ran Lu,Jin Huiming,Ch	中国卫生检验杂志	ese Journal of Health Inspe	20	12	3077-3080	2010	-
2821	成都市全球沙门菌监测	ts of the Chengdu Global S	碧容,陈竹,朱帆,陈仕晓,	ng,Chen Zhu,Zhu Fan,Chen	临床荟萃	clinical collection	25	10	863-864	2010	-
2822	圳医院急性感染性腹泻	ce results of acute infectio	王德,苏琪,杨虹,王丽.	g De,Suki,Yang Hong,Wan	热带医学杂志	journal of tropical medicine	10	1	64-65,68	2010	-
2823	南京地区食源性致病菌污	e pathogenic bacteria cont	金萍,江晓.	Jin Ping,Jiang Xiao.	中国医学创新	Chinese medical innovation	7	18	152-153	2010	sn.1674-4985.2010.18.094
2824	鸭沙门菌引起食物中毒	se of food poisoning cause	马吉辉,陈卫武.	Ma Jihui,Chen Weiwu.	中国当代医药	inese contemporary medic	17	23	117122	2010	sn.1674-4721.2010.23.074
2825	都柏林沙门菌引起的食	poisoning caused by Salm	林忠兰,廖慧.	Lin Zhonglan,Liao Hui.	海峡预防医学杂志	s Journal of Preventive Me	16	4	51-52	2010	-
2826	07-2009年重庆市沙门菌	Salmonella surveillance in	鸿燕,李勤,肖邦忠,李志锋,	n,Li Qin,Chopin Chung,Li Z	热带医学杂志	journal of tropical medicine	10	10	1230-1233	2010	-
2827	菌及沙门氏菌污染水源	y pathogenic Escherichia	张克志,赵严,陈宝杰.	g Kezhi,Zhao Yan,Chen Ba	中国初级卫生保健	primary health care in china	24	5	66	2010	n.1001-568X.2010.05.039
2828	鸡沙门氏菌病流行病学调	and analysis of chicken sal	林君,张国权,王国艳,张李	Zhang Guoquan,Wang Guo	山西农业科学	Shanxi Agricultural Sciences	38	9	58-62	2010	ssn.1002-2481.2010.09.18
2829	沙门菌引起食物中毒的	r food poisoning caused by	段朝标,李淑芳,李刚文.	Chaobiao,Li Shufang,Li Gan	中国当代医药	inese contemporary medic	17	27	129-130	2010	sn.1674-4721.2010.27.082
2830	单位食源性致病菌污染	ogenic bacteria contamina	以玥,王蒋丽,曾强,王伟,高	Jiangli,Zeng Qiang,Wang W	中国卫生检验杂志	ese Journal of Health Inspe	20	12	3418-3419	2010	-
2831	肉制食品中食源性致病菌	ria contamination in meat	柱,李雪梅,刘长宏,岳永杰	Xuemei,Liu Changhong,Yue	中国卫生检验杂志	ese Journal of Health Inspe	20	9	2278-2280	2010	-
2832	鸡沙门氏分离鉴定及防治	fication and prevention of	刘亚伟,冯运巩.	Liu Yawei,Feng Yungong.	今日畜牧兽医	ivestock Veterinarian Toda	-	3	40	2010	-
2833	某校学生食物中毒流行病	poisoning among student	王恒昌.	Wang Hengchang.	中国自然医学杂志	ese Journal of Natural Med	12	4	316	2010	-
2834	办宴席引发食物中毒事件	oisoning incident caused b	郑国旗,郭超,陈光旭.	Guoqi,Guo Chao,Chen Gua	中国食品卫生杂志	hinese Food Hygiene Journ	22	2	171-172	2010	-
2835	童血培养分离菌的变迁及	analysis of bacterial isolat	鞠樑.	Ju Liang.	吉林医学	Jilin Medicine	31	35	6506-6508	2010	sn.1004-0412.2010.35.058
2836	副伤寒临床与沙门菌药敏	paratyphoid A and drug su	陈忠明,冯舜.	Chen Zhongming,Feng Shun	中国现代药物应用	dern drug application in Ch	4	10	122-123	2010	sn.1673-9523.2010.10.111
2837	食源性沙门氏菌耐药及相	ted genes of foodborne Sa	进玲,席美丽,只帅,崔生辉,	eili,Only handsome,Cui She	微生物学报	Acta Microbiologica Sinica	50	6	788-796	2010	-
2838	产品的食源性致病微生物	ogenic microorganisms in	晓明,张海妍,张松山,孙宝	ang Haiyan,Zhang Songsha	肉类研究	meat research	-	7	50-53	2010	sn.1001-8123.2010.07.015
2839	沙门菌的分离鉴定及耐药	d drug resistance analysis	刘茜.	Liu Qian.	生物技术通报	biotechnology bulletin	-	5	145-148	2010	-
2840	省余姚市感染性腹泻沙门	lance results of infectious	张怡明,张建群,罗学辉.	Yiming,Zhang Jianqun,Luo	疾病监测	disease surveillance	25	11	894-896,899	2010	sn.1003-9961.2010.11.015
2841	儿园食物中毒爆发的调查	alysis of food poisoning ou	王玲.	Wang Ling.	中国校医	Chinese school doctor	24	8	570-571	2010	-
2842	患者血液培养病原体和	pathogens and drug suscep	唐秀文,张晶.	Tang Xiuwen,Zhang Jing.	广西医学	Guangxi Medicine	-	7	782-785	2010	sn.0253-4304.2010.07.009
2843	鸡鸡白痢沙门菌的分离鉴	tion of Salmonella pulloru	北宇,曹峰子,陈小玲,张培	ao Fengzi,Chen Xiaoling,Zh	动物医学进展	vances in Veterinary Medic	31	12	153-156	2010	sn.1007-5038.2010.12.037
2844	2004-2009年细菌性食物中	009Annual analysis of bact	周惠芳,朱海燕.	Zhou Huifang,Zhu Haiyan.	中国卫生检验杂志	ese Journal of Health Inspe	20	9	2274-2275	2010	-
2845	海洋贝类微生物污染状况	l contamination of marine	青云,潘长旺,梁韶晖,黄	Changwang,Liang Shaohu	南方医科大学学报	al of Southern Medical Univ	30	7	1624-1625	2010	sn.1673-4254.2010.07.037
2846	-2009年食品卫生微生物	lysis of annual food hygien	王雅琴,张仕华.	Wang Yaqin,Zhang Shihua.	中国卫生检验杂志	ese Journal of Health Inspe	20	6	1496-1497	2010	-
2847	食源性致病菌污染状况的	mination status of foodbor	曹斌,申峻松.	Cao Bin,Shen Junsong.	泰州职业技术学院学报	aizhou Vocational and Tech	10	1	57-58	2010	sn.1671-0142.2010.01.022

2848	08年临床食源性疾病监测	of annual clinical foodbor	李香亭,陈道湧.	Li Xiangting,Chen Daoyong	现代预防医学	nodern preventive medicin	37	5	936-938	2010	-
2849	寒沙门菌引起食物中毒的	a case of food poisoning ca	赵朝珍,张宗辉.	ao Chaozhen,Zhang Zongh	国际流行病学传染病学杂	rnal of Epidemiology and In	37	4	287	2010	sn.1673-4149.2010.04.024
2850	类肠道寄生虫及沙门菌感	sites and salmonella infecti	朱文刚.	Zhu Wengang.	中国媒介生物学及控制杂	ournal of Vector Biology an	21	3	187	2010	-
2851	登沙门菌引起的一起食源	s outbreak caused by Salm	华,杜春华,潘海燕,张玫,黄	unhua,Pan Haiyan,Zhang M	中国卫生检验杂志	ese Journal of Health Inspe	20	4	912-913	2010	-
2852	伤寒沙门菌脉冲电场凝胶	horesis typing analysis of Sa	海健,田克诚,游旅,韦小俞	,Tian Kecheng,Travel,Wei	首都医科大学学报	nal of Capital Medical Unive	31	3	388-392	2010	sn.1006-7795.2010.03.021
2853	株志贺菌沙门菌检测结果	ction results of Shigella str	王建兰,朱海燕.	Wang Jianlan,Zhu Haiyan.	中国卫生检验杂志	ese Journal of Health Inspe	20	11	2933-2934	2010	-
2854	肠道致泻菌的调查与药敏	ivity analysis of intestinal d	张玉霞,余淑华.	Zhang Yuxia,Yu Shuhua.	中国当代医药	inese contemporary medic	17	27	134-135	2010	sn.1674-4721.2010.27.086
2855	细菌性食物中毒的病原学	lysis of a case of bacterial f	徐妍,马智龙,蔡震,杨娟.	,Ma Zhilong,Cai Zhen,Yang	现代预防医学	nodern preventive medicin	37	24	4661-4662	2010	-
2856	沙门氏菌污染食品引起的	oisoning caused by Salmon	齐洪武,张利.	Qi Hongwu,Zhang Li.	中国热带医学	Chinese tropical medicine	10	8	1038-1038	2010	-
2857	市大兴区食品中食源性致	oodborne pathogenic bact	洁,王秀臣,王小英,潘峰,孟	Xiuchen,Wang Xiaoying,Pa	中国食品卫生杂志	hinese Food Hygiene Journ	22	5	435-437	2010	-
2858	09年濮阳市肠道致泻菌耐	resistance status of intesti	孙迎军.	Sun Yingjun.	中国实用医刊	ese Journal of Practical Med	37	23	84-85	2010	sn.1674-4756.2010.23.046
2859	8年徐州市食源性致病菌	nalysis of foodborne patho	升浩,薛诚,吴玲,葛小伍,肖	,Xue Cheng,Wu Ling,Ge Xi	中国校医	Chinese school doctor	24	7	483-484	2010	-
2860	从业人员肠道沙门氏菌携	Salmonella among caterin	海平,张宏萍,张伟,赵哲,褚	,Zhang Hongping,Zhang W	安徽预防医学杂志	i Journal of Preventive Med	16	2	91-92,112	2010	-
2861	门菌引发食物中毒的实验	food poisoning caused by	朱晶.	Zhu Jing.	现代医药卫生	nodern medicine and healt	26	22	3510-3511	2010	-
2862	空气细菌数量与乳房炎发生	nber of air bacteria and the	,马飞,谈亦奇,许威,孙晴,祁	Fei,Tan Yiqi,Xu Wei,Sun Qin	畜牧与兽医	Husbandry and Veterinary M	42	12	87-91	2010	-
2863	2007-2009年食物中毒事件	-2009Analysis of annual fo	杨丽宏.	Yang Lihong.	实用预防医学	ractical preventive medicin	17	3	507-508	2010	sn.1006-3110.2010.03.035
2864	化养猪场环境细菌的调查与	s of environmental bacteri	高颖,杜海燕,王开功,周碧	u Haiyan,Wang Kaigong,Zh	中国畜牧兽医	mal husbandry and veterina	37	8	199-203	2010	-
2865	寒沙门菌引起食物中毒流行	a case of food poisoning ca	曹妍,李丽.	Cao Yan,Li Li.	预防医学情报杂志	of Preventive Medicine Info	26	5	413-414	2010	-
2866	山夫登堡沙门菌流行菌株	cal epidemic strains of Saln	红,盛跃颖,冉陆,刁保卫,崔	Yueying,Ran Lu,Diao Baob	检验医学	laboratory medicine	25	10	797-800	2010	sn.1673-8640.2010.10.016
2867	门氏菌引起的食源性疾病的	nt status of a foodborne il	张明霞.	Zhang Mingxia.	中外健康文摘	hese and Foreign Health Di	7	3	26-27	2010	sn.1672-5085.2010.03.017
2868	用皮蛋引起的食物中毒的病	se of food poisoning cause	周江,向晓霞,张学健.	iang,Xiang Xiaoxia,Zhang X	中国卫生检验杂志	ese Journal of Health Inspe	20	2	422-422	2010	-
2869	利丹沙门菌引起的食物中毒	ry test report on food pois	雷兰芝,马名驹,邓勇.	Lanzhi,Ma Mingju,Deng Yo	四川医学	Sichuan Medicine	31	5	672	2010	sn.1004-0501.2010.05.063
2870	起沙门氏菌食物中毒调查分	nalysis of a case of salmon	王振海.	Wang Zhenhai.	疾病监测与控制	ease surveillance and cont	4	1	27-27	2010	-
2871	型副伤寒沙门菌引起的食	oisoning caused by Salmor	王小红,龚成林.	ang Xiaohong,Gong Chengl	中国卫生检验杂志	ese Journal of Health Inspe	20	6	1536	2010	-
2872	市售鸭肝主要微生物污染	ntamination status of duck	雁,谢英,刘华,朱良强,祁克	ie Ying,Liu Hua,Zhu Liangq	黑龙江农业科学	longjiang Agricultural Scien	-	4	120-121	2010	sn.1002-2767.2010.04.040
2873	沙门菌引起食物中毒的调查	of a case of food poisonin	许华,廖周勇.	Xu Hua,Liao Zhouyong.	现代预防医学	nodern preventive medicin	37	20	3944-3944	2010	-
2874	法确认方案在从业人员肠	nfirmation scheme for the	曾斤日,刘爱民,彭守柏.	g Jinri,Liu Aimin,Peng Shou	中国卫生检验杂志	ese Journal of Health Inspe	20	7	1711-1713	2010	-
2875	鼠伤寒沙门氏菌引起的食物	isoning caused by Salmone	王铁锚.	Wang tiemao.	中国保健	China health care	-	10	-	2010	-
2876	炎主要病原菌的分离鉴定	ermination of the main pat	抗,张为民,张彦明,王晶钰,	g Weimin,Zhang Yanming,W	西北农业学报	rnal of Northwest Agricult	19	7	30-34	2010	sn.1004-1389.2010.07.007
2877	学校感染性腹泻暴发原因	ses of an outbreak of infec	杨忠贤,张兵礼,李晓.	Zhongxian,Zhang Bingli,Li	河南预防医学杂志	Journal of Preventive Me	21	5	349-350	2010	-
2878	沙门氏菌食物中毒的病原	c bacteria in a case of Salm	阳艳.	Yang Yan.	医学信息（医药版）	nformation (Pharmaceutica	-	2	-	2010	-
2879	沙门氏菌引起食物中毒的	s of food poisoning caused	吴红玲,马红梅.	Wu Hongling,Ma Hongmei.	宁夏医学杂志	Ningxia Medical Journal	32	12	1256-1257	2010	sn.1001-5949.2010.12.116
2880	省太原市古交地区感染性	of infectious diarrhea in Gu	郝拉维,张美英.	Hao Lawei,Zhang Meiying.	中国医药指南	Chinese Medicine Guide	8	14	244-245	2010	sn.1671-8194.2010.14.157
2881	儿童大便沙门菌培养及药敏	analysis of Salmonella in s	林伟雄,丘芸,王宗杰.	Veixiong,Qiu Yun,Wang Zo	中国社区医师（医学专业）	munity physician (medical	12	19	157	2010	sn.1007-614x.2010.19.168
2882	中沙门菌和金黄色葡萄球菌	la and Staphylococcus aure	,席美丽,申进玲,石颖,只帅	Shen Jinling,Shi Ying,Only h	中华预防医学杂志	se Journal of Preventive Me	44	8	757-759	2010	sn.0253-9624.2010.08.019
2883	市大兴区市售食品食源性致	ne pathogenic bacteria in c	王小英,赵惠玲,王宏宇.	aoying,Zhao Huiling,Wang	预防医学论坛	Preventive Medicine Forum	16	9	828-830	2010	-
2884	浦区饮服从业人员沙门菌	a among catering industry	华哲云,王宇,崔冉,张前龙	un,Wang Yu,Cui Ran,Zhang	中国公共卫生	Chinese public health	26	3	373-374	2010	-

2885	鸭屠宰加工生产链中沙	nation in the meat duck sla	唐善虎,岑璐伽,李雪,陈诺	hanhu,Cen Lujia,Li Xue,Ch	食品科学	food science	31	22	326-331	2010	-
2886	寒沙门菌脉冲场凝胶电	s genotyping analysis of Sa	钱慧敏,周璐,谈忠鸣,鲍倡	imin,Zhou Lu,Tan Zhongmi	江苏卫生保健	Jiangsu Health Care	12	6	11-12	2010	sn.1008-7338.2010.06.006
2887	08年～2009年食源性致病	2009Annual Foodborne Pa	,戴月,符晓梅,乔昕,沈贇,	e,Fu Xiaomei,Qiao Xin,Shen	江苏预防医学	iangsu Preventive Medicin	21	4	1-3	2010	sn.1006-9070.2010.04.001
2888	07-2009年食源性致病菌	Annual foodborne pathoge	吴春敏,陈萍,周颖.	Chunmin,Chen Ping,Zhou	中国自然医学杂志	ese Journal of Natural Med	12	3	164-166	2010	-
2889	门菌感染引起的慢性心包	sion caused by Salmonella	庄熙晶,梁永才.	Zhuang Xijing,Liang Yongcai	医学信息（中旬刊）	cal information (mid-term	5	2	240-241	2010	1006-1959-C.2010.02.067
2890	液中分离出希林登沙门菌	nella Hillingdon isolated fr	,陈亚宝,沙鸭云,陆桃红,	abao,Merganser cloud,Lu	中华临床感染病杂志	ournal of Clinical Infectious	3	4	248	2010	sn.1674-2397.2010.04.019
2891	饲料中沙门氏菌的检测与	Analysis of Salmonella in	秋华,陈建辉,罗土炎,姚清	,Chen Jianhui,Luo Tuyan,Y	中国农村小康科技	ina Rural Well-off Technol	-	12	79-81	2010	sn.1007-7774.2010.12.026
2892	的检测及病原菌的分离	lation, identification and	拉达来,刘晓松,朱宪光,	ai,Liu Xiaosong,Zhu Xiangu	畜牧与饲料科学	Livestock and feed science	-	6	235-237	2010	sn.1672-5190.2010.06.083
2893	肉产品中沙门氏菌污染状	ella contamination in com	郑增忍,王玉东,龚振华,黄	gren,Wang Yudong,Gong Z	中国动物检疫	China Animal Quarantine	27	7	50-50	2010	n.1005-944X.2010.07.025
2894	地区沙门菌的分布及耐药	esistance analysis of Salmo	黄金娥,张萍,蒋慧.	ang Jin'e,Zhang Ping,Jiang	实用预防医学	ractical preventive medicin	17	7	1416-1417	2010	sn.1006-3110.2010.07.074
2895	染患者246例血培养阳性	ients246Analysis of positiv	关兴丽.	Guan Xingli.	中国医药指南	Chinese Medicine Guide	8	14	206-208	2010	sn.1671-8194.2010.14.128
2896	寒高发地区以人口为基数	areas with high incidence	壮,李永红,唐昌新,姚催华	i Yonghong,Tang Changxin	应用预防医学	pplied preventive medicin	16	3	133-135	2010	n.1673-758X.2010.03.002
2897	某高校水污染事件调查	water pollution incident i	刘建如,杨光耀,张旭辉.	nru,Yang Guangyao,Zhang	实用预防医学	ractical preventive medicin	17	6	1133-1134	2010	sn.1006-3110.2010.06.039
2898	儿童感染性腹泻病原学和	esistance of infectious diar	,甄景慧,刘锡清,许宏,宋	n Jinghui,Liu Xiqing,Xu Hon	临床儿科杂志	ournal of Clinical Pediatric	28	6	535-538	2010	sn.1000-3606.2010.06.008
2899	食品沙门氏菌分离与菌	bacterial film detection fr	,赵金龙,刘昕煜,朱超,杜	ao Jinlong,Liu Xinyu,Zhu C	食品研究与开发	od research and developm	31	12	136-139	2010	sn.1005-6521.2010.12.039
2900	247例甲型副伤寒耐药分	of drug resistance in cases	胡筱卿.	Hu Xiaoqing.	医药与保健（下旬版）	ine and Health Care (late e	-	4	-	2010	-
2901	江苏省食品中食源性致病	of foodborne pathogenic	,乔昕,袁宝君,戴月,符晓	Xin,Yuan Baojun,Dai Yue,F	中国食品卫生杂志	hinese Food Hygiene Jour	22	5	431-434	2010	-
2902	髓培养伤寒沙门菌1例报	culture Salmonella typhi1E	郭海波,陶丽旸,文雪	ibo,Tao Liyang,Wen Xue,F	检验医学与临床	oratory medicine and clin	7	13	1401-1402	2010	sn.1672-9455.2010.13.072
2903	株腹泻病原菌分布及耐药	and drug resistance of dia	路晓梅,高进霞.	Lu Xiaomei,Gao Jinxia.	检验医学与临床	oratory medicine and clin	7	12	1210-1211	2010	sn.1672-9455.2010.12.031
2904	沙门菌病的流行情况及防	vention and control strateg	邵华斌,艾地云,杨峻,罗玲	uabin,Ai Diyun,Yang Jun,L	中国家禽	Chinese poultry	32	12	51	2010	-
2905	染性腹泻2380例临床及病	rrhea2380Clinical and etio	,齐绪林,徐晓刚,樊晓明,	Xulin,Xu Xiaogang,Fan Xia	中华传染病杂志	se Journal of Infectious Dis	28	2	103-106	2010	sn.1000-6680.2010.02.009
2906	特沙门菌引起食物中毒的	a case of food poisoning ca	林毅.	Lin Yi.	中国卫生检验杂志	ese Journal of Health Inspe	20	11	30373039	2010	-
2907	便中沙门菌I类整合子与耐	thy pigsIDetection of integ	郁,姚健,谢倩,孙裴,王桂军	an,Xie Qian,Sun Pei,Wang	中国微生态学杂志	inese Journal of Microecol	22	7	594-598	2010	-
2908	沙门菌分离株耐药性分析	drug resistance of Salmon	刘红英,刘怀萍.	Liu Hongying,Liu Huaiping.	中国公共卫生	Chinese public health	26	5	633-634	2010	-
2909	沙门菌食物中毒事件的	f an incident of Salmonell	,方叶珍,徐丹戈,黄世旺,	ezhen,Xu Dange,Huang Sh	中国卫生检验杂志	ese Journal of Health Inspe	20	1	205-206	2010	-
2910	炎沙门菌家庭食物中毒	ood poisoning case caused	刘红丽.	Liu Hongli.	中国卫生检验杂志	ese Journal of Health Inspe	20	3	665-666	2010	-
2911	型副伤寒沙门菌引起食	rne illness caused by Salm	樊毅.	Fan Yi.	中国卫生检验杂志	ese Journal of Health Inspe	20	11	2978-2979	2010	-
2912	节液中培养出猪霍乱沙门	a choleraesuis from bone	陈坚,杨建华,张艳丽.	Jian,Yang Jianhua,Zhang	中华医院感染学杂志	ournal of Hospital Infectiou	20	11	1635	2010	-
2913	所从业人员沙门氏菌携带	lla carriage among employ	宝良,刘利香,雒引.	Baoliang,Liu Lixiang,Luo Yin	包头医学院学报	rnal of Baotou Medical Col	26	6	50-51	2010	n.1006-740X.2010.06.025
2914	食品沙门菌菌型分布及	istance of Salmonella in co	,熊海平,汤敏,张伟,赵哲,	aiping,Tang Min,Zhang W	江苏预防医学	iangsu Preventive Medicin	21	3	16-18	2010	sn.1006-9070.2010.03.006
2915	-2009年细菌性食物中毒	sis of annual bacterial food	葛少锋,倪丰安.	Ge Shaofeng,Ni Feng'an.	中国卫生检验杂志	ese Journal of Health Inspe	20	11	2945-2946	2010	-
2916	沙门菌污染糕点引发的食	ing caused by eating cakes	贺漓漓.	He Lili.	中国卫生检验杂志	ese Journal of Health Inspe	20	9	23592362	2010	-
2917	伤寒沙门菌感染40例临	ium infection in children40	刘永生.	Liu Yongsheng.	中国中西医结合儿科学	Chinese and Western Medi	2	1	89-90	2010	sn.1674-3865.2010.01.042
2918	农村学校伤寒暴发的调查	sis of a typhoid fever ou	向宏;向恩;	Xiang Hong;Xiang En;	公共卫生与预防医学	Health and Preventive M	21	5	71-72	2010	-
2919	一起伤寒暴发疫情调查分	n and analysis of a typh	祥;谭维莲;张建希;任永宏;	eilian;Zhang Jianxi;Ren	医学动物防制	medical animal control	26	10	942-944+946	2010	-
2920	中区光福镇一起食源性伤	oid fever in Guangfu To	刘涛;张钧;王桂平;钱迟华	ng Jun;Wang Guiping;Q	疾病监测	disease surveillance	25	8	673-674	2010	-
2921	甲型副伤寒暴发的流行病	tigation of an outbreak	涛;饶细林;张华林;金长	Xilin;Zhang Hualin;Jin	现代预防医学	odern preventive medici	37	15	2911-2912+2915	2010	-

2922	县一起伤寒暴发疫情调查	sis of a typhoid fever out	姚莹;	Yao Ying;	医学动物防制	medical animal control	26	5	454-455	2010	-
2923	市汉沽区某村伤寒暴发调查	hoid fever outbreak in a	刘国敏;	Liu Guomin;	河南预防医学杂志	Journal of Preventive M	21	2	149-150	2010	/j.cnki.hnjpm.2010.02.035
2924	校甲型副伤寒暴发疫情的调	is of an outbreak of para	易继宽;陈朴;胡儒元;	Jikuan;Chen Pu;Hu Ruyu	公共卫生与预防医学	Health and Preventive M	21	1	86-87	2010	-
2925	染导致伤寒暴发的流行病	n typhoid outbreak cause	舒进安;朱建刚;	Shu Jinan;Zhu Jiangang	实用心脑肺血管病杂志	rdiocerebral and Pulmon	18	1	50-51	2010	-
2926	2005-2010年细菌性食物病	y2005-2010Annual bacteri	杨慧,甘莉萍,金玉娟,李静娟	ui,Gan Liping,Jin Yujuan,Li J	职业与健康	Occupation and health	27	16	1847-1850	2011	-
2927	区伤寒副伤寒沙门菌株脉冲	yping of Salmonella typhi p	许玉玲,黄丽莉,郭大城,张	ng,Huang Lili,Guo Dacheng	河南预防医学杂志	n Journal of Preventive Me	22	4	246-248	2011	-
2928	-2010年腹泻病人及食品中	ysis of Salmonella detection	叶玉龙,钟海明.	Ye Yulong,Zhong Haiming.	中国卫生检验杂志	ese Journal of Health Inspe	21	11	2768-2769,2772	2011	-
2929	08年上海市食源性疾病监	ghai Foodborne Disease Su	陆冬磊,段胜钢,吴春峰,邢之	,Duan Shenggang,Wu Chun	中国食品卫生杂志	hinese Food Hygiene Journ	23	2	126-131	2011	-
2930	菌属临床分离株的耐药特点	demiology of clinical isolat	俏俏,段荣,柯江维,杨乐和	Duan Rong,Ke Jiangwei,Yan	中华检验医学杂志	se Journal of Laboratory Me	34	3	249-253	2011	sn.1009-9158.2011.03.013
2931	寒沙门菌引起食物中毒的病	a case of food poisoning ca	周莹,罗江秀.	Zhou Ying,Luo Jiangxiu.	按摩与康复医学（下旬刊）	nd Rehabilitation Medicine	2	7	249-250	2011	-
2932	边地区仔猪副伤寒的发病	aratyphoid fever in piglets i	叶润全,陈伟明.	Ye Runquan,Chen Weiming	黑龙江畜牧兽医（上半月）	ndry and Veterinary Medic	-	12	114-115	2011	-
2933	肉中三种食源性致病菌的耐	ree foodborne pathogenic b	侠,谭艾娟,吕世明,金志强,	n Aijuan,Lu Shiming,Jin Zhi	山地农业生物学报	l of Mountain Agricultural B	30	2	141-143,160	2011	sn.1008-0457.2011.02.010
2934	肠道传染病实验室监测结果	f intestinal infectious disea	飞,林志萍,梁桂玲,扈庆华	hiping,Liang Guiling,Hu Qin	中国医药科学	Chinese Medical Sciences	1	8	12-14,25	2011	-
2935	感染性肠炎的病原检测结	esults and analysis of acute	涂银萍,王晶,王久伶.	Yinping,Wang Jing,Wang Jiu	中国现代医学杂志	ese Journal of Modern Med	21	21	2608-2610	2011	-
2936	部分食品中食源性致病菌	us of foodborne pathogeni	闫爱莉,赵白雪,刘丽,时慧.	Aili,Zhao Baixue,Liu Li,Shi H	实用预防医学	ractical preventive medicin	18	10	1894-1895	2011	sn.1006-3110.2011.10.028
2937	腹泻病原菌监测及流行病学	cal characteristics of diarrh	海燕,张朝雄,周丽华,胡挺	n,Zhang Chaoxiong,Zhou Li	中国热带医学	Chinese tropical medicine	11	2	136-138	2011	-
2938	沙门菌引起的食物中毒调查	n a case of food poisoning	朱文军.	Zhu Wenjun.	江苏预防医学	Jiangsu Preventive Medicine	22	2	39-39	2011	sn.1006-9070.2011.02.023
2939	0年睢县致泻性病原菌监测	g results of diarrheal patho	宋信兵.	Song Xinbing.	河南预防医学杂志	n Journal of Preventive Me	22	5	370-371,382	2011	-
2940	市市售食品病原菌污染状况	tamination of commercially	周良君,陈果,王学军.	Liangjun,Chen Guo,Wang X	中国热带医学	Chinese tropical medicine	11	10	1234-1235	2011	-
2941	血流感染革兰阴性杆菌的耐	stance of Gram-negative ba	卢敏,闫川,汪安勇,蒋猛,沈	,Yan Chuan,Wang Anyong,	中国抗生素杂志	hinese Journal of Antibiotic	36	9	708-711	2011	-
2942	木齐市细菌性腹泻流行病学	nvestigation of bacterial di	,董路宁,李明,李力,仲英娜	g Luning,Li Ming,Li Li,Zhon	解放军预防医学杂志	tive Medicine of the People	29	3	192-193	2011	-
2943	儿童腹泻病原菌分布及耐	ce of pathogenic bacteria i	先斌,刘健龙,郭宽鹏,宋春	Jianlong,Guo Kuanpeng,So	实用预防医学	ractical preventive medicin	18	11	2195-2197	2011	sn.1006-3110.2011.11.069
2944	儿童败血症病原菌及耐药性	bacteria and drug resistan	张洲慧,彭珉娟,陈娟.	Zhouhui,Peng Minjuan,Che	四川医学	Sichuan Medicine	32	6	838-841	2011	sn.1004-0501.2011.06.018
2945	0年内蒙古通辽市食品污染	amination status in Tonglia	布仁巴图,吴凤云,王丽梅.	n Batu,Wu Fengyun,Wang L	疾病监测与控制	sease surveillance and cont	5	4	216-217	2011	-
2946	环境微生物致病性和耐药	ug resistance of environme	书科,黄秀梅,王玉东,李葳,	uang Xiumei,Wang Yudong	中国动物检疫	China Animal Quarantine	28	11	46-47	2011	n.1005-944X.2011.11.023
2947	封市2009年感染性腹泻监	e2009Annual surveillance	洪民,王德祥,张建萍,张翠	Wang Dexiang,Zhang Jianp	中国热带医学	Chinese tropical medicine	11	1	74-75	2011	-
2948	引起的食物中毒病原学检	analysis of a case of food p	王柏莲.	Wang Bolian.	医学动物防制	medical animal control	27	3	265267	2011	sn.1003-6245.2011.03.029
2949	门氏菌Ⅱ引起的食物中毒	ood poisoning incident cau	孙步武.	Sun Buwu.	应用预防医学	applied preventive medicine	17	1	64	2011	n.1673-758X.2011.01.025
2950	上海市金山区其他感染性	ics of other infectious diarr	宋灿磊,曹志诚,莫平华,朱	ei,Cao Zhicheng,Mo Pinghu	职业与健康	Occupation and health	27	23	2765-2767	2011	-
2951	胃患者非伤寒沙门氏菌监测	n-typhoid Salmonella in pa	罗泽燕,徐励琴,张健,黄超群	an,Xu Liqin,Zhang Jian,Hua	国际医药卫生导报	ational Medical and Health	17	20	2542-2545	2011	sn.1007-1245.2011.20.029
2952	门菌菌型分布和药敏分析	oodborne Salmonella in Sh	张毅,陈欣钦,宋昌彦.	g Yi,Chen Xinqin,Song Chan	现代生物医学进展	ogress in Modern Biomedic	11	20	3938-3941	2011	-
2953	副溶血性弧菌引起食物中	ood poisoning caused by S	胡全胜.	Hu Quansheng.	山东医学高等专科学校学报	nal of Shandong Medical Co	33	5	341-342	2011	sn.1674-0947.2011.05.009
2954	京市西城区食品食源性致	odborne pathogenic bacter	王敬辉,宋超,郭勇峰,杨宏	ui,Song Chao,Guo Yongfeng	职业与健康	Occupation and health	27	17	1967-1969	2011	-
2955	年上半年食源性致病菌监	borne pathogen monitorin	英姿,安静,侯元,刘建敏,王	Quiet,Hou Yuan,Liu Jianmin	中国卫生检验杂志	ese Journal of Health Inspe	21	10	2496-2497	2011	-
2956	省伤寒副伤寒高发地区综合	hoid fever in areas with hig	孙军玲,姚光海,田克诚,王	ling,Yao Guanghai,Tian Ked	预防医学情报杂志	of Preventive Medicine Info	27	7	483-487	2011	-
2957	畸胎瘤中分离出甲型副伤寒	onella paratyphi A from pa	方宁.	Fang Ning.	中华医院感染学杂志	ournal of Hospital Infectiou	21	4	673-673	2011	-
2958	湖区食品中食源性致病菌	nic bacteria contamination	吴水斌,周哲华,王恒辉.	uibin,Zhou Zhehua,Wang H	中国公共卫生管理	nese public health managem	27	1	101-102	2011	-

2959	210例婴幼儿腹泻流行病	ical analysis of diarrhea cas	曦,唐博恒,刘金华,王珊珊,	Boheng,Liu Jinhua,Wang S	广东医学	Guangdong Medicine	32	3	340-341	2011	sn.1001-9448.2011.03.027
2960	患者非伤寒沙门氏菌监	n-typhoid Salmonella in pa	泽燕,徐励琴,张健,黄超群	an,Xu Liqin,Zhang Jian,Hua	国际医药卫生导报	ational Medical and Health	17	19	2451-2454	2011	sn.1007-1245.2011.19.042
2961	乱沙门菌致腰部囊性肿块	aist caused by salmonella	,杨海梅,周玲,吴廷芳,王	mei,Zhou Ling,Wu Tingfang	临床检验杂志	journal of clinical testing	29	2	137-137	2011	-
2962	源沙门菌的鉴定及耐药性	ance detection of Salmone	张焕容,覃春红,马驰,魏峰	ng,Qin Chunhong,Ma Chi,W	中国家禽	Chinese poultry	33	16	61-63	2011	-
2963	沙门菌引起的食物中毒调	rt on a food poisoning cau	陈洪浩.	Chen Honghao.	中国社区医师（医学专业）	munity physician (medical	13	29	155-156	2011	sn.1007-614x.2011.29.152
2964	副伤寒沙门菌引起的食物	a Food Poisoning Caused	徐红.	Xu Hong.	中外医疗	Chinese and foreign medica	30	1	36-37	2011	sn.1674-0742.2011.01.019
2965	卤熟食食品中检出沙门菌	ction of Salmonella in brais	曾远伟,谢承恩,刘虹.	Yuanwei,Xie Chengen,Liu	中国医药指南	Chinese Medicine Guide	9	33	149-150	2011	sn.1671-8194.2011.33.115
2966	市食品中食源性致病菌污	status of foodborne patho	杨洋,苗艳芳,李涛,王瑶,曹	Miao Yanfang,Li Tao,Wang	中国卫生检验杂志	ese Journal of Health Inspe	21	7	1767-1768	2011	-
2967	监狱食品从业人员健康检	on results of food workers i	李佐伦,谭浪,陈文菊.	Zuolun,Tan Lang,Chen Wen	海峡预防医学杂志	s Journal of Preventive Me	17	1	48-49	2011	-
2968	食品微生物及微生物毒素	of microorganisms and mic	质文,马维兴,张健,韩丽娟	wen,Ma Weixing,Zhang Jian	检验检疫学刊	al of Inspection and Quara	21	2	5-10	2011	-
2969	染沙门菌耐药基因调查与	rug resistance genes of Sal	坚,吴劲松,徐英春,陈民钧	Jinsong,Xu Yingchun,Chen	中华检验医学杂志	e Journal of Laboratory Me	34	5	431-436	2011	sn.1009-9158.2011.05.009
2970	氏菌的分离鉴定及侵袭蛋	roteins of Salmonella in fee	瑞欣,曾昭文,朱天乐,陈合	in,Zeng Zhaowen,Zhu Tian	畜牧与兽医	Husbandry and Veterinary	43	11	76-79	2011	-
2971	010年食源性致病菌污染	nnual foodborne pathogen	张利焱.	Zhang Liyan.	医学动物防制	medical animal control	27	9	860862	2011	sn.1003-6245.2011.09.035
2972	食源性沙门菌菌型分布及	rug susceptibility results o	雪梅,廖国东,陈家图,黄水	Liao Guodong,Chen Jiatu,H	中国卫生检验杂志	ese Journal of Health Inspe	21	3	644-645,647	2011	-
2973	畜禽肉及凉拌菜中沙门氏	stock and poultry meat and	,师俊玲,只帅,席美丽,申	ng,Only handsome,Xi Meili	西北农业学报	rnal of Northwest Agricult	20	7	22-27	2011	sn.1004-1389.2011.07.006
2974	副伤寒沙门菌引起的食物	of food poisoning caused b	朱翔,包云娟.	Zhu Xiang,Bao Yunjuan.	浙江预防医学	hejiang Preventive Medicin	23	12	49-50	2011	sn.1007-0931.2011.12.019
2975	南宁畜禽食品及病禽中沙	ella in livestock and poultry	SCO CHIESA,韦平,梁冬	HIESA,Wei Ping,Liang Don	食品科学	food science	32	21	198-200	2011	-
2976	副伤寒沙门菌脉冲场凝胶	ctrophoresis of Salmonella	涛,瞿媛,杨建斌,尹建雯,	Yuan,Yang Jianbin,Yin Jian	现代预防医学	modern preventive medicin	38	14	2823-2825	2011	-
2977	50株沙门菌PFGE指纹图	e50SalmonellaPFGEFinger	劲松,陈建辉,郑金凤,陈爱	Chen Jianhui,Zheng Jinfeng	海峡预防医学杂志	s Journal of Preventive Me	17	3	7-9	2011	-
2978	速检测从业人员肠道致病	pid detection of intestinal	扬,刘海文,彭巨成,曾维,卢	aiwen,Peng Jucheng,Zeng	中国热带医学	Chinese tropical medicine	11	8	971-972	2011	-
2979	下积液检出1株都柏林沙门	of fluid under scalp1Salmo	陈荔.	Chen Li.	中外健康文摘	nese and Foreign Health Di	8	24	191-191	2011	sn.1672-5085.2011.24.162
2980	生食品源沙门氏菌的耐药	ance of Salmonella from ar	周佳,刘书亮,侯小刚,居华	,Liu Shuliang,Hou Xiaogang	中国畜牧兽医	mal husbandry and veterina	38	3	188-191	2011	-
2981	重复序列分析应用于中国	multilocus tandem repeat	妮,陈春霞,刁保卫,娄静,	n Chunxia,Diao Baobao,Lo	疾病监测	disease surveillance	26	4	264-270	2011	-
2982	门氏菌的分离鉴定与Dot-E	ation of Salmonella in feed	韩志辉,张红见.	Han Zhihui,Zhang Hongjian	安徽农业科学	Anhui Agricultural Sciences	39	26	16139-16140,16163	2011	sn.0517-6611.2011.26.121
2983	猪肉中沙门氏杆菌的耐药	etection of Salmonella in p	侠,谭艾娟,吕世明,金志强,	n Aijuan,Lu Shiming,Jin Zhi	贵州农业科学	uizhou Agricultural Science	39	4	133-135	2011	sn.1001-3601.2011.04.042
2984	消费食品食源性致病菌及	ne Pathogens and Coliform	黄丽华,唐保晖.	Huang Lihua,Tang Baohui.	医学动物防制	medical animal control	27	7	642-642,644	2011	sn.1003-6245.2011.07.023
2985	贵州省伤寒和副伤寒沙门	nce of Salmonella typhi and	旅,韦小瑜,马青,刘英,田克	Xiaoyu,Ma Qing,Liu Ying,Ti	医学动物防制	medical animal control	27	8	687-690	2011	sn.1003-6245.2011.08.001
2986	市龙岗区细菌性 食物中毒	enzhen CityAnalysis of path	慧,甘莉萍,陈应坚,金玉娟	Gan Liping,Chen Yingjian,Ji	疾病监测	disease surveillance	26	7	543-546,559	2011	sn.1003-9961.2011.07.013
2987	标本细菌及其L型分离培养	eria and theirLAnalysis of	杨俊,王云芬.	Yang Jun,Wang Yunfen.	检验医学与临床	boratory medicine and clini	8	1	95-96	2011	sn.1672-9455.2011.01.052
2988	部沙门氏菌食物中毒72例	ella Food Poisoning72Case	彭易根,张正群.	Peng Yigan,Zhang Zhengqun	现代医药卫生	modern medicine and healt	27	4	571-572	2011	-
2989	苏家屯区细菌性食物中毒	bacterial food poisoning in	张良.	Zhang Liang.	中外健康文摘	nese and Foreign Health Di	8	13	444-445	2011	sn.1672-5085.2011.13.448
2990	份临床血液标本细菌培养	culture results of clinical b	,储从家,管新龙,李杰芬,	u Congjia,Guan Xinlong,Li Ji	中国感染控制杂志	ese Journal of Infection Co	10	3	209-213	2011	sn.1671-9638.2011.03.013
2991	区腹泻病人沙门菌感染水	ion Levels in Diarrhea Patie	健君,罗泽燕,曹海燕,柯碧	un,Luo Zeyan,Cao Haiyan,K	热带医学杂志	journal of tropical medicine	11	1	90-92,101	2011	-
2992	江苏省食源性致病菌耐药	nitoring of foodborne path	赟,马恺,符晓梅,乔昕,王燕	Kai,Fu Xiaomei,Qiao Xin,W	江苏预防医学	iangsu Preventive Medicin	22	6	44,68	2011	sn.1006-9070.2011.06.025
2993	境废物原料病原微生物污	genic microorganisms in ra	吉城,李小波,相大鹏,张顺	,Li Xiaobo,Xiang Dapeng,Zh	中国卫生检验杂志	ese Journal of Health Inspe	21	6	1509-1511	2011	-
2994	恰地区动物性食品中沙门	in animal foods in Qabqa	王寿,李增魁.	Wang Shou,Li Zengkui.	畜牧与饲料科学	Livestock and feed science	-	6	31-33	2011	sn.1672-5190.2011.06.016
2995	病性大肠埃希菌及沙门菌	genic Escherichia coli and	贺晓龙,史福胜.	He Xiaolong,Shi Fusheng.	动物医学进展	vances in Veterinary Medic	32	11	123-126	2011	sn.1007-5038.2011.11.029

2996	食品食源性致病菌谱及耐	m and drug resistance stat	马智龙,蔡震.	Ma Zhilong,Cai Zhen.	中国卫生检验杂志	ese Journal of Health Inspe	21	9	2297-2299,2302	2011	-
2997	年洛阳市食品致病菌污染	athogenic bacteria contami	仝志琴.	gay piano.	预防医学情报杂志	of Preventive Medicine Info	27	11	934-935	2011	-
2998	泻739例病原菌分布和耐	stribution and drug resista	李晓云,吴永胜.	Li Xiaoyun,Wu Yongsheng.	西北国防医学杂志	west Journal of Defense Me	32	3	188-189	2011	-
2999	及散发腹泻患者沙门菌PF	poisoning and sporadic dia	彦,唐振柱,李秀桂,孙贵娟	ang Zhenzhu,Li Xiugui,Sun	应用预防医学	pplied preventive medicin	17	3	140-143	2011	n.1673-758X.2011.03.004
3000	病化疗并发脓毒症60例	atological diseases complic	何云燕,罗建明,刘强,李萌	an,Luo Jianming,Liu Qiang,	实用儿科临床杂志	ctical Pediatric Clinical Jou	26	6	412-414	2011	n.1003-515X.2011.06.009
3001	09年茂名市食源性致病菌	is of foodborne pathogeni	国东,黄水飞,许铭清,陈家	Huang Shuifei,Xu Mingqing	中国热带医学	Chinese tropical medicine	11	4	434-435	2011	-
3002	年深圳市南山区食品污染	sis of food contaminants in	袁月明,袁梦,程妍.	Yueming,Yuan Meng,Cheng	河南预防医学杂志	n Journal of Preventive Me	22	6	474-475,481	2011	-
3003	沙门氏菌引起食物中毒的	ase of food poisoning cause	廖斌,李怡,候敏,刘贵明.	Bin,Li Yi,Hou Min,Liu Guim	内蒙古中医药	ngolia Traditional Chinese	30	4	115-116	2011	sn.1006-0979.2011.04.140
3004	4-2008年伤寒、副伤寒流	ual epidemic characteristic	楼江红,吴铭宇.	Lou Jianghong,Wu Mingyu.	中国公共卫生管理	ese public health managen	27	3	264-265	2011	-
3005	在食品从业人员沙门氏菌	ube method in the detectio	张建新.	Zhang Jianxin.	江苏卫生保健	Jiangsu Health Care	13	3	17-18	2011	sn.1008-7338.2011.03.011
3006	水县市售食品食源性致病	orne pathogenic bacteria i	章红顺.	Zhang Hongshun.	河南预防医学杂志	n Journal of Preventive Me	22	5	385-386,393	2011	-
3007	0年四类食源性致病菌污	ion of four types of foodbo	,倪颇男,胡卓,石文松,卓	nan,Hu Zhuo,Shi Wensong,	医药前沿	Medical Frontier	1	23	178-178	2011	sn.2095-1752.2011.23.128
3008	性细菌感染性腹泻患儿沙	in Children with Acute Ba	磊燕,许学斌,宋建明,薛建	uebin,Song Jianming,Xue J	微生物与感染	Microbes and infections	6	3	139-143	2011	sn.1673-6184.2011.03.003
3009	区猪"高热病"流行特征及	n"High fever"Epidemic cha	陈金山,赵坤,胡建和,王自	han,Zhao Kun,Hu Jianhe,W	广东农业科学	angdong Agricultural Scien	38	15	92-95	2011	n.1004-874X.2011.15.034
3010	年全国伤寒和副伤寒监测	ce analysis of typhoid feve	伟东,闫梅英,王子军,张静	dong,Yan Meiying,Wang Zij	疾病监测	disease surveillance	26	4	256-260	2011	-
3011	10年食品中食源性致病菌	nitoring results of foodbor	张志祥,于雁,钱邢莹.	g Zhixiang,Yu Yan,Qian Xing	浙江预防医学	hejiang Preventive Medicin	23	11	45,70	2011	sn.1007-0931.2011.11.016
3012	菌和沙门菌药敏检验的临	g susceptibility testing of S	杨玉岑,冯扬.	Yang Yucen,Feng Yang.	健康必读（下旬刊）	ust-read for health (late iss	-	10	269261	2011	-
3013	海珠区市食品污染物监测	ant monitoring results in H	刘馨桦,郭宁晓,宋叶.	Xinhua,Guo Ningxiao,Song	医学动物防制	medical animal control	27	5	408-410	2011	sn.1003-6245.2011.05.006
3014	年麦盖提县伤寒、副伤寒	data of typhoid fever and	衣努尔·肉孜,阿布力肯·阿	Mayinur·Rouzi,Abricon·Ab	预防医学论坛	Preventive Medicine Forum	17	8	765-766,767	2011	-
3015	78例腹泻患者病原菌检测	thogenic bacteria detectio	曦,唐博恒,刘金华,王珊珊,	Boheng,Liu Jinhua,Wang S	华南预防医学	uth China Preventive Medic	37	4	80-81	2011	-
3016	血液中检出典型伤寒沙门	on of typical Salmonella ty	沈隽卿,曹钟艺.	Shen Junqing,Cao Zhongyi.	职业与健康	Occupation and health	27	10	1110-1111	2011	-
3017	年市售食品中食源性致病	lysis of foodborne pathoge	,杨小波,黄家兵,梅红,司徒	g Xiaobo,Huang Jiabing,plu	中外健康文摘	nese and Foreign Health Di	8	21	55-57	2011	sn.1672-5085.2011.21.047
3018	并鼠伤寒沙门菌肺炎误诊	ver Cirrhosis and Salmonell	耿志强,刘华雷.	Geng Zhiqiang,Liu Hualei.	河北医药	Hebei Medicine	33	17	2718	2011	sn.1002-7386.2011.17.100
3019	市售食品中食源性病原菌	borne pathogenic bacteria i	洪丽君.	Hong Lijun.	中国卫生检验杂志	ese Journal of Health Inspe	21	1	235-236	2011	-
3020	鸭沙门杆菌分离鉴定及耐	rug resistance analysis of S	婧瑶,范斌,曹羽,刘欢欢,王	ao,Fan Bin,Cao Yu,Liu Hua	黑龙江畜牧兽医（上半月）	ndry and Veterinary Medic	-	8	124-126	2011	-
3021	区2008－2010年食源性致	Annual surveillance analysi	王敬辉,简友平,高平,杨宏	hui,Jian Youping,Gao Ping,Y	中国预防医学杂志	se Journal of Preventive Me	12	8	709-710	2011	-
3022	系中大肠埃希菌和沙门菌	of Escherichia coli and Salm	,李珺峤,吴希阳,于辉,林立	Junqiao,Wu Xiyang,Yu Hui,	动物医学进展	vances in Veterinary Medic	32	6	96-100	2011	sn.1007-5038.2011.06.023
3023	岩市部分食源性致病菌污	tus of some foodborne pat	红,曹春远,何云,陈炳材,林	Chunyuan,He Yun,Chen Bin	预防医学论坛	Preventive Medicine Forum	17	7	603-605	2011	-
3024	鲫鱼肠道中沙门菌的调查	monella in the intestinal tr	晓,胡秀彩,祁越,王艺,吕	ao,Hu Xiucai,Qi Yue,Wang Y	中国微生态学杂志	inese Journal of Microecolo	23	12	1082-1083	2011	-
3025	患儿血液中检出猪霍乱沙	eraesuis was detected in th	郝君,孙解生.	Hao Jun,Sun Jiesheng.	国际检验医学杂志	onal journal of laboratory	32	1	142-143	2011	sn.1673-4130.2011.01.076
3026	性沙门氏菌的分离鉴定及	ivity testing of pathogenic	谭伟成,卢景.	Tan Weicheng,Lu Jing.	上海畜牧兽医通讯	nal Husbandry and Veterina	-	2	43-45	2011	sn.1000-7725.2011.02.019
3027	2010年沙门菌感染性腹泻	of Salmonella Infectious Di	浩淼,李康,崔艳红,胡松	o,Li Kang,Cui Yanhong,Hu S	现代预防医学	modern preventive medicin	38	17	3558-3560	2011	-
3028	直接入口食品中食源性致	ogenic bacteria in directly	封会茹,董晓根,余红,赵伟	ru,Dong Xiaogen,Yu Hong,Z	职业与健康	Occupation and health	27	11	1258-1260	2011	-
3029	脓肿穿刺液中分离出都柏	from a patient's parotid gl	徐霞,童明华,蔡华,梁日初	Tong Minghua,Cai Hua,Lian	检验医学与临床	boratory medicine and clini	8	16	2041-2042	2011	sn.1672-9455.2011.16.079
3030	感染性腹泻患者病原菌检	hogenic bacteria in outpat	爱玲,刘松山,管恩聚,赵淑	,Liu Songshan,Guan Enju,Z	青岛医药卫生	ingdao Medicine and Healt	43	1	35-36	2011	sn.1006-5571.2011.01.019
3031	原料奶沙门氏菌污染程度	onella contamination degr	文茹,陈庆森,庞广昌,朱晨	Qingsen,Pang Guangchan	食品科技	food technology	36	2	285-289	2011	-
3032	沙门菌混合感染的病原分	ibility test for mixed infecti	李卫,白家媛,蔡双双.	ei,Bai Jiayuan,Cai Shuangsh	动物医学进展	vances in Veterinary Medic	32	1	124-127	2011	sn.1007-5038.2011.01.030

3033	的耐药性研究及肠毒素基因	rrhea and analysis of the r	虽,余晓君,周红平,柯江维,乡	ojun,Zhou Hongping,Ke Jia	华临床医师杂志（电子版	urnal of Clinicians (electron	5	18	5360-5365	2011	sn.1674-0785.2011.18.027
3034	校周边市售凉拌食品微生物	mmercially available cold	周藜,朱玫,周倩,陈依江.	i,Zhu Mei,Zhou Qian,Chen Y	中国学校卫生	Chinese school health	32	9	1094-1095	2011	092/R.20110920.1229.050
3035	七年细菌性食物中毒的病原	cs of bacterial food poisoni	志爱,张欣强,庞杏林,刘俊	ang Xinqiang,Pang Xinglin,	中国卫生检验杂志	ese Journal of Health Inspe	21	3	622-624,627	2011	-
3036	中食源性病原菌污染现况	uation of Foodborne Patho	二,朱珠英,张素燕,陈文善,月	uying,Zhang Suyan,Chen W	浙江预防医学	hejiang Preventive Medicin	23	5	12-13,16	2011	sn.1007-0931.2011.05.004
3037	007～2010年沙门菌监测结	Analysis of annual Salmon	占,张元玲,李德华,薛镧,周汶	g Yuanling,Li Dehua,Xue La	国外医学（医学地理分册）	Medicine (Medical Geograp	32	4	251-253	2011	sn.1001-8883.2011.04.008
3038	北京市西城区肠道病原菌监	enteric pathogenic bacteri	王利萍,崔京辉,王永全,杨青	,Cui Jinghui,Wang Yongqu	职业与健康	Occupation and health	27	20	2311-2314	2011	-
3039	沙门菌和金黄色葡萄球菌	la and Staphylococcus aure	矛,王海燕,柯碧霞,谭海玲,李	g Haiyan,Ke Bixia,Tan Haili	中华流行病学杂志	inese Journal of Epidemiolo	32	9	918-923	2011	sn.0254-6450.2011.09.016
3040	市餐饮服务食品安全监督抽	upervision and inspection c	许金凤,王抒倩.	Xu Jinfeng,Wang Shuqian.	江苏预防医学	iangsu Preventive Medicine	22	6	39-40	2011	sn.1006-9070.2011.06.022
3041	新型副伤寒沙门氏菌感染1例	typhi C infection in infants	胡学惠.	Hu Xuehui.	中国健康月刊（A）	China Health Monthly (A)	-	5	-	2011	-
3042	业人员与腹泻患者沙门氏菌	s among catering workers	燕,曾健君,徐励琴,刘燕,冯	g Jianjun,Xu Liqin,Liu Yan,F	疾病监测与控制	sease surveillance and cont	5	5	257-258	2011	-
3043	市昌平区食品中食源性致	of foodborne pathogenic b	海玲,金文军,孙伟,刘小兵	Jin Wenjun,Sun Wei,Liu Xia	中国预防医学杂志	se Journal of Preventive Me	12	8	714-715	2011	-
3044	亚运会涉亚食品监测结果	od monitoring results at th	新才,陈坤才,陈纯,丁鹏,陈	Kuncai,Chen Chun,Ding Pe	中国卫生检验杂志	ese Journal of Health Inspe	21	11	2765-2767	2011	-
3045	市售凉拌菜的卫生微生物监	nitoring of commercially av	刘晓黎,肖双,周艺,杨赟,陈	oli,Xiao Shuang,Zhou Yi,Yan	中国卫生检验杂志	ese Journal of Health Inspe	21	10	2524-2525,2527	2011	-
3046	港市从业人员肠道沙门菌	almonella among Employee	柳丽江.	Liu Lijiang.	预防医学情报杂志	of Preventive Medicine Info	27	5	359-362	2011	-
3047	肠炎沙门菌肠炎致休克1例	eritidis Shock Caused by En	张俊兰,潘赟.	Zhang Junlan,Pan Yun.	中国社区医师（医学专业）	munity physician (medical	13	22	260	2011	sn.1007-614x.2011.22.251
3048	主要食源性致病菌污染现状	tion status of major foodbo	卓,石文松,钟艳,方英,杨林	Wensong,Zhong Yan,Fang Yi	热带病与寄生虫学	pical Diseases and Parasito	9	4	222-223,234	2011	sn.1672-2302.2011.04.014
3049	夏朝觐人群携带圣水的卫	gical testing of holy water	公,马红霞,徐勤伟,朱玉香,夏	ongxia,Xu Qinwei,Zhu Yuxi	中国国境卫生检疫杂志	ntier Health and Quarantin	34	3	155-157	2011	-
3050	外分离的猪霍乱沙门菌耐	ce of extraintestinal Salmo	祁粉琴,蒋海燕,葛锁华.	enqin,Jiang Haiyan,Ge Suol	临床医学工程	clinical medical engineering	18	4	610-612	2011	n.1674-4659.2011.04.0610
3051	菌药敏分析及其所致儿童	id Salmonella strains and c	,龙晓玲,梁展图,张泉山,倪	oling,Liang Zhantu,Zhang Q	微生物与感染	Microbes and infections	6	3	149-152	2011	sn.1673-6184.2011.03.005
3052	京市通州区肠道病原菌监测	ults of enteric pathogenic b	新,刘秀军,刘晓峰,王宝兰,	Xiujun,Liu Xiaofeng,Wang B	中华疾病控制杂志	hese Journal of Disease Con	15	10	869-871	2011	-
3053	南省食物中毒突发公共卫生	cs of food poisoning public	何景阳.	He Jingyang.	河南预防医学杂志	n Journal of Preventive Me	22	2	146-148	2011	-
3054	的耐药性及脉冲场凝胶电	d gel electrophoresis typing	董剑辉,熊惠军,宋立,陈瑞	nhui,Xiong Huijun,Song Li,C	动物医学进展	vances in Veterinary Medic	32	5	9-13	2011	sn.1007-5038.2011.05.003
3055	模养猪场主要病原菌的耐	athogenic bacteria in large	,王开功,嵇辛勤,马喆,何瑾	aigong,Ji Xinqin,Ma Zhe,He	贵州农业科学	uizhou Agricultural Science	39	8	132-135	2011	sn.1001-3601.2011.08.038
3056	生猪主要细菌学指标的调	e main bacteriological indi	张静,周源,韩玉虎,汪仁莉	g,Zhou Yuan,Han Yuhu,Wa	畜牧与饲料科学	Livestock and feed science	32	5	89-90	2011	sn.1672-5190.2011.05.045
3057	年细菌性食物中毒病原菌检	detection results of bacte	英,高文洁,王恒辉,陈黎霞,	Wenjie,Wang Henghui,Che	现代预防医学	modern preventive medicin	38	1	30-31,33	2011	-
3058	沙门氏菌引起食物中毒的调	is of a case of food poisoni	卫东,刘晓娟,郑秀清,赵玉	iu Xiaojuan,Zheng Xiuqing,	医学动物防制	medical animal control	27	1	56,58	2011	sn.1003-6245.2011.01.025
3059	食品中检出姆瓦林沙门菌	Mwarin was detected in fo	苏靖华,王闻卿,傅慧琴.	nghua,Wang Wenqing,Fu H	上海预防医学	hanghai Preventive Medicin	23	4	157-158	2011	sn.1004-9231.2011.04.006
3060	2009年肉类和水产品食源	ance of foodborne pathoge	,乔昕,符晓梅,沈赟,袁宝君	Xin,Fu Xiaomei,Shen Yun,Y	中国公共卫生	Chinese public health	27	5	539-541	2011	-
3061	腹泻患儿的病毒检验结果	virus test results in childre	王玉环.	Wang Yuhuan.	按摩与康复医学（下旬刊）	nd Rehabilitation Medicine	2	6	248-248	2011	-
3062	2007-2009年市售熟肉制品	gienic quality analysis of co	萧金长.	Xiao Jinchang.	华南预防医学	uth China Preventive Medic	37	3	71-72	2011	-
3063	细菌性食源性疾病的病因	he causes of bacterial food	徐晓红,朱芳芳.	Xu Xiaohong,Zhu Fangfang	中外健康文摘	hese and Foreign Health Di	8	33	12-13	2011	sn.1672-5085.2011.33.006
3064	,沙门菌致感染性腹主动脉	aneurysm caused by Salmo	张益辉,任嘉玮,王泽球.	g Yihui,Ren Jiawei,Wang Ze	中国感染与化疗杂志	urnal of Infection and Chen	11	2	159-160	2011	-
3065	起细菌性食物中毒的检验报	on a case of bacterial food	李红.	Li Hong.	中外医疗	Chinese and foreign medica	30	6	102-102	2011	sn.1674-0742.2011.06.077
3066	无锡市部分腹泻患者病原学	testing data of some diarr	贾兆国.	Jia Zhaoguo.	预防医学论坛	Preventive Medicine Forum	17	12	1133-1134	2011	-
3067	鸡蛋及死胚蛋中沙门氏菌的	nella in food eggs and dead	陈晓平,徐桂云,郑江霞,杨	iaoping,Xu Guiyun,Zheng J	农产品加工·学刊	ing of agricultural products	-	7	112-114	2011	671-9646(X).2011.07.032
3068	细菌性食源性疾病流行病	teristics of bacterial foodb	,刘淮玉,刘宏,胡国泉,单月	aiyu,Liu Hong,Hu Guoquan,	医学信息（上旬刊）	dical information (early iss	24	10	6403-6404	2011	sn.1006-1959.2011.10.069
3069	猪霍乱沙门菌败血症1例报	Salmonella septicemia1Ex	张玉臣,聂翠芳,张延美.	Yuchen,Nie Cuifang,Zhang Y	山东医药	Shandong Medicine	51	5	107	2011	n.1002-266X.2011.05.070

3070	饮、公共场所从业人员沙	Carrying Status among Em	超育,李文,梁炯明,宋斌,罗	Wen,Liang Jiongming,Song	预防医学论坛	Preventive Medicine Forum	17	11	1020-1022	2011	-
3071	布利丹沙门菌食物中毒的	a case of Salmonella bulid	可凡,祝小平,朱保平,马会来	aoping,Zhu Baoping,The ho	中华流行病学杂志	inese Journal of Epidemiolo	32	7	697-699	2011	sn.0254-6450.2011.07.014
3072	培养的病原菌分布及耐药	acteria distribution and dru	施茜.	Shi Qian.	中国医疗前沿	China Medical Frontier	6	23	53-56	2011	n.1673-5552.2011.23.0030
3073	~2009年沙门氏菌流行病学	almonella epidemiological	石一,颜虹,岳永杰,马国柱	an Hong,Yue Yongjie,Ma G	陕西医学杂志	Shaanxi Medical Journal	40	6	726-727	2011	sn.1000-7377.2011.06.034
3074	菌败血症伴感染性腹主动	port sepsis and infected ab	吴擘颋,戎瑞明.	Wu Bo Ting,Rong Ruiming.	中华传染病杂志	se Journal of Infectious Dis	29	9	523	2011	sn.1000-6680.2011.09.003
3075	性肠炎沙门氏菌的流行病学	tigation of chicken-borne	张荣武,徐彪,朱来华,肖西志	Rongwu,Xu Biao,Zhu Laihu	中国人兽共患病学报	Chinese Journal of Zoonose	27	5	455-458	2011	sn.1002-2694.2011.05.023
3076	市食品中食源性致病菌污	dborne pathogenic bacteria	吴晓芳,纪蕾,徐德顺,陈莉萍	ofang,Ji Lei,Xu Deshun,Che	中国卫生检验杂志	ese Journal of Health Inspe	21	1	217-218,220	2011	-
3077	菌和伦敦沙门菌引起食物	poisoning caused by Proteu	高亚色,李恩.	Gao Ya color,Li En.	中国卫生检验杂志	ese Journal of Health Inspe	21	9	2222-2223,2225	2011	-
3078	门菌引起的食物中毒暴发	outbreak of food poisoning	张建梅,梁小亮,宣迎梅,张晶	,Liang Xiaoliang,Xuan Yingr	海峡预防医学杂志	s Journal of Preventive Me	17	4	42-44	2011	-
3079	北京市沙门菌血清型和药	monella serotypes and dru	桂荣,黄芳,黄瑛,李洁,王全	uang Fang,Huang Ying,Li Ji	中国预防医学杂志	se Journal of Preventive Me	12	11	899-901	2011	-
3080	血培养病原菌的种类及耐药	d drug resistance of pathog	朱华山.	Zhu Huashan.	医学信息（下旬刊）	edical information (late issu	24	6	291-292	2011	-
3081	起沙门氏菌食物中毒的调	into a case of salmonella fo	李巧荣,李秋梅,张领弟.	aorong,Li Qiumei,Zhang Lin	中国公共卫生管理	ese public health managen	27	3	309	2011	-
3082	环境中主要病原菌耐药性	of major pathogenic bacte	嵇辛勤,文明,傅心亮,李世	n,civilization,Fu Xinliang,Li S	山地农业生物学报	l of Mountain Agricultural	30	6	554-557	2011	sn.1008-0457.2011.06.017
3083	宴席食物中毒的实验室检	lysis of four cases of food p	张汉菊.	Zhang Hanju.	中国卫生检验杂志	ese Journal of Health Inspe	21	11	2754-2755	2011	-
3084	慢性腹泻患者菌源性分析	rial origin in patients with	袁飞.	Yuan Fei.	中国实验诊断学	nese experimental diagnos	15	4	725-726	2011	sn.1007-4287.2011.04.060
3085	部沙门氏茵食物中毒72例	poisoning in Arizona72Cas	彭易根,张正群.	Peng Yigan,Zhang Zhengqun	现代医药卫生	modern medicine and healt	-	4	571-572	2011	-
3086	一起食物中毒调查	food poisoning investigatio	魏德清.	Wei Deqing.	预防医学情报杂志	of Preventive Medicine Info	27	3	238	2011	-
3087	柏林沙门氏菌引起的食物中	of food poisoning caused	张光筋,韦李明,李红军.	Guangjin,Wei Liming,Li Ho	应用预防医学	applied preventive medicin	17	3	191	2011	n.1673-758X.2011.03.023
3088	童败血症病原菌及耐药回	hogenic bacteria and drug	温柏平,肖曙芳,倪林仙,黄	aiping,Xiao Shufang,Ni Linxi	当代医学	contemporary medicine	17	36	93-94	2011	sn.1009-4393.2011.36.064
3089	市部分医院污水处理效果	age treatment effect of son	封永建,高文征.	eng Yongjian,Gao Wenzhen	中国消毒学杂志	hinese Journal of Disinfecti	28	1	76-78	2011	-
3090	市蛋鸡疾病流行情况调查与	n the prevalence of laying	崔大春,汪招雄,彭本英,张士	n,Wang Zhaoxiong,Peng Be	养殖与饲料	Breeding and feed	-	1	35-36	2011	n.1671-427X.2011.01.022
3091	服务行业人群中肠道致病菌	bacteria among people in	戚杰,戚婉婷.	Qi Jie,Qi Wanting.	中外医疗	Chinese and foreign medica	30	3	33-33	2011	sn.1674-0742.2011.03.022
3092	养常见病原菌分布及耐药性	resistance of common pat	吴润香,张银辉,林广城,何	xiang,Zhang Yinhui,Lin Guar	海南医学	Hainan Medicine	22	12	135-137	2011	sn.1003-6350.2011.12.058
3093	脓毒血症病原菌分布及药	tivity of pathogenic bacter	石艳,王西蓉,杨钊,苏先旭	Wang Xirong,Yang Zhao,Su	四川医学	Sichuan Medicine	32	4	526-529	2011	sn.1004-0501.2011.04.034
3094	北京市感染性腹泻病原学监	alysis of the etiology of infe	园,张新,李洁,严寒秋,高志	n,Zhang Xin,Li Jie,severe co	中华预防医学杂志	se Journal of Preventive Me	45	9	820-824	2011	sn.0253-9624.2011.09.012
3095	校甲型副伤寒疫情流行病	gation of a paratyphoid fev	艳,兰才燕,牙韩芬,郑德文,程	Caiyan,Ya Hanfen,Zheng De	中国学校卫生	Chinese school health	32	8	1006-1007	2011	092/R.20110816.1046.049
3096	炎患儿血中检出鼠伤寒沙	n blood of children with pu	樊卫红,安欧,刘杰.	Fan Weihong,Anou,Liu Jie.	中国误诊学杂志	inese Journal of Misdiagno	11	31	7751	2011	-
3097	混合感染引起关节炎的诊	of arthritis caused by mixe	莲,磨美兰,林广津,黄柏成,赵	lan,Lin Guangjin,Huang Bai	养禽与禽病防治	and poultry disease preven	-	4	37-39	2011	-
3098	门氏菌携带者引起人群急	cute diarrhea caused by Sa	殷淑权.	Yin Shuquan.	中国社区医师（医学专业）	munity physician (medical	13	31	326	2011	sn.1007-614x.2011.31.322
3099	水中嗜肺军团菌的脉冲场	gionella pneumophila in wa	梅,秦丽云,周吉坤,王苋,周	Liyun,Zhou Jikun,Wang Ra	环境与健康杂志	rnal of Environment and He	28	11	995-997	2011	-
3100	07年-2010年食源性致病菌	ual surveillance analysis of	叶艳华,江晓,王炜,丁洁.	hua,Jiang Xiao,Wang Wei,D	中国卫生检验杂志	ese Journal of Health Inspe	21	10	2487-2488	2011	-
3101	腹泻沙门茵分型与耐药机	mechanisms of Salmonella	林兰,丁宏,崔生辉,张庆生	Hong,Cui Shenghui,Zhang	中国药事	hinese pharmaceutical affai	25	8	836-840,847	2011	-
3102	河南省登封市感染性腹泻监	of infectious diarrhea in D	洪民,王德祥,张建萍,张翠	Wang Dexiang,Zhang Jianp	疾病预防控制通报	se Prevention and Control	26	4	28-29	2011	-
3103	市年食源性疾病危险因素	orne disease risk factor sur	梁秀川.	Liang Xiuchuan.	职业卫生与病伤	ccupational health and illne	26	2	73-77	2011	n.1006-172X.2011.02.004
3104	西州伤寒流行特征与病原	d etiological surveillance o	科,吴戈,姚华,孙灵芝,向王	Wu Ge,Yao Hua,Sun Lingzh	预防医学论坛	Preventive Medicine Forum	17	9	841-843	2011	-
3105	门氏菌新近分离株的耐药	of newly isolated pathoge	春杰,李银聚,吴庭才,李小	Chunjie,Li Yinju,Ngo Dinh	中国兽医科学	Chinese Veterinary Science	41	7	751-755	2011	-
3106	菌带菌情况调查及其血清	la in chickens and analysis	张宏海,方艳红,朱恒文,丁	hai,Fang Yanhong,Zhu Hen	中国微生态学杂志	inese Journal of Microecolo	23	7	582-585,588	2011	-

3107	西食源性致病菌监测结果	Guangxi Foodborne Patho	红,吕素玲,黄彦,诸葛石养	Suling,Huang Yan,Zhuge S	应用预防医学	pplied preventive medicin	17	4	239-240	2011	n.1673-758X.2011.04.018
3108	见模化养鸭主要疫病流行性	major diseases in large-sca	地云,罗玲,王红琳,温国元,	ing,Wang Honglin,Wen Gu	湖北农业科学	Hubei Agricultural Sciences	50	23	4896-4899	2011	sn.0439-8114.2011.23.042
3109	成格隆沙门菌引起的食	of food poisoning caused b	金锁,黄秋洁,莫国华,何金	o,Huang Qiujie,Mo Guohu	浙江预防医学	hejiang Preventive Medicin	23	8	44-45	2011	sn.1007-0931.2011.08.018
3110	肉中肠炎沙门氏菌的分离	tion of Salmonella Enteriti	明,王辉,陈智,刘增再,郑	ng Hui,Chen Zhi,Liu Zengza	肉类研究	meat research	25	3	1-3	2011	sn.1001-8123.2011.03.001
3111	门氏菌、志贺氏菌分离鉴	eptibility test of Salmonell	胡秀彩,胡宏晓,吕爱军.	Xiucai,Hu Hongxiao,Lu Aij	中国畜牧兽医	nal husbandry and veterina	38	4	210-213	2011	-
3112	阳市食品中沙门菌污染调	nonella contamination in f	刘虎生,白丽娜.	Liu Husheng,Bai Lina.	环境与健康杂志	nal of Environment and He	-	9	768	2011	-
3113	广州市天河区食源性致病	e pathogenic bacteria in Ti	柳勤.	Liu Qin.	当代医学	contemporary medicine	17	34	157-158	2011	sn.1009-4393.2011.34.112
3114	医院污水中检出婴儿沙门	almonella infantis from ho	储黎娟,周品众.	Chu Lijuan,Zhou Pinzhong.	中国医药科学	Chinese Medical Sciences	1	17	174-174,187	2011	-
3115	死马肉导致139人食物中毒	horse meat139Analysis and	刘金兰.	Liu Jinlan.	医学动物防制	medical animal control	27	1	63	2011	sn.1003-6245.2011.01.031
3116	沙门菌耐药酶检测及药物	nd drug sensitivity analysis	郭宝俊,曹小燕,何昱苇.	Baojun,Cao Xiaoyan,He Yu	中华医院感染学杂志	ournal of Hospital Infectiou	-	20	4396-4398	2011	-
3117	人口食品大肠菌群和致病	Food Coliform and Pathog	邱烨.	Qiu Ye.	医学信息（下旬刊）	edical information (late issu	24	3	353	2011	-
3118	致沙门氏菌食物中毒的流	ysis of Salmonella food poi	高春玉,苏俊锋,王善雨.	hunyu,Su Junfeng,Wang Sh	中国卫生监督杂志	se Journal of Health Super	18	3	279-282	2011	sn.1007-6131.2011.03.019
3119	010年食源性致病菌监测	is of annual foodborne pat	杨柳青,宋克云,苏良.	gliuqing,Song Keyun,Su Lia	实用预防医学	ractical preventive medicin	18	5	859-860	2011	sn.1006-3110.2011.05.030
3120	西安地区鸡肉源沙门氏菌	aracteristics of Salmonella	玲,席美丽,张秀丽,崔生辉,	eili,Zhang Xiuli,Cui Shengh	食品科学	food science	32	19	130-136	2011	-
3121	5年～2009年熟肉制品微	vestigation on microbial c	振昱,范晓静,刘北星,钱婷	yu,Fan Xiaojing,Liu Beixing	江苏预防医学	iangsu Preventive Medicin	22	3	48-49	2011	sn.1006-9070.2011.03.024
3122	市婴幼儿腹泻病原学调查	on the etiology of infant d	杨富强,康俊辉.	Yang Fuqiang,Kang Junhui.	现代检验医学杂志	l of Modern Laboratory Me	26	6	14-16	2011	sn.1671-7414.2011.06.005
3123	校食物中毒的病原学检测	s of the etiology of food po	李月华,叶菊莲,韦俊超.	Yuehua,Ye Julian,Wei Junch	中国预防医学杂志	se Journal of Preventive Me	12	3	290-291	2011	-
3124	老院肠炎沙门氏菌食物中	ella Enteritidis food poison	季洪斌.	Ji Hongbin.	中国初级卫生保健	primary health care in china	25	9	70-71	2011	n.1001-568X.2011.09.038
3125	型副伤寒沙门菌引起的食	ning caused by Salmonella	魏清文,张伟雄.	ei Qingwen,Zhang Weixion	中国职业医学	inese Occupational Medici	-	S1	88-89	2011	-
3126	院儿童沙门菌感染监测分	of salmonella infection in	石海丽,蒋武.	Shi Haili,Jiang Wu.	中外健康文摘	nese and Foreign Health Di	8	30	168-169	2011	sn.1672-5085.2011.30.163
3127	品中耐辐照沙门氏菌的筛	ation of radiation-resistant	王,张艺,吴军,潘俊,邵明玉	hang Yi,Wu Jun,Pan Jun,Sha	中国酿造	China Brewing	-	6	48-50	2011	-
3128	东省2008-2009年沙门菌监	nce2008-2009annual salm	柏生,谭海玲,何冬梅,刘美	n Hailing,He Dongmei,Liu M	中华流行病学杂志	inese Journal of Epidemiol	32	8	789-792	2011	sn.0254-6450.2011.08.012
3129	区儿童感染性腹泻病原学	rug resistance of infectious	董方,刘锡清,许宏,宋文琪	ng,Liu Xiqing,Xu Hong,Song	中国小儿急救医学	tric Emergency Medicine in	18	1	33-35	2011	sn.1673-4912.2011.01.012
3130	集体食物中毒事件的调查	nalysis of a collective food	徐里强.	Xu Liqiang.	中国民康医学	nese People's Health Medi	23	18	2324	2011	sn.1672-0369.2011.18.079
3131	从业人员沙门氏菌携带状	ug resistance spectrum am	马智龙.	Ma Zhilong.	医学动物防制	medical animal control	27	8	729-732	2011	sn.1003-6245.2011.08.017
3132	州市非发酵性豆制品的卫	status of non-fermented s	黄启红,李光宪,曾泳而.	Qihong,Li Guangxian,Zeng	商品与质量（学术观察）	y and quality (academic ob	-	2	260	2011	-
3133	感染性肠炎的病原检测结	esults and analysis of acute	涂银萍,王晶,王久伶.	Yinping,Wang Jing,Wang Jiu	中国现代医学杂志	ese Journal of Modern Med	21	21	2608-2610	2011	sn.1005-8982.2011.21.012
3134	区食品中沙门菌的监测与分	alysis of Salmonella in foo	柳勤,黄燕,张惠文.	Qin,Huang Yan,Zhang Huiw	中国医药指南	Chinese Medicine Guide	9	33	373-374	2011	sn.1671-8194.2011.33.293
3135	基在健康人群中沙门菌检	media for detection of Sa	,宋驰萍,骆玲飞,王海青,欧	hiping,Luo Lingfei,Wang Ha	中国实用医药	Chinese Practical Medicine	6	31	275-276	2011	sn.1673-7555.2011.31.236
3136	童血培养病原菌的分布及	pathogenic bacteria cultur	林,陈超群,张秋桂,李晓丽	hen Chaoqun,Zhang Qiugu	中华医院感染学杂志	ournal of Hospital Infectiou	-	19	4160-4162	2011	-
3137	沙门菌引起的感染性腹泻	infectious diarrhea case ca	,刘超梅,王民,李仕英,杜	Liu Chaomei,Wang Min,Li S	解放军医药杂志	s Liberation Army Medical	23	2	65-66	2011	n.2095-140X.2011.02.034
3138	省食源性沙门菌污染监测	Foodborne Salmonella Con	军,刘桂华,黄鑫,孔祥云,杨	uihua,Huang Xin,Kong Xian	中国卫生工程学	Chinese sanitary engineerin	10	1	58-59	2011	-
3139	年北京市沙门菌流行特征	stics and molecular typing	荣,崔志刚,严寒秋,刘元,	higang,severe cold autumn	中华预防医学杂志	se Journal of Preventive Me	45	2	113-117	2011	sn.0253-9624.2011.02.004
3140	林沙门菌引起泌尿道感染	lin causes urinary tract infe	徐涛,陈玉莲.	Xu Tao,Chen Yulian.	国际检验医学杂志	onal journal of laboratory	32	17	2042-2042	2011	sn.1673-4130.2011.17.067
3141	鼠伤寒沙门菌性肠炎患儿	treatment of children with	李云送,陈瑶.	Li Yunsong,Chen Yao.	医药前沿	Medical Frontier	1	21	30-32	2011	sn.2095-1752.2011.21.022
3142	10年江门市腹泻病原菌现	rrent Situation of Diarrhea	敏嘉,吴瑞英,蓝秀健,陈省	Wu Ruiying,Lan Xiujian,Che	热带医学杂志	ournal of tropical medicine	11	7	826-827	2011	-
3143	寒沙门菌引起食物中毒的	e of food poisoning caused	王均华,高海英.	Wang Junhua,Gao Haiying.	中国现代医生	modern chinese doctor	49	30	5,8	2011	sn.1673-9701.2011.30.003

3144	计我国食源性非伤寒沙门	non-typhoid salmonellosis	毛雪丹,胡俊峰,刘秀梅.	Xuedan,Hu Junfeng,Liu Xiu	中华疾病控制杂志	hese Journal of Disease Cor	15	7	622-625	2011	-
3145	食服务从业人员沙门菌监	results among catering ser	帅慧群,商晓春,张睿,赵雪琴	Shang Xiaochun,Zhang Rui	浙江预防医学	hejiang Preventive Medicin	23	12	57-58,73	2011	sn.1007-0931.2011.12.023
3146	地食堂引起沙门氏菌食物	ella food poisoning caused	季忠华.	Ji Zhonghua.	健康必读（下旬刊）	ust-read for health (late issu	-	8	393369	2011	-
3147	株中分离出肠沙门菌维尔	a enterica serotype Virchov	王利梅.	Wang Limei.	疾病监测与控制	sease surveillance and cont	5	11	667670	2011	-
3148	人感染猪霍乱沙门氏菌败	fection with choleraesuis, S	周正银,谢明映.	hou Zhengyin,Xie Mingying	广西医科大学学报	al of Guangxi Medical Univ	28	1	164-164	2011	n.1005-930X.2011.01.063
3149	市江干区食品中微生物污	mination status in food in	珍,方叶珍,邵景莺,黄世旺,金	Yezhen,Shao Jingying,Huar	中国预防医学杂志	se Journal of Preventive Me	12	7	610-613	2011	-
3150	猪霍乱沙门氏菌的分离鉴定	ptibility testing of Salmone	海棠,张志鹏,赵彩丽,申红	Zhang Zhipeng,Zhao Caili,Sh	河南农业科学	Henan Agricultural Sciences	40	4	146-148	2011	sn.1004-3268.2011.04.038
3151	区2010年腹泻病原菌耐药性	and analysis of drug resista	,吴静怡,李明,王黎,刘然,刘	ngyi,Li Ming,Wang Li,Liu Ra	传染病信息	fectious disease informatio	24	2	85-87	2011	sn.1007-8134.2011.02.007
3152	北京市丰台区食源性致病	borne pathogenic bacteria	燕燕,崔宝荣,封会茹,冯月	Cui Baorong,Feng Huiru,Fe	首都公共卫生	capital public health	5	4	161-164	2011	sn.1673-7830.2011.04.008
3153	阳市食品中沙门菌污染调查	almonella contamination in	刘虎生,白丽娜.	Liu Husheng,Bai Lina.	环境与健康杂志	rnal of Environment and He	28	9	768-768	2011	-
3154	源性沙门菌血清型分布及耐	d drug resistance of foodbo	岚,贾华云,张红,张林青,胡	uayun,Zhang Hong,Zhang L	实用预防医学	ractical preventive medicin	18	6	994-997	2011	sn.1006-3110.2011.06.007
3155	沙门氏菌食物中毒的调查	nalysis of a case of salmon	李红萍,邵志洪.	Li Hongping,Shao Zhihong.	中外健康文摘	hese and Foreign Health Di	8	32	50-51	2011	sn.1672-5085.2011.32.041
3156	沙门氏菌和奇异变形杆菌	d Proteus mirabilis class I in	进玲,杨保伟,席美丽,孟江	Yang Baowei,Xi Meili,Men	食品科学	food science	32	9	130-134	2011	-
3157	伤寒沙门菌感染聚集性发生	currence of Salmonella typ	姚新宝,丁清,易洪仪.	o Xinbao,Ding Qing,Yi Hong	中国感染控制杂志	ese Journal of Infection Co	10	1	57-58	2011	sn.1671-9638.2011.01.017
3158	州市惠阳区超市凉拌菜卫生	old dishes in supermarkets	杨维占,陶玉芬.	Yang Weizhan,Tao Yufen.	海峡预防医学杂志	s Journal of Preventive Me	17	1	66-67	2011	-
3159	部脓汁分离都柏林沙门菌	almonella Dublin from foot	红,周薇薇,王勇,郭宇航,张	u Weiwei,Wang Yong,Guo	中华医院感染学杂志	ournal of Hospital Infectiou	-	17	3538	2011	-
3160	群链球菌合并感染致孕妇	abscess in a pregnant wom	李佳俊,李国钢,周扬霄.	ajun,Li Guogang,Zhou Yang	中华传染病杂志	se Journal of Infectious Dis	29	12	758	2011	sn.1000-6680.2011.12.013
3161	沙门氏菌的健康带菌调查	sceptibility test of Salmone	储倩,朱晓霞,岳华,汤承.	n,Zhu Xiaoxia,Yuehua,Tang	四川畜牧兽医	nal Husbandry and Veterina	38	1	24-25,28	2011	sn.1001-8964.2011.01.011
3162	区腹泻患者沙门菌亚型分	n and drug resistance in pat	博,郭桐生,王欢,陈素明,,B	gsheng,Wang Huan,Chen Su	中国卫生检验杂志	ese Journal of Health Inspe	21	12	2937-2939	2011	-
3163	起因水源污染导致的伤寒暴	break caused by water sc	生;朱保平;马会来;陈福辉	oping;The horse will com	疾病监测	disease surveillance	26	7	579-581	2011	-
3164	校内伤寒暴发疫情的调查	alysis of a typhoid fever	邱国生;	Qiu Guosheng;	中外医学研究	se and foreign medical re	9	19	54-56	2011	33/j.cnki.cfmr.2011.19.009
3165	甲型副伤寒暴发疫情调查	nalysis of an outbreak of	韦克波;	wikepo;	医学动物防制	medical animal control	27	6	547-548	2011	-
3166	县1起伤寒暴发疫情的流行	Epidemiological analysis	;李朝霞;王立杰;卡日曼;李	aoxia;Wang Lijie;Karzma	职业与健康	Occupation and health	27	11	1288-1289	2011	9/j.cnki.zyyjk.2011.11.028
3167	市一起甲型副伤寒暴发疫情	break of paratyphoid feve	顾金军;	Gu Jinjun;	江苏卫生保健	Jiangsu Health Care	13	3	19-20	2011	-
3168	型副伤寒暴发疫情的调查	atment of an outbreak of	吴志明;王月萍;	Yu Zhiming;Wang Yuepin	江苏预防医学	angsu Preventive Medici	22	2	22-23	2011	-
3169	病合并沙门氏菌感染6例临	th salmonella infection6Ca	陶鹏飞,杨欣平,李惠琴.	Pengfei,Yang Xinping,Li Hu	中国医师进修杂志	hysician Continuing Educati	35	z1	152-153	2012	-
3170	菌致感染性心内膜炎一例	ditis caused by Salmonella	王磊利,沈定霞,叶丽艳.	ng Leili,Shen Dingxia,Ye Liy	中华检验医学杂志	se Journal of Laboratory Me	35	12	1195-1196	2012	sn.1009-9158.2012.12.033
3171	有毒牛肝菌引起食物中毒的	e of food poisoning cause	良伟,刘伦光,张丽杰,施国庆	iu Luguang,Zhang Lijie,Shi	中华流行病学杂志	inese Journal of Epidemiolo	33	12	1261-1264	2012	sn.0254-6450.2012.12.014
3172	伤寒流行现况、菌株耐药	typhoid epidemic status, ba	唐震,董晨,周璐,钱慧敏,汤	en,Dong Chen,Zhou Lu,Qia	中华流行病学杂志	inese Journal of Epidemiolo	33	12	1269-1272	2012	sn.0254-6450.2012.12.016
3173	髋关节伤寒沙门菌感染一例	almonella typhoid infection	陈东阳,史冬泉,戴进,秦江	yang,Shi Dongquan,Dai Jin,	中华外科杂志	Chinese Journal of Surgery	50	11	1053-1054	2012	sn.0529-5815.2012.11.027
3174	沙门菌引起的食物中毒的调	is of a case of food poisoni	李宏.	Li Hong.	医药前沿	Medical Frontier	-	33	359-360	2012	-
3175	株沙门菌、21株志贺菌检测	nella,twenty oneAnalysis of	吕芝平,王艳,周丽萍,张晋	ng,Wang Yan,Zhou Liping,Z	医药前沿	Medical Frontier	-	31	334	2012	-
3176	甲型副伤寒沙门菌流行菌株	revalent strains of Salmone	草,葛玉梅,俞骅,郑伟,张蔚	mei,Yu Hua,Zheng Wei,Zha	中华微生物学和免疫学杂志	urnal of Microbiology and Im	32	8	711-715	2012	sn.0254-5101.2012.08.009
3177	2010年感染性腹泻病原菌	e of pathogenic bacteria in	许磊,曾德唯,甘忠志,邓辉	eng Dewei,Gan Zhongzhi,D	重庆医学	Chongqing Medicine	41	23	2402-2404	2012	sn.1671-8348.2012.23.023
3178	1年市售食品食源性致病菌	sis of foodborne pathogeni	张帆.	Zhang Fan.	中国热带医学	Chinese tropical medicine	12	10	1270-1272	2012	-
3179	腹泻患者病原菌检测结果	ic bacteria detection result	王久伶,王晶,涂银萍.	ng Jiuling,Wang Jing,Tu Yinp	北京医学	Beijing Medicine	34	6	479-481	2012	-
3180	幼儿下呼吸道感染常见病原	wer respiratory tract infec	明晓波,丁刚,周翔,黄和明,谢	,Ding Gang,Zhou Xiang,Hua	海南医学	Hainan Medicine	23	21	14-17	2012	sn.1003-6350.2012.21.006

3181	的血清型、生物被膜形成能	ning ability and drug resista	素娟,张华,张晓平,田艳娜	hang Hua,Zhang Xiaoping,T	畜牧兽医学报	mal Husbandry and Veterin	43	10	1630-1638	2012	-
3182	D群沙门氏菌引起食物中	o cases of food poisoning c	王代良,吴娴,陈冲,李秀英	Jiang,Wu Xian,Chen Chong,	中国公共卫生管理	ese public health managen	28	3	343-345	2012	-
3183	败血症猪霍乱沙门菌感染的	ella choleraesuis infection i	黄凯,段荣,余晓君.	ang Kai,Duan Rong,Yu Xiaoj	实验与检验医学	mental and Laboratory Me	30	5	466-467	2012	sn.1674-1129.2012.05.017
3184	由沙门菌引起的食物中毒的	case of food poisoning cau	黄燕,何立华,彭玉金.	uang Yan,He Lihua,Peng Yuj	江西医药	Jiangxi Medicine	47	4	360364	2012	sn.1006-2238.2012.04.037
3185	冲场凝胶电泳分子分型在	field gel electrophoresis m	王晨,袁巧,邓小玲.	g Chen,Yuan Qiao,Deng Xia	国际流行病学传染病学杂志	rnal of Epidemiology and In	39	3	212-216	2012	sn.1673-4149.2012.03.019
3186	011年生食蔬菜中病原菌	alysis of pathogenic bacter	漓漓,杨小养,康富俊,李红	,Yang Xiaoyang,Kang Fujun	中国当代医药	inese contemporary medic	19	36	142-143	2012	sn.1674-4721.2012.36.074
3187	点污染引起食物中毒事件	poisoning incident caused	庞洁,施向东,龙兮,梁惠宁	Shi Xiangdong,Long Xi,Liang	医学动物防制	medical animal control	28	8	926928	2012	sn.1003-6245.2012.08.038
3188	主动脉瘤患者血中分离出1	s with descending aortic ps	刘美清,林玮,赵松,朱晓征	ng,Lin Wei,Zhao Song,Zhu X	检验医学与临床	boratory medicine and clini	9	12	1525-1526	2012	sn.1672-9455.2012.12.074
3189	门氏菌的分离鉴定及药敏	and drug susceptibility tes	龚大春,齐海涛.	Gong Dachun,Qi Haitao.	工大学学报（自科版）农学	(Autonomous Science Edit	9	8	23-24	2012	1673-1409(S).2012.08.008
3190	11年北海市海产品污染状	d pollution status in Beihai	黄坚,包贤艳.	Huang Jian,Bao Xianyan.	职业与健康	Occupation and health	28	19	2370-2371	2012	-
3191	区健康肉鸡携带沙门氏菌	lmonella carried by healthy	,宁宜宝,宋立,陈惠娟,习砚	g Yibao,Song Li,Chen Huiju	中国兽药杂志	ese Journal of Veterinary D	46	10	9-12	2012	-
3192	市部分伤寒副伤寒沙门菌	rug resistance of selected S	陈敏,张曦,陈洪友,席曼芳	,Zhang Xi,Chen Hongyou,X	中国卫生检验杂志	ese Journal of Health Inspe	22	2	285-286,289	2012	-
3193	培养病原菌菌群分布的变	s and drug resistance of pat	侯德凤,刘海燕,姜友珍.	Defeng,Liu Haiyan,Jiang You	临床合理用药杂志	l of Rational Clinical Use of	5	7	100-102	2012	sn.1674-3296.2012.07.083
3194	道病原微生物与粪便污染	ic microorganisms and fec	张崇淼,王晓昌,周进宏,吉铮	ao,Wang Xiaochang,Zhou J	环境科学学报	rnal of Environmental Scie	32	11	2789-2794	2012	-
3195	镇晨光村一起食物中毒调	se of food poisoning in Che	甘武丰,刘会芸.	Gan Wufeng,Liu Huiyun.	健康大视野	Healthy vision	20	7	274-274	2012	-
3196	145株沙门氏菌耐药性分析	of drug resistance of Salmo	罗琦,董柯,赵瑞臻,张月泉	ong Ke,Zhao Ruizhen,Zhang	河南预防医学杂志	n Journal of Preventive Me	23	4	256-258,261	2012	-
3197	门菌的监测及其脉冲场凝	ectrophoresis typing of Salm	,王燕梅,沈赟,符晓梅,甄世	Yanmei,Shen Yun,Fu Xiaom	中国卫生检验杂志	ese Journal of Health Inspe	22	3	514-516	2012	-
3198	饮业现榨果蔬汁微生物污染	shly squeezed fruit and veg	李艳灵.	Li Yanling.	中国卫生产业	China health industry	-	6	118	2012	-
3199	年广州市番禺区水产品污染	situation of aquatic produc	明,吴利楠,麦洁梅,张琪,杨	Wu Linan,Mai Jiemei,Zhang	热带医学杂志	journal of tropical medicine	12	9	1150-1153	2012	-
3200	病患者血培养病原菌分布	eria distribution results fro	谢宁.	Xie Ning.	海南医学	Hainan Medicine	23	9	89-91	2012	sn.1003-6350.2012.09.039
3201	服务从业人员沙门菌监测	e and analysis among cater	丽清,阮海花,涂基恒,杨振	,Ruan Haihua,Tu Jiheng,Yan	贵州医药	Guizhou Medicine	36	2	171-172	2012	N.1000-744X.2012.02.034
3202	患病动物源沙门菌的多重耐	sistance in ciprofloxacin-re	李健,任艳娜,汪天露,孙永学	en Yanna,Wang Tianlu,Sun	中国兽医科学	Chinese Veterinary Science	42	7	747-752	2012	-
3203	年宁夏地区沙门氏菌分布	distribution characteristic	郭邦成,闫立群,谢明英,梁俊	cheng,Yan Liqun,Xie Mingy	中国人兽共患病学报	Chinese Journal of Zoonose	28	5	506-508	2012	sn.1002-2694.2012.05.025
3204	血培养检出婴儿沙门菌1例	antis detected in blood cul	厉世笑,许春燕,沈定树.	ixiao,Xu Chunyan,Shen Ding	临床检验杂志	journal of clinical testing	30	9	720	2012	-
3205	例儿童腹泻病原学检测结果	gical detection results of c	康俊辉.	Kang Junhui.	国际检验医学杂志	onal journal of laboratory r	33	5	539-540,542	2012	sn.1673-4130.2012.05.013
3206	1年广西食品中沙门菌监	ce and analysis of Salmone	振柱,刘展华,李秀桂,黄立	hu,Liu Zhanhua,Li Xiugui,H	实用预防医学	ractical preventive medicin	19	12	1817-1820	2012	sn.1006-3110.2012.12.019
3207	区食品中食源性致病菌污	dborne pathogenic bacteria	章迎春,陆永梅,张雁.	Yingchun,Lu Yongmei,Zhan	中国卫生检验杂志	ese Journal of Health Inspe	22	8	1921-1923	2012	-
3208	1年永州市食源性致病菌监	oodborne pathogenic bact	陈蓉,唐旭辉.	Chen Rong,Tang Xuhui.	实用预防医学	ractical preventive medicin	19	7	1032-1034	2012	sn.1006-3110.2012.07.026
3209	0年中国鼠伤寒沙门菌分子	yping analysis of Salmonella	王淑京,娄静,刁保卫,阚飙	ing,Lou Jing,Diao Baobao,K	中国人兽共患病学报	Chinese Journal of Zoonose	28	12	1161-1166	2012	sn.1002-2694.2012.12.002
3210	学校一起疑似食源性疾病发	spected food-borne diseas	荣卿,白文河,李焕军,索凌	Bai Wen River,Li Huanjun,S	疾病预防控制通报	se Prevention and Control I	-	6	82-83	2012	-
3211	D群沙门氏菌引起食物中	on cases of food poisoning	王代良,吴娴,陈冲,李秀英	Jiang,Wu Xian,Chen Chong,	中国公共卫生管理	ese public health managen	-	3	343-345	2012	-
3212	污染致细菌感染性腹泻暴发	rial infectious diarrhea cau	张斌,靳桂才,林建维.	ang Bin,Jin Guicai,Lin Jianw	包头医学院学报	rnal of Baotou Medical Coll	28	1	26-28	2012	n.1006-740X.2012.01.012
3213	副伤寒沙门氏菌引起食物	poisoning caused by Salmo	戴婧.	Dai Jing.	青海医药杂志	Qinghai Medical Journal	42	7	71-72	2012	-
3214	生从业人员沙门菌带菌状	influencing factors among	邓志新,施咏凤.	Deng Zhixin,Shi Yongfeng.	江苏卫生保健	Jiangsu Health Care	14	2	27-28	2012	sn.1008-7338.2012.02.022
3215	集体聚餐和食物中毒现况	tuation of collective dining	高文东,胡小虎.	Gao Wendong,Hu Xiaohu.	健康必读（中旬刊）	-read for health (mid-term	11	12	83-84	2012	-
3216	门峡市食品中食源性致病菌	athogenic bacteria contami	赵建明,杨洁,郭琳娜.	o Jianming,Yang Jie,Guo Lin	河南预防医学杂志	n Journal of Preventive Me	23	3	191-193	2012	-
3217	品中食源性致病菌污染状况	odborne pathogenic bacter	郑成翠,刘桂华.	Zheng Chengcui,Liu Guihua	中国卫生工程学	Chinese sanitary engineerin	11	1	52-53	2012	-

3218	边市场部分市售食品的微	ercially available foods in r	陈朝琼.	Chen Chaoqiong.	中国农学通报	na Agricultural Science Bull	28	21	283-287	2012	sn.1000-6850.2012.21.051
3219	食食品中微生物污染状况	ysis of microbial contamina	,刘晓娟,郑秀清,赵丽萍,李	aojuan,Zheng Xiuqing,Zhao	医学动物防制	medical animal control	28	8	886-888	2012	sn.1003-6245.2012.08.021
3220	少门菌致1起食物中毒调查	ella1food poisoning invest	兰,周泽君,吴娴,朱毅,陈华	ou Zejun,Wu Xian,Zhu Yi,C	预防医学情报杂志	of Preventive Medicine Info	28	4	298-300	2012	-
3221	物中毒的病原学鉴定及药敏	lmonella food poisoning ar	黄永亮,张宏雅.	ang Yongliang,Zhang Hong	实用医学杂志	ournal of Practical Medicin	28	1	143-145	2012	sn.1006-5725.2012.01.062
3222	国部分地区鸡沙门氏菌的	monella chicken in some ar	张荣武.	Zhang Rongwu.	山东畜牧兽医	mal Husbandry and Veteri	33	5	83-84	2012	sn.1007-1733.2012.05.055
3223	少门菌PFGE分子分型和耐	GEStudy on the correlation	陈建辉,林杰,郑金凤,严延生	ui,Lin Jie,Zheng Jinfeng,Yan	中国人兽共患病学报	Chinese Journal of Zoonose	28	1	10-13	2012	sn.1002-2694.2012.01.003
3224	电泳检测一起汤卜逊沙门	is detects a case of Salmon	月华,叶菊莲,韦俊超,王雅	,Ye Julian,Wei Junchao,Wa	中国卫生检验杂志	ese Journal of Health Inspe	22	5	1170-1172	2012	-
3225	韦太夫雷登沙门菌引起的	poisoning caused by Salmor	刘永爱.	Liu Yongai.	中外健康文摘	nese and Foreign Health Di	9	14	199-200	2012	sn.1672-5085.2012.14.174
3226	东省食源性沙门菌血清分	ptibility analysis of foodbo	邵坤,关冰,侯配斌,张华宁	Guan Bing,Hou Peibin,Zhan	中国食品卫生杂志	hinese Food Hygiene Journ	24	1	9-13	2012	-
3227	市食品污染物中致病菌污	of pathogenic bacteria in f	吴凤云,张连娟,张博.	engyun,Zhang Lianjuan,Zha	疾病监测与控制	ease surveillance and cont	6	3	145-146	2012	-
3228	起沙门氏菌食物中毒调查分	nalysis of a case of salmon	李蓓,魏彩梅.	Li Bei,Wei Caimei.	疾病监测与控制	ease surveillance and cont	6	7	414-415	2012	-
3229	季度全国食物中毒致50人	arter, food poisoning cause	-	-	中国食品学报	urnal of Chinese Food Scien	-	10	15	2012	-
3230	1年桂林市零售食品中沙	eillance of Salmonella in ret	小养,康富俊,李红梅,王春	g,Kang Fujun,Li Hongmei,W	华夏医学	Chinese Medicine	25	3	301-303	2012	sn.1008-2409.2012.03.003
3231	菌感染性腹泻病原学监测	lance results of community	王辉,马焕丽,邱燕飞.	ang Hui,Ma Huanli,Qiu Yan	国际检验医学杂志	onal journal of laboratory	33	19	2404-2406	2012	sn.1673-4130.2012.19.057
3232	法在从业人员肠道致病菌	f methods in screening of i	钟学飘,朱淑萍,梁高洲,蔡	iao,Zhu Shuping,Liang Gao	中外健康文摘	nese and Foreign Health Di	-	48	97-98	2012	sn.1672-5085.2012.48.080
3233	年株洲市食源性致病菌监	ysis of foodborne pathogen	方英,胡卓,钟艳,杨林飞.	ng,Hu Zhuo,Zhong Yan,Yan	河南预防医学杂志	n Journal of Preventive Me	23	2	139-141	2012	-
3234	行业从业人员肠道致病菌	l pathogenic bacteria amo	,陈磊,盛丽,孙成铭,初永丽	ei,Shengli,Sun Chengming,	中国卫生检验杂志	ese Journal of Health Inspe	22	7	1653-1657	2012	-
3235	少门氏菌引起的食物中毒	is of a case of food poisoni	朱艳梅,汤艳.	Zhu Yanmei,Tang Yan.	中外健康文摘	nese and Foreign Health Di	-	34	80-81	2012	sn.1672-5085.2012.34.061
3236	东省沙门菌监测及其病原	ngdong Province and analy	,柯昌文,梁兆铭,谭海玲,李	wen,Liang Zhaoming,Tan H	中华预防医学杂志	se Journal of Preventive Me	46	5	424-429	2012	sn.0253-9624.2012.05.010
3237	区血流感染菌群调查及耐	analysis of bloodstream i	张悦英,蔡洁新,林淳,朱红军	ying,Cai Jiexin,Lin Chun,Zhu	医学检验与临床	edical Laboratory and Clini	23	4	34-36	2012	sn.1673-5013.2012.04.014
3238	办宴席引起食物中毒事件	oisoning incidents caused b	万霞,龚文胜,张金枝,罗敬	Gong Wensheng,Zhang Jinz	中国初级卫生保健	primary health care in china	26	4	63-64	2012	n.1001-568X.2012.04.025
3239	活体样本中沙门菌的药物	s of Salmonella in chicken	符晓梅,沈赟,乔昕,王燕梅	ei,Shen Yun,Qiao Xin,Wang	江苏卫生保健	Jiangsu Health Care	14	2	7,43	2012	sn.1008-7338.2012.02.005
3240	菌和粪肠球菌混合感染致	infection with Salmonella t	瞿如波,张昊,孙跃岭,邱广斌	Zhang Hao,Sun Yueling,Qiu	验和临床感染病杂志（电	ntal and Clinical Infectious	6	6	624	2012	sn.1674-1358.2012.06.030
3241	HINET血流感染的病原菌	d drug resistance of pathog	,陈中举,贾蓓,黄文祥,卓超	Jia Bei,Huang Wenxiang,Ex	中国感染与化疗杂志	urnal of Infection and Che	12	4	251-258	2012	sn.1009-7708.2012.04.002
3242	门氏菌污染状况和血清型	tribution and drug resistan	素玲,韦程媛,姚雪婷,李秀	Wei Chengyuan,Yao Xueting	应用预防医学	applied preventive medicin	18	3	137-141,170	2012	n.1673-758X.2012.03.004
3243	环介导等温扩增(LAMP)	monella in animal feed(LA	孙园园,赵鹏.	Sun Yuanyuan,Zhao Peng.	中国畜牧兽医	mal husbandry and veterina	39	1	32-36	2012	sn.1671-7236.2012.01.008
3244	肠炎沙门菌食物中毒检测	ort of Salmonella Enteritidis	潘新明.	Pan Xinming.	海峡预防医学杂志	s Journal of Preventive Me	18	4	44-45	2012	-
3245	0-2011年食源性致病菌监	nalysis of annual foodborne	马妮,赵虹,张旭,马景宏.	hao Hong,Zhang Xu,Ma Jin	中国公共卫生管理	nese public health managen	28	1	46-48	2012	-
3246	染性腹泻致病菌的培养结	athogenic bacteria causing	声,张丽华,林偲思,张丽,朱	,Zhang Lihua,Lin Sisi,Zhang	中国医药	Chinese Medicine	7	8	992-994	2012	sn.1673-4777.2012.08.032
3247	少门菌的分离鉴定及其药敏	nd drug susceptibility analy	徐伟红,陈士红.	Xu Weihong,Chen Shihong.	国际检验医学杂志	onal journal of laboratory	33	1	126-127	2012	sn.1673-4130.2012.01.064
3248	011年莱州市腹泻症候群	on pathogens of diarrhea s	刘倩,李金萍,尹海英,寇增	ian,Li Jinping,Yin Haiying,K	预防医学论坛	Preventive Medicine Forum	18	6	457-458	2012	-
3249	品安全风险监测食源性致	detection results from food	,邓赞民,黄英哲,覃善玲,黄	min,Huang Yingzhe,Tan Sh	中国医药实践杂志	nese Journal of Medical Pra	-	3	34	2012	-
3250	年临床送检鸡关节炎病例	port on arthritis cases in ch	东,徐家芳,韦平,王海源,刘	Jiafang,Wei Ping,Wang Hai	中国家禽	Chinese poultry	34	22	57-58	2012	sn.1004-6364.2012.22.018
3251	肉中五种食源性致病菌污	of five foodborne pathogen	谭艾娟,吕世明,金志强,方英	n,Lu Shiming,Jin Zhiqiang,F	山地农业生物学报	l of Mountain Agricultural	31	1	91-94	2012	sn.1008-0457.2012.01.021
3252	患者粪便细菌培养及临床	e and clinical characteristic	,王晓才,王蕻馨,王作梅,黄	ai,Wang Hongxin,Wang Zu	中国卫生检验杂志	ese Journal of Health Inspe	22	3	559-561	2012	-
3253	江苏部分地区鸡沙门氏菌	lla infection in chickens in s	龚建森,刘加圣,刘学贤.	Jiansen,Liu Jiasheng,Liu Xu	中国家禽	Chinese poultry	34	3	62-63	2012	sn.1004-6364.2012.03.019
3254	市饮食服务从业人员携带沙	monella carried by catering	林勤.	Lin Qin.	临床合理用药杂志	l of Rational Clinical Use of	5	33	20-21	2012	sn.1674-3296.2012.33.015

3255	血培养同时检出伤寒沙门	ected simultaneously in bo	陈名霞.	Chen Mingxia.	医学检验与临床	edical Laboratory and Clini	23	3	93-93	2012	sn.1673-5013.2012.03.044
3256	年福建省伤寒、副伤寒流	acteristics of typhoid fever	,陈爱平,谢芳钦,陈建辉,林	Aiping,Xie Fangqin,Chen Ji	预防医学论坛	Preventive Medicine Forum	18	5	341-343	2012	-
3257	齐菲尔德沙门菌食物中毒	case of Salmonella Lichfiel	张欣强,邓志爱,侯水平,胡	Xinqiang,Deng Zhiai,Hou I	中国热带医学	Chinese tropical medicine	12	6	684-687	2012	-
3258	童腹泻病原学特点分析与	drug resistance monitorin	王亚平,任振平,高志玲.	Yaping,Ren Zhenping,Gao Z	中国卫生产业	China health industry	-	14	145	2012	-
3259	市熟肉制品微生物污染状	tion status of cooked meat	谊,张健,刘俊华,肖丽红,罗	g Jian,Liu Junhua,Xiao Liho	医学动物防制	medical animal control	28	3	274-275	2012	sn.1003-6245.2012.03.018
3260	儿童腹泻130例临床分析	in children130Case clinical	岑小玲.	Cen Xiaoling.	中外医学研究	ese and foreign medical res	10	18	127-128	2012	sn.1674-6805.2012.18.096
3261	沙门氏菌检测及洁蛋对蛋	ction and research on the i	彦宇辉,刘燕荣,袁正东,杨宁	Liu Yanrong,Yuan Zhengdo	食品安全质量检测学报	f Food Safety and Quality I	3	5	475-480	2012	-
3262	鼠伤寒沙门菌多重耐药和	esistance and molecular typ	刘桂荣,李爽,贾蕾,李锡太	iu Guirong,Li Shuang,Jia Le	华临床医师杂志（电子版	urnal of Clinicians (electror	6	15	4477-4479	2012	sn.1674-0785.2012.15.075
3263	地区某鸡场鸡白痢的分离	pullorum from a chicken f	徐宁.	Xu Ning.	现代畜牧兽医	mal husbandry and veterina	-	9	33-35	2012	sn.1672-9692.2012.09.024
3264	寒沙门菌引起食物中毒的	a case of food poisoning ca	邓丽芳,王雪梅,许立新.	g Lifang,Wang Xuemei,Xu L	海峡预防医学杂志	s Journal of Preventive Me	18	1	55-56	2012	-
3265	门菌引起的食物中毒病原	of food poisoning caused	罗芸,叶菊莲,李琳,吴爱萍	Yun,Ye Julian,Li Lin,Wu Ai	中国卫生检验杂志	ese Journal of Health Inspe	22	5	1060-1062	2012	-
3266	市熟肉制品致病菌监测结	of pathogenic bacteria in c	方,贺连华,石晓路,陈妙玲,林	ianhua,Shi Xiaolu,Chen Mi	中国卫生检验杂志	ese Journal of Health Inspe	22	8	1916-1917	2012	-
3267	食物中毒事故流行病学调	al investigation of a late-re	沈广顺.	Shen Guangshun.	中国城乡企业卫生	ygiene in urban and rural a	27	4	105-106	2012	-
3268	省鸡沙门氏菌病流行病学	gation of chicken salmonel	,王申锋,许兰菊,朱金凤,孙	henfeng,Xu Lanju,Zhu Jinf	中国家禽	Chinese poultry	34	6	64-66	2012	sn.1004-6364.2012.06.019
3269	1年腹泻病症候群病原菌	urveillance results of diarrh	锦平,姜晓丽,范广智,董惠	ng Xiaoli,Fan Guangzhi,Do	宁夏医学杂志	Ningxia Medical Journal	34	7	672-673	2012	sn.1001-5949.2012.07.039
3270	殖场沙门氏菌药敏试验结	rug susceptibility test resu	冬娥,苏建晖,陈冠武,林宁	e,Su Jianhui,Chen Guanwu,	养殖技术顾问	reeding technical consultar	-	9	238-239	2012	sn.1673-1921.2012.09.226
3271	沙门菌多重耐药性及流行	stance and epidemiological	荣,李洁,贾蕾,李锡太,黎新	g,Li Jie,Jia Lei,Li Xitai,Li Xiny	中国热带医学	Chinese tropical medicine	12	3	309-311	2012	-
3272	2年-2011年沙门菌血清型	11Salmonella serotype and	,张鞠玲,王欢,陈素明,张成	,Wang Huan,Chen Suming,	中国卫生检验杂志	ese Journal of Health Inspe	22	9	2217-2219	2012	-
3273	大肠杆菌病病原的分离鉴	ction of colibacillosis patho	胡世君,郑凯,罗杰,张成建	n,Zheng Kai,Roger,Zhang C	黑龙江畜牧兽医（上半月）	ndry and Veterinary Medic	-	3	105-107	2012	-
3274	年食品中食源性致病菌污	ontamination status of foo	贤艳,黄坚,白海涛,李师鹏	yan,Huang Jian,Bai Haitao,	海峡预防医学杂志	s Journal of Preventive Me	18	3	60-61	2012	-
3275	年第2季度我院细菌的分析	rly analysis report of bacte	刘志雪.	Liu Zhixue.	中国社区医师（医学专业）	munity physician (medical	14	10	34	2012	sn.1007-614x.2012.10.029
3276	检测方法优化及在冷鲜猪	ethods and application in	,刘媛,方春,单颖,陈健舜,J	ang Chun,Shan Ying,Chen J	中国食品学报	urnal of Chinese Food Scie	12	6	141-147	2012	sn.1009-7848.2012.06.021
3277	上海市闵行区腹泻患者病	genic bacteria in diarrhea p	荣,刘芸,骆玲飞,陈国强,欧	iu Yun,Luo Lingfei,Chen Gu	职业与健康	Occupation and health	28	17	封2-封3	2012	-
3278	市肉食品食源性致病菌的	tus of foodborne pathogen	从克.	From grams.	河南预防医学杂志	n Journal of Preventive Me	23	4	308-310	2012	-
3279	泻患者病原菌分离及流行	al analysis of pathogenic b	勤爱,张苹,张培,于德宪,李	Zhang Ping,Zhang Pei,Yu D	中国微生态学杂志	inese Journal of Microecolo	24	6	521-522,526	2012	-
3280	部分养猪场常见病原菌的	mon pathogenic bacteria	翟方,顾武军,龚大弟,范德忠	u Wujun,Gong Dadi,Fan De	动物医学进展	vances in Veterinary Medic	33	10	25-28	2012	sn.1007-5038.2012.10.007
3281	苏省肉鸡沙门菌污染专项	Salmonella contamination	许晓梅,沈赟,马恺,倪云龙,汤	ei,Shen Yun,Ma Kai,Ni Yunl	中国食品卫生杂志	hinese Food Hygiene Journ	24	2	170-172	2012	-
3282	炎沙门氏菌引起食物中毒	of food poisoning caused	冯裕如.	Feng Yuru.	健康必读（中旬刊）	-read for health (mid-term	11	10	75-75	2012	-
3283	表面及医务人员手细菌污	ination of hospital object s	君,郭建华,孙利群,王艳秋,刘	o Jianhua,Sun Liqun,Wang	中国消毒学杂志	hinese Journal of Disinfecti	29	4	325-326	2012	-
3284	族聚居地区家宴食物中毒	ng at a family banquet in a	张志军,李亚琪,王晓玲.	ng Zhijun,Li Yaqi,Wang Xiao	宁夏医科大学学报	al of Ningxia Medical Unive	34	2	188-189	2012	sn.1674-6309.2012.02.032
3285	食品中5种食源性致病菌监	Analysis of monitoring resu	张桂芳,徐锡.	Zhang Guifang,Xu Xi.	宁夏医学杂志	Ningxia Medical Journal	34	3	276-277	2012	sn.1001-5949.2012.03.040
3286	慢性腹泻患者粪便标本的	microorganisms in fecal sa	,李敏,田敬华,张燕,王玉兴	Tian Jinghua,Zhang Yan,Wa	验和临床感染病杂志（电	ntal and Clinical Infectious	6	2	135-138	2012	sn.1674-1358.2012.02.013
3287	引起沙门氏菌食物中毒检	case of salmonella food po	王锐,王英,高志彬.	ang Rui,Wang Ying,Gao Zhib	中外健康文摘	nese and Foreign Health Dig	-	50	171-172	2012	sn.1672-5085.2012.50.164
3288	沙门菌的分离鉴定及其耐	stance analysis of Salmonel	,王申锋,许兰菊,朱金凤,孙	henfeng,Xu Lanju,Zhu Jinf	畜牧与兽医	Husbandry and Veterinary M	44	8	29-33	2012	-
3289	沙门氏菌引起食物中毒的	of food poisoning caused	元萍,吕文凤,郑永惠,薄立	g,Lu Wenfeng,Zheng Yong	中国城乡企业卫生	ygiene in urban and rural a	27	2	103-104	2012	-
3290	食品中沙门氏菌污染状况	contamination status in ei	琪木格.	Qimuge.	内蒙古医学杂志	ner Mongolia Medical Jour	44	2	封3	2012	sn.1004-0951.2012.02.056
3291	食源性沙门菌的分布特点	alence of foodborne Salmo	瑞伦,黄志广,莫嘉延,李凤	,Huang Zhiguang,Mo Jiayan	中国预防医学杂志	se Journal of Preventive Me	13	6	436-438	2012	-

3292	R检测方法对畜禽肉中沙门	Salmonella contamination	可,孙吉昌,严寒,谢智欣,尹德	ichang,severe cold,Xie Zhix	农产品质量与安全	ultural product quality and	-	z1	21-24	2012	sn.1674-8255.2012.z1.005
3293	例非伤寒沙门菌耐药谱和	ella in diarrhea cases in Gu	梅,谭海玲,王晨,梁兆铭,刘	,Wang Chen,Liang Zhaomi	中华微生物学和免疫学杂志	rnal of Microbiology and In	32	6	542-548	2012	sn.0254-5101.2012.06.012
3294	安区某街道其他感染性腹	r infectious diarrhea in a s	张克春,甘露,杨杰,刘灵辉	chun,honeydew,Yang Jie,Li	医学信息	medical information	25	9	396-397	2012	sn.1006-1959.2012.09.447
3295	品中微生物污染调查分析及	ontamination in exported a	孟秀梅,夏雪奎,袁文鹏,刘新	,Xia Xuekui,Yuan Wenpeng	农产品加工·学刊	ing of agricultural products	-	1	24-29	2012	1671-9646(X).2012.01.006
3296	甲型副伤寒暴发疫情的病	tiology of a paratyphoid fev	唐保晖,黄丽华,吴林洪.	Baohui,Huang Lihua,Wu Lin	职业与健康	Occupation and health	28	8	991-992	2012	-
3297	共场所从业人员沙门菌感染	ion status among employe	花蕾.	bud.	实验与检验医学	imental and Laboratory Me	30	1	71-72	2012	sn.1674-1129.2012.01.029
3298	15例腹泻病例病原菌检测	detection results of pathog	张梅,程小迎,许宁.	ng Mei,Cheng Xiaoying,Xu N	寄生虫病与感染性疾病	ic diseases and infectious d	10	1	54-56	2012	-
3299	饮单位自制凉菜细菌污染	contamination of homemad	高平,李丽,杨宏,吴金霞,王冰	i Li,Yang Hong,Wu Jinxia,W	中国食品卫生杂志	hinese Food Hygiene Journ	24	1	77-79	2012	-
3300	肠道中致病菌的分离鉴定	test of pathogenic bacteria	闫志强,王李萍,于忠垚,侯立	ng,Wang Liping,Yu Zhongya	当代畜牧	ntemporary animal husban	-	6	10-11	2012	-
3301	病原菌的分离鉴定及药敏试	d Drug Sensitivity Test of C	大伟,许兰菊,张光辉,尤晓	vei,Xu Lanju,Zhang Guangh	河南农业科学	Henan Agricultural Sciences	41	4	148-153	2012	sn.1004-3268.2012.04.036
3302	分市售调味酱的微生物检	of some commercially avai	汪洪涛,李小华,杨爱萍.	Hongtao,Li Xiaohua,Yang A	中国调味品	Chinese condiments	37	2	98-100	2012	sn.1000-9973.2012.02.024
3303	肠炎沙门菌引起食物中毒	of food poisoning caused I	张耘.	Zhang Yun.	上海预防医学	hanghai Preventive Medicin	24	2	71-72	2012	sn.1004-9231.2012.02.005
3304	2010-2011年感染性腹泻致	urveillance and analysis of	荣,赵伟,余红,秦萌,邢洪光	ao Wei,Yu Hong,Qin Meng,	实用预防医学	ractical preventive medicin	19	6	887-889	2012	sn.1006-3110.2012.06.034
3305	沙门菌致败血症并化脓性脑	ausing sepsis and purulent	王迁,李冠慧.	Wang Qian,Li Guanhui.	国际检验医学杂志	onal journal of laboratory r	33	9	1151-1152	2012	sn.1673-4130.2012.09.071
3306	沙门氏菌的分离鉴定及毒	commercially available fre	勤,芮弦,陈婷,李成山,张三	Chen Ting,Li Chengshan,Zha	食品科学	food science	33	16	154-158	2012	-
3307	2004-2011年食源性致病菌	-2011Annual Foodborne Pa	本康,李占裕,郑南才,潘钊	,Li Zhanyu,Zheng Nancai,P	医学动物防制	medical animal control	28	10	1074-1076	2012	sn.1003-6245.2012.10.004
3308	食品中食源性致病菌污染	hogenic bacteria contamin	佳璇,夏丹,林云万,侯水平,张	Chardin,Lin Yunwan,Hou le	中国卫生检验杂志	ese Journal of Health Inspe	22	8	1964-1966	2012	-
3309	州市餐饮业食品安全监测	monitoring results of cate	陈瑞英,蔡震,杨娟.	en Ruiying,Cai Zhen,Yang Ju	中国食品卫生杂志	hinese Food Hygiene Journ	24	1	63-67	2012	-
3310	鼠类沙门氏菌感染状况调	of Salmonella infection sta	李俭峰.	Li Jianfeng.	疾病监测与控制	sease surveillance and cont	6	6	356-357	2012	-
3311	本零售生鲜肉和产肉动物_	cteria on Japanese retail fre	-	-	中国家禽	Chinese poultry	-	22	69	2012	-
3312	0-2012年医疗机构消毒监测	annual disinfection monit	王孝正,段广才.	ng Xiaozheng,Duan Guang	中国药物经济学	harmacoeconomics in Chin	-	3	165-166	2012	sn.1673-5846.2012.03.092
3313	属流行血清型分型及耐药	ance analysis of prevalent s	陈金玲1,陈志晓1,麦宗良2	g1,Chen Zhixiao1,Mai Zong	检验医学与临床	boratory medicine and clini	-	23	-	2012	-
3314	染性腹泻疫情病原检测结	etection results in an infect	张娟.	Zhang Juan.	安徽预防医学杂志	i Journal of Preventive Med	18	2	154-155	2012	-
3315	年食品中食源性致病菌检	detection results of foodb	张彩虹.	Zhang Caihong.	医学动物防制	medical animal control	28	2	157-158	2012	sn.1003-6245.2012.02.016
3316	寒沙门氏菌分离鉴定和基因	genotyping results of som	继华,杨继先,解书润,吴小	Yang Jixian,Jie Shurun,Wu	中国医药科学	Chinese Medical Sciences	2	11	92-93	2012	-
3317	木齐市鸡鸭蛋中沙门氏菌的	lmonella in Chicken and D	F,麦麦提艾力·麦麦提,段进	meti Aili·Maimaiti,Duan Jin	新疆畜牧业	Xinjiang animal husbandry	-	5	23-24	2012	sn.1003-4889.2012.05.016
3318	厅从业人员肠道沙门菌属带	onella carriage among em	艳,程倩,梁新秀,王磊利,罗	Qian,Liang Xiuxin,Wang Le	中华医院感染学杂志	ournal of Hospital Infectiou	22	12	2576-2577	2012	-
3319	口人源沙门氏菌血清型分布	resistance of food-borne a	陈爱贞,骆艳婷,陈健,雷宇纳	Aizhen,Luo Yanting,Chen J	热带医学杂志	ournal of tropical medicine	12	8	955-958	2012	-
3320	内区2011年食源性致病菌监	Analysis of annual foodbor	吕秋艳,王志越,宋景红,李勇	,Wang Zhiyue,Song Jinghon	实用预防医学	ractical preventive medicin	19	11	1667-1668	2012	sn.1006-3110.2012.11.022
3321	～2011年食源性致病菌监	ysis of annual foodborne p	周汉洪,李红,薛镧,李德华	anhong,Li Hong,Xue Lan,Li	国外医学（医学地理分册）	Medicine (Medical Geograp	33	4	236-238	2012	sn.1001-8883.2012.04.005
3322	沙门氏菌的分离、鉴定及	drug resistance detection o	姚大伟,江芸,徐幸莲,周光宏	iang Yun,Xu Xinglian,Zhou	食品科学	food science	33	24	210-214	2012	-
3323	猪霍乱沙门菌所致败血症1	ed by Salmonella choleraes	彭鹏.	Peng Peng.	中国煤炭工业医学杂志	Journal of Coal Industry M	15	9	1425	2012	-
3324	肉鸡养殖场中死淘鸡病原菌	c bacteria of dead chickens	田华,齐静,魏甜甜,单虎,刘王	ng,Wei Tiantian,single tige	中国畜牧兽医	nal husbandry and veterina	39	1	213-217	2012	sn.1671-7236.2012.01.052
3325	地区成人感染性腹泻病原学	gy of adult infectious diarr	张再宽,张秀瑜,王云英,黄长	Zhang Xiuyu,Wang Yunying	中国微生态学杂志	inese Journal of Microecolo	24	9	781-785	2012	-
3326	009-2011年食源性致病菌	1Annual Foodborne Patho	国忠,高海英,蒋建章,王均	,Gao Haiying,Jiang Jianzhan	医学动物防制	medical animal control	28	4	378-380	2012	sn.1003-6245.2012.04.008
3327	源沙门菌的分离鉴定及耐	resistance detection of Sal	吕纯,覃春红,赖婧,舒刚,吴耿	hunhong,Lai Jing,Shu Gang	中国兽医杂志	Chinese Veterinary Journal	48	12	63-66,中插7	2012	sn.0529-6005.2012.12.025
3328	地区蛋鸡孵化场沙门氏菌	monella in laying hen hatch	李,张德胜,赖守勋,代友洪,叶	Desheng,Lai Shouxun,Dai Y	四川畜牧兽医	nal Husbandry and Veterina	39	4	20-21	2012	sn.1001-8964.2012.04.009

3329	氏菌的分离鉴定及药物敏	n and Drug Sensitivity Test	范俊娟.	Fan Junjuan.	辽宁农业职业技术学院学	Agricultural Vocational an	14	2	4-5	2012	sn.1671-0517.2012.02.002
3330	门菌PFGE分子分型及耐药	EMolecular typing and dru	迎慧,扈庆华,吕东月,邱亚	,Hu Qinghua,Lu Dongyue,C	热带医学杂志	journal of tropical medicine	12	6	750-753	2012	-
3331	沙门氏菌的分离鉴定及多重	from broiler carcasses and	,乔昕,符晓梅,沈贇,王燕梅	Xin,Fu Xiaomei,Shen Yun,W	医科大学学报（自然科学	Medical University (Natur	32	1	125-128	2012	-
3332	县市售食品食源性致病菌	genic bacteria contaminati	勤美,刘乙锟,王佳雯,陆震	ei,Liu Yikun,Wang Jiawen,L	中国卫生检验杂志	ese Journal of Health Inspe	22	1	141-143	2012	-
3333	花生引起的沙门菌食物中	nella food poisoning cause	王岩,李杨.	Wang Yan,Li Yang.	中外健康文摘	hese and Foreign Health Di	9	15	366-366	2012	sn.1672-5085.2012.15.339
3334	梧州市食品中食源性致病	of foodborne pathogenic b	冼桂江,彭美薇,盘珍梅.	uijiang,Peng Meiwei,Pan Zh	职业与健康	Occupation and health	28	17	2122-2124	2012	-
3335	肉中分离的136株沙门菌血	angxi Province136Serologi	,曾艳兵,刘成伟,朱应飞,王	Yanbing,Liu Chengwei,Zhu Y	实验与检验医学	imental and Laboratory Me	30	1	10-12	2012	sn.1674-1129.2012.01.004
3336	患者病原菌分布和耐药性	resistance of pathogenic b	王静霞.	Wang Jingxia.	湖南中医药大学学报	University of Traditional C	32	10	48-49	2012	070X.2012.10.022.048.02
3337	食服务业从业人员沙门氏菌	s among employees in pub	王正革.	Wang Zhengge.	医食参考	Medical and food reference	-	9	-	2012	-
3338	培阳性病原菌在血行感染	culture pathogens in the ev	王涛,李红.	Wang Tao,Li Hong.	健康必读（下旬刊）	ust-read for health (late iss	-	7	256-257	2012	-
3339	型副伤寒暴发疫情的流行病	stigation of an outbreak of	施远陆.	Shi Yuanlu.	应用预防医学	applied preventive medicin	18	1	64-64	2012	n.1673-758X.2012.01.025
3340	009-2011年医院消毒效果	nnual hospital disinfection	陈香郡,胡振龙,李小燕,刘	ounty,Hu Zhenlong,Li Xiao	中国医学创新	Chinese medical innovation	9	19	92-93	2012	sn.1674-4985.2012.19.056
3341	1年禽、畜养殖和屠宰过	alysis of annual poultry an	宁,叶瑞国,陆运龙,宋斌,	ng,Ye Ruiguo,Lu Yunlong,S	职业与健康	Occupation and health	28	16	1979-1981	2012	-
3342	大夫雷登沙门菌引起食物中	of food poisoning caused b	海燕,黄健利,钟凌,郭宝勇	,Huang Jianli,Zhong Ling,G	海峡预防医学杂志	s Journal of Preventive Me	18	1	58-59	2012	-
3343	患者血中分离出奥雷宁堡沙	isolated from blood of lym	,张浩,王培昌,闵嵘,李燕,	Hao,Wang Peichang,Min R	临床检验杂志	journal of clinical testing	30	9	718	2012	-
3344	的临床与病原学分布特点及	haracteristics and drug resi	蒋莹,庄天彦,郭祥萍.	ng,Zhuang Tianyan,Guo Xia	北京医学	Beijing Medicine	34	9	824-827	2012	-
3345	沙门菌引起的食物中毒实验	f a case of food poisoning	夏良贤,乔玉林.	Xia Liangxian,Qiao Yulin.	中国卫生检验杂志	ese Journal of Health Inspe	22	9	2247-2248	2012	-
3346	病患者合并沙门氏菌感染	IDS patients complicated w	兰玲鲜.	Lan Lingxian.	内科	Internal Medicine	7	2	143-144	2012	sn.1673-7768.2012.02.024
3347	433例婴幼儿腹泻病原微	and analysis of pathogenic	曜方,李莉,曹昌志,马晓珍,	ang,Li Li,Cao Changzhi,Ma	疾病监测	disease surveillance	27	2	97-100	2012	ssn.1003-9961.2012.2.005
3348	沙门氏菌引起食物中毒的	case of food poisoning cau	于洁,李东芝,李红梅.	u Jie,Li Dongzhi,Li Hongme	中国卫生产业	China health industry	-	2	95	2012	-
3349	年登封市肠道传染病监测	is of intestinal infectious d	德祥,李洪民,张建萍,张翠	ng,Li Hongmin,Zhang Jianp	河南预防医学杂志	n Journal of Preventive Me	23	1	65-67	2012	-
3350	带者分离株血清学及PFG	r isolate serology andPFG	,王红,唐振柱,李秀桂,孙	Hong,Tang Zhenzhu,Li Xiu	中国热带医学	Chinese tropical medicine	12	10	1176-1178,1188	2012	-
3351	夏秋季腹泻病例流行特征及	mmer and autumn diarrhe	小瑜,游旅,田克诚,唐光鹏	,Travel,Tian Kecheng,Tang	应用预防医学	applied preventive medicin	18	3	133-136	2012	n.1673-758X.2012.03.002
3352	售食品中食源性致病菌抽	ic bacteria in commercially	吴玉峰.	Wu Yufeng.	青海医药杂志	Qinghai Medical Journal	42	10	58-59	2012	-
3353	邮轮急性胃肠炎疾病暴发	cute gastroenteritis outbre	孙丽萍,韩辉.	Sun Liping,Han Hui.	中国国境卫生检疫杂志	ontier Health and Quarantin	35	2	104-107,111	2012	-
3354	滋病并发血流感染的临床分	AIDS complicated by bloo	励峰,朱翠云,黄琴,孙洪清	Zhu Cuiyun,Huang Qin,Sun	医学研究杂志	journal of medical research	41	12	32-35	2012	n.1673-548X.2012.12.012
3355	餐饮业现榨果蔬汁卫生状	d fruit and vegetable juices	陈美芬,裘立晓,徐奋奋.	n Meifen,Qiu Lixiao,Xu Fen	中国卫生检验杂志	ese Journal of Health Inspe	22	9	2198-2199	2012	-
3356	人员对腹泻病人诊疗行为	ent behavior of Shanghai m	明,袁政安,肖文佳,冉陆,顾	n Zheng'an,Xiao Wenjia,Ra	中国卫生资源	China health resources	15	3	268-270	2012	n.1007-953X.2012.03.038
3357	10年许昌市细菌性食物中	s of bacterial food poisoni	仝玉平,贾桂华,毛斐.	ng Yuping,Jia Guihua,Mao	职业与健康	Occupation and health	28	12	1476-1477	2012	-
3358	疆222株伤寒沙门菌耐药性	nd analysis of drug resista	木合亚提·胡塞英,刘清,木	r,muheyati·Hu Saiying,Liu	疾病预防控制通报	se Prevention and Control	-	2	35-36	2012	-
3359	深圳市细菌性食物中毒检	cterial food poisoning dete	邱亚群,石晓路,林一曼,耿	iu Yaqun,Shi Xiaolu,Lin Yim	华南预防医学	uth China Preventive Medi	38	2	67-68	2012	-
3360	市售食品食源性致病菌监	he pathogenic bacteria in c	杨毅,陈国利,吴亚英,曹华	i,Chen Guoli,Wu Yaying,Ca	中国医药指南	Chinese Medicine Guide	10	11	394-395	2012	sn.1671-8194.2012.11.316
3361	1年江西省食品中沙门菌污	y on Salmonella contamina	,曾艳兵,刘成伟,朱应飞,王	Yanbing,Liu Chengwei,Zhu Y	实验与检验医学	imental and Laboratory Me	30	2	126-129	2012	sn.1674-1129.2012.02.006
3362	沙门氏菌污染食品引起的食	oisoning caused by Salmon	金义民,周彦杰,王桂玲.	i-min,Zhou Yanjie,Wang G	医学信息	medical information	25	12	438-439	2012	sn.1006-1959.2012.12.472
3363	性沙门氏菌的分离鉴定及耐	ance analysis of pathogeni	周琼,刘芳萍,李昌文,刘立	Qiong,Liu Fangping,Li Cha	中国兽药杂志	ese Journal of Veterinary D	46	5	11-13	2012	sn.1002-1280.2012.05.003
3364	都柏林沙门氏菌的分离与	entification of a Dublin stra	孙晓红.	Sun Xiaohong.	中外健康文摘	hese and Foreign Health Di	9	49	163-164	2012	sn.1672-5085.2012.49.157
3365	起食物中毒的病原学毒力	tification of the etiology of	良,湛志飞,李广兵,黄良军	han Zhifei,Li Guangbing,Hu	实用预防医学	ractical preventive medicin	19	11	1721-1723	2012	sn.1006-3110.2012.11.047

3366	寒沙门菌引起食物中毒的	a case of food poisoning ca	邓丽芳,王雪梅,许立新.	g Lifang,Wang Xuemei,Xu L	海峡预防医学杂志	s Journal of Preventive Me	18	1	55-56	2012	-
3367	衢州市食源性沙门氏菌污	sis of Foodborne Salmonel	军,金莞尔,张建民,叶承华,	an'er,Zhang Jianmin,Ye Che	中国农村卫生事业管理	na's rural health managem	32	2	171-172	2012	-
3368	年北京市延庆县食源性致	of foodborne pathogenic b	任淑敏.	Ren Shumin.	首都公共卫生	capital public health	6	1	36-38	2012	sn.1673-7830.2012.01.009
3369	10年-2011年食源性致病菌	nual surveillance analysis o	陈晓勇,黄先慧,邓琴,陈梅	ong,Huang Xianhui,Deng Qi	中国卫生检验杂志	ese Journal of Health Inspe	22	7	1658-1659,1662	2012	-
3370	秋季腹泻大便标本致泻性	tool specimens of infants a	祝俭平,廖礼梅.	Zhu Jianping,Liao Limei.	检验医学与临床	boratory medicine and clini	9	3	355-356	2012	sn.1672-9455.2012.03.058
3371	大肠杆菌和沙门氏菌的分	herichia coli and Salmonel	妍君,杨莉,刘春华,肖平,	n,Yang Li,Liu Chunhua,Xiao	食品科学	food science	33	13	202-206	2012	-
3372	市售食品中食源性致病菌	borne pathogenic bacteria i	王颖,彭少杰,田明胜,王李	Peng Shaojie,Tian Mingshe	中国食品卫生杂志	hinese Food Hygiene Journ	24	3	264-267	2012	-
3373	沙门菌引起化脓性脑膜	nt meningitis caused by Sa	李冠慧,张静,郭艳霞,张丽	hui,Zhang Jing,Guo Yanxia,Z	中国综合临床	hina Comprehensive Clinic	28	5	551	2012	sn.1008-6315.2012.05.035
3374	艾滋病相关慢性腹泻的临	study on AIDS-related chro	玲,王玉光,李坪,刘景院,王	g Yuguang,Li Ping,Liu Jingy	验和临床感染病杂志（电	ntal and Clinical Infectious	6	3	191-194	2012	sn.1674-1358.2012.03.004
3375	淮安市食源性病原菌监测	ng results of foodborne pa	张蓉,熊建明,杨振宇.	Rong,Xiong Jianming,Yang Z	预防医学情报杂志	of Preventive Medicine Info	28	7	548-550	2012	-
3376	品、公共场所从业人员沙	ong workers in food and p	张珺,顾海燕,李波,高和平.	Jun,Gu Haiyan,Li Bo,Gao H	医学动物防制	medical animal control	28	3	272-273	2012	sn.1003-6245.2012.03.017
3377	控中心从鸡蛋到鸡肉全过	ors the entire process from	-	-	家禽科学	poultry science	-	7	54-54	2012	-
3378	感染性腹泻疫情的病原学	ection of an infectious diar	杨丽桦.	Yang Lihua.	亚太传统医药	ia Pacific Traditional Medic	8	4	43-44	2012	sn.1673-2197.2012.04.019
3379	1年腹泻病人沙门菌血清	a serotypes and drug resist	,董柯,安戈,赵瑞臻,张月泉	Ke,Ango,Zhao Ruizhen,Zha	中国人兽共患病学报	Chinese Journal of Zoonose	28	10	1017-1019	2012	sn.1002-2694.2012.10.012
3380	市售猪肉中食源性致病菌	pathogenic bacteria from c	以琨,谭艾娟,吕世明,方英	n,Tan Aijuan,Lu Shiming,Fa	天津农业科学	Tianjin Agricultural Sciences	18	2	72-74	2012	sn.1006-6500.2012.02.021
3381	门菌引起的中学生食物中	among middle school stude	李晔,贺天锋,张胜良,易波	e Tianfeng,Zhang Shenglian	中国学校卫生	Chinese school health	33	6	755-756,759	2012	-
3382	年食品污染状况及食源性	mination status and foodb	铨,方叶珍,朱晓霞,俞骅,文	Fang Yezhen,Zhu Xiaoxia,Y	中国农村卫生事业管理	na's rural health managem	32	7	741-743	2012	-
3383	氏菌的分离鉴定、耐药性	ysis and pathogenicity test	,郭旋,张民秀,兰家暖,廖	Zhang Minxiu,Lan Jianuan,	中国畜牧兽医	mal husbandry and veterina	39	1	172-177	2012	sn.1671-7236.2012.01.041
3384	链中沙门氏菌的污染调查	in the chicken slaughtering	魏建忠,刘军军,占松鹤,赵	Jianzhong,Liu Junjun,Zhan	食品科学	food science	33	17	48-53	2012	-
3385	鼠伤寒沙门菌分子流行病	l study of Salmonella typhi	,陈建辉,熊美琴,徐海滨,陈	Jianhui,Xiong Meiqin,Xu H	中国预防医学杂志	se Journal of Preventive Me	13	3	161-164	2012	-
3386	10年食品中食源性致病菌	contamination status of f	吴林洪,韦麒.	Wu Linhong,Wei Qi.	医学动物防制	medical animal control	28	4	418-420	2012	sn.1003-6245.2012.04.024
3387	海珠区2011年食品污染物	11Annual food contamina	吴琪,许少洪,曾雅.	Wu Qi,Xu Shaohong,Zeng Ya	海峡预防医学杂志	s Journal of Preventive Me	18	2	57-58	2012	-
3388	售食品食源性致病菌监测	ne pathogenic bacteria in c	张艳梅.	Zhang Yanmei.	中国保健营养（下旬刊）	ese health nutrition (late is	22	5	1071-1072	2012	-
3389	省食源性非伤寒沙门菌疾	id Salmonella disease burd	翌婧,吴高林,郑东宇,甄世	,Wu Gaolin,Zheng Dongyu,	江苏预防医学	iangsu Preventive Medicine	23	6	25-27	2012	sn.1006-9070.2012.06.010
3390	海珠区食品安全风险监测	sk monitoring results in Ha	丘春萍,郭宁晓.	Qiu Chunping,Guo Ningxiao	国际医药卫生导报	ational Medical and Health	18	12	1802-1806	2012	sn.1007-1245.2012.12.050
3391	某区禽畜肉中致病菌污染	hogenic bacteria in poultry	刘小祥,吴国莉.	Liu Xiaoxiang,Wu Guoli.	中国医药指南	Chinese Medicine Guide	10	4	93-94	2012	sn.1671-8194.2012.04.058
3392	-2010年急性腹泻患者沙门	of annual Salmonella surve	灿磊,叶玉龙,张媛媛,李澜	i,Ye Yulong,Zhang Yuanyua	公共卫生与预防医学	Health and Preventive Me	23	3	9-11	2012	-
3393	源性疾病监测中沙门菌的	Salmonella in foodborne di	,贺连华,石晓路,陈妙玲,	anhua,Shi Xiaolu,Chen Mia	中国卫生检验杂志	ese Journal of Health Inspe	22	3	581-583	2012	-
3394	年宿迁市食源性致病菌监	analysis of foodborne path	伟光,陈尚林,蔡丹丹,闫	g,Chen Shanglin,Cai Danda	江苏预防医学	iangsu Preventive Medicine	23	3	7-9	2012	sn.1006-9070.2012.03.003
3395	沙门氏菌引起的食物中毒	of a case of food poisoning	杜昌海,杨万群.	Du Changhai,Yang Wanqun	外医学（医学地理分册）	Medicine (Medical Geogra	33	3	189-190	2012	sn.1001-8883.2012.03.014
3396	沙门菌致感染性腹主动脉	aneurysm caused by Salmo	于翔,陈鹏,李博,于金燕,马	g,Chen Peng,Li Bo,Yu Jinyan	中国老年学杂志	hinese Journal of Gerontolo	32	17	3768-3769	2012	sn.1005-9202.2012.17.074
3397	柏林沙门菌致肝脓肿一例	liver abscess caused by Sa	张文瑾,崔恩博,张鞠玲,陈	Wenjin,Cui Eunbo,Zhang Ju	临床检验杂志（电子版）	f Clinical Testing (electronic	-	2	121-122	2012	-
3398	起沙门氏茵食物中毒调查	nalysis of a Case of Salmon	李蓓,魏彩梅.	Li Bei,Wei Caimei.	疾病监测与控制杂志	of Disease Surveillance and	6	7	414-415	2012	-
3399	年开平市细菌性食物中毒	ial food poisoning test resu	丽芳,许立新,王雪梅,林崇	Xu Lixin,Wang Xuemei,Lin	职业与健康	Occupation and health	28	13	1608-1609,1611	2012	-
3400	沙门菌致脓气胸一例并文	ed by Salmonella choleraes	斌,朱奕豪,姚宇锋,徐峻,	u Yihao,Yao Yufeng,Xu Jun	中华结核和呼吸杂志	l of Tuberculosis and Respi	35	9	683-686	2012	sn.1001-0939.2012.09.014
3401	中食源性致病菌定性及定	nd analysis of foodborne p	川,张雷,郭惠,王路梅,苗升	ng Lei,Guo Hui,Wang Lume	中国卫生检验杂志	ese Journal of Health Inspe	22	9	2104-2106	2012	-
3402	急性散发腹泻病例病原学	etiology of acute sporadic	继珊,庞晶晶,高波,崔海	,Pang Jingjing,Gao Bo,Cui H	中国卫生检验杂志	ese Journal of Health Inspe	22	8	1970-1972,1977	2012	-

3403	霍乱沙门氏菌败血症1例报	Salmonella septicemia1Ex	蔡华.	Cai Hua.	中外健康文摘	hese and Foreign Health Di	-	37	181-182	2012	sn.1672-5085.2012.37.185
3404	廊坊市食源性致病菌监测结	ults of foodborne pathoger	刘国良,邱贺民,肖利新,邱	liang,Qiu Hemin,Xiao Lixin,	中国美容医学	Chinese aesthetic medicine	21	18	848	2012	sn.1008-6455.2012.18.870
3405	市某县甲型副伤寒暴发队列	atyphoid A outbreak in a	黄革;李建明;	ellow leather;Li Jianming	疾病监测与控制	ease surveillance and co	6	11	653-655	2012	-
3406	-2012年细菌性食物中毒检	lysis of annual bacterial foo	胜中,叶雪仪,李宇聪,杨丽	zhong,Ye Xueyi,Li Yucong,Y	国际医药卫生导报	ational Medical and Health	19	19	3076-3078	2013	sn.1007-1245.2013.19.049
3407	医院院内感染病原菌分布	hogenic bacteria in general	谭斌,张弦,薛健,周锡鹏,李	Bin,Zhang Xian,Xue Jian,Zh	中华流行病学杂志	inese Journal of Epidemiol	34	11	1134-1138	2013	.0254-6450.2013.011.020
3408	寒并发多器官衰竭患者的	with enteric typhoid fever	邓长升,孙谋,赵子瑜,吴文轩	ng,Sun Mou,Zhao Ziyu,Wu	中华危重病急救医学	e critical care emergency m	25	12	763	2013	sn.2095-4352.2013.12.017
3409	年开封市肉鸡中沙门菌污染	mination status in broiler c	恭举,杨如璞,刘杰,巩飚,陈	ang Rupu,Liu Jie,Gong Biao	中华预防医学杂志	se Journal of Preventive Me	47	5	475-476	2013	sn.0253-9624.2013.05.023
3410	产加工环节沙门菌的污染及	distribution in broiler chic	丙,乔昕,杨小蓉,陈玉贞,裴晓	g Xiaorong,Chen Yuzhen,Pe	中华预防医学杂志	se Journal of Preventive Me	47	5	435-438	2013	sn.0253-9624.2013.05.012
3411	中国其他感染性腹泻监测现	uation of surveillance of ot	刘海霞,张静.	Liu Haixia,Zhang Jing.	中华预防医学杂志	se Journal of Preventive Me	47	4	328-332	2013	sn.0253-9624.2013.04.009
3412	我院儿童细菌性腹泻病原菌	nd drug susceptibility of ch	胡云海,范忠祥,杜成雪.	hai,Fan Zhongxiang,Du Che	医药前沿	Medical Frontier	-	11	70-71	2013	-
3413	寒、副伤寒高发区发热症状	with high incidence of typh	诚,张静,孙军玲,王子军,蒋	,Zhang Jing,Sun Junling,Wa	中华流行病学杂志	inese Journal of Epidemiol	34	3	254-258	2013	sn.0254-6450.2013.03.012
3414	区2011年和2012年食源性	anhe2012Annual surveillan	年,潘朝庆,黄华国,石威,谢	Chaoqing,Huang Huaguo,S	医药前沿	Medical Frontier	-	7	22-23	2013	-
3415	菌所致食源性疾病暴发疫	odborne disease outbreak	康,许静静,王路梅,童晶,张雷	gjing,Wang Lumei,Tong Jin	中华预防医学杂志	se Journal of Preventive Me	47	2	160-163	2013	sn.0253-9624.2013.02.014
3416	年虹口区生鲜食品沙门菌	detection of fresh food in	陈真,张静,沈静.	Chen Zhen,Zhang Jing,quiet	医药前沿	Medical Frontier	-	3	359	2013	-
3417	12年餐饮单位自制熟肉制	alysis on Testing Results o	凌琳,秦立强.	Ling Lin,Qin Liqiang.	医药前沿	Medical Frontier	-	3	368-369	2013	-
3418	糖尿病并发细菌感染的临床	ses of diabetes complicate	杨杰,李绍生.	Yang Jie,Li Shaosheng.	医药前沿	Medical Frontier	-	2	188	2013	-
3419	市售鲜鸭蛋细菌污染的调查	ntamination of commercia	洋,彭义,刘力,彭祥伟,朱艳	Peng Yi,Liu Li,Peng Xiangw	畜禽业	vestock and poultry indust	-	4	60-61	2013	sn.1008-0414.2013.04.043
3420	城市食品中食源性致病菌	orne pathogenic bacteria c	郭树荣,张岚,吴双霞,王静	g,Zhang Lan,Wu Shuangxia	长治医学院学报	nal of Changzhi Medical Co	27	1	19-21	2013	sn.1006-0588.2013.01.006
3421	市2008年腹泻病原菌监测	nnual surveillance analysis c	王润莲,刘宝莲.	Wang Runlian,Liu Baolian.	临床合理用药杂志	l of Rational Clinical Use of	6	21	20-21	2013	sn.1674-3296.2013.21.013
3422	沙门氏菌分离鉴定和药敏实	and drug susceptibility tes	段培培.	Duan Peipei.	中国畜牧兽医文摘	mal Husbandry and Veterin	-	8	34-34,35	2013	-
3423	血行感染革兰阴性杆菌的	monitoring of Gram-negativ	萍,徐俊,陶云珍,王运中,朱	,Tao Yunzhen,Wang Yunzh	临床儿科杂志	Journal of Clinical Pediatric	31	4	339-342	2013	sn.1000-3606.2013.04.011
3424	料中的大肠菌群及沙门氏菌	coliforms and salmonella i	志琴,张晓红,李春,马亚楠,	,Zhang Xiaohong,Li Chun,M	畜牧与饲料科学	Livestock and feed science	34	6	35-37	2013	sn.1672-5190.2013.06.013
3425	氏菌引起的食源性疾病的调	gation of a foodborne illnes	王琴.	Wang Qin.	疾病预防控制通报	se Prevention and Control	-	3	65-66	2013	-
3426	起校园细菌性食物中毒的核	of bacterial food poisoning	王晓英.	Wang Xiaoying.	健康必读（中旬刊）	-read for health (mid-term	12	5	116-116	2013	-
3427	感染性腹泻病原菌分布及	e of pathogenic bacteria in	秦萌,尉秀霞,杨军勇,耿荣,王	ei Xiuxia,Yang Junyong,Ger	实用预防医学	ractical preventive medicin	20	8	900-903	2013	sn.1006-3110.2013.08.002
3428	品及饲料添加剂中沙门氏菌	monella in feed products a	董鹏,李金磊,高延玲,邱富娟	Peng,Li Jinlei,Gao Yanling,C	上海畜牧兽医通讯	hal Husbandry and Veterin	-	2	10-11	2013	sn.1000-7725.2013.02.004
3429	肠杆菌科细菌感染分布及耐	n and drug resistance of E	丽,张丽华,张菊芬,朱学海,朱	ng Lihua,Zhang Jufen,Zhu	中国感染控制杂志	ese Journal of Infection Co	12	5	377-380	2013	sn.1671-9638.2013.05.015
3430	寒沙门氏菌引起的食物中毒	oning caused by Salmonella	罗雪,刘天慧,康燕.	uo Xue,Liu Tianhui,Kang Ya	最新医学信息文摘（电子	edical information abstract	-	20	344-344	2013	sn.1671-3141.2013.20.266
3431	大学教学设施卫生状况调	ealth status of teaching fac	邓怡卿,赵渝.	Deng Yiqing,Zhao Yu.	师范大学学报（自然科学	i Normal University (Natur	42	5	499-503	2013	sn.1000-5137.2013.05.010
3432	西灵山县食源性致病菌监测	of foodborne pathogenic b	丁梅,杨毅修,丁霞.	Ding Mei,Yang Yixiu,Ding Xia	中外健康文摘	hese and Foreign Health Di	-	25	111-112	2013	-
3433	地区域食品的食源性致病菌	detection results in food i	贺欣.	He Xin.	甘肃科技纵横	u science and technology a	42	7	143-145	2013	sn.1672-6375.2013.07.051
3434	区2008-2011年沙门菌感染	2011Epidemiological analy	东,王怡珺,韩颖俊,王曙,顾宝	Yijun,Han Yingjun,Wang Sh	上海预防医学	hanghai Preventive Medici	25	1	4-6,16	2013	sn.1004-9231.2013.01.002
3435	12年广西鸡沙门氏菌感染	on salmonella infection in	覃胜兰,陆文俊,邹联斌,粟艳	u Wenjun,Zou Lianbin,Su Y	上海畜牧兽医通讯	hal Husbandry and Veterin	-	6	4-6	2013	sn.1000-7725.2013.06.002
3436	门氏菌引起食物中毒的病原	of food poisoning caused	文天平.	Wen Tianping.	中国卫生产业	China health industry	-	14	98100	2013	-
3437	年全年市售食品食源性致	status of foodborne pathog	俊荣,王春东,毕红杰,赵玲	Vang Chuntong,Bi Hongjie,Z	山西医药杂志（下半月版）	cal Journal (Second Half M	42	6	269-270	2013	sn.0253-9926.2013.06.015
3438	扬州地区鸡白痢沙门氏菌耐	istance of Salmonella pullo	杨安龙.	Yang Anlong.	畜禽业	vestock and poultry indust	-	4	16-18	2013	sn.1008-0414.2013.04.010
3439	城县市售食品中食源性致病	borne pathogenic bacteria	永红,傅剑羽,黄佳慧,华冬	ianyu,Huang Jiahui,Chinese	现代医药卫生	modern medicine and healt	29	5	675-676,678	2013	sn.1009-5519.2013.05.015

3440	食品中食源性致病菌污染	genic bacteria contaminati	,徐子强,唐建红,钟启丽,刘	qiang,Tang Jianhong,Chung	中国保健营养（中旬刊）	e health nutrition (mid-tern	-	10	842-843	2013	-
3441	北京地区儿童腹泻病原菌	genic bacteria in children w	梅,冯燕玲,薛冠华,闫超,张	nling,Xue Guanhua,Yan Ch	国际检验医学杂志	onal journal of laboratory r	34	4	432-434	2013	sn.1673-4130.2013.04.020
3442	宴宴引起的沙门氏菌食物	monella food poisoning cau	秦田秀.	Qin Tianxiu.	广西医学	Guangxi Medicine	-	7	929-930	2013	ssn.0253-4304.2013.07.44
3443	沙门氏菌菌血症患者的用	use in patients with Salmo	刘云红,林海霞,官真水.	nhong,Lin Haixia,Guan Zhe	内蒙古中医药	ngolia Traditional Chinese	32	10	179-180	2013	sn.1006-0979.2013.10.203
3444	穿刺液中分离出肠炎沙门	Enteritidis isolated from hi	杨峰.	Yang Feng.	健康大视野	Healthy vision	21	16	508-508	2013	-
3445	宜宾市食源性致病菌污染	status of foodborne patho	张红,易松涛,何俊,赵杰.	Hong,Yi Songtao,He Jun,Zh	中国当代医药	inese contemporary medic	20	7	151-152,154	2013	sn.1674-4721.2013.07.075
3446	年餐饮单位熟肉制品微生	crobial monitoring results	王艳,周丽萍,张晋.	ang Yan,Zhou Liping,Zhang	医药前沿	Medical Frontier	-	26	370-370	2013	sn.2095-1752.2013.26.455
3447	沙门氏菌食物中毒调查及	ng in Nandan County and F	周青华.	Zhou Qinghua.	中国农村卫生	rural health in china	-	z1	319-320	2013	-
3448	沙门氏菌引起的食物中毒	case of food poisoning ca	曾甲庆,侯万乐,徐海峰.	Jiaqing,Hou Wanle,Xu Hai	医学信息	medical information	-	27	487-488	2013	-
3449	获得性腹泻病原菌菌谱及	of pathogenic bacteria in	严银燕,叶光勇.	Yan Yinyan,Ye Guangyong.	中国现代医生	modern chinese doctor	51	26	74-75,77	2013	-
3450	炎中沙门氏菌的分离鉴定	eptibility test of Salmonella	潘懿,黄宝学.	Pan Yi,Huang Baoxue.	广西畜牧兽医	nal Husbandry and Veterin	29	5	270-271	2013	-
3451	例伤寒病例的沙门菌分离	ation of Salmonella from a	徐瑞萍,蒋奎英,徐敏.	Ruiping,Jiang Kuiying,Xu M	健康必读（下旬刊）	ust-read for health (late issu	-	6	536-536	2013	-
3452	养标本检出病原菌类别及	ysis of pathogenic bacteria	王燕.	Wang Yan.	广西医学	Guangxi Medicine	35	1	98-99,103	2013	ssn.0253-4304.2013.01.35
3453	沙门氏菌的分离鉴定及其	udy of drug resistance of Sa	张秀芹,杜柏林,韩文瑜.	ang Xiuqin,dublin,Han Wen	饲料工业	Feed industry	-	18	46-48	2013	n.1001-991X.2013.18.012
3454	所从业人员沙门氏菌携带	ella carriage among employ	,梁玉潮,梁卫桥,冯婉丽,李	chao,Liang Weiqiao,Feng V	当代医学	contemporary medicine	-	23	160-160,161	2013	sn.1009-4393.2013.23.124
3455	例伤寒患者多项实验室检测	aboratory test results in pa	覃安汝.	Qin Anru.	中国医药指南	Chinese Medicine Guide	-	22	216-217	2013	-
3456	养猪场猪源沙门氏杆菌的	a originating from pigs in la	张剑勇,谭艾娟,吕世明,方英	yong,Tan Aijuan,Lu Shimin	贵州农业科学	Guizhou Agricultural Science	41	4	109-111	2013	sn.1001-3601.2013.04.033
3457	性食物中毒事故流行病学	on and analysis of a bacter	周立新.	Zhou Lixin.	中国保健营养（中旬刊）	e health nutrition (mid-tern	-	8	532-533	2013	-
3458	婚宴引起的沙门氏菌食物	onella food poisoning cau	杨凤娟.	Yang Fengjuan.	中外健康文摘	nese and Foreign Health Di	-	49	44-45	2013	-
3459	和金黄色葡萄球菌引起的	ing caused by Salmonella N	张桂芳,高斌,刘月淑,徐锡	Guifang,Gao Bin,Liu Yueshu	宁夏医科大学学报	nal of Ningxia Medical Univ	35	2	232,封3	2013	sn.1674-6309.2013.02.041
3460	儿腹泻病原菌分布及耐药	rug resistance of pathogen	春红,卢悦琳,刁文连,陈锐	ong,Lu Yuelin,Diao Wenliar	检验医学与临床	boratory medicine and clini	-	14	1847-1848	2013	sn.1672-9455.2013.14.036
3461	源性沙门氏菌分离鉴定及	nce study of poultry-derive	陈培荣,刘锦妮,何斌.	hen Peirong,Liu Jinni,He Bi	湖北农业科学	Hubei Agricultural Sciences	52	17	4161-4163	2013	sn.0439-8114.2013.17.039
3462	腹泻病原微生物的感染特	eristics of pathogenic micro	焦继光,刘会霞.	Jiao Jiguang,Liu Huixia.	中国医药指南	Chinese Medicine Guide	-	20	44-45	2013	sn.1671-8194.2013.20.028
3463	沙门菌的分离鉴定与耐药	d drug resistance analysis	郑风英.	Zheng Fengying.	黑龙江畜牧兽医（下半月）	dry and Veterinary Medicin	-	12	84-85	2013	-
3464	制品微生物污染状况调查与	of microbial contamination	陈求欢.	Chen Qiuhuan.	农业与技术	Agriculture and Technology	33	8	228	2013	n.1671-962X.2013.08.186
3465	暴露食物中毒事件的流行	sis of a multiple exposure f	吴永,陈米娜,胡依红.	u Yong,Mina Chen,Hu Yiho	中国保健营养（中旬刊）	e health nutrition (mid-tern	-	9	584-585	2013	-
3466	儿童腹泻54例诊治分析	ren54Case diagnosis and tr	张冰冰.	Zhang Bingbing.	中国实用医药	Chinese Practical Medicine	8	16	153	2013	sn.1673-7555.2013.16.110
3467	市食源性疾病哨点医院监	ults of foodborne disease s	陆祥彬,赵瑾.	Lu Xiangbin,Zhao Jin.	江苏预防医学	iangsu Preventive Medicin	24	1	21-23	2013	sn.1006-9070.2013.01.008
3468	门氏菌引起的食物中毒调	n a case of food poisoning	梁艳.	Liang Yan.	中外健康文摘	nese and Foreign Health Di	-	33	226-227	2013	-
3469	炎沙门菌引起的食物中毒	case of food poisoning ca	吴俊霞.	Wu Junxia.	江苏卫生保健	Jiangsu Health Care	15	5	29	2013	sn.1008-7338.2013.05.018
3470	源沙门菌的分离·鉴定和耐	n pigs in Guangxi·Identifica	蹇慧,刘芳,覃永长.	n Hui,Liu Fang,Qin Yongcha	安徽农业科学	Anhui Agricultural Sciences	41	3	1123-1125	2013	sn.0517-6611.2013.03.071
3471	公共场所从业人员沙门菌	g employees working in fo	严华娟.	Yan Huajuan.	市建设理论研究（电子版	ch on Urban Construction (	-	15	-	2013	-
3472	庆区芦阳镇熟肉制品致病	cteria sampling results of c	,杨成林,钟天辉,谭燕妮,X	henglin,Zhong Tianhui,Tan	职业卫生与病伤	ccupational health and illne	28	6	356-357	2013	-
3473	县与流产相关羊病的调查	sheep diseases related to a	成玉,韩赟,王戈平,王顺林,阳	an Yun,Wang Geping,Wan	青海畜牧兽医杂志	mal Husbandry and Veterin	43	3	4-6	2013	sn.1003-7950.2013.03.002
3474	沙门氏菌食物中毒调查及	ng in Nandan County and F	周青华.	Zhou Qinghua.	中国农村卫生	rural health in china	-	7	73-74	2013	-
3475	中分离出肠炎沙门氏菌的1	ritidis isolated from blood1	郭亚娜,齐林.	Guo Yana,Qi Lin.	最新医学信息文摘（电子	edical information abstract	-	26	194-194	2013	sn.1671-3141.2013.26.148
3476	熟肉制品中常见食源性致	f common foodborne path	秦武丽,吴林洪,徐雪英.	Wuli,Wu Linhong,Xu Xuey	医药前沿	Medical Frontier	-	18	389-390	2013	sn.2095-1752.2013.18.491

3477	鸽沙门氏菌的分离鉴定试验	ntification Test of Salmone	王伟.	Wang Wei.	中国畜牧兽医文摘	mal Husbandry and Veterin	-	5	50-50,3	2013	-
3478	11年北京市大兴区感染性	e of infectious diarrhea in	周丽敏,王斌,梁赢,白英丽	min,Wang Bin,Liang Ying,Ba	首都公共卫生	capital public health	7	3	124-127	2013	sn.1673-7830.2013.03.008
3479	肉产品中沙门菌血清型及耐	and drug resistance in poul	源,徐家芳,刘发东,辛秀克	uan,Xu Jiafang,Liu Fadong,S	中国家禽	Chinese poultry	35	13	55-57	2013	sn.1004-6364.2013.13.019
3480	菌属的细菌学感染与药敏	and drug susceptibility an	燕青,赵元勋,徐和平,马晓	ng,Zhao Yuanxun,Xu Hepin	实验与检验医学	imental and Laboratory Me	-	6	624-624	2013	sn.1674-1129.2013.06.048
3481	品中食源性致病菌污染状况	nce of foodborne pathogen	,王岚,胡旃,刘建琪,张林青	n,Hu Zhan,Liu Jianqi,Zhang	实用预防医学	ractical preventive medicin	20	1	16-18	2013	sn.1006-3110.2013.01.005
3482	家庄地区细菌性食物中毒	f pathogenic bacteria of ba	丽云,徐保红,王苋,吕国平	u Baohong,Wang Ran,Lu G	微生物学杂志	journal of microbiology	33	5	73-77	2013	sn.1005-7021.2013.05.016
3483	食源性疾病暴发的病原学	e etiology of a foodborne d	唐雨德,魏德江,周东明,梁洪	Wei Dejiang,Zhou Dongmin	东南国防医药	heast National Defense Med	15	4	335-337	2013	n.1672-271X.2013.04.005
3484	INET血培养临床分离菌的	on and drug resistance of c	倪语星,孙景勇,徐英春,张	g,Sun Jingyong,Xu Yingchun	中国感染与化疗杂志	urnal of Infection and Chen	13	4	241-247	2013	sn.1009-7708.2013.04.001
3485	年大连市食源性致病菌监测	nalysis of foodborne patho	郑晓南,李瑞,王凡.	eng Xiaonan,Li Rui,Wang Fa	中外健康文摘	hese and Foreign Health Di	-	25	146-147	2013	-
3486	门菌病病原分离鉴定及耐药	resistance detection of sal	建,陈绩,赵青剑,马鹏恩,王	en Ji,Zhao Qingjian,Ma Pen	中国家禽	Chinese poultry	35	11	45-46	2013	sn.1004-6364.2013.11.014
3487	炎临床特征及脑脊液分析	luid analysis of purulent me	林贵喜,郑建明,黄天文,姚锁	eng Jianming,Huang Tianw	医学信息	medical information	26	5	355-356	2013	sn.1006-1959.2013.05.370
3488	猪源鼠伤寒沙门菌的分离	of Salmonella typhimurium	贡嘎,查果,拉珍,索朗斯珠	Check the fruit,Rajan,solang	中国兽医杂志	Chinese Veterinary Journal	49	6	44-45	2013	sn.0529-6005.2013.06.015
3489	威镇1起细菌性食物中毒的	Investigation and analysis	蒋奎.	Jiang Kui.	职业卫生与病伤	ccupational health and illne	28	4	249-251	2013	n.1006-172X.2013.04.020
3490	东聊城市食源性致病菌污染	f foodborne pathogenic ba	红,董炳刚,梁胜楠,姜宁宁,	nggang,Liang Shengnan,Jia	中国保健营养（中旬刊）	e health nutrition (mid-tern	-	9	753-754	2013	-
3491	品大肠杆菌O157:H7和沙	ts in Dongguan, Guangdon	伟彬,李永福,张险朋,黄炳	Li Yongfu,Zhang Xianpeng,H	中国兽医杂志	Chinese Veterinary Journal	49	9	69-71	2013	-
3492	培养病原菌的种类分析与	ribution of pathogenic bact	谢宁,苏汉珍,孙华英.	Ning,Su Hanzhen,Sun Huay	临床和实验医学杂志	f Clinical and Experimental	12	3	216-217	2013	sn.1671-4695.2013.03.025
3493	德尔卑沙门菌耐药谱及P	Salmonella delphi in Hunan	云,陈帅,胡旃,张林青,刘建	n Shuai,Hu Zhan,Zhang Linc	实用预防医学	ractical preventive medicin	20	8	915-918	2013	sn.1006-3110.2013.08.007
3494	发性食物中毒的病原菌检	athogenic bacteria in two ca	杨梅霞.	Yang Meixia.	中国保健营养（中旬刊）	e health nutrition (mid-tern	-	5	534-534	2013	-
3495	寒沙门菌对23种抗生素的	hi vs.twenty threeAntibiotic	王云芳.	Wang Yunfang.	吉林医学	Jilin Medicine	34	5	873	2013	sn.1004-0412.2013.05.064
3496	区从业人员携带的沙门菌	ty analysis of Salmonella ca	陆祥彬,徐妍.	Lu Xiangbin,Xu Yan.	中国保健营养（中旬刊）	e health nutrition (mid-tern	-	8	27-28	2013	-
3497	门菌感染暴发流行病原的	ological agents in two outb	张婷,李国民,杨继先,陆洲	Ting,Li Guomin,Yang Jixian,	实用预防医学	ractical preventive medicin	20	1	96-98	2013	sn.1006-3110.2013.01.037
3498	沙门氏菌的危害分析、污染	gation and prevention and	王学硕,崔生辉,邢书霞,丁宏	uo,Cui Shenghui,Xing Shuxi	中国药事	hinese pharmaceutical affai	27	9	974-979	2013	-
3499	北塘区医院污水微生物检测	s of wastewater from Beita	朱惠芳.	Zhu Huifang.	医学信息	medical information	-	14	358-358	2013	sn.1006-1959.2013.14.471
3500	92例小儿腹泻的病原体分	s of pathogens in children v	李超强,朱凯欣,黄美燕,李丹	ang,Zhu Kaixin,Huang Meiy	检验医学与临床	boratory medicine and clini	-	24	3303-3304,3307	2013	sn.1672-9455.2013.24.026
3501	氏菌耐药基因检测与耐药	cken-derived Salmonella ar	李昌文,刘立新,李睿,罗鹏志	gwen,Liu Lixin,Li Rui,Luo P	中国预防兽医学报	rnal of Preventive Veterina	35	8	627-630	2013	ssn.1008-0589.2013.08.06
3502	河南省禽病流行态势与防控	ends and prevention and c	长庚,孙开冬,何向峰,慕桂	g,Sun Kaidong,He Xiangfen	河南畜牧兽医（市场版）	andry and Veterinary Medic	34	4	14-16	2013	sn.1004-5090.2013.04.006
3503	沙门菌PFGE分型及耐药性	PFGEType classification and	,刘万华,任娟娟,武宁,邱洪	anhua,Ren Juanjuan,Wunin	动物医学进展	vances in Veterinary Medic	-	11	1-5	2013	sn.1007-5038.2013.11.001
3504	市龙岗区感染性腹泻病原菌	athogenic bacteria in infect	村春燕,崔海燕,胡琴,陈日炳	yan,Cui Haiyan,Hu Qin,Che	现代检验医学杂志	l of Modern Laboratory Me	28	4	136-138	2013	sn.1671-7414.2013.04.050
3505	自办家宴引起食物中毒的	oisoning caused by a self-h	王慧华,王立华.	Wang Huihua,Wang Lihua.	蛇志	snake ambition	25	3	287-289	2013	sn.1001-5639.2013.03.017
3506	林沙门氏菌引起食物中毒	f a case of food poisoning c	张金华,王桂红.	hang Jinhua,Wang Guihong	东南大学学报（医学版）	Southeast University (Medi	32	1	29-31	2013	sn.1671-6264.2013.01.007
3507	性腹泻病原菌的分布及其耐	analysis of pathogenic bac	杨乐,赵建.	Yang Le,Zhao Jian.	中国实用医药	Chinese Practical Medicine	8	2	137-138	2013	sn.1673-7555.2013.02.111
3508	猪肉产业链中沙门氏菌的	monella in the pork industr	小刚,刘书亮,韩新锋,吴聪	Liu Shuliang,Han Xinfeng,W	食品科学	food science	34	11	250-253	2013	pkx1002-6630-201311054
3509	腹泻患儿的病毒检验结果	virus test results in childre	王东升,付锋.	Wang Dongsheng,Fu Feng.	国际检验医学杂志	onal journal of laboratory r	34	10	1319-1320	2013	sn.1673-4130.2013.10.061
3510	菌的血清型、耐药性和耐	nd drug resistance mechan	蕊蕊,张纯萍,徐士新,张启	Zhang Chunping,Xu Shixin,	农业生物技术学报	nal of Agricultural Biotechn	21	7	855-862	2013	sn.1674-7968.2013.07.012
3511	伤寒沙门菌致乳腺脓肿1	sed by Salmonella Paratyph	储平坤.	Chu Pingkun.	昆明医科大学学报	al of Kunming Medical Univ	34	1	145-146	2013	sn.1003-4706.2013.01.044
3512	鸽的沙门氏菌耐药性与血清	ing from apparently healthy	,邵颖,方艳红,魏建忠,王桂	ng,Fang Yanhong,Wei Jianz	安徽农业大学学报	al of Anhui Agricultural Univ	-	4	-	2013	-
3513	猪沙门氏菌病的诊断和药	ptibility testing of swine sa	李珉珉,许建国,王传锋,张俊	u Jianguo,Wang Chuanfeng	畜禽业	vestock and poultry indust	-	3	62-64	2013	sn.1008-0414.2013.03.038

3514	品大肠杆菌O157：H7和沙	in Dongguan, GuangdongC	伟彬,李永福,张险朋,黄炳	Li Yongfu,Zhang Xianpeng,H	中国兽医杂志	Chinese Veterinary Journal	49	9	69-71	2013	sn.0529-6005.2013.09.027
3515	市售食品安全动态监测结	ring results of commercial	红,赵瑾,陈瑞英,张雪梅,马	o Jin,Chen Ruiying,Zhang X	江苏预防医学	iangsu Preventive Medicin	24	1	59-60	2013	sn.1006-9070.2013.01.027
3516	年全年市售食品食源性致	status of foodborne pathog	俊荣,王春东,毕红杰,赵玲	Vang Chuntong,Bi Hongjie,Z	山西医药杂志（下半月版）	cal Journal (Second Half M	42	3	269-270	2013	-
3517	场牛舍环境中沙门氏菌的	Salmonella in the environm	战波,申欣,车宁宁,薛洋洋	hen Xin,Che Ningning,Xue Y	中国畜牧杂志	a Animal Husbandry Maga	49	8	20-23	2013	sn.0258-7033.2013.08.005
3518	西罗非鱼食源性致病菌及	bacteria and their drug sus	祥庆,吴伟军,吴明媛,黄国	ng,Wu Weijun,Wu Mingyua	南方农业学报	urnal of Southern Agricultu	44	11	1914-1918	2013	n.2095-1191.2013.11.1914
3519	不同规模牧场原料乳的微	w milk from pastures of diff	剧柠,柴佳丽,黄小晶.	Ning,Chai Jiali,Huang Xiaoji	农产品加工·学刊（下）	of agricultural products·Jou	-	8	114-115,118	2013	1671-9646(X).2013.08.066
3520	门菌感染血清型及药敏分	ion serotype and drug susc	廖晓林.	Liao Xiaolin.	西部医学	western medicine	25	2	286-287	2013	sn.1672-3511.2013.02.047
3521	鸡屠宰环节胴体沙门菌污	mination status of broiler c	符晓梅,倪云龙,郑东宇,沈	mei,Ni Yunlong,Zheng Dong	江苏预防医学	iangsu Preventive Medicin	24	3	4-6	2013	sn.1006-9070.2013.03.002
3522	院血培养阳性病原菌分布	pathogenic bacteria with p	云建,陈海,闫中强,艾晓曼	n Hai,Yan Zhongqiang,Ai Xi	华临床医师杂志（电子版	urnal of Clinicians (electron	-	18	8477-8480	2013	sn.1674-0785.2013.18.096
3523	场沙门氏菌分离鉴定及耐	g resistance analysis of Sal	玉,张金茹,郝丽丽,闫延华	ang Jinru,Hao Lili,Yan Yanhu	贵州畜牧兽医	mal Husbandry and Veterin	37	2	10-12	2013	sn.1007-1474.2013.02.004
3524	肠炎沙门菌引起食物中毒	of food poisoning caused	周晓红,严军,陈秀兰,姜洁	phong,Yan Jun,Chen Xiulan	江苏卫生保健	Jiangsu Health Care	15	6	25-26	2013	sn.1008-7338.2013.06.016
3525	起沙门菌食物中毒的调查	nalysis of a case of salmon	洪军,魏德江,李晶,张锦海	jun,Wei Dejiang,Li Jing,Zha	实用预防医学	ractical preventive medicin	20	6	716-718	2013	sn.1006-3110.2013.06.028
3526	沙门氏菌分离鉴定及耐药	-derived Salmonella and a	建华,吴植,刘俊栋,蔡丙严	ua,Wu Zhi,Liu Jundong,Cai	江苏农业科学	iangsu Agricultural Science	-	10	159-160,161	2013	sn.1002-1302.2013.10.061
3527	～2012年沙门菌流行特征	epidemic characteristics a	燕,孙长贵,陈晓,余斐,陈瑞	n Changgui,Chen Xiao,Yu F	临床检验杂志	journal of clinical testing	31	8	621-624	2013	-
3528	2009-2011年食品安全监	-2011Analysis of annual fo	丁惠萍,惠亚鹏,俞志丽.	g Huiping,Hui Yapeng,Yu Z	宁夏医学杂志	Ningxia Medical Journal	35	1	64-65	2013	sn.1001-5949.2013.01.031
3529	菌和沙门氏菌的分离鉴定	stance detection of Escheri	燕霞,陈冬杰,邹海鹏,迟明	en Dongjie,Zou Haipeng,Ch	中国动物检疫	China Animal Quarantine	30	7	63-67	2013	n.1005-944X.2013.07.023
3530	沙门氏菌的分离鉴定及药	ug susceptibility test of Sal	,张桉潮,李昆鹏,张秀丽,

Yuchao,Li Kunpeng,Zhang	中国畜牧兽医	mal husbandry and veterina	40	7	167-171	2013	sn.1671-7236.2013.07.039
3531	鼠伤寒沙门菌引起的食物	isoning caused by Salmon	礼,侯伟伟,肖倩茹,江连,刘	Weiwei,Xiao Qianru,Jiang	检验医学与临床	boratory medicine and clini	10	0	216	2013	-
3532	流感染沙门菌的血清型分	g resistance of Salmonella i	,梁超娟,孙华英,苏汉珍,黎	Chaojuan,Sun Huaying,Su	临床检验杂志	journal of clinical testing	31	6	480	2013	-
3533	儿腹泻病原菌分布及耐药	nce of pathogenic bacteria	春红,卢悦琳,刁文连,陈锐	ong,Lu Yuelin,Diao Wenlian	检验医学与临床	boratory medicine and clini	-	14	-	2013	-
3534	慢性腹泻粪便标本培养结	sults of stool specimens in	杨秀莲.	Yang Xiulian.	宁夏医科大学学报	al of Ningxia Medical Unive	35	3	317-318	2013	sn.1674-6309.2013.03.029
3535	年至2012年食品监测网结	Analysis of annual food m	郭勇峰,王敬辉,吉彦莉.	Yongfeng,Wang Jinghui,Ji Y	中国保健营养（中旬刊）	e health nutrition (mid-tern	-	12	589-590	2013	-
3536	年食源性致病菌污染监测	athogenic bacteria contam	李燕,郭华荣,高银平,何楠	Guo Huarong,Gao Yinping,H	卫生职业教育	Health Professions Educatio	31	10	132-134	2013	sn.1671-1246.2013.10.077
3537	明儿童医院小儿败血症病	and drug resistance of ped	,肖曙芳,李斌,倪林仙,黄海	Shufang,Li Bin,Ni Linxian,	华临床医师杂志（电子版	urnal of Clinicians (electron	-	13	6090-6092	2013	sn.1674-0785.2013.13.070
3538	病合并脓肿诊治2例报道及	th abscess in emergency d	高雨松,陈旭岩.	Gao Yusong,Chen Xuyan.	中国急救医学	mergency medicine in chin	33	11	1053-1056	2013	sn.1002-1949.2013.11.025
3539	腹泻的210例病原微生物检	ren210Analysis of pathoge	韩晶.	Han Jing.	中国社区医师（医学专业）	munity physician (medical	15	7	225	2013	sn.1007-614x.2013.07.220
3540	门菌Ⅱ食物中毒的追溯调	nd analysis of a case of Sal	卢俊荣,王春东,刘金华.	rong,Wang Chuntong,Liu J	山西医药杂志（下半月版）	cal Journal (Second Half M	42	16	883-884	2013	-
3541	霍乱沙门菌败血症死亡一	ath from choleraesuis salm	李璐,胥婕.	Li Lu,Xu Jie.	华临床医师杂志（电子版	urnal of Clinicians (electron	-	15	7328-7329	2013	sn.1674-0785.2013.15.152
3542	起D群沙门菌属食物中毒分	of food poisoning caused	曲永健,李兆国,陈秀香.	ngjian,Li Zhaoguo,Chen Xiu	中国民康医学	nese People's Health Medi	25	14	109111	2013	sn.1672-0369.2013.14.065
3543	市鸡致病性沙门菌的分离	on of pathogenic salmonel	辉,李鹏,张桂荣,张恒军,文	ng,Zhang Guirong,Zhang H	畜牧与兽医	Husbandry and Veterinary M	45	5	111-112	2013	-
3544	沙门氏菌对喹诺酮类药物	tance of Salmonella Indian	,赵红玉,刘宇,候晓林,吴国	Hongyu,Liu Yu,Hou Xiaolin,	中国人兽共患病学报	Chinese Journal of Zoonose	29	6	605-608	2013	sn.1002-2694.2013.06.016
3545	细菌性食物中毒病原菌检测	of Pathogens Caused by B	炳健,钟有光,黎柱荣,冼桂	ong Youguang,Lai Chu Win	健康之路	road to health	12	4	299-299	2013	sn.1671-8801.2013.04.340
3546	某地区98起细菌性食物中	Analysis of pathogenic bac	杨瑛.	Yang Ying.	中外医疗	Chinese and foreign medica	32	16	35-36	2013	sn.1674-0742.2013.16.021
3547	年咸阳市食源性致病菌监	ing results of foodborne pa	,王利,杨囡,秦龙,张俊君,王	i,Yang Nan,Qin Long,Zhang	食品安全质量检测学报	f Food Safety and Quality I	-	5	1525-1528	2013	-
3548	检便样中沙门氏菌的优化	onella in stool samples du	刘晓晨,徐品,张默宇.	Xiaochen,Xu Pin,Zhang Mo	口岸卫生控制	Port health control	18	3	21-23	2013	sn.1008-5777.2013.03.007
3549	菌的检测及其在肉骨粉中的	research on its growth ch	饶正华,王宏,张苏,肖志明	ua,Wang Hong,Zhang Su,Xi	农产品质量与安全	ltural product quality and	-	4	41-43	2013	sn.1674-8255.2013.04.010
3550	内容物沙门氏菌污染的检测	contents of egg shells sold	倩,姚明,刘媛,刘宇贺,李有	,Yao Ming,Liu Yuan,Liu Yu	食品安全质量检测学报	f Food Safety and Quality I	-	5	1505-1510	2013	-

3551	012年伤寒、副伤寒病原学	of annual typhoid and par	龚红明,曾玉明.	ong Hongming,Zeng Yumin	健康大视野	Healthy vision	21	10	39-40	2013	-
3552	乱沙门氏菌败血症27例临	septicemia in children27C	管建宏,沈莉,缪伶伶.	Jianhong,Shen Li,Miao Lin	山东医药	Shandong Medicine	53	10	97	2013	n.1002-266X.2013.10.039
3553	血流感染患者的病原菌分布	enic bacteria in patients wi	覃凌,王慕云,汪利娥.	Ling,Wang Muyun,Wang L	中国临床新医学	hinese new clinical medicin	-	10	971-973	2013	ssn.1674-3806.2013.10.15
3554	区禽源沙门菌的分布及血清	analysis of poultry-derived	华,石火英,吉贞颖,陆玮,杨雪	Huoying,Ji Zhenying,Lu Wei	畜牧兽医学报	mal Husbandry and Veterin	44	2	329-332	2013	-
3555	两省鸡白痢沙门菌的分离	nce analysis of Salmonella	华,石火英,吉贞颖,陆玮,杨雪	Huoying,Ji Zhenying,Lu Wei	中国家禽	Chinese poultry	35	4	17-20	2013	sn.1004-6364.2013.04.006
3556	大兴区水产品致病菌监测结	thogenic bacteria in aquati	国,周丽敏,王斌,高洁,赵颖	Limin,Wang Bin,Gao Jie,Zha	首都公共卫生	capital public health	7	1	35-37	2013	sn.1673-7830.2013.01.013
3557	市售食品中沙门菌污染状	ination in commercially ava	金晓凤,叶红萍.	Jin Xiaofeng,Ye Hongping.	浙江预防医学	hejiang Preventive Medicin	25	10	60-62	2013	sn.1007-0931.2013.10.018
3558	由沙门菌引起的食物中毒	case of food poisoning ca	杜航.	Du Hang.	社区医学杂志	urnal of Community Medici	11	22	73-74	2013	-
3559	感染性腹泻312例临床特点	rhea312Analysis of clinical	滕晔.	Teng Ye.	中外健康文摘	hese and Foreign Health Di	-	20	109-110	2013	sn.1672-5085.2013.20.100
3560	料、配合料中沙门氏菌的	monella in feed raw materi	阿力腾才斯克.	altencaisk.	新疆畜牧业	Xinjiang animal husbandry	-	3	32-33	2013	sn.1003-4889.2013.03.010
3561	区3种生吃蔬菜中肠道致	gation on intestinal pathog	丁宏伟,代露露,曹玉民,胡梅	ngwei,Dai Lulu,Cao Yumin,	实用预防医学	ractical preventive medicin	20	12	1444-1446	2013	sn.1006-3110.2013.12.012
3562	一起食物中毒的调查报告	on report on a case of food	李建庆.	Li Jianqing.	健康大视野	Healthy vision	21	18	690-690	2013	-
3563	鸡屠宰过程中肠炎沙门氏菌	nation during the slaughte	邓树轩,叶朗光,蔡铭升.	uxuan,Ye Langguang,Cai Mi	中国动物检疫	China Animal Quarantine	30	7	41-42	2013	n.1005-944X.2013.07.015
3564	门菌Ⅱ食物中毒的追溯调	nd analysis of a case of Sal	卢俊荣,王春东,刘金华.	rong,Wang Chuntong,Liu J	山西医药杂志（下半月版）	cal Journal (Second Half Mo	42	8	883-884	2013	-
3565	菌多重耐药和Ⅰ型整合子	resistance and type Ⅰ inte	赵红玉,张中文,候晓林,吴	u,Zhang Zhongwen,Hou Xi	中国农学通报	na Agricultural Science Bull	-	32	39-43	2013	sn.1000-6850.2013.32.008
3566	区常见动物传染病病原菌	enic bacteria of common a	李静姬,王健泽.	Li Jingji,Wang Jianze.	吉林农业科技学院学报	gricultural Science and Tec	22	1	1-3,6	2013	sn.1674-7852.2013.01.001
3567	预防控制中心食品及公共场	rking in food and public pl	俞建萍,魏良敏,朱芝萍.	nping,Wei Liangmin,Zhu Zh	健康之路	road to health	12	5	360-360	2013	sn.1671-8801.2013.05.406
3568	沙门菌引起食物中毒的检	of a case of food poisoning	谢文忠,郭加团,欧秀华.	enzhong,Guo Jiatuan,Ou Xi	实验与检验医学	imental and Laboratory Me	31	1	78-79	2013	sn.1674-1129.2013.01.030
3569	放养鸡及鸡蛋沙门氏菌带菌	arriage in free-range chicke	楠,陈晶,田祥宇,王忠,袁建	Jing,Tian Xiangyu,Wang Zh	中国动物检疫	China Animal Quarantine	-	10	61-65	2013	n.1005-944X.2013.10.025
3570	养基在从业人员健康体检	onella chromogenic culture	马永,陈建辉,杨宁.	ayon,Chen Jianhui,Yang Nin	医学信息（下旬刊）	edical information (late issu	26	12	91	2013	sn.1006-1959.2013.12.118
3571	一起伤寒沙门氏菌引起的	ood poisoning caused by Sa	赛娜瓦尔·巴合提,刘清.	Senavar·Bahti,Liu Qing.	疾病预防控制通报	se Prevention and Control	-	3	63-64,72	2013	-
3572	门菌引起食物中毒的检验结	of a case of food poisoning	陈玉红.	Chen Yuhong.	检验医学与临床	boratory medicine and clini	10	2	206-207	2013	sn.1672-9455.2013.02.038
3573	常州市市售食品食源性致病	nic contamination of comm	杜强,张露杰,徐晓怡.	Qiang,Zhang Lujie,Xu Xiao	江苏预防医学	iangsu Preventive Medicine	24	4	11-13	2013	sn.1006-9070.2013.04.005
3574	氏菌引起的群体性食物中毒	on a mass food poisoning c	马有俊,锁忠萍.	Ma Youjun,Suo Zhongping.	健康必读（中旬刊）	-read for health (mid-term	12	7	597-597	2013	-
3575	酒泉市食品中食源性致病菌	sis of foodborne pathogeni	李燕,鲁浩斌,高银平,王莹	u Haobin,Gao Yinping,War	中国卫生产业	China health industry	-	13	113-114	2013	-
3576	氏菌引起食物中毒事件的分	ysis of a food poisoning inc	唐爽,杨立娟.	Tang Shuang,Yang Lijuan.	中国伤残医学	Chinese Disability Medicine	-	12	364-364,365	2013	-
3577	年大连市食源性致病菌监测	nalysis of foodborne patho	王凡,郑晓南,张磊.	g Fan,Zheng Xiaonan,Zhang	中外健康文摘	hese and Foreign Health Di	-	20	76-77,78	2013	sn.1672-5085.2013.20.068
3578	本甲型副伤寒沙门菌检出	esistance of Salmonella Pa	王云芬,杨兴林.	Wang Yunfen,Yang Xinglin.	贵州医药	Guizhou Medicine	37	3	265-266	2013	N.1000-744X.2013.03.032
3579	州市食源性疾病哨点医院监	g results of foodborne dise	邹碧.	Zou Bi.	医学信息（下旬刊）	edical information (late issu	26	15	561	2013	sn.1006-1959.2013.15.800
3580	感染性腹泻的流行病学调	on and analysis of a case o	锦娟,谢青梅,李宗谨,李军	uan,Xie Qingmei,Li Zongjin	健康必读（下旬刊）	ust-read for health (late issu	-	1	355-355	2013	-
3581	食品中检出朗根萨尔查沙门	ella Langensalsa detected	小林,郑国顺,王顺东,罗德维	heng Guoshun,Wang Shun	中国保健营养	Chinese health nutrition	23	1	40-41	2013	sn.1004-7484.2013.01.040
3582	食水产品中微生物污染情况	obial contamination in raw	刘荣荣.	Liu Rongrong.	河北医药	Hebei Medicine	35	14	2193-2194	2013	sn.1002-7386.2013.14.065
3583	行区5岁以下儿童腹泻病病	s of pathogenic spectrum o	名,孙乔,傅益飞,朱渭萍,薛	Qiao,Fu Yifei,Zhu Weiping,	上海预防医学	hanghai Preventive Medicin	-	11	602-605	2013	-
3584	市场肉鸡屠宰过程中肠炎沙	aughtering process of broil	邓树轩,叶朗光.	Deng Shuxuan,Ye Langguang	畜牧与饲料科学	Livestock and feed science	34	2	128	2013	sn.1672-5190.2013.02.058
3585	宴引起疑似食物中毒的调	suspected food poisoning c	王晓东.	Wang Xiaodong.	健康大视野	Healthy vision	21	5	714	2013	-
3586	料中沙门菌的分离鉴定	esistance analysis of Salmo	余容,刘耀敏,杜雪莉,颜其贵	g,Liu Yaomin,Du Shirley,Ya	中国畜牧兽医	mal husbandry and veterina	40	12	204-207	2013	sn.1671-7236.2013.12.043
3587	2012年腹泻病症候群病原	ysis of annual diarrhea syn	张林,胡云晖.	Zhang Lin,Hu Yunhui.	内蒙古中医药	ngolia Traditional Chinese	32	33	82-83	2013	-

3588	区牦牛源沙门菌血清型及	of Salmonella originating f	张斌,朱晓霞,岳华,汤承.	in,Zhu Xiaoxia,Yuehua,Tang	畜牧兽医学报	mal Husbandry and Veteri	44	7	1167-1172	2013	sn.0366-6964.2013.07.024
3589	伤寒沙门氏菌引发食物中	se of food poisoning cause	,郑波,王君,刘平,邹小娟,	Bo,Wang Jun,Liu Ping,Zou X	健康大视野	Healthy vision	21	18	691-691	2013	-
3590	-2012年细菌性食物中毒检	lysis of annual bacterial fo	胜中,叶雪仪,李宇聪,杨丽	zhong,Ye Xueyi,Li Yucong,Y	国际医药卫生导报	ational Medical and Health	19	19	3076-3078	2013	sn.1007-1245.2013.19.049
3591	医院院内感染病原菌分布	hogenic bacteria in general	谭斌,张弦,薛健,周锡鹏,李	Bin,Zhang Xian,Xue Jian,Zh	中华流行病学杂志	inese Journal of Epidemiol	34	11	1134-1138	2013	n.0254-6450.2013.011.020
3592	寒并发多器官衰竭患者的	with enteric typhoid fever	邓长升,孙谋,赵子瑜,吴文	ng,Sun Mou,Zhao Ziyu,Wu	中华危重病急救医学	e critical care emergency m	25	12	763	2013	sn.2095-4352.2013.12.017
3593	年开封市肉鸡中沙门菌污	mination status in broiler c	恭举,杨如璞,刘杰,巩飚,陈	ang Rupu,Liu Jie,Gong Biao	中华预防医学杂志	se Journal of Preventive Me	47	5	475-476	2013	sn.0253-9624.2013.05.023
3594	产加工环节沙门菌的污染	distribution in broiler chic	,乔昕,杨小蓉,陈玉贞,裴	g Xiaorong,Chen Yuzhen,P	中华预防医学杂志	se Journal of Preventive Me	47	5	435-438	2013	sn.0253-9624.2013.05.012
3595	中国其他感染性腹泻监测	uation of surveillance of ot	刘海霞,张静.	Liu Haixia,Zhang Jing.	中华预防医学杂志	se Journal of Preventive Me	47	4	328-332	2013	sn.0253-9624.2013.04.009
3596	我院儿童细菌性腹泻病原	nd drug susceptibility of ch	胡云海,范忠祥,杜成雪.	hai,Fan Zhongxiang,Du Che	医药前沿	Medical Frontier	-	11	70-71	2013	-
3597	寒、副伤寒高发区发热症	with high incidence of typh	诚,张静,孙军玲,王子军,	,Zhang Jing,Sun Junling,Wa	中华流行病学杂志	inese Journal of Epidemiol	34	3	254-258	2013	sn.0254-6450.2013.03.012
3598	区2011年和2012年食源性	anhe2012Annual surveillan	年,潘朝庆,黄华国,石威,谢	Chaoqing,Huang Huaguo,S	医药前沿	Medical Frontier	-	7	22-23	2013	-
3599	菌所致食源性疾病暴发疫	odborne disease outbreak	,许静静,王路梅,童晶,张	gjing,Wang Lumei,Tong Jin	中华预防医学杂志	se Journal of Preventive Me	47	2	160-163	2013	sn.0253-9624.2013.02.014
3600	年虹口区生鲜食品沙门菌	detection of fresh food in	陈真,张静,沈静.	Chen Zhen,Zhang Jing,quiet	医药前沿	Medical Frontier	-	3	359	2013	-
3601	12年餐饮单位自制熟肉制	alysis on Testing Results o	凌琳,秦立强.	Ling Lin,Qin Liqiang.	医药前沿	Medical Frontier	-	3	368-369	2013	-
3602	糖尿病并发细菌感染的临	ses of diabetes complicate	杨杰,李绍生.	Yang Jie,Li Shaosheng.	医药前沿	Medical Frontier	-	2	188	2013	-
3603	市售鲜鸭蛋细菌污染的调	ntamination of commercia	洋,彭义,刘力,彭祥伟,朱艳	Peng Yi,Liu Li,Peng Xiangw	畜禽业	vestock and poultry indust	-	4	60-61	2013	sn.1008-0414.2013.04.043
3604	城市食品中食源性致病菌	orne pathogenic bacteria	郭树荣,张岚,吴双霞,王静	g,Zhang Lan,Wu Shuangxia	长治医学院学报	nal of Changzhi Medical Co	27	1	19-21	2013	sn.1006-0588.2013.01.006
3605	市2008年腹泻病原菌监测	nnual surveillance analysis	王润莲,刘宝莲.	Wang Runlian,Liu Baolian.	临床合理用药杂志	l of Rational Clinical Use of	6	21	20-21	2013	sn.1674-3296.2013.21.013
3606	沙门氏菌分离鉴定和药敏	and drug susceptibility tes	段培培.	Duan Peipei.	中国畜牧兽医文摘	mal Husbandry and Veterin	-	8	34-34,35	2013	-
3607	血行感染革兰阴性杆菌的	monitoring of Gram-negativ	萍,徐俊,陶云珍,王运中,朱	,Tao Yunzhen,Wang Yunzh	临床儿科杂志	Journal of Clinical Pediatric	31	4	339-342	2013	sn.1000-3606.2013.04.011
3608	料中的大肠菌群及沙门氏	coliforms and salmonella i	志琴,张晓红,李春,马亚楠,	,Zhang Xiaohong,Li Chun,M	畜牧与饲料科学	Livestock and feed science	34	6	35-37	2013	sn.1672-5190.2013.06.013
3609	氏菌引起的食源性疾病的	gation of a foodborne illne	王琴.	Wang Qin.	疾病预防控制通报	se Prevention and Control	-	3	65-66	2013	-
3610	起校园细菌性食物中毒的	of bacterial food poisoning	王晓英.	Wang Xiaoying.	健康必读（中旬刊）	read for health (mid-term	12	5	116-116	2013	-
3611	感染性腹泻病原菌分布及	of pathogenic bacteria in	萌,尉秀霞,杨军勇,耿荣,	ei Xiuxia,Yang Junyong,Ger	实用预防医学	ractical preventive medici	20	8	900-903	2013	sn.1006-3110.2013.08.002
3612	品及饲料添加剂中沙门氏	monella in feed products a	鹏,李金磊,高延玲,邱富	Peng,Li Jinlei,Gao Yanling,	上海畜牧兽医通讯	nal Husbandry and Veterin	-	2	10-11	2013	sn.1000-7725.2013.02.004
3613	肠杆菌科细菌感染分布及	n and drug resistance of E	,张丽华,张菊芬,朱学海,	ng Lihua,Zhang Jufen,Zhu	中国感染控制杂志	ese Journal of Infection Co	12	5	377-380	2013	sn.1671-9638.2013.05.015
3614	寒沙门氏菌引起的食物中	oning caused by Salmonell	罗雪,刘天慧,康燕.	uo Xue,Liu Tianhui,Kang Ya	最新医学信息文摘（电子	edical information abstrac	-	20	344-344	2013	sn.1671-3141.2013.20.266
3615	大学教学设施卫生状况调	ealth status of teaching fac	邓怡卿,赵渝.	Deng Yiqing,Zhao Yu.	师范大学学报（自然科学	i Normal University (Natur	42	5	499-503	2013	sn.1000-5137.2013.05.010
3616	西灵山县食源性致病菌监	of foodborne pathogenic b	丁梅,杨毅修,丁霞.	Ding Mei,Yang Yixiu,Ding Xi	中外健康文摘	hese and Foreign Health Di	-	25	111-112	2013	-
3617	地区域食品的食源性致病	detection results in food i	贺欣.	He Xin.	甘肃科技纵横	u science and technology a	42	7	143-145	2013	sn.1672-6375.2013.07.051
3618	2008-2011年沙门菌感染	2011Epidemiological analy	,王怡珺,韩颖俊,王曙,顾	Yijun,Han Yingjun,Wang Sh	上海预防医学	hanghai Preventive Medici	25	1	4-6,16	2013	sn.1004-9231.2013.01.002
3619	12年广西鸡沙门氏菌感染	on salmonella infection in	胜兰,陆文俊,邹联斌,粟	u Wenjun,Zou Lianbin,Su Y	上海畜牧兽医通讯	nal Husbandry and Veterin	-	6	4-6	2013	sn.1000-7725.2013.06.002
3620	门氏菌引起食物中毒的病	of food poisoning caused	文天平.	Wen Tianping.	中国卫生产业	China health industry	-	14	98100	2013	-
3621	年全年市售食品食源性致	status of foodborne pathog	俊荣,王春东,毕红杰,赵玲	Wang Chuntong,Bi Hongjie,	山西医药杂志（下半月版）	cal Journal (Second Half M	42	6	269-270	2013	sn.0253-9926.2013.06.015
3622	州地区鸡白痢沙门氏菌耐	istance of Salmonella pullo	杨安龙.	Yang Anlong.	畜禽业	vestock and poultry indust	-	4	16-18	2013	sn.1008-0414.2013.04.010
3623	县市售食品中食源性致病	borne pathogenic bacteria	永红,傅剑羽,黄佳慧,华冬	ianyu,Huang Jiahui,Chinese	现代医药卫生	modern medicine and healt	29	5	675-676,678	2013	sn.1009-5519.2013.05.015
3624	食品中食源性致病菌污染	genic bacteria contaminati	,徐子强,唐建红,钟启丽,	qiang,Tang Jianhong,Chung	中国保健营养（中旬刊）	e health nutrition (mid-ter	-	10	842-843	2013	-

3625	北京地区儿童腹泻病原菌	genic bacteria in children w	梅,冯燕玲,薛冠华,闫超,张	nling,Xue Guanhua,Yan Ch	国际检验医学杂志	onal journal of laboratory	34	4	432-434	2013	sn.1673-4130.2013.04.020
3626	宴引起的沙门氏菌食物	monella food poisoning cau	秦田秀.	Qin Tianxiu.	广西医学	Guangxi Medicine	-	7	929-930	2013	ssn.0253-4304.2013.07.44
3627	沙门氏菌菌血症患者的用	use in patients with Salmo	刘云红,林海霞,官真水.	nhong,Lin Haixia,Guan Zhe	内蒙古中医药	ngolia Traditional Chinese	32	10	179-180	2013	sn.1006-0979.2013.10.203
3628	刺液中分离出肠炎沙门	Enteritidis isolated from hi	杨峰.	Yang Feng.	健康大视野	Healthy vision	21	16	508-508	2013	-
3629	宜宾市食源性致病菌污染	status of foodborne path	张红,易松涛,何俊,赵杰.	Hong,Yi Songtao,He Jun,Zh	中国当代医药	inese contemporary medic	20	7	151-152,154	2013	sn.1674-4721.2013.07.075
3630	年餐饮单位熟肉制品微生	crobial monitoring results	王艳,周丽萍,张晋.	ang Yan,Zhou Liping,Zhang	医药前沿	Medical Frontier	-	26	370-370	2013	sn.2095-1752.2013.26.455
3631	门氏菌食物中毒调查及	ng in Nandan County and	周青华.	Zhou Qinghua.	中国农村卫生	rural health in china	-	z1	319-320	2013	-
3632	沙门氏菌引起的食物中毒	case of food poisoning ca	曾甲庆,侯万乐,徐海峰.	Jiaqing,Hou Wanle,Xu Hai	医学信息	medical information	-	27	487-488	2013	-
3633	获得性腹泻病原菌菌谱及	of pathogenic bacteria in	严银燕,叶光勇.	Yan Yinyan,Ye Guangyong.	中国现代医生	modern chinese doctor	51	26	74-75,77	2013	-
3634	炎中沙门氏茵的分离鉴定	eptibility test of Salmonella	潘懿,黄宝学.	Pan Yi,Huang Baoxue.	广西畜牧兽医	mal Husbandry and Veterin	29	5	270-271	2013	-
3635	伤寒病例的沙门菌分离	ation of Salmonella from a	徐瑞萍,蒋奎英,徐敏.	Ruiping,Jiang Kuiying,Xu M	健康必读（下旬刊）	st-read for health (late iss	-	6	536-536	2013	-
3636	养标本检出病原菌类别及	ysis of pathogenic bacteria	王燕.	Wang Yan.	广西医学	Guangxi Medicine	35	1	98-99,103	2013	ssn.0253-4304.2013.01.35
3637	门氏菌的分离鉴定及其	dy of drug resistance of Sa	张秀芹,杜柏林,韩文瑜.	ang Xiuqin,dublin,Han Wen	饲料工业	Feed industry	-	18	46-48	2013	n.1001-991X.2013.18.012
3638	从业人员沙门氏菌携带	lla carriage among employ	,梁玉潮,梁卫桥,冯婉丽,	chao,Liang Weiqiao,Feng	当代医学	contemporary medicine	-	23	160-160,161	2013	sn.1009-4393.2013.23.124
3639	伤寒患者多项实验室检	aboratory test results in pa	覃安汝.	Qin Anru.	中国医药指南	Chinese Medicine Guide	-	22	216-217	2013	-
3640	养猪场猪源沙门氏杆菌的	originating from pigs in la	剑勇,谭艾娟,吕世明,方	yong,Tan Aijuan,Lu Shimin	贵州农业科学	uizhou Agricultural Science	41	4	109-111	2013	sn.1001-3601.2013.04.033
3641	食物中毒事故流行病学	on and analysis of a bacter	周立新.	Zhou Lixin.	中国保健营养（中旬刊）	health nutrition (mid-tern	-	8	532-533	2013	-
3642	宴引起的沙门氏菌食物	onella food poisoning cau	杨凤娟.	Yang Fengjuan.	中外健康文摘	hese and Foreign Health Di	-	49	44-45	2013	-
3643	和金黄色葡萄球菌引起的	ing caused by Salmonella	张桂芳,高斌,刘月淑,徐锡	Guifang,Gao Bin,Liu Yueshu	宁夏医科大学学报	al of Ningxia Medical Univ	35	2	232,封3	2013	sn.1674-6309.2013.02.041
3644	腹泻病原菌分布及耐药	rug resistance of pathogen	春红,卢悦琳,刁文连,陈锐	ong,Lu Yuelin,Diao Wenlian	检验医学与临床	boratory medicine and clini	-	14	1847-1848	2013	sn.1672-9455.2013.14.036
3645	源性沙门氏菌分离鉴定及	nce study of poultry-derive	陈培荣,刘锦妮,何斌.	hen Peirong,Liu Jinni,He Bi	湖北农业科学	Hubei Agricultural Sciences	52	17	4161-4163	2013	sn.0439-8114.2013.17.039
3646	腹泻病原微生物的感染特	eristics of pathogenic micr	焦继光,刘会霞.	Jiao Jiguang,Liu Huixia.	中国医药指南	Chinese Medicine Guide	-	20	44-45	2013	sn.1671-8194.2013.20.028
3647	沙门菌的分离鉴定与耐药	d drug resistance analysis	郑风英.	Zheng Fengying.	黑龙江畜牧兽医（下半月）	dry and Veterinary Medicin	-	12	84-85	2013	-
3648	品微生物污染状况调查	of microbial contamination	陈求欢.	Chen Qiuhuan.	农业与技术	Agriculture and Technology	33	8	228	2013	n.1671-962X.2013.08.186
3649	暴露食物中毒事件的流行	sis of a multiple exposure f	吴永,陈米娜,胡依红.	u Yong,Mina Chen,Hu Yiho	中国保健营养（中旬刊）	health nutrition (mid-tern	-	9	584-585	2013	-
3650	儿童腹泻54例诊治分析	ren54Case diagnosis and tr	张冰冰.	Zhang Bingbing.	中国实用医药	Chinese Practical Medicine	8	16	153	2013	sn.1673-7555.2013.16.110
3651	市食源性疾病哨点医院监	ults of foodborne disease s	陆祥彬,赵瑾.	Lu Xiangbin,Zhao Jin.	江苏预防医学	iangsu Preventive Medicin	24	1	21-23	2013	sn.1006-9070.2013.01.008
3652	门氏菌引起的食物中毒调	n a case of food poisoning	梁艳.	Liang Yan.	中外健康文摘	hese and Foreign Health Di	-	33	226-227	2013	-
3653	炎沙门菌引起的食物中毒	case of food poisoning ca	吴俊霞.	Wu Junxia.	江苏卫生保健	Jiangsu Health Care	15	5	29	2013	sn.1008-7338.2013.05.018
3654	沙门菌的分离·鉴定和耐	n pigs in Guangxi·Identifica	蹇慧,刘芳,覃永长.	n Hui,Liu Fang,Qin Yongcha	安徽农业科学	Anhui Agricultural Sciences	41	3	1123-1125	2013	sn.0517-6611.2013.03.071
3655	公共场所从业人员沙门菌	g employees working in fo	严华娟.	Yan Huajuan.	市建设理论研究（电子版	ch on Urban Construction (	-	15	-	2013	-
3656	灾区芦阳镇熟肉制品致病	teria sampling results of c	,杨成林,钟天辉,谭燕妮,	henglin,Zhong Tianhui,Tan	职业卫生与病伤	ccupational health and illne	28	6	356-357	2013	-
3657	县与流产相关羊病的调查	heep diseases related to a	玉,韩赟,王戈平,王顺林,	an Yun,Wang Geping,Wan	青海畜牧兽医杂志	mal Husbandry and Veteri	43	3	4-6	2013	sn.1003-7950.2013.03.002
3658	门氏菌食物中毒调查及	ng in Nandan County and	周青华.	Zhou Qinghua.	中国农村卫生	rural health in china	-	7	73-74	2013	-
3659	分离出肠炎沙门氏菌的1	ritidis isolated from blood	郭亚娜,齐林.	Guo Yana,Qi Lin.	最新医学信息文摘（电子	edical information abstract	-	26	194-194	2013	sn.1671-3141.2013.26.148
3660	熟肉制品中常见食源性致	f common foodborne path	秦武丽,吴林洪,徐雪英.	Wuli,Wu Linhong,Xu Xuey	医药前沿	Medical Frontier	-	18	389-390	2013	sn.2095-1752.2013.18.491
3661	沙门氏菌的分离鉴定试	ntification Test of Salmone	王伟.	Wang Wei.	中国畜牧兽医文摘	mal Husbandry and Veterin	-	5	50-50,3	2013	-

3662	11年北京市大兴区感染性	e of infectious diarrhea in	周丽敏,王斌,梁赢,白英丽	min,Wang Bin,Liang Ying,Ba	首都公共卫生	capital public health	7	3	124-127	2013	sn.1673-7830.2013.03.008
3663	肉产品中沙门菌血清型及	and drug resistance in poul	源,徐家芳,刘发东,辛秀	uan,Xu Jiafang,Liu Fadong,S	中国家禽	Chinese poultry	35	13	55-57	2013	sn.1004-6364.2013.13.019
3664	菌属的细菌学感染与药敏	and drug susceptibility an	燕青,赵元勋,徐和平,马晓	ng,Zhao Yuanxun,Xu Heping	实验与检验医学	imental and Laboratory Me	-	6	624-624	2013	sn.1674-1129.2013.06.048
3665	品中食源性致病菌污染状	nce of foodborne pathogen	,王岚,胡旃,刘建琪,张林青	,Hu Zhan,Liu Jianqi,Zhang	实用预防医学	ractical preventive medicin	20	1	16-18	2013	sn.1006-3110.2013.01.005
3666	家庄地区细菌性食物中毒	f pathogenic bacteria of ba	丽云,徐保红,王苋,吕国平	u Baohong,Wang Ran,Lu G	微生物学杂志	journal of microbiology	33	5	73-77	2013	sn.1005-7021.2013.05.016
3667	食源性疾病暴发的病原学	etiology of a foodborne d	唐雨德,魏德江,周东明,梁	Wei Dejiang,Zhou Dongmin	东南国防医药	heast National Defense Me	15	4	335-337	2013	n.1672-271X.2013.04.005
3668	INET血培养临床分离菌的	n and drug resistance of c	倪语星,孙景勇,徐英春,张	g,Sun Jingyong,Xu Yingchun	中国感染与化疗杂志	urnal of Infection and Che	13	4	241-247	2013	sn.1009-7708.2013.04.001
3669	年大连市食源性致病菌监	nalysis of foodborne patho	郑晓南,李瑞,王凡.	eng Xiaonan,Li Rui,Wang F	中外健康文摘	hese and Foreign Health Di	-	25	146-147	2013	-
3670	门菌病病原分离鉴定及耐	resistance detection of sal	建,陈绩,赵青剑,马鹏恩,王	en Ji,Zhao Qingjian,Ma Per	中国家禽	Chinese poultry	35	11	45-46	2013	sn.1004-6364.2013.11.014
3671	炎临床特征及脑脊液分析	uid analysis of purulent m	林贵喜,郑建明,黄天文,姚	eng Jianming,Huang Tianw	医学信息	medical information	26	5	355-356	2013	sn.1006-1959.2013.05.370
3672	猪源鼠伤寒沙门菌的分离	of Salmonella typhimurium	贡嘎,查果,拉珍,索朗斯珠	Check the fruit,Rajan,solang	中国兽医杂志	Chinese Veterinary Journal	49	6	44-45	2013	sn.0529-6005.2013.06.015
3673	威镇1起细菌性食物中毒的	Investigation and analysis	蒋奎.	Jiang Kui.	职业卫生与病伤	ccupational health and illne	28	4	249-251	2013	n.1006-172X.2013.04.020
3674	东聊城市食源性致病菌污	f foodborne pathogenic ba	红,董炳刚,梁胜楠,姜宁宁,	nggang,Liang Shengnan,Jia	中国保健营养（中旬刊）	e health nutrition (mid-term	-	9	753-754	2013	-
3675	品大肠杆菌O157:H7和沙	ts in Dongguan, Guangdon	伟彬,李永福,张险朋,黄炳	Li Yongfu,Zhang Xianpeng,H	中国兽医杂志	Chinese Veterinary Journal	49	9	69-71	2013	-
3676	培养病原菌的种类分析与	ribution of pathogenic bact	谢宁,苏汉珍,孙华英.	Ning,Su Hanzhen,Sun Huay	临床和实验医学杂志	f Clinical and Experimental	12	3	216-217	2013	sn.1671-4695.2013.03.025
3677	德尔卑沙门菌耐药谱及P	Salmonella delphi in Hunan	云,陈帅,胡旃,张林青,刘	Shuai,Hu Zhan,Zhang Lind	实用预防医学	ractical preventive medicin	20	8	915-918	2013	sn.1006-3110.2013.08.007
3678	发性食物中毒的病原菌检	thogenic bacteria in two c	杨梅霞.	Yang Meixia.	中国保健营养（中旬刊）	e health nutrition (mid-term	-	5	534-534	2013	-
3679	寒沙门菌对23种抗生素的	hi vs.twenty threeAntibiotic	王云芳.	Wang Yunfang.	吉林医学	Jilin Medicine	34	5	873	2013	sn.1004-0412.2013.05.064
3680	区从业人员携带的沙门菌	ty analysis of Salmonella ca	陆祥彬,徐妍.	Lu Xiangbin,Xu Yan.	中国保健营养（中旬刊）	e health nutrition (mid-term	-	8	27-28	2013	-
3681	门菌感染暴发流行病原的	ological agents in two outb	张婷,李国民,杨继先,陆洲	Ting,Li Guomin,Yang Jixian,	实用预防医学	ractical preventive medicin	20	1	96-98	2013	sn.1006-3110.2013.01.037
3682	沙门氏菌的危害分析、污	gation and prevention and	学硕,崔生辉,邢书霞,丁宏	uo,Cui Shenghui,Xing Shuxi	中国药事	hinese pharmaceutical affa	27	9	974-979	2013	-
3683	北塘区医院污水微生物检	s of wastewater from Beita	朱惠芳.	Zhu Huifang.	医学信息	medical information	-	14	358-358	2013	sn.1006-1959.2013.14.471
3684	92例小儿腹泻的病原体分	s of pathogens in children	李超强,朱凯欣,黄美燕,李丹	ang,Zhu Kaixin,Huang Meiy	检验医学与临床	boratory medicine and clini	-	24	3303-3304,3307	2013	sn.1672-9455.2013.24.026
3685	氏菌耐药基因检测与耐药	cken-derived Salmonella ar	李昌文,刘立新,李睿,罗鹏志	gwen,Liu Lixin,Li Rui,Luo P	中国预防兽医学报	rnal of Preventive Veterina	35	8	627-630	2013	ssn.1008-0589.2013.08.06
3686	河南省禽病流行态势与防	ends and prevention and c	长庚,孙开冬,何向峰,慕桂	,Sun Kaidong,He Xiangfen	河南畜牧兽医（市场版）	andry and Veterinary Medi	34	4	14-16	2013	sn.1004-5090.2013.04.006
3687	沙门菌PFGE分型及耐药性	FGEType classification an	,刘万华,任娟娟,武宁,邱	anhua,Ren Juanjuan,Wunir	动物医学进展	vances in Veterinary Medi	-	11	1-5	2013	sn.1007-5038.2013.11.001
3688	市龙岗区感染性腹泻病原	thogenic bacteria in infect	春燕,崔海燕,胡琴,陈日	yan,Cui Haiyan,Hu Qin,Che	现代检验医学杂志	l of Modern Laboratory Me	28	4	136-138	2013	sn.1671-7414.2013.04.050
3689	自办家宴引起食物中毒的	oisoning caused by a self-h	王慧华,王立华.	Wang Huihua,Wang Lihua.	蛇志	snake ambition	25	3	287-289	2013	sn.1001-5639.2013.03.017
3690	林沙门氏菌引起食物中毒	f a case of food poisoning c	张金华,王桂红.	hang Jinhua,Wang Guihong	东南大学学报（医学版）	Southeast University (Medi	32	1	29-31	2013	sn.1671-6264.2013.01.007
3691	性腹泻病原菌的分布及其	analysis of pathogenic bac	杨乐,赵建.	Yang Le,Zhao Jian.	中国实用医药	Chinese Practical Medicine	8	2	137-138	2013	sn.1673-7555.2013.02.111
3692	猪肉产业链中沙门氏菌的	monella in the pork industr	小刚,刘书亮,韩新锋,吴聪	Liu Shuliang,Han Xinfeng,W	食品科学	food science	34	11	250-253	2013	pkx1002-6630-201311054
3693	腹泻患儿的病毒检验结果	virus test results in childre	王东升,付锋.	Wang Dongsheng,Fu Feng.	国际检验医学杂志	onal journal of laboratory	34	10	1319-1320	2013	sn.1673-4130.2013.10.061
3694	菌的血清型、耐药性和耐	nd drug resistance mechan	蕊蕊,张纯萍,徐士新,张启	Zhang Chunping,Xu Shixin,	农业生物技术学报	hal of Agricultural Biotechn	21	7	855-862	2013	sn.1674-7968.2013.07.012
3695	伤寒沙门菌致乳腺脓肿1	sed by Salmonella Paratyph	储平坤.	Chu Pingkun.	昆明医科大学学报	al of Kunming Medical Univ	34	1	145-146	2013	sn.1003-4706.2013.01.044
3696	的沙门氏菌耐药性与血清	ing from apparently healthy	,邵颖,方艳红,魏建忠,王	ng,Fang Yanhong,Wei Jianz	安徽农业大学学报	al of Anhui Agricultural Univ	-	4	-	2013	-
3697	猪沙门氏菌病的诊断和药	ptibility testing of swine sa	李珉珉,许建国,王传锋,张	u Jianguo,Wang Chuanfeng	畜禽业	vestock and poultry indust	-	3	62-64	2013	sn.1008-0414.2013.03.038
3698	品大肠杆菌O157：H7和沙	in Dongguan, GuangdongC	伟彬,李永福,张险朋,黄炳	Li Yongfu,Zhang Xianpeng,H	中国兽医杂志	Chinese Veterinary Journal	49	9	69-71	2013	sn.0529-6005.2013.09.027

3699	市售食品安全动态监测结	oring results of commercial	红,赵瑾,陈瑞英,张雪梅,马	o Jin,Chen Ruiying,Zhang X	江苏预防医学	iangsu Preventive Medicin	24	1	59-60	2013	sn.1006-9070.2013.01.027
3700	年全年市售食品食源性致	tatus of foodborne pathog	俊荣,王春东,毕红杰,赵玲	Vang Chuntong,Bi Hongjie,Z	山西医药杂志（下半月版）	cal Journal (Second Half Mo	42	3	269-270	2013	-
3701	场牛舍环境中沙门氏菌的i	Salmonella in the environm	战波,申欣,车宁宁,薛洋洋	hen Xin,Che Ningning,Xue Y	中国畜牧杂志	a Animal Husbandry Maga	49	8	20-23	2013	sn.0258-7033.2013.08.005
3702	西罗非鱼食源性致病菌及	bacteria and their drug sus	吴祥庆,吴伟军,吴明媛,黄国	ng,Wu Weijun,Wu Mingyua	南方农业学报	urnal of Southern Agricultu	44	11	1914-1918	2013	n.2095-1191.2013.11.1914
3703	不同规模牧场原料乳的微	v milk from pastures of diff	剧柠,柴佳丽,黄小晶.	Ning,Chai Jiali,Huang Xiaoji	农产品加工·学刊（下）	of agricultural products·Jou	-	8	114-115,118	2013	671-9646(X).2013.08.066
3704	门菌感染血清型及药敏分	ion serotype and drug susc	廖晓林.	Liao Xiaolin.	西部医学	western medicine	25	2	286-287	2013	sn.1672-3511.2013.02.047
3705	鸡屠宰环节胴体沙门菌污	mination status of broiler c	符晓梅,倪云龙,郑东宇,沈	mei,Ni Yunlong,Zheng Dong	江苏预防医学	iangsu Preventive Medicin	24	3	4-6	2013	sn.1006-9070.2013.03.002
3706	院血培养阳性病原菌分布	pathogenic bacteria with p	丹云建,陈海,闫中强,艾晓曼	n Hai,Yan Zhongqiang,Ai Xi	华临床医师杂志（电子版	urnal of Clinicians (electror	-	18	8477-8480	2013	sn.1674-0785.2013.18.096
3707	场沙门氏菌分离鉴定及耐	g resistance analysis of Sal	玉,张金茹,郝丽丽,闫延华	ang Jinru,Hao Lili,Yan Yanhu	贵州畜牧兽医	mal Husbandry and Veterin	37	2	10-12	2013	sn.1007-1474.2013.02.004
3708	肠炎沙门菌引起食物中毒的	of food poisoning caused	周晓红,严军,陈秀兰,姜洁	zhong,Yan Jun,Chen Xiulan	江苏卫生保健	Jiangsu Health Care	15	6	25-26	2013	sn.1008-7338.2013.06.016
3709	起沙门菌食物中毒的调查分	nalysis of a case of salmon	洪军,魏德江,李晶,张锦海	jun,Wei Dejiang,Li Jing,Zha	实用预防医学	ractical preventive medicin	20	6	716-718	2013	sn.1006-3110.2013.06.028
3710	沙门氏菌分离鉴定及耐药	-derived Salmonella and a	戚建华,吴植,刘俊栋,蔡丙严	ua,Wu Zhi,Liu Jundong,Cai	江苏农业科学	iangsu Agricultural Science	-	10	159-160,161	2013	sn.1002-1302.2013.10.061
3711	)～2012年沙门菌流行特征	epidemic characteristics a	杨燕,孙长贵,陈晓,余斐,陈瑜	in Changgui,Chen Xiao,Yu F	临床检验杂志	journal of clinical testing	31	8	621-624	2013	-
3712	市2009-2011年食品安全监	-2011Analysis of annual fo	丁惠萍,惠亚鹏,俞志丽.	g Huiping,Hui Yapeng,Yu Z	宁夏医学杂志	Ningxia Medical Journal	35	1	64-65	2013	sn.1001-5949.2013.01.031
3713	菌和沙门氏菌的分离鉴定	stance detection of Escheri	燕霞,陈冬杰,邹海鹏,迟明	en Dongjie,Zou Haipeng,Ch	中国动物检疫	China Animal Quarantine	30	7	63-67	2013	n.1005-944X.2013.07.023
3714	沙门氏菌的分离鉴定及药敏	ug susceptibility test of Sal	乐,张桉潮,李昆鹏,张秀丽,梁	Yuchao,Li Kunpeng,Zhang	中国畜牧兽医	mal husbandry and veterina	40	7	167-171	2013	sn.1671-7236.2013.07.039
3715	鼠伤寒沙门菌引起的食物	oisoning caused by Salmon	礼,侯伟伟,肖倩茹,江连,刘	Weiwei,Xiao Qianru,Jiang	检验医学与临床	boratory medicine and clini	10	0	216	2013	-
3716	流感染沙门菌的血清型分布	g resistance of Salmonella i	产,梁超娟,孙华英,苏汉珍,黎	Chaojuan,Sun Huaying,Su	临床检验杂志	journal of clinical testing	31	6	480	2013	-
3717	儿腹泻病原菌分布及耐药性	nce of pathogenic bacteria	春红,卢悦琳,刁文连,陈锐	ong,Lu Yuelin,Diao Wenliar	检验医学与临床	boratory medicine and clini	-	14	-	2013	-
3718	慢性腹泻粪便标本培养结	sults of stool specimens in	杨秀莲.	Yang Xiulian.	宁夏医科大学学报	al of Ningxia Medical Univ	35	3	317-318	2013	sn.1674-6309.2013.03.029
3719	F至2012年食品监测网结果	Analysis of annual food m	郭勇峰,王敬辉,吉彦莉.	Yongfeng,Wang Jinghui,Ji Y	中国保健营养（中旬刊）	e health nutrition (mid-tern	-	12	589-590	2013	-
3720	年食源性致病菌污染监测	athogenic bacteria contam	李燕,郭华荣,高银平,何楠	Guo Huarong,Gao Yinping,H	卫生职业教育	Health Professions Educatio	31	10	132-134	2013	sn.1671-1246.2013.10.077
3721	明儿童医院小儿败血症病	and drug resistance of ped	南,肖曙芳,李斌,倪林仙,黄海	Shufang,Li Bin,Ni Linxian,	华临床医师杂志（电子版	urnal of Clinicians (electror	-	13	6090-6092	2013	sn.1674-0785.2013.13.070
3722	病合并脓肿诊治2例报道及	th abscess in emergency d	高雨松,陈旭岩.	Gao Yusong,Chen Xuyan.	中国急救医学	mergency medicine in chin	33	11	1053-1056	2013	sn.1002-1949.2013.11.025
3723	腹泻的210例病原微生物检	ren210Analysis of pathoge	韩晶.	Han Jing.	中国社区医师（医学专业）	munity physician (medical	15	7	225	2013	sn.1007-614x.2013.07.220
3724	门菌Ⅱ食物中毒的追溯调	nd analysis of a case of Sal	卢俊荣,王春东,刘金华.	rong,Wang Chuntong,Liu J	山西医药杂志（下半月版）	cal Journal (Second Half Mo	42	16	883-884	2013	-
3725	霍乱沙门菌败血症死亡一	ath from choleraesuis salm	李璐,胥婕.	Li Lu,Xu Jie.	华临床医师杂志（电子版	urnal of Clinicians (electror	-	15	7328-7329	2013	sn.1674-0785.2013.15.152
3726	起D群沙门菌属食物中毒分	of food poisoning caused	曲永健,李兆国,陈秀香.	ngjian,Li Zhaoguo,Chen Xiu	中国民康医学	nese People's Health Medic	25	14	109111	2013	sn.1672-0369.2013.14.065
3727	市鸡致病性沙门菌的分离	on of pathogenic salmonel	辉,李鹏,张桂荣,张恒军,文	eng,Zhang Guirong,Zhang H	畜牧与兽医	Husbandry and Veterinary M	45	5	111-112	2013	-
3728	内沙门氏菌对喹诺酮类药物	tance of Salmonella Indian	彦,赵红玉,刘宇,候晓林,吴国	Hongyu,Liu Yu,Hou Xiaolin,	中国人兽共患病学报	Chinese Journal of Zoonose	29	6	605-608	2013	sn.1002-2694.2013.06.016
3729	细菌性食物中毒病原菌检测	of Pathogens Caused by B	炳健,钟有光,黎柱荣,冼桂	ong Youguang,Lai Chu Win	健康之路	road to health	12	4	299-299	2013	sn.1671-8801.2013.04.340
3730	某地区98起细菌性食物中毒	Analysis of pathogenic bac	杨瑛.	Yang Ying.	中外医疗	Chinese and foreign medica	32	16	35-36	2013	sn.1674-0742.2013.16.021
3731	年咸阳市食源性致病菌监	ring results of foodborne pa	犁,王利,杨囡,秦龙,张俊君,王	i,Yang Nan,Qin Long,Zhang	食品安全质量检测学报	f Food Safety and Quality In	-	5	1525-1528	2013	-
3732	检便样中沙门氏菌的优化	onella in stool samples du	刘晓晨,徐品,张默宇.	Xiaochen,Xu Pin,Zhang Mo	口岸卫生控制	Port health control	18	3	21-23	2013	sn.1008-5777.2013.03.007
3733	菌的检测及其在肉骨粉中的	research on its growth ch	饶正华,王宏,张苏,肖志明	ua,Wang Hong,Zhang Su,Xi	农产品质量与安全	ultural product quality and	-	4	41-43	2013	sn.1674-8255.2013.04.010
3734	内容物沙门氏菌污染的检测	contents of egg shells sold	付倩,姚明,刘媛,刘宇贺,李有	,Yao Ming,Liu Yuan,Liu Yuh	食品安全质量检测学报	f Food Safety and Quality In	-	5	1505-1510	2013	-
3735	012年伤寒、副伤寒病原学	of annual typhoid and par	龚红明,曾玉明.	ong Hongming,Zeng Yumin	健康大视野	Healthy vision	21	10	39-40	2013	-

3736	乱沙门氏菌败血症27例临	septicemia in children27C	管建宏,沈莉,缪伶伶.	Jianhong,Shen Li,Miao Lin	山东医药	Shandong Medicine	53	10	97	2013	n.1002-266X.2013.10.039
3737	血流感染患者的病原菌分	enic bacteria in patients wi	覃凌,王慕云,汪利娥.	Ling,Wang Muyun,Wang L	中国临床新医学	hinese new clinical medicir	-	10	971-973	2013	ssn.1674-3806.2013.10.15
3738	禽源沙门菌的分布及血	analysis of poultry-derived	,石火英,吉贞颖,陆玮,杨	uoying,Ji Zhenying,Lu Wei	畜牧兽医学报	mal Husbandry and Veteri	44	2	329-332	2013	-
3739	两省鸡白痢沙门菌的分离	nce analysis of Salmonella	,石火英,吉贞颖,陆玮,杨	uoying,Ji Zhenying,Lu Wei	中国家禽	Chinese poultry	35	4	17-20	2013	sn.1004-6364.2013.04.006
3740	大兴区水产品致病菌监测	thogenic bacteria in aquati	国,周丽敏,王斌,高洁,赵颖	Limin,Wang Bin,Gao Jie,Zh	首都公共卫生	capital public health	7	1	35-37	2013	sn.1673-7830.2013.01.013
3741	市售食品中沙门菌污染状	ination in commercially ava	金晓凤,叶红萍.	Jin Xiaofeng,Ye Hongping.	浙江预防医学	hejiang Preventive Medicin	25	10	60-62	2013	sn.1007-0931.2013.10.018
3742	由沙门菌引起的食物中毒	case of food poisoning ca	杜航.	Du Hang.	社区医学杂志	urnal of Community Medici	11	22	73-74	2013	-
3743	感染性腹泻312例临床特点	rhea312Analysis of clinical	滕晔.	Teng Ye.	中外健康文摘	hese and Foreign Health Di	-	20	109-110	2013	sn.1672-5085.2013.20.100
3744	料、配合料中沙门氏菌的	monella in feed raw materi	阿力腾才斯克.	altencaisk.	新疆畜牧业	Xinjiang animal husbandry	-	3	32-33	2013	sn.1003-4889.2013.03.010
3745	区3种生吃蔬菜中肠道致	gation on intestinal pathog	宏伟,代露露,曹玉民,胡	ngwei,Dai Lulu,Cao Yumin,	实用预防医学	ractical preventive medicin	20	12	1444-1446	2013	sn.1006-3110.2013.12.012
3746	一起食物中毒的调查报告	on report on a case of foo	李建庆.	Li Jianqing.	健康大视野	Healthy vision	21	18	690-690	2013	-
3747	鸡屠宰过程中肠炎沙门氏	nation during the slaughte	邓树轩,叶朗光,蔡铭升.	uxuan,Ye Langguang,Cai Mi	中国动物检疫	China Animal Quarantine	30	7	41-42	2013	n.1005-944X.2013.07.015
3748	门菌Ⅱ食物中毒的追溯调	nd analysis of a case of Sal	卢俊荣,王春东,刘金华.	rong,Wang Chuntong,Liu J	西医药杂志（下半月版）	cal Journal (Second Half M	42	8	883-884	2013	-
3749	菌多重耐药和Ⅰ型整合子	resistance and type Ⅰ int	赵红玉,张中文,候晓林,吴	u,Zhang Zhongwen,Hou Xi	中国农学通报	na Agricultural Science Bul	-	32	39-43	2013	sn.1000-6850.2013.32.008
3750	常见动物传染病病原菌	enic bacteria of common a	李静姬,王健泽.	Li Jingji,Wang Jianze.	吉林农业科技学院学报	gricultural Science and Tec	22	1	1-3,6	2013	sn.1674-7852.2013.01.001
3751	预防控制中心食品及公共	rking in food and public pl	俞建萍,魏良敏,朱芝萍.	nping,Wei Liangmin,Zhu Zh	健康之路	road to health	12	5	360-360	2013	sn.1671-8801.2013.05.406
3752	沙门菌引起食物中毒的检	of a case of food poisoning	谢文忠,郭加团,欧秀华.	enzhong,Guo Jiatuan,Ou X	实验与检验医学	mental and Laboratory Me	31	1	78-79	2013	sn.1674-1129.2013.01.030
3753	放养鸡及鸡蛋沙门氏菌带	rriage in free-range chicke	楠,陈晶,田祥宇,王忠,袁	Jing,Tian Xiangyu,Wang Zh	中国动物检疫	China Animal Quarantine	-	10	61-65	2013	n.1005-944X.2013.10.025
3754	养基在从业人员健康体检	onella chromogenic culture	马永,陈建辉,杨宁.	ayon,Chen Jianhui,Yang Ni	医学信息（下旬刊）	edical information (late iss	26	12	91	2013	sn.1006-1959.2013.12.118
3755	一起伤寒沙门氏菌引起的	od poisoning caused by Sa	赛娜瓦尔·巴合提,刘清.	Senavar·Bahti,Liu Qing.	疾病预防控制通报	se Prevention and Control	-	3	63-64,72	2013	-
3756	门菌引起食物中毒的检验	of a case of food poisoning	陈玉红.	Chen Yuhong.	检验医学与临床	boratory medicine and clini	10	2	206-207	2013	sn.1672-9455.2013.02.038
3757	常州市市售食品食源性致	nic contamination of comm	杜强,张露杰,徐晓怡.	Qiang,Zhang Lujie,Xu Xia	江苏预防医学	iangsu Preventive Medicin	24	4	11-13	2013	sn.1006-9070.2013.04.005
3758	氏菌引起的群体性食物中	on a mass food poisoning	马有俊,锁忠萍.	Ma Youjun,Suo Zhongping.	健康必读（中旬刊）	read for health (mid-term	12	7	597-597	2013	-
3759	酒泉市食品中食源性致病	sis of foodborne pathogen	李燕,鲁浩斌,高银平,王莹	u Haobin,Gao Yinping,Wa	中国卫生产业	China health industry	-	13	113-114	2013	-
3760	氏菌引起食物中毒事件的	ysis of a food poisoning in	唐爽,杨立娟.	Tang Shuang,Yang Lijuan.	中国伤残医学	Chinese Disability Medicine	-	12	364-364,365	2013	-
3761	年大连市食源性致病菌监	nalysis of foodborne patho	王凡,郑晓南,张磊.	g Fan,Zheng Xiaonan,Zhan	中外健康文摘	hese and Foreign Health Di	-	20	76-77,78	2013	sn.1672-5085.2013.20.068
3762	本甲型副伤寒沙门菌检出	resistance of Salmonella Pa	王云芬,杨兴林.	Wang Yunfen,Yang Xinglin.	贵州医药	Guizhou Medicine	37	3	265-266	2013	N.1000-744X.2013.03.032
3763	州市食源性疾病哨点医院	g results of foodborne dise	邹碧.	Zou Bi.	医学信息（下旬刊）	edical information (late iss	26	15	561	2013	sn.1006-1959.2013.15.800
3764	感染性腹泻的流行病学调	on and analysis of a case o	锦娟,谢青梅,李宗谨,李军	uan,Xie Qingmei,Li Zongjin	健康必读（下旬刊）	ust-read for health (late iss	-	1	355-355	2013	-
3765	食品中检出朗根萨尔查沙	ella Langensalsa detected	林,郑国顺,王顺东,罗德	heng Guoshun,Wang Shun	中国保健营养	Chinese health nutrition	23	1	40-41	2013	sn.1004-7484.2013.01.040
3766	食水产品中微生物污染情	obial contamination in raw	刘荣荣.	Liu Rongrong.	河北医药	Hebei Medicine	35	14	2193-2194	2013	sn.1002-7386.2013.14.065
3767	区5岁以下儿童腹泻病病	s of pathogenic spectrum	,孙乔,傅益飞,朱渭萍,薛	Qiao,Fu Yifei,Zhu Weiping,	上海预防医学	hanghai Preventive Medicir	-	11	602-605	2013	-
3768	市场肉鸡屠宰过程中肠炎	aughtering process of broi	邓树轩,叶朗光.	eng Shuxuan,Ye Langguan	畜牧与饲料科学	Livestock and feed science	34	2	128	2013	sn.1672-5190.2013.02.058
3769	宴引起疑似食物中毒的调	suspected food poisoning	王晓东.	Wang Xiaodong.	健康大视野	Healthy vision	21	5	714	2013	-
3770	料中沙门氏菌的分离鉴定	esistance analysis of Salm	容,刘耀敏,杜雪莉,颜其	g,Liu Yaomin,Du Shirley,Y	中国畜牧兽医	mal husbandry and veterin	40	12	204-207	2013	sn.1671-7236.2013.12.043
3771	2012年腹泻病症候群病原	ysis of annual diarrhea syn	张林,胡云晖.	Zhang Lin,Hu Yunhui.	内蒙古中医药	ngolia Traditional Chinese	32	33	82-83	2013	-
3772	区牦牛源沙门菌血清型及	of Salmonella originating f	张斌,朱晓霞,岳华,汤承.	in,Zhu Xiaoxia,Yuehua,Tan	畜牧兽医学报	mal Husbandry and Veteri	44	7	1167-1172	2013	sn.0366-6964.2013.07.024

3773	伤寒沙门氏菌引发食物中毒	se of food poisoning cause	,郑波,王君,刘平,邹小娟,	Bo,Wang Jun,Liu Ping,Zou X	健康大视野	Healthy vision	21	18	691-691	2013	-
3774	发危险因素1:2配对条件L	outbreaks1:2pairing condi	杨进;李永红;秦卫文;麦浩	Li Yonghong;Qin Weiwen	微生物学免疫学进展	s in Microbiology and Imr	41	3	41-44	2013	09/j.cnki.pmi.2013.03.023
3775	店镇一起伤寒暴发疫情的	break of typhoid fever in	;任达飞;冉启军;刘怡娅;	eng;Simon Yam;Ran Qiju	医学动物防制	medical animal control	29	2	162-164	2013	-
3776	县城区1起甲型副伤寒暴	ict1Investigation into the	黄革;李建明;	ellow leather;Li Jianming	预防医学论坛	reventive Medicine Foru	19	1	30-32	2013	sn.1672-9153.2013.01.025
3777	患者胆汁细菌群落限制性	hisms of bile bacterial com	黄洁,丁绍晖,孙敏,张捷.	ie,Ding Shaohui,Sun Min,Zh	中华消化杂志	Chinese Journal of Digestion	34	12	823-825	2014	sn.0254-1432.2014.12.007
3778	鼠伤寒沙门菌分子分型及其	nalysis of Salmonella typhi	,梁景涛,陈爱贞,廖华乐,何	Jingtao,Chen Aizhen,Liao H	中华流行病学杂志	inese Journal of Epidemiolo	35	9	1079-1080	2014	sn.0254-6450.2014.09.028
3779	物源性食品中沙门菌流行	ristics of Salmonella in anin	立冬,郭云昌,李薇薇,王岚,	Yunchang,Li Weiwei,Wang	中华预防医学杂志	se Journal of Preventive Me	48	8	699-704	2014	sn.0253-9624.2014.08.010
3780	疮合并感染性关节炎12例	with infectious arthritis12	乔琳,徐东,赵岩.	Qiao Lin,xudong,Zhao Yan.	中华风湿病学杂志	nese Journal of Rheumatol	18	9	589-592	2014	sn.1007-7480.2014.09.003
3781	年鼠伤寒沙门菌监测及菌	eillance of Salmonella Typh	霞,孙九峰,何冬梅,陈清,柯	ufeng,He Dongmei,Chen Q	中华流行病学杂志	inese Journal of Epidemiolo	35	8	917-924	2014	sn.0254-6450.2014.08.010
3782	94-2013年伤寒流行病学特	epidemiological characteris	进,林玫,闫梅英,曾竣,权怡	Mei,Yan Meiying,Zeng Jun,C	中华流行病学杂志	inese Journal of Epidemiolo	35	8	930-934	2014	sn.0254-6450.2014.08.012
3783	门菌耐药及分子流行病学	ecular epidemiological cha	传清,侯琦,黄峥,金汇明,肖	ng,Hou Qi,Huang Zheng,Jin	中华流行病学杂志	inese Journal of Epidemiolo	35	7	842-847	2014	sn.0254-6450.2014.07.019
3784	县食品中食源性致病菌监测	s of foodborne pathogenic	黄贤,旋惠娟,余贤国.	g Xian,Xuan Huijuan,Yu Xia	国际医药卫生导报	ational Medical and Health	20	4	478-479	2014	sn.1007-1245.2014.04.011
3785	大齐某农场沙门氏菌的分	ification of Salmonella fron	彭永刚,于倩,刘建华.	g Yonggang,Yu Qian,Liu Jian	中国动物检疫	China Animal Quarantine	-	3	55-57	2014	n.1005-944X.2014.03.022
3786	血清型特异性基因挖掘及生	type-specific gene mining	平,邓扬悟,郭燕华,梁长和	ng Yangwu,Guo Yanhua,Lian	江西理工大学学报	ngxi University of Science an	-	3	13-17	2014	cnki.jxlgdxxb.2014.03.003
3787	年自贡市食品食源性致病	ysis of foodborne pathogen	许烈英,崔敏,王红,陈燕梅,	,Cui Min,Wang Hong,Chen	预防医学情报杂志	of Preventive Medicine Info	30	6	494-497	2014	-
3788	性腹泻患者粪便标本的病原	organisms in fecal samples	苏映琼.	Su Yingqiong.	中国卫生产业	China health industry	11	6	24-25	2014	-
3789	猪伤寒沙门氏菌引起食物中	ng caused by Salmonella ty	汪伟.	Wang Wei.	中国现代药物应用	dern drug application in Ch	-	10	236-238	2014	-
3790	炎患儿尿中分离出一株伦敦	n isolated from the urine of	张静华,袁应华,孙奋勇.	inghua,Yuan Yinghua,Sun F	检验医学	laboratory medicine	-	5	492-492,497	2014	sn.1673-8640.2014.05.014
3791	年大连市食源性致病菌监测	nalysis of foodborne patho	郑晓南,陈玉凤,李瑞.	ng Xiaonan,Chen Yufeng,Li	微量元素与健康研究	e elements and health rese	31	5	42-43	2014	-
3792	宴引起的沙门氏菌食物中	nella food poisoning cause	周明,田本泉,王立军.	Ming,Tian Benquan,Wang	健康之路	road to health	-	9	366-366	2014	sn.1671-8801.2014.09.613
3793	德比尔沙门菌致食物中毒	oning caused by Salmonell	杜春兰,李少岩,高鹏.	Chunlan,Li Shaoyan,Gao Pe	河北医科大学学报	nal of Hebei Medical Unive	-	7	863-864	2014	sn.1007-3205.2014.07.047
3794	012年食源性致病菌监测结	s of annual foodborne path	袁丹,吴日明,王华洪.	Dan,Wu Riming,Wang Hua	国际检验医学杂志	onal journal of laboratory	-	5	622-624	2014	sn.1673-4130.2014.05.054
3795	州市市售食品食源性致病	rne pathogenic bacteria in	黄唐嘉.	Huang Tangjia.	中国城乡企业卫生	ygiene in urban and rural ar	29	4	34-36	2014	-
3796	患者血培养阳性病原菌分	ogenic bacteria positive in	梅红,陈冬梅,刘朝曦,张尉	Chen Dongmei,Liu Chaoxi,Z	中国老年学杂志	inese Journal of Gerontolo	34	5	1215-1217	2014	sn.1005-9202.2014.05.027
3797	市销鲜猪肉中致病菌的检	enic bacteria in fresh pork	王福红.	Wang Fuhong.	肉类工业	meat industry	-	9	38-40	2014	sn.1008-5467.2014.09.013
3798	沙门菌分离鉴定及系统进化	hylogenetic analysis of Salm	菊,刘书超,韩冠双,范才良	chao,Korean crown double	中国兽医杂志	Chinese Veterinary Journal	50	9	10-13,16	2014	sn.0529-6005.2014.09.003
3799	伤寒沙门菌感染腹泻患者	on-typhoid salmonella infe	兰芬,吴秀娟,孙各琴,慕月	u Xiujuan,Sun Geqin,Mu Y	中华医院感染学杂志	ournal of Hospital Infectiou	-	8	1874-1876	2014	.11816/cn.ni.2014-130196
3800	宝坻区食源性疾病监测结	orne disease surveillance r	耿素珍.	Geng Suzhen.	中国城乡企业卫生	ygiene in urban and rural ar	29	4	33-34	2014	-
3801	染性腹泻病原菌调查及其	ectious diarrhea in Tonglu C	震宇,王佳雯,沈芳,蒋爱萍	Wang Jiawen,Shen Fang,Jia	中国卫生检验杂志	ese Journal of Health Inspe	24	20	2977-2979	2014	-
3802	原菌的分离鉴定及药物敏	g sensitivity analysis of cani	红,王京仁,洪冬胜,刘媛媛,	Jingren,Hong Dongsheng,L	江苏农业科学	Jiangsu Agricultural Science	-	4	170-171,172	2014	sn.1002-1302.2014.04.065
3803	鞍山市食源性致病菌监测结	esults of foodborne patho	刘大江,尹晔.	Liu Dajiang,Yin Ye.	医学动物防制	medical animal control	30	10	1079-1081	2014	0.7629/yxdwfz201410006
3804	门氏菌引起食物中毒的病原	of food poisoning caused	马春平,马骏杰.	Ma Chunping,Ma Junjie.	深圳中西医结合杂志	egrated Traditional Chinese	24	11	21-22	2014	-
3805	管结石成分及微生物群落结	icrobial community structu	洁,丁绍晖,孙敏,许丁伟,张	g Shaohui,Sun Min,Xu Ding	世界华人消化杂志	rld Chinese Journal of Diges	-	17	2467-2472	2014	-
3806	门氏菌的分离、鉴定及其	nicity of Salmonella from c	利,赵红霞,李志芳,丁月霞	li,Zhao Hongxia,Li Zhifang,	中国畜牧兽医	mal husbandry and veterina	41	6	224-229	2014	-
3807	物致病菌沙门氏菌的测定能	mination of food microbial	严烨,舒静,刘静,邹力,张莉	,Shu Jing,Liu Jing,Zou Li,Zh	中外食品工业（下半月）	ign food industry (second h	-	10	10-12	2014	sn.1672-5336.2014.10.006
3808	炎沙门氏菌食物中毒事件	dis food poisoning incident	文,张晓健,谢明军,王芳,罗	iaojian,Xie Mingjun,Wang F	中国公共卫生管理	ese public health managen	-	1	63-64	2014	-
3809	012年食源性致病菌监测结	of annual foodborne patho	徐丹,肖冰,陈玉凤.	u Dan,Xiao Bing,Chen Yufen	医学动物防制	medical animal control	30	11	1275-1277	2014	0.7629/yxdwfz201411033

3810	中沙门氏菌的分离鉴定及	ella in the chilled chicken p	李静怡,冯赛祥,贺现辉,廖明	g Saixiang,He Xianhui,Liao	中国畜牧兽医	nal husbandry and veterina	41	12	62-66	2014	-
3811	狐狐沙门氏氏菌菌病的诊	treatment of salmonellosis	李亚龙.	Li Yalong.	现代农村科技	modern rural technology	-	1	39-39	2014	-
3812	兔沙门氏菌感染致结肠水肿	ma caused by Salmonella in	梁存军.	Liang Cunjun.	中国养兔	Raising rabbits in China	-	3	34	2014	sn.1005-6327.2014.03.011
3813	西沙门氏菌引起食物中毒的	case of food poisoning cau	王玮.	Wang Wei.	医学信息	medical information	-	29	336-337	2014	sn.1006-1959.2014.29.526
3814	源性肠炎沙门氏菌的鉴定分	s of Salmonella Enteritidis	宋志霞,王萍.	Song Zhixia,Wang Ping.	中国畜禽种业	estock and poultry breeding	10	10	146-147	2014	sn.1673-4556.2014.10.112
3815	东新区急性腹泻门诊病例	cs of acute diarrhea outpat	芒,薛曹怡,李文先,朱渭萍,朱	i,Li Wenxian,Zhu Weiping,Z	疾病监测	disease surveillance	29	5	349-353	2014	sn.1003-9961.2014.05.005
3816	鸽沙门菌病的病原分离与鉴	on of the pathogen of salm	亭,张文文,张莉,陆宇超,晏文	g Wenwen,Zhang Li,Lu Yuc	中国家禽	Chinese poultry	36	18	50-52	2014	sn.1004-6364.2014.18.014
3817	菌感染44例流行病学特征和	demiological characteristics	谭丽丽.	Tan Lili.	中国医药科学	Chinese Medical Sciences	-	10	120-121,197	2014	-
3818	菌血清分型及脉冲场凝胶	ectrophoresis fingerprint o	兴勇,刘成伟,朱应飞,周厚	g,Liu Chengwei,Zhu Yingfei,	中国食品卫生杂志	hinese Food Hygiene Journ	26	6	528-532	2014	0.13590/j.cjfh.2014.06.003
3819	料中沙门氏菌污染现状调	Salmonella contamination	吴宗芬,余萍.	Wu Zongfen,Yu Ping.	贵州畜牧兽医	nal Husbandry and Veterin	38	4	32-34	2014	sn.1007-1474.2014.04.012
3820	门氏菌引起的食物中毒分	esearch on food poisoning	史志瑛.	Shi Zhiying.	科技展望	Technology Outlook	-	18	210-210	2014	sn.1672-8289.2014.18.181
3821	沙门氏菌对抗菌药物的耐	monella Enteritidis from ch	彦,吕安,赵红玉,侯晓林,吴国	,Zhao Hongyu,Hou Xiaolin,	中国人兽共患病学报	Chinese Journal of Zoonose	30	1	17-22	2014	sn.1002-2694.2014.01.004
3822	海市食品中食源性致病菌	lts of foodborne pathogen	陈炯,顾其芳,刘诚,张曦.	ong,Gu Qifang,Liu Cheng,Zh	上海预防医学	hanghai Preventive Medicin	26	4	169-172	2014	-
3823	因聚餐引发食物中毒的调查	rt on food poisoning cause	徐基明,查正时.	Xu Jiming,Check timing.	热带病与寄生虫学	pical Diseases and Parasitol	-	4	252-253	2014	sn.1672-2302.2014.04.020
3824	产品出口中的沙门菌污染的	tamination in the export of	马艳,黄建生,林杰.	a Yan,Huang Jiansheng,Lin J	对外经贸实务	gn Economic and Trade Pra	-	12	68-69	2014	sn.1003-5559.2014.12.017
3825	蒙古自治区食源性致病菌	athogenic bacteria in Inner	金化瑞,乌伊罕,王利平.	n Huarui,Uyhan,Wang Lipin	中国卫生检验杂志	ese Journal of Health Inspe	24	11	1619-1622	2014	-
3826	阳市市售食品中食源性致	pathogenic bacteria in cor	吴晓红,罗赟,张代友,何玲玲	ng,Luo Yun,Zhang Daiyou,H	职业与健康	Occupation and health	30	13	1832-1833	2014	-
3827	内蒙古通辽市食源性致病	of foodborne pathogenic b	吴凤云,张连娟,张博,李慧静	n,Zhang Lianjuan,Zhang Bo	最新医学信息文摘（电子	edical information abstract	-	15	20-21,8	2014	sn.1671-3141.2014.15.010
3828	社区获得性细菌性腹泻病	alysis of pathogenic bacte	勇,贾天野,鲍春梅,张鞠玲,	,Jia Tianye,Bao Chunmei,Zh	传染病信息	fectious disease informatio	-	3	143-147	2014	-
3829	酒中微生物的卫生检验结	ction results of microorgan	高松.	Takamatsu.	河南预防医学杂志	n Journal of Preventive Me	-	4	345-346,353	2014	-
3830	伤寒沙门菌感染流行病学	burden of non-typhoid Saln	玮,黄蔚,梁骏华,卢玲玲,邓	ng Wei,Liang Junhua,Lu Lin	中国食品卫生杂志	hinese Food Hygiene Journ	26	3	217-222	2014	0.13590/j.cjfh.2014.03.004
3831	2011-2013年食源性致病菌	-2013Analysis of annual fo	周莹冰,罗昱玥,刘义萍,桂勇	bing,Luo Yuyue,Liu Yiping,	疾病监测与控制	sease surveillance and cont	8	7	405-407	2014	-
3832	年西安市细菌性腹泻病监	of bacterial diarrhea surveil	娟,魏晓光,吴守芝,刘继锋,	ei Xiaoguang,Wu Shouzhi,L	现代预防医学	modern preventive medicin	41	13	2465-2467	2014	-
3833	沙门氏菌耐药性及脉冲场	ophoresis typing of Salmon	广代,潘海建,苏旭东,吴丽萍	i,Pan Haijian,Su Xudong,Wu	中国畜牧兽医	nal husbandry and veterina	41	2	213-217	2014	-
3834	道感染病原菌分布及耐药	istance of pathogenic bact	菁,王春,许学斌,秦惠宏,孙	Chun,Xu Xuebin,Qin Huiho	中国感染与化疗杂志	ournal of Infection and Che	-	3	235-239	2014	-
3835	业链中沙门菌流行情况及其	sistance of Salmonella in th	英,赖婧,马立才,吴聪明,左	i Jing,Ma Licai,Wu Chongm	中国兽医学报	al of Chinese Veterinary Me	34	11	1790-1794	2014	-
3836	胚中沙门氏菌的分离及血清	entification of Salmonella f	赵玉梅,范玉霞.	Zhao Yumei,Fan Yuxia.	中国畜牧兽医文摘	nal Husbandry and Veterin	0	6	183	2014	-
3837	镇伤寒疫情暴发的流行病	igation of a typhoid fever c	,韦森,权怡,班秀花,曾竣,房	on,Quan Yi,Ban Xiuhua,Zen	应用预防医学	applied preventive medicine	20	2	87-89	2014	-
3838	院一起食物中毒的调查与	ghtenment of a Food Poiso	姚伟,尹红,王斌,李荣,韩红	in Hong,Wang Bin,Li Rong,	解放军预防医学杂志	tive Medicine of the People	32	6	527-528	2014	-
3839	感染性腹泻病原监测及流行	alysis of infectious diarrhe	媛,姜晓梅,王惠萱,覃敏,刘	iang Xiaomei,Wang Huixua	中国热带医学	Chinese tropical medicine	14	7	799-802	2014	-
3840	莱州市感染性腹泻病原菌	sis of Pathogens of Infectio	刘宗东,史玲杰,胡彬.	u Zongdong,Shi Lingjie,Hu B	中国保健营养（中旬刊）	e health nutrition (mid-term	24	2	906-907	2014	-
3841	牛场奶牛乳房炎病原菌分	c bacteria causing mastitis	张冰,朱江巍,张德显,杨彩霞	Zhu Jiangwei,Zhang Dexian,	现代畜牧兽医	nal husbandry and veterina	-	12	37-39	2014	sn.1672-9692.2014.12.011
3842	猪细菌性腹泻的病原分离与	of pathogens of bacterial	彭琼,杨胜坤,刘运平,肖明才	g Shengkun,Liu Yunping,Xia	中国畜禽种业	estock and poultry breeding	-	12	109-110	2014	sn.1673-4556.2014.12.085
3843	道微生物分离、鉴定及细	rganisms in captive giant p	娟,龙梅,汤纯香,周晓平,罗	ei,Tang Chunxiang,Zhou Xia	四川动物	sichuan animals	33	2	161-166	2014	sn.1000-7083.2014.02.001
3844	料的微生物污染监测及影	of experimental animal bed	张林青,胡旃,贾华云,王岚,汪	qing,Hu Zhan,Jia Huayun,W	实用预防医学	ractical preventive medicin	21	11	1300-1303	2014	n.1006-3110.2014.011.007
3845	正常携带沙门氏菌引起的食	soning caused by normally	李静.	Li Jing.	中国农村卫生	rural health in china	-	z2	379-379	2014	-
3846	仔猪沙门氏菌能分离鉴定	ceptibility test of Salmonel	黄晓慧,陆萍,焦安心,孙裴,姜	iaohui,Lu Ping,Jiao Anxin,S	猪业科学	Swine Science	31	1	94-96	2014	-

3847	菌血清型分布及药敏试验	rotype distribution and dr	郭志勤,王凤平,李丽娟,张	iqin,Wang Fengping,Li Lij	中国感染与化疗杂志	urnal of Infection and Che	-	6	532-534	2014	sn.1009-7708.2014.06.014
3848	国伤寒和副伤寒重点监测	urveillance data for typhoi	玲,张静,阚飙,闫梅英,常	g Jing,Kan Biao,Yan Meiyin	疾病监测	disease surveillance	29	11	875-879	2014	sn.1003-9961.2014.11.009
3849	市感染性腹泻流行特征及	nd pathogen surveillance	王艳琴,朱韩武.	Wang Yanqin,Zhu Hanwu.	社区医学杂志	urnal of Community Medic	12	12	10-11	2014	-
3850	江市早餐中食源性致病菌	us of foodborne pathogeni	刘磊,沙丽娜,吴双志,吕昌	ei,Sharina,Wu Shuangzhi,Lu	医药前沿	Medical Frontier	-	26	337-338	2014	sn.2095-1752.2014.26.335
3851	北京市海淀区食源性致病	lts of foodborne pathogen	光,谢利军,孙晓华,张海	ang,Xie Lijun,Sun Xiaohua,Z	现代检验医学杂志	l of Modern Laboratory M	29	2	154-155	2014	sn.1671-7414.2014.02.051
3852	鸡沙门菌和空肠弯曲菌污	ylobacter jejuni contamin	林,李娜,葛莉,刁习习,蔡	glin,Li Na,Ge Li,Diao Xixi,C	江苏预防医学	iangsu Preventive Medicin	25	1	75-76	2014	sn.1006-9070.2014.01.033
3853	梧州市区食品中食源性致	e pathogenic bacteria in f	莫桂姮,李梦映,周素芳.	uiheng,Li Mengying,Zhou S	职业与健康	Occupation and health	30	20	2905-2907	2014	-
3854	年盐城市食源性致病菌监	ance results of foodborne	殷俊,刘秀兰,吴巨飞.	Yin Jun,Liu Xiulan,Wu Jufei.	江苏预防医学	iangsu Preventive Medicin	25	2	78-79	2014	sn.1006-9070.2014.02.032
3855	年-2013年3种节日食品抽	Year3Analysis of sampling	文慧,王爱月,李永利,付玉	hui,Wang Aiyue,Li Yongli,Fu	中国卫生检验杂志	ese Journal of Health Insp	24	12	1794-1796	2014	-
3856	区鼠伤寒沙门菌耐药性和	typing of Salmonella typh	金玉娟,林文娟,刘渠,甘莉	ujuan,Lin Wenjuan,Liu Qu,G	中国病原生物学杂志	ese Journal of Pathogen Bi	9	4	343-346	2014	10.13350/j.cjpb.140414
3857	鼠伤寒沙门氏菌肠炎临床	sporadic cases of Salmonel	黄应希,周飞全.	Huang Yingxi,Zhou Feiquan	赣南医学院学报	nal of Gannan Medical Co	-	2	271-272	2014	-
3858	伤寒沙门菌败血症24例临	epticemia in Childrentwen	李迎飞,王险峰,王艳荣.	fei,Wang Xianfeng,Wang Y	吉林医学	Jilin Medicine	-	18	3911-3912	2014	-
3859	南平市食源性致病菌监测	esults of foodborne patho	周颖,郑鹃,吴春敏,郑雯菱	heng Juan,Wu Chunmin,Zh	中国初级卫生保健	primary health care in chin	28	4	89-91	2014	.1001-568X.2014.04.0037
3860	斯维的亚沙门菌引起食物	f food poisoning caused b	孟奇,刘中林,曹丽华,康丛	qi,Liu Zhonglin,Cao Lihua,K	中国卫生检验杂志	ese Journal of Health Insp	24	12	18161822	2014	-
3861	年辽宁省食源性致病菌监	esults of foodborne patho	文涛.	Wen Tao.	中国公共卫生管理	nese public health manage	30	5	739-740,743	2014	-
3862	起食物中毒病因的实验室	analysis of the cause of foo	盛冬萍,杨元斌,徐景野.	Dongping,Yang Yuanbin,Xu	中国食品卫生杂志	hinese Food Hygiene Jour	26	5	504-506	2014	.13590/j.cjfh.2014.05.024
3863	龙岗区小儿细菌病原学感	n status in children in Long	,邹惠英,陈日炳,贺天朵,	ying,Chen Ribing,He Tiandu	医学综述	medical review	20	6	1112-1114	2014	sn.1006-2084.2014.06.052
3864	锡市市售食品食源性致病	ne pathogenic bacteria in	高敏国,刘萍,孙钊,诸芸.	inguo,Liu Ping,Sun Zhao,Zh	江苏预防医学	iangsu Preventive Medicin	25	5	72-73	2014	sn.1006-9070.2014.05.033
3865	门菌食物中毒的检测与溯	ity analysis of a case of sal	,陈旭,李红叶,美丽克,左	en Xu,Li Hongye,Melic,Zuo	中国卫生检验杂志	ese Journal of Health Insp	24	23	3400-3401	2014	-
3866	并上消化道出血及伤寒临	e pancreatitis complicated	刘斌,潘蔚竑,李方,胡正雄	an Weihong,Li Fang,Hu Zhe	基层医学论坛	Primary Medicine Forum	-	25	3455-3456	2014	-
3867	省食源性非伤寒沙门菌疾	n of foodborne non-typhoi	让,周翌婧,郑东宇,吴高	Zhou Yijing,Zheng Dongyu,	江苏预防医学	iangsu Preventive Medicin	25	4	20-22	2014	sn.1006-9070.2014.04.008
3868	市部分酒店月饼微生物检	analysis of mooncakes in s	郭盈希,李丽华,王英姿.	o Yingxi,Li Lihua,Wang Yin	轻工科技	Light industry technology	30	3	105-106	2014	-
3869	市兴宾区市售食品中食源	rne pathogenic bacteria in	荣伟,梁战,巫紫琼,高翠文	g Zhan,Michelle Wu,Gao C	当代医药论丛	ontemporary Medicine Ser	-	20	179-180	2014	-
3870	区中小餐厅塑封消毒餐具	nfected tableware in small	张勇,桂燕华,陆岚.	ang Yong,Gui Yanhua,Lu La	中国初级卫生保健	primary health care in chin	28	1	87-88	2014	.1001-568X.2014.01.0033
3871	液中检出一株甲型副伤寒	lmonella Paratyphi A dete	李杰芬,陈静宜.	Li Jiefen,Chen Jingyi.	实用医技杂志	l of Practical Medical Tech	-	8	920-920	2014	-
3872	南省元江县甲型副伤寒流	on of paratyphoid A in Yua	松,张晓和,刘泽,周燕华,	ang Xiaohe,Liu Ze,Zhou Yan	寄生虫病与感染性疾病	c diseases and infectious	12	2	79-82	2014	-
3873	患者中非伤寒沙门菌分子	e of non-typhoidal Salmon	海,郭主声,张丽,陈桓练,周	sheng,Zhang Li,Chen Huan	中国食品卫生杂志	hinese Food Hygiene Jour	26	6	605-609	2014	.13590/j.cjfh.2014.06.022
3874	沙门菌感染致左髋关节滑	aused by salmonella chole	卢运照,韦丽艳.	Lu Yunzhao,Wei Liyan.	右江民族医学院学报	ujiang Medical College for	-	2	181-181	2014	sn.1001-5817.2014.02.060
3875	工地沙门氏菌食物中毒的	case of salmonella food p	吉喜军.	Ji Xijun.	最新医学信息文摘（电子	edical information abstrac	-	6	182-182	2014	sn.1671-3141.2014.06.135
3876	食非发酵豆制品微生物污	atus of ready-to-eat non-f	刘晓娟.	Liu Xiaojuan.	医学动物防制	medical animal control	30	8	905-906,908	2014	0.7629/yxdwfz201408028
3877	氏菌的分离鉴定及其耐药	and drug resistance analy	王晓楠,王俊峰,王龙,陈爽	han,Wang Junfeng,Wang L	东北农业大学学报	of Northeast Agricultural U	-	8	49-54,64	2014	sn.1005-9369.2014.08.008
3878	引发的肠炎沙门氏菌食物	Enteritidis food poisoning	王艳,张江萍.	Wang Yan,Zhang Jiangping	贵州医药	Guizhou Medicine	-	2	164-165	2014	N.1000-744X.2014.02.030
3879	起由沙门氏菌引起的食物	food poisoning caused by s	肖梅花,韩金安.	Xiao Meihua,Han Jinan.	内蒙古医学杂志	ner Mongolia Medical Jour	46	2	201-202	2014	-
3880	仔猪沙门氏菌的分离鉴定	ceptibility test of Salmonel	晓慧,陆萍,焦安心,孙裴,	iaohui,Lu Ping,Jiao Anxin,S	猪业科学	Swine Science	-	1	94-96	2014	sn.1673-5358.2014.01.027
3881	鸡沙门菌的携带与血清学	al infection status of Salmo	萍,宁宜宝,宋立,徐士新,高	ng,Ning Yibao,Song Li,Xu S	中国家禽	Chinese poultry	36	17	58-60	2014	sn.1004-6364.2014.17.018
3882	感染性腹泻细菌病原学监	ts of bacterial etiology of i	妹,周登仁,黄海,陈小佳,朱	ou Dengren,yellow sea,Ch	海南医学	Hainan Medicine	-	18	2776-2777	2014	.1003-6350.2014.18.1093
3883	食堂引起沙门氏菌食物中	almonella food poisoning	陈国庆.	Chen Guoqing.	中国保健营养（上旬刊）	a Health Nutrition (first iss	24	2	1088-1089	2014	1004-7484(s).2014.02.640

3884	物饲料中沙门氏菌分离鉴定	ation and Testing Report o	蒙振亩,潘懿.	Meng Zhenmu,Pan Yi.	饲料博览	Feed Expo	-	3	51-53	2014	-
3885	沙门菌流行特征及脉冲场	l electrophoresis molecula	王宏.	Wang Hong.	科技创新导报	and Technology Innovatior	-	1	254	2014	n.1674-098X.2014.01.209
3886	鸭沙门氏菌分离鉴定及药	usceptibility test of Salmor	颀,王传禹,谭红,许琳,赵蓉	Chuanyu,Tan Hong,Xu Lin	云南畜牧兽医	nal Husbandry and Veterina	-	4	10-12	2014	sn.1005-1341.2014.04.008
3887	化牛场犊牛沙门氏菌病的	t of salmonellosis in calves	黄仁惠,李霞,董文,徐建平	enhui,Li Xia,Dong Wen,Xu	中国畜禽种业	estock and poultry breeding	-	9	123-123	2014	sn.1673-4556.2014.09.099
3888	中金黄色葡萄球菌和沙门	reus and Salmonella from	陈萍,王佳男,冯书章,郭	ing,Wang Jianan,Feng Shu	中国兽药杂志	ese Journal of Veterinary D	-	10	14-17	2014	-
3889	县腹泻病症候群监测结果	syndrome surveillance resu	马翠琴,马丽,马楠.	Ma Cuiqin,Ma Li,Ma Nan.	现代预防医学	modern preventive medicin	41	9	1676-1677	2014	-
3890	制品和活禽中沙门氏菌血	of Salmonella in retail poul	郡慧霞,许学斌,周恒,刘志	u Xuebin,Zhou Heng,Liu Zh	中国畜牧兽医	mal husbandry and veterina	41	11	263-266	2014	-
3891	州市售食品致病菌监测与	ic bacteria in commercially	杨巧玲,王辉艳.	Yang Qiaoling,Wang Huiyan	中外健康文摘	hese and Foreign Health Di	-	12	25-26	2014	-
3892	寒沙门氏菌引起食物中毒	is of food poisoning caused	肖光明.	Xiao Guangming.	国际检验医学杂志	onal journal of laboratory	-	15	2120-2120	2014	sn.1673-4130.2014.15.077
3893	预包装食品常见致病菌分	mon pathogenic bacteria ir	顾春华,颜琪,仝伟建,杨晓	ua,Yan Qi,Tong Weijian,Yan	甘肃科技	ansu Science and Technolo	30	22	95-97	2014	sn.1000-0952.2014.22.033
3894	年我院食源性疾病监测结	borne disease surveillance	蒋拥军.	Jiang Yongjun.	医药与保健	Medicine and Healthcare	-	10	139-139	2014	-
3895	血中检出沙门氏菌的临床	Salmonella detected in the	史今凡.	Shi Jinfan.	内蒙古医科大学学报	f Inner Mongolia Medical U	-	s1	38-39	2014	-
3896	的非伤寒沙门菌分子分型	of non-typhoid Salmonella	王鸣柳,许学斌,崔晶花,杜	ingliu,Xu Xuebin,Cui Jinghu	疾病监测	disease surveillance	29	6	428-431	2014	sn.1003-9961.2014.06.004
3897	福州市健康人群携带沙门	nella serotypes carried by	梅,潘洁茹,齐文岚,庄玲,	n Jieru,Qi Wenlan,Zhuang L	职业与健康	Occupation and health	30	10	1400-1402	2014	-
3898	炎沙门菌食物中毒的调查	n on a case of Salmonella	韩永红.	Han Yonghong.	中国城乡企业卫生	ygiene in urban and rural a	29	4	53-54	2014	-
3899	沙门氏菌引起的食物中毒	n a case of food poisoning	陈翠华.	Chen Cuihua.	特别健康：下	Extraordinarily healthy: Nex	-	1	224	2014	-
3900	市零售鸡蛋沙门氏菌快速	n of Salmonella in retail eg	高娟,王晓亮,张传苹,叶绍	Xiaoliang,Zhang Chuanping	上海农业学报	hanghai Agricultural Journa	-	4	93-96	2014	sn.1000-3924.2014.04.027
3901	牛乳房炎病原菌分离鉴定	test of pathogenic bacteri	李清利,路延豪.	Li Qingli,Lu Yanhao.	北京农业	Beijing Agriculture	-	24	130-130	2014	sn.1000-6966.2014.24.113
3902	2年91株甲型副伤寒沙门菌	ysis of pathogenic characte	雯,古文鹏,李超群,杨建斌,	Wenpeng,Li Chaoqun,Yang	中国人兽共患病学报	Chinese Journal of Zoonose	30	1	49-53,57	2014	sn.1002-2694.2014.01.011
3903	耐药沙门菌PFGE分子分	in Liaoning ProvincePFGE	张旭,李雪,魏彤竹,刚宇晨	u,Li Xue,Wei Tongzhu,Gang	中国微生态学杂志	inese Journal of Microecolo	26	6	653-655	2014	381/j.cnki.cjm.201406008
3904	某县饮食服务从业人员沙	results of catering service	韦宏珍,张艳琼,梁秀媛.	zhen,Zhang Yanqiong,Liang	检验医学与临床	boratory medicine and clini	-	z2	219-221	2014	sn.1672-9455.2014.26.115
3905	广州市白云区食品微生物	bial contamination status i	苏碧华,徐建华,罗世武.	Bihua,Xu Jianhua,Luo Shiw	应用预防医学	applied preventive medicin	-	4	239-240	2014	n.1673-758X.2014.04.019
3906	猪霍乱沙门氏菌严重脓毒	fected with Salmonella ch	姚玲,王绍红,陈若冰.	g,Wang Shaohong,Chen Ru	贵州医药	Guizhou Medicine	-	12	1113-1114	2014	SN.1000-744X.2014.12.27
3907	门氏菌食物中毒事件流行	alysis of a salmonella food	牛,张世勇,刘立,庞志钊,	ang Shiyong,Liu Li,Pang Zhi	医学动物防制	medical animal control	30	10	1146-1147	2014	0.7629/yxdwfz201410026
3908	腹泻的致病菌及肠道病毒	eria and enteroviruses in in	谢春燕,胡琴,崔海燕,陈日	yan,Hu Qin,Cui Haiyan,Che	检验医学与临床	boratory medicine and clini	-	2	168-170	2014	sn.1672-9455.2014.02.010
3909	感染性腹泻病原学监测结	urveillance results of infect	育禾,徐丽萍,张朝阳,徐景	,Xu Liping,Zhang Chaoyang	现代实用医学	modern practical medicine	26	3	311-313	2014	sn.1671-0800.2014.03.043
3910	广西武鸣县食品微生物污	ontamination test results i	韦宏珍,张艳琼,梁秀媛.	zhen,Zhang Yanqiong,Liang	职业与健康	Occupation and health	30	15	2086-2088	2014	-
3911	腹泻常见病原菌的分离与	of common pathogenic ba	詹先强,俞建萍.	Zhan Xianqiang,Yu Jianping	福建畜牧兽医	al Husbandry and Veterina	-	1	7-9	2014	sn.1003-4331.2014.01.004
3912	沙门氏菌的分离与耐药性	tance analysis of Salmonel	杜雄伟,吴静,于凤娟,魏怡	iongwei,Wu Jing,Yu Fengju	江苏农业科学	iangsu Agricultural Science	-	10	278-279,280	2014	sn.1002-1302.2014.10.095
3913	源性病原菌主要流行株分	ain epidemic strains of food	,徐景野,胡荣华,沈玄艺,	gye,Hu Ronghua,Shen Xuar	中国卫生检验杂志	ese Journal of Health Inspe	24	10	1500-1503,1510	2014	-
3914	鸽源沙门氏菌的鉴定及	alysis of Salmonella origina	涛,纪桂云,张津津,江奕	uiyun,Zhang Jinjin,Jiang Yi	中国畜牧兽医	mal husbandry and veterina	41	7	207-211	2014	-
3915	该地生肉沙门氏菌污染	soning and Salmonella con	姬晓红,孙玲.	Ji Xiaohong,Sun Ling.	中国伤残医学	Chinese Disability Medicine	-	13	299-300	2014	.cnki.cjotadm.2014.13.275
3916	寒沙门氏菌污染皮蛋引起	ng preserved eggs contami	罗华.	Luo Hua.	中国保健营养（中旬刊）	e health nutrition (mid-term	24	4	2502-2503	2014	-
3917	饮单位自制凉菜细菌污染	nation of homemade cold	高璐,陈梦吟.	Gao Lu,Chen Mengyin.	中国卫生产业	China health industry	-	18	47-47,49	2014	-
3918	程中沙门氏菌的污染情况	and drug resistance durin	珍,刘书亮,赖海梅,韩新锋	en,Liu Shuliang,Lai Haimei,	食品科学	food science	35	17	214-219	2014	pkx1002-6630-201417041
3919	沙门氏菌引起食物中毒事件	od poisoning incident caus	邓远玲,陈丽萍,王平原,詹	anling,Chen Liping,Wang P	疾病监测与控制	ease surveillance and cont	8	8	488-489	2014	-
3920	毒血症病原菌及耐药性	genic bacteria and drug res	吴茜,李斌,李杨方,黄海林	ian,Li Bin,Li Yangfang,Huar	临床儿科杂志	Journal of Clinical Pediatric	-	7	629-632	2014	sn.1000-3606.2014.07.008

3921	省部分地区鸭源沙门菌分	ce of Salmonella originati	水斌,陈丽,梁秀川,黄玉兰	n,Chen Li,Liang Xiuchuan,M	现代预防医学	modern preventive medicin	41	17	3184-3187	2014	-
3922	学技术在一起食物中毒检测	ar biology techniques in fo	发,王爱红,杨元斌,叶硕,郭	Aihong,Yang Yuanbin,Ye S	中国食品卫生杂志	hinese Food Hygiene Journ	26	3	254-258	2014	.13590/j.cjfh.2014.03.011
3923	州市食品风险监测致病菌	teria results from food risk	陈宝林,王珥梅,盛军利.	Baolin,Wang Ermei,Sheng	现代预防医学	modern preventive medicin	41	21	3882-3883	2014	-
3924	淮区一次食品微生物污染	amination of primary food	高琦.	Gao Qi.	大家健康（中旬版）	one is healthy (mid-term ed	-	12	335-335	2014	-
3925	病人沙门菌血清学、耐药	ella serology, drug resistan	张晶,张欣强,梁兆铭,胡	g,Zhang Xinqiang,Liang Zha	现代预防医学	modern preventive medicin	41	15	2689-2692,2696	2014	-
3926	腹泻病中细菌的分离鉴定	eptibility test of bacteria in	栗卫东.	Li Weidong.	农民致富之友	riends of farmers getting ri	-	16	251-251	2014	sn.1003-1650.2014.16.228
3927	邯郸市熟肉制品微生物污	bial contamination of cook	郑秀清,赵丽萍,李卫东,	g Xiuqing,Zhao Liping,Li W	实用预防医学	ractical preventive medicin	21	3	330-331	2014	sn.1006-3110.2014.03.026
3928	菌临床分离株的耐药性及	homology analysis of clinic	夏梦,张智洁,刘勇.	Meng,Zhang Zhijie,Liu Yo	医学临床研究	medical clinical research	-	12	2311-2314,2317	2014	sn.1671-7171.2014.12.009
3929	门菌在人和动物间的传播	ent of Salmonella Aberdeen	陈智,周晓瑛,李晓虹,许	n Zhi,Zhou Xiaoying,Li Xia	疾病监测	disease surveillance	29	8	669-674	2014	-
3930	鸡生产加工环节沙门菌污	drug resistance in broiler	侯配斌,王勤,胡光春,李心	pin,Wang Qin,Hu Guangchu	卫生研究	health research	43	6	933-938	2014	-
3931	霍乱沙门菌耐药基因特点	resistance genes of Salmon	黄凯,余晓君,王晔.	uang Kai,Yu Xiaojun,Wang	实验与检验医学	imental and Laboratory Me	-	2	126-127,131	2014	sn.1674-1129.2014.02.007
3932	浩特市食品中五种食源性	status of five foodborne p	王素娟,王红霞.	Wang Sujuan,Wang Hongxia	最新医学信息文摘（电子	edical information abstract	-	3	6-8,13	2014	sn.1671-3141.2014.03.002
3933	染性腹泻主要肠道病原对	or intestinal pathogens cau	崔京辉,胡晓芬,吴本和,张	nghui,Hu Xiaofen,Wu Benh	疾病监测	disease surveillance	29	6	432-436	2014	sn.1003-9961.2014.06.005
3934	引起的肠炎沙门氏菌食物	nella Enteritidis food poison	冯秀,游胜,吴凯,曾勇.	Tourist attraction,Wu Kai,Z	应用预防医学	applied preventive medicine	-	3	194-194	2014	-
3935	年-2012年细菌性食物中毒	012Laboratory Analysis of	虞静,王洁,蒋逸群,戴林晔	g,Wang Jie,Jiang Yiqun,Dai	中国卫生检验杂志	ese Journal of Health Inspe	24	10	1465-1467	2014	-
3936	肠炎沙门氏菌食物中毒的	case of Salmonella Enterit	孙磊.	Sun Lei.	中国实用医药	Chinese Practical Medicine	-	23	273-274	2014	-
3937	场乳房炎的病原鉴定与药	and drug sensitivity test of	赵海涛.	Zhao Haitao.	中国畜禽种业	estock and poultry breeding	10	11	127-128	2014	sn.1673-4556.2014.11.097
3938	流市常见食品食源性致病	foodborne pathogenic bac	陈东军,赖兆斌,范谯萍.	Dongjun,Lai Zhaobin,fan谯	应用预防医学	applied preventive medicine	20	2	111-113	2014	-
3939	患者血中检出德比沙门	etected in the blood of a pa	胡黎娅,郭凤丽,周友全.	Liya,Guo Fengli,Zhou Youqu	海南医学	Hainan Medicine	-	13	2008-2008,2009	2014	n.1003-6350.2014.13.0780
3940	菌血清学分型及生物被膜	n formation ability of Salm	兰,艾佛德,刘芳,方小文,杨	,Liu Fang,Fang Xiaowen,Ya	畜牧与兽医	Husbandry and Veterinary M	46	7	1-5	2014	-
3941	伤寒沙门氏菌对21种抗生素	phi Atwenty oneAntibiotic s	梅鲜艳.	Plum blossoms.	大家健康（下旬版）	eryone is healthy (late editi	8	4	168	2014	-
3942	011年宜春市食品致病菌监	d analysis of food pathoge	魏雄杰,张众,尚春.	iongjie,Zhang Zhong,Shang	实验与检验医学	imental and Laboratory Me	-	2	209-211	2014	sn.1674-1129.2014.02.041
3943	牛乳房炎病原菌分离鉴定	test of pathogenic bacteri	雪梅,钱秋月,宋俊涛,陈	i,Qian Qiuyue,Song Juntao,	河南畜牧兽医（综合版）	y and Veterinary Medicine	35	10	6-7	2014	-
3944	食物中毒的流行病学分析	oratory investigation of Sa	陆月敏.	Lu Yuemin.	中国卫生检验杂志	ese Journal of Health Inspe	24	12	1788-1789	2014	-
3945	儿童病原学检测及药物敏	ction and drug sensitivity r	宋贤响,方代华,权艳秋.	anxiang,Fang Daihua,Quan	重庆医学	Chongqing Medicine	-	27	3684-3686	2014	sn.1671-8348.2014.27.053
3946	脏血管周上皮样细胞肿瘤	pithelioid cell tumor of the	白红莲,陈焕伟.	Bai Honglian,Chen Huanwe	中西医结合肝病杂志	ional Chinese and Western	-	3	176-176,193	2014	sn.1005-0264.2014.03.021
3947	急性腹泻病原微生物检验	microorganism test results f	阳正东.	Yang Zhengdong.	蚌埠医学院学报	rnal of Bengbu Medical Col	-	8	1107-1109,1110	2014	-
3948	染性腹泻患者沙门氏菌感	nella infection in patients w	南,汪伟山,林爱心,杨晓	ang Weishan,Lin Aixin,Yan	国际检验医学杂志	onal journal of laboratory	-	15	2049-2050	2014	sn.1673-4130.2014.15.035
3949	泻患者沙门菌同源性及耐	teristics of Salmonella in pa	学海,张丽,张丽华,郭主	,Zhang Li,Zhang Lihua,Guo	中国感染与化疗杂志	urnal of Infection and Che	-	4	319-322	2014	sn.1009-7708.2014.04.013
3950	12年大连市食源性致病菌	d analysis of foodborne pa	郑晓南,姜杰,李瑞.	heng Xiaonan,Jiang Jie,Li R	微量元素与健康研究	e elements and health rese	31	6	29-30	2014	-
3951	门氏菌菌型分布及耐药性	nella type distribution and	张广业,汪鹏,康倩.	Guangye,Wang Peng,Kang	医学信息	medical information	-	5	36-37	2014	sn.1006-1959.2014.05.042
3952	物中毒流行病学调查和实	ory testing analysis of food	韦宏珍,张艳琼,梁秀媛.	zhen,Zhang Yanqiong,Liang	医学动物防制	medical animal control	30	12	1412-1414	2014	0.7629/yxdwfz201412039
3953	感染性腹泻的志贺菌和沙	es and drug resistance in c	岳美娜,王云桥,赵仕勇,	a,Wang Yunqiao,Zhao Shiy	疾病监测	disease surveillance	29	5	364-368	2014	sn.1003-9961.2014.05.008
3954	沙门氏菌食物中毒事件的	of a salmonella food poiso	曾露滋,黄柏林,陆少佐.	Luzi,Huang Bolin,Lu Shao	内科	Internal Medicine	9	5	592-594	2014	-
3955	013年腹泻症候群病原菌检	results and analysis of pa	刘宗东,于九.	Liu Zongdong,Yu Jiu.	中国保健营养（中旬刊）	e health nutrition (mid-term	-	5	3247-3247	2014	-
3956	染性腹泻1080例病原体检	children1080Case pathog	黄玉华.	Huang Yuhua.	中国伤残医学	Chinese Disability Medicine	-	10	184-185	2014	-
3957	菌中志贺菌和沙门菌的分	tection methods of Shigell	轼,严杰,陈松,倪娜,顾洪	an Jie,Chen Song,Ni Na,Gu	中国微生态学杂志	inese Journal of Microecolo	26	10	1181-1184	2014	381/j.cnki.cjm.201410019

3958	同来源山夫登堡沙门菌分	nella sanfordenberg from c	李浩,梁蓓蓓,杨超杰,王建	g Beibei,Yang Chaojie,War	传染病信息	fectious disease informatic	-	5	292-295	2014	-
3959	病原菌检验结果104例临	athogen test results104Exa	戴小花.	Dai Xiaohua.	大家健康（下旬版）	eryone is healthy (late editi	-	11	418-419	2014	sn.1009-6019.2014.11.550
3960	江苏省食源性致病菌耐药	onitoring of foodborne path	沈赟,马恺,庄菱,符晓梅,郑	a Kai,Zhuang Ling,Fu Xiaon	江苏预防医学	iangsu Preventive Medicin	25	1	28-30	2014	sn.1006-9070.2014.01.010
3961	家庭聚餐食物中毒的调查	Analysis of Food Poisoning	王福春.	Wang Fuchun.	医学动物防制	medical animal control	30	10	1148-1149	2014	0.7629/yxdwfz201410027
3962	下不同血清型沙门氏菌	fferent serotypes of Salmo	冰璐,杨磊,孟祥芹,付晓平	ang Lei,Meng Xiangqin,Fu X	中国畜牧兽医	mal husbandry and veterina	41	10	230-237	2014	-
3963	相关多重耐药病原菌分析	bacteria associated with di	玲,王欢,陈素明,崔恩博,鲍	g,Wang Huan,Chen Suming	解放军医学院学报	People's Liberation Army M	35	9	887-889	2014	sn.2095-5227.2014.09.003
3964	市普陀区食源性致病菌监	lts of foodborne pathogeni	超,李勇,唐海丰,熊鹿言,李	i Yong,Tang Haifeng,Xiong	海南医学	Hainan Medicine	25	4	595-598	2014	.1003-6350.2014.04.0232
3965	-2013年志贺菌、沙门菌属	is of distribution and drug	刘红娟,查巍,祖方.	ongjuan,Zha Wei,ancestral	安徽医药	Anhui Medicine	-	10	1994-1995,1996	2014	sn.1009-6469.2014.10.065
3966	地区沙门菌食物中毒分离	racteristics of Salmonella f	云,郭玉梅,吕国平,王苋,剧	Yumei,Lu Guoping,Wang R	微生物学杂志	journal of microbiology	34	1	84-87	2014	sn.1005-7021.2014.01.016
3967	要病原菌的分离鉴定及耐	sistance analysis of major	燕燕,刘伟,林世光,王斌仙	n,Liu Wei,Lin Shiguang,War	上海畜牧兽医通讯	nal Husbandry and Veterina	-	6	48-49,51	2014	sn.1000-7725.2014.06.021
3968	宴席引起的混合细菌性食	rial food poisoning caused	揆,凯金祥,陈宇,王刚强,李	Jinxiang,Chen Yu,Wang Gar	疾病预防控制通报	se Prevention and Control	29	5	63-64	2014	15/j.cnki.jbyfkztb.1405006
3969	海区感染性腹泻病原谱及	racteristics of infectious di	东晓,谢益君,黄瑛,邓晓宇,	Yijun,Huang Ying,Deng Xiao	上海预防医学	hanghai Preventive Medicin	26	4	181-183	2014	-
3970	824例儿童腹泻病原分析	pathogenic factors of diarr	杨桂荣.	Yang Guirong.	古民族大学学报（自然科学	University for Nationalities	-	4	461-462	2014	sn.1671-0185.2014.04.027
3971	屠宰场及猪体内沙门氏菌	ella from slaughterhouses a	黄炳炽,徐振娜,黄育浩,袁	ngchi,Xu Zhenna,Huang Yu	中国动物检疫	China Animal Quarantine	-	9	45-47	2014	n.1005-944X.2014.09.016
3972	2010-2012年腹泻病原菌监	12Annual diarrhea pathoge	鹏,姜玉芳,刘宗东,胡彬,寇	g Yufang,Liu Zongdong,Hu	海峡预防医学杂志	s Journal of Preventive Me	20	3	36-37	2014	-
3973	食物中毒合并群体性心因	used by drinking water cor	罗华.	Luo Hua.	首都医药	Capital Medicine	-	24	64-64,65	2014	-
3974	检出罕见的姆瓦林血清型	ella Mwarin serotype dete	邱华红,成玲,陈惠瑜.	Huahong,Cheng Ling,Chen H	现代预防医学	modern preventive medicin	41	9	1651-1652,1655	2014	-
3975	泻病例沙门氏菌的分离与	cation of Salmonella from c	农,高新菊,姚大伟,丁阳,杨	Gao Xinju,Yao Dawei,Ding Y	畜牧与兽医	Husbandry and Veterinary M	46	6	109-113	2014	-
3976	寒沙门氏菌的分离鉴定及	ceptibility test of Salmonell	蒋勇军.	Jiang Yongjun.	中国畜牧兽医文摘	mal Husbandry and Veterin	-	11	46-47	2014	-
3977	2013年鸡肉沙门菌污染状	of Salmonella contaminatio	秦丽云,徐保红,张慧贤,王苋	u Baohong,Zhang Huixian,	中国卫生检验杂志	ese Journal of Health Inspe	24	19	2867-2868,2871	2014	-
3978	肠炎344例病原菌分布及药	tion of pathogenic bacteria	杨晓华,谭南,林爱心.	ng Xiaohua,Tan Nan,Lin Aix	中国感染与化疗杂志	urnal of Infection and Cher	-	2	149-152	2014	-
3979	奶及奶制品微生物污染状况	contamination of commer	,何永娣,朱伟双,龙文婷,钱	di,Zhu Weishuang,Long W	中国卫生检验杂志	ese Journal of Health Inspe	24	12	1783-1784,1787	2014	-
3980	年全州县食源性致病菌的监	ts of foodborne pathogeni	蒋叶伟,蒋艳凤,王燕红.	ewei,Jiang Yanfeng,Wang Y	应用预防医学	applied preventive medicin	-	4	236-238	2014	n.1673-758X.2014.04.018
3981	性结肠炎合并伦敦沙门菌感	plicated by Salmonella Lond	李丽,张蓓,朱旭慧,闫少珍,詹	Bei,Zhu Xuhui,Yan Shaozh	中国感染与化疗杂志	urnal of Infection and Cher	-	4	352-352	2014	sn.1009-7708.2014.04.025
3982	市感染性腹泻细菌和病毒病	e results of bacterial and vi	江佳,胡家瑜,吴寰宇,薛莹,潘	Hu Jiayu,Wu Huanyu,Xue Y	疾病监测	disease surveillance	29	11	880-883	2014	sn.1003-9961.2014.11.010
3983	地区食源性沙门氏菌耐药性	sistance of foodborne Salm	张文宇,杜雄伟,岳威威.	Wenyu,Du Xiongwei,Yue W	畜牧兽医科技信息	d Veterinary Science and Te	-	2	35-35,36	2014	N.1671-6027.2014.02.017
3984	省血流感染沙门菌的血清型	ance of Salmonella in bloo	徐成芬,吴晓,杨华强.	hengfen,Wu Xiao,Yang Huad	中国现代医生	modern chinese doctor	52	9	77-79,82	2014	-
3985	病原学检测结果的分析及	and discussion of clinical si	范晓琴,沈冬梅.	Fan Xiaoqin,Shen Dongmei	当代医药论丛	ontemporary Medicine Seri	-	16	280-281	2014	-
3986	寒沙门菌引起的食物中毒	k of food poisoning caused	峰,范建华,杨祖顺,韦蝶心,	an Jianhua,Yang Zushun,We	疾病监测	disease surveillance	29	11	920-924	2014	sn.1003-9961.2014.11.019
3987	印第安纳沙门菌多重耐药性	resistance of Salmonella In	,武岩,赵红玉,候晓林,吴国	n,Zhao Hongyu,Hou Xiaolin	中国兽医学报	al of Chinese Veterinary Me	34	9	1496-1500	2014	-
3988	引起的食源性疾病暴发事件	oodborne disease outbreak	锋,许丹,曹国科,侯水平,刘	,Xu Dan,Cao Guoke,Hou le	华南预防医学	uth China Preventive Medic	40	3	270-273	2014	.13217/j.scjpm.2014.0270
3989	食物中毒中脉冲场凝胶电泳	rophoresis technology in Sa	,占利,叶菊莲,罗芸,吴爱萍	,profit,Ye Julian,Luo Yun,W	浙江预防医学	hejiang Preventive Medicin	-	5	535-537	2014	-
3990	类食品中食源性致病菌监测	nalysis of foodborne patho	明,曹春远,陈炳材,林英华,袁	nyuan,Chen Bingcai,Lin Yin	河南预防医学杂志	n Journal of Preventive Me	25	6	414-416	2014	-
3991	波市镇海区感染性腹泻病	ic characteristics of infecti	东晓,谢益君,黄瑛,邓晓宇,	Yijun,Huang Ying,Deng Xiao	疾病监测	disease surveillance	29	4	287-290	2014	sn.1003-9961.2014.04.009
3992	门氏菌的分离鉴定及耐药	resistance detection of Sal	李凤梅,胡杰,屈素洁,莫胜兰	gmei,Hu Jie,Qu Sujie,Mo Sh	农业与技术	Agriculture and Technology	-	8	176-178	2014	n.1671-962X.2014.08.154
3993	婴幼儿腹泻的临床护理干	ervention observation on c	姚红岩.	Yao Hongyan.	中国药物经济学	harmacoeconomics in Chin	-	11	269-269	2014	-
3994	2年厦门市食源性病原菌	e and analysis of foodborne	张建梅,翁琴云,朱淦,汪家旭	ei,Weng Qinyun,Zhu Fu,Wa	现代预防医学	modern preventive medicin	41	13	2352-2355	2014	-

3995	甲型副伤寒疫情实验室分	port on Paratyphoid A Epid	仲春林,徐秀梅.	Zhongchunlin,Xu Xiumei.	医学信息文摘（连续型电	information abstract (cont	-	30	341-341,343	2014	sn.1671-3141.2014.30.276
3996	宜州市食品中食源性致病	odborne pathogenic bacte	韦丽娟,韦显东,杨微.	Lijuan,Wei Xiandong,Yang	职业与健康	Occupation and health	30	13	1841-1843	2014	-
3997	医院沙门菌临床分布及耐	n and drug resistance of Sa	王文娟,虞涛.	Wang Wenjuan,Yu Tao.	中国消毒学杂志	hinese Journal of Disinfecti	31	5	451-453	2014	-
3998	菌引起的食物中毒调查及	analysis of a case of food p	张振春,安秉站.	ang Zhenchun,Anbing Stati	中外健康文摘	hese and Foreign Health Di	-	13	74-74,75	2014	-
3999	检沙门/志贺氏菌检验PC	loyees/Shigella testPCRAp	欣,董雪,刘娜,李继耀,刘宏	g Xue,Liu Na,Li Jiyao,Liu H	宁大学学报（自然科学版	oning University (Natural Sc	41	2	172-175	2014	sn.1000-5846.2014.02.015
4000	鲜牛乳中微生物污染程度	icrobial contamination leve	赛都力,王一明,阿曼土尔,	en·Saduli,Wang Yiming,Om	龙江畜牧兽医（上半月）	ndry and Veterinary Medic	-	7	209-211	2014	-
4001	菌引起的食物中毒检测及	ed by Salmonella Enteritidi	静,竺稽定,夏颖萍,杨元	,Zhu Jiding,Xia Yingping,Ya	中国食品卫生杂志	hinese Food Hygiene Journ	26	5	489-491	2014	.13590/j.cjfh.2014.05.023
4002	中沙门氏菌的半定量风险	assessment study of Salm	吴云凤,袁宝君.	Wu Yunfeng,Yuan Baojun.	食品安全质量检测学报	f Food Safety and Quality I	-	12	4157-4162	2014	-
4003	平湖市沙门菌临床感染血	fection serotypes and drug	沈美萍,吴晓燕,王良平.	eiping,Wu Xiaoyan,Wang Li	疾病监测	disease surveillance	29	7	560-563	2014	sn.1003-9961.2014.07.014
4004	致病菌的分离鉴定、耐药	ogenicity test of pathogen	萍,陆风,黄福标,杨奕洁,陈	Feng,Huang Fubiao,Yang Yi	中国奶牛	Chinese cow	-	5	24-27	2014	sn.1004-4264.2014.05.007
4005	起肠炎沙门菌食物中毒报	Salmonella Enteritidis food	安平,潘刚雷,陈米娜,杨元	ing,Pan Ganglei,Mina Chen	浙江预防医学	hejiang Preventive Medicin	26	4	402-404	2014	-
4006	～2012年食品中致病菌监	of annual monitoring resul	劳希,盘珍梅,卢家友.	ausch,Pan Zhenmei,Lu Jiay	中国热带医学	Chinese tropical medicine	14	5	624-626	2014	-
4007	年达州市全球沙门菌监测	obal Salmonella Surveillan	李红,陈松,周汉洪.	ong,Chen Song,Zhou Hanh	预防医学情报杂志	of Preventive Medicine Inf	30	8	663-664	2014	-
4008	江省义乌市食品中食源性	foodborne pathogenic bac	方晔,陈黎,陈锦德,陈步青	Chen Li,Chen Jinde,Chen Bu	中国卫生检验杂志	ese Journal of Health Inspe	24	8	1159-1161	2014	-
4009	岗区致感染性腹泻沙门菌	ing of Salmonella causing i	江涛,王德全,陈应坚,金玉	Wang Dequan,Chen Yingjia	中国病原生物学杂志	ese Journal of Pathogen Bi	9	6	519-522	2014	10.13350/j.cjpb.140611
4010	1年猪肉生产链中沙门菌	nce characteristics and mo	刘书亮,侯小刚,陈荀,彭珍	,Hou Xiaogang,Chen Xun,P	中华流行病学杂志	inese Journal of Epidemiol	-	5	519-523	2014	sn.0254-6450.2014.05.011
4011	至2013年食品监测网结	Analysis of annual food m	郭勇峰,王敬辉,吉彦莉.	Yongfeng,Wang Jinghui,Ji Y	中国保健营养（中旬刊）	health nutrition (mid-term	24	3	1168-1169	2014	-
4012	密云县食源性疾病主动	nce results of foodborne di	杰,郑德生,宋晓立,郑若	eng Desheng,Song Xiaoli,Zh	首都公共卫生	capital public health	8	4	163-165	2014	-
4013	2012年食源性致病菌监测	s of annual foodborne pat	方道,盛维立,汪道发,唐静	iu,Sheng Weili,Wang Daofa	浙江预防医学	hejiang Preventive Medicin	-	8	832-834	2014	-
4014	沙门菌的分离鉴定及耐药	d drug resistance analysis	旭,戴增斌,刘梅,李建梅,姜	,Dai Zengbin,Liu Mei,Li Jian	中国家禽	Chinese poultry	36	14	48-51	2014	sn.1004-6364.2014.14.014
4015	德尔卑沙门菌临床株耐药	es of clinical strains of mul	,陈冬雅,黎毓光,林晓晖,	n Dongya,Li Yuguang,Lin X	中国热带医学	Chinese tropical medicine	14	6	652-656	2014	-
4016	超市中冰鲜鸡肉微生物污	mination of chilled chicken	红,李鑫,阳宇恒,李昂狄,郑	Xin,Yang Yuheng,Li Angdi,	中国食物与营养	Chinese food and nutrition	-	12	10-12,13	2014	sn.1006-9577.2014.12.002
4017	性沙门氏菌的分离及耐药	sistance research of anima	元冉,董鹏,李金磊,班付国	ran,Dong Peng,Li Jinlei,ban	畜牧与饲料科学	Livestock and feed science	-	7	11-13	2014	sn.1672-5190.2014.07.006
4018	沙门氏菌耐药表型与耐药	drug-resistant genes of pat	戴建华,吴植,袁维峰.	Jianhua,Wu Zhi,Yuan Weif	中国畜牧兽医	mal husbandry and veterin	41	4	245-248	2014	-
4019	禽畜肉及相关食品中致病	ria in raw poultry, livestoc	兰,陈伟鑫,陈培超,袁红,孙	n Weixin,Chen Peichao,Yua	上海预防医学	hanghai Preventive Medici	-	12	681-682	2014	-
4020	门菌江苏分离株的生物学	stics of Jiangsu isolates of	,韩先干,朱春红,周守长,	gan,Zhu Chunhong,Zhou S	中国预防兽医学报	rnal of Preventive Veterina	36	10	775-779	2014	ssn.1008-0589.2014.10.08
4021	制速冻面米制品食源性	nic bacteria in raw quick-f	慧霞,张秀丽,崔莹,李艳	xia,Zhang Xiuli,Cui Ying,Li	中国食品卫生杂志	hinese Food Hygiene Journ	26	5	500-503	2014	.13590/j.cjfh.2014.05.022
4022	即食生食动物性水产品	w animal aquatic products	卫兵,羌校君,赵荣梅,金峰	bing,Qiang Xiaojun,Zhao R	中国食物与营养	Chinese food and nutrition	20	8	17-19	2014	sn.1006-9577.2014.08.004
4023	12年26家医疗机构消毒灭	Analysis of Disinfection ar	江,何亚明,刘南,季恒青,	,He Yaming,Liu Nan,Ji Heng	重庆医学	Chongqing Medicine	-	28	3769-3771	2014	sn.1671-8348.2014.28.024
4024	腹泻病原菌分布特点及药	analysis of pathogenic ba	薛爱国,李海燕,宋冬,丁伟	uo,Li Haiyan,Song Dong,Di	中国现代药物应用	dern drug application in Ch	-	7	7-8,9	2014	-
4025	年我国其他感染性腹泻事	lysis of other infectious dia	高永军,丁凡,王霄晔,李群	gjun,Ding Fan,Wang Xiaoy	实用预防医学	ractical preventive medicin	21	6	695-697	2014	sn.1006-3110.2014.06.019
4026	服务单位自制凉菜细菌污	on of homemade cold dish	周新峰.	Zhou Xinfeng.	河南预防医学杂志	Journal of Preventive Me	-	3	240-242	2014	-
4027	氏菌感染者大便中酵母样	ngi in the stool of patients	云峰,江晓玲,杜利容,刘中	g,Jiang Xiaoling,Du Lijong,	西南军医	outhwest Military Medicin	-	4	409-410	2014	sn.1672-7193.2014.04.023
4028	市农村食品安全风险监测	od safety risk monitoring	李建勋.	Li Jianxun.	中国煤炭工业医学杂志	Journal of Coal Industry M	17	5	806-808	2014	yyx1007-9564 201405041
4029	邵阳市食源性致病菌污染	Foodborne Pathogen Con	善良,李广兵,黄良军,雷卫	Li Guangbing,Huang Liangj	河南预防医学杂志	Journal of Preventive Me	-	3	195-198,206	2014	-
4030	金州新区食源性致病菌检	ults of foodborne pathoge	周春景,薛慧丽,侯小娜.	Chunjing,Xue Huili,Hou Xi	疾病监测与控制	ease surveillance and cont	8	5	311310	2014	-
4031	分血液细菌培养与耐药性	terial culture and drug resis	张旻,卢少华.	Zhang Min,Lu Shaohua.	中国卫生产业	China health industry	-	3	43-44	2014	-

4032	京市海淀区细菌性食物中	food poisoning detection r	王菊光,谢利军,孙晓华,纪	ng Juguang,Xie Lijun,Sun Xi	预防医学情报杂志	of Preventive Medicine Info	30	10	857-860	2014	-
4033	寒沙门氏菌的分离鉴定及	ity observation of Salmone	丽,杨军,项玉,谷长勤,张万	ng Jun,Xiang Yu,Gu Changc	湖北农业科学	Hubei Agricultural Sciences	53	2	378-381	2014	sn.0439-8114.2014.02.035
4034	炎沙门菌引起的食物中毒	is of food poisoning caused	华,王东,刘思渊,黄淑华,温	ng Dong,Liu Siyuan,Huang	华南预防医学	uth China Preventive Medic	40	3	274-275	2014	).13217/j.scjpm.2014.0274
4035	餐饮食品中食源性致病微	s of foodborne pathogenic	周银柱,朱彩明,陈艳.	u Yinzhu,Zhu Caiming,Chen	实用预防医学	ractical preventive medicin	21	12	1476-1478	2014	n.1006-3110.2014.012.022
4036	已鼠伤寒沙门菌食物中毒	ase of Salmonella Typhimu	陈和娟,王伟望,张峰,李宁丰	Wang Weiwang,Zhang Fen	上海预防医学	hanghai Preventive Medicir	26	10	537-538	2014	-
4037	年昆明市食品安全监测结	food safety monitoring res	王健芳,黄文智,林嘉,赵俊丽	fang,Huang Wenzhi,Lin Jia,	中国卫生标准管理	a Health Standard Manager	-	23	2-6	2014	N.1674-9316.2014.23.002
4038	2013年伤寒沙门氏菌耐药	drug resistance analysis o	甫尔,李方,外力·沙塔尔,木	Fang,external force·Shatar	疾病预防控制通报	se Prevention and Control	29	5	49-51	2014	15/j.cnki.jbyfkztb.1402028
4039	菌耐药谱及质粒耐药基因	ug resistance spectrum an	冯婧嘉,施春雷,李可,史贤明	jia,Shi Chunlei,Li Ke,Shi Xia	中国食品学报	urnal of Chinese Food Scier	14	4	184-190	2014	-
4040	双流国际机场航空食品微	alysis of aviation food at C	玮,刘杨,黄筱宇,魏灿,邓晓	ang,Huang Xiaoyu,Wei Can	中国国境卫生检疫杂志	ontier Health and Quarantir	37	5	339-342,352	2014	-
4041	省肉鸡产业链中沙门菌污	tamination in the broiler in	崔莹,李艳芬,张秀丽,张丁	g,Li Yanfen,Zhang Xiuli,Zhar	中国卫生检验杂志	ese Journal of Health Inspe	24	20	3000-3003,3009	2014	-
4042	版纳州鼠型斑疹伤寒暴发	typhus in Xishuangbann	冯云;杨杜鹃;宋淼;白鹤鸣	ang Dujuan;Song Miao;	中国人兽共患病学报	hinese Journal of Zoonos	30	12	1272-1280	2014	-
4043	路水源引起的伤寒暴发疫	phoid fever outbreak cau	群勇;张娟;胡琴莉;周进升	;Zhang Juan;Hu Qinli;Zh	现代预防医学	odern preventive medicir	41	11	1972-1974	2014	-
4044	腹泻病原学检测及流行病	nd epidemiological analysis	张利,孙林春.	Zhang Li,Sun Linchun.	中国综合临床	hina Comprehensive Clinic	31	12	1143-1145	2015	sn.1008-6315.2015.12.027
4045	菌致幼儿腹泻的病例检测	s of diarrhea in young child	文鹏,尹建雯,张京云,周晓	eng,Yin Jianwen,Zhang Jing	中华预防医学杂志	se Journal of Preventive Me	49	11	1020-1022	2015	sn.0253-9624.2015.11.019
4046	月至2014年7月腹泻病例	7Study on the etiological s	家瑜,吴寰宇,潘浩,张曦,薛	u Huanyu,Pan Hao,Zhang X	中华流行病学杂志	inese Journal of Epidemiolo	36	10	1099-1103	2015	sn.0254-6450.2015.10.014
4047	3年沙门菌流行特征及分子	emic characteristics and m	煜,郑伟,陈建才,张蔚,黄利	Wei,Chen Jiancai,Zhang We	中华流行病学杂志	inese Journal of Epidemiolo	36	8	907-909	2015	sn.0254-6450.2015.08.030
4048	致食源性疾病暴发疫情的	sis of a foodborne disease	丽,马晓晨,滕仁明,赵耀,陈	aochen,Teng Renming,Zhao	中华预防医学杂志	se Journal of Preventive Me	49	1	60-62	2015	sn.0253-9624.2015.01.013
4049	市城区食源性疾病哨点医	of foodborne disease sentir	谭维维,安娜.	Tan Weiwei,Anna.	职业与健康	Occupation and health	31	20	2776-2779	2015	-
4050	门氏菌的分离鉴定及药敏	n and drug susceptibility te	魏静萍.	Wei Jingping.	养殖与饲料	Breeding and feed	-	2	16-19	2015	n.1671-427X.2015.02.005
4051	年医院感染病原菌监测与	g resistance analysis of pat	民,陈文萍,黄荣平,彭磊,王	ping,Huang Rongping,Peng	中华医院感染学杂志	ournal of Hospital Infectiou	-	20	4592-4593,4596	2015	.11816/cn.ni.2015-151341
4052	腹泻细菌性病原的分离鉴	analysis of bacterial patho	龙龙,毕峻龙,冯朝相,杨贵	g,Bi Junlong,Feng Chaoxian	中国畜牧兽医	mal husbandry and veterina	42	3	696-700	2015	ki.1671-7236.2015.03.029
4053	拌食品中微生物污染情况	of microbial contamination	雷少华.	Lei Shaohua.	中国卫生产业	China health industry	-	24	20-22	2015	ki.1672-5654.2015.24.020
4054	用槟榔微生物污染状况调	bial Contamination of Com	邓峥华,邓春明,李帮锐.	enghua,Deng Chunming,Li	海峡预防医学杂志	s Journal of Preventive Me	21	3	66-67	2015	-
4055	寒引起的肠炎沙门菌食物中	Enteritidis food poisoning	吴兴美,廖金宝,廖慧.	u Xingmei,Liao Jinbao,Liao H	海峡预防医学杂志	s Journal of Preventive Me	21	1	46-47	2015	-
4056	枝花市食品中食源性致病	ults of foodborne pathoge	陈嫣,沈来红,潘泓宇,陈鑫宝	hen Laihong,Pan Hongyu,C	现代预防医学	modern preventive medicin	42	21	3871-3873,3938	2015	-
4057	11-2014年市售婴幼儿食品	vey on hygiene status of c	长慧,秦三平,齐晓琴,王红雷	Qin Sanping,Qi Xiaoqin,Wa	中国保健营养	Chinese health nutrition	25	11	301	2015	-
4058	市西湖区食源性疾病哨点	ults of foodborne disease s	闽燕,沈利明,陈晖,吴亚平	n,Shen Liming,Chen Hui,W	中国卫生检验杂志	ese Journal of Health Inspe	25	22	3924-3926	2015	-
4059	~2014年食源性致病菌监	alysis of annual foodborne	吴占国,孙素梅,崔明.	Zhanguo,Sun Sumei,Cui M	标记免疫分析与临床	eled immunoassays and cli	22	9	924-926	2015	sn.1006-1703.2015.09.029
4060	吉街道从业人员沙门菌PFG	oyees in Buji Street, Shenz	海娟,金玉娟,吴永雄,张文	n Yujuan,Wu Yongxiong,Zh	中国热带医学	Chinese tropical medicine	15	3	281-284	2015	-
4061	鸡源沙门菌血清型鉴定及耐	nce analysis of Salmonella	婷,柴慧敏,李小龙,白洋,丁	Huimin,bruce lee,Bai Yang,	动物医学进展	vances in Veterinary Medic	-	11	121-124	2015	sn.1007-5038.2015.11.028
4062	学生餐中食源性致病菌污	amination status of foodb	王燕梅,唐震,乔昕,倪云龙	mei,Tang Zhen,Qiao Xin,N	现代预防医学	modern preventive medicin	42	23	4268-4270	2015	-
4063	菌引起的食源性疾病爆发的	foodborne disease outbre	郑悦康,叶志英,刘绮明,吴	Yuekang,Ye Zhiying,Liu Qi	现代生物医学进展	ogress in Modern Biomedic	15	31	6043-6045,6111	2015	41/j.cnki.pmb.2015.31.012
4064	济市零售牛羊肉中沙门氏菌	lmonella in retail beef and	天,邹弯,张亚南,葛坤,王智	n,Zou Wan,Zhang Yanan,G	食品安全质量检测学报	f Food Safety and Quality I	-	9	3480-3484	2015	-
4065	模化猪场环境中病原菌的	genic bacteria in large-scale	郑华,张邑帆,杨柳,沈克飞	Hua,Zhang Yifan,willow,She	上海畜牧兽医通讯	nal Husbandry and Veterina	-	5	2-4	2015	sn.1000-7725.2015.05.001
4066	贺州市市售食品食源性致	ne pathogenic bacteria in	芬,饶贵平,梁琴,黎振坚,郑	uiping,Liang Qin,Li Zhenjian	中国卫生检验杂志	ese Journal of Health Inspe	25	7	1060-1063	2015	-
4067	屠宰环节沙门菌毒力基因	atus of Salmonella in pig sl	赵建梅,曲志娜,王玉东,孙	Jianmei,Qu Zhina,Wang Y	中国兽医杂志	Chinese Veterinary Journal	51	12	83-85,88	2015	sn.0529-6005.2015.12.030
4068	深圳市龙岗区沙门菌分子	ogical analysis of Salmonell	,刘丽红,金玉娟,邹惠英,王	ihong,Jin Yujuan,Zou Huiyi	热带医学杂志	ournal of tropical medicine	15	9	1262-1265	2015	-

4069	大连市食源性致病菌监测结	g results of foodborne pat	吕秋月,栾明春,肖冰.	iuyue,Luan Mingchun,Xiao	医学动物防制	medical animal control	31	5	506-508	2015	0.7629/yxdwfz201505012
4070	市市售食品中食源性致病	thogenic bacteria contamin	熊莉萍,李通情,郭欣,刘腾,刘	g,Li Tongqing,Guo Xin,Liu T	现代预防医学	modern preventive medicin	42	10	1768-1771	2015	-
4071	行业从业人员肠道致病菌	bacteria among employees	王丹.	Wang Dan.	中国保健营养	Chinese health nutrition	25	14	241	2015	-
4072	养殖和屠宰销售环节沙门	broiler chicken hatching, br	益涛,梁永柱,刘德辉,王晓	o,Liang Yongzhu,Liu Dehui,	预防医学论坛	Preventive Medicine Forum	21	9	654-656,659	2015	-
4073	西城区沙门菌的监测、溯	ability of Salmonella in Xich	霍哲,高波,胡三梅,王宵雪	Gao Bo,Hu Sanmei,Wang Xi	中国卫生检验杂志	ese Journal of Health Inspe	25	10	1606-1608	2015	-
4074	源熟肉制品中食源性致病	enic bacteria in cooked me	黄贤.	Huang Xian.	疾病监测与控制	sease surveillance and cont	9	6	410-411	2015	-
4075	沙门氏菌的分离鉴定与药敏	n and drug susceptibility te	王海龙,秦国栋,张敏敏.	ailong,Qin Guodong,Zhang	中国畜禽种业	estock and poultry breeding	-	7	28-29	2015	sn.1673-4556.2015.07.020
4076	写门诊病例粪便的细菌菌	eptibility analysis of feces o	杨焕章,严育忠,范慧清,徐英	hang,Yan Yuzhong,Fan Huic	国际检验医学杂志	onal journal of laboratory r	-	12	1682-1683,1685	2015	sn.1673-4130.2015.12.019
4077	培养阳性病原菌分布及耐	ance analysis of blood cultu	,胡大春,卢赞,任宝军,王霞	chun,Luzan,Ren Baojun,W	中国执业药师	Chinese licensed pharmacis	-	4	11-15	2015	sn.1672-5433.2015.04.003
4078	中分离菌对β-内酰胺类的	eat sold in Zhengzhouβ-Re	钟杏好,刘保光,徐航,胡功	ghao,Liu Baoguang,Xu Hang	江西农业学报	ournal of Jiangxi Agricultur	27	10	108-111	2015	sn.1001-8581.2015.10.025
4079	屠宰场屠宰环节沙门氏菌	the slaughtering process o	,赵建梅,盖文燕,孙佳怡,	ao Jianmei,Gai Wenyan,Su	中国动物检疫	China Animal Quarantine	32	5	82-86	2015	n.1005-944X.2015.05.026
4080	13年杭州市腹泻病原谱分	diarrhea pathogen spectru	敏,孙长贵,陈晓,余斐,陈	un Changgui,Chen Xiao,Yu	临床检验杂志	journal of clinical testing	33	6	469-471	2015	602/j.cnki.jcls.2015.06.21
4081	猪沙门氏菌的分离鉴定及	ne determination of Salmo	杨贵燕,马树薇,王九峰.	Guiyan,Ma Shuwei,Wang Ji	畜牧与饲料科学	Livestock and feed science	36	6	223-228	2015	sn.1672-5190.2015.06.036
4082	不同污水处理系统中病原	pathogenic bacteria in diff	易鑫,李娟,黄京,刘新春.	,Li Juan,Huang Jing,Liu Xin	环境科学学报	rnal of Environmental Scie	35	6	1759-1767	2015	13671/j.hjkxxb.2014.1039
4083	丹江市食品中食源性致病	atus of foodborne pathoge	杨硕,吕昌武,王彦玲,冯春	Lu Changwu,Wang Yanling,	医学动物防制	medical animal control	31	7	773-775,778	2015	0.7629/yxdwfz201507022
4084	鸡养殖场主要疫病流行	on of main diseases in large	超,李明彦,陈培荣,赵瑜,	Li Mingyan,Chen Peirong,Z	中国家禽	Chinese poultry	37	5	66-68	2015	sn.1004-6364.2015.05.017
4085	江省食源性疾病监测现状	s of foodborne disease surv	吕鹏,章荣华,齐小娟,陈江	ang Ronghua,Qi Xiaojuan,	浙江预防医学	hejiang Preventive Medicin	-	8	828-829,837	2015	-
4086	市市售新鲜蔬菜微生物污	on of fresh vegetables com	马晨,陈雪华,钱程.	Chen,Chen Xuehua,Qian Ch	浙江农业科学	hejiang Agricultural Science	56	3	396-401	2015	.issn.0528-9017.20150336
4087	2013年沙门菌对环丙沙星	n the resistance characteris	碧霞,何冬梅,谭海玲,李柏	e Dongmei,Tan Hailing,Li B	华南预防医学	uth China Preventive Medic	41	1	1-5	2015	.13217/j.scjpm.2015.0001
4088	细菌性食物中毒的发生规	s and epidemic characteris	李玉芹.	Li Yuqin.	当代医药论丛	ontemporary Medicine Seri	-	16	235-236	2015	-
4089	江口岸水体中食源性致病	hogenic bacteria in water b	,吴瑕,许佳,袁良慧,赵谦,	Xu Jia,Yuan Lianghui,Zhao	中国国境卫生检疫杂志	ntier Health and Quarantin	38	4	269-272	2015	/j.1004-9770.2015.04.011
4090	半年家禽重点养殖区域疫	iseases in key poultry bree	沈元,崔玉娟,尤永君,梁武	n,Cui Yujuan,You Yongjun,	中国禽业导刊	China Poultry Industry Guid	32	16	42,44	2015	-
4091	2-2013年桶装饮用水微生	biological testing and analy	刘璀.	Liu Cui.	公共卫生与预防医学	Health and Preventive Me	26	4	103-104	2015	-
4092	水中沙门菌检测扩项评审	review of Salmonella detec	余芳,王旭琴,陈慧燕.	Fang,Wang Xuqin,Chen Hui	中国卫生工程学	Chinese sanitary engineerin	14	5	450-451	2015	-
4093	3年沈阳市食源性致病菌	ults of foodborne pathogen	赵常智.	Zhao Changzhi.	职业与健康	Occupation and health	31	7	979-980	2015	-
4094	2013-2014年感染性腹泻病	2014Annual infectious diar	陈辉,李博,鞠长燕,王婷.	Hui,Li Bo,Ju Changyan,Wan	中国热带医学	Chinese tropical medicine	15	7	839-841	2015	cnki.46-1064/r.2015.07.18
4095	主要食源性致病菌污染检	ction and distribution prob	,杜兰兰,雷娅,张宏宇,刘芳	nlan,Rhea,Zhang Hongyu,L	食品科学	food science	36	10	201-205	2015	pkx1002-6630-201510040
4096	食物中毒事件进行致病菌检	sis of pathogenic bacteria	孟克.	Monk.	医学信息文摘（连续型电	information abstract (cont	15	A2	222	2015	.1671-3141.2015.102.168
4097	市售奶粉细菌检测及分析	d analysis of commercially	,王东建,卢春霞,刘长彬,	ngjian,Lu Chunxia,Liu Char	中国乳品工业	China dairy industry	43	1	44-46	2015	sn.1001-2230.2015.01.012
4098	园肠炎沙门菌食物中毒事	of Salmonella Enteritidis Fo	溢联,赵帅,马洁,彭立昌,董	hao Shuai,Ma Jie,Peng Lich	疾病监测	disease surveillance	30	7	602-603	2015	sn.1003-9961.2015.07.020
4099	沙门菌的污染情况调查和耐	e analysis of Salmonella co	兴伟,周向阳,周秀锦,万婧	i,Zhou Xiangyang,Zhou Xiu	中国抗生素杂志	hinese Journal of Antibiotic	40	2	128-131	2015	sn.1001-8689.2015.02.012
4100	装糕点微生物污染状况调	crobial contamination statu	要三会.	Need three meetings.	医学信息文摘（连续型电	information abstract (cont	-	49	145-146	2015	sn.1671-3141.2015.49.126
4101	食物中毒事件中检出伤寒	ted in a wedding banquet f	胡冬菊.	Hu Dongju.	医学信息文摘（连续型电	information abstract (cont	15	35	251-252	2015	sn.1671-3141.2015.35.229
4102	儿童腹泻患儿致病菌流行特	tance of pathogenic bacter	于宝东,申红霞,刘畅,赵伟	ng,Shen Hongxia,Liu Chang,	儿科药学杂志	ournal of Pediatric Pharmac	21	11	41-43	2015	.1672-108X.2015.011.014
4103	性败血症52例临床特点和病	ical characteristics and pat	许世申,陈国伟.	Xu Shishen,Chen Guowei.	北方药学	Northern Pharmacy	-	5	165-166	2015	-
4104	物中毒致病因素的文献分	s on the causative factors o	郑建成,王育兵,杨凯.	Jiancheng,Wang Yubing,Ya	职业与健康	Occupation and health	31	23	3236-3239	2015	-
4105	南宁市哨点医院食源性疾	disease surveillance results	张静,刘军.	Zhang Jing,Liu Jun.	中国卫生检验杂志	ese Journal of Health Inspe	25	4	559-561	2015	-

4106	食源性非伤寒沙门菌感染	n-typhoid Salmonella infect	紫杭,翁创伟,余少珍,卢嘉	eng Chuangwei,Yu Shaozh	中国热带医学	Chinese tropical medicine	15	5	571-573	2015	-
4107	～2014年感染性腹泻沙门	al surveillance results of sa	卯青,欧新华,张如胜,李叶兰	g,Ou Xinhua,Zhang Rushen	中国热带医学	Chinese tropical medicine	15	4	500-502	2015	-
4108	肠炎沙门菌食物中毒流行	lmonella Enteritidis food p	建民,曹国平,蓝岳云,杨瑞	ao Guoping,Lan Yueyun,Ya	中国学校卫生	Chinese school health	36	10	1565-1567	2015	-
4109	肠炎32例临床特点与耐药	on clinical characteristics a	张笑稚.	Zhang Xiaozhi.	中外女性健康研究	nd foreign women's health	-	23	226229	2015	-
4110	病原谱及其与CD4+T淋巴	s and its relationship with	何盛华,周锐峰,蔡琳,张仁芳	hua,Zhou Ruifeng,Cai Lin,Z	中国艾滋病性病	AIDS and STDs in China	21	1	8-10	2015	-
4111	市4起学校食物中毒调查	tion and analysis of food p	刘萍,张峰,李凡,吴小燕.	g,Zhang Feng,Li Fan,Wu Xi	公共卫生与预防医学	Health and Preventive Me	26	6	81-83	2015	-
4112	腹泻中沙门菌血清型及耐药	erotypes and drug resistan	高昆,韩倩,黄惠婷.	Kun,Han Qian,Huang Huit	临床输血与检验	inical transfusion and testi	17	1	15-17	2015	sn.1671-2587.2015.01.005
4113	门菌L型的分离培养与in	adderLtype of isolation cul	文静,吴文娟,潘耀振,汤可	ng,Wu Wenjuan,Pan Yaozh	动物医学进展	vances in Veterinary Medic	-	3	25-27,28	2015	sn.1007-5038.2015.03.007
4114	细菌性食源性疾病病原菌	etection results of bacteria	高亚色,杨帆,王坤明.	a color,Yang Fan,Wang Kur	现代预防医学	modern preventive medicin	42	22	4074-4076	2015	-
4115	原微生物临床检验120例这	arrhea in infants and young	郑国荣.	Zheng Guorong.	医学信息文摘（连续型电	information abstract (cont	-	13	112-112,116	2015	sn.1671-3141.2015.13.092
4116	养殖场饲料中潜在风险	roorganisms in the feed of	,祁巨中,刘英玉,李琳,刘莉	hong,Liu Yingyu,Li Lin,Liu Li	新疆农业科学	Xinjiang Agricultural Science	52	9	1749-1756	2015	sn.1001-4330.2015.09.025
4117	南地区Whire Union血流感	ire UnionBloodstream infe	铁,陈知行,刘宝,费樱,杜艳	en Zhixing,Liu Bao,Fei Ying	四川大学学报（医学版）	Sichuan University (Medic	46	1	75-81	2015	-
4118	下婴幼儿腹泻感染现状临	tus of diarrheal infections	宪华,崔桂平,王丹丹,陈王	ui Guiping,Wang Dandan,C	河南预防医学杂志	Journal of Preventive Me	26	5	446-449	2015	m.1006-8414.2015.05.042
4119	起的食物中毒的病原检	y analysis of food poisonin	勇,龙佳丽,侯水平,张欣强	g,Long Jiali,Hou level,Zhang	检验医学与临床	boratory medicine and clini	-	14	2135-2136	2015	sn.1672-9455.2015.14.071
4120	菌的血清型、药敏结果及	ility results and multidrug	汪云霞,薛春玲,黄东平.	nxia,Xue Chunling,Huang D	齐齐哈尔医学院学报	rnal of Qiqihar Medical Coll	36	31	4738-4739	2015	-
4121	冷冻食品微生物监测分析	monitoring and analysis rep	周迪.	Zhou Di.	医学信息文摘（连续型电	information abstract (cont	-	5	127-127	2015	n.1671_3141.2015.05.094
4122	海珠区细菌性食物中毒病	f pathogenic bacteria of ba	琪,曾雅,许少洪,李映霞,王	Zeng Ya,Xu Shaohong,Li Yir	中国公共卫生管理	ese public health managen	31	2	157-159	2015	-
4123	市食源性致病菌污染状	odborne Pathogenic Bacteri	苏春丽,沈亮,杨柳.	u Chunli,Shen Liang,willow	现代预防医学	modern preventive medicin	42	5	817-818,840	2015	-
4124	013年大连市食源性疾病	ance analysis of foodborne	晓昀,安玉,蒋希宏,张磊,李	un,Anyu,Jiang Xihong,Zhan	预防医学论坛	Preventive Medicine Forum	21	10	731-734	2015	-
4125	2013年食品中食源性致病	annual monitoring results	周庆荣,郑建俊,郑升龙.	rong,Zheng Jianjun,Zheng	中国卫生检验杂志	ese Journal of Health Inspe	25	3	401-404	2015	-
4126	7株伤寒和副伤寒沙门菌	stance and molecular typin	坤,吕冰,梁志超,黄瑛,李钦	n,Lu Bing,Liang Zhichao,Hu	职业与健康	Occupation and health	31	12	1614-1617	2015	-
4127	业从业人员肠道沙门氏菌	test results for employees	李玉莹.	Li Yuying.	中国卫生产业	China health industry	-	12	25-26	2015	-
4128	腹泻患者病原菌分布与耐药	stance of pathogenic bacte	陈素菜,张爱鸣,张颖,林建芬	i,Zhang Aiming,Zhang Ying	中华医院感染学杂志	ournal of Hospital Infectiou	-	23	5338-5340	2015	.11816/cn.ni.2015-144147
4129	区儿童细菌性腹泻病原菌	ce of pathogenic bacteria in	肖元华.	Xiao Yuanhua.	医学信息	medical information	-	48	114-114	2015	sn.1006-1959.2015.48.143
4130	013年熟肉制品风险监测结	k Monitoring Results of Ar	刘晓辉,王欣,徐清,黄河清	ui,Wang Xin,Xu Qing,Huan	中国卫生检验杂志	ese Journal of Health Inspe	25	13	2207-2209,2212	2015	-
4131	市其他感染性腹泻流行病	haracteristics of other infe	陈恩晃.	Chen Enhuang.	中国医学工程	Chinese Medical Engineerin	23	7	104107	2015	-
4132	4年感染性腹泻病原学监	f annual infectious diarrhe	张月芬,王玉财,李玉忠.	Yuefen,Wang Yucai,Li Yuz	大家健康（中旬版）	one is healthy (mid-term ed	-	7	15-15,16	2015	-
4133	非伤寒沙门菌的分子分型	g resistance of foodborne	张晓嫒,王迪,陈倩.	ng Xiaoai,Wang Di,Chen Q	中国食品卫生杂志	hinese Food Hygiene Journ	27	3	232-237	2015	).13590/j.cjfh.2015.03.003
4134	事件的病原学检测及结果	result analysis of food pois	曾世培,古廷思.	Zeng Shipei,Gu Tingsi.	医学信息文摘（连续型电	information abstract (cont	-	6	165-166	2015	.1671-3141.2015.06.0142
4135	的耐药性分析及氟苯尼考	ived Salmonella and identi	黄骏,杨林,鞠勇,朱相儒,孙	Jun,Yang Lin,Ju Yong,Zhu X	中国畜牧兽医	mal husbandry and veterina	42	2	459-466	2015	ki.1671-7236.2015.02.035
4136	中分离出沙门氏菌的FAFL	attle during slaughterFAFL	衍伟,梁荣蓉,牛乐宝,李三	Yanwei,Liang Rongrong,Ni	现代食品科技	Modern food technology	31	9	31-37	2015	nfst.1673-9078.2015.9.006
4137	头孢噻肟双耐药印第安纳	cefotaxime dual-resistant	陈秋霞,刘桂华,陈倩,周刚	,Liu Guihua,Chen Qian,Gar	中华预防医学杂志	se Journal of Preventive Me	-	8	716-721	2015	sn.0253-9624.2015.08.010
4138	-2014年食物中毒事件原因	and evaluation of causes c	雪华,李月欢,杜济民,周慧	hua,Li Yuehuan,Du Jimin,Ch	中国热带医学	Chinese tropical medicine	15	12	1441-1444	2015	cnki.46-1064/r.2015.12.07
4139	肠道门诊腹泻患者病原	um in patients with diarrhe	张兰荣,甄博珺,李朝辉,吴	anrong,Zhen Bojun,Li Chao	中华传染病杂志	se Journal of Infectious Dis	-	8	460-464	2015	sn.1000-6680.2015.08.004
4140	者血培养阳性结果的临床	itive blood culture results i	陈滔.	Chen Tao.	医学信息	medical information	-	48	250-250	2015	sn.1006-1959.2015.48.352
4141	引起的幼儿园幼儿中毒事	ng incident of kindergarten	,李晓霞,周洁,游杰,杨贵清	aoxia,Zhou Jie,You Jie,Yang	中国食品卫生杂志	hinese Food Hygiene Journ	27	5	590-593	2015	).13590/j.cjfh.2015.05.023
4142	14年新疆食源性致病菌监	d analysis of foodborne pat	艳,于烽,苏静,远雪梅,杨勤	Feng,Su Jing,Yuan Xuemei,Y	疾病预防控制通报	se Prevention and Control	30	4	78-79	2015	15/j.cnki.jbyfkztb.1505008

4143	南山区腹泻患者沙门菌血	f Salmonella in patients wi	燕,潘伟光,黄锐敏,王婷,阝	Veiguang,Huang Ruimin,W	疾病监测	disease surveillance	30	1	30-34	2015	sn.1003-9961.2015.01.009
4144	门菌引起食源性疾病暴发	odborne disease outbreak	,王鲁茜,唐雅清,舒高林,孙	g Luqian,Tang Yaqing,Shu G	疾病监测	disease surveillance	30	10	883-885	2015	sn.1003-9961.2015.10.021
4145	霍乱沙门菌致羊膜腔感染	infection caused by Salmo	毛红霞,王琳.	Mao Hongxia,Wang Lin.	中华围产医学杂志	inese Journal of Perinatolo	-	4	304-305	2015	sn.1007-9408.2015.04.013
4146	腹泻患者沙门菌病原学特	resistance of Salmonella in	,冯宝立,郑兰紫,蒋会婷,周	Baoli,Zheng Lanzi,Jiang Hu	中国卫生检验杂志	ese Journal of Health Inspe	25	24	4347-4349	2015	-
4147	料用肉粉微生物污染调查	s of Microbial Contaminati	敏,柏凡,郭春华,柏雪,刘	Fan,Guo Chunhua,Bai Xue	畜牧与兽医	Husbandry and Veterinary M	47	2	19-24	2015	-
4148	阳市食品中食源性致病菌	ults of foodborne pathoge	英,罗赟,吴晓红,何玲玲,凡	Luo Yun,Wu Xiaohong,He L	预防医学情报杂志	of Preventive Medicine Info	31	12	943-946	2015	-
4149	性肠炎的病原菌分布及药	bility analysis of pathogeni	医峰,袁琼琼,侯一民,干安	uan Qiongqiong,Hou Yimin	中华医院感染学杂志	ournal of Hospital Infectiou	-	8	1735-1737	2015	.11816/cn.ni.2015-144891
4150	门菌食物中毒实验室检测	ity analysis of a case of Sal	民,杨瑞军,陈旭富,吕磊,赵	ang Ruijun,Chen Xufu,Lu Le	中国卫生检验杂志	ese Journal of Health Inspe	25	20	3541-3542,3545	2015	-
4151	沙门氏菌的分离与耐药性	tance analysis of Salmonel	雄伟, 狄文婷, 吴静, 江洁,	ei ,Di Wenting ,Wu Jing ,Ji J	农业科学与技术（英文版）	cience and Technology (En	-	10	2286-2289	2015	sn.1009-4229.2015.10.048
4152	沙门菌和大肠杆菌耐药性	ance of Salmonella and Esc	刘艳红,李颖,吕淑霞.	u Yanhong,Li Ying,Lu Shuxi	畜牧与兽医	Husbandry and Veterinary M	47	12	160-161	2015	-
4153	江省食源性疾病事件流行	characteristics of foodbor	,张荷香,齐小娟,朱大方,王	ang Hexiang,Qi Xiaojuan,Zh	中国食品卫生杂志	hinese Food Hygiene Journ	27	2	120-123	2015	).13590/j.cjfh.2015.02.005
4154	原料奶食品病原微生物检	hogenic microorganisms in	李涛.	Li Tao.	预防医学情报杂志	of Preventive Medicine Info	31	10	803-805	2015	-
4155	深圳市食物中毒流行病学	iological characteristics of	彭朝琼,刘建平,黄海雄,王丹	ong,Liu Jianping,Huang Hai	江苏预防医学	iangsu Preventive Medicine	-	3	81-82	2015	sn.1006-9070.2015.03.032
4156	登堡沙门氏菌引起的食物	od poisoning caused by Sal	钱霞,任新红,肖美,李尤佳	ia,Ren Xinhong,Xiao Mei,Li	医药前沿	Medical Frontier	-	2	126-126,127	2015	sn.2095-1752.2015.02.090
4157	复发成人血栓性微血管病	mbotic microangiopathy in	,陈凤梅,王瑞强,翟子涵,周	gmei,Wang Ruiqiang,Zhai	中华实用诊断与治疗杂志	nal of Practical Diagnosis ar	29	4	360363	2015	sn.1674-3474.2015.04.017
4158	蛋沙门菌污染检测及分离	in eggs and analysis of dru	华,张正帆,刘少文,张颖,陈	g Zhengfan,Liu Shaowen,Zh	畜牧与兽医	Husbandry and Veterinary M	47	6	109-112	2015	-
4159	来源沙门菌血清型分布及	g resistance characteristics	凌云,廖和壮,权怡,黄君,曾	o Hezhuang,Quan Yi,Huan	中国媒介生物学及控制杂志	ournal of Vector Biology an	26	3	266-270	2015	sn.1003.4692.2015.03.012
4160	中沙门菌的定量检测及风	d risk assessment of Salmo	张玲,刘桂华.	Zhang Ling,Liu Guihua.	中国微生态学杂志	inese Journal of Microecolo	27	8	907-909	2015	381/j.cnki.cjm.201508010
4161	物中毒疫情的实验室诊断	ability analysis of a preserv	余军,何雪连,蒋双,陈倚.	,He Xuelian,Jiang Shuang,C	饮食保健	Diet and health care	2	16	5-6	2015	-
4162	市市售食品中食源性致病	odborne pathogenic bacter	泽燕,刘思超,杨剑英,方巧	u Sichao,Yang Jianying,Fang	实用预防医学	ractical preventive medicin	22	7	812-815	2015	sn.1006-3110.2015.07.013
4163	京市密云县腹泻患者病原	genic bacteria in diarrhea	,冯宝立,郑兰紫,蒋会婷,周	Baoli,Zheng Lanzi,Jiang Hu	职业与健康	Occupation and health	31	18	2473-2475	2015	-
4164	淀区食源性疾病主动监测	results of foodborne disea	伟,纪黎黎,陈玉,孙晓华,谢	Wei,Ji Lili,Chen Yu,Sun Xiaoh	现代预防医学	modern preventive medicin	42	19	3498-3500	2015	-
4165	泻患者病原菌检测与流行	nalysis of pathogenic bacter	黄薇,李敏璐,施晓莺,韩英	Wei,Li Minlu,Shi Xiaoying,H	医学信息	medical information	-	30	256-256	2015	sn.1006-1959.2015.30.383
4166	鸡中沙门菌和弯曲菌协同	lmonella and Campylobact	胡豫杰,王晔茹,李凤琴.	u Yujie,Wang Yeru,Li Fengq	卫生研究	health research	44	1	68-72,76	2015	-
4167	餐饮业自制食品微生物污	al contamination of homem	萍,谢强,况伟,赖纪年,朱	e Qiang,Kuang Wei,Lai Jinn	中国药事	hinese pharmaceutical affai	-	4	353-356	2015	-
4168	市细菌感染性腹泻病原菌	results of bacterial infectio	任启智,刘洪博,张燕,吴艳	,Liu Hongbo,Zhang Yan,Wu	中国公共卫生管理	ese public health managen	31	4	561-562	2015	-
4169	门菌食物中毒的实验室诊	nd analysis of a case of salr	于慧霞,杜小丽,崔志刚,梁	uixia,Du Xiaoli,Cui Zhigang	疾病监测	disease surveillance	30	5	381-384	2015	sn.1003-9961.2015.05.010
4170	病症候群细菌性病原学监	bacterial pathogens of diar	李蓉,虎凤侠.	Rong,Tiger and Phoenix Ma	安徽预防医学杂志	i Journal of Preventive Med	21	5	366-367	2015	-
4171	-2014年鸡源沙门菌耐药性	hanges in drug resistance o	岳秀英,汪开毓.	Yue Xiuying,Wang Kaiyu.	中国兽医科学	Chinese Veterinary Science	45	5	544-550	2015	-
4172	源沙门氏菌的分离鉴定及	stance status of Salmonella	吴雪娇,吴科敏,焦鹏涛,韦平	ao,Wu Kemin,Jiao Pengtao	广西畜牧兽医	nal Husbandry and Veterin	-	5	227-230	2015	sn.1002-5235.2015.05.001
4173	区食品中致病菌监测与流	sis of pathogenic bacteria i	野,闫鹏,杨元斌,章丹阳,胡	g,Yang Yuanbin,Zhang Dan	中国食品卫生杂志	hinese Food Hygiene Journ	27	5	562-568	2015	).13590/j.cjfh.2015.05.018
4174	物中毒事件的调查处置与	rmation release of a cross-	展宏,蒋洪明,陆雪娣,宋兵	,Jiang Hongming,Lu Xuedi,	中国食品卫生杂志	hinese Food Hygiene Journ	27	6	703-706	2015	).13590/j.cjfh.2015.06.024
4175	仙台沙门菌疾病的微生物	sis of Salmonella Sendai di	李维萍,刘岳,高娴,王代录	ping,Liu Yue,Gao Xian,Wang	黑龙江畜牧兽医（下半月）	dry and Veterinary Medicin	-	1	122-124	2015	-
4176	新疆伤寒、副伤寒流行病	characteristics of typhoid	塞英,夏依旦·吾甫尔,顾本	ying,Xia Yidan·Wufu'er,Gu	现代预防医学	modern preventive medicin	42	15	2694-2696	2015	-
4177	分住院儿童感染性腹泻的	ctious diarrhea in some ho	杨全英,张梦寒,杨波,傅春玲	Zhang Menghan,Yang Bo,Fu	职业与健康	Occupation and health	31	3	358-361	2015	-
4178	2013年感染性腹泻病原体	lysis of annual infectious d	高桂玲,吴毅凌,奚超,张清慧	ng,Wu Yiling,Xi Chao,Zhang	医学信息	medical information	-	20	198-199	2015	-
4179	感染革兰阴性杆菌的构成	f Gram-negative bacilli in c	郑华月,郑春阳,刘纳.	Huayue,Zheng Chunyang,L	中华医院感染学杂志	ournal of Hospital Infectiou	-	22	5236-5238	2015	.11816/cn.ni.2015-142973

4180	年滁州市食源性致病菌监	ysis of foodborne pathogen	磊,陈秀红,朱慧琳,杨步	hen Xiuhong,Zhu Huilin,Ya	食品安全质量检测学报	f Food Safety and Quality I	-	1	372-376	2015	-
4181	房食品污染引起食品安全	incident caused by food co	符艳,徐艳钢.	Fu Yan,Xu Yangang.	现代预防医学	modern preventive medicin	42	12	2167-2169	2015	-
4182	3年饮食从业人员肠道沙门	f enteric Salmonella testing	青,黄飞,吴科明,蒙国伟,刘	Fei,Wu Keming,Meng Guo	右江民族医学院学报	ujiang Medical College for	-	3	476-477	2015	sn.1001-5817.2015.03.055
4183	食品中微生物致病菌污染	hogenic bacteria contamin	曹迎春,高儒,李幂,王丽霞	ngchun,Gao Ru,Li Mi,Wang	中国卫生产业	China health industry	-	13	166-168	2015	-
4184	加工、运输、销售过程中	ing broiler breeding, slaugh	慧萍,孟丽,牛世文,张雪	g,Meng Li,Niu Shiwen,Zhar	河南预防医学杂志	n Journal of Preventive Me	26	4	324-326	2015	m.1006-8414.2015.04.030
4185	染沙门菌血清型分布和耐	distribution and drug resi	潘慧芳,李艳红,刘艳梅.	Huifang,Li Yanhong,Liu Yan	中国妇幼保健	ina maternal and child hea	30	8	1196-1197	2015	ssn.1001-4411.2015.08.17
4186	年其他感染性腹泻流行病	epidemiological characteris	郭丽丽,曾育富,谭小华,张	li,Zeng Yufu,Tan Xiaohua,Z	华南预防医学	uth China Preventive Medic	41	3	233-237	2015	).13217/j.scjpm.2015.0233
4187	市涉及公共卫生从业人员	monella testing results am	吴集才,张敏,吴建英,曹健	i,Zhang Min,Wu Jianying,C	实验与检验医学	imental and Laboratory Me	-	2	242-244	2015	sn.1674-1129.2015.02.045
4188	儿童沙门菌感染流行病	sis of Salmonella infection	张建群,罗学辉,黄绍军.	ianqun,Luo Xuehui,Huang S	疾病监测	disease surveillance	30	9	776-779	2015	sn.1003-9961.2015.09.018
4189	2014年儿童沙门菌分离特	eristics and drug resistance	潘慧芳.	Pan Huifang.	山西医药杂志	Shanxi Medical Journal	-	9	1073-1074	2015	-
4190	区山羊沙门氏菌健康带菌	d drug resistance of Salmor	王晶晶,夏宾雁,岳华,汤承	gjing,Xia Binyan,Yuehua,Ta	中国畜牧兽医	mal husbandry and veterina	42	2	472-477	2015	ki.1671-7236.2015.02.037
4191	链细菌耐药性及其耐药基	ial resistance and drug-resi	吴艺影,曲道峰,韩剑众.	iying,Qu Daofeng,Han Jianz	中国畜牧杂志	na Animal Husbandry Maga	51	6	78-83	2015	sn.0258-7033.2015.06.016
4192	牦牛沙门菌的分离鉴定与	susceptibility test of Salmo	逯玉,谢秀梅.	Luyu,Xie Xiumei.	黑龙江畜牧兽医（下半月）	dry and Veterinary Medicin	-	10	121-123	2015	-
4193	门氏菌的分离鉴定及药敏	tion and drug susceptibility	周忠坤.	Zhou Zhongkun.	现代农业	modern agriculture	-	9	94-96	2015	sn.1008-0708.2015.09.071
4194	售水产品食源性致病菌污	ne pathogenic bacteria in c	仪,张维蔚,邓志爱,余超,林	Weiwei,Deng Zhiai,Yu Chao	中国食品卫生杂志	hinese Food Hygiene Journ	27	3	294-297	2015	).13590/j.cjfh.2015.03.016
4195	肉中沙门菌的血清型及耐	ce of Salmonella in comme	娇,莫国东,焦鹏涛,王海源	uodong,Jiao Pengtao,Wang	黑龙江畜牧兽医（上半月）	ndry and Veterinary Medic	-	8	281-284	2015	-
4196	奶牛子宫内膜炎病原菌敏	nic bacteria of endometrit	丽,李静,杨利国,郭爱珍,李	Jing,Yang Liguo,Guo Aizher	中国奶牛	Chinese cow	-	21	16-19	2015	sn.1004-4264.2015.21.006
4197	夫登堡沙门菌耐药性及脉	ophoresis typing of Salmor	民,杨筱薇,石维敏,陶燕,潘	ng Xiaowei,Shi Weimin,Tac	华南农业大学学报	f South China Agricultural U	-	1	113-116	2015	-
4198	港市感染性腹泻监测病例	nfectious diarrhea surveilla	秀,何德辉,孔庆麟,邓星超	hui,Kong Qinglin,Deng Xin	应用预防医学	applied preventive medicin	-	4	253-254	2015	n.1673-758X.2015.04.014
4199	品生加工沙门氏菌污染的	amination in the productio	马艳,陈迪.	Ma Yan,Chen Di.	福建水产	Fujian aquatic products	37	2	153-156	2015	12/j.cnki.fjsc.2015.02.009
4200	沙门菌感染流行病学特征	cs and drug resistance of S	修宁宁,郑碧英,辛青松.	gning,Zheng Biying,Xin Qin	华临床医师杂志（电子版	urnal of Clinicians (electron	-	13	2530-2534	2015	sn.1674-0785.2015.13.018
4201	土壤肠道菌分离鉴定及药	ensitivity analysis of soil in	莎,赵珈禾,余华军,孙裕	Zhao Jiahe,Yu Huajun,Sun Y	中国兽医杂志	Chinese Veterinary Journal	51	2	76-80	2015	sn.0529-6005.2015.02.028
4202	市售食品中致病菌检测结	lts of pathogenic bacteria i	李辉,郑卉,陈福尊,李增敏	Zheng Hui,Chen Fuzun,Li Ze	医学动物防制	medical animal control	31	7	779-781	2015	0.7629/yxdwfz201507024
4203	培养法检测食品从业人员	he detection of Salmonella	韩毅,孙燕萍,周虹,毛菲菲	un Yanping,Zhou Hong,Ma	中国食品卫生杂志	hinese Food Hygiene Journ	27	2	132-135	2015	).13590/j.cjfh.2015.02.008
4204	江从进口鱼粉中检出沙门	tected in imported fishmea	麦文伟.	Mai Wenwei.	海洋与渔业	Oceans and Fisheries	0	9	13	2015	-
4205	泻常见病原菌的分布和耐	nalysis of common pathoge	,祝慧华,沈英莲,朱玉龙,	ihua,Shen Yinglian,Zhu Yul	中国乡村医药	Chinese rural medicine	22	23	65-66	2015	sn.1006-5180.2015.23.038
4206	深圳市食品中沙门菌血清	ypes and drug resistance o	平芳,陈妙玲,冼慧霞,林爱	ang,Chen Miaoling,Xian Hu	中国卫生检验杂志	ese Journal of Health Inspe	25	2	289-291,294	2015	-
4207	菌病的病原分离·鉴定及药	l disease in minks·Identific	飞,王赠超,邹玲,李文立,任	g Xiancao,Zou Ling,Li Wen	安徽农业科学	Anhui Agricultural Sciences	-	12	391-392	2015	sn.0517-6611.2015.12.134
4208	贺菌感染的流行趋势及耐	g resistance patterns of Sh	新宝,王晓红,金汇明,胡家	o,Wang Xiaohong,Jin Huim	中国感染与化疗杂志	urnal of Infection and Che	-	1	32-37	2015	sn.1009-7708.2015.01.008
4209	京市门头沟区肠道病原菌	sults of intestinal pathogen	吕秋艳,王志越,褚添,苏健	yan,Wang Zhiyue,Chu Tian,	热带医学杂志	journal of tropical medicine	15	8	1141-1143	2015	-
4210	014年食源性疾病暴发事件	ological analysis of annual	杨华.	Yang Hua.	卫生软科学	health soft sciences	29	12	798-801	2015	-
4211	从业人员大便培养沙门菌	e of Salmonella in stool cul	朱惠芳.	Zhu Huifang.	中国卫生检验杂志	ese Journal of Health Inspe	25	15	2635-2636,2638	2015	-
4212	自办宴席引发食物中毒的	od poisoning caused by sel	芦丽嫦,张峰,戴正,顾敏霞	ng,Zhang Feng,Dai Zheng,G	中国农村卫生事业管理	na's rural health managem	35	4	459-461	2015	-
4213	4年常熟市食品安全风险	fety risk monitoring analys	冬月,王俭,常伟冰,陆卫	e,Wang Jian,Chang Weibin	现代预防医学	modern preventive medicin	42	21	3880-3882,3888	2015	-
4214	幼儿腹泻沙门菌感染状况	status and drug resistance	林伟雄,覃卫娟,黄卫彤.	xiong,Qin Weijuan,Huang V	应用预防医学	applied preventive medicin	-	4	232-233,231	2015	n.1673-758X.2015.04.005
4215	腹泻病人沙门菌血清分型	es and drug resistance in p	勇,龙佳丽,侯水平,张欣强	g,Long Jiali,Hou level,Zhang	热带医学杂志	journal of tropical medicine	15	7	975-977	2015	-
4216	市售食品食源性致病菌调	pathogenic bacteria in con	高恒波,王慰.	Gao Hengbo,Wang Wei.	中国病原生物学杂志	ese Journal of Pathogen Bi	10	10	928-930,939	2015	10.13350/j.cjpb.151015

4217	沙门氏菌引起的学校食源性	odborne illness outbreak c	吴高芳,孙敏红.	Wu Gaofang,Sun Minhong.	医学与社会	medicine and society	28	0	42-43	2015	-
4218	州地区肠炎沙门菌主动监	ic characteristics analysis o	晶,刘庭君,梁俊如,邓丽华	iu Tingjun,Liang Junru,Tere	中国卫生检验杂志	ese Journal of Health Inspe	25	21	3707-3708,3711	2015	-
4219	省饲用油脂微生物污染状	ntamination of feed oils a	华,陈甫,邹玲,朱连勤,徐	Chen Fu,Zou Ling,Zhu Lianq	中国畜牧杂志	a Animal Husbandry Maga	51	12	58-61,67	2015	sn.0258-7033.2015.12.013
4220	中沙门菌污染状况及其血	its serotype and molecula	王勤,刘军,张杰,王延东.	in,Liu Jun,Zhang Jie,Wang Y	现代预防医学	modern preventive medicin	42	16	2924-2926,2929	2015	-
4221	京某城区感染性腹泻患者	bacteria in patients with i	,刘淑岭,高波,胡三梅,霍	uling,Gao Bo,Hu Sanmei,H	中国感染控制杂志	ese Journal of Infection Co	-	6	361-365	2015	sn.1671-9638.2015.06.001
4222	市某酒店连续4起食物中毒	City continued4food poiso	喜,秦友燕,蒋铁翼,何柳	xi,Qin Youyan,Jiang Tieyi,H	华夏医学	Chinese Medicine	28	5	127-128	2015	-
4223	起的食物中毒的流行病学	test analysis of a case of fo	璟,申艳琴,甘连军,张慧	hen Yanqin,Gan Lianjun,Zh	中国卫生检验杂志	ese Journal of Health Inspe	25	5	756-758	2015	-
4224	血培养中病原菌检出及构	sition analysis of pathoge	,卢赞,任宝军,王霞,尹丽	,Ren Baojun,Wang Xia,Yin	医学检验与临床	edical Laboratory and Clini	-	2	41-43	2015	sn.1673-5013.2015.02.016
4225	吸综合征病原菌的分离与	of pathogenic bacteria of s	娟,王奇惠,时永强,曲伟	Wang Qihui,Shi Yongqiang,	畜牧与饲料科学	Livestock and feed science	36	6	155-158	2015	sn.1672-5190.2015.06.022
4226	区鸭源沙门氏菌分离鉴定	lysis of Salmonella originat	文灿,程平,谭天,许凯迪,	can,Cheng Ping,Tan Tian,X	中国预防兽医学报	rnal of Preventive Veterina	37	4	262-265	2015	ssn.1008-0589.2015.04.06
4227	寒沙门菌食物中毒疫情的	epidemic of Salmonella typ	李晓莉,蒲剑,黄静.	Li Xiaoli,Pu Jian,Huang Jing.	中国卫生检验杂志	ese Journal of Health Inspe	25	13	2123-2125	2015	-
4228	食源性沙门菌脉冲场凝胶	ctrophoresis Typing of Foo	赵薇,刘桂华,遇晓杰.	o Wei,Liu Guihua,Meet Xia	中国卫生检验杂志	ese Journal of Health Inspe	25	13	2171-2172,2175	2015	-
4229	服务业从业人员体检沙门	ults of physical examinatio	海燕,秦剑秋,覃巍巍,黄	,Qin Jianqiu,Tan Weiwei,H	应用预防医学	applied preventive medicin	-	3	190-191	2015	n.1673-758X.2015.03.020
4230	合并细菌性腹泻的病原学	nalysis of rotavirus combin	罗伟波,林静,陈钧顺.	Weibo,Lin Jing,Chen Junsh	黑龙江医学	Heilongjiang Medicine	-	3	294-295	2015	sn.1004-5775.2015.03.037
4231	感染猪霍乱沙门菌8例临	on with Salmonella cholera	宗萍,詹震泽.	Zong Ping,Zhan Zhenze.	吉林医学	Jilin Medicine	-	3	470-470	2015	sn.1004-0412.2015.03.056
4232	副溶血性弧菌食物中毒1例	haemolyticus food poisoni	姚淑连,韦小民.	Yao Shulian,Wei Xiaomin.	河北医学	Hebei Medicine	-	2	352-352	2015	sn.1006-6233.2015.02.062
4233	氏菌的分离鉴定及血清型	sensitivity analysis of path	凤梅,邹联斌,许心婷,王	ei,Zou Lianbin,Xu Xinting,	中国畜牧兽医	nal husbandry and veterina	42	8	2160-2168	2015	ki.1671-7236.2015.08.034
4234	某种禽场鸡胚沙门氏菌的	bryonated chickens in a ce	张丽芳,肖桥,罗薇.	ang Lifang,Xiao Qiao,Luo W	中国畜牧兽医	nal husbandry and veterina	42	6	1571-1579	2015	ki.1671-7236.2015.06.037
4235	培养病原菌的分布与耐药	esistance of pathogenic ba	邱付兰,钟荣荣,黄浩南.	n,Zhong Rongrong,Huang	实验与检验医学	imental and Laboratory Me	-	2	185-187	2015	sn.1674-1129.2015.02.020
4236	食品中食源性致病菌监测	s of foodborne pathogenic	良,张丽,陈秀英,梅建华,柳	ang Li,Chen Xiuying,Mei Jia	中国卫生检验杂志	ese Journal of Health Inspe	25	1	105-107	2015	-
4237	2013年-2015年食源性致病	-2015Annual prevalence st	骆骋,牛蔚霞.	Luo Cheng,Niu Weixia.	医药前沿	Medical Frontier	-	21	336-337	2015	sn.2095-1752.2015.21.320
4238	014年广西2市2县脑膜炎症	city2County Meningitis Sy	柏青,王鸣柳,曾竣,廖和	,Wang Mingliu,Zeng Jun,Li	应用预防医学	applied preventive medicin	-	5	281-285	2015	n.1673-758X.2015.05.001
4239	枝花市食品中重要食源性	foodborne pathogenic bac	来红,陈嫣,刘相琨,陈鑫	g,Chen Yan,Liu Xiangkun,C	预防医学情报杂志	of Preventive Medicine Info	31	9	703-705	2015	-
4240	2年-2014年食源性致病菌	of annual foodborne path	仁增卓玛.	Rigzin Dolma.	青海医药杂志	Qinghai Medical Journal	45	3	73-74	2015	-
4241	武威市腹泻症候群病例监	diarrhea syndrome cases in	昊,张宏山,杨越,董冰,俞亚	g Hongshan,Yang Yue,Dong	疾病预防控制通报	se Prevention and Control	30	4	59-61	2015	15/j.cnki.jbyfkztb.1503048
4242	区腹泻病病例病原及流行	aracteristics of diarrhea ca	珠,王曙,俪佳莹,舒敏,沈	u,Wang Shu,Li Jiaying,Shu M	职业与健康	Occupation and health	31	18	2504-2507	2015	-
4243	011-2013年食源性致病菌	13Annual foodborne patho	虎生,安静,赵常智,侯元,魏	Quiet,Zhao Changzhi,Hou Y	海峡预防医学杂志	s Journal of Preventive Me	21	2	78-79	2015	-
4244	武威市熟肉制品微生物检	and analysis of cooked mea	董晓芳,黄爱霞.	Dong Xiaofang,Huang Aixia	疾病预防控制通报	se Prevention and Control	30	1	81-82	2015	15/j.cnki.jbyfkztb.1410003
4245	禽源沙门氏菌的分离与鉴	identification of Salmonella	苏战强,沈月,周文宁,王伟,	en Yue,Zhou Wenning,Wan	新疆畜牧业	Xinjiang animal husbandry	-	1	33-34,25	2015	sn.1003-4889.2015.01.013
4246	年吉林省食源性疾病监测	nalysis of foodborne disea	光大,邢扬,孙平辉,翟前	a,Xing Yang,Sun Pinghui,Zh	实用预防医学	ractical preventive medicin	22	7	886-888,封3	2015	sn.1006-3110.2015.07.040
4247	湖北省某高校一起伤寒暴	phoid fever outbreak in a u	军,吴杨,蒋庆,赵球平,叶	Yang,Jiang Qing,Zhao Qiup	疾病监测	disease surveillance	30	11	979-981	2015	sn.1003-9961.2015.11.020
4248	起鼠伤寒沙门氏菌食物中	phimurium food poisoning	温晋华,马玉春.	Wen Jinhua,Ma Yuchun.	河北医学	Hebei Medicine	-	12	2120-2122	2015	sn.1006-6233.2015.12.082
4249	州地区儿童腹泻病原菌分	istance of pathogenic bact	,刘海英,谢永强,钟华敏,	ing,Xie Yongqiang,Zhong H	国际检验医学杂志	onal journal of laboratory	-	10	1351-1353	2015	sn.1673-4130.2015.10.013
4250	门氏菌所引起的食物中毒	case of food poisoning ca	彤,李钞,熊进超,罗婕,周	i Chao,Xiong Jinchao,Luo Ji	医学美学美容（中旬刊）	esthetics and Cosmetology	24	6	840	2015	-
4251	源性致病菌污染状况及相	amination status and relat	,张有良,王义平,何军霞,	ouliang,Wang Yiping,He Ju	疾病预防控制通报	se Prevention and Control	30	6	66-68,74	2015	15/j.cnki.jbyfkztb.1509005
4252	屠宰环节沙门菌流行病学	analysis of Salmonella in b	娟,黄秀梅,王君玮,杨瑞梅	g Xiumei,Wang Junwei,Yan	中国家禽	Chinese poultry	37	7	67-70	2015	sn.1004-6364.2015.07.017
4253	甘南州食品中食源性致病	borne pathogenic bacteria	吕卫民,苏玲玉,刘芳,杨勇	min,Su Lingyu,Liu Fang,Yan	职业与健康	Occupation and health	31	18	2479-2481,2484	2015	-

4254	部分畜禽养殖场沙门氏菌的	monella in some livestock	向前,魏强,胡龙明,叶承	n,Wei Qiang,Hu Longming,	交通大学学报（农业科学	ao Tong University (Agricu	33	6	36-40	2015	N.1671-9964.2015.06.006
4255	馆餐服人员肠道沙门氏菌	ric Salmonella in catering st	,丛黎明,兰智杰,任硕,李	ong Liming,Lan Zhijie,Ren	河南预防医学杂志	n Journal of Preventive Me	26	1	14-16,23	2015	m.1006-8414.2015.01.004
4256	儿感染性腹泻的病原学及	d drug susceptibility of infe	张蔚.	Zhang Wei.	医学信息	medical information	-	16	292-292	2015	sn.1006-1959.2015.16.446
4257	开封市肉鸡产业链沙门菌	contamination in the broi	,陈磊,张春艳,阎学燕,刘	ei,Zhang Chunyan,Yan Xue	职业与健康	Occupation and health	31	16	2211-2213	2015	-
4258	表水细菌污染状况与水质	ation status of typical surf	然,杨忠委,刘美霞,钟启平,	g Zhongwei,Liu Meixia,Zho	环境与健康杂志	rnal of Environment and He	32	5	408-411	2015	ki.1001-5914.2015.05.009
4259	部分市售婴幼儿食品卫生	tus of some commercially a	王卓,郑文龙,赵帅,江国虹	eng Wenlong,Zhao Shuai,J	现代预防医学	modern preventive medicin	42	20	3682-3683,3709	2015	-
4260	直医院沙门菌属菌群分布	ution and drug resistance i	黄宝兴,马东礼.	Huang Baoxing,Ma Dongli.	实用预防医学	ractical preventive medicin	22	7	880-881,872	2015	sn.1006-3110.2015.07.037
4261	工生产链中沙门氏菌的污	on in the broiler slaughteri	保林,张春艳,黄淑华,陈磊,	ng Chunyan,Huang Shuhua	医学动物防制	medical animal control	31	11	1206-1209	2015	0.7629/yxdwfz201511007
4262	12-2014年食源性致病菌的	contamination status of fo	强,罗瑜,杨美红,李文伟,李	Luo Yu,Yang Meihong,Li W	公共卫生与预防医学	Health and Preventive Me	26	5	89-91	2015	-
4263	鸡源致病性沙门氏菌的耐	hogenic Salmonella origina	彬彬,刘慧,李冰,李培,郭桂	in,Liu Hui,Li Bing,Li Pei,Gu	黑龙江畜牧兽医（上半月）	ndry and Veterinary Medic	-	11	173-175	2015	-
4264	门氏菌血清型及对β-内酰	imal sources and their resp	,林居纯,张飞,舒刚,赵玲,	un,Zhang Fei,Shu Gang,Zha	食品科学	food science	36	7	101-104	2015	pkx1002-6630-201507019
4265	鸡生产过程中沙门菌污染	a contamination during bro	刘军.	Liu Jun.	中国食品卫生杂志	hinese Food Hygiene Journ	27	6	661-664	2015	.13590/j.cjfh.2015.06.014
4266	区鸭蛋沙门氏菌的分离鉴	tibility test of Salmonella f	邢春华,马晓龙,柏雪,张正	a,Ma Xiaolong,Bai Xue,Zha	食品工业科技	Food Industry Technology	36	6	72-74,85	2015	sn1002-0306.2015.06.007
4267	速冻水饺、汤圆等制品卫	ick-frozen dumplings, gluti	樊亚丽.	Fan Yali.	河南预防医学杂志	n Journal of Preventive Me	26	1	84-85	2015	m.1006-8414.2015.01.028
4268	立自制熟肉制品中致病菌	ation in homemade cooke	瑞英,马永华,单宏,高春	ng,Ma Yonghua,Shan Hong	食品工程	food engineering	-	2	41-44,59	2015	sn.1673-6044.2015.02.013
4269	鲜鸡类食品中沙门氏菌的	a contamination in retail fr	吴云凤,袁宝君.	Wu Yunfeng,Yuan Baojun.	医科大学学报（自然科学	Medical University (Natur	35	2	284-287	2015	655/NYDXBNS20150235
4270	门菌分离株的毒力及耐药	stance characteristics of sw	杰,王俊峰,王晓楠,王龙,	g Junfeng,Wang Xiaonan,W	中国兽医科学	Chinese Veterinary Science	45	2	190-194	2015	-
4271	013年食品安全风险监测	ysis of annual food safety r	徐丽红.	Xu Lihong.	中国卫生检验杂志	ese Journal of Health Inspe	25	5	712-714	2015	-
4272	~2014年疑似细菌性食物	2014Analysis of suspected	苏兰妹,徐伟兵,袁花艳.	nmei,Xu Weibing,Yuan Hu	甘肃医药	Gansu Medicine	-	10	782-784	2015	-
4273	炎沙门氏菌分离株毒力基	of Salmonella Enteritidis is	,周秀娟,许学斌,史贤明,	u Xiujuan,Xu Xuebin,Shi Xi	食品科学	food science	36	14	165-170	2015	pkx1002-6630-201514032
4274	京市顺义区感染性腹泻病	athogenic bacteria of infec	涛,王园园,李颖,朱美娟,王	g Yuanyuan,Li Ying,Zhu Me	实用预防医学	ractical preventive medicin	22	10	1244-1246	2015	.1006-3110.2015.010.030
4275	沙门氏菌Ⅰ类整合子与耐	gene cassette of Salmone	谭艾娟,吕世明,孙硕,华夏	Aijuan,Lu Shiming,Sun Shuo	山东农业科学	handong Agricultural Scienc	-	1	105-108	2015	sn.1001-4942.2015.01.024
4276	鸡主要疫病发生及流行情	prevalence of major disea	佩国,张建文,张香斋,史秋	ianwen,Zhang Xiangzhai,S	黑龙江畜牧兽医（下半月）	dry and Veterinary Medicin	-	8	92-94	2015	-
4277	从业人员致泻性大肠埃希	nic Escherichia coli among	邓艳华.	Deng Yanhua.	实验与检验医学	imental and Laboratory Me	-	2	161-162	2015	sn.1674-1129.2015.02.011
4278	新罗歇尔沙门菌致败血症1	ew Rochelle causing septice	,高波,霍哲,任艳芳,何悦,	,Huo Zhe,Ren Yanfang,He	临床检验杂志	journal of clinical testing	33	2	160	2015	602/j.cnki.jcls.2015.02.23
4279	泰安市市售膨化食品卫生	atus of commercially availa	张新峰,郑金华,蔺胜田.	infeng,Zheng Jinhua,Lin Sh	中国卫生检验杂志	ese Journal of Health Inspe	25	2	247-249	2015	-
4280	呼和浩特市食源性疾病致	d analysis of foodborne dis	罗云,赵锡安,王亚丽,杨月	un,Zhao Xian,Wang Yali,Yan	疾病监测与控制	ease surveillance and cont	9	6	412-413	2015	-
4281	鸡源沙门菌血清型及耐药	istance status of Salmonell	娇,王海源,吴科敏,莫国东,	g Haiyuan,Wu Kemin,Mo G	中国兽医杂志	Chinese Veterinary Journal	51	9	88-90	2015	sn.0529-6005.2015.09.033
4282	区农村集体聚餐熟肉制品	at products at rural collectiv	陈志利,朱力涛,刘辉.	Chen Zhili,Zhu Litao,Liu Hui	职业与健康	Occupation and health	31	23	3240-3242	2015	-
4283	肃省武威市食品中食源性	dborne pathogenic bacteri	雷诚,常全娥.	Lei Cheng,Chang Quan'e.	疾病预防控制通报	se Prevention and Control	30	6	61-63	2015	15/j.cnki.jbyfkztb.1507016
4284	并发噬血细胞综合征及肠	hemophagocytic syndrom	从超凡,杨京.	rom extraordinary,Yang Jin	中华传染病杂志	se Journal of Infectious Dis	-	9	560-561	2015	sn.1000-6680.2015.09.012
4285	101例食源性疾病监测检	01Study on the results of fo	况强华,周军华,赖玉珍.	Qianghua,Zhou Junhua,Lai	饮食保健	Diet and health care	2	11	23-24	2015	-
4286	年大连市食源性致病菌污	dborne pathogenic bacteria	徐丹,栾明春,陈玉凤.	an,Luan Mingchun,Chen Yu	中国卫生工程学	Chinese sanitary engineerin	14	1	45-46,49	2015	-
4287	2～2015年鸡关节炎病例	n and Analysis of Pathogen	,侯雪娇,吴科敏,陈果,刘	uejiao,Wu Kemin,Chen Gu	中国家禽	Chinese poultry	37	23	68-70	2015	sn.1004-6364.2015.23.018
4288	菌致脊柱骨髓炎并椎旁脓	s and paravertebral absces	,霍娜,王艳,蔺小红,万华,	ang Yan,Lin Xiaohong,Wan	中华传染病杂志	se Journal of Infectious Dis	-	7	402-402	2015	sn.1000-6680.2015.07.006
4289	县9例都柏林型沙门氏菌	ity9Analysis of case data o	巍,郑德生,滕克强,张杰,冯	g Desheng,Teng Keqiang,Z	首都公共卫生	capital public health	9	4	173-175,182	2015	-
4290	龙岩市食源性致病菌监测	ults of foodborne pathoge	红,何云,林伟,张彦锋,曹	Yun,Lin Wei,Zhang Yanfeng	基层医学论坛	Primary Medicine Forum	-	32	4566-4567	2015	-

4291	市光明新区感染性腹泻病	of infectious diarrhea path	,罗淑华,汪东篱,姚炜,吴补	uhua,Wang Dongli,Yao Wei	职业与健康	Occupation and health	31	3	354-357	2015	-
4292	腹泻细菌性病原的分离鉴	alysis of Bacterial Pathoge	刘永明,王慧,王胜义,崔东安	ing,Wang Hui,Wang Sheng	西北农业学报	urnal of Northwest Agricult	24	12	35-39	2015	sn.1004-1389.2015.12.005
4293	年广东省肠炎沙门菌耐药	ance of Salmonella Enteriti	何冬梅,效拟,肖媛,孙九峰,柯	imitate,Xiao Yuan,Sun Jiufe	中华疾病控制杂志	hese Journal of Disease Cor	19	3	269-272	2015	/j.cnki.zhjbkz.2015.03.015
4294	牡丹江市食品中沙门氏菌	he distribution of Salmonel	,刘洋,胡锋,吕昌武,刘凤海	Yang,Hu Feng,Lu Changwu,	医学动物防制	medical animal control	31	9	1005-1007	2015	0.7629/yxdwfz201509020
4295	源性疾病主动监测系统监测	f Changsha Foodborne Dis	陈艳,朱彩明,周银柱.	n Yan,Zhu Caiming,Zhou Yin	实用预防医学	ractical preventive medicin	22	9	1115-1117	2015	sn.1006-3110.2015.09.031
4296	沙门菌食物中毒实验室检测	sceptibility analysis of Saln	吴南卫,邓瑶,莫丽娟.	u Nanwei,Deng Yao,Mo Liju	现代预防医学	modern preventive medicin	42	17	3216-3217,3224	2015	-
4297	连市食品中食源性致病菌	on status of foodborne pat	陈玉凤,栾明春,吕秋月,任月	g,Luan Mingchun,Lu Qiuyu	医学动物防制	medical animal control	31	1	7-9	2015	0.7629/yxdwfz201501003
4298	玛依市夏季细菌性腹泻病	erial diarrheal diseases in s	,黄玉莲,帕力旦·吾孜克,袁	ang Yulian,Paridan·Wuzike	疾病预防控制通报	se Prevention and Control	30	6	19-20,48	2015	15/j.cnki.jbyfkztb.1507011
4299	引起胃部不适症状投诉的	s about Stomach Discomfo	祝贵才.	Zhu Guicai.	基层医学论坛	Primary Medicine Forum	19	17	2397-2398	2015	-
4300	市入境水产品致病菌污染	thogenic bacteria in aquati	繁星,曾爱英,潘光合,苏德	ng,Zeng Aiying,Pan Guangh	中国国境卫生检疫杂志	ontier Health and Quarantin	38	4	273-275,278	2015	/j.1004-9770.2015.04.012
4301	食水产品致病菌污染及安	ety evaluation of raw aqua	元,秦峥,吴世杰,李寅生,丁	Zheng,Wu Shijie,Li Yinshen	食品安全质量检测学报	f Food Safety and Quality I	-	4	1434-1439	2015	-
4302	堡沙门氏菌致老年患者腹	berg causes diarrhea in eld	盛,陈俊,邹晓艳,张义,朱珍	,Chen Jun,Zou Xiaoyan,Zha	临床消化病杂志	nal of Clinical Gastroentero	-	2	116-116	2015	ssn.1005-541X.2015.02.18
4303	微生物污染情况及大肠菌群	ial contamination and colif	李梓娴,速存芬,陈磊,刘辉光	,Sucunfen,Chen Lei,Liu Hu	中国卫生产业	China health industry	-	12	192-194	2015	-
4304	级医院310例食源性腹泻病	310Detection and Analysis	章溢峰,柏品清,傅灵菲.	ng Yifeng,Bai Pinqing,Fu Lin	上海预防医学	hanghai Preventive Medicin	27	11	715-717	2015	-
4305	儿感染性腹泻病原学特征	ristics of infectious diarrhe	商晓春,周晓红,帅慧群.	ochun,Zhou Xiaohong,Shua	疾病监测	disease surveillance	30	10	855-860	2015	sn.1003-9961.2015.10.015
4306	猪源沙门氏菌及耐药性变	la originating from pigs an	毓,吴晓岚,陆强,孙吉,宁宜	Wu Xiaolan,Lu Qiang,Sun J	四川动物	sichuan animals	34	5	707-713	2015	.issn.1000-7083.20150099
4307	生鲜鸡肉沙门氏菌污染状况	onella contamination in fr	,刘杰,阎学燕,黄淑华,陈磊	,Yan Xueyan,Huang Shuhu	河南预防医学杂志	n Journal of Preventive Me	26	6	438-439,443	2015	m.1006-8414.2015.06.013
4308	沙门菌耐药分子流行病学	cal characteristics of drug	汤泓,李颖,石维敏,张静,李勇	Li Ying,Shi Weimin,Zhang J	疾病监测	disease surveillance	30	1	50-57	2015	sn.1003-9961.2015.01.014
4309	冷鲜猪肉中沙门氏菌污染	lla contamination in chilled	心,陈正涛,王方昆,王淑静	Zhengtao,Wang Fangkun,W	山东畜牧兽医	mal Husbandry and Veteri	-	9	60-61,62	2015	sn.1007-1733.2015.09.039
4310	沙门氏菌的平板计数检测	unting detection method o	柯璐,林霖,陈彬,戴晓丽,张	in Lin,Chen Bin,Dai Xiaoli,Z	中国饲料	china feed	-	1	25-26,32	2015	nki.cn11-2975/s.20150107
4311	宴餐引起沙门菌食物中毒	Salmonella Food Poisoning	李凤元.	Li Fengyuan.	现代医药卫生	modern medicine and healt	-	z1	111-113	2015	sn.1009-5519.2015.25.058
4312	源沙门菌的耐药基因流行情	ce gene prevalence of poul	小坚,廖晓萍,黄婷,方亮星,刘	Xiaoping,Huang Ting,Fang L	中国家禽	Chinese poultry	37	18	63-65	2015	sn.1004-6364.2015.18.019
4313	市售食品中食源性致病菌监	pathogenic bacteria in com	,葛少锋,倪丰安,强丽红,冯	feng,Ni Feng'an,Strong red	疾病预防控制通报	se Prevention and Control	30	4	70-73	2015	15/j.cnki.jbyfkztb.1412046
4314	市昌平区其他感染性腹泻流	eristics of other infectious	马树波,王秀云,唐雅清.	hubo,Wang Xiuyun,Tang Ya	职业与健康	Occupation and health	31	14	1935-1938	2015	-
4315	13年食源性疾病主动监测	of annual foodborne disea	虞吉寅,张博,任宜,赵建刚	n,Zhang Bo,Ren Yi,Zhao Jia	中国卫生检验杂志	ese Journal of Health Inspe	25	4	557-558	2015	-
4316	酒店两起沙门菌食物中毒的	ases of salmonella food poi	陈清凉,肖锦晖,张震文,茌静	,Xiao Jinhui,Zhang Zhenwe	中国卫生检验杂志	ese Journal of Health Inspe	25	12	1946-1947,1951	2015	-
4317	0-2012年腹泻病例沙门菌	hella surveillance and mole	灵,陈真,姚红,王斌,汤显,陈	hen,Yao Hong,Wang Bin,Ta	上海预防医学	hanghai Preventive Medicin	-	3	119-124	2015	-
4318	年宝鸡市食源性致病菌监测	g results of foodborne path	,武永平,杨海峰,薛莉,张克	ngping,Yang Haifeng,Xue L	医学动物防制	medical animal control	31	12	1324-1327	2015	0.7629/yxdwfz201512009
4319	门菌、志贺菌血清凝集的肠	eriaceae Serum Agglutinate	凌勤芳.	Ling Qinfang.	上海预防医学	hanghai Preventive Medicin	27	11	727-728	2015	-
4320	沙门菌胃肠道感染235例分	trointestinal infection235E	绪林,林东昉,徐晓刚,卜淑	in Dongfang,Xu Xiaogang,E	中国临床医学	Chinese clinical medicine	-	3	370-373	2015	-
4321	腹泻病原菌检测分析及耐药	ce research on diarrhea pa	冯军军,姚素霞.	Feng Junjun,Yao Suxia.	中国基层医药	China primary medicine	-	16	2441-2442,2443	2015	sn.1008-6706.2015.16.013
4322	血培养阳性病原菌构成及耐	drug resistance of pathoge	,卢赞,任宝军,王霞,尹丽民	,Ren Baojun,Wang Xia,Yin	国际检验医学杂志	onal journal of laboratory	-	6	740-742	2015	sn.1673-4130.2015.06.009
4323	细菌学指标和重金属、兽药	, heavy metals and veterin	魏玉明,钱振波,齐明,魏炳成	,Qian Zhenbo,Qi Ming,Wei	畜牧兽医杂志	mal Husbandry and Veteri	34	6	13-16	2015	sn.1004-6704.2015.06.004
4324	腹泻病人中食源性致病菌检	e pathogenic bacteria amo	沈玄艺,杨元斌,闫鹏,章丹阳	ang Yuanbin,Yan Peng,Zhar	中国农村卫生事业管理	na's rural health managem	35	4	469-473	2015	-
4325	及牛肉制品沙门氏菌污染	n of Salmonella contamina	包常明.	Bao Changming.	学园	Academy	-	33	136-137	2015	sn.1674-4810.2015.33.064
4326	猪和家禽屠宰环节沙门菌	ypes in pig and poultry slau	王君玮,盖文燕,颜世敢,黄	g Junwei,Gai Wenyan,Yan S	中国食品卫生杂志	hinese Food Hygiene Journ	27	5	503-508	2015	.13590/j.cjfh.2015.05.004
4327	一起沙门菌食物中毒调查	into a case of salmonella fo	林忠.	Lin Zhong.	海峡预防医学杂志	s Journal of Preventive Me	21	2	59-60	2015	-

4328	市腹泻患者沙门菌检测与	f Salmonella in patients wi	张建群,黄绍军,袁士杰.	ianqun,Huang Shaojun,Yua	中国预防医学杂志	se Journal of Preventive Me	16	9	720-722	2015	-
4329	源沙门菌毒力基因检测及	stance analysis of Salmone	,康元环,曹亮,单晓枫,王	huan,Cao Liang,Shan Xiaof	中国兽医杂志	Chinese Veterinary Journal	51	11	82-84	2015	sn.0529-6005.2015.11.029
4330	10年-2014年感染性腹泻病	surveillance analysis of pat	梅,钱海坤,林长缨,黄瑛,	Haikun,Lin Changying,Hu	中国卫生检验杂志	ese Journal of Health Inspe	25	22	3913-3917	2015	-
4331	县一起沙门氏菌食物中毒	e of salmonella food poiso	吴强,田维丽,谷永香.	Qiang,Tian Weili,Gu Yongxi	微量元素与健康研究	e elements and health rese	32	6	41	2015	-
4332	细菌性感染性腹泻病原学	esults of bacterial infectiou	程周祥,许亚冰,吴国富,陈	Zhouxiang,Xu Yabing,Wu G	安徽预防医学杂志	i Journal of Preventive Me	21	2	92-94	2015	-
4333	致病性沙门氏菌耐药性分	monella originating from d	瑞丽,李志芳,丁月霞,李	,Li Zhifang,Ding Yuexia,Li P	中国畜牧兽医	mal husbandry and veterina	42	8	2150-2159	2015	ki.1671-7236.2015.08.033
4334	宝鸡市食源性致病菌的污	of foodborne pathogenic b	萍,席桂绒,武永平,杨海	Guirong,Wu Yongping,Yan	江苏预防医学	iangsu Preventive Medicin	26	1	99-100	2015	sn.1006-9070.2015.01.043
4335	衢州地区感染性腹泻病原	is of pathogenic bacteria o	陆军,白永凤,余旭良,祝进	Bai Yongfeng,Yu Xuliang,Z	中国卫生检验杂志	ese Journal of Health Inspe	25	9	1416-1418,1422	2015	-
4336	殖场猪粪源沙门氏菌的耐	monella originating from pi	萍,夏利宁,苏战强,林亚	g,Xia Lining,Su Zhanqiang,L	中国农学通报	na Agricultural Science Bull	31	35	23-26	2015	-
4337	沙门菌多重耐药性与整合	g resistance and integrons	冒群,熊海平,张宏萍,张洁	group,Xiong Haiping,Zhang	中国卫生检验杂志	ese Journal of Health Inspe	25	6	892-894,898	2015	-
4338	市夏季肠道传染病腹泻病	cteria of intestinal infectio	贾妮娜.	Janina.	健康之路	road to health	-	1	353-353	2015	sn.1671-8801.2015.01.617
4339	镇江市食源性致病菌监测	sults of foodborne pathog	丽丽,刘丽萍,巢秀琴,徐	Lili,Liu Liping,Chao Xiuqin,X	中国卫生检验杂志	ese Journal of Health Inspe	25	13	2182-2183,2186	2015	-
4340	省腹泻人群志贺菌、沙门	onella serotypes among pe	李艳芬,许贝贝,杨芳,张秀	nfen,Xu Beibei,Yang Fang,Z	中国卫生检验杂志	ese Journal of Health Inspe	25	8	1259-1261	2015	-
4341	海市腹泻儿童沙门菌感染	of Salmonella infection in c	李桦,汪伟山,周玉球.	ua,Wang Weishan,Zhou Yu	国际检验医学杂志	onal journal of laboratory	-	18	2640-2642	2015	sn.1673-4130.2015.18.010
4342	源性致病菌监测与鉴定结	tification results of foodbo	罗兰秀,陶小春.	Luo Lanxiu,Tao Xiaochun.	中国保健营养	Chinese health nutrition	25	9	327-328	2015	-
4343	克苏地区肠道传染病病原	stinal infectious disease pa	娜尔克孜·阿布扎力汗,穆	n,Nargiz·Abu Zali Khan,Mu	疾病预防控制通报	se Prevention and Control	30	4	62-63	2015	15/j.cnki.jbyfkztb.1503005
4344	部队医院腹泻病原菌耐药	arrhea pathogenic bacteri	波,张培,李曦,洪文艳,刘	hang Pei,Li Xi,Hong Wenya	解放军预防医学杂志	tive Medicine of the People	33	6	644-645	2015	-
4345	2-2013年食源性致病菌监	ysis of annual foodborne pa	瑞,侯君,张磊,宋晓昀,陈玉	n,Zhang Lei,Song Xiaoyun,	实用预防医学	ractical preventive medicin	22	3	340-342	2015	sn.1006-3110.2015.03.028
4346	林市市售食品受食源性致	borne pathogenic bacteria	,贺漓漓,李红梅,王彦文,	He Lili,Li Hongmei,Wang Y	右江医学	Youjiang Medicine	43	2	225-230	2015	sn.1003-1383.2015.02.023
4347	镜蛇腐烂性皮肤病的病原	of the pathogen of putrid s	接瑜,廖黎黎,冯世文,曾	u,Liao Lili,Feng Shiwen,Zen	动物医学进展	vances in Veterinary Medic	-	4	121-123,124	2015	sn.1007-5038.2015.04.028
4348	州市食品中食源性致病菌	of foodborne pathogenic	邓志爱,吴继彬,张健,张晶	iai,Wu Jibin,Zhang Jian,Zha	华南预防医学	uth China Preventive Medic	41	2	192-194	2015	.13217/j.scjpm.2015.0192
4349	段腹泻儿童病原微生物检	sm detection results in chil	杨斐.	Yang Fei.	临床合理用药杂志	l of Rational Clinical Use of	-	6	101-102	2015	nki.13-1389/r.2015.06.065
4350	地区宠物重要人兽共患细	notic bacterial diseases in	,赵莎莎,刘静,陈文芳,蒋	asha,Liu Jing,Chen Wenfan	江苏农业科学	iangsu Agricultural Science	43	3	198-200	2015	sn.1002-1302.2015.03.064
4351	儿食品中食源性致病菌污	a contamination in infant a	,甄世祺,唐震,乔昕,秦思,	n Shiqi,Tang Zhen,Qiao Xin	现代预防医学	modern preventive medicin	42	9	1592-1595	2015	-
4352	感染败血症病原菌及感染	epticemia in children's hos	鹏程,金江兵,梁亚峰,卢	heng,Jin Jiangbing,Liang Ya	温州医科大学学报	al of Wenzhou Medical Univ	45	11	834-837	2015	sn.2095-9400.2015.11.014
4353	13年成都市食源性疾病	ive surveillance of foodbor	刘艳,何志凡,曾学民,冯敏	,He Zhifan,Zeng Xuemin,Fe	预防医学情报杂志	of Preventive Medicine Info	31	7	544-547	2015	-
4354	门氏菌耐药性及脉冲场凝	l electrophoresis typing of	维谊,沈莉萍,徐峰,齐新	iyi,Shen Liping,Xu Feng,Qi	中国动物检疫	China Animal Quarantine	-	2	78-81	2015	n.1005-944X.2015.02.027
4355	市食品及公共场所从业人	mination results of food an	马奎,费奉龙.	Magui,Fei Fenglong.	职业卫生与病伤	ccupational health and illne	30	5	272-275	2015	-
4356	食品6种食源性致病菌污	on contamination status of	,贺连华,石晓路,陈妙玲,	anhua,Shi Xiaolu,Chen Mia	热带医学杂志	journal of tropical medicine	15	7	989-991	2015	-
4357	波特沙门菌引起的食物中	e of food poisoning caused	罗冬香,林小红.	uo Dongxiang,Lin Xiaohong	海峡预防医学杂志	s Journal of Preventive Me	21	6	54-55	2015	-
4358	沙门氏菌食物中毒的确诊	report of salmonella food	王晓荫.	Wang Xiaoyin.	大家健康（中旬版）	one is healthy (mid-term e	-	8	272-273	2015	-
4359	城区感染性腹泻病例病原	d epidemic patterns of infe	李继珊,高波,霍哲,崔海洋	an,Gao Bo,Huo Zhe,Cui Ha	现代预防医学	modern preventive medicin	42	21	3995-3998	2015	-
4360	人粪便标本中沙门菌分离	Salmonella from fecal sam	方欣,李春.	Fang Xin,Li Chun.	中国人兽共患病学报	Chinese Journal of Zoonose	31	2	135-138	2015	sn.1002-2694.2015.02.009
4361	集中供水污染引起的伤寒	used by contamination o	富强;易子又;黄艳;唐光	i Ziyou;Huang Yan;Tang	实用预防医学	actical preventive medici	22	10	1227-1229+1219	2015	-
4362	市肉鸡生产链中沙门菌分	lar typing of Salmonella in	雪,白莉,李薇薇,闫军,王	Xue,Baili,Li Weiwei,Yan Ju	中华流行病学杂志	inese Journal of Epidemiol	37	12	1630-1638	2016	sn.0254-6450.2016.12.015
4363	儿非伤寒沙门菌菌型分布	bility analysis of non-typh	卫明,唐玲玲,王海燕,黄艳	ang Lingling,Wang Haiyan,	国际医药卫生导报	ational Medical and Health	22	20	3157-3161	2016	sn.1007-1245.2016.20.036
4364	4年食源性疾病主动监测的	iological characteristics of	玲,曾洪辉,杨彤,李柏生,梁	nghui,Yang Tong,Li Baishe	中华流行病学杂志	inese Journal of Epidemiol	37	10	1373-1378	2016	sn.0254-6450.2016.10.012

4365	境中沙门菌流行特征和分	lecular typing of Salmonel	,赵嘉咏,王泽乾,夏胜利,黄	ayong,Wang Zeqian,Xia Sh	中华流行病学杂志	inese Journal of Epidemiol	37	10	1404-1408	2016	sn.0254-6450.2016.10.017
4366	015年非伤寒沙门菌病原	illance of etiology and drug	姣,张白帆,夏胜利,苏佳,黄	ang Baifan,Xia Shengli,Su Ji	中华流行病学杂志	inese Journal of Epidemiol	37	9	1253-1256	2016	sn.0254-6450.2016.09.014
4367	养殖和生产加工环节中沙	Chicken Breeding, Product	杰,郑晓华,董锐,刘玉文,薛	ng Xiaohua,Dong Rui,Liu Yu	中华预防医学杂志	se Journal of Preventive Me	50	9	833-835	2016	sn.0253-9624.2016.09.018
4368	市肉鸡生产链中沙门菌分	lar typing of Salmonella in	雪,白莉,李薇薇,闫军,王开	Xue,Baili,Li Weiwei,Yan Jun	中华流行病学杂志	inese Journal of Epidemiol	37	12	1630-1638	2016	sn.0254-6450.2016.12.015
4369	儿非伤寒沙门菌菌型分布	bility analysis of non-typho	卫明,唐玲玲,王海燕,黄艳	ang Lingling,Wang Haiyan,	国际医药卫生导报	ational Medical and Health	22	20	3157-3161	2016	sn.1007-1245.2016.20.036
4370	年食源性疾病主动监测的	iological characteristics of	玲,曾洪辉,杨彤,李柏生,梁	nghui,Yang Tong,Li Baishen	中华流行病学杂志	inese Journal of Epidemiol	37	10	1373-1378	2016	sn.0254-6450.2016.10.012
4371	养殖和生产加工环节中沙	Chicken Breeding, Product	杰,郑晓华,董锐,刘玉文,薛	ng Xiaohua,Dong Rui,Liu Yu	中华预防医学杂志	se Journal of Preventive Me	50	9	833-835	2016	sn.0253-9624.2016.09.018
4372	015年非伤寒沙门菌病原	illance of etiology and drug	姣,张白帆,夏胜利,苏佳,黄	ang Baifan,Xia Shengli,Su Ji	中华流行病学杂志	inese Journal of Epidemiol	37	9	1253-1256	2016	sn.0254-6450.2016.09.014
4373	境中沙门菌流行特征和分	lecular typing of Salmonel	,赵嘉咏,王泽乾,夏胜利,黄	ayong,Wang Zeqian,Xia Sh	中华流行病学杂志	inese Journal of Epidemiol	37	10	1404-1408	2016	sn.0254-6450.2016.10.017
4374	沙门菌肠道感染的临床流	drug resistance patterns of	秋,常海岭,李月芳,许学斌,	hanghailing,Li Yuefang,Xu X	中华儿科杂志	Chinese Journal of Pediatric	54	7	489-495	2016	sn.0578-1310.2016.07.003
4375	15年甲型副伤寒沙门菌耐	5Salmonella paratyphi A d	,穆玉姣,张白帆,夏胜利,黄	u Yujiao,Zhang Baifan,Xia	中华流行病学杂志	inese Journal of Epidemiol	37	5	714-717	2016	sn.0254-6450.2016.05.026
4376	年伤寒副伤寒沙门菌分子	olecular typing and drug re	强,穆玉姣,苏佳,夏胜利,黄	Mu Yujiao,Su Jia,Xia Shengl	中华流行病学杂志	inese Journal of Epidemiol	37	1	111-114	2016	sn.0254-6450.2016.01.024
4377	海产品中常见食源性致病	mmon foodborne pathoge	娟,沈飚,周秀锦,徐君辉,张	en Biao,Zhou Xiujin,Xu Jun	中国卫生检验杂志	ese Journal of Health Inspe	26	5	746-747,752	2016	-
4378	群细菌分离鉴定及药敏试	drug susceptibility test of	陈巨清,杨振燕.	Chen Juqing,Yang Zhenyan.	兽药市场指南	eterinary Drug Market Guid	0	8	28-29	2016	-
4379	重庆市流动早餐食源性致	dborne pathogenic bacteria	钦培,罗书全,向新志,王红	uo Shuquan,Xiang Xinzhi,W	实用预防医学	ractical preventive medicin	23	12	1523-1526	2016	sn.1006-3110.2016.12.033
4380	菌类群分布及抗菌、抗肿瘤	tibacterial and antitumor a	利琼,谢华蓉,邓祖军,金小	n,Xie Huarong,Deng Zujun,	广东药学院学报	Guangdong Pharmaceutica	32	6	683-689	2016	ki.1006-8783.2016092601
4381	沙门菌污染对居民健康影	f Salmonella contaminatio	利,宋筱瑜,薛成玉,郑晓华	ng Xiaoyu,Xue Chengyu,Zh	中国食品卫生杂志	hinese Food Hygiene Journ	0	5	671-676	2016	-
4382	性腹泻病例饮食相关因素	-related factors in children	桑向来.	Sang Xianglai.	中国初级卫生保健	primary health care in china	30	8	62-63	2016	.1001-568X.2016.08.0025
4383	-2013年食物中毒事件流行	iological analysis of annua	沈秀莲,王俊瑛,阮元,周勤	n,Wang Junying,Ruan Yuan	中国公共卫生	Chinese public health	32	4	535-537	2016	847/zgggws2016-32-04-32
4384	市动物性食品微生物状况	microbial status of animal f	陈祥.	Chen Zhan.	上海畜牧兽医通讯	nal Husbandry and Veterina	-	6	36-37	2016	-
4385	门氏菌血清型鉴定及其对	strains derived from yak m	英,周晏阳,罗薇,孙吉,杨晓	ou Yanyang,Luo Wei,Sun J	黑龙江畜牧兽医（下半月）	dry and Veterinary Medicin	-	6	108-110	2016	-
4386	生食蔬菜沙门氏菌污染状	ntamination status of raw	吕新,陈丽华,李玥仁.	u Xin,Chen Lihua,Li Yueren	福建农业学报	ournal of Fujian Agriculture	31	3	297-300	2016	sn.1008-0384.2016.03.017
4387	2年-2015年食源性疾病监	Analysis of annual foodborn	张静,刘军,张秉慧.	ang Jing,Liu Jun,Zhang Bingl	中国卫生检验杂志	ese Journal of Health Inspe	26	22	3290-3292,3295	2016	-
4388	从业人员肛拭标本及食品	ure media for the detectio	徐妍,马智龙,杨娟,蔡震.	n,Ma Zhilong,Yang Juan,Cai	中国卫生产业	China health industry	13	2	95-97	2016	ki.1672-5654.2016.02.095
4389	南陆港口岸蝇类及其携带	and the pathogens they ca	,王丽,王晓梦,张芳,王凯,	Wang Xiaomeng,Zhang Fan	中国国境卫生检疫杂志	ntier Health and Quarantin	39	3	207-209,212	2016	/j.1004-9770.2016.03.016
4390	013年大连市食源性致病	nce of foodborne pathogen	冰,徐丹,陈玉凤,吕秋月,于	u Dan,Chen Yufeng,Lu Qiu	医学动物防制	medical animal control	32	4	427-429,432	2016	0.7629/yxdwfz201604025
4391	沙门氏菌对抗生素及消毒	ella to antibiotics and disi	国艳,龙梅,邓雯文,王红宁	,Long Mei,Deng Wenwen,	遗传	genetics	38	10	948-956	2016	10.16288/j.yczz.16-185
4392	鸡源沙门氏菌血清及脉冲	phoresis typing of Salmone	珍,罗薇,孔雪英,葛润洲,	uo Wei,Kong Xueying,Ge R	中国畜牧兽医	mal husbandry and veterina	43	11	2873-2879	2016	ki.1671-7236.2016.11.010
4393	锡市感染性腹泻监测分析	arly warning effect evaluat	石平,钱燕华,施超.	i Ping,Qian Yanhua,Shi Cha	职业与健康	Occupation and health	32	5	641-644	2016	-
4394	绵阳市食源性致病菌监测	sults of foodborne pathog	王乐,姚楠,陈果,周良君.	,Yao Nan,Chen Guo,Zhou L	医学动物防制	medical animal control	32	2	234-236	2016	0.7629/yxdwfz201602038
4395	殖过程中沙门氏菌病的防	on and treatment of salmo	杨万郊.	Yang Wanjiao.	甘肃畜牧兽医	al Husbandry and Veterina	46	2	54-55	2016	n.1006-799X.2016.02.032
4396	尿液中分离出伦敦沙门菌	olated from urine of pregn	周杰,陈开森,徐康立,张娟,	,Chen Kaisen,Xu Kangli,Zha	临床检验杂志	journal of clinical testing	34	2	160	2016	602/j.cnki.jcls.2016.02.22
4397	菌感染后对微生物检验结果	rial infection of blood spec	冉瑞芳.	Ran Ruifang.	西医结合心血管病电子杂	Diseases Integrated Traditi	4	22	150	2016	sn.2095-6681.2016.22.121
4398	门氏菌耐药表型与耐药基	otypes and drug-resistant	敏,莫国东,刘书宏,言天久,	uodong,Liu Shuhong,Yan T	食品科学	food science	37	19	166-170	2016	pkx1002-6630-201619028
4399	动早餐中食源性致病菌污染	odborne pathogenic bacte	王燕梅,唐震,乔昕,倪云龙	mei,Tang Zhen,Qiao Xin,N	中国卫生检验杂志	ese Journal of Health Inspe	26	3	432-433,436	2016	-
4400	肠炎沙门菌食物中毒的流	vestigation and analysis o	缪国忠.	Miao Guozhong.	疾病监测与控制	ease surveillance and cont	10	5	348-349	2016	-
4401	市市售食品中食源性致病	dborne pathogenic bacteri	,刘于飞,梁伯衡,李迎月,刘	fei,Liang Boheng,Li Yingyu	医学动物防制	medical animal control	32	11	1190-1192	2016	0.7629/yxdwfz201611004

4402	染性腹泻病人病原菌检测	teria detection results in pa	张学昌,吴旭东,刘宗东.	uechang,Wu Xudong,Liu Zo	中国保健营养	Chinese health nutrition	26	11	56-56	2016	-
4403	卖配送餐中细菌性污染情	alysis of bacterial contami	杨庆文,杨萍,杨祖顺,许燕	gwen,Yang Ping,Yang Zushu	中国卫生检验杂志	ese Journal of Health Inspe	26	17	2536-2539	2016	-
4404	养法检测从业人员沙门菌	onella and Shigella among	莉,张鲍虎,徐康立,黄伟忠	ng Baohu,Xu Kangli,Huang	中国卫生检验杂志	ese Journal of Health Inspe	26	23	3377-3378,3381	2016	-
4405	校、家庭、酒店冰箱内表	contamination of inner su	,刘鹰航,郑南才,李艳清,许	Liu Yinghang,Zheng Nanca	社区医学杂志	urnal of Community Medici	14	19	13-16	2016	-
4406	门氏菌食物中毒事件的流	lity of a cross-regional Saln	超,薛一凡,王丽丽,吴阳博	o,Xue Yifan,Wang Lili,Wu Y	现代预防医学	modern preventive medicin	43	19	3479-3482	2016	-
4407	沙门菌食物中毒病原菌检测	is of the Pathogen of Salmo	杨顺革.	Yang Shunge.	中国城乡企业卫生	ygiene in urban and rural ar	31	3	48-49	2016	/j.1003-5052.2016.03.021
4408	中沙门菌污染水平定量	f Salmonella contamination	,胡豫杰,马柯,汪兆国,李庆	Hu Yujie,Ma Ke,Wang Zhao	中国食品卫生杂志	hinese Food Hygiene Journ	28	6	714-719	2016	).13590/j.cjfh.2016.06.006
4409	寒沙门菌的监测及其脉冲	yphi in Dalian and its pulse	肖冰,王晓炜,吕秋月,王晓	g,Wang Xiaowei,Lu Qiuyue,	实用预防医学	ractical preventive medicin	23	6	743-745	2016	sn.1006-3110.2016.06.033
4410	门菌引起的食源性疾病调查	ysis of a foodborne illness	建全,李艳红,姚云波,徐艳	nquan,Li Yanhong,Yao Yun	中国卫生检验杂志	ese Journal of Health Inspe	26	7	1046-1047,1056	2016	-
4411	宴引起的沙门氏菌食源性	llness outbreak caused by a	坤,许建明,白赟,李旭东,	u Jianming,Bai Yun,Li Xudo	现代预防医学	modern preventive medicin	43	13	2462-2465	2016	-
4412	吉林市非发酵豆制品检测结	ults of non-fermented soyb	成,李怀伟,张歌,李生福,	Huaiwei,Zhang Ge,Li Shen	中国城乡企业卫生	ygiene in urban and rural ar	31	7	166-168	2016	/j.1003-5052.2016.07.077
4413	菌性腹泻病原微生物及药敏	s and drug susceptibility t	梁洁红.	Liang Jiehong.	中国医学工程	Chinese Medical Engineerin	24	7	123-124	2016	sn.1672-2019.2016.07.057
4414	牛肉与牛肉制品沙门氏菌血	serotypes in beef and bee	包常明.	Bao Changming.	食品界	food industry	-	4	70-71	2016	-
4415	沙门氏菌污染引起的食物中	ood poisoning caused by S	傅荣生,陈建中.	Rongsheng,Chen Jianzhon	今日健康	health today	15	6	5,9	2016	sn.1671-5160.2016.06.004
4416	物中毒和同期散发腹泻的	ood poisoning caused by S	吴守芝,王增国,袁军,魏晓	hi,Wang Zengguo,Yuan Jun	中国病原生物学杂志	ese Journal of Pathogen Bio	11	12	1122-1125	2016	10.13350/j.cjpb.161216
4417	产品中沙门氏菌血清型与	nce of Salmonella in livesto	葛秀清,柯艳坤,罗国强,黄	ing,Ke Yankun,Luo Guoqian	食品安全质量检测学报	f Food Safety and Quality In	7	6	2252-2257	2016	-
4418	江省哨点医院急性腹泻患	n patients with acute diarrh	伟,杨先知,谢国良,王忆	ng Xianxian,Xie Guoliang,W	中华预防医学杂志	e Journal of Preventive Me	50	12	1084-1090	2016	sn.0253-9624.2016.12.012
4419	口鱼粉中沙门菌监测与分	nalysis of Salmonella in Im	嫦娇,林素洁,白泉阳,郑鹏	gjiao,Lin Sujie,bai quanyang	中国兽医杂志	Chinese Veterinary Journal	52	3	95-97	2016	sn.0529-6005.2016.03.038
4420	2014年食品安全风险监测	alysis of annual food safety	晓辉,李成涛,许浩,朱国庆	hui,Li Chengtao,Xu Hao,Zhu	大家健康（上旬版）	ryone is healthy (early edit	10	4	21-21	2016	-
4421	门菌的耐药性分析及质粒	nella in Tibetan chickens ar	,蒋忠荣,冯卫东,徐志文,李	Zhongrong,Feng Weidong,	中国兽医科学	Chinese Veterinary Science	46	1	84-90	2016	sn.1673-4696.2016.01.015
4422	14年学生餐食源性致病菌	us of foodborne pathogeni	红,罗书全,向新志,陈京蓉	uo Shuquan,Xiang Xinzhi,Ch	中国学校卫生	Chinese school health	37	8	1273-1274	2016	ki.1000-9817.2016.08.051
4423	四川省食源性致病菌监测	s of foodborne pathogenic	文,张普,马梦婷,兰真,蒋恩	g Yu,Ma Mengting,Lan Zhe	预防医学情报杂志	of Preventive Medicine Info	32	12	1311-1314	2016	-
4424	畜产品中沙门氏菌污染状	lla contamination in livesto	丽,蔺露,姚璐,叶洁莹,梁望	ao Lu,Ye Jieying,Liang Wan	中国动物检疫	China Animal Quarantine	33	8	27-30	2016	n.1005-944X.2016.08.007
4425	猪肉中沙门氏菌的耐药性	drug resistance of Salmone	琪,李焱,唐文彦,耿亚娟,王	n,Tang Wenyan,Geng Yajua	中国动物检疫	China Animal Quarantine	33	10	23-25,30	2016	n.1005-944X.2016.10.006
4426	杆菌和沙门氏菌分离及药敏	ty test analysis of poultry E	王志国,王春明.	ang Zhiguo,Wang Chunmin	中国动物保健	China animal health	18	11	29	2016	sn.1008-4754.2016.11.014
4427	县沙门菌感染的流行病学	rug resistance analysis of Sa	杨伟才.	Yang Weicai.	中国疗养医学	hinese convalescent medici	25	12	1336-1339	2016	17/j.cnki.ccm.2016.12.046
4428	制中心微生物实验室2015	ntrol and Prevention, Nanj	窦雪如.	Dou Xueru.	疾病监测与控制	sease surveillance and cont	10	10	830-832	2016	-
4429	地区31批保健食品中污染	d identification of contami	彦斌,高瑞霞,滕赟,岳佳,	in,Gao Ruixia,Teng Yun,Yue	食品安全质量检测学报	f Food Safety and Quality In	7	4	1483-1488	2016	-
4430	市金州新区食源性致病菌	oodborne pathogenic bact	王忠智.	Wang Zhongzhi.	基层医学论坛	Primary Medicine Forum	-	4	436-438	2016	-
4431	氏菌的PFGE分型及耐药	FGETyping and drug resist	杨毅,王超,巴特尔,吴亚英	Yi,Wang Chao,Battle,Wu Ya	医学动物防制	medical animal control	32	10	1134-1136	2016	0.7629/yxdwfz201610024
4432	年淮安市食品中致病菌检	ion results of pathogenic b	兵兵,刘靓,刘纯成,邢亚	,Liu Liang,Liu Chuncheng,X	公共卫生与预防医学	Health and Preventive Me	27	4	23-25	2016	-
4433	自市仔猪腹泻致病性细菌	athogens of piglet diarrhea	韦,李芳,张英,张艳,高宏清	hang Ying,Zhang Yan,Gao H	上海畜牧兽医通讯	al Husbandry and Veterina	-	2	25-27	2016	sn.1000-7725.2016.02.009
4434	致病菌污染饮用水引起的	nteric pathogenic bacteria	甘映群.	Gan Yingqun.	保健文汇	Health Wenhui	-	4	189	2016	sn.1671-5217.2016.04.172
4435	家庭自办宴席引起的食物	isoning caused by a banque	杨树博,王力,马源,刘武.	Shubo,Wang Li,Ma Yuan,Li	医学动物防制	medical animal control	32	7	799-800	2016	0.7629/yxdwfz201607031
4436	沙门菌耐药谱及耐药基因	spectrum and drug resistan	宵雪,霍哲,安希龙,白书娥	ue,Huo Zhe,An Xilong,Bai Sh	中国卫生检验杂志	ese Journal of Health Inspe	26	1	136-138	2016	-
4437	省哨点医院食源性疾病病	f foodborne disease cases i	郭学斌.	Guo Xuebin.	现代预防医学	modern preventive medicin	43	22	4192-4195	2016	-
4438	食品安全风险监测微生物	ual food safety risk monito	杨艳艳.	Yang Yanyan.	临床研究	clinical research	-	1	121-121	2016	-

4439	源性沙门菌的多位点序列	sequence typing of aquatic	玲丽,周向阳,周宇芳,邵宏	u Xiangyang,Zhou Yufang,S	中国卫生检验杂志	ese Journal of Health Inspe	26	2	159-162	2016	-
4440	儿童病原学检测及药敏试	n and drug susceptibility te	王文雅,张秀敏,杜会双.	Venya,Zhang Xiumin,Du Hu	医学信息文摘（连续型电	information abstract (cont	16	8	23-23,25	2016	sn.1671-3141.2016.08.011
4441	肠炎沙门菌食物中毒的调	monella Enteritidis food po	谢雯,高海英.	Xie Wen,Gao Haiying.	医学信息	medical information	29	27	236-237	2016	sn.1006-1959.2016.27.194
4442	食品食源性致病菌监测结果	odborne pathogenic bacte	李跃中,徐水祥,张文元,刘放	,Xu Shuixiang,Zhang Weny	中国卫生检验杂志	ese Journal of Health Inspe	26	21	3049-3052	2016	-
4443	3年昌吉州食源性致病菌检	results of foodborne patho	朱建疆,祁玮,刘晓玉.	u Jianjiang,Qi Wei,Liu Xiao	中国卫生检验杂志	ese Journal of Health Inspe	26	6	797-799	2016	-
4444	2014年双流县食物中毒情	od poisoning situation in S	红宏,郭冬梅,刘昌玲,赵志	Guo Dongmei,Liu Changling	预防医学情报杂志	of Preventive Medicine Info	32	10	1112-1116	2016	-
4445	食源性疾病哨点医院监测	ts of Zhangjiagang Foodbo	柳丽江,朱芳,樊飞,钱惠芬	ang,Zhu Fang,Fan Fei,Qian	中国卫生检验杂志	ese Journal of Health Inspe	26	5	733-735	2016	-
4446	菌分离鉴定及药敏试验案	tion and drug susceptibility	陈巨清,杨振燕.	Chen Juqing,Yang Zhenyan.	今日畜牧兽医	ivestock Veterinarian Toda	-	10	35-36	2016	-
4447	门菌的血清学分型与耐药	sistance analysis of Salmon	张林吉,任士飞,吴艳涛.	ng Linji,Ren Shifei,Wu Yant	中国家禽	Chinese poultry	38	7	61-63	2016	sn.1004-6364.2016.07.016
4448	鼠伤寒沙门菌暴发流行的	is of an outbreak of Salmon	,曲梅,张海艳,严寒秋,黄玥	ng Haiyan,severe cold autu	疾病监测	disease surveillance	31	2	171-173	2016	sn.1003-9961.2016.02.019
4449	2014年安康市食品风险监	of food risk monitoring in	刘斌,李湘平,屈娅荣,周慧萍	Xiangping,Qu Yarong,Zhou	食品与发酵科技	d and Fermentation Techno	52	4	72-74	2016	n.1674-506X.2016.04-017
4450	血流感染病原菌构成及耐	drug resistance of pathoge	红娟,杜艳,胡大春,钱净,尹	an,Du Yan,Hu Dachun,Qian	中国抗生素杂志	hinese Journal of Antibiotic	41	2	137-143	2016	sn.1001-8689.2016.02.011
4451	者血培养病原菌分布及耐药	tribution and drug resistan	,徐春泉,周翠,张雪青,余方	unquan,Zhou Cui,Zhang Xu	温州医科大学学报	al of Wenzhou Medical Univ	46	3	211-214	2016	sn.2095-9400.2016.03.014
4452	民散发性沙门菌感染因素	adic salmonella infection a	浩,袁国平,茅俭英,沈隽卿	Guoping,Mao Jianying,Shen	职业与健康	Occupation and health	32	5	652-654,658	2016	-
4453	急性腹泻患者志贺菌、沙门	Salmonella in patients wit	肖宇平.	Xiao Yuping.	中国社区医师	hinese community physicia	32	24	127-128	2016	ssn.1007-614x.2016.24.77
4454	性疾病的细菌分离鉴定及药	g susceptibility test of bact	盛贤均,郭成华,贺永建.	ianjun,Guo Chenghua,He Y	甘肃畜牧兽医	al Husbandry and Veterina	46	17	83-87	2016	n.1006-799X.2016.17.047
4455	沙门菌污染对居民健康影	almonella contamination i	开利,宋筱瑜,薛成玉,郑晓华	ong Xiaoyu,Xue Chengyu,Zh	中国食品卫生杂志	hinese Food Hygiene Journ	28	5	671-676	2016	0.13590/j.cjfh.2016.05.025
4456	细菌性腹泻的病原微生物	genic microorganism test r	芦珂,秋洁.	Lu Ke,Qiu Jie.	中国医药指南	Chinese Medicine Guide	14	27	155-155	2016	-
4457	2010-2014年食源性致病菌	4Analysis of annual foodbo	曹展,姚盛英,霍哲.	Zhan,Yao Shengying,Huo Z	中国预防医学杂志	se Journal of Preventive Me	17	10	786-788	2016	5/j.1009-6639.2016.10.015
4458	地区某猪场沙门氏菌的分离	ation of Salmonella from a	赛涛,郭骏啸,吴夏智,何鑫	Saitao,Guo Junxiao,Wu Xia	中国畜牧兽医文摘	mal Husbandry and Veterin	32	3	59-60	2016	-
4459	肠炎沙门氏菌耐药性分析及	of Salmonella Enteritidis in	赵建梅,李玉清,黄秀梅,王君	mei,Li Yuqing,Huang Xium	食品科学	food science	37	3	120-124	2016	pkx1002-6630-201603023
4460	节置换术后都柏林沙门菌感	fection after hip replaceme	凡炼炼,于倩,白凤芝.	day refining,Yu Qian,Bai Fe	中国实验诊断学	nese experimental diagnos	20	4	688-688	2016	-
4461	隆沙门菌的抗菌药物敏感	d virulence genes of clinica	邓娟,师伟,吴晓妹,张利娟,韩	Wei,Wu Xiaomei,Zhang Liju	中华微生物学和免疫学杂志	rnal of Microbiology and In	36	8	582-587	2016	sn.0254-5101.2016.08.005
4462	年余杭区腹泻病例监测结果	rhea case surveillance resu	张玲玲,李飞,王海英.	ng Lingling,Li Fei,Wang Haiy	中国农村卫生事业管理	na's rural health managem	36	1	65-67	2016	-
4463	东新区生鲜食品中食源性致	genic bacteria in fresh food	K,苏靖华,黄红,张勇琪,朱林	Su Jinghua,yellow red,Zhan	中国食品卫生杂志	hinese Food Hygiene Journ	28	6	791-795	2016	0.13590/j.cjfh.2016.06.023
4464	粉类淀粉制品微生物污染状	obial contamination status	魁,廖兴广,张丁,王永,王法	guang,Zhang Ding,Wang Yo	现代预防医学	modern preventive medicin	43	9	1578-1581	2016	-
4465	寒并发噬血细胞综合征1例	emophagocytic syndrome i	温智新,何颜霞,付丹.	Wen Zhixin,He Yanxia,Fu Da	临床儿科杂志	Journal of Clinical Pediatrics	34	10	737-739	2016	sn.1000-3606.2016.10.005
4466	门菌耐药特征及分子分型织	acteristics and molecular t	周旋,李明友,黄基伟,刘桂治	,Li Mingyou,Huang Jiwei,Liu	中国人兽共患病学报	Chinese Journal of Zoonose	32	6	553-557	2016	sn.1002-2694.2016.06.009
4467	医院(南院)儿童急性腹泻病	d)Surveillance and analysis	敏,乔荆,孙建萍,郑洁芳,江雪	ng,Sun Jianping,Zheng Jiefa	寄生虫病与感染性疾病	ic diseases and infectious d	14	2	81-83	2016	-
4468	性与人源沙门菌耐药性与血	d serotypes of food-borne	刘翔,张燕飞,郝琼,沈梅,田	Zhang Yanfei,Hao Qiong,Sh	中国抗生素杂志	hinese Journal of Antibiotic	41	9	707-709,717	2016	sn.1001-8689.2016.09.012
4469	氏菌引起的自助餐食物中毒	food poisoning at a buffet	冬颖,赵伟,宋杰,范尉尉,白	ing,Zhao Wei,Song Jie,Fan W	现代预防医学	modern preventive medicin	43	19	3609-3610,3619	2016	-
4470	无锡市锡山区食源性疾病	he disease surveillance resu	沈梅云,张晴.	Shen Meiyun,Zhang Qing.	中国校医	Chinese school doctor	30	10	756-757	2016	-
4471	贵阳市辣椒粉微生物污染状	contamination status of ch	俊,刘敏,马韵韵,周映佑,肖	Min,Ma Yunyun,Zhou Ying	食品研究与开发	od research and developme	37	7	198-199	2016	sn.1005-6521.2016.07.048
4472	航道水域致病性细菌的毒	notypes of pathogenic bact	管峰,陈吉刚,毛芝娟,杨季芳	,Chen Jigang,Mao Zhijuan,Y	应用海洋学学报	rnal of Applied Oceanograp	35	2	183-189	2016	N.2095-4972.2016.02.005
4473	感染性肠炎病原检测结果与	teritis pathogen detection	任立军,王晶,沈建飞.	n Lijun,Wang Jing,Shen Jian	中国病原生物学杂志	ese Journal of Pathogen Bio	11	3	262-264	2016	10.13350/j.cjpb.160315
4474	门氏菌分离、鉴别方法的	ation and identification met	王宇,刘洋,欧静堃,蔡军,李慧	,Liu Yang,Ou Jingkun,Cai Ju	食品安全质量检测学报	f Food Safety and Quality In	7	10	4163-4168	2016	-
4475	淮市伤寒沙门菌耐药及分子	nce and molecular typing o	春,王晓炜,李柏生,孙茂利,	un,Wang Xiaowei,Li Baishen	中国微生态学杂志	inese Journal of Microecolo	28	4	396-399	2016	381/j.cnki.cjm.201604006

4476	建省福州市食源性致病菌	odborne pathogenic bacte	廖冬冬,刘建,张昊.	Dongdong,Liu Jian,Zhang	中国食物与营养	Chinese food and nutrition	22	12	26-28	2016	sn.1006-9577.2016.12.006
4477	吉林省食源性疾病流行病	ological characteristics of f	,寇泊洋,张丽薇,吕乐,金	Kou Boyang,Zhang Liwei,Lu	中国食品卫生杂志	hinese Food Hygiene Journ	28	5	589-593	2016	.13590/j.cjfh.2016.05.006
4478	售食品致病菌污染状况调	bacteria contamination in c	王巍,王书祺.	Wang Wei,Wang Shuqi.	中国卫生工程学	Chinese sanitary engineerin	15	4	392-393	2016	-
4479	年昆明市凉拌菜检测结果	he test results of cold vege	乐晶晶,周建德.	Le Jingjing,Zhou Jiande.	疾病监测与控制	sease surveillance and cont	10	10	789-790	2016	-
4480	寒沙门氏引起食物中毒的	e of food poisoning caused	孙延芳,张云仙.	Sun Yanfang,Zhang Yunxian	中国卫生产业	China health industry	13	20	112-113	2016	ki.1672-5654.2016.20.112
4481	伤寒沙门菌分离鉴定及药	ptibility test of Salmonella	悦,刘梅,李建梅,姜逸,赵宝	Liu Mei,Li Jianmei,Jiang Yi,	中国兽医杂志	Chinese Veterinary Journal	52	8	99-100	2016	sn.0529-6005.2016.08.037
4482	两所高校细菌性痢疾暴	l dysentery outbreaks in tw	冯智,洪镭,周蕾,马会来,祖	g Lei,Zhou Lei,The horse w	中华卫生应急电子杂志	ealth Emergency Electronic	2	6	358-364	2016	sn.2095-9133.2016.06.005
4483	寒沙门氏菌的分离鉴定	sistance study of Salmonell	军朝,邸涛,邹宇靖,王彦	,Di Tao,Zou Yujing,Wang Y	龙江畜牧兽医（下半月）	dry and Veterinary Medicin	-	8	131-133	2016	-
4484	沙门菌耐药特点及鉴定方	stics and identification met	新,宋文琪,苏建荣,董方,	Wenqi,Su Jianrong,Dong Fa	首都医科大学学报	hal of Capital Medical Unive	37	3	364-369	2016	sn.1006-7795.2016.03.023
4485	源性患者粪便中致病菌检	genic bacteria in the feces	李顺姬.	Lee Soon Hee.	中国微生态学杂志	inese Journal of Microecolo	28	12	1389-1392	2016	381/j.cnki.cjm.201612007
4486	张掖市食源性致病菌监	ysis of foodborne pathogen	晓梅,王泽平,王敏,郭立	i,Wang Zeping,Wang Min,	外医学（医学地理分册）	Medicine (Medical Geograp	37	1	54-56	2016	sn.1001-8883.2016.01.012
4487	寒沙门氏菌的分离?鉴定?	rom free-range chickens?i	建,张丹俊,潘孝成,赵瑞宏,	Danjun,Pan Xiaocheng,Zh	安徽农业科学	Anhui Agricultural Sciences	44	27	110-113,211	2016	sn.0517-6611.2016.27.038
4488	年CHINET沙门菌属细菌	Surveillance of drug resist	东,李金,卓超,苏丹虹,张朝	idong,Li Jin,Excellent,suda	中国感染与化疗杂志	urnal of Infection and Che	16	3	294-301	2016	/j.1009-7708.2016.03.009
4489	门诊血流感染154例病原学	n in fever clinic154Analysi	李晓光,胥婕,姚贝.	Li Xiaoguang,Xu Jie,Yao Bei	中国感染与化疗杂志	urnal of Infection and Che	16	2	123-128	2016	/j.1009-7708.2016.02.003
4490	从业人员食源性致病菌带	enic bacteria carriage amo	王丽萍.	Wang Liping.	医药前沿	Medical Frontier	6	17	331-332	2016	-
4491	罗湖区腹泻患者沙门菌流	nd drug resistance of Salm	华,龙冬玲,卓菲,赵洁玲,沈	ongling,Zhuo Fei,Zhao Jieli	中国卫生检验杂志	ese Journal of Health Inspe	26	3	447-449	2016	-
4492	瓜蒌饮片的微生物污染	tus of Trichosanthes tricho	桢,林永强,林林,焦阳,刘	n Yongqiang,Lin Lin,Jiao Ya	中国药学杂志	inese Pharmaceutical Jour	51	22	1962-1966	2016	10.11669/cpj.2016.22.015
4493	民医院283株沙门菌培养	al283Analysis of culture re	宜,陈金玲,黎青梅,陈冬	hen Jinling,Li Qingmei,Che	检验医学与临床	boratory medicine and clini	13	17	2468-2471	2016	sn.1672-9455.2016.17.024
4494	华市食源性疾病主动监测	veillance results of foodbo	王鸽,申屠平平,朱珈慧.	igeon,Shentu Pingping,Zhu	中国卫生检验杂志	ese Journal of Health Inspe	26	1	113-114,131	2016	-
4495	人员携带的沙门氏菌耐药	ular typing of Salmonella c	,杨建国,潘志明,焦新安,	g Jianguo,Pan Zhiming,Jiao	现代预防医学	modern preventive medicin	43	17	3110-3115	2016	-
4496	市食源性致病菌污染状	odborne pathogenic bacte	袁珣,李铁墙,冯梅.	an Xun,Li Tieqiang,Feng M	中国卫生检验杂志	ese Journal of Health Inspe	26	13	1949-1950	2016	-
4497	州市肉及肉制品卫生监测	nitoring results of meat an	边瑞岩,刘风仙.	Bian Ruiyan,Liu Fengxian.	中国卫生检验杂志	ese Journal of Health Inspe	26	5	730-732	2016	-
4498	南省鼠伤寒沙门菌耐药与	d molecular typing of Salm	玉姣,谢志强,苏佳,张白	jiao,Xie Zhiqiang,Su Jia,Zha	中国人兽共患病学报	Chinese Journal of Zoonose	32	1	56-60	2016	sn.1002-2694.2016.01.012
4499	门菌和志贺菌的分布及	analysis of Salmonella and	孙佰秀,阮田田,田园园.	xiu,Ruan Tiantian,pastoral	中国小儿急救医学	ric Emergency Medicine in	23	9	639-640,644	2016	sn.1673-4912.2016.09.015
4500	要食源性致病微生物与	microorganisms and expo	孙建云,刘兴荣.	Sun Jianyun,Liu Xingrong.	中国公共卫生	Chinese public health	32	5	605-608	2016	847/zgggws2016-32-05-11
4501	新生儿院内感染病原体分	of neonatal nosocomial in	玲,曾子耘,徐萱,李迎丽,邱	,Zeng Ziyun,Xu Xuan,Li Yin	重庆医学	Chongqing Medicine	-	3	380-382,386	2016	sn.1671-8348.2016.03.029
4502	年伊宁市食物中毒实验室	Analysis of Food Poisonin	宏英,闻捷,张逸静,古军	ing,Wen Jie,Zhang Yijing,Gu	疾病预防控制通报	se Prevention and Control	31	5	73-74,86	2016	15/j.cnki.jbyfkztb.1605004
4503	2013-2015年食品污染物	-2015Analysis of annual fo	田,杨梅,陈网旋,李汉锋,李	erry,Chen Wangxuan,Li Ha	中国热带医学	Chinese tropical medicine	16	8	843-845	2016	cnki.46-1064/r.2016.08.27
4504	市龙华新区感染性腹泻病	of infectious diarrhea path	晓玲,蔡剑辉,何小媚,刘	ing,Cai Jianhui,He Xiaomei,	热带医学杂志	ournal of tropical medicine	16	11	1455-1457	2016	sn.1672-3619.2016.11.031
4505	牛乳房炎病原菌调查与敏	cow mastitis and sensitive	燕,张涛,彭彰,王潘,陈鲁	ang Tao,Peng Zhang,Wang	中国草食动物科学	Chinese herbivore science	36	4	46-48,49	2016	sn.2095-3887.2016.04.013
4506	缓冲蛋白胨水培养基筛选	mparison of buffered pept	韩镌竹,李欣南,宿艳芳.	Zhuzhu,Li Xinnan,Su Yanf	龙江畜牧兽医（上半月）	ndry and Veterinary Medic	-	5	276-278,288	2016	-
4507	152份淡水龙虾中致病菌	Analysis of distribution and	兵,刘纯成,邢亚东,刘靓,侯	uncheng,Xing Yadong,Liu	现代预防医学	modern preventive medicin	43	17	3134-3138	2016	-
4508	腹泻病原菌分布及耐药性	g resistance of pathogenic	豪,刘慧,刘丽莎,泰淑红,邵	u Hui,Lisa Liu,Tai Shuhong,	医学检验与临床	edical Laboratory and Clini	27	5	43-44	2016	sn.1673-5013.2016.05.014
4509	外籍沙门菌感染患者流	nella infection among Chin	汤泓,黄峥,张岭,徐云蕴,侯	g,Huang Zheng,Zhang Ling,	中国热带医学	Chinese tropical medicine	16	5	475-477,491	2016	cnki.46-1064/r.2016.05.18
4510	查病人血液中分离出伤寒	the blood of a patient with	扎西央宗.	Tashi Yangzong.	西藏科技	Tibet Technology	-	10	57	2016	sn.1004-3403.2016.10.020
4511	氏菌中磺胺类药物耐药基	istance genes in Salmonell	晓菲,牛鑫鑫,廖聪,孙颖,	ofei,Niu Xinxin,Liao Cong,S	家禽科学	poultry science	-	3	10-12	2016	sn.1673-1085.2016.03.004
4512	沙门菌感染血清型分布	drug resistance of Salmone	余新华.	Yu Xinhua.	中国现代药物应用	dern drug application in Ch	10	1	166-167	2016	i.cn11-5581/r.2016.01.131

4513	少门菌的分离鉴定与耐药性	resistance analysis of Salm	,周雯,李芙蓉,陆广富,朱国	Wen,Li Furong,Lu Guangfu,	中国家禽	Chinese poultry	38	5	60-63	2016	sn.1004-6364.2016.05.016
4514	食源性致病菌污染状况调	e pathogenic bacteria in se	宫春波,王朝霞,董峰光.	unbo,Wang Chaoxia,Dong F	中国食品卫生杂志	hinese Food Hygiene Journ	28	1	103-106	2016	).13590/j.cjfh.2016.01.023
4515	2013年食品中食源性致病	nual monitoring results of	,刘翔,郝琼,曹守勤,闫立君	ng,Hao Qiong,Cao Shouqin	医学动物防制	medical animal control	32	1	31-34	2016	0.7629/yxdwfz201601011
4516	年常熟市食源性疾病主动	e surveillance of foodborne	月,王俭,常伟冰,沈明珠,	ng Jian,Chang Weibing,She	公共卫生与预防医学	Health and Preventive Me	27	2	71-73	2016	-
4517	胶电泳技术在一起食物中	ld gel electrophoresis tech	于烽,刘艳,苏静,远雪梅,	,Liu Yan,Su Jing,Yuan Xuem	中国卫生检验杂志	ese Journal of Health Inspe	26	4	546-548	2016	-
4518	门菌引起食物中毒的病原	of food poisoning caused	张甜.	Zhang Tian.	中国城乡企业卫生	ygiene in urban and rural a	31	8	54-56	2016	/j.1003-5052.2016.08.022
4519	4年大连市金州新区食品	bial monitoring in Jinzhou N	宫晓君.	Gong Xiaojun.	预防医学论坛	Preventive Medicine Forum	22	2	封2,154	2016	-
4520	年深圳市腹泻疾病的病原	udy on the etiology of diar	韦美玲,李卫宁,李迎慧,张海	Li Weining,Li Yinghui,Zhan	现代检验医学杂志	l of Modern Laboratory Me	31	3	143-146,149	2016	sn.1671-7414.2016.03.040
4521	区腹泻患者中食源性病原	analysis of foodborne path	郑剑,叶硕,沈玄艺,杨元斌,	an,Ye Shuo,Shen Xuanyi,Ya	中国卫生检验杂志	ese Journal of Health Inspe	26	15	2235-2239	2016	-
4522	水环境中沙门菌分布特征	resistance of Salmonella in	启发,高红,章丹阳,罗学辉	ao Hong,Zhang Danyang,Lu	中国食品卫生杂志	hinese Food Hygiene Journ	28	6	709-713	2016	).13590/j.cjfh.2016.06.005
4523	肠炎沙门菌家庭食物中毒	ood poisoning case caused	张梅.	Zhang Mei.	大家健康（中旬版）	one is healthy (mid-term e	10	8	49-50	2016	-
4524	炎沙门菌食物中毒的实验	of a case of Salmonella Ent	玲,周文革,张志魁,魏美石	ou Wenge,Zhang Zhikui,W	海峡预防医学杂志	s Journal of Preventive Me	22	4	89-90	2016	-
4525	多种病毒和细菌感染状况	al infections in large-scale	明薇,程悦宁,王建科,赵航	eng Yuening,Wang Jianke,Z	特产研究	Specialty research	38	3	1-4	2016	720/j.cnki.tcyj.2016.03.001
4526	5年湖北省突发中毒事件	acteristics of sudden poison	卫婷婷,姚永祥,梅良英.	gting,Yao Yongxiang,Mei Li	公共卫生与预防医学	Health and Preventive Me	27	6	52-55	2016	-
4527	015年食源性致病菌监测	s of annual foodborne path	方英,钟艳,胡卓,刘育兰.	ing,Zhong Yan,Hu Zhuo,Liu	微量元素与健康研究	e elements and health rese	33	6	41-42	2016	-
4528	3年-2015年感染性腹泻病	nalysis of annual surveillan	光清,肖锦晖,陈清凉,茌静	ao Jinhui,Chen Qingliang,Ch	中国卫生检验杂志	ese Journal of Health Inspe	26	10	1458-1461	2016	-
4529	对喹诺酮类药物耐药性及	of resistance to quinolone	明远,古丽娜孜,王威,张亚	gyuan,Gulinazi,Wang Wei,Z	食品科学	food science	37	17	150-155	2016	pkx1002-6630-201617025
4530	合征合并血流感染143例	ith bloodstream infection1	梁欣,柳明波,李春玫.	ng Xin,Liu Mingbo,Li Chunn	中国感染与化疗杂志	urnal of Infection and Che	16	3	252-256	2016	/j.1009-7708.2016.03.003
4531	同餐次两批聚餐人群食物	ng two groups of people d	,许舒乐,杨荣兴,张强,张	,Yang Rongxing,Zhang Qia	中国热带医学	Chinese tropical medicine	16	2	137-140	2016	cnki.46-1064/r.2016.02.11
4532	2016年南京市食源性沙门	6Identification and analysi	超,程婷婷,江晓,叶艳华,张	Tingting,Jiang Xiao,Ye Yanh	饮食保健	Diet and health care	3	20	197-199	2016	sn.2095-8439.2016.20.239
4533	广州市即食食品微生物的	amination of ready-to-eat	志爱,胡玉山,陈佳璇,张	i,Hu Yushan,Chen Jiaxuan,Z	医学动物防制	medical animal control	32	10	1071-1073,1076	2016	0.7629/yxdwfz201610003
4534	婴幼儿分离株耐药基因及	enes of Salmonella typhim	进,彭子欣,张宏元,赵熙,	ng Zixin,Zhang Hongyuan,Z	中国食品卫生杂志	hinese Food Hygiene Journ	28	5	567-575	2016	).13590/j.cjfh.2016.05.002
4535	015年食品中食源性致病	annual survey results of foo	刘世芸,王圣庆,刘璞.	Shiyun,Wang Shengqing,Liu	安徽预防医学杂志	i Journal of Preventive Me	22	2	107-109	2016	-
4536	无锡市感染性腹泻的病原	iology of infectious diarrhe	管红霞,肖勇,沙丹.	uan Hongxia,Xiao Yong,sata	检验医学	laboratory medicine	31	11	953-958	2016	n.1673-8640.2016.011.006
4537	源性疾病主动监测结果分	of active surveillance of fo	谭云鹤.	Tan Yunhe.	内科	Internal Medicine	11	2	289-291	2016	ki.cn45-1347/r.2016.02.46
4538	生餐食源性致病菌污染状	tus of foodborne pathogen	姚雪婷,刘展华,李秀桂.	Xueting,Liu Zhanhua,Li Xiu	中国学校卫生	Chinese school health	37	11	1688-1689,1693	2016	ki.1000-9817.2016.11.028
4539	京市哨点医院监测食源性	of foodborne diseases mo	云,江晓,杜雪飞,叶艳华,王	g Xiao,Du Xuefei,Ye Yanhua	职业与健康	Occupation and health	32	21	2939-2942	2016	-
4540	14年食源性致病菌污染监	and analysis of foodborne p	,栾阳,彭雁,张晔,魏晓光,	Peng Yan,Zhang Ye,Wei Xi	中国卫生检验杂志	ese Journal of Health Inspe	26	4	573-575,581	2016	-
4541	产链中沙门菌耐药性分析	n of Salmonella in the broi	娟,黄秀梅,王君玮,杨瑞梅	g Xiumei,Wang Junwei,Yan	中国家禽	Chinese poultry	38	24	22-27	2016	sn.1004-6364.2016.24.005
4542	婴幼儿腹泻病原学检测结	ogical detection results of i	宪华,陈王征,崔桂平,王丹	hen Wangzheng,Cui Guipin	国际检验医学杂志	onal journal of laboratory	37	15	2186-2188	2016	sn.1673-4130.2016.15.057
4543	菌斯坦利沙门菌的分子	alysis of Salmonella Stanle	王丽丽,陈倩.	Wang Lili,Chen Qian.	中国食品卫生杂志	hinese Food Hygiene Journ	28	1	27-31	2016	).13590/j.cjfh.2016.01.006
4544	急性腹泻儿童的肠道病原	ce in children with acute d	洁皓,郭家胤,许学斌,王	ehao,Guo Jiayin,Xu Xuebin,	中华传染病杂志	se Journal of Infectious Dis	34	1	19-22	2016	sn.1000-6680.2016.01.005
4545	年贵州省伤寒和副伤寒流	cteristics of typhoid fever a	荷,姚光海,黄艳萍,韦小瑜	o Guanghai,Huang Yanpin	华南预防医学	uth China Preventive Medi	42	6	562-564	2016	).13217/j.scjpm.2016.0562
4546	控中心细菌性食物中毒检	g Testing at Primary Center	曾丽,孙秋香,高艳,吕郁豪	i,Sun Qiuxiang,Gao Yan,Lu	疾病监测与控制	ease surveillance and cont	10	11	916-917	2016	-
4547	市饼干、糕点微生物安全	ety risk monitoring results	胡津津,汪少敏.	Hu Jinjin,Wang Shaomin.	现代农业科技	odern agricultural technolo	-	20	269-271	2016	sn.1007-5739.2016.20.154
4548	食品中沙门菌的检测方法	ection methods for Salmon	孙玉祝,陈江楠,夏兆飞.	uzhu,Chen Jiangnan,Xia Zh	中国兽医杂志	Chinese Veterinary Journal	52	1	87-89	2016	sn.0529-6005.2016.01.030
4549	省甲型副伤寒沙门菌分子	g and drug resistance of Sa	帆,穆玉姣,苏佳,夏胜利,黄	Mu Yujiao,Su Jia,Xia Sheng	中国病原生物学杂志	ese Journal of Pathogen Bi	11	8	701-704	2016	10.13350/j.cjpb.160806

4550	食品中食源性致病菌污染	of foodborne pathogenic ba	,王东健,刘长勇,罗瑞峰,	gjian,Liu Changyong,Luo R	预防医学情报杂志	of Preventive Medicine Info	32	7	700-703	2016	-
4551	年江阴市伤寒副伤寒流行	characteristics of typhoid a	姚建香,马焰,钱程.	Jianxiang,Ma Yan,Qian Che	职业与健康	Occupation and health	32	20	2835-2838	2016	-
4552	品中金黄色葡萄球菌及沙	s aureus and Salmonella in	素燕,姜娴,王晓红,周珊慧	iang Xian,Wang Xiaohong,	中国卫生检验杂志	ese Journal of Health Inspe	26	5	665-667	2016	-
4553	道公共场所从业人员沙门	tion among employees in	枫,何必隆,陈夏明,武照燕	e Bilong,Chen Xiaming,Wu	今日健康	health today	15	3	377-377	2016	-
4554	兵团哨点医院食源性疾病	e results of foodborne dise	罗建忠,王建平.	uo Jianzhong,Wang Jianpin	预防医学情报杂志	of Preventive Medicine Info	32	11	1145-1148	2016	-
4555	5年廊坊市食物中毒报告	food poisoning reports in	李红,李瑞丽.	Li Hong,Li Ruili.	中国保健营养	Chinese health nutrition	26	3	367	2016	-
4556	腹泻的105例病原微生物检	ren105Analysis of pathoge	航,张晓丽,张吉生,王勇,王	iaoli,Zhang Jisheng,Wang Y	医学信息	medical information	29	8	197-198	2016	sn.1006-1959.2016.08.144
4557	年黔西南州食源性致病菌	f foodborne pathogenic ba	凯金祥,姚丽,潘梅.	Kai Jinxiang,Yao Li,Pan Mei	职业与健康	Occupation and health	32	6	764-766	2016	-
4558	蒙得维的亚沙门菌流行特	l characteristics of Salmone	,王爱敏,黄峥,石维敏,胡雪	Aimin,Huang Zheng,Shi W	疾病监测	disease surveillance	31	7	540-545	2016	sn.1003-9961.2016.07.004
4559	山县生乳致病菌污染状况分	status of raw milk pathoge	谢向长.	Xie Xiangchang.	医药前沿	Medical Frontier	6	1	32-33	2016	sn.2095-1752.2016.01.023
4560	、弱雏中沙门氏菌的分离	monella from Tibetan chick	琪,赵瑾,李霞辉,徐超,王志	Jin,Li Xiahui,Xu Chao,Wan	四川农业大学学报	of Sichuan Agricultural Un	34	3	354-358	2016	sn.1000-2650.2016.03.016
4561	从业人员食源性致病菌带菌	athogenic bacteria among f	雯,焦文志,高曼,宋晓敏,方	Venzhi,Goldman,Song Xiao	世界临床医学	world clinical medicine	10	3	7-8	2016	-
4562	门菌和阿贡纳沙门菌耐药	ping of Salmonella Delphi	姣,谢志强,潘静静,夏胜利	,Xie Zhiqiang,Pan Jingjing,X	中国病原生物学杂志	ese Journal of Pathogen Bio	11	6	517-521	2016	10.13350/j.cjpb.160609
4563	海市宝山区沙门菌监测分	nalysis and capability asses	平,金凯,茅俭英,彭真真,朱	ncaid,Mao Jianying,Peng Zh	疾病监测	disease surveillance	31	7	546-553	2016	sn.1003-9961.2016.07.005
4564	门菌的血清型分布及耐药	and drug resistance analys	陈东杰,胡辛兰,伍严安.	n Dongjie,Hu Xinlan,Wu Ya	创伤与急诊电子杂志	ma and emergency e-maga	4	1	25-27	2016	nki.11-9332/r.2016.01.007
4565	2014年市售水产品受食源	ntamination status of comm	华,赵洁玲,卓菲,龙冬玲,沈	eling,Zhuo Fei,Long Dongli	中国卫生检验杂志	ese Journal of Health Inspe	26	9	1310-1312	2016	-
4566	津某猪场沙门氏菌的分离鉴	ication of Salmonella from	晓伟,蒙晓雷,郭恋,王健春	,Meng Xiaolei,Guo Lian,Wa	养猪	pig raising	-	3	105-108	2016	sn.1002-1957.2016.03.034
4567	京市房山区肠道多病原监测	lts of multiple intestinal pa	波,王云霞,史文凤,黄少平,	unxia,Shi Wenfeng,Huang S	职业与健康	Occupation and health	32	17	2420-2422	2016	-
4568	鸡养殖和屠宰加工环节中	cken Breeding, Slaughtering	杰,薛成玉,谢平会,郑晓华,	e Chengyu,Xie Pinghui,Zhen	中国食品卫生杂志	hinese Food Hygiene Journ	28	4	485-488	2016	0.13590/j.cjfh.2016.04.016
4569	家宴沙门菌食物中毒的调查	sis of Salmonella food pois	李净,林建维,靳桂才.	Li Jing,Lin Jianwei,Jin Guicai	疾病监测与控制	sease surveillance and cont	10	8	650-651	2016	-
4570	沙门菌的分离及其生物学	characteristics of Salmonel	伟,贾雪波,肖芹,窦新红,邹	ia Xuebo,Xiao Qin,Dou Xin	中国家禽	Chinese poultry	38	6	53-55	2016	sn.1004-6364.2016.06.013
4571	市县餐饮食品微生物污染	test results of catering food	符天晓,陈婉娃,梁书敏.	nxiao,Chen Wanwa,Liang S	首都食品与医药	Capital Food and Medicine	-	2	18-19	2016	-
4572	年余姚市沙门菌菌型分布	distribution and drug susc	黄邵军,张建群,罗学辉.	Shaojun,Zhang Jianqun,Luo	中国卫生检验杂志	ese Journal of Health Inspe	26	21	3111-3113	2016	-
4573	海产品中常见致病菌的检	common pathogenic bacte	,李盛鹏,林奕华,高素虹,	engpeng,Lin Yihua,Gao Su	中国卫生检验杂志	ese Journal of Health Inspe	26	6	805-807	2016	-
4574	分省市外卖盒饭中微生物	on in take-out lunch boxes	燕,闫琳,杨舒然,胡静,李宁	Yan Lin,Yang Shuran,Hu Jin	卫生研究	health research	45	6	1010-1012	2016	-
4575	氨基糖苷类耐药表型及耐	phenotypes and resistanc	福星,刘莉,戴建华,蔡丙严	ng,Liu Li,Dai Jianhua,Cai Bi	中国家禽	Chinese poultry	38	17	56-58	2016	sn.1004-6364.2016.17.013
4576	氏菌的分离鉴定及流行血清	t pigeons in Shanghai and	伟芳,詹泽强,任行星,林嘉	ang,Zhan Zeqiang,any plane	中国畜牧兽医	mal husbandry and veterina	43	12	3322-3328	2016	ki.1671-7236.2016.12.035
4577	的韦太夫雷登沙门菌在全球	nonella Wetefreden origina	隽卿,李迎慧,张建民,周晓	qing,Li Yinghui,Zhang Jian	疾病监测	disease surveillance	31	1	73-81	2016	sn.1003-9961.2016.01.017
4578	门菌感染引发脓毒血症致	h caused by sepsis caused	明元,范云飞,苟放,徐朝霞	yuan,Fan Yunfei,Keep quiet	西南国防医药	west National Defense Me	26	11	1355-1356	2016	sn.1004-0188.2016.11.053
4579	要病原菌的分离鉴定与药	sceptibility Test of Importa	珂,李小慧,林静,洪莹莹,金	Xiaohui,Lin Jing,Hong Ying	天津农学院学报	l of Tianjin Agricultural Uni	23	4	5-8	2016	sn.1008-5394.2016.04.002
4580	制品中沙门菌的半定量风	dy of Salmonella in cooked	周少君,梁辉,黄芮.	u Shaojun,Liang Hui,Huang	食品科技	food technology	41	4	324-328	2016	-
4581	中普沙门菌耐药性与毒力基	ce and virulence genes of	晓霞,杨杰,吴晓妹,张利娟	,Yang Jie,Wu Xiaomei,Zhan	天津医药	Tianjin Medicine	44	11	1387-1390	2016	10.11958/20160630
4582	传染病医院血流感染细菌构	g resistance of bloodstrea	,王启明,李青峰,刘应芬,陈	g Qiming,Li Qingfeng,Liu Y	现代预防医学	modern preventive medicin	43	15	2806-2810	2016	-
4583	年随州市食品中致病菌污	genic bacteria contaminati	,李月,聂文武,王永光,陈平	Nie Wenwu,Wang Yonggua	中国卫生检验杂志	ese Journal of Health Inspe	26	14	2085-2086	2016	-
4584	口饲料中沙门氏菌的分离	tion and identification of S	汪霖.	Wang Lin.	科技与企业	Technology and Enterprise	-	7	251-251	2016	sn.1004-9207.2016.07.211
4585	金黄色葡萄球菌和沙门菌	sistance of Staphylococcus	湘晖,刘帅仁,萧福元,陈子	ui,Liu Shuairen,Xiao Fuyuan	实用预防医学	ractical preventive medicin	23	8	989-991	2016	sn.1006-3110.2016.08.030
4586	沙门菌喹诺酮耐药分子机	alysis of quinolone resistan	,于兆阳,冯宝立,郑兰紫,	haoyang,Feng Baoli,Zheng	中国卫生检验杂志	ese Journal of Health Inspe	26	7	1050-1053	2016	-

4587	肠炎患儿病原菌分析与预	h on pathogenic bacteria in	麦海亮,王红吉,杨露,王肖	g,Wang Hongji,Yang Lu,Wa	中华医院感染学杂志	ournal of Hospital Infectiou	26	1	197-199	2016	.11816/cn.ni.2016-152422
4588	症病原菌及耐药性10年的	of septicemia in children1	黄梦,李克诚,徐旭.	uang Meng,Li Kecheng,Xu X	中国生化药物杂志	ese Journal of Biochemical	-	2	165-168	2016	-
4589	居民主要食品食源性致病	ogenic bacteria in the main	开春,胡晓宁,甘连军,权玉	n,Hu Xiaoning,Gan Lianjun,	中国卫生检验杂志	ese Journal of Health Inspe	26	24	3593-3595	2016	-
4590	门菌食物中毒病原学检测	f the etiology of a case of S	,王睿,查涛,刘文,孙永,邓	Rui,Cha Tao,Liu Wen,Sun Y	中国卫生检验杂志	ese Journal of Health Inspe	26	6	872-874	2016	-
4591	日市食品中沙门氏菌污染	onella contamination in fo	磊,刘玉婷,郎小倩,翁庆	iu Yuting,Lang Xiaoqian,W	师范大学学报（自然科学	u Normal University (Natur	34	5	36-40	2016	sn.1004-5570.2016.05.006
4592	液标本中检出伤寒沙门	s detected in the patient's	宫晓君.	Gong Xiaojun.	中国冶金工业医学杂志	rnal of Metallurgical Indust	33	4	496	2016	-
4593	上海市宝山区腹泻病监测	sease surveillance results i	伦辉,沈隽卿,金凯,杨兴	i,Shen Junqing,Kincaid,Yan	实用预防医学	ractical preventive medicin	23	2	215-218	2016	sn.1006-3110.2016.02.029
4594	门菌血清型分布及耐药情	ribution and drug resistanc	书媛,胡三梅,庞晶晶,高	yuan,Hu Sanmei,Pang Jing	河北医药	Hebei Medicine	-	3	449-451	2016	sn.1002-7386.2016.03.044
4595	期死鸡胚沙门氏杆菌的分	onella from dead chicken	李遵瑞.	Li Zunrui.	中国畜禽种业	estock and poultry breeding	12	3	141-143	2016	sn.1673-4556.2016.03.116
4596	监测工作在基层哨点医院	e surveillance of foodborne	王晓岚.	Wang Xiaolan.	航空航天医学杂志	urnal of Aerospace Medici	27	3	387-388	2016	sn.2095-1434.2016.03.065
4597	急性感染性腹泻病原菌分	ance of pathogenic bacteri	姜哲,迟维维.	Jiang Zhe,Chi Weiwei.	临床医药文献电子杂志	edical literature electronic	3	44	8863-8863	2016	sn.2095-8242.2016.44.126
4598	沙门菌感染临床特点及耐	ug resistance patterns of Sa	王庭庭,徐飞,李玫,郭红梅	ingting,Xu Fei,Li Mei,Guo H	临床儿科杂志	Journal of Clinical Pediatric	34	4	246-248	2016	sn.1000-3606.2016.04.002
4599	沙门菌引起的食物中毒实	of a case of food poisoning	朋,王海欣,姜颍丽,郭影,杨	g Haixin,Jiang Yingli,Guo Yi	医药论坛杂志	Medical Forum Magazine	37	1	117-118	2016	-
4600	病原菌分布、药物敏感性	d drug resistance changes	樊红丽,张米,高丽.	an Hongli,Zhang Mi,Goryed	检验医学与临床	boratory medicine and clini	13	z2	247-250	2016	sn.1672-9455.2016.26.105
4601	鸡生产链中沙门菌的分离	tion of Salmonella in broile	思俊,王娟,赵建梅,李玉清	ang Juan,Zhao Jianmei,Li Yu	中国兽医杂志	Chinese Veterinary Journal	-	6	-	2016	-
4602	顶山市食品中食源性致病	lts of foodborne pathogeni	李艳艳,王雨,席慧霞,王国	yan,Wang Yu,Xi Huixia,Wa	现代预防医学	modern preventive medicin	43	4	620-622,648	2016	-
4603	广东省熟肉制品致病菌污	ontamination status of coo	,赖蔚苳,杨冰,王海燕,陈	iruan,Yang Bing,Wang Haiy	华南预防医学	uth China Preventive Medic	42	2	192-195	2016	.13217/j.scjpm.2016.0192
4604	房山区沙门菌血清学鉴定	ug resistance analysis of Sa	,史文凤,黄少平,阚震,刘	enfeng,Huang Shaoping,Kan	中国卫生检验杂志	ese Journal of Health Inspe	26	6	811-812	2016	-
4605	在从业人员伤寒痢疾快速	gy in rapid screening of typ	,魏丽娜,吴步东,冯贞玉,	Lina,Wu Butong,Feng Zhen	中国卫生产业	China health industry	13	2	104-106	2016	ki.1672-5654.2016.02.104
4606	沙门菌表型特征及PFGE分	cteristics of Salmonella in	欧新华,贾华云,苏良,刘晓	hua,Jia Huayun,Su Liang,Liu	实用预防医学	ractical preventive medicin	23	1	39-42	2016	sn.1006-3110.2016.01.010
4607	物中毒病原菌调查与预防	acterial food poisoning and	曾德兴,黄思思,陈应坚.	Dexing,Huang Sisi,Chen Yin	现代诊断与治疗	dern Diagnosis and Treatm	27	8	1518-1520	2016	-
4608	乳粉中致病菌污染状况检	bacteria contamination in	大进,杨舒然,胡静,李宁,	ang Shuran,Hu Jing,Li Ning,	中国公共卫生	Chinese public health	32	5	602-604	2016	847/zgggws2016-32-05-10
4609	生肉与生肉制品致病菌污	us of commercially availab	学燕,许姣,黄淑华,陈磊,	an,Xu Jiao,Huang Shuhua,C	河南预防医学杂志	n Journal of Preventive Me	27	12	955-958	2016	m.1006-8414.2016.12.024
4610	头市细菌性食物中毒与病	d poisoning and pathogeni	,江素红,林奕华,高素虹,	g Suhong,Lin Yihua,Gao Su	预防医学情报杂志	of Preventive Medicine Info	32	1	77-80	2016	-
4611	151株志贺菌分布特征与耐	distribution characteristics	高庆双,高春燕,张晓玲.	shuang,Gao Chunyan,Zhang	现代预防医学	modern preventive medicin	43	1	60-61,110	2016	-
4612	上的食品和水源性疾病回	food and waterborne disea	,佘燕燕,张玲娟,刘晓荣,	Yanyan,Zhang Lingjuan,Liu	职业与健康	Occupation and health	32	3	338-341	2016	-
4613	菌分离鉴定及药敏试验案	tion and drug susceptibility	陈巨清,杨振燕.	Chen Juqing,Yang Zhenyan.	兽医导刊	veterinary guide	-	13	65-66,67	2016	sn.1673-8586.2016.13.038
4614	海西地区学生营养早餐食	ms in nutritional breakfast	郭海艳.	Guo Haiyan.	中国校医	Chinese school doctor	30	7	506509	2016	-
4615	菌引起的食物中毒检测及	sed by Salmonella Newport	,何春荣,林英华,廖琳虹,	unrong,Lin Yinghua,Liao Lin	中国食品卫生杂志	hinese Food Hygiene Journ	28	2	172-174	2016	.13590/j.cjfh.2016.02.007
4616	鼠伤寒沙门氏菌的分离鉴	aracteristics of Salmonella	王传宝.	Wang Chuanbao.	农家科技（下旬刊）	cience and Technology (lat	-	3	202-203	2016	-
4617	年禹州市食源性致病菌的	sis of foodborne pathogen	王爱香,杨秀范,马晓静,王	xiang,Yang Xiufan,Ma Xiaoj	河南预防医学杂志	n Journal of Preventive Me	27	9	666-668	2016	m.1006-8414.2016.09.009
4618	道沙门菌双相亚利桑那亚	rrhea-causing Salmonella e	,吕冰,张新,严寒秋,钱海	hang Xin,severe cold autum	中国预防医学杂志	se Journal of Preventive Me	17	6	405-408	2016	/j.1009-6639.2016.06.002
4619	宁波市肠炎沙门菌分子分	istance characteristics of S	元斌,宋启发,闫鹏,高红,叶	,Song Qifa,Yan Peng,Gao H	疾病监测	disease surveillance	31	12	1007-1011	2016	-
4620	伤寒沙门氏菌食物中毒事	of Salmonella Typhimurium	梁少溢,林鑫,韦冬萍.	g Shaoyi,Lin Xin,Wei Dongp	医学动物防制	medical animal control	32	8	902-904,907	2016	0.7629/yxdwfz201608025
4621	水污染致感染性腹泻病原	y of infectious diarrhea cau	李海余,唐丽群,田祖建.	Haiyu,Tang Liqun,Tian Zujia	医学理论与实践	Medical theory and practice	29	10	1369-1370	2016	-
4622	区感染性腹泻细菌病原学	l etiology of infectious diar	李平,陈懿,徐景野.	Li Ping,Chen Yi,Xu Jingye.	中国卫生检验杂志	ese Journal of Health Inspe	26	3	397-399,402	2016	-
4623	寒沙门菌引起的食源性疾	dborne illness caused by Sa	和仙.	He Xian.	中国保健营养	Chinese health nutrition	26	33	339-340	2016	sn.1004-7484.2016.33.565

4624	由肠炎沙门菌引起的食物	oning caused by Salmonella	蒋奎英,徐敏,高日红.	g Kuiying,Xu Min,Gao Riho	预防医学论坛	Preventive Medicine Forum	22	4	318,封3	2016	-
4625	15年度101例食源性疾病监	r101Analysis of foodborne	周军华,况强华.	hou Junhua,Kuang Qianghu	实验与检验医学	imental and Laboratory Me	-	1	115-117	2016	sn.1674-1129.2016.01.045
4626	肉中沙门氏菌的污染及耐药	nd drug resistance of Salm	娟,吕世明,刘芸,刘玉梅,张	Shiming,Liu Yun,Liu Yumei,	黑龙江畜牧兽医（上半月）	ndry and Veterinary Medic	-	2	257-259	2016	-
4627	人源沙门氏菌的血清分布	and drug resistance analys	邵燕,钱玉春,胡中旺,张文艳	Yuchun,Hu Zhongwang,Zh	安徽预防医学杂志	i Journal of Preventive Med	22	5	291-293,329	2016	-
4628	泻120例病原微生物和药敏	pathogenic microorganism	刘宗生.	Liu Zongsheng.	医学信息	medical information	29	29	264-264	2016	sn.1006-1959.2016.29.211
4629	副伤寒甲临床特征和耐药	eristics and drug resistance	王辉,秦波,高源.	ang Hui,Play waves,Gao Yu	重庆医学	Chongqing Medicine	45	22	3083-3085	2016	sn.1671-8348.2016.22.021
4630	定病原体的食源性疾病病	dborne disease cases of sp	王黎荔,山若青,高四海,蔡	Mountains are green,Gao S	中国食品卫生杂志	hinese Food Hygiene Journ	28	3	378-381	2016	).13590/j.cjfh.2016.03.023
4631	市朝阳区食源性腹泻病例	on of foodborne diarrhea c	明捷,魏云芳,巴蕾,姜薇,林	Chiet,Wei Yunfang,Bale,Jian	职业与健康	Occupation and health	32	18	2517-2519	2016	-
4632	省哨点医院食源性疾病病	borne disease cases in sent	强,万青青,王晓雯,阮元,赵	Qingqing,Wang Xiaowen,Ru	职业与健康	Occupation and health	32	12	1674-1677	2016	-
4633	沙门菌β-内酰胺类抗生素耐	th diarrheaβ-Analysis of la	邵启民,赵士勇,吴亦栋,厉小	min,Zhao Shiyong,Wu Yido	浙江临床医学	Zhejiang Clinical Medicine	18	3	479-480	2016	-
4634	幼儿细菌性腹泻的病原学分	of bacterial diarrhea in infa	王惠姣,徐娇君,朱根林.	g Huijiao,Xu Jiaojun,Zhu Ge	浙江临床医学	Zhejiang Clinical Medicine	18	4	728-729	2016	-
4635	州地区沙门菌的耐药情况研	g resistance of Salmonella	慧芬,郑文力,胡立成,包其	fen,Zheng Wenli,Hu Licher	中国卫生检验杂志	ese Journal of Health Inspe	26	2	283-286	2016	-
4636	细菌感染检测及其生物学	n Fujian Province and deter	华,刘荣昌,万春和,施少华,	gchang,Wan Chunhe,Shi Sh	中国兽医杂志	Chinese Veterinary Journal	52	11	10-13	2016	-
4637	生食动物性水产品中食源	borne pathogenic bacteria i	宫春波,王朝霞,董峰光.	unbo,Wang Chaoxia,Dong F	实用预防医学	ractical preventive medicin	23	12	1440-1444	2016	sn.1006-3110.2016.12.009
4638	年重庆市渝北区早餐食品	enic Quality of Breakfast Fo	海英,龚志华,胡迪.	Haiying,Gong Zhihua,Woody	中国卫生检验杂志	ese Journal of Health Inspe	26	10	1502-1503,1508	2016	-
4639	小学食堂餐（饮）具消毒	d utensils in canteens of p	兵,吴健,高永文,亢玲玲,王聿	n,Gao Yongwen,Kang Lingli	牡丹江医学院学报	al of Mudanjiang Medical C	37	4	123-124	2016	-
4640	区哨点医院成人急性腹泻	s in adults with acute diarr	晶,刘岩红,张万菊,何静,朱	ng,Zhang Wanju,He Jing,Zh	中华医院感染学杂志	ournal of Hospital Infectiou	26	22	5100-5103	2016	.11816/cn.ni.2016-160548
4641	肠道感染疾病临床特征及	esistance analysis of comm	郑海淑,黄永存.	heng Haishu,Huang Yongcu	临床医药文献电子杂志	edical literature electronic	3	20	4018-4018,4019	2016	-
4642	泻病例沙门菌的血清型分	stribution and drug resista	晶,张欣强,侯水平,吴新伟	Zhang Xinqiang,Hou level,	实用预防医学	ractical preventive medicin	23	5	616-617,623	2016	sn.1006-3110.2016.05.035
4643	道门诊2376例患者的临床分	clinic2376Clinical analysis o	陆群.	Lu Qun.	饮食保健	Diet and health care	3	18	27-28	2016	-
4644	测海产品中副溶血性弧菌、	of Vibrio parahaemolyticus	沈飚,余辉,周秀锦,张静,召	afterglow,Zhou Xiujin,Zhan	中国食品卫生杂志	hinese Food Hygiene Journ	28	4	440-444	2016	).13590/j.cjfh.2016.04.007
4645	013年阳江市沙门菌监测结	Salmonella Surveillance Re	天才,黄焕宜,陈怡静,孙亚	i,Huang Huanyi,Chen Yijing	实用预防医学	ractical preventive medicin	23	3	372-373,379	2016	sn.1006-3110.2016.03.039
4646	年食品中食源性致病菌监	monitoring results of foodb	丽萍,吕广波,刘晓娟,黄亮	Lu Guangbo,Liu Xiaojuan,H	医学动物防制	medical animal control	32	6	680-682	2016	0.7629/yxdwfz201606030
4647	奉贤区感染性腹泻病原菌分	enic bacteria in infectious d	方文娟,唐群力.	Fang Wenjuan,Tang Qunli.	中国消毒学杂志	hinese Journal of Disinfecti	33	11	1090-1092	2016	sn.1001-7658.2016.11.020
4648	患者沙门菌血清型及其耐药	ypes and drug resistance in	白帆,赵嘉咏,谢志强,夏胜	n,Zhao Jiayong,Xie Zhiqian	中国卫生检验杂志	ese Journal of Health Inspe	26	9	1335-1338	2016	-
4649	感染性主动脉瘤1例	cted aortic aneurysm1exan	靳丽妍,朱光发.	Jin Liyan,Zhu Guangfa.	中国感染与化疗杂志	urnal of Infection and Chen	16	6	785-787	2016	3/j.1009-7708.2016.06.019
4650	海北州食源性致病菌监测结	sults of foodborne pathoge	刘萍,晏伟.	Liu Ping,Yan Wei.	青海医药杂志	Qinghai Medical Journal	46	3	53-55	2016	-
4651	血性弧菌和沙门氏菌主动	f Vibrio parahaemolyticus	皆添,宋景红,刘海涛,庄国良	ng Jinghong,Liu Haitao,Zhu	食品安全导刊	food safety guide	-	16	65-67	2016	-
4652	寒沙门菌聚集性病例的病	and traceability analysis o	廖玉姣,苏佳,黄学勇,夏胜利	jiao,Su Jia,Huang Xueyong,	中国人兽共患病学报	Chinese Journal of Zoonose	32	12	1122-1125	2016	.1002-2694.2016.012.015
4653	京市崇文区食品中食源性致	of foodborne pathogenic	张旭,常志荣,吴伟.	ng Xu,Chang Zhirong,Wu W	中国卫生检验杂志	ese Journal of Health Inspe	26	22	3300-3303	2016	-
4654	沙门氏菌耐药性分析及PFG	sistance of Salmonella stra	刘艳,苏静,马鑫,远雪梅,杨	,Su Jing,Ma Xin,Yuan Xuem	疾病预防控制通报	se Prevention and Control	31	6	9-12,49	2016	15/j.cnki.jbyfkztb.1605044
4655	要细菌性病原体食源性疾病	e diseases caused by majo	福,祝白春,金迪,李小成,王艳	Baichun,Jindi,Li Xiaocheng,	现代预防医学	modern preventive medicin	43	21	3880-3883,3895	2016	-
4656	年浙江省仙居腹泻病沙门菌	urveillance results of diarrh	陈华斌,吴灵芝,余央云,赵斐	Huabin,Wu Lingzhi,Yu Yang	中国卫生检验杂志	ese Journal of Health Inspe	26	13	1933-1936	2016	-
4657	院临床分离沙门菌耐药性与	ng of clinically isolated Saln	周厚德,刘洋,姜茗,刘道峰	ude,Liu Yang,Jiang Ming,Liu	中国消毒学杂志	hinese Journal of Disinfecti	33	12	1200-1202	2016	sn.1001-7658.2016.12.021
4658	市售奶粉微生物污染状况	ontamination of milk powd	谢爱蓉,洪程基,李毅,胡玉琴	ong,Hong Chengji,Li Yi,Hu	中国卫生检验杂志	ese Journal of Health Inspe	26	20	3013-3015	2016	-
4659	区2012-2014年食品微生物	012-2014Analysis of annua	蒋艺梅.	Jiang Yimei.	交通医学	traffic medicine	30	4	402-403	2016	-
4660	儿童感染鼠伤寒沙门氏菌临	es of children infected with	梁燕霞,李小南,伍绍东.	Yanxia,Li Xiaonan,Wu Shao	中国实用医药	Chinese Practical Medicine	11	17	44-45	2016	nki.11-5547/r.2016.17.022

4661	患者非伤寒沙门菌血清分	s of non-typhoid Salmonell	王鸣柳,林玫,廖和壮,黎冬梅	ngliu,Lin Mei,Liao Hezhuan	中国卫生检验杂志	ese Journal of Health Inspe	26	19	2861-2864	2016	-
4662	童粪便分离的459株沙门菌	Hospital459Distribution ch	雷旻,周高枫,张交生,彭宝	ou Gaofeng,Zhang Jiaoshen	中国感染控制杂志	ese Journal of Infection Co	15	11	817-820	2016	sn.1671-9638.2016.11.003
4663	区食品安全风险监测微生	nalysis of food safety risk r	柳勤,叶新,黄燕,张惠文.	,Ye Xin,Huang Yan,Zhang H	中国卫生检验杂志	ese Journal of Health Inspe	26	10	1415-1416,1419	2016	-
4664	生食肉类产品中食源性致	bacteria in raw meat and r	欧阳以恒.	Ouyang Yiheng.	今日健康	health today	15	2	397-397,398	2016	-
4665	服务沙门氏菌食物中毒的	is of salmonella food poiso	吉喜军.	Ji Xijun.	中国保健营养	Chinese health nutrition	26	28	378-379	2016	sn.1004-7484.2016.28.512
4666	禽畜肉中沙门菌和单核细	monocytogenes contamina	董溢峰,傅灵菲,张晨,沈惠平	eng,Fu Lingfei,Zhang Chen,	中国卫生检验杂志	ese Journal of Health Inspe	26	11	1588-1590,1593	2016	-
4667	农村聚餐引起的沙门菌暴	lmonella outbreak caused	周兴余,陈曦,袁伟,张丽杰	ngyu,Chen Xi,Yuan Wei,Zha	预防医学情报杂志	of Preventive Medicine Info	32	2	145-149	2016	-
4668	猪沙门菌的分离鉴定及其耐	ug resistance analysis of Sa	刘海燕,侯巍,王远微,杨发龙	Hou Wei,Wang Yuanwei,Ya	中国兽医杂志	Chinese Veterinary Journal	-	6	86-88	2016	-
4669	患者血流感染病原菌分布	thogenic bacteria in bloods	青峰,刘应芬,王启明,陈莲,朱	Yingfen,Wang Qiming,Che	中华传染病杂志	se Journal of Infectious Dis	34	10	609-612	2016	sn.1000-6680.2016.10.008
4670	菌所致食物中毒的病原学	ysis of two cases of food po	,陈前进,林英华,廖琳虹,何	n Qianjin,Lin Yinghua,Liao	中国食品卫生杂志	hinese Food Hygiene Journ	28	1	32-36	2016	).13590/j.cjfh.2016.01.007
4671	品从业人员沙门菌血清学	almonella serology andPFG	,王艳,侯健国,殷宏梅,张	,Hou Jianguo,Yin Hongmei	实用预防医学	ractical preventive medicin	23	9	1131-1133	2016	sn.1006-3110.2016.09.034
4672	圳市龙岗区感染性腹泻病	of infectious diarrhea path	应坚,金玉娟,刘渠,李静媚,	gjian,Jin Yujuan,Liu Qu,Li Ji	中国卫生检验杂志	ese Journal of Health Inspe	26	24	3584-3587	2016	-
4673	州市瓯海区食品中食源性致	of foodborne pathogenic b	笑南,章忠辉,缪蔚蔚,林笑	Zhang Zhonghui,Miao Weiw	中国卫生检验杂志	ese Journal of Health Inspe	26	20	2989-2991,2993	2016	-
4674	沙门菌聚集性病例的病原	analysis of a Salmonella St	宝林,谢志强,黄学勇,穆玉	Xie Zhiqiang,Huang Xueyo	中国人兽共患病学报	Chinese Journal of Zoonose	32	5	497-501	2016	sn.1002-2694.2016.05.016
4675	沙门氏菌属和志贺氏菌属	teric Salmonella and Shigel	冯贤贵.	Feng Xiangui.	医药前沿	Medical Frontier	6	21	369-370	2016	-
4676	某市五类食品食源性致病菌	f foodborne pathogenic ba	杨国然,茅乃玲,刘斌.	g Guoran,Mao Nailing,Liu	现代预防医学	modern preventive medicin	43	5	818-820	2016	-
4677	5株沙门菌耐药表型与分子	sistance phenotype and m	晓,刘菊梅,方艳平,姜长宏	Jumei,Fang Yanping,Jiang	现代预防医学	modern preventive medicin	43	4	696-699	2016	-
4678	门氏菌的分离鉴定与耐药	ug resistance analysis of Sa	碧君,胡兴义,张海,文明,和	un,Hu Xingyi,Zhang Hai,civ	贵州畜牧兽医	mal Husbandry and Veterin	40	6	1-4	2016	sn.1007-1474.2016.06.001
4679	州市沙门菌临床分离株血清	stance analysis of Salmonel	,杨小蓉,周汉洪,田巍威,李	Xiaorong,Zhou Hanhong,Tia	预防医学情报杂志	of Preventive Medicine Info	32	8	781-785	2016	-
4680	中非伤寒沙门菌血清型及	nce of non-typhoidal Salmo	卿,傅慧琴,赵冰,黄红,章	u Huiqin,Zhao Bing,yellow	职业与健康	Occupation and health	32	1	52-54,58	2016	-
4681	14年食源性疾病主动监测	of annual foodborne diseas	陈艳,朱芳仪.	Chen Yan,Zhu Fangyi.	中国卫生检验杂志	ese Journal of Health Inspe	26	7	1025-1026	2016	-
4682	寒沙门氏菌食物中毒的流	a case of food poisoning ca	付秀影,黄露.	Fu Xiuying,yellow dew.	首都公共卫生	capital public health	10	2	86-89	2016	-
4683	年北京市房山区食源性疾病	orne disease surveillance re	王云霞,阚震,史文凤.	g Yunxia,Kan Zhen,Shi Wen	中国卫生检验杂志	ese Journal of Health Inspe	26	10	1469-1470,1473	2016	-
4684	州市服务行业人员沙门菌	monella carriage among se	建辉,黄梦颖,杨劲松,陈爱	uang Mengying,Yang Jinson	中国卫生检验杂志	ese Journal of Health Inspe	26	14	2081-2084	2016	-
4685	伤寒沙门菌肠炎临床分析	nce monitoring of non-typh	蔡和花,李桦,王慧,杨莉莉	ehua,Li Hua,Wang Hui,Yan	四川生理科学杂志	n Journal of Physiological S	38	2	76-79	2016	-
4686	长春市食品中食源性致病	of foodborne pathogenic	静鸿,龚云伟,李月婷,武艳	ng,Gong Yunwei,Li Yueting	食品安全质量检测学报	f Food Safety and Quality I	7	1	27-32	2016	-
4687	院感染病房病原菌分布及耐	ion and drug resistance in	红梅,王和平,张交生,齐利峰	,Wang Heping,Zhang Jiaosh	国际检验医学杂志	onal journal of laboratory	37	19	2702-2704,2707	2016	sn.1673-4130.2016.19.017
4688	耐药表型与耐药基因、血	drug-resistant genes and s	施开创,李凤梅,王海清,张珍	ang,Li Fengmei,Wang Haiq	动物医学进展	vances in Veterinary Medic	37	3	25-32	2016	sn.1007-5038.2016.03.006
4689	规模猪场环境中主要致病	teria in the environment o	张维谊,夏炉明,徐峰,沈莉萍	Weiyi,Xia Luming,Xu Feng,Sh	畜禽业	vestock and poultry indust	-	10	54-55	2016	sn.1008-0414.2016.10.031
4690	环节沙门菌分离株毒力基因	e analysis of Salmonella is	,赵建梅,曲志娜,黄秀梅,	Jianmei,Qu Zhina,Huang Xi	畜牧与兽医	Husbandry and Veterinary	48	9	103-107	2016	-
4691	滨地区鸡源沙门菌耐药性	of Salmonella originating fr	鑫鑫,黄皓,艾显辉,王晓菲	,Huang Hao,Ai Xianhui,Wa	动物医学进展	vances in Veterinary Medic	37	3	131-133	2016	sn.1007-5038.2016.03.029
4692	成人急性腹泻病例病原谱	s of Pathogen Spectrum in	黄建芳,黄海滨,度尧,马瑨	anfang,Huang Haibin,Du Ya	中国医药导报	China Medical Herald	13	20	60-63	2016	-
4693	市食源性疾病病原微生物	of foodborne disease path	蔡彦秋,马智龙,董洪燕.	nqiu,Ma Zhilong,Dong Hon	现代预防医学	modern preventive medicin	43	17	3131-3133,3143	2016	-
4694	屠宰场三种食源性细菌的	n of three foodborne bacte	林,欧秀玲,蒲敬伟,李爱巧,	Xiuling,Pu Jingwei,Li Aiqiao	甘肃畜牧兽医	al Husbandry and Veterina	46	21	91-92	2016	n.1006-799X.2016.21.045
4695	安市感染性腹泻细菌病原	nce results of bacterial etio	张辉,王春娟,谢龙,魏晓光	ang Chunjuan,Xie Long,We	现代预防医学	modern preventive medicin	43	22	4200-4203,4207	2016	-
4696	武威市474例食源性疾病监	vince474Foodborne diseas	让山,黄爱霞,马艳,满世军	untain,Huang Aixia,Ma Yan	疾病预防控制通报	se Prevention and Control	31	1	82-83	2016	15/j.cnki.jbyfkztb.1507020
4697	015年白银市食源性疾病监	ce analysis of foodborne d	,马菊红,杨军鹏,张景辉,李	Juhong,Yang Junpeng,Zhan	疾病预防控制通报	se Prevention and Control	31	5	58-60,63	2016	15/j.cnki.jbyfkztb.1605035

4698	2~2014年食源性沙门菌监	odborne Salmonella survei	平宗,王小龙,罗水斌,李传	,Wang Xiaolong,Luo Shuibi	检验医学与临床	boratory medicine and clini	-	1	4-5	2016	sn.1672-9455.2016.01.002
4699	内酰胺类抗生素耐药相关	and identification of lactar	,王继彤,曾博江,王少华,王	ong,Zeng Bojiang,Wang Sh	北京农学院学报	al of Beijing Agricultural Co	31	4	71-75	2016	issn.1002-3186.2016.0415
4700	省余姚市主城区河水沙门菌	water in the main urban are	群,黄邵军,罗学辉,苗超,杨	Shaojun,Luo Xuehui,Miao	疾病监测	disease surveillance	31	12	1023-1027	2016	-
4701	外卖快餐盒饭卫生状况调	ic Conditions of Takeaway	孙启明,何翠华.	Sun Qiming,He Cuihua.	海南医学	Hainan Medicine	27	12	2033-2034,2035	2016	sn.1003-6350.2016.12.049
4702	夫登堡沙门菌耐药性及分	epidemiological character	新,吕冰,钱海坤,严寒秋,鄧	Bing,Qian Haikun,severe c	现代预防医学	modern preventive medicin	43	14	2497-2501	2016	-
4703	伤寒沙门氏菌食物中毒的	e of food poisoning caused	鑫,许风兰,梁宏章,谢海鑫	Fenglan,Liang Hongzhang,X	医学动物防制	medical animal control	32	5	571-572,575	2016	0.7629/yxdwfz201605036
4704	年宁夏食源性疾病监测结	odborne disease surveillanc	袁秀娟,马娟,刘翔.	an Xiujuan,Ma Juan,Liu Xia	宁夏医科大学学报	al of Ningxia Medical Unive	38	9	1082-1084	2016	sn1674-6309.2016.09.032
4705	感染性腹泻病原学及流行	y and epidemiological char	姚建香,束方燕,梁峰.	anxiang,Shu Fangyan,Liang	医学动物防制	medical animal control	32	3	325-327	2016	0.7629/yxdwfz201603029
4706	疆兵团食品中食源性致病	esults of foodborne pathog	王建平,罗建忠.	Vang Jianping,Luo Jianzhon	现代预防医学	modern preventive medicin	43	12	2167-2170	2016	-
4707	河南省肠炎沙门菌耐药与	nd molecular typing of Sal	强,潘静静,苏佳,穆玉姣,黄	n Jingjing,Su Jia,Mu Yujiao,	中华预防医学杂志	se Journal of Preventive Me	50	3	261-265	2016	sn.0253-9624.2016.03.014
4708	感染性腹泻沙门菌的血清	stance analysis of Salmone	健宁,吴佳音,李舒宁,黄建	g,Wu Jiayin,Li Shuning,Hua	现代预防医学	modern preventive medicin	43	13	2435-2438,2452	2016	-
4709	伤寒沙门菌肠毒素基因序	nce characteristics of Salm	凤,张舟听,王忠发,付松维,	houting,Wang Zhongfa,Fu	中国卫生检验杂志	ese Journal of Health Inspe	26	12	1757-1760	2016	-
4710	婴幼儿腹泻病原微生物监	f pathogenic microorganis	毕小朵,郭妍妍.	Bi Xiaoduo,Guo Yanyan.	中国病原生物学杂志	ese Journal of Pathogen Bio	11	12	1118-1121	2016	10.13350/j.cjpb.161215
4711	寒沙门菌分子分型及耐药	resistance characteristics o	喈,柏彩英,邓秋莲,袁彩虹,	iying,Deng Qiulian,Yuan Ca	国际检验医学杂志	onal journal of laboratory r	37	12	1601-1603	2016	sn.1673-4130.2016.12.004
4712	沙门菌引起的食物中毒的	s of food poisoning caused	黎剑华,夏伟,卓菲,杨贵清	ua,Xia Wei,Zhuo Fei,Yang G	当代医学	contemporary medicine	22	36	196-196	2016	sn.1009-4393.2016.36.134
4713	份食品中食源性致病菌监	nitoring results of foodbor	郭海艳,李春林.	Guo Haiyan,Li Chunlin.	青海医药杂志	Qinghai Medical Journal	46	7	71-72	2016	-
4714	辖区食品中细菌污染状况	rial contamination in food	慧,楼永良,王晓红,张素燕,	ngliang,Wang Xiaohong,Zh	中国卫生检验杂志	ese Journal of Health Inspe	26	15	2252-2253,2258	2016	-
4715	门菌的血清学分型与耐药	sistance analysis of Salmon	张林吉,任士飞,吴艳涛.	ng Linji,Ren Shifei,Wu Yant	中国家禽	Chinese poultry	38	7	61-63	2016	sn.1004-6364.2016.07.016
4716	鼠伤寒沙门菌暴发流行的	is of an outbreak of Salmon	,曲梅,张海艳,严寒秋,黄琪	ng Haiyan,severe cold autu	疾病监测	disease surveillance	31	2	171-173	2016	sn.1003-9961.2016.02.019
4717	2014年安康市食品风险监	of food risk monitoring in	斌,李湘平,屈娅荣,周慧萍	Xiangping,Qu Yarong,Zhou	食品与发酵科技	d and Fermentation Techno	52	4	72-74	2016	n.1674-506X.2016.04-017
4718	血流感染病原菌构成及耐	drug resistance of pathoge	红娟,杜艳,胡大春,钱净,尹	an,Du Yan,Hu Dachun,Qian	中国抗生素杂志	hinese Journal of Antibiotic	41	2	137-143	2016	sn.1001-8689.2016.02.011
4719	者血培养病原菌分布及耐	tribution and drug resistan	,徐春泉,周翠,张雪青,余灵	unquan,Zhou Cui,Zhang Xu	温州医科大学学报	al of Wenzhou Medical Univ	46	3	211-214	2016	sn.2095-9400.2016.03.014
4720	民散发性沙门菌感染因素	adic salmonella infection a	浩,袁国平,茅俭英,沈隽卿	Guoping,Mao Jianying,Shen	职业与健康	Occupation and health	32	5	652-654,658	2016	-
4721	急性腹泻患者志贺菌、沙门	Salmonella in patients wit	肖宇平.	Xiao Yuping.	中国社区医师	hinese community physicia	32	24	127-128	2016	ssn.1007-614x.2016.24.77
4722	性疾病的细菌分离鉴定及药	susceptibility test of bact	盛贤均,郭成华,贺永建.	ianjun,Guo Chenghua,He Y	甘肃畜牧兽医	al Husbandry and Veterina	46	17	83-87	2016	n.1006-799X.2016.17.047
4723	沙门菌污染对居民健康影	almonella contamination i	利,宋筱瑜,薛成玉,郑晓华	ng Xiaoyu,Xue Chengyu,Zh	中国食品卫生杂志	hinese Food Hygiene Journ	28	5	671-676	2016	).13590/j.cjfh.2016.05.025
4724	细菌性腹泻的病原微生物	genic microorganism test r	芦珂,秋洁.	Lu Ke,Qiu Jie.	中国医药指南	Chinese Medicine Guide	14	27	155-155	2016	-
4725	2010-2014年食源性致病菌	4Analysis of annual foodbo	曹展,姚盛英,霍哲.	Zhan,Yao Shengying,Huo Z	中国预防医学杂志	se Journal of Preventive Me	17	10	786-788	2016	5/j.1009-6639.2016.10.015
4726	地区某猪场沙门氏菌的分	ation of Salmonella from a	赛涛,郭骏啸,吴夏智,何鑫	Saitao,Guo Junxiao,Wu Xia	中国畜牧兽医文摘	mal Husbandry and Veterin	32	3	59-60	2016	-
4727	肠炎沙门氏菌耐药性分析及	f Salmonella Enteritidis in	赵建梅,李玉清,黄秀梅,王春	mei,Li Yuqing,Huang Xium	食品科学	food science	37	3	120-124	2016	pkx1002-6630-201603023
4728	置换术后都柏林沙门菌感	fection after hip replacem	凡炼炼,于倩,白凤芝.	day refining,Yu Qian,Bai Fe	中国实验诊断学	nese experimental diagnos	20	4	688-688	2016	-
4729	隆沙门菌的抗菌药物敏感	d virulence genes of clinica	娟,师伟,吴晓妹,张利娟,韩	Wei,Wu Xiaomei,Zhang Lij	中华微生物学和免疫学杂志	rnal of Microbiology and Im	36	8	582-587	2016	sn.0254-5101.2016.08.005
4730	年余杭区腹泻病例监测结果	rhea case surveillance resu	张玲玲,李飞,王海英.	ng Lingling,Li Fei,Wang Haiy	中国农村卫生事业管理	na's rural health managem	36	1	65-67	2016	-
4731	东新区生鲜食品中食源性致	genic bacteria in fresh food	,苏靖华,黄红,张勇琪,朱林	Su Jinghua,yellow red,Zhan	中国食品卫生杂志	hinese Food Hygiene Journ	28	6	791-795	2016	).13590/j.cjfh.2016.06.023
4732	粉类淀粉制品微生物污染	obial contamination status	魁,廖兴广,张丁,王永,王法	guang,Zhang Ding,Wang Yo	现代预防医学	modern preventive medicin	43	9	1578-1581	2016	-
4733	菌并发噬血细胞综合征1例	emophagocytic syndrome i	温智新,何颜霞,付丹.	Ven Zhixin,He Yanxia,Fu Da	临床儿科杂志	Journal of Clinical Pediatrics	34	10	737-739	2016	sn.1000-3606.2016.10.005
4734	门菌耐药特征及分子分型	acteristics and molecular t	旋,李明友,黄基伟,刘桂治	Li Mingyou,Huang Jiwei,Liu	中国人兽共患病学报	Chinese Journal of Zoonose	32	6	553-557	2016	sn.1002-2694.2016.06.009

4735	医院(南院)儿童急性腹泻病	d)Surveillance and analysis	刘,乔荆,孙建萍,郑洁芳,江雪	g,Sun Jianping,Zheng Jiefa	寄生虫病与感染性疾病	ic diseases and infectious d	14	2	81-83	2016	-
4736	性与人源沙门菌耐药性与血	d serotypes of food-borne	刘翔,张燕飞,郝琼,沈梅,田	Zhang Yanfei,Hao Qiong,Sh	中国抗生素杂志	hinese Journal of Antibiotic	41	9	707-709,717	2016	sn.1001-8689.2016.09.012
4737	氏菌引起的自助餐食物中毒	food poisoning at a buffet	冬颖,赵伟,宋杰,范尉尉,白	ing,Zhao Wei,Song Jie,Fan	现代预防医学	modern preventive medicin	43	19	3609-3610,3619	2016	-
4738	无锡市锡山区食源性疾病	he disease surveillance resu	沈梅云,张晴.	Shen Meiyun,Zhang Qing.	中国校医	Chinese school doctor	30	10	756-757	2016	-
4739	贵阳市辣椒粉微生物污染	contamination status of ch	俊,刘敏,马韵韵,周映佑,肖	Min,Ma Yunyun,Zhou Ying	食品研究与开发	od research and developme	37	7	198-199	2016	sn.1005-6521.2016.07.048
4740	航道水域致病性细菌的毒	otypes of pathogenic bact	管峰,陈吉刚,毛芝娟,杨季芳	Chen Jigang,Mao Zhijuan,Y	应用海洋学学报	rnal of Applied Oceanogra	35	2	183-189	2016	N.2095-4972.2016.02.005
4741	感染性肠炎病原检测结果与	teritis pathogen detection	任立军,王晶,沈建飞.	n Lijun,Wang Jing,Shen Jian	中国病原生物学杂志	ese Journal of Pathogen Bio	11	3	262-264	2016	10.13350/j.cjpb.160315
4742	门氏菌分离、鉴别方法的	tion and identification met	王宇,刘洋,欧静堃,蔡军,李慧	,Liu Yang,Ou Jingkun,Cai Ju	食品安全质量检测学报	f Food Safety and Quality I	7	10	4163-4168	2016	-
4743	连市伤寒沙门菌耐药及分子	nce and molecular typing o	春,王晓炜,李柏生,孙茂利,	n,Wang Xiaowei,Li Baishen	中国微生态学杂志	inese Journal of Microecolo	28	4	396-399	2016	381/j.cnki.cjm.201604006
4744	建省福州市食源性致病菌	oodborne pathogenic bacte	廖冬冬,刘建,张昊.	Dongdong,Liu Jian,Zhang	中国食物与营养	Chinese food and nutrition	22	12	26-28	2016	sn.1006-9577.2016.12.006
4745	吉林省食源性疾病流行病	ological characteristics of f	王,寇泊洋,张丽薇,吕乐,金英	Kou Boyang,Zhang Liwei,Lu	中国食品卫生杂志	hinese Food Hygiene Journ	28	5	589-593	2016	).13590/j.cjfh.2016.05.006
4746	售食品致病菌污染状况调	bacteria contamination in c	王巍,王书祺.	Wang Wei,Wang Shuqi.	中国卫生工程学	Chinese sanitary engineerin	15	4	392-393	2016	-
4747	年昆明市凉拌菜检测结果	he test results of cold vege	乐晶晶,周建德.	Le Jingjing,Zhou Jiande.	疾病监测与控制	sease surveillance and cont	10	10	789-790	2016	-
4748	寒沙门氏引起食物中毒的	e of food poisoning caused	孙延芳,张云仙.	Sun Yanfang,Zhang Yunxian	中国卫生产业	China health industry	13	20	112-113	2016	ki.1672-5654.2016.20.112
4749	伤寒沙门菌分离鉴定及药	ptibility test of Salmonella	悦,刘梅,李建梅,姜逸,赵宝	Liu Mei,Li Jianmei,Jiang Yi,Z	中国兽医杂志	Chinese Veterinary Journal	52	8	99-100	2016	sn.0529-6005.2016.08.037
4750	市两所高校细菌性痢疾暴发	l dysentery outbreaks in tw	冯智,洪镭,周蕾,马会来,祖	g Lei,Zhou Lei,The horse w	中华卫生应急电子杂志	ealth Emergency Electronic	2	6	358-364	2016	sn.2095-9133.2016.06.005
4751	伤寒沙门氏菌的分离鉴定及	sistance study of Salmonell	李军朝,邸涛,邹宇靖,王彦红	,Di Tao,Zou Yujing,Wang Y	黑龙江畜牧兽医（下半月）	dry and Veterinary Medicin	-	8	131-133	2016	-
4752	沙门菌耐药特点及鉴定方	stics and identification met	新,宋文琪,苏建荣,董方,王	Wenqi,Su Jianrong,Dong Fa	首都医科大学学报	nal of Capital Medical Unive	37	3	364-369	2016	sn.1006-7795.2016.03.023
4753	源性患者粪便中致病菌检测	genic bacteria in the feces	李顺姬.	Lee Soon Hee.	中国微生态学杂志	inese Journal of Microecolo	28	12	1389-1392	2016	381/j.cnki.cjm.201612007
4754	年张掖市食源性致病菌监测	ysis of foodborne pathogen	刘晓梅,王泽平,王敏,郭立娟	i,Wang Zeping,Wang Min,G	国外医学（医学地理分册）	Medicine (Medical Geograp	37	1	54-56	2016	sn.1001-8883.2016.01.012
4755	寒沙门氏菌的分离?鉴定?	rom free-range chickens?id	建,张丹俊,潘孝成,赵瑞宏,	Danjun,Pan Xiaocheng,Zha	安徽农业科学	Anhui Agricultural Sciences	44	27	110-113,211	2016	sn.0517-6611.2016.27.038
4756	年CHINET沙门菌属细菌耐	Surveillance of drug resist	东,李金,卓超,苏丹虹,张朝	idong,Li Jin,Excellent,suda	中国感染与化疗杂志	urnal of Infection and Che	16	3	294-301	2016	3/j.1009-7708.2016.03.009
4757	门诊血流感染154例病原学	n in fever clinic154Analysi	李晓光,胥婕,姚贝.	Li Xiaoguang,Xu Jie,Yao Bei	中国感染与化疗杂志	urnal of Infection and Che	16	2	123-128	2016	3/j.1009-7708.2016.02.003
4758	从业人员食源性致病菌带	enic bacteria carriage amo	王丽萍.	Wang Liping.	医药前沿	Medical Frontier	6	17	331-332	2016	-
4759	罗湖区腹泻患者沙门菌流行	nd drug resistance of Salm	华,龙冬玲,卓菲,赵洁玲,沈	ongling,Zhuo Fei,Zhao Jieli	中国卫生检验杂志	ese Journal of Health Inspe	26	3	447-449	2016	-
4760	区瓜蒌饮片的微生物污染状	tus of Trichosanthes tricho	丁桢,林永强,林林,焦阳,刘洪	n Yongqiang,Lin Lin,Jiao Ya	中国药学杂志	inese Pharmaceutical Jour	51	22	1962-1966	2016	10.11669/cpj.2016.22.015
4761	人民医院283株沙门菌培养	al283Analysis of culture re	焕宜,陈金玲,黎青梅,陈冬玲	hen Jinling,Li Qingmei,Che	检验医学与临床	boratory medicine and clini	13	17	2468-2471	2016	sn.1672-9455.2016.17.024
4762	华市食源性疾病主动监测	veillance results of foodbo	王鸽,申屠平平,朱珈慧.	igeon,Shentu Pingping,Zhu	中国卫生检验杂志	ese Journal of Health Inspe	26	1	113-114,131	2016	-
4763	人员携带的沙门氏菌耐药	ular typing of Salmonella ca	燕,杨建国,潘志明,焦新安,马	g Jianguo,Pan Zhiming,Jiao	现代预防医学	modern preventive medicin	43	17	3110-3115	2016	-
4764	安市食源性致病菌污染状况	oodborne pathogenic bacte	袁珣,李铁墙,冯梅.	an Xun,Li Tieqiang,Feng M	中国卫生检验杂志	ese Journal of Health Inspe	26	13	1949-1950	2016	-
4765	州市肉及肉制品卫生监测	nitoring results of meat an	边瑞岩,刘风仙.	Bian Ruiyan,Liu Fengxian.	中国卫生检验杂志	ese Journal of Health Inspe	26	5	730-732	2016	-
4766	河南省鼠伤寒沙门菌耐药与	d molecular typing of Salm	廖玉姣,谢志强,苏佳,张白帆	jiao,Xie Zhiqiang,Su Jia,Zha	中国人兽共患病学报	Chinese Journal of Zoonose	32	1	56-60	2016	sn.1002-2694.2016.01.012
4767	沙门菌和志贺菌的分布及耐	analysis of Salmonella and	孙佰秀,阮田田,田园园.	xiu,Ruan Tiantian,pastoral	中国小儿急救医学	tric Emergency Medicine in	23	9	639-640,644	2016	sn.1673-4912.2016.09.015
4768	主要食源性致病微生物与暴	microorganisms and expo	孙建云,刘兴荣.	Sun Jianyun,Liu Xingrong.	中国公共卫生	Chinese public health	32	5	605-608	2016	847/zgggws2016-32-05-11
4769	新生儿院内感染病原体分	of neonatal nosocomial in	玲,曾子耘,徐萱,李迎丽,邱	,Zeng Ziyun,Xu Xuan,Li Ying	重庆医学	Chongqing Medicine	-	3	380-382,386	2016	sn.1671-8348.2016.03.029
4770	5年伊宁市食物中毒实验室	Analysis of Food Poisonin	李宏英,闻捷,张逸静,古军梅	ing,Wen Jie,Zhang Yijing,Gu	疾病预防控制通报	se Prevention and Control	31	5	73-74,86	2016	15/j.cnki.jbyfkztb.1605004
4771	区2013-2015年食品污染物	-2015Analysis of annual fo	田,杨梅,陈网旋,李汉锋,李	erry,Chen Wangxuan,Li Ha	中国热带医学	Chinese tropical medicine	16	8	843-845	2016	cnki.46-1064/r.2016.08.27

4772	市龙华新区感染性腹泻病	of infectious diarrhea path	晓玲,蔡剑辉,何小媚,刘爱	ing,Cai Jianhui,He Xiaomei,	热带医学杂志	Journal of tropical medicine	16	11	1455-1457	2016	sn.1672-3619.2016.11.031
4773	牛乳房炎病原菌调查与敏	cow mastitis and sensitive	燕,张涛,彭彰,王潘,陈鲁	ang Tao,Peng Zhang,Wang	中国草食动物科学	Chinese herbivore science	36	4	46-48,49	2016	sn.2095-3887.2016.04.013
4774	缓冲蛋白胨水培养基筛选	mparison of buffered pept	韩镌竹,李欣南,宿艳芳.	Zhuzhu,Li Xinnan,Su Yanf	黑龙江畜牧兽医（上半月）	ndry and Veterinary Medic	-	5	276-278,288	2016	-
4775	152份淡水龙虾中致病菌	Analysis of distribution and	兵,刘纯成,邢亚东,刘靓,侯	uncheng,Xing Yadong,Liu L	现代预防医学	modern preventive medicin	43	17	3134-3138	2016	-
4776	腹泻病原菌分布及耐药性	g resistance of pathogenic	豪,刘慧,刘丽莎,泰淑红,邵	u Hui,Lisa Liu,Tai Shuhong,	医学检验与临床	edical Laboratory and Clini	27	5	43-44	2016	sn.1673-5013.2016.05.014
4777	中外籍沙门菌感染患者流行	nella infection among Chin	汤泓,黄峥,张岭,徐云蕴,侯	g,Huang Zheng,Zhang Ling,	中国热带医学	Chinese tropical medicine	16	5	475-477,491	2016	cnki.46-1064/r.2016.05.18
4778	查病人血液中分离出伤寒	the blood of a patient with	扎西央宗.	Tashi Yangzong.	西藏科技	Tibet Technology	-	10	57	2016	sn.1004-3403.2016.10.020
4779	氏菌中磺胺类药物耐药基	istance genes in Salmonell	王晓菲,牛鑫鑫,廖聪,孙颖,	ofei,Niu Xinxin,Liao Cong,S	家禽科学	poultry science	-	3	10-12	2016	sn.1673-1085.2016.03.004
4780	省沙门菌感染血清型分布及	drug resistance of Salmone	余新华.	Yu Xinhua.	中国现代药物应用	dern drug application in Ch	10	1	166-167	2016	i.cn11-5581/r.2016.01.131
4781	沙门菌的分离鉴定与耐药性	resistance analysis of Salm	,周雯,李芙蓉,陆广富,朱国	Wen,Li Furong,Lu Guangfu,	中国家禽	Chinese poultry	38	5	60-63	2016	sn.1004-6364.2016.05.016
4782	食源性致病菌污染状况调	e pathogenic bacteria in se	宫春波,王朝霞,董峰光.	unbo,Wang Chaoxia,Dong F	中国食品卫生杂志	hinese Food Hygiene Journ	28	1	103-106	2016	0.13590/j.cjfh.2016.01.023
4783	2013年食品中食源性致病	nual monitoring results of	,刘翔,郝琼,曹守勤,闫立君	ng,Hao Qiong,Cao Shouqin	医学动物防制	medical animal control	32	1	31-34	2016	0.7629/yxdwfz201601011
4784	年常熟市食源性疾病主动	e surveillance of foodborne	月,王俭,常伟冰,沈明珠,	ng Jian,Chang Weibing,She	公共卫生与预防医学	Health and Preventive Me	27	2	71-73	2016	-
4785	胶电泳技术在一起食物中	ld gel electrophoresis tech	于烽,刘艳,苏静,远雪梅,	,Liu Yan,Su Jing,Yuan Xuem	中国卫生检验杂志	ese Journal of Health Inspe	26	4	546-548	2016	-
4786	门菌引起食物中毒的病原	of food poisoning caused	张甜.	Zhang Tian.	中国城乡企业卫生	ygiene in urban and rural a	31	8	54-56	2016	/j.1003-5052.2016.08.022
4787	4年大连市金州新区食品	bial monitoring in Jinzhou N	宫晓君.	Gong Xiaojun.	预防医学论坛	Preventive Medicine Forum	22	2	封2,154	2016	-
4788	年深圳市腹泻疾病的病原	udy on the etiology of diar	美玲,李卫宁,李迎慧,张海	Li Weining,Li Yinghui,Zhan	现代检验医学杂志	l of Modern Laboratory Me	31	3	143-146,149	2016	sn.1671-7414.2016.03.040
4789	区腹泻患者中食源性病原	analysis of foodborne path	郑剑,叶硕,沈玄艺,杨元斌,	an,Ye Shuo,Shen Xuanyi,Ya	中国卫生检验杂志	ese Journal of Health Inspe	26	15	2235-2239	2016	-
4790	水环境中沙门菌分布特征	resistance of Salmonella in	启发,高红,章丹阳,罗学辉	ao Hong,Zhang Danyang,Lu	中国食品卫生杂志	hinese Food Hygiene Journ	28	6	709-713	2016	0.13590/j.cjfh.2016.06.005
4791	肠炎沙门菌家庭食物中毒	food poisoning case caused	张梅.	Zhang Mei.	大家健康（中旬版）	one is healthy (mid-term e	10	8	49-50	2016	-
4792	炎沙门菌食物中毒的实验	of a case of Salmonella Ent	玲,周文革,张志魁,魏美	ou Wenge,Zhang Zhikui,W	海峡预防医学杂志	s Journal of Preventive Me	22	4	89-90	2016	-
4793	多种病毒和细菌感染状况	al infections in large-scale	明薇,程悦宁,王建科,赵	eng Yuening,Wang Jianke,Z	特产研究	Specialty research	38	3	1-4	2016	20/j.cnki.tcyj.2016.03.001
4794	5年湖北省突发中毒事件	acteristics of sudden poiso	卫婷婷,姚永祥,梅良英.	gting,Yao Yongxiang,Mei Li	公共卫生与预防医学	Health and Preventive Me	27	6	52-55	2016	-
4795	015年食源性致病菌监测	s of annual foodborne path	方英,钟艳,胡卓,刘育兰.	ing,Zhong Yan,Hu Zhuo,Liu	微量元素与健康研究	e elements and health rese	33	6	41-42	2016	-
4796	3年-2015年感染性腹泻病	nalysis of annual surveillan	光清,肖锦晖,陈清凉,荏静	ao Jinhui,Chen Qingliang,C	中国卫生检验杂志	ese Journal of Health Inspe	26	10	1458-1461	2016	-
4797	对喹诺酮类药物耐药性及	of resistance to quinolone	明远,古丽娜孜,王威,张亚	gyuan,Gulinazi,Wang Wei,Z	食品科学	food science	37	17	150-155	2016	pkx1002-6630-201617025
4798	综合征合并血流感染143例	ith bloodstream infection1	梁欣,柳明波,李春玫.	g Xin,Liu Mingbo,Li Chunn	中国感染与化疗杂志	urnal of Infection and Che	16	3	252-256	2016	/j.1009-7708.2016.03.003
4799	同餐次两批聚餐人群食物	ng two groups of people d	,许舒乐,杨荣兴,张强,张	,Yang Rongxing,Zhang Qia	中国热带医学	Chinese tropical medicine	16	2	137-140	2016	cnki.46-1064/r.2016.02.11
4800	2016年南京市食源性沙门	6Identification and analysi	超,程婷婷,江晓,叶艳华,张	Tingting,Jiang Xiao,Ye Yanh	饮食保健	Diet and health care	3	20	197-199	2016	sn.2095-8439.2016.20.239
4801	广州市即食食品微生物的	amination of ready-to-eat	邓志爱,胡玉山,陈佳璇,张	i,Hu Yushan,Chen Jiaxuan,Z	医学动物防制	medical animal control	32	10	1071-1073,1076	2016	0.7629/yxdwfz201610003
4802	婴幼儿分离株耐药基因及	enes of Salmonella typhim	进,彭子欣,张宏元,赵熙,	ng Zixin,Zhang Hongyuan,Z	中国食品卫生杂志	hinese Food Hygiene Journ	28	5	567-575	2016	0.13590/j.cjfh.2016.05.002
4803	015年食品中食源性致病	annual survey results of foo	刘世芸,王圣庆,刘璞.	Shiyun,Wang Shengqing,Liu	安徽预防医学杂志	i Journal of Preventive Me	22	2	107-109	2016	-
4804	无锡市感染性腹泻的病原	iology of infectious diarrhe	管红霞,肖勇,沙丹.	uan Hongxia,Xiao Yong,sata	检验医学	laboratory medicine	31	11	953-958	2016	.1673-8640.2016.011.006
4805	源性疾病主动监测结果分	of active surveillance of fo	谭云鹤.	Tan Yunhe.	内科	Internal Medicine	11	2	289-291	2016	ki.cn45-1347/r.2016.02.46
4806	生餐食源性致病菌污染状	tus of foodborne pathogen	姚雪婷,刘展华,李秀桂.	Xueting,Liu Zhanhua,Li Xiu	中国学校卫生	Chinese school health	37	11	1688-1689,1693	2016	ki.1000-9817.2016.11.028
4807	南京市哨点医院监测食源性	of foodborne diseases mo	云,江晓,杜雪飞,叶艳华,王	g Xiao,Du Xuefei,Ye Yanhua	职业与健康	Occupation and health	32	21	2939-2942	2016	-
4808	14年食源性致病菌污染监	and analysis of foodborne	,栾阳,彭雁,张晔,魏晓光,	,Peng Yan,Zhang Ye,Wei Xi	中国卫生检验杂志	ese Journal of Health Inspe	26	4	573-575,581	2016	-

4809	产链中沙门菌耐药性分	n of Salmonella in the broi	娟,黄秀梅,王君玮,杨瑞梅	g Xiumei,Wang Junwei,Yan	中国家禽	Chinese poultry	38	24	22-27	2016	sn.1004-6364.2016.24.005
4810	婴幼儿腹泻病原学检测结	ogical detection results of i	宪华,陈王征,崔桂平,王丹	hen Wangzheng,Cui Guipin	国际检验医学杂志	onal journal of laboratory	37	15	2186-2188	2016	sn.1673-4130.2016.15.057
4811	菌斯坦利沙门菌的分子	alysis of Salmonella Stanle	王丽丽,陈倩.	Wang Lili,Chen Qian.	中国食品卫生杂志	hinese Food Hygiene Journ	28	1	27-31	2016	).13590/j.cjfh.2016.01.006
4812	急性腹泻儿童的肠道病原	ce in children with acute d	蔡洁皓,郭家胤,许学斌,王	ehao,Guo Jiayin,Xu Xuebin,	中华传染病杂志	se Journal of Infectious Dis	34	1	19-22	2016	sn.1000-6680.2016.01.005
4813	年贵州省伤寒和副伤寒流	cteristics of typhoid fever a	荷,姚光海,黄艳萍,韦小	o Guanghai,Huang Yanpin	华南预防医学	th China Preventive Medi	42	6	562-564	2016	).13217/j.scjpm.2016.0562
4814	控中心细菌性食物中毒检	g Testing at Primary Center	曾丽,孙秋香,高艳,吕郁豪	i,Sun Qiuxiang,Gao Yan,Lu	疾病监测与控制	sease surveillance and cont	10	11	916-917	2016	-
4815	市饼干、糕点微生物安全	ety risk monitoring results	胡津津,汪少敏.	Hu Jinjin,Wang Shaomin.	现代农业科技	odern agricultural technolo	-	20	269-271	2016	sn.1007-5739.2016.20.154
4816	食品中沙门菌的检测方法	ection methods for Salmo	孙玉祝,陈江楠,夏兆飞.	uzhu,Chen Jiangnan,Xia Zh	中国兽医杂志	Chinese Veterinary Journal	52	1	87-89	2016	sn.0529-6005.2016.01.030
4817	省甲型副伤寒沙门菌分子	g and drug resistance of Sa	帆,穆玉姣,苏佳,夏胜利,黄	Mu Yujiao,Su Jia,Xia Sheng	中国病原生物学杂志	ese Journal of Pathogen Bi	11	8	701-704	2016	10.13350/j.cjpb.160806
4818	食品中食源性致病菌污染	of foodborne pathogenic b	,王东健,刘长勇,罗瑞峰,	gjian,Liu Changyong,Luo R	预防医学情报杂志	of Preventive Medicine Inf	32	7	700-703	2016	-
4819	年江阴市伤寒副伤寒流行	characteristics of typhoid a	姚建香,马焰,钱程.	Jianxiang,Ma Yan,Qian Ch	职业与健康	Occupation and health	32	20	2835-2838	2016	-
4820	品中金黄色葡萄球菌及沙	s aureus and Salmonella in	素燕,姜娴,王晓红,周珊慧	iang Xian,Wang Xiaohong,	中国卫生检验杂志	ese Journal of Health Inspe	26	5	665-667	2016	-
4821	道公共场所从业人员沙门	tion among employees in p	枫,何必隆,陈夏明,武照燕	e Bilong,Chen Xiaming,Wu	今日健康	health today	15	3	377-377	2016	-
4822	兵团哨点医院食源性疾病	e results of foodborne dise	罗建忠,王建平.	uo Jianzhong,Wang Jianpin	预防医学情报杂志	of Preventive Medicine Inf	32	11	1145-1148	2016	-
4823	5年廊坊市食物中毒报告	food poisoning reports in	李红,李瑞丽.	Li Hong,Li Ruili.	中国保健营养	Chinese health nutrition	26	3	367	2016	-
4824	腹泻的105例病原微生物检	ren105Analysis of pathoge	航,张晓丽,张吉生,王勇,王	iaoli,Zhang Jisheng,Wang Y	医学信息	medical information	29	8	197-198	2016	sn.1006-1959.2016.08.144
4825	年黔西南州食源性致病菌	f foodborne pathogenic ba	凯金祥,姚丽,潘梅.	Kai Jinxiang,Yao Li,Pan Mei	职业与健康	Occupation and health	32	6	764-766	2016	-
4826	蒙得维的亚沙门菌流行特	l characteristics of Salmon	,王爱敏,黄峥,石维敏,胡雪	Aimin,Huang Zheng,Shi W	疾病监测	disease surveillance	31	7	540-545	2016	sn.1003-9961.2016.07.004
4827	山县生乳致病菌污染状况	status of raw milk pathoge	谢向长.	Xie Xiangchang.	医药前沿	Medical Frontier	6	1	32-33	2016	sn.2095-1752.2016.01.023
4828	、弱雏中沙门氏菌的分	monella from Tibetan chick	琪,赵瑾,李霞辉,徐超,王志	Jin,Li Xiahui,Xu Chao,Wan	四川农业大学学报	of Sichuan Agricultural Un	34	3	354-358	2016	sn.1000-2650.2016.03.016
4829	从业人员食源性致病菌带	athogenic bacteria among f	雯,焦文志,高曼,宋晓敏,方	Venzhi,Goldman,Song Xiao	世界临床医学	world clinical medicine	10	3	7-8	2016	-
4830	门菌和阿贡纳沙门菌耐药	ping of Salmonella Delphi	姣,谢志强,潘静静,夏胜利	,Xie Zhiqiang,Pan Jingjing,X	中国病原生物学杂志	ese Journal of Pathogen Bi	11	6	517-521	2016	10.13350/j.cjpb.160609
4831	海市宝山区沙门菌监测分	nalysis and capability asses	平,金凯,茅俭英,彭真真,朱	ncaid,Mao Jianying,Peng Z	疾病监测	disease surveillance	31	7	546-553	2016	sn.1003-9961.2016.07.005
4832	门菌的血清型分布及耐药	and drug resistance analy	陈东杰,胡辛兰,伍严安.	n Dongjie,Hu Xinlan,Wu Ya	创伤与急诊电子杂志	ma and emergency e-maga	4	1	25-27	2016	nki.11-9332/r.2016.01.007
4833	014年市售水产品受食源	ntamination status of com	华,赵洁玲,卓菲,龙冬玲,沈	eling,Zhuo Fei,Long Dongli	中国卫生检验杂志	ese Journal of Health Inspe	26	9	1310-1312	2016	-
4834	津某猪场沙门氏菌的分离	ication of Salmonella from	晓伟,蒙晓雷,郭恋,王健	i,Meng Xiaolei,Guo Lian,W	养猪	pig raising	-	3	105-108	2016	sn.1002-1957.2016.03.034
4835	市房山区肠道多病原监	lts of multiple intestinal p	波,王云霞,史文凤,黄少平,	nxia,Shi Wenfeng,Huang S	职业与健康	Occupation and health	32	17	2420-2422	2016	-
4836	鸡养殖和屠宰加工环节中	cken Breeding, Slaughterin	杰,薛成玉,谢平会,郑晓华,	e Chengyu,Xie Pinghui,Zhe	中国食品卫生杂志	hinese Food Hygiene Journ	28	4	485-488	2016	).13590/j.cjfh.2016.04.016
4837	家宴沙门菌食物中毒的调	sis of Salmonella food pois	李净,林建维,靳桂才.	Li Jing,Lin Jianwei,Jin Guica	疾病监测与控制	sease surveillance and cont	10	8	650-651	2016	-
4838	沙门菌的分离及其生物学	characteristics of Salmone	伟,贾雪波,肖芹,窦新红,邹	ia Xuebo,Xiao Qin,Dou Xin	中国家禽	Chinese poultry	38	6	53-55	2016	sn.1004-6364.2016.06.013
4839	市县餐饮食品微生物污染	test results of catering foo	符天晓,陈婉娃,梁书敏.	nxiao,Chen Wanwa,Liang S	首都食品与医药	Capital Food and Medicine	-	2	18-19	2016	-
4840	年余姚市沙门菌菌型分布	distribution and drug susc	黄邵军,张建群,罗学辉.	Shaojun,Zhang Jianqun,Luo	中国卫生检验杂志	ese Journal of Health Inspe	26	21	3111-3113	2016	-
4841	海产品中常见致病菌的检	common pathogenic bacte	,李盛鹏,林奕华,高素虹,	engpeng,Lin Yihua,Gao Su	中国卫生检验杂志	ese Journal of Health Inspe	26	6	805-807	2016	-
4842	分省市外卖盒饭中微生物	on in take-out lunch boxes	燕,闫琳,杨舒然,胡静,李	Yan Lin,Yang Shuran,Hu Jin	卫生研究	health research	45	6	1010-1012	2016	-
4843	氨基糖苷类耐药表型及耐	phenotypes and resistan	福星,刘莉,戴建华,蔡丙	ng,Liu Li,Dai Jianhua,Cai Bi	中国家禽	Chinese poultry	38	17	56-58	2016	sn.1004-6364.2016.17.013
4844	氏菌的分离鉴定及流行血	t pigeons in Shanghai and	伟芳,詹泽强,任行星,林	ng,Zhan Zeqiang,any plan	中国畜牧兽医	nal husbandry and veterin	43	12	3322-3328	2016	ki.1671-7236.2016.12.035
4845	韦太夫雷登沙门菌在全	onella Wetefreden origin	隽卿,李迎慧,张建民,周	qing,Li Yinghui,Zhang Jian	疾病监测	disease surveillance	31	1	73-81	2016	sn.1003-9961.2016.01.017

4846	门菌感染引发脓毒血症致	h caused by sepsis caused	余明元,范云飞,苟放,徐朝	yuan,Fan Yunfei,Keep quiet	西南国防医药	west National Defense Me	26	11	1355-1356	2016	sn.1004-0188.2016.11.053
4847	要病原菌的分离鉴定与药	sceptibility Test of Importa	珂,李小慧,林静,洪莹莹,金	Xiaohui,Lin Jing,Hong Yingy	天津农学院学报	l of Tianjin Agricultural Uni	23	4	5-8	2016	sn.1008-5394.2016.04.002
4848	制品中沙门菌的半定量风	dy of Salmonella in cooked	周少君,梁辉,黄芮.	u Shaojun,Liang Hui,Huang	食品科技	food technology	41	4	324-328	2016	-
4849	普沙门菌耐药性与毒力	ce and virulence genes of	晓霞,杨杰,吴晓妹,张利娟	,Yang Jie,Wu Xiaomei,Zhar	天津医药	Tianjin Medicine	44	11	1387-1390	2016	10.11958/20160630
4850	染病医院血流感染细菌构	g resistance of bloodstrea	,王启明,李青峰,刘应芬,陈	g Qiming,Li Qingfeng,Liu Yi	现代预防医学	nodern preventive medicin	43	15	2806-2810	2016	-
4851	年随州市食品中致病菌污	genic bacteria contaminati	,李月,聂文武,王永光,陈平	Nie Wenwu,Wang Yonggua	中国卫生检验杂志	ese Journal of Health Inspe	26	14	2085-2086	2016	-
4852	口饲料中沙门氏菌的分离	tion and identification of S	汪霖.	Wang Lin.	科技与企业	Technology and Enterprise	-	7	251-251	2016	sn.1004-9207.2016.07.211
4853	金黄色葡萄球菌和沙门菌	sistance of Staphylococcus	湘晖,刘帅仁,萧福元,陈子	ui,Liu Shuairen,Xiao Fuyuar	实用预防医学	ractical preventive medicin	23	8	989-991	2016	sn.1006-3110.2016.08.030
4854	沙门菌喹诺酮耐药分子机	alysis of quinolone resistan	,于兆阳,冯宝立,郑兰紫,	haoyang,Feng Baoli,Zheng	中国卫生检验杂志	ese Journal of Health Inspe	26	7	1050-1053	2016	-
4855	肠炎患儿病原菌分析与预	h on pathogenic bacteria in	麦海亮,王红吉,杨露,王肖	g,Wang Hongji,Yang Lu,Wa	中华医院感染学杂志	ournal of Hospital Infectiou	26	1	197-199	2016	.11816/cn.ni.2016-152422
4856	症病原菌及耐药性10年的	of septicemia in children1	黄梦,李克诚,徐旭.	uang Meng,Li Kecheng,Xu X	中国生化药物杂志	ese Journal of Biochemical	-	2	165-168	2016	-
4857	居民主要食品食源性致病	genic bacteria in the main	开春,胡晓宁,甘连军,权玉	n,Hu Xiaoning,Gan Lianjun,	中国卫生检验杂志	ese Journal of Health Inspe	26	24	3593-3595	2016	-
4858	门菌食物中毒病原学检测	f the etiology of a case of S	,王睿,查涛,刘文,孙永,邓	Rui,Cha Tao,Liu Wen,Sun Y	中国卫生检验杂志	ese Journal of Health Inspe	26	6	872-874	2016	-
4859	市食品中沙门氏菌污染	onella contamination in fo	磊,刘玉婷,郎小倩,翁庆	iu Yuting,Lang Xiaoqian,We	师范大学学报（自然科学	u Normal University (Natur	34	5	36-40	2016	sn.1004-5570.2016.05.006
4860	液标本中检出伤寒沙门	s detected in the patient's	宫晓君.	Gong Xiaojun.	中国冶金工业医学杂志	rnal of Metallurgical Indust	33	4	496	2016	-
4861	上海市宝山区腹泻病监测	sease surveillance results i	伦辉,沈隽卿,金凯,杨兴	i,Shen Junqing,Kincaid,Yan	实用预防医学	ractical preventive medicin	23	2	215-218	2016	sn.1006-3110.2016.02.029
4862	门菌血清型分布及耐药情	ribution and drug resistan	书媛,胡三梅,庞晶晶,高	yuan,Hu Sanmei,Pang Jing	河北医药	Hebei Medicine	-	3	449-451	2016	sn.1002-7386.2016.03.044
4863	死鸡胚沙门氏杆菌的分	onella from dead chicken	李遵瑞.	Li Zunrui.	中国畜禽种业	estock and poultry breeding	12	3	141-143	2016	sn.1673-4556.2016.03.116
4864	监测工作在基层哨点医院	e surveillance of foodborne	王晓岚.	Wang Xiaolan.	航空航天医学杂志	urnal of Aerospace Medici	27	3	387-388	2016	sn.2095-1434.2016.03.065
4865	性感染性腹泻病原菌分	ance of pathogenic bacteri	姜哲,迟维维.	Jiang Zhe,Chi Weiwei.	临床医药文献电子杂志	edical literature electronic	3	44	8863-8863	2016	sn.2095-8242.2016.44.126
4866	沙门菌感染临床特点及耐	ug resistance patterns of Sa	王庭庭,徐飞,李玫,郭红梅	ngting,Xu Fei,Li Mei,Guo H	临床儿科杂志	Journal of Clinical Pediatric	34	4	246-248	2016	sn.1000-3606.2016.04.002
4867	沙门菌引起的食物中毒实	of a case of food poisoning	朋,王海欣,姜颖丽,郭影,杨	g Haixin,Jiang Yingli,Guo Yi	医药论坛杂志	Medical Forum Magazine	37	1	117-118	2016	-
4868	病原菌分布、药物敏感性	d drug resistance changes	樊红丽,张米,高丽.	an Hongli,Zhang Mi,Goryec	检验医学与临床	boratory medicine and clini	13	z2	247-250	2016	sn.1672-9455.2016.26.105
4869	生产链中沙门菌的分离	tion of Salmonella in broile	思俊,王娟,赵建梅,李玉清	ang Juan,Zhao Jianmei,Li Yu	中国兽医杂志	Chinese Veterinary Journal	-	6	-	2016	-
4870	顶山市食品中食源性致病	lts of foodborne pathogeni	艳艳,王雨,席慧霞,王国	yan,Wang Yu,Xi Huixia,Wa	现代预防医学	nodern preventive medicin	43	4	620-622,648	2016	-
4871	广东省熟肉制品致病菌污	ontamination status of coo	,赖蔚苳,杨冰,王海燕,陈	iruan,Yang Bing,Wang Haiy	华南预防医学	uth China Preventive Medi	42	2	192-195	2016	.13217/j.scjpm.2016.0192
4872	房山区沙门菌血清学鉴定	ug resistance analysis of Sa	,史文凤,黄少平,阚震,刘	nfeng,Huang Shaoping,Kar	中国卫生检验杂志	ese Journal of Health Inspe	26	6	811-812	2016	-
4873	在从业人员伤寒痢疾快速	gy in rapid screening of typ	,魏丽娜,吴步东,冯贞玉,	Lina,Wu Butong,Feng Zher	中国卫生产业	China health industry	13	2	104-106	2016	ki.1672-5654.2016.02.104
4874	沙门菌表型特征及PFGE分	cteristics of Salmonella in	欧新华,贾华云,苏良,刘晓	hua,Jia Huayun,Su Liang,Liu	实用预防医学	ractical preventive medicin	23	1	39-42	2016	sn.1006-3110.2016.01.010
4875	物中毒病原菌调查与预防	acterial food poisoning and	曾德兴,黄思思,陈应坚.	Dexing,Huang Sisi,Chen Yin	现代诊断与治疗	dern Diagnosis and Treatm	27	8	1518-1520	2016	-
4876	粉中致病菌污染状况检	bacteria contamination in	大进,杨舒然,胡静,李宁,	ang Shuran,Hu Jing,Li Ning	中国公共卫生	Chinese public health	32	5	602-604	2016	847/zgggws2016-32-05-10
4877	生肉与生肉制品致病菌污	us of commercially availab	学燕,许姣,黄淑华,陈磊,	an,Xu Jiao,Huang Shuhua,C	河南预防医学杂志	n Journal of Preventive Me	27	12	955-958	2016	m.1006-8414.2016.12.024
4878	头市细菌性食物中毒与病	d poisoning and pathogeni	,江素红,林奕华,高素虹,	g Suhong,Lin Yihua,Gao Su	预防医学情报杂志	of Preventive Medicine Info	32	1	77-80	2016	-
4879	151株志贺菌分布特征与耐	distribution characteristics	高庆双,高春燕,张晓玲.	shuang,Gao Chunyan,Zhang	现代预防医学	nodern preventive medicin	43	1	60-61,110	2016	-
4880	上的食品和水源性疾病回	food and waterborne disea	,佘燕燕,张玲娟,刘晓荣,	anyan,Zhang Lingjuan,Liu	职业与健康	Occupation and health	32	3	338-341	2016	-
4881	菌分离鉴定及药敏试验案	tion and drug susceptibility	陈巨清,杨振燕.	Chen Juqing,Yang Zhenyan	兽医导刊	veterinary guide	-	13	65-66,67	2016	sn.1673-8586.2016.13.038
4882	西地区学生营养早餐食	ms in nutritional breakfast	郭海艳.	Guo Haiyan.	中国校医	Chinese school doctor	30	7	506509	2016	-

4883	菌引起的食物中毒检测及	sed by Salmonella Newport	,何春荣,林英华,廖琳虹,引	nrong,Lin Yinghua,Liao Lin	中国食品卫生杂志	hinese Food Hygiene Journ	28	2	172-174	2016	).13590/j.cjfh.2016.02.007
4884	鼠伤寒沙门氏菌的分离鉴	racteristics of Salmonella t	王传宝.	Wang Chuanbao.	农家科技（下旬刊）	cience and Technology (lat	-	3	202-203	2016	-
4885	5年禹州市食源性致病菌的	sis of foodborne pathogen	王爱香,杨秀范,马晓静,王	kiang,Yang Xiufan,Ma Xiaoj	河南预防医学杂志	n Journal of Preventive Me	27	9	666-668	2016	m.1006-8414.2016.09.009
4886	道沙门菌双相亚利桑那亚	rrhea-causing Salmonella e	,吕冰,张新,严寒秋,钱海坤	hang Xin,severe cold autum	中国预防医学杂志	se Journal of Preventive Me	17	6	405-408	2016	5/j.1009-6639.2016.06.002
4887	宁波市肠炎沙门菌分子分	istance characteristics of S	元斌,宋启发,闫鹏,高红,叶	,Song Qifa,Yan Peng,Gao H	疾病监测	disease surveillance	31	12	1007-1011	2016	-
4888	伤寒沙门氏菌食物中毒事	of Salmonella Typhimuriun	梁少溢,林鑫,韦冬萍.	g Shaoyi,Lin Xin,Wei Dongp	医学动物防制	medical animal control	32	8	902-904,907	2016	0.7629/yxdwfz201608025
4889	水污染致感染性腹泻病原学	y of infectious diarrhea cau	李海余,唐丽群,田祖建.	Haiyu,Tang Liqun,Tian Zujia	医学理论与实践	Medical theory and practice	29	10	1369-1370	2016	-
4890	区感染性腹泻细菌病原学	l etiology of infectious diar	李平,陈懿,徐景野.	Li Ping,Chen Yi,Xu Jingye.	中国卫生检验杂志	ese Journal of Health Inspe	26	3	397-399,402	2016	-
4891	寒沙门菌引起的食源性疾	dborne illness caused by Sa	和仙.	He Xian.	中国保健营养	Chinese health nutrition	26	33	339-340	2016	sn.1004-7484.2016.33.565
4892	由肠炎沙门菌引起的食物	oning caused by Salmonella	蒋奎英,徐敏,高日红.	ng Kuiying,Xu Min,Gao Riho	预防医学论坛	Preventive Medicine Forum	22	4	318,封3	2016	-
4893	15年度101例食源性疾病监	r101Analysis of foodborne	周军华,况强华.	hou Junhua,Kuang Qianghu	实验与检验医学	imental and Laboratory Me	-	1	115-117	2016	sn.1674-1129.2016.01.045
4894	肉中沙门氏菌的污染及耐药	nd drug resistance of Salm	娟,吕世明,刘芸,刘玉梅,张	Shiming,Liu Yun,Liu Yumei,	黑龙江畜牧兽医（上半月）	ndry and Veterinary Medic	-	2	257-259	2016	-
4895	人源沙门氏菌的血清分布	and drug resistance analys	邵燕,钱玉春,胡中旺,张文艳	Yuchun,Hu Zhongwang,Zh	安徽预防医学杂志	i Journal of Preventive Med	22	5	291-293,329	2016	-
4896	泻120例病原微生物和药敏	pathogenic microorganism	刘宗生.	Liu Zongsheng.	医学信息	medical information	29	29	264-264	2016	sn.1006-1959.2016.29.211
4897	副伤寒甲临床特征和耐药	eristics and drug resistance	王辉,秦波,高源.	ang Hui,Play waves,Gao Yu	重庆医学	Chongqing Medicine	45	22	3083-3085	2016	sn.1671-8348.2016.22.021
4898	定病原体的食源性疾病病	dborne disease cases of sp	王黎荔,山若青,高四海,蔡	Mountains are green,Gao S	中国食品卫生杂志	hinese Food Hygiene Journ	28	3	378-381	2016	).13590/j.cjfh.2016.03.023
4899	市朝阳区食源性腹泻病例	on of foodborne diarrhea c	明捷,魏云芳,巴蕾,姜薇,林	Chiet,Wei Yunfang,Bale,Jiar	职业与健康	Occupation and health	32	18	2517-2519	2016	-
4900	省哨点医院食源性疾病病	borne disease cases in sent	强,万青青,王晓雯,阮元,赵	Qingqing,Wang Xiaowen,Ru	职业与健康	Occupation and health	32	12	1674-1677	2016	-
4901	沙门菌β-内酰胺类抗生素耐	th diarrheaβ-Analysis of lac	邵启民,赵士勇,吴亦栋,厉小	min,Zhao Shiyong,Wu Yido	浙江临床医学	Zhejiang Clinical Medicine	18	3	479-480	2016	-
4902	幼儿细菌性腹泻的病原学分	of bacterial diarrhea in infa	王惠姣,徐娇君,朱根林.	g Huijiao,Xu Jiaojun,Zhu Ge	浙江临床医学	Zhejiang Clinical Medicine	18	4	728-729	2016	-
4903	州地区沙门菌的耐药情况研	g resistance of Salmonella	慧芬,郑文力,胡立成,包其	ifen,Zheng Wenli,Hu Licher	中国卫生检验杂志	ese Journal of Health Inspe	26	2	283-286	2016	-
4904	细菌感染检测及其生物学	n Fujian Province and deter	华,刘荣昌,万春和,施少华	gchang,Wan Chunhe,Shi Sh	中国兽医杂志	Chinese Veterinary Journal	52	11	10-13	2016	-
4905	生食动物性水产品中食源	borne pathogenic bacteria i	宫春波,王朝霞,董峰光.	unbo,Wang Chaoxia,Dong F	实用预防医学	ractical preventive medicin	23	12	1440-1444	2016	sn.1006-3110.2016.12.009
4906	年重庆市渝北区早餐食品	enic Quality of Breakfast Fo	海英,龚志华,胡迪.	aiying,Gong Zhihua,Wood	中国卫生检验杂志	ese Journal of Health Inspe	26	10	1502-1503,1508	2016	-
4907	小学食堂餐（饮）具消毒	d utensils in canteens of p	,吴健,高永文,亢玲玲,王月	n,Gao Yongwen,Kang Lingli	牡丹江医学院学报	al of Mudanjiang Medical C	37	4	123-124	2016	-
4908	区哨点医院成人急性腹泻	s in adults with acute diarr	,刘岩红,张万菊,何静,朱华	g,Zhang Wanju,He Jing,Zh	中华医院感染学杂志	ournal of Hospital Infectiou	26	22	5100-5103	2016	.11816/cn.ni.2016-160548
4909	肠道感染疾病临床特征及	esistance analysis of comm	郑海淑,黄永存.	heng Haishu,Huang Yongcu	临床医药文献电子杂志	edical literature electronic	3	20	4018-4018,4019	2016	-
4910	泻病例沙门菌的血清型分	stribution and drug resista	张晶,张欣强,侯水平,吴新伟	Zhang Xinqiang,Hou level,	实用预防医学	ractical preventive medicin	23	5	616-617,623	2016	sn.1006-3110.2016.05.035
4911	道门诊2376例患者的临床分	clinic2376Clinical analysis o	陆群.	Lu Qun.	饮食保健	Diet and health care	3	18	27-28	2016	-
4912	湖海产品中副溶血性弧菌	of Vibrio parahaemolyticus	,沈飚,余辉,周秀锦,张静,王	afterglow,Zhou Xiujin,Zhar	中国食品卫生杂志	hinese Food Hygiene Journ	28	4	440-444	2016	).13590/j.cjfh.2016.04.007
4913	013年阳江市沙门菌监测结	Salmonella Surveillance Re	天才,黄焕宜,陈怡静,孙亚	i,Huang Huanyi,Chen Yijing	实用预防医学	ractical preventive medicin	23	3	372-373,379	2016	sn.1006-3110.2016.03.039
4914	年食品中食源性致病菌监	monitoring results of foodb	赵丽萍,吕广波,刘晓娟,黄勇	Lu Guangbo,Liu Xiaojuan,H	医学动物防制	medical animal control	32	6	680-682	2016	0.7629/yxdwfz201606030
4915	奉贤区感染性腹泻病原菌分	enic bacteria in infectious c	方文娟,唐群力.	Fang Wenjuan,Tang Qunli.	中国消毒学杂志	hinese Journal of Disinfecti	33	11	1090-1092	2016	sn.1001-7658.2016.11.020
4916	患者沙门菌血清型及其耐药	ypes and drug resistance in	白帆,赵嘉咏,谢志强,夏胜利	n,Zhao Jiayong,Xie Zhiqian	中国卫生检验杂志	ese Journal of Health Inspe	26	9	1335-1338	2016	-
4917	感染性主动脉瘤1例	cted aortic aneurysm1exan	靳丽妍,朱光发.	Jin Liyan,Zhu Guangfa.	中国感染与化疗杂志	urnal of Infection and Cher	16	6	785-787	2016	3/j.1009-7708.2016.06.019
4918	海北州食源性致病菌监测结	sults of foodborne pathoge	刘萍,晏伟.	Liu Ping,Yan Wei.	青海医药杂志	Qinghai Medical Journal	46	3	53-55	2016	-
4919	血性弧菌和沙门氏菌主动	f Vibrio parahaemolyticus	者添,宋景红,刘海涛,庄国民	ng Jinghong,Liu Haitao,Zhu	食品安全导刊	food safety guide	-	16	65-67	2016	-

4920	寒沙门菌聚集性病例的病	and traceability analysis o	廖玉姣,苏佳,黄学勇,夏胜利	jiao,Su Jia,Huang Xueyong,	中国人兽共患病学报	Chinese Journal of Zoonose	32	12	1122-1125	2016	n.1002-2694.2016.012.015
4921	市崇文区食品中食源性致	of foodborne pathogenic	张旭,常志荣,吴伟.	ng Xu,Chang Zhirong,Wu W	中国卫生检验杂志	ese Journal of Health Inspe	26	22	3300-3303	2016	-
4922	门氏菌耐药性分析及PFG	sistance of Salmonella stra	刘艳,苏静,马鑫,远雪梅,杨	,Su Jing,Ma Xin,Yuan Xuem	疾病预防控制通报	se Prevention and Control	31	6	9-12,49	2016	15/j.cnki.jbyfkztb.1605044
4923	要细菌性病原体食源性疾	ne diseases caused by majo	福,祝白春,金迪,李小成,王	Baichun,Jindi,Li Xiaocheng,	现代预防医学	modern preventive medicin	43	21	3880-3883,3895	2016	-
4924	浙江省仙居腹泻病沙门菌	rveillance results of diarrh	陈华斌,吴灵芝,余央云,赵	Huabin,Wu Lingzhi,Yu Yang	中国卫生检验杂志	ese Journal of Health Inspe	26	13	1933-1936	2016	-
4925	院临床分离沙门菌耐药性	ng of clinically isolated Sal	周厚德,刘洋,姜茗,刘道峰	ude,Liu Yang,Jiang Ming,Liu	中国消毒学杂志	hinese Journal of Disinfecti	33	12	1200-1202	2016	sn.1001-7658.2016.12.021
4926	市售奶粉微生物污染状况	ontamination of milk powd	谢爱蓉,洪程基,李毅,胡玉琴	ong,Hong Chengji,Li Yi,Hu	中国卫生检验杂志	ese Journal of Health Inspe	26	20	3013-3015	2016	-
4927	2012-2014年食品微生物	012-2014Analysis of annua	蒋艺梅.	Jiang Yimei.	交通医学	traffic medicine	30	4	402-403	2016	-
4928	儿童感染鼠伤寒沙门氏菌临	es of children infected with	梁燕霞,李小南,伍绍东.	Yanxia,Li Xiaonan,Wu Shao	中国实用医药	Chinese Practical Medicine	11	17	44-45	2016	nki.11-5547/r.2016.17.022
4929	患者非伤寒沙门菌血清分	s of non-typhoid Salmonell	王鸣柳,林玫,廖和壮,黎冬梅	ngliu,Lin Mei,Liao Hezhuan	中国卫生检验杂志	ese Journal of Health Inspe	26	19	2861-2864	2016	-
4930	童粪便分离的459株沙门菌	Hospital459Distribution ch	雷旻,周高枫,张交生,彭宝珍	ou Gaofeng,Zhang Jiaoshe	中国感染控制杂志	ese Journal of Infection Co	15	11	817-820	2016	sn.1671-9638.2016.11.003
4931	区食品安全风险监测微生物	nalysis of food safety risk	柳勤,叶新,黄燕,张惠文.	,Ye Xin,Huang Yan,Zhang H	中国卫生检验杂志	ese Journal of Health Inspe	26	10	1415-1416,1419	2016	-
4932	生食肉类产品中食源性致病	bacteria in raw meat and r	欧阳以恒.	Ouyang Yiheng.	今日健康	health today	15	2	397-397,398	2016	-
4933	服务沙门氏菌食物中毒的	is of salmonella food poiso	吉喜军.	Ji Xijun.	中国保健营养	Chinese health nutrition	26	28	378-379	2016	sn.1004-7484.2016.28.512
4934	禽畜肉中沙门菌和单核细	monocytogenes contamina	董溢峰,傅灵菲,张晨,沈惠平	eng,Fu Lingfei,Zhang Chen,	中国卫生检验杂志	ese Journal of Health Inspe	26	11	1588-1590,1593	2016	-
4935	农村聚餐引起的沙门菌暴发	lmonella outbreak caused	周兴余,陈曦,袁伟,张丽杰	ngyu,Chen Xi,Yuan Wei,Zha	预防医学情报杂志	of Preventive Medicine Info	32	2	145-149	2016	-
4936	猪沙门菌的分离鉴定及其耐	ug resistance analysis of Sa	刘海燕,侯巍,王远微,杨发龙	Hou Wei,Wang Yuanwei,Ya	中国兽医杂志	Chinese Veterinary Journal	-	6	86-88	2016	-
4937	患者血流感染病原菌分布	hogenic bacteria in bloods	晴峰,刘应芬,王启明,陈莲,朱	Yingfen,Wang Qiming,Che	中华传染病杂志	se Journal of Infectious Dis	34	10	609-612	2016	sn.1000-6680.2016.10.008
4938	菌所致食物中毒的病原学	ysis of two cases of food po	,陈前进,林英华,廖琳虹,何	n Qianjin,Lin Yinghua,Liao	中国食品卫生杂志	hinese Food Hygiene Journ	28	1	32-36	2016	0.13590/j.cjfh.2016.01.007
4939	品从业人员沙门菌血清学	lmonella serology andPFG	,王艳,侯健国,殷宏梅,张有	,Hou Jianguo,Yin Hongmei	实用预防医学	ractical preventive medicin	23	9	1131-1133	2016	sn.1006-3110.2016.09.034
4940	川市龙岗区感染性腹泻病	of infectious diarrhea path	应坚,金玉娟,刘渠,李静媚	gjian,Jin Yujuan,Liu Qu,Li Ji	中国卫生检验杂志	ese Journal of Health Inspe	26	24	3584-3587	2016	-
4941	市瓯海区食品中食源性致	of foodborne pathogenic b	笑南,章忠辉,缪蔚蔚,林笑	hang Zhonghui,Miao Weiw	中国卫生检验杂志	ese Journal of Health Inspe	26	20	2989-2991,2993	2016	-
4942	沙门菌聚集性病例的病原	analysis of a Salmonella St	宝林,谢志强,黄学勇,穆玉	Xie Zhiqiang,Huang Xueyo	中国人兽共患病学报	Chinese Journal of Zoonose	32	5	497-501	2016	sn.1002-2694.2016.05.016
4943	沙门氏菌属和志贺氏菌属	teric Salmonella and Shigel	冯贤贵.	Feng Xiangui.	医药前沿	Medical Frontier	6	21	369-370	2016	-
4944	市五类食品食源性致病菌	f foodborne pathogenic ba	杨国然,茅乃玲,刘斌.	g Guoran,Mao Nailing,Liu	现代预防医学	modern preventive medicin	43	5	818-820	2016	-
4945	株沙门菌耐药表型与分子	sistance phenotype and m	晓,刘菊梅,方艳平,姜长宏	Jumei,Fang Yanping,Jiang	现代预防医学	modern preventive medicin	43	4	696-699	2016	-
4946	门氏菌的分离鉴定与耐药	ug resistance analysis of Sa	周碧君,胡兴义,张海,文明,程	un,Hu Xingyi,Zhang Hai,civ	贵州畜牧兽医	mal Husbandry and Veterin	40	6	1-4	2016	sn.1007-1474.2016.06.001
4947	市沙门菌临床分离株血清	tance analysis of Salmonel	,杨小蓉,周汉洪,田巍威,李	Xiaorong,Zhou Hanhong,Tia	预防医学情报杂志	of Preventive Medicine Info	32	8	781-785	2016	-
4948	中非伤寒沙门菌血清型及	nce of non-typhoidal Salmo	卿,傅慧琴,赵冰,黄红,章红	Huiqin,Zhao Bing,yellow	职业与健康	Occupation and health	32	1	52-54,58	2016	-
4949	14年食源性疾病主动监测	of annual foodborne diseas	陈艳,朱芳仪.	Chen Yan,Zhu Fangyi.	中国卫生检验杂志	ese Journal of Health Inspe	26	7	1025-1026	2016	-
4950	寒沙门氏菌食物中毒的流行	a case of food poisoning ca	付秀影,黄露.	Fu Xiuying,yellow dew.	首都公共卫生	capital public health	10	2	86-89	2016	-
4951	北京市房山区食源性疾病	rne disease surveillance re	王云霞,阚震,史文凤.	g Yunxia,Kan Zhen,Shi Wen	中国卫生检验杂志	ese Journal of Health Inspe	26	10	1469-1470,1473	2016	-
4952	州市服务行业人员沙门菌	monella carriage among se	建辉,黄梦颖,杨劲松,陈爱	uang Mengying,Yang Jinso	中国卫生检验杂志	ese Journal of Health Inspe	26	14	2081-2084	2016	-
4953	伤寒沙门菌肠炎临床分析	nce monitoring of non-typh	蔡和花,李桦,王慧,杨莉莉	ehua,Li Hua,Wang Hui,Yan	四川生理科学杂志	n Journal of Physiological S	38	2	76-79	2016	-
4954	长春市食品中食源性致病	of foodborne pathogenic	静鸿,龚云伟,李月婷,武艳	ng,Gong Yunwei,Li Yueting	食品安全质量检测学报	f Food Safety and Quality I	7	1	27-32	2016	-
4955	院感染病房病原菌分布及耐	ion and drug resistance in	红梅,王和平,张交生,齐利峰	Wang Heping,Zhang Jiaosh	国际检验医学杂志	onal journal of laboratory	37	19	2702-2704,2707	2016	sn.1673-4130.2016.19.017
4956	耐药表型与耐药基因、血	drug-resistant genes and s	施开创,李凤梅,王海清,张珍	ang,Li Fengmei,Wang Haiq	动物医学进展	vances in Veterinary Medic	37	3	25-32	2016	sn.1007-5038.2016.03.006

4957	规模猪场环境中主要致病	teria in the environment o	维谊,夏炉明,徐峰,沈莉	eiyi,Xia Luming,Xu Feng,Sh	畜禽业	vestock and poultry indust	-	10	54-55	2016	sn.1008-0414.2016.10.031
4958	节沙门菌分离株毒力基	e analysis of Salmonella is	,赵建梅,曲志娜,黄秀梅,	Jianmei,Qu Zhina,Huang Xi	畜牧与兽医	Husbandry and Veterinary	48	9	103-107	2016	-
4959	滨地区鸡源沙门菌耐药性	of Salmonella originating f	鑫鑫,黄皓,艾显辉,王晓	,Huang Hao,Ai Xianhui,Wa	动物医学进展	vances in Veterinary Medic	37	3	131-133	2016	sn.1007-5038.2016.03.029
4960	人急性腹泻病例病原谱	s of Pathogen Spectrum in	黄建芳,黄海滨,度尧,马瑨	anfang,Huang Haibin,Du Ya	中国医药导报	China Medical Herald	13	20	60-63	2016	-
4961	食源性疾病病原微生物	of foodborne disease path	蔡彦秋,马智龙,董洪燕.	nqiu,Ma Zhilong,Dong Hor	现代预防医学	modern preventive medicin	43	17	3131-3133,3143	2016	-
4962	宰场三种食源性细菌的	n of three foodborne bacte	林,欧秀玲,蒲敬伟,李爱巧,	Xiuling,Pu Jingwei,Li Aiqiao	甘肃畜牧兽医	al Husbandry and Veterina	46	21	91-92	2016	n.1006-799X.2016.21.045
4963	安市感染性腹泻细菌病原	nce results of bacterial etic	张辉,王春娟,谢龙,魏晓光	ang Chunjuan,Xie Long,We	现代预防医学	modern preventive medicin	43	22	4200-4203,4207	2016	-
4964	武威市474例食源性疾病	vince474Foodborne diseas	让山,黄爱霞,马艳,满世	untain,Huang Aixia,Ma Yan	疾病预防控制通报	se Prevention and Control	31	1	82-83	2016	15/j.cnki.jbyfkztb.1507020
4965	15年白银市食源性疾病监	ce analysis of foodborne d	,马菊红,杨军鹏,张景辉,	Juhong,Yang Junpeng,Zhar	疾病预防控制通报	se Prevention and Control	31	5	58-60,63	2016	15/j.cnki.jbyfkztb.1605035
4966	~2014年食源性沙门菌监	odborne Salmonella survei	平宗,王小龙,罗水斌,李传	,Wang Xiaolong,Luo Shuibi	检验医学与临床	boratory medicine and clini	-	1	4-5	2016	sn.1672-9455.2016.01.002
4967	内酰胺类抗生素耐药相关	and identification of lacta	,王继彤,曾博江,王少华,	ong,Zeng Bojiang,Wang Sh	北京农学院学报	al of Beijing Agricultural C	31	4	71-75	2016	issn.1002-3186.2016.0415
4968	省余姚市主城区河水沙门	water in the main urban are	群,黄邵军,罗学辉,苗超,杨	Shaojun,Luo Xuehui,Miao	疾病监测	disease surveillance	31	12	1023-1027	2016	-
4969	外卖快餐盒饭卫生状况调	c Conditions of Takeaway	孙启明,何翠华.	Sun Qiming,He Cuihua.	海南医学	Hainan Medicine	27	12	2033-2034,2035	2016	sn.1003-6350.2016.12.049
4970	夫登堡沙门菌耐药性及分	epidemiological character	新,吕冰,钱海坤,严寒秋,	Bing,Qian Haikun,severe c	现代预防医学	modern preventive medicin	43	14	2497-2501	2016	-
4971	寒沙门氏菌食物中毒的	e of food poisoning caused	鑫,许风兰,梁宏章,谢海鑫	Fenglan,Liang Hongzhang,	医学动物防制	medical animal control	32	5	571-572,575	2016	0.7629/yxdwfz201605036
4972	宁夏食源性疾病监测结	dborne disease surveillanc	袁秀娟,马娟,刘翔.	an Xiujuan,Ma Juan,Liu Xia	宁夏医科大学学报	al of Ningxia Medical Univ	38	9	1082-1084	2016	sn1674-6309.2016.09.032
4973	感染性腹泻病原学及流行	y and epidemiological char	姚建香,束方燕,梁峰.	anxiang,Shu Fangyan,Liang	医学动物防制	medical animal control	32	3	325-327	2016	0.7629/yxdwfz201603029
4974	疆兵团食品中食源性致病	esults of foodborne pathog	王建平,罗建忠.	ang Jianping,Luo Jianzhon	现代预防医学	modern preventive medicin	43	12	2167-2170	2016	-
4975	河南省肠炎沙门菌耐药与	nd molecular typing of Sal	强,潘静静,苏佳,穆玉姣,黄	n Jingjing,Su Jia,Mu Yujiao,	中华预防医学杂志	se Journal of Preventive Me	50	3	261-265	2016	sn.0253-9624.2016.03.014
4976	感染性腹泻沙门菌的血清	stance analysis of Salmone	健宁,吴佳音,李舒宁,黄建	g,Wu Jiayin,Li Shuning,Hua	现代预防医学	modern preventive medicin	43	13	2435-2438,2452	2016	-
4977	伤寒沙门菌肠毒素基因序	nce characteristics of Salm	凤,张舟听,王忠发,付松维,	houting,Wang Zhongfa,Fu	中国卫生检验杂志	ese Journal of Health Inspe	26	12	1757-1760	2016	-
4978	婴幼儿腹泻病原微生物监	f pathogenic microorganis	毕小朵,郭妍妍.	Bi Xiaoduo,Guo Yanyan.	中国病原生物学杂志	ese Journal of Pathogen Bi	11	12	1118-1121	2016	10.13350/j.cjpb.161215
4979	寒沙门菌分子分型及耐药	resistance characteristics	谐,柏彩英,邓秋莲,袁彩虹,	iying,Deng Qiulian,Yuan Ca	国际检验医学杂志	onal journal of laboratory	37	12	1601-1603	2016	sn.1673-4130.2016.12.004
4980	门菌引起的食物中毒的	s of food poisoning caused	黎剑华,夏伟,卓菲,杨贵清	ua,Xia Wei,Zhuo Fei,Yang	当代医学	contemporary medicine	22	36	196-196	2016	sn.1009-4393.2016.36.134
4981	份食品中食源性致病菌监	nitoring results of foodbor	郭海艳,李春林.	Guo Haiyan,Li Chunlin.	青海医药杂志	Qinghai Medical Journal	46	7	71-72	2016	-
4982	区食品中细菌污染状况	rial contamination in food	慧,楼永良,王晓红,张素燕,	ngliang,Wang Xiaohong,Zh	中国卫生检验杂志	ese Journal of Health Inspe	26	15	2252-2253,2258	2016	-
4983	境污染引起甲型副伤寒	break Caused by Public	胡儒元;王恩;	Hu Ruyuan;Wang En;	现代预防医学	odern preventive medici	43	23	4260-4263	2016	-
4984	污染引起伤寒暴发疫情的	typhoid outbreak caused	何坤;徐泽海;顾华康;	Kun;Xu Zehai;Gu Huaka	职业卫生与应急救援	onal health and emergen	34	1	72-73	2016	sn.1007-1326.2016.01.025
4985	10-2011年一起甲型副伤寒	nvestigation and treatmen	晓和,阳波,吴强,阚飙,周燕	,Yang Bo,Wu Qiang,Kan Bia	中华流行病学杂志	inese Journal of Epidemiol	38	2	200-204	2017	sn.0254-6450.2017.02.013
4986	食生鲜果蔬病原微生物	microorganisms in ready-t	学飞,张军锋,郭大城,尚	hang Junfeng,Guo Dachen	河南预防医学杂志	n Journal of Preventive Me	28	5	325-328	2017	m.1006-8414.2017.05.002
4987	售生禽肉中沙门菌血清分	r typing of Salmonella in ra	霞,吴玲玲,邱正勇,张秀丽,	gling,Qiu Zhengyong,Zhang	中国食品卫生杂志	hinese Food Hygiene Journ	29	6	679-683	2017	.13590/j.cjfh.2017.06.009
4988	年漳州市食源性致病菌	n results of foodborne pat	凌,郭宝羡,姚海燕,张丽	Guo Baoxian,Yao Haiyan,Zh	中国卫生检验杂志	ese Journal of Health Inspe	27	24	3613-3615	2017	-
4989	年中国甲型副伤寒沙门菌	al spread of Salmonella pa	王嘉正.	Wang Jiazheng.	中华预防医学杂志	se Journal of Preventive Me	51	12	1141	2017	-
4990	区居民食源性疾病流行	of foodborne diseases am	梅,杜田,谢思柔,李玥,陈网	Tian,Xie Sirou,Li Yue,Chen	公共卫生与预防医学	Health and Preventive Me	28	1	118-119	2017	-
4991	门氏菌引起的食物中毒原	uses of food poisoning cau	萍,刘香娥,燕玲,昝永芳,杨	iang'e,Yanling,Zan Yongfan	大家健康（上旬版）	ryone is healthy (early edit	11	5	105-106	2017	-
4992	丽江市食品中食源性致病	esults of foodborne pathog	杨晓忠,和丽毅,杨瑞娟.	Xiaozhong,He Liyi,Yang Ru	食品安全质量检测学报	f Food Safety and Quality I	8	10	3846-3849	2017	sn.2095-0381.2017.10.027
4993	5年咸宁市食源性致病菌	ults of foodborne pathogen	候少华,安锋涛,张莉,李静	aohua,An Fengtao,Zhang Li	职业与健康	Occupation and health	33	9	1193-1196	2017	-

4994	2016年安康市食物中毒检	food poisoning detection i	刘万静,刘斌,李湘平.	Wanjing,Liu Bin,Li Xiangpi	食品安全质量检测学报	f Food Safety and Quality I	8	11	4496-4499	2017	sn.2095-0381.2017.11.070
4995	鸡场鸡源沙门氏菌耐药性	enes of Salmonella origina	关茹飞,江萍,高超,夏利宁	Rufei,Jiang Ping,superb,Xia	中国农业科技导报	ultural Science and Techno	19	10	28-35	2017	13304/j.nykjdb.2017.0064
4996	湛江市出口罗非鱼微生物	ial contamination of tilapi	,聂芳红,孙良娟,雷晓凌,孙	anghong,Sun Liangjuan,Lei	中国卫生检验杂志	ese Journal of Health Inspe	27	5	718-721,727	2017	-
4997	诊腹泻儿童沙门菌感染状况	drug resistance in children	冰,张新,钱海坤,贾蕾,严	g Xin,Qian Haikun,Jia Lei,se	现代预防医学	nodern preventive medicin	44	22	4091-4095	2017	-
4998	年败血症患儿病原菌与耐	enic bacteria and drug resi	蔡晓燕,王朝媛,谭薇.	aoyan,Wang Chaoyuan,Tan	中华医院感染学杂志	ournal of Hospital Infectiou	27	16	3791-3794	2017	.11816/cn.ni.2017-170255
4999	珠海市市售食品中病原菌	s of pathogenic bacteria in	艳梅,袁筱茜,董莉,魏泉德	ei,Yuan Xiaoqian,Dong Li,W	中国卫生检验杂志	ese Journal of Health Inspe	27	15	2158-2161	2017	-
5000	州市食源性疾病哨点医院	results of foodborne diseas	苗升浩,张娟,吴玲,薛诚.	ghao,Zhang Juan,Wu Ling,X	实用预防医学	ractical preventive medicin	24	3	352-354,封3	2017	sn.1006-3110.2017.03.028
5001	雷登沙门菌引起的食物中	Poisoning Caused by Salmo	袁飞.	Yuan Fei.	国际检验医学杂志	onal journal of laboratory	38	15	2062-2064	2017	sn.1673-4130.2017.15.017
5002	-2015年食品中食源性致病	nitoring and analysis of foo	陈敏,罗信昌,陈惠龙.	Min,Luo Xinchang,Chen Hu	海峡预防医学杂志	s Journal of Preventive Me	23	3	68-69	2017	-
5003	里利沙门菌耐药特征和分子	istics and molecular typing	艳梅,杨春晓,张丽荣,魏泉	ang Chunxiao,Zhang Lirong	中国热带医学	Chinese tropical medicine	17	9	862-866	2017	cnki.46-1064/r.2017.09.03
5004	市津南区食源性疾病哨点	ults of foodborne disease s	刘坚龄,赵文,聂小才.	Jianling,Zhao Wen,Nie Xiao	中国卫生检验杂志	ese Journal of Health Inspe	27	1	109-111	2017	-
5005	放牧与舍饲育肥牦牛食源性	tween free grazing and ho	立,刘少文,陈宇,钟金城,王	owen,Chen Yu,bell jincheng	食品科学	food science	38	17	66-71	2017	pkx1002-6630-201717012
5006	医院感染性腹泻常见病原	pathogenic bacteria in infe	,马凤龙,戚金荣,刘大伟,文	Fenglong,Qi Jinrong,Liu Dav	实用预防医学	ractical preventive medicin	24	1	65-67	2017	sn.1006-3110.2017.01.019
5007	脓性关节炎病原体及药物	nd drug sensitivity of hip se	冯彦华,王康,郝建宗,张文	Vang Kang,Hao Jianzong,Zh	国际生物医学工程杂志	nal journal of biomedical e	40	6	432-436	2017	sn.1673-4181.2017.06.006
5008	分地市养殖场鲜鸡蛋微生	ation of fresh eggs in farm	东,王增国,王小强,陈雅丽	Zengguo,Wang Xiaoqiang,	医学动物防制	medical animal control	33	7	709-713	2017	0.7629/yxdwfz201707001
5009	肠埃希氏菌和沙门氏菌的	of enteropathogenic Esche	涛,文明,马光强,王伟,胡兴	vilization,Ma Guangqiang,V	中国畜牧兽医	mal husbandry and veterina	44	12	3625-3633	2017	ki.1671-7236.2017.12.033
5010	安市儿童细菌性腹泻病原	ults of pathogenic bacteria	雷超,刘继锋,魏晓光,刘南	ao,Liu Jifeng,Wei Xiaoguan	现代预防医学	nodern preventive medicin	44	15	2877-2880	2017	-
5011	市市售食品中食源性致病	dborne pathogenic bacteri	告,张娟,吴玲,许静静,童晶	g Juan,Wu Ling,Xu Jingjing,	现代预防医学	nodern preventive medicin	44	9	1589-1592	2017	-
5012	分市售食品中沙门氏菌和	s contamination in some c	春艳,黄淑华,陈磊,许姣,文	an,Huang Shuhua,Chen Lei	医学动物防制	medical animal control	33	4	393-395	2017	0.7629/yxdwfz201704013
5013	年感染性腹泻监测结果分	l infectious diarrhea survei	云,袁永娟,朱建民,凌勤芳	an Yongjuan,Zhu Jianmin,L	中国卫生检验杂志	ese Journal of Health Inspe	27	3	400-403	2017	-
5014	致病菌的食源性疾病暴发认	xploration of causes when	黄琼,张冬生,周荃,王铁强	g Dongsheng,Zhou Quan,W	中国食品卫生杂志	hinese Food Hygiene Journ	29	1	100-104	2017	0.13590/j.cjfh.2017.01.023
5015	腹泻非伤寒沙门菌耐药监	typing of non-typhoid Salm	新峰,王蕾,徐贵永,李静,胡	ng,Wang Lei,Xu Guiyong,Li	中国人兽共患病学报	Chinese Journal of Zoonose	33	4	337-342	2017	sn.1002-2694.2017.04.009
5016	餐饮从业人员带菌状况调	olecular typing of bacteria	子华,乐银辉,刘洋,周厚德	hua,Le Yinhui,Liu Yang,Zho	现代预防医学	nodern preventive medicin	44	19	3492-3495	2017	-
5017	餐饮业现榨果汁卫生状况调	tatus of freshly squeezed ju	汪艳玲,赵怀荣.	Vang Yanling,Zhao Huairong	食品安全质量检测学报	f Food Safety and Quality I	8	5	1938-1941	2017	sn.2095-0381.2017.05.070
5018	年北京市房山区肠道门诊	outpatient clinic cases in F	李海娇,田竞,董瑞强,石磊	aijiao,Tian Jing,Dong Ruiqia	现代预防医学	nodern preventive medicin	44	7	1316-1318,1344	2017	-
5019	宁市食品中食源性致病菌	esults of foodborne pathog	夏燕平,陈器,汪勇,楚涛.	ping,Chen Qi,Wang Yong,C	现代预防医学	nodern preventive medicin	44	21	3871-3874	2017	-
5020	门氏菌及大肠杆菌混合感	ion of Salmonella and Esch	淑红,张华清,王京仁,彭敬	Zhang Huaqing,Wang Jingr	黑龙江畜牧兽医（下半月）	dry and Veterinary Medicin	-	5	100-102	2017	-
5021	源沙门氏菌的分离鉴定与	yping of Salmonella origina	过效民.	Guo Xiaomin.	河南畜牧兽医（综合版）	y and Veterinary Medicine	38	10	6-7	2017	-
5022	自北京口岸进出口食品的	nella isolates from import	薇,汪琦,赵晓娟,陈鑫,杨丽	Qi,Zhao Xiaojuan,Chen Xi	中国食品卫生杂志	hinese Food Hygiene Journ	29	6	653-657	2017	0.13590/j.cjfh.2017.06.004
5023	肉制品生产环节致病菌检	eria in the production proc	兵兵,刘靓,刘纯成,邢亚东	,Liu Liang,Liu Chuncheng,X	中国卫生检验杂志	ese Journal of Health Inspe	27	9	1328-1330,1340	2017	-
5024	售整鸡沙门菌分离株耐药	anism of Salmonella isolate	杰,赫英英,白瑶,李凤琴,徐	He Yingying,Bai Yao,Li Feng	卫生研究	health research	46	4	538-545	2017	-
5025	院污水耐热大肠菌群监测与	is of heat-resistant colifor	茅国峰,梁美春,王清.	uofeng,Liang Meichun,Wan	中华医院管理杂志	se Hospital Management J	33	12	944-947	2017	sn.1000-6672.2017.12.016
5026	年上海市腹泻病综合监测	e surveillance results of di	昊寰宇,宫霄欢,肖文佳,胡家	,Gong Xiaohuan,Xiao Wen	中国公共卫生	Chinese public health	32	10	1507-1511	2017	847/zgggws2017-33-10-23
5027	412例感染性腹泻患者沙	lysis of salmonella infectio	思柔,莫浩联,张勇,赵凌国	Mo Haolian,Zhang Yong,Zh	中国热带医学	Chinese tropical medicine	17	7	691-693	2017	cnki.46-1064/r.2017.07.12
5028	沙门菌耐药基因、毒力基	e genes of Salmonella in re	文燕,黄秀梅,曲志娜,王玉	nyan,Huang Xiumei,Qu Zhi	中国家禽	Chinese poultry	39	22	76-80	2017	sn.1004-6364.2017.22.019
5029	圈养野生动物几种脏器带	ying conditions in several c	,王才益,陈玎玎,龚利洋,卢	g Caiyi,Chen Dingding,Gon	野生动物学报	Journal of Wildlife	38	3	360-366	2017	-
5030	京市门头沟区肠道门诊腹泻	in intestinal clinics in Men	李龙建,吕秋艳,刘英.	Longjian,Lu Qiuyan,Liu Yin	热带医学杂志	ournal of tropical medicine	17	9	1251-1253,1278	2017	sn.1672-3619.2017.09.033

5031	肉储存与销售环节食源性	cteria in the storage and sa	任皓,李爱巧,杨泽林,史茜	ao,Li Aiqiao,Yang Zelin,Shi	中国卫生检验杂志	ese Journal of Health Inspe	27	11	1618-1620	2017	-
5032	原市食品中食源性致病菌	odborne pathogenic bacter	张慧.	Zhang Hui.	食品安全质量检测学报	f Food Safety and Quality I	8	1	122-125	2017	sn.2095-0381.2017.01.020
5033	泻病原菌分布特点及耐药	esistance analysis of diarrh	永,张鞠玲,崔恩博,贾天野,	,Zhang Juling,Cui Eunbo,Jia	中国抗生素杂志	hinese Journal of Antibiotic	42	7	561-565	2017	sn.1001-8689.2017.07.004
5034	术在一起肠炎沙门氏菌食	for traceability analysis in	国忠,章剑,何政,徐超,张宏	hang Jian,He Zheng,Xu Cha	医学动物防制	medical animal control	33	12	1288-1289,1292	2017	0.7629/yxdwfz201712019
5035	年北京市大兴区食品中致病	c bacteria contamination in	潘峰,韩林,赵惠玲.	an Feng,Han Lin,Zhao Huilin	职业与健康	Occupation and health	33	10	1335-1338	2017	-
5036	沙门菌血清型分布及耐药	ution and drug resistance o	妍,邓娟,闰东辉,曹阳,闰梅	,Deng Juan,Run Donghui,C	临床和实验医学杂志	f Clinical and Experimental	16	3	299-302	2017	sn.1671-4695.2017.03.032
5037	包装糕点微生物污染状况	contamination status of pr	吴琼,宋安东.	Wu Qiong,Song Andong.	食品安全质量检测学报	f Food Safety and Quality I	8	1	345-349	2017	sn.2095-0381.2017.01.060
5038	售熟肉制品的微生物含量	robial content of cooked m	代艳梅,郑吉和.	Dai Yanmei,Zheng Jihe.	医药前沿	Medical Frontier	7	14	385-386	2017	sn.2095-1752.2017.14.342
5039	对多粘菌素的耐药现况及	non-typhoidal Salmonella i	晓杰,韩营营,李杰,阚飙,	aojie,Hanyingying,Li Jie,Kan	疾病监测	disease surveillance	32	5	365-371	2017	sn.1003-9961.2017.05.005
5040	6年云南省食源性致病菌	genic bacteria contaminati	敏,刘志涛,李娟娟,赵江,张	hitao,Li Juanjuan,Zhao Jian	职业与健康	Occupation and health	33	21	2943-2946	2017	-
5041	年河南省肠炎沙门菌耐药	tatus and molecular typing	白帆,穆玉姣,苏佳,黄学勇	ifan,Mu Yujiao,Su Jia,Huan	中华传染病杂志	se Journal of Infectious Dis	35	8	481-485	2017	sn.1000-6680.2017.08.007
5042	数儿童急性腹泻病的多中	acute diarrhea in children	张秀琴,朱忠生,黄永生,马	,Zhu Zhongsheng,Huang Yo	国际儿科学杂志	rnational journal of paedia	44	8	570-573	2017	sn.1673-4408.2017.08.015
5043	的HIV感染患者粪便标本病	etection and analysis of fe	郭静.	Guo Jing.	中国继续医学教育	a continuing medical educa	9	13	63-64	2017	sn.1674-9308.2017.13.032
5044	菌分离株生物被膜与耐药	film and drug resistance of	,周雪雁,王勇祥,邹文成,	n,Wang Yongxiang,Zou We	中国家禽	Chinese poultry	39	2	22-27	2017	sn.1004-6364.2017.02.005
5045	雷登沙门菌食物中毒的分	a case of food poisoning c	飞,谢成彬,张锡峰,王频佳	e Chengbin,Zhang Xifeng,W	现代预防医学	modern preventive medicin	44	19	3500-3503	2017	-
5046	门菌产超广谱β-内酰胺酶	xtended spectrumβ-Discus	厉小玉,刘荻,吴亦栋,胡雪	aoyu,Liu Di,Wu Yidong,Hu X	中国卫生检验杂志	ese Journal of Health Inspe	27	4	594-597	2017	-
5047	区儿童腹泻病原学检测及	nd drug resistance of childr	张秀香.	Zhang Xiuxiang.	中国保健营养	Chinese health nutrition	27	11	329	2017	sn.1004-7484.2017.11.494
5048	场所从业人员肠道沙门菌	onella carriage among wor	陈超,周渝蓉,黄平.	Chao,Zhou Yurong,Huang	预防医学情报杂志	of Preventive Medicine Info	33	9	894-896	2017	-
5049	鼠伤寒沙门菌血清型和分	aracteristics of food-borne	莹,吕素玲,杜悦,韦程媛,	u Suling,Du Yue,Wei Cheng	中国卫生检验杂志	ese Journal of Health Inspe	27	17	2445-2447,2451	2017	-
5050	门菌脉冲场凝胶电泳分型	d distribution characteristic	,王超,陈国利,巴特尔,吴	ng Chao,Chen Guoli,Battle,	医学动物防制	medical animal control	33	3	307-309	2017	0.7629/yxdwfz201703020
5051	门氏菌的毒力基因检测与	ug resistance analysis of Sa	娟,赵建梅,洪军,李月华,	Zhao Jianmei,Hong Jun,Li Y	中国动物检疫	China Animal Quarantine	34	5	40-46	2017	n.1005-944X.2017.05.011
5052	株洲市感染性腹泻细菌和	ults of bacterial and viral e	贺放晴,曹正安,罗誉皓.	ngqing,Cao Zhengan,Luo Y	河南预防医学杂志	n Journal of Preventive Me	28	9	691-692,封4	2017	m.1006-8414.2017.09.019
5053	年赤峰市食品中食源性致	alysis of foodborne pathog	曲桂娟,吴凤华.	Qu Guijuan,Wu Fenghua.	中国农村卫生	rural health in china	-	12	43-44	2017	n.1674-361X.2017.12.041
5054	15年感染性腹泻病原流行	miological characteristics o	佳,宫霄欢,庄源,匡小舟,	huan,Zhuang Yuan,Kuang	中华预防医学杂志	se Journal of Preventive Me	51	12	1113-1117	2017	sn.0253-9624.2017.12.012
5055	岸进口美国鸡肉粉连续检	cken meal imported from t	-	-	人人健康	Health for all	-	14	30	2017	-
5056	州市售食品细菌性污染情	l contamination of food so	段江丽,胡汝源,杨红菊.	Jiangli,Hu Ruyuan,Yang Ho	中国卫生检验杂志	ese Journal of Health Inspe	27	6	869-871	2017	-
5057	山市某区市售食品中致病	genic bacteria in commerci	通,区子阳,何瑜,陈卓仪,	u Ziyang,He Yu,Chen Zhuo	华南预防医学	uth China Preventive Medi	43	4	389-390,393	2017	.13217/j.scjpm.2017.0389
5058	畜禽产品中5种病原微生物	ducts5Monitoring and evalu	潘虎.	Pan Hu.	西藏农业科技	gricultural Science and Tec	39	3	34-37	2017	sn.1005-2925.2017.03.008
5059	密云地区腹泻病原菌耐药	f diarrhea pathogenic bact	于兆阳,张巍巍.	Yu Zhaoyang,Zhang Weiwei	中国卫生检验杂志	ese Journal of Health Inspe	27	5	751-753	2017	-
5060	细菌性腹泻病原的分离培养	tification of bacterial diarr	郝玺媛.	Hao Xiyuan.	畜牧兽医科技信息	d Veterinary Science and Te	-	9	126	2017	N.1671-6027.2017.09.113
5061	长沙门菌引起的食物中毒	a case of food poisoning ca	彭立昌,马骏.	Peng Lichang,Ma Jun.	中国食品卫生杂志	hinese Food Hygiene Journ	29	2	233-237	2017	.13590/j.cjfh.2017.02.025
5062	沙门氏菌的耐药性检测与	f drug resistance of Salmon	江洁,李焱,唐文彦,耿亚娟,	Yan,Tang Wenyan,Geng Ya	大连民族大学学报	of Dalian University for Nati	19	1	32-35	2017	n.1009-315X.2017.01.008
5063	州省仔猪腹泻的流行病掌	emic situation of piglet dia	双翔,周碧君,唐宇,何贤海,	xiang,Zhou Bijun,Tang Yu,H	黑龙江畜牧兽医（下半月）	dry and Veterinary Medicin	-	7	136-139	2017	-
5064	性腹泻患者粪便标本细菌	ibution in fecal samples of	任章银,陈敢敢,江舰.	hangyin,Chen Gangan,Jiang	检验医学与临床	boratory medicine and clini	14	10	1469-1472	2017	sn.1672-9455.2017.10.041
5065	流感染病原菌分布与耐药	istance characteristics of b	卢赞,尹利民,伏改芬.	Luzan,Yin Limin,Fu Gaifen.	国际检验医学杂志	onal journal of laboratory	38	10	1354-1357	2017	sn.1673-4130.2017.10.024
5066	源沙门氏菌的耐药性分析	tance of Salmonella from	周妮妮,韩敏敏,魏建忠,孙	u Nini,Han Minmin,Wei Jia	中国预防兽医学报	rnal of Preventive Veterina	39	7	544-549	2017	ssn.1008-0425.2017.07.07
5067	门菌血清型、耐药性及毒	stance and virulence genes	,刘红,齐文岚,王腾,林萍,	ng,Qi Wenlan,Wang Teng,L	热带医学杂志	journal of tropical medicine	17	1	11-14,28	2017	sn.1672-3619.2017.01.004

5068	同来源沙门菌耐药性及耐	ance spectrum of Salmone	莹春,霍细香,李文芳,刘准,	un,Huo Xixiang,Li Wenfang,	现代预防医学	modern preventive medicin	44	6	1011-1014,1022	2017	-
5069	郡市成华区食源性疾病监	ase surveillance results in	雅,李思齐,文艳群,文敏,许	Siqi,Wen Yanqun,Wen Mi	中国保健营养	Chinese health nutrition	27	20	287	2017	sn.1004-7484.2017.20.421
5070	次学生食物中毒的病因分	the cause of a student's foo	王秀琼.	Wang Xiuqiong.	中国保健营养	Chinese health nutrition	27	15	393	2017	sn.1004-7484.2017.15.637
5071	食品中致病微生物的调查	analysis of pathogenic mic	苏远科,王志宏,吕斐,罗晓	nke,Wang Zhihong,Lu Fei,Lu	口岸卫生控制	Port health control	22	1	53-54,52	2017	sn.1008-5777.2017.01.017
5072	年乐山市食源性致病菌监	results of foodborne path	刘珊,李俐漫,王加莉.	Liu Shan,Li Liman,Wang Jial	现代预防医学	modern preventive medicin	44	15	2723-2726	2017	-
5073	下婴幼儿沙门菌感染血清	of serotypes of Salmonella	,潘俊均,邓沛汶,庄云菁,	un,Deng Peiwen,Zhuang Y	国际检验医学杂志	onal journal of laboratory	38	11	1502-1504	2017	sn.1673-4130.2017.11.022
5074	源非伤寒沙门菌的分子分	nalysis of non-typhoid Salm	晓媛,王迪,崔霞,张诣,陈	i,Wang Di,Cui Xia,Zhang Yi	首都公共卫生	capital public health	11	4	150-154	2017	-
5075	生食物中毒的微生物学检	nination and analysis of bac	颖梅,谭海芳,林凤,苏乐	mei,Tan Haifang,Lin Feng,	疾病监测与控制	sease surveillance and cont	11	2	95-97	2017	-
5076	泻患者排泄物细菌培养及	ristics of fecal matter in pa	李叶静.	Li Yejing.	热带医学杂志	journal of tropical medicine	17	2	191-193	2017	sn.1672-3619.2017.02.014
5077	食品中食源性致病菌污染	tus of foodborne pathoger	张康.	Zhang Kang.	中国保健营养	Chinese health nutrition	27	4	307	2017	sn.1004-7484.2017.04.443
5078	年深圳市食源性疾病暴发流	analysis of foodborne dise	袁清连,李俊彦,黄薇,张锦	ian,Li Junyan,Huang Wei,Zh	公共卫生与预防医学	Health and Preventive Me	28	2	6-9	2017	-
5079	场鸡源沙门菌的分离鉴定	est of Salmonella originatin	邵伟娜,路振香,刘利晓,龚争	,Lu Zhenxiang,Liu Lixiao,G	动物医学进展	vances in Veterinary Medic	38	6	107-111	2017	-
5080	年湛江市出口虾微生物污染	s on Microbial Contaminat	雷晓凌,李红权,杨劲,刘骁	ling,Li Hongquan,Yang Jin,	中国卫生检验杂志	ese Journal of Health Inspe	27	14	2102-2105	2017	-
5081	广西焙烤食品食源性致病菌	tus of foodborne pathogen	婷,谢艺红,李秀桂,苏奕成,	ie Yihong,Li Xiugui,Su Yiche	现代预防医学	modern preventive medicin	44	16	2937-2939,2947	2017	-
5082	河南省食源性疾病沙门氏菌	illance Situation of Foodbo	艳芬,戚浩彧,张广伟,邱正勇	Haoyu,Zhang Guangwei,Q	中国人兽共患病学报	Chinese Journal of Zoonose	33	8	748-752	2017	sn.1002-2694.2017.08.017
5083	源沙门菌血清型鉴定及毒力	virulence genes of Salmon	晶晶,汤承,谭烁,冯帆,陈	,Tang Cheng,Tan Shuo,Fen	畜牧兽医学报	mal Husbandry and Veteri	48	11	2157-2165	2017	sn.0366-6964.2017.11.017
5084	术在肠道沙门氏菌快速检	he application of technolog	郭丹桂,许少洪.	Guo Dangui,Xu Shaohong.	医学动物防制	medical animal control	33	8	904-906	2017	0.7629/yxdwfz201708030
5085	12—2014年熟肉制品微生	nalysis of microbial testing	秦就成.	Qin Jiucheng.	医药前沿	Medical Frontier	7	16	351-352	2017	sn.2095-1752.2017.16.296
5086	永康市食品安全风险监测	safety risk monitoring res	吕春景,支红峰,石晓波.	unjing,Zhi Hongfeng,Shi Xi	中国卫生检验杂志	ese Journal of Health Inspe	27	16	2379-2380,2385	2017	-
5087	儿腹泻病原微生物检验结果	genic microorganisms in in	常桂丽.	Chang Guili.	临床医药文献电子杂志	edical literature electronic	4	74	14502-14503	2017	sn.2095-8242.2017.74.028
5088	和2016年餐饮具卫生指标	ysis of annual tableware hy	冯春艳,李建丽.	Feng Chunyan,Li Jianli.	食品安全导刊	food safety guide	-	18	145-147	2017	sn.1674-0270.2017.18.124
5089	氏菌对喹诺酮类药物的耐	of Salmonella originating f	保伟,吴浩天,王威,张亚南	i,Wu Haotian,Wang Wei,Z	食品科学	food science	38	4	107-112	2017	pkx1002-6630-201704018
5090	在冷冻禽畜肉类沙门氏菌	echnology in rapid salmone	菲菲,戴赛飞,刘子音,卓一	ong,Dai Saifei,Liu Ziyin,Zhu	食品安全导刊	food safety guide	-	9	87-88	2017	sn.1674-0270.2017.09.062
5091	沙门菌致感染性腹主动脉	rtic aneurysm caused by Sa	心洁,林冬群,王侃,郑远,林	n Dongqun,Wang Kan,Zhe	安徽医药	Anhui Medicine	21	7	1279-1280	2017	sn.1009-6469.2017.07.030
5092	家医院感染性腹泻来源非	nce of non-typhoid Salmo	梅英,崔志刚,黎剑华,王	eiying,Cui Zhigang,Li Jianhu	中华预防医学杂志	se Journal of Preventive Me	51	12	1118-1121	2017	sn.0253-9624.2017.12.013
5093	安全风险监测样品微生物	ical test results of food saf	敏,丁启能,韩英,段云权,王	heng,Han Ying,Duan Yunqu	河南预防医学杂志	n Journal of Preventive Me	28	11	878-881	2017	m.1006-8414.2017.11.023
5094	快速检测方法的建立及其	lmonella Enteritidis from c	军,许保疆,翟崇凯,郁川,王	Baojiang,Zhai Chongkai,Ika	河南农业科学	Henan Agricultural Sciences	46	10	137-142	2017	ki.1004-3268.2017.10.025
5095	肉类食品中沙门菌污染状	nella contamination in mea	辽朴,张小然,牛世文,周晓	hang Xiaoran,Niu Shiwen,Z	河南预防医学杂志	n Journal of Preventive Me	28	10	771-772,778	2017	m.1006-8414.2017.10.015
5096	16年杭州市医院污水监测	age monitoring results of H	曾文芳,岑斌,胡薇薇,沈寅	nfang,Cen Bin,Hu Weiwei,S	中国农村卫生事业管理	na's rural health managem	37	10	1208-1209	2017	sn.1005-5916.2017.10.024
5097	2002-2014年食源性疾病监	4Annual Foodborne Diseas	张静,张秉慧.	Zhang Jing,Zhang Binghui.	实用预防医学	ractical preventive medicin	24	4	486-488	2017	sn.1006-3110.2017.04.030
5098	者的感染病原菌分布及其	ance of infectious pathoge	罗云飞.	Luo Yunfei.	中国保健营养	Chinese health nutrition	27	21	290	2017	sn.1004-7484.2017.21.449
5099	株洲市食源性致病菌污染	tatus of foodborne pathog	艳,罗誉皓,胡卓,方英,刘育	uo Yuhao,Hu Zhuo,Fang Yi	河南预防医学杂志	n Journal of Preventive Me	28	10	769-770	2017	m.1006-8414.2017.10.014
5100	业人员预防性健康检查沙	of preventive health exami	芬,邱峰,曾国,杨智旭,廖	Feng,Zeng Guo,Yang Zhixu	心电图杂志（电子版）	diogram Magazine (electro	6	2	229-230	2017	-
5101	禽源沙门氏菌分离鉴定与	nce research of Salmonella	丹俊,潘孝成,赵瑞宏,戴	un,Pan Xiaocheng,Zhao Rui	中国畜牧兽医	mal husbandry and veterin	44	12	3663-3669	2017	ki.1671-7236.2017.12.038
5102	州市食源性疾病暴发事件	nce data on foodborne dise	王黎荔,高四海,山若青,蔡	Gao Sihai,Mountains are gr	卫生研究	health research	46	5	837-840	2017	-
5103	门菌引起的食源性疾病暴	oodborne illness outbreak	陈慧中,常春祥.	en Huizhong,Chang Chunxia	基层医学论坛	Primary Medicine Forum	21	17	2274-2276	2017	/j.1672-1721.2017.17.078
5104	袭性沙门菌属感染23例临	fections in adultstwenty thr	郭益群,王鹏,杨春霞,谷丽	un,Wang Peng,Yang Chunx	首都医科大学学报	nal of Capital Medical Unive	38	5	744-749	2017	sn.1006-7795.2017.05.021

5105	年自贡市食源性疾病主动	llance results of foodborne	刘桂丹,范正轩,谢飞.	Guidan,Fan Zhengxuan,Xie	职业与健康	Occupation and health	33	20	2792-2794,2799	2017	-
5106	猪源沙门菌血清型与耐药	of Salmonella originating f	,王伟芳,詹泽强,张红霞,	,Zhan Zeqiang,Zhang Hong	动物医学进展	vances in Veterinary Medic	38	2	32-36	2017	-
5107	狼疮继发沙门菌化脓性	osteomyelitis secondary to	陈家丽,何菁,安媛,陈适.	n Jiali,He Jing,Anyuan,Chen	中华风湿病学杂志	nese Journal of Rheumatol	21	1	53-55	2017	sn.1007-7480.2017.01.013
5108	门菌的血清分布类型和耐药	nd drug resistance status o	陈琴,张瑞中,郭少燕.	Qin,Zhang Ruizhong,Guo Sh	护理研究	nursing research	31	32	4170-4172	2017	sn.1009-6493.2017.32.040
5109	014年食源性致病菌监测	s of annual foodborne path	陈萍.	Chen Ping.	中国卫生标准管理	a Health Standard Manager	8	27	1-2	2017	sn.1674-9316.2017.27.001
5110	菌引起食物中毒的病原鉴	pathogen causing food po	瑞娥,姚素霞,张秋香,王	Yao Suxia,Zhang Qiuxiang,V	中国卫生检验杂志	ese Journal of Health Inspe	27	14	2023-2025	2017	-
5111	国家禽主要疫病的流行	tion and control strategies	尤永君.	You Yongjun.	中国禽业导刊	China Poultry Industry Guid	34	7	24-26	2017	-
5112	区羔羊腹泻主要病原菌的	genic bacteria causing dia	雪玲,美合日古丽·阿卜杜	n,Li Xueling,Meihe Riguli·A	新疆农垦科技	Agricultural Reclamation Te	40	8	27-29	2017	n.1001-361X.2017.08.011
5113	-2015年感染性腹泻病原	surveillance analysis of inf	金丹,孙传武.	golden elixir,Sun Chuanwu	中国初级卫生保健	primary health care in china	31	2	56-57,64	2017	.1001-568X.2017.02.0021
5114	年市售食品的食源性致病	pathogenic contaminatio	王君,韩蓓,陈小宁,任越.	un,Han Bei,Chen Xiaoning,F	中国热带医学	Chinese tropical medicine	17	10	991-996	2017	cnki.46-1064/r.2017.10.09
5115	食品中沙门菌污染与耐药	tamination and drug resist	周厚德,刘洋,姜茗,刘道峰	ude,Liu Yang,Jiang Ming,Liu	中国消毒学杂志	hinese Journal of Disinfecti	34	9	850-853	2017	sn.1001-7658.2017.09.017
5116	湖北省桶装饮用水微生物	icrobial Contamination of B	,刘艳,杨硕,郭鹏程,邵翠	Yang Shuo,Guo Pengcheng	公共卫生与预防医学	Health and Preventive Me	28	3	49-51	2017	-
5117	苗对抗菌药物及消毒剂	la vaccine derived from pig	凌丁,张业怀.	Ling Ding,Zhang Yehuai.	黑龙江畜牧兽医（下半月）	dry and Veterinary Medicin	-	9	109-112	2017	-
5118	人员腹泻病原体快速检测	on process for diarrhea pat	汝雯,史蕾,苏影,张小川,	n,Shi Lei,Su Ying,Zhang Xia	中国国境卫生检疫杂志	ontier Health and Quarantin	40	3	153-155,190	2017	/j.1004-9770.2017.03.001
5119	方法对肠道沙门菌的检测	s of Salmonella enterica be	刘志冰.	Liu Zhibing.	华夏医学	Chinese Medicine	30	3	106-108	2017	ki.1008-2409.2017-03-032
5120	进口牛奶微生物检验结果	ical test results of imported	超,周毅,程积进,王凤芝,	hou Yi,Cheng Jijin,Wang Fe	中国奶牛	Chinese cow	-	7	50-53	2017	nki.11-3009/s.2017.07.013
5121	院57例腹泻儿童沙门菌感	ical characteristics of Salm	刘伟东,蒋丰智.	Liu Weidong,Jiang Fengzhi.	牡丹江医学院学报	al of Mudanjiang Medical C	38	4	131-132,139	2017	-
5122	部分鸡场鸡沙门氏菌病流	chicken salmonellosis in so	,次仁多吉,拉巴次旦,曾江	ering Dorje,Raba Cidan,Zer	中国动物检疫	China Animal Quarantine	34	8	23-25	2017	n.1005-944X.2017.08.006
5123	左地区食品微生物安全风	microbial safety risk moni	农晓妮.	Nong Xiaoni.	大家健康（中旬版）	one is healthy (mid-term ed	11	1	51-52	2017	sn.1009-6019.2017.01.066
5124	装熟肉制品生产加工过	uction and processing of p	,李艳芬,邱正勇,炊慧霞,	nfen,Qiu Zhengyong,Cui H	卫生研究	health research	46	5	834-836	2017	-
5125	010-2015年食品中食源性	nual monitoring and analy	秋丽,谭志熹,付丽,陈若	Qiuli,Tan Zhixi,Fu Li,Chen Ru	实用预防医学	ractical preventive medicin	24	1	95-97	2017	sn.1006-3110.2017.01.030
5126	食品加工从业人员带菌状	atus among food processin	王乐,陈果,周良君.	ng Le,Chen Guo,Zhou Liang	中国卫生检验杂志	ese Journal of Health Inspe	27	17	2566-2568	2017	-
5127	链中沙门氏菌风险评估	nella risk assessment in ra	霞,杜建萍,张春艳,刘箐,董	anping,Zhang Chunyan,Liu	生物加工过程	bioprocessing	15	2	72-78	2017	sn.1672-3678.2017.02.012
5128	食物中毒的微生物学检	ination and analysis of bac	包浙宁.	Bao Zhening.	大家健康（上旬版）	ryone is healthy (early edit	11	12	77	2017	-
5129	部分地区猪沙门菌的耐药	Salmonella swine in some a	王豪男,张洪峰,常宇慧,郭	onan,Zhang Hongfeng,Cha	中国兽医学报	al of Chinese Veterinary Me	37	7	1283-1287	2017	nki.1005-4545.2017.07.14
5130	山市食品中食源性致病	sults of foodborne pathog	,王丽,刘本周,赵金燕,杨	u Zhouzhou,Zhao Jinyan,Ya	食品安全质量检测学报	f Food Safety and Quality I	8	10	3701-3709	2017	sn.2095-0381.2017.10.001
5131	门峡市食品致病菌监测	ng results of food pathoge	帅清,刘栋楠,王娜,卫成,	ing,Liu Dongnan,Wang Na,	实用预防医学	ractical preventive medicin	24	7	787-789	2017	sn.1006-3110.2017.07.006
5132	门菌引起食物中毒的病原	of food poisoning caused	卢波.	Lupo.	世界临床医学	world clinical medicine	11	13	233	2017	-
5133	015年伤寒、副伤寒流行	acteristics and bacterial typ	许国章,杨元斌,章丹阳,徐	ng,Yang Yuanbin,Zhang Da	中国卫生检验杂志	ese Journal of Health Inspe	27	7	1020-1024	2017	-
5134	沙门氏菌MLST和血清分	handong ProvinceMLSTan	源,李璐璐,刘玉洁,王永明	,Li Lulu,Liu Yujie,Wang Yo	中国人兽共患病学报	Chinese Journal of Zoonose	33	9	793-799	2017	sn.1002-2694.2017.09.008
5135	坡头区哨点医院食源性	dborne diseases in sentine	高敏贤,刘萍,段晓娟.	Minxian,Liu Ping,Duan Xiao	宁夏医学杂志	Ningxia Medical Journal	39	12	1231-1232	2017	j.1001-5949.2017.12.1231
5136	09-2014年甲型副伤寒疫情	demiological investigation	胡儒元,王恩.	Hu Ruyuan,Wang En.	中国学校卫生	Chinese school health	38	5	735-738,741	2017	ki.1000-9817.2017.05.027
5137	6年新疆食源性疾病主动	ctive surveillance of foodb	尔地,梁继红,艾依热提·	Turdi,Liang Jihong,Aiyireti·	疾病预防控制通报	se Prevention and Control	32	5	23-26	2017	15/j.cnki.jbyfkztb.1707012
5138	加工从业人员沙门菌带	r status among food proce	袁帅,朱维维,马锋.	an Shuai,Zhu Weiwei,Ma Fe	现代医药卫生	modern medicine and healt	33	18	2791-2792,2795	2017	sn.1009-5519.2017.18.018
5139	规模化猪场沙门氏菌分离	ntion of Salmonella in a larg	涛,王玉超,刘兆虎,孟凡	Wang Yuchao,Liu Zhaohu,N	中国饲料添加剂	China feed additives	0	7	26-31	2017	-
5140	-2016年腹泻症候群细菌	nalysis on the bacterial etic	于晓芳.	Yu Xiaofang.	中国卫生检验杂志	ese Journal of Health Inspe	27	22	3297-3298	2017	-
5141	氏菌病原学检测及药敏试	gen detection and drug sus	魏晓光,王增国,李芳.	iaoguang,Wang Zengguo,Li	医学动物防制	medical animal control	33	9	946-948	2017	0.7629/yxdwfz201709011

5142	人员沙门氏菌Real time PC	onella in workersReal time	罗卫红,曹刚,何靖.	o Weihong,Cao Gang,He Ji	河南预防医学杂志	n Journal of Preventive Me	28	6	432-434	2017	m.1006-8414.2017.06.009
5143	链中沙门氏菌污染源分	ination sources and hazard	宋德义.	Song Deyi.	上海畜牧兽医通讯	nal Husbandry and Veterina	-	3	50-52	2017	-
5144	病患者院内感染病原菌的	ocomial pathogenic bacte	晓丽,申娟,刘利君,马长	oli,Shen Juan,Liu Lijun,Ma	四川医学	Sichuan Medicine	38	12	1377-1380	2017	sn1004-0501-2017.12.006
5145	沙门氏菌的分离鉴定与药	ty test analysis of pathoge	召兴,庞洪泽,张文举,吉志	ing,Pang Hongze,Zhang We	河北科技师范学院学报	Science and Technology N	31	4	56-59	2017	N.1672-7983.2017.04.011
5146	东省沙门菌监测及其耐	e and drug resistance ana	挥,杨彤,梁宇恒,谭海玲,李	ong,Liang Yuheng,Tan Hai	中华微生物学和免疫学杂	rnal of Microbiology and I	37	8	611-617	2017	sn.0254-5101.2017.08.010
5147	市东丽区食源性疾病监测	orne disease surveillance e	张甜.	Zhang Tian.	中国城乡企业卫生	ygiene in urban and rural a	32	2	44-45	2017	/j.1003-5052.2017.02.017
5148	灌肠食品生产链中沙门菌	ation in an enema food pro	陈炳智,杨鹏飞,李双姝,刘	gzhi,Yang Pengfei,Li Shuan	现代预防医学	modern preventive medicin	44	24	4437-4441	2017	-
5149	民日常餐饮食品中食源性	orne pathogenic bacteria i	泽燕,徐励琴,杨剑英,冯伟	Xu Liqin,Yang Jianying,Feng	实用预防医学	ractical preventive medicin	24	7	776-779	2017	sn.1006-3110.2017.07.003
5150	沙门菌的耐药性分析及ES	f Salmonella strains derive	,齐亚如,韩文龙,韩雨奇,	Yaru,Han Wenlong,Han Yu	中国兽医杂志	Chinese Veterinary Journal	53	12	15-19,23	2017	-
5151	菌引发的食源性疾病暴发	odborne illness outbreaks	陈慧中,刘博.	Chen Huizhong,Liu Bo.	预防医学论坛	Preventive Medicine Forum	23	8	591-592,595	2017	-
5152	阜阳市食源性致病菌监测	esults of foodborne patho	侠,孟昭倩,李国兰,卜戈,	eng Zhaoqian,Li Guolan,Bu	中国卫生检验杂志	ese Journal of Health Inspe	27	23	3481-3483	2017	-
5153	人员体检沙门氏菌检测结	test results of physical exa	陈海平,冼桂江.	Chen Haiping,Xian Guijiang	大家健康（中旬版）	one is healthy (mid-term e	11	2	53-54	2017	sn.1009-6019.2017.02.064
5154	门菌PFGE分子分型及耐药	FGEMolecular typing and	,李美华,陈海滨,廖琳虹,	eihua,Chen Haibin,Liao Li	中华微生物学和免疫学杂	rnal of Microbiology and I	37	7	534-539	2017	sn.0254-5101.2017.07.010
5155	球菌和沙门菌的分离鉴	analysis of Staphylococcus	青,李博文,高彤,王豪举,丁	Bowen,Gao Tong,Wang H	中国兽医杂志	Chinese Veterinary Journal	53	3	82-85	2017	-
5156	阳市康平县感染性腹泻病	sults of infectious diarrhea	苗杨.	Miao Yang.	航空航天医学杂志	urnal of Aerospace Medici	28	1	108-110	2017	sn.2095-1434.2017.01.052
5157	酱卤肉制品生产加工过	oduction and processing o	艳,蒋君,朱昱锟,盛楠,林雪	g Jun,Zhu Yukun,Sheng Na	国际医药卫生导报	ational Medical and Health	23	9	1463-1465	2017	sn.1007-1245.2017.09.058
5158	广州市食品中食源性致病	results of foodborne path	爱,伍业健,陈惠玲,刘巧	i,Wu Yejian,Chen Huiling,L	中国卫生检验杂志	ese Journal of Health Inspe	27	11	1608-1609,1612	2017	-
5159	感染引起的食源性疾病暴	dborne disease outbreak c	洪佳冬,潘淑贤.	Hong Jiadong,Pan Shuxian.	华南预防医学	uth China Preventive Medi	43	6	556-559	2017	.13217/j.scjpm.2017.0556
5160	某市食源性致病菌污染状	g resistance of foodborne	磊,刘杰,张春艳,阎学燕,	iu Jie,Zhang Chunyan,Yan	华南预防医学	uth China Preventive Medi	43	4	377-380	2017	.13217/j.scjpm.2017.0377
5161	携带的动物性食品中化学	d pathogenic bacteria in a	琦,张妍,葛婷,阿热阿依·海	uo Jiaqi,Zhang Yan,Ge Ting	动物医学进展	vances in Veterinary Medi	38	10	51-55	2017	sn.1007-5038.2017.10.009
5162	顺义区947份感染性腹泻	sis of surveillance results o	朱美娟,李颖,王彦波.	Meijuan,Li Ying,Wang Yan	现代预防医学	modern preventive medicin	44	4	734-737,754	2017	-
5163	区食源性疾病病例监测结	sults of foodborne disease	刚雷,杨庆伟,邵钰莹,洪	i,Yang Qingwei,Shao Yuyin	中国卫生检验杂志	ese Journal of Health Inspe	27	19	2836-2839	2017	-
5164	东佛山高明区食品致病菌	contamination in Gaomin	,麦国通,黄志权,黄庆超,	g,Huang Zhiquan,Huang Q	公共卫生与预防医学	Health and Preventive Me	28	2	93-94	2017	-
5165	龙岩市即食散装熟肉制品	ysis of ready-to-eat bulk c	,金建潮,袁丹茅,张景平,	hao,Yuan Danmao,Zhang J	河南预防医学杂志	n Journal of Preventive Me	28	12	952-953	2017	m.1006-8414.2017.12.024
5166	布鲁菌病等3种人畜共患	area, etc.3Survey on the p	沈辰峰,马晓菁,叶锋,薛晶	eng,Ma Xiaojing,Ye Feng,X	畜牧与兽医	Husbandry and Veterinary	49	9	98-100	2017	-
5167	云南省食物中毒事件流行	ical characteristics of food	戚艳波,郑尔达,郑艳,杨静	o,Zheng Erda,Zheng Yan,Y	现代预防医学	modern preventive medicin	44	20	3691-3695	2017	-
5168	伊宁市11类食品致病菌监	11Analysis of monitoring s	,王小平,闻捷,古军梅,迪	ang Xiaoping,Wen Jie,Gu J	医学动物防制	medical animal control	33	4	462-464	2017	0.7629/yxdwfz201704037
5169	年食品中食源性致病菌监	l monitoring results of foo	杨蕙,周亚娟,王娅芳,朱殊	Zhou Yajuan,Wang Yafang	微量元素与健康研究	e elements and health rese	34	5	45-47	2017	-
5170	区食品中食源性致病菌污	dborne pathogenic bacteri	尤育杰,胡喜梅.	You Yujie,Hu Ximei.	中国校医	Chinese school doctor	31	7	493-494	2017	-
5171	猪血制品引起沙门菌食物	ella food poisoning cause	孔祥初,贾明生,苏冰.	Xiangchu,Jia Mingsheng,Su	中国公共卫生管理	ese public health manage	33	3	431-432,437	2017	cnki.23-1318.2017.03.045
5172	渝2012-2015年腹泻病原	gqing2012-2015Analysis o	黎黎,周晓芳,尹洁,尹建	ou Xiaofang,Yin Jie,Yin Jia	国际流行病学传染病学杂	nal of Epidemiology and I	44	5	292-295	2017	sn.1673-4149.2017.05.002
5173	杭州萧山国际机场航空食	od hygiene testing at Hang	魏莹,朱加进.	Wei Ying,Zhu Jiajin.	现代食品	modern food	-	18	17-21	2017	cn41-1434/ts.2017.18.005
5174	株洲市主要食源性致病菌	s of major foodborne path	刘育兰,钟艳,胡卓,方英.	an,Zhong Yan,Hu Zhuo,Fa	医药前沿	Medical Frontier	7	19	378-379	2017	sn.2095-1752.2017.19.329
5175	标本中沙门菌与志贺菌的	Salmonella and Shigella in	李世云,戴薇.	Li Shiyun,Dai Wei.	医疗装备	medical equipment	30	23	61-63	2017	sn.1002-2376.2017.23.033
5176	吉林市食品安全风险监测	od safety risk monitoring	孙寒松,王德宇.	Sun Hansong,Wang Deyu.	食品安全质量检测学报	f Food Safety and Quality I	8	1	111-115	2017	sn.2095-0381.2017.01.018
5177	染性腹泻216例临床及病	ea216Clinical and etiologi	朱勇,李丽,张跃新.	hu Yong,Li Li,Zhang Yuexi	世界华人消化杂志	ld Chinese Journal of Dige	25	2	190-193	2017	10.11569/wcjd.v25.i2.190
5178	建省部分腹泻患者沙门菌	ypes and drug resistance i	,黄梦颖,李曲文,徐海滨,	Mengying,Li Quwen,Xu Ha	预防医学论坛	Preventive Medicine Forum	23	7	489-491	2017	-

5179	品中5种食源性致病菌污染	on contamination status of	杜宇艇.	Du Yuping.	中国国境卫生检疫杂志	ntier Health and Quarantin	40	1	43-44,71	2017	/j.1004-9770.2017.01.010
5180	氏菌耐药基因检测方法应	ion methods for Salmonell	余超亚.	Yu Chaoya.	魅力中国	Charming China	-	20	298-299	2017	-
5181	亭湖区疾控中心致病菌检验	a testing at the Tinghu Dist	杨远锋.	Yang Yuanfeng.	河南预防医学杂志	n Journal of Preventive Me	28	11	887-889	2017	m.1006-8414.2017.11.025
5182	源性沙门氏菌耐药性及分	molecular typing of foodbo	李兵兵,李双姝,刘纯成,邢	ng,Li Shuangshu,Liu Chuncl	徐州医科大学学报	al of Xuzhou Medical Univ	37	10	664-667	2017	sn.1000-2065.2017.10.009
5183	菌感染后对微生物检验结果	ial infection of blood speci	廖德君.	Liao Dejun.	大家健康（上旬版）	ryone is healthy (early edit	11	7	16	2017	-
5184	沙门菌败血症并胸椎脓肿	onella typhi sepsis and th	刘雯,臧珂,杨晓艳,张国强	ng Ke,Yang Xiaoyan,Zhang	中华传染病杂志	se Journal of Infectious Dis	35	4	242	2017	sn.1000-6680.2017.04.014
5185	门菌血清型分布特征及耐	stribution characteristics a	马月佳,袁雕,汪华丽,王建瑛	an Diao,Wang Huali,Wang	中国卫生检验杂志	ese Journal of Health Inspe	27	11	1554-1556	2017	-
5186	泻便病原菌患者检验结果	esults of pathogenic bacter	吴祥铭,刘能江.	Vu Xiangming,Liu Nengjiang	影像研究与医学应用	research and medical appl	1	8	209-211	2017	sn.2096-3807.2017.08.138
5187	鸡肉中沙门菌污染调查和	of Salmonella contaminati	沈旭,遇晓杰,闫军,薛成玉,	Meet Xiaojie,Yan Jun,Xue Ch	中国公共卫生管理	ese public health managen	33	3	313-315	2017	cnki.23-1318.2017.03.007
5188	肺炎、马腺疫、马流产沙门	ine bubonic disease, and e	,马陈,赵亚南,马玉辉,朱义	hen,Zhao Yanan,Ma Yuhui,	中国预防兽医学报	rnal of Preventive Veterina	39	7	540-543	2017	ssn.1008-0425.2017.07.06
5189	性腹泻的病原分离及血清	fication of pathogenic bac	志,吴位珩,徐景峨,杨胜坤	u Weiheng,Xu Jing'e,Yang	龙江畜牧兽医（下半月）	dry and Veterinary Medicin	-	4	100-102	2017	-
5190	深圳市宝安区感染性腹泻	c bacteria in infectious diar	桂玲,李苑,钟景成,靳兴汉	ling,Li Yuan,Zhong Jingcher	医学动物防制	medical animal control	33	3	267-270,274	2017	0.7629/yxdwfz201703009
5191	见血清型科瓦利斯沙门菌	ng of rare serotype Salmor	慧霞,吴玲玲,邱正勇,张秀	Wu Lingling,Qiu Zhengyon	现代预防医学	modern preventive medicin	44	16	3044-3047	2017	-
5192	品和公共场所从业人员沙	detecting Salmonella in por	罗卫红,曹刚,田斌.	o Weihong,Cao Gang,Tian E	国际检验医学杂志	onal journal of laboratory	38	9	1273-1275	2017	sn.1673-4130.2017.09.048
5193	2009年-2015年食品监测结	-2015Analysis of annual fo	叶恒平.	Ye Hengping.	中国卫生检验杂志	ese Journal of Health Inspe	27	2	247-249	2017	-
5194	头雁和天鹅源沙门菌的耐	onella originating from gee	润洲,罗薇,杨怀珍,孔雪英	,Luo Wei,Yang Huaizhen,Ko	动物医学进展	vances in Veterinary Medic	38	9	49-54	2017	sn.1007-5038.2017.09.011
5195	市场鸡群沙门氏菌的分离鉴	alysis of Salmonella from c	,沈莉萍,王晓旭,徐锋,张维	Liping,Wang Xiaoxu,Xu Fen	上海畜牧兽医通讯	al Husbandry and Veterina	-	5	42-45	2017	-
5196	塔基沙门氏菌食物中毒事	Kentucky salmonella food	牛惠敏,杨玲,王艳芬.	Huimin,Yang Ling,Wang Yar	医学动物防制	medical animal control	33	8	888-889	2017	0.7629/yxdwfz201708024
5197	藏鸡沙门菌病流行情况调	prevalence of salmonellosi	,夏晨阳,吴金措姆,四朗玉	ia Chenyang,Wu Jintsomu,	中国兽医杂志	Chinese Veterinary Journal	53	12	49-50	2017	-
5198	家医疗机构污水消毒监测	sewage disinfection monito	黄云.	Huang Yun.	中国消毒学杂志	hinese Journal of Disinfecti	34	3	246-248	2017	sn.1001-7658.2017.03.016
5199	沙门菌的分离、鉴定及毒力	nce gene detection of Salm	,卢建洲,齐亚如,邓同炜,徐	anzhou,Qi Yaru,Deng Tongv	现代牧业	Modern animal husbandry	1	4	5-8	2017	sn.1008-3111.2017.04.002
5200	及市售禽肉样品中沙门菌	in human food poisoning,	红,蔡文华,王燕,杨新英,郭	nhua,Wang Yan,Yang Xinyi	中国人兽共患病学报	Chinese Journal of Zoonose	33	7	637-641	2017	sn.1002-2694.2017.07.012
5201	腹泻病原菌分布及耐药性	g resistance of pathogenic	罗燕华.	Luo Yanhua.	医学检验与临床	edical Laboratory and Clini	28	3	32-33,22	2017	sn.1673-5013.2017.03.010
5202	源沙门氏菌耐药性及耐药基	g-resistant genes of Salmo	萍,关茹飞,夏利宁,林亚军	ng,Guan Rufei,Xia Lining,Li	中国畜牧兽医	al husbandry and veterina	44	3	896-903	2017	ki.1671-7236.2017.03.038
5203	布利丹沙门菌引起食物中毒	e of food poisoning caused	兴斌,李文珍,陈凯琦,叶招	n,Li Wenzhen,Chen Kaiqi,Y	海峡预防医学杂志	s Journal of Preventive Me	23	6	81-82	2017	-
5204	宰环节沙门氏菌血清型及	resistance testing in Shan	赵建梅,曲志娜,黄秀梅,王	anmei,Qu Zhina,Huang Xiu	中国人兽共患病学报	Chinese Journal of Zoonose	33	6	517-521	2017	sn.1002-2694.2017.06.009
5205	感染性腹泻患者临床特点	characteristics of patients v	邓建刚,李全瑞,罗艳丽,王晶	ang,Li Quanrui,Luo Yanli,W	中国医刊	Chinese Medical Journal	52	5	59-62	2017	sn.1008-1070.2017.05.019
5206	317例食源性疾病病例流行	demiological investigation o	蔡圆圆,高四海,李毅,章乐	Yuanyuan,Gao Sihai,Li Yi,Z	中国预防医学杂志	se Journal of Preventive Me	18	1	35-38	2017	/j.1009-6639.2017.01.009
5207	泻80例病原微生物和药敏	pathogenic microorganism	余长发.	Yu Changfa.	当代医学	contemporary medicine	23	11	113-114	2017	sn.1009-4393.2017.11.059
5208	产链中沙门菌耐药基因检	of Salmonella in the broile	娟,黄秀梅,王君玮,杨瑞梅	g Xiumei,Wang Junwei,Yan	中国兽医学报	l of Chinese Veterinary Me	37	3	479-484	2017	nki.1005-4545.2017.03.17
5209	葡萄球菌和 沙门氏菌快速	roducts andA new system	,封丽霞,冯晓,王红坤,李春	ixia,Feng Xiao,Wang Hong	食品安全导刊	food safety guide	-	34	72-74	2017	sn.1674-0270.2017.34.030
5210	南充市食品中食源性致病	is of foodborne pathogenic	李天蓉,张明洪,张艳,蒋君	,Zhang Minghong,Zhang Ya	预防医学情报杂志	of Preventive Medicine Info	33	4	348-351	2017	-
5211	性腹泻的病原微生物检验	athogenic microorganisms	范明.	Fan Ming.	中外医学研究	se and foreign medical res	15	36	85-86	2017	3/j.cnki.cfmr.2017.36.044
5212	肠道细菌分离鉴定及药敏	susceptibility testing of inte	李国雄,刘芳,鲁红林,段纲	ong,Liu Fang,Lu Honglin,Du	中兽医医药杂志	raditional Chinese Veterina	36	6	52-54	2017	/j.cnki.jtcvm.2017.06.015
5213	市食品中食源性致病菌检	of foodborne pathogenic	刘彩春,梁廷基.	Liu Caichun,Liang Tingji.	中国卫生检验杂志	ese Journal of Health Inspe	27	21	3142-3144	2017	-
5214	南省食源性C群沙门菌耐药	e FoodborneCSalmonella	贾华云,王岚,甚志飞,张红	un,Wang Lan,Very Zhifei,Zh	实用预防医学	ractical preventive medicin	24	11	1281-1284	2017	sn.1006-3110.2017.11.001
5215	腹泻的临床特征及流行病	emiological investigation o	梁玉彩,周瑞清,孙文琴.	Yucai,Zhou Ruiqing,Sun We	中国妇幼保健	ina maternal and child hea	32	14	3303-3305	2017	ssn.1001-4411.2017.14.75

5216	年北京市大兴区食源性疾	sease surveillance results i	赵惠玲,潘峰,韩林.	hao Huiling,Pan Feng,Han Li	职业与健康	Occupation and health	33	10	1332-1334,1338	2017	-
5217	发生急性腹泻的常见病原	athogenic microorganisms	丽娟,陆军,邹婷婷,刘庆	an,army,Zou Tingting,Liu Q	中外医学研究	ese and foreign medical res	15	25	64-65	2017	3/j.cnki.cfmr.2017.25.033
5218	源沙门氏菌的分离 鉴定及	in Shandong ProvinceIdent	王晓艺,李涛,吕新新,张敏	Xiaoyi,Li Tao,Lu Xinxin,Zhar	家禽科学	poultry science	-	4	51-53	2017	sn.1673-1085.2017.04.023
5219	新区市售水产品中食源性	in commercially available a	灵菲,章溢峰,胡卉,沈惠	fei,Zhang Yifeng,Hu Hui,Sh	卫生研究	health research	46	1	162-164	2017	-
5220	市食品中食源性致病菌监	s of foodborne pathogenic	,刘晶晶,吴玲玲,张秀丽,	gjing,Wu Lingling,Zhang Xi	河南预防医学杂志	n Journal of Preventive Me	28	11	882-886	2017	m.1006-8414.2017.11.024
5221	菌血清型、药敏性及部分	partial drug resistance ge	伟,肖英平,杨华,曹晨阳,杨	ingping,Yang Hua,Cao Cher	食品科学	food science	38	15	140-146	2017	pkx1002-6630-201715023
5222	市售熟肉制品微生物污染	s of commercially availabl	琴,魏明敏,李通情,张系	Mingmin,Li Tongqing,Zhar	医学动物防制	medical animal control	33	6	610-613	2017	0.7629/yxdwfz201706006
5223	沙门氏菌分离鉴定过程中	isolation and identificatio	秀敏,尹玮璐,蒋佳希,张彬	n,Yin Weilu,Jiang Jiaxi,Zhar	食品安全质量检测学报	f Food Safety and Quality I	8	4	1287-1292	2017	sn.2095-0381.2017.04.033
5224	感染性腹泻来源沙门菌耐	Salmonella originating fro	宏,吴劲松,郝红雨,蒋淑萍,	u Jinsong,Hao Hongyu,Jian	中国人兽共患病学报	Chinese Journal of Zoonose	33	10	897-902	2017	sn.1002-2694.2017.10.009
5225	沙门氏菌检测能力验证的	nella detection proficiency	君杰,胡同静,葛晴颖,朱振	,Hutong quiet,Ge Qingying,	养禽与禽病防治	and poultry disease preven	-	11	10-12	2017	-
5226	州区食源性疾病监测结果	ance results of foodborne d	王力.	Wang Li.	中国保健营养	Chinese health nutrition	27	29	151	2017	sn.1004-7484.2017.29.212
5227	售生禽肉沙门菌和弯曲杆	cter contamination of raw	慧霞,吴玲玲,李艳芬,张秀	a,Wu Lingling,Li Yanfen,Zh	中国家禽	Chinese poultry	39	11	77-80	2017	sn.1004-6364.2017.11.019
5228	都铁路辖区从业人员沙门	carriage among employees	王珍凤,冯红,徐原能,张静	eng,Feng Hong,Xu Yuaneng	重庆医学	Chongqing Medicine	46	16	2249-2250	2017	sn.1671-8348.2017.16.026
5229	年食品微生物致病因子风	oring research and applicat	晓,孙菊芳,李荣发,陈琼	Sun Jufang,Li Rongfa,Chen	食品安全质量检测学报	f Food Safety and Quality I	8	10	3858-3863	2017	sn.2095-0381.2017.10.030
5230	体检快速筛查沙门志贺氏	g for Salmonella and Shigel	朱素芬,谭翰清.	Zhu Sufen,Tan Hanqing.	国际检验医学杂志	onal journal of laboratory	38	23	3268-3269,3273	2017	sn.1673-4130.2017.23.018
5231	漯河市城区发生的食物中	se of food poisoning in urb	闫晓军.	Yan Xiaojun.	预防医学情报杂志	of Preventive Medicine Info	33	2	165-168	2017	-
5232	014年食源性致病因子监	nnual surveillance results	王宁红,程艳君.	ang Ninghong,Cheng Yanju	心理医生	Psychologist	23	7	250-251	2017	-
5233	省6类即食食品中食源性	tion of foodborne pathoge	誉,马梦婷,陈文,兰真,张立	engting,Chen Wen,Lan Zh	预防医学情报杂志	of Preventive Medicine Info	33	5	471-474	2017	-
5234	健康体检中沙门菌检验方	a detection methods in pre	韦宏珍.	Wei Hongzhen.	检验医学与临床	boratory medicine and clini	14	Z1	117-118	2017	sn.1672-9455.2017.25.053
5235	产品食源性致病菌污染的	borne Pathogenic Bacteria	柯永贵.	Ke Yonggui.	中国医药科学	Chinese Medical Sciences	7	19	180-182,193	2017	sn.2095-0616.2017.19.054
5236	中山市感染性腹泻病原学	results of infectious diarrh	王翠玲,王曼,李雷,师舞阳	iling,Wang Man,Li Lei,Shi W	现代预防医学	modern preventive medicin	44	16	3040-3043,3047	2017	-
5237	菜中沙门氏菌污染情况调	dy on Salmonella contami	朱金艳,李莉.	Zhu Jinyan,Li Li.	新农业	new agriculture	-	9	4-5	2017	sn.1002-4298.2017.09.001
5238	鼠伤寒沙门菌感染40例临	ium infection in children40	叶蓓,蒋茂莹,邵启民.	Bei,Jiang Maoying,Shao Qin	浙江中西医结合杂志	grated Traditional Chinese	27	7	611-613	2017	sn.1005-4561.2017.07.026
5239	株洲市食源性疾病散发状	situation of foodborne dis	胡卓,石文松,刘育兰,方英	,Shi Wensong,Liu Yulan,Fa	微量元素与健康研究	e elements and health rese	34	6	41-42	2017	-
5240	6年启东市食物中毒实验	sis of Qidong Food Poisoni	张海英.	Zhang Haiying.	中国卫生检验杂志	ese Journal of Health Inspe	27	11	1613-1615	2017	-
5241	食品加工从业人员食源性	e pathogenic bacteria amo	钟天辉,张辉,谭燕妮,郑玲	nhui,Zhang Hui,Tan Yanni,Z	预防医学情报杂志	of Preventive Medicine Info	33	3	285-288	2017	-
5242	年丽水市食源性致病菌监	g results of foodborne path	高,徐翠红,刘爱霞,林德,黄	,Xu Cuihong,Liu Aixia,Linde	中国卫生检验杂志	ese Journal of Health Inspe	27	20	3007-3009	2017	-
5243	贡纳沙门菌耐药情况和毒	lence genes of Salmonella	,刘晓霞,吴晓妹,张利娟,贾	oxia,Wu Xiaomei,Zhang Liju	中华传染病杂志	se Journal of Infectious Dis	35	11	693-698	2017	sn.1000-6680.2017.11.011
5244	年云南省肉类制品致病菌	bacteria contamination of	路,国译丹,汤晓召,邹颜秋	dan,Tang Xiaozhao,Zou Ya	食品安全质量检测学报	f Food Safety and Quality I	8	10	3893-3897	2017	sn.2095-0381.2017.10.037
5245	儿童产ESBLs沙门菌耐药性	ESBLsPreliminary study o	邵启民,赵仕勇,吴亦栋,厉	min,Zhao Shiyong,Wu Yido	中国卫生检验杂志	ese Journal of Health Inspe	27	1	141-144	2017	-
5246	与小儿感染性腹泻病原学	ip between value and etiol	何建军,杭亚萍.	He Jianjun,Hang Yaping.	临床医学研究与实践	al Medical Research and Pr	2	6	115121	2017	nki.2096-1413.201706060
5247	沙门菌检测结果分析及P	ella test results among em	陈明明,王芯,葛美霞.	Mingming,Wang Xin,Ge M	微量元素与健康研究	e elements and health rese	34	6	43-44,46	2017	-
5248	菌污染的煮花生中毒事故	ort on boiled peanut poiso	杨建新,郑广军.	ang Jianxin,Zheng Guangju	医学动物防制	medical animal control	33	9	1009-1011	2017	0.7629/yxdwfz201709033
5249	起肠炎沙门氏菌食物中	onella Enteritidis Food Poi	李文常.	Li Wenchang.	养生保健指南	Health and Wellness Guide	-	38	85201	2017	sn.1006-6845.2017.38.076
5250	炎沙门菌食物中毒事件的	of an incident of Salmonell	生,邱冠雄,邱秀青,潘玮,	u Guanxiong,Qiu Xiuqing,P	海峡预防医学杂志	s Journal of Preventive Me	23	5	90-92	2017	-
5251	肉与肉制品中食源性致病	c bacteria in commercially	爱霞,申艳琴,银菊香,吴	en Yanqin,Silver chrysanth	疾病预防控制通报	se Prevention and Control	32	6	41-44	2017	15/j.cnki.jbyfkztb.1709001
5252	海市187株沙门菌Ⅰ耐药性	of drug resistance and mol	艳梅,魏泉德,张丽荣,袁筱	i,Wei Quande,Zhang Liron	现代预防医学	modern preventive medicin	44	23	4407-4411	2017	-

5253	腹泻患儿的病原菌分布与	stribution and drug resista	阮静文,梅冠炫,史苗年.	gwen,Mei Guanxuan,Shi M	中国妇幼保健	ina maternal and child hea	32	11	2412-2414	2017	ssn.1001-4411.2017.11.49
5254	和屠宰加工过程沙门菌污	ion during pig breeding an	张丁,张秀丽,郭晓玲,刘杰,	Ding,Zhang Xiuli,Guo Xiaol	中国卫生检验杂志	ese Journal of Health Inspe	27	16	2373-2375	2017	-
5255	食用鹌鹑蛋食物中毒事件	se of food poisoning from	赖招霞,谢超,陈荣添.	haoxia,Xie Chao,Chen Rong	海峡预防医学杂志	s Journal of Preventive Me	23	5	92-93	2017	-
5256	肠道门诊大便细菌培养及	eptibility analysis in childre	廖炀,林凯,张泓,张婷.	ng,Lin Kai,Zhang Hong,Zhar	儿科药学杂志	ournal of Pediatric Pharma	23	2	39-42	2017	p.1672-108X.2017.02.013
5257	品安全省级监测点微生物	t results of national food sa	进,白莉,王伟,胡豫杰,李业	Baili,Wang Wei,Hu Yujie,Li Y	卫生研究	health research	46	3	429-433	2017	-
5258	3260份血培养结果分析	Analysis of blood culture re	肖振清,幕博斌.	Xiao Zhenqing,Mu Bobin.	新疆医学	Xinjiang Medicine	47	2	176-177	2017	-
5259	的沙门氏菌耐药基因检测	ysis of drug resistance corr	黄秀梅,曲志娜,王君玮,杨玥	mei,Qu Zhina,Wang Junwei	中国动物检疫	China Animal Quarantine	34	1	35-39,42	2017	n.1005-944X.2017.01.010
5260	北京市怀柔区食源性疾病	veillance of foodborne dise	彬,闫雪,赵明星,郭浩,范义	ue,Zhao Mingxing,Guo Hao,	职业与健康	Occupation and health	33	18	2494-2496,2501	2017	-
5261	牦牛与黄牛源沙门氏菌的分	almonella originating from	春华,刘少文,谭子璇,陈宇,	a,Liu Shaowen,Tan Zixuan,	食品工业科技	Food Industry Technology	38	2	196-199,204	2017	sn1002-0306.2017.02.029
5262	浙江省宁海县感染性腹泻	n of infectious diarrhea su	章根华,洪因之,张峰,张丹	ua,Hong Yinzhi,Zhang Feng	中国卫生检验杂志	ese Journal of Health Inspe	27	21	3145-3147	2017	-
5263	汉市渍水水质变化过程监	lysis of Water Quality Chan	玲,何振宇,汪耀,熊燕,梁锦	enyu,Wang Yao,Xiong Yan,	卫生研究	health research	46	6	956-959	2017	-
5264	高校餐饮食品卫生状况调	d hygiene status of catering	吴琼.	Wu Qiong.	食品安全质量检测学报	f Food Safety and Quality I	8	9	3694-3697	2017	sn.2095-0381.2017.09.071
5265	肉供应链中沙门氏菌风险	Salmonella Risks in Pork S	唐瑞霞,马丽.	Tang Ruixia,Ma Li.	黑龙江畜牧兽医（下半月）	dry and Veterinary Medicin	-	2	91-92	2017	-
5266	市巴南区食源性致病菌检	of foodborne pathogenic b	黄雪梅,陈小玲.	Huang Xuemei,Chen Xiaolin	医学动物防制	medical animal control	33	7	798-800	2017	0.7629/yxdwfz201707030
5267	门氏菌分离鉴定及药物敏	ug sensitivity testing of Sal	杰,马弘财,王燕,夏晨阳,冯	Hongcai,Wang Yan,Xia Che	现代畜牧兽医	mal husbandry and veterin	-	6	41-44	2017	-
5268	沙门菌fosA3基因的流行	rcesfoA3Gene prevalence	文慧,张莉娟,杨玲,傅嘉莉	nhui,Zhang Lijuan,Yang Lin	中国兽医科学	Chinese Veterinary Science	47	4	514-522	2017	sn.1673-4696.2017.04.018
5269	市售水产品中食源性致病	tus of foodborne pathoge	,庄茂强,于连龙,宋柬,李明	g,Yu Lianlong,Song and Car	现代预防医学	modern preventive medicin	44	22	4069-4072	2017	-
5270	年-2016年食品中致病菌限	al survey and analysis of pa	慧萍,任三秋,刘瑞萍,王彦	Ren Sanqiu,Liu Ruiping,Wa	中国卫生检验杂志	ese Journal of Health Inspe	27	13	1952-1955	2017	-
5271	冷冻鱼糜制品中常见食源	pathogenic bacteria in com	,胡静,裴晓燕,杨大进,张	g,Pei Xiaoyan,Yang Dajin,Z	卫生研究	health research	46	2	328-330	2017	-
5272	规模化猪场沙门菌携带情	ella carriage in large-scale	陈龙,王好,崔冰冰,张贺亮	ang Hao,Cui Bingbing,Zhar	吉林畜牧兽医	l Husbandry and Veterinar	-	6	62,64	2017	-
5273	京城北哨点医院食源性疾	surveillance situation in N	丽,陆敏,徐东宁,赵虞虹,姚	Xu Dongning,Zhao Yuhong	中国食品卫生杂志	hinese Food Hygiene Journ	29	1	93-96	2017	).13590/j.cjfh.2017.01.021
5274	云南省学生餐细菌污染情	contamination of student	,杨萍,杨祖顺,高菊美,董坚	Ping,Yang Zushun,Gao Jum	中国食品卫生杂志	hinese Food Hygiene Journ	29	2	223-229	2017	).13590/j.cjfh.2017.02.023
5275	腹泻病例沙门菌血清型分	lla serotype distribution an	,杨春晓,张丽荣,袁筱茜,燕	unxiao,Zhang Lirong,Yuan X	华南预防医学	uth China Preventive Medi	43	5	474-477	2017	).13217/j.scjpm.2017.0474
5276	腔脓肿检出鼠伤寒沙门菌	murium detected in pelvic	永祥,崔晓琳,王咏梅,韩淑	ng,Cui Xiaolin,Wang Yongm	青岛大学医学院学报	f Qingdao University Medi	53	2	160	2017	13361/j.qdyxy.201702011
5277	患者粪便细菌培养及临床	e and clinical characteristic	黄杰之,韦柳春,马文斌.	g Jiezhi,Wei Liuchun,Ma W	数理医药学杂志	rnal of Mathematical Medi	30	5	685-686	2017	sn.1004-4337.2017.05.026
5278	性腹泻86例病原微生物检	dren86Analysis of pathoge	林高平,肖勍,邹立新.	Gaoping,Xiao Qing,Zou Lix	基层医学论坛	Primary Medicine Forum	21	8	963-964	2017	5/j.1672-1721.2017.08.049
5279	昌平区沙门菌临床菌株分	esistance of Salmonella cli	华,闻艳红,徐代庆,吴杨,庄	Yanhong,Xu Daiqing,Wu Ya	职业与健康	Occupation and health	33	12	1619-1622	2017	-
5280	区细菌性食源性疾病哨点	odborne disease sentinel h	江,肖贵勇,王佳佳,马晓曼	g,Xiao Guiyong,Wang Jiajia	职业与健康	Occupation and health	33	18	2502-2505	2017	-
5281	杭州地区甲型副伤寒沙门	ular typing of Salmonella P	皓秋,郑伟,俞骅,张蔚,潘劲	Zheng Wei,Yu Hua,Zhang W	中华微生物学和免疫学杂	rnal of Microbiology and I	37	1	57-61	2017	sn.0254-5101.2017.01.009
5282	沙门菌感染性主动脉瘤患	with aortic aneurysm infect	黄英男,潘珏,胡必杰.	ang Yingnan,Pan Jue,Hu Bi	中华医院感染学杂志	ournal of Hospital Infectiou	27	12	2718-2721	2017	.11816/cn.ni.2017-171126
5283	区部分鸡场沙门菌流行病	almonella in some chicken	张勇,程素平.	Zhang Yong,Cheng Suping.	中国禽业导刊	China Poultry Industry Guid	34	23	62-63	2017	-
5284	雏鸡鸡白痢沙门菌的分离	ection of Salmonella pullor	伟,康西,任志军,陈颖彬,刘	ei,Kangxi,Ren Zhijun,Chen Y	中国兽医杂志	Chinese Veterinary Journal	53	12	88-91	2017	-
5285	年6市县网络实验室送检菌	bacterial strains submitted	郭长红,景伟力.	Guo Changhong,Jing Weili.	海峡预防医学杂志	s Journal of Preventive Me	23	4	59-60	2017	-
5286	市13类市售食品微生物监	onitoring and analysis of si	曹啸.	Cao Xiao.	当代医学	contemporary medicine	23	34	28-30	2017	sn.1009-4393.2017.34.011
5287	肠炎沙门菌食物中毒事件	Salmonella Enteritidis food	林忠兰,吴兴美,吴光渠.	onglan,Wu Xingmei,Wu Gu	海峡预防医学杂志	s Journal of Preventive Me	23	2	89-91	2017	-
5288	细菌性腹泻病原菌及耐药	and analysis of bacterial d	汪鹏,康倩,张慧敏,于德山	g,Kang Qian,Zhang Huimin,	中国卫生检验杂志	ese Journal of Health Inspe	27	4	570-571	2017	-
5289	中国大陆食品的1070株沙	a food1070Analysis of dru	飞,甘辛,吕涵阳,刘畅,杨大	Ganxin,Lu Hanyang,Liu Cha	中国食品卫生杂志	hinese Food Hygiene Journ	29	6	647-652	2017	).13590/j.cjfh.2017.06.003

5290	1～2015年食品中食源性	al surveillance of foodborn	苟伟民,徐雪梅.	Gou Weimin,Xu Xuemei.	食品安全质量检测学报	f Food Safety and Quality I	8	1	126-130	2017	sn.2095-0381.2017.01.021
5291	院食源性疾病主动监测的	surveillance of foodborne	董和桂,高颖慧,闫立青.	g Hegui,Gao Yinghui,Yan Li	泰山医学院学报	rnal of Taishan Medical Col	38	1	44-45	2017	sn.1004-7115.2017.01.014
5292	氏菌污染引起食物中毒的	ase of food poisoning caus	胡鹏,唐开来.	Hu Peng,Tang Kailai.	医药前沿	Medical Frontier	7	16	229-230	2017	sn.2095-1752.2017.16.185
5293	市顺义区市售食品中食源	athogenic bacteria in comr	宛,吕金昌,李永进,刘秀峰	van,Lu Jinchang,Li Yongjin,	卫生研究	health research	46	1	159-161,164	2017	-
5294	015年学校食源性疾病时	nd Analysis of Foodborne I	杰,王岗,杨淑香,李向云,郭	ng,Yang Shuxiang,Li Xiangy	中国学校卫生	Chinese school health	38	1	82-85	2017	ki.1000-9817.2017.01.025
5295	哈瓦那沙门氏菌的耐药性	stance genes of Salmonell	,尹明远,田歌,马文瑞,王	Yin Mingyuan,Tian Ge,Ma	现代食品科技	Modern food technology	33	10	37-44	2017	fst.1673-9078.2017.10.006
5296	感染乙型副伤寒沙门氏菌	n an infant infected with S	陶墨奎,李强.	Tao Mokui,Li Qiang.	中国农村卫生	rural health in china	-	24	68	2017	n.1674-361X.2017.24.070
5297	病区域普查在肉鸭生产过	bacterial diseases in the p	伟,崔大鹏,朱继昌,李宝群,	Cui Dapeng,Zhu Jichang,Li	中国家禽	Chinese poultry	39	10	60-62	2017	sn.1004-6364.2017.10.015
5298	门氏菌引起的食物中毒的	is of a case of food poisoni	梁秀敏,罗晓菊,孙碧江.	g Xiumin,Luo Xiaoju,Sun Bij	中国保健营养	Chinese health nutrition	27	18	372	2017	sn.1004-7484.2017.18.578
5299	建省食品中沙门菌的分布	eristics of Salmonella in fo	伟伟,叶玲清,吴春敏,杨育	,Ye Lingqing,Wu Chunmin,	海峡预防医学杂志	s Journal of Preventive Me	23	2	65-67	2017	-
5300	食源性疾病暴发事件的调	report on a foodborne dise	王晓丽.	Wang Xiaoli.	中国城乡企业卫生	ygiene in urban and rural a	32	7	51-52	2017	5/j.1003-5052.2017.07.020
5301	业鲜榨果汁卫生质量调查	gienic Quality of Freshly Sq	周藜,张清,周倩,朱玫.	i,Zhang Qing,Zhou Qian,Zh	中国卫生检验杂志	ese Journal of Health Inspe	27	3	422-424	2017	-
5302	测方法在预防性体检肛拭	od of Salmonella in preven	郑少梅.	Zheng Shaomei.	中外医疗	Chinese and foreign medica	36	20	87-89	2017	ki.1674-0742.2017.20.087
5303	2016年食源性疾病病例监	nual Foodborne Disease Ca	苏玉.	Su Yu.	中国城乡企业卫生	ygiene in urban and rural a	32	8	149-150	2017	5/j.1003-5052.2017.08.064
5304	市昌平区感染性腹泻病原	ts of infectious diarrhea pa	彭华,徐代庆,李楠,高进玺	g Hua,Xu Daiqing,Li Nan,Ga	现代预防医学	modern preventive medicin	44	13	2444-2447,2452	2017	-
5305	生肉食品中致病菌污染状	cteria contamination in raw	斌,胡元玮,方琼楼,陈志	Yuanwei,Fang Qionglou,Ch	中国卫生检验杂志	ese Journal of Health Inspe	27	13	1931-1933	2017	-
5306	朱食物中毒沙门菌的耐药	e characteristics and mole	云,徐保红,王燕,杨新英,孙	ong,Wang Yan,Yang Xinyin	中国食品卫生杂志	hinese Food Hygiene Journ	29	5	539-543	2017	).13590/j.cjfh.2017.05.004
5307	副伤寒并无反应性结核病	A and unreactive tuberculo	梁紫茵,杨京.	Liang Ziyin,Yang Jing.	贵州医科大学学报	al of Guizhou Medical Univ	42	3	371-372	2017	ki.1000-2707.2017.03.029
5308	屠宰场分离沙门氏菌血清	sistance analysis of Salmon	怡,伍宏凯,方秋华,肖田安,	ongkai,Fang Qiuhua,Xiao Ti	中国兽药杂志	ese Journal of Veterinary D	51	7	17-22	2017	-
5309	感染引起的食源性疾病暴	rne illness outbreaks caus	朱海兰,徐智,徐然.	Zhu Hailan,Xu Zhi,Xu Ran.	预防医学论坛	Preventive Medicine Forum	23	5	398-399	2017	-
5310	腹泻的临床特征及流行病	and epidemiological studie	孟琨,岳英明,王蕾.	g Kun,Yue Yingming,Wang	中国临床保健杂志	se Journal of Clinical Healt	20	6	751-754	2017	sn.1672-6790.2017.06.036
5311	14年非伤寒沙门菌血清型	of serotype and molecular	真,杨红,茅俭英,朱倩如,陈	Hong,Mao Jianying,Zhu Qi	上海预防医学	hanghai Preventive Medici	29	8	622-624	2017	-
5312	性腹泻流行特征、病原及	pathogens and drug resista	李建明,陈莉,吴伟波,杨柳青	ing,Chen Li,Wu Weibo,Yan	中国实用医药	Chinese Practical Medicine	12	10	7-9	2017	nki.11-5547/r.2017.10.003
5313	2016年兰州市腹泻病例监	nd analysis of diarrhea case	,高金霞,李守禹,王龄庆,	Jinxia,Li Shouyu,Wang Ling	疾病预防控制通报	se Prevention and Control	32	5	20-22,70	2017	15/j.cnki.jbyfkztb.1706020
5314	2015年-2016年食源性疾病	Year-2016Analysis of annua	张峰,顾敏霞,章根华.	g Feng,Gu Minxia,Zhang Ge	中国卫生检验杂志	ese Journal of Health Inspe	27	15	2251-2253	2017	-
5315	年伤寒/副伤寒沙门菌耐药	search on drug resistance a	晨,嵇红,汪洋,鲍倡俊,霍	Hong,Wang Yang,Bao Chan	中华流行病学杂志	inese Journal of Epidemiol	38	11	1546-1550	2017	sn.0254-6450.2017.11.022
5316	016年滨州市水产品卫生监	u aquatic product hygiene	刘风仙,聂勇光,孙巧平.	gxian,Nie Yongguang,Sun Q	职业与健康	Occupation and health	33	23	3237-3239	2017	-
5317	金华市食源性疾病监测结	borne disease surveillance	王鸽,申屠平平,朱珈慧.	igeon,Shentu Pingping,Zhu	中国食品卫生杂志	hinese Food Hygiene Journ	29	1	97-100	2017	).13590/j.cjfh.2017.01.022
5318	肠杆菌和沙门氏菌的分离	ection of Escherichia coli a	杰,文娟,朱子凤,罗薇,杨	juan,Zhu Zifeng,Luo Wei,Y	黑龙江畜牧兽医（下半月）	dry and Veterinary Medicin	-	11	112-114,119	2017	-
5319	染4种人畜共患病原菌危险	stigation and analysis of ris	,蒲敬伟,徐敏,陈国利,陈	gwei,Xu Min,Chen Guoli,C	医学动物防制	medical animal control	33	5	542-544	2017	0.7629/yxdwfz201705023
5320	葫芦岛市区食源性致病菌	ults of foodborne pathogen	曹啸.	Cao Xiao.	国际检验医学杂志	onal journal of laboratory	38	21	3048-3050	2017	sn.1673-4130.2017.21.044
5321	小型猪主要病原微生物学	anisms and parasitology of	凡贵,吴瑞可,陈梅玲,王希	i,Wu Ruike,Chen Meiling,W	中国比较医学杂志	e Journal of Comparative M	27	10	69-73,79	2017	sn.1671-7856.2017.10.014
5322	市哨点医院食源性疾病监	ease surveillance results in	桂强,邢玉芳,宫春波,董峰	ng Yufang,Gong Chunbo,D	现代预防医学	modern preventive medicin	44	20	3798-3800,3812	2017	-
5323	入境口岸食品及饲料中沙	monella in food and feed a	薇,汪琦,赵晓娟,陈鑫,杨丽	Qi,Zhao Xiaojuan,Chen Xi	中国食品卫生杂志	hinese Food Hygiene Journ	29	5	534-538	2017	).13590/j.cjfh.2017.05.003
5324	10-2011年一起甲型副伤寒	nvestigation and treatmen	晓和,阳波,吴强,阚飙,周燕	,Yang Bo,Wu Qiang,Kan Bi	中华流行病学杂志	inese Journal of Epidemiol	38	2	200-204	2017	sn.0254-6450.2017.02.013
5325	即食生鲜果蔬病原微生物	microorganisms in ready-t	邵学飞,张军锋,郭大城,尚	Zhang Junfeng,Guo Dachen	河南预防医学杂志	n Journal of Preventive Me	28	5	325-328	2017	m.1006-8414.2017.05.002
5326	售生禽肉中沙门菌血清分	r typing of Salmonella in ra	霞,吴玲玲,邱正勇,张秀丽,	gling,Qiu Zhengyong,Zhang	中国食品卫生杂志	hinese Food Hygiene Journ	29	6	679-683	2017	).13590/j.cjfh.2017.06.009

5327	年漳州市食源性致病菌检	n results of foodborne pat	中凌,郭宝羡,姚海燕,张丽蓉	Guo Baoxian,Yao Haiyan,Zh	中国卫生检验杂志	ese Journal of Health Inspe	27	24	3613-3615	2017	-
5328	西渭南市食源性致病菌监测	of foodborne pathogenic b	张系忠,刘玮,郭欣,刘腾,王	hong,Liu Wei,Guo Xin,Liu Te	医学动物防制	medical animal control	33	10	1048-1050	2017	0.7629/yxdwfz201710010
5329	年中国甲型副伤寒沙门菌	nal spread of Salmonella pa	王嘉正.	Wang Jiazheng.	中华预防医学杂志	se Journal of Preventive Me	51	12	1141	2017	-
5330	社区居民食源性疾病流行病	s of foodborne diseases am	梅,杜田,谢思柔,李玥,陈网	u Tian,Xie Sirou,Li Yue,Chen	公共卫生与预防医学	Health and Preventive Me	28	1	118-119	2017	-
5331	门氏菌引起的食物中毒原	uses of food poisoning cau	萍,刘香娥,燕玲,昝永芳,杨	iang'e,Yanling,Zan Yongfan	大家健康（上旬版）	ryone is healthy (early edit	11	5	105-106	2017	-
5332	丽江市食品中食源性致病	esults of foodborne pathog	杨晓忠,和丽毅,杨瑞娟.	Xiaozhong,He Liyi,Yang Ru	食品安全质量检测学报	f Food Safety and Quality I	8	10	3846-3849	2017	sn.2095-0381.2017.10.027
5333	5年咸宁市食源性致病菌	ults of foodborne pathogen	候少华,安锋涛,张莉,李静	aohua,An Fengtao,Zhang Li	职业与健康	Occupation and health	33	9	1193-1196	2017	-
5334	广东省深圳市其他感染性	other infectious diarrhea i	媛,路滟,江敏,张海龙,梅树	Yan,Jiang Min,Zhang Hailo	疾病监测	disease surveillance	32	10	836-841	2017	003-9961.2017.10/11.013
5335	2016年安康市食物中毒检	food poisoning detection i	刘万静,刘斌,李湘平.	Wanjing,Liu Bin,Li Xiangpi	食品安全质量检测学报	f Food Safety and Quality I	8	11	4496-4499	2017	sn.2095-0381.2017.11.070
5336	鸡场鸡源沙门氏菌耐药性	enes of Salmonella origina	关茹飞,江萍,高超,夏利宁	Rufei,Jiang Ping,superb,Xia	中国农业科技导报	ultural Science and Techno	19	10	28-35	2017	13304/j.nykjdb.2017.0064
5337	湛江市出口罗非鱼微生物	ial contamination of tilapia	,聂芳红,孙良娟,雷晓凌,孙	anghong,Sun Liangjuan,Lei	中国卫生检验杂志	ese Journal of Health Inspe	27	5	718-721,727	2017	-
5338	诊腹泻儿童沙门菌感染状况	drug resistance in children	冰,张新,钱海坤,贾蕾,严	g Xin,Qian Haikun,Jia Lei,se	现代预防医学	modern preventive medicin	44	22	4091-4095	2017	-
5339	患者标本中沙门菌与志贺菌	almonella and Shigella in sp	雯,王若静,阎红,李娟,张传	Ruojing,Yan Hong,Li Juan,Zh	世界最新医学信息文摘	he world's latest medical in	-	64	-	2017	ki.1671-3141.2017.64.002
5340	年败血症患儿病原菌与耐	genic bacteria and drug resi	蔡晓燕,王朝媛,谭薇.	aoyan,Wang Chaoyuan,Tan	中华医院感染学杂志	ournal of Hospital Infectiou	27	16	3791-3794	2017	.11816/cn.ni.2017-170255
5341	珠海市市售食品中病原菌	s of pathogenic bacteria in	艳梅,袁筱茜,董莉,魏泉德	ei,Yuan Xiaoqian,Dong Li,W	中国卫生检验杂志	ese Journal of Health Inspe	27	15	2158-2161	2017	-
5342	州市食源性疾病哨点医院	results of foodborne diseas	苗升浩,张娟,吴玲,薛诚.	ghao,Zhang Juan,Wu Ling,X	实用预防医学	ractical preventive medicin	24	3	352-354,封3	2017	sn.1006-3110.2017.03.028
5343	雷登沙门菌引起的食物中	Poisoning Caused by Salmo	袁飞.	Yuan Fei.	国际检验医学杂志	onal journal of laboratory	38	15	2062-2064	2017	sn.1673-4130.2017.15.017
5344	特沙门菌引起化脓性脑膜	nster causes purulent men	余夏.	Yu Xia.	医学检验与临床	edical Laboratory and Clini	28	10	59-60	2017	sn.1673-5013.2017.10.020
5345	-2015年食品中食源性致病	nitoring and analysis of foo	陈敏,罗信昌,陈惠龙.	Min,Luo Xinchang,Chen Hu	海峡预防医学杂志	s Journal of Preventive Me	23	3	68-69	2017	-
5346	利沙门菌耐药特征和分子	istics and molecular typing	艳梅,杨春晓,张丽荣,魏泉	ang Chunxiao,Zhang Lirong	中国热带医学	Chinese tropical medicine	17	9	862-866	2017	cnki.46-1064/r.2017.09.03
5347	在从业人员伤寒痢疾快速	gy in rapid screening of typ	玉,高峰,李伟宏,贾海江,潘	,peak,Li Weihong,Jia Haijia	中国卫生产业	China health industry	14	19	64-65,90	2017	ki.1672-5654.2017.19.064
5348	市津南区食源性疾病哨点	ults of foodborne disease s	刘坚龄,赵文,聂小才.	Jianling,Zhao Wen,Nie Xiao	中国卫生检验杂志	ese Journal of Health Inspe	27	1	109-111	2017	-
5349	医院感染性腹泻常见病原	pathogenic bacteria in infe	江,马凤龙,戚金荣,刘大伟,文	Fenglong,Qi Jinrong,Liu Dav	实用预防医学	ractical preventive medicin	24	1	65-67	2017	sn.1006-3110.2017.01.019
5350	脓性关节炎病原体及药物	nd drug sensitivity of hip se	冯彦华,王康,郝建宗,张文	Vang Kang,Hao Jianzong,Zh	国际生物医学工程杂志	nal journal of biomedical e	40	6	432-436	2017	sn.1673-4181.2017.06.006
5351	安市儿童细菌性腹泻病原	ults of pathogenic bacteria	雷超,刘继锋,魏晓光,刘南	ao,Liu Jifeng,Wei Xiaoguan	现代预防医学	modern preventive medicin	44	15	2877-2880	2017	-
5352	市市售食品中食源性致病	dborne pathogenic bacteria	告,张娟,吴玲,许静静,童晶	g Juan,Wu Ling,Xu Jingjing,	现代预防医学	modern preventive medicin	44	9	1589-1592	2017	-
5353	分市售食品中沙门氏菌和	s contamination in some c	张春艳,黄淑华,陈磊,许姣,文	an,Huang Shuhua,Chen Lei	医学动物防制	medical animal control	33	4	393-395	2017	0.7629/yxdwfz201704013
5354	学生餐微生物污染状况监	microbial contamination in	何源,王红,刘晓朋,王文斟	ng Hong,Liu Xiaopeng,Wan	中国学校卫生	Chinese school health	38	3	430-432	2017	ki.1000-9817.2017.03.033
5355	本细菌感染后对微生物检验	rial infection of blood spec	孔祥慧.	Kong Xianghui.	中国保健营养	Chinese health nutrition	27	23	388-389	2017	sn.1004-7484.2017.23.560
5356	年感染性腹泻监测结果分	l infectious diarrhea survei	陈云,袁永娟,朱建民,凌勤为	an Yongjuan,Zhu Jianmin,L	中国卫生检验杂志	ese Journal of Health Inspe	27	3	400-403	2017	-
5357	腹泻非伤寒沙门菌耐药监	typing of non-typhoid Salm	新峰,王蕾,徐贵永,李静,胡	ng,Wang Lei,Xu Guiyong,Li	中国人兽共患病学报	Chinese Journal of Zoonose	33	4	337-342	2017	sn.1002-2694.2017.04.009
5358	餐饮从业人员带菌状况调	olecular typing of bacteria	张子华,乐银辉,刘洋,周厚德	hua,Le Yinhui,Liu Yang,Zho	现代预防医学	modern preventive medicin	44	19	3492-3495	2017	-
5359	年北京市房山区肠道门诊	outpatient clinic cases in F	李海娇,田竞,董瑞强,石磊	aijiao,Tian Jing,Dong Ruiqia	现代预防医学	modern preventive medicin	44	7	1316-1318,1344	2017	-
5360	宁市食品中食源性致病菌	esults of foodborne pathog	夏燕平,陈器,汪勇,楚涛.	ping,Chen Qi,Wang Yong,C	现代预防医学	modern preventive medicin	44	21	3871-3874	2017	-
5361	门氏菌及大肠杆菌混合感	ion of Salmonella and Esch	李淑红,张华清,王京仁,彭敬	Zhang Huaqing,Wang Jingr	黑龙江畜牧兽医（下半月）	dry and Veterinary Medicin	-	5	100-102	2017	-
5362	菌血清型快速PCR鉴定方法	apidPCREstablishment of id	方婷子,史贤明,施春雷.	Tingzi,Shi Xianming,Shi Ch	中国食品学报	urnal of Chinese Food Scier	17	2	212-219	2017	/j.1009-7848.2017.02.028
5363	源沙门氏菌的分离鉴定与	yping of Salmonella origina	过效民.	Guo Xiaomin.	河南畜牧兽医（综合版）	y and Veterinary Medicine	38	10	6-7	2017	-

5364	自北京口岸进出口食品的	onella isolates from import	薇,汪琦,赵晓娟,陈鑫,杨丽	Qi,Zhao Xiaojuan,Chen Xi	中国食品卫生杂志	hinese Food Hygiene Journ	29	6	653-657	2017	.13590/j.cjfh.2017.06.004
5365	肉制品生产环节致病菌检	eria in the production proc	兵兵,刘靓,刘纯成,邢亚东	,Liu Liang,Liu Chuncheng,X	中国卫生检验杂志	ese Journal of Health Inspe	27	9	1328-1330,1340	2017	-
5366	售整鸡沙门菌分离株耐药	anism of Salmonella isolate	杰,赫英英,白瑶,李凤琴,徐	e Yingying,Bai Yao,Li Feng	卫生研究	health research	46	4	538-545	2017	-
5367	人沙门菌的病原学特征及	molecular typing of Salm	丹,李泓,管红霞,冯微宏,肖	,Guan Hongxia,Feng Weiho	中国人兽共患病学报	Chinese Journal of Zoonose	33	4	378-381	2017	sn.1002-2694.2017.04.017
5368	年上海市腹泻病综合监测	e surveillance results of di	寰宇,宫霄欢,肖文佳,胡家	,Gong Xiaohuan,Xiao Wen	中国公共卫生	Chinese public health	32	10	1507-1511	2017	847/zgggws2017-33-10-23
5369	412例感染性腹泻患者沙	lysis of salmonella infectio	思柔,莫浩联,张勇,赵凌国	Mo Haolian,Zhang Yong,Zh	中国热带医学	Chinese tropical medicine	17	7	691-693	2017	cnki.46-1064/r.2017.07.12
5370	沙门菌耐药基因、毒力基	e genes of Salmonella in re	文燕,黄秀梅,曲志娜,王玉	nyan,Huang Xiumei,Qu Zhi	中国家禽	Chinese poultry	39	22	76-80	2017	sn.1004-6364.2017.22.019
5371	沙门菌分离鉴定与耐药性	ug resistance analysis of Sa	增海,徐耀辉,邓同炜,赵攀	Xu Yaohui,Deng Tongwei,Z	动物医学进展	vances in Veterinary Medic	38	11	121-125	2017	sn.1007-5038.2017.11.026
5372	京市门头沟区肠道门诊腹	in intestinal clinics in Men	李龙建,吕秋艳,刘英.	Longjian,Lu Qiuyan,Liu Yin	热带医学杂志	journal of tropical medicine	17	9	1251-1253,1278	2017	sn.1672-3619.2017.09.033
5373	肉储存与销售环节食源性	cteria in the storage and sa	任皓,李爱巧,杨泽林,史茜	ao,Li Aiqiao,Yang Zelin,Shi	中国卫生检验杂志	ese Journal of Health Inspe	27	11	1618-1620	2017	-
5374	京市食品中食源性致病菌	odborne pathogenic bacter	张慧.	Zhang Hui.	食品安全质量检测学报	f Food Safety and Quality I	8	1	122-125	2017	sn.2095-0381.2017.01.020
5375	泻病原菌分布特点及耐药	esistance analysis of diarrh	永,张鞠玲,崔恩博,贾天野,	,Zhang Juling,Cui Eunbo,Jia	中国抗生素杂志	hinese Journal of Antibiotic	42	7	561-565	2017	sn.1001-8689.2017.07.004
5376	在一起肠炎沙门氏菌食	for traceability analysis in	国忠,章剑,何政,徐超,张宏	hang Jian,He Zheng,Xu Cha	医学动物防制	medical animal control	33	12	1288-1289,1292	2017	0.7629/yxdwfz201712019
5377	北京市大兴区食品中致病	bacteria contamination in	潘峰,韩林,赵惠玲.	n Feng,Han Lin,Zhao Huilin	职业与健康	Occupation and health	33	10	1335-1338	2017	-
5378	沙门菌血清型分布及耐药	ution and drug resistance o	妍,邓娟,闰东辉,曹阳,闰梅	,Deng Juan,Run Donghui,C	临床和实验医学杂志	f Clinical and Experimental	16	3	299-302	2017	sn.1671-4695.2017.03.032
5379	加工过程中沙门氏菌污染	pe analysis during broiler c	兴广,王法云,朱海华,炊慧	ng,Wang Fayun,Zhu Haihu	河南预防医学杂志	n Journal of Preventive Me	28	9	718-720,封4	2017	m.1006-8414.2017.09.029
5380	包装糕点微生物污染状况	contamination status of p	吴琼,宋安东.	Wu Qiong,Song Andong.	食品安全质量检测学报	f Food Safety and Quality I	8	1	345-349	2017	sn.2095-0381.2017.01.060
5381	售熟肉制品的微生物含量	robial content of cooked m	代艳梅,郑吉和.	Dai Yanmei,Zheng Jihe.	医药前沿	Medical Frontier	7	14	385-386	2017	sn.2095-1752.2017.14.342
5382	对多粘菌素的耐药现况及	non-typhoidal Salmonella i	晓杰,韩营营,李杰,阚飙,	ojie,Hanyingying,Li Jie,Kan	疾病监测	disease surveillance	32	5	365-371	2017	sn.1003-9961.2017.05.005
5383	6年云南省食源性致病菌	genic bacteria contaminati	敏,刘志涛,李娟娟,赵江,张	hitao,Li Juanjuan,Zhao Jian	职业与健康	Occupation and health	33	21	2943-2946	2017	-
5384	年河南省肠炎沙门菌耐药	tatus and molecular typing	白帆,穆玉姣,苏佳,黄学勇	ifan,Mu Yujiao,Su Jia,Huan	中华传染病杂志	se Journal of Infectious Dis	35	8	481-485	2017	sn.1000-6680.2017.08.007
5385	儿童急性腹泻病的多中	acute diarrhea in children	张秀琴,朱忠生,黄永生,马	,Zhu Zhongsheng,Huang Y	国际儿科学杂志	rnational journal of paedia	44	8	570-573	2017	sn.1673-4408.2017.08.015
5386	的HIV感染患者粪便标本	etection and analysis of fe	郭静.	Guo Jing.	中国继续医学教育	a continuing medical educa	9	13	63-64	2017	sn.1674-9308.2017.13.032
5387	菌分离株生物被膜与耐药	film and drug resistance o	,周雪雁,王勇祥,邹文成,	n,Wang Yongxiang,Zou W	中国家禽	Chinese poultry	39	2	22-27	2017	sn.1004-6364.2017.02.005
5388	超市购物用具卫生状况调	nic status of shopping uter	芳,陈云,朱建民,袁永娟,胡	Yun,Zhu Jianmin,Yuan Yon	中国卫生检验杂志	ese Journal of Health Inspe	27	12	1797-1798,1802	2017	-
5389	登沙门菌食物中毒的分	a case of food poisoning c	飞,谢成彬,张锡峰,王频佳	e Chengbin,Zhang Xifeng,W	现代预防医学	modern preventive medicin	44	19	3500-3503	2017	-
5390	区儿童腹泻病原学检测及	nd drug resistance of childr	张秀香.	Zhang Xiuxiang.	中国保健营养	Chinese health nutrition	27	11	329	2017	sn.1004-7484.2017.11.494
5391	门菌产超广谱β-内酰胺酶	xtended spectrumβ-Discus	厉小玉,刘荻,吴亦栋,胡雪	aoyu,Liu Di,Wu Yidong,Hu X	中国卫生检验杂志	ese Journal of Health Inspe	27	4	594-597	2017	-
5392	染性腹泻细菌分布及耐药	n and drug resistance in ch	滑鑫,刘瑜,赵亚玲.	Hua Xin,Liu Yu,Zhao Yaling.	中国保健营养	Chinese health nutrition	27	20	376	2017	sn.1004-7484.2017.20.565
5393	场所从业人员肠道沙门菌	onella carriage among wor	陈超,周渝蓉,黄平.	Chao,Zhou Yurong,Huang	预防医学情报杂志	of Preventive Medicine Info	33	9	894-896	2017	-
5394	鼠伤寒沙门菌血清型和分	aracteristics of food-borne	莹,吕素玲,杜悦,韦程媛,	u Suling,Du Yue,Wei Cheng	中国卫生检验杂志	ese Journal of Health Inspe	27	17	2445-2447,2451	2017	-
5395	门氏菌的毒力基因检测与	ug resistance analysis of Sa	娟,赵建梅,洪军,李月华,	Zhao Jianmei,Hong Jun,Li Y	中国动物检疫	China Animal Quarantine	34	5	40-46	2017	n.1005-944X.2017.05.011
5396	门菌脉冲场凝胶电泳分型	d distribution characteristic	,王超,陈国利,巴特尔,吴亚	ng Chao,Chen Guoli,Battle,	医学动物防制	medical animal control	33	3	307-309	2017	0.7629/yxdwfz201703020
5397	株洲市感染性腹泻细菌和	ults of bacterial and viral e	贺放晴,曹正安,罗誉皓.	ngqing,Cao Zhengan,Luo Y	河南预防医学杂志	n Journal of Preventive Me	28	9	691-692,封4	2017	m.1006-8414.2017.09.019
5398	年赤峰市食品中食源性致	alysis of foodborne pathog	曲桂娟,吴凤华.	Qu Guijuan,Wu Fenghua.	中国农村卫生	rural health in china	-	12	43-44	2017	n.1674-361X.2017.12.041
5399	15年感染性腹泻病原流行	miological characteristics o	佳,宫霄欢,庄源,匡小舟,	huan,Zhuang Yuan,Kuang	中华预防医学杂志	se Journal of Preventive Me	51	12	1113-1117	2017	sn.0253-9624.2017.12.012
5400	山市某区市售食品中致病	genic bacteria in commerc	通,区子阳,何瑜,陈卓仪,	u Ziyang,He Yu,Chen Zhuo	华南预防医学	uth China Preventive Medic	43	4	389-390,393	2017	.13217/j.scjpm.2017.0389

5401	禽产品中5种病原微生物	ducts5Monitoring and evalu	潘虎.	Pan Hu.	西藏农业科技	gricultural Science and Tech	39	3	34-37	2017	sn.1005-2925.2017.03.008
5402	67株甲型副伤寒沙门菌分	ecular typing and drug resi	勇,张白帆,穆玉姣,苏佳,黄	g,Zhang Baifan,Mu Yujiao,S	中华预防医学杂志	se Journal of Preventive Me	51	10	881-885	2017	sn.0253-9624.2017.10.003
5403	门菌流行病学调查与耐药	drug resistance analysis o	,苏金辉,朱要宏,赵亚荣,王	nhui,Zhu Yaohong,Zhao Ya	中国兽医杂志	Chinese Veterinary Journal	53	10	17-19	2017	-
5404	细菌性腹泻病原的分离培养	tification of bacterial diarr	郝玺媛.	Hao Xiyuan.	畜牧兽医科技信息	d Veterinary Science and Te	-	9	126	2017	N.1671-6027.2017.09.113
5405	密云地区腹泻病原菌耐药	f diarrhea pathogenic bact	于兆阳,张巍巍.	Yu Zhaoyang,Zhang Weiwei	中国卫生检验杂志	ese Journal of Health Inspe	27	5	751-753	2017	-
5406	症状的老年都柏林沙门	elderly Dublin patient with	程燕,车炜.	Cheng Yan,Che Wei.	中国保健营养	Chinese health nutrition	27	21	151	2017	sn.1004-7484.2017.21.212
5407	沙门菌引起的食物中毒	case of food poisoning ca	彭立昌,马骏.	Peng Lichang,Ma Jun.	中国食品卫生杂志	hinese Food Hygiene Journ	29	2	233-237	2017	).13590/j.cjfh.2017.02.025
5408	沙门氏菌的耐药性检测与	drug resistance of Salmon	江洁,李焱,唐文彦,耿亚娟,	Yan,Tang Wenyan,Geng Ya	大连民族大学学报	of Dalian University for Nati	19	1	32-35	2017	n.1009-315X.2017.01.008
5409	鸡沙门氏菌的分离鉴定与	and prevention of Salmone	华玉新.	Hua Yuxin.	兽医导刊	veterinary guide	-	22	124-125	2017	-
5410	州省仔猪腹泻的流行病掌	emic situation of piglet dia	双翔,周碧君,唐宇,何贤海,	xiang,Zhou Bijun,Tang Yu,H	黑龙江畜牧兽医（下半月）	dry and Veterinary Medicin	-	7	136-139	2017	-
5411	性腹泻患者粪便标本细菌	ibution in fecal samples of	任章银,陈敢敢,江舰.	hangyin,Chen Gangan,Jiang	检验医学与临床	boratory medicine and clini	14	10	1469-1472	2017	sn.1672-9455.2017.10.041
5412	流感染病原菌分布与耐药	istance characteristics of b	卢赞,尹利民,伏改芬.	Luzan,Yin Limin,Fu Gaifen.	国际检验医学杂志	onal journal of laboratory r	38	10	1354-1357	2017	sn.1673-4130.2017.10.024
5413	源沙门氏菌的耐药性分析	tance of Salmonella from p	周妮妮,韩敏敏,魏建忠,孙	u Nini,Han Minmin,Wei Jia	中国预防兽医学报	rnal of Preventive Veterina	39	7	544-549	2017	ssn.1008-0425.2017.07.07
5414	门菌血清型、耐药性及毒	stance and virulence genes	,刘红,齐文岚,王腾,林萍,	ng,Qi Wenlan,Wang Teng,L	热带医学杂志	journal of tropical medicine	17	1	11-14,28	2017	sn.1672-3619.2017.01.004
5415	同来源沙门菌耐药性及耐	ance spectrum of Salmone	莹春,霍细香,李文芳,刘准,	un,Huo Xixiang,Li Wenfang,	现代预防医学	modern preventive medicin	44	6	1011-1014,1022	2017	-
5416	都市成华区食源性疾病监	ase surveillance results in	雅,李思齐,文艳群,文敏,许	Siqi,Wen Yanqun,Wen Min	中国保健营养	Chinese health nutrition	27	20	287	2017	sn.1004-7484.2017.20.421
5417	次学生食物中毒的病因分	the cause of a student's foo	王秀琼.	Wang Xiuqiong.	中国保健营养	Chinese health nutrition	27	15	393	2017	sn.1004-7484.2017.15.637
5418	食品中致病微生物的调查	analysis of pathogenic mic	苏远科,王志宏,吕斐,罗晓	nke,Wang Zhihong,Lu Fei,Lu	口岸卫生控制	Port health control	22	1	53-54,52	2017	sn.1008-5777.2017.01.017
5419	年乐山市食源性致病菌监	results of foodborne path	刘珊,李俐漫,王加莉.	Liu Shan,Li Liman,Wang Jial	现代预防医学	modern preventive medicin	44	15	2723-2726	2017	-
5420	下婴幼儿沙门菌感染血清	of serotypes of Salmonella	,潘俊均,邓沛汶,庄云菁,温	un,Deng Peiwen,Zhuang Yu	国际检验医学杂志	onal journal of laboratory r	38	11	1502-1504	2017	sn.1673-4130.2017.11.022
5421	源非伤寒沙门菌的分子分	alysis of non-typhoid Salm	晓媛,王迪,崔霞,张诣,陈	i,Wang Di,Cui Xia,Zhang Yi	首都公共卫生	capital public health	11	4	150-154	2017	-
5422	食品中食源性致病菌污染	tus of foodborne pathogen	张康.	Zhang Kang.	中国保健营养	Chinese health nutrition	27	4	307	2017	sn.1004-7484.2017.04.443
5423	深圳市食源性疾病暴发流	analysis of foodborne dise	袁清连,李俊彦,黄薇,张锦	ian,Li Junyan,Huang Wei,Zh	公共卫生与预防医学	Health and Preventive Me	28	2	6-9	2017	-
5424	沙门菌的分离、鉴定及生	haracteristics analysis of Sa	刘加圣.	Liu Jiasheng.	中国禽业导刊	China Poultry Industry Guid	34	24	56-57	2017	-
5425	场鸡源沙门菌的分离鉴定	est of Salmonella originati	邵伟娜,路振香,刘利晓,龚争	,Lu Zhenxiang,Liu Lixiao,Go	动物医学进展	vances in Veterinary Medic	38	6	107-111	2017	-
5426	湛江市出口虾微生物污染	s on Microbial Contaminat	雷晓凌,李红权,杨劲,刘骁,	ling,Li Hongquan,Yang Jin,L	中国卫生检验杂志	ese Journal of Health Inspe	27	14	2102-2105	2017	-
5427	西焙烤食品食源性致病菌	tus of foodborne pathogen	婷,谢艺红,李秀桂,苏奕成,	ie Yihong,Li Xiugui,Su Yiche	现代预防医学	modern preventive medicin	44	16	2937-2939,2947	2017	-
5428	南省食源性疾病沙门氏菌	illance Situation of Foodbo	艳芬,戚浩彧,张广伟,邱正勇	Haoyu,Zhang Guangwei,Qi	中国人兽共患病学报	Chinese Journal of Zoonose	33	8	748-752	2017	sn.1002-2694.2017.08.017
5429	源沙门菌血清型鉴定及毒力	virulence genes of Salmon	晶晶,汤承,谭烁,冯帆,陈	,Tang Cheng,Tan Shuo,Fen	畜牧兽医学报	mal Husbandry and Veteri	48	11	2157-2165	2017	sn.0366-6964.2017.11.017
5430	某市沙门菌监测及其病原	f its etiological characteris	郑悦康,区金结.	Zheng Yuekang,Ou Jinjie.	中国当代医药	inese contemporary medic	24	22	148-150,162	2017	sn.1674-4721.2017.22.049
5431	12—2014年熟肉制品微生	alysis of microbial testing	秦就成.	Qin Jiucheng.	医药前沿	Medical Frontier	7	16	351-352	2017	sn.2095-1752.2017.16.296
5432	康市食品安全风险监测	safety risk monitoring res	吕春景,支红峰,石晓波.	unjing,Zhi Hongfeng,Shi Xi	中国卫生检验杂志	ese Journal of Health Inspe	27	16	2379-2380,2385	2017	-
5433	腹泻病原微生物检验结果	genic microorganisms in in	常桂丽.	Chang Guili.	临床医药文献电子杂志	edical literature electronic	4	74	14502-14503	2017	sn.2095-8242.2017.74.028
5434	氏菌对喹诺酮类药物的耐	of Salmonella originating f	保伟,吴浩天,王威,张亚南	i,Wu Haotian,Wang Wei,Z	食品科学	food science	38	4	107-112	2017	pkx1002-6630-201704018
5435	沙门菌致感染性腹主动脉	rtic aneurysm caused by Sa	心洁,林冬群,王侃,郑远,林	n Dongqun,Wang Kan,Zhe	安徽医药	Anhui Medicine	21	7	1279-1280	2017	sn.1009-6469.2017.07.030
5436	家医院感染性腹泻来源非	nce of non-typhoid Salmo	梅英,崔志刚,黎剑华,王	eiying,Cui Zhigang,Li Jianhu	中华预防医学杂志	se Journal of Preventive Me	51	12	1118-1121	2017	sn.0253-9624.2017.12.013
5437	流行病学及对喹诺酮耐药	miology of Salmonella infe	顾兵,秦婷婷.	Gu Bing,Qin Tingting.	传染病信息	fectious disease informati	30	5	282-285	2017	sn.1007-8134.2017.05.009

5438	全风险监测样品微生物检	ical test results of food safe	敏,丁启能,韩英,段云权,王	heng,Han Ying,Duan Yunqu	河南预防医学杂志	n Journal of Preventive Me	28	11	878-881	2017	m.1006-8414.2017.11.023
5439	快速检测方法的建立及其	lmonella Enteritidis from c	军,许保疆,翟崇凯,郁川,王	Baojiang,Zhai Chongkai,Ika	河南农业科学	Henan Agricultural Sciences	46	10	137-142	2017	ki.1004-3268.2017.10.025
5440	肉类食品中沙门菌污染状	ella contamination in mea	辽朴,张小然,牛世文,周晓	hang Xiaoran,Niu Shiwen,Zh	河南预防医学杂志	n Journal of Preventive Me	28	10	771-772,778	2017	m.1006-8414.2017.10.015
5441	门氏菌耐药性和耐药基因	drug-resistant genes of Sa	萍,关茹飞,夏利宁,徐志勇	g,Guan Rufei,Xia Lining,Xu	中国农业大学学报	al of China Agricultural Univ	22	9	55-63	2017	ssn.1007-4333.2017.09.07
5442	6年杭州市医院污水监测	age monitoring results of H	曾文芳,岑斌,胡薇薇,沈寅	nfang,Cen Bin,Hu Weiwei,S	中国农村卫生事业管理	na's rural health managem	37	10	1208-1209	2017	sn.1005-5916.2017.10.024
5443	株洲市食源性致病菌污染	tatus of foodborne pathog	艳,罗誉皓,胡卓,方英,刘育	uo Yuhao,Hu Zhuo,Fang Yi	河南预防医学杂志	n Journal of Preventive Me	28	10	769-770	2017	m.1006-8414.2017.10.014
5444	2002-2014年食源性疾病监	4Annual Foodborne Diseas	张静,张秉慧.	Zhang Jing,Zhang Binghui.	实用预防医学	ractical preventive medicin	24	4	486-488	2017	sn.1006-3110.2017.04.030
5445	业人员预防性健康检查沙	of preventive health exami	芬,邱峰,曾国,杨智旭,廖	Feng,Zeng Guo,Yang Zhixu	心电图杂志（电子版）	diogram Magazine (electror	6	2	229-230	2017	-
5446	源沙门氏菌分离鉴定与	nce research of Salmonella	丹俊,潘孝成,赵瑞宏,戴银	un,Pan Xiaocheng,Zhao Rui	中国畜牧兽医	mal husbandry and veterina	44	12	3663-3669	2017	ki.1671-7236.2017.12.038
5447	州市食源性疾病暴发事件	nce data on foodborne dise	王黎荔,高四海,山若青,蔡	Gao Sihai,Mountains are gr	卫生研究	health research	46	5	837-840	2017	-
5448	门菌引起的食源性疾病暴	oodborne illness outbreak	陈慧中,常春祥.	en Huizhong,Chang Chunxia	基层医学论坛	Primary Medicine Forum	21	17	2274-2276	2017	/j.1672-1721.2017.17.078
5449	袭性沙门菌属感染23例临	ections in adultstwenty thr	郭益群,王鹏,杨春霞,谷丽	un,Wang Peng,Yang Chunx	首都医科大学学报	hal of Capital Medical Unive	38	5	744-749	2017	sn.1006-7795.2017.05.021
5450	年威海市食源性疾病主动	e surveillance results of fo	徐莉,肖辉川,陈稳稳,闫兆	uichuan,Chen Wenwen,Yar	中国卫生检验杂志	ese Journal of Health Inspe	27	18	2693-2695,2698	2017	-
5451	年自贡市食源性疾病主动	llance results of foodborne	刘桂丹,范正轩,谢飞.	Guidan,Fan Zhengxuan,Xie	职业与健康	Occupation and health	33	20	2792-2794,2799	2017	-
5452	猪源沙门菌血清型与耐	of Salmonella originating f	,王伟芳,詹泽强,张红霞,	,Zhan Zeqiang,Zhang Hong	动物医学进展	vances in Veterinary Medic	38	2	32-36	2017	-
5453	门菌的血清分布类型和耐	nd drug resistance status o	陈琴,张瑞中,郭少燕.	Qin,Zhang Ruizhong,Guo Sh	护理研究	nursing research	31	32	4170-4172	2017	sn.1009-6493.2017.32.040
5454	014年食源性致病菌监测	s of annual foodborne path	陈萍.	Chen Ping.	中国卫生标准管理	a Health Standard Manage	8	27	1-2	2017	sn.1674-9316.2017.27.001
5455	菌引起食物中毒的病原鉴	pathogen causing food po	瑞娥,姚素霞,张秋香,王	Yao Suxia,Zhang Qiuxiang,V	中国卫生检验杂志	ese Journal of Health Inspe	27	14	2023-2025	2017	-
5456	我国家禽主要疫病的流行	tion and control strategies	尤永君.	You Yongjun.	中国禽业导刊	China Poultry Industry Guid	34	7	24-26	2017	-
5457	标本病原菌的监测及药敏	drug sensitivity analysis of	苏丽霞.	Su Lixia.	包头医学院学报	rnal of Baotou Medical Coll	33	3	30-31	2017	n.1006-740X.2017.03.014
5458	区羔羊腹泻主要病原菌的	genic bacteria causing diar	雪玲,美合日古丽·阿卜杜	n,Li Xueling,Meihe Riguli·A	新疆农垦科技	Agricultural Reclamation Te	40	8	27-29	2017	n.1001-361X.2017.08.011
5459	3-2015年感染性腹泻病原	surveillance analysis of inf	金丹,孙传武.	golden elixir,Sun Chuanwu.	中国初级卫生保健	primary health care in china	31	2	56-57,64	2017	.1001-568X.2017.02.0021
5460	年市售食品的食源性致病	pathogenic contamination	王君,韩蓓,陈小宁,任越.	un,Han Bei,Chen Xiaoning,R	中国热带医学	Chinese tropical medicine	17	10	991-996	2017	cnki.46-1064/r.2017.10.09
5461	湖北省桶装饮用水微生物	icrobial Contamination of B	,刘艳,杨硕,郭鹏程,邵翠翠	Yang Shuo,Guo Pengcheng	公共卫生与预防医学	Health and Preventive Me	28	3	49-51	2017	-
5462	食品中沙门菌污染与耐药	tamination and drug resist	周厚德,刘洋,姜茗,刘道峰	ude,Liu Yang,Jiang Ming,Liu	中国消毒学杂志	hinese Journal of Disinfectio	34	9	850-853	2017	sn.1001-7658.2017.09.017
5463	溪市一起伤寒暴发的流行	a typhoid fever outbreak	军;陈恩富;缪梓萍;叶建杰	Chen Enfu;Miao Ziping;	疾病监测	disease surveillance	32	3	258-262	2017	-
5464	检验在季节性腹泻检验中	routine stool examination	陈义波,刘芳芳.	Chen Yibo,Liu Fangfang.	健康大视野	Healthy vision	-	21	225-226	2018	sn.1005-0019.2018.21.339
5465	感染性食源性疾病流行特	alysis of infectious foodbo	王曙,沈福杰.	Wang Shu,Shen Fujie.	健康大视野	Healthy vision	-	20	278-279	2018	sn.1005-0019.2018.20.402
5466	沙门菌感染的流行病学调	istance analysis of Salmon	李国钢,李佳俊,赵升.	Guogang,Li Jiajun,Zhao She	中国现代医生	modern chinese doctor	56	6	135-137,141	2018	-
5467	童肠道感染非伤寒沙门菌	stance of non-typhoid Salm	涛,付四毛,谢广清,梁展图	ay 40 cents,Xie Guangqing	中国小儿急救医学	tric Emergency Medicine in	25	2	132-135	2018	sn.1673-4912.2018.02.011
5468	区177例食源性疾病的细菌	sis of bacterial detection r	王维娟,荆丽萍.	Wang Weijuan,Jing Liping.	职业卫生与应急救援	ional health and emergenc	36	1	51-53	2018	sn.1007-1326.2018.01.015
5469	食物中毒标本进行实验室	ry test results on bacterial	段绯.	Duan Fei.	家庭医药	home medicine	-	1	74-75	2018	sn.1671-4954.2018.01.096
5470	门菌感染的临床特点和药	nsitivity analysis of invasiv	,黄宝兴,周高枫,雷炎玲,	g Baoxing,Zhou Gaofeng,Le	临床儿科杂志	Journal of Clinical Pediatrics	36	10	741-744	2018	sn.1000-3606.2018.10.004
5471	门菌引起关节感染一例并	used by Salmonella typhi a	董秀涛.	Dong Xiutao.	医学检验与临床	edical Laboratory and Clini	29	10	58-59	2018	sn.1673-5013.2018.10.021
5472	鼠伤寒沙门菌耐药谱特征	ctrum characteristics of Sa	,陈建辉,黄梦颖,杨劲松,	hui,Huang Mengying,Yang	预防医学论坛	Preventive Medicine Forum	24	10	721-724	2018	-
5473	烤食品中微生物污染状	contamination in commerc	晓燕,杨舒然,李莹,张磊,	n,Yang Shuran,Li Ying,Zha	中国卫生检验杂志	ese Journal of Health Inspe	28	12	1499-1501	2018	-
5474	菌血清学与病原学监测结	a serological and pathogen	传彬,霍斯琪,刘玉良,张倩	bin,Huo Siqi,Liu Yuliang,Zh	中国兽医杂志	Chinese Veterinary Journal	54	2	3-6,11	2018	-

5475	市门头沟区食源性疾病主	nce of foodborne diseases	香菊,褚添,吕秋艳,王志	gju,Chu Tian,Lu Qiuyan,Wa	职业与健康	Occupation and health	34	23	3217-3219	2018	-
5476	从业人员伤寒痢疾快速筛	echnology in rapid screenir	吕志刚.	Lui Chi-gang.	医药前沿	Medical Frontier	8	22	345	2018	sn.2095-1752.2018.22.298
5477	中国市售酱油微生物污染	nination status of commerc	长会,闫琳,廖春,申志新,赵	,Yan Lin,Liao Chun,Shen Zh	中国调味品	Chinese condiments	43	12	191-195	2018	sn.1000-9973.2018.12.038
5478	鸡源沙门氏菌血清型与耐药	nce of Salmonella originati	玲,韩镌竹,李欣南,高铎,武	n Zhuzhu,Li Xinnan,Gao Du	现代畜牧兽医	mal husbandry and veterina	-	8	38-42	2018	-
5479	验在季节性腹泻检验 中的	and testing for seasonal dia	王珊珊.	Wang Shanshan.	黑龙江医药	Heilongjiang Medicine	31	3	611-613	2018	5/j.cnki.hljyy.2018.03.071
5480	性感染性腹泻病原菌及耐	nd drug resistance of acute	牛玉峰,施城东,问亚锋.	ufeng,Shi Chengdong,Ask Y	中国国境卫生检疫杂志	ntier Health and Quarantin	41	6	399-401	2018	/j.1004-9770.2018.06.003
5481	儿童沙门菌感染状况调查	and drug resistance analysis	秀蓉,张力,王洁琳,高飞,龙	,tension,Wang Jielin,fly hig	国际检验医学杂志	onal journal of laboratory	39	0	127-131	2018	-
5482	鸡生产全过程沙门菌的污染	s of Salmonella in the who	,张秀丽,遇晓杰,唐震,毕	Meet Xiaojie,Tang Zhen,Bi	中华预防医学杂志	se Journal of Preventive Me	52	4	352-357	2018	sn.0253-9624.2018.04.005
5483	食物中毒病原学及微生物	soning etiology and microb	李晓燕.	Li Xiaoyan.	中国卫生标准管理	a Health Standard Manager	9	1	6-8	2018	sn.1674-9316.2018.01.003
5484	洲市食源性疾病散发监测	rveillance results of foodbo	胡卓,刘育兰,郑玉峰.	Zhuo,Liu Yulan,Zheng Yufe	河南预防医学杂志	n Journal of Preventive Me	29	12	939-940	2018	m.1006-8414.2018.12.023
5485	仔猪肠道沙门氏菌耐药性	nce of enteric Salmonella ir	琦,张双翔,王伟,胡兴义,张	huangxiang,Wang Wei,Hu X	畜牧与兽医	Husbandry and Veterinary M	50	3	53-58	2018	-
5486	寒沙门菌肠炎的临床特征	nd drug resistance of Salmo	,万涵,符佳,邓慧玲,王军,徐	Han,Fu Jia,Deng Huiling,W	中国小儿急救医学	tric Emergency Medicine in	25	8	637-640	2018	sn.1673-4912.2018.08.019
5487	门菌耐药性分析及质粒基	nid gene detection of Salmo	蒋卫国.	Jiang Weiguo.	中国畜禽种业	estock and poultry breeding	14	3	36	2018	sn.1673-4556.2018.03.026
5488	查一起小学教职工及家属	outbreak of salmonella fo	周焕宁.	Zhou Huanning.	微量元素与健康研究	e elements and health rese	35	2	49-50	2018	-
5489	6个省市食源性沙门菌耐药	characteristics of foodbor	王美美,甘辛,徐进,李凤琴,杨	eimei,Ganxin,Xu Jin,Li Feng	中国食品卫生杂志	hinese Food Hygiene Journ	30	5	456-461	2018	.13590/j.cjfh.2018.05.002
5490	性食物中毒实验室检测结果	tory test results for bacteri	魏巍.	Wei Wei.	中国保健营养	Chinese health nutrition	28	6	364-365	2018	sn.1004-7484.2018.06.584
5491	染性腹泻分离菌株的分布和	nalysis of isolates of bacte	尚艳,万玛措,张毅斌.	ng Yan,Wanmacuo,Zhang Y	中国儿童保健杂志	ese Journal of Child Health	26	5	566-569	2018	52/zgetbjzz2018-26-05-28
5492	源性腹泻患者粪便细菌病原	f fecal bacteria in patients w	周丽华,王萍,强鑫华,柳秋霞	a,Wang Ping,Qiang Xinhua,	浙江临床医学	Zhejiang Clinical Medicine	20	6	1135-1136	2018	-
5493	区鸡沙门氏菌的分离鉴定及	ptibility test of Salmonella	张阿娥.	Zhang A'e.	福建畜牧兽医	al Husbandry and Veterina	40	4	6-8	2018	sn.1003-4331.2018.04.004
5494	膝关节多次术后肿痛1例报	ltiple postoperative swellin	石大鹏,潘守成.	Shi Dapeng,Pan Shoucheng	临床医药文献电子杂志	edical literature electronic	5	75	188	2018	sn.2095-8242.2018.75.138
5495	市嘉定区食品中食源性致	of foodborne pathogenic b	彭慧,孙思飞,彭谦,孙攀,陈	Sun Sifei,Peng Qian,Sun Pa	食品安全质量检测学报	f Food Safety and Quality I	9	15	4180-4183	2018	sn.2095-0381.2018.15.051
5496	便病原菌检验结果的临床	test results of pathogenic	张丽.	Zhang Li.	中国医药指南	Chinese Medicine Guide	16	24	51-52	2018	-
5497	沙门氏菌的分离鉴定与药	ug susceptibility test of Sal	宁,苏玉铭,魏澍,任玉峰,李	u Yuming,Wei Shu,Ren Yufe	现代畜牧兽医	mal husbandry and veterina	-	11	14-19	2018	-
5498	羊源沙门菌的分离鉴定及	ance analysis of Salmonella	王英,黄忍,李英林,邵靓,赵	g,Huang Ren,Li Yinglin,Shao	四川畜牧兽医	mal Husbandry and Veterina	45	11	23-25	2018	-
5499	龙山县感染性腹泻病原学	ious diarrhea etiology surv	张孝洪,罗垲炜,赵善露,吴	Xiaohong,Luo Weiwei,Zhao	实用预防医学	ractical preventive medicin	25	12	1462-1465	2018	sn.1006-3110.2018.12.015
5500	丁沙门菌致老年性尿路感	nfection in the elderly caus	李云,张健,武超玉,李霞.	n,Zhang Jian,Wu Chaoyu,L	世界最新医学信息文摘	he world's latest medical ir	-	44	-	2018	ki.1671-3141.2018.44.108
5501	点品种食品安全状况监测	ts of food safety status of	徐伟兵,叶石兴,李伟旗.	Weibing,Ye Shixing,Li Wei	心理医生	Psychologist	24	28	353-355	2018	-
5502	聚集性沙门氏菌食物中毒	sis of a family cluster of sal	严娟,谢文奇,刘承焱.	Juan,Xie Wenqi,Liu Chengy	赣南医学院学报	rnal of Gannan Medical Col	38	12	1228-1229,1238	2018	sn.1001-5779.2018.12.014
5503	古自治区食源性致病菌监	pathogenic bacteria in Inn	王利平,张冰冰,乌伊罕.	g Liping,Zhang Bingbing,Uy	现代预防医学	modern preventive medicin	45	18	3407-3410	2018	-
5504	市大兴区食品中食源性致	s of foodborne pathogenic	贾昆,余焕玲.	Jaqen,Yu Huanling.	中国医药导报	China Medical Herald	15	8	175-178	2018	-
5505	预冷后肉鸡胴体沙门菌污	els and drug resistance sta	,韩海红,刘继开,付萍,张	ihong,Liu Jikai,Fu Ping,Zhan	中华预防医学杂志	se Journal of Preventive Me	52	2	124-128	2018	sn.0253-9624.2018.02.002
5506	获得性革兰阴性杆菌血流	d gram-negative bacilli blo	,吴丹,张昕,石磊,谢杨新,	hang Xin,Shi Lei,Xie Yangxi	中华肝脏病杂志	hinese Journal of Hepatolog	26	1	23-27	2018	sn.1007-3418.2018.01.007
5507	工处理环节沙门氏菌污染	contamination in differen	王宏辉.	Wang Honghui.	中国畜禽种业	estock and poultry breeding	14	8	159-160	2018	sn.1673-4556.2018.08.140
5508	市售食品中食源性致病菌	of foodborne pathogenic b	平,汪洋,杨爱青,王淑田,杨	ang,Yang Aiqing,Wang Shu	中国公共卫生管理	ese public health managen	34	1	102-105	2018	cnki.23-1318.2018.01.030
5509	员43株肠道沙门菌血清分	ing and drug resistance sta	陈臣,朱丹,曾筱,杜昊,乔甫	,Zhu Dan,Zeng Xiao,Du Ha	现代预防医学	modern preventive medicin	45	10	1750-1752,1758	2018	-
5510	沙门菌引起食物中毒的溯源	f a case of food poisoning	宗华,许磊,曾德唯.	ong Hua,Xu Lei,Zeng Dewe	中国卫生检验杂志	ese Journal of Health Inspe	28	14	1771-1773	2018	-
5511	食源性腹泻病例监测病原	tion of foodborne diarrhea	婷婷,温晓红,诸小飞,周萍	Wen Xiaohong,Zhu Xiaofei,Z	中国现代医生	modern chinese doctor	56	24	98-101	2018	-

5512	庄地区食物中毒株沙门菌	yping of food poisoning str	保红,王燕,杨新英,郭玉梅	Wang Yan,Yang Xinying,Gu	中国卫生检验杂志	ese Journal of Health Inspe	28	9	1060-1062,1066	2018	-
5513	年上海市闵行区食源性疾	ts of foodborne diseases in	玉萍,朱效宁,王宙云,张兆	u Xiaoning,Wang Zhouyun,	职业与健康	Occupation and health	34	9	1223-1226	2018	-
5514	南省沙门菌污染分布状况	mination distribution and c	懿,赵嘉咏,尤爱国,夏胜利	ao Jiayong,You Aiguo,Xia Sh	中国人兽共患病学报	Chinese Journal of Zoonose	34	8	748-752	2018	sn.1002-2694.2018.00.119
5515	地区感染性腹泻病原菌	enic bacteria of infectious	孙立珍,孙立平,丁淑芹.	Lizhen,Sun Liping,Ding Shu	热带医学杂志	journal of tropical medicine	18	1	97-99,103	2018	sn.1672-3619.2018.01.026
5516	胚中沙门菌的分离鉴定	analysis of Salmonella fro	如,韩文龙,韩雨奇,匡秀华,	enlong,Han Yuqi,Kuang Xiul	中国兽医学报	al of Chinese Veterinary Me	38	7	1321-1326	2018	nki.1005-4545.2018.07.11
5517	伤寒沙门菌基因组流行病	c epidemiology of Salmone	秋,俞骅,郑伟,郑之北,潘	u Hua,Zheng Wei,Zheng Zh	中华微生物学和免疫学杂志	rnal of Microbiology and In	38	2	116-123	2018	sn.0254-5101.2018.02.007
5518	年辽宁省沙门菌耐药菌谱	ar typing of Salmonella dru	飞,于淼,魏彤竹,李雪,马景	ao,Wei Tongzhu,Li Xue,Ma	中国微生态学杂志	inese Journal of Microecolo	30	10	1142-1146	2018	381/j.cnki.cjm.201810007
5519	肠炎沙门菌引起的食物中毒	of food poisoning caused	赵健,李文玲.	Zhao Jian,Li Wenling.	中国城乡企业卫生	ygiene in urban and rural ar	33	11	76-78	2018	/j.1003-5052.2018.11.027
5520	年吉林省食源性致病菌监	g results of foodborne pat	薇,孙景昱,杨修军,石奔,黄	n Jingyu,Yang Xiujun,Shi Be	食品安全质量检测学报	f Food Safety and Quality I	9	4	947-950	2018	sn.2095-0381.2018.04.044
5521	门菌肠炎临床用药疗效比	ical drug efficacy in childre	绮筠,陈桂铃,汤耀光,曾丽	Chen Guiling,Tang Yaoguan	医学信息	medical information	31	1	127-129,132	2018	sn.1006-1959.2018.01.049
5522	3所学校食物中毒的溯源	ysis and drug susceptibility	斟,许磊,张鹏,成文,熊鹰,李	Lei,Zhang Peng,written,Xio	预防医学情报杂志	of Preventive Medicine Info	34	8	1050-1053	2018	-
5523	淮安市食品中食源性致病	g results of foodborne path	乒乓,刘靓,李军鹰,汪艳玲	,Liu Liang,Li Junying,Wang	中国卫生检验杂志	ese Journal of Health Inspe	28	6	709-711	2018	-
5524	临床鼠伤寒沙门氏菌的耐药	of clinical Salmonella typhi	李永瑞,李艳.	Li Yongrui,Li Yan.	南科技大学学报（医学版	ersity of Science and Techn	36	3	218-221	2018	sn1672-688x.2018.03.015
5525	全国食物中毒事件流行特	cteristics of food poisoning	晔,任婧寰,王哲,翁熹君,王	n Jinghuan,Wang Zhe,Weng	疾病监测	disease surveillance	33	5	359-364	2018	sn.1003-9961.2018.05.004
5526	鉴定、药敏试验及毒力基	drug susceptibility testing	春香,陈苗,刘本金,郭伟娜,文	hen Miao,Liu Benjin,Guo W	中国微生态学杂志	inese Journal of Microecolo	30	6	635-639,646	2018	381/j.cnki.cjm.201806004
5527	学生餐食源性致病菌污染	of foodborne pathogenic b	李艳芬,炊慧霞,张濛,张秀	,Cui Huixia,Zhang Meng,Zh	中国学校卫生	Chinese school health	39	1	54-56	2018	ki.1000-9817.2018.01.016
5528	腰椎感染波茨坦沙门菌1例	Infection Salmonella Potsc	,郭健莲,张阳根,江先海,徐	nlian,Zhang Yanggen,Jiang	临床检验杂志	journal of clinical testing	36	1	75	2018	602/j.cnki.jcls.2018.01.21
5529	养方式下牛源沙门氏菌的	f bovine Salmonella under	马淑一,何珊,张竹强,高芳,	,He Shan,Zhang Zhuqiang,G	包头医学院学报	rnal of Baotou Medical Coll	34	2	80-82	2018	3/j.cnki.jbmc.2018.02.037
5530	污染沙门氏菌菌株毒力基因	ains contaminated with po	李晨阳,赵思俊,赵建梅,赵格	g,Zhao Sijun,Zhao Jianmei,	中国人兽共患病学报	Chinese Journal of Zoonose	34	8	697-702	2018	sn.1002-2694.2018.00.132
5531	伤寒沙门菌感染的临床特	ceptibility analysis of non-t	刘芙蓉,于四景,罗如平.	Furong,Yu Sijing,Luo Rupi	医学临床研究	medical clinical research	35	7	1372-1374	2018	sn.1671-7171.2018.07.043
5532	不同来源的肠炎沙门菌耐药	lmonella Enteritidis from d	明,袁丹茅,李美华,曹春远,陈	anmao,Li Meihua,Cao Chur	河南预防医学杂志	n Journal of Preventive Me	29	1	43-45	2018	m.1006-8414.2018.01.013
5533	同饲养方式下牛源沙门氏菌	monella under different fe	一,何珊,张竹强,高芳,于敬	han,Zhang Zhuqiang,Gao F	包头医学院学报	rnal of Baotou Medical Coll	34	1	89-91	2018	3/j.cnki.jbmc.2018.01.043
5534	慢性腹泻患者粪便标本病原	microorganisms in fecal sa	黄凡华,田琪.	Huang Fanhua,Tian Qi.	家庭医药	home medicine	-	11	117	2018	sn.1671-4954.2018.11.148
5535	海地区腹泻致病菌及药敏分	eria and drug susceptibility	振波,卞志伟,高娟娟,黄君	Bian Zhiwei,Gao Juanjuan,H	中国国境卫生检疫杂志	ontier Health and Quarantin	41	2	84-86	2018	/j.1004-9770.2018.02.003
5536	动物性水产品中食源性致	genic bacteria in commerc	慧霞,吴玲玲,邱正勇,张秀	Wu Lingling,Qiu Zhengyong,	中国卫生检验杂志	ese Journal of Health Inspe	28	3	338-339,342	2018	-
5537	地区鸡沙门氏菌病流行病学	tigation of chicken salmone	刘佳.	Liu Jia.	中国动物保健	China animal health	20	7	40-42	2018	sn.1008-4754.2018.07.029
5538	市熟肉制品加工过程微生	mination during cooked me	张艳,蒋君,伏子奇.	Zhang Yan,Jiang Jun,Fu Ziqi	预防医学情报杂志	of Preventive Medicine Info	34	11	1410-1413	2018	-
5539	部分地区猪源沙门菌的分	onella originating from pig	晓刚,吴丽云,张玥,孙彤,李	ng,Wu Liyun,Zhang Yue,Su	畜牧与兽医	Husbandry and Veterinary M	50	1	79-85	2018	-
5540	后感染相关因素分析及其	fter gastrointestinal endos	智敏,钟惠梅,余秀晶,方彩	n,Zhong Huimei,Yu Xiujing,	现代诊断与治疗	dern Diagnosis and Treatm	29	20	3248-3250	2018	sn.1001-8174.2018.20.029
5541	地区沙门菌感染流行病学	acteristics and drug resista	汤勇才,吴斌,黄小媛,廖军	cai,Wu Bin,Huang Xiaoyua	国际检验医学杂志	onal journal of laboratory	39	5	635-637	2018	sn.1673-4130.2018.05.035
5542	沙门氏菌在长春南湖水体中	a Typhimurium in Changch	儒,丁美月,张蕊,李佳航,马	Meiyue,Zhang Rui,Li Jiaha	科学技术与工程	nce Technology and Engine	18	8	338-342	2018	sn.1671-1815.2018.08.057
5543	17年沙门氏菌血清型分布	17Salmonella serotype dist	,李伟莉,林珊珊,李娟美,吴	Weili,Lin Shanshan,Li Juann	医学动物防制	medical animal control	34	11	1118-1121	2018	0.7629/yxdwfz201811030
5544	加工过程中沙门菌污染定量	monella contamination dur	华,王娟,曲志娜,盖文燕,黄	Juan,Qu Zhina,Gai Wenya	中国动物检疫	China Animal Quarantine	35	4	26-31	2018	n.1005-944X.2018.04.007
5545	年泰安市食源性疾病监测	odborne disease surveillan	桂,唐秀芹,吴鹏,徐红霞,肖	Xiuqin,Wu Peng,Xu Hongx	实用预防医学	ractical preventive medicin	25	1	78-82	2018	sn.1006-3110.2018.01.022
5546	养病原菌的分布及主要病	ulture of children and analy	詹志祥.	Zhan Zhixiang.	现代预防医学	modern preventive medicin	45	8	1524-1527	2018	-
5547	蛋鸡19种疫病流行病学调	pidemiological investigatio	李桂喜,何永光,梁磊,魏曼,	He Yongguang,Liang Lei,W	家禽科学	poultry science	-	10	39-41	2018	sn.1673-1085.2018.10.012
5548	深圳市龙岗区感染性腹泻病	ous diarrhea pathogens in	平,刘凤仁,刘渠,金玉娟,李	g,Liu Fengren,Liu Qu,Jin Yu	热带医学杂志	journal of tropical medicine	18	7	977-980	2018	sn.1672-3619.2018.07.032

5549	原性疾病监测病例流行特征	fluencing factors of foodbo	士军,欧阳艳昊,赖天兵,柳恒	uyang Yanhao,Lai Tianbing,	实用预防医学	ractical preventive medicin	25	12	1433-1437,1444	2018	sn.1006-3110.2018.12.007
5550	沙门菌单相变异株的鉴定	lysis of single-phase mutan	雯,屠丽红,张雯霞,张曦,陈	ihong,Zhang Wenxia,Zhang	检验医学	laboratory medicine	33	9	798-802	2018	sn.1673-8640.2018.09.006
5551	性腹泻的病原菌分布与耐	ance of pathogenic bacteria	魏寒松.	Wei Hansong.	世界华人消化杂志	ld Chinese Journal of Diges	26	11	680-686	2018	0.11569/wcjd.v26.i11.680
5552	7年食品安全风险监测微生	is of annual food safety risk	罗伟倩,潘伟芬,黄群娣.	Veiqian,Pan Weifen,Huang C	食品安全导刊	food safety guide	-	25	67-69	2018	sn.1674-0270.2018.25.028
5553	沙门菌食物中毒事件流行	n on an incident of Salmone	陈亚娜,曹信金.	Chen Yana,Cao Xinjin.	海峡预防医学杂志	s Journal of Preventive Me	24	1	88-89	2018	-
5554	5年食源性疾病病原学监测	f annual foodborne disease	周银柱,朱彩明,陈艳,苏良	zhu,Zhu Caiming,Chen Yan,	海峡预防医学杂志	s Journal of Preventive Me	24	1	77-79	2018	-
5555	同城乡类型地区细菌性腹	acterial diarrhea pathogen	秦帅,段然,景怀琦,王鑫.	ai,Duan Ran,Jing Huaiqi,Wa	中华流行病学杂志	inese Journal of Epidemiolo	39	5	651-655	2018	sn.0254-6450.2018.05.021
5556	伤寒沙门菌血清型分布、耐	n of non-typhoidal Salmone	李迎慧,邱亚群,陈琼城,江敏	Yaqun,Chen Qiongcheng,Ji	热带医学杂志	journal of tropical medicine	18	1	79-81,96	2018	sn.1672-3619.2018.01.021
5557	大连市伤寒沙门菌耐药性及	tance and molecular typing	明玉,陈玉凤,侯君,杨世宏,	,Chen Yufeng,Hou Jun,Yang	中国微生态学杂志	inese Journal of Microecolo	30	6	683-686	2018	381/j.cnki.cjm.201806014
5558	017年市售食品中食源性致	analysis of foodborne path	胡凤清,郑雯菱,叶丽丹.	engqing,Zheng Wenling,Ye L	海峡预防医学杂志	s Journal of Preventive Me	24	4	62-64	2018	-
5559	腹泻患者病原菌检测与结果	ts of pathogenic bacteria in	吴斌,袁登学.	Wu Bin,Yuan Dengxue.	心理月刊	Psychological Monthly	-	10	188	2018	-
5560	起沙门氏菌食物中毒调查分	nalysis of a case of salmon	楷城,黄葵青,朱世瑜,陈锦	g,Huang Kuiqing,Zhu Shiyu	中国农村卫生事业管理	na's rural health managem	38	2	204-206	2018	sn.1005-5916.2018.02.027
5561	泉州市哨点医院食源性疾病	sease surveillance results i	升浩,许猛,王悦然,张娟,吴	Xu Meng,Wang Yueran,Zha	现代预防医学	modern preventive medicin	45	24	4500-4503,4512	2018	-
5562	区腹泻患者沙门菌耐药性	l flight mass spectrometry i	海波,王云霞,史文凤,黄少	ng Yunxia,Shi Wenfeng,Hu	职业与健康	Occupation and health	34	9	1170-1173	2018	-
5563	寒沙门氏菌引起的食物中	case of food poisoning caus	王婧,何旭鑫.	Wang Jing,He Xuxin.	医学信息文摘（连续型电	information abstract (cont	18	51	254256	2018	ki.1671-3141.2018.51.149
5564	处理环节沙门氏菌污染情	a contamination in differen	刘彬.	Liu Bin.	乡村科技	Rural Technology	-	17	105-106	2018	sn.1674-7909.2018.17.064
5565	地区沙门菌属细菌耐药及	characteristics of Salmonel	瑜华,阿祥仁,鲁卫平,季萍,张	A Xiangren,Lu Weiping,Ji P	中国抗生素杂志	hinese Journal of Antibiotic	43	9	1073-1078	2018	sn.1001-8689.2018.09.002
5566	水产品食源性病原微生物	borne pathogenic microorg	衣明.	Yi Ming.	中国保健营养	Chinese health nutrition	28	10	197-198	2018	sn.1004-7484.2018.10.241
5567	沙门氏菌耐药性及耐药基	ug-resistant genes of Salmo	鹏,张宝珠,韩芮,刘福星,郭	ang Baozhu,Han Rui,Liu Fux	当代畜禽养殖业	y livestock and poultry bree	-	10	11	2018	sn.1005-5959.2018.10.007
5568	郴州市哨点医院食源性疾病	disease surveillance results	军,刘勋,李征莉,段朝标,关	Kun,Li Zhengli,Duan Chaobi	中国卫生检验杂志	ese Journal of Health Inspe	28	2	240-243	2018	-
5569	菌耐药性及耐药基因的检测	g resistance and drug-resis	张林吉,任士飞,孙朋,吴艳涛	nji,Ren Shifei,Sun Peng,Wu	中国家禽	Chinese poultry	40	14	62-65	2018	sn.1004-6364.2018.14.014
5570	泻小儿肠道病原体分布特	tics of intestinal pathogens	李玲.	Li Ling.	河南医学高等专科学校学报	rnal of Henan Medical Coll	30	5	517-519	2018	sn.1008-9276.2018.05.036
5571	5-2016年食源性疾病监测	on annual foodborne disea	黄晓媚,董勤,黄杰周,李家杰	omei,Dong Qin,Huang Jiezh	中国公共卫生管理	ese public health managen	34	2	208-211	2018	cnki.23-1318.2018.02.018
5572	区245例腹泻患儿沙门菌亚	sis of Salmonella subtype d	张育超.	Zhang Yuchao.	中国医药科学	Chinese Medical Sciences	8	19	125-127	2018	sn.2095-0616.2018.19.034
5573	门菌的耐药表型与耐药基	d drug-resistant genes of S	宏,齐亚如,韩雨奇,匡秀华,蒋	Yaru,Han Yuqi,Kuang Xiuhu	中国家禽	Chinese poultry	40	1	51-55	2018	sn.1004-6364.2018.01.011
5574	电泳技术在饲料中沙门菌检	trophoresis technology in t	刘鸿鹤,韩镌竹,武凤娇,李	onghe,Han Zhuzhu,Wu Feng	饲料研究	feed research	-	4	41-45	2018	sn1002-2813.2018.04.011
5575	年桐庐县食源性特定病原菌	results of foodborne specif	康义,陆震宇,王秋英,郑卢	u Zhenyu,Wang Qiuying,Zh	中国卫生检验杂志	ese Journal of Health Inspe	28	23	2918-2920	2018	-
5576	泉市市售食品中食源性致	orne pathogenic bacteria in	高银平.	Gao Yinping.	中国食物与营养	Chinese food and nutrition	24	8	18-20	2018	sn.1006-9577.2018.08.004
5577	门菌感染临床特点及耐药	ics and drug resistance of S	陈小丽,吴佳音,连清荣,叶	Xiaoli,Wu Jiayin,Lian Qingr	中国当代儿科杂志	Journal of Contemporary P	20	11	921-924	2018	sn.1008-8830.2018.11.008
5578	禽沙门菌的分离鉴定与血清	and serotype analysis of wa	荣旭,张溢珊,曹梦蕊,陈建	ang Yishan,Cao Mengrui,Ch	中国人兽共患病学报	Chinese Journal of Zoonose	34	6	546-549	2018	sn.1002-2694.2018.00.085
5579	氏菌引起的食源性疾病暴	dborne disease outbreak c	韩祖源.	Han Zuyuan.	心理医生	Psychologist	24	14	348-349	2018	-
5580	132例沙门菌耐药性检测及	ples of Salmonella drug res	黄邀,陈玲玲,陆卫民.	ng Yao,Chen Lingling,Lu We	中国基层医药	China primary medicine	25	3	356-359	2018	sn.1008-6706.2018.03.023
5581	鼠伤寒沙门菌药物敏感性	of the method in drug susc	军,黄梦颖,杨劲松,徐海滨,罗	Mengying,Yang Jinsong,Xu H	预防医学论坛	Preventive Medicine Forum	24	7	481-482,486	2018	-
5582	年郴州市鲜禽畜肉食品风险	oring results of fresh poultr	兆令辉,郑文,欧社祥,贺莉萍	ui,Zheng Wen,Ou Shexiang	中国卫生检验杂志	ese Journal of Health Inspe	28	24	3030-3033	2018	-
5583	食品微生物及其致病因子	microorganisms and their	毕红杰,张玉梅.	Bi Hongjie,Zhang Yumei.	职业与健康	Occupation and health	34	14	1917-1920	2018	-
5584	鸡肉源沙门氏菌的分离鉴定	e analysis of Salmonella orig	海依拉提,郜敏,克然木·托乎	,Gao Min,Kerim·Tohuti,Len	食品安全质量检测学报	f Food Safety and Quality I	9	7	1524-1528	2018	sn.2095-0381.2018.07.010
5585	加工中沙门菌和弯曲杆菌	bacter contamination in br	洋,安雅洁,周莉,王法云,闫	n Yajie,Zhou Li,Wang Fayu	中国家禽	Chinese poultry	40	4	80-84	2018	sn.1004-6364.2018.04.018

5586	朱山夫登堡沙门菌的分子分	typing and drug resistance	吴鹏程,樊飞,施爱萍,钱惠	gcheng,Fan Fei,Shi Aiping,C	现代预防医学	modern preventive medicin	45	20	3774-3777	2018	-
5587	谱区食源性疾病监测状况	disease surveillance status	军华,李培松,桂九凤,况强	,Li Peisong,Gui Jiufeng,Kua	实验与检验医学	imental and Laboratory Me	36	5	778-780	2018	sn.1674-1129.2018.05.047
5588	乙型副伤寒沙门菌暴发疫情	nto an outbreak of Salmone	赵虹.	Zhao Hong.	海峡预防医学杂志	s Journal of Preventive Me	24	4	96-97	2018	-
5589	售牛肉中沙门菌毒力基因	onella virulence genes in X	阿依·海依拉提,张晓玲,克	ayi·Haierati,Zhang Xiaoling	食品安全质量检测学报	f Food Safety and Quality I	9	17	4675-4680	2018	sn.2095-0381.2018.17.034
5590	种肠炎沙门菌分离株的生物	Salmonella Enteritidis isol	林,鲍晓伟,李富祥,严红亚,王	aowei,Li Fuxiang,Yan Hong	中国家禽	Chinese poultry	40	17	61-63	2018	sn.1004-6364.2018.17.015
5591	寒沙门菌感染的流行病学分	den probability of non-typ	李华,宋夏,陆冬磊,吴春峰,陈	ong Xia,Lu Donglei,Wu Chu	中国食品卫生杂志	hinese Food Hygiene Journ	30	1	12-17	2018	0.13590/j.cjfh.2018.01.003
5592	腹泻病多重PCR方法建立	sheepPCRMethod establis	娥,蒲龄,余波,王璇,杨莉,朱	termath,Wang Xuan,Yang	中国畜禽种业	estock and poultry breeding	14	12	147-149	2018	sn.1673-4556.2018.12.117
5593	区猪源沙门菌的分离鉴定及	e analysis of Salmonella ori	李汉源,张宇航,肖雨屹,史浩	n,Zhang Yuhang,Xiao Yuyi,S	畜牧与兽医	Husbandry and Veterinary M	50	7	82-87	2018	-
5594	食源性腹泻病原体检测结	ults of foodborne diarrhea	陈荣富,张海芳,张森荣,孟华	hang Haifang,Zhang Senro	中国卫生检验杂志	ese Journal of Health Inspe	28	8	934-937	2018	-
5595	白痢沙门氏菌的分离鉴定与	nce analysis of Salmonella	翡雅,马邯生,刘彦威,刘娜,李	a Hansheng,Liu Yanwei,Liu	黑龙江畜牧兽医（上半月）	ndry and Veterinary Medic	-	2	169-172,259	2018	cnki.hljxmsy.2017.03.0089
5596	驴场几种重要传染病的调	eral important infectious di	陶永梅,刘宪斌,曲洪磊,刘	Yongmei,Liu Xianbin,Qu Ho	动物医学进展	vances in Veterinary Medic	39	4	128-132	2018	sn.1007-5038.2018.04.027
5597	年北京地区实验动物质量抽	uality inspection of experin	洪,王吉,李晓波,冯育芳,王	g Ji,Li Xiaobo,Feng Yufang,W	实验动物科学	Laboratory animal science	35	5	60-66	2018	sn.1006-6179.2018.05.012
5598	副溶血性弧菌、非伤寒沙	emolyticus and non-typhoi	蒂,廖宁波,齐小娟,陈莉莉,王	iao Ningbo,Qi Xiaojuan,Che	中国预防医学杂志	se Journal of Preventive Me	19	1	49-52	2018	6/j.1009-6639.2018.01.012
5599	市肉与肉制品中食源性致	foodborne pathogenic bact	姜慧仙,毛丽仙,郑升龙.	uixian,Mao Lixian,Zheng She	中国卫生检验杂志	ese Journal of Health Inspe	28	22	2798-2800	2018	-
5600	鸡场沙门氏菌的耐药分析及	medication of Salmonella i	郭战勇.	Guo Zhanyong.	农业知识（科学养殖）	ural knowledge (scientific b	-	10	46	2018	-
5601	鸡屠宰加工和销售过程中沙	ing broiler slaughtering, pr	田延,杨加祥,宋红芹.	Yan,Yang Jiaxiang,Song Hon	中国家禽	Chinese poultry	40	13	69-72	2018	sn.1004-6364.2018.13.017
5602	沙门菌对黏菌素的耐药性及	ating from pigs and poultry	传周,赵建梅,李月华,迟良	zhou,Zhao Jianmei,Li Yueh	动物医学进展	vances in Veterinary Medic	39	12	20-26	2018	sn.1007-5038.2018.12.005
5603	写患者病原菌流行趋势及耐	esistance of pathogenic bac	纟,李修远,顾海彤,孙宇峰,鲁	Xiuyuan,Gu Haitong,Sun Y	临床检验杂志	journal of clinical testing	36	4	314-316	2018	3602/j.cnki.jcls.2018.04.19
5604	品生产企业的生产加工环节	cteristics in the production	宋燕,徐迎春,伍海燕,宫月华	u Yingchun,Wu Haiyan,Gon	食品安全质量检测学报	f Food Safety and Quality I	9	7	1538-1541	2018	sn.2095-0381.2018.07.013
5605	腹泻的病原菌分布特点及	ance analysis of pathogeni	张海玉.	Zhang Haiyu.	实用临床医药杂志	nal of Practical Clinical Med	22	23	88-90,94	2018	10.7619/jcmp.201823026
5606	中沙门菌荧光定量PCR检测	d animal-derived feedPCR	华,张宁,黄翠琴,王武军,俞	g Ning,Huang Cuiqin,Wang	中国兽医科学	Chinese Veterinary Science	48	2	167-174	2018	issn.1673-4696.2018.0026
5607	部分食品微生物污染检测	ntamination test results of	晶,沈晓丽,耿文宁,闫明俞,杨	Xiaoli,Geng Wenning,Yan M	食品安全质量检测学报	f Food Safety and Quality I	9	1	210-217	2018	sn.2095-0381.2018.01.035
5608	起群发性急性胃肠炎的调	a cluster of acute gastroent	许保生,王晓燕,张春燕.	heng,Wang Xiaoyan,Zhang C	解放军预防医学杂志	tive Medicine of the People	36	6	806-807,809	2018	-
5609	鼠伤寒沙门菌同源性分析及	han community of Guangd	严海忠,王娟,罗锡华,冯雪琴	ng,Wang Juan,Luo Xihua,Fe	现代检验医学杂志	l of Modern Laboratory Me	33	4	63-66,69	2018	sn.1671-7414.2018.04.016
5610	市售禽畜肉中沙门菌污染	ltry and livestock meat sold	刘靓,李双姝,范乃来,侯海	ng,Li Shuangshu,Fan Nailai	卫生研究	health research	47	2	260-265,300	2018	-
5611	沙门氏菌血清型、耐药性	esistance genes of Salmon	周庆安,陈泽祥,许力士,柳	Qingan,Chen Zexiang,Xu Lis	中国畜牧兽医	mal husbandry and veterina	45	3	770-780	2018	ki.1671-7236.2018.03.027
5612	市食源性疾病流行病学特	tics and etiological analysis	王喜,赵永琪,孟伟,张卫文	hao Yongqi,Meng Wei,Zhan	河南预防医学杂志	n Journal of Preventive Me	29	5	397-399	2018	m.1006-8414.2018.05.029
5613	性相关腹泻病原菌感染监测	pathogenic bacterial infec	杰,陈志杭,王海明,叶斌,葛	Zhihang,Wang Haiming,Ye	浙江临床医学	Zhejiang Clinical Medicine	20	9	1525-1527	2018	-
5614	因食用超市熟食所致疾病的	lness caused by eating sup	江海棠,汪斌,陈荣凯,毕嘉瑶	aitang,Wang Bin,Chen Rong	中国公共卫生管理	nese public health managen	34	1	52-54	2018	cnki.23-1318.2018.01.015
5615	氏菌的分离鉴定及其耐药性	cks in Chengdu area and a	凡,邢天,巫梦雨,黄勇,邹立	tian,Wu Mengyu,Huang Yo	中国人兽共患病学报	Chinese Journal of Zoonose	34	3	217-223	2018	sn.1002-2694.2018.00.037
5616	5—2017年食源性疾病监测	lysis of annual foodborne d	国红,李云云,许雅鑫,李兴	hong,Li Yunyun,Xu Yaxin,L	心理医生	Psychologist	24	12	297-298	2018	-
5617	区甲型副伤寒沙门菌优势克	ups of Salmonella Paratyph	小莉,刁保卫,闫梅英,阚飙,徐	ao Baobao,Yan Meiying,Ka	中国预防医学杂志	se Journal of Preventive Me	19	1	70-74	2018	6/j.1009-6639.2018.01.016
5618	污染肉制品引起的食源性疾	s outbreak caused by Salm	张民,段德水,于瑞洪,钟庆,孙	uan Deshui,Yu Ruihong,Zho	预防医学论坛	Preventive Medicine Forum	24	11	846-848	2018	-
5619	4年-2017年食源性疾病监测	alysis of annual foodborne	申屠平平,吕恭进,朱珈慧.	u Pingping,Lu Gongjin,Zhu J	中国卫生检验杂志	ese Journal of Health Inspe	28	22	2803-2806	2018	-
5620	染性腹泻病例中沙门菌血清	stance analysis of Salmonel	小凤,庄春红,郑友限,陈韵	ang Chunhong,Zheng Youx	中国食品卫生杂志	hinese Food Hygiene Journ	30	4	368-372	2018	0.13590/j.cjfh.2018.04.006
5621	三河市餐服人员沙门氏菌	tuation among catering sta	项,王海霞,王宇卓,王敏娣,梁	Haixia,Wang Yuzhuo,Wang	医学动物防制	medical animal control	34	1	97-98	2018	10.7629/yxdwfz201801032
5622	引起的食物中毒的实验室	report on food poisoning ca	傅晓花.	Fu Xiaohua.	健康必读	Health must read	-	21	233	2018	-

5623	杨梅微生物污染调查及限	tion of bayberry in Zhejian	英平,杨桂玲,牛灵,赵慧宇	ng,Yang Guiling,Niu Ling,Zh	浙江农业科学	hejiang Agricultural Science	59	7	1261-1263,1266	2018	.issn.0528-9017.20180756
5624	生肉中食源性沙门菌的	istance of foodborne Salmo	刘军,吴莹,张杰.	Liu Jun,Wu Ying,Zhang Jie.	现代预防医学	modern preventive medicin	45	8	1508-1511,1527	2018	-
5625	市食品从业人员肠道致病	nal pathogenic bacteria am	舜珊,尤倩媚,陈焕娟,郑悦	u Qianmei,Chen Huanjuan	热带医学杂志	journal of tropical medicine	18	10	1378-1381	2018	sn.1672-3619.2018.10.029
5626	合并胃肠道血管炎继发肠	astrointestinal vasculitis se	繁奇,薛阿利,吴素叶,赵	Fanqi,Xue Ali,Wu Suye,Zha	温州医科大学学报	al of Wenzhou Medical Univ	48	10	773-775	2018	sn.2095-9400.2018.10.016
5627	源性疾病监测医院工作现	s of Xinjiang foodborne dis	胡晓琴,王雯雷,马龙.	Xiaoqin,Wang Wenlei,Malo	新疆医学	Xinjiang Medicine	48	12	1330-1334	2018	-
5628	内江市鼠伤寒沙门菌监测	typing of Salmonella typhi	媛媛,叶帮芬,古月,代东	yuan,Ye Bangfen,Gu Yue,Da	疾病监测	disease surveillance	33	5	397-401	2018	sn.1003-9961.2018.05.011
5629	染性腹泻监测病例中沙门	lmonella in surveillance ca	瑜,李世军,王斌,黄荷,唐	n,Wang Bin,Huang He,Tan	中国人兽共患病学报	Chinese Journal of Zoonose	34	8	712-715	2018	sn.1002-2694.2018.00.124
5630	禽源沙门氏菌的分离鉴定	ysis of Salmonella originati	荣旭,张溢珊,陈建红,黄鹤	ang Yishan,Chen Jianhong,	黑龙江畜牧兽医（下半月）	dry and Veterinary Medicin	-	5	116-119	2018	nki.hljxmsy.2017.11.0435
5631	区腹泻患者弯曲菌流行	g resistance of Campyloba	李颖,张爽,张彦春,马红梅	g Shuang,Zhang Yanchun,	疾病监测	disease surveillance	33	12	1048-1053	2018	sn.1003-9961.2018.12.018
5632	海市市售食品中食源性致	odborne pathogenic bacte	张化江,徐莉,于京平,肖辉	iang,Xu Li,Yu Jingping,Xiao	中国卫生检验杂志	ese Journal of Health Inspe	28	6	727-730	2018	-
5633	年家庭内食源性疾病暴发	nnual foodborne disease o	昌,李薇薇,潘娜,鲍小波,耿	eiwei,Panna,Bao Xiaobo,Ge	现代预防医学	modern preventive medicin	45	8	1499-1503,1519	2018	-
5634	污染引起食物中毒的实验	a case of food poisoning c	张欣,苏新霞,王亚丽.	hang Xin,Su Xinxia,Wang Ya	河南预防医学杂志	n Journal of Preventive Me	29	3	227-228	2018	m.1006-8414.2018.03.022
5635	年引起食源性腹泻的主要	ogenic bacteria causing foo	志涛,张强,万蓉,万青青,	hang Qiang,Wanrong,Wan	昆明医科大学学报	al of Kunming Medical Univ	39	3	109-114	2018	sn.1003-4706.2018.03.022
5636	鸡中沙门菌血清型分布	eristics of Salmonella seroty	,王美美,甘辛,王伟,闫韶	Meimei,Ganxin,Wang Wei	中华预防医学杂志	se Journal of Preventive Me	52	4	372-377	2018	sn.0253-9624.2018.04.008
5637	腹膜透析相关性腹膜炎二	ated peritonitis caused by	军,周柳沙,俞东容,童孟立	u Liusha,Yu Dongrong,Ton	中华肾脏病杂志	hinese Journal of Nephrolo	34	10	783-784	2018	sn.1001-7097.2018.10.012
5638	区网络外卖餐饮食品微	of online takeaway caterin	,卢嘉明,曾玉梅,梁暄,宋	ng,Zeng Yumei,Liang Xuan,	中国食品卫生杂志	hinese Food Hygiene Journ	30	5	514-518	2018	.13590/j.cjfh.2018.05.014
5639	水煮花生引起食物中毒的	ood poisoning caused by e	王岩,贾永华.	Wang Yan,Jia Yonghua.	河南预防医学杂志	n Journal of Preventive Me	29	1	73,78	2018	m.1006-8414.2018.01.025
5640	食品中不同微生物的污染	of different microorganisr	陈宇鸿,陈飞荣,林建伟,邵	hong,Chen Feirong,Lin Jian	中国卫生检验杂志	ese Journal of Health Inspe	28	14	1776-1778	2018	-
5641	菌耐药基因与毒力基因筛	s and virulence genes in co	志娜,赵思俊,李月华,王	,Zhao Sijun,Li Yuehua,War	中国动物检疫	China Animal Quarantine	35	11	81-86	2018	n.1005-944X.2018.11.022
5642	相关噬血细胞综合征1例	emophagocytic syndrome1	刘海峰,王季石,吴莉.	iu Haifeng,Wang Jishi,Wu L	现代医药卫生	modern medicine and healt	34	9	1438-1440	2018	sn.1009-5519.2018.09.059
5643	年上海市静安区食源性疾	ysis of foodborne diseases	玲玲,陈毅琼,王向东,俞沈	hen Yiqiong,Wang Xiangdo	公共卫生与预防医学	Health and Preventive Me	29	3	101-103	2018	sn.1006-2483.2018.03.026
5644	海市金山区其他感染性腹	ristics of other infectious d	李俊,宋灿磊.	Li Jun,Song Canlei.	职业与健康	Occupation and health	34	2	225-228	2018	-
5645	者下肢乙型副伤寒沙门菌	he lower limbs of a patient	尤聪,谢春,叶小英.	u Cong,Xie Chun,Ye Xiaoyi	重庆医学	Chongqing Medicine	47	26	3483-3484	2018	sn.1671-8348.2018.26.033
5646	源沙门氏菌对四环素耐药	resistance of Salmonella o	张博.	Zhang Bo.	中国畜禽种业	estock and poultry breeding	14	5	38	2018	sn.1673-4556.2018.05.032
5647	区2014—2015年食源性	ce2014—2015Analysis of a	马良良.	Ma Liangliang.	现代医药卫生	modern medicine and healt	34	18	2828-2830	2018	sn.1009-5519.2018.18.015
5648	区腹泻病例中沙门菌流行	lecular typing characteristi	爽,王彦波,张彦春,张赫,	ang Yanbo,Zhang Yanchun,	疾病监测	disease surveillance	33	10	803-808	2018	sn.1003-9961.2018.10.004
5649	猪场4种细菌耐药性和毒	e4Detection of bacterial re	琦,彭子欣,李凤琴,黎娜,葛	eng Zixin,Li Fengqin,Lina,G	中国食品卫生杂志	hinese Food Hygiene Journ	30	6	577-581	2018	.13590/j.cjfh.2018.06.005
5650	品中沙门菌检测及生物学	racteristics Analysis of Saln	军,岳文喜,朱克永,李俊	n,Yue Wenxi,Zhu Keyong,L	中国兽医杂志	Chinese Veterinary Journal	54	3	70-73	2018	-
5651	年湖南省感染性腹泻病原	ectrum characteristics of i	志红,欧阳云,张孝洪,龙奇	g,Ouyang Yun,Zhang Xiaoh	热带病与寄生虫学	pical Diseases and Parasito	16	2	71-74	2018	sn.1672-2302.2018.02.003
5652	年深圳市食物中毒流行病	emiological characteristics	,张锦周,刘建平,潘柳波,	Jinzhou,Liu Jianping,Pan L	中国食品卫生杂志	hinese Food Hygiene Journ	30	4	372-375	2018	.13590/j.cjfh.2018.04.007
5653	沙门氏菌血清分型方法的	tion of serotyping method	梅,李月华,宋传周,赵格,曲	Yuehua,Song Chuanzhou,Zh	中国动物检疫	China Animal Quarantine	35	1	73-77	2018	n.1005-944X.2018.01.021
5654	16年人源沙门菌流行现状	epidemic status and etiolog	,何冬梅,谭海玲,李柏生,	gmei,Tan Hailing,Li Baishen	中华流行病学杂志	inese Journal of Epidemiolo	39	1	63-66	2018	sn.0254-6450.2018.01.013
5655	门菌血清型、耐药谱及P	types, drug resistance spec	妮,耿英芝,魏彤竹,李雪,	eng Yingzhi,Wei Tongzhu,L	中国微生态学杂志	inese Journal of Microecolo	30	3	268-272	2018	381/j.cnki.cjm.201803005
5656	常见病原菌分布和耐药	ce characteristics of comm	王靖然.	Wang Jingran.	基层医学论坛	Primary Medicine Forum	22	7	961-962	2018	/j.1672-1721.2018.07.060
5657	腹泻沙门菌分离鉴定与耐	g resistance analysis of Sal	刘燕.	Liu Yan.	中国兽医杂志	Chinese Veterinary Journal	54	6	96-98	2018	-
5658	地区儿童感染性腹泻病原	veillance results of childho	雪飞,叶艳华,金萍,王炜,丁	Ye Yanhua,Jin Ping,Wang W	实用预防医学	ractical preventive medicin	25	12	1508-1510	2018	sn.1006-3110.2018.12.029
5659	婴幼儿食源性腹泻流行病	thogenic factors of foodbo	刘大晶,郭学斌,陈少岩.	ajing,Guo Xuebin,Chen Sha	中国食品卫生杂志	hinese Food Hygiene Journ	30	3	279-282	2018	.13590/j.cjfh.2018.03.012

5660	沙门菌分离株的耐药谱及	related genes of Salmonel	峰,方毅,杨丽华,刘文健,赵	ang Yi,Yang Lihua,Liu Wenj	检验医学	laboratory medicine	33	12	1098-1102	2018	sn.1673-8640.2018.12.009
5661	4年-2017年食源性疾病监	Analysis of annual foodbor	戴黎萍.	Dai Liping.	中国卫生检验杂志	ese Journal of Health Inspe	28	21	2655-2657	2018	-
5662	-2016年哨点医院食源性疾	sis of foodborne disease su	郑晓燕.	Zheng Xiaoyan.	海峡预防医学杂志	s Journal of Preventive Me	24	3	40-42	2018	-
5663	门菌的分离鉴定、血清分	g resistance analysis of Sal	斌,邱荣超,张丽娜,符颖,引	Rongchao,Zhang Lina,Fu Yi	中国兽医杂志	Chinese Veterinary Journal	54	4	79-82,86	2018	-
5664	菌的耐药表型、基因型及	otypes and epidemic char	建皓,张小荣,曹永忠,巢国	ng Xiaorong,Cao Yongzhong	中国人兽共患病学报	Chinese Journal of Zoonose	34	2	150-157,164	2018	sn.1002-2694.2018.00.018
5665	区重点品种食品安全状况	from the monitoring of fo	叶石兴,徐伟兵.	Ye Shixing,Xu Weibing.	中国医药科学	Chinese Medical Sciences	8	14	213-215	2018	sn.2095-0616.2018.14.067
5666	粪便分离的肠炎沙门菌的	and aggregation typing of S	兴,李宁沙,陈立华,徐丹,夏	i Ningsha,Chen Lihua,Xu Da	实用预防医学	ractical preventive medicin	25	4	499-501	2018	sn.1006-3110.2018.04.032
5667	自治区伤寒沙门菌脉冲场	sis molecular typing of Sal	周海健,刁保卫,木塔里甫·	Haijian,Diao Baobao,Mutal	疾病监测	disease surveillance	33	5	402-406	2018	sn.1003-9961.2018.05.012
5668	健康体检沙门氏菌检测结果	esults of annual employee	胡静华.	Hu Jinghua.	中国卫生产业	China health industry	15	3	126-127	2018	ki.1672-5654.2018.03.126
5669	鸡蛋沙门氏菌污染的研究	of local eggs in Wenzhou	ATED HOSPITAL OF WE	ST AFFILIATED HOSPIT	温州科技职业学院学报	Vocational College of Scie	-	4	26-29	2018	-
5670	肉鸡沙门菌血清型分布及	tion of Salmonella serotype	,周莉,李向力,谭静,平洋,	Li,Li Xiangli,Tan Jing,Pingy	中国家禽	Chinese poultry	40	5	76-80	2018	sn.1004-6364.2018.05.018
5671	5年学校食源性疾病暴发	analysis of annual school f	,杨淑香,付萍,韩海红,刘	uxiang,Fu Ping,Han Haihon	中国学校卫生	Chinese school health	39	4	570-572,576	2018	ki.1000-9817.2018.04.026
5672	市售肉制品中沙门菌耐药	typing of Salmonella in co	刘诚,朱颖莹,刘弘,陈敏,许	u Yingying,Liu Hong,Chen	中国食品卫生杂志	hinese Food Hygiene Journ	30	2	132-138	2018	).13590/j.cjfh.2018.02.002
5673	市宠物源沙门氏菌耐药性	istant genes of Salmonella	亚军,郭菲,夏利宁,姚晓	un,Guo Fei,Xia Lining,Yao X	中国农业大学学报	al of China Agricultural Univ	23	7	75-83	2018	ssn.1007-4333.2018.07.09
5674	宰环节沙门菌污染的定量	of Salmonella contaminati	黄秀梅,李月华,盖文燕,	ng Xiumei,Li Yuehua,Gai W	农产品质量与安全	ultural product quality and	-	2	21-25	2018	sn.1674-8255.2018.02.005
5675	用汉堡引起食源性疾病事	ident of foodborne illness	王玉俊.	Wang Yujun.	中国卫生标准管理	a Health Standard Manage	9	3	15-16	2018	sn.1674-9316.2018.03.008
5676	国伤寒/副伤寒流行特征及	ic characteristics and mole	凤,廖巧红,李杰,刁保卫,樊	ao Qiaohong,Li Jie,Diao Ba	中华流行病学杂志	inese Journal of Epidemiol	39	3	337-341	2018	sn.0254-6450.2018.03.017
5677	汉中市食品中沙门氏菌污	almonella Contamination i	雅丽,刘红丽,高洁,张木	Yali,Liu Hongli,Gao Jie,Zha	医学动物防制	medical animal control	34	1	31-33	2018	0.7629/yxdwfz201801009
5678	广州市4大类食品食源性致	n status of foodborne pat	晓华,余超,罗健梅,李迎	ua,Yu Chao,Luo Jianmei,Li	职业与健康	Occupation and health	34	3	332-335	2018	-
5679	川省德尔卑沙门菌耐药与	e and molecular typing of S	雷高鹏,黄伟峰,黄玉兰,杨	eng,Huang Weifeng,Magno	中国食品卫生杂志	hinese Food Hygiene Journ	30	6	570-576	2018	).13590/j.cjfh.2018.06.004
5680	重耐药肯塔基沙门菌的检	ltidrug-resistant Salmonel	高红,章丹阳,闫鹏,沈玄艺	g,Zhang Danyang,Yan Peng	疾病监测	disease surveillance	33	6	510-514	2018	-
5681	性心内膜炎1例病例报告	endocarditis1Case report	彪,王永刚,田雅茹,牟劲松	ang Yonggang,Tian Yaru,M	中国社区医师	hinese community physicia	34	29	79-81,83	2018	sn.1007-614x.2018.29.048
5682	腹泻患者病原微生物监测	croorganism monitoring re	,郭珊,刘玲,曹献芹,袁燕,	an,Liu Ling,Cao Xianqin,Yua	中国病原生物学杂志	ese Journal of Pathogen Bi	13	5	536-538,542	2018	10.13350/j.cjpb.180519
5683	泻患者粪便标本病原菌检	alysis of pathogenic bacter	邢亚东,刘纯成,李兵兵,金	dong,Liu Chuncheng,Li Bing	现代预防医学	modern preventive medicin	45	6	1095-1101	2018	-
5684	骨髓及胸腹水标本病原菌	teria distribution and drug	沈晖,邬靖敏.	Shen Hui,Wu Jingmin.	中国艾滋病性病	AIDS and STDs in China	24	3	227-229,264	2018	419/j.cnki.aids.2018.03.04
5685	引起的都柏林沙门氏菌食	se of salmonella food pois	,王春艳,祖文刚,左志平,	Chunyan,Zu Wengang,Zuo	医学动物防制	medical animal control	34	1	77-79	2018	0.7629/yxdwfz201801026
5686	市售5类食品中5种食源性	5in foods5Analysis of mon	,余峰玲,苗升浩,许猛,张	g,Miao Shenghao,Xu Meng	实用预防医学	ractical preventive medicin	25	12	1524-1527	2018	sn.1006-3110.2018.12.034
5687	某市医疗机构污水消毒效	stewater Disinfection Effe	芳盈,杜俊,谭琳琳,刘晓	angying,Du Jun,Tan Linlin,	中国消毒学杂志	hinese Journal of Disinfecti	35	8	635-637	2018	sn.1001-7658.2018.08.029
5688	道感染沙门菌检测及药物	tion in children in Tianjin a	梁帆,穆云.	Liang Fan,Mu Yun.	中国处方药	Chinese prescription drugs	16	11	45-46	2018	n.1671-945X.2018.11.027
5689	食物中毒患者微生物学检	al test results in patients w	齐莹莹.	Qi Yingying.	河南预防医学杂志	n Journal of Preventive Me	29	12	928-929	2018	m.1006-8414.2018.12.018
5690	鸡沙门氏菌的分离鉴定及	esistance investigation of	颜卫,安楠,魏雨琼,周宇婷	,An Nan,Wei Yuqiong,Zhou	现代农业科技	odern agricultural technolo	-	1	225232	2018	sn.1007-5739.2018.01.137
5691	鸡肉与烧鸡中沙门氏菌污	nation in chilled chicken a	,闫龙刚,朱瑶迪,袁乾乾,	gang,Zhu Yaodi,Yuan Qian	安徽农学通报	ui Agricultural Science Bull	24	20	96-97,111	2018	sn.1007-7731.2018.20.039
5692	北京市海淀区食源性疾病	veillance of foodborne dis	婧,张娜,刘伟,韩思媛,纪黎	Zhang Na,Liu Wei,Han Siyu	职业与健康	Occupation and health	34	16	2207-2211	2018	-
5693	省沙门菌血清型分布特征	ristics and molecular typin	祖顺,曹国林,金莉,任翔,	shun,Cao Guolin,Jinli,Ren X	中华微生物学和免疫学杂	rnal of Microbiology and I	38	4	285-292	2018	sn.0254-5101.2018.04.008
5694	株肠炎沙门菌耐药情况分	g resistance of Salmonella	招福,李美华,曹春远,廖琳	Li Meihua,Cao Chunyuan,L	河南预防医学杂志	n Journal of Preventive Me	29	10	737-739	2018	m.1006-8414.2018.10.005
5695	性腹泻病原菌分子分型及	analysis of pathogenic bact	魏晓光,徐纪茹,栾阳.	i Xiaoguang,Xu Jiru,Luanya	中国病原生物学杂志	ese Journal of Pathogen Bi	13	12	1327-1334	2018	10.13350/j.cjpb.181207
5696	合征合并败血症患者66例	mbined with sepsis66Clinic	,梁飞立,李玫蓉,方敏,吴	g Feili,Li Meirong,Fang Mi	验和临床感染病杂志（电	ntal and Clinical Infectious	12	3	296-300	2018	sn.1674-1358.2018.03.019

5697	肉及肉制品中沙门氏菌污	ntamination status and dru	靓,李兵兵,邢亚东,李军康	i Bingbing,Xing Yadong,Li J	食品安全质量检测学报	f Food Safety and Quality I	9	3	659-663	2018	sn.2095-0381.2018.03.034
5698	对体检人员健康体检结果	tion results of physical exa	王敏.	Wang Min.	中国卫生产业	China health industry	15	4	143-144	2018	ki.1672-5654.2018.04.143
5699	惠阳区重点品种食品安全	d safety status of key varie	徐伟兵,叶石兴,李伟旗.	Weibing,Ye Shixing,Li Wei	中国医药科学	Chinese Medical Sciences	8	7	135-137,200	2018	sn.2095-0616.2018.07.041
5700	西省食源性沙门菌分子分	resistance analysis of foo	彭思露,刘道峰,游兴勇,文	ilu,Liu Daofeng,You Xingyo	中国消毒学杂志	hinese Journal of Disinfecti	35	10	764-769	2018	sn.1001-7658.2018.10.015
5701	沙门菌多位点序列及耐药	ug resistance characteristi	王庭庭,谈华,李玫,金玉,郭	Talk about Hua,Li Mei,gol	临床儿科杂志	Journal of Clinical Pediatric	36	7	520-523	2018	sn.1000-3606.2018.07.011
5702	一起普沙门菌引起的食物中毒	of food poisoning caused	王清,王小林,白梅.	Qing,Wang Xiaolin,white	医学食疗与健康	dical Diet Therapy and Hea	-	2	64,67	2018	-
5703	病合并都柏林沙门菌感染	AIDS combined with Salm	杨翠先,高丽,李正伦,谢袓	Cuixian,Goryeo,Li Zhenglun	检验医学与临床	boratory medicine and clini	15	20	3161-3163	2018	sn.1672-9455.2018.20.052
5704	西藏农区犊牛死亡原因调查	uses of death of calves in a	赵丽,陈晓英.	Zhao Li,Chen Xiaoying.	畜牧与饲料科学	Livestock and feed science	39	12	92-94	2018	-
5705	4-2016年食源性致病菌监	sis of annual foodborne pa	张丽萍,高涛,薛莉,武永平,	ng,Gao Tao,Xue Li,Wu Yon	实用预防医学	ractical preventive medici	25	10	1239-1241	2018	sn.1006-3110.2018.10.025
5706	市延庆区感染性腹泻病原	the etiology of infectious d	敢,王全意,曲梅,刘凡,赵璐	g Quanyi,Qu Mei,Liu Fan,Z	中国卫生检验杂志	ese Journal of Health Inspe	28	7	855-858	2018	-
5707	市吴江区食品中食源性致	foodborne pathogenic bac	严坤.	Yan Kun.	医学信息文摘（连续型电	information abstract (cont	18	68	27-28,31	2018	ki.1671-3141.2018.68.012
5708	炎沙门菌耐药分析与分子	and molecular typing of Sal	张秋香,杨红霞,王洋,韩吉	g,Yang Hongxia,Wang Yan	中华微生物学和免疫学杂	rnal of Microbiology and I	38	7	505-509	2018	sn.0254-5101.2018.07.005
5709	治疗儿童鼠伤寒沙门菌肠	dium in the treatment of S	付毅.	Fu Yi.	当代医药论丛	ontemporary Medicine Seri	16	16	114-115	2018	sn.2095-7629.2018.16.080
5710	直辖市)市售生畜肉中常见	contamination status of co	杰,杨庆文,陈伟伟,杨小蓉,	Qingwen,Chen Weiwei,Ya	卫生研究	health research	47	3	498-501,511	2018	-
5711	禽肉蛋产品中食源性致病	ic bacteria in livestock, po	树秋,王文博,邓立刚,李鸳	qiu,Wang Wenbo,Deng Lig	山东农业科学	andong Agricultural Scienc	50	7	38-41	2018	sn.1001-4942.2018.07.007
5712	南昌市食源性致病菌监测	n foodborne pathogen surv	华,赖玉珍,裴小丽,周丽,刘	a,Lai Yuzhen,Pei Xiaoli,Zho	医学食疗与健康	dical Diet Therapy and Hea	-	6	165	2018	-
5713	年防城港市食源性致病菌	ults of foodborne pathoge	,李叶青,李林柯,蒙国伟,文	eqing,Li Linke,Meng Guow	职业与健康	Occupation and health	34	3	329-331	2018	-
5714	沙门氏菌引起的食物中毒	e of food poisoning cause	苏芸.	Su Yun.	中国社区医师	hinese community physicia	34	17	137139	2018	sn.1007-614x.2018.17.085
5715	伤寒沙门菌肠炎临床特点	nd drug resistance of Salm	杜敏,谢晓丽.	Du Min,Xie Xiaoli.	儿科药学杂志	ournal of Pediatric Pharma	24	9	12-14	2018	p.1672-108X.2018.09.004
5716	京市顺义区腹泻病例中沙	of Salmonella among diarr	李颖,张爽,王园园,朱美娟	g Shuang,Wang Yuanyuan	首都公共卫生	capital public health	12	5	256-258	2018	-
5717	江阴市1起食源性伤寒暴发	gation into the outbreak of	姚建香,顾敏华,颜雅娟.	ianxiang,Gu Minhua,Yan Ya	职业与健康	Occupation and health	34	14	1986-1988	2018	-
5718	宁县域内食源性疾病监测	alysis of foodborne disease	陈彩丽.	Chen Caili.	饮食保健	Diet and health care	5	31	282	2018	sn.2095-8439.2018.31.353
5719	沙门氏菌污染状况及血清	on status and serotyping a	谢作蓉,林宝英,高云慨.	uorong,Lin Baoying,Gao Yu	现代食品	modern food	-	11	96-99	2018	cn41-1434/ts.2018.11.032
5720	奉贤区188株沙门菌分子分	i188Study on molecular ty	张宏伟,陈洪友,王仁刚,陶力	wei,Chen Hongyou,Wang R	预防医学情报杂志	of Preventive Medicine Info	34	11	1391-1395	2018	-
5721	省家禽疫病流行情况初步	epidemic situation of pou	,和春林,赵蓉,李珂,李敏,林	Chunlin,Zhao Rong,Li Ke,Li	云南畜牧兽医	nal Husbandry and Veterina	-	5	9-10	2018	sn.1005-1341.2018.05.006
5722	医院感染性腹泻常见病原	thogenic bacteria in infecti	丁丽萍,柏雪莲.	Ding Liping,Bai Xuelian.	预防医学论坛	Preventive Medicine Forum	24	7	541-543	2018	-
5723	自治区食源性沙门菌的耐	spectrum of foodborne S	吕素玲,杜悦,韦程媛,瞿聪	ling,Du Yue,Wei Chengyua	中国食品卫生杂志	hinese Food Hygiene Journ	30	1	22-27	2018	.13590/j.cjfh.2018.01.005
5724	伤寒沙门菌血清凝集及PFG	murium serum agglutinati	崔仑标,周品众,张宏宾,高	,Zhou Pinzhong,Zhang Ho	医学动物防制	medical animal control	34	1	46-48	2018	0.7629/yxdwfz201801014
5725	病和食源性疾病患者粪便中	lla in the feces of patients	群,黄邵军,苗超,袁士杰,罗	g Shaojun,Miao Chao,Yua	疾病监测	disease surveillance	33	11	950-954	2018	sn.1003-9961.2018.11.016
5726	市食源性疾病致病病原体	racteristics of foodborne d	陈晓敏,刘晗,过晓阳,甄世祺	in,Liu Han,Guo Xiaoyang,Z	职业与健康	Occupation and health	34	1	48-51	2018	-
5727	州市网络订餐餐饮食品卫生	situation of online orderi	周露,丁清龙,梁颖思,梁秋华	g Qinglong,Liang Yingsi,Lia	食品安全质量检测学报	f Food Safety and Quality I	9	12	2935-2940	2018	sn.2095-0381.2018.12.010
5728	来源的非伤寒沙门菌对β-	yphoidal Salmonella specie	詹晔斐,许兆军,宋启发.	n Yefei,Xu Zhaojun,Song Q	中国食品卫生杂志	hinese Food Hygiene Journ	30	3	239-244	2018	.13590/j.cjfh.2018.03.004
5729	丰阿勒泰市细菌性腹泻病	cterial diarrheal disease su	徐德英,汤景荣.	Xu Deying,Tang Jingrong.	中国卫生检验杂志	ese Journal of Health Inspe	28	19	2404-2405	2018	-
5730	从业人员携带沙门菌血清	es and drug susceptibility	李桃,张毓瑜,李学红,张柱	hang Yuyu,Li Xuehong,Zha	中国卫生检验杂志	ese Journal of Health Inspe	28	21	2602-2604	2018	-
5731	分地区生鲜畜禽肉中食源	odborne pathogenic bacte	安,冯世文,陈泽祥,柳锋,胡	ng Shiwen,Chen Zexiang,L	动物医学进展	vances in Veterinary Medic	39	4	132-136	2018	sn.1007-5038.2018.04.028
5732	沙门菌感染性主动脉瘤1例	a-infected aortic aneurysm	成朔,曹莉芳,王立旗,邓娟	shuo,Cao Lifang,Wang Liqi,	中国感染与化疗杂志	urnal of Infection and Che	18	2	209-210	2018	/j.1009-7708.2018.02.016
5733	菌的血清型分布和对黏菌	in resistance of Salmonella	萍,宋立,崔明全,赵琪,徐	g,Song Li,Cui Mingquan,Zh	中国兽药杂志	ese Journal of Veterinary D	52	1	13-18	2018	SN.1002-1280.2018.01.03

5734	沙门菌引起食物中毒的病	case of food poisoning cau	刘佳,夏玛丽,胡俊.	Liu Jia,Mary Xia,Hu Jun.	中国卫生检验杂志	ese Journal of Health Inspe	28	10	1254-1255	2018	-
5735	又中食品中食源性致病菌	s of foodborne pathogenic	张志强,刘红丽,高洁,张木林	ang,Liu Hongli,Gao Jie,Zhar	预防医学情报杂志	of Preventive Medicine Info	34	4	445-448	2018	-
5736	源性相关腹泻沙门菌种分	Salmonella species in food	叶映月,陈志杭,王海明,叶	Chen Zhihang,Wang Haimi	中国卫生检验杂志	ese Journal of Health Inspe	28	24	2979-2981	2018	-
5737	年我国婴幼儿配方乳粉抽	ection situation of infant fo	黄湘鹭,邢书霞.	Huang Xianglu,Xing Shuxia.	食品安全质量检测学报	f Food Safety and Quality I	9	9	2245-2251	2018	sn.2095-0381.2018.09.045
5738	珠海市肠炎沙门菌分子分	ping and drug resistance o	艳梅,魏泉德,陈红涛,张晟	ei Quande,Chen Hongtao,Z	中国卫生检验杂志	ese Journal of Health Inspe	28	13	1573-1575,1595	2018	-
5739	国大陆食源性疾病暴发监	disease outbreak surveilla	进军,刘长青,熊鹰,李宁,徐	u Changqing,Xiong Ying,Li	中国食品卫生杂志	hinese Food Hygiene Journ	30	3	293-298	2018	).13590/j.cjfh.2018.03.015
5740	北猪场沙门菌的分离鉴定	analysis of Salmonella fro	斌,邵靓,梁璐琪,周莉媛,邓	Liang,Liang Luqi,Zhou Liyu	畜禽业	vestock and poultry indust	29	8	1-3	2018	ki.1008-0414.2018.08.001
5741	环城某区食源性疾病病原	ase pathogen detection re	孟庆贺,王淑惠,石明英.	Qinghe,Wang Shuhui,Shi Mi	河南预防医学杂志	n Journal of Preventive Me	29	11	841-843,849	2018	m.1006-8414.2018.11.013
5742	成都市双流区57株人源沙	engdu57Characteristic ana	,郭冬梅,杨小蓉,刘昌玲,	mei,Yang Xiaorong,Liu Cha	职业与健康	Occupation and health	34	17	2343-2346	2018	-
5743	为患者沙门菌感染状况和	tus and drug resistance in	金金,杨虹,吴延杰,罗泳仪,	,Yang Hong,Wu Yanjie,Luo	中国人兽共患病学报	Chinese Journal of Zoonose	34	6	532-536,541	2018	sn.1002-2694.2018.00.092
5744	市腹泻病综合监测体系构	urveillance based on Shan	,肖文佳,黎健,林声,陈敏,	enjia,Li Jian,Lin Sheng,Chen	中华传染病杂志	se Journal of Infectious Dis	36	6	327-332	2018	sn.1000-6680.2018.06.002
5745	品加工从业人员沙门氏菌	status among food process	徐伟兵,叶石兴.	Xu Weibing,Ye Shixing.	首都食品与医药	Capital Food and Medicine	25	13	76-77	2018	sn.1005-8257.2018.13.068
5746	的耐药性及超广谱β-内酰	um of clinically isolated Sal	黄瑛,张新,吕冰,钱海坤,王	,Zhang Xin,Lu Bing,Qian Ha	中国感染控制杂志	ese Journal of Infection Co	17	6	496-501	2018	sn.1671-9638.2018.06.007
5747	伤寒106例临床分析	oid fever106Case clinical ar	陈维迪,陆亚君.	Chen Weidi,Lu Yajun.	中国乡村医药	Chinese rural medicine	25	17	9-10	2018	sn.1006-5180.2018.17.005
5748	封市地方食品花生糕微生	Microbial Contamination of	冯辉,黄淑华,许娇.	ng Hui,Huang Shuhua,Xu Ji	河南预防医学杂志	n Journal of Preventive Me	29	12	950-952	2018	m.1006-8414.2018.12.027
5749	畜类食品中沙门氏菌污染	and livestock foods in Jiadi	敏,陈培超,陈伟鑫,顾丽萍,	Peichao,Chen Weixin,Gu L	上海预防医学	hanghai Preventive Medici	30	9	755-758	2018	28/j.cnki.sjpm.2018.18418
5750	沙门菌食物中毒34例临床	itidis food poisoning34Cas	袁强,田辉.	Yuan Qiang,Tian Hui.	中华传染病杂志	se Journal of Infectious Dis	36	8	496-498	2018	sn.1000-6680.2018.08.008
5751	年郴州市食品中食源性致	ysis of foodborne pathoge	军,郑文,李征莉,段朝标,	ng Wen,Li Zhengli,Duan Ch	中国卫生检验杂志	ese Journal of Health Inspe	28	8	1013-1015,1022	2018	-
5752	腹泻的260例病原微生物检	ren260Analysis of pathoge	张仙云.	Zhang Xianyun.	黑龙江中医药	jiang Traditional Chinese M	47	5	165-166	2018	-
5753	区肉鸡场饮用水细菌污染	mination of drinking water	袁东芳,沈美艳,李汝春,李	fang,Shen Meiyan,Li Ruch	黑龙江畜牧兽医（下半月）	dry and Veterinary Medicin	-	2	91-92	2018	cnki.hljxmsy.2017.03.0483
5754	市售食品常见食源性致病	tus of common foodborne	顾文辉,刘军.	Gu Wenhui,Liu Jun.	河南预防医学杂志	n Journal of Preventive Me	29	7	561-564	2018	m.1006-8414.2018.07.030
5755	门氏菌耐药性及耐药基因	drug-resistant genes of Sa	海燕,仇永凤,胡素娟,卫玲玲	Qiu Yongfeng,Hu Sujuan,We	生物技术通报	biotechnology bulletin	34	3	170-176	2018	otech.bull.1985.2017-0743
5756	菌感染的临床特点及其对	lla infection in children and	黄振华,叶丽娟.	Huang Zhenhua,Ye Lijuan.	抗感染药学	Anti-infective Pharmacy	15	1	30-34	2018	sn.1672-7878.2018.01-009
5757	治区市售肉及肉制品食源	cteria in commercially sold	艺红,蒋玉艳,李秀桂,钟延	,Jiang Yuyan,Li Xiugui,Zhon	中国食品卫生杂志	hinese Food Hygiene Journ	30	4	411-414	2018	).13590/j.cjfh.2018.04.015
5758	京市特色小吃中食源性致	sults of foodborne pathoge	王迪,赵耀,张晓媛,陈倩,崔	ao Yao,Zhang Xiaoai,Chen	中国卫生检验杂志	ese Journal of Health Inspe	28	17	2162-2164	2018	-
5759	餐饮从业人员沙门菌带菌	rrier status among catering	勇,刘正奇,周厚德,刘洋,刘	Zhengqi,Zhou Houde,Liu Y	中国消毒学杂志	hinese Journal of Disinfecti	35	6	448-450	2018	sn.1001-7658.2018.06.016
5760	肉牛源沙门菌的分离鉴定	alysis of Salmonella origina	斌,张晓玲,姚刚,克然木·	oling,Yao Gang,Kerim·Tohu	动物医学进展	vances in Veterinary Medic	39	8	15-19	2018	sn.1007-5038.2018.08.004
5761	寒沙门氏菌PFGE分子分型	nnan ProvincePFGEMolec	邹颜秋硕,金莉,赵江,任翔,	nqiushuo,Jinli,Zhao Jiang,Re	昆明医科大学学报	al of Kunming Medical Univ	39	8	23-27	2018	sn.1003-4706.2018.08.006
5762	深圳市食源性疾病流行病	haracteristics of foodborne	刘建平,张锦周,潘柳波,梁	g,Zhang Jinzhou,Pan Liubo,	职业与健康	Occupation and health	34	21	2913-2916	2018	-
5763	食堂及小卖部食品和 餐具	d canteen food andAnalysi	郭正梅,杜楠,黄敏.	Zhengmei,Du Nan,Huang	实用预防医学	ractical preventive medicin	25	8	946-949	2018	sn.1006-3110.2018.08.013
5764	香炉山鸡白痢血清学调查	estigation of pullorum in Xi	涛,吴冬梅,刘铭英,杨先富,	ongmei,Liu Mingying,Yang	贵州畜牧兽医	mal Husbandry and Veterin	42	6	60-61	2018	sn.1007-1474.2018.06.021
5765	年鼠伤寒沙门菌感染的脉	el electrophoresis analysis	,陈曦,肖波,王斌,孙松松,	Xiao Bo,Wang Bin,Sun Song	实用预防医学	ractical preventive medicin	25	1	82-84	2018	sn.1006-3110.2018.01.023
5766	区儿童腹泻病原菌分布及	sis of pathogenic bacteria i	杨英贤,虞斌,李玉珍,温伟	xian,Yu Bin,Li Yuzhen,Wen	深圳中西医结合杂志	egrated Traditional Chinese	28	3	198-198,封3	2018	ki.1007-0893.2018.03.098
5767	导等温扩增方法在食品卫	ing-mediated isothermal a	朱根忠.	Zhu Genzhong.	心血管外科杂志（电子版）	rdiovascular Surgery (elect	7	4	755-756	2018	sn.2095-2260.2018.04.105
5768	便培养菌群构成比及耐药	g resistance analysis of ped	卓丽文.	Zhuo Liwen.	包头医学院学报	rnal of Baotou Medical Coll	34	8	21-22,34	2018	3/j.cnki.jbmc.2018.08.007
5769	年-2015年医疗机构污水监	of annual wastewater mor	何学军,李金,蒋国钦,傅利	ejun,Li Jin,Jiang Guoqin,Fu	中国卫生检验杂志	ese Journal of Health Inspe	28	11	1381-1383	2018	-
5770	省地方特色食品微生物污	ntamination of local specia	吴玲玲,邱正勇,崔莹,张秀	,Qiu Zhengyong,Cui Ying,Zh	河南预防医学杂志	n Journal of Preventive Me	29	7	553-557	2018	m.1006-8414.2018.07.028

5771	区腹泻样本细菌病原谱学	pectrum monitoring results	尹德秀,郝向荣.	Yin Dexiu,Hao Xiangrong.	医学信息文摘（连续型电	information abstract (cont	18	A2	238240	2018	i.1671-3141.2018.102.117
5772	明地区儿童血源性感染细	nce characteristics of bloo	斌,张铁松,肖曙芳,周琳,苏	Tiesong,Xiao Shufang,Zhou	儿科药学杂志	ournal of Pediatric Pharmac	24	9	46-48	2018	p.1672-108X.2018.09.015
5773	炎沙门菌所致食物中毒的	of food poisoning caused	可云,陈海滨,曹春远,廖琳虹	Haibin,Cao Chunyuan,Liao	河南预防医学杂志	n Journal of Preventive Me	29	11	862-864	2018	m.1006-8414.2018.11.020
5774	鸡死胚中沙门菌的分离鉴定	is of Salmonella from dead	炜,卢建洲,陈益,张冲,刘永	zhou,Chen Yi,Zhang Chong,	中国预防兽医学报	rnal of Preventive Veterina	40	10	891-896	2018	ssn.1008-0589.201805001
5775	门菌血清分型及脉冲场凝胶	unnan Province and finger	,闵向东,蔡同建,韩兴孟,杨	angdong,Cai Tongjian,Han	昆明医科大学学报	al of Kunming Medical Univ	39	2	30-33	2018	sn.1003-4706.2018.02.007
5776	污染引起的食物中毒的实验	a case of food poisoning c	白李明,杨志强,叶聿程.	ming,Yang Zhiqiang,Ye Yuc	食品安全导刊	food safety guide	-	27	84-85	2018	sn.1674-0270.2018.27.065
5777	寒沙门氏菌引起的食物中毒	oisoning incident caused b	世雄,赖天兵,蔡亚辉,娄常	Lai Tianbing,Cai Yahui,Lou C	医学动物防制	medical animal control	34	9	879-882	2018	0.7629/yxdwfz201809018
5778	6年阜新市食源性致病菌监	toring results of foodborne	,修敏,任妍妍,蔡楠楠,刘文	Min,Ren Yanyan,Cai Nannan	河北医学	Hebei Medicine	24	9	1577-1582	2018	sn.1006-6233.2018.09.046
5779	门菌污染聚餐食品所致食物	ood Poisoning Caused by S	郝林会,徐闻,古文鹏,字桂	hui,Xu Wen,Gu Wenpeng,Z	中国食品卫生杂志	hinese Food Hygiene Journ	30	6	659-662	2018	).13590/j.cjfh.2018.06.021
5780	携带沙门菌的血清型分布	sceptibility test of Salmone	鲍虎,张莉,杨书才,周杰,张	,Zhang Li,Yang Shucai,Zhou	检验医学与临床	boratory medicine and clini	15	12	1741-1743	2018	sn.1672-9455.2018.12.013
5781	快速诊断伤寒沙门菌血流	monella typhi bloodstream	逸敏,马逸珉,金嘉琳,张文	Ma Yimin,Jin Jialin,Zhang W	中华传染病杂志	se Journal of Infectious Dis	36	1	48-49	2018	sn.1000-6680.2018.01.015
5782	年间沙门氏菌食物中毒事件	teristics of Salmonella food	,高雪丽,郭卫芸,王永辉,孙	ueli,Guo Weiyun,Wang Yon	食品工业	food industry	39	5	253-255	2018	-
5783	病合并沙门菌骨髓炎一例并	omplicated by Salmonella	覃文,忻霞菲,周丽,王庭辉	n,Xin Xiafei,Zhou Li,Wang T	中国全科医学	Chinese general medicine	21	5	616-619	2018	sn.1007-9572.2017.00.163
5784	伤寒沙门氏菌培养结果与	of Salmonella typhoid and	陈蔚冰,王立芸.	Chen Weibing,Wang Liyun.	医学信息	medical information	31	9	157-158	2018	sn.1006-1959.2018.09.051
5785	市售发酵与非发酵豆制品	ilable fermented and non-	,王路梅,苗升浩,许猛,王悦	i,Miao Shenghao,Xu Meng,	食品安全质量检测学报	f Food Safety and Quality I	9	20	5525-5530	2018	sn.2095-0381.2018.20.044
5786	至2017年食源性疾病暴发	2017Annual surveillance an	闵向东,张强,万蓉,蔡同建,刘	ong,Zhang Qiang,Wanrong,	昆明医科大学学报	al of Kunming Medical Univ	39	6	118-123	2018	sn.1003-4706.2018.06.025
5787	鼠伤寒沙门菌感染临床分析	onella typhimurium infecti	罗文雄,高利伟,刘鸿.	Wenxiong,Gao Liwei,Liu H	中国社区医师	hinese community physicia	34	30	21,24	2018	sn.1007-614x.2018.30.010
5788	鲁木齐市肠炎沙门菌耐药	nce and molecular typing	,王超,陈国利,巴特尔,吴亚	ng Chao,Chen Guoli,Battle,	医学动物防制	medical animal control	34	10	962-964,968	2018	0.7629/yxdwfz201810014
5789	肠炎沙门菌的监测及耐药	hickens in Guangzhou and	双,杨伟聪,张丽娜,符颖,詹	,Yang Weicong,Zhang Lina	畜牧兽医学报	mal Husbandry and Veterir	49	2	388-395	2018	sn.0366-6964.2018.02.018
5790	年腹泻病患者沙门菌分离	e of Salmonella isolates fro	颖华,陈秀华,刘芸,童锐,陈	Chen Xiuhua,Liu Yun,Tong	中国卫生检验杂志	ese Journal of Health Inspe	28	11	1288-1292,1298	2018	-
5791	广西报告的食源性疾病暴	borne disease outbreaks r	,石萌萌,姚雪婷,钟延旭,	gmeng,Yao Xueting,Zhong	现代预防医学	modern preventive medicin	45	21	4013-4017	2018	-
5792	上海三林地区儿童腹泻病	athogenic spectrum of chil	华,刘芳,陈敏,孙建萍,丁	Fang,Chen Min,Sun Jianpi	实用预防医学	ractical preventive medicin	25	7	886-889	2018	sn.1006-3110.2018.07.035
5793	6年盐城市食源性致病菌	lts of foodborne pathogen	郜明明,陆金凤,吴巨飞.	Mingming,Lu Jinfeng,Wu J	职业与健康	Occupation and health	34	15	2060-2063	2018	-
5794	及动物医院猪源沙门菌分	ating from pigs in some sla	晓雷,周金柱,王小波,高清	ai Xiaolei,Zhou Jinzhu,Wan	中国兽医学报	al of Chinese Veterinary Me	38	3	508-514	2018	nki.1005-4545.2018.03.14
5795	鸡、猪源沙门氏菌血清型	Salmonella originating fro	月华,赵建梅,张青青,曲志	hao Jianmei,Zhang Qingqin	中国动物检疫	China Animal Quarantine	35	6	30-36	2018	n.1005-944X.2018.06.009
5796	台市哨点医院食源性疾病	disease surveillance results	,孙月琳,王朝霞,宫春波,董	in,Wang Chaoxia,Gong Chu	现代预防医学	modern preventive medicin	45	18	3414-3417,3433	2018	-
5797	原菌流行病学及耐药特征	ristics and detection techn	霞,张树永,王慧珠,娄金丽	ang Shuyong,Wang Huizhu	中国抗生素杂志	hinese Journal of Antibiotic	43	9	1083-1088	2018	sn.1001-8689.2018.09.004
5798	武汉市食源性疾病监测结	borne disease surveillance	雅婷,石梦蝶,王肖,王怀记	Shi Mengdie,Wang Xiao,W	公共卫生与预防医学	Health and Preventive Me	29	5	93-96	2018	sn.1006-2483.2018.05.025
5799	患者血液培养病原体分布	ution and drug resistance i	储从家,吴惠玲.	Chu Congjia,Wu Huiling.	实用预防医学	ractical preventive medicin	25	7	872-873	2018	sn.1006-3110.2018.07.030
5800	腹泻的150例病原微生物检	ren150Analysis of pathoge	李佳,尹庆霞,赖佼.	Li Jia,Yin Qingxia,Lai Jiao.	医药前沿	Medical Frontier	8	16	210	2018	sn.2095-1752.2018.16.177
5801	治区部分地区鲜奶吧乳品	milk bars in some areas o	乌伊罕,王利平,张冰冰.	an,Wang Liping,Zhang Bing	中国卫生检验杂志	ese Journal of Health Inspe	28	21	2682-2684	2018	-
5802	猪源沙门氏菌的分离鉴定	analysis of Salmonella origi	艾鑫,董欣炜.	Ai Xin,Dong Xinwei.	四川畜牧兽医	nal Husbandry and Veterin	-	9	28-30	2018	-
5803	肠炎沙门菌耐药性及分子分	g characteristics of Salmon	玲玲,刘学升,王博,孙英伟	u Xuesheng,Wang Bo,Sun Y	中国卫生检验杂志	ese Journal of Health Inspe	28	5	534-536	2018	-
5804	海南省餐饮等食品微生物	nation status of catering an	柳贤勤,何剑,邢坤,冯礼明	nqin,He Jian,Xing Kun,Feng	实用预防医学	ractical preventive medicin	25	3	261-263,344	2018	sn.1006-3110.2018.03.002
5805	源性疾病哨点医院主动监测	results of foodborne disea	华,杜雪飞,江晓,郭宝福,金	Du Xuefei,Jiang Xiao,Guo B	公共卫生与预防医学	Health and Preventive Me	29	5	100-103	2018	sn.1006-2483.2018.05.027
5806	1-2017年食品中沙门菌污染	nual survey on Salmonella	赵忠俊,王晓威,梁永柱.	ngjun,Wang Xiaowei,Liang	中国城乡企业卫生	ygiene in urban and rural a	33	4	53-54	2018	5/j.1003-5052.2018.04.022
5807	电泳技术对肠炎沙门菌食物	idis food poisoning using p	劭劼,黄智瑜,王凤美,蔡洪	ie,Huang Zhiyu,Wang Feng	中国食品卫生杂志	hinese Food Hygiene Journ	30	5	543-546	2018	).13590/j.cjfh.2018.05.020

5808	糕引起集体食品安全事件	e food safety incidents cau	,肖新才,谢朝军,李泳光,	Xincai,Xie Chaojun,Li Yong	医学动物防制	medical animal control	34	6	604-605	2018	0.7629/yxdwfz201806027
5809	源沙门氏菌耐药性及相关	e and correlation of Salmon	萍,王艳,王超,巢国祥,张	Yan,Wang Chao,Chao Guo	食品安全质量检测学报	f Food Safety and Quality I	9	7	1513-1517	2018	sn.2095-0381.2018.07.008
5810	食物中毒的微生物检验结	bial test results for bacteri	陈静宜,廖飞.	Chen Jingyi,Liao Fei.	首都食品与医药	Capital Food and Medicine	25	6	34	2018	sn.1005-8257.2018.06.033
5811	南省地方食品微生物污染	f microbial contamination	李艳芬,炊慧霞,张濛,张秀	,Cui Huixia,Zhang Meng,Zh	中国卫生检验杂志	ese Journal of Health Inspe	28	3	343-344,349	2018	-
5812	源性伤寒暴发92例临床分	phoid outbreak92Case cl	;陈建新;张建春;翁锡定;X	xin;Zhang Jianchun;Wen	中外医学研究	se and foreign medical re	16	31	174-175	2018	3/j.cnki.cfmr.2018.31.083
5813	预防在伤寒暴发流行控制	rug prevention in epidem	林云翔;	Lin Yunxiang;	基层医学论坛	Primary Medicine Forum	22	22	3169-3170	2018	/j.1672-1721.2018.22.081
5814	品沙门氏菌生化型和血清	e analysis of Salmonella in	马丽萍,张素艳,蒋健.	Liping,Zhang Suyan,Jiang J	宁波农业科技	Agricultural Science and Te	-	4	8-9	2019	-
5815	沙门菌致小儿败血症的	ysis of a case of pediatric s	蔚,李萍,王恒辉,郭金磊,高	i Ping,Wang Henghui,Guo	疾病监测	disease surveillance	34	12	1122-1125	2019	sn.1003-9961.2019.12.019
5816	年甘南州市售食品致病菌	enic bacteria in commercia	王辉艳.	Wang Huiyan.	疾病预防控制通报	se Prevention and Control	34	6	50-53	2019	15/j.cnki.jbyfkztb.1906005
5817	肉中肯塔基沙门菌的流行	monella Kentucky in chick	,陈政权,张红霞,温俊平,	engyi,Zhang Hongxia,Wen	畜牧兽医学报	mal Husbandry and Veteri	50	12	2509-2517	2019	sn.0366-6964.2019.12.015
5818	肿瘤住院患者合并铜绿	atients with malignant tum	何宁.	He Ning.	中国当代医药	inese contemporary medic	26	36	185-188	2019	sn.1674-4721.2019.36.053
5819	沙门氏菌血清型、耐药	lence characteristics of foo	,段合波,吴清,郭婧,张英子	Hebo,Wu Qing,Guo Jing,Zh	武汉轻工大学学报	f Wuhan University of Light	38	6	1-7	2019	sn.2095-7386.2019.06.001
5820	感染性腹泻人群病原构	tance in other people with	燕,林俊香,蔡剑辉,何小	Yan,Lin Junxiang,Cai Jianhu	热带医学杂志	ournal of tropical medicine	19	12	1554-1556,1560	2019	sn.1672-3619.2019.12.024
5821	肠炎沙门氏菌引发的食物	poisoning caused by Salmo	赵堡宁,田疆.	Zhao Baoning,Tianjiang.	宁夏医科大学学报	nal of Ningxia Medical Univ	41	12	1294-1296	2019	sn1674-6309.2019.12.026
5822	针穿刺后脓液标本中细	pus samples from patients	群,张业,陈培佳,郭文伟,郑	ng Ye,Chen Peijia,Guo We	抗感染药学	Anti-infective Pharmacy	16	12	2103-2105	2019	sn.1672-7878.2019.12-021
5823	广东鸽源大肠杆菌、沙门	scherichia coli and Salmone	吕敏娜,孙铭飞,沈浩铎.	inna,Sun Mingfei,Shen Hao	畜牧兽医科技信息	Veterinary Science and Te	-	12	46-48	2019	N.1671-6027.2019.12.034
5824	寒沙门菌感染的临床特征	nd treatment of non-typho	晋华,胡鹏娟,赵青,原慧云,	,Hu Pengjuan,Zhao Qing,Yu	医药前沿	Medical Frontier	9	36	110-111	2019	-
5825	蟑螂侵害及病原携带状	festation and pathogen ca	,哈丽娜,邸师红,杨凤琴,	alina,Di Shihong,Yang Feng	中华卫生杀虫药械	ygienic Insecticide and Equ	25	6	569-572	2019	-
5826	要致病菌的多重定量PCR	chicken meat in Hebei Prov	子龙,宋阔阔,张欣,王瑾,	ng Kuokuo,Zhang Xin,Wan	中国兽医科学	Chinese Veterinary Science	49	12	1549-1554	2019	issn.1673-4696.2019.0197
5827	菌引起的食源性疾病暴发	foodborne disease outbrea	,徐俊,徐瑞雪,魏泽明,林	Xu Ruixue,Wei Zeming,Lin	首都公共卫生	capital public health	13	6	296-298	2019	-
5828	起的食源性疾病暴发事	foodborne disease outbre	明捷,翁曦君,王银会,巴	h Chiet,Weng Xijun,Wang	首都公共卫生	capital public health	13	6	299-301	2019	-
5829	地区犊牛腹泻病原流行	ion on calves’ diarrhea pat	孙力,覃杰.	Sun Li,Qin Jie.	龙江畜牧兽医（下半月）	dry and Veterinary Medicin	-	12	80-82	2019	nki.hljxmsy.2019.03.0025
5830	氏菌食源性疾病事件的实	is of a Salmonella Enteritid	海涛,吕秋艳,赵香菊,王晨	u Qiuyan,Zhao Xiangju,Wa	首都公共卫生	capital public health	13	6	301-303	2019	-
5831	区动物源沙门菌的检测与	of Salmonella of animal o	牟豪,余远迪,翟少钦.	u Hao,Yu Yuandi,Zhai Shao	养殖与饲料	Breeding and feed	-	12	69-72	2019	n.1671-427X.2019.12.029
5832	和水库水源水微生物污染	ator bacteria in source wa	萍,孙宗科,张伟,陈西平,李	un Zongke,Zhang Wei,Chen	环境与健康杂志	nal of Environment and He	36	11	1000-1002	2019	ki.1001-5914.2019.11.014
5833	生儿化脓性脑膜炎病原	bacteria in neonatal purul	王华伟.	Wang Huawei.	临床医药文献电子杂志	edical literature electronic	6	86	28,44	2019	-
5834	沙门菌肠炎66例临床特点	dren66Clinical characterist	洁雯,马丙南,林菁,黄丽	en,Ma Bingnan,Lin Jing,Hu	甘肃医药	Gansu Medicine	38	11	984-986	2019	-
5835	利丹沙门菌的鉴定及耐药	sistance analysis of Salmon	陈革,缪卫媛,高尚,杨跃飞	Miao Weiyuan,noble,Yang	中国兽医杂志	Chinese Veterinary Journal	55	10	91-92	2019	-
5836	口市食品安全风险微生物	food safety risk microbial	,孙晓岩,郭素英,安静,单	aoyan,Guo Suying,Quiet,Si	社区医学杂志	urnal of Community Medic	17	19	1181-1184	2019	90/j.cnki.JCM.2019.19.05
5837	南省微生物性食源性疾病	teristics of microbial foodb	张强,万青青,万蓉,闵向东,	,Wan Qingqing,Wanrong,M	职业与健康	Occupation and health	35	18	2493-2498	2019	-
5838	省余姚市非伤寒沙门菌监	a and analysis of drug resis	袁士杰,张建群,黄邵军.	ijie,Zhang Jianqun,Huang S	疾病监测	disease surveillance	34	8	725-730	2019	sn.1003-9961.2019.08.011
5839	型副伤寒疫情交叉暴发的	sis of cross-outbreaks of pa	,李红叶,王炳全,巩盼龙,	ye,Wang Bingquan,Gong P	疾病监测	disease surveillance	34	8	721-724	2019	sn.1003-9961.2019.08.010
5840	铁路冷藏盒饭微生物变化	l changes in railway refrige	柴洪艳,陈亮,付璇.	i Hongyan,Chen Liang,Fu X	铁路节能环保与安全卫生	ation, environmental prote	9	4	25-28,32	2019	.issn2095-1671.2019.0045
5841	年镇江市食源性致病菌监	results of foodborne path	,巢秀琴,韦镇平,曹文婷,	Xiuqin,Wei Zhenping,Cao	现代预防医学	modern preventive medicin	46	16	3020-3022,3062	2019	-
5842	区不同来源沙门菌分离株	of Salmonella isolates fro	霞,郭凯纯,许少洪,黄芳,	aichun,Xu Shaohong,Huan	中国食品卫生杂志	hinese Food Hygiene Journ	31	4	356-359	2019	.13590/j.cjfh.2019.04.011
5843	病性沙门菌分离鉴定与药	rug susceptibility test of pa	王恒月.	Wang Hengyue.	中国兽医杂志	Chinese Veterinary Journal	55	7	64-65	2019	-
5844	病原菌检验结果90例临	pathogen test results90Ca	王佳.	Wang Jia.	健康大视野	Healthy vision	-	13	225227	2019	sn.1005-0019.2019.13.378

5845	沙门氏菌食物中毒的病原	analysis of a case of Salmon	超,葛藤,杜雪飞,王燕,丁	udzu,Du Xuefei,Wang Yan,	现代预防医学	modern preventive medicin	46	9	1711-1714	2019	-
5846	患儿继发抗生素相关性	ic-associated diarrhea in ch	婧,俞栋,尹惠梅,杨琳,葛	u Dong,Yin Huimei,Yang Li	中华医院感染学杂志	ournal of Hospital Infectiou	29	8	1214-1217	2019	.11816/cn.ni.2019-180762
5847	区熟肉制品中食源性致病	enic bacteria in cooked mea	叶石兴,徐伟兵,游文彪.	nixing,Xu Weibing,You Wen	食品安全导刊	food safety guide	-	12	82-83	2019	043/j.cnki.cfs.2019.12.062
5848	年广东省食源性疾病监测	orne disease surveillance re	李雪琪,梁骏华,刘志婷,邓小	iang Junhua,Liu Zhiting,Der	华南预防医学	uth China Preventive Medic	45	2	152-155	2019	.13217/j.scjpm.2019.0152
5849	膜中沙门氏菌实时荧光PC	n food plastic packaging film	王莹.	Wang Ying.	农村科学实验	Rural Science Experiment	-	27	83-84	2019	-
5850	割鸡肉中沙门菌污染状况	g characteristics of Salmon	勤贵,刘晨星,杨洋,曹晨阳	Liu Chenxing,Yang Yang,Ca	中国食品卫生杂志	hinese Food Hygiene Journ	31	5	423-428	2019	.13590/j.cjfh.2019.05.005
5851	性腹泻病原微生物检验的	enic microorganisms in acu	唐骐.	Tang Qi.	医学信息文摘（连续型电	information abstract (cont	19	74	195-196	2019	ki.1671-3141.2019.74.132
5852	食品中主要致病菌的快速	jor pathogenic bacteria in	华,宋夏,徐碧瑶,罗宝章,刘	g Xia,Xu Biyao,Luo Baozhar	现代预防医学	modern preventive medicin	46	10	1757-1760	2019	-
5853	疾病事件的病原学及分子	y and molecular biology of	沈静,张静,王姗,金虹飞.	hang Jing,Wang Shan,Jin H	医药前沿	Medical Frontier	9	34	242-243	2019	-
5854	大陆食源性疾病暴发事件	data on foodborne disease	森,陈江,白光大,徐粒子,王	ang,Bai Guangda,Xu particl	中国食品卫生杂志	hinese Food Hygiene Journ	31	1	64-70	2019	.13590/j.cjfh.2019.01.014
5855	贵州省食源性疾病病例监	ce results of foodborne dis	王娅芳,周亚娟,朱姝,杨蕙	fang,Zhou Yajuan,Zhu Shu,	现代预防医学	modern preventive medicin	46	4	723-727	2019	-
5856	6-2017年4类食品食源性	Contamination status of foo	曹舜珊,陈夏威,何彬洪.	unshan,Chen Xiawei,He Bi	海峡预防医学杂志	s Journal of Preventive Me	25	2	78-79	2019	-
5857	贮藏、销售环节中沙门	in the production, storage	郭耀东,张英华,韩笑.	aodong,Zhang Yinghua,Har	湖北农业科学	Hubei Agricultural Sciences	58	16	115-117	2019	sn0439-8114.2019.16.026
5858	江市人民医院沙门菌监测	its etiological characteristic	玲,陈志晓,林飞燕,关志	en Zhixiao,Lin Feiyan,Kwar	海南医学	Hainan Medicine	30	13	1697-1700	2019	sn.1003-6350.2019.13.017
5859	感染性腹泻沙门菌监测结	urveillance results in outpa	熊玉华,丁恒.	Xiong Yuhua,Ding Heng.	保健文汇	Health Wenhui	-	4	168-169	2019	sn.1671-5217.2019.04.087
5860	市鸡沙门氏菌耐药质粒	sistant plasmids of Salmon	杰,王燕,马宏财,冯静,元	g Yan,Ma Hongcai,Feng Jing	现代畜牧兽医	mal husbandry and veterina	-	2	5-8	2019	-
5861	年上海市松江区食源性疾	ase surveillance results in S	邱香,盛峰松,孙中兴.	ng,Sheng Fengsong,Sun Zho	职业与健康	Occupation and health	35	13	1774-1777	2019	-
5862	市腹泻症候群病例监测	alysis of diarrhea syndrome	马菊红,曾同霞,杨军鹏.	hong,Zeng Tongxia,Yang Ju	疾病预防控制通报	se Prevention and Control	34	4	36-38,68	2019	15/j.cnki.jbyfkztb.1902019
5863	年湖州市食源性疾病监测	analysis of foodborne disea	伟,徐德顺,吴晓芳,陈莉	Xu Deshun,Wu Xiaofang,Ch	中国卫生检验杂志	ese Journal of Health Inspe	29	13	1613-1615,1618	2019	-
5864	场沙门氏菌的分离鉴定及	ance analysis of Salmonella	华,林雪玲,赖宝色,林秋敏,	Lin Xueling,Lai Baose,Lin Qi	畜禽业	vestock and poultry indust	30	9	3-5	2019	ki.1008-0414.2019.09.002
5865	宁堡沙门菌致儿童血流感	causes bloodstream infect	兰,刘辉,陈丽萍,陈媛,钟	i,Chen Liping,Chen Yuan,Z	中国抗生素杂志	hinese Journal of Antibiotic	44	2	270-272	2019	sn.1001-8689.2019.02.022
5866	杆菌及沙门氏杆菌的分离	is of Escherichia coli and Sa	陈培培,齐心,张灿.	Peipei,Work together,Zhan	黑龙江畜牧兽医（下半月）	dry and Veterinary Medicin	-	11	76-79,180	2019	cnki.hljxmsy.2019.01.0292
5867	市感染性腹泻病毒病原学	pidemic characteristics of i	何楚婕,胡贵方.	He Chujie,Hu Guifang.	热带医学杂志	journal of tropical medicine	19	2	240-243	2019	sn.1672-3619.2019.02.029
5868	州省食源性沙门氏菌血清	drug resistance of foodbo	,张豫,蔡娜,安丽娜,向婧	,Zhang Yu,Cai Na,Alina,Xia	中华微生物学和免疫学杂	rnal of Microbiology and I	39	10	737-742	2019	sn.0254-5101.2019.10.003
5869	沙门菌引起的食源性疾病	foodborne illness caused	哲,王永全,徐俊,苗芳,李明	Yongquan,Xu Jun,Miao Fa	中国卫生检验杂志	ese Journal of Health Inspe	29	24	2969-2970,2974	2019	-
5870	猪源沙门菌分离鉴定及耐	ance detection of Salmonel	,张婷,严昕,韩笑,张萍,晏	ing,Yan Xin,Han Xiao,Zhan	金陵科技学院学报	ling Institute of Science an	35	3	89-92	2019	ki.32-1722/n.2019.03.019
5871	食及其制品致食源性疾病	orne disease cases caused	贵,张黎黎,王娅芳,朱姝,	ang Lili,Wang Yafang,Zhu S	医学信息	medical information	32	21	121-124	2019	sn.1006-1959.2019.21.039
5872	患者细菌病原学检测及	and serotype analysis in pa	,陈娟仙,韩安棣,陈维增,	uanxian,Han Andi,Chen W	中国卫生检验杂志	ese Journal of Health Inspe	29	9	1056-1059	2019	-
5873	区感染性腹泻标本常见病	bacteria in infectious diarr	维钧,舒高林,彭华,李东迅	jun,Shu Gaolin,Peng Hua,L	实用预防医学	ractical preventive medicin	26	10	1274-1277	2019	sn.1006-3110.2019.10.034
5874	微生物检验质量控制探讨	n food microbiological insp	颜淑芳.	Yan Shufang.	食品界	food industry	-	8	73-75	2019	n.2095-638X.2019.08.048
5875	厂花生酱和芝麻酱加工过	uring peanut butter and se	晓蓉,王慧雯,魏茂琪,龚	ng,Wang Huiwen,Wei Mao	食品安全质量检测学报	f Food Safety and Quality I	10	22	7610-7615	2019	-
5876	沈阳市食源性疾病主动监	rveillance results of foodb	慧中,刘博,刘丰瑜,刘雪梅	zhong,Liu Bo,Liu Fengyu,Li	实用预防医学	ractical preventive medicin	26	9	1040-1042	2019	sn.1006-3110.2019.09.005
5877	苏省无锡市感染性腹泻病	sults of infectious diarrhea	霞,沙丹,冯微宏,齐倩倩,	atan,Feng Weihong,Qi Qian	疾病监测	disease surveillance	34	9	795-799	2019	sn.1003-9961.2019.09.006
5878	门菌的分离鉴定与PFGE	of Salmonella from waterf	爱贞,梁景涛,麦晓周,李荣	iang Jingtao,Mai Xiaozhou,	中国兽医学报	al of Chinese Veterinary Me	39	10	1994-2000	2019	nki.1005-4545.2019.10.18
5879	边县鸡病主要流行情况调	idemic situation of chicken	丽芬,董平,王宇慧,武艳,	Dong Ping,Wang Yuhui,Wu	今日畜牧兽医	ivestock Veterinarian Toda	35	1	8-9	2019	sn.1673-4092.2019.01.007
5880	昌平区感染性腹泻监测结	ance results of infectious di	李东迅,彭华,王维钧,赵菊	n,Peng Hua,Wang Weijun,Z	中国校医	Chinese school doctor	33	12	905-909	2019	-
5881	氏菌的分离鉴定及药物敏	n and drug sensitivity analy	宁,杨勇,王改丽,刘泽余,	,Yang Yong,Wang Gaili,Liu	中国畜牧兽医	mal husbandry and veterina	46	2	635-641	2019	ki.1671-7236.2019.02.036

5882	南省肉类制品致病菌污	hogenic bacteria contami	,杨菁,汤晓召,邹颜秋硕,	ng,Tang Xiaozhao,Zou Yand	食品安全质量检测学报	f Food Safety and Quality I	10	22	7596-7600	2019	-
5883	瘤的腔内治疗-单中心8年	rtic aneurysm-single cente	岩,朱杰昌,戴向晨,罗宇	u Jiechang,Dai Xiangchen,	中华普通外科杂志	ese Journal of General Sur	34	5	402-405	2019	n.1007-631X.2019.05.007
5884	菌引起的食源性疾病爆发	a foodborne illness outbrea	倪婕,刘峰,杨华.	Ni Jie,Liu Feng,Yang Hua.	西医结合心血管病电子杂	Diseases Integrated Traditi	7	28	94103	2019	-
5885	年烟台市食源性疾病主动	lts of active surveillance o	,孙月琳,王朝霞,宫春波,	in,Wang Chaoxia,Gong Chu	中国卫生检验杂志	ese Journal of Health Inspe	29	6	747-750	2019	-
5886	禽动物源性沙门菌血清型	ing from livestock and pou	段瑶,李杰,阚飙,闫梅英.	Yao,Li Jie,Kan Biao,Yan Me	疾病监测	disease surveillance	34	4	295-302	2019	sn.1003-9961.2019.04.005
5887	水源性沙门菌血清型与	ug resistance of human- an	建群,黄邵军,苗超,袁士	n,Huang Shaojun,Miao Cha	中国卫生检验杂志	ese Journal of Health Inspe	29	14	1694-1696	2019	-
5888	6—2017年食源性疾病监	alysis of annual foodborne	宇,叶倩,郑卢萍,郑伟,杨	n,Zheng Luping,Zheng We	中国乡村医药	Chinese rural medicine	26	5	53-54	2019	sn.1006-5180.2019.05.034
5889	体检沙门氏菌检测结果分	preventive management	黄羡华.	Huang Xianhua.	临床检验杂志（电子版）	f Clinical Testing (electroni	8	1	82-83	2019	-
5890	性腹泻病原微生物检验	genic microorganisms in	吴淑华,苗锐.	Wu Shuhua,Miao Rui.	中国社区医师	hinese community physicia	35	4	149-150	2019	sn.1007-614x.2019.04.098
5891	检验在季节性腹泻诊治	ination in the diagnosis an	胡灵莉.	Hu Lingli.	健康前沿	health frontier	28	1	282	2019	sn.9128-6509.2019.01.258
5892	市食源性疾病患者分离沙	monella isolates from patie	岳峰,方正,孙永,崔志刚.	ng,Founder,Sun Yong,Cui Z	疾病监测	disease surveillance	34	4	312-315	2019	sn.1003-9961.2019.04.008
5893	儿童粪便分离沙门菌的流	nce of Salmonella isolates	黄盈敏.	Huang Yingmin.	实用临床医学	practical clinical medicine	20	6	94-96	2019	64/j.cnki.lcsy.2019.06.035
5894	区出口水产品微生物检	sis of Exported Aquatic Pro	,林嘉欣,刘莹,孙雪莲,孙	axin,Liu Ying,Sun Xuelian,Su	农产品加工（上半月）	oducts processing (first hal	-	6	56-58	2019	671-9646(X).2019.06.016
5895	肠杆菌和沙门氏菌分离	alysis of Escherichia coli ar	骆海朋,任秀,陈怡文,余文	ipeng,Ren Xiu,Chen Yiwen,	食品安全质量检测学报	f Food Safety and Quality I	10	1	48-53	2019	sn.2095-0381.2019.01.008
5896	～2017年食品安全风险监	nalysis of annual food safe	宇鸿,金春秋,陈飞荣,王益	,Jin Chunqiu,Chen Feirong,	安徽预防医学杂志	i Journal of Preventive Me	25	2	105-107,117	2019	-
5897	产超广谱β-内酰胺酶沙门	-broad spectrumβ-Analysis	陈素菜,沈丽珍.	Chen Sucai,Shen Lizhen.	疾病监测	disease surveillance	34	1	62-65	2019	sn.1003-9961.2019.01.015
5898	肠炎沙门氏菌食物中毒	monella Enteritidis food po	礼钦,张建新,张丽芬,张	n,Zhang Jianxin,Zhang Lifer	食品安全质量检测学报	f Food Safety and Quality I	10	5	1394-1400	2019	sn.2095-0381.2019.05.052
5899	食品的污染及其导致的	tion of food and its resulti	刘祎婷.	Liu Yiting.	中国保健营养	Chinese health nutrition	29	22	329	2019	-
5900	区健康牛沙门氏菌携带情	tance in healthy cattle in ty	海燕,孙栋良,陈德美,武	yan,Sun Dongliang,Chen D	解放军预防医学杂志	ive Medicine of the People	37	7	61-62,65	2019	-
5901	源沙门氏菌分离鉴定、血	resistance research of Salm	贇,张彦斌,卜红宇,岳佳,崔	g Yanbin,Bu Hongyu,Yue Jia	食品安全质量检测学报	f Food Safety and Quality I	10	6	1705-1709	2019	sn.2095-0381.2019.06.050
5902	7年湖州市食源性疾病流	ution characteristics of foo	付云,袁瑞,宋臻鹏.	Yun,Yuan Rui,Song Zhenpe	中国卫生检验杂志	ese Journal of Health Inspe	29	20	2537-2540	2019	-
5903	市清城区一起食源性疾	utbreak in Qingcheng Distr	王美欢,凌水权,赵泽辉.	eihuan,Lingshui Quan,Zha	中国热带医学	Chinese tropical medicine	19	11	1063-1067	2019	cnki.46-1064/r.2019.11.13
5904	珠海市10岁及以下儿童沙	Salmonella infection in ch	张亮,邱炜,张秋平.	g Liang,Qiu Wei,Zhang Qiu	公共卫生与预防医学	Health and Preventive Me	30	5	99-102	2019	sn.1006-2483.2019.05.025
5905	起肠炎沙门氏菌食物中	t of Salmonella Enteritidis	田渝,黄治兰,袁玲燕.	Yu,Huang Zhilan,Yuan Ling	现代医药卫生	modern medicine and healt	35	14	2254-2256	2019	sn.1009-5519.2019.14.064
5906	检验在腹泻患儿中的临	tion effect of microbial tes	任强.	Ren Qiang.	健康必读	Health must read	-	23	16	2019	-
5907	性疾病主动监测特定病原	ce characteristics of active	红,张琰,章丹阳,杨元斌,叶	Yan,Zhang Danyang,Yang	中国卫生检验杂志	ese Journal of Health Inspe	29	24	3049-3052,3055	2019	-
5908	儿童医院门诊感染性腹泻	ea in the outpatient clinic	,除文健,黄辉,邓莉,钟雪	enjian,Huang Hui,Deng Li,	中华检验医学杂志	e Journal of Laboratory Me	42	5	359-364	2019	sn.1009-9158.2019.05.008
5909	沙门氏杆菌的分离鉴定及	raesuis from pork in slaug	,邱燕,张升波,徐丽,李昌	Zhang Shengbo,Xu Li,Li Ch	龙江畜牧兽医（下半月）	dry and Veterinary Medici	-	3	73-77,171	2019	cnki.hljxmsy.2018.05.0336
5910	部分地区食源性沙门氏菌	f foodborne Salmonella in	王德宇,周凤岩.	Wang Deyu,Zhou Fengyan.	食品安全质量检测学报	f Food Safety and Quality I	10	4	973-976	2019	sn.2095-0381.2019.04.026
5911	感染性腹泻的病原体特点	ens and epidemic characte	,张晓蕾,李金平,朱东,张	iaolei,Li Jinping,Zhu Dong,	中华医院感染学杂志	ournal of Hospital Infectio	29	11	1732-1736	2019	.11816/cn.ni.2019-181467
5912	伤寒暴发疫情的耐药分析	d molecular typing of a fo	华,姚建香,刘晓骏,钱程,颜	anxiang,Liu Xiaojun,Qian C	医学动物防制	medical animal control	35	11	1033-1036	2019	0.7629/yxdwfz201911004
5913	便病原菌检验结果的临床	test results of pathogenic	纵瑞艳.	Zong Ruiyan.	东方药膳	Oriental medicinal food	-	15	132-133	2019	-
5914	添加剂中微生物污染情况	f microbial contamination	鹏,李金磊,杨希祥,方忠意	Jinlei,Yang Xixiang,Fang Zh	饲料工业	Feed industry	40	18	20-24	2019	3302/j.cnki.fi.2019.18.005
5915	市龙岗区细菌性食源性疾	logy of bacterial foodborn	丽红,吴洁,陈日炳,钟文	Wu Jie,Chen Ribing,Zhong	热带医学杂志	ournal of tropical medicine	19	3	373-375	2019	sn.1672-3619.2019.03.028
5916	沙门氏菌食物中毒事件	almonella food poisoning	,古丽斯,章志斌,陈远峰,	hang Zhibin,Chen Yuanfen	实用预防医学	ractical preventive medici	26	8	947-950	2019	sn.1006-3110.2019.08.014
5917	腹泻病原菌的分离鉴定及	ibility test of pathogenic b	港,侯俊丽,陈志军,程淑	Hou Junli,Chen Zhijun,Che	畜牧兽医科学（电子版）	y and Veterinary Science (E	-	1	11-12	2019	sn.2096-3637.2019.01.004
5918	携带沙门氏菌血清型分	ug resistance of Salmonell	,贾森泉,周永明,李学红,	ngs,Zhou Yongming,Li Xue	食品安全质量检测学报	f Food Safety and Quality I	10	22	7570-7575	2019	-

5919	菌血流感染临床特点、病	eristics and prognosis of AI	,石亚玲,蔡卫平,唐小平,	ling,Cai Weiping,Tang Xiao	实用医学杂志	ournal of Practical Medicin	35	14	2234-2237	2019	sn.1006-5725.2019.14.009
5920	地区鸭源沙门菌流行情况	nce of Salmonella originati	杏芬,肖英平,戴贤君,夏	Xiao Yingping,Dai Xianjun,	中国家禽	Chinese poultry	-	24	74-79	2019	sn.1004-6364.2019.24.018
5921	市售食品食源性致病菌监	rne pathogenic bacteria in	张妍.	Zhang Yan.	医学信息文摘（连续型电	information abstract (cont	19	86	181-182	2019	ki.1671-3141.2019.86.103
5922	区2015-2017年沙门菌相关	015-2017Analysis of Salmo	鲁瑶,林晓晖,黄少隆,喻聪	n Xiaohui,Huang Shaolong,	热带医学杂志	ournal of tropical medicine	19	9	1184-1186,1190	2019	sn.1672-3619.2019.09.031
5923	流感染者病原菌分布及临	ution and clinical characteri	曦悦,石明巧,李冬冬,肖玉	hi Mingqiao,Li Dongdong,	中国艾滋病性病	AIDS and STDs in China	25	2	187-188	2019	419/j.cnki.aids.2019.02.21
5924	生物检测及血清IFN-γIL-6	of infants and young childr	潘淑,张婧,马瑜珊.	n Shu,Zhang Jing,Ma Yusha	河北医学	Hebei Medicine	25	3	671-675	2019	sn.1006-6233.2019.03.037
5925	圳市食源性沙门菌血清分	and drug resistance of foo	,夏俊杰,梁焯南,贺连华,	jie,Liang Zhuonan,He Lianh	实用预防医学	ractical preventive medicin	26	4	495-496	2019	sn.1006-3110.2019.04.032
5926	鸡源沙门氏菌的分离鉴	e analysis of Salmonella or	夏宇飞,周汝顺,黎满香.	ufei,Zhou Rushun,Li Manxi	湖南农业科学	Hunan Agricultural Science	-	7	4-8	2019	cnki.hnnykx.2019.007.002
5927	清学检验在沙门菌临床检	biochemical reactions and	杨娅,杨正禄.	Yang Ya,Yang Zhenglu.	中国保健营养	Chinese health nutrition	29	2	103	2019	sn.1004-7484.2019.02.124
5928	沙门氏菌食物中毒流行病	stigation of a case of salmo	韩京娣,张丹梅.	Han Jingdi,Zhang Danmei.	养生保健指南	Health and Wellness Guide	-	27	369	2019	-
5929	寒沙门氏菌引起食物中毒	e of food poisoning caused	于美英,祝永英,冯海燕.	eiying,Zhu Yongying,Feng H	中国农村卫生	rural health in china	11	10	42-43	2019	n.1674-361X.2019.10.046
5930	学校甲型副伤寒暴发疫情	outbreak of paratyphoid fe	顾敏华,姚建香,李骏.	Minhua,Yao Jianxiang,Li Ju	中国学校卫生	Chinese school health	40	4	616-618	2019	ki.1000-9817.2019.04.039
5931	地区市售调味品质量调查	ality of commercially availa	陈艳,颜一雪,雷圆圆.	n Yan,Yan Yixue,Lei Yuanyu	食品与发酵科技	and Fermentation Techno	55	5	112-116,120	2019	n.1674-506X.2019.05-022
5932	州市沙门菌污染及其感染	tamination and infection d	何伟佳,赵瑾,李晨,董庆利	eijia,Zhao Jin,Li Chen,Dong	中华疾病控制杂志	hese Journal of Disease Co	23	7	877-880	2019	/j.cnki.zhjbkz.2019.07.026
5933	昆明市医疗机构污水消毒	wage disinfection treatmen	周卫民,任艳,韩瑞萍.	Weimin,Ren Yan,Han Rui	中国消毒学杂志	hinese Journal of Disinfecti	36	11	846-847,851	2019	sn.1001-7658.2019.11.014
5934	料中沙门菌快速检测方法	etection methods of Salmo	忠意,李金磊,董鹏,狄元冉	ngyi,Li Jinlei,Dong Peng,Di Y	上海畜牧兽医通讯	al Husbandry and Veterina	-	6	10-13	2019	-
5935	2016年沙门氏菌的PFGE分	16SalmonellaPFGEMolecu	春红,吴小凤,郑迎翔,郑友	Wu Xiaofeng,Zheng Yingxia	医学动物防制	medical animal control	35	6	540-543	2019	0.7629/yxdwfz201906008
5936	株洲市其他感染性腹泻病	nce results of other infecti	志,何礼,胡世雄,高立冬,李	He Li,Hu Shixiong,Gao Lidon	疾病监测	disease surveillance	34	9	788-794	2019	sn.1003-9961.2019.09.005
5937	门菌血流感染伴疑似导管	Salmonella typhi bloodstre	,石卫峰,刘皋林,范国荣,	eifeng,Liu Gaolin,Fan Guor	中国医药导报	China Medical Herald	16	2	131-134	2019	-
5938	童专科医院儿童细菌性腹	ogenic bacteria in children	,许红梅,张明强,熊菀,赵	ei,Zhang Mingqiang,Xiong	中国循证儿科杂志	ournal of Evidence-Based	14	3	176-180	2019	sn.1673-5501.2019.03.003
5939	市食源性疾病流行病学特	characteristics of foodbor	翟光富,冯亚琴,赵存喜.	Guangfu,Feng Yaqin,Zhao	安徽医学	Anhui Medicine	40	10	1176-1180	2019	sn.1000-0399.2019.10.029
5940	细菌性食物中毒发生状况	erial food poisoning in a ce	万磊.	Wan Lei.	河南预防医学杂志	Journal of Preventive Me	30	5	380-382,419	2019	-
5941	子水平的变化、病原学特	logical characteristics and	殷跃文.	Yin Yuewen.	中国病原生物学杂志	ese Journal of Pathogen Bi	14	6	716-720	2019	10.13350/j.cjpb.190621
5942	壮族自治区市售食品中沙	ata in commercially availab	雪婷,蒋玉艳,谭冬梅,吕素	g,Jiang Yuyan,Tan Dongme	中国食品卫生杂志	hinese Food Hygiene Journ	31	5	449-455	2019	.13590/j.cjfh.2019.05.010
5943	市学校饮用水微生物污染	obial contamination of drin	李腊梅,吴攀,杨芸,陈天林	ei,Wu Pan,Yang Yun,Chen T	现代预防医学	modern preventive medicin	46	12	2265-2268	2019	-
5944	物中毒的病原菌调查与预	analysis of preventive coun	金艳珍.	Jin Yanzhen.	首都食品与医药	Capital Food and Medicine	26	4	71	2019	sn.1005-8257.2019.04.065
5945	鲁木齐市鼠伤寒沙门菌耐	d molecular typing of Salm	蕾,牟文婷,姜亦华,王超,	u Wenting,Jiang Yihua,Wa	世界最新医学信息文摘	he world's latest medical in	-	78	267-268	2019	ki.1671-3141.2019.78.174
5946	门菌暴发疫情的病原学检	eability analysis of an outb	,管英,姚楠,林杨,陈果,周	g,Yao Nan,Lin Yang,Chen G	中国卫生检验杂志	ese Journal of Health Inspe	29	5	617-620	2019	-
5947	市生活用纸中细菌的耐药	nce of bacteria in househol	清,王航,牛慧君,华团坡,刘	ang Hang,Niu Huijun,huatu	河南医学研究	Henan Medical Research	28	7	1190-1193	2019	n.1004-437X.2019.07.012
5948	染性腹泻病原微生物及耐	nd drug resistance of infect	于淼.	Yu Miao.	饮食保健	Diet and health care	6	20	17-18	2019	-
5949	天津市急性腹泻患者病原	d epidemic characteristics	高璐,李佳萌.	Gao Lu,Li Jiameng.	中华传染病杂志	se Journal of Infectious Dis	37	4	209-213	2019	sn.1000-6680.2019.04.004
5950	门菌诱导自噬的分子机制	mechanism of autophagy	庄晶,刘鹏刚,王晶香,孙敬	Liu Penggang,Wang Jingxia	中国家禽	Chinese poultry	-	18	44-52	2019	sn.1004-6364.2019.18.009
5951	市伤寒沙门菌耐药监测及分	d molecular typing analysi	香,张宏宾,高海英,周品众	ng Hongbin,Gao Haiying,Zh	中华医院感染学杂志	ournal of Hospital Infectiou	29	23	3530-3534	2019	.11816/cn.ni.2019-186720
5952	泻儿童非伤寒沙门菌感染	atus and drug resistance in	俊英,商丽红,邓林,谢晓	ying,Shang Lihong,Deng Lin	国际检验医学杂志	onal journal of laboratory	40	11	1325-1329	2019	sn.1673-4130.2019.11.011
5953	腹泻患者沙门菌分布特征	g resistance of Salmonella	伊怀文,张恒.	Yi Huaiwen,Zhang Heng.	中国国境卫生检疫杂志	ntier Health and Quarantin	42	2	137-138,141	2019	/j.1004-9770.2019.02.017
5954	河源市腹泻病例沙门菌感	nella Infection in Diarrhea	燕红,黄丰光,高淑萍,陈俏	g Fengguang,Gao Shuping,	华南预防医学	uth China Preventive Medi	45	1	45-47,51	2019	.13217/j.scjpm.2019.0045
5955	年潍坊市食源性致病菌监	g results of foodborne pat	徐斌,张康,刘德辉,赵忠俊	ang Kang,Liu Dehui,Zhao Z	社区医学杂志	urnal of Community Medic	17	12	687-689	2019	90/j.cnki.JCM.2019.12.01

5956	儿童伤寒和副伤寒沙门氏菌	a typhoid and paratyphoid	俊英,谢晓丽,熊励晶,商丽	ng,Xie Xiaoli,Xiong Lijing,Sh	四川医学	Sichuan Medicine	40	9	881-885	2019	sn1004-0501-2019.09.003
5957	氏菌和葡萄球菌耐药性及	stance genes of Salmonella	亚军,高超,吾买尔江·牙合	/ajun,superb,Wumaierjiang	林科技大学学报（自然科	vest A&F University (Natura	47	3	14-20	2019	j.cnki.jnwafu.2019.03.003
5958	仔猪细菌性腹泻病原的	bacterial diarrhea pathoge	李虹瑾,李国江,沙万里,尹	ngjin,Li Guojiang,Shawanli,	黑龙江畜牧兽医（下半月）	dry and Veterinary Medicin	-	3	88-89,94,172	2019	nki.hljxmsy.2018.04.0071
5959	离出沙门氏菌的脉冲场凝	lated from beef cattle dur	敏,毛衍伟,梁荣蓉,韩广星,	o Yanwei,Liang Rongrong,	食品科学	food science	40	13	171-176	2019	1002-6630-20180709-129
5960	门氏杆菌耐药表型与耐药	enotype and drug resistan	天泽,李明玥,刘通,张洁,	nze,Li Mingyue,Liu Tong,Z	黑龙江畜牧兽医（上半月）	ndry and Veterinary Medic	-	11	93-97,179	2019	nki.hljxmsy.2018.12.0412
5961	住院儿童腹泻患者沙门菌	infections in hospitalized c	李享宸,陈佳雪.	Li Xiangchen,Chen Jiaxue.	医药前沿	Medical Frontier	9	12	245-246	2019	-
5962	年郑州市食源性致病菌监	ng results of foodborne pat	松强,李倩,孙丽梅,牛卫东	ang,Li Qian,Sun Limei,Niu W	实用预防医学	ractical preventive medicin	26	12	1450-1453	2019	sn.1006-3110.2019.12.011
5963	肉销售点五种常见致病菌	on pathogenic bacteria in	,苏月,王春梅,阿里木江·	u Yue,Wang Chunmei,alim	新疆畜牧业	Xinjiang animal husbandry	34	6	22-25	2019	5/j.cnki.xjxmy.2019.6.005
5964	性腹泻的病原微生物检验	athogenic microorganisms	治芩,吴相川,马松涛,陈兴	u Xiangchuan,Ma Songtao,	饮食保健	Diet and health care	6	31	2	2019	-
5965	滋病合并沙门氏菌败血症	s of AIDS complicated with	玖香,石亚玲,陈星,陈万山	g,Shi Yaling,Chen Xing,Chen	热带医学杂志	journal of tropical medicine	19	11	1371-1374,1420	2019	sn.1672-3619.2019.11.012
5966	理州市售食品中细菌性污	of bacterial contamination	红菊,胡汝源,张昆仑,吴艳	,Hu Ruyuan,Zhang Kunlun,	食品安全质量检测学报	f Food Safety and Quality I	10	22	7762-7767	2019	-
5967	地区儿童感染性腹泻病原	illance results of infectious	阮洋,莫丽亚,邹爱军.	Ruan Yang,Moria,Zou Aijun	实用预防医学	ractical preventive medicin	26	11	1372-1374	2019	sn.1006-3110.2019.11.025
5968	病原菌检验的结果和临床	significance of pathogenic	古丽·吾甫尔,艾尼完尔·	uli·Wufu'er,Aini Wan'er·M	健康之友	health friend	-	15	25-26	2019	-
5969	河州食品风险监测中熟肉	at products monitoring in f	李青,吴春梅,周涛,谈星延	Chunmei,Zhou Tao,Talk abo	家庭医药	home medicine	-	8	368	2019	-
5970	从业人员食源性致病菌带	ong food processing emplo	,吴玲玲,李艳芬,炊慧霞,	Wu Lingling,Li Yanfen,Cui H	中国卫生产业	China health industry	16	11	155-157,160	2019	ki.1672-5654.2019.11.155
5971	沙门菌感染性肠炎患儿临	cteristics of children with n	覃四妹,宋晓玲.	Tan Simei,Song Xiaoling.	当代医药论丛	ontemporary Medicine Seri	17	12	101-102	2019	-
5972	菌引起的1起食源性疾病	nteritidis1Analysis of food	李红霞.	Li Hongxia.	现代医药卫生	modern medicine and healt	35	17	2759-2760	2019	sn.1009-5519.2019.17.062
5973	物危险性评价对食源性	assessment in food safety	申文佳,杨珊一.	Shen Wenjia,Yang Shanyi.	首都食品与医药	Capital Food and Medicine	26	16	191-192	2019	sn.1005-8257.2019.16.165
5974	猪场4种细菌的流行状况及	y on the prevalence and ty	蕾,张倩,杨俊,彭子欣,李凤	Qian,Yang Jun,Peng Zixin,	陕西农业科学	haanxi Agricultural Science	65	7	63-69	2019	sn.0488-5368.2019.07.020
5975	餐饮业过桥米线微生物污	ion status of cross-bridge r	梅琼,聂玉敏,段云权,丁启	,Nie Yumin,Duan Yunquan,	食品安全质量检测学报	f Food Safety and Quality I	10	12	4009-4012	2019	sn.2095-0381.2019.12.063
5976	市外卖店食品致病菌监	ts of food pathogenic bact	鹏伟,赵俊君,袁志敏,朱文	,Zhao Junjun,Yuan Zhimin,	实用预防医学	ractical preventive medicin	26	7	860-862	2019	sn.1006-3110.2019.07.024
5977	引起沙门菌食物中毒事件	f salmonella food poisonin	吕鸿鑫,温豪,何林,王金明	xin,Wen Hao,He Lin,Wang	热带医学杂志	journal of tropical medicine	19	4	509-511	2019	sn.1672-3619.2019.04.029
5978	17年肠炎沙门菌多重耐药	ultidrug resistance and mol	毕磊,黄峥,林征,张岭.	uang Zheng,Lin Zheng,Zha	上海预防医学	hanghai Preventive Medici	31	10	808-812	2019	28/j.cnki.sjpm.2019.19075
5979	区不同来源肠炎沙门菌耐	of Salmonella Enteritidis fr	,郭勇峰,崔京辉,李达,徐	Yongfeng,Cui Jinghui,Li Da	职业与健康	Occupation and health	35	16	2184-2186,2190	2019	-
5980	市滨海新区某地区食源性	orne diseases in a certain a	刘敏,杨宇鹏.	Liu Min,Yang Yupeng.	职业与健康	Occupation and health	35	12	1638-1640	2019	-
5981	脓性胰腺脓肿一例报道并	purulent pancreatic absce	孟雪,郑吉敏,王玉珍.	Xue,Zheng Jimin,Wang Yu	中国全科医学	Chinese general medicine	22	33	4150-4153	2019	sn.1007-9572.2019.00.210
5982	中致病性病原微生物检测	sis of pathogenic microorga	杨全中,张煜,谢永生.	uanzhong,Zhang Yu,Xie Yon	中国兽医杂志	Chinese Veterinary Journal	55	4	99-102	2019	-
5983	自治区食品中沙门菌血清	ution and drug resistance i	利平,乌兰,张冰冰,乌伊	iping,Ulan,Zhang Bingbing,	中国卫生检验杂志	ese Journal of Health Inspe	29	23	2847-2850	2019	-
5984	肠杆菌、沙门氏杆菌耐药	e of chicken-derived Esche	陈春林,冯刚,秦杨,付利芝	unlin,Feng Gang,Qin Yang,	黑龙江畜牧兽医（下半月）	dry and Veterinary Medicin	-	6	85-88	2019	nki.hljxmsy.2018.07.0325
5985	年绵阳市食源性疾病事件	ults of foodborne disease	刘颜,何玲玲,罗赟,吴晓红	,He Lingling,Luo Yun,Wu Xi	职业与健康	Occupation and health	35	17	2341-2345	2019	-
5986	餐中食源性致病菌及毒素	ria and toxin contaminatio	梅,唐震,甄世祺,乔昕,倪	ang Zhen,Zhen Shiqi,Qiao	中国卫生检验杂志	ese Journal of Health Inspe	29	9	1119-1121	2019	-
5987	食品中沙门菌污染状况监	aracteristics of Salmonella	,莫浩联,杨梅,李玲玲,刘	o Haolian,bayberry,Li Lingl	热带医学杂志	journal of tropical medicine	19	7	913-917	2019	sn.1672-3619.2019.07.025
5988	地区野禽沙门菌的分离鉴	drug resistance analysis of	,冯政,张伟伟,李超,李刚,	ng,Zhang Weiwei,Li Chao,	中国家禽	Chinese poultry	-	9	33-37	2019	sn.1004-6364.2019.09.006
5989	常见沙门菌的脉冲场凝胶	ce spectrum characteristics	锋,熊燕,陈智,王芳,张卉,赵	an,Chen Zhi,Wang Fang,Zh	中科技大学学报（医学版	versity of Science and Tech	48	5	549-555	2019	sn.1672-0741.2019.05.011
5990	沙门菌的分型及同源性	ance analysis of Salmonella	周俊英,田宏攀.	Zhou Junying,Tian Hongpan	现代检验医学杂志	l of Modern Laboratory Me	34	1	83-84,88	2019	sn.1671-7414.2019.01.021
5991	菌快速分型检验中实时荧	st of frozen poultry and live	李丹妮.	Li Danni.	食品界	food industry	-	2	75,77	2019	n.2095-638X.2019.02.049
5992	狼疮并沙门菌感染致髋关	temic lupus erythematosus	海彬,何伟,张庆文,陈镇	,He Wei,Zhang Qingwen,C	华关节外科杂志（电子版	nal of Joint Surgery (electr	13	4	517-520	2019	n.1674-134X.2019.04.023

5993	菌鉴别培养基和PCR检测方	on Medium andPCREstabl	艳,徐煜琳,鞠孜敬,崔治中	an,Xu Yulin,Ju Zijing,Cui Zhi	山东畜牧兽医	mal Husbandry and Veteri	40	10	1-3	2019	sn.1007-1733.2019.10.001
5994	沧州市食品中食源性致病	oodborne pathogenic bact	玉梅,任海鹏,王春东,卢俊	n Haipeng,Wang Chuntong	医学动物防制	medical animal control	35	3	263-266	2019	0.7629/yxdwfz201903016
5995	病牛沙门菌致盆腔脓肿1例	aused by Salmonella in sick	周辉,高硕,张之烽,周万青	ao Shuo,Zhang Zhifeng,Zho	临床检验杂志	journal of clinical testing	37	7	557-558	2019	602/j.cnki.jcls.2019.07.19
5996	18年吉安市水禽新发病调	w diseases of waterfowl i	,匡贤珠,胡爱明,刘建林,李	g Xianzhu,Hu Aiming,Liu Ji	江西畜牧兽医杂志	mal Husbandry and Veterin	-	1	49-50	2019	sn.1004-2342.2019.01.017
5997	食源性疾病暴发事件的流行	traceability of a foodborne	三桃,杨红霞,李雪原,王洋	dao,Yang Hongxia,Li Xueyu	中国药物与临床	hinese medicine and clinica	19	4	553-555	2019	1655/zgywylc2019.04.008
5998	5年全国血流感染细菌耐药	ream Infection Bacterial R	郑琳,顾蓓青,董海新,宛传	in,Gu Beiqing,Dong Haixin,	中华临床感染病杂志	ournal of Clinical Infectious	12	1	24-37	2019	sn.1674-2397.2019.01.005
5999	人员沙门氏菌的血清型分布	distribution of Salmonella	梁炎,何燕,王婷.	iang Yan,He Yan,Wang Ting	临床检验杂志（电子版）	f Clinical Testing (electroni	8	2	6-7	2019	-
6000	区2018年食源性疾病主动	ysis of annual foodborne d	齐迹,李鹏高.	All traces,Li Penggao.	食品安全质量检测学报	f Food Safety and Quality I	10	12	3760-3764	2019	sn.2095-0381.2019.12.022
6001	验在季节性腹泻检验中的	f routine stool examination	邹晓旭.	Zou Xiaoxu.	养生保健指南	Health and Wellness Guide	-	46	32	2019	-
6002	嘉兴市属医院粪便检出沙	lmonella detected in feces	陈兴英,王琼琼.	en Xingying,Wang Qiongqio	中国卫生检验杂志	ese Journal of Health Inspe	29	9	1060-1062	2019	-
6003	腹泻病例中沙门氏菌分子	d drug resistance of Salmo	迪,徐可心,郭海燕,程利红,	Kexin,Guo Haiyan,Cheng Li	中国人兽共患病学报	Chinese Journal of Zoonose	35	10	915-921	2019	sn.1002-2694.2019.00.109
6004	鲁木齐市细菌性腹泻病监测	al diarrheal disease surveil	牟文婷,杨毅,王启果.	Wenting,Yang Yi,Wang Qi	医学信息文摘（连续型电	information abstract (con	19	46	49-51	2019	ki.1671-3141.2019.46.021
6005	年云南省食源性沙门氏菌	nce surveillance of foodbo	祖顺,田云屏,国译丹,任翔,	ian Yunping,Guoyidan,Ren	食品安全质量检测学报	f Food Safety and Quality I	10	22	7601-7605	2019	-
6006	血液标本后对微生物检验结	tion on blood specimens o	高艳萍.	Gao Yanping.	实用医技杂志	l of Practical Medical Tech	26	5	579-580	2019	ki.1671-5098.2019.05.020
6007	年武汉市食源性疾病致病因	is of pathogenic factors of	婷,王肖,石梦蝶,孙言凤,王	Xiao,Shi Mengdie,Sun Yan	现代预防医学	modern preventive medicin	46	7	1199-1202,1237	2019	-
6008	肠杆菌、沙门氏杆菌耐药	e of chicken-derived Esche	陈春林,冯刚,秦杨,付利芝	hunlin,Feng Gang,Qin Yang,	黑龙江畜牧兽医	nimal Husbandry and Veter	-	12	85-88	2019	nki.hljxmsy.2018.07.0325
6009	阴市其他感染性腹泻病的	cteristics of other infectiou	华,马焰,姚建香,颜雅娟,李	,Ma Yan,Yao Jianxiang,Yan	现代预防医学	modern preventive medicin	46	8	1357-1359,1381	2019	-
6010	8年感染性腹泻致病菌检出	detection rate of pathoger	石晓娈,徐勇.	Shi Xiaojuan,Xu Yong.	家庭医药	home medicine	-	11	394	2019	-
6011	养殖场沙门菌污染状况及	ation status and bacterial	玲清,陈伟伟,李闽真,马群	,Chen Weiwei,Li Minzhen,	海峡预防医学杂志	s Journal of Preventive Me	25	1	75-76	2019	-
6012	验在季节性腹泻检验中的	e stool examination in det	张会品.	Zhang Huipin.	科口腔医学杂志（电子版	General Dentistry (electron	6	25	183190	2019	-
6013	泻病例中肠炎沙门菌和鼠	la enteritidis and Salmonel	国强,李颖,马红梅,张松建	,Li Ying,Ma Hongmei,Zhan	疾病监测	disease surveillance	34	11	987-993	2019	sn.1003-9961.2019.11.009
6014	区食源性疾病主动监测实	disease active surveillance	李丽燕,李静虹.	Li Liyan,Li Jinghong.	临床检验杂志（电子版）	f Clinical Testing (electroni	8	2	16-18	2019	-
6015	北京市某院感染性腹泻的病	l characteristics of infectio	王玫,李佐君.	Wang Mei,Li Zuojun.	河北医药	Hebei Medicine	41	23	3650-3653	2019	sn.1002-7386.2019.23.034
6016	者的病原微生物检验结果及	t results and drug sensitivit	李义斌.	Li Yibin.	中外女性健康研究	nd foreign women's health	-	3	123180	2019	-
6017	氏菌分离鉴定、血清分型及	ensitivity analysis of Salmo	元冉,袁聪,李红婵,高延玲	uan Cong,Li Hongchan,Gao	中国动物检疫	China Animal Quarantine	36	4	74-78	2019	n.1005-944X.2019.04.018
6018	门氏杆菌分离鉴定及体外抑	vitro bacteriostatic test of S	侯美东.	Hou Meidong.	畜牧兽医科学（电子版）	y and Veterinary Science (E	-	24	21-22	2019	-
6019	在临床诊断季节性腹泻中的	utine stool examination in	金波.	Jinbo.	航空航天医学杂志	urnal of Aerospace Medici	30	6	687-689	2019	sn.2095-1434.2019.06.020
6020	性食物中毒的微生物学检验	nination and analysis of bac	张磊.	Zhang Lei.	当代医学	contemporary medicine	25	1	50-52	2019	sn.1009-4393.2019.01.019
6021	沙门氏菌流行现状调查及耐	us of Salmonella and analy	丁丽军,卢劲晔,羊建平,沈	ijun,Lu Jinye,Yang Jianping,	江苏农业科学	iangsu Agricultural Science	47	2	157-159	2019	sn.1002-1302.2019.02.039
6022	对一起肠炎沙门菌食物中	monella Enteritidis food po	春敏,叶素贞,胡凤清,叶丽	min,Ye Suzhen,Hu Fengqing	海峡预防医学杂志	s Journal of Preventive Me	25	3	73-74	2019	-
6023	腹泻病中沙门氏菌的感染	stics of Salmonella in adult	晓芸,施怡茹,张相猛,徐兴	hi Yiru,Zhang Xiangmeng,X	检验医学与临床	boratory medicine and clini	16	21	3077-3080	2019	sn.1672-9455.2019.21.002
6024	利斯沙门菌耐药特征及分	cs and molecular typing of	丽,黄胜中,叶雪仪,张莉萍	g Shengzhong,Ye Xueyi,Zha	华南预防医学	uth China Preventive Medic	45	1	58-61	2019	.13217/j.scjpm.2019.0058
6025	坊市食源性沙门氏菌分子	esistance monitoring of foo	卓,张丽敏,王敏娣,陈忠建,	ng Limin,Wang Mindi,Chen	医学动物防制	medical animal control	35	3	217-221	2019	0.7629/yxdwfz201903004
6026	鸡源沙门菌分离鉴定及耐	ce analysis of Salmonella	阳,张天姿,刘维,周祖涛,徐	hang Tianzi,Liu Wei,Zhou Z	中国畜牧兽医	mal husbandry and veterina	46	3	931-939	2019	ki.1671-7236.2019.03.035
6027	军总医院沙门菌分子分型和	rug resistance gene charac	,叶坤,李鑫,叶丽艳,张樱,仲	,Li Xin,Ye Liyan,Zhang Ying	中华检验医学杂志	e Journal of Laboratory Me	42	3	209-213	2019	sn.1009-9158.2019.03.012
6028	市通州区感染性腹泻细菌	al pathogen spectrum of in	高翔,赵凤玲,高洁,张扬,李	g,Zhao Fengling,Gao Jie,Pu	疾病监测	disease surveillance	34	9	800-804	2019	sn.1003-9961.2019.09.007
6029	服务行业健康人群携带沙门	among healthy people in	朱莹莹.	Zhu Yingying.	中国卫生标准管理	a Health Standard Manage	10	6	5-7	2019	sn.1674-9316.2019.06.003

6030	肠加工过程中微生物风险	robial risks during ham sau	林,裴晓燕,朱海明,黄玉兰,	Xiaoyan,Zhu Haiming,Mag	中国食品卫生杂志	hinese Food Hygiene Journ	31	1	59-63	2019	).13590/j.cjfh.2019.01.013
6031	病患者院内感染病原菌分	athogenic bacteria in noso	叶晓洁,季显琼.	Ye Xiaojie,Ji Xianqiong.	中国医院统计	Chinese hospital statistics	26	1	50-53	2019	sn.1006-5253.2019.01.014
6032	学校感染性腹泻事件病原	of an infectious diarrhea i	,林春燕,朱斐莉,王明昌,	hunyan,Zhu Lili,Wang Ming	中国学校卫生	Chinese school health	40	8	1267-1269	2019	ki.1000-9817.2019.08.042
6033	的致病因素的临床治疗方	and effects of causative fa	李艳萍.	Li Yanping.	智慧健康	Smart health	5	10	132-133	2019	ki.2096-1219.2019.10.056
6034	南省外卖餐饮的微生物污	tamination status of take-	范璐,汤小召,杨菁,何玉凤,	ng Xiaozhao,Yang Jing,He Y	食品安全质量检测学报	f Food Safety and Quality I	10	22	7633-7638	2019	-
6035	3种即食类地方食品的致	pathogenic bacteria conta	正勇,李艳芬,炊慧霞,张秀	yong,Li Yanfen,Cui Huixia,Z	中国卫生检验杂志	ese Journal of Health Inspe	29	9	1122-1123,1152	2019	-
6036	鸡源沙门氏菌耐药性与致	resistance and pathogenici	王孝德,黎宗强,尹彦文,屈	Wang Xiaode,Li Zongqiang,	南方农业学报	urnal of Southern Agricultu	50	10	2350-2358	2019	ssn.2095-1191.2019.10.28
6037	博市1088份市售食品监测	Analysis of monitoring resu	洋,杨爱青,徐一凡,杨洪汲	Yang Aiqing,Xu Yifan,Yang	医学动物防制	medical animal control	35	5	476-478,481	2019	0.7629/yxdwfz201905020
6038	链中沙门菌优势血清型	in virulence factors in the p	萍,刘静,刘英姿,高慎阳,周	iu Jing,Liu Yingzi,Gao Sheny	中国兽医学报	al of Chinese Veterinary Me	39	8	1540-1544,1565	2019	nki.1005-4545.2019.08.19
6039	食品中沙门氏菌污染状况	status and serotype distrib	志琴,路立立,徐礼扬,杨晓	o,Lu Lili,Xu Liyang,Yang Xia	河南预防医学杂志	n Journal of Preventive Me	30	10	797-800	2019	.1006-8414.2019.010.023
6040	年吉林地区仔猪腹泻流行	e prevalence of piglet diar	东旭,沙万里,尹柏双,李国	,Shawanli,Yin Baishuang,L	黑龙江畜牧兽医（上半月）	ndry and Veterinary Medic	-	8	90-93	2019	nki.hljxmsy.2018.09.0261
6041	食品中沙门菌污染状况及	ination status and characte	蓉,章乐怡,李毅,胡玉琴,吴	,Zhang Leyi,Li Yi,Hu Yuqin,	中国食品卫生杂志	hinese Food Hygiene Journ	31	5	461-464	2019	).13590/j.cjfh.2019.05.012
6042	湖南省其他感染性腹泻流	characteristics of other in	红,孙倩莱,杨浩,曾舸,张	Sun Qianlai,Yang Hao,Zeng	实用预防医学	ractical preventive medicin	26	1	51-54	2019	sn.1006-3110.2019.01.013
6043	型分子检测靶点筛选及多	xing of Salmonella Delphi s	,杨剑婷,李永泉,王水平,	anting,Li Yongquan,Wang l	食品与发酵工业	od and Fermentation Indus	45	7	269-275	2019	5/j.cnki.11-1802/ts.019243
6044	的污染及其导致的食源性	food by Salmonella and th	张丹.	Zhang Dan.	中国保健营养	Chinese health nutrition	29	12	396	2019	sn.1004-7484.2019.12.377
6045	菌肠道感染的流行病学特	susceptibility results of non	雷旻,周高枫,雷炎玲,谭锟	Jin,Zhou Gaofeng,Lei Yanlin	儿科药学杂志	ournal of Pediatric Pharmac	25	2	37-40	2019	p.1672-108X.2019.02.011
6046	性肠炎沙门菌流行病学特	mology analysis of food-b	凤岩,凤淳雅,郭世杰,陶	yan,Feng Chunya,Guo Shij	中国生物制品学杂志	se Journal of Biological Pro	32	9	1001-1005	2019	-
6047	市部分地区羊肉制品食源	nic contamination of mutt	李鹏.	Li Peng.	中国草食动物科学	Chinese herbivore science	39	1	52-54	2019	sn.2095-3887.2019.01.011
6048	四川省家庭内食源性疾病	rne disease outbreaks in h	陈文,兰真,程刚,张誉,宋阳	n Zhen,Cheng Gang,Zhang	现代预防医学	modern preventive medicin	46	23	4391-4395	2019	-
6049	清凝集分型和分子血清分	nation typing and molecula	悦,胡凤月,张熙雅,巩有博,	engyue,Zhang Xiya,Gong Y	食品安全质量检测学报	f Food Safety and Quality I	10	18	6068-6077	2019	-
6050	市门头沟区感染性腹泻病	sults of infectious diarrhea	艳,李龙建,刘海涛,褚添,赵	ongjian,Liu Haitao,Chu Tian	疾病监测	disease surveillance	34	6	514-518	2019	sn.1003-9961.2019.06.010
6051	市售食品中食源性致病菌	results of foodborne path	马娟.	Ma Juan.	中国保健营养	Chinese health nutrition	29	8	278-279	2019	sn.1004-7484.2019.08.352
6052	常见沙门菌血清型分离株	tes in Wuhan and NingboP	熊燕,陈智,陈中举,王芳,方	hen Zhi,Chen Zhongju,War	中华医院感染学杂志	ournal of Hospital Infectiou	29	20	3041-3049	2019	.11816/cn.ni.2019-191950
6053	中全面生化反应和血清学	biochemical reactions and	谭君.	Tan Jun.	养生保健指南	Health and Wellness Guide	-	22	112	2019	sn.1006-6845.2019.22.103
6054	古自治区肉及肉制品中食	bacteria in meat and meat	乌伊罕,王利平,张冰冰.	an,Wang Liping,Zhang Bing	现代预防医学	modern preventive medicin	46	1	44-47	2019	-
6055	门菌流行趋势与耐药情况	rends and drug resistance	骆艳,李中跃.	Luo Yan,Li Zhongyue.	中国当代医药	inese contemporary medic	26	14	41-44	2019	sn.1674-4721.2019.14.013
6056	食物中毒脉冲场凝胶电泳	drug susceptibility analysis	峰,王红,赵婷,廖春艳,代	ang Hong,Zhao Ting,Liao C	国际检验医学杂志	onal journal of laboratory	40	17	2151-2154	2019	sn.1673-4130.2019.17.025
6057	利斯沙门菌药物敏感试验	lecular typing analysis of f	王雪竹,炊慧霞,赵一真.	Xuezhu,Cui Huixia,Zhao Yi	食品安全质量检测学报	f Food Safety and Quality I	10	3	583-588	2019	sn.2095-0381.2019.03.005
6058	蛋鸡养殖场常见疫病感染	ease infections in a green-s	马光强.	Ma Guangqiang.	黑龙江畜牧兽医（上半月）	ndry and Veterinary Medic	-	5	91-95,182	2019	nki.hljxmsy.2018.03.0411
6059	沙门氏菌院内感染病例病	mial infection cases caused	,谢清梅,谢志强,李艳艳,	Qingmei,Xie Zhiqiang,Li Yan	医学动物防制	medical animal control	35	5	461-463	2019	0.7629/yxdwfz201905015
6060	氏杆菌的临床分离鉴定及	n and drug resistance analy	贾荣玲,刘耀东,李生涛.	ongling,Liu Yaodong,Li Shen	黑龙江畜牧兽医（上半月）	ndry and Veterinary Medic	-	9	101-104	2019	nki.hljxmsy.2018.10.0417
6061	熟肉制品生产加工过程	n and processing of sauce-	国,金迪,王艳莉,江晓,叶	o,Jindi,Wang Yanli,Jiang Xia	中国食品卫生杂志	hinese Food Hygiene Journ	31	5	465-469	2019	).13590/j.cjfh.2019.05.013
6062	坊市食品中沙门氏菌污染	contamination status and	卓,张丽敏,王敏娣,陈忠健,	ng Limin,Wang Mindi,Chen	医学动物防制	medical animal control	35	2	134-136	2019	0.7629/yxdwfz201902008
6063	人感染性腹泻肠道致病菌	hogenic bacteria in adults	阳,杨江辉,王利君,段宁,	ng Jianghui,Wang Lijun,Dua	标记免疫分析与临床	eled immunoassays and cli	26	5	777-780	2019	sn.1006-1703.2019.05.014
6064	区即食米粉食源性致病	nic bacteria contamination	罗素梅,陈媚,何礼,龙奇志.	l May,Chen Mei,He Li,Long	河南预防医学杂志	n Journal of Preventive Me	30	7	555-557	2019	m.1006-8414.2019.07.024
6065	沙门氏菌(ATCC13076)的	on lettuce(ATCC13076)a	,马文娟,李晚宁,王佳,王	njuan,Li Wanning,Wang Jia,	现代食品科技	Modern food technology	35	11	170-175,309	2019	fst.1673-9078.2019.11.024
6066	株洲市感染性腹泻病原学	ce results of infectious diar	志,龙英姿,胡世雄,何礼,罗	ng Yingzi,Hu Shixiong,He L	实用预防医学	ractical preventive medicin	26	5	584-587	2019	sn.1006-3110.2019.05.020

6067	四环素类药物耐药性和耐	resistance and resistance g	建华,吴宁鹏,贺丹丹,潘玉	u Ningpeng,He Dandan,Pa	中国兽医学报	l of Chinese Veterinary Me	39	10	1927-1933	2019	nki.1005-4545.2019.10.08
6068	菌致新生儿败血症及细菌性	a and bacterial meningitis	张金晶,王亚娟,刘蕾,邵芳	jing,Wang Yajuan,Liu Lei,Sl	华新生儿科杂志（中英文	al of Neonatology (Chinese	34	2	142-143	2019	sn.2096-2932.2019.02.014
6069	2017年沙门菌耐药菌谱及	f drug-resistant bacterial s	悦康,刘绮明,区金结,仇绮	Yuekang,Liu Qiming,Ou Jin	中国热带医学	Chinese tropical medicine	19	5	459-462	2019	cnki.46-1064/r.2019.05.13
6070	品食源性致病菌污染状况及	nation status and related r	胡汝源,陈冉,杨红菊.	Ruyuan,Chen Ran,Yang Hor	食品安全质量检测学报	f Food Safety and Quality I	10	22	7756-7761	2019	-
6071	Y县某酒店疑似食物中毒	suspected food poisoning ir	李俊,吴静,王帅.	Li Jun,Wu Jing,Wang Shuai.	百科论坛电子杂志	pedia Forum Electronic Ma	-	8	536	2019	-
6072	年襄阳市食源性疾病监测	dborne disease surveillance	汪雪洋,邓万霞,张金枝.	ueyang,Deng Wanxia,Zhan	公共卫生与预防医学	Health and Preventive Me	30	3	95-98	2019	sn.1006-2483.2019.03.022
6073	性腹泻的病原微生物检验	organism test results for b	杨颖,李付广.	Yang Ying,Li Fuguang.	保健文汇	Health Wenhui	-	7	177-178	2019	sn.1671-5217.2019.07.088
6074	市西城区肠炎沙门菌耐药	molecular typing of Salmo	哲,王永全,徐俊,苗芳,崔京	Yongquan,Xu Jun,Miao Fa	职业与健康	Occupation and health	35	8	1055-1058	2019	-
6075	感染暴发的病原学分析及	olecular typing of an outbre	朱艳,贾宇驰,郭文学,祁伟	an,Jia Yuchi,Guo Wenxue,Q	中华传染病杂志	se Journal of Infectious Dis	37	6	353-356	2019	sn.1000-6680.2019.06.007
6076	市售食品中食源性致病菌	orne pathogenic bacteria ir	麟,余超,刘于飞,梁伯衡,林	hao,Liu Yufei,Liang Boheng	食品安全质量检测学报	f Food Safety and Quality I	10	12	3975-3980	2019	sn.2095-0381.2019.12.057
6077	区2016年-2017年食源性疾	ear-2017Annual Foodborne	赵静,肖贵勇,王佳佳,马晓	ing,Xiao Guiyong,Wang Jiaj	中国卫生检验杂志	ese Journal of Health Inspe	29	4	490-493	2019	-
6078	性感染性腹泻的病原菌及耐	d drug resistance of acute	红,彭玲,王春清,李锋,王	g Ling,Wang Chunqing,Li F	中国国境卫生检疫杂志	ntier Health and Quarantin	42	5	345-348	2019	/j.1004-9770.2019.05.012
6079	海市食品中食源性致病菌	of foodborne pathogenic	昭宁,白海涛,李师勤,胡小	aoning,Bai Haitao,Li Shiqin,	中国卫生检验杂志	ese Journal of Health Inspe	29	16	2017-2019	2019	-
6080	-2017年市售食品微生物检	robiological testing results	李芳,郑升龙.	Li Fang,Zheng Shenglong.	中国公共卫生管理	ese public health managen	35	1	111-113	2019	cnki.23-1318.2019.01.031
6081	海市疑似细菌性食物中毒	sults of suspected bacterial	昭宁,白海涛,李师勤,胡小	hing,Bai Haitao,Li Shiqin,Hu	食品安全导刊	food safety guide	-	6	169-170,175	2019	-
6082	伤寒沙门氏杆菌的分离鉴定	nce analysis of Salmonella t	天伦,徐磊,杨跃飞,王彦红	u Lei,Yang Yuefei,Wang Ya	黑龙江畜牧兽医（上半月）	ndry and Veterinary Medic	-	1	81-83	2019	nki.hljxmsy.2017.12.0240
6083	2016年食品中沙门菌污染	l monitoring and analysis c	松,刘勋,郑文,李征莉,肖秀	g,Liu Xun,Zheng Wen,Li Zhe	中国卫生检验杂志	ese Journal of Health Inspe	29	5	621-622,626	2019	-
6084	上半年重要禽病检测结果	of important poultry disea	,杨沛沛,王洋洋,习向锋,赵	ei,Wang Yangyang,Xi Xiang	中国动物保健	China animal health	21	8	10-13	2019	sn.1008-4754.2019.08.007
6085	儿童医院临床分离细菌分布	nitoring of clinically isolated	崔晓燕,陈虹宇,孙丽芳,杨	Xiaoyan,Chen Hongyu,Sun	中国感染与化疗杂志	urnal of Infection and Cher	19	4	417-424	2019	/j.1009-7708.2019.04.015
6086	型沙门氏菌共同引起的食源	he illness outbreak caused	邵伟,胡世雄,贾华云,王岚,	Shixiong,Jia Huayun,Wang	实用预防医学	ractical preventive medicin	26	7	857-860	2019	sn.1006-3110.2019.07.023
6087	患者机会性感染病原菌监	of pathogenic bacteria in pa	张帮林,郑琳.	Zhang Banglin,Zheng Lin.	热带医学杂志	ournal of tropical medicine	19	3	333-336	2019	sn.1672-3619.2019.03.018
6088	沙门菌分子分型、毒力基因	resistance determination	美华,曹春远,廖琳虹,陈海	Chunyuan,Liao Linhong,C	中华微生物学和免疫学杂志	rnal of Microbiology and Ir	39	11	805-811	2019	sn.0254-5101.2019.11.001
6089	玉溪市售肉及肉制品细菌性	tamination of meat and m	,马惆卿,向正华,李旭东,	gqing,Xiang Zhenghua,Li X	食品安全质量检测学报	f Food Safety and Quality I	10	22	7682-7693	2019	-
6090	年婴幼儿腹泻病原因子及	es and characteristics of dia	,仇广翠,唐芹方,顾春森,	gcui,Tang Qinfang,Gu Chur	江苏预防医学	iangsu Preventive Medicine	-	5	553-554	2019	sn.1006-9070.2019.05.028
6091	18年无锡市食源性疾病监	dborne disease surveillance	玲芬,殷俊,刘萍,任梁,周伟	,Yin Jun,Liu Ping,Ren Liang	职业与健康	Occupation and health	35	21	2944-2947	2019	-
6092	中全面生化反应和血清学	hemical reactions and sero	何琳.	He Lin.	医学美学美容	edical Aesthetics and Beau	28	15	82	2019	-
6093	-2018年腹泻病人沙门菌血	ing and molecular typing o	张旭,牛娜,陈洪友,许学斌	,Niu Na,Chen Hongyou,Xu	中国热带医学	Chinese tropical medicine	19	6	503-507	2019	cnki.46-1064/r.2019.06.01
6094	卑沙门菌脉冲场凝胶电泳	nd drug resistance of Salm	云屏,任翔,邹颜秋硕,汤晓	Ren Xiang,Zou Yanqiushuo	食品安全质量检测学报	f Food Safety and Quality I	10	22	7707-7714	2019	-
6095	沙门氏菌血清型分布及分	ype distribution and molec	慧欣,陈泽辉,蔡薇,游小伟	ixin,Chen Zehui,Cai Wei,Yo	现代预防医学	nodern preventive medicin	46	7	1248-1254	2019	-
6096	门菌血流感染致脊膜脊神经	litis caused by Salmonella	丹,安康,姚远,牟鈃雨,李双	eing,Yao Yuan,Mou Linyu,Li	中华全科医师杂志	e Journal of General Practi	18	12	1169-1170	2019	sn.1671-7368.2019.12.012
6097	的临床分布及其对抗菌素的	of Salmonella Paratyphi A	梁绪东.	Liang Xudong.	健康必读	Health must read	-	12	130	2019	-
6098	鸡场沙门菌流行病学调查和	sistance analysis of Salmon	,刘桂凤,张健,王凡,刘博	uifeng,Zhang Jian,Wang Fa	中国家禽	Chinese poultry	-	2	49-52	2019	sn.1004-6364.2019.02.011
6099	县某镇一起伤寒暴发疫情	typhoid fever outbreak in a	夏军林.	Xia Junlin.	河南预防医学杂志	Journal of Preventive Me	30	7	541-543	2019	m.1006-8414.2019.07.019
6100	中沙门氏菌感染状况及耐	nd drug resistance status a	莉,朱晓露,马会会,巩方文	Xiaolu,Jockey Club,Gong F	检验医学与临床	oratory medicine and clini	16	17	2523-2525	2019	sn.1672-9455.2019.17.031
6101	山州市售食品食源性致病	odborne pathogenic bacte	张练,白丽晶,田会涛.	ang Lian,Bai Lijing,Tian Huit	食品安全质量检测学报	f Food Safety and Quality I	10	22	7649-7655	2019	-
6102	品中沙门氏菌血清分型及脉	d gel electrophoresis typin	玲,倪刚,徐晋莹,吴春梅,李	ng,Xu Jinying,Wu Chunmei,	食品安全质量检测学报	f Food Safety and Quality I	10	22	7694-7700	2019	-
6103	275例儿童感染沙门菌的临	bution and drug resistance a	曾莉,景春梅.	Zeng Li,Jing Chunmei.	重庆医学	Chongqing Medicine	48	23	4131-4133	2019	sn.1671-8348.2019.23.045

6104	食品风险微生物及致病因	s of food risk microorganis	廖怀平,张鹏.	Liao Huaiping,Zhang Peng.	中国保健营养	Chinese health nutrition	29	14	282-283	2019	-
6105	双流国际机场航空食品微	alysis of aviation food at C	邓晓东,钟玮,高国龙,张青	ng,Zhong Wei,Gao Guolong	中国国境卫生检疫杂志	ntier Health and Quarantin	42	3	185-187	2019	3/j.1004-9770.2019.03.010
6106	院2016-2018年腹泻病原	2018Annual surveillance an	俊,许姜姜,严剑婷,刘永斌,	ngjiang,Yan Jianting,Liu Yon	实用预防医学	ractical preventive medicin	26	11	1369-1372	2019	sn.1006-3110.2019.11.024
6107	泻临床特征与病原菌分布	d drug resistance of patho	胡玲,王宝香,梅红,赵玉霞	Vang Baoxiang,plum red,Zh	中华医院感染学杂志	ournal of Hospital Infectiou	29	14	2237-2240	2019	.11816/cn.ni.2019-181796
6108	份高铁快餐盒饭微生物指	ial contamination status of	王昭妍,丛楠楠.	Vang Zhaoyan,Cong Nannan	预防医学情报杂志	of Preventive Medicine Info	35	2	165-167	2019	-
6109	人源与食源性沙门菌血清	nce characteristics of huma	伟伟,杨劲松,徐海滨,罗朝	ei,Yang Jinsong,Xu Haibin,L	疾病监测	disease surveillance	34	4	316-321	2019	sn.1003-9961.2019.04.009
6110	腹泻病主要病原菌的分离	on of the main pathogenic	兴民,张焕容,毛从剑,彭纯	ang Huanrong,Mao Congjia	黑龙江畜牧兽医（下半月）	dry and Veterinary Medicin	-	2	78-82	2019	cnki.hljxmsy.2018.01.0395
6111	源沙门氏菌的分离鉴定及	ce analysis of Salmonella o	宋欣媛,谭珊,吴培福.	g Xinyuan,Tan Shan,Wu Pe	现代畜牧兽医	mal husbandry and veterina	-	4	1-5	2019	-
6112	泻的80例病原微生物检验	0Analysis of pathogenic m	漆兆李.	Qi Zhaoli.	健康必读	Health must read	-	9	71-72	2019	-
6113	地区小儿腹泻常见致病因素	tive factors of diarrhea in c	张贤梓,黄会平.	Zhang Xianzi,Huang Huiping	中国妇幼保健	ina maternal and child hea	34	6	1291-1293	2019	ssn.1001-4411.2019.06.27
6114	理白族自治州食源性疾病暴	se outbreak surveillance re	尹顺珠.	Yin Soon-joo.	中华灾害救援医学	inese disaster relief medici	7	5	245-248	2019	sn.2095-6274.2019.05.002
6115	台区肠道致病菌检出及耐药	resistance of intestinal pa	,余红,尉秀霞,石婧,曲梅,	Hong,Wei Xiuxia,Shi Jing,Qu	中国卫生检验杂志	ese Journal of Health Inspe	29	8	928-930,936	2019	-
6116	区生产及零售端鸡肉源沙门	a from Chicken Production	,肖英平,赵瑞婷,刘晨星,杨	ingping,Zhao Ruiting,Liu Cl	浙江农业学报	urnal of Zhejiang Agricultu	31	7	1161-1169	2019	ssn.1004-1524.2019.07.17
6117	区儿童感染性腹泻病原菌	bacteria in children with inf	余玮,孙攀,彭谦,宣栋樑.	Sun Pan,Peng Qian,Xuando	中国卫生标准管理	a Health Standard Manager	10	7	82-85	2019	sn.1674-9316.2019.07.035
6118	市龙华区食源性所致感染	food-borne infectious diarr	何林,梅树江,廖玉学,卿文	,meishujiang,Liao Yuxue,Qi	预防医学情报杂志	of Preventive Medicine Info	35	5	439-445	2019	-
6119	加血清学在沙门菌临床检验	ve biochemical reactions a	王昭晴.	Wang Zhaoqing.	临床检验杂志（电子版）	f Clinical Testing (electroni	8	1	170	2019	-
6120	菌β-内酰胺耐药表型及ES	eβ-Lactam resistance phe	陈建辉,黄梦颖,杨劲松,林杰	,Huang Mengying,Yang Jin	中国人兽共患病学报	Chinese Journal of Zoonose	35	10	944-949	2019	sn.1002-2694.2019.00.096
6121	市食源性疾病主动监测细菌	s of active surveillance of fo	郭凯,刘晓琳,王伟栋.	Kai,Liu Xiaolin,Wang Weid	中国卫生检验杂志	ese Journal of Health Inspe	29	2	242-244	2019	-
6122	童腹泻流行特征及致泻性	hea in Shanghai and analys	顾文超,孔令娜,王瑞,蒋潇	hao,Kong Lingna,Wang Rui	疾病监测	disease surveillance	34	6	559-564	2019	sn.1003-9961.2019.06.019
6123	区沙门菌流行病学及耐药	g resistance analysis of Sal	,杨登辉,王栋,田明星,丁	ui,Wang Dong,Tian Mingxi	中国动物传染病学报	ournal of Animal Infectious	27	1	49-54	2019	-
6124	病患儿进行病原微生物检	nic microorganism test resu	施瑜.	Shi Yu.	当代医药论丛	ontemporary Medicine Seri	17	3	184-185	2019	-
6125	郑州市食源性致病菌监测	results of foodborne patho	蒋诗苑,赵瑞臻,孙丽梅,牛	yuan,Zhao Ruizhen,Sun Lim	中国卫生检验杂志	ese Journal of Health Inspe	29	2	248-250,253	2019	-
6126	毒检测阴性的腹泻患儿病	children with diarrhea who	劲涛,彭朝阳,厉小玉,徐丽	,Peng Chaoyang,Li Xiaoyu,	中华检验医学杂志	e Journal of Laboratory Me	42	8	688-693	2019	sn.1009-9158.2019.08.019
6127	肠炎沙门菌致败血症1例	la enteritidis causes sepsis	王艳,刘宏蕴,翁绳凤.	Yan,Liu Hongyun,Weng She	中国乡村医药	Chinese rural medicine	26	23	36	2019	sn.1006-5180.2019.23.022
6128	痘合并脓毒血症回顾性分	severe chickenpox complic	凌,王福祥,杨柳青,孟令香	Fuxiang,Yangliuqing,Meng	中国社区医师	hinese community physicia	35	29	12-13	2019	sn.1007-614x.2019.29.007
6129	市80份食品中食源性致病	onitoring results of foodbo	蕾,马蕾,王宜凤,王勇,孟	Lei,Wang Yifeng,Wang Yon	食品安全导刊	food safety guide	-	25	59-61	2019	043/j.cnki.cfs.2019.25.019
6130	年新乡市食源性致病菌监测	ysis of foodborne pathogen	红霞,史晓娟,芦天成,王伟	,Shi Xiaojuan,Lu Tiancheng	河南预防医学杂志	n Journal of Preventive Me	30	12	956-958	2019	m.1006-8414.2019.12.026
6131	序中沙门菌和大肠杆菌污	a and E. coli contamination	光,梁茂文,赵瑞生,王呈,	g Maowen,Zhao Ruisheng,	畜牧与饲料科学	Livestock and feed science	40	1	19-23	2019	-
6132	氏菌的分离鉴定、耐药及	ce and virulence analysis of	晓莉,程凯慧,张亮,楚会萌	eng Kaihui,Zhang Liang,Ch	山东农业科学	andong Agricultural Scienc	51	11	143-148	2019	sn.1001-4942.2019.11.029
6133	市北仑区熟肉制品风险监	esults of cooked meat prod	书奇,胡群雄,徐明敏,王雪	u Qunxiong,Xu Mingmin,W	河南预防医学杂志	n Journal of Preventive Me	30	10	801-802,封3	2019	n.1006-8414.2019.010.024
6134	泻病原微生物临床检验分	ganisms and preventive me	刘永凤.	Liu Yongfeng.	健康之友	health friend	-	3	180-181	2019	-
6135	年家庭食源性疾病暴发事	characteristics of annual ho	,李薇薇,杨淑香,郭云昌,	eiwei,Yang Shuxiang,Guo Yu	中国公共卫生	Chinese public health	35	10	1379-1382	2019	10.11847/zgggws1123875
6136	腹泻患者便常规检验诊断	e of routine stool tests in p	宣萍.	Xuan Ping.	医药前沿	Medical Frontier	9	23	95	2019	-
6137	菌性食物中毒的病原菌与	f bacterial food poisoning a	王玉梅.	Wang Yumei.	中国医药指南	Chinese Medicine Guide	17	33	390-391	2019	-
6138	坡头区感染性腹泻细菌病	acterial pathogen spectrum	杨吉凯,李悦丰,吕红旭.	ng Jikai,Li Yuefeng,Lu Hong	宁夏医科大学学报	al of Ningxia Medical Univ	41	9	926-929	2019	ssn1674-6309.2019.09.014
6139	市市售食品中食源性致病	borne pathogenic bacteria	志强,刘红丽,高洁,张木	ang,Liu Hongli,Gao Jie,Zhan	医学动物防制	medical animal control	35	2	117-120,124	2019	0.7629/yxdwfz201902004
6140	6～2018年沙门氏菌监测	16~2018Annual Salmonell	曾献莹,韦程媛,吕素玲,李	anying,Wei Chengyuan,Lu S	安徽预防医学杂志	i Journal of Preventive Me	25	2	88-92	2019	-

6141	血症的临床特征与 病原菌	S complicated with sepsisA	林锋,肖芙蓉,吴彪,张秀春,	Xiao Furong,Wu Biao,Zhang	中华医院感染学杂志	ournal of Hospital Infectiou	29	23	3544-3547,3572	2019	.11816/cn.ni.2019-184136
6142	康体检后沙门氏菌的检测	ve management measures	马翠平.	Ma Cuiping.	医学信息文摘（连续型电	information abstract (cont	19	99	352	2019	ki.1671-3141.2019.99.211
6143	北京市伤寒、副伤寒沙门	teristics of Salmonella typh	瑛,张新,吕冰,贾蕾,田祎,	Zhang Xin,Lu Bing,Jia Lei,Ti	首都公共卫生	capital public health	13	2	57-61	2019	-
6144	龙华区从业人员沙门菌携	of Salmonella among empl	可小媚,蔡剑辉,李贻汉,李志	Xiaomei,Cai Jianhui,Li Yiha	检验医学与临床	boratory medicine and clini	16	20	2921-2923	2019	sn.1672-9455.2019.20.003
6145	分鸡场禽沙门菌血清学检	is of poultry salmonella in	成倩,孙凤萍,高骏,姚惠娟,	,Sun Fengping,Gao Jun,Yao	上海畜牧兽医通讯	nal Husbandry and Veterina	-	1	56-57	2019	-
6146	道非伤寒沙门菌的感染特	stics and drug susceptibility	罗锡华,卢兰芬,王娟,慕月	hua,Lu Lanfen,Wang Juan,	实用检验医师杂志	of Practical Laboratory Phy	11	1	4-8	2019	sn.1674-7151.2019.01.002
6147	体检沙门氏菌检测结果分	preventive management	仇秀琴.	Qiu Xiuqin.	保健文汇	Health Wenhui	-	4	244-245	2019	sn.1671-5217.2019.04.125
6148	物检验中PCR技术运用价值	progressPCRTechnology a	日正强,颜鲁伟,杨晶,赵慧敏	ang,Yan Luwei,Yang Jing,Zh	甘肃科技纵横	u science and technology a	48	9	81-82,85	2019	sn.1672-6375.2019.09.025
6149	海地区牦牛腹泻病调查与分	nd analysis of yak diarrhea	李娟.	Li Juan.	今日畜牧兽医	ivestock Veterinarian Toda	35	11	23	2019	sn.1673-4092.2019.11.016
6150	39株沙门菌毒力基因检测	virulence gene detection	前进,陈小东,钟叶平,李美	,Chen Xiaodong,Zhong Ye	中国人兽共患病学报	Chinese Journal of Zoonose	35	8	720-725,731	2019	sn.1002-2694.2019.00.133
6151	西省阳泉市食源性多病原	athogen surveillance and t	李荣,郝瑞娥,姚素霞,许学斌	Hao Rui'e,Yao Suxia,Xu Xue	中国卫生检验杂志	ese Journal of Health Inspe	29	10	1153-1156,1160	2019	-
6152	年-2017年细菌性食物中毒	alysis of annual bacterial f	佳明,朱善友,吕永祥,李和	ing,Zhu Shanyou,Lu Yongx	中国卫生检验杂志	ese Journal of Health Inspe	29	8	1007-1010	2019	-
6153	寒沙门菌脉冲场凝胶电泳	drug resistance of Salmone	鹏,黄玉兰,吕虹,黄伟峰,杨	nolia,Lu Hong,Huang Weife	中国食品卫生杂志	hinese Food Hygiene Journ	31	5	441-444	2019	).13590/j.cjfh.2019.05.008
6154	源沙门氏菌分子生物学鉴	serotyping of Salmonella o	刘会杰,马弘财,曾江勇,元	ie,Ma Hongcai,Zeng Jiangy	中国动物检疫	China Animal Quarantine	36	2	63-67	2019	n.1005-944X.2019.02.017
6155	康性沙门菌血清分型及PFG	lmonella in Hanzhong City	志强,刘红丽,高洁,汤进,黄	Liu Hongli,Gao Jie,Tang Jin,	职业与健康	Occupation and health	35	19	2623-2626	2019	-
6156	州市食源性疾病病原学监测	e results of foodborne dise	公强,蒋诗苑,孙丽梅,牛卫东	Jiang Shiyuan,Sun Limei,Ni	现代预防医学	modern preventive medicin	46	4	715-717,759	2019	-
6157	7年龙游县食源性疾病监测	odborne disease surveillan	周慧,姜国良.	Zhou Hui,Jiang Guoliang.	中国卫生检验杂志	ese Journal of Health Inspe	29	13	1619-1622	2019	-
6158	国网售自制即食食品中微	us of homemade ready-to-	何,陈秋霞,张华宁,遇晓杰,闫	uxia,Zhang Huaning,Meet X	卫生研究	health research	48	1	144-146,150	2019	-
6159	市伤寒副伤寒流行病学特	teristics and drug resistanc	K,王晓立,侯君,陈玉凤,栾明	Xiaoli,Hou Jun,Chen Yufeng	实用预防医学	ractical preventive medicin	26	1	103-105	2019	sn.1006-3110.2019.01.029
6160	南省楚雄州食源性疾病流	uses of foodborne disease	琼,白光平,陈强国,李菊,杨	Guangping,Chen Qiangguo,	职业与健康	Occupation and health	35	17	2337-2340,2345	2019	-
6161	川省微生物性食源性疾病	bial foodborne disease ou	陈文,林黎,张誉.	Chen Wen,Lin Li,Zhang Yu.	现代预防医学	modern preventive medicin	46	1	14-16,24	2019	-
6162	检测提高沙门菌检测准确	ns and serological testing	柏柳.	Bailiu.	临床医药文献电子杂志	edical literature electronic	6	25	126-127	2019	-
6163	浙江省食源性疾病监测结	ne disease surveillance res	娟,张政,陈莉莉,孙亮,廖宁	Zheng,Chen Lili,Sun Liang	中国预防医学杂志	se Journal of Preventive Me	20	1	6-10	2019	5/j.1009-6639.2019.01.004
6164	源和人源汤卜逊沙门菌耐	ella Thompson from food	,陈帅,湛志飞,张红,梁进军	i,Zhan Zhifei,Zhang Hong,L	热带医学杂志	journal of tropical medicine	19	10	1221-1224	2019	sn.1672-3619.2019.10.008
6165	便病原菌检验结果的临床	test results of pathogenic	吕幸福.	Lu Xingfu.	临床医药文献电子杂志	edical literature electronic	6	67	44-45	2019	-
6166	样品中四种常见病原的检	f four common pathogens	智宇,朱建国,张孟涛,余磊	Zhu Jianguo,Zhang Mengtao	山东畜牧兽医	mal Husbandry and Veteri	40	1	1-3	2019	sn.1007-1733.2019.01.001
6167	食源性疾病病原菌分布与耐	sistance of foodborne disea	姚燕,陆英,张孟奇.	o Yan,Lu Ying,Zhang Meng	中国卫生检验杂志	ese Journal of Health Inspe	29	23	2900-2902	2019	-
6168	某医院沙门菌的药敏分析	nterotoxin gene detection	向蓉,陈荣忠,黄雪珍.	ng,Chen Rongzhong,Huang	中国抗生素杂志	hinese Journal of Antibiotic	44	2	254-259	2019	sn.1001-8689.2019.02.019
6169	肠炎沙门菌食物中毒事件	Salmonella Enteritidis food	,杨万美,游银行,朱焕新,卢	g Wanmei,travel bank,Zhu	海峡预防医学杂志	s Journal of Preventive Me	25	3	69-70	2019	-
6170	感染性腹泻门诊病例的季	and etiological characteris	佳,林声,庄源,匡小舟,袁政	ng,Zhuang Yuan,Kuang Xiao	中华流行病学杂志	inese Journal of Epidemiolo	40	8	889-894	2019	sn.0254-6450.2019.08.004
6171	病监测中1,4,[5],12:i:-沙门	e1,4,[5],12:i:-Analysis of e	宇,沈赟,周翌婧,吴高林,	hen Yun,Zhou Yijing,Wu Ga	公共卫生与预防医学	Health and Preventive Me	30	4	29-32	2019	sn.1006-2483.2019.04.007
6172	源沙门氏菌的耐药性及相关	esistance genes of Salmone	,徐春志,景书灏,杨峰,陈	unzhi,Jing Shuhao,Yang Fe	养猪	pig raising	-	3	105-107	2019	sn.1002-1957.2019.03.054
6173	沙门氏菌耐药性监测及毒力	monitoring and virulence ge	启发,何守魁,徐景野,石秀	fa,He Shoukui,Xu Jingye,Sh	中国食品学报	urnal of Chinese Food Scien	19	2	216-222	2019	)/j.1009-7848.2019.02.027
6174	培养病原菌的分布及耐药	e analysis of pathogenic ba	梅,张映春,师宏,李杰芬,宋	ng Yingchun,Shi Hong,Li Jie	中国医学创新	Chinese medical innovation	16	24	164-168	2019	sn.1674-4985.2019.24.044
6175	市售食品中食源性致病菌	rne pathogenic bacteria in	,栾旭波,王伟丽,马建敏,史	n Xubo,Wang Weili,Ma Jian	河南预防医学杂志	n Journal of Preventive Me	30	2	159-161	2019	m.1006-8414.2019.02.029
6176	品中食源性致病菌的污染	stics of foodborne pathoge	,姚素霞,王洋,韩吉婷,杨	Suxia,Wang Yang,Han Jitin	中华微生物学和免疫学杂	urnal of Microbiology and In	39	10	731-736	2019	sn.0254-5101.2019.10.002
6177	部分地区禽源沙门氏菌流	nce analysis of poultry-der	青青,赵格,王娟,刘娜,黄秀	,Zhao Ge,Wang Juan,Liu Na	中国动物检疫	China Animal Quarantine	36	8	27-35	2019	n.1005-944X.2019.08.007

6178	年食源性疾病主动监测的	ogical characteristics of an	素霞,张秋香,杨红霞,王洋	ang Qiuxiang,Yang Hongxia	中国人兽共患病学报	Chinese Journal of Zoonose	35	9	852-856	2019	sn.1002-2694.2019.00.094
6179	江省细菌性腹泻症候群病	diarrhea syndrome in Heilo	,任启智,刘玉文,白晶,苑	i,Liu Yuwen,white crystal,	生物信息学	bioinformatics	17	2	116-121	2019	ssn.1672-5565.201903001
6180	鸡病原菌的分离鉴定与	d weak Taihang chicks and	龙,王斌,刘娜,杨俊奇,李博	ng Bin,Liu Na,Yang Junqi,L	龙江畜牧兽医（下半月）	dry and Veterinary Medicin	-	8	107-111,182	2019	nki.hljxmsy.2018.09.0387
6181	验在季节性腹泻检验中	e stool examination in dete	迟祥.	Chi Xiang.	中国医疗器械信息	na medical device informat	25	8	22-23	2019	sn.1006-6586.2019.08.011
6182	乱弧菌和沙门菌等病原	teria such as Vibrio choler	,王莹,田园,纪雪,孙洋,初	Ying,rural,Ji Xue,Sun Yang	中国人兽共患病学报	Chinese Journal of Zoonose	35	3	212-215,222	2019	sn.1002-2694.2019.00.008
6183	分型综合分析在一起甲型	alysis of pathogen mole	;闫梅英;王志刚;阳波;吴	ing;Wang Zhigang;Yang	中国公共卫生	Chinese public health	35	3	341-344	2019	-
6184	应用PCR法与传统细菌培	nalysis of detection metho	刘莉莉.	Liu Lili.	医学食疗与健康	dical Diet Therapy and Hea	18	24	174-175	2020	-
6185	菌污染引起的食源性疾病	a foodborne illness caused	王雨荷.	Wang Yuhe.	山西医药杂志	Shanxi Medical Journal	49	24	3400-3402	2020	sn.0253-9926.2020.24.010
6186	伤寒沙门菌耐药与分子	lecular typing of Salmonell	霞,张秋香,杨红霞,王洋,韩	xiang,Yang Hongxia,Wang	华微生物学和免疫学杂	rnal of Microbiology and I	40	12	903-908	2020	n112309-20200806-00392
6187	门氏菌肠炎的临床分析	tance monitoring of Salmo	保明,田根全,凌丽,关丽	ing,Tian Genquan,Ling Li,G	中国处方药	Chinese prescription drugs	18	12	165-166	2020	n.1671-945X.2020.12.084
6188	-2018年三起甲型副伤寒	eptibility test of strains of	曾竣,陆宝,顾桂敏,黄君,王	Lu Bao,Gu Guimin,Huang J	中国热带医学	Chinese tropical medicine	20	12	1134-1137,1148	2020	cnki.46-1064/r.2020.12.03
6189	无锡市肠炎沙门菌药物敏	d molecular typing of Salm	沙丹,李娟,赵晓菲,郑东宇	Juan,Zhao Xiaofei,Zheng D	疾病监测	disease surveillance	35	12	1146-1151	2020	sn.1003-9961.2020.12.018
6190	沙门菌致食物中毒的病	se of food poisoning cause	王艳玲.	Wang Yanling.	中国城乡企业卫生	ygiene in urban and rural a	35	12	12-14	2020	/j.1003-5052.2020.12.005
6191	印第安纳沙门菌的耐药性	esistant genes of Salmone	雨倩,王存敏,詹泽强,高	Wang Cunmin,Zhan Zeqiang	中国动物传染病学报	ournal of Animal Infectious	28	6	96-101	2020	-
6192	门氏菌引起的食物中毒事	d poisoning incident cause	鹏程,施爱萍,钱惠芬,徐兰,	ng,Shi Aiping,Qian Huifen,	医学动物防制	medical animal control	36	12	1171-1173	2020	0.7629/yxdwfz202012014
6193	源性腹泻患者沙门氏菌	and drug resistance in pat	思露,周厚德,刘道峰,刘成	ou Houde,Liu Daofeng,Liu	现代预防医学	modern preventive medicin	47	24	4452-4455	2020	-
6194	售食品中食源性致病菌	orne pathogenic bacteria i	刘于飞,张维蔚,李燕,周琴,	,Zhang Weiwei,Li Yan,Zhou	食品安全质量检测学报	f Food Safety and Quality I	11	24	9396-9401	2020	-
6195	食品中沙门氏菌的污染	d serotype distribution of S	洁,赵薇,王艳秋,李可维,	o Wei,Wang Yanqiu,Li Kew	食品安全质量检测学报	f Food Safety and Quality I	11	24	9377-9382	2020	-
6196	粪便标本病原微生物检	rug resistance analysis of f	董莉.	Dong Li.	河南医学研究	Henan Medical Research	29	36	6863-6865	2020	n.1004-437X.2020.36.057
6197	疾控中心细菌性食物中毒	of bacterial food poisoning	邓秀.	Deng Xiu.	家有孕宝	re is a pregnant baby at h	2	24	160	2020	-
6198	副伤寒沙门菌脉冲场凝胶	ular typing and drug resist	云屏,邹颜秋硕,任翔,田云	Zou Yanqiushuo,Ren Xiang,	食品安全质量检测学报	f Food Safety and Quality I	11	24	9434-9439	2020	-
6199	售食品中食源性致病菌污	of foodborne pathogenic b	明敏,刘腾,杨欢欢,王露	,Liu Teng,Yang Huanhuan,	医学动物防制	medical animal control	36	12	1142-1145	2020	0.7629/yxdwfz202012006
6200	福建沙门氏菌和弯曲杆菌	Salmonella and Campyloba	罗怡,施杏芬,胡骥,陈怡飞,	Yi,Shi Xingfen,Hu Ji,Chen Yi	浙江农业学报	urnal of Zhejiang Agricultu	32	12	2218-2225	2020	ssn.1004-1524.2020.12.13
6201	因组序列中外排泵耐药基	p resistance genes in the	沈赟,秦思,侯海燕.	Shen Yun,Qin Si,Hou Haiyan	食品安全质量检测学报	f Food Safety and Quality I	11	24	9361-9365	2020	-
6202	族地区特色食品安全风险	control of specialty food i	张静,施向东,庞洁,秦剑秋	g,Shi Xiangdong,Pang Jie,Q	中国卫生检验杂志	ese Journal of Health Inspe	30	24	3013-3016	2020	-
6203	血清型、药敏分析及脉冲	ld gel electrophoresis mol	林,高艳,刘丹,焦洋,段杉,张	,Liu Dan,Jiao Yang,Duan Sh	食品安全质量检测学报	f Food Safety and Quality I	11	24	9415-9420	2020	-
6204	门氏菌的分离鉴定与生物	ological Characteristics Ana	,杜冬冬,王国红,黄新,张	ng,Wang Guohong,Huang	新疆农垦科技	Agricultural Reclamation Te	43	12	38-40	2020	n.1001-361X.2020.12.024
6205	儿童中的感染流行特征及	drug resistance analysis of	勇,李泓,冯微宏,管红霞,	Hong,Feng Weihong,Guan	中国卫生检验杂志	ese Journal of Health Inspe	30	24	2981-2983,2987	2020	-
6206	规检验对季节性腹泻的检	onal diarrhea by routine st	陈宗斌.	Chen Zongbin.	健康必读	Health must read	-	36	198	2020	-
6207	鸡及其商品鸡中沙门氏	ent breeder chickens and c	宏庆,刘莉,姚志兰,王永	ing,Liu Li,Yao Zhilan,Wang	微生物学通报	Bulletin of Microbiology	47	12	4105-4112	2020	/j.microbiol.china.200631
6208	9年肠炎沙门菌分子流行	ecular epidemiological char	晶,仲伟麒,李鑫,郑晓华,	hong Weiqi,Li Xin,Zheng Xia	中国公共卫生管理	nese public health managen	36	6	846-848	2020	cnki.23-1318.2020.06.025
6209	8年餐饮食品食源性致病	monitoring results of food	崔杰,孟灿,陈志飞,李卫东	Meng Can,Chen Zhifei,Li W	安徽预防医学杂志	i Journal of Preventive Me	26	6	411-414	2020	7/j.cnki.ahyf.2020.06.001
6210	7-2018年感染性腹泻病原	veillance analysis of infecti	张建群,罗学辉,袁士杰.	Jianqun,Luo Xuehui,Yuan	中国公共卫生管理	nese public health managen	36	6	868-871	2020	cnki.23-1318.2020.06.032
6211	菌性腹泻的病原微生物检	icroorganism testing for ba	王娟.	Wang Juan.	中国医药指南	Chinese Medicine Guide	18	35	47-48	2020	-
6212	沙门菌食物中毒1例调查	eritidis food poisoning1Cas	梦颖,姚婧,李廷荣,黄莉	ing,Yao Jing,Li Tingrong,Hu	现代医药卫生	modern medicine and healt	36	23	3886-3888	2020	sn.1009-5519.2020.23.053
6213	者粪便常规临床检验结果	clinical examination result	何梦婷,吴勋.	He Mengting,Wu Xun.	健康必读	Health must read	-	35	2	2020	-
6214	及肉制品食源性致病菌监	dborne pathogenic bacter	刘思洁,赵薇,杨修军,石奔	,Zhao Wei,Yang Xiujun,Shi	食品安全质量检测学报	f Food Safety and Quality I	11	23	9033-9038	2020	-

6215	市食品中食源性致病菌监	lts of foodborne pathogen	,王恒辉,高雯洁,陈黎霞,李	Henghui,Gao Wenjie,Cher	中国卫生检验杂志	ese Journal of Health Inspe	30	23	2921-2924	2020	-
6216	单核细胞增生李斯特菌和	steria monocytogenes and	范一灵,宋明辉,秦峰,刘浩	g,Song Minghui,Qin Feng,L	食品安全质量检测学报	f Food Safety and Quality I	11	23	9016-9020	2020	-
6217	食品中沙门氏菌快速检测	ection method for Salmone	王雨,石晓丽,杨桂娥.	Vang Li,Shi Xiaoli,Yang Gui'	食品研究与开发	od research and developme	41	23	179-184	2020	sn.1005-6521.2020.23.030
6218	感染性腹泻病原学及其	hea in children in Zunyi are	庆,李茂花,甘旭,蹇亚丹,杨	Maohua,Gan Xu,Jian Yada	中华医院感染学杂志	ournal of Hospital Infectiou	30	23	3668-3672	2020	.11816/cn.ni.2020-200819
6219	地区水禽主要细菌性疾病	or bacterial diseases of wat	董永毅,卢凤英,吴坤,孙华伟	ngyi,Lu Fengying,Wu Kun,Su	江苏农业科学	iangsu Agricultural Science	48	23	172-174	2020	sn.1002-1302.2020.23.034
6220	细菌性食物中毒的微生物	cal test results of cases of b	张步芳.	Zhang Bufang.	中国保健食品	Chinese health food	-	12	83	2020	-
6221	2018-2019年食源性疾病监	9Annual Foodborne Diseas	赵娅丽.	Zhao Yali.	养生保健指南	Health and Wellness Guide	-	48	278	2020	-
6222	性腹泻病原微生物检验结	ts of pathogenic microorga	张蕾蕾.	Zhang Leilei.	中国保健营养	Chinese health nutrition	30	34	35	2020	-
6223	查应用于秋冬季腹泻患儿	n in the diagnosis of childre	刘云霞.	Liu Yunxia.	科学养生	Scientific health care	-	12	167	2020	-
6224	性腹泻452例临床和病原学	452Case clinical and etiolo	伊凯.	ikei.	家庭医药.就医选药	he.Medical treatment and d	-	12	53	2020	-
6225	滋病患者血培养病原菌检	ution of pathogenic bacteri	蒲大朋,熊倩妤,杨兴林,杨俊	,Xiong Qianyu,Yang Xinglin	基层医学论坛	Primary Medicine Forum	24	34	4967-4969	2020	5/j.1672-1721.2020.34.043
6226	腹泻检验中便常规检验意	f routine stool examination	乔丽娜.	Jolina.	科学养生	Scientific health care	-	12	105	2020	-
6227	市I4,[5],12 : i : -沙门菌流行	-Salmonella epidemic char	水飞,彭淑萍,许铭清,廖国	Peng Shuping,Xu Mingqing	中国食品卫生杂志	hinese Food Hygiene Journ	32	6	681-685	2020	).13590/j.cjfh.2020.06.017
6228	沙门菌污染三明治引起食	aused by Salmonella Enteri	加贝,王虹玲,陈艳,虞艳,刘	ang Hongling,Chen Yan,Yu	中国食品卫生杂志	hinese Food Hygiene Journ	32	6	708-712	2020	).13590/j.cjfh.2020.06.022
6229	产过程微生物污染状况监	raceability analysis of prep	霞,戚浩彧,张秀丽,刘岩,王	Haoyu,Zhang Xiuli,Liu Yan,\	中国食品卫生杂志	hinese Food Hygiene Journ	32	6	686-691	2020	).13590/j.cjfh.2020.06.018
6230	新区食源性疾病主动监测	racteristics of active survei	任亚萍,沈惠平,瞿凤,柏品清	g,Shen Huiping,Qu Feng,Ba	中国食品卫生杂志	hinese Food Hygiene Journ	32	6	676-680	2020	).13590/j.cjfh.2020.06.016
6231	类市售食品中微生物及其	ts of microorganisms and t	陈加贝,黄丽君,朱芳仪,陈艳	bei,Huang Lijun,Zhu Fangyi	中国食品卫生杂志	hinese Food Hygiene Journ	32	6	670-675	2020	).13590/j.cjfh.2020.06.015
6232	腹泻患儿细菌培养结果及干	intervention strategies in	孟文芳,章承红,王晶晶.	fang,Zhang Chenghong,Wa	现代消化及介入诊疗	and interventional diagno	25	11	1512-1515	2020	sn.1672-2159.2020.11.023
6233	菌血症病例中分离的乌撒	au isolated from the first c	杨晓蓉,雷高鹏,封正娟,沈	rong,Lei Gaopeng,Feng Zhe	中国人兽共患病学报	Chinese Journal of Zoonose	36	11	913-917	2020	sn.1002-2694.2020.00.147
6234	源性致病菌监测结果分析	ng results of foodborne pa	魏建萍.	Wei Jianping.	抗感染药学	Anti-infective Pharmacy	17	11	1653-1655	2020	sn.1672-7878.2020.11-030
6235	食源性沙门菌的分布及耐药	nce analysis of foodborne	晓霞,陈雅莉,孔花娟,柏莹	,Chen Yali,Kong Huajuan,B	检验医学	laboratory medicine	35	11	1161-1164	2020	sn.1673-8640.2020.11.018
6236	省食品中疑似鼠伤寒沙门	ion of Suspected Salmonell	黄玉兰,黄伟峰,张林,刘丽	a,Huang Weifeng,Zhang Lin	预防医学情报杂志	of Preventive Medicine Info	36	11	1418-1423	2020	-
6237	寒沙门氏菌引起的食物中	poisoning incident caused	陈伟,秦友燕,韩丹丹,黄辉	,Qin Youyan,Han Dandan,H	广西医科大学学报	al of Guangxi Medical Univ	37	11	2074-2078	2020	nki.45-1211/r.2020.11.024
6238	宁省食品中食源性致病微	of foodborne pathogenic m	竹,刘海霞,李雪,耿英芝,	u Haixia,Li Xue,Geng Yingzh	食品安全质量检测学报	f Food Safety and Quality I	11	22	8566-8571	2020	-
6239	例南宁市食源性疾病特征	acteristics of foodborne di	,施向东,龙兮,马玲玲,秦	Xiangdong,Long Xi,Ma Lingl	现代预防医学	modern preventive medicin	47	22	4072-4075,4079	2020	-
6240	起的肠炎沙门氏菌食物中	ning incident caused by Sal	程文娟,薛琳,周坤,吴景文	njuan,Xue Lin,Zhou Kun,W	实用预防医学	ractical preventive medicin	27	11	1379-1380	2020	sn.1006-3110.2020.11.027
6241	海螺蛸饮片的微生物污染	mination status of sea octo	妮娜,白雯静,张彩霞,宋玉	Bai Wenjing,Zhang Caixia,S	中国卫生检验杂志	ese Journal of Health Inspe	30	22	2777-2779	2020	-
6242	年间血培养分离细菌的分布	stance spectrum analysis o	双珠,马众仙,龙湖波,杨宝	ongxian,Longhu wave,Yang	中国抗生素杂志	hinese Journal of Antibiotic	45	11	1153-1160	2020	sn.1001-8689.2020.11.010
6243	及肉制品食源性致病菌检	pathogenic bacteria in mea	邹春萍,杨修军,石奔,孙景	nping,Yang Xiujun,Shi Ben,	食品安全质量检测学报	f Food Safety and Quality I	11	22	8551-8555	2020	-
6244	生急性腹泻的常见病原微	athogenic microorganisms	李中春,李俊梅.	Li Zhongchun,Li Junmei.	养生保健指南	Health and Wellness Guide	-	47	251	2020	-
6245	鸡源沙门菌的流行性及耐	sistance of Salmonella origi	,张纯萍,李伟杰,赵琪,徐	ng Chunping,Li Weijie,Zhao	中国兽医杂志	Chinese Veterinary Journal	56	11	89-92	2020	-
6246	菌和沙门氏菌的分离鉴定	e analysis of Escherichia co	薛瑞林,李守湖,柳纪省,唐	e Ruilin,Li Shouhu,Liuji Pro	中国兽药杂志	ese Journal of Veterinary D	54	11	14-21	2020	SN.1002-1280.2020.11.03
6247	门菌属细菌220株的分布特	.220Distribution characteri	中举,田磊,闫少珍,孙自镛	gju,Tian Lei,Yan Shaozhen,S	中国感染与化疗杂志	urnal of Infection and Cher	20	6	659-664	2020	3/j.1009-7708.2020.06.013
6248	门菌鉴定、分子分型及耐药	drug resistance analysis of	王瑶,张焕容,罗薇.	g Yao,Zhang Huanrong,Luo	动物医学进展	vances in Veterinary Medic	41	11	53-57	2020	sn.1007-5038.2020.11.010
6249	肠沙门菌血清型鉴定与毒力	nd drug resistance detecti	楠,呼延含蓉,李男,胡博,田	uyan Hanrong,Li Nan,Hu Bo	动物医学进展	vances in Veterinary Medic	41	11	58-61	2020	sn.1007-5038.2020.11.011
6250	性食物中毒的微生物学检验	nination and analysis of bac	李荔婷.	Li Liting.	饮食保健	Diet and health care	-	47	243	2020	-
6251	9年肠道门诊腹泻患者病	etection results in patients	柳秋霞,强鑫华.	Liu Qiuxia,Qiang Xinhua.	中国乡村医药	Chinese rural medicine	27	21	56-57	2020	sn.1006-5180.2020.21.037

6252	疗机构污水消毒处理的卫	disinfection treatment in m	英,郑剑,徐明,马晓,孙斌,高	eng Jian,Xu Ming,Ma Xiao,	中国消毒学杂志	hinese Journal of Disinfecti	37	11	848-850,854	2020	sn.1001-7658.2020.11.015
6253	生菜致病微生物污染现状	ogenic microbial contamina	,王珍,何天,刘思怡,肖英平	hen,What day,Liu Siyi,Xiao	浙江农业科学	hejiang Agricultural Science	61	11	2333-2335	2020	.issn.0528-9017.20201144
6254	鸡群中芙蓉沙门氏菌的分	of Salmonella hibiscus from	张开臣,崔璐璐,孔德军.	ai Chen,Cui Lulu,Kong Deju	家禽科学	poultry science	-	11	50-52	2020	sn.1673-1085.2020.11.016
6255	饮业现榨果汁微生物污染	tus of freshly squeezed jui	袁瑞,付云,宋臻鹏,沈建勇.	u Yun,Song Zhenpeng,Shen	中国卫生检验杂志	ese Journal of Health Inspe	30	21	2659-2662	2020	-
6256	上海市浦东新区食源性致	of foodborne pathogenic ba	锦,桂燕华,赵冰,陆岚,丁明	ui Yanhua,Zhao Bing,Lu Lan	职业与健康	Occupation and health	36	21	2936-2940	2020	-
6257	食源性沙门菌血清分布及	distribution and drug susce	诸佳辉,罗学辉,黄邵军.	ahui,Luo Xuehui,Huang Sha	中国乡村医药	Chinese rural medicine	27	20	49-50	2020	sn.1006-5180.2020.20.032
6258	门菌血清型、药物敏感性	d virulence genes of clinical	东晓,王若南,成军,孙长贵,郭	ng Ruonan,into an army,Su	中华传染病杂志	se Journal of Infectious Dis	38	10	646-650	2020	n311365-20191220-00420
6259	直腹泻患者分离的非伤寒沙	non-typhoid Salmonella iso	晓,尤爱国,张白帆,赵嘉咏,	guo,Zhang Baifan,Zhao Jiay	中华微生物学和免疫学杂志	urnal of Microbiology and In	40	10	787-791	2020	n112309-20200222-00070
6260	沙门菌食物中毒90例临床	ning in children90Analysis	李珊珊,齐颖,宋萍,付丹.	nshan,Qi Ying,Song Ping,Fu	医学检验与临床	edical Laboratory and Clinic	31	10	31-34	2020	sn.1673-5013.2020.10.008
6261	余姚市河水环境中沙门菌	tance of Salmonella in the r	黄邵军,罗学辉,诸佳辉.	g Shaojun,Luo Xuehui,Zhu J	疾病监测	disease surveillance	35	10	946-952	2020	sn.1003-9961.2020.10.016
6262	酸奶质量安全的污染因素	llution factors in yogurt qu	龙,李继荣,张唐伟,吴雪莲	irong,Zhang Tangwei,Wu X	华南农业大学学报	f South China Agricultural U	41	5	115-123	2020	ssn.1001-411X.202001013
6263	厦市哨点医院食源性疾病	urveillance of foodborne d	时秀,杨康,高景枝,李玉芳	ang Kang,Gao Jingzhi,Li Yu	公共卫生与预防医学	Health and Preventive Me	31	5	57-60	2020	sn.1006-2483.2020.05.015
6264	毒的病原学情况和微生物	nd microbial test results of	薛喜梅,王永生.	Xue Ximei,Wang Yongsheng	口岸卫生控制	Port health control	25	5	43-45	2020	sn.1008-5777.2020.05.011
6265	龟暴露致幼儿感染波摩那	ona infection in young chil	崔琪奇,叶楚楚,桂燕华,苏	ui Qiqi,Ye Chuchu,Gui Yanh	疾病监测	disease surveillance	35	10	957-960	2020	sn.1003-9961.2020.10.018
6266	食品中沙门氏菌的测定	rmination of Salmonella in	,宋勇强,仝伟建,杨晓楠,付	ngqiang,Tong Weijian,Yang	甘肃畜牧兽医	al Husbandry and Veterina	50	10	60-63,70	2020	n.1006-799X.2020.10.020
6267	源沙门菌的分子分型及耐药	sistance research on Salm	艳,张小荣,曹永忠,吴艳涛	ng Xiaorong,Cao Yongzhong	中国病原生物学杂志	ese Journal of Pathogen Bio	15	10	1131-1136,1143	2020	10.13350/j.cjpb.201003
6268	血流感染病原体分布及耐药	rug resistance of AIDS bloo	志珊,林永年,陈晓婷,张汉	,Lin Yongnian,Chen Xiaoting	中国艾滋病性病	AIDS and STDs in China	26	10	1057-1060	2020	419/j.cnki.aids.2020.10.07
6269	—2019年食物中毒致病菌	is of the annual detection o	张晓琦,张育英,李燕.	ng Xiaoqi,Zhang Yuying,Li Y	河南医学研究	Henan Medical Research	29	30	5641-5643	2020	n.1004-437X.2020.30.028
6270	腹泻患儿菌株的分布和耐药	nalysis of bacterial strains i	袁阳.	Yuan Yang.	实用中西医结合临床	ation of Traditional Chinese	20	10	110-111,157	2020	sn.1671-4040.2020.10.056
6271	校周边即食米面食品微生物	ready-to-eat rice and nood	瑞,黄铮,宋臻鹏,朱幸,沈建	heng,Song Zhenpeng,Zhu X	中国学校卫生	Chinese school health	41	10	1576-1578	2020	ki.1000-9817.2020.10.037
6272	术后败血症与骨髓抑制相	ation between postoperativ	刘海英,陈峰,吴宁.	Haiying,Chen Feng,Wu Nin	介入放射学杂志	rnal of Interventional Radio	29	10	1033-1035	2020	n.1008-794X.2020.10.016
6273	儿腹泻病原微生物检验结果	genic microorganisms in in	曹忠海.	Cao Zhonghai.	中国保健营养	Chinese health nutrition	30	30	375-376	2020	-
6274	年哨点医院食源性疾病主	foodborne disease surveilla	王子友,周海慧,郑涛涛.	Ziyou,Zhou Haihui,Zheng T	中国卫生检验杂志	ese Journal of Health Inspe	30	20	2546-2548,2551	2020	-
6275	源沙门氏杆菌的分离鉴定	ty analysis of Salmonella or	刘雪姣,李海花,王倩,盛清凯	o,Li Haihua,Wang Qian,She	黑龙江畜牧兽医（下半月）	dry and Veterinary Medicin	-	10	89-92,160	2020	cnki.hljxmsy.2019.11.0093
6276	性疾病流行病学分析和基	foodborne diseases in Nann	张静,马玲玲.	Zhang Jing,Ma Lingling.	职业与健康	Occupation and health	36	20	2793-2796	2020	-
6277	蛋糕引起的肠炎沙门氏菌	ella Enteritidis food poison	刘永兴.	Liu Yongxing.	东方药膳	Oriental medicinal food	-	19	96	2020	-
6278	源沙门氏菌的分离鉴定及耐	istance study of Salmonell	涛,吴冬梅,刘铭英,杨先富	Dongmei,Liu Mingying,Yan	安徽农业科学	Anhui Agricultural Sciences	48	19	109-111	2020	sn.0517-6611.2020.19.028
6279	检测方法在预防性体检便检	ds for Salmonella in preven	韦瑞吉.	Verigi.	特别健康	Very healthy	-	29	98-99	2020	-
6280	金黄色葡萄球菌混合引起的	ning caused by a mixture of	骆业巧,王进,司波.	Luo Yeqiao,Wang Jin,Shiba	食品安全质量检测学报	f Food Safety and Quality In	11	19	6947-6951	2020	-
6281	地区沙门菌血清型分布特征	characteristics of Salmone	何迎峰,管红霞,沙丹,严昕宇	feng,Guan Hongxia,satan,Y	中国卫生检验杂志	ese Journal of Health Inspe	30	19	2330-2332	2020	-
6282	省不同来源沙门氏菌耐药性	Salmonella from different	张铭琰,耿英芝,于淼,张眉眉	an,Geng Yingzhi,Yu Miao,Zh	食品安全质量检测学报	f Food Safety and Quality In	11	19	7142-7146	2020	-
6283	发的肠炎沙门氏菌食物中	hella Enteritidis food poison	黄洁.	Huang Jie.	医学信息文摘（连续型电	information abstract (cont	20	81	278-279	2020	sn.1671-3141.2020.81.136
6284	泻的临床特征、病原菌分	of pathogenic bacteria and	黄雪霞.	Huang Xuexia.	智慧健康	Smart health	6	28	53-54,57	2020	ki.2096-1219.2020.28.022
6285	透析合并伤寒沙门氏菌感染	g maintenance hemodialys	何俊琪,张嘉欣.	He Junqi,Zhang Jiaxin.	特别健康	Very healthy	-	28	210-211	2020	-
6286	年陕西省发病猪群主要疫病	lysis of Major Diseases in P	仇薪鑫,孟姣,张振仓.	nxin,Meng Jiao,Zhang Zhen	江苏农业科学	iangsu Agricultural Science	48	19	172-176	2020	sn.1002-1302.2020.19.038
6287	区市售牛羊肉食源性致病菌	ia contamination of comme	李鹏.	Li Peng.	中国草食动物科学	Chinese herbivore science	40	5	53-55	2020	sn.2095-3887.2020.05.011
6288	市售雪花酥的品质评价	n of commercially available	古明亮.	Gu Mingliang.	粮食加工	Grain processing	45	5	74-78	2020	-

6289	新区市售生禽畜肉中6种食	dong New District, Shangh	慧,胡卉,邬天凤,王筱,沈	Hu Hui,Wu Tianfeng,Wang	职业与健康	Occupation and health	36	19	2645-2648,2653	2020	-
6290	生食肉制品及相关产品中	ion in raw meat products a	陈祎清,宋昌彦,袁波,杨君,	ing,Song Changyan,Yuan B	中国食品卫生杂志	hinese Food Hygiene Journ	32	5	530-535	2020	).13590/j.cjfh.2020.05.011
6291	者粪便中紧密连接蛋白水	of patients with infectious	何方,杜海涛,赵敏,曹静.	re,Du Haitao,Zhao Min,Cao	中国医师杂志	Chinese Physician Journal	22	9	1422-1424	2020	n431274-20190619-00729
6292	沙门菌感染的临床特点及	sistance research of intesti	黄雪霞,黄振鹏,曾惠华.	uexia,Huang Zhenpeng,Zen	临床检验杂志（电子版）	f Clinical Testing (electroni	9	3	235-236	2020	-
6293	18年江西省临床分离非伤	rug resistance of clinically	,周厚德,刘洋,刘道峰,胡凯	ude,Liu Yang,Liu Daofeng,H	中国食品卫生杂志	hinese Food Hygiene Journ	32	5	493-498	2020	).13590/j.cjfh.2020.05.004
6294	中非伤寒沙门菌定量风险	assessment of non-typhoi	贾华云,董庆利,赵格,白莉	uayun,Dong Qingli,Zhao Ge	中国食品卫生杂志	hinese Food Hygiene Journ	32	5	576-581	2020	).13590/j.cjfh.2020.05.020
6295	R技术在临床微生物检测中	application value of techno	蒋雪纷.	Jiang Xuefen.	临床检验杂志（电子版）	f Clinical Testing (electroni	9	3	344-345	2020	-
6296	起伊桑吉沙门菌引起的食	outbreak caused by Salmo	群,胡津津,孙成玺,苗超,李	u Jinjin,Sun Chengxi,Miao	疾病监测	disease surveillance	35	9	866-870	2020	sn.1003-9961.2020.09.021
6297	新乡市食品安全风险监测	d safety risk monitoring res	红霞,芦天成,史晓娟,王伟	,Lu Tiancheng,Shi Xiaojuan	河南预防医学杂志	n Journal of Preventive Me	31	9	710-712	2020	m.1006-8414.2020.09.022
6298	染急性腹泻患儿预后及危	for children with acute dia	方,姜勇超,王丽,涂丹娜,陈	ang Yongchao,Wang Li,Tu D	热带医学杂志	journal of tropical medicine	20	9	1236-1239	2020	sn.1672-3619.2020.09.028
6299	中沙门氏菌全基因组分析	ar traceability of Salmonel	晗,严礼,郭焜鹏,张海韵,王	Yanli,Guo Kunpeng,Zhang H	食品与机械	Food and Machinery	36	9	55-62,169	2020	sn.1003-5788.2020.09.009
6300	天津市津南区食源性疾病	rne disease surveillance re	小雅娜,李龙玉,李胜强,郑梅	,Li Longyu,Li Shengqiang,Zh	中国城乡企业卫生	ygiene in urban and rural ar	35	9	31-33	2020	5/j.1003-5052.2020.09.011
6301	菌感染患儿160例临床特征	hengdu160Clinical charact	小蓉,张玉霞,黄伟峰,商丽	Zhang Yuxia,Huang Weifen	预防医学情报杂志	of Preventive Medicine Info	36	9	1119-1123,1130	2020	-
6302	痢疾患者黏液脓血便病原	ction in patients with muc	张雪艳.	Zhang Xueyan.	内蒙古医学杂志	ner Mongolia Medical Jour	52	9	1031-1032	2020	i.nmgyxzz.2020.52.09.003
6303	劳德尔卑沙门菌的耐药特	enetic diversity of Salmonel	建辉,邱玉锋,杨劲松,柯自	Jianhui,Qiu Yufeng,Yang Ji	中国病原生物学杂志	ese Journal of Pathogen Bi	15	9	1046-1052	2020	10.13350/j.cjpb.200911
6304	本中病原菌的分布特征及	ples of children with bacte	苏航.	Su Hang.	抗感染药学	Anti-infective Pharmacy	17	9	1297-1299	2020	sn.1672.7878.2020.09-017
6305	沙门氏菌的分离、鉴定及	n and pathogenicity study o	,李华德,付雪,张兰,翟亚	uade,Fu Xue,Zhang Lan,Zha	川大学学报（自然科学版	huan University (Natural Sc	57	5	1022-1028	2020	sn.0490-6756.2020.05.030
6306	测在儿童细菌性腹泻中的	thogenic microorganism de	王华伟,郑敏红,王淑虹.	awei,Zheng Minhong,Wang	医学信息文摘（连续型电	information abstract (cont	20	78	227-228	2020	sn.1671-3141.2020.78.097
6307	制品中食源性致病菌的污	odborne pathogenic bacter	,朱静静,金富红,杨海鑫,黄	ingjing,Jin Fuhong,Yang Ha	食品安全质量检测学报	f Food Safety and Quality I	11	18	6552-6556	2020	-
6308	封市鼓楼区食源性疾病监	isease surveillance results	赵云清,殷锋科.	Zhao Yunqing,Yin Fengke.	实用预防医学	ractical preventive medicin	27	9	1135-1137	2020	sn.1006-3110.2020.09.032
6309	地区食源性人兽共患病原	tus of foodborne zoonotic	庆,李啸雪,卢贤忠,徐学	i Xiaoxue,Lu Xianzhong,Xu	中国兽医杂志	Chinese Veterinary Journal	56	9	63-67	2020	-
6310	性食物中毒的微生物学检	ination and analysis of bac	邢军华,文蔚,尹明.	g Junhua,Wen Wei,Yin Mi	西医结合心血管病电子杂	Diseases Integrated Traditi	8	27	95103	2020	-
6311	年重庆市其他感染性腹泻	mic characteristics of other	寒,李勤,李柏松,易娟,熊	an,Li Qin,Li Baisong,Yi Juan	热带病与寄生虫学	pical Diseases and Parasito	18	3	151-154,187	2020	sn.1672-2302.2020.03.005
6312	天津市津南区食源性疾病	of foodborne disease path	孙雅娜,刘坚龄.	Sun Yana,Liu Jianling.	职业与健康	Occupation and health	36	18	2486-2489	2020	-
6313	生鲜湿面中腐败菌的分离	oilage bacteria in commerc	李刚凤,夏欣欣,马莉敏,吴	angfeng,Xia Xinxin,Ma Limi	中国酿造	China Brewing	39	9	148-151	2020	sn.0254-5071.2020.09.028
6314	性食物中毒的微生物学检	ination and analysis of bac	韦瑾平.	Wei Jinping.	东方药膳	Oriental medicinal food	-	17	17-18	2020	-
6315	-2018年食品风险监测致病	l food risk monitoring path	邹翔.	Zou Xiang.	中国卫生检验杂志	ese Journal of Health Inspe	30	17	2164-2166	2020	-
6316	8年度腹泻症候群病例监	of annual surveillance of di	陈清.	Chen Qing.	中国卫生检验杂志	ese Journal of Health Inspe	30	17	2173-2174,封3	2020	-
6317	市沙滩浴场水体病原微生	roorganisms in water bodi	军,高霞,杜荐如,李淑华,叶	,Gao Xia,Du Jianru,Li Shuhu	中国初级卫生保健	primary health care in china	34	9	102-104	2020	.1001-568X.2020.09.0031
6318	沙门菌的分离鉴定及其耐	cks in Xuzhou and surroun	任士飞,张林吉,迟兰,房超	ifei,Zhang Linji,Chi Lan,Fan	畜牧与兽医	Husbandry and Veterinary M	52	9	99-104	2020	-
6319	性腹泻病原微生物检验结	ts of pathogenic microorga	于金英,栗秀娥.	Yu Jinying,Li Xiu'e.	糖尿病天地	Diabetes World	17	9	161	2020	-
6320	松类食品制作过程中非伤	in the preparation process	峰,姚云英,夏伟,胡虹雨,汤	,Yao Yunying,Xia Wei,Hu H	养生保健指南	Health and Wellness Guide	-	36	289-290	2020	-
6321	沙门氏菌性肠炎和乳糖不	salmonella enteritis and la	丽霞,陈卓杰,邓筹芬,江洪	en Zhuojie,Deng Choufen,J	中国实用医药	Chinese Practical Medicine	15	24	57-59	2020	nki.11-5547/r.2020.24.023
6322	染患者血标本的细菌培养	of antibacterial drugs in blo	徐雪勤.	Xu Xueqin.	抗感染药学	Anti-infective Pharmacy	17	8	1165-1167	2020	sn.1672-7878.2020.08-023
6323	临床血流感染病例检出特	Province for the first time	,胡辛兰,陈建辉,杨劲松,黄	lan,Chen Jianhui,Yang Jins	中国人兽共患病学报	Chinese Journal of Zoonose	36	8	643-645,684	2020	sn.1002-2694.2020.00.096
6324	8年上海市奉贤区腹泻病	istics of diarrheal diseases	清,胡晓丹,陈爽,李星成,王	dan,Chen Shuang,Li Xingch	热带医学杂志	journal of tropical medicine	20	8	1106-1109	2020	sn.1672-3619.2020.08.029
6325	鼠伤寒沙门菌的流行病学	racteristics of Salmonella t	郭惠,童晶,吴畏畏,刘庭君	Tong Jing,Wu Weiwei,Liu T	中国人兽共患病学报	Chinese Journal of Zoonose	36	8	636-642	2020	sn.1002-2694.2020.00.128

6326	浙江省金华市伤寒副伤寒	ristics of typhoid and parat	龙志峰,金学怡,金祝平,吴	ifeng,Jin Xueyi,Jin Zhuping,	疾病监测	disease surveillance	35	8	757-761	2020	sn.1003-9961.2020.08.017
6327	区藏猪源沙门菌分离鉴定	enotype of Salmonella origi	燕娟,焦新安,潘志明,索朗斯	,Jiao Xinan,Pan Zhiming,so	中国兽医学报	l of Chinese Veterinary Me	40	8	1506-1510	2020	nki.1005-4545.2020.08.13
6328	市腹泻患者中非伤寒沙门	e of non-typhoid Salmonell	,陈谨,王蓉,罗颖,杨晨,陈	n,Wang Rong,Luo Ying,Yan	中国人兽共患病学报	Chinese Journal of Zoonose	36	8	646-653	2020	sn.1002-2694.2020.00.094
6329	年开封市鼓楼区食源性疾	sis of foodborne diseases i	赵云清,殷锋科.	Zhao Yunqing,Yin Fengke.	河南预防医学杂志	n Journal of Preventive Me	31	8	643-645	2020	m.1006-8414.2020.08.030
6330	沙门菌血清型分布及药	ella serotype distribution a	黄黎俐,刘五高,吴宁俊.	ng Lili,Liu Wugao,Wu Ning	中国卫生检验杂志	ese Journal of Health Inspe	30	16	1952-1954	2020	-
6331	物检验在儿童腹泻诊断中	microbial testing in the dia	韩吉粉.	Hanji powder.	保健文汇	Health Wenhui	-	21	182-183	2020	sn.1671-5217.2020.21.142
6332	低温肉灌肠生产加工过	he production and process	余超,李迎月,刘于飞,李燕	hao,Li Yingyue,Liu Yufei,Li	食品安全质量检测学报	f Food Safety and Quality I	11	16	5583-5587	2020	-
6333	地区生鲜牛乳中微生物的	sis of microorganisms in fr	孙春玲,朱静,郭元晟,包世	unling,Zhu Jing,Guo Yuansh	农产品加工（下半月）	ducts processing (second ha	-	8	75-78,82	2020	1671-9646(X).2020.08.054
6334	交周边部分鲜奶吧乳品监	esults of some fresh milk b	李颖,刘辉,王一平,于维森	iu Hui,Wang Yiping,Yu Wei	医学动物防制	medical animal control	36	8	815-816,封3	2020	0.7629/yxdwfz202008029
6335	泻患儿粪便培养及抗菌药	eptibility analysis of childre	张杉,林莎莎.	Zhang Shan,Lin Shasha.	河南医学研究	Henan Medical Research	29	24	4576-4578	2020	n.1004-437X.2020.24.070
6336	沙门菌引起食物中毒的	f food poisoning caused by	令玲,周文革,张志魁,魏美	Zhou Wenge,Zhang Zhikui,	中国卫生检验杂志	ese Journal of Health Inspe	30	16	2032-2033,封3	2020	-
6337	17年沙门菌分布特征及	ella distribution characteris	玉龙,阙凤霞,董兆鹏,袁佳	e Fengxia,Dong Zhaopeng,	中国卫生检验杂志	ese Journal of Health Inspe	30	16	2006-2008	2020	-
6338	场分离的常见细菌对消毒	acteria isolated from some	林秋敏,赖宝色,杨慧,吴樟	,Lai Baose,Yang Hui,Wu Zh	黑龙江畜牧兽医（下半月）	dry and Veterinary Medicin	-	8	83-86	2020	cnki.hljxmsy.2019.10.0306
6339	起鼠伤寒沙门菌食物中毒	onella Typhimurium food	丽芳,张宏亮,张景平,张志	g Hongliang,Zhang Jingpin	海峡预防医学杂志	s Journal of Preventive Me	26	4	87-89	2020	-
6340	年—2019年食源性疾病监	9Analysis of annual foodb	魏新红.	Wei Xinhong.	基层医学论坛	Primary Medicine Forum	24	23	3374-3375	2020	/j.1672-1721.2020.23.077
6341	庭进餐引起沙门氏菌食物	poisoning incident caused	梅丽敏,余林,袁敏.	Mei Limin,Yu Lin,Yuan Min.	食品安全导刊	food safety guide	-	23	36-37	2020	-
6342	食源性疾病主动监测病例	nd analysis of active survei	周爱民,刘旭晨.	Zhou Aimin,Liu Xuchen.	临床医学研究与实践	l Medical Research and Pr	5	23	10-12	2020	nki.2096-1413.202023005
6343	区儿童腹泻沙门氏菌的感	status of Salmonella diarrh	沈赟,秦思,霍翔.	Shen Yun,Qin Si,Huo Xiang.	食品安全质量检测学报	f Food Safety and Quality I	11	15	5150-5155	2020	-
6344	区817肉雏鸡沙门氏菌溯	ation on the traceability of	姗姗,王雨蒙,李洁,冀笑	n,Wang Yumeng,Li Jie,Ji Xi	家禽科学	poultry science	-	8	45-48	2020	sn.1673-1085.2020.08.017
6345	装糕点微生物质量情况调	ysis of microbial quality of	唐轶君,李晶,李靖媛,宁鑫	Yijun,Li Jing,Li Jingyuan,Ni	食品安全质量检测学报	f Food Safety and Quality I	11	15	5195-5202	2020	-
6346	市市售猪肉食源性致病菌	pathogenic bacteria conta	李鹏.	Li Peng.	养猪	pig raising	-	4	3-4	2020	sn.1002-1957.2020.04.006
6347	性疾病特定病原体主动监	lts of specific pathogens o	张建群,苗超,诸佳辉.	g Jianqun,Miao Chao,Zhu J	中国卫生检验杂志	ese Journal of Health Inspe	30	15	1892-1895	2020	-
6348	养殖场沙门菌的分离鉴定	e detection of Salmonella f	昕萌,林琦杰,陈凯风,张建	g,Lin Qijie,Chen Kaifeng,Zh	养禽与禽病防治	and poultry disease preven	-	8	11-15	2020	-
6349	腹泻的常见病原微生物检	on pathogenic microorgani	滕勇胜,刘冰,马丽丽.	g Yongsheng,Liu Bing,Ma	中国保健营养	Chinese health nutrition	30	22	88	2020	-
6350	汉市食源性疾病流行特征	ristics and pathogenic facto	记,吴晓旻,伍雅婷,王肖,王	u Xiaomin,Wu Yating,Wang	职业与健康	Occupation and health	36	15	2061-2064	2020	-
6351	菌性食物中毒的微生物检	ical testing for bacterial foo	于淑莲.	Yu Shulian.	中国保健营养	Chinese health nutrition	30	22	85	2020	-
6352	区成年人急性感染性腹泻	in Changping District, Beiji	华,张皓,舒高林,金文军,王	Hao,Shu Gaolin,Jin Wenju	中国基层医药	China primary medicine	27	14	1770-1774	2020	sn.1008-6706.2020.14.025
6353	门菌对抗生素和消毒剂耐	almonella typhimurium to	爱平,何利,杨勇,敖晓琳,周	e Li,Yang Yong,Ao Xiaolin,Z	中国食品卫生杂志	hinese Food Hygiene Journ	32	4	356-363	2020	).13590/j.cjfh.2020.04.002
6354	市非伤寒沙门菌感染流行	racteristics of non-typhoid	段胜钢,齐辰,陆冬磊,刘弘	nggang,Qi Chen,Lu Donglei	中国食品卫生杂志	hinese Food Hygiene Journ	32	4	422-426	2020	).13590/j.cjfh.2020.04.014
6355	型多态性与PFGE分型及其	hisms andPFGEResearch o	锦江,卢晓芸,姚正原,陈洪	Xiaoyun,Yao Zhengyuan,Ch	检验医学与临床	boratory medicine and clini	17	14	2017-2019	2020	sn.1672-9455.2020.14.021
6356	引起的食物中毒事件的病	of a food poisoning incide	永艳,薛琳,王伟,樊国印,倪	n,Xue Lin,Wang Wei,Fan G	中国热带医学	Chinese tropical medicine	20	7	640-644	2020	cnki.46-1064/r.2020.07.13
6357	00份市售食品中食源性致	toring results of foodborne	曲强.	Qu Qiang.	抗感染药学	Anti-infective Pharmacy	17	7	1021-1023	2020	sn.1672-7878.2020.07-028
6358	地区急性感染性腹泻常见	ce of common pathogenic	玛依拉·艾尼瓦尔,桑国耀	iang Jing,Maira·Anivar,San	实用药物与临床	ractical medicine and clinic	23	7	641-645	2020	053/j.cnki.ppcr.202007014
6359	门菌食物中毒的病原学检	ty analysis of a case of Sal	张君,张冬民.	Zhang Jun,Zhang Dongmin.	河南预防医学杂志	n Journal of Preventive Me	31	7	567-568,572	2020	m.1006-8414.2020.07.031
6360	食源性沙门菌感染的血清	dborne Salmonella infectio	萍,李波,廖国东,许铭清,黄	o,Liao Guodong,Xu Mingqi	中国热带医学	Chinese tropical medicine	20	7	661-665	2020	cnki.46-1064/r.2020.07.18
6361	江省余姚市沙门菌血清型	type changes and drug resi	张建群,袁士杰,苗超.	g Jianqun,Yuan Shijie,Miao	疾病监测	disease surveillance	35	7	627-632	2020	sn.1003-9961.2020.07.016
6362	所致儿童肠炎的临床特点	nce of children with enteri	,龙晓玲,梁展图,张泉山,	oling,Liang Zhantu,Zhang	中国现代药物应用	dern drug application in Ch	14	14	248-250	2020	i.cn11-5581/r.2020.14.114

6363	17-2019年食源性疾病流行	019Annual epidemic chara	陈高尚,申屠平平,张涛.	oshang,Shentu Pingping,Zh	中国学校卫生	Chinese school health	41	7	1084-1087	2020	ki.1000-9817.2020.07.035
6364	售猪肉馅及冷鲜鸡中沙门	ntamination in pork stuffin	亮,刘雨蒙,李永波,刘帅,张	Yumeng,Li Yongbo,Liu Shu	食品安全质量检测学报	f Food Safety and Quality I	11	14	4714-4718	2020	-
6365	菌的分离鉴定、耐药表型	enotype analysis and drug	王旭东,李九彬,丁红雷,杨	Xudong,Li Jiubin,Ding Hong	中国畜牧兽医	nal husbandry and veterina	47	7	2256-2263	2020	ki.1671-7236.2020.07.032
6366	基在沙门氏菌检验中的检	n effect of different culture	梅,黄焕宜,黎莉,何国华,陈	ng Huanyi,Li Li,He Guohua,	医药前沿	Medical Frontier	10	20	110-112	2020	-
6367	脏病患儿围术期感染鼠伤	Salmonella typhimurium in	夏云.	Xia Yun.	当代护士（下旬刊）	ntemporary Nurses (late iss	27	7	151-153	2020	ki.1006-6411.2020.21.062
6368	南省沙门氏菌感染引起食	ne diarrhea caused by Saln	董海燕,杨彦玲,彭敏,苏玮	Haiyan,Yang Yanling,Peng M	食品安全质量检测学报	f Food Safety and Quality I	11	13	4253-4257	2020	-
6369	省食源性疾病哨点医院主	results of foodborne disea	陈文,兰真,杨小蓉,张誉.	n,Lan Zhen,Yang Xiaorong,	现代预防医学	modern preventive medicin	47	13	2466-2470	2020	-
6370	省预包装食品中致病菌污	ogenic bacteria contamina	林碧莲,柯振华.	Lin Bilian,Ke Zhenhua.	食品安全质量检测学报	f Food Safety and Quality I	11	13	4207-4213	2020	-
6371	市食源性疾病流行特征及	and dominant bacterial gr	东,王松强,戴蕾,陈欣然,孙	ng Songqiang,Dai Lei,Chen	新乡医学院学报	nal of Xinxiang Medical Co	37	7	626-629	2020	7683/xxyxyxb.2020.07.006
6372	腹泻病原检测分析与临床	etection and clinical micro	沈国武.	Shen Guowu.	中国保健营养	Chinese health nutrition	30	19	342-343	2020	-
6373	壁市11类食品致病菌监测	sis of monitoring status of	梁莹莹.	Liang Yingying.	临床研究	clinical research	28	7	3-5	2020	-
6374	泻诊断中大便常规检验的	e stool examination in the	宋秋玲.	Song Qiuling.	东方药膳	Oriental medicinal food	-	12	95	2020	-
6375	不同动物源耐药沙门氏菌	fferent animal sources in Y	亚军,王舒丰,李淑娴,向志	Wang Shufeng,Li Shuxian,X	中国农业科技导报	ultural Science and Techno	22	6	111-122	2020	13304/j.nykjdb.2019.0204
6376	引起幼儿长湾泥沙门菌感	Salmonella infection in you	,吴畏畏,张传昤,郭惠,郑	wei,Zhang Chuanhao,Guo	中国人兽共患病学报	Chinese Journal of Zoonose	36	6	520-524	2020	sn.1002-2694.2020.00.070
6377	菌群分析与腐败菌种分	-to-eat jellyfish and isolati	琳,林瑜,缪松,曾绍校,林	u,Mouzon,Zeng Shaoxiao,	食品与生物技术学报	nal of Food and Biotechno	39	6	55-60	2020	sn.1673-1689.2020.06.008
6378	州地区儿童肠道沙门菌感	infection status and drug r	,武庆斌,陆惠钢,葛海霞	ingbin,Lu Huigang,Ge Haix	中国血液流变学杂志	nese Journal of Hemorheol	30	2	217-223	2020	n.1009-881X.2020.02.024
6379	省猪源沙门菌的分离鉴定	stance analysis of Salmone	王湘如,杨斓,杨丹,王斐,华	ngru,Yang Lan,Yang Dan,W	中国兽医学报	l of Chinese Veterinary Me	40	6	1142-1147,1152	2020	nki.1005-4545.2020.06.12
6380	州市市售食品食源性致病	odborne pathogenic bact	海麟,刘于飞,梁伯衡,林晓	Liu Yufei,Liang Boheng,Lin	公共卫生与预防医学	Health and Preventive Me	31	3	76-79	2020	sn.1006-2483.2020.03.019
6381	售冷冻羊肉沙门氏菌污染	contamination in commer	李鹏.	Li Peng.	中国动物传染病学报	ournal of Animal Infectious	28	3	91-96	2020	-
6382	株鸽源沙门氏菌致病性观	genicity of Salmonella strai	伊薇,张焕容,任玉鹏,杨劲松	ang Huanrong,Ren Yupeng	中国动物传染病学报	ournal of Animal Infectious	28	3	7-13	2020	-
6383	及电离飞行时间质谱与血	ation time-of-flight mass sp	凌娟,钟国权,温伟洪,李玉	,Zhong Guoquan,Wen Wei	中国热带医学	Chinese tropical medicine	20	6	495-499	2020	cnki.46-1064/r.2020.06.01
6384	东地区家禽沙门菌的分离	and drug resistance analy	乐,费中杰,杨铜,钱程,张凯	ngjie,Yang Tong,Qian Chen,	中国预防兽医学报	rnal of Preventive Veterina	42	6	549-554	2020	ssn.1008-0589.201910001
6385	规模化鸡场沙门氏菌病流	almonellosis in large-scale	薛邦玉.	Xue Bangyu.	河南畜牧兽医（市场版）	andry and Veterinary Medic	41	6	36-37	2020	sn.1004-5090.2020.06.016
6386	检测方法在预防性体检便	ods for Salmonella in preve	徐明治.	Xu Mingzhi.	特别健康	Very healthy	-	18	43	2020	-
6387	宝鸡市市售肉及肉制品致病	acteria in commercially ava	任婧,陈喆霄,魏雯,梁文革	Chen Zhexiao,Wei Wen,Lian	医学动物防制	medical animal control	36	6	561-563	2020	0.7629/yxdwfz202006015
6388	门菌引起的食源性疾病调	alysis of a foodborne illness	梅,杨红霞,王洋,张晓华,赵	ongxia,Wang Yang,Zhang X	中国卫生检验杂志	ese Journal of Health Inspe	30	12	1529-1531	2020	-
6389	赴港澳旅行团食源性疾病	e diseases in a mainland to	陈光中,卢志敏,卢志慧.	Guangzhong,Lu Zhimin,Lu Z	中国国境卫生检疫杂志	ontier Health and Quarantir	43	3	182-183,194	2020	3/j.1004-9770.2020.03.010
6390	-2019年学校食源性疾病流	Epidemiological survey of f	,陈江,陈莉莉,齐小娟,张	ang,Chen Lili,Qi Xiaojuan,Z	中国学校卫生	Chinese school health	41	6	901-903,907	2020	ki.1000-9817.2020.06.027
6391	南地方鸡沙门氏菌血清学	ation of Salmonella in local	王天葆,李廷翠,陈富珍,张	i Tingcui,Chen Fuzhen,Zha	云南畜牧兽医	nal Husbandry and Veterina	-	3	1-4	2020	sn.1005-1341.2020.03.001
6392	年湖南省沙门菌耐药状况	resistance status and dist	云,任国峰,梁进军,刘晓	en Guofeng,Liang Jinjun,Liu	中国抗生素杂志	hinese Journal of Antibioti	45	6	621-626	2020	sn.1001-8689.2020.06.014
6393	周边地区仔猪腹泻的临床	n piglet diarrhea in surrou	张明洋,况玲.	hang Mingyang,Kuang Ling	畜牧兽医科技信息	Veterinary Science and Te	-	6	32-34	2020	N.1671-6027.2020.06.020
6394	牛场犊牛腹泻沙门氏菌的	Salmonella from calf diar	李淑红,刘勇,孙明兰,王京	,Liu Yong,Sun Minglan,Wa	江苏农业科学	iangsu Agricultural Science	48	12	167-169	2020	sn.1002-1302.2020.12.036
6395	患者粪便常规临床检验结	examination results of sto	林立群.	Lin Liqun.	临床医药文献电子杂志	edical literature electronic	7	49	138140	2020	-
6396	沙门氏菌引起食物中毒的	case of food poisoning ca	全生,雷务年,秦燕妮,蒋秋	g,year of thunder,Qin Yann	保健文汇	Health Wenhui	-	17	278-279	2020	sn.1671-5217.2020.17.182
6397	沙门菌感染临床特征及抗	atment progress of non-ty	石国露,李中跃.	Shi Guolu,Li Zhongyue.	中华实用儿科临床杂志	Clinical Journal of Practical	35	11	874-877	2020	n101070-20200526-00892
6398	羽肉种鸡场沙门氏菌的分	lmonella in large-scale wh	旭升,刘鹏,刘国华,曲贞	g,Liu Peng,Liu Guohua,Qu	家禽科学	poultry science	-	6	53-57	2020	sn.1673-1085.2020.06.020
6399	西双版纳州食源性致病因	lts of foodborne pathogen	路,王宇,李光明,杨雅麟,吴	Yu,Li Guangming,Yang Yal	食品安全质量检测学报	f Food Safety and Quality I	11	11	3654-3659	2020	-

6400	市食源性疾病病例流行特	istics and dietary history o	陆冬磊,段胜钢,齐辰,刘弘	i,Duan Shenggang,Qi Chen	现代预防医学	nodern preventive medicin	47	11	1970-1974	2020	-
6401	儿伤寒沙门菌感染54例临	ium infection in children54	智群,林菁,朱炜春,马丙南	n,Lin Jing,Zhu Weichun,Ma	当代医学	contemporary medicine	26	16	147-149	2020	sn.1009-4393.2020.16.062
6402	性食物中毒的微生物学检	ination and analysis of bac	崔忠心.	Cui Zhongxin.	家庭医药.就医选药	ne.Medical treatment and	-	6	359	2020	-
6403	治区市售婴幼儿食品食源	nic bacteria in commercial	婷,赵鹏,蒋玉艳,李秀桂,韦	Peng,Jiang Yuyan,Li Xiugu	中国食品卫生杂志	hinese Food Hygiene Journ	32	3	288-293	2020	).13590/j.cjfh.2020.03.014
6404	年我院沙门菌感染情况和	infection situation and drug	黄璐.	Huang Lu.	海峡药学	Straits Pharmacy	32	5	210-212	2020	sn.1006-3765.2020.05.093
6405	汤道门诊成年人急性细菌	acute bacterial diarrhea ar	吕冰,曲梅,霍达,张新,黄瑛	u Mei,Hoda,Zhang Xin,Hua	疾病监测	disease surveillance	35	5	411-415	2020	sn.1003-9961.2020.05.010
6406	开封市售食品食源性致病	dborne pathogenic bacteria	许姣,陈磊,巩飙.	Xu Jiao,Chen Lei,Gong Biao	河南预防医学杂志	n Journal of Preventive Me	31	5	408-411	2020	m.1006-8414.2020.05.026
6407	内2019年食品污染菌检测	19Analysis of annual food	李锋涛.	Li Fengtao.	抗感染药学	Anti-infective Pharmacy	17	5	703-706	2020	sn.1672-7878.2020.05-022
6408	验验在季节性腹泻检验中的	he stool examination in det	杜思菊.	Du Siju.	养生保健指南	Health and Wellness Guide	-	21	14	2020	-
6409	伤寒沙门菌感染治疗特点及	eptibility results of complic	赵夜,林菁,赖宗乐.	Zhao Ye,Lin Jing,Lai Zongle.	中国医学创新	Chinese medical innovation	17	15	121-125	2020	sn.1674-4985.2020.15.031
6410	陵市食品中食源性致病菌	sults of foodborne pathoge	华,汪道发,盛维立,王蓓蕾	a,Wang Daofa,Sheng Weili,	现代预防医学	nodern preventive medicin	47	10	1774-1777	2020	-
6411	畜禽肉中沙门氏菌污染情况	la contamination in fresh li	郭焜鹏,张海韵,王芳斌,唐	peng,Zhang Haiyun,Wang F	食品安全导刊	food safety guide	-	15	97-98	2020	-
6412	区2014—2016年腹泻病例监	4-2016Analysis of annual di	戚玉萍,栾晶,王宙云,朱效宁	,Luan Jing,Wang Zhouyun,	上海预防医学	hanghai Preventive Medicir	32	5	408-411	2020	28/j.cnki.sjpm.2020.18932
6413	某区食源性疾病主动监测	active surveillance of food	陈静.	Chen Jing.	中国医药指南	Chinese Medicine Guide	18	14	16-18	2020	-
6414	菌肠炎临床特征和血清型及	e and drug resistance analy	李佐君,钱冬梅.	Li Zuojun,Qian Dongmei.	中国临床研究	clinical research in china	33	5	654-657	2020	29/j.cnki.cjcr.2020.05.020
6415	沙门氏菌引起的食源性	oodborne illness caused by	李静,徐贵永,张静.	Jing,Xu Guiyong,Zhang Jin	健康教育与健康促进	Education and Health Pror	15	3	324-325	2020	cnki.31-1974/r.202003034
6416	伤寒沙门菌的耐药性检测	enotyping of non-typhoid S	李立巍.	Li Liwei.	临床医药文献电子杂志	edical literature electronic	7	40	17,47	2020	-
6417	性腹泻的病原微生物检验	athogenic microorganisms	向慧.	Xiang Hui.	东方药膳	Oriental medicinal food	-	9	105	2020	-
6418	肠杆菌科细菌耐药性分析	resistance of Enterobacte	唐泌然.	Tang Biran.	东方药膳	Oriental medicinal food	-	9	89	2020	-
6419	急性腹泻病原菌检验结果	bacteria test results for acu	王贞美.	Wang Zhenmei.	医药前沿	Medical Frontier	10	14	80-81	2020	-
6420	仙桃市伤寒沙门菌分子分型	drug resistance analysis of	敏,邢学森,吕静,何飞,周海	ng Xuesen,Lu Jing,He Fei,Z	国际检验医学杂志	onal journal of laboratory r	41	9	1042-1046,1051	2020	sn.1673-4130.2020.09.005
6421	规检验在季节性腹泻诊治中	ination in the diagnosis an	张婷婷,李柯芬,燕晶晶.	g Tingting,Li Kefen,Yan Jing	西医结合心血管病电子杂	Diseases Integrated Traditi	8	14	94	2020	-
6422	8年食品监测常见食源性致	n of common foodborne p	陆子春,蒋一,曾宏.	u Zichun,Jiang Yi,Zeng Hong	中国初级卫生保健	primary health care in china	34	5	84-87	2020	.1001-568X.2020.05.0025
6423	家哨点医院主动监测的食	of active surveillance of fo	王悦,时晨,于良.	Wang Yue,morning,Yu Liang	食品安全质量检测学报	f Food Safety and Quality I	11	9	3029-3034	2020	-
6424	菌和德尔卑沙门菌耐药性	aracteristics of Salmonella	张彬,章乐怡,李毅,谢爱蓉	Bin,Zhang Leyi,Li Yi,Xie Air	中国卫生检验杂志	ese Journal of Health Inspe	30	9	1056-1058,1062	2020	-
6425	我国进口食品食源性致病菌	atus of foodborne pathoge	进玲,宁雪,王新,蒋原,杨捷	Ning Xue,Wang Xin,Jiang Yu	食品安全质量检测学报	f Food Safety and Quality I	11	9	2930-2935	2020	-
6426	江西省食源性疾病病原学	he disease etiology surveill	德,刘洋,彭思露,刘道峰,文	iu Yang,Peng Silu,Liu Daofe	现代预防医学	nodern preventive medicin	47	9	1678-1683	2020	-
6427	黄色葡萄球菌污染状况及	drug resistance of Staphyl	汤越超,邹颜秋硕,任翔,汤晓	,Zou Yanqiushuo,Ren Xiang	食品安全质量检测学报	f Food Safety and Quality I	11	9	2958-2962	2020	-
6428	感染性腹泻临床特点及病	hogen analysis of bacterial	王萍萍,刘荻,金雪锋.	ng Pingping,Liu Di,Jin Xuefe	儿科药学杂志	ournal of Pediatric Pharmac	26	5	26-28	2020	p.1672-108X.2020.05.008
6429	年湖州地区食源性疾病病	n of pathogenic bacteria of	华,周丽华,王萍,嵇龙飞,郑	hou Lihua,Wang Ping,Ji Lor	中国卫生检验杂志	ese Journal of Health Inspe	30	9	1118-1120	2020	-
6430	东省市售鸡蛋中沙门氏菌的	ssment of Salmonella in co	岚,陈洪升,梁骏华,闻剑,梁	ongsheng,Liang Junhua,We	食品安全质量检测学报	f Food Safety and Quality I	11	9	2969-2973	2020	-
6431	省动物性水产品7种致病微	dong Province7Analysis of	岚,黄盼盼,卢玲玲,梁辉,闻	g Panpan,Lu Lingling,Liang	食品安全质量检测学报	f Food Safety and Quality I	11	9	2980-2985	2020	-
6432	市房山区弯曲菌监测结果及	sults and drug resistance a	刘颖,王云霞,史文凤,黄少	g Yunxia,Shi Wenfeng,Hua	职业与健康	Occupation and health	36	9	1197-1199,1204	2020	-
6433	售散装即食食品食源性致	c bacteria contamination st	江南,王双,周景林,赵凤玲,王	huang,Zhou Jinglin,Zhao Fe	预防医学情报杂志	of Preventive Medicine Info	36	4	417-420	2020	-
6434	菌感染患者的临床特征及耐	ics and drug resistance in p	鲁稻,徐秀亮,潮燕,刘小玲	,Xu Xiuliang,Chaoyan,Liu X	公共卫生与预防医学	Health and Preventive Me	31	2	141-144	2020	sn.1006-2483.2020.02.035
6435	宁河区哨点医院食源性疾	oodborne diseases in senti	董兴华.	Dong Xinghua.	中国城乡企业卫生	ygiene in urban and rural ar	35	4	4-6	2020	/j.1003-5052.2020.04.002
6436	标本细菌培养及其药敏结	fecal samples of children w	赵秀芝.	Zhao Xiuzhi.	抗感染药学	Anti-infective Pharmacy	17	4	520-522	2020	sn.1672-7878.2020.04-019

6437	青岛市腹泻病例中沙门菌	e analysis of Salmonella in	凯,刘晓琳,王伟栋,曲剑	u Xiaolin,Wang Weidong,Q	疾病监测	disease surveillance	35	4	345-349	2020	sn.1003-9961.2020.04.015
6438	血清型鉴定及CTX-M型ES	n in slaughtered meat and	岩,张莉娟,常满霞,赵秋	,Zhang Lijuan,Chang Manx	中国兽医学报	l of Chinese Veterinary Me	40	4	707-712	2020	nki.1005-4545.2020.04.08
6439	区其他感染性腹泻病流行	ics of other infectious diar	,秦宏兴,潘燕莹,彭志勇,	gxing,Pan Yanying,Peng Zh	疾病预防控制通报	se Prevention and Control	35	2	41-43	2020	15/j.cnki.jbyfkztb.2002005
6440	性结膜炎患者病原学及流	epidemiological characteri	存,范雪松,华近,周林,郎	an Xuesong,Huajin,Zhou L	国际病毒学杂志	ternational journal of virolo	27	2	163-166	2020	sn.1673-4092.2020.02.018
6441	区空气中细菌总数分布与	etection of common patho	储燕萍,赵望,刘思秀.	u Yanping,Zhao Wang,Liu Si	复旦学报（自然科学版）	dan University (Natural Sci	59	2	240-244	2020	-
6442	谱区食源性疾病监测结果	disease surveillance results	况强华,邓虹,周军华.	Qianghua,Deng Hong,Zhou	医学信息	medical information	33	8	135-136	2020	sn.1006-1959.2020.08.044
6443	例细菌性腹泻病原学检测	etection of etiology of bact	曲识.	Quji.	航空航天医学杂志	urnal of Aerospace Medici	31	4	477-478	2020	sn.2095-1434.2020.04.050
6444	年病例沙门菌检出率ARIM	ction rate among annual ca	任国峰,赖天兵,梁进军,刘	ofeng,Lai Tianbing,Liang Jin	中国卫生统计	China health statistics	37	2	272-275	2020	-
6445	年河北省食物中毒事件流	c characteristics of food po	,陈磊,罗晓燕,赵永丽,刘	i,Luo Xiaoyan,Zhao Yongli,	现代预防医学	modern preventive medicin	47	8	1381-1384	2020	-
6446	染性腹泻临床特点及病原	etiological surveillance resu	张冬雨.	Zhang Dongyu.	中国中西医结合儿科学	Chinese and Western Medi	12	2	176-180	2020	sn.1674-3865.2020.02.024
6447	市蛋类样品沙门氏菌脉冲	esistance analysis of Salm	云,褚红娜,王伟,王蒋丽,高	Hongna,Wang Wei,Wang Jia	医学动物防制	medical animal control	36	4	352-355	2020	0.7629/yxdwfz202004015
6448	9年5类食品中食源性致病	ntamination status of food	李霞.	Li Xia.	饮食保健	Diet and health care	7	17	280-281	2020	-
6449	区猪肉食品中致病性沙门	f pathogenic Salmonella in	赵维章,张正英.	o Weizhang,Zhang Zhengy	中国兽医杂志	Chinese Veterinary Journal	56	4	80-82,86	2020	-
6450	食源性致病菌检测结果分	borne pathogenic bacteria	范霞.	Fan Xia.	食品安全导刊	food safety guide	-	12	99-100	2020	-
6451	非伤寒沙门菌感染的临床	nce analysis of intestinal n	仕勇,汤卫红,王萍萍,魏金	g,Tang Weihong,Wang Pin	现代实用医学	modern practical medicine	32	4	461-463,封3	2020	sn.1671-0800.2020.04.019
6452	性食物中毒的微生物学检	ination and analysis of bac	柏海兰.	Bai Hailan.	中国农村卫生	rural health in china	12	8	47	2020	n.1674-361X.2020.08.045
6453	门氏杆菌病原分离鉴定及	n and drug resistance analy	懿,玉耀贤,许光明,农忠	aoxian,Xu Guangming,Non	中国动物保健	China animal health	22	4	29-30	2020	sn.1008-4754.2020.04.026
6454	染性腹泻疾病的流行及病	etiological characteristics c	郭雪鸿.	Guo Xuehong.	中国卫生标准管理	a Health Standard Manage	11	7	16-18	2020	sn.1674-9316.2020.07.006
6455	市售食品中食源性病原微	results of foodborne path	魏夺.	Wei Duo.	海峡预防医学杂志	s Journal of Preventive Me	26	2	53-55	2020	-
6456	8年山西省伤寒副伤寒流	aracteristics of typhoid an	国华,高雪芬,李晓清,赵维	,Gao Xuefen,Li Xiaoqing,Zh	实用医技杂志	l of Practical Medical Tech	27	4	473-474	2020	ki.1671-5098.2020.04.029
6457	门菌菌血症首诊的非霍奇	a first diagnosed with non-	晔,张昕宇,周志安,王立	hang Xinyu,Zhou Zhian,Wa	验和临床感染病杂志（电	ntal and Clinical Infectious	14	2	167-171	2020	sn.1674-1358.2020.02.015
6458	菌性食物中毒的微生物学	ical testing of bacterial foo	刘小燕.	Liu Xiaoyan.	医学美学美容	edical Aesthetics and Beau	29	7	108	2020	-
6459	炎沙门菌食物中毒事件的	g incident involving Salmo	李映霞,张欣强,许少洪,黄	Zhang Xinqiang,Xu Shaoho	海峡预防医学杂志	s Journal of Preventive Me	26	2	47-48	2020	-
6460	医院神经内科多重耐药	l infections in the Departm	王捷,王跃平.	Wang Jie,Wang Yueping.	中国保健营养	Chinese health nutrition	30	11	275-276	2020	-
6461	性腹泻检验中大便常规	f routine stool testing in se	李莉.	Li Li.	中国保健营养	Chinese health nutrition	30	11	320-321	2020	-
6462	性腹泻患者中分离沙门菌	almonella isolates from pa	丽,王君,马琳,石一,张铮,刘	Jun,Ma Lin,Shi Yi,Zhang Zh	现代预防医学	modern preventive medicin	47	7	1281-1284	2020	-
6463	螺蛳粉沙门氏菌实时荧光	wder Salmonella real-time	龙,覃巧思,罗璐,韦维,韦	Qin Qiaosi,Luo Lu,Weiwei,W	食品安全质量检测学报	f Food Safety and Quality I	11	7	2197-2203	2020	-
6464	食源性疾病病原菌监测结	sults of foodborne disease	佳辉,罗学辉,张建群,黄邵	o Xuehui,Zhang Jianqun,Hu	中国卫生检验杂志	ese Journal of Health Inspe	30	7	884-887	2020	-
6465	年绍兴市越城区食品安全	k monitoring analysis in Yu	迎春,孙一富,殷德荣,秦	gchun,Sun Yifu,Yin Derong,	中国卫生检验杂志	ese Journal of Health Inspe	30	7	856-858	2020	-
6466	相芯片技术与实时荧光定	uantificationP CComparis	广进,张福真,胡亮杉,曹	jin,Zhang Fuzhen,Hu Liangs	基础医学与临床	Basic medicine and clinical	40	4	523-527	2020	sn.1001-6325.2020.04.018
6467	动监测工作在哨点医院监	of active foodborne disease	张苗,王雪梅,王银平.	Miao,Wang Xuemei,Wang Y	中国保健营养	Chinese health nutrition	30	10	378	2020	-
6468	018年食源性腹泻患者病	the detection of pathogen	,强鑫华,周丽华,王萍,郑	Xinhua,Zhou Lihua,Wang P	中国乡村医药	Chinese rural medicine	27	6	58-59	2020	sn.1006-5180.2020.06.034
6469	糜制品中常见食源性致病	thogenic bacteria in comm	舒然,闫琳,裴晓燕,杨大	uran,Yan Lin,Pei Xiaoyan,Ya	中国食品卫生杂志	hinese Food Hygiene Journ	32	2	180-183	2020	).13590/j.cjfh.2020.02.014
6470	腹泻病例沙门菌血清型分	nella serotype distribution	悦康,吴灿权,刘绮明,仇绮	kang,Wu Canquan,Liu Qimi	中国食品卫生杂志	hinese Food Hygiene Journ	32	2	134-138	2020	).13590/j.cjfh.2020.02.005
6471	市一起伤寒疫情的实验	nalysis of a typhoid fever e	阚飙,周海健,罗迪,施超,钱	ao,Zhou Haijian,Roddy,Shi	中华预防医学杂志	se Journal of Preventive Me	54	3	323-326	2020	sn.0253-9624.2020.03.015
6472	2013-2017年感染性腹泻病	n the epidemic characteris	张子科,余建兴,叶楚楚,薛	g Zike,Yu Jianxing,Ye Chuch	中华流行病学杂志	inese Journal of Epidemiolo	41	3	417-422	2020	sn.0254-6450.2020.03.026
6473	病监测中病原微生物检验	organism test results in fo	韩进兰.	Han Jinlan.	临床检验杂志（电子版）	f Clinical Testing (electroni	9	1	122	2020	-

6474	广活性污泥中细菌的分离鉴	nalysis of bacteria in activa	苹,范紫娟,陈艳媛,吴爽爽,饶	uan,Chen Yanyuan,Wu Shua	基因组学与应用生物学	enomics and Applied Biolog	39	3	1191-1197	2020	0.13417/j.gab.039.001191
6475	2017年人源沙门菌血清型	aracteristics and molecula	陈悠,樊飞,施菊萍,钱惠芬	n You,Fan Fei,Shi Juping,Qia	中国热带医学	Chinese tropical medicine	20	3	236-240	2020	cnki.46-1064/r.2020.03.09
6476	少门菌血清种类的区位熵	py model and deviation of	曾献莹,韦程媛,吕素玲,李	anying,Wei Chengyuan,Lu S	中国热带医学	Chinese tropical medicine	20	3	259-262,266	2020	cnki.46-1064/r.2020.03.14
6477	测基线预测广西玉林市从	s in Yulin City, Guangxi bas	丽,王芳,梁雯,王鸣柳,施春雷	ang,Liang Wen,Wang Ming	疾病监测	disease surveillance	35	3	212-217	2020	sn.1003-9961.2020.03.008
6478	医院儿童腹泻患者沙门菌感	almonella infection in child	付永强,虢艳,邓秋连,周珍文	g,Guo Yan,Deng Qiulian,Zh	热带医学杂志	journal of tropical medicine	20	3	407-409	2020	sn.1672-3619.2020.03.032
6479	光明新区感染性腹泻病原	infectious diarrhea pathog	氏,蒋杨,李燕,姚炜,汪东篱,吴	ang,Li Yan,Yao Wei,Wang	医学信息	medical information	33	6	128-130	2020	sn.1006-1959.2020.06.040
6480	市食源性疾病住院病例流行	thogen distribution of hos	陈艳,李鹏,王虹玲,张永利	Li Peng,Wang Hongling,Zha	中国卫生检验杂志	ese Journal of Health Inspe	30	6	738-739,742	2020	-
6481	速食品中病原微生物的检	thogenic microorganisms i	钱坤,任连泉.	Qian Kun,Ren Lianquan.	肉类工业	meat industry	-	3	43-46	2020	sn.1008-5467.2020.03.009
6482	院2014～2018年肠道门诊	demiological analysis of ga	田媛媛,苏建荣,王克迪,杜宇	nyuan,Su Jianrong,Wang Ke	临床和实验医学杂志	f Clinical and Experimental	19	6	666-671	2020	sn.1671-4695.2020.06.029
6483	韦泰夫雷登沙门氏菌食物中	a Wetevreden food poisoni	秋琼,单桂花,彭明益,陈庆	le Osmanthus,Peng Mingyi	医学动物防制	medical animal control	36	3	300-304	2020	0.7629/yxdwfz202003028
6484	全保障中21家酒店微生物	entstwenty onehotel micro	冯春艳,冯晨韵,李建丽.	Chunyan,Feng Chenyun,Li	食品安全导刊	food safety guide	-	9	107-108,110	2020	-
6485	主要流行血清型耐药性的	resistance of main prevale	郑林,祝令伟,郭学军,陈萍	,Zhu Lingwei,Guo Xuejun,C	江苏农业科学	iangsu Agricultural Science	48	6	8-12	2020	sn.1002-1302.2020.06.002
6486	病原学特征及PCT、CD64和	diarrhea in preschool child	旭鑫,何慧君,张凤宜,何天	He Huijun,Zhang Fengyi,He	现代生物医学进展	ogress in Modern Biomedic	20	8	1506-1510	2020	41/j.cnki.pmb.2020.08.023
6487	京市房山区腹泻监测病例	ts of diarrhea surveillance	黄少平,李爱军,赵霞,周彦明	bing,Li Aijun,Zhao Xia,Zhou	职业与健康	Occupation and health	36	6	749-751,756	2020	-
6488	食生鲜果蔬病原微生物污染	contamination of ready-to-	宁,王珍,蔡铮,肖英平,杨华	en,Cai Zheng,Xiao Yingping	浙江农业科学	hejiang Agricultural Science	61	3	528-530	2020	.issn.0528-9017.20200343
6489	年四川省沙门菌食源性疾	nella foodborne disease ou	周良君,陈文,汪勇,梁秀川	n,Chen Wen,Wang Yong,Lia	现代预防医学	modern preventive medicin	47	5	769-771,775	2020	-
6490	2018年腹泻症候群监测病	s of etiology of annual diar	刘新凤,蒋小娟,王琪,张蕾洁	Jiang Xiaojuan,Wang Qi,Zh	中华疾病控制杂志	nese Journal of Disease Cor	24	3	303-307,347	2020	/j.cnki.zhjbkz.2020.03.011
6491	和血清学在沙门菌临床检验	ve biochemical reactions a	张玉德.	Zhang Yude.	大医生	great doctor	5	5	125-126	2020	-
6492	疾病主动监测实验室检测结	results for active surveillar	张苗,王雪梅,王银平.	Miao,Wang Xuemei,Wang Y	中国保健营养	Chinese health nutrition	30	7	275-276	2020	-
6493	上海市青浦区食源性疾病病	surveillance of foodborne d	高红梅,吴丽珠,马英.	o Hongmei,Wu Lizhu,Ma Yi	职业与健康	Occupation and health	36	5	608-612,617	2020	-
6494	医院食源性疾病主动监测结	nce results of foodborne di	王雪梅,张苗.	Wang Xuemei,Zhang Miao.	中国保健营养	Chinese health nutrition	30	7	277	2020	-
6495	检验在季节性腹泻检验中的	e stool examination in dete	姜铭.	Jiang Ming.	中国医药指南	Chinese Medicine Guide	18	6	169-170	2020	-
6496	年定西市食品中食源性致病	ysis of foodborne pathogen	赵娟,马成,田菊梅.	ao Juan,Ma Cheng,Tian Jum	疾病预防控制通报	se Prevention and Control	35	1	41-43	2020	15/j.cnki.jbyfkztb.1911011
6497	蔬菜中Pb、Cd污染评价和健	enterPb,cdContamination a	春霞,唐伟,李亚辉,杨海峰,张	Tang Wei,Li Yahui,Yang Ha	环境污染与防治	nmental pollution and prev	42	2	227-231	2020	ki.1001-3865.2020.02.019
6498	宴引起肠炎沙门菌感染的	ella Enteritidis infection ca	王贵奇,邹婷,林钢,彭尚明	iqi,Zou Ting,Lin Gang,Peng	预防医学情报杂志	of Preventive Medicine Info	36	2	240-244	2020	-
6499	氏菌引起食物中毒事件的	f a food poisoning incident	杨敬金.	Yang Jingjin.	继续医学教育	ontinuing medical educatio	34	2	142-143	2020	sn.1004-6763.2020.02.078
6500	病例中的分离沙门菌耐药	ectrum and drug resistance	朱,陈晴晴,张竹慧,撒楠,袁媛	qing,Zhang Zhuhui,Sanan,Y	中华预防医学杂志	se Journal of Preventive Me	54	2	187-191	2020	sn.0253-9624.2020.02.014
6501	定点屠宰场和农贸市场的沙	Salmonella originating fron	睿鹏,郑晓琴,朱梦含,胥兰	heng Xiaoqin,Zhu Menghan	新疆农业科学	Xinjiang Agricultural Science	57	2	326-332	2020	sn.1001-4330.2020.02.014
6502	儿粪便中病原微生物的检验	thogenic microorganisms i	陈岗,郭红,蒋军,郭方.	ang,Guo Hong,Jiang Jun,Gu	检验医学与临床	boratory medicine and clini	17	4	555-558	2020	sn.1672-9455.2020.04.038
6503	中细菌培养结果及主要致	e diarrhea and analysis of t	刘振华.	Liu Zhenhua.	抗感染药学	Anti-infective Pharmacy	17	2	183-186	2020	sn.1672-7878.2020.02-009
6504	江阴市从业人员肠道沙门菌	la carriage among employe	晓骏,周品众,张宏宾,汤丽	hou Pinzhong,Zhang Hongb	医学动物防制	medical animal control	36	2	194-196	2020	0.7629/yxdwfz202002026
6505	门菌引起食物中毒的检验	of food poisoning caused b	李志军,程光.	Li Zhijun,Cheng Guang.	特别健康	Very healthy	-	6	53-54	2020	-
6506	白事宴席引发的食物中毒	food poisoning caused by	程家国,谭晓东,刘敏,杨佑坤	uo,Tan Xiaodong,Liu Min,Ya	医学动物防制	medical animal control	36	2	175-178	2020	0.7629/yxdwfz202002020
6507	地区儿童血流感染病原菌	ce of pathogenic bacteria in	嘉慧,王双杰,岑贞娇,曾尚娟	angjie,Cen Zhenjiao,Zeng S	实用医学杂志	ournal of Practical Medicin	36	4	527-531	2020	sn.1006-5725.2020.04.022
6508	鼠伤寒沙门菌食物中毒事件	of salmonella typhimurium	陈艳,邬其欢,李科峰.	hen Yan,Wu Qihuan,Li Kefer	中国卫生检验杂志	ese Journal of Health Inspe	30	4	499-501	2020	-
6509	岸水源性传染病致病菌分	genic bacteria of waterborn	洪烨,郑夔,师永霞,李小波	Zheng Kui,Shi Yongxia,Li Xia	中国国境卫生检疫杂志	ntier Health and Quarantin	43	1	53-57	2020	3/j.1004-9770.2020.01.018
6510	乳房炎致病菌地域分布及	nges of pathogenic bacteri	张立梅,顾小龙,张檬,霍文琳	Gu Xiaolong,Zhang Meng,H	中国兽医杂志	Chinese Veterinary Journal	56	2	22-26	2020	-

6511	伤寒沙门菌的分离鉴定及	eptibility test of Salmonell	铭,高尚,徐磊,王彦红,王晓	ble,Xu Lei,Wang Yanhong,W	中国兽医杂志	Chinese Veterinary Journal	56	2	82-84,88	2020	-
6512	验在季节性腹泻检验中的	of routine stool examinati	宋洁.	Song Jie.	健康必读	Health must read	-	6	30,29	2020	-
6513	菌性食物中毒的微生物学	ical testing of bacterial foo	马力.	horsepower.	食品安全导刊	food safety guide	-	6	147	2020	-
6514	年食源性腹泻病例病原体	sentinel surveillance result	慧琳,朱磊,陈秀红,李祥,张	hu Lei,Chen Xiuhong,Li Xian	安徽预防医学杂志	i Journal of Preventive Me	26	1	15-18	2020	37/j.cnki.ahyf.2020.01.005
6515	临床沙门菌分离株血清型	types and drug resistance c	,傅祎欣,叶玲清,李闽真,陈	ixin,Ye Lingqing,Li Minzhen	海峡预防医学杂志	s Journal of Preventive Me	26	1	48-50	2020	-
6516	54例病原微生物检验情况	f pathogenic microbial test	孙春涛,盛优静.	Sun Chuntao,Sheng Youjing	宁夏医学杂志	Ningxia Medical Journal	42	2	162-163	2020	j.1001-5949.2020.02.0162
6517	大肠杆菌和沙门氏菌性仔	i and salmonella piglet diar	万里,高雪,刘东旭,李国江,	o Xue,Liu Dongxu,Li Guojia	安徽农业科学	Anhui Agricultural Sciences	48	3	89-90,100	2020	sn.0517-6611.2020.03.026
6518	在腹泻诊断中应用及对治	esting in the diagnosis of d	徐石秀,李尤,曾冬梅.	Shixiu,Li You,Zeng Dongm	东方药膳	Oriental medicinal food	-	3	103	2020	-
6519	肠炎沙门菌食物中毒调	easures of Salmonella Ente	杭生,邱春晖,李丽丽,吴兴	heng,Qiu Chunhui,Li Lili,Wu	海峡预防医学杂志	s Journal of Preventive Me	26	1	79-80	2020	-
6520	菌肠炎患儿恢复期带菌危	hildren with Salmonella ty	丙南,林菁,周洁雯,高智群	an,Lin Jing,Zhou Jiewen,Ga	山东医药	Shandong Medicine	60	5	79-81	2020	n.1002-266X.2020.05.021
6521	者粪便常规临床检验结果	clinical examination result	苏文文.	Su Wenwen.	临床医药文献电子杂志	edical literature electronic	7	14	55	2020	-
6522	类中沙门氏菌全基因组分	g resistance analysis of Sal	向莹,杨磊,赵琢,李小林,史	ng,Yang Lei,Zhao Zhuo,Li Xi	食品安全质量检测学报	f Food Safety and Quality I	11	3	783-791	2020	-
6523	中志贺菌和沙门菌的分布	is of Shigella and Salmonel	会海,张盼,张海谱,秦立	hai,Zhang Pan,Zhang Haipu	海南医学	Hainan Medicine	31	3	295-298	2020	sn.1003-6350.2020.03.007
6524	儿粪便标本病原微生物检	nd drug resistance in fecal	魏喜典,贾红丽,秦新峰,刘	i Xidian,Jia Hongli,Qin Xinfe	河南医学研究	Henan Medical Research	29	4	711-713	2020	n.1004-437X.2020.04.072
6525	验验在季节性腹泻诊断中	e stool examination in the	陈光.	Chen Guang.	医药前沿	Medical Frontier	10	4	92-93	2020	-
6526	寒沙门菌所致伤寒症的病	atment of typhoid fever ca	马玉兰,刘淑梅.	Ma Yulan,Liu Shumei.	特别健康	Very healthy	-	4	76	2020	-
6527	陆良县哺乳仔猪腹泻病原	factors of diarrhea in suckli	董昱廷,王乔平.	Dong Yuting,Wang Qiaoping	兽医导刊	veterinary guide	-	2	152	2020	-
6528	源性疾病主动监测病原学	gical characteristics of acti	丽红,陈安明,张景辉,王彦	en Anming,Zhang Jinghui,W	中国食品卫生杂志	hinese Food Hygiene Journ	32	1	72-76	2020	).13590/j.cjfh.2020.01.014
6529	肉鸡相关样品中沙门菌	nella in broiler-related sam	彦霏,王银,黄巾凌,彭子欣	Wang Yin,Yellow Turban Li	中国食品卫生杂志	hinese Food Hygiene Journ	32	1	66-71	2020	).13590/j.cjfh.2020.01.013
6530	性腹泻中沙门菌血清型及	es and drug resistance in c	俊文,杨俊梅,高凯杰,王颖	,Yang Junmei,Gao Kaijie,W	中国妇幼健康研究	maternal and child health r	31	1	96-99	2020	sn.1673-5293.2020.01.021
6531	年急性传染性腹泻致病菌	pathogenic bacteria in acu	陶理智.	Tao Lili.	现代消化及介入诊疗	and interventional diagno	25	1	16-20	2020	sn.1672-2159.2020.01.004
6532	觉县饮用水中微生物检	rganisms in drinking water	果布次仁,索朗曲珍.	bu Tsering,Suolang Quzhe	农技服务	gricultural technical service	37	1	83-84	2020	-
6533	体微生物、蛋鸭粪便微生	microorganisms, laying du	宇,叶胜强,王丽霞,邓兵,陈	engqiang,Wang Lixia,Deng	家畜生态学报	Journal of Livestock Ecology	41	1	37-41	2020	sn.1673-1182.2020.01.008
6534	鼠伤寒沙门菌病原学与P	typhimurium in Minhang	秀华,刘芸,张颖华,王小光	u Yun,Zhang Yinghua,Wang	中国卫生检验杂志	ese Journal of Health Inspe	30	2	136-140	2020	-
6535	病病例实验室检测结果分	esistance monitoring resu	爱霞,申艳琴,吴燕,曹莉	Aixia,Shen Yanqin,Wu Yan,	中国卫生检验杂志	ese Journal of Health Inspe	30	2	235-238	2020	-
6536	18年其他感染性腹泻流行	e epidemic characteristics	彭,汪慧,陈纯,王大虎,李美	g Hui,Chen Chun,Wang Da	医学动物防制	medical animal control	36	1	32-35	2020	0.7629/yxdwfz202001010
6537	州市网络外卖配送餐微生	atus of online takeout deliv	维蔚,李迎月,林晓华,黄	Veiwei,Li Yingyue,Lin Xiaoh	环境与职业医学	mental and Occupational M	37	1	57-62	2020	13/j.cnki.jeom.2020.19530
6538	苏省学校食物中毒事件流	characteristics of school f	宗雯琦,吴雨晨,戴月.	ng Wenqi,Wu Yuchen,Dai Y	食品安全质量检测学报	f Food Safety and Quality I	11	2	655-659	2020	-
6539	心医院50例腹泻儿童鼠伤	inical analysis of Salmonell	马志平,陈瑶.	Ma Zhiping,Chen Yao.	广州医药	Guangzhou Medicine	51	1	66-69	2020	sn.1000-8535.2020.01.015
6540	源沙门菌的分离鉴定及药	susceptibility test of Salm	变华,贾刘数,吕一格,王智	ua,Jia Liushu,Lu Yige,Wang	动物医学进展	vances in Veterinary Medic	41	1	125-130	2020	sn.1007-5038.2020.01.024
6541	检验中应用大便常规检验	e stool examination in sea	栗艳芳.	Li Yanfang.	医学信息文摘（连续型电	information abstract (cont	20	6	175-176	2020	sn.1671-3141.2020.06.090
6542	菌临床检验方法及检验结	l testing methods and anal	张玉梅.	Zhang Yumei.	医学信息文摘（连续型电	information abstract (cont	20	4	176178	2020	nki.1671-3141.2020.4.113
6543	规检验在季节性腹泻诊治	ination in the diagnosis an	徐芳芫.	Xu Fangqian.	中国医药指南	Chinese Medicine Guide	18	1	35	2020	-
6544	年句容市食源性疾病监测	orne disease surveillance r	,岁源,曾萍,曹俊,刘敏,吴	an,Zeng Ping,Cao Jun,Liu M	现代预防医学	modern preventive medicin	47	1	132-135	2020	-
6545	城区菜市场熟食中致病菌	nic bacteria in cooked foo	刘培培.	Liu Peipei.	医学信息	medical information	33	1	130-132	2020	sn.1006-1959.2020.01.040
6546	门菌食物中毒的病原学检	the etiology of Salmonella	娟,洪敏丽,刘渠,甘莉萍,李	,Hong Minli,Liu Qu,Gan Lip	现代预防医学	modern preventive medicin	47	1	123-126	2020	-
6547	门氏菌引起化脓性脑膜炎	itis caused by Salmonella t	杨乐,李静.	Yang Le,Li Jing.	医药前沿	Medical Frontier	10	1	252-253	2020	-

6548	津市河北区食源性疾病主	illance results of foodborne	黎珊珊,李莎涛,杨正宇.	anshan,Li Shatao,Yang Zhe	河南预防医学杂志	n Journal of Preventive Me	31	2	128-130,133	2020	m.1006-8414.2020.02.016
6549	建省食源性疾病沙门氏菌	illance Situation of Foodbo	伟伟,傅祎欣,叶玲清,李闽	wei,Fu Yixin,Ye Lingqing,Li	中国人兽共患病学报	Chinese Journal of Zoonose	36	3	223-228	2020	sn.1002-2694.2019.00.146
6550	金属和微生物含量检测及	icrobial content in crayfish	伟,杨章力,曾云想,刘国强	Zhangli,Zeng Yunxiang,Liu	分析仪器	Analytical Instruments	-	2	61-69	2020	sn.1001-232x.2020.02.013
6551	生食物中毒的微生物学检	iological examination of ba	张正丽.	Zhang Zhengli.	自我保健	self care	-	22	99-100	2020	-
6552	年郑州大学附属儿童医院	nonella in Children's Hospit	俊文,贾静,阙蔚鹏,杨俊梅	ven,Jia Jing,Que Weipeng,Y	中华医院感染学杂志	ournal of Hospital Infectiou	30	1	120-124	2020	.11816/cn.ni.2020-186775
6553	验在季节性腹泻检验中的	of routine stool examinatio	吴丽霞.	Wu Lixia.	中外医疗	Chinese and foreign medica	39	1	183-185	2020	ki.1674-0742.2020.01.183
6554	性腹泻的病原微生物检验	st results and significance o	刘苹.	Liu Ping.	健康管理	health management	-	16	133	2020	-
6555	海市宝山区食源性疾病病	ease pathogen surveillance	利,何平,沈先标,杨兴堂,沈	Shen Xianbiao,Yang Xingta	职业与健康	Occupation and health	36	1	38-41	2020	-
6556	品沙门氏菌生化型和血清	e analysis of Salmonella in	马丽萍,张素艳,蒋健.	Liping,Zhang Suyan,Jiang J	浙江畜牧兽医	mal Husbandry and Veterin	45	1	40-41	2020	-
6557	的优势血清型沙门菌耐药	of dominant Salmonella se	谭冬梅,瞿聪,梁炎,高海惠	mei,Qu Cong,Liang Yan,Ga	中国卫生检验杂志	ese Journal of Health Inspe	30	1	116-119	2020	-
6558	感染非伤寒沙门菌分子分	yphoid Salmonella infectio	谭冬梅,刘光武,瞿聪,谭琳	gmei,Liu Guangwu,Qu Con	职业与健康	Occupation and health	36	4	470-473	2020	-
6559	食物中毒的微生物学检验	iological examination of ba	陈帆.	Chen Fan.	探索科学	Explore Science	-	1	239	2020	-
6560	年肉与肉制品中食源性致	ction results of foodborne	曹铁红,刘桂华.	Cao Tiehong,Liu Guihua.	中国卫生工程学	Chinese sanitary engineerin	19	1	39-41	2020	-
6561	南州某县一起伤寒暴发的	er outbreak in a county i	黄荷;李梦;姚光海;	ng He;Li Meng;Yao Guan	海峡预防医学杂志	Journal of Preventive M	26	4	42-44	2020	-
6562	伤寒暴发菌株的基因组遗	sistance characteristics o	;李玲;刘晓骏;葛以跃;赵	g;Liu Xiaojun;Ge Yiyue;Z	江苏预防医学	angsu Preventive Medici	31	4	371-374	2020	sn.1006-9070.2020.04.006
6563	沙门菌感染临床特征及药	g susceptibility results o	祥英;张桂花;王开梅;何	;Zhang Guihua;Wang K	医药论坛杂志	Medical Forum Magazine	42	23	82-85	2021	-
6564	伤寒沙门菌耐药性及耐药	nd drug-resistant genes	水;王越;郎兴莹;司虹;薄志	g Yue;Lang Xingying;Si H	中国微生态学杂志	nese Journal of Microeco	33	12	1403-1405+1412	2021	381/j.cnki.cjm.202112007
6565	北地区鸡源沙门菌流行病	g resistance analysis of	尧;何云凤;李蕴玉;李佩国	He Yunfeng;Li Yunyu;Li P	中国预防兽医学报	rnal of Preventive Veterin	43	9	924-929	2021	-
6566	非伤寒沙门菌临床分离株	e and molecular typing o	李世军;王丹;黄俊飞;童毅	g Dan;Huang Junfei;Tong	中国人兽共患病学报	hinese Journal of Zoonos	37	7	603-610	2021	-
6567	伤寒沙门菌MLVA分型流行	inceMLVAAnalysis of po	;李可维;郭丰;石奔;杨修	ewei;Guo Feng;Shi Ben;	中国实验诊断学	ese experimental diagno	25	5	737-740	2021	-
6568	源鼠伤寒沙门氏菌的分离	of Salmonella typhimuriu	伟东;刘琪;梁瑞英;贾亚	g;Liu Qi;Liang Ruiying;Ji	动物医学进展	ances in Veterinary Medi	42	2	122-127	2021	ki.1007-5038.2021.02.023
6569	菌脑膜炎3例临床分析并	dren3Clinical analysis of	春珍;周明明;汪洪姣;谢永	Mingming;Wang Hongjia	临床儿科杂志	ournal of Clinical Pediatri	39	12	934-937	2021	-
6570	腹泻沙门氏菌血清型、耐	molecular typing of Sal	-	-	现代预防医学	dern preventive medici	-	-	-	2021	-
6571	区食源性疾病致病菌监测	oodborne disease patho	-	-	预防医学	Preventive Medicine	-	-	-	2021	-
6572	绵阳市食品安全风险监测	ety risk monitoring re	-	-	职业与健康	Occupation and health	-	-	-	2021	-
6573	花市沙门菌临床分离株血	istance analysis of Salmone	-	-	医学动物防制	medical animal control	-	-	-	2021	-
6574	省开封市0～5岁儿童食源	ovince0~5Foodborne disea	-	-	河南预防医学杂志	n Journal of Preventive Me	-	-	-	2021	-
6575	湖南省食源性沙门菌流行	ogical characteristics of foo	-	-	实用预防医学	ractical preventive medicin	-	-	-	2021	-
6576	18—2020年多中心临床分	resistance of clinical isolate	-	-	中国抗生素杂志	hinese Journal of Antibioti	-	-	-	2021	-
6577	染性腹泻病原菌监测及耐	lysis of pathogenic bacteri	-	-	疾病预防控制通报	se Prevention and Control	-	-	-	2021	-
6578	菌临床分离株血清型和分	ing characteristics of clinic	-	-	检验医学	laboratory medicine	-	-	-	2021	-
6579	省沙门氏菌PFGE分子分型	ityPFGEResearch on mole	-	-	中国人兽共患病学报	Chinese Journal of Zoonose	-	-	-	2021	sn.1002-2694.2021.00.130
6580	猪腹泻源沙门氏菌流行病	alysis of Salmonella origina	建新,左文兵,刘小娟,王	o Wenbing,Liu Xiaojuan,W	养猪	pig raising	-	2	105-108	2021	sn.1002-1957.2021.02.035
6581	三级医院感染性腹泻的病	e results of infectious diarr	卫华,周龙珠,曹新梅,黄俐	Zhou Longzhu,Cao Xinmei,	海南医学	Hainan Medicine	32	7	940-943	2021	sn.1003-6350.2021.07.031
6582	菌性腹泻的病原微生物检	hogenic microbial testing i	韩振龙,李红岩,宋英杰.	henlong,Li Hongyan,Song Y	医学检验与临床	edical Laboratory and Clini	32	3	55-56,9	2021	sn.1673-5013.2021.03.015
6583	氏菌骨髓炎伴化脓性关节	nella osteomyelitis accomp	文晶,王志芳,韩冬,杨变	Wang Zhifang,Han Dong,Ya	中国临床新医学	hinese new clinical medici	14	3	307-309	2021	ssn.1674-3806.2021.03.18
6584	微生物污染状况调查及大	dishes in Guangzhou and s	张娟,黄宝莹,赵玲,杨纯佳	n,Huang Baoying,Zhao Ling	食品安全质量检测学报	f Food Safety and Quality I	12	6	2485-2490	2021	-

6585	门氏菌食物中毒分离株的	cteristics of a London Salm	陶霞,王安娜,周勇,和鹏,吴	,Anna Wang,Zhou Yong,He	食品安全质量检测学报	f Food Safety and Quality I	12	6	2474-2478	2021	-
6586	检测中实时荧光定量PCR	in food Salmonella detecti	林桂英.	Lin Guiying.	保健文汇	Health Wenhui	22	9	204-205	2021	-
6587	畜禽生鲜肉沙门氏菌分离	nella from fresh livestock a	邵定乾,罗瑛,洪枫,满初日	ian,Luo Ying,Hong Feng,Ma	热带生物学报	Acta Tropical Biology	12	1	103-109	2021	-
6588	罗县重点品种食品安全微	monitoring results of key va	骆玉萍,陈晓能,李锡康.	Yuping,Chen Xiaoneng,Li Xi	食品安全导刊	food safety guide	-	9	106-107,111	2021	-
6589	人急性感染性腹泻常见致	of common pathogenic ba	詹永婧,李全瑞,田耕.	Yongjing,Li Quanrui,Tian G	中国病案	Chinese medical records	22	3	99-103	2021	sn.1672-2566.2021.03.034
6590	食物中毒病原学检测及临	n and clinical analysis of ba	于洁.	Yu Jie.	实用检验医师杂志	of Practical Laboratory Ph	13	1	22-24	2021	sn.1674-7151.2021.01.008
6591	猪肉源伦敦沙门菌的流行	monella London originating	茜,张丽娜,符颖,张建民,廖	Lina,Fu Ying,Zhang Jianmi	畜牧兽医学报	mal Husbandry and Veteri	52	2	488-497	2021	sn.0366-6964.2021.02.021
6592	测网2014-2019年粪便标本	rk2014-2019Annual stool s	全国细菌耐药监测网.	cterial Resistance Monitori	中国感染控制杂志	ese Journal of Infection Co	20	2	168-174	2021	.issn.1671-9638.20216175
6593	新疆动物源鼠伤寒沙门菌	ce of Salmonella typhimuri	强,刘雪连,宋超慧,徐琦琦,	ang,Liu Xuelian,Song Chaoh	中国农业大学学报	al of China Agricultural Univ	26	2	88-97	2021	ssn.1007-4333.2021.02.11
6594	年武汉市监测食源性疾病	mic characteristics of food	月,曾敬,李敏,梁韡斌,郭建	eng Jing,Li Min,Liang Weib	中华医院感染学杂志	ournal of Hospital Infectiou	31	4	631-635	2021	.11816/cn.ni.2021-201951
6595	潍坊市生畜禽肉食源性致	orne pathogenic bacteria i	艳,张辉,赵忠俊,王晓威,梁	ui,Zhao Zhongjun,Wang Xi	食品安全导刊	food safety guide	-	6	103-104	2021	-
6596	腹泻病原微生物的临床检	alysis of pathogenic micro	林仁贵,谢琳峰.	Lin Rengui,Xie Linfeng.	中外医疗	Chinese and foreign medica	40	6	186-188	2021	ki.1674-0742.2021.06.186
6597	院食源性疾病病原学监测	lance results of foodborne	姝中,魏小萍,孟灵,郭文琴	,Wei Xiaoping,Meng Ling,	中国公共卫生管理	ese public health managen	37	1	102-105	2021	nki.23-1318.2021.01.0026
6598	区多重耐药沙门氏菌的耐	ics of multidrug-resistant S	玮,汪皓秋,俞骅,潘劲草,陈	ang Haoqiu,Yu Hua,Pan Jir	微生物学通报	Bulletin of Microbiology	48	2	536-544	2021	4/j.microbiol.china.200151
6599	屠宰场猪肉中3种食源性致	se pork3Detection of food	马广宇,丁立,邓森荣,魏琦	,Ding Li,Deng Senrong,We	动物医学进展	vances in Veterinary Medic	42	2	50-54	2021	sn.1007-5038.2021.02.009
6600	伤寒沙门菌肠炎的临床特	nd drug resistance of Salm	邵莉,林乐欣,李路冷,廖素	,Lin Lexin,Li Luleng,Liao Su	中国卫生标准管理	a Health Standard Manage	12	3	7-10	2021	sn.1674-9316.2021.03.003
6601	细菌性食物中毒的检验方	's testing methods for bac	戴晴.	Dai Qing.	中国保健营养	Chinese health nutrition	31	5	271	2021	-
6602	猪沙门氏菌的分离鉴定及	y analysis of Salmonella or	蔺俐仲,杨源,刘艳,景书灏	zhong,Yang Yuan,Liu Yan,Ji	养猪	pig raising	-	1	95-97	2021	sn.1002-1957.2021.01.031
6603	阳市食品中食源性致病菌	pathogenic bacteria in foo	高颖.	Gao Ying.	基层医学论坛	Primary Medicine Forum	25	4	581-582	2021	5/j.1672-1721.2021.04.066
6604	宴食物中毒调查分析及防	ing at rural family banque	林亚娜.	Lin Yana.	中国继续医学教育	a continuing medical educ	13	3	89-92	2021	sn.1674-9308.2021.03.026
6605	校园周边小食品的微生物	robial contamination of sn	任泓燕.	Ren Hongyan.	微量元素与健康研究	e elements and health rese	38	1	39-41	2021	-
6606	炎沙门菌引起的食物中毒	case of food poisoning ca	艳,张栗,滕毅,王玲,刘朝发	g Li,Teng Yi,Wang Ling,Liu	预防医学情报杂志	of Preventive Medicine Info	37	1	48-52,57	2021	-
6607	百日鸡群沙门菌的鉴定及	eristics of Salmonella from	顾小雪,蒋智宇,朱晨,杨杰,	e,Jiang Zhiyu,Zhu Chen,Yar	中国预防兽医学报	rnal of Preventive Veterina	43	1	21-28	2021	ssn.1008-0589.202003030
6608	耐药性及其耐消毒剂基因	rug resistance and its disin	,黄校樑,何杏欣,罗侠平,	aoliang,He Xingxin,Luo Xiap	热带医学杂志	journal of tropical medicine	21	1	40-43	2021	sn.1672-3619.2021.01.010
6609	7年广西食源性疾病暴发	foodborne disease outbre	银品,钟延旭,杨永鹏,蒙浩	g Yanxu,Yang Yongpeng,M	广西医科大学学报	al of Guangxi Medical Univ	38	1	178-182	2021	nki.45-1211/r.2021.01.029
6610	其他细菌性感染性腹泻临	cteristics of other bacteria	莉,胡明,刘悦,何中华,沈	ng,Liu Yue,He Zhonghua,Sh	现代消化及介入诊疗	and interventional diagno	26	1	32-36	2021	sn.1672-2159.2021.01.007
6611	山市食品和公共场所从业	sults of employees working	张晓琦,王延峰,岳伟玲.	Xiaoqi,Wang Yanfeng,Yue W	河南预防医学杂志	n Journal of Preventive Me	32	1	87-89	2021	m.1006-8414.2021.01.029
6612	增法检测蔬菜产地环境	tion of Salmonella in envir	新,刘兰英,陈丽华,李玥	Liu Lanying,Chen Lihua,Li Y	食品安全质量检测学报	f Food Safety and Quality I	12	2	492-498	2021	-
6613	2017年山西省老年病人疾	e disease spectrum of elde	高华,韩颖,张佳莹,左京骅	Han Ying,Zhang Jiaying,Zuo	护理研究	nursing research	35	2	319-324	2021	sn.1009-6493.2021.02.025
6614	检测中荧光定量PCR技术	al microbiological testingP	张月玲,马涛,庞世超,郭改玲	ing,Ma Tao,Pang Shichao,G	航空航天医学杂志	urnal of Aerospace Medici	32	1	58-59	2021	sn.2095-1434.2021.01.035
6615	谱技术检测不同乳制品常	r detection of common foo	刘玉林,刘秀,尹建军,宋全	Liu Xiu,Yin Jianjun,Song Qu	食品安全质量检测学报	f Food Safety and Quality I	12	2	546-551	2021	-
6616	沙门氏菌分子检测方法的	ar detection methods for S	连海,董彬,赵婷婷,周琦,张	ong Bin,Zhao Tingting,Zhou	食品安全质量检测学报	f Food Safety and Quality I	12	2	576-583	2021	-
6617	肠炎沙门氏菌的分离鉴定	haracteristics of Salmonella	亚,常志顺,李珂,覃袖伟,	hishun,Li Ke,Qin Xiuwei,Zh	微生物学通报	Bulletin of Microbiology	48	1	82-92	2021	4/j.microbiol.china.200329
6618	8年急性传染性腹泻致病	of pathogenic bacteria in a	邵婷婷.	Shao Tingting.	中国卫生检验杂志	ese Journal of Health Inspe	31	1	124-128	2021	-
6619	康状态和饲养环境中宠物	nella from pets in different	凯,吾买尔江•牙合甫,高	Kai,Wumaierjiang•Yahefu	畜牧与兽医	Husbandry and Veterinary	53	1	106-112	2021	-
6620	区食源性沙门氏菌耐药特	haracteristics of foodborn	鸣秋,刘艳,李诗瑶,罗彤,付	gqiu,Liu Yan,Li Shiyao,Luo T	食品安全质量检测学报	f Food Safety and Quality I	12	1	78-85	2021	-
6621	腹泻的病原微生物检验结	of pathogenic microorganis	薛顺虹.	Xue Shunhong.	健康之友	health friend	-	1	116	2021	-

6622	儿童胸腔积液的病因和临床	al characteristics of pleural	张蘅辞,邹培远,朱晓萍.	Hengci,Zou Peiyuan,Zhu Xia	贵州医科大学学报	al of Guizhou Medical Univ	46	8	937-942	2021	ki.2096-8388.2021.08.011
6623	业从业人员传染病现状调	nt situation of infectious di	于强.	Yu Qiang.	食品安全导刊	food safety guide	-	15	5-6	2021	-
6624	沙门氏菌和链球菌感染情	ella and Streptococcus infe	王大春,李庆丰.	Wang Dachun,Li Qingfeng.	养猪	pig raising	-	3	121-123	2021	sn.1002-1957.2021.03.042
6625	绞股蓝的微生物污染调查及	mination of Gynostemma	陈玉珍,赵子冰,胡翮.	en Yuzhen,Zhao Zibing,Hu	中国药品标准	Chinese drug standards	22	5	439-445	2021	0.19778/j.chp.2021.05.009
6626	染性腹泻患儿的临床特点及	pathogens of children with	夏荣钧.	Xia Rongjun.	家庭生活指南	Family Living Guide	37	10	113-114	2021	-
6627	性感染性腹泻病原菌及其	s diarrhea in outpatient cli	张文胜,李道平,黄浩,吴娜	nsheng,Li Daoping,Huang H	中华医院感染学杂志	ournal of Hospital Infectiou	31	15	2241-2245	2021	.11816/cn.ni.2021-203224
6628	牛源沙门菌的分离鉴定及耐	esistance analysis of Salmo	吴丹,卢姊豪,尹力鸿,吕家	hant,Lu Sisterhao,Yin Lihon	中国兽医学报	al of Chinese Veterinary Me	41	10	1976-1982	2021	nki.1005-4545.2021.10.15
6629	寒性脊柱炎误诊胸椎结核	misdiagnosed as thoracic tu	,张礼仁,刘家明,张志宏,文	g Liren,Liu Jiaming,Zhang Z	南昌大学学报（医学版）	Nanchang University (Medi	61	4	100-102	2021	4/j.cnki.ncdm.2021.04.023
6630	015-2019年食源性疾病病	nalysis of annual foodborne	婧,韩喜荣,韩思媛,施丽飞	ng,Han Xirong,Han Siyuan,	海峡预防医学杂志	s Journal of Preventive Me	27	3	54-56	2021	-
6631	染性腹主动脉瘤的诊疗体	experience of infected ab	小岩,吴学君,张十一,孔祥	u Xuejun,Zhang Shiyi,Kong	河北医科大学学报	nal of Hebei Medical Unive	42	8	959-962	2021	sn.1007-3205.2021.08.020
6632	宁堡沙门菌食源性疾病暴	almonella Orenenburg foo	王丁丁,梅建华,陈秀英,王	ngding,Mei Jianhua,Chen X	预防医学	Preventive Medicine	33	6	602-606	2021	ssn2096-5087.2021.06.015
6633	州市食源性疾病流行特征分	haracteristics of foodborne	,高四海,蔡圆圆,山若青,蔡	ai Yuanyuan,Mountains are	预防医学	Preventive Medicine	33	3	306-308	2021	ssn2096-5087.2021.03.024
6634	引起食源性聚集性病例的溯	ceability of a food-borne c	5,许海燕,赵哲,茅丽婷,熊	Haiyan,Zhao Zhe,Mao Liting	医学动物防制	medical animal control	37	7	621-623,627	2021	0.7629/yxdwfz202107003
6635	四川省阿贡纳沙门菌分子分	resistance analysis of Salm	忧,刘丽,雷高鹏,黄玉兰,黄	,Lei Gaopeng,Magnolia,Hua	中国食品卫生杂志	hinese Food Hygiene Journ	33	5	553-558	2021	0.13590/j.cjfh.2021.05.006
6636	[5],12:i:-沙门菌和鼠伤寒沙	dentification and molecula	安戈,李羿,赵雪蕾,张晓蕾,	Yi,Zhao Xuelei,Zhang Xiao	预防医学	Preventive Medicine	33	8	797-800	2021	ssn2096-5087.2021.08.010
6637	微生物性食源性疾病暴发	s of microbial foodborne di	徐粒子,孟灿,李卫东.	particles,Meng Can,Li Weid	现代预防医学	modern preventive medicin	48	6	1007-1011	2021	-
6638	生物检验在儿童腹泻诊断中	icrobial testing in the diagn	杨毅军.	Yang Yijun.	医学美学美容	edical Aesthetics and Beau	30	11	78	2021	-
6639	沙门菌食物中毒事件的病	and traceability of Salmon	春,苏良,刘晓蕾,杨柳青,袁	,Su Liang,Liu Xiaolei,Yangli	现代预防医学	modern preventive medicin	48	6	1103-1106	2021	-
6640	省沙门菌血清型、分子分	olecular typing and drug re	张眉眉,耿英芝,张铭琰,范	mei,Geng Yingzhi,Zhang M	中国微生态学杂志	inese Journal of Microecolo	33	5	538-541	2021	381/j.cnki.cjm.202105008
6641	门菌食物中毒的流行病学	ility analysis of Salmonella	华,夏威,施惠军,陆逊,王	arvey,Shi Huijun,Lu Xun,Wa	医学动物防制	medical animal control	37	2	175-178,182	2021	0.7629/yxdwfz202102020
6642	武清区餐饮食品食源性主	genic bacteria contaminati	盛楠.	Sheng Nan.	寄生虫病与感染性疾病	ic diseases and infectious d	19	3	113-115,119	2021	-
6643	起肠炎沙门菌食源性疾病	of Salmonella Enteritidis f	强,徐云龙,宋士利,郑双来,	Yunlong,Song Shili,Zheng S	中国食品卫生杂志	hinese Food Hygiene Journ	33	5	583-586	2021	0.13590/j.cjfh.2021.05.011
6644	泻患者沙门菌流行病学特	e analysis of Salmonella in	余玮,孙攀,陈培超,高肖芳	un Pan,Chen Peichao,Gao	上海预防医学	hanghai Preventive Medicin	33	10	929-934	2021	28/j.cnki.sjpm.2021.20536
6645	区餐饮具消毒效果分析与	e disinfection effect of tabl	朱玉娇,李微微.	Zhu Yujiao,Li Weiwei.	食品安全导刊	food safety guide	-	20	42-43	2021	-
6646	应用于一起肠炎沙门菌引起	sis in investigation of food	,贾肇一,孙汝春,刘晴,王	aoyi,Sun Ruchun,Liu Qing,W	医学动物防制	medical animal control	37	2	149-151	2021	0.7629/yxdwfz202102013
6647	沙门菌食物中毒事件的流行	of an incident of Salmone	,张金金,许玉成,张桂欣,周	hang Jinjin,Xu Yucheng,Zha	医学动物防制	medical animal control	37	1	23-26	2021	0.7629/yxdwfz202101006
6648	区儿童非伤寒沙门菌感染	stics of non-typhoid Salmo	刘瑜华,张又祥,胡家奇.	Yuhua,Zhang Youxiang,Hu J	广东医学	Guangdong Medicine	42	8	936-939	2021	820/j.cnki.gdyx.20200787
6649	泻患儿粪便标本中主要致	nce of major pathogenic ba	刘绍辉.	Liu Shaohui.	抗感染药学	Anti-infective Pharmacy	18	5	689-691	2021	sn.1672-7878.2021.05-017
6650	-2018年二七区其他感染性	8Other infectious diarrhea	孙花荣.	Sun Huarong.	医药论坛杂志	Medical Forum Magazine	42	2	79-82	2021	-
6651	引起的沙门菌食源性疾病	dborne disease outbreak ca	牛蓓,田美娜,陈磊,罗晓燕	Tian Mina,Chen Lei,Luo Xia	医学动物防制	medical animal control	37	1	88-91	2021	0.7629/yxdwfz202101025
6652	鸭主要细菌病调查与耐药	analysis of major bacterial	锋,樊祐卿,王文秀,朱峰亮,	Huqing,Wang Wenxiu,Zhu	家禽科学	poultry science	-	9	52-56	2021	sn.1673-1085.2021.09.019
6653	在儿童急性细菌性脑膜炎	mic sequencing in children	师,查剑,孙小兰,章静静,陈	a Jian,Sun Xiaolan,Zhang Ji	中华实用儿科临床杂志	Clinical Journal of Practical	36	13	1007-1010	2021	n101070-20200217-00180
6654	氏菌分型鉴定及常用抗生素	from Tibetan chickens and	旺马泽郎,黄家旗,李霞,赵	a Zelang,huangjiaqi,Li Xia,Z	高原农业	plateau agriculture	5	3	272-278	2021	707/j.cnki.jpa.2021.03.010
6655	7年熟肉制品生产加工过程	of pathogenic bacteria in th	梅,乔昕,倪云龙,田亭,郑	ao Xin,Ni Yunlong,Tian Tin	现代预防医学	modern preventive medicin	48	18	3430-3433	2021	-
6656	性食物中毒微生物检验结果	bial test results for bacteri	崔宪.	Cui Xian.	首都食品与医药	Capital Food and Medicine	28	16	116-118	2021	sn.1005-8257.2021.16.063
6657	儿粪便标本病原微生物检验	nic microorganisms in fecal	王晓静.	Wang Xiaojing.	河南医学研究	Henan Medical Research	30	26	4963-4966	2021	n.1004-437X.2021.26.051
6658	林市饮服从业人员沙门菌	typing of Salmonella among	罗铭,蒋宁,张耀平,陈小梅	g Ning,Zhang Yaoping,Che	热带医学杂志	journal of tropical medicine	21	6	797-799,806	2021	sn.1672-3619.2021.06.029

6659	医疗机构污水消毒效果监	water Disinfection Effect of	],王琼妹,陈小佳,李琼梅,剑	Qiongmei,Chen Xiaojia,Li Qi	中国消毒学杂志	hinese Journal of Disinfecti	38	6	472-473	2021	sn.1001-7658.2021.06.024
6660	从业人员沙门菌携带情况	employees in some public	职建军.	ocational Construction Arm	临床研究	clinical research	29	9	149-150	2021	-
6661	检验在季节性腹泻检验中	outine Stool Testing in Seas	刂利鹏,洪岩,史文凤,黄少平	Hongyan,Shi Wenfeng,Huan	特别健康	Very healthy	-	20	141-142	2021	-
6662	菌性食物中毒的致病菌分	pathogenic bacteria in a ca	李海遥,郑姝豪,黄渊,肖礼	),Zheng Shuhao,Huang Yua	饮食保健	Diet and health care	-	24	258	2021	-
6663	少门氏菌的分离鉴定及耐ž	istance analysis of Salmon	雷,刘欣,刘立英,侯春雪,万	u Xin,Liu Liying,Hou Chunx	现代畜牧兽医	mal husbandry and veterina	-	6	72-74	2021	-
6664	验在临床诊断季节性腹泻中	stool examination in clinica	李大伟.	Li Dawei.	中国保健营养	Chinese health nutrition	31	18	288	2021	-
6665	省其他感染性腹泻病病原	characteristics of other infe	军,王学文,马斌忠,曹海兰,	g Xuewen,Ma Binzhong,Ca	现代预防医学	modern preventive medicin	48	17	3073-3076	2021	-
6666	售生食动物性水产品食源	e pathogenic bacteria in ra	刘于飞,张维蔚,李燕,周琴,	,Zhang Weiwei,Li Yan,Zhou	食品安全质量检测学报	f Food Safety and Quality I	12	8	3113-3117	2021	-
6667	验在感染控制中的应用及	ng in infection control and	李晓彤.	Li Xiaotong.	中国城乡企业卫生	ygiene in urban and rural a	36	7	96-98	2021	5/j.1003-5052.2021.07.036
6668	滨区细菌性食物中毒的粪	teria in fecal samples cause	乔春晓,张欣.	Qiao Chunxiao,Zhang Xin.	临床研究	clinical research	29	9	147-149	2021	-
6669	猪肉中沙门菌定量检测方	contamination risk of Salm	,凌秀梅,李莹,李瑶,崔生	g Xiumei,Li Ying,Li Yao,Cui	中华预防医学杂志	se Journal of Preventive Me	55	8	999-1005	2021	n112150-20210302-00209
6670	起伤寒疫情的病原特征及	molecular typing of a typh	姣,胡晓,宋威蓉,张白帆,叶	iao,Song Weirong,Zhang Ba	河南预防医学杂志	n Journal of Preventive Me	32	6	413-415,432	2021	m.1006-8414.2021.06.004
6671	温核酸扩增方法快速检测	ic acid amplification metho	邓雨,刘常宏,卜元卿,薛雅蓉	Changhong,Bu Yuanqing,X	微生物学通报	Bulletin of Microbiology	48	3	1041-1047	2021	4/j.microbiol.china.200410
6672	禽肉中沙门菌污染状况及	tus and pathogenic charac	,李闽真,洪锦春,郑盈翔,陈	,Hong Jinchun,Zheng Yingx	中国食品卫生杂志	hinese Food Hygiene Journ	33	5	587-590	2021	).13590/j.cjfh.2021.05.012
6673	直腹泻大肠埃希菌的分离	nalysis of Escherichia coli c	,纪雪,梁冰,姜博文,孙洋,刘	Liang Bing,Jiang Bowen,Sun	中国病原生物学杂志	ese Journal of Pathogen Bio	16	3	305-309	2021	10.13350/j.cjpb.210313
6674	某学校一起沙门菌食物中毒	food poisoning incident in	沈惠平,潘丽峰,胡卉,秦存	uiping,Pan Lifeng,Hu Hui,Q	中国食品卫生杂志	hinese Food Hygiene Journ	33	5	627-632	2021	).13590/j.cjfh.2021.05.021
6675	宿州市食品微生物安全风	icrobial safety risk monitor	胡雪影.	Hu Xueying.	职业与健康	Occupation and health	37	11	1552-1555	2021	-
6676	检测结果,分析其临床微	ildren,Analysis and discuss	陈培幸,刘卫平.	Chen Peixing,Liu Weiping.	特别健康	Very healthy	-	19	93-94	2021	-
6677	河大骨鸡沙门菌病原分离、	nella pathogen from Zhuan	张日欣,范强,贺永明,俞美子	in,Fan Qiang,He Yongming	中国兽医杂志	Chinese Veterinary Journal	57	2	78-81	2021	-
6678	菌的血清型分布、分子分型	ng characteristics and drug	石红,王毅.	Shi Hong,Wang Yi.	海南医学	Hainan Medicine	32	18	2392-2394	2021	sn.1003-6350.2021.18.022
6679	沙门菌病人分离株的耐药	nd molecular typing of Salr	琴超,陈金堃,陈吉铭,何琴	ao,Chen Jinkun,Chen Jimin	中国食品卫生杂志	hinese Food Hygiene Journ	33	5	542-548	2021	).13590/j.cjfh.2021.05.004
6680	菌1,4,[5],12 ：i：-的耐药性	,4,[5],12：i：-Analysis of	霞,李柏生,何冬梅,刘哲,李	aisheng,He Dongmei,Liu Zh	疾病监测	disease surveillance	36	5	501-508	2021	).3784/jbjc.202012020405
6681	医院2016-2018年食源性疾	-2018Analysis of annual fo	萍,施菊萍,吴鹏程,樊飞,徐	i Juping,Wu Pengcheng,Fa	医学动物防制	medical animal control	37	1	64-67	2021	0.7629/yxdwfz202101018
6682	性沙门氏菌的血清和耐药	ug resistance levels of food	屠博文,薛银刚,赵莹,黎俊	en,Xue Yingang,Zhao Ying,L	公共卫生与预防医学	Health and Preventive Me	32	3	63-67	2021	sn.1006-2483.2021.03.015
6683	5起食用"猪活血"中毒事件	blood circulation"Investig	林玫,蒋玉艳,石萌萌,苏奕	ng Yuyan,Shi Mengmeng,S	应用预防医学	applied preventive medicin	27	3	225-226,229	2021	n.1673-758X.2021.03.012
6684	性腹泻患儿病原微生物分	of pathogenic microorganis	曹玉红.	Cao Yuhong.	深圳中西医结合杂志	egrated Traditional Chinese	31	1	58-59	2021	ki.1007-0893.2021.01.026
6685	售食用螺类中食源性致病	dborne pathogenic bacteria	张濛,李艳芬,吴玲玲,邱正勇	Yanfen,Wu Lingling,Qiu Zh	河南预防医学杂志	n Journal of Preventive Me	32	9	666-668	2021	m.1006-8414.2021.09.006
6686	8年-2019年沙门菌血清型	19Salmonella serotype and	张柱梁,刘敏,邹智敏,张毓	iang,Liu Min,Zou Zhimin,Zh	中国卫生检验杂志	ese Journal of Health Inspe	31	3	311-313,317	2021	-
6687	粪便标本病原微生物分布	ogenic microorganisms in	黄英英.	Huang Yingying.	药品评价	drug evaluation	18	12	747-750	2021	nki.1672-2809.2021.12.14
6688	门氏菌MLST分型及毒力	tryMLSTTyping and virule	晖,柳明,李小林,万晓楠,	iu Ming,Li Xiaolin,Wan Xiao	食品研究与开发	od research and developme	42	13	174-179	2021	sn.1005-6521.2021.13.026
6689	门氏菌携带状况、耐药性	ug resistance and drug res	,王鑫盛,郝勇航,王亚宾,	heng,Hao Yonghang,Wang	食品安全质量检测学报	f Food Safety and Quality I	12	8	3034-3040	2021	-
6690	2019年小儿感染性腹泻细	anges in bacterial spectrum	冯莉茹,赵青.	Feng Liru,Zhao Qing.	实用临床医药杂志	nal of Practical Clinical Med	25	16	45-48,53	2021	10.7619/jcmp.20211991
6691	市市售保健食品微生物学	ts and analysis of commer	杰,王珏,陈卓,杨灿,徐璐,郝	ue,Chen Zhuo,Yang Can,Xu	中国食品卫生杂志	hinese Food Hygiene Journ	33	2	170-174	2021	).13590/j.cjfh.2021.02.009
6692	市感染性腹泻细菌病原谱	spectrum and epidemic ch	吕冰,张新,严寒秋,贾蕾,王	ang Xin,severe cold autum	中国预防医学杂志	se Journal of Preventive Me	22	2	93-97	2021	5/j.1009-6639.2021.02.003
6693	患者粪便沙门菌血清学分	bility of Salmonella in feces	冯文南,黄丽庆,吴浪波.	Wennan,Huang Liqing,Wu L	医学食疗与健康	edical Diet Therapy and Hea	19	14	230-231	2021	-
6694	木齐宠物源常见病原菌的	f common pathogenic bact	几,王舒丰,姚晓慧,马木尔·	Wang Shufeng,Yao Xiaohui,	中国兽医杂志	Chinese Veterinary Journal	57	5	70-73,78	2021	-
6695	内禽病态势及2021年禽病	situation and2021Annual p	叶亦见.	Ye Yijian.	中国禽业导刊	China Poultry Industry Guid	38	2	19-22	2021	-

6696	食物中毒实验室检测的研	n Laboratory Detection of	苏小芸,张春蕾.	Su Xiaoyun,Zhang Chunlei.	中国卫生产业	China health industry	18	13	182-185	2021	ki.1672-5654.2021.13.182
6697	反应在检测冷冻禽、畜肉	ain reaction in the detectio	乔玉林.	Qiao Yulin.	临床研究	clinical research	29	9	141-142	2021	-
6698	验方法对食品中沙门氏菌	nd research on testing met	岩,胥小荣,庞婕,王艳英,苗	Xiaorong,Pang Jie,Wang Ya	食品安全导刊	food safety guide	-	26	69-70	2021	-
6699	分地区蛇源性细菌的耐药	e of snake-derived bacteria	晨,陶飞,白慧丽,李常挺,	n,Tao Fei,Bai Huili,Li Chang	今日畜牧兽医	ivestock Veterinarian Toda	37	7	11-13	2021	sn.1673-4092.2021.07.009
6700	门菌和副溶血性弧菌的分	almonella and Vibrio parah	茂荣,钟凌,姚海燕,郭宝	,Zhong Ling,Yao Haiyan,Gu	热带医学杂志	journal of tropical medicine	21	2	175-180	2021	sn.1672-3619.2021.02.012
6701	学附属北京中医医院感染	ea pathogens in Beijing Ho	翔,杨春娇,田敬华,张会	ng Chunjiao,Tian Jinghua,Z	中华预防医学杂志	se Journal of Preventive Me	55	7	879-883	2021	n112150-20200714-01005
6702	食品监测常见食源性致病	ommon foodborne pathog	仇文轩.	Qiu Wenxuan.	养生保健指南	Health and Wellness Guide	-	42	291-292	2021	-
6703	非伤寒沙门菌血清型和耐	drug resistance of invasive	艳,王伟,崔鑫楠,何琼玉,李	ng Wei,Cui Xinnan,He Qion	中华流行病学杂志	inese Journal of Epidemiol	42	3	549-554	2021	n112338-20200316-00361
6704	省腹泻病人沙门菌血清型	sistance of Salmonella sero	楠,马泰,汪梦,吴小珉,王	atai,Wang Meng,Wu Xiaom	中国食品卫生杂志	hinese Food Hygiene Journ	33	5	536-541	2021	).13590/j.cjfh.2021.05.003
6705	性腹泻病病原菌分布特征	cteria of foodborne diarrhe	王维,王林.	Wang Wei,Wang Lin.	现代检验医学杂志	l of Modern Laboratory Me	36	4	51-54,80	2021	sn.1671-7414.2021.04.011
6706	生素相关性腹泻主要病原	thogenic bacteria in antibi	齐武,王媛媛,张文竹,卢金	Yuanyuan,Zhang Wenzhu,	热带医学杂志	journal of tropical medicine	21	5	549-551,595	2021	sn.1672-3619.2021.05.005
6707	上海市某区食源性疾病监	foodborne disease surveilla	张雪英,龚华.	Zhang Xueying,Gong Hua.	上海预防医学	hanghai Preventive Medici	33	8	720-725	2021	28/j.cnki.sjpm.2021.20142
6708	区2020年从业人员沙门菌	020Annual survey on Salm	,朱春秀,陈永东,龙小莉,	nxiu,Chen Yongdong,Long	海峡预防医学杂志	s Journal of Preventive Me	27	3	69-71	2021	-
6709	食源性疾病暴发的流行病	tion of foodborne disease	李卫华,马佳平.	Li Weihua,Ma Jiaping.	健康大视野	Healthy vision	-	19	23,25	2021	-
6710	餐饮食品中食源性致病菌	rne pathogenic bacteria in	婷,彭鑫,宋晓立,高志奇,	Peng Xin,Song Xiaoli,Gao Z	食品安全质量检测学报	f Food Safety and Quality I	12	7	2691-2694	2021	-
6711	伤寒、副伤寒沙门菌血清	tance of Salmonella typhi a	乔玉林.	Qiao Yulin.	临床研究	clinical research	29	1	138-139	2021	-
6712	海市杨浦区肉及肉制品中	genic bacteria in meat and	婷,肖建伟,徐红,曹健,庄	o Jianwei,Xu Hong,Cao Jiar	现代食品	modern food	-	15	128-130,133	2021	.cn41-1434/ts.2021.15.035
6713	炎沙门菌食源性疾病暴发	almonella Enteritidis foodb	徐皓轩,蓝岳云,张茜,万圣	an Yueyun,Zhang Qian,Hal	预防医学	Preventive Medicine	33	6	621-623	2021	ssn2096-5087.2021.06.020
6714	子标本中病原菌的培养及	en with foodborne diarrhea	陈苏巧.	Chen Suqiao.	抗感染药学	Anti-infective Pharmacy	18	3	401-403	2021	sn.1672-7878.2021.03-026
6715	源性食品中ST198型肯塔	ng CityST198Research on	,侯轩,周梦蓉,邓明惠,段	hou Mengrong,Deng Ming	食品安全质量检测学报	f Food Safety and Quality I	12	17	6756-6762	2021	-
6716	源性沙门菌监测数据的S-	ed Salmonella in Nanning	兰,曾献莹,韦程媛,李秀	ng Xianying,Wei Chengyuar	中国预防医学杂志	se Journal of Preventive Me	22	2	140-145	2021	/j.1009-6639.2021.02.012
6717	售冷藏冷冻动物源食品致	organisms in commercially	黄莉萍,罗书全.	Huang Liping,Luo Shuquan	食品安全质量检测学报	f Food Safety and Quality I	12	10	4286-4291	2021	-
6718	菌1,4,[5],12:i:-耐药性及分	2:i:-Research on drug resis	吕素玲,瞿聪,蓝兰,谭冬梅	uling,Qu Cong,Lanlan,Tan	中国食品卫生杂志	hinese Food Hygiene Journ	33	5	558-565	2021	).13590/j.cjfh.2021.05.007
6719	年黔江地区儿童腹泻常见	non pathogens of childhoo	何小阳,王永红.	e Xiaoyang,Wang Yonghon	饮食保健	Diet and health care	-	20	273-274	2021	-
6720	场沙门氏菌分离鉴定及	Resistance Analysis of Salm	周政.	Zhou Zheng.	养猪	pig raising	-	4	106-108	2021	sn.1002-1957.2021.04.032
6721	毒的病原学情况与微生物	d microbial test results of	刘利克.	Liu Like.	中外食品工业	nese and foreign food indu	-	12	103-104	2021	sn.1672-5336.2021.12.047
6722	童细菌性腹泻的病原微生	croorganism testing for ba	何义,张颖.	He Yi,Zhang Ying.	中国农村卫生	rural health in china	13	6	15-16	2021	n.1674-361X.2021.06.009
6723	沙门氏菌的分离鉴定及毒	analysis of Salmonella orig	振书,张凯悦,管玉堂,庞喆	hang Kaiyue,Guan Yutang,	中国畜牧兽医	nal husbandry and veterina	48	8	3069-3078	2021	ki.1671-7236.2021.08.040
6724	沙门菌流行特征及指纹图	olymorphism analysis of h	玉,李敬晶,董锐,郑晓华,谢	ing,Dong Rui,Zheng Xiaohu	疾病监测	disease surveillance	36	6	605-610	2021	).3784/jbjc.202103110118
6725	山西省沙门氏菌分子分型	typing and drug resistance	娥,王洋,张秋香,杨红霞,韩	ng,Zhang Qiuxiang,Yang Ho	中国人兽共患病学报	Chinese Journal of Zoonose	37	9	815-820	2021	sn.1002-2694.2021.00.119
6726	患儿非伤寒沙门菌感染的	hoid Salmonella infection i	冠桦,李慧雯,陈佩瑜,耿岚	,Li Huiwen,Chen Peiyu,Ger	中华实用儿科临床杂志	Clinical Journal of Practical	36	10	744-747	2021	n101070-20200317-00432
6727	226例尿培养病原菌种类及	pathogenic bacteria types	汪美英.	Wang Meiying.	药品评价	drug evaluation	18	6	362-365	2021	nki.1672-2809.2021.06.14
6728	省臭豆腐微生物污染监	ination monitoring results	召,国译丹,杨菁,邹颜秋硕	uoyidan,Yang Jing,Zou Yanc	食品安全质量检测学报	f Food Safety and Quality I	12	10	4309-4314	2021	-
6729	浙江省湖州市食源性疾病	disease surveillance results	芳,徐德顺,纪蕾,查赟峰,陈	u Deshun,Ji Lei,Zha Yunfer	疾病监测	disease surveillance	36	9	958-962	2021	).3784/jbjc.202105150270
6730	沙门菌的流行与耐药性	g resistance analysis of Sa	孙先谱,孙凡,徐正中,焦新	,Sun Fan,Xu Zhengzhong,J	中国人兽共患病学报	Chinese Journal of Zoonose	37	9	795-800	2021	sn.1002-2694.2021.00.108
6731	泻常见病原菌分布及影	actors of common pathoge	杜笑颖,李莉.	Du Xiaoying,Li Li.	山西医药杂志	Shanxi Medical Journal	50	9	1412-1415	2021	sn.0253-9926.2021.09.002
6732	徽省某市食源性疾病病例	results of foodborne disea	琴,翟光富,陈柳,朱小琼,谢	i Guangfu,Chen Liu,Zhu Xia	现代预防医学	modern preventive medicin	48	20	3808-3814	2021	-

6733	年大连市食源性疾病监测	foodborne disease surveil	凤,潘微,栾明春,宋晓昀,薄	Wei,Luan Mingchun,Song X	职业与健康	Occupation and health	37	11	1473-1477	2021	-
6734	包装辣条类调味面制品微	ckaged spicy strips and sea	赵雯婧,炊慧霞,马笑菲.	Wenjing,Cui Huixia,Ma Xia	中国卫生检验杂志	ese Journal of Health Inspe	31	12	1523-1527,1536	2021	-
6735	区近4年食物中毒事件致病	tribution of pathogenic bac	宋晓青,王欢欢,彭雄.	oqing,Wang Huanhuan,Per	实用中西医结合临床	ation of Traditional Chinese	21	9	117-119	2021	sn.1671-4040.2021.09.059
6736	门菌肠炎患儿临床特点及	nd drug resistance in childr	付宽慈.	Fu Kuanci.	当代医药论丛	ontemporary Medicine Seri	19	12	51-53	2021	sn.2095-7629.2021.12.029
6737	郎区沙门氏菌食物中毒事件	monella Food Poisoning Inc	廖婵,曹应琼,梅丽敏.	Chan,Cao Yingqiong,Mei Li	医学信息	medical information	34	10	158-160	2021	sn.1006-1959.2021.10.046
6738	聚餐引起的沙门氏菌食物中	food poisoning incident cau	王世萍.	Wang Shiping.	寄生虫病与感染性疾病	ic diseases and infectious d	19	1	38-41	2021	-
6739	菌引起家庭食源性疾病暴	hold foodborne disease out	陈桂芳,陈雪芳.	Chen Guifang,Chen Xuefang	医学食疗与健康	edical Diet Therapy and Hea	19	15	221-223	2021	-
6740	杂鸡胚胎带菌情况调查及	ures on bacterial infection i	王小康,王方昆,常维山,庚	ang,Wang Fangkun,Chang V	家禽科学	poultry science	-	6	37-41	2021	sn.1673-1085.2021.06.012
6741	伤寒沙门菌(iNTS)基因组和	onella(iNTS)Genomic and	伟,许学斌,王艳,崔鑫楠,徐	u Xuebin,Wang Yan,Cui Xin	中国食品卫生杂志	hinese Food Hygiene Journ	33	3	249-255	2021	).13590/j.cjfh.2021.03.001
6742	安市食源性疾病病原学主	surveillance results of foo	刘萍,张锋,刘冬.	u Ping,Zhang Feng,Liu Don	公共卫生与预防医学	Health and Preventive Me	32	6	36-39	2021	sn.1006-2483.2021.06.009
6743	血性弧菌食物中毒的实验室	lysis of a case of Vibrio par	邱艺燕,欧秀华.	Qiu Yiyan,Ou Xiuhua.	中国卫生标准管理	a Health Standard Manager	12	12	58-61	2021	sn.1674-9316.2021.12.019
6744	腹泻样本中食源性致病菌分	odborne pathogenic bacte	江晓,叶艳华,刘品,王燕,吴	Ye Yanhua,Liu Pin,Wang Ya	中国临床研究	clinical research in china	34	8	1077-1080	2021	29/j.cnki.cjcr.2021.08.016
6745	县食品食源性致病菌检测	Foodborne Pathogenic Bac	雯,梁志强,顾时平,叶炜,张	g Zhiqiang,Gu Shiping,Ye V	预防医学	Preventive Medicine	33	7	732-734,737	2021	sn2096-5087.2021.07.021
6746	性食物中毒的微生物学检验	ination and analysis of bac	王霞.	Wang Xia.	健康必读	Health must read	-	22	290	2021	-
6747	市顺义区成年人食源性疾	e foodborne disease survei	昌,陈东宛,高彭,何朝,刘秀	Dongwan,Gao Peng,He Dy	医学信息	medical information	34	16	159-162	2021	sn.1006-1959.2021.16.045
6748	门菌和弯曲杆菌的流行性调	of Salmonella and Campyl	研,张纯萍,白玉惠,李泽坤,张	ang Chunping,Bai Yuhui,Li Z	中国家禽	Chinese poultry	43	6	61-66	2021	sn.1004-6364.2021.06.011
6749	2020年消毒餐(饮)具微生物	isinfection meal(drink)With	海珍,魏万彩,石红霞,祁叠	n,Wei Wancai,Shi Hongxia,	现代食品	modern food	27	9	117-119	2021	cn41-1434/ts.2021.09.033
6750	门菌污染对居民健康影响的	mpact of salmonella conta	王晔茹,王彝白纳,刘阳泰,李	Yeru,Wang Yibaina,Liu Yar	卫生研究	health research	50	4	646-652,664	2021	eishengyanjiu.2021.04.018
6751	定量PCR法检测中药制剂	Detection of Salmonella in	德,张文婷,易巧,熊骏,刘练	ng Wenting,Yi Qiao,Xiong J	中国现代应用药学	nese modern applied pharm	38	7	836-840	2021	sn1007-7693.2021.07.011
6752	养分离菌的临床分布特点	nd drug susceptibility result	朱德永,袁雕,陆兴热.	Deyong,Yuan Diao,Lu Xing	检验医学与临床	boratory medicine and clini	18	16	2403-2405	2021	sn.1672-9455.2021.16.028
6753	的细菌性病原分离及16S rR	al pathogens of porcupine	周玉照,张小苗,张以芳.	zhao,Zhang Xiaomiao,Zhan	野生动物学报	Journal of Wildlife	42	4	1177-1182	2021	sn.1000-0127.2021.04.032
6754	市食源性疾病主动监测流行	etiology of active surveillan	富,刘小华,蒋家俊,江美琴	fu,Liu Xiaohua,Jiang Jiajun,	贵州医药	Guizhou Medicine	45	9	1431-1433	2021	n.1000-744X.2021.09.044
6755	场蛋鸡沙门氏菌分离鉴定及	alysis of Salmonella from la	玉,张毅,张凌,艾柯代·吐鲁	,Zhang Yi,Zhang Ling,Ecod	动物医学进展	vances in Veterinary Medic	42	11	60-64	2021	sn.1007-5038.2021.11.011
6756	年康平县食源性致病菌监	results of foodborne patho	杨伟才.	Yang Weicai.	中外女性健康研究	nd foreign women's health	-	17	197-198	2021	-
6757	市同期五起伤寒病原学研究	ity of five cases of typhoid	王彦青,李燕,宋燕,邹晓楠,	anqing,Li Yan,Song Yan,Zou	中华预防医学杂志	se Journal of Preventive Me	55	1	78-83	2021	n112150-20200624-00922
6758	区腹泻病例分离沙门氏菌耐	of Salmonella isolates from	,张赫,张爽,冀国强,马红梅	,Zhang Shuang,Ji Guoqiang	中国食品卫生杂志	hinese Food Hygiene Journ	33	3	295-302	2021	).13590/j.cjfh.2021.03.010
6759	地区沙门氏菌病原学特征及	aracteristics and drug resis	李惠娟,魏琼,张燕飞.	uijuan,Wei Qiong,Zhang Ya	宁夏医学杂志	Ningxia Medical Journal	43	8	767-768,前插1	2021	j.1001-5949.2021.08.0767
6760	斗瓦利斯沙门菌的耐药性及	ecular typing of Salmonella	强,黄雪欢,瞿孝云,马叶本,	Xuehuan,Qu Xiaoyun,Ma Ye	中国兽医杂志	Chinese Veterinary Journal	57	4	75-80	2021	-
6761	季节性腹泻160例患者诊	rhea160Clinical research in	吴祥铭.	Wu Xiangming.	自我保健	self care	-	11	210	2021	-
6762	隆阳区细菌性食物中毒检	ial food poisoning detectio	陈思行.	Chen Sixing.	世界最新医学信息文摘	he world's latest medical in	21	65	315-316	2021	sn.1671-3141.2021.65.156
6763	后细菌性病原的分离鉴定与	istance Analysis of Bacterial	,柏传茂,杨生明,郭永久,质	nao,Yang Shengming,Guo J	江苏农业科学	iangsu Agricultural Science	49	11	127-131	2021	sn.1002-1302.2021.11.023
6764	规检验在季节性腹泻诊治中	ination in the diagnosis an	赵春雨.	Zhao Chunyu.	自我保健	self care	-	4	80-81	2021	-
6765	省肠炎沙门菌分离株分子	resistance of Salmonella E	芝,于淼,李飞,张铭琰,张	Miao,Li Fei,Zhang Mingyan	中国微生态学杂志	inese Journal of Microecolo	33	7	770-773,778	2021	381/j.cnki.cjm.202107005
6766	中沙门氏菌的分离鉴定及其	ug susceptibility analysis o	娅绯,李思思,张强,陈皆	i,Li Sisi,Zhang Qiang,Chen J	中国动物检疫	China Animal Quarantine	38	10	45-50	2021	n.1005-944X.2021.10.009
6767	食品、餐具食源性致病菌监	e pathogenic bacteria in ca	李羽翡,芮文君,傅雷,李羽翠	ufei,Rui Wenjun,Fu Lei,Li Yu	中国食物与营养	Chinese food and nutrition	27	7	12-16,89	2021	sn.1006-9577.2021.07.002
6768	年深圳市食物中毒事件流行	cal analysis of food poisoni	平,张锦周,梁浩,潘柳波,质	ng Jinzhou,Liang Hao,Pan	公共卫生与预防医学	Health and Preventive Me	32	3	41-44	2021	sn.1006-2483.2021.03.010
6769	沟区食源性疾患分离沙门	Salmonella isolated from	刘海涛,吕秋艳,赵香菊.	Haitao,Lu Qiuyan,Zhao Xian	养生保健指南	Health and Wellness Guide	-	35	283	2021	-

6770	天津市宁河区食源性疾病	rne disease surveillance res	么立满,刘爽.	Mo Liman,Liu Shuang.	职业与健康	Occupation and health	37	13	1757-1760	2021	-
6771	京市海淀区感染性腹泻病	of infectious diarrhea patho	邵云平,郭黎,蔡伟.	hao Yunping,Guo Li,Cai We	职业与健康	Occupation and health	37	14	1958-1962	2021	-
6772	门菌引起食源性疾病暴发	borne disease outbreak ca	玲玲,炊慧霞,李艳芬,崔	g,Cui Huixia,Li Yanfen,Cui	中国卫生检验杂志	ese Journal of Health Inspe	31	9	1037-1039,1043	2021	-
6773	沙门菌暴发疫情快速检	agnosis analysis of a Salmo	庆,封正娟,谭琳,陈嫣,刘	Zhengjuan,Tan Lin,Chen	预防医学情报杂志	of Preventive Medicine Info	37	7	1007-1010	2021	-
6774	的沙门菌耐药性监测及	f Salmonella from differen	华,张炜煜,王艳秋,龚云	hang Weiyu,Wang Yanqiu,	中国卫生工程学	Chinese sanitary engineerin	20	3	377-380	2021	sn.1671-4199.2021.03.008
6775	沙门氏菌分离鉴定及药敏	drug susceptibility test of	乜英青.	What Yingqing.	中国畜禽种业	estock and poultry breeding	17	4	53-54	2021	sn.1673-4556.2021.04.031
6776	成人腹泻病原微生物的检	ogenic microorganisms in	唐翠连.	Tang Cuilian.	家庭生活指南	Family Living Guide	37	15	117-118	2021	-
6777	菌性食物中毒的微生物学	ical testing of bacterial foo	李嫔.	Li Bin.	饮食保健	Diet and health care	-	26	265	2021	-
6778	细菌性食源性疾病主要	ics of main pathogens of b	丽,孙婷,林旭,段德水,刘铁	Ting,Lin Xu,Duan Deshui,Li	华南预防医学	uth China Preventive Medic	47	3	339-342	2021	.12183/j.scjpm.2021.0339
6779	相关病原流行特征及腹泻	ogens and the drug resista	,冯宝立,王金龙,韩雅俊,	Baoli,Wang Jinlong,Han Y	疾病监测	disease surveillance	36	4	381-386	2021	-
6780	感染性腹泻病人来源沙门	ting from patients with acu	金金,吴炫桢,许玉成,欧泳	Wu Xuanzhen,Xu Yucheng,	热带医学杂志	ournal of tropical medicine	21	3	377-381	2021	sn.1672-3619.2021.03.027
6781	沙门氏菌耐药性监测及ES	originating from pets in Be	东,王凡,周冰倩,杨梅,丁	ng Fan,Zhou Bingqian,bayb	微生物学通报	Bulletin of Microbiology	48	8	2714-2722	2021	4/j.microbiol.china.210357
6782	菌性食物中毒病原菌的调	and prevention of pathoge	吴世娟.	Wu Shijuan.	健康必读	Health must read	-	11	187	2021	-
6783	氏菌鉴定和分型方法比对	identification and typing	婧,王立平,杨红莲,薛晨玉,	iping,Yang Honglian,Xue C	现代食品	modern food	27	14	146-150	2021	cn41-1434/ts.2021.14.044
6784	菌感染伴抗菌药过敏患者	h salmonella infection and	邓敏,李鹏鹏,丁楠,王卓.	n,Li Juanjuan,Ding Nan,Wa	药学实践杂志	ournal of Pharmacy Practic	39	5	468-471	2021	ssn.1006-0111.202103009
6785	菏泽市食品中食源性致病	g results of foodborne pat	刘伟,刘艳荣.	Liu Wei,Liu Yanrong.	中国卫生检验杂志	ese Journal of Health Inspe	31	5	618-623	2021	-
6786	门氏菌污染引起的群体食	borne diarrhea caused by S	丽,杨洋,次仁卓玛,陈洲	ng Yang,Tsering Dolma,Ch	医药前沿	Medical Frontier	11	18	193-194	2021	-
6787	生疾病应用抗生素治疗的	ation of antibiotics in the t	裴桂霞,刘士刚.	Pei Guixia,Liu Shigang.	中国畜禽种业	estock and poultry breeding	17	5	163-164	2021	sn.1673-4556.2021.05.099
6788	氏菌的分离鉴定及PCR快	Salmonella from porkPCR	崔霞.	Cui Xia.	食品安全导刊	food safety guide	-	24	65-66	2021	-
6789	食品厂芝麻酱生产加工环	the production and proce	邓涛,梅淑华,李林,杨光武	o,Mei Shuhua,Li Lin,Yang G	寄生虫病与感染性疾病	ic diseases and infectious d	19	1	47-50,56	2021	-
6790	病菌Taqman多重荧光定	tiplex fluorescence quanti	陈秀琴,林甦,郑敏,黄梅清	in,Lin Su,Zheng Min,Huang	福建畜牧兽医	al Husbandry and Veterina	43	3	22-27	2021	sn.1003-4331.2021.03.008
6791	生致病菌带菌情况检测结	ion results of foodborne pa	曲彩红.	Qu Caihong.	临床研究	clinical research	29	9	137-139	2021	-
6792	动态固定术后感染的临床	s of postoperative infectio	李培,赵晨,高永建,王羿阳	Chen,Gao Yongjian,Wang Y	中华骨科杂志	hinese Journal of Orthopedi	41	17	1275-1282	2021	n121113-20210316-00244
6793	门菌引起的学校食源性疾	ol foodborne illness outbre	建,吴燕飞,刘东妹,方艳梅,	Yanfei,Liu Dongmei,Fang Y	中国食品卫生杂志	hinese Food Hygiene Journ	33	5	595-599	2021	.13590/j.cjfh.2021.05.014
6794	中心食源性致病菌检验室	borne pathogen laboratory	,刘越连,杨舒筠,杨枫,王	an,Yang Shujun,Yang Feng,	中国药物与临床	hinese medicine and clinica	21	7	1198-1199	2021	1655/zgywylc2021.07.066
6795	炎沙门菌食物中毒事件病	of a Salmonella Enteritidis	,宋秋坤,石岩,李艳艳,郭	Qiukun,Shi Yan,Li Yanyan,	医学动物防制	medical animal control	37	8	771-774	2021	0.7629/yxdwfz202108016
6796	中沙门菌的传播特点和	in pig slaughterhouses and	通,潘明星,孟闯,焦新安,潘	Mingxing,Meng Chuang,Jia	畜牧与兽医	Husbandry and Veterinary M	53	5	127-132	2021	-
6797	门县食源性疾病监测结果	e disease surveillance resu	祁先群,王子友,郑涛涛.	nqun,Wang Ziyou,Zheng Ta	预防医学	Preventive Medicine	33	6	631-633	2021	sn2096-5087.2021.06.023
6798	制品食源性致病菌污染	nd pathogenic characterist	宇,向婧姝,张德著,张豫,安	g Jingshu,Written by Zhan	现代预防医学	modern preventive medicin	48	11	1951-1954,2060	2021	-
6799	反应检测沙门氏菌的诊断	f comprehensive biochemi	周宇轩,全颖莹.	Zhou Yuxuan,Quan Yingying	医学食疗与健康	edical Diet Therapy and Hea	19	12	157-158	2021	-
6800	童病例病原学检出情况及	and characteristics of chi	播先莉,李敏,王文慧,占达	nli,Li Min,Wang Wenhui,Zh	华南预防医学	uth China Preventive Medic	47	3	284-287	2021	.12183/j.scjpm.2021.0284
6801	R检测在肠道致病菌检测	esting in the detection of e	饶小惠.	Rao Xiaohui.	医学美学美容	edical Aesthetics and Beau	-	18	132-133	2021	-
6802	王食品沙门氏菌分离鉴定	tection methods in the iso	何志勇.	He Zhiyong.	微量元素与健康研究	e elements and health rese	38	4	49-50	2021	-
6803	感染性腹泻监测病例细菌	m surveillance results of i	,皮海轮,张文增,史继新,	i Lun,Zhang Wenzeng,Shi J	医学信息	medical information	34	10	145-148	2021	sn.1006-1959.2021.10.042
6804	2020年细菌性食物中毒的	nalysis of etiological detect	黄晓平,翁奕敏,史煜曼.	Xiaoping,Weng Yimin,Shi Y	河南医学研究	Henan Medical Research	30	18	3319-3322	2021	n.1004-437X.2021.18.014
6805	引起的肠炎沙门氏菌食物	nella Enteritidis food poiso	赵雯婧,王轶,郭晏强,李爱	enjing,Wang Yi,Guo Yanqia	河南预防医学杂志	Journal of Preventive Me	32	3	252-255	2021	m.1006-8414.2021.03.027
6806	019年沙门菌血清型分布	nnual Salmonella serotype	绮琳,郑悦康,刘绮明,区金	Zheng Yuekang,Liu Qiming	中国热带医学	Chinese tropical medicine	21	8	758-763	2021	cnki.46-1064/r.2021.08.09

6807	川省食源性疾病哨点医院	ults of foodborne disease	周玉锦,张誉,陈文,兰祎婷	jin,Zhang Yu,Chen Wen,La	预防医学情报杂志	of Preventive Medicine Info	37	6	792-797	2021	-
6808	鼠伤寒沙门菌肠炎的临床	eristics of Salmonella typh	李孟珠,袁林,卓志强.	engzhu,Yuan Lin,Zhuo Zhiq	中国小儿急救医学	tric Emergency Medicine in	28	6	537-539	2021	sn.1673-4912.2021.06.020
6809	非伤寒沙门菌临床分离株	ce and molecular typing of	李世军,王丹,黄俊飞,童毅	ng Dan,Huang Junfei,Tong	中国人兽共患病学报	Chinese Journal of Zoonose	37	7	603-610	2021	sn.1002-2694.2021.00.099
6810	病监测中病原微生物检验	athogenic microorganisms	林志永,陈积月.	Lin Zhiyong,Chen Jiyue.	饮食保健	Diet and health care	-	22	260	2021	-
6811	酶链反应法检测沙门菌与	ive polymerase chain react	曹原.	Cao Yuan.	实用医技杂志	l of Practical Medical Tech	28	7	897-899	2021	ki.1671-5098.2021.07.022
6812	州区食源性疾病主动监测	of foodborne disease active	,张萍,吴建军,张海鹏,马晓	ing,Wu Jianjun,Zhang Haiju	职业与健康	Occupation and health	37	13	1761-1763,1767	2021	-
6813	四川省食源性疾病暴发事	dborne disease outbreaks	陈文,许毅,林黎.	Chen Wen,Xu Yi,Lin Li.	预防医学情报杂志	of Preventive Medicine Info	37	8	1064-1068,1074	2021	-
6814	沙门菌和轮状病毒肠炎的	ristics and medications of	利,李韬,宋晋,范鹏斌,王丽	,Song and Jin,Fan Pengbin,	世界最新医学信息文摘	he world's latest medical in	21	36	218-219,221	2021	sn.1671-3141.2021.36.088
6815	山市食品中食源性致病菌	ults of foodborne pathoge	张敏,孙延芳.	Zhang Min,Sun Yanfang.	中国卫生产业	China health industry	18	9	178-180	2021	ki.1672-5654.2021.09.178
6816	市售食品致病性微生物监	croorganisms in commercia	玉婷,彭慧,程仁杰,季莹,袁	eng Hui,Cheng Renjie,Ji Yin	预防医学	Preventive Medicine	33	5	532-535	2021	sn2096-5087.2021.05.026
6817	面生化反应和血清学检验	ehensive biochemical react	李惠芳,王红梅.	Li Huifang,Wang Hongmei.	保健文汇	Health Wenhui	22	25	196-197	2021	-
6818	年食源性疾病主动监测流	aracteristics of Foodborne	金迪,郭宝福,孙桂菊.	Jindi,Guo Baofu,Sun Guiju.	中国公共卫生	Chinese public health	37	3	564-567	2021	10.11847/zgggws1128260
6819	食源性疾病流行特征和重	orne diseases and infectio	强,薛春洪,何远东,朱永义	ue Chunhong,He Yuandong	医学动物防制	medical animal control	37	5	450-453	2021	0.7629/yxdwfz202105011
6820	0年某医院食源性疾病主动	surveillance of foodborne	乔红英.	Qiao Hongying.	河南医学研究	Henan Medical Research	30	11	1974-1977	2021	n.1004-437X.2021.11.014
6821	门菌食物中毒的病原学检	y analysis of a case of Salm	芬,吴鹏程,施菊萍,施爱萍,	Pengcheng,Shi Juping,Shi	医学动物防制	medical animal control	37	5	434-437	2021	0.7629/yxdwfz202105007
6822	东省牛羊疫病报告及监测	emic disease reports and s	,陈峰,渠珂,吕桂霞,徐栋,	Feng,Qu Ke,Lu Guixia,Xu D	中国动物检疫	China Animal Quarantine	38	7	25-30	2021	n.1005-944X.2021.07.007
6823	儿童急性腹泻流行病学调	stance analysis of acute dia	王敏.	Wang Min.	中国国境卫生检疫杂志	ntier Health and Quarantin	44	3	181-183	2021	/j.1004-9770.2021.03.008
6824	菌性食物中毒的微生物学检	ical testing of bacterial foo	雷慧欣,郭凡.	Lei Huixin,Guo Fan.	中外食品工业	nese and foreign food indu	-	7	113-114	2021	sn.1672-5336.2021.07.060
6825	津市津南区食源性致病菌	ity analysis of foodborne p	,梁广忠,李胜强,李龙玉,关	zhong,Li Shengqiang,Li Lon	中国食品卫生杂志	hinese Food Hygiene Journ	33	5	548-552	2021	0.13590/j.cjfh.2021.05.005
6826	病患者肠道感染情况及耐	al infections and drug resis	谢璐蔓,黄葵,覃善芳.	uman,Huang Kui,Qin Shan	新发传染病电子杂志	ournal of Emerging Infectio	6	3	220-224	2021	j.cnki.xfcrbzz.2021.03.012
6827	机会性感染病原菌分布及	nce of pathogenic bacteria	易银,陈铭,张立丽,张世斌	hen Ming,Zhang Lili,Zhang	标记免疫分析与临床	eled immunoassays and cli	28	7	1084-1091	2021	sn.1006-1703.2021.07.002
6828	市儿童腹泻感染病原谱	ectrum of diarrheal infectio	莉,史东坡,于德山,马建中	i Dongpo,Yu Deshan,Ma Ji	中国卫生检验杂志	ese Journal of Health Inspe	31	13	1567-1569,1573	2021	-
6829	海淀区食源性腹泻病原监	of foodborne diarrhea path	刘伟,尹可欣,赵振.	iu Wei,Yin Kexin,Zhao Zhen	寄生虫病与感染性疾病	ic diseases and infectious d	19	3	138-142	2021	-
6830	沙门菌耐药性分析及毒力	virulence gene detection o	利峰,郭晓芳,张晓华,梁丽	o Xiaofang,Zhang Xiaohua,	中国药物与临床	hinese medicine and clinica	21	18	3089-3092	2021	1655/zgywylc2021.18.009
6831	沟区感染性腹泻患者便样	stool samples from patien	秋艳,李龙建,刘海涛,赵香	,Li Longjian,Liu Haitao,Zha	首都公共卫生	capital public health	15	2	106-107,114	2021	-
6832	～2020年细菌性食物中毒	ysis of annual bacterial foo	张艳红,刘冰.	Zhang Yanhong,Liu Bing.	首都食品与医药	Capital Food and Medicine	28	12	146-148	2021	sn.1005-8257.2021.12.081
6833	验在季节性腹泻检验中的	of routine stool examinatio	项少黎,吴金良.	Xiang Shaoli,Wu Jinliang.	中国保健营养	Chinese health nutrition	31	29	61	2021	-
6834	菌腹泻临床特征、病原体	serology and drug resistan	南,杨晓华,何嘉麟,简晓	,Yang Xiaohua,He Jialin,Jia	医学检验与临床	edical Laboratory and Clini	32	7	29-32	2021	sn.1673-5013.2021.07.008
6835	城区1起幼儿园札如病毒	y1Investigation into Zaravi	,吴向青,杨静静,季顺峰,谌	Xiangqing,Yang Jingjing,Ji S	江苏预防医学	iangsu Preventive Medicin	32	4	477-478	2021	sn.1006-9070.2021.04.033
6836	省动物源常见细菌耐药性	e of common bacteria of a	周沁怡,李锐,杨华,郝力力,	Qinyi,Li Rui,Yang Hua,Hao	动物医学进展	vances in Veterinary Medic	42	8	107-111	2021	sn.1007-5038.2021.08.020
6837	并败血症患者病原菌分布	drug sensitivity in elderly pa	立罡,叶加建,薛小英,江	,Ye Jiajian,Xue Xiaoying,Jia	中国微生态学杂志	inese Journal of Microecolo	33	3	331-334	2021	381/j.cnki.cjm.202103016
6838	童感染性腹泻沙门菌和志	bility results of Salmonella	海林,李小娟,尹建雯,蒋鸿	Li Xiaojuan,Yin Jianwen,Jia	昆明医科大学学报	al of Kunming Medical Univ	42	7	83-87	2021	sn.2095-610X.S20210713
6839	血培养常见病原菌耐药性	common pathogenic bacte	彭军,黄孝天,柯江维,周艳	Huang Xiaotian,Ke Jiangwei	实验与检验医学	imental and Laboratory Me	39	3	589-592,602	2021	sn.1674-1129.2021.03.026
6840	9年金山区食源性致病菌	of foodborne pathogenic b	,韩东方,钟海明,俞丹丹,叶	ongfang,Zhong Haiming,Yu	实用预防医学	ractical preventive medicin	28	8	1006-1008	2021	sn.1006-3110.2021.08.029
6841	乳房炎病原菌的分离鉴定	alysis of pathogenic bacteri	张楠,张博,石玉祥.	ang Nan,Zhang Bo,Shi Yuxia	中国奶牛	Chinese cow	-	9	40-43	2021	nki.11-3009/s.2021.09.010
6842	单相变异株引起食源性疾	utbreak caused by a single-	秀川,赵士程,勾红梅,王艳	,Zhao Shicheng,Gouhongm	预防医学情报杂志	of Preventive Medicine Info	37	6	809-812	2021	-
6843	市食源性疾病主动监测病	ts of active surveillance of	郭娟,袁帅,邹莉,朱月潜.	an,Yuan Shuai,Zou Li,Zhu Y	职业与健康	Occupation and health	37	8	1056-1060	2021	-

6844	20年多重耐药肯塔基沙门	ic characteristics of multidr	瑛,田祎,张新,贾蕾,吕冰,	Tian Yi,Zhang Xin,Jia Lei,Lu	中华流行病学杂志	inese Journal of Epidemiolo	42	7	1252-1259	2021	n112338-20201206-01382
6845	19年沙门氏菌的血清型和	of serotypes and drug resi	沈赟,秦思,郑东宇,霍翔.	n,Qin Si,Zheng Dongyu,Hu	现代预防医学	modern preventive medicin	48	12	2263-2267	2021	-
6846	法在从业人员健康体检肠	d of anal swab specimens i	赵卫,刘萌萌.	Zhao Wei,Liu Mengmeng.	首都食品与医药	Capital Food and Medicine	28	13	96-97	2021	sn.1005-8257.2021.13.052
6847	餐馆饮食食品微生物抽样	ampling of food and bever	马韵婕.	Ma Yunjie.	现代食品	modern food	29	20	210-212	2021	.cn41-1434/ts.2021.20.057
6848	毒患者病原学情况及微生	th bacterial food poisoning	王天斯.	Wang Tiansi.	中国现代医生	modern chinese doctor	59	13	131-134	2021	-
6849	年-2019年生畜肉沙门菌监	is of Salmonella Monitorin	磊,赵士光,郑水萍,陈卫国	higuang,Zheng Shuiping,Ch	中国卫生检验杂志	ese Journal of Health Inspe	31	8	992-994	2021	-
6850	北地区鸡源沙门菌流行病	rug resistance analysis of S	尧,何云凤,李蕴玉,李佩国	,He Yunfeng,Li Yunyu,Li Pe	中国预防兽医学报	rnal of Preventive Veterina	43	9	924-929	2021	ssn.1008-0589.202101031
6851	菌食物中毒事件的病原检	traceability of a salmonella	燕,张文艳,杜小莉,周海	ang Wenyan,Du Xiaoli,Zho	医学动物防制	medical animal control	37	4	405-407	2021	0.7629/yxdwfz202104024
6852	014—2020年5岁以下儿童	Year5Analysis of infectiou	宋明,张丽,王晓博,李林军.	Dynasties,Zhang Li,Wang X	北方学院学报（自然科学	Northern University (Natura	37	12	19-23	2021	sn.1673-1492.2021.12.005
6853	菌血清型调查及其对小鼠	onella originating from mi	朱倩,时文健,袁朋,姜世金	an,Shi Wenjian,Yuan Peng,	兽类学报	ournal of Veterinary Scienc	41	3	245-253	2021	10.16829/j.slxb.150468
6854	3-2019年食源性疾病哨点	ysis of annual foodborne di	赵上瑛.	Zhao Shangying.	世界最新医学信息文摘	he world's latest medical i	21	39	327-328	2021	n.1671-3141.2021.39.162
6855	新都区鼠伤寒沙门菌耐药	s and molecular typing of S	群,舒玉聪,宋志琴,秦超,黄	ucong,Song Zhiqin,Qin Cha	中国食品卫生杂志	hinese Food Hygiene Journ	33	1	69-74	2021	0.13590/j.cjfh.2021.01.014
6856	菌引起食物中毒的病原学	ogy testing for food poison	方静.	Fang Jing.	健康必读	Health must read	-	25	92-93	2021	-
6857	肉馅中食源性致病菌污染	dborne pathogenic bacteri	王敏娣,邱颖,王宇卓,梁慧	ndi,Qiu Ying,Wang Yuzhuo,	医学动物防制	medical animal control	37	2	191-193	2021	0.7629/yxdwfz202102024
6858	腹泻病原菌的分离鉴定及	e study of diarrhea pathog	,穷珍,巴桑,次旺罗杰,石次	oor treasure,Basan,Tsewa	甘肃畜牧兽医	al Husbandry and Veterina	51	5	43-45,51	2021	n.1006-799X.2021.05.013
6859	在诊断儿童细菌性腹泻中	ic microbial testing in the	张霜.	Zhang Shuang.	实用医技杂志	l of Practical Medical Tech	28	8	1018-1020	2021	ki.1671-5098.2021.08.027
6860	人源性沙门菌数据的动态	Salmonella data in Xijiang	曾献莹,诸葛石养,韦程媛,	ing,Zhuge Shiyang,Wei Ch	中国热带医学	Chinese tropical medicine	21	6	552-558	2021	cnki.46-1064/r.2021.06.10
6861	20年饮用水水质卫生安全	t situation of drinking wate	蒋玉蕊.	Jiang Yurui.	健康必读	Health must read	-	18	274-275	2021	-
6862	菌食源性疾病暴发事件调	sis of a Salmonella Ente	;陈惠龙;陈上士;李闽真;	Huilong;Sergeant Chen;L	海峡预防医学杂志	Journal of Preventive M	27	6	73-75	2021	-
6863	沙门菌所致食物中毒的流	investigation on food poi	石锦鸿;张宇;杨秀程;李杰	g;Zhang Yu;Yang Xiuch	预防医学情报杂志	f Preventive Medicine In	37	12	1660-1663	2021	-
6864	一起食物中毒事件的调查	analysis of a food poison	郭邦成;刘翔;郝琼;田晓伟	ng;Liu Xiang;Hao Qiong;	疾病预防控制通报	e Prevention and Contro	36	6	30-33+73	2021	15/j.cnki.jbyfkztb.2108041
6865	菌引起食物中毒的病原学	gy testing for food poiso	凌玲[1]	Lingling[1]	技期刊数据库（全文版）	Journal Database (Full	-	6	106-107	2021	-
6866	沙门菌引起食物中毒菌株	in causing food poisonin	昌海[1];周其开[1];左荣余	hai[1];Zhou Qikai[1];Zuo	中国科技期刊数据库 医药	and Technology Journal Da	-	5	183-183	2021	-
6867	技术对两个家庭肠炎沙门	poisoning in two familie	[1];盘珍梅[1];蔡周梅[1];冼	Zhenmei[1];Cai Zhoume	技期刊数据库（全文版）	Journal Database (Full	-	4	239-240	2021	-
6868	起的肠炎沙门菌食物中毒	nella Enteritidis food poi	春艳[1];崔立周[1];祖文刚	yan[1];Cui Lizhou[1];Zu	医学动物防制	medical animal control	37	10	1000-1003	2021	-
6869	组测序在腹泻暴发调查中	mic sequencing in diarrh	赵嘉咏,张剑峰,周海健,李	g,Zhang Jianfeng,Zhou	疾病监测	disease surveillance	36	6	616-621	2021	0.3784/jbjc.202104060171
6870	疑似食源性疾病事件调查	t on a suspected foodbo	韩秋启,马辉.	Han Qiuqi,Ma Hui.	健康管理	health management	-	36	190-192	2021	-
6871	沙门菌引起的跨区食源性	orne disease outbreak c	伟;祁莉;周军波;赵滢;黄	Li;Zhou Junbo;Zhao Yi	疾病监测	disease surveillance	37	11	1495-1501	2022	-
6872	门菌食物中毒分离株的病	cteristics of Salmonella	楠;包小兵;栾旭波;宋艳	Xiaobing;Luan Xubo;So	江苏预防医学	angsu Preventive Medici	33	6	731-732	2022	sn.1006-9070.2022.06.035
6873	甲型副伤寒沙门菌暴发疫	nella Paratyphi A outbre	李毅;谢爱蓉;谢中必;	Yi;Xie Airong;Xie Zhong	中国人兽共患病学报	hinese Journal of Zoonos	38	10	898-905	2022	-
6874	门菌食物中毒的病原鉴定	ity analysis of a case of	兰芳;吕均;杨康;李艳丽;杜	;Lu Jun;Yang Kang;Li Y	医学动物防制	medical animal control	38	11	1039-1042	2022	-
6875	起的校园食源性疾病暴发	ampus foodborne diseas	;彭飞;彭子欣;范自军;李	ng Fei;Peng Zixin;Fan Z	中国食品卫生杂志	inese Food Hygiene Jou	34	5	1089-1094	2022	0.13590/j.cjfh.2022.05.036
6876	菌引起食源性暴发事件的	sis of a foodborne outbr	棕棕;骆水娟;夏雅红;楼丽	g;Luo Shuijuan;Xia Yah	中国卫生检验杂志	se Journal of Health Insp	32	18	2218-2221	2022	-
6877	门菌引起大学生食物中毒	isoning among college s	锋;马良良;吕婷;李欣欣;余	iangliang;Lu Ting;Li Xin	中国校医	Chinese school doctor	36	9	681-683+690	2022	-
6878	食源性疾病暴发事件的关	ability analysis of foodb	玲玲;炊慧霞;戚浩彧;李保	;Cui Huixia;Qi Haoyu;Li	河南预防医学杂志	Journal of Preventive M	33	9	650-653	2022	m.1006-8414.2022.09.003
6879	引起的食源性疾病暴发的	of an outbreak of foodb	学香;马健;赵媛坤;张丽	Ma Jian;Zhao Yuankun;Z	医学动物防制	medical animal control	38	9	902-905	2022	-
6880	罕见的萨奥沙门氏菌的分	ation of a rare strain of S	李海遥;	Li Haiyao;	食品安全导刊	food safety guide	-	23	59-61	2022	043/j.cnki.cfs.2022.23.019

6881	少门氏菌感染引起的食源性	k suspected to be caused	康冬花;汪生虎;沈作芳;	hua;Wang Shenghu;She	疾病预防控制通报	e Prevention and Control	37	4	53-55	2022	15/j.cnki.jbyfkztb.2202006
6882	门菌食源性疾病暴发事件的	of a Salmonella Enteritid	;邹文燕;张梦寒;王小龙;†	n;Zhang Menghan;Wang	上海预防医学	anghai Preventive Medic	34	7	665-670	2022	28/j.cnki.sjpm.2022.21564
6883	菌食源性疾病事件的调查和	analysis of a salmonella	;李柏生;黄友志;邱福明;吴	eng;Huang Youzhi;Qiu F	应用预防医学	pplied preventive medicin	28	3	225-228	2022	-
6884	塔基沙门菌引起聚集性腹泻	traceability analysis of c	娟;张良军;赵辉;刘凡;苏东	g Liangjun;Zhao Hui;Liu	职业与健康	Occupation and health	38	11	1460-1464	2022	329/j.cnki.zyyjk.2022.0312
6885	邓柏林沙门氏菌食源性疾病	Salmonella foodborne il	肖林;	Xiao Lin;	中国校医	Chinese school doctor	36	4	280-282	2022	-
6886	沙门菌食物中毒的实验室	sis of a case of Salmone	欧秀华;邱艺燕;	Ou Xiuhua;Qiu Yiyan;	热带病与寄生虫学	cal Diseases and Parasit	20	2	89-90+98	2022	-
6887	后沙门菌食物中毒的病原特	eristics of a case of Salm	李彩云;宗华;殷静;罗青梅	;Zong Hua;Yin Jing;Luo	中国卫生检验杂志	se Journal of Health Insp	32	7	818-820+831	2022	-
6888	包引起的肠炎沙门氏菌食	dent Caused by Salmone	刘颜;张先德;王乐;何玲玲	hang Xiande;Wang Le;H	中国食品卫生杂志	inese Food Hygiene Jour	34	2	359-364	2022	).13590/j.cjfh.2022.02.027
6889	炎沙门菌食物中毒事件的	resistance of suspected	家瑞;张梦寒;王小龙;邹文	ang Menghan;Wang Xia	江苏预防医学	angsu Preventive Medici	33	2	192-193+196	2022	sn.1006-9070.2022.02.022
6890	者引起沙门菌食源性疾病暴	Salmonella foodborne il	平;张拥军;李志峰;王红;彭	g Yongjun;Li Zhifeng;Wa	预防医学情报杂志	f Preventive Medicine In	38	3	369-376	2022	-
6891	菌和两种沙门氏菌引起的食	k caused by Vibrio para	莉;孙中兴;蒋元强;盛峰松	gxing;Jiang Yuanqiang;S	实用预防医学	actical preventive medici	29	3	350-352	2022	-
6892	沙门菌食物中毒的调查和	alysis of a case of Salmo	静媚;洪敏丽;陈永东;龙小	i;Hong Minli;Chen Yong	海峡预防医学杂志	Journal of Preventive M	28	1	93-95	2022	-
6893	门菌污染蛋糕致食源性疾	utbreak caused by salm	;李美华;廖琳虹;陈海滨;陈	hua;Liao Linhong;Chen H	预防医学论坛	reventive Medicine Foru	28	2	155-157+161	2022	sn.1672-9153.2022.02.023
6894	氏菌食物中毒的溯源分析及	sceptibility testing of a c	慧钰;时玉雯;刘铭;刘岚铮	Shi Yuwen;Liu Ming;Liu	保健医学研究与实践	th care research and pra	19	1	42-45	2022	-
6895	门菌食源性疾病暴发事件	Outbreak of Salmonella	徐皓轩;蓝岳云;张茜;万圣	n Yueyun;Zhang Qian;H	实用预防医学	actical preventive medici	29	1	85-87	2022	-
6896	门氏菌污染的皮蛋引起的食	used by eating preserve	唐琳[1]	Tang Lin[1]	技期刊数据库（文摘版）医	Journal Database (Abst	-	6	205-207	2022	-
6897	食物中毒事件中的卫生监督	n and handling of a food	[1];董德荣[1];王勇[1];刘	g Derong[1];Wang Yong	中国消毒学杂志	nese Journal of Disinfec	39	8	639-640	2022	-
6898	性食物中毒的快速处理及	aceability analysis of a cl	王君[1];严梅[1]	Wang Jun[1];Yan Mei[1]	中国科技期刊数据库 医药	and Technology Journal Da	-	3	116-118	2022	-
6899	中学食物中毒检验与调查	and investigation proces	罗江淑[1]	Luo Jiangshu[1]	技期刊数据库（文摘版）医	Journal Database (Abst	-	5	209-211	2022	-
6900	起食源性肠道传染病的调	and analysis of foodborn	胡峻[1]	Hu Jun[1]	保健医学研究与实践	th care research and pra	19	11	33-36	2022	-
6901	中毒的病原学情况及微生	robial test results of bac	高展[1]	Gao Zhan[1]	现代食品	modern food	28	15	226-228	2022	-
6902	物中毒病原学情况及微生物	bial testing analysis of b	1];曾利华[1];黄兰芳[1];邓	g Lihua[1];Huang Lanfang	医学信息	medical information	35	22	86-88	2022	-
6903	菌合并副溶血弧菌引起食	g caused by Salmonella	滨;李芬香;陈荣华;邹琦;谢	ang;Chen Ronghua;Zou	实用预防医学	actical preventive medici	30	2	202-205	2023	-
6904	起的食物中毒事件的病原	of food poisoning incide	蒋丽;李彩云;高雅;修俊娟	ngli;Li Caiyun;elegant;Xiu	医学动物防制	medical animal control	39	2	198-201	2023	-
6905	门氏菌食源性疾病事件暴发	utbreak of Salmonella E	颜;何玲玲;王乐;汪洋;张先	ling;Wang Le;Wang Yan	实用预防医学	actical preventive medici	30	1	84-87	2023	-
6906	沙门氏菌引起的食源性疾	se outbreak caused by S	熊飞;喻瑾;罗禹;肖伦;	g Fei;Yu Jin;Luo Yu;Xiao	中国食品卫生杂志	inese Food Hygiene Jour	35	2	289-293	2023	).13590/j.cjfh.2023.02.023

Sheet2: English publication						
No.	Title	Authors	Journal/Book	Publication Year	PMID	DOI
1	ns of Salmonella anatum iso	FOURNIER J.	Bull Soc Pathol Exot Filiale	1947	20270442	-
2	nfections in Shanghai durin	FOURNIER J, TUO MK.	Chin Med J	1949	18128807	-
3	East, with especial referen	J, SUNG C, KOANG NK,	Chin Med J	1950	15434883	-
4	SEROTYPES ISOLATED	JANG CT, CHAN-TOEH C	J Trop Med Hyg	1964	14135619	-
5	ntrahepatic typhoid carrier	McFadzean AJ, Ong GB.	Br Med J	1966	5940221	10.1136/bmj.1.5503.1567
6	with Salmonella choleraesu	Huang CT, Lo CB.	J Hyg (Lond)	1967	20475876	1017/s0022172400045654
7	laboratory patterns of typho	i FH, Wang HC, Wang W	st Asian J Trop Med Publi	1971	5002825	-
8	a worthington transmitted b	WK, Chau PY, Tse D, Teo	J Hyg (Lond)	1976	1069811	1017/s0022172400055650
9	and bacteria in the etiology	uinnan G, Portnoy B, Olson	J Infect Dis	1977	198481	10.1093/infdis/136.3.383
10	consumption in Hong Kong	PY, Shortridge KF, Huang	J Hyg (Lond)	1977	265345	1017/s002217240005614x
11	Salmonellosis in Hong Kon	Chau PY, Huang CT.	Public Health	1977	854567	6/s0033-3506(77)80005-x
12	d its possible role in the co	Chau PY, Tse D, Sin WK	J Hyg (Lond)	1977	264495	1017/s0022172400055996
13	consumption in Hong Kong	PY, Shortridge KF, Huang	J Hyg (Lond)	1977	265345	1017/s002217240005614x
14	sis, brucellosis, tuberculosi	Shanks RA, McAllister TA,	Br Med J	1977	843867	10.1136/bmj.1.6062.676
15	Salmonellosis in Hong Kon	Chau PY, Huang CT.	Public Health	1977	854567	6/s0033-3506(77)80005-x
16	d its possible role in the co	Chau PY, Tse D, Sin WK	J Hyg (Lond)	1977	264495	1017/s0022172400055996
17	clinical specimens in Taip	Chung TC.	Min Guo Wei Sheng Wu X	1978	583034	-
18	les, gallbladder wall and je	el PH, Su JF, Chiu TC, Ch	Min Guo Wei Sheng Wu X	1978	581572	-
19	ella johannesburg in Hong	hau PY, Wong WT, Fok Y	J Hyg (Lond)	1978	252536	1017/s0022172400025225
20	tis in Hong Kong--a clinica	Chang WK, Fok TF, Teoh-	J Trop Med Hyg	1979	469979	-
21	I2S-positive Salmonella ser	Chau PY, Huang CT.	Microbiol Immunol	1979	481258	348-0421.1979.tb00449.x
22	openhagen infection in pig	Sawa H, Hirai K.	Nihon Juigaku Zasshi	1981	7026856	10.1292/jvms1939.43.277
23	arriers in the Chinese army	Show YS, Chao SH, Tai FH	Guo Wei Sheng Wu Ji Mia	1982	7183423	-
24	to chloramphenicol-resista	Jing Q.	ghua Liu Xing Bing Xue Z	1982	7185448	-
25	ics of human Salmonella st	Hu ZL.	ghua Liu Xing Bing Xue Z	1982	7185447	-
26	g resistance of salmonella	u FS, Chuech LL, Shen YI	Guo Wei Sheng Wu Ji Mia	1983	6675900	-
27	onella typhi isolated from	-	ghua Liu Xing Bing Xue Z	1983	6675857	-
28	outbreak of Salmonella ariz	Zhou QS.	nghua Yu Fang Yi Xue Za	1983	6675986	-
29	of Salmonella strains in H	Yu WB.	ghua Liu Xing Bing Xue Z	1984	6518483	-
30	ical survey on an outbreak	Xu WX.	ghua Liu Xing Bing Xue Z	1984	6744401	-
31	stant Salmonellae isolated f	Ling J, Chau PY, Rowe B.	Epidemiol Infect	1987	3678394	1017/s0950268800067777
32	almonella isolates from dia	Ling J, Chau PY.	Epidemiol Infect	1987	3315706	1017/s0950268800067789

33	tance of S. typhi and an ou	Zheng QS.	ghua Liu Xing Bing Xue Z	1987	3442791	-
34	stant Salmonellae isolated f	Ling J, Chau PY, Rowe B.	Epidemiol Infect	1987	3678394	1017/s0950268800067777
35	age typing and antimicrobi	Xiao QL.	ghua Liu Xing Bing Xue Z	1987	3449213	-
36	almonella isolates from dia	Ling J, Chau PY.	Epidemiol Infect	1987	3315706	1017/s0950268800067789
37	nella isolated during an epi	Li ZX.	Zhonghua Yi Xue Za Zhi	1988	3228727	-
38	n of a typhoid outbreak in	, You SL, Chuang YC, Hu	Int J Epidemiol	1989	2656560	10.1093/ije/18.1.254
39	nical bacteria isolated in T	JL, Wang JJ, Wang AM, L	Guo Wei Sheng Wu Ji Mia	1989	2791722	-
40	al gastroenteritis in paediat	BC, Tam J, Ng MH, Yeung	J Hosp Infect	1989	2575634	16/0195-6701(89)90075-3
41	on of typhoid and paratyph	Wang DS.	ghua Liu Xing Bing Xue Z	1989	2516479	-
42	f antibiotic resistance in S	deen F, Teoh-Chan CH, Li	Eur J Epidemiol	1989	2504618	10.1007/BF00156832
43	n of a typhoid outbreak in	, You SL, Chuang YC, Hu	Int J Epidemiol	1989	2656560	10.1093/ije/18.1.254
44	of Salmonella typhi-muriu	Chen KC.	ghua Liu Xing Bing Xue Z	1989	2661008	-
45	d clinical evaluation of Sa	n TY, Huang SC, Huang	Changgeng Yi Xue Za Zhi	1990	2282566	-
46	Septicaemia in Hong Kong	, Cheng AF, Duthie R, Co	J Antimicrob Chemother	1990	2347772	0.1093/jac/25.suppl_c.115
47	comial infection by Salmon	Shi J.	ghua Liu Xing Bing Xue Z	1990	2261617	-
48	tions on an outbreak of typ	Yin Y.	ghua Liu Xing Bing Xue Z	1990	2124514	-
49	to Salmonella typhimuriu	C, Xie HH, Tan XP, Wan	J Diarrhoeal Dis Res	1990	2243183	-
50	Septicaemia in Hong Kong	, Cheng AF, Duthie R, Co	J Antimicrob Chemother	1990	2347772	0.1093/jac/25.suppl_c.115
51	oning caused by multiple S	ing MJ, Hsieh SC, Lin MI	Guo Wei Sheng Wu Ji Mia	1991	1845344	-
52	in infants with Salmonella	Huang SH, Chen SH, Hsu	Min Guo Xiao Er Ke Yi Xu	1991	1823512	-
53	remia strains grow in bloo	Chiang TM, Chang TY.	nghua Yi Xue Za Zhi (Tai	1991	1848461	-
54	duction of Hong Kong isol	, Zhou GM, Woo TH, Fre	J Antimicrob Chemother	1991	1816184	10.1093/jac/28.6.877
55	y of septicaemia in hospita	AF, Fok TF, Duthie R, Fre	J Trop Med Hyg	1991	1942206	-
56	duction of Hong Kong isol	, Zhou GM, Woo TH, Fre	J Antimicrob Chemother	1991	1816184	10.1093/jac/28.6.877
57	oning caused by multiple S	ing MJ, Hsieh SC, Lin MI	Guo Wei Sheng Wu Ji Mia	1991	1845344	-
58	y of septicaemia in hospita	AF, Fok TF, Duthie R, Fre	J Trop Med Hyg	1991	1942206	-
59	r of age at a children's hos	talergs O, Changchawalit S	Trans R Soc Trop Med Hyg	1991	1781004	16/0035-9203(91)90389-g
60	f childhood diarrhea in Bei	, He X, de Hua G, Ge YA,	J Clin Microbiol	1991	1993771	1128/jcm.29.1.90-95.1991
61	remia strains grow in bloo	Chiang TM, Chang TY.	nghua Yi Xue Za Zhi (Tai	1991	1848461	-
62	l Salmonella infection by g	Wang SJ, Liao SQ, Hsu CY	oxiong Yi Xue Ke Xue Za	1992	1294760	-
63	vein thrombosis: report of	ang YC, Young CD, Cheng	Kansenshogaku Zasshi	1992	1402120	ogakuzasshi1970.66.1125
64	f Salmonella serotypes in s	Peng CF.	oxiong Yi Xue Ke Xue Za	1992	1619701	-
65	uct stones: a cause of chron	n RC, Cheng AF, Sung JY	Am J Gastroenterol	1992	1519582	-
66	oprim-resistance in Salmon	, Hui YW, Cheng AF, Fre	Pathology	1992	1437292	109/00313029209063171

67	f Salmonella serotypes in s	Peng CF.	oxiong Yi Xue Ke Xue Za	1992	1619701	-
68	8 cases of nontyphoid Saln	Huang TC, Leu HH.	Changgeng Yi Xue Za Zhi	1993	8313209	-
69	ctious diarrhoea in Hong K	Ling JM, Cheng AF.	J Trop Med Hyg	1993	8459483	-
70	urrently with the first prese	, Cohen MG, Ho AK, Cher	Br J Rheumatol	1993	8422563	093/rheumatology/32.1.66
71	utbreak of Salmonella typh	Wu SX, Tang Y.	Chin Med J (Engl)	1993	8222893	-
72	l poisoning by Salmonella l	Li HB.	nghua Yu Fang Yi Xue Za	1993	8243173	-
73	ctious diarrhoea in Hong K	Ling JM, Cheng AF.	J Trop Med Hyg	1993	8459483	-
74	805 children with Salmonel	Wu S, Fan SZ, Lin F.	Zhonghua Yi Xue Za Zhi	1993	8389228	-
75	-typhi Salmonella: analysis	, Yang PH, Shieh WB, Las	Clin Infect Dis	1994	7803634	10.1093/clinids/19.4.693
76	infection in renal transplar	uang CC, Lai MK, Chu SH	Transplant Proc	1994	8066703	-
77	lla infections in infants and	Yang MT, Chi CS.	nghua Yi Xue Za Zhi (Tai	1994	8087721	-
78	osthetic valve endocarditis	Lai LP, Shyu KG, Kuan P,	Angiology	1994	8129208	77/000331979404500312
79	y of human salmonellosis i	uen KY, Yam WC, Lee TY	Epidemiol Infect	1994	7995352	1017/s0950268800068436
80	infection in renal transplar	uang CC, Lai MK, Chu SH	Transplant Proc	1994	8066703	-
81	n in 20 provinces, autonom	He XQ, Liu XL, Yang L.	nghua Yu Fang Yi Xue Za	1994	7842866	-
82	pericarditis and empyema:	Chen KJ, Tseng HH, Yang	nghua Yi Xue Za Zhi (Tai	1995	8854443	-
83	nella psoas abscess--a case	iao YS, Shih HN, Hsu RW	Changgeng Yi Xue Za Zhi	1995	7641111	-
84	cotic aneurysm of the aorta	CW, Huang JJ, Chuang Y	J Infect	1995	7636278	6/s0163-4453(95)80007-7
85	due to non-typhi salmonella	MY, Wang JH, Chen YS.	Clin Infect Dis	1996	8909837	10.1093/clinids/23.4.743
86	ess caused by Salmonella: a	TH, Jeng CJ, Su SC, Wan	nghua Yi Xue Za Zhi (Tai	1996	8803311	-
87	ne disease outbreaks in Tai	Y, Huang HC, Wang TK,	J Formos Med Assoc	1996	8688712	-
88	eningitis complicated by br	Huang LT.	Clin Infect Dis	1996	8825008	10.1093/clinids/22.1.194
89	e 10-year analysis of 134 c	CH, Tseng HH, Chen KJ, L	Scand J Infect Dis	1996	8792485	109/00365549609049070
90	iarrhoea in hospitalized ch	on DJ, Nelson EA, Lau D,	Trop Med Int Health	1996	8911454	365-3156.1996.tb00095.x
91	Salmonella enterica seroty	YM, Kam KM, Ma CH, V	Int J Antimicrob Agents	1996	18611751	6/s0924-8579(96)00316-0
92	ibiotic susceptibilities of sa	Kam KM.	Chin Med J (Engl)	1996	8758287	-
93	tients with diarrhea in Suix	Chen ZD, Shen XS, Lu H.	ghua Liu Xing Bing Xue Z	1996	9387572	-
94	the infection of Salmonella	Zhou G, Ru WP, Zhang J.	ghua Liu Xing Bing Xue Z	1996	9387571	-
95	iarrhoea in hospitalized ch	on DJ, Nelson EA, Lau D,	Trop Med Int Health	1996	8911454	365-3156.1996.tb00095.x
96	due to non-typhi salmonella	MY, Wang JH, Chen YS.	Clin Infect Dis	1996	8909837	10.1093/clinids/23.4.743
97	ibiotic susceptibilities of sa	Kam KM.	Chin Med J (Engl)	1996	8758287	-
98	in a six-month-old infant: a	CS, Chiu CH, Lin TY, Lin	Changgeng Yi Xue Za Zhi	1997	9397614	-
99	inical experience of third-g	Huang LT, Ko SF, Lui CC.	Acta Paediatr	1997	9350883	651-2227.1997.tb14806.x
100	ftriaxone therapy for childr	Chiu CH, Lin TY, Ou JT.	Changgeng Yi Xue Za Zhi	1997	9260371	-

101	almonella-infected aneurys	Luo CY, Yang YJ.	J Formos Med Assoc	1997	9170822	-
102	ella infection--conservativ	ui HF, Chiu KH, Leung K	Can J Surg	1997	9030084	-
103	f Salmonella in some anim	G, Zhao G, Wang C, Sun	Wei Sheng Yan Jiu	1997	10325636	-
104	e among clinical isolates o	, Wang SM, Wu JJ, Huang	r J Clin Microbiol Infect D	1998	10052556	10.1007/s100960050213
105	advanced human immuno	PR, Hsieh SM, Liu CJ, Ch	J Formos Med Assoc	1998	9830279	-
106	ystemic lupus erythematos	Luo SF, Wu YJ, Wang CM	Clin Rheumatol	1998	9776109	10.1007/BF01451006
107	lasty in Salmonella coxitis:	S, Ueng WN, Shih CH, Lu	Changgeng Yi Xue Za Zhi	1998	9607275	-
108	is in bone marrow transpla	, Yuen KY, Wong SS, Le	Clin Infect Dis	1998	9709891	10.1086/514659
109	Salmonella enterica seroty	M, Koo IC, Kam KM, Che	J Clin Microbiol	1998	9620402	CM.36.6.1693-1699.1998
110	ntary tract microbes in bon	, Chen FF, Wong SS, Lau	Diagn Microbiol Infect Dis	1998	9554172	6/s0732-8893(97)00213-7
111	ystemic lupus erythematos	Luo SF, Wu YJ, Wang CM	Clin Rheumatol	1998	9776109	10.1007/BF01451006
112	Salmonella enterica seroty	M, Koo IC, Kam KM, Che	J Clin Microbiol	1998	9620402	CM.36.6.1693-1699.1998
113	Salmonella isolates by sero	ng LC, Yang DI, Cheng C	Acta Paediatr Taiwan	1999	10927958	-
114	ion-associated hemophago	PI, Huang LM, Lee CY, L	Acta Paediatr Taiwan	1999	10910545	-
115	ction of non-typhoidal Sal	Chiu CH, Lin TY, Ou JT.	Int J Clin Pract	1999	10665125	-
116	subdural empyema in chil	n MH, Huang YC, Chou M	J Microbiol Immunol Infec	1999	10650495	-
117	of antibiotic resistance in T	, Lauderdale TL, Yeh LL,	J Microbiol Immunol Infec	1999	10650488	-
118	n of septicemia and periton	Tain YL, Lin G, Cher TW.	Pediatr Nephrol	1999	10603131	10.1007/s004670050710
119	ting aortic aneurysm and s	Chung SL, Ding YA.	onghua Yi Xue Za Zhi (Tai	1999	10575811	-
120	ospinal fluid of patients wi	Chiu CH, Ou JT.	Clin Infect Dis	1999	10452664	10.1086/517773
121	lar activity of antibiotics a	Chiu CH, Lin TY, Ou JT.	Int J Antimicrob Agents	1999	10389647	6/s0924-8579(99)00038-2
122	monella iliopsoas abscess:	Yu WL.	onghua Yi Xue Za Zhi (Tai	1999	10367486	-
123	l tool for analysis of sporad	, Lin JS, Hu HH, Liu PR, V	J Appl Microbiol	1999	10347870	.1365-2672.1999.00720.x
124	oleraesuis bacteremia in so	L, Su YC, Hwang KP, Tsa	Kaohsiung J Med Sci	1999	10330799	-
125	nan salmonellosis during 1	Tsai JJ, Hwang KP, Lu PL,	Kaohsiung J Med Sci	1999	10224836	-
126	y using selenite brilliant gr	Shen HC, Li AM, Chen Cy	J Clin Microbiol	1999	10565941	CM.37.12.4120-4123.1999
127	monella iliopsoas abscess:	Yu WL.	onghua Yi Xue Za Zhi (Tai	1999	10367486	-
128	l tool for analysis of sporad	, Lin JS, Hu HH, Liu PR, V	J Appl Microbiol	1999	10347870	.1365-2672.1999.00720.x
129	oleraesuis bacteremia in so	L, Su YC, Hwang KP, Tsa	Kaohsiung J Med Sci	1999	10330799	-
130	nan salmonellosis during 1	Tsai JJ, Hwang KP, Lu PL,	Kaohsiung J Med Sci	1999	10224836	-
131	r in children: a fourteen-ye	u CH, Tsai JR, Ou JT, Lin	Acta Paediatr Taiwan	2000	10910556	-
132	nfection after a Chinese yea	ung TG, Chiu NY, Horng	J Microbiol Immunol Infec	2000	10806963	-
133	ping of Salmonella enterica	o YM, Kam KM, Hoa NT,	J Clin Microbiol	2000	10618104	8/JCM.38.1.292-300.2000
134	itis in a teaching hospital i	un W, Chan WT, Lee HC,	Acta Paediatr Taiwan	2001	11729707	-

135	among clinical isolates of S	Kuo AJ, Chia JH, Sun CF,	Epidemiol Infect	2001	11693497	1017/s0950268801005957
136	soning cases in Taiwan by p	Tsen HY, Lin JS.	J Appl Microbiol	2001	11442716	.1365-2672.2001.01343.x
137	of ciprofloxacin as second	eh SM, Hsiao CF, Chen M	AIDS	2001	11317004	002030-200103300-00016
138	is of the hip caused by Sal	hiu CH, Lin TY, Luo CC,	Ann Trop Paediatr	2001	11284255	080/02724930020028993
139	of Salmonella enterica sero	ng JM, Chan EW, Cheng A	J Infect	2001	11531322	10.1053/jinf.2001.0806
140	e typhimurium from Hong	ing JM, Koo IC, Cheng AF	Scand J Infect Dis	2001	11345219	080/003655401300077289
141	iterica serotype Typhi varia	ng AM, Wong SS, Tsoi HW	J Clin Microbiol	2001	11230457	CM.39.3.1190-1194.2001
142	of Salmonella enterica sero	ng JM, Chan EW, Cheng A	J Infect	2001	11531322	10.1053/jinf.2001.0806
143	ents with biliary atresia: in	Ni YH, Lee PI, Hsu HY, L	Pediatr Surg Int	2001	11527173	10.1007/s003830000573
144	c of China, 1999: estimati	H, Park JK, Pan YF, Kim Y	J Infect Dis	2001	11372030	10.1086/320729
145	e typhimurium from Hong	ing JM, Koo IC, Cheng AF	Scand J Infect Dis	2001	11345219	080/003655401300077289
146	iterica serotype Typhi varia	ng AM, Wong SS, Tsoi HW	J Clin Microbiol	2001	11230457	CM.39.3.1190-1194.2001
147	: evidence for interspecies	hiu CH, Ko WC, Chuang C	J Formos Med Assoc	2002	12645197	-
148	almonella enterica serovar	, Chu C, Chia JH, Kuo AJ,	Microbiol Immunol	2002	12597357	348-0421.2002.tb02770.x
149	of chicken hatcheries in Ta	ee TE, Wang EM, Cho TJ,	J Microbiol Immunol Infec	2002	12542249	-
150	uated Salmonella enterica s	CF, Lin HC, Liao CL, Sytw	J Microbiol Immunol Infec	2002	12542248	-
151	Salmonella typhimurium:	Su DH, Huang TS.	Thyroid	2002	12490081	089/105072502320908376
152	onella typhi osteomyelitis	F, Szabó G, Abed R, Lová	Scand J Infect Dis	2002	12477333	080/00365540260348590
153	am-negative bacilli: clinica	iang PC, Su TM, Tsai YD,	Clin Neurol Neurosurg	2002	12445926	6/s0303-8467(02)00103-8
154	ated by empyema caused b	o WT, Wang CC, Chu ML	Eur J Pediatr	2002	12424580	.1007/s00431-002-1051-4
155	d Salmonella enterocolitis	LH, He CC, Jaing TH, Luo	Pediatr Surg Int	2002	12415367	.1007/s00383-002-0830-3
156	irysm presenting as acute p	JK, Lin JL, Huang CC, Yu	Ren Fail	2002	12380916	10.1081/jdi-120013974
157	nal failure in a patient with	VR, Chang CT, Yen TH, L	Ren Fail	2002	12212834	10.1081/jdi-120006781
158	-typhoid Salmonella infect	YY, Su LH, Wu TL, Chan	J Microbiol Immunol Infec	2002	12099339	-
159	y for non-typhoid Salmone	H, Huang YC, Chiu CH, Li	J Microbiol Immunol Infec	2002	12099342	-
160	epidemiology, timing of a	N, Chang CJ, Cheng BC, L	Clin Neurol Neurosurg	2002	12140104	6/s0303-8467(02)00052-5
161	emia in children with nonty	MC, Wang SM, Wu JJ, Ch	r J Clin Microbiol Infect D	2002	12072940	.1007/s10096-002-0715-3
162	r salmonella infection in sy	CH, Chen CY, Ou LS, Hua	J Rheumatol	2002	12064838	-
163	esistant Salmonella enterica	I, Chu C, Su LH, Wu WY,	ntimicrob Agents Chemoth	2002	11959613	AAC.46.5.1604-1606.2002
164	nonella within hepatocellul	ee CC, Poon SK, Chen GH	Dig Dis Sci	2002	11911347	0.1023/a:1017924321058
165	uinolone resistance in Saln	LH, Chu C, Chia JH, Kuo A	N Engl J Med	2002	11832529	10.1056/NEJMoa012261
166	erial pathogens isolated fro	, Yu YJ, Tai WL, Kao WL	Chang Gung Med J	2003	15008323	-
167	a subspecies houtenae in a	JS, Chen PY, Lau YJ, Chi	J Microbiol Immunol Infec	2003	14723260	-
168	al abscess in a child: case r	I, Chen PT, Chiu YC, Che	Pediatr Infect Dis J	2003	14506379	.inf.0000083828.70413.fd

169	in perinatal and childhood	, Hung PL, Ho HH, Chang	QJM	2003	14500862	10.1093/qjmed/hcg128
170	f aortic aneurysm infected	B, Tsay YG, Wang SS, Ch	Br J Surg	2003	12945075	10.1002/bjs.4170
171	onella mycotic aneurysm of	CY, Hong GJ, Lee KC, Tsa	Eur J Cardiothorac Surg	2003	12895636	6/s1010-7940(03)00294-x
172	roiditis in a patient with ch	hang H, Peng MY, Ho CL.	Ann Hematol	2003	12879283	.1007/s00277-003-0702-5
173	onship of causative pathog	Wang SM, Yang YJ, Tsai C	Microbiol Immunol Infec	2003	12741732	-
174	g an occult mycotic aneury	Chen KS, Tsai MF, Ng SH,	Scand J Infect Dis	2003	12751718	0/00365540210000026997
175	patients without acquired	B, Tsay YG, Chen RJ, Ch	Clin Infect Dis	2003	12652381	10.1086/367932
176	es and rapid spread of plas	VC, Chiu CH, Tsai SH, Wu	Emerg Infect Dis	2003	12643826	10.3201/eid0903.010410
177	xone resistance in Salmon	CH, Chu C, Wang MH, Ch	ntimicrob Agents Chemoth	2003	12543660	3/AAC.47.2.563-567.2003
178	adult male-successful mana	Singh S, Aggarwal A, Agg	Orthop Surg (Hong Kong	2003	14676351	77/230949900301100220
179	nes of fluoroquinolone-resi	I, Chan EW, Lam AW, Ch	ntimicrob Agents Chemoth	2003	14576119	AC.47.11.3567-3573.2003
180	(Dongjiang) water, with p	Ho KC, Chow YL, Yau JT.	Chemosphere	2003	12867174	6/S0045-6535(03)00481-8
181	ed gallbladder pseudolithia	n HH, Huang YK, Zheng C	Acta Paediatr Taiwan	2004	15868813	-
182	s of Salmonella enterica se	ma A, Ishihara K, Asai T,	Jpn J Infect Dis	2004	15623959	-
183	cteremia in adult patients w	Hsu RB, Chen RJ, Chu SH	Am J Med Sci	2004	15599326	6/s0002-9629(15)33940-9
184	reak caused by Salmonella	IJ, Tung YL, Hwang SJ, L	BMC Infect Dis	2004	15541186	10.1186/1471-2334-4-48
185	monella enterica serotype C	H, Wu TL, Su LH, Liu JW,	Emerg Infect Dis	2004	15498176	10.3201/eid1009.030596
186	bscess from Salmonella: a c	in HY, Chiang PC, Chen K	J Reprod Med	2004	15493571	-
187	c granulomatous disease: re	Weng JD, Shyur SD.	Acta Paediatr Taiwan	2004	15493736	-
188	aused by Salmonella enteri	u CP, Chen SY, Chen SC,	J Formos Med Assoc	2004	15278192	-
189	patients with systemic lup	C, Yao TC, Yeh KW, Hua	J Rheumatol	2004	15229953	-
190	rysms: clinical outcome an	B, Chen RJ, Wang SS, Ch	J Vasc Surg	2004	15218459	10.1016/j.jvs.2004.03.020
191	aracteristics of typhoid fev	u CP, Chen YC, Chang SC	Microbiol Immunol Infec	2004	15181493	-
192	oleraesuis infection in a m	Chiu S, Chiu CH, Lin TY.	Microbiol Immunol Infec	2004	15181491	-
193	hildren: clinical manifestati	I, Hsieh KS, Liu YC, Wu T	Pediatr Gastroenterol Nut	2004	15097441	005176-200405000-00011
194	erotype choleraesuis resista	u C, Chia JH, Wu TL, Lin	Lancet	2004	15094275	6/S0140-6736(04)16003-0
195	erica Typhimurium and Ch	ng D, Shyr JM, Yu KW, V	Emerg Infect Dis	2004	15078598	10.3201/eid1001.030171
196	nic abscess in southern Ta	CH, Leu HS, Hu TH, Liu	Microbiol Immunol Infec	2004	15060686	-
197	the epidemiology, clinical	YC, Hung PL, Lu CH, Ch	Brain Dev	2004	15030905	6/S0387-7604(03)00122-0
198	f serogroup D nontyphoidal	Su LH, Hung CC, Chen K	J Clin Microbiol	2004	14715794	8/JCM.42.1.415-417.2004
199	disease in Hong Kong, usin	JS, Yu LM, Glass RI, Para	Epidemiol Infect	2004	15310163	1017/s0950268804002250
200	cotic aneurysms of the thor	AC, Cheng SW, Ho P, Poo	Eur J Cardiothorac Surg	2004	15201010	).1016/j.ejcts.2004.03.035
201	disease in Hong Kong, usin	JS, Yu LM, Glass RI, Para	Epidemiol Infect	2004	15310163	1017/s0950268804002250
202	tance gene cassettes among	uo S, Zhang X, Yamasaki S	Microbiol Immunol	2004	15383699	348-0421.2004.tb03473.x

203	food borne pathogens and	ang M, Ran L, Wang Z, Li	Wei Sheng Yan Jiu	2004	15098478	-
204	the human isolates of Salı	u CS, Schroeter A, Guerra	J Appl Microbiol	2005	16313420	.1365-2672.2005.02749.x
205	fever and typhoid hepatitis	H, Tseng SP, Teng LJ, Ho	Epidemiol Infect	2005	16274504	017/S0950268805004048
206	n-resistant Salmonella ente	S, Lee YT, Guo SM, Hsue	J Infect	2005	16230198	0.1016/j.jinf.2004.12.011
207	lmonella in a woman with	H, Chen CP, Chiang S, Ch	J Reprod Med	2005	16220772	-
208	neningitis associated with c	Thien PF, Liaw SC, Fung C	J Clin Microbiol	2005	16208031	CM.43.10.5400-5402.2005
209	ovascular infection due to r	Hsu RB, Lin FY.	QJM	2005	16203825	10.1093/qjmed/hci126
210	a-lactamase-producing clir	Nakazaki N, Hirata Y, Ino	Diagn Microbiol Infect Dis	2005	16165001	diagmicrobio.2005.04.004
211	gen tests based on clinical r	ang YJ, Kuo PH, Wang SM	r J Clin Microbiol Infect D	2005	16096776	.1007/s10096-005-1373-z
212	ella adrenal abscess in an H	PT, Liu CP, Lee CM, Hung	Scand J Infect Dis	2005	16051576	080/00365540510034527
213	sistance in salmonella-infe	in FY, Chen RJ, Hsueh PR	Ann Thorac Surg	2005	16039199	/j.athoracsur.2005.02.046
214	d by multiple organ involv	ang CM, Ko WC, Huang Y	J Infect	2005	16038753	0.1016/j.jinf.2004.08.018
215	es in Northern Taiwan attri	CP, Weng LC, Wang NY	J Clin Microbiol	2005	16000441	CM.43.7.3237-3243.2005
216	illness outbreaks in norther	Chiu SI, Tsai JL, Lee CL, I	J Infect Chemother	2005	15990979	.1007/s10156-005-0383-1
217	with surgical repair of myc	Chang HH, Hsu CP, Lai ST	J Chin Med Assoc	2005	15984820	/S1726-4901(09)70148-0
218	ciprofloxacin resistance in	u CS, Lauderdale TL, Tsa	Emerg Infect Dis	2005	15963294	10.3201/eid1106.041153
219	lla enterica Serovar Choler	KS, Chiu CH, Chu C, Chi	J Clin Microbiol	2005	15956400	CM.43.6.2798-2804.2005
220	nella bacteremia in patients	Hsu RB, Chen RJ, Chu SH	Am J Med Sci	2005	15894865	00441-200505000-00004
221	in Salmonella isolates fror	TL, Chia JH, Chu C, Kuo A	J Antimicrob Chemother	2005	15872047	10.1093/jac/dki116
222	serovar Choleraesuis, a hi	, Hu S, Bao Q, Yu J, Chou	Nucleic Acids Res	2005	15781495	10.1093/nar/gki297
223	rk carcass microbiological	Yeh KS, Chen SP, Lin JH.	J Food Prot	2005	15771166	4315/0362-028x-68.3.458
224	eptibilities of Salmonella a	Tsai HJ, Hsiang PH.	J Vet Med Sci	2005	15699587	10.1292/jvms.67.7
225	parC, and parE in Salmon	s-Dancla E, Cloeckaert A,	ntimicrob Agents Chemoth	2005	15673791	/AAC.49.2.862-863.2005
226	odiscitis in patients withou	Chang IC.	Clin Orthop Relat Res	2005	15662331	blo.0000137561.82099.d5
227	elitis among children in a r	NC, Li WC, Chi H, Lee Y	J Formos Med Assoc	2005	15660174	-
228	infections caused by ceftr	WL, Lee HC, Lee NY, Wa	Clin Infect Dis	2005	15655754	10.1086/426593
229	rent Salmonella species in	ang TK, Ho PL, Wong SS	New Microbiol	2005	16035260	-
230	axime in clinical isolates o	YW, Chu MY, Ma CH, Yu	J Antimicrob Chemother	2005	16033804	10.1093/jac/dki250
231	dicitis caused by an H1-j s	, Chan CY, Woo WL, Wo	J Clin Microbiol	2005	15750137	CM.43.3.1470-1472.2005
232	liated multiple antimicrobi	Guo YC, Du CM, Liu XM	Wei Sheng Yan Jiu	2005	16329607	-
233	a-lactamase-producing clir	Nakazaki N, Hirata Y, Ino	Diagn Microbiol Infect Dis	2005	16165001	diagmicrobio.2005.04.004
234	ute gastroenteritis in hospit	M, Ni YH, Chen HL, Char	J Formos Med Assoc	2006	17185238	/S0929-6646(09)60280-1
235	cteremia due to Salmonella	ng CC, Cheng SN, Lo WT	J Microbiol Immunol Infec	2006	17164956	-
236	one-resistant Salmonella sp	eh KS, Chang CC, Hsuan S	Emerg Infect Dis	2006	17080579	10.3201/eid1202.050629

237	almonella enterica serotype	an SS, Wang JY, Hsueh P	J Microbiol Immunol Infec	2006	17066196	-
238	ren: etiology, clinical mani	C, Chen CC, Chen SY, C	Expert Rev Anti Infect The	2006	17009942	0.1586/14787210.4.4.629
239	ous erythematosus: salmon	ng JJ, Wu KC, Lee WI, Ch	Semin Arthritis Rheum	2006	16887469	6/j.semarthrit.2006.04.003
240	serovar typhimurium phag	u CH, Wang MH, Yeh CM	J Clin Microbiol	2006	16825349	10.1128/JCM.00171-06
241	otype Choleraesuis infecti	Chuang CH, Chiu S, Su L	Pediatrics	2006	16717121	10.1542/peds.2005-2251
242	n a large cohort of patients	H, Hsieh MY, Kuo ML, Li	J Clin Immunol	2006	16783466	.1007/s10875-006-9013-7
243	Salmonella isolates recove	ang YC, Chen YT, Yang C	J Food Prot	2006	16541703	4315/0362-028x-69.3.674
244	ca serotype Choleraesuis in	wang JJ, Hsu CN, Lin LC	Epidemiol Infect	2006	16569283	017/S095026880600608X
245	scherichia coli and Salmon	ang JC, Hsuan-Yuan CH, C	Int J Antimicrob Agents	2006	16621462	/j.ijantimicag.2005.11.020
246	fferent serotypes of clinica	Lai JF, Wang HY, Shiau Y	Diagn Microbiol Infect Dis	2006	16529903	diagmicrobio.2006.01.002
247	obial-resistant Salmonella	S, Abbott J, Kiessling C, A	J Food Prot	2006	16541678	4315/0362-028x-69.3.500
248	lla infection appearing as a	heng SP, Liu CL, Chang Y	J Ultrasound Med	2006	16439795	0.7863/jum.2006.25.2.277
249	arrying bla(CMY-2) amon	Chia JH, Liu SY, Chu C,	J Antimicrob Chemother	2006	16396917	10.1093/jac/dki478
250	A3 in clinical isolates of Sa	K, Ng TK, Tsang D, To W	J Antimicrob Chemother	2006	16943211	10.1093/jac/dkl340
251	monella spp. in Hong Kong	Jin Y, Ling JM.	J Med Microbiol	2006	16914655	10.1099/jmm.0.46637-0
252	tem and pulsed-field gel el	i YJ, Wang YP, Cui SH, J	ghua Liu Xing Bing Xue Z	2006	17415987	-
253	i A isolates from four prov	n KC, Zhong HJ, Zhu FC,	ghua Liu Xing Bing Xue Z	2006	17343181	-
254	rovar paratyphi A occurred	da M, Suzuki M, Kitazume	Kansenshogaku Zasshi	2006	17176854	hogakuzasshi1970.80.674
255	ium multidrug resistance a	Q, Liu WH, Guo AZ, Chen	Wei Sheng Wu Xue Bao	2006	17172030	-
256	inical bacteria isolated fro	L, Zhu X, Ma Y, Li J, She	ong Univ Sci Technolog M	2006	16961303	10.1007/BF02829585
257	A3 in clinical isolates of Sa	K, Ng TK, Tsang D, To W	J Antimicrob Chemother	2006	16943211	10.1093/jac/dkl340
258	ory workers--Huizhou, Gua	Luo HM, Fontaine R; Cent	MMWR Suppl	2006	16645581	-
259	carcinoma in a diabetic an	en CS, Chiu KW, Kuo CM	Liver Int	2006	16629655	.1478-3231.2006.01247.x
260	scherichia coli and Salmon	ang JC, Hsuan-Yuan CH, C	Int J Antimicrob Agents	2006	16621462	/j.ijantimicag.2005.11.020
261	ystitis: A rare presentation	CK, Chin C, Lin HH, Chi	Scand J Infect Dis	2006	16500779	080/00365540500372655
262	usceptibility of Salmonella	, Wang MH, Chu C, Su LH	J Microbiol Immunol Infec	2007	17932601	-
263	mia in previously healthy	Chiu CH, Yen MH, Chang	Pediatr Infect Dis J	2007	17901796	7/INF.0b013e318127189b
264	ella enterica serotype Ente	ng PH, Chiu YL, Hsueh P	J Microbiol Immunol Infec	2007	17639171	-
265	ults with Salmonella enteri	HC, Lee NY, Shih HI, Le	J Microbiol Immunol Infec	2007	17639165	-
266	yphoidal Salmonella isolat	ng YJ, Wang SM, Chiou C	J Microbiol Immunol Infec	2007	17639164	-
267	oidal Salmonella bacteremi	hen TL, Chen YY, Lin ML	J Microbiol Immunol Infec	2007	17639163	-
268	enterica in poultry hatcheri	H, Yeh CM, Chou SJ, Chu	Poult Sci	2007	17626809	10.1093/ps/86.8.1651
269	t suggests a major world-w	Helmuth R, Schroeter A, C	Int J Food Microbiol	2007	17379345	j.ijfoodmicro.2006.05.024
270	acters in household and str	ang HC, Lin CM, Lien YY	Vet Res Commun	2007	17285243	.1007/s11259-007-0009-4

271	erichia coli: genetic correla	, Su LH, Chiu CH, Kuo A.	Int J Antimicrob Agents	2007	17300921	/j.ijantimicag.2006.12.008
272	ectrum beta-lactamase from	LH, Yeh YL, Chu C, Lai JC	Int J Antimicrob Agents	2007	17275265	/j.ijantimicag.2006.11.013
273	ntyphoid Salmonella bacter	NY, Lee NY, Lee HC, Shi	J Intern Med	2007	17222172	.1365-2796.2006.01748.x
274	hoid osteomyelitis of the sp	Khan FY, El-Hiday AH.	Hong Kong Med J	2007	17548921	-
275	monella mycotic aneurysm	g SP, Lai TK, Ng WL, Luk	Hong Kong Med J	2007	17548914	-
276	otype Typhi isolates by puls	uey KY, Chiu AW, Law CI	Foodborne Pathog Dis	2007	17378707	10.1089/fpd.2006.59
277	typhi in asia and molecular	ghui Y, Anh DD, Canh DG	ntimicrob Agents Chemoth	2007	17908946	10.1128/AAC.00294-07
278	ce of foodborne pathogens	G, Zhou X, Jiao X, Qian X.	Foodborne Pathog Dis	2007	17883311	10.1089/fpd.2007.0088
279	rne disease outbreaks in Ch	g S, Duan H, Zhang W, Li	MS Immunol Med Microb	2007	17666075	.1574-695X.2007.00305.x
280	a serovars isolated from me	Jiao XA, Liu XW, Chen X	Wei Sheng Wu Xue Bao	2007	17552224	-
281	monella mycotic aneurysm	g SP, Lai TK, Ng WL, Luk	Hong Kong Med J	2007	17548914	-
282	otype Typhi isolates by puls	uey KY, Chiu AW, Law CI	Foodborne Pathog Dis	2007	17378707	10.1089/fpd.2006.59
283	ombining serological tests i	ng M, Kim D, Ochiai RL,	Epidemiol Infect	2007	17217551	017/S0950268806007801
284	ibility of Salmonella enteric	g YJ, Su LH, Hsu CH, Fu Y	J Microbiol Immunol Infec	2008	19255695	-
285	e thyroiditis and ruptured ac	HR, Tsao SM, Wu YL, Ya	Transplant Proc	2008	19100483	.transproceed.2008.06.057
286	outhern Taiwan: a medical	Hwang KP, Kuo HC, Hua	Pediatr Neonatol	2008	19054916	/S1875-9572(08)60025-X
287	onella infection in heart tra	Hsu RB, Lin FY.	Am J Med Sci	2008	19011395	/MAJ.0b013e31816a8973
288	stics of mycotic aneurysms	u PJ, Lee CH, Lee FY, Liu	J Microbiol Immunol Infec	2008	18787739	-
289	monella and Salmonella en	h CM, Lo DY, Su YC, Hor	Poult Sci	2008	18648047	10.3382/ps.2008-00018
290	Salmonella infection in a p	KM, Lin TY, Chen JS, Ch	Lupus	2008	18250138	.1177/0961203307085165
291	monella enterica serovar Ty	ang KC, Chang YF, Chiou	J Clin Microbiol	2008	18077649	10.1128/JCM.00709-07
292	ss of the ulna caused by Sa	p KC, Lam YL, Chang RY	Hong Kong Med J	2008	18382026	-
293	ce of Salmonella from retai	V, Zhi S, Cui SH, Xi ML, Y	nghua Yu Fang Yi Xue Za	2008	19178840	-
294	is in a food-borne outbreak	Hu QH, Lan QX, Zhang Q	nghua Yu Fang Yi Xue Za	2008	19176144	-
295	is on non-typhoidal Salmo	Chen JL, Ke CW, Guo C, C	ghua Liu Xing Bing Xue Z	2008	19173963	-
296	assessment of Salmonella	Fan YX, Liu XM.	nghua Yu Fang Yi Xue Za	2008	18844078	-
297	istance tendency of pathoge	EB, Guo TS, Bao CM, Li	Zhonghua Nei Ke Za Zhi	2008	18843955	-
298	health emergency in four re	Li X, Huang J, Zhang H.	BMC Public Health	2008	18803860	10.1186/1471-2458-8-319
299	cus sequence typing system	Li Y, Ji R, Wang Y, Jiang T	Wei Sheng Yan Jiu	2008	18421863	-
300	Salmonella enterica seroty	un Z, Hu C, Jin S, Guo Y,	Emerg Infect Dis	2008	18325271	10.3201/eid1403.070857
301	gene-positive Salmonella is	g J, Wang Q, Pei ZF, Zhan	Int J Antimicrob Agents	2008	18178065	/j.ijantimicag.2007.10.023
302	ver in south China: Guangx	Jin Y.	J Infect Dev Ctries	2008	19741290	10.3855/jidc.223
303	ella enteritidis B in a famil	nn SR, Lee SJ, Wang YH,	J Microbiol Immunol Infec	2009	20422139	-
304	sk factor of Salmonella cho	Chen CM, Hsu YM, Chio	J Environ Health	2009	20063610	-

305	rovar Braenderup, but not t	hen SW, Chang YF, Weng	BMC Microbiol	2009	20017951	10.1186/1471-2180-9-264
306	Salmonella enterica serotyp	Lin HH, Chen YS, Tien N	J Formos Med Assoc	2009	19864196	6/S0929-6646(09)60403-4
307	cin and ceftriaxone among	Carlos C, Tan BH, Kuma	ntimicrob Agents Chemoth	2009	19332677	10.1128/AAC.01297-08
308	ltiple joints in a girl with a	uang YC, Tsai MH, Chiu C	Pediatr Neonatol	2009	19326836	6/S1875-9572(09)60027-9
309	rian abscess in a patient wi	hao AS, Wang TH, Liu LP	Fertil Steril	2009	19304287	16/j.fertnstert.2008.04.037
310	carcasses in Taiwan and de	g YC, Chang CC, Hsuan S	J Food Prot	2009	19205458	0.4315/0362-028x-72.1.14
311	bacteremia in children wit	TY, Liu MC, Hsu CF, Lin	r J Clin Microbiol Infect D	2009	18949496	.1007/s10096-008-0641-0
312	ce of Salmonella enterica s	singhe G, So T, VAN PH,	Epidemiol Infect	2009	18474127	017/S0950268808000745
313	n antibiotic-resistant Salm	Jin Y, Ling JM.	Jpn J Infect Dis	2009	19934534	-
314	hogens in clinical specimen	Ling JM.	Hong Kong Med J	2009	19258630	-
315	ular typing of Salmonella e	J, Gu BK, Chen M, Ran L	ghua Liu Xing Bing Xue Z	2009	20193231	-
316	ric infectious diseases duri	Q, Gao ZY, Dou XF, Zhan	nghua Yu Fang Yi Xue Za	2009	20137562	-
317	n antibiotic-resistant Salm	Jin Y, Ling JM.	Jpn J Infect Dis	2009	19934534	-
318	istics of typhoid and paraty	M, Jin CG, Mao GH, Shi Y	ghua Liu Xing Bing Xue Z	2009	19642380	-
319	le ribs and thoracic vertebr	X, Wang J, Wu C, Mehbo	Spine J	2009	19660991	1016/j.spinee.2009.06.013
320	e typhimurium from outpat	hang LS, Xia SL, Yang WZ	Biomed Environ Sci	2009	19618691	6/S0895-3988(09)60036-2
321	-resistant Salmonella in fis	Broughton EI, Walker DG.	Foodborne Pathog Dis	2009	19292686	10.1089/fpd.2008.0196
322	enterica subspecies enteric	eng S, Chen X, Pan W, C	Vet Microbiol	2009	19128897	016/j.vetmic.2008.11.015
323	resistance gene cassettes an	, Nawaz MS, Cheng CM, I	Appl Environ Microbiol	2009	19074612	10.1128/AEM.02054-08
324	bility of Salmonella isolate	Huang L, Zhang J, Guo W	J Clin Microbiol	2009	19073874	10.1128/JCM.01099-08
325	r, China, in 1994-1995 and	XL, Sun XR, Han XH, Che	Environ Pollut	2009	19027211	016/j.envpol.2008.10.004
326	enterica isolates from infan	Z, Hu C, Jin S, Li F, Guo Y	J Antimicrob Chemother	2009	18984647	10.1093/jac/dkn452
327	urface waters by QPCR in	u YJ, Zhang CM, Wang X	Environ Monit Assess	2009	18931927	.1007/s10661-008-0602-1
328	ositis and dermatomyositis:	HH, Liou LB, Chen JY, K	Rheumatology (Oxford)	2010	20837496	093/rheumatology/keq279
329	t graft implantation for a S	S, Chiu KM, Chu SH, Hua	Ann Thorac Surg	2010	20667384	6/j.athoracsur.2009.09.085
330	eat analysis for fine typing	I, Watanabe H, Tung SK, T	Int J Food Microbiol	2010	20573417	j.ijfoodmicro.2010.06.001
331	yphoid Salmonella in a pati	C, Wang JT, Tsai CW, Wu	Yonsei Med J	2010	20376908	0.3349/ymj.2010.51.3.472
332	a serovars from different br	ou CS, Lee CY, Yeh CM, Y	BMC Microbiol	2010	20307324	10.1186/1471-2180-10-86
333	almonella within the gluteu	Chen YS, Chia WT.	Orthop Nurs	2010	20142688	/NOR.0b013e3181c8cd1c
334	sceptibility of salmonellae	C, Lai YH, Tung KC, Chi	J Vet Diagn Invest	2010	20093681	77/104063871002200107
335	aditional marketplaces in T	wang WZ, Tsai SJ, Hsih Y	Poult Sci	2010	20075291	10.3382/ps.2009-00001
336	ella-infected left ventricula	, Hung WC, Chiu CA, Wa	Kaohsiung J Med Sci	2010	20040471	/S1607-551X(10)70006-1
337	eus with preservation of gr	ang JW, Tsai HL, Yang L	Clin Nephrol	2010	20040356	10.5414/cnp73072
338	l burkholderiosis associate	W, Lee R, Que TL, Tse H,	Clin Vaccine Immunol	2010	20445006	10.1128/CVI.00053-10
