# Supplementary material for: Nationwide trends and features of human salmonellosis outbreaks in China
Source: Emerg Microbes Infect. 2024 Jun 26;13(1):2372364. doi: 10.1080/22221751.2024.2372364 (PMC11259058; doi:10.1080/22221751.2024.2372364)
Supplement: Supplemental Material [file TEMI_A_2372364_SM2458.pdf]

Table S2: Extracted information obtained from literature included in the systematic evaluation or meta-analysis
Sheet1: Extracted information for systematic review
Sheet2: Extracted information for meta-analysis
Sheet3: Reference standard
Sheet4: Antimicrobial annotation

Sheet1: Extracted information for systematic review

Publication information

No.	Author	Author_E.T.	Title	Title_E.T.	Journal	Journal_E.T.	Volume	Issue	Page	Year	DOI
1	霞,王安娜,周勇,和	a Wang,Zhou Yo	菌食物中毒分离株	stics of a London	品安全质量检测学	d Safety and Qua	12	6	2474-2478	2021	-
2	张要,滕毅,王玲,刘	Teng Yi,Wang Lin	门菌引起的食物中	of food poisoni	预防医学情报杂志	eventive Medicin	37	1	48-52,57	2021	-
3	丁丁,梅建华,陈秀	ing,Mei Jianhua,C	沙门菌食源性疾	nella Orenenbur	预防医学	reventive Medici	33	6	602-606	2021	.cnki.issn2096-5087.2
4	苏良,刘晓蕾,杨柳	Liang,Liu Xiaolei,Y	门菌食物中毒事件	traceability of Sal	现代预防医学	rn preventive me	48	6	1103-1106	2021	-
5	苏良,刘晓蕾,杨柳	Liang,Liu Xiaolei,Y	门菌食物中毒事件	traceability of Sal	现代预防医学	rn preventive me	48	6	1103-1106	2021	-
6	夏威,施惠军,陆孙	v,Shi Huijun,Lu Xu	菌食物中毒的流行	analysis of Salmo	医学动物防制	edical animal con	37	2	175-178,182	2021	.7629/yxdwfz202102
7	徐云龙,宋士利,郑	ong,Song Shili,Zh	肠炎沙门菌食源性	almonella Enteriti	中国食品卫生杂志	e Food Hygiene	33	5	583-586	2021	13590/j.cjfh.2021.05.
8	孙汝春,刘	Sun Ruchun,Liu C	于一起肠炎沙门菌	n investigation of	医学动物防制	edical animal con	37	2	149-151	2021	.7629/yxdwfz202102
9	余金,许玉成,张桂	Jinjin,Xu Yuchen	菌食物中毒事件的	n incident of Sal	医学动物防制	edical animal con	37	1	23-26	2021	.7629/yxdwfz202101
10	茜,田羊娜,陈磊,罗	Ming,Chen Lei,Lu	起的沙门菌食源性	ne disease outbre	医学动物防制	edical animal con	37	1	88-91	2021	.7629/yxdwfz202101
11	海遥,郑姝豪,黄	ng Shuhao,Huan	食物中毒的致病	ogenic bacteria i	饮食保健	iet and health ca	-	24	258	2021	-
12	惠平,潘丽峰,胡	g,Pan Lifeng,Hu	校一起沙门菌食物	d poisoning incid	中国食品卫生杂志	e Food Hygiene	33	5	627-632	2021	13590/j.cjfh.2021.05.
13	李卫华,马佳平,	Weihua,Ma Jiapi	源性疾病暴发的流	of foodborne di	健康大视野	Healthy vision	-	19	23,25	2021	-
14	洪轩,蓝岳云,张茜	uevun,Zhang Qia	门菌食源性疾病暴	nella Enteritidis f	预防医学	reventive Medici	33	6	621-623	2021	.cnki.issn2096-5087.2
15	康锉,曹应琼,梅丽	h,Cao Yingqiong,I	沙门氏菌食物中毒	ella Food Poisoni	医学信息	nedical informatic	34	10	158-160	2021	/j.issn.1006-1959.202
16	王世萍	Wang Shiping	引起的沙门氏菌食	poisoning incide	生虫病与感染性疾	eases and infecti	19	1	38-41	2021	-
17	陈桂芳,陈雪芳,	Guifang,Chen Xu	引起家庭食源性疾	foodborne disea	医学食疗与健康	l Diet Therapy an	19	15	221-223	2021	-
18	珍,炊慧霞,李艳芬	i Huixia,Li Yanfen	引起食源性疾病暴	e disease outbre	中国卫生检验杂志	ournal of Health	31	9	1037-1039,1043	2021	-
19	封正娟,谭琳,陈	engjuan,Tan Lin,C	门菌暴发疫情快速	sis analysis of a	预防医学情报杂志	eventive Medicin	37	7	1007-1010	2021	-
20	杨洋,次仁卓玛,陈	ang,Tsering Doln	菌污染引起的群体	e diarrhea cause	医药前沿	Medical Frontier	11	18	193-194	2021	-
21	吴燕飞,刘东妹,方	ei,Liu Dongmei,F	引起的学校食源性	odborne illness o	中国食品卫生杂志	e Food Hygiene	33	5	595-599	2021	13590/j.cjfh.2021.05.
22	吴鹏程,施菊萍,施	acheng,Shi Jupin	菌食物中毒的病原	alysis of a case of	医学动物防制	edical animal con	37	5	434-437	2021	.7629/yxdwfz202105
23	川,赵士程,勾红梅	o Shicheng,Gouh	相变异株引起食源	eak caused by a	预防医学情报杂志	eventive Medicin	37	6	809-812	2021	-
24	张文艳,杜小莉,周	Wenyan,Du Xiaol	食物中毒事件的病	eability of a salm	医学动物防制	edical animal con	37	4	405-407	2021	.7629/yxdwfz202104
25	王雨荷,	Wang Yuhe,	污染引起的食源性	dborne illness ca	山西医药杂志	anxi Medical Jou	49	24	3400-3402	2020	/j.issn.0253-9926.202
26	王艳玲,	Wang Yanling,	门菌致食物中毒的	food poisoning	中国城乡企业卫生	e in urban and ru	35	12	12-14	2020	286/j.1003-5052.2020
27	婧,姚婧,李廷荣,黄	ao Jing,Li Tingror	门菌食物中毒1例调	is food poisoning	现代医药卫生	rn medicine and	36	23	3886-3888	2020	/j.issn.1009-5519.202
28	贝,王虹玲,陈艳,虞	Hongling,Chen Y	门菌污染三明治引	d by Salmonella	中国食品卫生杂志	e Food Hygiene	32	6	708-712	2020	13590/j.cjfh.2020.06.
29	伟,秦友燕,韩丹丹	Youyan,Han Dand	沙门氏菌引起的食	oning incident ca	广西医科大学学报	Guangxi Medical	37	11	2074-2078	2020	0/j.cnki.45-1211/r.202
30	文娟,薛琳,周坤,吴	n Xue Lin,Zhou K	肠炎沙门氏菌食物	incident caused	实用预防医学	cal preventive me	27	11	1379-1380	2020	/j.issn.1006-3110.202
31–171	[illegible]										

Outbreak information — Spatiotemporal background (Time; Space) — Patient information — Case collection

No.	Year (onset)	HB / y	Month	Duration	Economic region	Province	Prefecture	Latitude / °N	Longitude / °E	Setting	nber of New Case	ple Exposed at sta	ttack Rate (AR) /	Minimum age / y	Maximum age / y	Mean Age / y	Mean Age_SD / y	Male	Female	ender Ratio (GR) /
1	2020	1	8	1	Eastern	Guangdong	Guangzhou	23.08	113.14	Restaurant	4	N/A	N/A	N/A	N/A	N/A	N/A	N/A	N/A	N/A
2	2019	2	7	1	Western	Sichuan	Chengdu	30.4	104.04	School	26	270	9.60	18	37	22	N/A	17	9	65.38
3	2019	2	6	4	Eastern	Zhejiang	Lishui	28.27	119.54	Residential area	18	280	6.43	45	70	N/A	N/A	9	9	50.00
4	2019	2	6	N/A	Central	Hunan	Changsha	28.12	112.59	Hospital	7	12	58.33	N/A	N/A	N/A	N/A	N/A	N/A	N/A
5	2019	2	11	N/A	Central	Hunan	Changsha	28.12	112.59	School	40	N/A	N/A	N/A	N/A	N/A	N/A	N/A	N/A	N/A
6	2018	3	6	8	Eastern	Jiangsu	Zhangjiagang	31.52	120.32	School	47	4200	1.12	19	22	N/A	N/A	22	25	46.81
7	2017	4	8	3	Eastern	Zhejiang	Hangzhou	30.16	120.1	Nursing home	23	407	5.65	21	80	43	N/A	17	6	73.91
8	2016	5	5	1	Eastern	Hebei	Xinji	37.54	115.12	Residential area	3	4	75.00	N/A	N/A	N/A	N/A	N/A	N/A	N/A
9	2019	2	8	2	Eastern	Guangdong	Shenzhen	22.33	114.07	Restaurant	11	16	68.75	14	50	25	N/A	1	10	9.09
10	2019	2	5	2	Eastern	Hebei	Shijiazhuang	38.02	114.3	Restaurant	68	104	65.38	2	78	N/A	N/A	41	27	60.29
11	2021	0	5	2	Western	Sichuan	Luzhou	28.54	105.24	Restaurant	6	N/A	N/A	N/A	N/A	N/A	N/A	N/A	N/A	N/A
12	2018	3	9	3	Eastern	Shanghai	Pudong	31.14	121.29	Restaurant	51	1270	4.02	11	18	12	N/A	21	30	41.18
13	2019	2	8	1	Eastern	Shandong	Dezhou	37.26	116.17	Office	15	98	15.31	21	67	45.12	4.66	9	6	60.00
14	2019	2	9	3	Eastern	Zhejiang	Quzhou	28.58	118.52	School	71	3602	1.97	N/A	N/A	N/A	N/A	45	26	63.38
15	2020	1	6	2	Western	Sichuan	Chengdu	30.4	104.04	Residential area	7	22	31.82	3	76	47	N/A	1	6	14.29
16	2019	2	5	1	Western	Sichuan	Xichang	27.54	102.16	Residential area	28	200	14.00	22	66	N/A	N/A	14	14	50.00
17	2018	3	6	1	Eastern	Guangdong	Chaozhou	23.4	116.38	mobile street stall	9	N/A	N/A	14	65	N/A	N/A	3	6	33.33
18	2019	2	2	3	Central	Henan	Pingdingshan	33.44	113.17	Restaurant	62	260	23.85	N/A	N/A	N/A	N/A	28	34	45.16
19	2019	2	6	3	Western	Sichuan	Panzhihua	26.34	101.43	Restaurant	27	N/A	N/A	N/A	N/A	N/A	N/A	N/A	N/A	N/A
20	2019	2	7	2	Western	Tibet	Ali	32.5	80.11	School	15	N/A	N/A	8	12	N/A	N/A	5	10	33.33
21	2020	1	10	8	Eastern	Guangdong	Zhuhai	22.17	113.34	School	74	3974	1.86	N/A	N/A	N/A	N/A	41	33	55.41
22	2019	2	4	2	Eastern	Jiangsu	Zhangjiagang	31.52	120.32	Factory	10	N/A	N/A	N/A	N/A	N/A	N/A	N/A	N/A	N/A
23	2019	2	5	2	Western	Sichuan	Guangyuan	32.28	105.51	Residential area	27	30	90.00	N/A	N/A	N/A	N/A	N/A	N/A	N/A
24	2018	3	7	N/A	Central	Anhui	Hefei	31.52	117.17	Office	20	N/A	N/A	N/A	N/A	N/A	N/A	N/A	N/A	N/A
25	2020	0	5	2	Central	Anhui	Bengbu	32.56	117.21	Restaurant	55	85	64.71	4	78	N/A	N/A	39	16	70.91
26	2020	0	5	2	Eastern	Tianjin	Jizhou	39.02	117.12	Restaurant	4	12	33.33	N/A	N/A	N/A	N/A	N/A	N/A	N/A
27	2020	0	7	4	Western	Chongqing	Shapingba	29.35	106.33	mobile street stall	12	N/A	N/A	21	39	29.8	N/A	4	8	33.33
28	2019	1	6	3	Eastern	Zhejiang	Zhoushan	30.01	122.06	School	37	N/A	N/A	16	18	N/A	N/A	23	14	62.16
29	2018	2	8	4	Western	Guangxi	Guilin	25.17	110.17	Restaurant	295	520	56.73	8	71	26	N/A	183	112	62.03
30	2019	1	10	5	Central	Jiangxi	Nanchang	28.4	115.55	mobile street stall	596	N/A	N/A	1	89	N/A	N/A	231	365	38.76
31–171	[illegible]																			

Incidence rates (IR) of designated symptoms / % — Laboratory test: Pathogen (*Salmonella*); Identification Method (Y: yes) — Clinical management: Treatment; Prognosis — Event cluster

No.	Diarrhea	Fever	Stomach cramps	Nausea	Vomiting	Headache	Source (food)	Serogroup	Serovar	Sequence type	nicrobial resistance	elective culture (S	emical identificatio	munological test (	ld gel electrophore	rase chain reactio	scopic examinatio	Other	Medication	Recovery	Death	Cluster type
1	100.00	100.00	100.00	N/A	N/A	N/A	Frozen food	E1	London	N/A	MP, NAL, CZO, SA	Y	Y	Y	Y	Y	Y	N/A	CAZ	4	0	Food poisoning
2	76.92	61.54	46.67	100.00	100.00	100.00	Meat products	D.	Enteritidis	N/A	N/A	Y	Y	Y	N/A	N/A	N/A	N/A	N/A	N/A	N/A	Food poisoning
3	100.00	38.89	100.00	11.11	5.56	16.67	Meat products	C1	Oranienburg	N/A	N/A	Y	N/A	Y	Y	N/A	N/A	N/A	N/A	N/A	N/A	Food poisoning
4	100.00	57.14	71.43	100.00	28.57	N/A	Pastries	D1	Enteritidis	N/A	NAL	Y	Y	Y	Y	N/A	N/A	N/A	N/A	N/A	N/A	-associated infection
5	100.00	80.00	90.00	100.00	20.00	N/A	Pastries	D1	Enteritidis	N/A	AL, AMP, SAM, C	Y	Y	Y	Y	N/A	N/A	N/A	N/A	N/A	N/A	Food poisoning
6	100.00	93.62	72.34	53.19	25.53	8.51	Pastries	D1	Enteritidis	N/A	MK, GEN, ATM, TC	Y	Y	Y	Y	N/A	N/A	N/A	N/A	N/A	N/A	Food poisoning
7	86.96	91.30	8.70	N/A	100.00	N/A	N/A	D1	Enteritidis	N/A	N/A	Y	Y	Y	Y	N/A	N/A	N/A	LVX, CRO	23	0	-associated infection
8	100.00	100.00	100.00	100.00	100.00	N/A	Egg products	D1	Enteritidis	N/A	N/A	Y	Y	Y	Y	N/A	Y	N/A	N/A	N/A	N/A	Food poisoning
9	100.00	100.00	100.00	90.91	63.64	81.82	rocessed grain produ	D1	Enteritidis	N/A	N/A	Y	N/A	Y	N/A	N/A	N/A	N/A	N/A	N/A	N/A	Food poisoning
10	85.29	100.00	95.59	100.00	44.12	100.00	Aquatic products	D1	Enteritidis	N/A	N/A	Y	N/A	Y	N/A	Y	N/A	N/A	N/A	N/A	N/A	Food poisoning
11	100.00	100.00	33.33	N/A	N/A	N/A	Pastries	D1	Gueuletapee	N/A	N/A	Y	Y	Y	N/A	N/A	N/A	N/A	N/A	N/A	N/A	Food poisoning
12	100.00	41.18	78.43	35.29	23.53	58.82	Pastries	C1	Mbandaka、Thomps	N/A	N/A	Y	N/A	Y	Y	N/A	N/A	N/A	N/A	N/A	N/A	Food poisoning
13	86.67	100.00	93.33	80.00	66.67	40.00	Meat products	D1	Enteritidis	N/A	N/A	Y	Y	Y	N/A	N/A	N/A	N/A	N/A	15	0	Food poisoning
14	63.38	45.07	91.55	38.03	30.99	N/A	Pastries	D1	Enteritidis	N/A	N/A	Y	Y	Y	Y	N/A	N/A	N/A	N/A	N/A	N/A	Food poisoning
15	100.00	57.14	70.43	N/A	28.57	N/A	Egg products	B	Typhimurium	N/A	N/A	Y	Y	Y	N/A	N/A	N/A	N/A	N/A	N/A	N/A	Food poisoning
16	82.14	42.85	64.29	46.43	57.14	N/A	Meat products	E1	London	N/A	N/A	Y	N/A	Y	N/A	N/A	N/A	N/A	N/A	28	0	Food poisoning
17	100.00	100.00	100.00	N/A	100.00	100.00	Fruits	E1	Weltevreden	N/A	N/A	Y	N/A	Y	N/A	N/A	N/A	N/A	N/A	N/A	N/A	Food poisoning
18	77.42	70.97	53.23	48.39	N/A	56.45	Others	D1	Enteritidis	N/A	AMP, NAL	Y	Y	Y	Y	Y	N/A	MALDI-TOF-MS	N/A	N/A	N/A	Food poisoning
19	100.00	100.00	100.00	100.00	100.00	N/A	Meat products	B	Stanley	N/A	N/A	Y	Y	Y	Y	Y	N/A	N/A	N/A	N/A	N/A	Food poisoning
20	100.00	100.00	100.00	63.20	84.20	N/A	Meat products	C1	N/A	N/A	N/A	Y	Y	Y	N/A	Y	Y	N/A	N/A	N/A	N/A	Food poisoning
21	83.78	63.51	78.38	43.24	22.97	31.08	Egg products	D1	Enteritidis	N/A	N/A	Y	Y	Y	Y	Y	N/A	N/A	N/A	74	0	Food poisoning
22	100.00	100.00	50.00	40.00	20.00	N/A	Egg products	B	Typhimurium	N/A	), CTT, AMK, GEN,	Y	Y	Y	Y	Y	N/A	N/A	N/A	N/A	N/A	Food poisoning
23	100.00	N/A	33.33	70.37	55.56	37.04	Meat products	B	Typhimurium 4,5,12,i:	N/A	AMP, TET, CHL, SX	Y	Y	Y	Y	Y	N/A	N/A	N/A	N/A	N/A	Food poisoning
24	100.00	100.00	N/A	N/A	N/A	N/A	Pastries	D1	Enteritidis	N/A	NAL, ERY	Y	Y	Y	Y	Y	N/A	N/A	N/A	N/A	N/A	Food poisoning
25	61.82	94.55	50.91	60.00	47.27	N/A	Aquatic products	E1	Kalina	N/A	N/A	Y	N/A	Y	N/A	N/A	N/A	N/A	N/A	55	0	Food poisoning
26	100.00	75.00	75.00	N/A	N/A	N/A	Meat products	D1	Enteritidis	N/A	N/A	Y	Y	Y	N/A	N/A	Y	N/A	N/A	4	0	Food poisoning
27	100.00	100.00	91.67	50.00	58.33	83.33	Pastries	D1	Enteritidis	N/A	N/A	Y	Y	Y	N/A	Y	N/A	N/A	N/A	12	0	Food poisoning
28	70.27	54.05	51.35	45.95	37.84	N/A	Pastries	D1	Enteritidis	N/A	NAL	Y	Y	Y	Y	N/A	N/A	N/A	N/A	N/A	N/A	Food poisoning
29	100.00	99.32	89.15	55.93	64.41	88.81	Meat products	D1	Enteritidis	N/A	N/A	Y	Y	Y	Y	N/A	N/A	N/A	N/A	N/A	N/A	Food poisoning
30	98.32	87.75	74.16	60.07	44.80	64.77	Pastries	D1	Enteritidis	N/A	NAL, SOX, SM, AM	Y	Y	Y	Y	Y	N/A	N/A	N/A	N/A	N/A	Food poisoning
31–171	[illegible]																					

172	史志瑛.	Shi Zhiying.	沙门氏菌引起的食物中毒	ch on food poiso	科技展望	echnology Outlo	-	18	210-210	2014	/j.issn.1672-8289.201	2013	1	8	1	Central	Shanxi	Datong
173	徐基明,查正时.	Jiming.Check tim	一起聚餐引发食物中毒的	h food poisoning	热带病与寄生虫学	Diseases and Par	-	4	252-253	2014	/j.issn.1672-2302.201	2012	2	8	3	Central	Anhui	Huangshan
174	[illegible]	Duan Yi Ban Xiuhu	[illegible]	ion of a typhoid f	应用预防医学	ed preventive me	20	2	87-89	2014	-	2013	1	7	42	Western	Guangxi	Hechi
175	[illegible]	ong.Wang Bin.Li R	一起食物中毒的调查	nment of a Food	解放军预防医学杂志	Medicine of the P	32	6	527-528	2014	-	2011	3	9	1	Eastern	Beijing	N/A
176	李静.	Li Jing.	[illegible]	na caused by nor	中国农村卫生	ural health in chir	-	z2	379-379	2014	-	2013	1	5	2	Eastern	Shandong	Taian
177	[illegible]	u Zhonglin.Cao Li	[illegible]	od poisoning cau	中国卫生检验杂志	ournal of Health	24	12	18,161,822	2014	-	2013	1	5	2	Eastern	Hebei	Shijiazhuang
178	[illegible]	u Li Hongye Meli	[illegible]	nalysis of a case	中国卫生检验杂志	ournal of Health	24	23	3400-3401	2014	-	2013	1	8	N/A	Western	Xinjiang	Urumqi
179	吉喜军.	Ji Xijun.	[illegible]	of salmonella fo	新医学信息文摘（E	al information ab	-	6	182-182	2014	/j.issn.1671-3141.201	2012	2	6	3	Western	Inner mongolia	Wulanchabu
180	王艳,张江萍.	a Yan.Zhang Jianc	[illegible]	ritidis food poiso	贵州医药	Guizhou Medicin	-	2	164-165	2014	j.ISSN.1000-744X.20	2011	3	5	2	Western	Guizhou	Guiyang
181	陈国庆.	Chen Guoqing.	[illegible]	nella food poiso	国保健营养（上旬刊	ealth Nutrition (fi	24	2	1088-1089	2014	.issn.1004-7484(s).20	2012	2	6	1	Northeastern	Jilin	Changchun
182	韩永红.	Han Yonghong.	[illegible]	a case of Salmo	中国城乡企业卫生	he in urban and ru	29	4	53-54	2014	-	2013	1	9	2	Eastern	Hebei	Tangshan
183	[illegible]	Shiyong.Liu Li.Par	[illegible]	s of a salmonella	医学动物防制	edical animal con	30	10	1146-1147	2014	.7629/yxdwfz201410	2013	1	6	3	Eastern	Hebei	Shijiazhuang
184	姬晓红,孙玲.	Xiaohong.Sun Lin	[illegible]	a and Salmonell	中国伤残医学	ese Disability Me	-	13	299-300	2014	14/j.cnki.cjotadm.201	2013	1	8	N/A	Western	Shaanxi	Weinan
185	姬晓红,孙玲.	Xiaohong.Sun Lin	[illegible]	a and Salmonell	中国伤残医学	ese Disability Me	-	13	299-300	2014	14/j.cnki.cjotadm.201	2013	1	9	N/A	Western	Shaanxi	Huayin
186	罗华.	Luo Hua.	[illegible]	reserved eggs c	国保健营养（中旬刊	alth nutrition (mid	24	4	2502-2503	2014	-	2009	5	6	1	Western	Chongqing	Wanzhou
187	[illegible]	g.Chen Liping.W	[illegible]	oisoning inciden	疾病监测与控制	surveillance and	8	8	488-489	2014	-	2012	2	5	2	Eastern	Guangdong	Qingyuan
188	[illegible]	ist attraction.Wu	[illegible]	Enteritidis food	应用预防医学	ed preventive me	-	3	194-194	2014	-	2014	0	12	3	Central	Jiangxi	Pingxiang
189	孙磊.	Sun Lei.	[illegible]	of Salmonella E	中国实用医药	ese Practical Me	-	23	273-274	2014	-	2012	2	5	3	Central	Henan	Luoyang
190	陆月敏.	Lu Yuemin.	食物中毒的流行病学	ory investigation	中国卫生检验杂志	ournal of Health	24	12	1788-1789	2014	-	2013	1	5	2	Eastern	Zhejiang	Yongkang
191	[illegible]	zi.Huang Bolin.Lu	[illegible]	salmonella food	内科	Internal Medicin	9	5	592-594	2014	-	2011	3	7	1	Western	Guangxi	Baise
192	王福春.	Wang Fuchun.	[illegible]	lysis of Food Pois	医学动物防制	edical animal con	30	10	1148-1149	2014	.7629/yxdwfz201410	2013	1	8	2	Western	Guangxi	Jingxi
193	[illegible]	ng.Chen Yu.Wan	[illegible]	od poisoning ca	疾病预防控制通报	evention and Cor	29	5	63-64	2014	215/j.cnki.jbyfkztb.14	2014	0	1	3	Western	Guizhou	Qianxinan
194	[illegible]	anhua.Yang Zushi	[illegible]	food poisoning c	疾病监测	isease surveillan	29	11	920-924	2014	/j.issn.1003-9961.201	2012	2	10	10	Western	Yunnan	Xishuangbanna
195	[illegible]	Dan.Cao Guoke.H	的食源性疾病暴发	orne disease ou	华南预防医学	hina Preventive	40	3	270-273	2014	13217/j.scjpm.2014.0	2013	1	6	2	Eastern	Guangdong	Guangzhou
196	[illegible]	nachunlin Xu Xiu	[illegible]	on Paratyphoid A	学信息文摘（连续	rmation abstract	-	30	341-341,343	2014	/j.issn.1671-3141.201	2010	4	7	61	Eastern	Jiangsu	Yangzhou
197	[illegible]	Zhenchun.Anbing	[illegible]	sis of a case of f	中外健康文摘	and Foreign Heal	-	13	74-74,75	2014	-	2013	1	7	3	Northeastern	Liaoning	Shenyang
198	[illegible]	Jiding.Xia Yingpi	[illegible]	y Salmonella Ent	中国食品卫生杂志	e Food Hygiene	26	5	489-491	2014	13590/j.cjfh.2014.05.	2011	3	11	N/A	Eastern	Zhejiang	Ningbo
199	[illegible]	Pan Ganglei.Ming	[illegible]	onella Enteritidis	浙江预防医学	ng Preventive Me	26	4	402-404	2014	-	2013	1	5	3	Eastern	Zhejiang	Ningbo
200	[illegible]	ong.Liu Siyuan.H	[illegible]	ood poisoning c	华南预防医学	hina Preventive	40	3	274-275	2014	13217/j.scjpm.2014.0	2013	1	6	2	Eastern	Guangdong	Dongguan
201	[illegible]	a Weiwang.Zhang	[illegible]	of Salmonella Typ	上海预防医学	hai Preventive M	26	10	537-538	2014	-	2013	1	12	3	Eastern	Zhejiang	Ningbo
202	[illegible]	.Wang Lumei.To	[illegible]	orne disease out	中华预防医学杂志	urnal of Preventiv	47	2	160-163	2013	ma.j.issn.0253-9624.2	2010	3	5	3	Eastern	Jiangsu	Xuzhou
203	王琴	Wang Qin	[illegible]	n of a foodborne	疾病预防控制通报	evention and Cor	-	3	65-66	2013	-	2012	1	6	5	Western	Gansu	Lanzhou
204	[illegible]	ue.Liu Tianhui.Kai	[illegible]	a caused by Salm	新医学信息文摘（E	al information ab	-	20	344-344	2013	/j.issn.1671-3141.201	2011	2	8	1	Western	Sichuan	Panzhihua
205	文天平.	Wen Tianping.	[illegible]	ood poisoning ca	中国卫生产业	hina health indus	-	14	98,100	2013	-	2012	1	3	N/A	Central	Hunan	Liuyang
206	秦田秀.	Qin Tianxiu.	[illegible]	ella food poisoni	广西医学	Guangxi Medicin	-	7	929-930	2013	5/j.issn.0253-4304.20	2009	4	8	6	Western	Guangxi	Guilin
207	周青华	Zhou Qinghua	[illegible]	Nandan County	中国农村卫生	ural health in chir	-	z1	319-320	2013	-	2011	2	4	7	Western	Guangxi	Hechi
208	周立新.	Zhou Lixin.	[illegible]	nd analysis of a b	国保健营养（中旬刊	alth nutrition (mid	-	8	532-533	2013	-	2012	1	1	2	Eastern	Shandong	Dezhou
209	杨凤娟.	Yang Fengjuan.	[illegible]	lla food poisonin	中外健康文摘	and Foreign Heal	-	49	44-45	2013	-	2012	1	11	3	Western	Yunnan	Dali
210	[illegible]	ang.Gao Bin.Liu Y	[illegible]	caused by Salmo	宁夏医科大学学报	Ningxia Medical	35	2	232,封3	2013	/j.issn.1674-6309.201	2011	2	5	2	Western	Ningxia	Yinchuan
211	梁艳.	Liang Yan.	[illegible]	ase of food pois	中外健康文摘	and Foreign Heal	-	33	226-227	2013	-	2011	2	10	1	Western	Yunnan	Baoshan
212	吴俊霞.	Wu Junxia.	[illegible]	of food poisoni	江苏卫生保健	iangsu Health Ca	15	5	29	2013	/j.issn.1008-7338.201	2012	1	6	2	Eastern	Jiangsu	Zhenjiang
213	蒋奎.	Jiang Kui.	[illegible]	tigation and anal	职业卫生与病伤	ational health and	28	4	249-251	2013	/j.issn.1006-172X.201	2011	2	8	4	Western	Sichuan	Panzhihua
214	杨梅霞	Yang Meixia	[illegible]	enic bacteria in	国保健营养（中旬刊	alth nutrition (mid	-	5	534-534	2013	-	2008	5	5	N/A	Western	Sichuan	Dazhou
215	王慧华,王立华.	a Huihua.Wang L	[illegible]	ning caused by a	蛇志	snake ambition	25	3	287-289	2013	/j.issn.1001-5639.201	2010	3	8	2	Eastern	Jiangsu	Changzhou
216	张金华,王梓红.	Jinhua.Wang Gu	[illegible]	se of food poiso	南大学学报（医学	heast University (	32	1	29-31	2013	/j.issn.1671-6264.201	2011	2	8	1	Eastern	Hebei	Chengde
217	[illegible]	g.Yan Jun.Chen X	[illegible]	od poisoning ca	江苏卫生保健	iangsu Health Ca	15	6	25-26	2013	/j.issn.1008-7338.201	2013	0	4	4	Eastern	Jiangsu	Huaian
218	[illegible]	Wei Dejiang.Li Jin	[illegible]	sis of a case of sal	实用预防医学	cal preventive me	20	6	716-718	2013	/j.issn.1006-3110.201	2011	2	9	1	Eastern	Jiangsu	Nanjing
219	[illegible]	wei.Xiao Qianru.J	[illegible]	ng caused by Sal	检验医学与临床	tory medicine and	10	0	216	2013	-	2012	1	7	N/A	Eastern	Shanghai	N/A
220	[illegible]	.Wang Chuntong	[illegible]	nalysis of a case	医药杂志（下半月	ournal (Second H	42	16	883-884	2013	-	2012	1	9	2	Eastern	Hebei	Cangzhou
221	[illegible]	n.Li Zhaoguo.Che	[illegible]	ood poisoning ca	中国民康医学	People's Health	25	14	109,111	2013	/j.issn.1672-0369.201	2011	2	7	N/A	Eastern	Shandong	Jinan
222	杜航.	Du Hang.	[illegible]	of food poisoni	社区医学杂志	of Community M	11	22	73-74	2013	-	2013	0	7	2	Central	Hubei	Xiangyang
223	李建庆.	Li Jianqing.	[illegible]	port on a case of	健康大视野	Healthy vision	21	18	690-690	2013	-	2012	1	7	2	Eastern	Zhejiang	Shaoxing
224	[illegible]	navar·Bahti.Liu Q	[illegible]	oisoning caused	疾病预防控制通报	evention and Cor	-	3	63-64,72	2013	-	2012	1	7	6	Western	Xinjiang	Kashgar
225	陈玉红	Chen Yuhong	[illegible]	case of food pois	检验医学与临床	tory medicine and	10	2	206-207	2013	/j.issn.1672-9455.201	2011	2	12	1	Eastern	Jiangsu	Rugao
226	[illegible]	Youjun.Suo Zhong	[illegible]	mass food poiso	健康必读（中旬刊）	l for health (mid-	12	7	597-597	2013	-	2013	0	5	1	Western	Ningxia	Wuzhong
227	[illegible]	a Shuang.Yang Li	[illegible]	of a food poisoni	中国伤残医学	ese Disability Me	-	12	364-364,365	2013	-	2012	1	8	2	Northeastern	Jilin	Changchun
228	[illegible]	Xie Qingmei.Li Zo	[illegible]	and analysis of a	健康必读（下旬刊）	ead for health (la	-	1	355-355	2013	-	2012	1	4	4	Central	Henan	Pingdingshan
229	王晓东.	Wang Xiaodong	[illegible]	ected food poiso	健康大视野	Healthy vision	21	5	714	2013	-	2012	1	6	2	Central	Hubei	Huanggang
230	[illegible]	Yang Jun.Liu Ping.	[illegible]	f food poisoning	健康大视野	Healthy vision	21	18	691-691	2013	-	2013	0	9	2	Eastern	Shandong	Anqiu
231	李宏.	Li Hong.	[illegible]	a case of food po	医药前沿	Medical Frontier	-	33	359-360	2012	-	2010	2	7	N/A	Western	Chongqing	Tongnan
232	[illegible]	Yan He Lihua Per	[illegible]	of food poisoni	江西医药	Jiangxi Medicine	47	4	360,364	2012	/j.issn.1006-2238.201	2011	1	6	1	Central	Jiangxi	Dexing
233	[illegible]	angdong.Long Xi	[illegible]	oning incident ca	医学动物防制	edical animal con	28	8	926,928	2012	/j.issn.1003-6245.201	2010	2	4	1	Western	Guangxi	Nanning
234	[illegible]	n Wufeng.Liu Hui	[illegible]	food poisoning i	健康大视野	Healthy vision	20	7	274-274	2012	-	2011	1	7	2	Central	Hubei	Enshi
235	[illegible]	Bin.Jin Guicai.Lin	[illegible]	nfectious diarrhe	包头医学院学报	of Baotou Medica	28	1	26-28	2012	/j.issn.1006-740X.201	2009	3	7	9	Western	Inner mongolia	Chifeng
236	戴婧	Dai Jing	[illegible]	oning caused by	青海医药杂志	ghai Medical Jou	42	7	71-72	2012	-	2010	2	6	1	Western	Qinghai	Haidong
237	[illegible]	Zejun.Wu Xian.Zhu	[illegible]	food poisoning i	预防医学情报杂志	eventive Medicin	28	4	298-300	2012	-	2011	1	7	3	Western	Sichuan	Yibin
238	李蓓,魏彩梅.	Li Bei.Wei Caimei	[illegible]	sis of a case of sal	疾病监测与控制	surveillance and	6	7	414-415	2012	-	2011	1	7	3	Western	Shaanxi	Shangluo
239	朱艳梅,汤艳.	u Yanmei.Tang Y	[illegible]	a case of food po	中外健康文摘	and Foreign Heal	-	34	80-81	2012	/j.issn.1672-5085.201	2011	1	7	2	Central	Hubei	Xiaogan
240	[illegible]	Wensheng.Zhan	[illegible]	ing incidents cau	中国初级卫生保健	ary health care in	26	4	63-64	2012	/j.issn.1001-568X.201	2011	1	11	3	Central	Hubei	Xiangyang
241	潘新明.	Pan Xinming.	[illegible]	f Salmonella Ente	海峡预防医学杂志	urnal of Preventiv	18	4	44-45	2012	-	2011	1	9	N/A	Central	Hubei	Wuhan
242	[illegible]	qiang.Deng Zhiai.	[illegible]	of Salmonella Lic	中国热带医学	nese tropical med	12	6	684-687	2012	-	2011	1	7	2	Eastern	Guangdong	Guangzhou
243	[illegible]	ang Wang Xueme	[illegible]	e of food poison	海峡预防医学杂志	urnal of Preventiv	18	1	55-56	2012	-	2010	2	8	1	Eastern	Guangdong	Kaiping
244	[illegible]	.Ye Julian.Li Lin.W	[illegible]	ood poisoning ca	中国卫生检验杂志	ournal of Health	22	5	1060-1062	2012	-	2011	1	8	2	Eastern	Zhejiang	Hangzhou
245	冯裕如.	Feng Yuru.	[illegible]	ood poisoning ca	健康必读（中旬刊）	l for health (mid-	11	10	75-75	2012	-	2011	1	7	3	Western	Sichuan	Suining
246	[illegible]	jun.Li Yaqi.Wang	[illegible]	t a family banque	宁夏医科大学学报	Ningxia Medical	34	2	188-189	2012	/j.issn.1674-6309.201	2011	1	5	2	Western	Ningxia	Guyuan
247	[illegible]	ui Wang Ying Ga	[illegible]	of salmonella fo	中外健康文摘	and Foreign Heal	-	50	171-172	2012	/j.issn.1672-5085.201	2012	0	5	1	Eastern	Shandong	Zibo
248	[illegible]	Wenfeng.Zheng	[illegible]	ood poisoning ca	中国城乡企业卫生	he in urban and ru	27	2	103-104	2012	-	2010	2	8	4	Eastern	Tianjin	Xiqing
249	张耘.	Zhang Yun.	[illegible]	ood poisoning ca	上海预防医学	hai Preventive M	24	2	71-72	2012	/j.issn.1004-9231.201	2011	1	7	2	Eastern	Jiangsu	Suzhou
250	张娟.	Zhang Juan.	[illegible]	tion results in an	安徽预防医学杂志	rnal of Preventive	18	2	154-155	2012	-	2011	1	9	N/A	Central	Anhui	Wuhu
251	王岩,李杨.	Wang Yan.Li Yang	[illegible]	food poisoning c	中外健康文摘	and Foreign Heal	9	15	366-366	2012	/j.issn.1672-5085.201	2011	1	6	1	Central	Henan	Xuchang
252	[illegible]	ng Jianli.Zhong L	[illegible]	od poisoning cau	海峡预防医学杂志	urnal of Preventiv	18	1	58-59	2012	-	2010	2	6	2	Eastern	Fujian	Zhangzhou
253	夏良贤,乔玉林.	Liangxian.Qiao Y	[illegible]	se of food poiso	中国卫生检验杂志	ournal of Health	22	9	2247-2248	2012	-	2008	4	6	1	Central	Henan	Zhumadian
254	[illegible]	Li Dongzhi Li Ho	[illegible]	of food poisonin	中国卫生产业	hina health indus	-	2	95	2012	-	2011	1	4	2	Eastern	Shandong	Zibo
255	[illegible]	n.Zhou Yanjie.Wa	[illegible]	ning caused by Sa	医学信息	medical informati	25	12	438-439	2012	/j.issn.1006-1959.201	2008	4	7	2	Northeastern	Liaoning	Fuxin
256	孙晓红.	Sun Xiaohong.	[illegible]	cation of a Dubli	中外健康文摘	and Foreign Heal	9	49	163-164	2012	/j.issn.1672-5085.201	2011	1	8	3	Northeastern	Liaoning	Beipiao
257	[illegible]	hifei.Li Guangbin	[illegible]	tion of the etiolo	实用预防医学	cal preventive me	19	11	1721-1723	2012	/j.issn.1006-3110.201	2011	1	8	3	Central	Hunan	Shaoyang
258	杨丽桦.	Yang Lihua.	[illegible]	n of an infectiou	亚太传统医药	cific Traditional M	8	4	43-44	2012	/j.issn.1673-2197.201	2010	2	6	N/A	Eastern	Hainan	Haikou
259	[illegible]	nfeng.Zhang She	[illegible]	g middle school	中国学校卫生	hinese school hea	33	6	755-756,759	2012	-	2011	1	12	3	Eastern	Zhejiang	Ningbo
260	[illegible]	hanghai.Yang Wa	[illegible]	ase of food poiso	医学（医学护理分	icine (Medical Ge	33	3	189-190	2012	/j.issn.1001-8883.201	2011	1	2	3	Western	Yunnan	Chuxiong
261	[illegible]	ou Ying.Luo Jiang	[illegible]	of food poisoni	与康复医学（下旬	ehabilitation Med	2	7	249-250	2011	-	2010	1	8	2	Western	Sichuan	Ganzi
262	朱文军.	Zhu Wenjun.	[illegible]	ase of food poiso	江苏预防医学	su Preventive Me	22	2	39-39	2011	/j.issn.1006-9070.201	2010	1	8	2	Eastern	Jiangsu	Huaian
263	孙步武.	Sun Buwu.	[illegible]	poisoning incide	应用预防医学	ed preventive me	17	1	64	2011	/j.issn.1673-758X.201	2010	1	11	7	Western	Guangxi	Baise
264	胡全胜.	Hu Quansheng.	[illegible]	poisoning caused	医学高等专科学校	Shandong Medi	33	5	341-342	2011	/j.issn.1674-0947.201	2010	1	9	3	Eastern	Shandong	Linyi
265	陈洪涛	Chen Honghao	[illegible]	a food poisonin	社区医师（医学专	ity physician (me	13	29	155-156	2011	/j.issn.1007-614x.201	2009	2	6	2	Northeastern	Jilin	Liaoyuan
266	朱翔,包云娟.	u Xiang.Bao Yunju	[illegible]	od poisoning cau	浙江预防医学	ng Preventive Me	23	12	49-50	2011	/j.issn.1007-0931.201	2010	1	3	3	Eastern	Zhejiang	Jinhua
267	彭易根,张正群.	Yigen.Zhang Zhe	[illegible]	Food Poisoning72	现代医药卫生	rn medicine and	27	4	571-572	2011	-	2010	1	6	N/A	Eastern	Jiangsu	Huaian
268	[illegible]	Li Yi.Hou Min.Liu	[illegible]	f food poisoning	内蒙古中医药	ia Traditional Chi	30	4	115-116	2011	/j.issn.1006-0979.201	2008	3	9	N/A	Western	Yunnan	Kunming
269	[illegible]	aojuan.Zheng Xiu	[illegible]	a case of food po	医学动物防制	edical animal con	27	1	56,58	2011	/j.issn.1003-6245.201	2009	2	4	2	Eastern	Hebei	Handan
270	李红.	Li Hong.	[illegible]	case of bacteria	中外医疗	se and foreign m	30	6	102-102	2011	/j.issn.1674-0742.201	2010	1	8	2	Northeastern	Liaoning	Anshan
271	[illegible]	g.Zhu Baoping.T	[illegible]	se of Salmonella	中华流行病学杂志	Journal of Epide	32	7	697-699	2011	ma.j.issn.0254-6450.2	2009	2	8	3	Western	Sichuan	Liangshan
272	高亚色,李恩	Gao Ya color Li E	[illegible]	ning caused by P	中国卫生检验杂志	ournal of Health	21	9	2222-2223,2225	2011	-	2010	1	9	2	Eastern	Fujian	Xiamen
273	[illegible]	a Xiaoliang.Xuan	[illegible]	eak of food pois	海峡预防医学杂志	urnal of Preventiv	17	4	42-44	2011	-	2010	1	10	2	Eastern	Fujian	Xiamen
274	[illegible]	ng.Li Qiumei.Zha	[illegible]	a case of salmone	中国公共卫生管理	public health ma	27	3	309	2011	-	2008	3	7	1	Western	Inner mongolia	Baotou
275	张汉菊.	Zhang Hanju.	[illegible]	of four cases of f	中国卫生检验杂志	ournal of Health	21	11	2754-2755	2011	-	2008	3	5	N/A	Western	Yunnan	Lincang
276	张汉菊	Zhang Hanju	[illegible]	of four cases of f	中国卫生检验杂志	ournal of Health	21	11	2754-2756	2011	-	2011	0	5	N/A	Western	Yunnan	Lincang
277	魏德清.	Wei Deqing.	一起食物中毒调查	poisoning invest	预防医学情报杂志	eventive Medicin	27	3	238	2011	-	2008	3	7	2	Western	Sichuan	Luzhou
278	[illegible]	ngjin.Wei Liming.	[illegible]	ood poisoning ca	应用预防医学	ed preventive me	17	3	191	2011	/j.issn.1673-758X.201	2011	0	4	4	Western	Guangxi	Baise
279	[illegible]	uang Qiujie.Mo G	[illegible]	od poisoning cau	浙江预防医学	ng Preventive Me	23	8	44-45	2011	/j.issn.1007-0931.201	2008	3	7	2	Eastern	Zhejiang	Hangzhou
280	刘金兰.	Liu Jinlan.	[illegible]	meat139Analysi	医学动物防制	edical animal con	27	1	63	2011	/j.issn.1003-6245.201	1984	27	7	3	Western	Inner mongolia	Wulanchabu
281	[illegible]	ua.Ye Julian.Wei J	[illegible]	the etiology of fo	中国预防医学杂志	urnal of Preventiv	12	3	290-291	2011		2009	2	5	3	Eastern	Zhejiang	Jinhua
282	季洪斌.	Ji Hongbin.	[illegible]	nteritidis food p	中国初级卫生保健	ary health care in	25	9	70-71	2011	/j.issn.1001-568X.201	2011	0	4	1	Eastern	Jiangsu	Nantong
283	[illegible]	ingwen Zhang We	[illegible]	caused by Salmo	中国职业医学	e Occupational M	-	S1	88-89	2011	-	2009	2	5	2	Eastern	Guangdong	Shanwei
284	徐里强.	Xu Liqiang.	[illegible]	sis of a collective	中国民康医学	People's Health	23	18	2324	2011	/j.issn.1672-0369.201	2011	0	5	2	Eastern	Jiangsu	Changshu
285	[illegible]	a Junhua.Gao Ha	[illegible]	food poisoning	中国现代医生	dern chinese do	49	30	5,8	2011	/j.issn.1673-9701.201	2008	3	7	2	Eastern	Jiangsu	Wuxi
286	[illegible]	gping.Shao Zhi	[illegible]	sis of a case of sal	中外健康文摘	and Foreign Heal	8	32	50-51	2011	/j.issn.1672-5085.201	2010	1	9	1	Central	Jiangxi	Nanchang
287	[illegible]	bao.Ding Qing.Yi	[illegible]	ence of Salmonel	中国感染控制杂志	ournal of Infectio	10	1	57-58	2011	/j.issn.1671-9638.201	2009	2	3	5	Western	Xinjiang	Urumqi
288	王金锋.	Wang Jinfeng.	[illegible]	of food poisoning	医学动物防制	edical animal con	26	6	556	2010	/j.issn.1003-6245.201	2008	2	6	2	Eastern	Hebei	Tangshan
289	[illegible]	ao Hong.Wang S	[illegible]	of food poisonir	医学信息（下旬刊）	l information (lat	23	2	280-280	2010	-	2007	3	5	N/A	Eastern	Shanghai	Hongkou
290	程徽.	Cheng Hui.	[illegible]	100 people caus	中国卫生检验杂志	ournal of Health	20	11	3036	2010	-	2009	1	4	2	Western	Gansu	Lanzhou
291	[illegible]	ao Jin.Yaofang.Lu	[illegible]	the source of Sal	预防医学情报杂志	eventive Medicin	26	11	855-857	2010	-	2008	2	7	N/A	Western	Sichuan	Guangyuan
292	[illegible]	Qin.Wang Yandon	[illegible]	dborne pathoge	中国卫生检验杂志	ournal of Health	20	10	2609-2610	2010	-	2009	1	7	1	Eastern	Shandong	Weifang
293	舒开奎.	Shu Kaikui.	[illegible]	od poisoning cau	中国药业	Pharmaceutical I	19	12	6-6	2010	/j.issn.1006-4931.201	2009	1	5	2	Western	Chongqing	Yuzhong
294	刘英民	Liu Yingmin	[illegible]	of food poisoning	临床合理用药杂志	Rational Clinical	3	15	28	2010	/j.issn.1674-3296.201	2009	1	6	1	Eastern	Hebei	Xingtai
295	郭皓,陈松.	uo Hao.Chen Sor	[illegible]	food poisoning	预防医学情报杂志	eventive Medicin	26	3	230-230	2010	-	2009	1	8	2	Western	Sichuan	Dazhou
296	[illegible]	Yunxia.Tian Li.Li T	[illegible]	students caused	中国医药指南	nese Medicine G	8	3	75-76	2010	/j.issn.1671-8194.201	2008	2	6	5	Northeastern	Heilongjiang	Jixi
297	王清,代彦,张勇.	Qing.Dai Yan.Zhan	[illegible]	tected in food po	现代预防医学	rn preventive me	37	20	3914-3916	2010	-	2009	1	7	2	Western	Sichuan	Dazhou
298	李岳良.	Li Yueliang.	[illegible]	and analysis of Sa	中国卫生检验杂志	ournal of Health	20	4	908,945	2010	-	2010	0	9	2	Eastern	Zhejiang	Ningbo
299	陈松,郭皓.	hen Song.Guo Ha	[illegible]	of a case of salm	预防医学情报杂志	eventive Medicin	26	3	222-223	2010	-	2008	2	6	2	Western	Sichuan	Dazhou
300	[illegible]	e Julian.Wei Junc	[illegible]	onella food poiso	中国卫生检验杂志	ournal of Health	20	10	2555-2557	2010	-	2010	0	3	3	Eastern	Zhejiang	Dongyang
301	[illegible]	ng Dai Chuanhua	[illegible]	food poisoning	职业与健康	cupation and he	26	5	517-518	2010	-	2008	2	3	2	Eastern	Jiangsu	Wuxi
302	刘惠红.	Liu Huihong.	[illegible]	food poisoning c	河南预防医学杂志	urnal of Preventiv	21	3	封3	2010	-	2006	4	3	1	Western	Sichuan	Liangshan
303	[illegible]	amin.Guo Dawei.	[illegible]	is of a Salmonella	海峡预防医学杂志	urnal of Preventiv	16	1	61	2010	-	2009	1	8	2	Eastern	Fujian	Longyan
304	[illegible]	jing.Zhang Xueba	[illegible]	almonella infecti	实用预防医学	cal preventive me	17	1	54-56	2010	/j.issn.1006-3110.201	2009	1	3	3	Eastern	Guangdong	Zhuhai
305	宋冬梅	Song Dongmei	[illegible]	of food poisonir	中外健康文摘	and Foreign Heal	-	-	-	2010	/j.issn.1672-5085.201	2009	1	8	1	Northeastern	Jilin	Changchun
306	[illegible]	a Jihui.Chen Wei	[illegible]	f food poisoning	中国当代医药	contemporary m	17	23	117,122	2010	/j.issn.1674-4721.201	2009	1	10	2	Western	Yunnan	Honghe
307	[illegible]	Zhonglan.Liao H	[illegible]	soning caused by	海峡预防医学杂志	urnal of Preventiv	16	4	51-52	2010	-	2009	1	5	2	Eastern	Fujian	Sanming
308	[illegible]	ezhi.Zhao Yan.Ch	[illegible]	athogenic Escher	中国初级卫生保健	ary health care in	24	5	66	2010	/j.issn.1001-568X.201	2006	4	8	2	Western	Yunnan	Dali
309	[illegible]	biao.Li Shufang.L	[illegible]	d poisoning caus	中国当代医药	contemporary m	17	27	129-130	2010	/j.issn.1674-4721.201	2009	1	5	2	Central	Hunan	Chenzhou
310	王恒昌.	Wang Hengchang	[illegible]	soning among st	中国自然医学杂志	ournal of Natural	12	4	316	2010	-	2009	1	6	2	Western	Yunnan	N/A
311	[illegible]	gai.Guo Chao.Che	[illegible]	ning incident cau	中国食品卫生杂志	e Food Hygiene	22	2	171-172	2010	-	2009	1	6	3	Western	Yunnan	Baoshan
312	[illegible]	haozhen Zhang Z	[illegible]	of food poisoni	流行病学传染病学	of Epidemiology	37	4	287	2010	ma.j.issn.1673-4149.2	2009	1	6	2	Western	Sichuan	Nanchong
313	[illegible]	ua.Pan Haiyan.Zha	[illegible]	tbreak caused by	中国卫生检验杂志	ournal of Health	20	4	912-913	2010	-	2009	1	2	2	Western	Yunnan	Yuxi
314	[illegible]	Zhilong.Cai Zher	[illegible]	of a case of bact	现代预防医学	rn preventive me	37	24	4661-4662	2010	-	2009	1	10	2	Eastern	Jiangsu	Taizhou
315	齐洪武,张利.	i Hongwu.Zhang	[illegible]	ning caused by Sa	中国热带医学	nese tropical med	10	8	1038-1038	2010	4/j.cnki.46-1064/r.201	2005	5	7	2	Northeastern	Liaoning	Fuxin
316	朱晶.	Zhu Jing.	[illegible]	d poisoning caus	现代医药卫生	rn medicine and	26	22	3510-3511	2010	-	2009	1	8	2	Eastern	Jiangsu	Taizhou
317	曹妍,李丽.	Cao Yan.Li Li.	[illegible]	e of food poison	预防医学情报杂志	eventive Medicin	26	5	413-414	2010	-	2007	3	7	2	Western	Sichuan	Nanchong
318	张明霞.	Zhang Mingxia.	[illegible]	tatus of a foodbo	中外健康文摘	and Foreign Heal	7	3	26-27	2010	/j.issn.1672-5085.201	2009	1	4	3	Eastern	Shandong	Yantai
319	王振海.	Wang Zhenhai.	[illegible]	sis of a case of sal	疾病监测与控制	surveillance and	4	1	27-27	2010	-	2007	3	8	3	Western	Inner mongolia	Chifeng
320	王小红,龚成林.	Xiaohong.Gong C	[illegible]	ning caused by Sa	中国卫生检验杂志	ournal of Health	20	6	1536	2010	-	2009	1	5	2	Western	Gansu	Lanzhou
321	许华,廖周勇.	Hua.Liao Zhouyo	[illegible]	case of food poi	现代预防医学	rn preventive me	37	20	3944-3944	2010	-	2009	1	8	4	Western	Sichuan	Nanchong
322	阳艳.	Yang Yan.	[illegible]	cteria in a case of	医学信息（医药版）	mation (Pharmac	-	2	-	2010	-	2008	2	6	1	Western	Sichuan	Zigong
323	吴红玲,马红梅	Hongling Ma Hon	[illegible]	ood poisoning ca	宁夏医学杂志	gxia Medical Jou	32	12	1256-1257	2010	/j.issn.1001-5949.201	2009	1	7	2	Western	Ningxia	Guyuan
324	[illegible]	Yang Guangyao.Z	[illegible]	ter pollution inci	实用预防医学	cal preventive me	17	6	1133-1134	2010	/j.issn.1006-3110.201	2009	1	5	17	Central	Hunan	Changsha
325	林毅.	Lin Yi.	[illegible]	e of food poison	中国卫生检验杂志	ournal of Health	20	11	30,373,039	2010	-	2009	1	9	2	Eastern	Shandong	Taian
326	刘红丽.	Liu Hongli.	[illegible]	poisoning case ca	中国卫生检验杂志	ournal of Health	20	3	665-666	2010	-	2005	5	7	2	Western	Shaanxi	Hanzhong
327	樊毅.	Fan Yi.	[illegible]	illness caused by	中国卫生检验杂志	ournal of Health	20	11	2978-2979	2010	-	2009	1	3	2	Western	Chongqing	Wanzhou
328	贺漓漓.	He Lili.	[illegible]	caused by eating	中国卫生检验杂志	ournal of Health	20	9	23,592,362	2010	-	2009	1	9	2	Western	Guangxi	Guilin
329	[illegible]	ng Xishan.Fu Yuli	[illegible]	of salmonella foo	医药杂志（下半月	ournal (Second H	38	8	381	2009	-	2008	1	5	3	Central	Shanxi	Lvliang
330	[illegible]	ng Ye Hengping	[illegible]	ase of food pois	公共卫生与预防医	lth and Preventiv	20	5	76-77	2009	-	2009	0	5	2	Central	Hubei	Xiantao
331	魏强,田野.	Wei Qiang.field.	水源污染致腹泻暴	ak caused by wat	预防医学论坛	entive Medicine F	15	3	287-288	2009	-	2007	2	7	4	Western	Inner mongolia	Tongliao
332	[illegible]	Wu Shaoqun.Wen	[illegible]	fruit shaved ice"A	海峡预防医学杂志	urnal of Preventiv	15	3	48-49	2009	-	2008	1	9	3	Eastern	Guangdong	Jieyang
333	[illegible]	u Junping.Yue Ga	[illegible]	etiology of a cas	公共卫生与预防医	lth and Preventiv	20	5	82-83	2009	-	2008	1	4	N/A	Central	Henan	Zhengzhou
334	[illegible]	uan Xing Suqin Y	[illegible]	poisoning caused	预防医学论坛	entive Medicine F	15	4	封3	2009	-	2008	1	3	3	Western	Inner mongolia	Chifeng
335	[illegible]	i.Shi Zhiyi.Li Junvi	[illegible]	d poisoning caus	国农村卫生事业管	rural health man	29	1	61-62	2009	-	2008	1	5	5	Western	Ningxia	Guyuan
336	[illegible]	jpu.Yu Mei.Mai Li	[illegible]	food poisoning	地方病通报	mic disease notifi	-	3	66	2009	-	2007	2	8	4	Western	Xinjiang	Ili
337	刘宏声.	Liu Hongsheng.	[illegible]	ase of food pois	海峡预防医学杂志	urnal of Preventiv	-	1	48-49	2009	-	2006	3	5	2	Eastern	Guangdong	Heyuan
338	[illegible]	Yunhong.Chen Yi	[illegible]	ella food poisoni	现代预防医学	rn preventive me	36	20	38,593,862	2009	-	2008	1	9	1	Central	Jiangxi	Nanchang
339	[illegible]	Jingmei.Gan Lipi	[illegible]	rne illness caused	职业与健康	cupation and he	25	4	382-383	2009	-	2008	1	6	2	Eastern	Guangdong	Shenzhen
340	[illegible]	Yan Hanqing.Ding	门菌PFGE分子分型	EMolecular typir	中国卫生检验杂志	ournal of Health	19	9	2039-2041	2009	-	2005	4	12	N/A	Eastern	Guangdong	Zhaoqing
341	[illegible]	Jinghui Shi Yong	[illegible]	isoning caused b	中国卫生检验杂志	ournal of Health	19	12	29,853,015	2009	-	2008	1	10	2	Eastern	Hebei	Tangshan
342	[illegible]	Mingyan.Zhang	[illegible]	ning caused by Sa	中国实用医药	ese Practical Me	4	9	247	2009	/j.issn.1673-7555.200	2007	2	6	4	Northeastern	Heilongjiang	Qiqihar
343	[illegible]	a Zhongwei.Yang	[illegible]	food poisoning	医学（医学护理分	icine (Medical Ge	30	4	185-186	2009	/j.issn.1001-8883.200	2009	0	8	N/A	Western	Shaanxi	Weinan
344	[illegible]	ai.Lan Quanxue.L	[illegible]	ility of pathogen	中国热带医学	nese tropical med	9	6	1144-1145,1005	2009	-	2008	1	6	2	Eastern	Guangdong	Shenzhen
345	[illegible]	Wang Hongxia	[illegible]	cular traceability	上海预防医学	hai Preventive M	21	12	593-596	2009	/j.issn.1004-9231.200	2007	2	10	10	Eastern	Shanghai	Huangpu
346	[illegible]	n Jinghai.Yu Xiao	[illegible]	isoning incident	首都公共卫生	apital public heal	3	5	219-221	2009	/j.issn.1673-7830.200	2009	0	4	2	Eastern	Beijing	Dongcheng
347	[illegible]	ong Ling Ye Lingc	[illegible]	esis in investigati	海峡预防医学杂志	urnal of Preventiv	15	3	54-55	2009	-	2006	3	4	2	Eastern	Fujian	Zhangzhou

348	林.郭宗成.牛金	n.Guo Dingcheng	沙门氏菌导致的食物	poisoning cause	药与保健（下旬版	nd Health Care (I	17	8	48-49	2009	-	2008	1	8	2	Western	Gansu	Tianshui
349	刘国营.左晨.	Guorong.Zuo Ch	为食物中毒突发公共	epidemic that tu	中国预防医学杂志	urnal of Preventiv	10	2	160-161	2009	-	2006	3	6	2	Eastern	Beijing	Changping
350	马世武.何海英.雨	iwu He Haiying Yu	起甲型副伤寒暴发	k of paratyphoid	中华临床感染病杂志	al of Clinical Infec	2	5	314-315	2009	ma.j.issn.1674-2397.2	2008	1	9	69	Western	Yunnan	Kunming
351	许华.	Xu Hua.	门氏菌引起食物中毒	case of food po	医学信息（医药版）	mation (Pharmac	-	11	-	2009	-	2009	0	5	1	Western	Sichuan	Nanchong
352	-	-	引起细菌性食物中毒	cterial food poiso	中国中医药资讯	al Chinese Medic	-	4	-	2009	-	2007	2	7	1	Western	Gansu	Linxia
353	李卫光.朱其凤.	Weiguang.Zhu Qif	医院阿伯丁沙门菌医	berdeen nosocor	中华医院感染学杂志	al of Hospital Infe	19	15	1954-1955	2009	/j.issn:1005-4529.200	2007	2	3	18	Eastern	Shandong	Jinan
354	尹薇蓉.王群	Xuerong Wang (	起由猪霍乱沙门氏菌	g caused by Salm	地方病通报	mic disease notifi	24	1	63-63	2009	-	2005	4	4	N/A	Western	Xinjiang	Ili
355	吴吟涓.唐飞.吴维邦	juan.Tang Fei.Wu	O9引起细菌性食物	on and analysis of	中国基层医药	na primary medi	16	7	1294-1295	2009	ma.j.issn.1008-6706.2	2007	2	5	2	Eastern	Guangdong	Shenzhen
356	子媛媛.朱海兰.	Yuanyuan.Zhu Hai	门菌引起的食物中毒	food poisoning c	预防医学论坛	entive Medicine F	15	5	478-479	2009	-	2008	1	7	3	Western	Yunnan	Dali
357	周玖英.	Zhou Jiuying.	门菌引起的食物中毒	ng and drug resi	医学信息	medical informatio	22	12	2718-2719	2009	/j.issn.1006-1959.200	2008	1	12	1	Central	Hubei	Enshi
358	.高翠文.朱怀甫.李	ven.Zhu Huaifu.Li	沙门氏菌引起食物中	poisoning cause	中外健康文摘	and Foreign Heal	6	33	18-19	2009	/j.issn.1672-5085.200	2005	4	8	2	Western	Guangxi	Laibin
359	庞惠勇.田锦萍.孙祝	iyong.Tian Jinpin	沙门菌引起食物中毒	food poisoning c	中国卫生检验杂志	ournal of Health I	19	12	2980-2981	2009	-	2009	0	6	2	Eastern	Jiangsu	Lianyungang
360	刘万郁.	Liu Wanyu.	沙门氏菌引起食物中	ood poisoning ca	现代预防医学	rn preventive me	36	24	4675-4675,4683	2009	-	2008	1	7	3	Western	Sichuan	Guangyuan
361	苗晓红	Miao Xiaohong	门氏菌引起的食物中	a case of food po	中国现代药物应用	drug application	3	14	193	2009	/j.issn.1673-9523.200	2006	3	6	4	Northeastern	Heilongjiang	Mudanjiang
362	黄继贵.章光提.	Jigui.Zhang Gua	鸭蛋引起沙门氏菌	od poisoning cau	现代预防医学	rn preventive me	35	9	17,271,730	2008	/j.issn.1003-8507.200	2007	1	6	3	Central	Hubei	Jingzhou
363	王兵.	Wang Bing.	伤寒沙门氏菌食物	phimurium food	泸州医学院学报	of Luzhou Medica	31	6	656-657	2008	/j.issn.1000-2669.200	2006	2	7	3	Western	Sichuan	Luzhou
364	滕鹏祺.李玉厘.	engpangi.Li Yuhe	门氏菌肠炎调查报	n reports on case	中国初级卫生保健	ary health care in	22	7	59	2008	/j.issn.1001-568X.200	2004	4	8	2	Northeastern	Heilongjiang	Heihe
365	何政.	He Zheng.	门菌污染引起的食	ng caused by Sal	职业与健康	cupation and hea	24	3	233-233	2008	/j.issn.1004-1257.200	2006	2	8	2	Eastern	Jiangsu	Wuxi
366	李怀玉.	Li Huaiyu.	沙门氏菌引起食物中	food poisoning c	中国医药指南	nese Medicine G	6	10	86-87	2008	/j.issn.1671-8194.200	2006	2	5	2	Western	Yunnan	Dali
367	刘丽华.刘加学.王晓	a.Liu Jiaxue.Wang	牛肠炎沙门氏菌	caused by Salmo	宁夏医学院学报	of Ningxia Medica	30	2	235-236	2008	/j.issn.1674-6309.200	2006	2	5	2	Western	Ningxia	Shizuishan
368	营.孔庆麟.王宇峰.	Kong Qinglin.Wa	染性腹泻暴发疫情	lysis of an infecti	应用预防医学	ed preventive me	14	2	102-103	2008	/j.issn.1673-758X.200	2007	1	9	8	Western	Guangxi	Guigang
369	石峻.	Shi Jun.	引起鼠伤寒沙门菌	sed by Salmonell	现代预防医学	rn preventive me	35	2	250,252	2008	/j.issn.1003-8507.200	2003	5	5	4	Western	Sichuan	Chengdu
370	李海燕.周宗俞.周飞	an.Zhou Zongyu.Z	寒沙门菌引起的	hing caused by sa	现代预防医学	rn preventive me	-	S1	22-23	2008	-	2008	0	7	2	Western	Chongqing	Yongchuan
371	余惠琴.王晓艳.	Huiqin.Wang Xiao	门氏菌集体食物中	food poisoning	大理学院学报	rnal of Dali Unive	7	2	49-50,53	2008	/j.issn.1672-2345.200	2006	2	8	N/A	Western	Yunnan	Dali

[illegible]

524	津,肖邦忠,邓学英,张	h Chung,Deng Xu	寨副伤寒暴发的调	f outbreaks of tv	预防医学情报杂志	eventive Medicin	19	1	12-14	2003	/j.issn.1006-4028.200	2001	2	9	N/A	Western	Chongqing	Zhongxian
525	谢德海,陈祈斌,彭辰	ai.Chen Qibin,Per	伤菌引起食物中毒	l poisoning incid	中国卫生监督杂志	ournal of Health S	10	1	24-25	2003	/j.issn.1077-6131.200	2002	1	4	2	Eastern	Guangdong	Shantou
526	赵耀林,史可梅	ao Yaolin Shi Ken	门氏菌属食物中毒	poisoning cause	长治医学院学报	f Changzhi Medic	17	1	20-21	2003	/j.issn.1006-0588.200	2002	1	10	3	Central	Shanxi	Changzhi
527	刘彤,李秀彬,杨东	a.Li Xiubin.Yang	沙门菌食物中毒的	se of Salmonella	中国卫生检验杂志	ournal of Health	13	6	779-779	2003	/j.issn.1004-8685.200	2001	2	6	2	Northeastern	Heilongjiang	Harbin
528	谢峰,纪红	Xie Feng,Ji Hong	菌引起婚宴食物中	od poisoning at	中国卫生监督杂志	ournal of Health S	10	2	70-71	2003	/j.issn.1077-6131.200	2002	1	5	2	Western	Ningxia	Yinchuan
529	赵鸣德,张旭	ao Mingdi,Zhang	氏沙门菌引起的食	oning caused by S	预防医学文献信息	edicine literatur	9	6	709-709	2003	/j.issn.1672-9153.200	2002	1	9	1	Northeastern	Liaoning	Dalian
530	李秀兰,孙凤琪	i Xiulan Sun Feng	纽沙门菌引起的食	oisoning case cau	中华流行病学杂志	Journal of Epide	24	12	1085-1085	2003	/j.issn:0254-6450.200	2003	0	5	3	Eastern	Guangdong	Guangzhou
531	津,邓化君,曾子同,余	a Huajun,Zeng Zit	肠感染性腹泻暴发	o an infectious d	实用预防医学	cal preventive me	10	4	584-584	2003	/j.issn.1006-3110.200	2002	1	6	15	Central	Hunan	Changde
532	魏月兴,张树锡	Yuexing,Zhang S	鼠伤寒沙门菌交叉	nial cross-infectic	中国乡村医药	inese rural medic	10	4	54	2003	/j.issn.1006-5180.200	2000	3	10	23	Eastern	Shandong	Binzhou
533	廖达升	Liao Dasheng	汝沙门氏菌食物中	e of Salmonella E	广西预防医学	axi Preventive Me	-	S1	41-42	2003	-	2001	2	9	1	Western	Guangxi	Chongzuo
534	王新村,李建芳	ng Xincun,Li Jianf	沙门菌食物中毒的	a case of salmone	职业与健康	cupation and hea	19	5	50-50	2003	/j.issn.1004-1257.200	2001	2	9	2	Eastern	Shandong	Binzhou
535	张长勇,夏淑青,董玉	ong,Xia Shuqing,I	污染真空软包装扒	la bulidan contan	预防医学文献信息	edicine literatur	9	2	189-189	2003	/j.issn.1672-9153.200	2002	1	5	2	Eastern	Shandong	Jinan
536	媛,周媛媛,马明	Zhou Yuanyuan,M	菌引起食物中毒暴	eak of food pois	第三军医大学学报	Third Military Me	25	22	2014-2014	2003	/j.issn:1000-5404.200	2003	0	2	1	Eastern	Beijing	N/A
537	黄纪明,支亚渊	ng Jiming Zhi Yav	业人员带菌引起的	caused by bact	中华卫生监督与健	alth Supervision	2	1	36	2003	-	2002	1	1	1	Eastern	Zhejiang	Shengzhou
538	张中昌,聂鸿雁	hongchang,Nie H	门氏菌所致食物中	oisoning caused b	宜春学院学报	nal of Yichun Univ	25	4	103-103	2003	/j.issn.1671-380X.200	2002	1	12	1	Eastern	Shandong	Rizhao
539	陈诗安,刘云会	hen Shi'an,Liu Yun	菌引起食物中毒34	uses food poiso	中华医学实践杂志	Journal of Medica	2	4	372-373	2003	-	2002	1	10	2	Central	Henan	Anyang
540	燕,严纪文,黄吉城	Jiwen,Huang Jich	门菌引起食物中毒	ase of food pois	华南预防医学	hina Preventive M	29	5	52-52	2003	/j.issn.1671-5039.200	2002	1	7	7	Eastern	Guangdong	Lianzhou
541	卢翠玲,王国志	uizhen Wang Gu	起的食物中毒调查	ng caused by sal	中华卫生监督与健	alth Supervision	2	3	45	2003	-	2002	1	9	N/A	Eastern	Hebei	Zunhua
542–699	[illegible]	[illegible]	[illegible]	[illegible]	[illegible]	[illegible]	[illegible]	[illegible]	[illegible]	[illegible]	[illegible]	[illegible]	[illegible]	[illegible]	[illegible]	[illegible]	[illegible]	[illegible]

[illegible]

700	张桂珍,李影林,	ng Guizhen,Li Yin	沙门氏菌引起的82	ella Dublin82Hum	中华医学检验杂志	l of Medical Labo	15	3	163-164	1992	-	1990	2	6	2	Northeastern	Jilin	Changchun	43.54	125.19	School	82	120	68.33	N/A	N/A	N/A	N/A	N/A
701	王淑敏,	Wang Shumin.	[illegible]	f food poisoning	中国食品卫生杂志	e Food Hygiene	-	3	36	1992	-	1990	2	7	N/A	Western	Gansu	Lanzhou	36.04	103.51	Military region	165	216	76.39	N/A	N/A	N/A	N/A	N/A
702	冉丽莎	Ran Lisa	[illegible]	on a case of salm	昌都科技	Qamdo Technolog	-	1	68-69	1992	-	1984	8	5	1	Western	Tibet	Changdu	30.92	97.73	Restaurant	8	48	16.67	N/A	N/A	N/A	N/A	6
703	朱健铭,吴晋兰,	u Jianming,Wu Jir	[illegible]	oning caused by	[illegible]	e Medicine and [illegible]	-	4	24-25	1992	-	1991	1	6	1	Eastern	Zhejiang	Hangzhou	30.16	120.1	Restaurant	38	40	95.00	N/A	N/A	N/A	N/A	N/A
704	[illegible]	ngyi,Sun Yi,Xu Xia	[illegible]	utbreak in hospit	中国医院感染学杂志	al of Hospital Infe	-	4	220-222	1992	-	1988	4	2	39	Central	Henan	Luoyang	34.41	112.27	Hospital	40	N/A	40.63	<1	14	2.3	2.9	22
705	[illegible]	ulan,Gao Jieqian,W	[illegible]	oisoning caused	河南预防医学杂志	urnal of Preventiv	-	2	67-70	1992	-	1983	9	5	3	Central	Henan	Nanyang	33	112.32	Residential area	258	260	99.23	N/A	N/A	N/A	N/A	N/A
706	[illegible]	onan,Shao Yaning	[illegible]	a nosocomial inf	中国卫生检验杂志	ournal of Health	-	1	62-63	1992	-	1988	4	4	N/A	Central	Henan	Kaifeng	34.47	114.21	Hospital	N/A	N/A	N/A	<1	N/A	N/A	N/A	N/A
707	[illegible]	yuan,Wang Shufa	[illegible]	f food poisoning	中国卫生检验杂志	ournal of Health	-	3	180-181	1992	-	1989	3	11	N/A	Western	Sichuan	Neijiang	29.36	105.02	mobile street stall	294	300	98.00	N/A	N/A	N/A	N/A	N/A
708	刘凤云,	Liu Fengyun.	[illegible]	nella Typhimuriur	河南预防医学杂志	urnal of Preventiv	-	6	375	1992	-	1988	4	9	3	Central	Henan	Hebi	35.54	114.11	Residential area	36	50	72.00	N/A	N/A	N/A	N/A	N/A
709	[illegible]	uang Xiaowei,Zha	[illegible]	ss-regional Salmo	食品科学	food science	-	9	47-50	1992	-	1991	1	1	70	Eastern	Jiangsu	Taizhou	32.3	119.54	mobile street stall	344	N/A	N/A	4	68	35	N/A	229
710	[illegible]	Huang Chuanzi,D	[illegible]	-wide infection w	上海医学	Shanghai Medicin	-	5	295	1992	-	1989	3	9	4	Eastern	Shanghai	Putuo	31.14	121.29	School	112	N/A	N/A	6	8	7	0.33	16
711	黄彪,	Huang Biao.	[illegible]	on a case of salm	广东卫生防疫	Health and Epider	-	4	83-84	1991	-	1990	1	10	2	Eastern	Guangdong	Zhuhai	22.17	113.34	Restaurant	61	150	40.67	22	64	43	N/A	35
712	[illegible]	Chongshan,Zhan	[illegible]	n Food Poisoning	中国食品卫生杂志	e Food Hygiene	-	3	37-38	1991	-	1990	1	5	3	Western	Inner mongolia	Hohhot	40.48	111.41	Farm	45	50	90.00	N/A	N/A	N/A	N/A	N/A
713	[illegible]	Pingyi,Wang Shin	[illegible]	n of a case of Salr	人民军医	ple's military med	-	10	10-11	1991	-	1989	2	12	4	Eastern	Jiangsu	Nanjing	32.03	118.46	School	242	499	48.50	N/A	N/A	N/A	N/A	N/A
714	刘亚男,闫琳,	Liu Yanan,Yan Lin	[illegible]	aused by Salmor	中国卫生检验杂志	ournal of Health	-	4	252-253	1991	-	1990	1	7	3	Northeastern	Heilongjiang	Hulunbuir	49.8	121.2	Residential area	275	300	91.67	N/A	N/A	N/A	N/A	N/A
715	郑丁华,	Zheng Dinghua.	[illegible]	eport on salmone	广东卫生防疫	Health and Epider	-	4	85-86	1991	-	1988	3	10	2	Eastern	Guangdong	Zhanjiang	21.11	110.24	mobile street stall	60	N/A	N/A	N/A	N/A	N/A	N/A	N/A
716	丁文静,王玉,朱玲,	enjing,Wang Yu,Z	[illegible]	outbreak in nurs	安徽医学	Anhui Medicine	-	6	32-33	1990	-	1988	2	8	1	Central	Anhui	Maanshan	31.43	118.28	Hospital	41	N/A	N/A	<1	N/A	<1	N/A	18
717	邓春萍,	Deng Qinglian.	[illegible]	a poisoning cause	云南畜牧兽医	usbandry and Ve	-	4	35	1990	-	1989	1	3	2	Western	Yunnan	Dali	25.34	100.13	Residential area	51	55	92.73	N/A	N/A	N/A	N/A	N/A
718	[illegible]	a Liuzi,Fan Qiniing	[illegible]	se of Salmonella	中国食品卫生杂志	e Food Hygiene	-	2	62-63	1990	-	1988	2	4	3	Central	Henan	Luoyang	34.41	112.27	Residential area	370	480	77.08	1	48	24	N/A	235
719	常天官,蔡佩钦,	ang Tianyi,Cai Pei	[illegible]	e of salmonella fo	新疆医学院学报	f Xinjiang Medica	13	4	260-261	1990	-	1989	1	11	3	Western	Xinjiang	Urumqi	43.45	87.36	Restaurant	106	126	84.13	2	54	32	N/A	36
720	余献,吴建中,	Xian,Wu Jianzho	[illegible]	Salmonella Thor	中国食品卫生杂志	e Food Hygiene	-	2	60-61	1990	-	1988	2	11	2	Eastern	Zhejiang	Hangzhou	30.16	120.1	Residential area	43	160	26.88	N/A	N/A	N/A	N/A	N/A
721	罗凤珍,王太梅,	Fengzhen,Wang T	[illegible]	lla typhimurium i	新生儿科杂志	urnal of Neonatol	5	2	75-76,86	1990	-	1988	2	7	240	Eastern	Beijing	Haidian	39.55	116.24	Hospital	40	N/A	N/A	<1	<1	<1	<1	10
722	[illegible]	in,Li Qinglin,Chen	[illegible]	teritis in obstetri	新生儿科杂志	urnal of Neonatol	5	5	201-202	1990	-	1989	1	7	57	Eastern	Shanghai	Baoshan	31.14	121.29	Hospital	24	141	17.02	<1	<1	<1	<1	2
723	[illegible]	uan,Zhao Ying,Ya	[illegible]	ning caused by Sa	包头医学院学报	of Baotou Medica	16	4	275-276	2000	j.issn.1006-740X.200	2000	0	8	1	Western	Inner mongolia	Baotou	40.39	109.49	Construction site	22	23	95.65	18	42	30	N/A	21
724	[illegible]	Fanwei,Deng Fuyi	[illegible]	of food poisoni	[illegible]	medicine literatur	6	4	374-375	2000	j.issn.1672-9153.200	1999	1	1	1	Eastern	Fujian	Longyan	25.06	117.01	Residential area	32	186	17.20	N/A	N/A	N/A	N/A	N/A
725	[illegible]	iaoping,Jiang Xue	[illegible]	Salmonella Salmo	浙江预防医学	ng Preventive Me	12	3	27	2000	j.issn.1007-0931.200	1996	4	5	1	Eastern	Zhejiang	Zhoushan	30.01	122.06	Military region	N/A	N/A	N/A	N/A	N/A	N/A	N/A	N/A
726	[illegible]	u Airong,Zhang A	[illegible]	oop from the fec	[illegible]	Journal of Epide	17	2	98	1996	ma.j.issn.0254-6450.1	1993	3	7	N/A	Western	Gansu	Wuwei	37.56	102.39	School	65	N/A	N/A	N/A	N/A	N/A	N/A	N/A
727	[illegible]	ng Aiping,Li Zuol	[illegible]	caused by salmo	中华医学检验杂志	l of Medical Labo	19	2	102	1996	-	1991	5	5	9	Northeastern	Jilin	Huadian	42.58	126.44	Residential area	8	N/A	N/A	N/A	N/A	N/A	N/A	N/A
728	[illegible]	Li,Fu Yukun,Zhan	[illegible]	ected in feces of	中华医学检验杂志	l of Medical Labo	17	4	239	1994	-	1993	1	5	N/A	Northeastern	Liaoning	Shenyang	41.48	123.25	School	N/A	N/A	N/A	N/A	N/A	N/A	N/A	N/A
729	蔡正选,	Cai Zhengxuan.	[illegible]	ood poisoning ca	[illegible]	urnal of Preventiv	27	2	128	1993	-	1990	3	10	1	Eastern	Hainan	Qionghai	19.14	110.28	Residential area	30	N/A	N/A	N/A	N/A	N/A	N/A	N/A
730	[illegible]	hchun,Zhang Li	[illegible]	land causes famil	[illegible]	l of Medical Labo	15	3	166	1992	-	1989	3	10	2	Northeastern	Liaoning	Benxi	41.18	123.46	Restaurant	10	N/A	N/A	28	74	51	N/A	3
731	[illegible]	Zhengliang,Yang	[illegible]	al outbreak of ne	[illegible]	Journal of Epide	12	5	301	1991	ma.j.issn.0254-6450.1	1989	2	9	24	Central	Hunan	Xiangtan	27.52	112.53	Hospital	16	16	100.00	<1	<1	<1	<1	N/A
732	[illegible]	Baoliang,Yan Qin	[illegible]	d poisoning caus	[illegible]	l of Medical Labo	14	4	223	1991	-	1990	1	10	1	Western	Xinjiang	Urumqi	43.45	87.36	Restaurant	2	N/A	N/A	14	N/A	N/A	N/A	2
733	[illegible]	en Jianjun,Wei Pi	[illegible]	of Salmonella A	[illegible]	urnal of Preventiv	24	2	69	1990	-	1988	2	6	2	Eastern	Hebei	Chengde	40.59	117.57	mobile street stall	254	254	100.00	N/A	N/A	N/A	N/A	N/A
734	[illegible]	u Jianhua,He Wei	[illegible]	nella typhimuriur	[illegible]	urnal of Preventiv	24	4	237	1990	-	1989	1	7	58	Central	Henan	Xinyang	32.07	114.04	Hospital	36	N/A	N/A	<1	3	1.5	N/A	20
735	[illegible]	uiyou,Yan Lifen,M	[illegible]	of choleraesuis	河南预防医学杂志	urnal of Preventiv	-	3	188	2000	j.issn.1006-8414.200	1999	1	1	2	Central	Henan	Zhengzhou	34.46	113.4	Residential area	35	85	41.18	3	65	34	N/A	22
736	[illegible]	iving,Zhong Ling	[illegible]	a Ramatogen was	福建医药杂志	lian Medical Jour	22	Z1	189	2000	j.issn.1002-2600.200	1998	2	12	3	Eastern	Fujian	Minqing	27.03	118.2	Residential area	121	216	56.02	N/A	N/A	N/A	N/A	N/A
737	[illegible]	sheng,Li Xianjun	[illegible]	ood poisoning ca	[illegible]	medicine literatur	6	2	139	2000	j.issn.1672-9153.200	1999	1	10	1	Central	Henan	Anyang	36.06	114.21	mobile street stall	96	103	93.20	2	62	32	N/A	53
738	吴佩琳,	Wu Peilin.	[illegible]	ning caused by Sa	河南医科大学学报	f Henan Medical	-	1	64	1993	-	1987	6	4	6	Central	Henan	Xinyang	32.07	114.04	Residential area	127	225	56.44	1	73	37	N/A	67
739	彭亚力,	Peng Yali.	[illegible]	nonella St. Paul fo	铁道医学	Railway Medicine	-	5	302	1994	-	1993	1	9	4	Western	Qinghai	Xining	36.38	101.48	mobile street stall	59	78	75.64	N/A	N/A	N/A	N/A	N/A
740	[illegible]	ngqi,Lu Baoxin,Sh	[illegible]	of food poisonin	[illegible]	ebei Workers' Me	-	4	32	1999	-	1999	0	4	2	Eastern	Hebei	Baoding	38.51	115.3	Restaurant	48	140	34.29	3	70	33	N/A	20
741	[illegible]	Shuning,Jin Guoq	[illegible]	c diarrhea caused	宁夏医科大学学报	Ningxia Medical	-	1	-	1996	-	1994	2	5	15	Western	Ningxia	Yinchuan	38.27	106.16	Hospital	13	39	33.33	<1	<1	<1	<1	6
742	李长玉,	Li Changyu.	[illegible]	Salmonella typh	宁夏医科大学学报	Ningxia Medical	-	4	-	1994	-	1991	3	11	111	Western	Ningxia	Yinchuan	38.27	106.16	Hospital	18	393	4.58	<1	12	3	N/A	12
743	[illegible] HEFELANHUI	Chunming,HEFE	[illegible]	Salmonella typhi	中国医院感染学杂志	al of Hospital Infe	-	3	138-140	1992	-	1989	3	10	210	Central	Anhui	Hefei	31.52	117.17	Hospital	23	N/A	N/A	<1	4	2	N/A	14
744	肖安保,	Xiao Anbao.	[illegible]	analysis of a case	疾病控制杂志	nal of disease co	4	3	278	2000	j.issn.1674-3679.200	1999	1	12	2	Central	Anhui	Maanshan	31.43	118.28	Residential area	28	36	77.78	19	56	38	N/A	8
745	刘巨多,	Liu Juduo.	[illegible]	oning among din	广东卫生防疫	Health and Epider	-	2	64	1999	-	1998	1	10	2	Eastern	Guangdong	Guangzhou	23.08	113.14	Restaurant	5	5	100.00	N/A	N/A	N/A	N/A	N/A
746	李春声,	Li Chunsheng.	[illegible]	of food poisonir	广东卫生防疫	Health and Epider	-	1	52-53	1999	-	1997	2	2	2	Eastern	Guangdong	Qingyuan	23.42	113.01	Residential area	166	200	83.00	4	70	37	N/A	66
747	[illegible]	Jin Ren Nugget	[illegible]	oisoning caused	中国卫生检验杂志	ournal of Health	-	6	49-51	1998	-	1995	3	10	N/A	Eastern	Zhejiang	Wenzhou	28.01	120.39	Residential area	96	N/A	N/A	N/A	N/A	N/A	N/A	N/A
748	于芳,	Yu Fang.	[illegible]	of poisoning ca	广东卫生防疫	Health and Epider	-	2	68-69	1998	-	1993	5	5	2	Eastern	Guangdong	Shenzhen	22.33	114.07	Office	25	N/A	N/A	N/A	N/A	N/A	N/A	N/A
749	唐燕飞,	Tang Yanfei.	[illegible]	oisoning caused	桂林医学杂志	ilin Medical Jour	14	3	181	1998	-	1996	2	8	N/A	Western	Guangxi	Guilin	25.17	110.17	mobile street stall	16	24	66.67	N/A	N/A	N/A	N/A	N/A
750	[illegible]	ng,He Guangli,Ch	[illegible]	aused by mixed	广东卫生防疫	Health and Epider	-	4	56-57	1997	-	1996	1	10	4	Eastern	Guangdong	Foshan	23.02	113.06	Restaurant	64	350	18.29	N/A	N/A	N/A	N/A	N/A
751	[illegible]	Chen Yingjian,Ji	[illegible]	the etiology of a	广东卫生防疫	Health and Epider	-	2	41-42	1996	-	1995	1	5	30	Eastern	Guangdong	Shenzhen	22.33	114.07	Factory	72	N/A	N/A	N/A	N/A	N/A	N/A	N/A
752	[illegible]	Chengshou,Wu H	[illegible]	tbreak of typhoid	[illegible]	rnal of Preventiv	-	3	69	1996	-	1994	2	4	120	Central	Anhui	Wuhu	31.19	118.22	Residential area	22	401	5.49	25	34	N/A	N/A	N/A
753	[illegible]	ansheng,Tao Xiul	[illegible]	of a typhoid feve	[illegible]	medicine literatur	-	2	113-114	1996	-	1994	2	7	21	Eastern	Shandong	Qingdao	36.03	120.18	Construction site	14	559	2.50	20	49	35	N/A	14
754	[illegible]	Xie Jie,Horse so	[illegible]	on report on foo	[illegible]	rnal of Preventiv	-	3	67-68	1996	-	1995	1	9	1	Central	Anhui	Fuyang	32.54	115.48	Restaurant	38	40	95.00	N/A	N/A	N/A	N/A	N/A
755	[illegible]	na Lei,Zhang Gua	[illegible]	nto a neonatal hc	疾病监测	isease surveillan	10	2	49	1995	-	1994	1	4	17	Western	Gansu	N/A	36.04	103.51	Hospital	33	44	75.00	<1	<1	<1	<1	15
756	[illegible]	Zhenru,Chen We	[illegible]	analysis of a nos	蚌埠医药	Bengbu Medicin	-	2	35	1995	-	1986	9	9	26	Central	Anhui	Bengbu	32.56	117.21	Hospital	27	34	79.41	<1	<1	<1	<1	12
757	杨会芹,	Yang Huiqin.	[illegible]	f food poisoning	职业与健康	cupation and hea	-	3	38-39	1995	-	1994	1	9	2	Eastern	Tianjin	Baodi	39.02	117.12	Restaurant	13	15	86.67	N/A	N/A	N/A	N/A	N/A
758	张国荣,	Zhang Guorong	[illegible]	erial food poison	[illegible]	eventive Medicin	-	S1	117	1993	-	1992	1	8	1	Western	Sichuan	Luzhou	28.54	105.24	Residential area	45	47	95.74	N/A	N/A	N/A	N/A	N/A

876	蓝岳云,张讲	an Yuevun,Zhang	中甲型副伤寒暴发调	phoid A Outbrea	疾病监测	isease surveillanc	-	9	1	2005	-	2003	2	4	70	Eastern	Zhejiang	Quzhou	[illegible]
877	木上丽,李少华,祝庆	ali,Li Shaohua,Zhu	瑞中学副伤寒暴发	phoid outbreak in	中国自然医学杂志	ournal of Natural	-	3	2	2005	-	2004	1	9	26	Central	Jiangxi	Shangrao	[illegible]
878	坤,李顺祥,刘红雁	Shunxiang Liu H	起甲型副伤寒暴发	tbreak of paratyp	预防医学情报杂志	eventive Medicin	-	4	478	2005	-	2005	0	4	23	Western	Yunnan	Yuxi	[illegible]
879	钟志成,谢起理,罗卫	heng,Xie Qili,Luo	中学水型伤寒暴发	f waterborne typ	预防医学论坛	entive Medicine F	-	3	359-360	2005	-	2003	2	6	36	Central	Jiangxi	Ganzhou	[illegible]
880	胡富宇,彭道法	Hu Fuyu,Peng Dao	一起甲型副伤寒暴发	aratyphoid fever A	中国热带医学	nese tropical med	-	5	1005-1006	2005	-	2003	2	9	169	Eastern	Zhejiang	Taizhou	[illegible]
881	章跃炎	Zhang Yueyan	暴发流行病例血清	ds for epidemic	中国卫生检验杂志	ournal of Health	-	5	614-615	2005	-	2004	1	10	58	Eastern	Zhejiang	Taizhou	[illegible]
882	军,陈平,于顺明,任	n Ping,Yu Shunmi	伤寒暴发的流行病学	tigation of an out	预防医学情报杂志	eventive Medicin	-	2	188-189	2005	-	2003	2	11	45	Western	Sichuan	Dazhou	[illegible]
883	陶丹	Tao Dan	起水型伤寒暴发调	outbreak of wate	预防医学情报杂志	eventive Medicin	-	2	191-192	2005	-	2004	1	8	56	Eastern	Zhejiang	Shaoxing	[illegible]
884	福,王树坤,曾丽萍	ng Shukun,Zeng	型副伤寒暴发流行	analysis of an out	中国热带医学	nese tropical med	-	1	77-133	2005	-	2003	2	6	51	Western	Yunnan	Yuxi	[illegible]
885	黎明强,殷凯,刘金	g Qiang,Yin Kai,L	副伤寒暴发流行的	on an outbreak o	广西预防医学	xi Preventive Me	-	5	317	2004	-	2004	0	5	80	Western	Guangxi	Liuzhou	[illegible]
886	福,王树坤,钦绍德	ng Shukun,chinsa	起甲型副伤寒暴发的	tion and Analysis	预防医学情报杂志	eventive Medicin	-	5	557-558	2004	-	2003	1	7	11	Western	Yunnan	Yuxi	[illegible]
887	开,黄运能,黄少新	Yunneng,Huang	发疫情流行因素的	epidemic factor	中国自然医学杂志	ournal of Natural	-	3	129-130	2004	-	2002	2	9	31	Western	Guangxi	Guilin	[illegible]
888	田京城,潘忠伦,欧建	,Pan Zhonglun,Ou	染引起的甲型副伤	utbreak caused b	预防医学情报杂志	eventive Medicin	-	4	455-456	2004	-	2001	3	6	61	Western	Guizhou	Qiandongnan	[illegible]
889	回甫,李华民,谢尚	Li Huamin,Xie Sh	型伤寒暴发的流行	n report on a livi	湖北预防医学杂志	rnal of Preventiv	-	4	34-35	2004	-	2003	1	8	65	Central	Hubei	Chibi	[illegible]
890	吉力根甫,阿地力	u Jili Tipu,Adili,A	勒县一起学校伤寒	fever outbreak i	疾病监测	isease surveillanc	-	6	235	2004	-	2003	1	6	16	Western	Xinjiang	Kashgar	[illegible]
891	利,李瑞英,张成恩	Ruiying,Zhang C	甲型副伤寒暴发的	the outbreak of	预防医学文献信息	edicine literatur	-	3	278-279	2004	-	2003	1	2	68	Eastern	Shandong	Weihai	[illegible]
892	杨春梅,王福元,张	mei,Wang Fuyuan	暴发的流行病学	gation and analys	解放军预防医学杂志	edicine of the P	-	1	69	2004	-	2002	2	6	41	Western	Gansu	N/A	[illegible]
893	袁萍,李诗佩	Yuan Ping,Li Shipe	暴发地区住院伤寒	cases in typhoid	预防医学文献信息	edicine literatur	-	1	115-116	2004	-	1999	5	10	61	Eastern	Zhejiang	Shaoxing	[illegible]
894–1051	[illegible]	[illegible]	[illegible]	[illegible]	[illegible]	[illegible]	[illegible]	[illegible]	[illegible]	[illegible]	[illegible]	[illegible]	[illegible]	[illegible]	[illegible]	[illegible]	[illegible]	[illegible]	[illegible]	[illegible]

1052	王莉敏;	Wang Limin;	湾西村伤寒暴发的	ver outbreak in L	皖南医学院学报	f Wannan Medic	-	4	75-76	1990	-	1988	2	7	106	Central	Anhui	Wuhu	31.19	118.22	Residential area	9	198	4.55	13	40	N/A	N/A	5	4	55.56	N/A	N/A	N/A	N/A	N/A	N/A	N/A	D1	Typhi	N/A	CHL	Y	N/A	Y	N/A	N/A	N/A	Phage infection test	AMK	9	0	id/paratyphoid fever
1053	姜美君;李红兵;	a Meijun;Li Hong	一起食物型伤寒暴	d-borne typhoid	西藏医药杂志	etan Medical Jou	-	1	32-34	1990	-	1988	2	11	30	Western	Tibet	Lhasa	29.39	91.08	Office	127	222	57.21	4	63	N/A	N/A	N/A	N/A	N/A	17.28	96.30	50.62	N/A	N/A	N/A	Meat products	D1	Typhi	N/A	N/A	Y	N/A	Y	N/A	N/A	N/A	N/A	CHL, FRZ, SXT	127	0	er & Food poisoning
1054	李照坤	Li Zhaokun	型肠伤寒暴发的流行	on of a waterbor	解放军预防医学杂志	Medicine of the P	-	1	59-61	1990	-	1986	4	4	62	Western	Guizhou	Guiyang	26.35	106.42	Hospital	133	815	16.32	4	78	23.4	N/A	83	50	62.41	30.83	100.00	13.31	N/A	N/A	81.20	N/A	D1	Typhi	N/A	N/A	Y	N/A	Y	N/A	N/A	N/A	Phage infection test	N/A	131	2	-associated infection
1055	蒋兴发;	Jiang Xingfa;	起中学伤寒暴发流行	d fever outbreak	学校卫生	school health	-	5	28-29	1989	-	1988	1	5	61	Central	Hunan	Huaihua	27.33	109.58	School	39	843	4.63	N/A	N/A	N/A	N/A	N/A	N/A	N/A	100.00	100.00	N/A	N/A	N/A	100.00	N/A	D1	Typhi	N/A	N/A	Y	N/A	Y	N/A	N/A	N/A	N/A	N/A	39	0	er & Food poisoning
1056	马风生;马龙宝;赵重	Malong Bao;Zhac	游一起水型伤寒暴	hoid fever occur	学校卫生	school health	-	5	29	1989	-	1988	1	9	94	Eastern	Jiangsu	Zhenjiang	32.11	119.27	School	122	798	15.29	N/A	N/A	N/A	N/A	54	32	44.26	N/A	N/A	N/A	N/A	N/A	N/A	N/A	D1	Typhi	N/A	N/A	Y	N/A	Y	N/A	N/A	N/A	N/A	N/A	N/A	N/A	id/paratyphoid fever
1057	王复国;	Wang Fuguo;	伤寒暴发流行的调	ort on an outbrea	铁道医学	Railway Medicine	-	2	98	1989	-	1987	2	7	69	Northeastern	Heilongjiang	Jixi	45.17	130.57	Restaurant	62	18029	0.34	N/A	N/A	N/A	N/A	33	29	53.23	N/A	N/A	N/A	N/A	N/A	N/A	N/A	D1	Typhi	N/A	N/A	Y	N/A	Y	N/A	N/A	N/A	N/A	N/A	62	0	id/paratyphoid fever
1058	程;王颖新;白志清;张	a Yingxi;Bai Zhiji	次水型伤寒暴发的	outbreak of wate	内蒙古医学杂志	Mongolia Medical	-	1	36-37	1988	-	1978	10	6	90	Western	Inner mongolia	Wuhai	39.4	106.48	Residential area	60	170	35.29	N/A	N/A	N/A	N/A	N/A	N/A	N/A	N/A	100.00	N/A	N/A	N/A	N/A	N/A	D1	Typhi	N/A	N/A	Y	N/A	Y	N/A	N/A	N/A	N/A	N/A	60	0	id/paratyphoid fever
1059	志明;曾财娣;刘树	ing;Zeng Caidi;Li	起伤寒暴发的流行	on of a typhoid f	广州医药	uangzhou Medici	-	6	2-4+6	1987	-	1986	1	1	18	Eastern	Guangdong	Guangzhou	23.08	113.14	School	23	814	2.83	18	26	N/A	N/A	20	3	86.96	14.30	100.00	19.10	23.81	23.81	90.50	N/A	D1	Typhi	N/A	N/A	Y	N/A	Y	N/A	N/A	N/A	N/A	N/A	23	0	id/paratyphoid fever
1060	顾清礼;	Gu Qingli;	寒暴发流行临床特	haracteristics of a	宁夏医学院学报	f Ningxia Medica	-	2	28+30	1987	-	1978	9	10	45	Western	Ningxia	Wuzhong	37.59	106.11	Construction site	51	N/A	N/A	16	40	N/A	N/A	51	0	100.00	45.10	100.00	39.22	9.80	9.80	62.75	N/A	D1	Typhi	N/A	N/A	Y	N/A	Y	N/A	N/A	N/A	N/A	N/A	N/A	N/A	id/paratyphoid fever
1061	劫;杨金龙;米芳苏;	ng Jinlong;Mi Fan	型伤寒暴发流行的	n an outbreak of	河南预防医学杂志	rnal of Preventiv	-	4	34-36	1987	-	1986	1	12	112	Central	Henan	Xinyang	32.07	114.04	Residential area	76	26456	0.29	1	63	N/A	N/A	39	37	51.32	N/A	100.00	N/A	N/A	N/A	N/A	N/A	D1	Typhi	N/A	N/A	Y	N/A	Y	N/A	N/A	N/A	N/A	CHL, AMP	76	0	id/paratyphoid fever
1062	民;付卓杰;吴凤义;	n;Fu Zhuojie;Wu	起水型肠伤寒暴发	of water-type e	黑龙江医药科学	jiang Medical S	-	2	116-118	1987	-	1985	2	8	36	Northeastern	Heilongjiang	Jiamusi	46.47	130.22	Residential area	40	3589	1.11	5	45	N/A	N/A	20	20	50.00	N/A	N/A	N/A	N/A	N/A	N/A	N/A	D1	Typhi	N/A	N/A	Y	N/A	Y	N/A	N/A	N/A	N/A	N/A	N/A	N/A	id/paratyphoid fever
1063	志;王彦厘;郑钧;郑	a Yanhou;Zheng	一次鼠伤寒暴发流	d fever outbreak	临沂医专学报	of Linyi Medical	-	Z1	61-63	1986	-	1985	1	2	61	Eastern	Shandong	Linyi	35.03	118.2	Hospital	64	N/A	N/A	<1	3	N/A	N/A	36	28	56.25	51.56	78.13	N/A	N/A	N/A	N/A	N/A	B	Typhimurium	N/A	N/A	Y	N/A	Y	N/A	N/A	N/A	N/A	EN, KAN, ERY, C	56	8	-associated infection
1064	俊俏;林永健;罗平全	Yongjian;Luo Pin	伤寒暴发的流行病	eport on an outbr	右江医学	Youjiang Medicin	-	1	1-4	1985	-	1984	1	7	87	Western	Guangxi	Baise	23.54	106.36	Residential area	98	183	53.55	1	73	N/A	N/A	44	54	44.90	N/A	100.00	100.00	N/A	N/A	100.00	N/A	D1	Typhi	N/A	N/A	N/A	N/A	Y	N/A	N/A	N/A	N/A	CHL	96	2	id/paratyphoid fever
1065	贾荣盛 董春红	gsheng Dong Ch	发流行624例临床分	reak624Case clinic	山东医药	handong Medici	-	7	3	1984	-	1983	1	8	61	Eastern	Shandong	Binzhou	37.22	118.02	Residential area	624	N/A	N/A	N/A	N/A	N/A	N/A	N/A	N/A	N/A	N/A	100.00	N/A	N/A	N/A	100.00	N/A	D1	Typhi	N/A	N/A	Y	N/A	Y	N/A	N/A	N/A	N/A	N/A	623	1	id/paratyphoid fever
1066	杨龙献;赵九根;曲春	xian;Zhao Jiuxian	村伤寒暴发流行	k of typhoid feve	河南预防医学杂志	rnal of Preventiv	-	3	21-24	1984	-	1983	1	8	109	Central	Henan	Luoyang	34.41	112.27	Residential area	72	388	18.56	1	65	N/A	N/A	43	29	59.72	59.72	100.00	47.22	30.56	26.39	72.22	N/A	D1	Typhi	N/A	CHL	Y	N/A	Y	N/A	N/A	N/A	N/A	CHL, GEN, SXT	69	3	id/paratyphoid fever
1067	世昌;鲍明荣;周天	g;Bao Mingrong;	季水型伤寒暴发流	a winter waterbo	人民军医	ple's military med	-	2	29-30	1984	-	1981	3	12	45	Central	Anhui	Luan	31.44	116.28	Military region	14	459	3.05	10	31	N/A	N/A	N/A	N/A	N/A	100.00	100.00	N/A	N/A	N/A	N/A	N/A	D1	Typhi	N/A	N/A	Y	N/A	Y	N/A	N/A	N/A	N/A	CHL, SXT	14	0	id/paratyphoid fever
1068	苏静君;蒋国伟;陈	n;Jiang Guowei;C	室鼠伤寒暴发流行	typhoid fever ou	贵州医药	Guizhou Medicin	-	1	2	1983	-	1982	1	10	21	Western	Guizhou	Guiyang	26.35	106.42	Hospital	65	137	47.45	<1	<1	<1	<1	36	29	55.38	100.00	100.00	N/A	N/A	N/A	N/A	N/A	B	Typhimurium	N/A	N/A	Y	N/A	Y	N/A	N/A	N/A	N/A	N/A	53	12	-associated infection
1069	叶云凌;李明;	Ye Yunling;Li Min	寒暴发流行的菌型	rial types prevale	浙江医学	Zhejiang Medicin	-	5	41-43	1981	-	1977	4	2	48	Eastern	Zhejiang	Hangzhou	30.16	120.1	Office	111	N/A	N/A	N/A	N/A	N/A	N/A	N/A	N/A	N/A	N/A	N/A	N/A	N/A	N/A	N/A	N/A	B	Typhimurium	N/A	N/A	Y	Y	Y	N/A	N/A	N/A	N/A	N/A	N/A	N/A	N/A
1070	泉;王建利;黄再添;	n;Wang Jianli;Hua	沃角伤寒暴发流行	k of typhoid feve	福建医药杂志	jian Medical Jour	-	2	30-32	1981	-	1977	4	7	64	Eastern	Fujian	Zhangzhou	24.31	117.39	Residential area	152	1974	7.70	N/A	N/A	N/A	N/A	69	83	45.39	N/A	N/A	N/A	N/A	N/A	N/A	N/A	D1	Typhi	N/A	N/A	Y	Y	Y	N/A	N/A	N/A	N/A	N/A	N/A	N/A	id/paratyphoid fever
1071	沙介荣;顾益明;	a Jierong;Gu Yim	暴发43例临床及流	eak43Clinical and	浙江医学	Zhejiang Medicin	-	4	25-26+14	1980	-	1978	2	4	57	Eastern	Zhejiang	Jinhua	29.07	119.39	Residential area	43	N/A	N/A	3	66	N/A	N/A	26	17	60.47	65.12	93.02	65.12	34.88	34.88	74.42	N/A	D1	Typhi	N/A	N/A	Y	N/A	Y	N/A	N/A	N/A	N/A	HL, AMP, FRZ, SX	N/A	N/A	id/paratyphoid fever
1072	宋清林;	Song Qinglin;	型伤寒暴发流行的	an outbreak of f	人民军医	ple's military med	-	7	34-36	1980	-	1978	2	5	18	Eastern	Beijing	N/A	39.55	116.24	Factory	22	174	12.64	N/A	N/A	N/A	N/A	N/A	N/A	N/A	18.18	100.00	45.45	N/A	N/A	100.00	Vegetable products	D1	Typhi	N/A	N/A	Y	Y	Y	N/A	N/A	Y	N/A	N/A	N/A	N/A	er & Food poisoning
1073	顾清礼;	Gu Qingli;	染引起的一次家庭	amily caused by	宁夏医学院学报	f Ningxia Medica	-	2	6-7	1980	-	1978	2	12	5	Western	Ningxia	Wuzhong	37.59	106.11	Residential area	8	8	100.00	<1	31	11.55	11.37	3	5	37.50	N/A	100.00	100.00	N/A	N/A	100.00	N/A	D1	Typhi	N/A	N/A	Y	N/A	Y	N/A	N/A	N/A	N/A	CHL, TMP	8	0	-associated infection
1074	郭安荣;李志成;	Anrong;Li Zhich	农场伤寒暴发流行	reak of typhoid f	人民军医	ple's military med	-	7	14-16	1979	-	1978	1	7	30	Western	Sichuan	Luzhou	28.54	105.24	Farm	51	109	46.79	N/A	N/A	N/A	N/A	N/A	N/A	N/A	35.29	100.00	78.43	78.43	29.41	92.15	N/A	D1	Typhi	N/A	N/A	Y	N/A	Y	N/A	N/A	N/A	N/A	CHL	N/A	N/A	id/paratyphoid fever
1075	刘立峰;	Liu Lifeng;	04例伤寒暴发的调	tigation report o	河南预防医学杂志	rnal of Preventiv	-	3	61-64	1979	-	1978	1	6	41	Central	Henan	Anyang	36.06	114.21	Residential area	104	3400	3.06	2	69	N/A	N/A	42	62	40.38	12.50	100.00	41.67	N/A	N/A	100.00	N/A	D1	Typhi	N/A	N/A	Y	N/A	Y	N/A	N/A	N/A	N/A	CHL	103	1	id/paratyphoid fever
1076	-	-	××厂伤寒暴发的调	report on typho	重庆医药	hongqing Medici	-	5	8-10+20	1977	-	1976	1	10	60	Western	Chongqing	Jiangbei	29.36	105.02	Factory	53	649	8.17	3	54	N/A	N/A	29	24	54.72	32.07	100.00	16.98	N/A	N/A	43.39	N/A	D1	Typhi	N/A	N/A	Y	N/A	Y	N/A	N/A	N/A	N/A	CHL, SOX	53	0	id/paratyphoid fever
1077	乔富泽	Qiao Fuqu	伤寒暴发流行49例	ne typhoid fever	陕西新医药	aanxi New Medic	-	3	17-18+20	1975	-	1973	2	6	14	Western	Shaanxi	Xianyang	34.2	108.43	Factory	49	514	11.89	N/A	N/A	N/A	N/A	N/A	N/A	N/A	100.00	100.00	100.00	N/A	N/A	100.00	N/A	D1	Typhi	N/A	N/A	Y	N/A	Y	N/A	N/A	N/A	N/A	SYN, CHL	49	0	er & Food poisoning
1078	-	-	乙型副伤寒暴发流	eak of paratyphoi	山东医刊	ndong Medical Jo	-	5	16+15	1961	-	1960	1	5	17	Eastern	Shandong	Qingzhou	36.42	118.28	Residential area	149	373	39.95	3	77	N/A	N/A	N/A	N/A	N/A	100.00	100.00	100.00	100.00	N/A	100.00	Meat products	B	Paratyphi B	N/A	N/A	Y	N/A	Y	N/A	N/A	N/A	N/A	N/A	149	0	er & Food poisoning
1079	孔庆长.	Kong Qingchang	门氏菌急性感染122	ute infection122E	中华卫生杂志	inese Health Jour	6	3	164-167	1958	-	1956	2	8	3	Northeastern	Jilin	Dehui	44.32	125.42	mobile street stall	122	N/A	N/A	3	78	N/A	N/A	86	36	70.49	91.23	89.47	96.49	76.32	66.67	91.23	Meat products	B	Typhimurium	N/A	N/A	Y	Y	Y	N/A	N/A	N/A	N/A	N/A	115	7	Food poisoning
1080	丽;李彩云;高雅;修	Li Caiyun;elegant	沟食物中毒事件的	ood poisoning in	医学动物防制	edical animal con	39	2	198-201	2023	-	2021	2	9	2	Eastern	Hebei	Chengde	40.59	117.57	Hospital	22	N/A	N/A	27	88	N/A	N/A	4	18	18.18	100.00	86.36	63.64	N/A	40.91	N/A	Others	D1	Enteritidis	N/A	AM, NAL, POL-E,	Y	Y	Y	Y	N/A	N/A	N/A	N/A	22	0	-associated infection
1081	何玲玲;王乐;汪洋;	;Wang Le;Wang	菌食源性疾病事件	reak of Salmonel	实用预防医学	cal preventive me	30	1	84-87	2023	-	2021	2	6	2	Western	Sichuan	Mianyang	31.3	104.42	School	198	395	50.13	16	18	N/A	N/A	79	119	39.90	94.40	87.88	66.16	56.06	33.33	N/A	Pastries	D1	Enteritidis	N/A	N/A	Y	Y	Y	Y	N/A	N/A	N/A	N/A	198	0	Food poisoning
1082	邵莉;周军波;赵洋;	Zhou Junbo;Zhao	门菌引起的辖区食	e disease outbre	疾病监测	lisease surveillanc	37	11	1495-1501	2022	-	2021	1	5	2	Central	Hubei	Wuhan	30.35	114.17	Residential area	8	N/A	N/A	21	51	N/A	N/A	4	4	50.00	100.00	75.00	100.00	N/A	50.00	N/A	Meat products	D1	Enteritidis	11	CZO, FOX, CXM, G	Y	Y	Y	Y	N/A	N/A	WGS	N/A	8	0	Food poisoning
1083	包小兵;李旭波;宋	aobing;Luan Xuh	菌食物中毒分离株	istics of Salmone	江苏预防医学	su Preventive Me	33	6	731-732	2022	8/j.issn.1006-9070.20	2021	1	5	3	Central	Henan	Anyang	36.06	114.21	Residential area	24	N/A	N/A	N/A	N/A	N/A	N/A	N/A	N/A	N/A	91.67	95.83	83.33	79.17	45.83	37.50	Nut products	B	Abony	N/A	GEN, CZO	Y	Y	Y	Y	Y	N/A	MALDI-TOF-MS	CRO, CIP	23	1	Food poisoning
1084	李毅;谢爱霞;谢中	Xie Airong;Xie Zh	副伤寒沙门菌暴发	a Paratyphi A out	中国人兽共患病学	se Journal of Zoo	38	10	898-905	2022	-	2020	2	12	21	Eastern	Zhejiang	Wenzhou	28.01	120.39	School	12	N/A	N/A	19	23	N/A	N/A	3	9	25.00	66.67	100.00	N/A	N/A	N/A	83.33	N/A	A	Paratyphi A	129	NAL, SM	Y	Y	Y	Y	Y	N/A	ALDI-TOF-MS、W	N/A	N/A	N/A	er & Food poisoning
1085	晓飞;喻瑾;罗玉;肖	ei;Yu Jin;Luo Yu;	门氏菌引起的食源	outbreak caused	中国食品卫生杂志	e Food Hygiene	-	-	9	2021	-	2021	0	5	3	Western	Chongqing	Jiulongpo	29.35	106.33	Office	55	73	75.34	6	79	N/A	N/A	37	18	67.27	89.09	43.64	78.18	N/A	29.09	30.90	Pastries	D1	Enteritidis	N/A	N/A	Y	Y	Y	N/A	N/A	N/A	N/A	N/A	N/A	N/A	Food poisoning
1086	玉;吕均;杨康;李艳	Jun;Yang Kang;L	菌食物中毒的病原	analysis of a case	医学动物防制	edical animal con	38	11	1039-1042	2022	-	2021	1	5	N/A	Central	Hubei	Shiyan	32.4	110.47	School	17	3930	0.43	N/A	N/A	N/A	N/A	N/A	N/A	N/A	100.00	70.59	N/A	N/A	47.06	N/A	Meat products	D1	Enteritidis	11	AMP, SAM, NAL	Y	Y	Y	Y	Y	N/A	N/A	N/A	N/A	N/A	Food poisoning
1087	飞;彭子欣;范自军;	Fei;Peng Zixin;Fa	校园食源性疾病	ous foodborne di	中国食品卫生杂志	e Food Hygiene	34	5	1089-1094	2022	13590/j.cjfh.2022.05.	2018	4	4	3	Central	Hubei	Enshi	30.16	109.29	School	71	2206	3.22	N/A	N/A	N/A	N/A	N/A	N/A	N/A	100.00	73.24	71.83	23.94	38.03	36.62	Pastries	D1	Enteritidis	N/A	N/A	Y	Y	Y	Y	N/A	N/A	N/A	N/A	N/A	N/A	Food poisoning
1088	骆水娟;夏雅红;杨	Luo Shuijuan;Xia	起食源性暴发事件	of a foodborne c	中国卫生检验杂志	ournal of Health	32	18	2218-2221	2022	-	2021	1	3	N/A	Eastern	Zhejiang	Hangzhou	30.16	120.1	Office	N/A	N/A	N/A	N/A	N/A	N/A	N/A	N/A	N/A	N/A	N/A	N/A	N/A	N/A	N/A	N/A	Egg products	B	Typhimurium	N/A	, CXM, FOX, AMK,	Y	Y	Y	Y	N/A	N/A	MALDI-TOF-MS	N/A	N/A	N/A	Food poisoning
1089	马良良;吕婷;李欣	aliang;Lu Ting;Li	菌引起大学生食物	oning among coll	中国校医	inese school doc	36	9	681-683+690	2022	-	2021	1	10	2	Eastern	Jiangsu	Xuzhou	34.15	117.11	School	15	N/A	N/A	18	24	19.6	1.58	4	11	26.67	100.00	93.33	33.33	N/A	33.33	N/A	Vegetable products	C1	Lomita	N/A	NAL, CIP	Y	Y	Y	Y	Y	N/A	N/A	N/A	15	0	Food poisoning
1090	珍;炊慧霞;戚浩;…	i Huixia;Qi Haoyu	性疾病暴发事件的	ity analysis of foo	河南预防医学杂志	rnal of Preventiv	33	9	650-653	2022	nki.hnjpm.1006-8414	2020	2	5	4	Central	Henan	Pingdingshan	33.44	113.17	Restaurant	89	475	18.74	N/A	N/A	N/A	N/A	N/A	N/A	N/A	100.00	100.00	100.00	41.57	31.46	N/A	Meat products	B	Paratyphi B	N/A	N/A	Y	Y	Y	Y	Y	N/A	MALDI-TOF-MS	N/A	89	0	er & Food poisoning
1091	香;马健;赵媛坤;张	Jian;Zhao Yuanku	的食源性疾病暴发	an outbreak of fo	医学动物防制	edical animal con	38	9	902-905	2022	-	2020	2	5	2	Western	Gansu	Longnan	33.6	105.22	Residential area	25	N/A	N/A	6	69	N/A	N/A	10	15	40.00	100.00	100.00	N/A	N/A	100.00	N/A	Others	D1	Enteritidis	N/A	N/A	Y	Y	Y	Y	N/A	N/A	N/A	N/A	N/A	N/A	Food poisoning
1092	冬花;汪生虎;沈作	;Wang Shenghu;	氏菌感染引起的食	uspected to be ca	疾病预防控制通报	evention and Cor	37	4	53-55	2022	215/j.cnki.jbyfkztb.2	2020	2	10	3	Western	Gansu	Dingxi	35.13	104.45	Residential area	35	230	15.22	7	70	51	N/A	25	10	71.43	100.00	77.10	97.10	88.60	87.10	N/A	Meat products	D1	Enteritidis	N/A	N/A	Y	Y	Y	N/A	N/A	N/A	N/A	N/A	35	0	Food poisoning
1093	文燕;张梦寒;王小	hang Menghan;V	食源性疾病暴发事	Salmonella Ente	上海预防医学	hai Preventive M	34	7	665-670	2022	428/j.cnki.sjpm.2022	2021	1	6	4	Eastern	Jiangsu	Suzhou	31.19	120.37	Office	201	420	47.86	N/A	N/A	N/A	N/A	N/A	N/A	N/A	82.65	60.20	13.27	N/A	21.43	50.00	Egg products	D1	Enteritidis	N/A	, SAM, CZO, NAL, I	Y	Y	Y	Y	N/A	N/A	N/A	N/A	201	0	Food poisoning
1094	柏生;黄友志;邱	;Huang Youzhi;Q	源性疾病事件的调	alysis of a salmor	应用预防医学	ed preventive me	28	3	225-228	2022	-	2020	2	10	3	Eastern	Guangdong	Maoming	21.4	110.53	School	43	N/A	N/A	N/A	N/A	N/A	N/A	N/A	N/A	N/A	100.00	100.00	70.00	N/A	N/A	70.00	Meat products	E1	London、Weltevrede	N/A	N/A	Y	Y	Y	Y	N/A	N/A	N/A	N/A	N/A	N/A	Food poisoning
1095	张良军;赵辉;刘凡	iangjun;Zhao Hui	沙门菌引起聚集性	ceability analysis	职业与健康	cupation and hea	38	11	1460-1464	2022	329/j.cnki.zyyjk.202	2020	2	7	3	Eastern	Beijing	Yanqing	39.55	116.24	Construction site	10	N/A	N/A	29	64	42	N/A	10	0	100.00	100.00	100.00	100.00	100.00	100.00	N/A	N/A	C3	Kentucky	N/A	NAL, GEN, CIP, AZ	Y	Y	Y	Y	Y	Y	ALDI-TOF-MS、W	N/A	N/A	N/A	N/A
1096	欧秀华;邱艺燕;	u Xiuhua;Qiu Yiya	门菌食物中毒的实	of a case of Salm	热带病与寄生虫学	Diseases and Par	20	2	89-90+98	2022	-	2020	2	8	6	Eastern	Fujian	Xiamen	24.27	118.06	Restaurant	7	N/A	N/A	N/A	N/A	N/A	N/A	N/A	N/A	N/A	100.00	100.00	100.00	100.00	100.00	100.00	ocessed grain produ	D1	Enteritidis	N/A	N/A	Y	Y	Y	N/A	N/A	Y	N/A	N/A	N/A	N/A	Food poisoning
1097	彩云;宗华;殷静;罗	ng Hua;Yin Jing;L	门菌食物中毒的病	tics of a case of S	中国卫生检验杂志	ournal of Health	32	7	818-820+831	2022	-	2020	2	8	N/A	Western	Chongqing	Nanan	29.35	106.33	Restaurant	N/A	N/A	N/A	N/A	N/A	N/A	N/A	N/A	N/A	N/A	N/A	N/A	N/A	N/A	N/A	N/A	Others	E1	Give	N/A	TET, SXT, AMP, AZ	Y	Y	Y	Y	N/A	N/A	N/A	N/A	N/A	N/A	Food poisoning
1098	张耕军;李志峰;王	ongjun;Li Zhifeng	起沙门菌食源性疾	lmonella foodbo	预防医学情报杂志	eventive Medicin	38	3	369-376	2022	-	2020	2	9	6	Western	Chongqing	Changshou	29.35	106.33	mobile street stall	51	N/A	N/A	N/A	N/A	N/A	N/A	N/A	N/A	N/A	100.00	100.00	100.00	100.00	100.00	N/A	Pastries	D1	Enteritidis	N/A	SAM, POL, CZO, CI	Y	Y	Y	Y	N/A	N/A	MALDI-TOF-MS	N/A	N/A	N/A	Food poisoning
1099	孙中兴;蒋元强;盛	ng;Jiang Yuanqiar	两种沙门氏菌引起	caused by Vibrio	实用预防医学	cal preventive me	29	3	350-352	2022	-	2020	2	5	2	Eastern	Shanghai	Songjiang	31.14	121.29	Restaurant	7	70	10.00	23	86	N/A	N/A	5	2	71.43	100.00	N/A	100.00	42.86	52.86	N/A	Aquatic products	B、E1	London、Typhimuriu	N/A	N/A	Y	Y	Y	Y	N/A	N/A	N/A	N/A	N/A	N/A	Food poisoning
1100	媚;洪敏丽;陈永东;	ong Minli;Chen Yo	门菌食物中毒的调	sis of a case of Sa	海峡预防医学杂志	rnal of Preventiv	28	1	93-95	2022	-	2019	3	5	3	Eastern	Guangdong	Shenzhen	22.33	114.07	School	14	N/A	N/A	17	22	18	N/A	10	4	71.43	100.00	100.00	85.71	N/A	64.29	N/A	Pastries	D1	Enteritidis	N/A	N/A	Y	Y	Y	Y	Y	N/A	N/A	N/A	14	0	Food poisoning
1101	钰;时玉雯;刘铭;刘	Yuwen;Liu Ming	食物中毒的溯源分	tibility testing o	保健医学研究与实践	are research and	19	1	42-45	2022	-	2019	3	8	1	Eastern	Shandong	Jinan	36.4	117	Restaurant	6	N/A	N/A	7	77	31.83	24.29	1	5	16.67	100.00	100.00	100.00	100.00	100.00	100.00	Meat products	E1	Fuhlsbuettel*	N/A	TET, MNO, SMZ, A	Y	Y	Y	Y	N/A	N/A	N/A	N/A	N/A	N/A	Food poisoning
1102	跃轩;董岳云;张震;	ueyun;Zhang Qia	食源性疾病暴发	utbreak of Salmo	实用预防医学	cal preventive me	29	1	85-87	2022	-	2018	4	8	3	Eastern	Zhejiang	Quzhou	28.58	118.52	Restaurant	72	198	36.36	N/A	N/A	N/A	N/A	N/A	N/A	N/A	84.72	90.28	58.33	31.94	30.56	34.72	Pastries	D1	Enteritidis	N/A	N/A	Y	Y	Y	Y	N/A	N/A	N/A	N/A	N/A	N/A	Food poisoning
1103	康龙;陈上士;李国	ong;Sergeant Ch	食源性疾病暴发事	of a Salmonella E	海峡预防医学杂志	rnal of Preventiv	27	6	73-75	2021	-	2019	2	6	5	Eastern	Fujian	Yongan	25.58	117.23	Restaurant	28	N/A	N/A	1	55	N/A	N/A	7	21	25.00	100.00	100.00	100.00	N/A	100.00	N/A	Pastries	D1	Enteritidis	N/A	N/A	Y	Y	Y	Y	N/A	N/A	N/A	N/A	N/A	N/A	Food poisoning
1104	滨;张宇;杨秀程;	Zhang Yu;Yang Xi	门菌所致食物中毒	estigation on foo	预防医学情报杂志	eventive Medicin	37	12	1660-1663	2021	-	2020	1	9	3	Western	Sichuan	Barkam	32.01	102.02	Residential area	29	172	16.86	3	79	43	N/A	13	16	44.83	72.41	68.97	93.10	55.17	27.59	N/A	Meat products	N/A	N/A	N/A	N/A	Y	Y	N/A	N/A	N/A	N/A	N/A	N/A	28	0	Food poisoning
1105	邵成;刘翔;郝琦;田	Liu Xiang;Hao Qi	食物中毒事件的调	ysis of a food poi	疾病预防控制通报	evention and Cor	36	6	30-33+73	2021	215/j.cnki.jbyfkztb.21	2020	1	9	3	Western	Ningxia	N/A	38.27	106.16	Residential area	13	23	56.52	10	72	N/A	N/A	6	7	46.15	84.62	84.62	100.00	76.92	30.77	54.55	Meat products	B	Typhimurium 4,5,12,i:	N/A	T, NAL, CHL, CTX	Y	Y	Y	Y	N/A	N/A	N/A	N/A	12	1	Food poisoning
1106	唐琳[1]	Tang Lin[1]	菌污染的皮蛋引起	ed by eating pres	刊数据库（文摘版	urnal Database (A	-	6	205-207	2022	-	2021	1	6	2	Western	Sichuan	Yibin	28.47	104.34	Residential area	6	18	33.33	N/A	N/A	N/A	N/A	1	5	16.67	100.00	66.67	100.00	50.00	16.67	N/A	Egg products	B	Typhimurium	N/A	N/A	Y	Y	Y	N/A	N/A	N/A	N/A	N/A	6	0	Food poisoning
1107	王君[1];严梅[1]	ang Jun[1];Yan Me	食物中毒的快速处	ability analysis of	期科技期刊数据库	l Technology Journal	-	3	116-118	2022	-	2015	7	1	3	Western	Chongqing	Bishan	29.35	106.33	Restaurant	234	660	35.45	N/A	N/A	N/A	N/A	N/A	N/A	N/A	100.00	100.00	100.00	100.00	100.00	100.00	N/A	D1	Enteritidis	N/A	N/A	Y	Y	Y	N/A	N/A	N/A	N/A	N/A	N/A	N/A	Food poisoning
1108	罗江湖[1]	Luo Jianghu[1]	学食物中毒检验与	investigation pr	刊数据库（文摘版	urnal Database (A	-	5	209-211	2022	-	2020	2	3	1	Western	Guizhou	Qiandongnan	26.51	108.56	School	72	N/A	N/A	N/A	N/A	N/A	N/A	N/A	N/A	N/A	100.00	N/A	100.00	100.00	100.00	N/A	Others	E4	Senftenberg	N/A	N/A	Y	Y	Y	N/A	N/A	Y	N/A	N/A	72	0	Food poisoning
1109	胡峻[1]	Hu Jun[1]	食源性肠道传染病	analysis of food	保健医学研究与实践	are research and	19	11	33-36	2022	-	2020	2	6	3	Central	Jiangxi	Ganzhou	28.52	114.56	Restaurant	68	N/A	N/A	3	60	N/A	N/A	30	38	44.12	100.00	100.00	29.41	30.88	58.82	27.94	Meat products	D1	Enteritidis	N/A	N/A	Y	Y	Y	N/A	Y	N/A	N/A	N/A	68	0	Food poisoning
1110	海[1];周其开[1];方	[1];Zhou Qikai[1];Z	门菌引起食物中毒	causing food pois	期科技期刊数据库	l Technology Journal	-	5	183-183	2021	-	2019	2	12	N/A	Western	Yunnan	Chuxiong	25.01	101.32	Residential area	N/A	N/A	N/A	N/A	N/A	N/A	N/A	N/A	N/A	N/A	N/A	N/A	N/A	N/A	N/A	N/A	Meat products	D1	Enteritidis	N/A	N/A	Y	Y	Y	N/A	N/A	Y	N/A	N/A	N/A	N/A	Food poisoning
1111	盘珍梅[1];蔡周梅[	enmei[1];Cai Zhou	对两个家庭肠炎沙	soning in two fam	刊数据库（全文版	urnal Database (Fu	-	4	239-240	2021	-	2020	1	8	N/A	Western	Guangxi	Wuzhou	23.29	111.2	Residential area	N/A	N/A	N/A	N/A	N/A	N/A	N/A	N/A	N/A	N/A	N/A	N/A	N/A	N/A	N/A	N/A	Meat products	D1	Enteritidis	N/A	N/A	Y	Y	Y	Y	N/A	N/A	N/A	N/A	N/A	N/A	Food poisoning
1112	婧[1];崔立周[1];祖	[1];Cui Lizhou[1];	肠炎沙门菌食物中	a Enteritidis food	医学动物防制	edical animal con	37	10	1000-1003	2021	-	2019	2	5	3	Eastern	Hebei	Baoding	38.51	115.3	Restaurant	80	248	32.26	2	81	35	N/A	48	32	60.00	77.50	96.25	68.75	N/A	25.00	N/A	Aquatic products	D1	Enteritidis	N/A	N/A	Y	Y	Y	Y	Y	N/A	N/A	N/A	80	0	Food poisoning
1113	o YS, Chiou CS, Ch	-	French toast sandwic	-	Epidemiol Infect	-	-	-	-	2020	1017/S095026882000	2018	2	4	10	Eastern	Taiwan	N/A	25.03	121.3	Restaurant	47	98	47.96	1	68	N/A	N/A	32	15	68.09	100.00	68.09	65.96	N/A	N/A	N/A	Egg products	D1	Enteritidis	N/A	N/A	Y	N/A	Y	Y	N/A	N/A	WGS	N/A	46	1	Food poisoning
1114	ng AS, Liao YS, Liu	-	ontaminated with mul	-	Foodborne Pathog D	-	-	-	-	2014	10.1089/fpd.2013.166	2010	4	7	4	Eastern	Taiwan	N/A	25.03	121.3	mobile street stall	324	886	36.57	1	85	N/A	N/A	134	190	41.36	89.20	47.53	69.75	N/A	17.28	32.72	Pastries	C1、D1	Enteritidis、Virchow	N/A	N/A	Y	N/A	Y	Y	N/A	N/A	N/A	N/A	324	0	Food poisoning
1115	, Tung YL, Hwang S	-	caused by Salmone	-	BMC Infect Dis	-	-	-	-	2004	0.1186/1471-2334-4-	2001	3	7	2	Eastern	Taiwan	N/A	25.03	121.3	mobile street stall	28	34	82.35	3	71	N/A	N/A	12	16	42.86	100.00	100.00	100.00	N/A	85.71	7.14	Pastries	D1	Enteritidis	N/A	N/A	Y	N/A	Y	Y	N/A	N/A	N/A	N/A	N/A	N/A	Food poisoning
1116	You SL, Chuang YC,	-	of a typhoid outbreak	-	Int J Epidemiol	-	-	-	-	1989	10.1093/ije/18.1.254	1983	6	7	34	Eastern	Taiwan	Hsinchu	24.79	120.95	Residential area	52	2772	1.88	1	40	N/A	N/A	24	28	46.15	N/A	N/A	N/A	N/A	N/A	N/A	N/A	D1	Typhi	N/A	N/A	Y	Y	Y	N/A	N/A	N/A	N/A	N/A	N/A	N/A	id/paratyphoid fever
1117	Yang C, Liu H, Du X,	-	rica Serovar Enteritic	-	Front Microbiol	-	-	-	-	2021	3389/fmicb.2021.779	2021	0	3	3	Eastern	Beijing	N/A	39.55	116.24	Office	225	324	69.44	N/A	N/A	24.1	N/A	223	2	99.11	100.00	100.00	63.11	51.11	63.56	68.44	Egg products	D1	Enteritidis	N/A	IP, SOX, NAL, TET	Y	Y	Y	Y	Y	N/A	WGS	N/A	N/A	N/A	Food poisoning
1118	Y, Wang YQ, Zou X	-	caused by Salmonell	-	Chin Med J (Engl)	-	-	-	-	2020	97/CM9.0000000000	2018	2	6	3	Eastern	Shandong	Yantai	37.32	121.24	Office	75	N/A	N/A	N/A	N/A	N/A	N/A	33	42	44.00	89.33	52.00	89.33	65.33	45.33	N/A	N/A	F	Aberdeen	N/A	CZO	Y	N/A	Y	Y	Y	N/A	N/A	CTX, LVX	75	0	Food poisoning
1119	Zhou X, Xu X, Yang	-	Caused by Multidrug	-	Front Microbiol	-	-	-	-	2020	.3389/fmicb.2020.00	2017	3	5	3	Central	Shanxi	Changzhi	36.11	113.06	Residential area	11	12	91.67	1	50	N/A	N/A	4	7	36.36	90.91	81.82	54.55	45.45	36.36	36.36	N/A	B	Typhimurium	N/A	AMP, NAL, SDI, SM	Y	Y	Y	Y	N/A	N/A	WGS	N/A	11	0	Food poisoning
1120	van PSL, Li Y, Lin Y	-	eritidis Isolates in Ou	-	Emerg Infect Dis	-	-	-	-	2020	0.3201/eid2604.1914	2018	2	6	4	Eastern	Guangdong	Shenzhen	22.33	114.07	Restaurant	10	N/A	N/A	N/A	N/A	N/A	N/A	N/A	N/A	N/A	100.00	100.00	N/A	70.00	70.00	N/A	Meat products	D1	Enteritidis	N/A	N/A	Y	N/A	Y	Y	N/A	N/A	WGS	N/A	N/A	N/A	Food poisoning
1121	u Q, Zhu Y, Que F, I	-	reak and Sporadic Ill	-	Foodborne Pathog D	-	-	-	-	2019	10.1089/fpd.2018.260	2018	1	8	1	Eastern	Shanghai	Jinshan	31.14	121.29	Office	28	N/A	N/A	N/A	N/A	N/A	N/A	9	19	32.14	78.57	N/A	78.75	85.71	85.71	N/A	N/A	C1	Livingstone	N/A	N/A	Y	Y	Y	Y	N/A	N/A	N/A	N/A	28	0	Food poisoning
1122	Ye Y, Guo B, Wang	-	ion of an outbreak of	-	Int J Biol Macromol	-	-	-	-	2018	016/j.ijbiomac.2017.0	2016	2	4	N/A	Eastern	Jiangsu	Nanjing	32.03	118.46	N/A	N/A	N/A	N/A	N/A	N/A	N/A	N/A	N/A	N/A	N/A	N/A	N/A	N/A	N/A	N/A	N/A	Meat products	B	Schwarzengrund	N/A	SMZ	Y	N/A	Y	Y	N/A	N/A	WGS	N/A	N/A	N/A	Food poisoning
1123	, Zhang HN, Jiang K	-	Serotype Isangi Isola	-	Foodborne Pathog D	-	-	-	-	2017	10.1089/fpd.2016.226	2016	1	4	4	Eastern	Shandong	Yantai	37.32	121.24	Residential area	33	74	44.59	27	77	N/A	N/A	16	17	48.48	93.90	57.60	87.90	66.67	30.30	24.30	Meat products	C1	Isangi	N/A	N/A	Y	N/A	Y	Y	N/A	N/A	WGS	N/A	33	0	Food poisoning
1124	X, Zhou Y, Pang B,	-	yphoid Fever Caused	-	PLoS Negl Trop Dis	-	-	-	-	2015	016/j.meegid.2015.1	2010	5	5	365	Western	Yunnan	N/A	25.04	102.42	Residential area	601	N/A	N/A	N/A	N/A	N/A	N/A	N/A	N/A	N/A	N/A	100.00	N/A	N/A	N/A	84.00	N/A	A	Paratyphi A	N/A	N/A	Y	Y	Y	Y	N/A	N/A	WGS	N/A	N/A	N/A	id/paratyphoid fever
1125	Z, Li L, Li Z, Chen	-	h a contaminated foo	-	Epidemiol Infect	-	-	-	-	2016	371/journal.pone.013	2013	3	6	4	Western	Sichuan	Meishan	29.36	103.29	School	399	1268	31.47	5	17	11.3	0.46	262	137	65.66	56.00	95.00	38.00	22.00	N/A	51.00	Meat products	B	Typhimurium	N/A	N/A	Y	N/A	Y	Y	N/A	N/A	N/A	CAZ, ATM	399	0	Food poisoning
1126	Z, Su Ch, Huang J,	-	ferent Salmonella ser	-	Jpn J Infect Dis	-	-	-	-	2015	97/INF.00000000000	2012	3	10	4	Eastern	Fujian	Xiamen	24.27	118.06	School	56	22404	0.25	17	22	N/A	N/A	20	36	35.71	N/A	84.00	89.00	80.00	43.00	N/A	Pastries	B、D1	Chester、Enteritidis	N/A	N/A	Y	N/A	Y	Y	Y	N/A	N/A	N/A	56	0	Food poisoning
1127	g ML, Yang J, Wu C	-	ng school: a commur	-	Epidemiol Infect	-	-	-	-	2010	1017/S095026881000	2004	6	11	14	Western	Guangxi	Guilin	25.17	110.17	School	394	1092	36.08	N/A	N/A	N/A	N/A	203	191	51.52	28.00	15.00	12.16	N/A	N/A	59.00	N/A	A	Paratyphi A	N/A	NAL	Y	N/A	Y	N/A	N/A	N/A	N/A	X, CTX, AMK, OFX	394	0	id/paratyphoid fever
1128	HM,FontaineR;Cent	-	ry workers--Huizhou	-	MMWRSuppl	-	-	-	-	2006	-	2004	2	7	6	Eastern	Guangdong	Huizhou	23.05	114.22	Factory	199	1496	13.30	17	50	N/A	N/A	154	55	77.39	87.00	66.00	78.00	28.00	18.00	N/A	Pastries	D1	Enteritidis	N/A	N/A	Y	N/A	Y	N/A	N/A	N/A	N/A	N/A	N/A	N/A	Food poisoning
1129	C,XieHH,TanXP,Wa	-	Salmonella typhimu	-	JDiarrhoealDisRes	-	-	-	-	1990	-	1987	3	8	5	Eastern	Fujian	Yongan	25.58	117.23	Residential area	1113	265605	0.42	N/A	N/A	N/A	N/A	N/A	N/A	N/A	94.80	95.00	84.50	N/A	48.20	N/A	Frozen food	B	Typhimurium	N/A	N/A	Y	N/A	Y	N/A	N/A	N/A	N/A	GEN, FRZ	1112	1	Food poisoning
1130	Wu Y, Guo Y, Shi Y,	-	of Salmonella Enteriti	-	Front Microbiol	-	-	-	-	2022	3389/fmicb.2022.772	2019	3	9	N/A	Eastern	Guangdong	Dongguan	23.02	113.45	School	157	N/A	N/A	2	61	N/A	N/A	73	48	46.50	89.30	98.40	87.60	N/A	79.30	N/A	Pastries	D1	Enteritidis	N/A	N/A	Y	Y	Y	N/A	Y	N/A	WGS	N/A	N/A	N/A	Food poisoning
1131	u G, Liu B, Lin C, Zh	-	eak by Waterborne I	-	China CDC Wkly	-	-	-	-	2022	.46234/ccdcw2022.(	2022	0	1	35	Eastern	Beijing	N/A	39.55	116.24	Residential area	23	106	21.70	22	45	N/A	N/A	8	15	34.78	47.83	82.61	100.00	100.00	100.00	100.00	N/A	D1	Typhi	N/A	IP, CHL, SXT, ATM	Y	N/A	Y	N/A	N/A	N/A	WGS	MEM	N/A	N/A	id/paratyphoid fever
1132	, Ruan F, Li Z, Huar	-	fection in a tourist gro	-	J Infect	-	-	-	-	2022	.1016/j.jinf.2022.02.(	2019	3	6	1	Eastern	Hong kong	N/A	21.23	115.12	Restaurant	6	18	33.33	42	62	N/A	N/A	3	3	50.00	100.00	100.00	83.33	100.00	N/A	66.67	Meat products	I	Hvittingfoss	N/A	N/A	Y	N/A	Y	Y	N/A	N/A	N/A	N/A	N/A	N/A	Food poisoning
1133	, Zhao L, Ji S, Li T, K	-	hoid Outbreak Cause	-	Front Med (Lausann	-	-	-	-	2022	.3389/fmed.2022.753	2016	6	1	120	Eastern	Shandong	Linyi	35.03	118.2	Residential area	31	N/A	N/A	1	55	N/A	N/A	14	17	45.16	N/A	N/A	N/A	N/A	N/A	N/A	N/A	D1	Typhi	N/A	SXT	Y	N/A	Y	Y	Y	N/A	ALDI-TOF-MS、W	N/A	N/A	N/A	id/paratyphoid fever
1134	Lu MC, Lai YC, Che	-	ely drug-resistant Sa	-	Glob Antimicrob Res	-	-	-	-	2022	.1016/j.jgar.2022.03.	2020	2	12	29	Eastern	Taiwan	N/A	25.03	121.3	Hospital	10	N/A	N/A	N/A	N/A	N/A	N/A	N/A	N/A	N/A	N/A	N/A	N/A	N/A	N/A	N/A	N/A	C2	Goldcoast	N/A	CRO, FEP, TZP, ET	Y	N/A	Y	Y	N/A	N/A	WGS	ETP, AZI	9	1	-associated infection

Sheet2: Extracted information for meta-analysis

Publication information | Outbreak information — Spatiotemporal background (Time; Space); Patient information — Case collection

	Author	Author_E.T.	Title	Title_E.T.	Journal	Journal_E.T.	Volume	Issue	Page	Year	DOI	Year (onset)	HB / y	Month	Duration	Economic regio	Province	Prefecture	Latitude / °N	Longitude / °E	Setting	ber of New Case	le Exposed at st	tack Rate (AR)	Minimum age /	Maximum age /
2	栗.滕毅.王玲.刘	ng Yi.Wang Li	门菌引起的食物	of food poison	预防医学情报杂	ventive Medici	37	1	48-52,57	2021	-	2019	2	7	1	Western	Sichuan	Chengdu	30.4	104.04	School	26	270	9.60	18	37
3	丁.梅建华.陈秀	g.Mei Jianhua.	沙门菌食源性疾	ella Orenenbu	预防医学	eventive Medic	33	6	602-606	2021	ki.issn2096-508	2019	2	6	4	Eastern	Zhejiang	Lishui	28.27	119.54	Residential area	18	280	6.43	45	70
6	星威.施惠军.陆冠	Shi Huijun.Lu X	食物中毒的流行	nalysis of Salm	医学动物防制	lical animal co	37	2	175-178,182	2021	529/yxdwfz2021	2018	3	6	8	Eastern	Jiangsu	Zhangjiagang	31.52	120.32	School	47	4200	1.12	19	22
7	云龙.宋士利.郑	ng.Song Shili.Z	炎沙门菌食源性	monella Enter	中国食品卫生杂	Food Hygiene	33	5	583-586	2021	590/j.cjfh.2021.	2017	4	8	3	Eastern	Zhejiang	Hangzhou	30.16	120.1	Nursing home	23	407	5.65	21	80
9	金金.许玉成.张	injin.Xu Yuche	食物中毒事件	incident of Sa	医学动物防制	lical animal co	37	1	23-26	2021	529/yxdwfz2021	2019	2	8	2	Eastern	Guangdong	Shenzhen	22.33	114.07	Restaurant	11	16	68.75	14	50
10	.田美娜.陈磊.罗	lina.Chen Lei.L	的沙门菌食源性	disease outb	医学动物防制	lical animal co	37	1	88-91	2021	529/yxdwfz2021	2019	2	5	2	Eastern	Hebei	Shijiazhuang	38.02	114.3	Restaurant	68	104	65.38	2	78
12	平.潘丽峰.胡卉.	.Pan Lifeng.Hu	一起沙门菌食	poisoning inci	中国食品卫生杂	Food Hygiene	33	5	627-632	2021	590/j.cjfh.2021.	2018	3	9	3	Eastern	Shanghai	Pudong	31.14	121.29	Restaurant	51	1270	4.02	11	18
13	李卫华.马佳平.	Weihua.Ma Jia	性疾病暴发的流	of foodborne d	健康大视野	Healthy vision	-	19	23,25	2021	-	2019	2	8	1	Eastern	Shandong	Dezhou	37.26	116.17	Office	15	98	15.31	21	67
14	轩.蓝岳云.张茜.	eyun.Zhang Q	门菌食源性疾病	ella Enteritidis	预防医学	eventive Medic	33	6	621-623	2021	ki.issn2096-508	2019	2	9	3	Eastern	Zhejiang	Quzhou	28.58	118.52	School	71	3602	1.97	N/A	N/A
15	婵.曹应琼.梅丽	Cao Yingqiong	沙门氏菌食物中	la Food Poisor	医学信息	edical informat	34	10	158-160	2021	issn.1006-1959.2	2020	1	6	2	Western	Sichuan	Chengdu	30.4	104.04	Residential area	7	22	31.82	3	76
16	王世萍.	Wang Shiping	引起的沙门氏菌	oisoning incid	寄生虫病与感染性	ases and infec	19	1	38-41	2021	-	2019	2	5	1	Western	Sichuan	Xichang	27.54	102.16	Residential area	28	200	14.00	22	66
18	令.炊慧霞.李艳芬	Huixia.Li Yanfe	引起食源性疾病	disease outbr	中国卫生检验杂	urnal of Health	31	9	1037-1039,1043	2021	-	2019	2	2	3	Central	Henan	Pingdingshan	33.44	113.17	Restaurant	62	260	23.85	N/A	N/A
21	燕飞.刘东妹.方	.Liu Dongmei.	引起的学校食源	dborne illness	中国食品卫生杂	Food Hygiene	33	5	595-599	2021	590/j.cjfh.2021.	2020	1	10	8	Eastern	Guangdong	Zhuhai	22.17	113.34	School	74	3974	1.86	N/A	N/A
25	王雨荷.	Wang Yuhe.	染引起的食源性	borne illness c	山西医药杂志	xi Medical Jou	49	24	3400-3402	2020	issn.0253-9926.2	2020	0	5	2	Central	Anhui	Bengbu	32.56	117.21	Restaurant	55	85	64.71	4	78
29	.秦友燕.韩丹丹.	ouyan.Han Dan	门氏菌引起的食	ning incident c	广西医科大学学	uangxi Medic	37	11	2074-2078	2020	.cnki.45-1211/r.2	2018	2	8	4	Western	Guangxi	Guilin	25.17	110.17	Restaurant	295	520	56.73	8	71
31	刘永兴.	Liu Yongxing.	引起的肠炎沙门	teritidis food	东方药膳	ntal medicinal	-	19	96	2020	-	2020	0	11	3	Western	Chongqing	Tongliang	29.35	106.33	Restaurant	27	343	7.87	<1	80
33	黄洁.	Huang Jie.	肠炎沙门氏菌食	nteritidis food	信息文摘（连续	mation abstrac	20	81	278-279	2020	issn.1671-3141.2	2018	2	7	2	Eastern	Shandong	Qingdao	36.03	120.18	Restaurant	37	224	16.52	8	72
34	津津.孙成玺.苗	n.Sun Chengxi	桑吉沙门菌引起	ak caused by	疾病监测	sease surveillan	35	9	866-870	2020	issn.1003-9961.2	2018	2	7	2	Eastern	Zhejiang	Yuyao	30.02	121.1	Factory	6	6	100.00	N/A	N/A
37	.张宏亮.张景平.	qliang.Zhang	伤寒沙门菌食物	Typhimurium	海峡预防医学杂	nal of Preventi	26	4	87-89	2020	-	2019	1	11	4	Eastern	Fujian	Longyan	25.06	117.01	mobile street stal	67	580	11.55	1	66
38	海丽敏.余林.袁	min.Yu Lin.Yua	餐引起沙门氏菌	oisoning incident	食品安全导刊	ood safety guic	-	23	36-37	2020	-	2019	1	8	2	Western	Sichuan	Chengdu	30.4	104.04	Residential area	3	3	100.00	15	69
41	沱中.卢志敏.卢	zhong.Lu Zhin	旅行团食源性	ses in a mainl	国境卫生检疫	Health and Qu	43	3	182-183,194	2020	/j.1004-9770.20	2019	1	7	2	Eastern	Macau	N/A	21.33	115.07	Restaurant	13	42	30.95	27	57
42	.雷务年.秦燕妮.	of thunder.Qi	氏菌引起食物中	of food poison	保健文汇	Health Wenhu	-	17	278-279	2020	issn.1671-5217.2	2019	1	7	1	Western	Guangxi	Guiping	23.22	110.04	Residential area	24	50	48.00	4	87
45	.单桂花.彭明益.	nanthus.Peng	大雷登沙门氏菌	vreden food p	医学动物防制	lical animal co	36	3	300-304	2020	529/yxdwfz2020	2018	2	6	1	Eastern	Guangdong	Guangzhou	23.08	113.14	Restaurant	17	30	56.67	17	44
46	奇.邹婷.林钢.彭	u Ting.Lin Gan	起肠炎沙门菌感	nteritidis infec	预防医学情报杂	ventive Medici	36	2	240-244	2020	-	2019	1	1	3	Western	Sichuan	Chengdu	30.4	104.04	Restaurant	82	603	13.60	5	73
49	.邱春晖.李丽丽.	Qiu Chunhui.Li	沙门菌食物中	s of Salmonell	海峡预防医学杂	nal of Preventi	26	1	79-80	2020	-	2019	1	5	2	Eastern	Fujian	Yongan	25.58	117.23	Residential area	14	24	58.33	1	68
50	敏丽.刘渠.甘莉	Minli.Liu Qu.G	食物中毒的病原	iology of Salm	现代预防医学	n preventive m	47	1	123-126	2020	-	2017	3	5	2	Eastern	Guangdong	Shenzhen	22.33	114.07	Residential area	8	13	61.54	5	68
51	赵保宁.田疆.	Baoning.Tian	沙门氏菌引发的	ing caused by	宁夏医科大学学	Ningxia Medica	41	12	1294-1296	2019	ki.issn1674-630	2017	2	9	3	Western	Ningxia	Yinchuan	38.27	106.16	School	47	110	42.73	17	19
52	良.徐瑞雪.魏泽	ixue.Wei Zemi	起的食源性疾病	orne disease c	首都公共卫生	pital public hea	13	6	296-298	2019	-	2018	1	7	2	Eastern	Beijing	Xicheng	39.55	116.24	Restaurant	10	17	58.82	23	75
57	静.张静.王姗.金	ling.Wang Sha	事件的病原学及	molecular biol	医药前沿	Medical Fronti	9	34	242-243	2019	-	2019	0	6	1	Eastern	Shanghai	Hongkou	31.14	121.29	Residential area	3	3	100.00	27	85
59	次.张建新.张丽	ng Jianxin.Zhan	沙门氏菌食物	Enteritidis fo	食品安全质量检测	Safety and Qu	10	5	1394-1400	2019	issn.2095-0381.2	2018	1	3	4	Eastern	Jiangsu	Suzhou	31.19	120.37	Factory	67	10221	0.66	N/A	N/A
60	庭欢.凌水权.赵	n.Lingshui Qu	清城区一起食源	k in Qingcher	中国热带医学	se tropical me	19	11	1063-1067	2019	j.cnki.46-1064/r.	2019	0	2	3	Eastern	Guangdong	Qingyuan	23.42	113.01	Restaurant	90	1250	7.20	5	75
62	建香.刘晓骏.钱	g.Liu Xiaojun.	暴发疫情的耐药	lecular typing	医学动物防制	lical animal co	35	11	1033-1036	2019	529/yxdwfz2019	2016	3	6	115	Eastern	Jiangsu	Jiangyin	31.54	120.17	Residential area	92	460000	0.02	2	69
64	韩京娣.张丹梅.	ngdi.Zhang D	氏菌食物中毒流	ion of a case o	养生保健指南	and Wellness	-	27	369	2019	-	2015	4	5	4	Western	Ningxia	Yinchuan	38.27	106.16	Restaurant	64	100	64.00	1	56
66	徽华.姚建香.李	ua.Yao Jianxia	甲型副伤寒暴发	eak of paratyp	中国学校卫生	nese school he	40	4	616-618	2019	cnki.1000-9817.	2016	3	12	29	Eastern	Jiangsu	Jiangyin	31.54	120.17	School	28	1712	1.64	N/A	N/A
67	乐.姚楠.林杨.陈	Nan.Lin Yang.	暴发疫情的病原	ty analysis of	中国卫生检验杂	urnal of Health	29	5	617-620	2019	-	2017	2	1	3	Western	Sichuan	Mianyang	31.3	104.42	Residential area	18	32	56.25	N/A	N/A
68	李红霞.	Li Hongxia.	起的1起食源性	idis1Analysis c	现代医药卫生	medicine and	35	17	2759-2760	2019	issn.1009-5519.2	2018	1	7	2	Western	Chongqing	Dadukou	29.35	106.33	Restaurant	8	20	40.00	N/A	N/A
71	李俊.吴静.王帅	Wu Jing.Wang	某酒店疑似食物	ted food poisc	医科论坛电子杂	Forum Electro	-	8	536	2019	-	2017	2	9	2	Eastern	Hainan	Haikou	20.02	110.2	Restaurant	25	984	2.54	5	56
73	胡世雄.贾华云.沈	ng.Jia Huayun	门氏菌共同引起	ess outbreak c	实用预防医学	al preventive m	26	7	857-860	2019	issn.1006-3110.2	2015	4	6	4	Central	Hunan	Liuyang	28.09	113.37	School	155	1439	10.77	10	51
74	夏军林.	Xia Junlin.	镇一起伤寒暴发	d fever outbre	河南预防医学杂	nal of Preventi	30	7	541-543	2019	i.hnjpm.1006-84	2018	1	5	164	Central	Henan	Zhumadian	32.58	114.01	Residential area	123	55909	0.22	2	85
76	周焕宁.	Zhou Huanning	小学教职工及	reak of salmon	微量元素与健康研	nents and heal	35	2	49-50	2018	-	2016	2	6	4	Eastern	Guangdong	Guangzhou	23.08	113.14	School	73	175	41.71	6	75
77	娟.谢文奇.刘承	Xie Wenqi.Liu	沙门氏菌食物	family cluster	赣南医学院学报	Gannan Medi	38	12	1228-1229,1238	2018	issn.1001-5779.2	2018	0	5	1	Central	Jiangxi	Ruijin	25.53	116.01	Residential area	8	8	100.00	7	68
78	赵健.李文玲.	ao Jian.Li Wen	沙门菌引起的食	d poisoning c	中国城乡企业卫	in urban and	33	11	76-78	2018	/j.1003-5052.20	2018	0	3	4	Eastern	Tianjin	Peace	39.02	117.12	Restaurant	99	252	39.29	2	85
79	陈亚娜.曹信金.	n Yana.Cao Xi	菌食物中毒事件	incident of S	海峡预防医学杂	nal of Preventi	24	1	88-89	2018	-	2017	1	5	2	Eastern	Fujian	Xiamen	24.27	118.06	School	36	1125	3.20	7	11
80	.黄葵青.朱世瑜.	ng Kuiqing.Zh	门氏菌食物中毒	s of a case of s	中国农村卫生事业	ural health ma	38	2	204-206	2018	issn.1005-5916.2	2012	6	7	1	Eastern	Zhejiang	Yiwu	29.18	120.04	Restaurant	18	26	69.23	26	62
81	王婧.何旭鑫.	ang Jing.He Xu	门氏菌引起的食	food poisonir	信息文摘（连续	mation abstrac	18	51	254,256	2018	cnki.1671-3141.	2017	1	7	2	Eastern	Hebei	Chengde	40.59	117.57	Residential area	19	19	100.00	18	60
82	韩祖源.	Han Zuyuan.	引起的食源性疾	disease outb	心理医生	Psychologist	24	14	348-349	2018	-	2017	1	8	2	Western	Guangxi	Wuzhou	23.29	111.2	Restaurant	50	440	11.36	13	84
83	赵虹.	Zhao Hong.	副伤寒沙门菌暴	outbreak of S	海峡预防医学杂	nal of Preventi	24	4	96-97	2018	-	2016	2	4	2	Western	Yunnan	Baoshan	25.08	99.1	Residential area	164	410	40.00	6	62
84	荣生.王晓燕.张	Vang Xiaoyan.	发性急性胃肠炎	er of acute ga	解放军预防医学杂	edicine of the	36	6	806-807,809	2018	-	2017	1	7	2	Eastern	Shandong	Qingzhou	36.42	118.28	Military region	27	106	25.47	23	25
85	斌.汪斌.陈荣凯.	.Wang Bin.Che	超市熟食所致	caused by eati	中国公共卫生管	ublic health ma	34	1	52-54	2018	j.cnki.23-1318.2	2016	2	9	1	Eastern	Guangdong	Shenzhen	22.33	114.07	Residential area	9	11	81.82	6	38
87	欣.苏新霞.王亚	in.Su Xinxia.W	引起食物中毒的	e of food poisc	河南预防医学杂	nal of Preventi	29	3	227-228	2018	i.hnjpm.1006-84	2016	2	10	2	Central	Henan	Sanmenxia	34.47	111.12	Restaurant	56	126	44.44	2	75
89	王玉俊.	Wang Yujun.	引起食源性疾病	of foodborne il	中国卫生标准管	th Standard M	9	3	15-16	2018	issn.1674-9316.2	2017	1	6	1	Eastern	Shandong	Dezhou	37.26	116.17	School	46	119	38.66	17	18
90	春艳.祖文刚.左	an.Zu Wengar	成都柏林沙门氏	salmonella foo	医学动物防制	lical animal co	34	1	77-79	2018	529/yxdwfz2018	2016	2	9	4	Eastern	Hebei	Baoding	38.51	115.3	Restaurant	63	100	63.00	2	65
94	李明.杨志强.叶	ang Zhiqiang.	引起的食物中毒	e of food poisc	食品安全导刊	ood safety guic	-	27	84-85	2018	issn.1674-0270.2	2018	0	9	1	Eastern	Fujian	Quanzhou	24.56	118.36	Residential area	3	5	60.00	7	55
95	.赖天兵.蔡亚辉.	nbing.Cai Yah	门氏菌引起的食	ng incident ca	医学动物防制	lical animal co	34	9	879-882	2018	529/yxdwfz2018	2016	2	5	4	Central	Hunan	Changsha	28.12	112.59	Restaurant	67	79	84.81	6	82
96	会.徐闻.古文鹏.	Wen.Gu Wen	染聚餐食品所致	isoning Cause	中国食品卫生杂	Food Hygiene	30	6	659-662	2018	590/j.cjfh.2018.	2016	2	8	4	Western	Yunnan	Dali	25.34	100.13	Residential area	138	640	31.56	<1	84
98	袁飞.	Yuan Fei.	沙门菌引起的食	ing Caused by	国际检验医学杂	ournal of labor	38	15	2062-2064	2017	issn.1673-4130.2	2016	1	5	2	Western	Sichuan	Chengdu	30.4	104.04	School	69	69	100.00	21	47
100	彭立昌.马骏.	g Lichang.Ma	门菌引起的食物	of food poison	中国食品卫生杂	Food Hygiene	29	2	233-237	2017	590/j.cjfh.2017.	2015	2	3	3	Eastern	Tianjin	Dongli	39.02	117.12	School	81	168	48.21	N/A	N/A
101	陈慧中.常春祥.	zhong.Chang C	引起的食源性疾	rne illness out	基层医学论坛	ary Medicine F	21	17	2274-2276	2017	/j.1672-1721.20	2015	2	12	3	Northeastern	Liaoning	Shenyang	41.48	123.25	School	91	690	13.19	N/A	N/A
104	陈慧中.刘博.	n Huizhong.Li	发的食源性疾病	ne illness outb	预防医学论坛	ative Medicine	23	8	591-592,595	2017	-	2016	1	8	2	Northeastern	Liaoning	Shenyang	41.48	123.25	Residential area	35	121	28.93	N/A	N/A
105	洪佳冬.潘淑贤.	Jiadong.Pan S	引起的食源性疾	disease outb	华南预防医学	ina Preventive	43	6	556-559	2017	217/j.scjpm.201	2016	1	9	3	Eastern	Guangdong	Guangzhou	23.08	113.14	Restaurant	54	664	8.13	N/A	N/A
106	.祥初.贾明生.苏	hu.Jia Mingsh	制品引起沙门菌	od poisoning	中国公共卫生管	ublic health ma	33	3	431-432,437	2017	j.cnki.23-1318.2	2016	1	7	2	Western	Guangxi	Liuzhou	23.19	109.24	Residential area	59	88	67.04	2	87
107	.惠敏.杨玲.王艳	.Yang Ling.W	沙门氏菌食物中	ucky salmonell	医学动物防制	lical animal co	33	8	888-889	2017	529/yxdwfz2017	2016	1	4	1	Eastern	Hebei	Shijiazhuang	38.02	114.3	Office	5	22	22.73	30	60
111	李文常.	Li Wenchang.	肠炎沙门氏菌食	Enteritidis Fo	养生保健指南	and Wellness	-	38	85,201	2017	issn.1006-6845.2	2013	4	12	2	Western	Gansu	Baiyin	36.33	104.12	Restaurant	94	290	32.41	1	73
112	邱冠雄.邱秀青.温	nxiong.Qiu Xiu	门菌食物中毒事	ncident of Saln	海峡预防医学杂	nal of Preventi	23	5	90-92	2017	-	2017	0	2	3	Eastern	Fujian	Yongan	25.58	117.23	Residential area	48	540	8.89	1	65
115	胡鹏.唐开来.	Peng.Tang Ka	污染引起食物中	food poisonin	医药前沿	Medical Fronti	7	16	229-230	2017	issn.2095-1752.2	2015	2	5	2	Central	Hubei	Xiaogan	30.56	113.54	Residential area	16	130	12.31	7	60
117	王晓丽.	Wang Xiaoli.	性疾病暴发事件	on a foodbor	中国城乡企业卫	in urban and	32	7	51-52	2017	/j.1003-5052.20	2016	1	6	2	Eastern	Shandong	Rongcheng	37.1	122.25	Office	23	46	50.00	22	53
118	缪国忠.	Miao Guozhong	沙门菌食物中毒	ation and anal	疾病监测与控制	surveillance an	10	5	348-349	2016	-	2015	1	5	3	Eastern	Jiangsu	Jiangyin	31.54	120.17	Office	15	76	19.74	18	56
121	之.李艳红.姚云波	.Li Yanhong.Y	引起的食源性疾	f a foodborne	中国卫生检验杂	urnal of Health	26	7	1046-1047,1056	2016	-	2014	2	3	3	Western	Yunnan	Yuxi	24.22	102.32	mobile street stal	22	22	100.00	5	63
122	许建明.白贤.李旭	ming.Bai Yun.L	引起的沙门氏菌	outbreak cause	现代预防医学	n preventive m	43	13	2462-2465	2016	-	2015	1	9	2	Western	Yunnan	Yuxi	24.22	102.32	Residential area	80	735	10.88	6	79
123	傅荣生.陈建中.	sheng.Chen Ji	氏菌污染引起的	oisoning caus	今日健康	health today	15	6	5,9	2016	issn.1671-5160.2	2012	4	4	2	Western	Chongqing	Wanzhou	29.35	106.33	Residential area	45	310	14.52	2	71
124	芝.王增国.袁军.	ng Zengguo.Y	暑和同期散发腹	oisoning cause	中国病原生物学杂	urnal of Patho	11	12	1122-1125	2016	13350/j.cjpb.161	2015	1	5	6	Western	Shaanxi	Xian	34.17	108.57	School	15	62	24.19	7	11
126	树博.王力.马源.	.Wang Li.Ma Y	自办宴席引起的	caused by a	医学动物防制	lical animal co	32	7	799-800	2016	529/yxdwfz2016	2014	2	8	3	Western	Gansu	Baiyin	36.33	104.12	Residential area	55	200	27.50	2	70
132	净.林建维.靳桂	.Lin Jianwei.Jin	沙门菌食物中毒	Salmonella foo	疾病监测与控制	surveillance an	10	8	650-651	2016	-	2015	1	5	3	Western	Inner mongolia	Chifeng	42.17	118.58	Residential area	25	40	62.50	11	74
138	余.陈曦.袁伟.张	Chen Xi.Yuan V	婚宴引起的沙门	lla outbreak c	预防医学情报杂	ventive Medici	32	2	145-149	2016	-	2013	3	5	3	Western	Sichuan	Zigong	29.23	104.46	Restaurant	44	50	88.00	N/A	N/A
139	前进.林英华.廖	jin.Lin Yinghu	致食物中毒的病	two cases of f	中国食品卫生杂	Food Hygiene	28	1	32-36	2016	590/j.cjfh.2016.	2014	2	10	2	Eastern	Fujian	Longyan	25.06	117.01	Restaurant	25	570	4.39	5	71
140	付秀影.黄露.	Xiuying.yellow	门氏菌食物中毒	of food poisor	首都公共卫生	pital public hea	10	2	86-89	2016	-	2015	1	5	3	Eastern	Beijing	Dongcheng	39.55	116.24	Office	66	1252	5.27	25	59
141	许风兰.梁宏章.	n.Liang Hong	沙门氏菌食物中	od poisoning	医学动物防制	lical animal co	32	-	-	2016	529/yxdwfz2016	2015	1	2	3	Western	Guangxi	Jingxi	23.15	106.31	ublic sports venu	39	60	65.00	8	58
142	华.夏伟.卓菲.杨	Wei.Zhuo Fei.	引起的食物中	od poisoning	当代医学	emporary med	22	-	-	2016	issn.1009-4393.2	2015	1	10	1	Eastern	Guangdong	Shenzhen	22.33	114.07	Residential area	6	6	100.00	21	48
143	洪乐.杨荣兴.张	Rongxing.Zha	次两批聚餐人群	groups of pe	中国热带医学	se tropical me	16	-	-	2016	j.cnki.46-1064/r.	2014	2	9	2	Eastern	Guangdong	Shenzhen	22.33	114.07	Restaurant	96	642	14.95	N/A	N/A
145	孟克.	Monk.	毒事件进行致病	pathogenic ba	信息文摘（连续	mation abstrac	15	A2	222	2015	ssn.1671-3141.2	2011	4	7	1	Western	Inner mongolia	Ordos	39.43	108.63	Residential area	7	7	100.00	7	72
152	君.何雪连.蒋双.	elian.Jiang Sh	毒疫情的实验室	analysis of a p	饮食保健	ct and health c	2	16	5-6	2015	-	2015	0	7	1	Western	Sichuan	Luzhou	28.54	105.24	Residential area	2	2	100.00	N/A	N/A
155	吴高芳.孙敏红.	aofang.Sun Mi	氏菌引起的学校	rne illness outl	医学与社会	dicine and soc	28	0	42-43	2015	-	2014	1	6	3	Central	Hubei	Enshi	30.16	109.29	School	77	2420	3.18	N/A	N/A
156	秦友燕.蒋铁翼.	Youyan.Jiang	酒店连续4起食物	ntinued4food	华夏医学	hinese Medicin	28	5	127-128	2015	-	2015	0	1	4	Western	Guangxi	Guilin	25.17	110.17	Restaurant	94	600	15.67	N/A	N/A
158	晨杨.蒋庆.赵球	iang Qing.Zha	省某高校一起伤	fever outbreak	疾病监测	sease surveillan	30	11	979-981	2015	issn.1003-9961.2	2013	2	12	36	Central	Hubei	Xiantao	30.22	113.27	School	74	7069	1.05	15	28
159	温晋华.马玉春.	Jinhua.Ma Yu	伤寒沙门氏菌食	urium food po	河北医学	Hebei Medicin	-	12	2120-2122	2015	issn.1006-6233.2	2015	0	5	3	Eastern	Hebei	Chengde	40.59	117.57	Office	66	1252	5.27	25	59
161	李凤元.	Li Fengyuan.	引起沙门菌食物	nella Food Poi	现代医药卫生	medicine and	-	z1	111-113	2015	issn.1009-5519.2	2013	2	8	2	Western	Guangxi	Chongzuo	22.51	107.27	Residential area	32	50	64.00	N/A	N/A
163	.强.田维丽.谷永	.Tian Weili.Gu	起沙门氏菌食物	almonella food	微量元素与健康研	nents and heal	32	6	41	2015	-	2015	0	4	2	Western	Guizhou	Qiannan	26.02	107.25	Residential area	27	300	9.00	3	60
164	罗冬香.林小红.	ngxiang.Lin Xi	沙门菌引起的食	od poisoning c	海峡预防医学杂	nal of Preventi	21	6	54-55	2015	-	2014	1	9	2	Eastern	Fujian	Longyan	25.06	117.01	Residential area	25	200	12.50	44	66
166	汪伟.	Wang Wei.	沙门氏菌引起	sed by Salmo	中国现代药物应	rug applicatic	-	10	236-238	2014	-	2012	2	5	4	Central	Henan	Xinyang	32.07	114.04	Restaurant	79	200	39.50	10	60
170	晓健.谢明军.王	.Xie Mingjun.W	沙门氏菌食物中	nella Enteritid	中国公共卫生管	ublic health ma	-	1	63-64	2014	-	2013	1	3	3	Central	Henan	Pingdingshan	33.44	113.17	Restaurant	67	225	29.78	2	80
172	史志瑛.	Shi Zhiying.	菌引起的食物中	on food pois	科技展望	chnology Outl	-	18	210-210	2014	issn.1672-8289.2	2013	1	8	1	Central	Shanxi	Datong	40.06	113.17	Restaurant	19	46	41.30	N/A	N/A
173	徐基明.查正时.	ming.Check ti	引发食物中毒	food poisoning	热带病与寄生虫	Diseases and Pa	-	4	252-253	2014	issn.1672-2302.2	2012	2	8	3	Central	Anhui	Huangshan	29.43	118.18	Residential area	30	72	41.67	7	75
174	东.权怡.班秀花.	an Yi.Ban Xiuh	寒疫情暴发的流	n of a typhoid	应用预防医学	preventive m	20	2	87-89	2014	-	2013	1	7	42	Western	Guangxi	Hechi	24.42	108.03	School	64	1576	4.06	6	19
179	吉喜军.	Ji Xijun.	沙门氏菌食物中	of salmonella	医学信息文摘	information a	-	6	182-182	2014	issn.1671-3141.2	2012	2	6	3	Western	Inner mongolia	Wulanchabu	41.7	112.44	Construction site	17	17	100.00	19	52
180	王艳.张江萍.	Yan.Zhang Jiar	的肠炎沙门氏菌	tidis food pois	贵州医药	uizhou Medici	-	2	164-165	2014	SSN.1000-744X.	2011	3	5	2	Western	Guizhou	Guiyang	26.35	106.42	Restaurant	29	220	13.18	7	67
181	陈国庆.	Chen Guoqing	引起沙门氏菌食	nella food pois	保健营养（上旬	alth Nutrition	24	2	1088-1089	2014	sn.1004-7484(s).	2012	2	6	1	Northeastern	Jilin	Changchun	43.54	125.19	Construction site	4	23	17.39	20	50
183	张世勇.刘立.庞	aiyong.Liu Li.P	菌食物中毒事件	of a salmonell	医学动物防制	lical animal co	30	10	1146-1147	2014	529/yxdwfz2014	2013	1	6	3	Eastern	Hebei	Shijiazhuang	38.02	114.3	Construction site	18	21	85.71	16	60
186	罗华.	Luo Hua.	门氏菌污染皮蛋	served eggs c	保健营养（中旬	h nutrition (m	24	4	2502-2503	2014	-	2009	5	6	1	Western	Chongqing	Wanzhou	29.35	106.33	Residential area	8	9	88.89	10	62
189	孙磊.	Sun Lei.	沙门氏菌食物中	of Salmonella	中国实用医药	se Practical Me	-	23	273-274	2014	-	2012	2	5	3	Central	Henan	Luoyang	34.41	112.27	Restaurant	55	234	23.50	24	69
190	陆月敏.	Lu Yuemin.	中毒的流行病学	y investigatio	中国卫生检验杂	urnal of Health	24	12	1788-1789	2014	-	2013	1	5	2	Eastern	Zhejiang	Yongkang	29.54	120.01	Office	19	19	100.00	18	56
191	黄滋.黄柏林.陆少	Huang Bolin.L	氏菌食物中毒事	lmonella food	内科	nternal Medici	9	5	592-594	2014	-	2011	3	7	1	Western	Guangxi	Baise	23.54	106.36	Residential area	12	23	52.17	6	70
192	王福春.	Wang Fuchun	聚餐食物中毒的	sis of Food Po	医学动物防制	lical animal co	30	10	1148-1149	2014	529/yxdwfz2014	2013	1	8	2	Western	Guangxi	Jingxi	23.15	106.31	Residential area	24	70	34.29	4	80
193	凯金祥.陈宇.王刚	g.Chen Yu.Wa	引起的混合细菌	od poisoning c	疾病预防控制通	vention and Co	29	5	63-64	2014	5/j.cnki.jbyfkztb.	2014	0	1	3	Western	Guizhou	Qianxinan	25.27	105.46	Residential area	38	408	9.31	1	76
194	建华.杨祖顺.韦	hua.Yang Zus	门菌引起的食物	od poisoning	疾病监测	sease surveillan	29	11	920-924	2014	issn.1003-9961.2	2012	2	10	10	Western	Yunnan	Xishuangbanna	22.11	100.89	School	151	3012	5.01	12	18
195	丹.曹国科.侯水	an.Cao Guoke	食源性疾病暴发	orne disease o	华南预防医学	ina Preventive	40	3	270-273	2014	217/j.scjpm.201	2013	1	6	2	Eastern	Guangdong	Guangzhou	23.08	113.14	Office	56	140	40.00	20	45
197	张振春.安秉站.	enchun.Anbin	起的食物中毒调	is of a case of	中外健康文摘	nd Foreign He	-	13	74-74,75	2014	-	2013	1	7	3	Northeastern	Liaoning	Shenyang	41.48	123.25	Restaurant	59	150	39.33	N/A	N/A
200	王东.刘思渊.黄	ng.Liu Siyuan.	门菌引起的食物	od poisoning	华南预防医学	ina Preventive	40	3	274-275	2014	217/j.scjpm.201	2013	1	6	2	Eastern	Guangdong	Dongguan	23.02	113.45	Office	36	1100	3.27	22	38
201	昌.王伟望.张峰.	Weiwang.Zhan	伤寒沙门菌食物	Salmonella Ty	上海预防医学	ai Preventive M	26	10	537-538	2014	-	2013	1	12	3	Eastern	Zhejiang	Ningbo	29.52	121.33	Residential area	41	68	60.29	18	87
202	静.王路梅.童	Wang Lumei.T	致食源性疾病暴	ne disease out	中华预防医学杂	rnal of Prevent	47	2	160-163	2013	.j.issn.0253-9624	2010	3	5	3	Eastern	Jiangsu	Xuzhou	34.15	117.11	School	135	754	17.90	N/A	N/A
203	王琴.	Wang Qin.	引起的食源性疾	of a foodborn	疾病预防控制通	vention and Co	-	3	65-66	2013	-	2012	1	6	5	Western	Gansu	Lanzhou	36.04	103.51	Residential area	50	226	22.12	11	70
204	罗雪.刘天慧.康	.Liu Tianhui.K	门氏菌引起的食	caused by Salr	医学信息文摘	information a	-	20	344-344	2013	issn.1671-3141.2	2011	2	8	1	Western	Sichuan	Panzhihua	26.34	101.43	Restaurant	5	9	55.56	30	42
206	秦田秀.	Qin Tianxiu.	引起的沙门氏菌	a food poison	广西医学	uangxi Medici	-	7	929-930	2013	j.issn.0253-4304.	2009	4	8	6	Western	Guangxi	Guilin	25.17	110.17	Residential area	107	350	30.57	1	75
209	杨凤娟.	Yang Fengjuan	引起的沙门氏菌	food poisonir	中外健康文摘	nd Foreign He	-	49	44-45	2013	-	2012	1	11	3	Western	Yunnan	Dali	25.34	100.13	Residential area	407	1440	28.26	2	75
211	梁艳.	Liang Yan.	菌引起的食物中	se of food pois	中外健康文摘	nd Foreign He	-	33	226-227	2013	-	2011	2	10	1	Western	Yunnan	Baoshan	25.08	99.1	Residential area	74	194	38.14	7	90
212	吴俊霞.	Wu Junxia.	门菌引起的食物	of food poison	江苏卫生保健	ngsu Health C	15	5	29	2013	issn.1008-7338.2	2012	1	6	2	Eastern	Jiangsu	Zhenjiang	32.11	119.27	Residential area	22	160	13.75	27	80
215	王慧华.王立华.	Huihua.Wang	家宴引起食物中	ng caused by	蛇志	snake ambitio	25	3	287-289	2013	issn.1001-5639.2	2010	3	8	2	Eastern	Jiangsu	Changzhou	31.47	119.58	Residential area	36	250	14.40	10	63
216	张金华.王桂红.	Jinhua.Wang C	门氏菌引起食物	of food poisc	大学学报（医学	east University	32	1	29-31	2013	issn.1671-6264.2	2011	2	8	1	Eastern	Hebei	Chengde	40.59	117.57	School	63	276	22.83	N/A	N/A
217	红.严军.陈秀兰.	.Yan Jun.Chen	沙门菌引起食物	d poisoning ca	江苏卫生保健	ngsu Health C	15	6	25-26	2013	issn.1008-7338.2	2013	0	4	4	Eastern	Jiangsu	Huaian	33.3	119.09	Residential area	20	90	22.22	20	60
222	杜航.	Du Hang.	门菌引起的食物	of food poison	社区医学杂志	of Community	11	22	73-74	2013	-	2013	0	7	2	Central	Hubei	Xiangyang	31.93	111.94	Office	19	107	17.76	19	53
223	李建庆.	Li Jianqing.	食物中毒的调查	ort on a case	健康大视野	Healthy vision	21	18	690-690	2013	-	2012	1	7	2	Eastern	Zhejiang	Shaoxing	30	120.34	Office	15	30	50.00	25	49
225	陈玉红.	Chen Yuhong	引起食物中毒的	ase of food po	检验医学与临床	ry medicine ar	10	2	206-207	2013	issn.1672-9455.2	2011	2	12	1	Eastern	Jiangsu	Rugao	32.23	120.33	Residential area	6	6	100.00	8	52
228	.谢青梅.李宗谨.	e Qingmei.Li Z	性腹泻的流行病	d analysis of a	健康必读（下旬刊	ad for health (l	-	1	355-355	2013	-	2012	1	4	4	Central	Henan	Pingdingshan	33.44	113.17	Hospital	4	12	33.33	<1	<1
229	王晓东.	Vang Xiaodon	起疑似食物中毒	ted food poisc	健康大视野	Healthy vision	21	5	714	2013	-	2012	1	6	2	Central	Hubei	Huanggang	30.72	115.34	Residential area	45	220	20.45	7	76
234	甘武丰.刘会芸.	Wufeng.Liu Hu	光村一起食物中	ood poisoning	健康大视野	Healthy vision	20	7	274-274	2012	-	2011	1	7	2	Central	Hubei	Enshi	30.16	109.29	Residential area	8	21	38.10	12	72
237	翻泽君.吴娴.朱	jun.Wu Xian.Z	菌致1起食物中毒	ood poisoning	预防医学情报杂	ventive Medici	28	4	298-300	2012	-	2011	1	7	3	Western	Sichuan	Yibin	28.47	104.34	Residential area	61	380	16.05	5	85
238	李蓓.魏彩梅.	Bei.Wei Caim	门氏菌食物中毒	s of a case of s	疾病监测与控制	surveillance an	6	7	414-415	2012	-	2011	1	7	3	Western	Shaanxi	Shangluo	33.65	109.9	Residential area	21	100	21.00	14	59
239	朱艳梅.汤艳.	Yanmei.Tang	氏菌引起的食物	case of food p	中外健康文摘	nd Foreign He	-	34	80-81	2012	issn.1672-5085.2	2011	1	7	2	Central	Hubei	Xiaogan	30.56	113.54	Residential area	16	290	5.52	12	62
240	.龚文胜.张金枝.	Wensheng.Zha	引起食物中毒	incidents ca	中国初级卫生保	y health care i	26	4	63-64	2012	ssn.1001-568X.2	2011	1	11	3	Central	Hubei	Xiangyang	31.93	111.94	Residential area	96	138	69.57	1	76
243	丽芳.王雪梅.许	g.Wang Xuem	门菌引起食物中	of food poiso	海峡预防医学杂	nal of Preventi	18	1	55-56	2012	-	2010	2	8	1	Eastern	Guangdong	Kaiping	22.22	112.4	Restaurant	13	15	86.67	15	55
245	冯裕如.	Feng Yuru.	门氏菌引起食物	d poisoning c	健康必读（中旬刊	for health (mic	11	10	75-75	2012	-	2011	1	7	3	Western	Sichuan	Suining	30.31	105.33	Residential area	42	87	48.28	3	71
248	.吕文凤.郑永惠.	Wenfeng.Zhen	氏菌引起食物中	od poisoning c	中国城乡企业卫	in urban and	27	2	103-104	2012	-	2010	2	8	4	Eastern	Tianjin	Xiqing	39.02	117.12	Factory	95	1700	5.59	18	59
252	燕.黄健利.钟凌	g Jianli.Zhong	鼠伤寒沙门菌引起	d poisoning ca	海峡预防医学杂	nal of Preventi	18	1	58-59	2012	-	2010	2	6	2	Eastern	Fujian	Zhangzhou	24.31	117.39	Residential area	15	22	68.18	3	82
257	甚志飞.李广兵.	ifei.Li Guangbi	物中毒的病原学	ion of the etiol	实用预防医学	al preventive m	19	11	1721-1723	2012	issn.1006-3110.2	2011	1	8	3	Central	Hunan	Shaoyang	27.14	111.28	Residential area	93	450	20.67	4	82
259	.贺天锋.张胜良.	eng.Zhang Sh	引起的中学生食	a middle schoo	中国学校卫生	nese school he	33	6	755-756,759	2012	-	2011	1	12	3	Eastern	Zhejiang	Ningbo	29.52	121.33	School	105	1211	9.37	12	16
260	杜昌海.杨万群.	anghai.Yang W	氏菌引起的食物	se of food pois	医学（医学地理	ine (Medical G	33	3	189-190	2012	issn.1001-8883.2	2011	1	2	3	Western	Yunnan	Chuxiong	25.01	101.32	Residential area	78	480	16.25	6	80
263	孙步武.	Sun Buwu.	菌Ⅱ引起的食物	oisoning incid	应用预防医学	preventive m	17	1	64	2011	ssn.1673-758X.2	2010	1	11	7	Western	Guangxi	Baise	23.54	106.36	Residential area	161	550	29.27	2	83
266	朱翔.包云娟.	Xiang.Bao Yur	寒沙门菌引起的	d poisoning ca	浙江预防医学	g Preventive M	23	12	49-50	2011	issn.1007-0931.2	2010	1	3	3	Eastern	Zhejiang	Jinhua	29.07	119.39	Residential area	16	76	21.05	8	71
274	巧荣.李秋梅.张	g.Li Qiumei.Zh	门氏菌食物中毒	case of salmo	中国公共卫生管	ublic health ma	27	3	309	2011	-	2008	3	7	1	Western	Inner mongolia	Baotou	40.39	109.49	Restaurant	23	120	19.17	8	51
279	.黄秋洁.莫国华.	ng Qiujie.Mo	降沙门菌引起	d poisoning ca	浙江预防医学	g Preventive M	23	8	44-45	2011	issn.1007-0931.2	2008	3	7	2	Eastern	Zhejiang	Hangzhou	30.16	120.1	Restaurant	27	49	55.10	2	63

Patient information (continued) — Incidence rates (IR) of designated symptoms / %; Laboratory test — Pathogen (*Salmonella*), Identification Method (Y: yes); Clinical management — Treatment, Prognosis; Event cluster

	Mean Age / y	Mean Age_SD /	Male	Female	nder Ratio (GR)	Diarrhea	Fever	Stomach cramp	Nausea	Vomiting	Headache	Source (food)	Serogroup	Serovar	Sequence type	crobial resistan	lective culture (	mical identificat	nunological test	l gel electrophor	ase chain reacti	copic examinati	Other	Medication	Recovery	Death	Cluster type
2	22	N/A	17	9	65.38	76.92	61.54	46.67	100.00	100.00	100.00	Meat products	D_1	Enteritidis	N/A	N/A	Y	Y	Y	N/A	N/A	N/A	N/A	N/A	N/A	N/A	Food poisoning
3	N/A	N/A	9	9	50.00	100.00	38.89	100.00	11.11	5.56	16.67	Meat products	C1	Oranienburg	N/A	N/A	Y	N/A	Y	Y	N/A	N/A	N/A	N/A	N/A	N/A	Food poisoning
6	N/A	N/A	22	25	46.81	100.00	93.62	72.34	53.19	25.53	8.51	Pastries	D1	Enteritidis	N/A	K, GEN, ATM, T	Y	Y	Y	Y	N/A	N/A	N/A	N/A	N/A	N/A	Food poisoning
7	43	N/A	17	6	73.91	86.96	91.30	8.70	N/A	100.00	N/A	N/A	D1	Enteritidis	N/A	N/A	Y	Y	Y	Y	N/A	N/A	N/A	LVX, CRO	23	0	ociated infection
9	25	N/A	1	10	9.09	100.00	100.00	100.00	90.91	63.64	81.82	cessed grain proc	D1	Enteritidis	N/A	N/A	Y	N/A	Y	N/A	N/A	N/A	N/A	N/A	N/A	N/A	Food poisoning
10	N/A	N/A	41	27	60.29	85.29	100.00	95.59	100.00	44.12	100.00	Aquatic products	D1	Enteritidis	N/A	N/A	Y	N/A	Y	N/A	Y	N/A	N/A	N/A	N/A	N/A	Food poisoning
12	12	N/A	21	30	41.18	100.00	41.18	78.43	35.29	23.53	58.82	Pastries	C1	andaka、Thomp	N/A	N/A	Y	N/A	Y	Y	N/A	N/A	N/A	N/A	N/A	N/A	Food poisoning
13	45.12	4.66	9	6	60.00	86.67	100.00	93.33	80.00	66.67	40.00	Meat products	D1	Enteritidis	N/A	N/A	Y	Y	Y	N/A	N/A	N/A	N/A	N/A	15	0	Food poisoning
14	N/A	N/A	45	26	63.38	63.38	45.07	91.55	38.03	30.99	N/A	Pastries	D1	Enteritidis	N/A	N/A	Y	Y	Y	Y	N/A	N/A	N/A	N/A	N/A	N/A	Food poisoning
15	47	N/A	1	6	14.29	100.00	57.14	70.43	N/A	28.57	N/A	Egg products	B	Typhimurium	N/A	N/A	Y	Y	Y	N/A	N/A	N/A	N/A	N/A	N/A	N/A	Food poisoning
16	N/A	N/A	14	14	50.00	82.14	42.85	64.29	46.43	57.14	N/A	Meat products	E1	London	N/A	N/A	Y	N/A	Y	N/A	N/A	N/A	N/A	N/A	28	0	Food poisoning
18	N/A	N/A	28	34	45.16	77.42	70.97	53.23	48.39	N/A	56.45	Others	D1	Enteritidis	N/A	AMP, NAL	Y	Y	Y	Y	Y	N/A	MALDI-TOF-M	N/A	N/A	N/A	Food poisoning
21	N/A	N/A	41	33	55.41	83.78	63.51	78.38	43.24	22.97	31.08	Egg products	D1	Enteritidis	N/A	N/A	Y	Y	Y	Y	Y	N/A	N/A	N/A	74	0	Food poisoning
25	N/A	N/A	39	16	70.91	61.82	94.55	50.91	60.00	47.27	N/A	Aquatic products	E1	Kalina	N/A	N/A	Y	N/A	Y	N/A	N/A	N/A	N/A	N/A	55	0	Food poisoning
29	26	N/A	183	112	62.03	100.00	99.32	89.15	55.93	64.41	88.81	Meat products	D1	Enteritidis	N/A	N/A	Y	Y	Y	Y	N/A	N/A	N/A	N/A	N/A	N/A	Food poisoning
31	N/A	N/A	16	11	59.26	100.00	70.37	77.78	29.63	33.33	37.04	Pastries	D1	Enteritidis	N/A	N/A	Y	Y	Y	N/A	Y	N/A	N/A	N/A	N/A	N/A	Food poisoning
33	40.7	N/A	14	23	37.84	100.00	100.00	100.00	100.00	100.00	N/A	Meat products	D1	Enteritidis	N/A	N/A	Y	Y	Y	N/A	N/A	N/A	N/A	N/A	37	0	Food poisoning
34	N/A	N/A	5	1	83.33	100.00	100.00	100.00	N/A	N/A	N/A	Meat products	C1	Isangi	216	SXT	Y	Y	Y	Y	N/A	N/A	WGS	NOR, CTF	6	0	Food poisoning
37	N/A	N/A	25	42	37.31	91.04	74.63	73.13	50.75	62.69	38.81	Pastries	B	Typhimurium	N/A	N/A	Y	Y	Y	Y	N/A	N/A	N/A	N/A	67	0	Food poisoning
38	48.33	23.8	2	1	66.67	100.00	66.67	66.67	N/A	66.67	100.00	Egg products	B	Typhimurium	N/A	N/A	Y	N/A	Y	N/A	Y	N/A	N/A	N/A	3	0	Food poisoning
41	N/A	N/A	0	13	0.00	100.00	76.92	84.62	53.85	46.15	69.23	Pastries	D1	Enteritidis	N/A	N/A	Y	Y	Y	N/A	N/A	N/A	N/A	N/A	13	0	Food poisoning
42	N/A	N/A	11	13	45.83	100.00	79.17	100.00	100.00	100.00	100.00	Meat products	D1	Enteritidis	N/A	N/A	Y	Y	Y	N/A	N/A	N/A	N/A	N/A	24	0	Food poisoning
45	N/A	N/A	11	6	64.71	100.00	94.12	76.47	N/A	38.46	N/A	Meat products	E1	Weltevreden	N/A	DX, MNO, DOX	Y	Y	Y	Y	N/A	N/A	N/A	N/A	17	0	Food poisoning
46	46	N/A	40	42	48.78	96.34	59.76	79.27	71.95	57.32	46.34	Others	D1	Enteritidis	N/A	N/A	Y	Y	Y	N/A	Y	N/A	N/A	N/A	82	0	Food poisoning
49	N/A	N/A	5	9	35.71	71.43	71.43	85.71	42.86	64.29	50.00	Meat products	D1	Enteritidis	N/A	N/A	Y	Y	Y	N/A	N/A	N/A	N/A	N/A	N/A	N/A	Food poisoning
50	27	N/A	3	5	37.50	100.00	100.00	100.00	N/A	37.50	N/A	Pastries	D1	Enteritidis	N/A	NAL, CIP, AMK	Y	Y	Y	Y	Y	Y	N/A	N/A	8	0	Food poisoning
51	N/A	N/A	18	29	38.30	100.00	76.60	N/A	82.98	N/A	82.98	N/A	D1	Enteritidis	N/A	N/A	Y	Y	Y	N/A	N/A	N/A	N/A	N/A	N/A	N/A	Food poisoning
52	N/A	N/A	6	4	60.00	80.00	100.00	70.00	90.00	10.00	50.00	N/A	D1	Enteritidis	N/A	NAL, CIP	Y	Y	Y	Y	N/A	N/A	N/A	N/A	N/A	N/A	Food poisoning
57	54	23.85	1	2	33.33	100.00	100.00	N/A	N/A	N/A	N/A	Meat products	D1	Enteritidis	N/A	ZO, NAL, SOX,	Y	Y	Y	Y	N/A	N/A	N/A	N/A	N/A	N/A	Food poisoning
59	N/A	N/A	55	12	82.09	83.58	79.10	76.12	25.37	10.45	71.64	Meat products	D1	Enteritidis	N/A	AMP, CHL, NAL	Y	Y	Y	Y	N/A	N/A	N/A	N/A	67	0	Food poisoning
60	N/A	N/A	55	35	61.11	98.89	100.00	95.56	N/A	37.78	N/A	Others	D1	Enteritidis	N/A	N/A	Y	N/A	Y	N/A	N/A	N/A	N/A	N/A	90	0	Food poisoning
62	N/A	N/A	42	50	45.65	28.26	100.00	13.04	17.39	13.04	66.30	N/A	D1	Typhi	N/A	NAL, RIF	Y	Y	Y	Y	Y	N/A	N/A	N/A	N/A	N/A	Food poisoning
64	35	N/A	45	19	70.31	100.00	100.00	100.00	50.00	35.94	N/A	Meat products	D1	Enteritidis	N/A	N/A	Y	N/A	Y	N/A	N/A	N/A	N/A	N/A	N/A	N/A	Food poisoning
66	N/A	N/A	13	15	46.43	25.00	95.00	20.00	35.00	10.00	80.00	Vegetable produc	A	Paratyphi A	N/A	N/A	Y	N/A	Y	Y	N/A	N/A	N/A	N/A	28	0	Food poisoning
67	N/A	N/A	8	10	44.44	100.00	100.00	100.00	100.00	100.00	N/A	Others	D1	Enteritidis	N/A	N/A	Y	Y	Y	Y	N/A	N/A	N/A	N/A	N/A	N/A	Food poisoning
68	N/A	N/A	4	4	50.00	87.50	87.50	37.50	12.50	12.50	N/A	Meat products	D1	Enteritidis	N/A	N/A	Y	N/A	Y	N/A	N/A	N/A	N/A	N/A	N/A	N/A	Food poisoning
71	N/A	N/A	13	12	52.00	80.00	88.00	88.00	84.00	56.00	40.00	Egg products	D1	Dublin	N/A	N/A	Y	N/A	Y	N/A	N/A	N/A	N/A	N/A	N/A	N/A	Food poisoning
73	N/A	N/A	84	71	54.19	98.84	94.19	69.03	58.71	58.06	94.19	Meat products	B、D1	ritidis、Typhimu	N/A	N/A	Y	Y	Y	Y	N/A	N/A	N/A	N/A	N/A	N/A	Food poisoning
74	32	N/A	78	45	63.41	N/A	100.00	N/A	N/A	N/A	100.00	Vegetable produc	D1	Typhi	N/A	CIP, SOX, SM	Y	Y	Y	Y	N/A	N/A	N/A	N/A	123	0	Food poisoning
76	36	N/A	21	52	28.77	84.93	95.89	N/A	46.58	27.40	N/A	Pastries	D1	Enteritidis	N/A	N/A	Y	Y	Y	N/A	Y	N/A	N/A	N/A	N/A	N/A	Food poisoning
77	N/A	N/A	4	4	50.00	100.00	87.50	100.00	100.00	75.00	N/A	Egg products	D1	Enteritidis	N/A	N/A	Y	Y	Y	N/A	N/A	N/A	N/A	CFP, AMK, CT	8	0	Food poisoning
78	39	N/A	43	56	43.43	100.00	90.91	94.95	75.76	71.72	N/A	Meat products	D1	Enteritidis	N/A	N/A	Y	Y	Y	N/A	N/A	N/A	N/A	N/A	N/A	N/A	Food poisoning
79	N/A	N/A	18	18	50.00	91.67	94.44	97.22	N/A	72.22	36.11	Pastries	D1	Enteritidis	N/A	N/A	Y	Y	Y	N/A	N/A	N/A	N/A	N/A	36	0	Food poisoning
80	N/A	N/A	6	12	33.33	100.00	94.44	92.20	61.11	38.89	27.78	Meat products	B	Typhimurium	N/A	N/A	Y	Y	Y	N/A	N/A	N/A	N/A	N/A	18	0	Food poisoning
81	N/A	N/A	10	8	52.63	100.00	100.00	100.00	63.16	84.21	N/A	Meat products	B	Typhimurium	N/A	N/A	Y	Y	Y	N/A	Y	N/A	N/A	N/A	19	0	Food poisoning
82	N/A	N/A	30	20	60.00	94.00	22.00	96.00	80.00	62.00	20.00	Meat products	B	Typhimurium	N/A	N/A	Y	Y	Y	N/A	N/A	N/A	N/A	N/A	50	0	Food poisoning
83	43	N/A	97	67	59.15	99.39	7.93	99.39	N/A	15.85	N/A	Vegetable produc	B	Paratyphi B	N/A	N/A	Y	Y	Y	N/A	N/A	N/A	N/A	N/A	164	0	Food poisoning
84	N/A	N/A	27	0	100.00	100.00	100.00	100.00	N/A	100.00	N/A	Meat products	N/A	N/A	N/A	N/A	Y	N/A	N/A	N/A	N/A	N/A	N/A	N/A	27	0	Food poisoning
85	20.6	N/A	5	4	55.56	100.00	100.00	100.00	100.00	100.00	100.00	Egg products	D1	Enteritidis	N/A	N/A	Y	Y	Y	N/A	N/A	N/A	N/A	N/A	N/A	N/A	Food poisoning
87	N/A	N/A	13	16	23.21	100.00	100.00	100.00	100.00	100.00	N/A	N/A	C1	Choleraesuis	N/A	N/A	Y	Y	Y	N/A	N/A	N/A	N/A	N/A	N/A	N/A	Food poisoning
89	N/A	N/A	24	22	52.17	93.48	91.30	93.48	100.00	N/A	N/A	Pastries	D1	Enteritidis	N/A	N/A	Y	Y	Y	N/A	N/A	N/A	N/A	N/A	46	0	Food poisoning
90	N/A	N/A	32	31	50.79	100.00	100.00	100.00	69.84	58.73	77.78	Seasonings	D1	Dublin	N/A	N/A	Y	Y	Y	N/A	N/A	N/A	N/A	N/A	63	0	Food poisoning
94	31	N/A	2	1	66.67	100.00	100.00	100.00	100.00	100.00	N/A	Vegetable produc	N/A	N/A	N/A	N/A	Y	Y	N/A	Y	Y	Y	N/A	N/A	N/A	N/A	Food poisoning
95	N/A	N/A	37	30	55.22	79.10	16.43	89.55	41.79	10.45	64.18	Meat products	B	Typhimurium	N/A	N/A	Y	N/A	Y	Y	N/A	N/A	N/A	N/A	N/A	N/A	Food poisoning
96	N/A	N/A	62	76	44.93	99.28	43.48	92.75	21.01	15.94	39.13	Meat products	B	N/A	N/A	N/A	Y	Y	Y	Y	N/A	N/A	N/A	N/A	138	0	Food poisoning
98	N/A	N/A	54	15	78.26	100.00	100.00	100.00	N/A	N/A	N/A	N/A	E1	Weltevreden	N/A	N/A	Y	Y	Y	N/A	Y	N/A	N/A	N/A	69	0	Food poisoning
100	N/A	N/A	46	35	56.79	98.77	100.00	97.53	N/A	69.14	N/A	Egg products	D1	Enteritidis	N/A	NAL	Y	Y	Y	Y	N/A	N/A	N/A	N/A	N/A	N/A	Food poisoning
101	N/A	N/A	62	29	68.13	94.51	100.00	85.71	69.23	98.90	78.02	Egg products	N/A	N/A	N/A	N/A	Y	Y	Y	N/A	N/A	N/A	N/A	AZI	N/A	N/A	Food poisoning
104	N/A	N/A	19	16	54.29	88.57	97.14	100.00	85.71	82.86	N/A	Meat products	N/A	N/A	N/A	N/A	Y	Y	Y	N/A	N/A	N/A	N/A	N/A	35	0	Food poisoning
105	N/A	N/A	18	36	33.33	98.15	79.63	98.15	38.89	37.04	61.11	Egg products	D1	Enteritidis	N/A	N/A	Y	Y	Y	N/A	Y	N/A	N/A	N/A	54	0	Food poisoning
106	N/A	N/A	29	26	49.15	81.36	30.51	77.97	57.63	47.46	N/A	Meat products	B	N/A	N/A	N/A	Y	Y	Y	N/A	N/A	N/A	N/A	N/A	N/A	N/A	Food poisoning
107	N/A	N/A	5	0	100.00	100.00	100.00	100.00	100.00	100.00	100.00	on-frozen bevera	C3	Kentucky	N/A	N/A	Y	Y	Y	N/A	N/A	N/A	N/A	N/A	5	0	Food poisoning
111	N/A	N/A	39	55	41.49	98.84	100.00	98.84	100.00	100.00	31.91	Meat products	D1	Enteritidis	N/A	TET, NAL	Y	Y	Y	Y	N/A	N/A	N/A	N/A	94	0	Food poisoning
112	N/A	N/A	25	23	52.08	100.00	77.08	100.00	41.67	22.92	54.17	Meat products	D1	Enteritidis	N/A	N/A	Y	Y	Y	N/A	N/A	N/A	N/A	N/A	48	0	Food poisoning
115	N/A	N/A	9	7	56.25	100.00	12.50	100.00	81.25	12.50	100.00	N/A	B	Typhimurium	N/A	N/A	Y	Y	Y	N/A	N/A	Y	N/A	N/A	16	0	Food poisoning
117	32.7	N/A	9	14	39.13	95.65	78.26	86.96	82.61	82.61	N/A	Legume products	D1	Enteritidis	N/A	N/A	Y	Y	Y	N/A	N/A	N/A	N/A	N/A	N/A	N/A	Food poisoning
118	N/A	N/A	8	7	53.33	100.00	60.00	100.00	33.33	26.67	N/A	Nut products	D1	Enteritidis	N/A	N/A	Y	Y	Y	N/A	Y	N/A	N/A	N/A	N/A	N/A	Food poisoning
121	N/A	N/A	12	10	54.55	100.00	36.36	90.91	40.91	31.82	N/A	Meat products	E1	London	N/A	F, CXM, CMZ, I	Y	Y	Y	Y	N/A	N/A	N/A	N/A	22	0	Food poisoning
122	N/A	N/A	29	51	36.25	100.00	100.00	100.00	100.00	100.00	66.25	Meat products	E1	Weltevreden	N/A	N/A	Y	Y	Y	N/A	N/A	N/A	N/A	N/A	N/A	N/A	Food poisoning
123	N/A	N/A	18	27	40.00	86.67	75.56	82.22	33.33	22.22	62.22	Meat products	N/A	N/A	N/A	N/A	Y	Y	N/A	N/A	N/A	N/A	N/A	N/A	N/A	N/A	Food poisoning
124	N/A	N/A	9	6	60.00	100.00	100.00	100.00	100.00	100.00	N/A	Frozen food	D1	Enteritidis	N/A	O, CTT, AMK,	Y	Y	Y	Y	N/A	N/A	N/A	N/A	N/A	N/A	Food poisoning
126	N/A	N/A	38	17	69.09	65.45	72.73	67.27	49.09	21.82	70.91	Meat products	D1	Enteritidis	N/A	N/A	Y	Y	Y	N/A	N/A	N/A	N/A	N/A	55	0	Food poisoning
132	44.9	N/A	18	7	72.00	100.00	84.00	100.00	44.00	32.00	64.00	Meat products	E1	London	N/A	N/A	Y	Y	Y	N/A	N/A	N/A	N/A	N/A	25	0	Food poisoning
138	N/A	N/A	18	26	40.91	75.00	100.00	84.09	56.82	40.91	72.73	Meat products	E1	Weltevreden	N/A	N/A	Y	Y	Y	Y	N/A	N/A	N/A	N/A	44	0	Food poisoning
139	36.9	N/A	14	11	56.00	100.00	80.00	96.00	72.00	24.00	68.00	Meat products	D1	Enteritidis	N/A	ZO, AMK, GE	Y	Y	Y	Y	N/A	N/A	N/A	N/A	25	0	Food poisoning
140	38.9	N/A	44	22	66.67	100.00	50.00	62.12	22.76	3.03	N/A	Meat products	B	Typhimurium	N/A	HL, SOX, AMC	Y	Y	Y	Y	Y	N/A	N/A	N/A	66	0	Food poisoning
141	N/A	N/A	39	0	100.00	100.00	100.00	100.00	100.00	N/A	100.00	Meat products	B	Typhimurium	N/A	N/A	Y	Y	Y	N/A	N/A	N/A	N/A	N/A	39	0	Food poisoning
142	37.59	4.32	3	3	50.00	100.00	100.00	100.00	100.00	100.00	100.00	Meat products	D1	Enteritidis	N/A	N/A	Y	Y	Y	N/A	N/A	N/A	N/A	N/A	N/A	N/A	Food poisoning
143	36.67	N/A	67	29	69.79	100.00	100.00	100.00	100.00	100.00	N/A	cessed grain proc	D1	Enteritidis	N/A	N/A	Y	Y	Y	Y	N/A	N/A	N/A	N/A	N/A	N/A	Food poisoning
145	31.8	2.6	4	3	57.14	100.00	100.00	100.00	100.00	100.00	N/A	Meat products	N/A	N/A	N/A	N/A	Y	Y	Y	N/A	N/A	Y	N/A	N/A	7	0	Food poisoning
152	N/A	N/A	1	1	50.00	100.00	100.00	100.00	N/A	N/A	N/A	Egg products	B	Typhimurium	N/A	N/A	Y	Y	Y	Y	N/A	N/A	N/A	N/A	N/A	N/A	Food poisoning
155	N/A	N/A	31	46	40.26	94.81	94.81	89.61	N/A	42.86	75.32	Pastries	D1	Dublin	N/A	N/A	Y	Y	Y	N/A	Y	N/A	N/A	N/A	N/A	N/A	Food poisoning
156	N/A	N/A	40	54	42.55	89.36	65.96	93.62	100.00	47.87	N/A	Meat products	D1	Enteritidis	N/A	N/A	Y	Y	Y	N/A	N/A	N/A	N/A	N/A	N/A	N/A	Food poisoning
158	N/A	N/A	44	30	59.46	N/A	100.00	N/A	N/A	N/A	N/A	N/A	D1	Typhi	N/A	N/A	Y	N/A	Y	N/A	N/A	N/A	N/A	N/A	74	0	aratyphoid fever
159	38.8	N/A	44	22	66.67	100.00	50.00	62.12	22.73	3.03	N/A	Meat products	B	Typhimurium	N/A	N/A	Y	Y	Y	N/A	N/A	N/A	N/A	N/A	66	0	Food poisoning
161	N/A	N/A	20	12	62.50	100.00	25.00	100.00	100.00	100.00	100.00	Meat products	D1	Enteritidis	N/A	N/A	Y	Y	Y	N/A	N/A	N/A	N/A	N/A	32	0	Food poisoning
163	N/A	N/A	11	16	40.74	100.00	100.00	100.00	100.00	100.00	100.00	Meat products	N/A	N/A	N/A	N/A	Y	Y	N/A	N/A	N/A	N/A	N/A	OFX, CRO	27	0	Food poisoning
164	N/A	N/A	10	15	40.00	100.00	N/A	100.00	100.00	100.00	100.00	Meat products	C2	Newport	N/A	ZO, AMK, GE	Y	Y	Y	Y	N/A	N/A	N/A	AMK, GEN, NO	25	0	Food poisoning
166	N/A	N/A	33	46	41.77	96.20	87.34	70.89	44.30	63.29	40.51	Meat products	C1	Typhisuis	N/A	N/A	Y	Y	Y	N/A	N/A	N/A	N/A	CRO, OFX	N/A	N/A	Food poisoning
170	N/A	N/A	27	40	40.30	76.11	77.61	71.64	59.70	50.74	67.16	Others	D1	Enteritidis	N/A	N/A	Y	Y	Y	N/A	N/A	N/A	N/A	N/A	67	0	Food poisoning
172	35	N/A	6	13	31.58	100.00	N/A	100.00	100.00	100.00	N/A	Meat products	N/A	N/A	N/A	N/A	Y	Y	N/A	N/A	N/A	N/A	N/A	N/A	19	0	Food poisoning
173	N/A	N/A	13	17	43.33	100.00	90.00	63.33	N/A	43.33	N/A	Meat products	N/A	N/A	N/A	N/A	Y	Y	N/A	N/A	N/A	N/A	N/A	CIP, CAZ	30	0	Food poisoning
174	N/A	N/A	33	31	51.56	32.81	100.00	56.25	100.00	100.00	68.75	N/A	D1	Typhi	N/A	NAL, FOX, SM	Y	Y	Y	Y	N/A	N/A	N/A	N/A	N/A	N/A	aratyphoid fever
179	N/A	N/A	17	0	100.00	94.12	58.82	100.00	64.71	64.71	58.82	Meat products	C2	Bovismorbificans	N/A	N/A	Y	Y	Y	N/A	N/A	N/A	N/A	N/A	17	0	Food poisoning
180	38	N/A	12	17	41.38	100.00	100.00	100.00	100.00	N/A	N/A	cessed grain proc	D1	Enteritidis	N/A	N/A	Y	Y	Y	N/A	N/A	N/A	N/A	N/A	29	0	Food poisoning
181	N/A	N/A	4	0	100.00	100.00	50.00	N/A	100.00	100.00	N/A	N/A	D1	Enteritidis	N/A	N/A	Y	N/A	Y	N/A	N/A	N/A	N/A	N/A	4	0	Food poisoning
183	N/A	N/A	16	2	88.89	100.00	100.00	83.33	77.78	55.66	72.22	Egg products	N/A	N/A	N/A	N/A	Y	Y	Y	N/A	N/A	N/A	N/A	N/A	18	0	Food poisoning
186	N/A	N/A	4	4	50.00	100.00	100.00	10.00	75.00	62.50	50.00	Egg products	B	Typhimurium	N/A	R, OFX, AMP, A	Y	Y	Y	N/A	N/A	N/A	N/A	N/A	8	0	Food poisoning
189	N/A	N/A	26	29	47.27	90.91	38.18	92.73	81.82	81.82	N/A	Others	D1	Enteritidis	N/A	N/A	Y	Y	Y	N/A	N/A	N/A	N/A	N/A	55	0	Food poisoning
190	N/A	N/A	6	13	31.58	100.00	89.47	100.00	100.00	100.00	100.00	Egg products	D1	Blegdam	N/A	N/A	Y	Y	Y	N/A	N/A	N/A	N/A	LVX	19	0	Food poisoning
191	N/A	N/A	2	10	16.67	100.00	58.33	11.00	50.00	50.00	N/A	N/A	D1	Enteritidis	N/A	N/A	Y	Y	Y	N/A	N/A	N/A	N/A	N/A	12	0	Food poisoning
192	N/A	N/A	8	16	33.33	91.67	79.17	75.00	70.83	37.50	66.67	Meat products	B	Typhimurium	N/A	N/A	Y	Y	Y	N/A	N/A	N/A	N/A	N/A	24	0	Food poisoning
193	32	N/A	21	17	55.26	100.00	68.42	86.84	68.42	39.47	60.53	Egg products	N/A	N/A	N/A	N/A	Y	Y	Y	N/A	N/A	N/A	N/A	N/A	38	0	Food poisoning
194	N/A	N/A	47	104	31.13	100.00	86.75	38.41	N/A	36.42	82.12	N/A	B	Typhimurium	N/A	IP, ATM, IMP, T	Y	Y	Y	Y	Y	N/A	N/A	N/A	N/A	N/A	Food poisoning
195	N/A	N/A	18	38	32.14	100.00	82.14	82.14	100.00	46.43	100.00	Meat products	D1	Enteritidis	N/A	N/A	Y	Y	Y	Y	Y	N/A	N/A	N/A	56	0	Food poisoning
197	N/A	N/A	28	31	47.46	88.14	64.41	91.53	100.00	74.58	N/A	Meat products	D1	Typhi	N/A	N/A	Y	Y	Y	N/A	N/A	N/A	N/A	N/A	59	0	Food poisoning
200	N/A	N/A	15	21	41.67	100.00	61.11	58.33	100.00	100.00	100.00	cessed grain proc	D1	Enteritidis	N/A	N/A	Y	Y	Y	N/A	N/A	N/A	N/A	N/A	36	0	Food poisoning
201	55	15.2	28	13	68.29	100.00	39.02	70.73	N/A	36.59	39.02	Meat products	B	Typhimurium	N/A	N/A	Y	Y	Y	N/A	N/A	N/A	N/A	OFX	41	0	Food poisoning
202	N/A	N/A	76	59	56.30	59.72	100.00	84.72	100.00	91.67	100.00	Aquatic products	D1	Enteritidis	N/A	N/A	Y	Y	Y	Y	Y	N/A	hage infection te	N/A	135	0	Food poisoning
203	41	N/A	28	22	56.00	100.00	100.00	100.00	100.00	100.00	100.00	Meat products	N/A	N/A	N/A	N/A	N/A	N/A	N/A	N/A	N/A	N/A	N/A	N/A	50	0	Food poisoning
204	N/A	N/A	3	2	60.00	100.00	100.00	100.00	100.00	100.00	N/A	Egg products	B	Typhimurium	N/A	N/A	Y	Y	Y	N/A	N/A	Y	N/A	N/A	5	0	Food poisoning
206	N/A	N/A	69	38	64.49	88.89	82.72	85.19	25.93	17.28	61.73	N/A	D1	Dublin	N/A	N/A	Y	Y	Y	N/A	N/A	N/A	N/A	N/A	107	0	Food poisoning
209	N/A	N/A	166	241	40.79	100.00	100.00	100.00	100.00	100.00	N/A	Meat products	N/A	N/A	N/A	N/A	Y	Y	Y	N/A	N/A	N/A	N/A	N/A	407	0	Food poisoning
211	N/A	N/A	29	45	39.19	100.00	N/A	100.00	100.00	100.00	N/A	Meat products	N/A	N/A	N/A	N/A	Y	Y	N/A	N/A	N/A	N/A	N/A	N/A	74	0	Food poisoning
212	52.3	N/A	11	11	50.00	100.00	100.00	100.00	100.00	100.00	N/A	Meat products	D1	Enteritidis	N/A	N/A	Y	Y	Y	N/A	N/A	N/A	N/A	N/A	22	0	Food poisoning
215	N/A	N/A	20	16	55.56	97.22	77.78	91.67	61.11	77.78	N/A	N/A	N/A	N/A	N/A	N/A	Y	Y	N/A	N/A	N/A	N/A	N/A	N/A	36	0	Food poisoning
216	N/A	N/A	25	38	39.68	100.00	100.00	100.00	100.00	100.00	100.00	Meat products	D1	Dublin	N/A	N/A	Y	Y	Y	N/A	N/A	Y	N/A	N/A	63	0	Food poisoning
217	N/A	N/A	9	11	45.00	100.00	100.00	N/A	N/A	N/A	100.00	Others	D1	Enteritidis	N/A	N/A	Y	Y	Y	N/A	N/A	Y	N/A	N/A	20	0	Food poisoning
222	28.3	1.8	7	12	36.84	100.00	78.95	89.47	N/A	68.42	36.84	Meat products	D1	Enteritidis	N/A	N/A	Y	Y	Y	N/A	N/A	N/A	N/A	N/A	19	0	Food poisoning
223	N/A	N/A	10	5	66.67	100.00	N/A	100.00	100.00	100.00	N/A	N/A	N/A	N/A	N/A	N/A	Y	Y	N/A	N/A	N/A	N/A	N/A	N/A	15	0	Food poisoning
225	36	N/A	4	2	66.67	100.00	100.00	100.00	100.00	100.00	100.00	Meat products	D1	Enteritidis	N/A	N/A	Y	Y	Y	N/A	N/A	N/A	N/A	CIP	6	0	Food poisoning
228	<1	<1	2	2	50.00	100.00	100.00	N/A	N/A	N/A	N/A	N/A	B	Stanley	N/A	TX, FEP, CAZ,	Y	Y	Y	Y	N/A	N/A	N/A	N/A	4	0	ociated infection
229	N/A	N/A	25	20	55.56	100.00	4.44	77.78	73.33	11.11	71.11	N/A	N/A	N/A	N/A	N/A	Y	Y	N/A	N/A	N/A	N/A	N/A	N/A	45	0	Food poisoning
234	41.25	18.38	6	2	75.00	100.00	12.50	75.00	100.00	100.00	N/A	Egg products	N/A	N/A	N/A	N/A	Y	N/A	N/A	N/A	N/A	N/A	N/A	N/A	8	0	Food poisoning
237	N/A	N/A	24	37	39.34	100.00	100.00	100.00	100.00	100.00	100.00	Meat products	D1	N/A	N/A	N/A	Y	Y	Y	N/A	N/A	N/A	N/A	N/A	61	0	Food poisoning
238	N/A	N/A	12	9	57.14	100.00	100.00	100.00	100.00	100.00	100.00	N/A	N/A	N/A	N/A	N/A	Y	N/A	N/A	N/A	N/A	N/A	N/A	N/A	21	0	Food poisoning
239	N/A	N/A	5	11	31.25	100.00	6.25	100.00	18.75	18.75	N/A	Aquatic products	N/A	N/A	N/A	N/A	Y	Y	Y	N/A	N/A	Y	N/A	N/A	16	0	Food poisoning
240	N/A	N/A	55	41	57.29	97.92	84.38	93.75	68.75	51.04	81.25	Others	N/A	N/A	N/A	N/A	Y	Y	N/A	N/A	N/A	N/A	N/A	N/A	96	0	Food poisoning
243	N/A	N/A	11	2	84.62	100.00	100.00	100.00	100.00	100.00	N/A	Egg products	D1	Typhi	N/A	N/A	Y	Y	Y	N/A	N/A	Y	N/A	N/A	15	0	Food poisoning
245	N/A	N/A	17	25	40.48	97.62	58.33	61.90	26.19	26.19	35.71	Meat products	D1	Enteritidis	N/A	N/A	Y	Y	Y	N/A	N/A	N/A	N/A	N/A	42	0	Food poisoning
248	N/A	N/A	33	62	34.74	100.00	100.00	100.00	100.00	100.00	100.00	Meat products	D1	Enteritidis	N/A	N/A	Y	Y	Y	N/A	N/A	Y	N/A	N/A	N/A	N/A	Food poisoning
252	N/A	N/A	9	6	60.00	100.00	53.33	100.00	100.00	26.67	60.00	Meat products	E1	Weltevreden	N/A	N/A	Y	Y	Y	N/A	N/A	Y	N/A	NOR, SXT	15	0	Food poisoning
257	N/A	N/A	49	44	52.69	93.55	64.52	77.42	50.54	43.01	60.22	N/A	D1	Moscow	N/A	ID, AMP, PIP, S	Y	Y	Y	Y	Y	N/A	N/A	N/A	93	0	Food poisoning
259	13.64	N/A	46	59	43.81	54.30	74.30	68.00	47.60	25.70	53.30	Others	D1	Enteritidis	N/A	N/A	Y	Y	Y	N/A	N/A	N/A	N/A	N/A	N/A	N/A	Food poisoning
260	N/A	N/A	33	45	42.31	100.00	100.00	100.00	100.00	100.00	100.00	Meat products	E1	Anatum	N/A	PEN	Y	Y	Y	N/A	N/A	Y	N/A	N/A	N/A	N/A	Food poisoning
263	N/A	N/A	76	85	47.20	88.20	67.70	88.20	47.20	47.20	67.70	Meat products	D1	N/A	N/A	AMX, CIP, AMP	Y	Y	Y	N/A	N/A	Y	N/A	N/A	161	0	Food poisoning
266	N/A	N/A	10	6	62.50	100.00	100.00	100.00	100.00	100.00	N/A	Meat products	C1	Paratyphi C	N/A	AMP	Y	Y	Y	N/A	N/A	N/A	N/A	N/A	16	0	Food poisoning
274	N/A	N/A	9	14	39.13	100.00	4.35	100.00	100.00	100.00	100.00	Meat products	N/A	N/A	N/A	N/A	Y	Y	Y	N/A	N/A	N/A	N/A	N/A	N/A	N/A	Food poisoning
279	29	N/A	13	14	48.15	100.00	85.19	77.78	55.56	44.44	N/A	on-frozen bevera	B	Schwarzengrund	N/A	N/A	Y	Y	Y	N/A	N/A	Y	N/A	N/A	27	0	Food poisoning

282	季洪斌.	Ji Hongbin.	肠炎沙门氏菌食	teritidis food	中国初级卫生保	y health care i	25	9	70-71	2011	ssn.1001-568X.2	2011	0	4	1	Eastern	Jiangsu	Nantong	[illegible]	[illegible]	[illegible]	[illegible]	[illegible]	[illegible]	[illegible]	[illegible]	[illegible]	[illegible]
283	魏清文.张伟雄.	qwen.Zhang V	伤寒沙门菌引起	caused by Salm	中国职业医学	Occupational	-	S1	88-89	2011	-	2009	2	5	2	Eastern	Guangdong	Shanwei	[illegible]	[illegible]	[illegible]	[illegible]	[illegible]	[illegible]	[illegible]	[illegible]	[illegible]	[illegible]
284	徐里强.	Xu Liqiang.	食物中毒事件的	s of a collectiv	中国民康医学	eople's Health	23	18	2324	2011	issn.1672-0369.2	2011	0	5	2	Eastern	Jiangsu	Changshu	31.39	120.43	Office	17	30	56.67	23	47	N/A	N/A
285	王均华.高海英.	Junhua.Gao H	门菌引起食物中	ood poisoning	中国现代医生	lern chinese d	49	30	5,8	2011	issn.1673-9701.2	2008	3	7	2	Eastern	Jiangsu	Wuxi	31.34	120.18	Residential area	4	4	100.00	23	58	N/A	N/A
288	王金锋.	Wang Jinfeng	门氏菌引起食物	f food poisonir	医学动物防制	lical animal co	26	6	556	2010	issn.1003-6245.2	2008	2	6	2	Eastern	Hebei	Tangshan	39.36	118.11	Residential area	20	20	100.00	15	59	N/A	N/A
295	郭皓.陈松.	o Hao.Chen Sc	沙门菌引起的	ood poisoning	预防医学情报杂	ventive Medici	26	3	230-230	2010	-	2009	1	8	2	Western	Sichuan	Dazhou	31.21	107.5	Residential area	20	120	16.67	10	70	N/A	N/A
301	王兴.代传焕.孟	.Dai Chuanhu	门菌引起的食物	ood poisoning	职业与健康	upation and h	26	5	517-518	2010	-	2008	2	3	2	Eastern	Jiangsu	Wuxi	31.34	120.18	Residential area	19	300	6.33	N/A	N/A	35	N/A
306	马吉辉.陈卫武.	Jihui.Chen We	沙门菌引起食物	food poisoning	中国当代医药	contemporary	17	23	117,122	2010	issn.1674-4721.2	2009	1	10	2	Western	Yunnan	Honghe	23.48	101.85	mobile street sta	37	61	60.66	5	72	N/A	N/A
308	克志.赵严.陈宝	hi.Zhao Yan.C	沙门氏菌污染	hogenic Esche	中国初级卫生保	y health care i	24	5	66	2010	issn.1001-568X.2	2006	4	8	2	Western	Yunnan	Dali	25.34	100.13	Residential area	213	600	35.50	1	75	N/A	N/A
309	明标.李淑芳.李	iao.Li Shufang	门菌引起食物中	poisoning ca	中国当代医药	contemporary	17	27	129-130	2010	issn.1674-4721.2	2009	1	5	2	Central	Hunan	Chenzhou	25.46	113.02	Residential area	12	30	40.00	4	71	N/A	N/A
311	国旗.郭超.陈光	i.Guo Chao.Ch	席引发食物中毒	ng incident ca	中国食品卫生杂	Food Hygiene	22	2	171-172	2010	-	2009	1	6	3	Western	Yunnan	Baoshan	25.08	99.1	Residential area	95	200	47.50	4	80	40	N/A
313	春华.潘海燕.张	.Pan Haiyan.Z	门菌引起的一起	reak caused b	中国卫生检验杂	urnal of Health	20	4	912-913	2010	-	2009	1	2	2	Western	Yunnan	Yuxi	24.22	102.32	Residential area	85	536	15.86	N/A	N/A	N/A	N/A
316	朱晶.	Zhu Jing.	引发食物中毒的	poisoning cau	现代医药卫生	n medicine and	26	22	3510-3511	2010	-	2009	1	8	2	Eastern	Jiangsu	Taizhou	32.3	119.54	Restaurant	30	50	60.00	8	46	N/A	N/A
317	曹妍.李丽.	Cao Yan.Li Li.	门菌引起食物中	of food poiso	预防医学情报杂	ventive Medici	26	5	413-414	2010	-	2007	3	7	2	Western	Sichuan	Nanchong	30.49	106.04	School	28	284	9.86	17	19	N/A	N/A
319	王振海.	Wang Zhenhai	门氏菌食物中毒	s of a case of s	疾病监测与控制	surveillance an	4	1	27-27	2010	-	2007	3	8	3	Western	Inner mongolia	Chifeng	42.17	118.58	Office	212	895	23.69	N/A	N/A	N/A	N/A
320	王小红.龚成林.	aohong.Gong	伤寒沙门菌引起	g caused by S	中国卫生检验杂	urnal of Health	20	6	1536	2010	-	2009	1	5	2	Western	Gansu	Lanzhou	36.04	103.51	Restaurant	15	15	100.00	N/A	N/A	N/A	N/A
321	许华.廖周勇.	Hua.Liao Zhou	菌引起食物中毒	ase of food po	现代预防医学	n preventive m	37	20	3944-3944	2010	-	2009	1	8	4	Western	Sichuan	Nanchong	30.49	106.04	Restaurant	64	325	19.69	N/A	N/A	N/A	N/A
323	吴红玲.马红梅.	ongling.Ma Hc	氏菌引起食物中	od poisoning	宁夏医学杂志	xia Medical Jc	32	12	1256-1257	2010	issn.1001-5949.2	2009	1	7	2	Western	Ningxia	Guyuan	35.92	106.18	Residential area	36	66	54.55	3	66	N/A	N/A
324	建如.杨光耀.张	ng Guangyao.	高校水污染事件	r pollution inc	实用预防医学	al preventive m	17	6	1133-1134	2010	issn.1006-3110.2	2009	1	5	17	Central	Hunan	Changsha	28.12	112.59	School	234	11790	1.98	N/A	N/A	N/A	N/A
327	樊毅.	Fan Yi.	副伤寒沙门菌引	ness caused by	中国卫生检验杂	urnal of Health	20	11	2978-2979	2010	-	2009	1	3	2	Western	Chongqing	Wanzhou	29.35	106.33	Residential area	22	298	7.38	8	71	N/A	N/A
331	魏强.田野.	Wei Qiang.fiel	水源污染致腹泻	caused by wa	预防医学论坛	ntive Medicine	15	3	287-288	2009	-	2007	2	7	4	Western	Inner mongolia	Tongliao	43.37	122.16	School	516	1900	27.15	17	21	N/A	N/A
334	佳娟.邢素琴.于	an.Xing Suqin.	门氏菌引起的食	oisoning cause	预防医学论坛	ntive Medicine	15	4	封3	2009	-	2008	1	3	3	Western	Inner mongolia	Chifeng	42.17	118.58	Restaurant	33	130	25.38	6	64	N/A	N/A
335	石志翼.李军义.	hi Zhiyi.Li Jun	门氏菌引起食物	poisoning cau	中国农村卫生事业	ural health ma	29	1	61-62	2009	-	2008	1	5	5	Western	Ningxia	Guyuan	35.92	106.18	Residential area	26	51	50.98	3	66	N/A	N/A
336	友铺.于美.麦丽	u.Yu Mei.Mai	沙湾县一起食物	food poisoning	地方病通报	ic disease noti	-	3	66	2009	-	2007	2	8	4	Western	Xinjiang	Ili	43.46	82.05	Restaurant	180	250	72.00	3	88	N/A	N/A
338	黄运红.陈应平.	unhong.Chen	引起沙门氏菌食	a food poison	现代预防医学	n preventive m	36	20	38,593,862	2009	-	2008	1	9	1	Central	Jiangxi	Nanchang	28.4	115.55	Residential area	5	5	100.00	6	55	N/A	N/A
341	王静辉.史永刚.	inghui.Shi Yor	宴席引起的肠炎	oning caused	中国卫生检验杂	urnal of Health	19	12	29,853,015	2009	-	2008	1	10	2	Eastern	Hebei	Tangshan	39.36	118.11	Residential area	176	582	30.24	N/A	N/A	N/A	N/A
348	建林.郭定成.牛	Guo Dingchen	门氏菌导致的食	oisoning caus	药与保健（下旬）	d Health Care	17	8	48-49	2009	-	2008	1	8	2	Western	Gansu	Tianshui	34.37	105.42	Residential area	20	208	9.62	11	65	N/A	N/A
350	马世武.何海英.	u.He Haiying.	甲型副伤寒暴发	of paratyphoi	华临床感染病杂	of Clinical Inf	2	5	314-315	2009	.j.issn.1674-2397	2008	1	9	69	Western	Yunnan	Kunming	25.04	102.42	School	105	2776	3.78	18	45	22.3	N/A
351	许华.	Xu Hua.	氏菌引起食物中	case of food p	学信息（医药版	ation (Pharma	-	11	-	2009	-	2009	0	5	1	Western	Sichuan	Nanchong	30.49	106.04	Residential area	2	2	100.00	N/A	N/A	65	N/A
353	李卫光.朱其凤.	eiguang.Zhu Q	病房阿伯丁沙门菌	erdeen nosoc	华医院感染学杂	of Hospital In	19	15	1954-1955	2009	issn:1005-4529.2	2007	2	3	18	Eastern	Shandong	Jinan	36.4	117	Hospital	22	30	73.33	<1	<1	<1	<1
354	尹薛荣.王群.	uerong.Wang	由猪霍乱沙门氏	caused by Sal	地方病通报	ic disease noti	24	1	63-63	2009	-	2005	4	4	N/A	Western	Xinjiang	Ili	43.46	82.05	Residential area	16	16	100.00	5	61	N/A	N/A
355	吟涓.唐飞.吴维	an.Tang Fei.W	例引起细菌性食	and analysis c	中国基层医药	a primary med	16	7	1294-1295	2009	.j.issn.1008-6706	2007	2	5	2	Eastern	Guangdong	Shenzhen	22.33	114.07	Factory	8	450	1.78	21	37	N/A	N/A
358	高翠文.朱怀甫.	n.Zhu Huaifu.	门氏菌引起食物	oisoning cause	中外健康文摘	nd Foreign He	6	33	18-19	2009	issn.1672-5085.2	2005	4	8	2	Western	Guangxi	Laibin	23.65	109.43	Residential area	58	140	41.43	8	84	N/A	N/A
359	惠勇.田锦萍.孙	ong.Tian Jinpi	门菌引起食物中	ood poisoning	中国卫生检验杂	urnal of Health	19	12	2980-2981	2009	-	2009	0	6	2	Eastern	Jiangsu	Lianyungang	34.63	119.11	Residential area	7	8	87.50	3	66	N/A	N/A
360	刘万郁.	Liu Wanyu.	门氏菌引起食物	d poisoning c	现代预防医学	n preventive m	36	24	4675-4675,4683	2009	-	2008	1	7	3	Western	Sichuan	Guangyuan	32.28	105.51	Residential area	28	64	43.75	7	67	N/A	N/A
361	苗晓红.	Miao Xiaohonc	氏菌引起的食物	case of food p	中国现代药物应	rug applicatic	3	14	193	2009	issn.1673-9523.2	2006	3	6	4	Northeastern	Heilongjiang	Mudanjiang	44.35	129.36	Construction site	25	25	100.00	18	54	N/A	N/A
362	黄继贵.章光斌.	Jiqui.Zhang Gu	宴引起沙门氏菌	poisoning cau	现代预防医学	n preventive m	35	9	17,271,730	2008	issn.1003-8507.2	2007	1	6	3	Central	Hubei	Jingzhou	29.86	112.66	Residential area	51	85	60.00	6	88	N/A	N/A
363	王兵.	Wang Bing.	伤寒沙门氏菌食	himurium food	泸州医学院学报	Luzhou Medic	31	6	656-657	2008	issn.1000-2669.2	2006	2	7	3	Western	Sichuan	Luzhou	28.54	105.24	Residential area	86	98	87.76	N/A	N/A	N/A	N/A
364	滕益棋.李玉厚.	ngpanqi.Li Yul	门氏菌肠炎调查报	reports on cas	中国初级卫生保	y health care i	22	7	59	2008	issn.1001-568X.2	2004	4	8	2	Northeastern	Heilongjiang	Heihe	50.14	127.29	Residential area	7	32	21.88	25	43	N/A	N/A
365	何政.	He Zheng.	门菌污染引起的	g caused by Sa	职业与健康	upation and h	24	3	233-233	2008	issn.1004-1257.2	2006	2	8	2	Eastern	Jiangsu	Wuxi	31.34	120.18	Restaurant	25	50	50.00	8	40	N/A	N/A
367	丽华.刘加学.王	Liu Jiaxue.Wan	生肠炎沙门氏菌	used by Salmo	宁夏医学院学报	Ningxia Medi	30	2	235-236	2008	issn.1674-6309.2	2006	2	5	2	Western	Ningxia	Shizuishan	39.02	106.22	Construction site	6	11	54.55	18	42	N/A	N/A
369	石峻.	Shi Jun.	起鼠伤寒沙门菌	d by Salmonel	现代预防医学	n preventive m	35	2	250,252	2008	issn.1003-8507.2	2003	5	5	4	Western	Sichuan	Chengdu	30.4	104.04	Residential area	53	65	81.54	3	76	N/A	N/A
371	余惠琴.王晓艳.	uigin.Wang Xi	氏菌集体食物中	ood poisoning	大理学院学报	al of Dali Univ	7	2	49-50,53	2008	issn.1672-2345.2	2006	2	8	N/A	Western	Yunnan	Dali	25.34	100.13	Residential area	55	76	72.37	1	71	N/A	N/A
372	吾福尔.李方.木	u.Li Fang.woo	伤寒爆发疫情分子	ion of typhoid	中国热带医学	se tropical me	8	10	1699-1700,1703	2008	issn.1009-9727.2	2007	1	8	45	Western	Xinjiang	N/A	43.45	87.36	Prison	57	2484	2.29	18	46	N/A	N/A
374	余惠琴.严文芳.	uiqin.Yan Wer	氏菌引起的食物	oisoning caus	云南预防医学杂	rnal of Prevent	13	2	65	2008	-	2005	3	8	3	Western	Yunnan	Dali	25.34	100.13	Residential area	15	15	100.00	3	84	N/A	N/A

612	朱墙.李秀昵.李	g.Li Xiuni.Li Sh	沙门氏菌食物中毒	e of Salmonella	中国卫生检验杂	urnal of Health	11	5	637	2001	issn.1004-8685.2	2000	1	9	1	Western
617	昌海.杨周祥.段	Yang Zhouxiar	登沙门氏菌引起	d poisoning ca	中国卫生监督杂	urnal of Health	8	1	8-9	2001	issn.1077-6131.2	2000	1	2	3	Western
618	旧仁贵.罗建平.李	ngui.Luo Jianp	门氏菌食物中毒	a case of saln	实用预防医学	al preventive m	8	4	298-298	2001	issn.1006-3110.2	2000	1	9	5	Central
619	添发.王淑文.周	Wang Shuwen	氏菌食物中毒的	s of a case of s	中国公共卫生管	ublic health ma	17	2	155-156	2001	issn.1001-9561.2	1999	2	7	1	Eastern
620	.吴建明.马占礼.	nming.Ma Zha	门氏菌引起的食	oisoning caus	中国学校卫生	nese school he	-	4	306	2001	issn.1000-9817.2	2000	1	6	4	Western
621	宋整风.黄崇艾.	ngfeng.Huanc	污染熟肉丸引	y salmonella c	放军预防医学杂	edicine of the	18	1	69	2000	issn.1001-5248.2	1999	1	3	1	Eastern
625	焕昭.李柏桂.林	nzhao.Li Baiqu	引起食物中毒	ood poisoning	国人兽共患病杂	e Journal of Zc	9	3	115	1999	issn.1002-2694.1	1998	1	5	1	Central
629	维健.尉向海.王	.Wei Xianghai.	门氏菌食物中毒	case of severe	学学报（医学	niversity (Med	-	1	73-76	2000	-	1999	1	11	2	Central
630	鞠勇.	Ju Yong.	门氏菌所致的食	soning caused	预防医学情报杂	ventive Medici	-	2	162	2000	-	1998	2	7	4	Western
631	宇.桂立新.孙玉	Gui Lixin.Sun Y	门氏菌引起的食	soning caused	安徽预防医学杂	nal of Preventi	-	2	159	2000	-	1999	1	9	3	Central
633	.谢红辉.罗亮宇.	Ionghui.Luo Li	沙门氏菌引起的	d poisoning ca	实用预防医学	al preventive m	7	3	212	2000	issn.1006-3110.2	1999	1	9	1	Central
637	董云.李柏桂.朱	gyun.Li Baiqui.	林沙门氏菌阿	a dublin Accra	现代预防医学	n preventive m	-	1	104	1999	-	1997	2	9	N/A	Central
638	李长庆.胡艳冰.	ngqing.Hu Ya	菌引起家庭暴发	of food poisor	华南国防医学杂	nal of National	-	2	64	1999	-	1997	2	1	1	Eastern
639	亚平.杨春秀.郑	g.Yang Chun	卢普沙门氏菌食	Salmonella Br	预防医学文献信	edicine literatu	-	1	14-15,17	1999	-	1998	1	4	3	Central
645	郭秀翠.付鹤迎.	Xiucui.Fu He	沙门氏菌病流	estigation of S	山东预防医学	ng Preventive I	18	1	23-23,22	1998	-	1995	3	5	210	Eastern
647	孙敬中.尹志晶.	ngzhong.Yin Z	沙门氏菌食物中	f food poisoni	山东预防医学	ng Preventive I	18	3	176	1998	-	1997	1	7	2	Eastern
648	.褚保林.胡志林.	Baolin.Hu Zhi	沙门氏菌引起	g caused by S	现代预防医学	n preventive m	-	1	84-85	1998	-	1997	1	8	3	Central
650	继荣.程永森.梁	eng Yongsen.L	沙门氏菌所致食	ning caused b	安徽预防医学杂	nal of Preventi	-	1	46	1998	-	1997	1	11	2	Central
651	赵淑兰.林化同.	Shulan.Lin Hu	门氏菌引起的食	d poisoning ca	代中西医结合杂	d Traditional C	-	11	0	1998	-	1997	1	4	3	Eastern
652	金志善.梁玉梅.	Ji Sun.Liang Yu	沙门氏菌食物中	of Salmonella	山东预防医学	ng Preventive I	17	1	34	1997	-	1995	2	8	3	Eastern
653	吴松涛.	Wu Songtao.	沙门氏菌致食物	oisoning cause	安徽预防医学杂	nal of Preventi	-	4	60	1997	-	1996	1	11	2	Central

[illegible]

948	许秀勇.张嘉祥	ıyong.Zhang J	引起伤寒暴发流	alence of typh	海峡预防医学杂	nal of Preventi	-	4	29-13	1999	-	1996	3	8	38	Eastern	Fujian	Sanming	26.13	117.36	Residential area	64	386	16.58	4	72	N/A	N/A	42	22	65.63	N/A	100.00	100.00	N/A	N/A	100.00	N/A	D1	Typhi	N/A	N/A	Y	N/A	Y	N/A	N/A	N/A	N/A	N/A	N/A	N/A	aratyphoid fever
951	司来.梁永春.吉	ai.Liang Yonq	寒暴发的流行病	qation of an o	江苏预防医学	u Preventive M	-	3	77-78	1999	-	1998	1	4	24	Eastern	Jiangsu	Yangzhou	32.23	119.26	School	17	1837	0.93	5	20	N/A	N/A	11	6	64.71	N/A	100.00	N/A	N/A	N/A	N/A	N/A	D1	Typhi	N/A	N/A	Y	N/A	Y	N/A	N/A	N/A	N/A	N/A	N/A	N/A	aratyphoid fever
955	建华.杜其筠.欧	hua.Du Qijun.	寒暴发疫情的调	rt on an outbre	现代预防医学	n preventive m	-	1	1	1999	-	1997	2	11	36	Western	Sichuan	Chengdu	30.4	104.04	School	18	2299	0.78	N/A	N/A	20.3	N/A	7	11	38.89	100.00	100.00	N/A	N/A	N/A	100.00	N/A	D1	Typhi	N/A	N/A	Y	N/A	Y	N/A	N/A	N/A	N/A	N/A	18	0	aratyphoid fever
956	王立富.严先民	g Lifu.Yan Xia	引起的伤寒暴发	hoid outbreak	疾病监测	sease surveilla	-	12	1	1998	-	1997	1	8	18	Eastern	Shandong	Jining	35.23	116.33	Residential area	81	645	12.56	3	64	N/A	N/A	42	39	51.85	N/A	100.00	100.00	N/A	N/A	N/A	N/A	D1	Typhi	N/A	N/A	Y	N/A	Y	N/A	N/A	N/A	N/A	N/A	N/A	N/A	: Food poisoning
958	小垒.唐昌新.唐	Tang Changxin	甲型副伤寒暴发	tbreak of para	广西预防医学	xi Preventive M	-	6	1	1998	-	1996	2	9	38	Western	Guangxi	Guilin	25.17	110.17	School	10	807	1.24	12	16	N/A	N/A	6	4	60.00	100.00	100.00	100.00	N/A	N/A	100.00	N/A	A	Paratyphi A	N/A	N/A	Y	N/A	Y	N/A	N/A	N/A	N/A	N/A	N/A	N/A	aratyphoid fever
959	.李航文.张栓虎	ngwen.Zhang	暴发的流行病学	ation and anal	河南预防医学杂	nal of Preventi	-	5	1	1998	-	1997	1	6	64	Central	Henan	Anyang	36.06	114.21	Residential area	57	120	47.50	4	70	N/A	N/A	33	24	57.89	59.09	100.00	59.09	N/A	N/A	20.45	N/A	D1	Typhi	N/A	N/A	Y	N/A	Y	N/A	N/A	N/A	N/A	N/A	57	0	aratyphoid fever
961	瑞民.崔顺领.郭	in.Cui Soonlin	起水型伤寒暴发	outbreak of wa	河南预防医学杂	nal of Preventi	-	4	1	1998	-	1996	2	6	28	Central	Henan	Puyang	35.44	115.01	Residential area	24	1951	1.23	1	37	N/A	N/A	15	9	62.50	50.00	100.00	N/A	N/A	N/A	N/A	N/A	D1	Typhi	N/A	N/A	Y	N/A	Y	N/A	N/A	N/A	N/A	N/A	N/A	N/A	aratyphoid fever
962	张子平	Zhang Ziping	型伤寒暴发流行	outbreak of wa	疾病监测	sease surveilla	-	6	3	1998	-	1995	3	11	35	Eastern	Jiangsu	Taizhou	32.3	119.54	School	32	1621	1.97	N/A	N/A	N/A	N/A	32	0	100.00	100.00	100.00	100.00	N/A	N/A	N/A	N/A	D1	Typhi	N/A	N/A	Y	N/A	Y	N/A	N/A	N/A	N/A	N/A	N/A	N/A	aratyphoid fever
966	其伟.刘子杰.李	iwei.Liu Zijie.L	库区伤寒暴发、	ce of typhoid	预防医学文献信	edicine literatu	-	2	138-139	1998	nt.issn.1672-915	1996	2	7	26	Central	Henan	Nanyang	33	112.32	Residential area	178	1957	9.10	3	83	N/A	N/A	83	95	46.63	70.22	100.00	70.22	N/A	N/A	N/A	N/A	D1	Typhi	N/A	N/A	Y	N/A	Y	N/A	N/A	N/A	N/A	CHL, SXT, AMI	N/A	N/A	aratyphoid fever
969	项慧	Xiang Hui	菌污染井水引起	by salmonella	浙江预防医学	g Preventive M	-	6	63	1997	ki.issn1007-093	1995	2	6	21	Eastern	Zhejiang	Taizhou	28.41	121.27	Residential area	26	354	7.34	7	66	N/A	N/A	8	18	30.77	N/A	100.00	60.00	N/A	N/A	N/A	N/A	D1	Typhi	N/A	N/A	Y	Y	Y	N/A	N/A	N/A	N/A	N/A	26	0	aratyphoid fever
972	黄益民	Huang Yimin	山区伤寒暴发的	eak of typhoid	疾病监测	sease surveilla	-	9	2	1997	-	1996	1	3	231	Western	Chongqing	Pengshui	29.35	106.33	Residential area	1397	24726	5.65	1	94	N/A	N/A	750	147	53.69	100.00	100.00	N/A	N/A	N/A	100.00	N/A	D1	Typhi	N/A	N/A	Y	N/A	Y	N/A	N/A	N/A	N/A	N/A	1396	1	aratyphoid fever
973	余录根	Yu Lugen	寒暴发的疫区处	outbreak of ty	疾病监测	sease surveilla	-	7	2	1997	-	1996	1	6	11	Eastern	Jiangsu	Nanjing	32.03	118.46	Residential area	17	1460	1.16	3	50	N/A	N/A	6	11	35.29	N/A	100.00	100.00	N/A	N/A	N/A	N/A	D1	Typhi	N/A	N/A	Y	N/A	Y	N/A	N/A	N/A	N/A	N/A	N/A	N/A	aratyphoid fever
974	詹根龙	Zhan Genlong	寒暴发流行的调	lysis of an out	疾病控制杂志	al of disease c	-	2	145	1997	-	1996	1	8	102	Central	Anhui	Luan	31.44	116.28	School	115	2176	5.28	8	36	N/A	N/A	90	35	78.26	100.00	100.00	100.00	N/A	N/A	N/A	N/A	D1	Typhi	N/A	N/A	Y	N/A	Y	N/A	N/A	N/A	N/A	N/A	115	0	aratyphoid fever
977	曾纯青.谢元甫	Chunqing.Xie	个单位伤寒暴发	of typhoid feve	实用预防医学	al preventive m	-	2	102	1997	-	1996	1	7	36	Central	Hunan	Loudi	27.44	111.59	Office	36	326	11.04	18	44	N/A	N/A	31	5	86.11	33.33	100.00	33.33	52.78	52.78	97.22	N/A	D1	Typhi	N/A	N/A	Y	N/A	Y	N/A	N/A	N/A	N/A	N/A	N/A	N/A	aratyphoid fever
978	.付盛元.刘建湘	engyuan.Liu Ji	学校水型伤寒暴	r-type typhoid	实用预防医学	al preventive m	-	1	14	1997	-	1994	3	4	23	Central	Hunan	Hengyang	26.53	112.37	School	58	1200	4.83	N/A	N/A	N/A	N/A	52	6	89.66	14.63	100.00	24.39	14.63	7.32	65.85	N/A	D1	Typhi	N/A	N/A	Y	N/A	Y	N/A	N/A	N/A	N/A	N/A	N/A	N/A	aratyphoid fever
979	王玉萍.白永华	Yuping.Bai Yo	县伤寒暴发流行	yphoid fever o	疾病监测	sease surveilla	-	2	2	1997	-	1994	3	11	211	Western	Qinghai	Huangnan	34.86	101.57	Residential area	131	44992	0.29	1	68	N/A	N/A	83	48	63.36	100.00	100.00	N/A	N/A	N/A	N/A	N/A	D1	Typhi	N/A	N/A	Y	N/A	Y	N/A	N/A	N/A	N/A	N/A	130	1	aratyphoid fever
980	翠云.林玫.董柏	Lin Mei.Dong	伤寒暴发的调查	analysis of typ	广西预防医学	xi Preventive M	-	1	23-24	1997	-	1996	1	10	33	Western	Guangxi	Hechi	24.42	108.03	School	341	1845	18.48	N/A	N/A	N/A	N/A	142	193	41.64	100.00	100.00	100.00	N/A	N/A	100.00	N/A	D1	Typhi	N/A	ERY, PEN	Y	N/A	Y	N/A	N/A	N/A	N/A	CHL	341	0	aratyphoid fever
982	莫洪群	mohongqun	洋村伤寒暴发流	d fever outbre	黔南民族医专学	nan National I	-	4	38-39	1996	-	1994	2	3	60	Western	Guizhou	Qiannan	26.02	107.25	Residential area	97	661	14.67	5	60	N/A	N/A	47	50	48.45	46.39	57.73	20.62	N/A	N/A	N/A	N/A	D1	Typhi	N/A	N/A	Y	N/A	Y	N/A	N/A	N/A	N/A	N/A	N/A	N/A	aratyphoid fever
983	王二梅，王新生	rmei, Wang Xi	第四中学伤寒暴	outbreak in N	疾病监测	sease surveilla	-	10	2	1996	-	1995	1	10	42	Central	Henan	Zhumadian	32.58	114.01	School	19	2196	0.87	6	44	N/A	N/A	4	15	21.05	100.00	100.00	N/A	N/A	100.00	N/A	N/A	D1	Typhi	N/A	N/A	Y	N/A	Y	N/A	N/A	N/A	N/A	N/A	18	1	aratyphoid fever
985	王获章，王章云	hang, Wang Z	乙型副伤寒暴发	outbreak of in	中国公共卫生	nese public he	-	9	1	1996	-	1995	1	6	57	Central	Hunan	Zhangjiajie	29.08	110.29	Residential area	45	378	11.90	21	59	N/A	N/A	23	22	51.11	N/A	100.00	N/A	N/A	N/A	100.00	N/A	B	Paratyphi B	N/A	N/A	Y	Y	Y	N/A	N/A	N/A	N/A	CHL, AMP	45	0	aratyphoid fever
986	忠，于凤太，贾	hong, Yu Feng	守所伤寒暴发调	d fever outbrea	疾病监测	sease surveilla	-	6	2	1996	-	1994	2	7	64	Eastern	Tianjin	Jizhou	39.02	117.12	Prison	72	346	20.81	17	47	N/A	N/A	72	0	100.00	58.33	94.44	55.56	N/A	45.83	81.94	N/A	D1	Typhi	N/A	N/A	Y	N/A	Y	N/A	N/A	N/A	N/A	N/A	72	0	aratyphoid fever
988	，夏伟，陈坚，	Wei, Chen Jia	发生一起伤寒暴	k of typhoid fe	实用预防医学	al preventive m	-	2	2	1996	-	1995	1	3	27	Central	Hunan	Changsha	28.12	112.59	School	61	11305	0.54	18	24	N/A	N/A	57	4	93.44	13.00	100.00	13.00	6.00	6.00	94.00	N/A	D1	Typhi	N/A	SXT	Y	N/A	Y	N/A	N/A	N/A	N/A	N/A	N/A	N/A	aratyphoid fever
989	绍文.梁建中.石	en.Liang Jianz	由洪灾引起的伤	typhoid fever	广西预防医学	xi Preventive M	-	3	2	1996	-	1994	2	2	230	Western	Guangxi	Hezhou	24.36	111.36	Residential area	87	635	13.70	N/A	N/A	N/A	N/A	41	46	47.13	100.00	100.00	100.00	N/A	N/A	100.00	N/A	D1	Typhi	N/A	N/A	Y	N/A	Y	N/A	N/A	N/A	N/A	CHL, NOR	N/A	N/A	aratyphoid fever
990	李中华，潘沫	onghua, Pan M	物型伤寒暴发流	an outbreak o	疾病监测	sease surveilla	-	4	1	1996	-	1994	2	10	36	Central	Hunan	Changsha	28.12	112.59	Residential area	57	3984	1.43	6	70	N/A	N/A	21	36	36.84	33.33	100.00	33.33	28.07	28.07	N/A	N/A	D1	Typhi	N/A	N/A	Y	N/A	Y	N/A	N/A	N/A	N/A	N/A	57	0	: Food poisoning
991	清，吴自林，芮	ng, Wu Zilin, R	水污染引起的副	hoid caused b	广西预防医学	xi Preventive M	-	2	2	1996	-	1995	1	6	70	Western	Guangxi	Liuzhou	23.19	109.24	Residential area	100	2268	4.41	5	60	N/A	N/A	48	52	48.00	100.00	100.00	100.00	100.00	100.00	100.00	N/A	A、C1	typhi A、Paraty	N/A	TET, SPM, LIN	Y	N/A	Y	N/A	N/A	N/A	N/A	N/A	N/A	N/A	aratyphoid fever
992	赵世年，郭夏	Shinian, Guo X	常生活型伤寒暴	break of fly-typ	河南预防医学杂	nal of Preventi	-	1	1	1996	-	1994	2	8	53	Central	Henan	Luoyang	34.41	112.27	Residential area	50	495	10.10	2	78	N/A	N/A	22	28	44.00	100.00	100.00	100.00	N/A	N/A	100.00	N/A	D1	Typhi	N/A	N/A	Y	N/A	Y	N/A	N/A	N/A	N/A	N/A	50	0	aratyphoid fever
994	戴和波	Dai Hebo	村水型伤寒暴发	typhoid fever	实用预防医学	al preventive m	-	4	2	1995	-	1993	2	9	39	Central	Hunan	Yongzhou	26.13	111.37	Residential area	62	754	8.22	6	74	N/A	N/A	28	34	45.16	48.38	100.00	16.12	17.74	17.74	37.09	N/A	D1	Typhi	N/A	N/A	Y	N/A	Y	N/A	N/A	N/A	N/A	CHL	62	0	aratyphoid fever
996	建人.项惠.虞昌	.Xiang Hui.Yu	污染引起伤寒暴	of typhoid feve	浙江预防医学	g Preventive M	-	5	6-7	1995	ki.issn1007-093	1994	1	6	20	Eastern	Zhejiang	Linhai	28.51	121.08	Residential area	26	354	7.34	7	66	N/A	N/A	8	18	30.77	N/A	100.00	60.00	N/A	N/A	N/A	N/A	D1	Typhi	N/A	N/A	Y	N/A	Y	N/A	N/A	N/A	N/A	CHL, NOR	N/A	N/A	aratyphoid fever
999	宁，仁青多杰，	Renqing Duoji	族自治县伤寒暴	hoid fever in H	青海医药杂志	hai Medical Jo	-	7	2	1995	-	1991	4	5	457	Western	Qinghai	Haungnan	34.86	101.57	Residential area	122	26000	0.47	<1	67	N/A	N/A	65	57	53.28	N/A	100.00	N/A	N/A	N/A	52.46	N/A	D1	Typhi	N/A	N/A	Y	N/A	Y	N/A	N/A	N/A	N/A	CHL	120	2	aratyphoid fever
1000	骆云德，谢显	hank you for y	制造厂伤寒暴发	id fever in Sha	实用预防医学	al preventive m	-	2	1	1995	-	1994	1	3	27	Eastern	Guangdong	Shenzhen	22.33	114.07	Factory	16	46	34.78	19	46	N/A	N/A	15	1	93.75	75.00	100.00	100.00	N/A	N/A	100.00	N/A	D1	Typhi	N/A	N/A	Y	N/A	Y	N/A	N/A	N/A	N/A	N/A	N/A	N/A	: Food poisoning
1002	无，焦金鸿，郑	ao Jinhong, Zh	型伤寒暴发流行	outbreak of wa	放军预防医学杂	edicine of the	-	1	2	1995	-	1992	3	7	115	Central	Henan	Zhengzhou	34.46	113.4	Residential area	235	2136	11.00	2	72	N/A	N/A	102	133	43.40	N/A	100.00	N/A	N/A	N/A	N/A	N/A	D1	Typhi	N/A	ERY	Y	N/A	Y	N/A	N/A	N/A	N/A	CHL	235	0	aratyphoid fever
1003	海景杰，曹承来	jie, Cao Cheng	伤寒沙门氏菌食	imurium food	中国公共卫生	nese public he	-	7	302	1994	-	1993	1	9	3	Western	Inner mongolia	Tongliao	43.37	122.16	Restaurant	118	184	64.13	5	55	N/A	N/A	43	75	36.44	100.00	100.00	N/A	100.00	100.00	100.00	Aquatic products	B	Typhimurium	N/A	N/A	Y	Y	Y	N/A	N/A	Y	N/A	N/A	N/A	N/A	Food poisoning
1006	饶浩然，李仁	ao Haoran, Li R	暴发的流行病学	on report on a	湖北预防医学杂	nal of Preventi	-	3	1	1994	-	1993	1	3	27	Central	Hubei	Wuhan	30.35	114.17	Residential area	68	151	45.03	<1	75	N/A	N/A	30	38	44.12	N/A	100.00	100.00	100.00	100.00	100.00	N/A	D1	Typhi	N/A	N/A	Y	N/A	Y	N/A	N/A	N/A	N/A	N/A	67	1	: Food poisoning
1007	王世华，杨秀芸	Shihua, Yang	县一次伤寒暴发	break of typho	卫生管理干部学	Health Manage	-	3	3	1994	-	1987	7	8	28	Western	Sichuan	Liangshan	27.9	102.08	Residential area	28	617	4.54	7	64	N/A	N/A	15	13	53.57	N/A	100.00	N/A	N/A	N/A	100.00	N/A	D1	Typhi	N/A	N/A	Y	N/A	Y	N/A	N/A	N/A	N/A	N/A	28	0	: Food poisoning
1009	李慧玲，王新	Li Huiling, War	专性伤寒暴发的	f an outbreak	中国公共卫生	nese public he	-	8	1	1994	-	1993	1	11	34	Northeastern	Liaoning	Panjin	41.07	122.03	Residential area	98	594	16.50	<1	47	N/A	N/A	51	47	52.04	N/A	90.82	26.53	23.47	20.41	64.29	N/A	D1	Typhi	N/A	N/A	Y	N/A	Y	N/A	N/A	N/A	N/A	N/A	N/A	N/A	aratyphoid fever
1014	陈国忠，吴志明	uozhong, Wu	红工人伤寒暴发	of typhoid fe	苏州医学院学报	Suzhou Medic	-	2	2	1994	-	1992	2	9	89	Eastern	Jiangsu	Suzhou	31.19	120.37	Factory	42	330	12.73	16	19	N/A	N/A	0	42	0.00	N/A	100.00	100.00	N/A	N/A	100.00	N/A	D1	Typhi	N/A	N/A	Y	Y	Y	N/A	N/A	N/A	N/A	N/A	N/A	N/A	aratyphoid fever
1018	启高.周庆礼.刘	.Zhou Qingli.L	寒暴发流行的调	the outbreak	蚌埠医学院学报	Bengbu Medic	-	3	216	1993	-	1992	1	8	71	Central	Anhui	Suzhou	33.38	116.58	Office	26	267	97.38	17	61	N/A	N/A	26	0	100.00	38.46	100.00	57.69	N/A	N/A	N/A	N/A	D1	Typhi	N/A	N/A	Y	N/A	Y	N/A	N/A	N/A	N/A	CHL, TMP, AM	25	1	aratyphoid fever
1020	.傅岳山.郑雪兰	n.Zheng Xuel	发流行调查及预	ever outbreak	预防医学与疾病	Medicine and	-	4	15-17	1993	ki.issn1007-093	1991	2	1	57	Eastern	Zhejiang	Jinhua	29.07	119.39	Factory	49	762	6.43	7	45	N/A	N/A	14	35	28.57	N/A	100.00	N/A	N/A	N/A	N/A	N/A	D1	Typhi	N/A	SM	Y	N/A	Y	N/A	N/A	N/A	Phage infection te	N/A	N/A	N/A	aratyphoid fever
1021	李水芸	Li Shuiyun	一起伤寒暴发	typhoid fever	中国学校卫生	nese school he	-	4	222	1993	-	1991	2	5	12	Central	Hunan	Chenzhou	25.46	113.02	School	538	1051	51.19	N/A	N/A	N/A	N/A	369	169	68.59	N/A	100.00	N/A	N/A	N/A	100.00	N/A	D1	Typhi	N/A	N/A	Y	N/A	Y	N/A	N/A	N/A	N/A	N/A	538	0	aratyphoid fever
1024	刘德兴;	Liu Dexing;	水型伤寒暴发	phoid fever ou	放军预防医学杂	edicine of the	-	2	132-133	1993	-	1990	3	8	26	Northeastern	Jilin	Jilin	43.54	125.19	Military region	55	2605	2.11	1	60	N/A	N/A	35	20	63.64	50.00	84.62	38.46	73.08	42.31	96.15	N/A	D1	Typhi	N/A	N/A	Y	N/A	Y	N/A	N/A	N/A	N/A	AMP, CHL, NO	54	1	ociated infection
1029	罗淦祥;	Luo Ganxiang	寒暴发流行的临	on an outbreal	广州医药	angzhou Medi	-	4	42	1992	-	1990	2	8	31	Eastern	Guangdong	Guangzhou	23.08	113.14	Prison	28	510	5.49	16	39	N/A	N/A	28	0	100.00	42.86	100.00	50.00	35.71	10.71	50.00	N/A	D1	Typhi	N/A	N/A	Y	N/A	Y	N/A	N/A	N/A	N/A	AMP, NOR	28	0	aratyphoid fever
1030	敢齐.李义平.谭	qi.Li Yiping.Tar	引起伤寒暴发流	id outbreak ca	预防医学与疾病	Medicine and	-	3	22-23	1992	ki.issn1007-093	1991	1	8	38	Eastern	Zhejiang	Huzhou	30.52	120.06	Residential area	83	1158	7.17	1	58	N/A	N/A	37	46	44.58	100.00	100.00	100.00	N/A	N/A	100.00	N/A	D1	Typhi	N/A	N/A	Y	N/A	Y	N/A	N/A	N/A	N/A	NOR	N/A	N/A	aratyphoid fever
1031	补团.王义.耿	.Wang Yi.Genc	型伤寒暴发流行	outbreak of wa	放军预防医学杂	edicine of the	-	2	3	1992	-	1989	3	11	8	Western	Gansu	Lanzhou	36.04	103.51	Military region	49	114	43.98	N/A	N/A	N/A	N/A	48	1	97.96	14.29	100.00	24.49	N/A	N/A	97.96	N/A	D1	Typhi	N/A	N/A	Y	N/A	Y	N/A	N/A	N/A	N/A	CHL, SXT	49	0	aratyphoid fever
1037	-	-	扬伤寒暴发流行	enteric typhoi	广东卫生防疫	ealth and Epid	-	4	24-26	1991	-	1989	2	7	43	Eastern	Guangdong	Lianzhou	24.48	112.23	Residential area	40	1265	3.16	2	63	N/A	N/A	23	17	57.50	N/A	100.00	N/A	N/A	N/A	100.00	N/A	D1	Typhi	N/A	N/A	Y	N/A	Y	N/A	N/A	N/A	N/A	N/A	N/A	N/A	aratyphoid fever
1038	-	-	扬伤寒暴发流行	enteric typhoi	广东卫生防疫	ealth and Epid	-	4	24-26	1991	-	1990	1	12	53	Eastern	Guangdong	Luoding	22.46	111.33	Residential area	81	507	15.98	1	74	N/A	N/A	34	47	41.98	100.00	100.00	N/A	100.00	100.00	100.00	N/A	D1	Typhi	N/A	N/A	Y	N/A	Y	N/A	N/A	N/A	N/A	CHL	81	0	aratyphoid fever
1040	树林;陈武泽;王	en Wuze;Wan	队医院伤寒暴发	id fever outbre	人民军医	e's military me	-	7	19-20	1991	-	1988	3	6	41	Central	Anhui	Hefei	31.52	117.17	Military region	25	786	3.18	20	29	N/A	N/A	16	9	64.00	N/A	100.00	N/A	N/A	N/A	100.00	N/A	D1、E1	London、Typhi	N/A	N/A	Y	N/A	Y	N/A	N/A	N/A	N/A	N/A	25	0	aratyphoid fever
1041	;管懋钧;刘惠敏	Maojun;Liu Hui	藏区伤寒暴发流	yphoid fever i	放军预防医学杂	edicine of the	-	3	220	1991	-	1989	2	7	12	Western	Yunnan	Xuanwei	26.13	104.06	Residential area	30	32	93.75	6	68	21.5	N/A	10	20	33.33	66.67	100.00	66.67	N/A	27.78	100.00	N/A	D1	Typhi	N/A	N, ERY, AMP, (	Y	N/A	Y	N/A	N/A	N/A	N/A	N, CHL, SXT, C	30	0	aratyphoid fever
1042	卫.杨祚弥.唐文	.Yang Zuomi.T	水型伤寒暴发调	n outbreak of	重庆医药	ongqing Medi	-	3	186	1991	-	1990	1	5	33	Western	Sichuan	Dazhou	31.21	107.5	Residential area	31	52	59.62	<1	76	N/A	N/A	10	21	32.26	N/A	100.00	N/A	N/A	N/A	100.00	N/A	D1	Typhi	N/A	SXT, AMP	Y	N/A	Y	N/A	N/A	N/A	N/A	N/A	31	0	aratyphoid fever
1043	谢锦梅;刘文生	nmei;Liu Wens	校副伤寒暴发流	tbreak of para	中国学校卫生	nese school he	-	3	164-165	1991	-	1989	2	11	25	Central	Jiangxi	Ganzhou	28.52	114.56	School	39	459	8.50	N/A	N/A	N/A	N/A	36	3	92.31	100.00	100.00	100.00	N/A	N/A	100.00	N/A	B	Paratyphi B	N/A	N/A	Y	Y	Y	N/A	N/A	N/A	N/A	N/A	39	0	aratyphoid fever
1048	陈祖府.吴光财	Zufu.Wu Gua	沾水型伤寒暴发	eak of low-wa	放军预防医学杂	edicine of the	-	4	397-398	1990	-	1985	5	12	45	Western	Guizhou	Qiannan	26.02	107.25	Residential area	69	338	20.41	1	75	N/A	N/A	38	31	55.07	15.94	100.00	N/A	15.94	N/A	100.00	N/A	D1	Typhi	N/A	N/A	Y	N/A	Y	N/A	N/A	N/A	N/A	N/A	69	0	aratyphoid fever
1054	李照坤	Li Zhaokun	肠伤寒暴发的流	n of a waterbo	放军预防医学杂	edicine of the	-	1	59-61	1990	-	1986	4	4	62	Western	Guizhou	Guiyang	26.35	106.42	Hospital	133	815	16.32	4	78	23.4	N/A	83	50	62.41	30.83	100.00	13.31	N/A	N/A	81.20	N/A	D1	Typhi	N/A	N/A	Y	N/A	Y	N/A	N/A	N/A	Phage infection te	N/A	131	2	ociated infection
1059	志明;曾财娣;刘	g;Zeng Caidi;	起伤寒暴发的流	n of a typhoid	广州医药	angzhou Medi	-	6	2-4+6	1987	-	1986	1	1	18	Eastern	Guangdong	Guangzhou	23.08	113.14	School	23	814	2.83	18	26	N/A	N/A	20	3	86.96	14.30	100.00	19.10	23.81	23.81	90.50	N/A	D1	Typhi	N/A	N/A	Y	N/A	Y	N/A	N/A	N/A	N/A	N/A	23	0	aratyphoid fever
1061	;杨金龙;米芳苏	Jinlong;Mi Fa	伤寒暴发流行的	an outbreak c	河南预防医学杂	nal of Preventi	-	4	34-36	1987	-	1986	1	12	112	Central	Henan	Xinyang	32.07	114.04	Residential area	76	26456	0.29	1	63	N/A	N/A	39	37	51.32	N/A	100.00	N/A	N/A	N/A	N/A	N/A	D1	Typhi	N/A	N/A	Y	N/A	Y	N/A	N/A	N/A	N/A	CHL, AMP	76	0	aratyphoid fever
1064	俏;林永健;罗平	ongjian;Luo Pi	寒暴发的流行病	port on an outl	右江医学	ujiang Medic	-	1	1-4	1985	-	1984	1	7	87	Western	Guangxi	Baise	23.54	106.36	Residential area	98	183	53.55	1	73	N/A	N/A	44	54	44.90	N/A	100.00	100.00	N/A	N/A	100.00	N/A	D1	Typhi	N/A	N/A	N/A	N/A	Y	N/A	N/A	N/A	N/A	CHL	96	2	aratyphoid fever
1066	龙献;赵九现;曲	an;Zhao Jiuxia	村伤寒暴发流行	of typhoid feve	河南预防医学杂	nal of Preventi	-	3	21-24	1984	-	1983	1	8	109	Central	Henan	Luoyang	34.41	112.27	Residential area	72	388	18.56	1	65	N/A	N/A	43	29	59.72	59.72	100.00	47.22	30.56	26.39	72.22	N/A	D1	Typhi	N/A	CHL	Y	N/A	Y	N/A	N/A	N/A	N/A	CHL, GEN, SXT	69	3	aratyphoid fever
1068	静君;蒋国伟;陈	Jiang Guowei;	鼠伤寒暴发流行	yphoid fever o	贵州医药	uizhou Medici	-	1	2	1983	-	1982	1	10	21	Western	Guizhou	Guiyang	26.35	106.42	Hospital	65	137	47.45	<1	<1	<1	<1	36	29	55.38	100.00	100.00	N/A	N/A	N/A	N/A	N/A	B	Typhimurium	N/A	N/A	Y	N/A	Y	N/A	N/A	N/A	N/A	N/A	53	12	ociated infection
1073	顾清礼;	Gu Qingli;	染引起的一次家	mily caused by	宁夏医学院学报	Ningxia Medi	-	2	6-7	1980	-	1978	2	12	5	Western	Ningxia	Wuzhong	37.59	106.11	Residential area	8	8	100.00	<1	31	11.55	11.37	3	5	37.50	N/A	100.00	100.00	N/A	N/A	100.00	N/A	D1	Typhi	N/A	N/A	Y	N/A	Y	N/A	N/A	N/A	N/A	CHL, TMP	8	0	ociated infection
1075	刘立峰;	Liu Lifeng;	例伤寒暴发的调	igation report	河南预防医学杂	nal of Preventi	-	3	61-64	1979	-	1978	1	6	41	Central	Henan	Anyang	36.06	114.21	Residential area	104	3400	3.06	2	69	N/A	N/A	42	62	40.38	12.50	100.00	41.67	N/A	N/A	100.00	N/A	D1	Typhi	N/A	N/A	Y	N/A	Y	N/A	N/A	N/A	N/A	CHL	103	1	aratyphoid fever
1076	-	-	×厂伤寒暴发的	report on typh	重庆医药	ongqing Medi	-	5	8-10+20	1977	-	1976	1	10	60	Western	Chongqing	Jiangbei	29.36	105.02	Factory	53	649	8.17	3	54	N/A	N/A	29	24	54.72	32.07	100.00	16.98	N/A	N/A	43.39	N/A	D1	Typhi	N/A	N/A	Y	N/A	Y	N/A	N/A	N/A	N/A	CHL, SOX	53	0	aratyphoid fever
1081	玲玲;王乐;汪洋	Wang Le;Wang	菌食源性疾病事	eak of Salmone	实用预防医学	al preventive m	30	1	84-87	2023	-	2021	2	6	2	Western	Sichuan	Mianyang	31.3	104.42	School	198	395	50.13	16	18	N/A	N/A	79	119	39.90	94.40	87.88	66.16	56.06	33.33	N/A	Pastries	D1	Enteritidis	N/A	N/A	Y	Y	Y	Y	N/A	N/A	N/A	N/A	198	0	Food poisoning
1085	飞;喻瑾;罗禹;肖	;Yu Jin;Luo Yu	氏菌引起的食源	utbreak caused	中国食品卫生杂	Food Hygiene	-	-	9	2021	-	2021	0	5	3	Western	Chongqing	Jiulongpo	29.35	106.33	Office	55	73	75.34	6	79	N/A	N/A	37	18	67.27	89.09	43.64	78.18	N/A	29.09	30.90	Pastries	D1	Enteritidis	N/A	N/A	Y	Y	Y	N/A	N/A	N/A	N/A	N/A	N/A	N/A	Food poisoning
1092	冬花;汪生虎;沈	Wang Shengh	氏菌感染引起的	pected to be c	疾病预防控制通	vention and Co	37	4	53-55	2022	5/j.cnki.jbyfkztb.	2020	2	10	3	Western	Gansu	Dingxi	35.13	104.45	Residential area	35	230	15.22	7	70	51	N/A	25	10	71.43	100.00	77.10	97.10	88.60	87.10	N/A	Meat products	D1	Enteritidis	N/A	N/A	Y	Y	Y	N/A	N/A	N/A	N/A	N/A	35	0	Food poisoning
1099	孙中兴;蒋元强;	;Jiang Yuanqi	两种沙门氏菌引	used by Vibric	实用预防医学	al preventive m	29	3	350-352	2022	-	2020	2	5	2	Eastern	Shanghai	Songjiang	31.14	121.29	Restaurant	7	70	10.00	23	86	N/A	N/A	5	2	71.43	100.00	N/A	100.00	42.86	52.86	N/A	Aquatic products	B、E1	ndon、Typhimur	N/A	N/A	Y	Y	Y	Y	N/A	N/A	N/A	N/A	N/A	N/A	Food poisoning
1104	鸿;张宇;杨秀程;	hang Yu;Yang	门菌所致食物中	tigation on fo	预防医学情报杂	ventive Medici	37	12	1660-1663	2021	-	2020	1	9	3	Western	Sichuan	Barkam	32.01	102.02	Residential area	29	172	16.86	3	79	43	N/A	13	16	44.83	72.41	68.97	93.10	55.17	27.59	N/A	Meat products	N/A	N/A	N/A	N/A	Y	Y	N/A	N/A	N/A	N/A	N/A	N/A	28	0	Food poisoning
1105	成;刘翔;郝琼;田	u Xiang;Hao C	食物中毒事件的	sis of a food p	疾病预防控制通	vention and Co	36	6	30-33+73	2021	5/j.cnki.jbyfkztb.	2020	1	9	3	Western	Ningxia	N/A	38.27	106.16	Residential area	13	23	56.52	10	72	N/A	N/A	6	7	46.15	84.62	84.62	100.00	76.92	30.77	54.55	Meat products	B	phimurium 4,5,12	N/A	NAL, CHL, CT	Y	Y	Y	Y	N/A	N/A	N/A	N/A	12	1	Food poisoning
1106	唐琳[1]	Tang Lin[1]	菌污染的皮蛋引	by eating pre	数据库（文摘	nal Database (	-	6	205-207	2022	-	2021	1	6	2	Western	Sichuan	Yibin	28.47	104.34	Residential area	6	18	33.33	N/A	N/A	N/A	N/A	1	5	16.67	100.00	66.67	100.00	50.00	16.67	N/A	Egg products	B	Typhimurium	N/A	N/A	Y	Y	Y	N/A	N/A	N/A	N/A	N/A	6	0	Food poisoning
1112	[1];崔立周[1];祖	;Cui Lizhou[1	肠炎沙门菌食物	Enteritidis foo	医学动物防制	lical animal co	37	10	1000-1003	2021	-	2019	2	5	3	Eastern	Hebei	Baoding	38.51	115.3	Restaurant	80	248	32.26	2	81	35	N/A	48	32	60.00	77.50	96.25	68.75	N/A	25.00	N/A	Aquatic products	D1	Enteritidis	N/A	N/A	Y	Y	Y	Y	Y	N/A	N/A	N/A	80	0	Food poisoning
1113	YS, Chiou CS, C	-	ench toast sandw	-	Epidemiol Infect	-	-	-	-	2020	17/S0950268820	2018	2	4	10	Eastern	Taiwan	N/A	25.03	121.3	Restaurant	47	98	47.96	1	68	N/A	N/A	32	15	68.09	100.00	68.09	65.96	N/A	N/A	N/A	Egg products	D1	Enteritidis	N/A	N/A	Y	N/A	Y	Y	N/A	N/A	WGS	N/A	46	1	Food poisoning
1114	AS, Liao YS, Li	-	aminated with m	-	odborne Pathog	-	-	-	-	2014	.1089/fpd.2013.1	2010	4	7	4	Eastern	Taiwan	N/A	25.03	121.3	mobile street stal	324	886	36.57	1	85	N/A	N/A	134	190	41.36	89.20	47.53	69.75	N/A	17.28	32.72	Pastries	C1、D1	nteritidis、Virch	N/A	N/A	Y	N/A	Y	Y	N/A	N/A	N/A	N/A	324	0	Food poisoning
1115	ung YL, Hwang	-	caused by Salmo	-	BMC Infect Dis	-	-	-	-	2004	1186/1471-2334-	2001	3	7	2	Eastern	Taiwan	N/A	25.03	121.3	mobile street stal	28	34	82.35	3	71	N/A	N/A	12	16	42.86	100.00	100.00	100.00	N/A	85.71	7.14	Pastries	D1	Enteritidis	N/A	N/A	Y	N/A	Y	Y	N/A	N/A	N/A	N/A	N/A	N/A	Food poisoning
1117	ng C, Liu H, Du	-	a Serovar Enterit	-	Front Microbiol	-	-	-	-	2021	89/fmicb.2021.7	2021	0	3	3	Eastern	Beijing	N/A	39.55	116.24	Office	225	324	69.44	N/A	N/A	24.1	N/A	223	2	99.11	100.00	100.00	63.11	51.11	63.56	68.44	Egg products	D1	Enteritidis	N/A	, SOX, NAL, TE	Y	Y	Y	Y	Y	N/A	WGS	N/A	N/A	N/A	Food poisoning
1119	hou X, Xu X, Y	-	used by Multidru	-	Front Microbiol	-	-	-	-	2020	389/fmicb.2020.(	2017	3	5	3	Central	Shanxi	Changzhi	36.11	113.06	Residential area	11	12	91.67	1	50	N/A	N/A	4	7	36.36	90.91	81.82	54.55	45.45	36.36	36.36	N/A	B	Typhimurium	N/A	MP, NAL, SDI, S	Y	Y	Y	Y	N/A	N/A	WGS	N/A	11	0	Food poisoning
1123	Zhang HN, Jiang	-	erotype Isangi Iso	-	odborne Pathog	-	-	-	-	2017	.1089/fpd.2016.2	2016	1	4	4	Eastern	Shandong	Yantai	37.32	121.24	Residential area	33	74	44.59	27	77	N/A	N/A	16	17	48.48	93.90	57.60	87.90	66.67	30.30	24.30	Meat products	C1	Isangi	N/A	N/A	Y	N/A	Y	Y	N/A	N/A	WGS	N/A	33	0	Food poisoning
1125	, Li L, Li Z, Che	-	a contaminated f	-	Epidemiol Infect	-	-	-	-	2016	71/journal.pone.0	2013	3	6	4	Western	Sichuan	Meishan	29.36	103.29	School	399	1268	31.47	5	17	11.3	0.46	262	137	65.66	56.00	95.00	38.00	22.00	N/A	51.00	Meat products	B	Typhimurium	N/A	N/A	Y	N/A	Y	Y	N/A	N/A	N/A	CAZ, ATM	399	0	Food poisoning
1126	, Su Ch, Huang J	-	ent Salmonella se	-	Jpn J Infect Dis	-	-	-	-	2015	/INF.000000000	2012	3	10	4	Eastern	Fujian	Xiamen	24.27	118.06	School	56	22404	0.25	17	22	N/A	N/A	20	36	35.71	N/A	84.00	89.00	80.00	43.00	N/A	Pastries	B、D1	hester、Enteritic	N/A	N/A	Y	N/A	Y	Y	Y	N/A	N/A	N/A	56	0	Food poisoning
1127	ML, Yang J, Wu	-	school: a comm	-	Epidemiol Infect	-	-	-	-	2010	17/S0950268810	2004	6	11	14	Western	Guangxi	Guilin	25.17	110.17	School	394	1092	36.08	N/A	N/A	N/A	N/A	203	191	51.52	28.00	15.00	12.16	N/A	N/A	59.00	N/A	A	Paratyphi A	N/A	NAL	Y	N/A	Y	N/A	N/A	N/A	N/A	CTX, AMK, OF	394	0	aratyphoid fever
1128	HM,FontaineR;C	-	workers--Huizho	-	MMWRSuppl	-	-	-	-	2006	-	2004	2	7	6	Eastern	Guangdong	Huizhou	23.05	114.22	Factory	199	1496	13.30	17	50	N/A	N/A	154	55	77.39	87.00	66.00	78.00	28.00	18.00	N/A	Pastries	D1	Enteritidis	N/A	N/A	Y	N/A	Y	N/A	N/A	N/A	N/A	N/A	N/A	N/A	Food poisoning
1131	G, Liu B, Lin C,	-	by Waterborne	-	China CDC Wkl	-	-	-	-	2022	46234/ccdcw2022	2022	0	1	35	Eastern	Beijing	N/A	39.55	116.24	Residential area	23	106	21.70	22	45	N/A	N/A	8	15	34.78	47.83	82.61	100.00	100.00	100.00	100.00	N/A	D1	Typhi	N/A	CHL, SXT, AT	Y	N/A	Y	N/A	N/A	N/A	WGS	MEM	N/A	N/A	aratyphoid fever
1132	Ruan F, Li Z, Hu	-	tion in a tourist g	-	J Infect	-	-	-	-	2022	016/j.jinf.2022.0	2019	3	6	1	Eastern	Hong kong	N/A	21.23	115.12	Restaurant	6	18	33.33	42	62	N/A	N/A	3	3	50.00	100.00	100.00	83.33	100.00	N/A	66.67	Meat products	I	Hvittingfoss	N/A	N/A	Y	N/A	Y	Y	N/A	N/A	N/A	N/A	N/A	N/A	Food poisoning

Sheet3: Reference standard		
**Objective**	**Reference**	**Weblink**
**Antimicrobial category**	Clinical and Laboratory Standards Institute M100-ED33	http://em100.edaptivedocs.net
**Designated symptoms**	U.S. Department of Health & Human Services	https://www.cdc.gov/salmonella/general/salmonella-symptoms.html
**Economic region**	National Bureau of Statistics of China	http://www.stats.gov.cn/zt_18555/zthd/sjtjr/dejtjkfr/tjkp/202302/t20230216_1909741.htm
**Food category**	China Food Production Licence Classification Catalogue	https://www.gov.cn/zhengce/zhengceku/2020-03/27/content_5496236.htm
	FDA Product Categories and Products	https://www.fda.gov/product-categories-and-products

Sheet4: Antimicrobial annotation		
**Chinese name**	**English name**	**Abbreviation**
阿莫西林	Amoxicillin	AMX
阿莫西林-克拉维酸钾	Amoxicillin and clavulanate potassium	AMC
氨苄西林-克拉维酸	Ampicillin - clavulanic acid	AMC
替卡西林-克拉维酸	Ticarcillin-clavulnic acid	TCC
氨苄西林	Ampicillin	AMP
氨苄西林-舒巴坦	Ampicillin and sulbactam sodium	SAM
哌拉西林	Piperacillin	PIP
哌拉西林-他唑巴坦	Piperacillin and tazobactam sodium	TZP
羧苄西林	Carbenicillin	SRB
苯唑西林	Oxacillin	OXA
替卡西林	Ticarcillin	TLC
美洛西林	Mezlocillin	MEZ
邻氯西林	Cloxacillin	CLO
奈替米星	Netilmicin	NET
阿米卡星	Amikacin	AMK
环丙沙星	Ciprofloxacin	CIP
诺氟沙星	Norfloxacin	NOR
氧氟沙星	Ofloxacin	OFX
左氧氟沙星	Levofloxacin	LVX
四环素	Tetracycline	TET
米诺环素	Minocycline	MIN
多西环素	Doxycycline	DOX
替加环素	Tigecycline	TGC
青霉素	Penicillin	PEN
链霉素	Streptomycin	SM
氯霉素	Chloramphenicol	CHL
红霉素	Erythromycin	ERY
新霉素	Neomycin	NEO
土霉素	Oxytetracycline	OXY
卡那霉素	Kanamycin	KAN
阿奇霉素	Azithromycin	AZI
林可霉素	Lincomycin	LIN
克林霉素	Clindamycin	CLI
庆大霉素	Gentamicin	GEN
麦迪霉素	Midecamycin	MID
美满霉素	Minocycline	MIN
妥布霉素	Tobramycin	TOB
万古霉素	Vancomycin	VAN
吉他霉素	Kitasamycin	KIT
新生霉素	Novobiocin	NOV
交沙霉素	Josamycin	JOS
头孢曲松	Ceftriaxone	CRO
头孢拉定	Cefradine	CED
头孢氨苄	Cephalexin	LEX
头孢克洛	Cefaclor	CEC
头孢噻吩	Cefalotin	CEP
头孢噻啶	Cefaloridine	RID
头孢哌酮	Cefoperazone	CFP
头孢曲松	Ceftriaxone	CRO
头孢呋辛	Cefuroxime	CXM
头孢美唑	Cefmetazole	CMZ
头孢替安	Cefotiam	CTF
头孢他啶	Ceftazidime	CAZ
头孢西丁	Cefoxitin	FOX

头孢唑啉	Cefazolin	CZO
头孢替坦	Cefotetan	CTT
头孢吡肟	Cefepime	FEP
头孢噻肟	Cefotaxime	CTX
呋喃唑酮	Furazolidone	FRZ
呋喃妥因	Nitrofurantoin	NIT
萘啶酸	Nalidixic acid	NAL
亚胺培南	Imipenem	IMP
厄他培南	Ertapenem	ETP
氨曲南	Aztreonam	ATM
磺胺嘧啶	Sulfadiazine	SDI
磺胺异恶唑	Sulfisoxazole	SOX
磺胺甲恶唑	Sulfamethoxazole	SMZ
复方新诺明	Trimethoprim/sulphamethoxazole	SXT
氟苯尼考	Florfenicol	FF
乙酰螺旋霉素	Acetylspiramycin	SPM
甲硝唑	Metronidazole	MTR
吡哌酸	Pipemidic acid	PPA
杆菌肽	Bacitracin	BAC
多粘菌素B	Polymyxin B	POL-B
