# Supplementary material for: Nationwide trends and features of human salmonellosis outbreaks in China
Source: Emerg Microbes Infect. 2024 Jun 26;13(1):2372364. doi: 10.1080/22221751.2024.2372364 (PMC11259058; doi:10.1080/22221751.2024.2372364)
Supplement: Supplemental Material [file TEMI_A_2372364_SM2456.pdf]

**Supplementary Table 3. Checklist for evaluating epidemiological studies of *Salmonella* outbreaks in China from 1949 to 2022 with high-quality evidence.**

| Criterion | Option | Score |
|---|---|---|
| **1. Event overview:** An outbreak investigation was conducted on all suspected cases within specific spatiotemporal boundaries, with the precise time and location reported on an independent basis. | **A.** Yes<br>**B.** Unclear | 2<br>1 |
| **2. Study population*:** An accurate description of the new case population, including number, age range, etc. | **A.** Yes<br>**B.** Unclear | 2<br>1 |
| **3. Identification method:** Definitive laboratory results confirm that the outbreak was caused by *Salmonella* infection and spread. | **A.** Yes<br>**B.** Unclear | 2<br>1 |
| **4. Description of statistical analysis:** The epidemiological investigation data were collected, organized, and subjected to statistical analysis to calculate the attack rates and gender ratios. | **A.** Yes<br>**B.** Unclear | 2<br>1 |
| **5. Clinical information:** The number of affected individuals presenting with characteristic symptoms was recorded to calculate the incidence rate. | **A.** Yes<br>**B.** Unclear | 2<br>1 |

* Studies with small sample sizes were included if the suspected cases were reported during the outbreak investigation within a well-defined timeframe. We used the binary random-effects model (SJ: Sidik-Jonkman) for the meta-analysis because the heterogeneity may lead to higher weights and wider confidence intervals for the studies with smaller sample sizes.

Method of scoring and assessment of the risk of bias and study quality.

| Calculation (No. of criteria × score) | Total score | Risk of bias | Quality of study |
|---|---|---|---|
| (5 × 2) + (0 × 1) | 10 | Low | High |
| (4 × 2) + (1 × 1) | 9 | Low | High |
| (3 × 2) + (2 × 1) | 8 | Moderate | Moderate |
| (2 × 2 ) + (3 × 1) | 7 | Moderate | Moderate |
| (1 × 2 ) + (4 × 1) | 6 | High | Low |
| (0 × 2 ) + (5 × 1) | 5 | High | Low |
