# Supplementary material for: Nationwide trends and features of human salmonellosis outbreaks in China
Source: Emerg Microbes Infect. 2024 Jun 26;13(1):2372364. doi: 10.1080/22221751.2024.2372364 (PMC11259058; doi:10.1080/22221751.2024.2372364)
Supplement: Supplemental Material [file TEMI_A_2372364_SM2452.pdf]

Table S4: Public datasets and reference sources utilized for correlation analysis
Sheet1: Sources of reference public data
Sheet2: Year-related public data
Sheet3: Month-related public data
Sheet4: Province-related public data

Table S4: Public datasets and reference sources utilized for correlation analysis				
	**Data name**	**Source**	**Weblink**	**Explanatory note**
**(1)**	Total population (TP) / 10^4 person	China Statistical Yearbook	http://www.stats.gov.cn/sj/ndsj/2022/indexch.htm	-
**(2)**	Rate of natural increase (RNI) / %	China Statistical Yearbook	http://www.stats.gov.cn/sj/ndsj/2022/indexch.htm	-
**(3)**	Urbanization rate (UR) / %	China Statistical Yearbook	http://www.stats.gov.cn/sj/ndsj/2022/indexch.htm	-
**(4)**	Gross domestic product (GDP) / 10^8 CNY	China Statistical Yearbook	http://www.stats.gov.cn/sj/ndsj/2022/indexch.htm	-
**(5)**	Proportion of primary industry (PPI) / %	China Statistical Yearbook	http://www.stats.gov.cn/sj/ndsj/2022/indexch.htm	-
**(6)**	Proportion of secondary industry (PSI) / %	China Statistical Yearbook	http://www.stats.gov.cn/sj/ndsj/2022/indexch.htm	-
**(7)**	Proportion of tertiary industry (PTI) / %	China Statistical Yearbook	http://www.stats.gov.cn/sj/ndsj/2022/indexch.htm	-
**(8)**	Total value of imports (TVI) / 10^8 CNY	China Statistical Yearbook	http://www.stats.gov.cn/sj/ndsj/2022/indexch.htm	-
**(9)**	Resident consumption level (RCL) / CNY	China Statistical Yearbook	http://www.stats.gov.cn/sj/ndsj/2022/indexch.htm	-
**(10)**	Temperature (T, nationwide annual averages) / ℃	China Climate Bulletin	https://www.cma.gov.cn/zfxxgk/gknr/qxbg	-
**(11)**	Precipitation (P, nationwide annual averages) / mm	China Climate Bulletin	https://www.cma.gov.cn/zfxxgk/gknr/qxbg	-
**(12)**	Temperature (T, nationwide monthly averages) / ℃	China Statistical Yearbook	http://www.stats.gov.cn/sj/ndsj/2022/indexch.htm	-
**(13)**	Relative humidity (RH, nationwide monthly averages) / %	China Statistical Yearbook	http://www.stats.gov.cn/sj/ndsj/2022/indexch.htm	-
**(14)**	Precipitation (P, nationwide monthly averages) / mm	China Statistical Yearbook	http://www.stats.gov.cn/sj/ndsj/2022/indexch.htm	-
**(15)**	Gross domestic product (GDP, 2021) / 10^8 CNY	China Statistical Yearbook	http://www.stats.gov.cn/sj/ndsj/2022/indexch.htm	-
**(16)**	Total population (TP, 2021) / 10^4 person	China Statistical Yearbook	http://www.stats.gov.cn/sj/ndsj/2022/indexch.htm	-
**(17)**	Mean years of schooling (MYS) / year	the Seventh National Population Census	http://www.stats.gov.cn/sj/pcsj/rkpc/7rp/indexch.htm	UNESCO (Hong Kong and Macau, http://uis.unesco.org)
**(18)**	Licensed physician ratio (LPR, 2021) / ‰	China Statistical Yearbook	http://www.stats.gov.cn/sj/ndsj/2022/indexch.htm	-
**(19)**	Human development index (HDI, 2019)	United Nations Development Programme	https://www.undp.org/zh/china	Global Data Lab (proofreader, https://globaldatalab.org)
**(20)**	Mean latitude (ML) / °N	Latitude and Longitude Finder	https://www.latlong.net	-
**(21)**	Temperature (T, cumulative annual averages by province) / ℃	National Meteorological Information Centre	https://data.cma.cn/data/weatherBk.html	Central Weather Bureau (Taiwan, https://npd.cwb.gov.tw),<br>Hong Kong Observatory (Hong Kong, https://wap.hko.gov.hk/tc/index.html),<br>and Macao Meteorological and Geophysical Bureau (Macau, https://www.smg.gov.mo)
**(22)**	Precipitation (P, cumulative annual averages by province) / mm	National Meteorological Information Centre	https://data.cma.cn/data/weatherBk.html	Central Weather Bureau (Taiwan, https://npd.cwb.gov.tw),<br>Hong Kong Observatory (Hong Kong, https://wap.hko.gov.hk/tc/index.html),<br>and Macao Meteorological and Geophysical Bureau (Macau, https://www.smg.gov.mo)

Sheet2: Year-related public data

Year	(1) Total population (TP) / 10^4 person	(2) Rate of natural increase (RNI) / %	(3) Urbanization rate (UR) / %	(4) Gross domestic product (GDP) / 10^8 CNY	(5) Proportion of primary industry (PPI) / %	(6) Proportion of secondary industry (PSI) / %	(7) Proportion of tertiary industry (PTI) / %	(8) Total value of imports (TVI) / 10^8 CNY	(9) Resident consumption level (RCL) / CNY	(10) Temperature (T, nationwide annual averages) / ℃	(11) Precipitation (P, nationwide annual averages) / mm
**2022**	141175	-0.6	0.652176377	1210207	0.07299998	0.399241	0.527759	181024	-	10.51	606.1
**2021**	141260	0.34	0.647210817	1149237	0.07241022	0.392908	0.534682	173634.3	31013	10.53	672.1
**2020**	141212	1.45	0.638897544	1013567	0.07698643	0.378428	0.544585	142936.4	27439	10.25	694.8
**2019**	141008	3.32	0.627099172	986515.2	0.07143691	0.385874	0.542689	143253.7	27504	10.34	645.5
**2018**	140541	3.78	0.615002028	919281.1	0.07043025	0.39687	0.5327	140881.3	25245	10.09	673.8
**2017**	140011	5.58	0.602402668	832035.9	0.0746356	0.398517	0.526847	124789.8	22968	10.39	641.3
**2016**	139232	6.53	0.588399219	746395.1	0.08057288	0.395806	0.523621	104967.2	20801	10.36	730
**2015**	138326	4.93	0.573297862	688858.2	0.08387009	0.408413	0.507717	104336.1	18857	10.5	648.8
**2014**	137646	6.71	0.557502579	643563.1	0.08643488	0.430856	0.482709	120358	17220	10.11	636.2
**2013**	136726	5.9	0.544900019	592963.2	0.08942899	0.441767	0.468804	121037.5	15586	10.17	653.5
**2012**	135922	7.43	0.531003075	538580	0.09113706	0.45423	0.454633	114801	14074	9.42	669.3
**2011**	134916	6.13	0.518300276	487940.2	0.09177662	0.465293	0.442931	113161.4	12668	9.3	556.8
**2010**	134091	4.79	0.499496611	412119.3	0.09325164	0.464978	0.44177	94699.5	10575	9.5	681
**2009**	133450	4.87	0.48341701	348517.7	0.09636182	0.459571	0.444067	68618.37	9249	9.9	574
**2008**	132802	5.08	0.469895032	319244.6	0.10169037	0.469712	0.428598	79526.53	8504	9.7	649.8
**2007**	132129	5.17	0.458892446	270092.3	0.10246164	0.468842	0.428697	73296.93	7454	10.1	607.9
**2006**	131448	5.28	0.443430102	219438.5	0.10625756	0.475574	0.418168	63376.86	6319	10	596.7
**2005**	130756	5.89	0.429899966	187318.9	0.11641484	0.470226	0.413359	54273.68	5688	9.76	625.6
**2004**	129988	5.87	0.417600086	161840.2	0.1291663	0.459002	0.411832	46435.76	5071	9.96	601
**2003**	129227	6.01	0.405302298	137422	0.12348969	0.456228	0.420282	34195.56	4555	9.9	637.4
**2002**	128453	6.45	0.390897838	121717.4	0.13301467	0.444506	0.422479	24430.27	4270	10.04	660
**2001**	127627	6.95	0.376597428	110863.1	0.13983462	0.447934	0.412231	20159.18	3968	9.91	603.5
**2000**	126743	7.58	0.362197518	100280.1	0.14676292	0.455362	0.397877	18638.81	3712	9.53	625.6
**1999**	125786	8.18	0.347797052	90564.4	0.16064811	0.453599	0.385753	13736.46	3340	10.09	631.2
**1998**	124761	9.14	0.333501655	85195.5	0.17159005	0.457976	0.370434	11626.14	3122	10.28	713.2
**1997**	123626	10.06	0.319099542	79715	0.17895252	0.47099	0.350057	11806.56	2974	9.62	609.9
**1996**	122389	10.42	0.304798634	71813.6	0.19325448	0.471043	0.335702	11557.43	2763	9.11	639.1
**1995**	121121	10.55	0.290403811	61339.9	0.19596543	0.467505	0.33653	11048.13	2329	9.47	628.6
**1994**	119850	11.21	0.285098039	48637.5	0.19474274	0.461629	0.343626	9960.06	1799	9.71	649.5
**1993**	118517	11.45	0.279900774	35673.2	0.19307491	0.461767	0.345161	5986.21	1332	9.08	656
**1992**	117171	11.6	0.274598663	27194.5	0.21328945	0.431153	0.355557	4443.33	1057	9.04	603.5
**1991**	115823	12.98	0.26940245	22005.6	0.24033882	0.414876	0.344785	3398.65	916	9.36	622.7
**1990**	114333	14.39	0.26409698	18872.9	0.2658415	0.410329	0.323829	2574.28	831	9.64	675.2
**1989**	112704	15.04	0.262102499	17179.7	0.24611606	0.424961	0.328923	2199.86	785	9.33	605.3
**1988**	111026	15.73	0.25814674	15180.4	0.25237807	0.435245	0.312376	2055.07	684	9.21	616.7
**1987**	109300	16.61	0.253193047	12174.6	0.26321193	0.433181	0.303608	1614.21	558	9.29	645.7
**1986**	107507	15.57	0.245249147	10376.2	0.26638847	0.43514	0.298472	1498.26	496	8.91	569.5
**1985**	105851	14.26	0.237069088	9098.9	0.27934146	0.427129	0.29353	1257.85	440	8.76	647.7
**1984**	104357	13.08	0.230142683	7278.5	0.31539466	0.429305	0.2553	620.47	356	8.52	630
**1983**	103008	13.29	0.216235632	6020.9	0.32568221	0.442293	0.232042	421.82	315	9.07	674
**1982**	101654	15.68	0.211305015	5373.4	0.32785573	0.446198	0.225928	357.54	284	9.23	602.3
**1981**	100072	14.55	0.201564873	4935.8	0.31316099	0.459703	0.227136	367.73	264	8.97	631.5
**1980**	98705	11.87	0.193911149	4587.6	0.29634231	0.480578	0.22308	298.8	238	8.94	626.5
**1979**	97542	11.61	0.189610629	4100.5	0.30703573	0.469528	0.223412	242.9	208	9.07	607.9
**1978**	96259	12	0.179152079	3678.7	0.27686411	0.477098	0.246038	187.39	184	9.07	588.5
**1977**	94974	12.12	0.175511193	3250	0.28990769	0.467015	0.243108	132.8	175	8.82	624.8
**1976**	93717	12.72	0.174365377	2988.6	0.32359633	0.450378	0.226059	129.3	171	8.36	606.7
**1975**	92420	15.77	0.173447306	3039.5	0.31952624	0.453594	0.226879	147.4	167	9.09	647.4
**1974**	90859	17.57	0.171639573	2827.7	0.3342646	0.424302	0.241433	152.8	163	8.62	618.5
**1973**	89211	20.99	0.172007936	2756.2	0.32925767	0.428235	0.242508	103.6	162	9.13	691.3
**1972**	87177	22.27	0.171318123	2552.4	0.32416549	0.427676	0.248119	64	155	8.74	591.1
**1971**	85229	23.4	0.172605569	2456.9	0.33631812	0.419187	0.244454	52.4	150	8.77	599
**1970**	82992	25.95	0.173799884	2279.7	0.34798438	0.402728	0.249287	56.1	147	8.53	656
**1969**	80671	26.19	0.174994732	1962.2	0.37519111	0.354194	0.270615	47.2	142	8.38	624.4
**1968**	78534	27.5	0.176203937	1744.1	0.41643254	0.311106	0.272519	50.9	139	8.55	601.3
**1967**	76368	25.65	0.177404148	1794.2	0.39806042	0.33887	0.26307	53.4	143	8.31	609.9
**1966**	74542	26.34	0.178597301	1888.7	0.37179012	0.378779	0.249431	61.1	139	8.86	585.2
**1965**	72538	28.5	0.179836775	1734	0.3754902	0.350923	0.273587	55.3	133	8.8	593.2
**1964**	70499	27.78	0.183690549	1469.9	0.38029798	0.353289	0.266413	42.1	127	8.7	672.5
**1963**	69172	33.5	0.168362921	1248.3	0.39854202	0.33069	0.270688	35.7	124	9.02	601.1
**1962**	67296	27.14	0.173249524	1162.2	0.38986405	0.313113	0.296937	33.8	126	8.77	615.3
**1961**	65859	3.8	0.192942498	1232.3	0.35794855	0.319322	0.32273	43	124	9.12	669.2
**1960**	66207	-4.57	0.197456462	1470.1	0.23175294	0.443915	0.324332	65.1	111	8.83	620.3
**1959**	67207	10.19	0.184073088	1447.5	0.2651468	0.426045	0.308808	71.2	104	8.77	667.8
**1958**	65994	17.24	0.162454162	1312.3	0.33978511	0.368513	0.291702	61.7	111	8.68	602.5
**1957**	64653	23.23	0.153883037	1071.4	0.40134404	0.295501	0.303155	50	108	8.26	605.3
**1956**	62828	20.5	0.14619278	1030.7	0.43067818	0.272048	0.297371	53	104	8.56	620.8
**1955**	61465	20.32	0.134792158	911.6	0.46182536	0.242979	0.295195	61.1	99	9.2	604.1
**1954**	60266	24.79	0.136876514	859.8	0.45591998	0.245173	0.298907	44.7	92	8.95	720
**1953**	58796	23	0.133104293	824.4	0.45851528	0.232411	0.309073	46.1	91	8.02	569
**1952**	57482	20	0.124612922	679.1	0.504933	0.207775	0.287292	37.5	80	7.9	549.2
**1951**	56300	20	0.117797513	-	-	-	-	35.3	-	8.26	544.8
**1950**	55196	19	0.111765345	-	-	-	-	21.3	-	-	-
**1949**	54167	16	0.106430114	-	-	-	-	-	-	-	-

Sheet3: Month-related public data			
	(12)	(13)	(14)
Month	Temperature (T, nationwide monthly averages) / ℃	Relative humidity (RH, nationwide monthly averages) / %	Precipitation (P, nationwide monthly averages) / mm
1	1.041176471	56.91176471	8.308823529
2	6.882352941	60.64705882	32.41764706
3	10.77352941	64.32352941	53.09411765
4	19.00588235	62.17647059	59.75294118
5	20.60588235	62.02941176	121.1411765
6	24.43529412	67.47058824	119.1058824
7	26.71176471	72.88235294	211.0264706
8	25.09705882	74.85294118	174.6941176
9	22.86764706	74.23529412	117.2323529
10	15.22058824	71.29411765	90.94117647
11	8.658823529	65.58823529	38.98235294
12	3.705882353	60.70588235	11.89705882

Sheet4: Province-related public data

	(15)	(16)	(17)	(18)	(19)	(20)	(21)	(22)
Province	Gross domestic product (GDP, 2021) / 10^8 CNY	Total population (TP, 2021) / 10^4 person	Mean years of schooling (MYS) / year	Licensed physician ratio (LPR, 2021) / ‰	Human development index (HDI, 2019)	Mean latitude (ML) / °N	Temperature (T, cumulative annual averages by province) / ℃	Precipitation (P, cumulative annual averages by province) / mm
Anhui	45045	6113	9.35	2.82	0.744	32.0237655	16.19166667	1000.8
Beijing	41611	2189	12.64	5.14	0.907	40.250521	13.16666667	531.8
Chongqing	29129	3212	9.8	2.87	0.774	30.1844385	18.275	1018.7
Fujian	53109.9	4187	9.66	2.65	0.775	25.908957	20.475	1588.9
Gansu	11201.6	2490	9.13	2.84	0.693	37.69543	8.533333333	171.1
Guangdong	129118.6	12684	10.38	2.52	0.799	22.871612	22.80833333	1801.2
Guangxi	26300.9	5037	9.54	2.62	0.734	23.645417	21	1341.3
Guizhou	20164.6	3852	8.75	2.74	0.69	26.922629	14.55	1072.8
Hainan	6818.2	1020	10.1	2.91	0.769	14.23175	24.61666667	2000.1
Hebei	42370.4	7448	9.84	3.41	0.744	39.3320855	12.34166667	513.6
Heilongjiang	15901	3125	9.93	3.1	0.743	48.491947	2.933333333	609
Henan	61345.1	9883	9.79	3.01	0.748	33.875454	15.05833333	753.7
Hong Kong	23470	741.3	12.23	2.1	0.952	22.3507405	24.304	1895.444
Hubei	53734.9	5830	10.02	2.91	0.775	31.1528225	16.35	951.2
Hunan	48670.4	6622	9.88	2.91	0.762	27.3858945	17.64166667	1382.9
Inner Mongolia	23158.7	2400	10.08	3.51	0.777	45.370213	7.325	396.4
Jiangsu	122875.6	8505	10.21	3.21	0.81	32.9437385	15.35833333	1043.4
Jiangxi	32074.7	4517	9.7	2.47	0.747	24.6142975	18	1613.5
Jilin	13070.2	2375	10.17	3.68	0.751	43.5831795	6.125	644.6
Liaoning	28975.1	4229	10.34	3.12	0.78	41.105044	9.016666667	721.5
Macao	1929	68.3	10.48	2.8	0.922	22.163088	22.4	2133.4
Ningxia	5069.6	725	9.81	3.11	0.734	37.31314	9.508333333	259.9
Qinghai	3610.1	594	8.85	3.16	0.695	35.4066335	3.408333333	217.6
Shaanxi	32772.7	3954	10.26	3.05	0.768	35.644697	10.80833333	594.2
Shandong	87435.1	10170	9.75	3.37	0.765	36.3886425	12.525	689.5
Shanghai	44652.8	2489	11.81	3.38	0.88	31.2831675	16.91666667	1259.3
Shanxi	25642.6	3480	10.45	3.26	0.758	37.6643685	10.41666667	423.3
Sichuan	56749.8	8372	9.24	2.99	0.74	30.181723	17.21666667	1039.6
Taiwan	49986	2337.5	14	2.93	0.926	23.917885	23.16666667	1429.7
Tianjin	16311.3	1373	11.29	3.77	0.844	39.4032945	12.9	511.5
Tibet	2132.6	366	6.75	2.9	0.614	31.668991	2.066666667	478
Xinjiang	17741.3	2589	10.11	2.73	0.738	41.7596215	12.03333333	59.2
Yunnan	28954.2	4690	8.82	2.68	0.697	25.183799	16.26666667	951.6
Zhejiang	77715.4	6540	9.79	3.56	0.801	28.9130905	17.625	1445.8
