# Supplementary material for: Nationwide trends and features of human salmonellosis outbreaks in China
Source: Emerg Microbes Infect. 2024 Jun 26;13(1):2372364. doi: 10.1080/22221751.2024.2372364 (PMC11259058; doi:10.1080/22221751.2024.2372364)
Supplement: Supplemental Material [file TEMI_A_2372364_SM2440.pdf]

Table S5: Results of meta-analysis estimating attack rates, gender ratios, and incidence rates of designated symptoms

Sheet1: Attack rate
Sheet2: Gender ratio
Sheet3: Incidence rate of diarrhea
Sheet4: Incidence rate of fever
Sheet5: Incidence rate of headache
Sheet6: Incidence rate of nausea
Sheet7: Incidence rate of abdominal cramps
Sheet8: Incidence rate of vomiting

Sheet1: Attack rate

Meta-analysis

Scale	Estimate	Lower bound	Upper bound	Std. error	p-Value	tau^2	Q(df=505)	Het. p-Value	I^2 / %
Overall	0.3666	0.3388	0.3945	0.0142	<0.0001	0.0992	117465.4969	<0.0001	99.9999
FP	0.457	0.424	0.49	0.0168	<0.0001	0.0971	99469.4737	<0.0001	100
EF	0.1667	0.1317	0.2017	0.0179	<0.0001	0.0524	21587.2974	<0.0001	100
HAI	0.4415	0.3063	0.5766	0.069	<0.0001	0.1005	2177.6268	<0.0001	99.6218
**Top serovars**									
Typhi	0.1508	0.1139	0.1877	0.0188	<0.0001	0.0412	13802.6861	<0.0001	100
Enteritidis	0.4089	0.3556	0.4622	0.0272	<0.0001	0.0787	15395.168	<0.0001	99.9689
Typhimurium	0.57	0.4915	0.6484	0.04	<0.0001	0.1076	22820.8406	<0.0001	99.6905
Paratyphi A	0.0842	0.0413	0.127	0.0219	<0.0001	0.0162	2857.8137	<0.0001	99.9949
Dublin	0.5089	0.3525	0.6653	0.0798	<0.0001	0.1036	3029.7528	<0.0001	99.848
Choleraesuis	0.5343	0.3469	0.7217	0.0956	<0.0001	0.088	981.4168	<0.0001	99.2591
Paratyphi B	0.6121	0.3916	0.8326	0.1125	<0.0001	0.1209	1863.1327	<0.0001	99.3581
Weltevreden	0.4809	0.253	0.7087	0.1163	<0.0001	0.1139	5091.441	<0.0001	99.6939
Blegdam	0.4191	0.0784	0.7599	0.1739	0.0159	0.1804	1113.1096	<0.0001	99.7367
Agona	0.3848	0.1162	0.6535	0.1371	0.005	0.109	541.3122	<0.0001	99.3263

Subgroup-analysis

Economic region	Studies	Estimate	Lower bound	Upper bound	Std. error	p-Val	z-Val	Q(df)	Het. p-Val	I^2 / %
Western	202	0.3455	0.3038	0.3871	0.0212	<0.0001	16.2595	52692.9191(201)	<0.0001	100
Eastern	183	0.379	0.3314	0.4266	0.0243	<0.0001	15.6094	38575.8049(182)	<0.0001	100
Central	103	0.3811	0.3162	0.446	0.0331	<0.0001	11.5131	20619.8588(102)	<0.0001	100
Northeastern	18	0.3955	0.2384	0.5526	0.0802	<0.0001	4.9341	2915.0610(17)	<0.0001	99.88
Overall	506	0.3666	0.3388	0.3945	0.0142	<0.0001	25.7998	117465.4969(505)	<0.0001	100
**Province**										
Sichuan	41	0.4615	0.368	0.5551	0.0477	<0.0001	9.6706	16132.7957(40)	<0.0001	99.63
Zhejiang	35	0.2611	0.1705	0.3517	0.0462	<0.0001	5.6505	2984.2609(34)	<0.0001	99.98
Jiangsu	28	0.3055	0.1802	0.4308	0.0639	<0.0001	4.7773	2931.9185(27)	<0.0001	100
Guangdong	35	0.3633	0.2548	0.4717	0.0553	<0.0001	6.5662	2782.087(34)	<0.0001	99.95
Hebei	15	0.5618	0.3917	0.7318	0.0868	<0.0001	6.4745	3711.5087(14)	<0.0001	99.5
Shanghai	6	0.2295	0.0058	0.4532	0.1141	0.0444	2.0105	113.1596(5)	<0.0001	99.38
Shandong	26	0.5456	0.41	0.6813	0.0692	<0.0001	7.8837	18569.315(25)	<0.0001	99.83
Henan	26	0.2985	0.197	0.4	0.0518	<0.0001	5.7626	5326.8534(25)	<0.0001	100
Anhui	16	0.4646	0.2733	0.6558	0.0976	<0.0001	4.7612	3346.648(15)	<0.0001	99.95
Guangxi	38	0.2987	0.2188	0.3786	0.0408	<0.0001	7.3257	5118.074(37)	<0.0001	99.96
Chongqing	13	0.2998	0.1349	0.4647	0.0841	0.0004	3.5636	1231.5382(12)	<0.0001	99.98
Fujian	16	0.3086	0.1608	0.4563	0.0754	<0.0001	4.0939	1061.9921(15)	<0.0001	99.9
Macau	1	0.3095	0.1697	0.4493	0.0713	NA	NA	NA	NA	NA
Ningxia	12	0.4234	0.2734	0.5734	0.0765	<0.0001	5.5325	734.6395(11)	<0.0001	98.9
Beijing	8	0.5544	0.3271	0.7817	0.116	<0.0001	4.7806	906.9032(7)	<0.0001	98.71
Hainan	2	0.283	-0.3065	0.8724	0.3008	0.3468	0.9408	5.5495(1)	0.0185	80.31
Hunan	25	0.4235	0.2831	0.5639	0.0716	<0.0001	5.9122	6996.6106(24)	<0.0001	99.99
Jiangxi	11	0.3538	0.1191	0.5886	0.1198	0.0031	2.9542	813.6497(10)	<0.0001	99.99
Tianjin	5	0.3409	0.1497	0.5321	0.0976	0.0005	3.4942	296.2674(4)	<0.0001	98.8
Yunnan	29	0.2968	0.1877	0.406	0.0557	<0.0001	5.3312	5151.4807(28)	<0.0001	99.98
Liaoning	12	0.4512	0.2642	0.6382	0.0954	<0.0001	4.7296	939.0034(11)	<0.0001	99.47
Gansu	11	0.4118	0.228	0.5957	0.0938	<0.0001	4.3906	1548.3389(10)	<0.0001	99.2
Hubei	21	0.3205	0.1934	0.4476	0.0648	<0.0001	4.9433	2877.395(20)	<0.0001	99.95
Shaanxi	4	0.3333	0.1121	0.5545	0.1129	0.0031	2.9531	15.7829(3)	0.0013	94.35
Inner Mongolia	10	0.5619	0.3663	0.7575	0.0998	<0.0001	5.6306	767.6091(9)	<0.0001	99.28
Guizhou	27	0.133	0.085	0.181	0.0245	<0.0001	5.4295	2058.3442(26)	<0.0001	99.96
Shanxi	4	0.7315	0.5045	0.9585	0.1158	<0.0001	6.3153	25.7385(3)	<0.0001	89.73
Jilin	3	0.0453	-0.04	0.1306	0.0435	0.298	1.0408	5.4479(2)	0.0656	99.49
Xinjiang	10	0.4	0.16	0.64	0.1224	0.0011	3.2665	2594.3934(9)	<0.0001	100
Heilongjiang	3	0.4983	0.0216	0.975	0.2432	0.0405	2.0486	284.6704(2)	<0.0001	99.1
Qinghai	5	0.575	0.1168	1.0332	0.2338	0.0139	2.4597	6160.9875(4)	<0.0001	100
Tibet	2	0.0746	-0.0808	0.23	0.0793	0.3467	0.9409	9.4507(1)	0.0021	88.64
Taiwan	5	0.5031	0.2584	0.7479	0.1249	<0.0001	4.029	248.7767(4)	<0.0001	98.99
Hong Kong	1	0.3333	0.1156	0.5511	0.1111	NA	NA	NA	NA	NA
Overall	506	0.3666	0.3388	0.3945	0.0142	<0.0001	25.7998	117465.4969(505)	<0.0001	100
**Setting**										
School	85	0.1849	0.1343	0.2356	0.0258	<0.0001	7.1588	29388.9118(84)	<0.0001	99.99
Residential area	240	0.381	0.3395	0.4225	0.0212	<0.0001	17.9982	55107.6589(239)	<0.0001	100
Nursing home	1	0.0565	0.0341	0.0789	0.0114	NA	NA	NA	NA	NA
Restaurant	85	0.4534	0.3944	0.5124	0.0301	<0.0001	15.0529	7809.3706(84)	<0.0001	99.34
Office	21	0.3535	0.2286	0.4783	0.0637	<0.0001	5.5481	2085.8820(20)	<0.0001	99.73
Factory	14	0.1921	0.0457	0.3385	0.0747	0.0101	2.5721	740.2360(13)	<0.0001	99.98
Mobile street stall	11	0.7298	0.5662	0.8933	0.0834	<0.0001	8.7454	1923.6130(10)	<0.0001	98.96
Military region	12	0.3351	0.1689	0.5012	0.0848	<0.0001	3.9526	851.9508(11)	<0.0001	99.94
Public sports venues	1	0.65	0.5293	0.7707	0.0616	NA	NA	NA	NA	NA
Construction site	14	0.6008	0.4004	0.8013	0.1023	<0.0001	5.874	4968.8890(13)	<0.0001	99.85
Hospital	19	0.459	0.3244	0.5936	0.0687	<0.0001	6.6833	1396.8914(18)	<0.0001	98.84
Prison	3	0.0936	-0.0168	0.2041	0.0564	0.0967	1.6612	77.9849(2)	<0.0001	99.03
Overall	506	0.3666	0.3388	0.3945	0.0142	<0.0001	25.7998	117465.4969(505)	<0.0001	100

Sheet2: Gender ratio

Meta-analysis

Scale	Estimate	Lower bound	Upper bound	Std. error	p-Value	tau^2	Q(df=505)	Het. p-Value	I^2 / %
Overall	0.5519	0.5347	0.569	0.0087	<0.0001	0.0317	21827.077	<0.0001	94.1922
FP	0.5421	0.5219	0.5624	0.0103	<0.0001	0.0302	12828.5108	<0.0001	92.5814
EF	0.5739	0.5449	0.603	0.0148	<0.0001	0.0317	8408.2985	<0.0001	95.5232
HAI	0.5735	0.497	0.65	0.039	<0.0001	0.0224	135.5173	<0.0001	75.5829
**Top serovars**									
Typhi	0.5652	0.5299	0.6005	0.018	<0.0001	0.0339	5860.9543	<0.0001	95.5089
Enteritidis	0.547	0.5109	0.583	0.0184	<0.0001	0.0302	3914.0387	<0.0001	93.1049
Typhimurium	0.552	0.5082	0.5958	0.0223	<0.0001	0.0259	2743.6103	<0.0001	90.251
Paratyphi A	0.5999	0.5369	0.6629	0.0321	<0.0001	0.0314	1782.4954	<0.0001	95.8538
Dublin	0.4942	0.4006	0.5877	0.0477	<0.0001	0.0297	80.7633	<0.0001	88.8088
Choleraesuis	0.4461	0.355	0.5373	0.0465	<0.0001	0.0149	44.0081	<0.0001	78.8511
Paratyphi B	0.6154	0.5105	0.7202	0.0535	<0.0001	0.0184	64.9795	<0.0001	78.389
Weltevreden	0.5065	0.4076	0.6054	0.0505	<0.0001	0.0152	46.41	<0.0001	75.4363
Blegdam	0.5196	0.4133	0.6259	0.0543	<0.0001	0.0094	9.915	0.0777	54.0632
Agona	0.5375	0.4136	0.6614	0.0632	<0.0001	0.0159	16.1478	0.0064	71.8302

Subgroup-analysis

Economic region	Studies	Estimate	Lower bound	Upper bound	Std. error	p-Val	z-Val	Q(df)	Het. p-Val	I^2 / %
Western	202	0.5393	0.5151	0.5635	0.0123	<0.0001	43.7024	4738.3173(201)	<0.0001	92.13
Eastern	183	0.5471	0.5158	0.5785	0.016	<0.0001	34.2058	13425.7963(182)	<0.0001	95.85
Central	103	0.5565	0.5203	0.5927	0.0185	<0.0001	30.1398	1776.3592(102)	<0.0001	91.27
Northeastern	18	0.7142	0.6205	0.808	0.0478	<0.0001	14.935	305.9571(17)	<0.0001	94.12
Overall	506	0.5519	0.5347	0.569	0.0087	<0.0001	63.1766	21827.0770(505)	<0.0001	94.19
**Province**										
Sichuan	41	0.5127	0.4612	0.5641	0.0262	<0.0001	19.533	549.4491(40)	<0.0001	84.67
Zhejiang	35	0.4992	0.4441	0.5543	0.0281	<0.0001	17.7609	169.0703(34)	<0.0001	81.04
Jiangsu	28	0.5489	0.4672	0.6305	0.0417	<0.0001	13.1765	1609.2679(27)	<0.0001	92.67
Guangdong	35	0.6154	0.5392	0.6916	0.0389	<0.0001	15.8303	1248.9075(34)	<0.0001	96.25
Hebei	15	0.5591	0.4595	0.6587	0.0508	<0.0001	11.0011	128.4698(14)	<0.0001	89.11
Shanghai	6	0.3397	0.1587	0.5207	0.0923	0.0002	3.6786	36.2033(5)	<0.0001	86.22
Shandong	26	0.5534	0.4589	0.6478	0.0482	<0.0001	11.4833	2021.7677(25)	<0.0001	96.06
Henan	26	0.524	0.4531	0.5949	0.0362	<0.0001	14.4862	631.8529(25)	<0.0001	92.83
Anhui	16	0.611	0.5143	0.7076	0.0493	<0.0001	12.391	256.5960(15)	<0.0001	89.46
Guangxi	38	0.52	0.4622	0.5778	0.0295	<0.0001	17.6432	1077.7312(37)	<0.0001	92.97
Chongqing	13	0.4962	0.4218	0.5706	0.038	<0.0001	13.0747	29.4422(12)	0.0034	89.2
Fujian	16	0.5451	0.4581	0.6321	0.0444	<0.0001	12.2824	439.2155(15)	<0.0001	86.25
Macau	1	0.0357	-0.0615	0.1329	0.0496	NA	NA	NA	NA	NA
Ningxia	12	0.5438	0.4591	0.6286	0.0432	<0.0001	12.584	25.4280(11)	0.0079	65.83
Beijing	8	0.5902	0.4273	0.7532	0.0831	<0.0001	7.0995	129.4283(7)	<0.0001	88.72
Hainan	2	0.5185	0.3301	0.707	0.0962	<0.0001	5.3925	0.0030(1)	0.9566	0
Hunan	25	0.5606	0.488	0.6331	0.037	<0.0001	15.1525	563.5807(24)	<0.0001	93.37
Jiangxi	11	0.6625	0.5401	0.7849	0.0624	<0.0001	10.6091	67.5748(10)	<0.0001	90.04
Tianjin	5	0.5743	0.3489	0.7996	0.115	<0.0001	4.9942	338.5249(4)	<0.0001	97.23
Yunnan	29	0.5098	0.4452	0.5744	0.033	<0.0001	15.4674	376.3954(28)	<0.0001	92.39
Liaoning	12	0.6784	0.5593	0.7975	0.0608	<0.0001	11.1629	260.6444(11)	<0.0001	95.23
Gansu	11	0.597	0.4637	0.7304	0.068	<0.0001	8.7751	421.7998(10)	<0.0001	95.42
Hubei	21	0.5304	0.4678	0.5929	0.0319	<0.0001	16.6184	86.6334(20)	<0.0001	75.92
Shaanxi	4	0.6627	0.5459	0.7796	0.0596	<0.0001	11.1172	1.8796(3)	0.5978	13.82
Inner Mongolia	10	0.6428	0.495	0.7905	0.0754	<0.0001	8.5263	376.6242(9)	<0.0001	96.7
Guizhou	27	0.5461	0.5038	0.5884	0.0216	<0.0001	25.2998	83.7992(26)	<0.0001	74.23
Shanxi	4	0.364	0.1635	0.5644	0.1023	0.0004	3.5584	14.3683(3)	0.0024	73.76
Jilin	3	0.6901	0.5	0.8803	0.097	<0.0001	7.1138	3.7612(2)	0.1525	58.69
Xinjiang	10	0.5699	0.4363	0.7036	0.0682	<0.0001	8.3581	795.7480(9)	<0.0001	97.12
Heilongjiang	3	0.8686	0.6834	1.0538	0.0945	<0.0001	9.1919	17.1128(2)	0.0002	86.98
Qinghai	5	0.6246	0.4327	0.8166	0.0979	<0.0001	6.3775	232.1429(4)	<0.0001	97.07
Tibet	2	0.5798	0.3227	0.8369	0.1312	<0.0001	4.4197	2.5452(1)	0.1106	58.77
Taiwan	5	0.5698	0.3943	0.7453	0.0896	<0.0001	6.3625	82.4177(4)	<0.0001	92.66
Hong Kong	1	0.5	0.0999	0.9001	0.2041	NA	NA	NA	NA	NA
Overall	506	0.5519	0.5347	0.569	0.0087	<0.0001	63.1766	21827.0770(505)	<0.0001	94.19
**Setting**										
School	85	0.564	0.525	0.603	0.0199	<0.0001	28.3405	2023.9164(84)	<0.0001	94.54
Residential area	240	0.4967	0.4801	0.5134	0.0085	<0.0001	58.5189	1050.8522(239)	<0.0001	75.61
Nursing home	1	0.7391	0.5597	0.9186	0.0916	NA	NA	NA	NA	NA
Restaurant	85	0.5362	0.4939	0.5785	0.0216	<0.0001	24.8465	1177.2119(84)	<0.0001	88.89
Office	21	0.5829	0.4699	0.6959	0.0577	<0.0001	10.1085	5054.4623(20)	<0.0001	98.4
Factory	14	0.5445	0.386	0.7031	0.0809	<0.0001	6.7311	959.6656(13)	<0.0001	97.48
Mobile street stall	11	0.5033	0.4193	0.5873	0.0429	<0.0001	11.7443	31.5766(10)	0.0005	81.29
Military region	12	0.9272	0.8579	0.9965	0.0354	<0.0001	26.2276	48.5069(11)	<0.0001	96.64
Public sports venues	1	0.9875	0.9531	1.0219	0.0176	NA	NA	NA	NA	NA
Construction site	14	0.938	0.8879	0.988	0.0255	<0.0001	36.7527	14.6225(13)	0.3315	74.67
Hospital	19	0.5667	0.4827	0.6508	0.0429	<0.0001	13.2116	129.5615(18)	<0.0001	76.76
Prison	3	0.9916	0.9774	1.0058	0.0073	<0.0001	136.6054	0.1599(2)	0.9232	0.82
Overall	506	0.5519	0.5347	0.569	0.0087	<0.0001	63.1766	21827.0770(505)	<0.0001	94.19

Sheet3: Incidence rate of diarrhea

Meta-analysis

Scale	Estimate	Lower bound	Upper bound	Std. error	p-Value	tau^2	Q(df=505)	Het. p-Value	I^2 / %
Overall	0.7581	0.7278	0.7885	0.0155	<0.0001	0.1185	1799566.225	<0.0001	99.9523
FP	0.8774	0.8541	0.9007	0.0119	<0.0001	0.0475	676547.8094	<0.0001	99.8161
EF	0.4299	0.3664	0.4935	0.0324	<0.0001	0.1736	1555166.605	<0.0001	99.9802
HAI	0.8752	0.7723	0.978	0.0525	<0.0001	0.0577	475.6343	<0.0001	98.7021
**Top serovars**									
Typhi	0.4255	0.3514	0.4995	0.0378	<0.0001	0.1666	934058.8532	<0.0001	99.9778
Enteritidis	0.9144	0.8894	0.9394	0.0128	<0.0001	0.0158	6980.9662	<0.0001	99.1045
Typhimurium	0.9466	0.9246	0.9686	0.0112	<0.0001	0.0069	486.4114	<0.0001	97.273
Paratyphi A	0.2664	0.1423	0.3904	0.0633	<0.0001	0.139	62081.1753	<0.0001	99.9684
Dublin	0.9695	0.9455	0.9935	0.0123	<0.0001	0.0014	30.7275	0.0146	95.6544
Choleraesuis	0.9387	0.8775	0.9998	0.0312	<<0.0001	0.0086	33.2074	0.0003	96.9367
Paratyphi B	0.5535	0.2948	0.8121	0.132	<<0.0001	0.1721	4689.727	<0.0001	99.7102
Weltevreden	0.9631	0.9145	1.0117	0.0248	<<0.0001	0.0042	17.3755	0.0264	94.3275
Blegdam	0.9774	0.9533	1.0015	0.0123	<<0.0001	0.0002	2.4717	0.7808	19.5301
Agona	0.7363	0.4832	0.9894	0.1291	<<0.0001	0.0971	174.4446	<0.0001	99.5022

Subgroup-analysis

Economic region	Studies	Estimate	Lower bound	Upper bound	Std. error	p-Val	z-Val	Q(df)	Het. p-Val	I^2 / %
Western	202	0.7531	0.7046	0.8016	0.0247	<0.0001	30.4406	1056464.9929(201)	<0.0001	99.97
Eastern	183	0.7672	0.7174	0.817	0.0254	<0.0001	30.2027	174625.4847(182)	<0.0001	99.81
Central	103	0.7397	0.6706	0.8088	0.0353	<0.0001	20.9758	363340.7641(102)	<0.0001	99.93
Northeastern	18	0.8281	0.6821	0.9742	0.0745	<0.0001	11.1139	13351.8186(17)	<0.0001	99.64
Overall	506	0.7581	0.7278	0.7885	0.0155	<0.0001	48.9649	1799566.2245(505)	<0.0001	99.95
**Province**										
Sichuan	41	0.8369	0.7616	0.9122	0.0384	<0.0001	21.7861	4144.4519(40)	<0.0001	99.77
Zhejiang	35	0.5976	0.4532	0.742	0.0737	<0.0001	8.1106	38882.9422(34)	<0.0001	99.87
Jiangsu	28	0.6842	0.5342	0.8342	0.0765	<0.0001	8.9414	14399.8968(27)	<0.0001	99.72
Guangdong	35	0.8193	0.7272	0.9115	0.047	<0.0001	17.4284	4338.6575(34)	<0.0001	99.34
Hebei	15	0.9505	0.9133	0.9878	0.019	<0.0001	50.0007	68.2765(14)	<0.0001	94.98
Shanghai	6	0.9879	0.9648	1.0111	0.0118	<0.0001	83.649	1.4855(5)	0.9147	38.76
Shandong	26	0.8192	0.699	0.9395	0.0614	<0.0001	13.3523	37857.8942(25)	<0.0001	99.77
Henan	26	0.7348	0.6043	0.8653	0.0666	<0.0001	11.0338	81895.5091(25)	<0.0001	99.92
Anhui	16	0.7885	0.6189	0.958	0.0865	<0.0001	9.1151	20190.4055(15)	<0.0001	99.8
Guangxi	38	0.7714	0.6577	0.8852	0.058	<0.0001	13.2891	180900.7817(37)	<0.0001	99.96
Chongqing	13	0.8366	0.6866	0.9866	0.0765	<0.0001	10.9317	5567.0659(12)	<0.0001	99.93
Fujian	16	0.7609	0.5717	0.9501	0.0965	<0.0001	7.8819	15815.4429(15)	<0.0001	99.81
Macau	1	0.9643	0.8671	1.0615	0.0496	NA	NA	NA	NA	NA
Ningxia	12	0.8636	0.7084	1.0188	0.0792	<0.0001	10.9043	170.9375(11)	<0.0001	99.6
Beijing	8	0.7743	0.6012	0.9475	0.0884	<0.0001	8.764	83.7311(7)	<0.0001	99.28
Hainan	2	0.8041	0.657	0.9511	0.075	<0.0001	10.7185	0.0211(1)	0.8845	0.02
Hunan	25	0.6855	0.5323	0.8387	0.0782	<0.0001	8.7701	164215.9409(24)	<0.0001	99.96
Jiangxi	11	0.745	0.5176	0.9723	0.116	<0.0001	6.4233	4753.4117(10)	<0.0001	99.88
Tianjin	5	0.9123	0.759	1.0655	0.0782	<0.0001	11.6644	50.1409(4)	<0.0001	99.54
Yunnan	29	0.7966	0.6721	0.921	0.0635	<0.0001	12.5442	300031.9303(28)	<0.0001	99.97
Liaoning	12	0.8791	0.721	1.0373	0.0807	<0.0001	10.8938	12544.4293(11)	<0.0001	99.65
Gansu	11	0.7036	0.4522	0.9549	0.1282	<0.0001	5.4862	12468.3463(10)	<0.0001	99.91
Hubei	21	0.744	0.5785	0.9095	0.0844	<0.0001	8.8116	16556.0186(20)	<0.0001	99.82
Shaanxi	4	0.958	0.9084	1.0076	0.0253	<0.0001	37.886	1.7255(3)	0.6313	17.75
Inner Mongolia	10	0.9142	0.8432	0.9853	0.0362	<0.0001	25.2285	294.2128(9)	<0.0001	98.6
Guizhou	27	0.3905	0.2315	0.5495	0.0811	<0.0001	4.8129	122492.7038(26)	<0.0001	99.96
Shanxi	4	0.8827	0.778	0.9875	0.0535	<0.0001	16.5107	13.5458(3)	0.0036	64.25
Jilin	3	0.4688	-0.0156	0.9533	0.2472	0.0578	1.8969	60.0181(2)	<0.0001	97.08
Xinjiang	10	0.8036	0.6377	0.9694	0.0846	<0.0001	9.4973	931.5958(9)	<0.0001	99.81
Heilongjiang	3	0.9856	0.9567	1.0144	0.0147	<0.0001	67.0577	0.4091(2)	0.815	8.8
Qinghai	5	0.7059	0.3234	1.0885	0.1952	0.0003	3.6171	26580.5356(4)	<0.0001	99.98
Tibet	2	0.8013	0.5319	1.0708	0.1375	<0.0001	5.8294	6.9865(1)	0.0082	84.45
Taiwan	5	0.8402	0.6156	1.0648	0.1146	<0.0001	7.3316	109.4917(4)	<0.0001	99.14
Hong Kong	1	0.9286	0.7378	1.1194	0.0973	NA	NA	NA	NA	NA
Overall	506	0.7581	0.7278	0.7885	0.0155	<0.0001	48.9649	1799566.2245(505)	<0.0001	99.95
**Setting**										
School	85	0.68	0.5988	0.7612	0.0414	<0.0001	16.4204	498487.841(84)	<0.0001	99.97
Residential area	240	0.7079	0.6597	0.7561	0.0246	<0.0001	28.7672	1153807.3057(239)	<0.0001	99.97
Nursing home	1	0.8696	0.7319	1.0072	0.0702	NA	NA	NA	NA	NA
Restaurant	85	0.9489	0.9326	0.9652	0.0083	<0.0001	114.2586	269.0323(84)	<0.0001	95.21
Office	21	0.9058	0.8306	0.9809	0.0383	<0.0001	23.6257	158.4974(20)	<0.0001	99.52
Factory	14	0.5298	0.3017	0.758	0.1164	<0.0001	4.5516	10329.2685(13)	<0.0001	99.8
Mobile street stall	11	0.92	0.8579	0.9822	0.0317	<0.0001	29.0249	100.1849(10)	<0.0001	95.72
Military region	12	0.5874	0.3475	0.8273	0.1224	<0.0001	4.7991	2243.3643(11)	<0.0001	99.56
Public sports venues	1	0.9875	0.9531	1.0219	0.0176	NA	NA	NA	NA	NA
Construction site	14	0.8238	0.6447	1.0028	0.0914	<0.0001	9.0158	1496.1776(13)	<0.0001	98.91
Hospital	19	0.936	0.8661	1.0059	0.0356	<0.0001	26.2576	284.0479(18)	<0.0001	97.05
Prison	3	0.677	0.3489	1.0051	0.1674	<0.0001	4.0442	80.771(2)	<0.0001	96.91
Overall	506	0.7581	0.7278	0.7885	0.0155	<0.0001	48.9649	1799566.2245(505)	<0.0001	99.95

Sheet4: Incidence rate of fever

Meta-analysis

Scale	Estimate	Lower bound	Upper bound	Std. error	p-Value	tau^2	Q(df=505)	Het. p-Value	I^2 / %
Overall	0.8467	0.8253	0.868	0.0109	<0.0001	0.0566	99807.3721	<0.0001	99.9175
FP	0.8008	0.7732	0.8284	0.0141	<0.0001	0.0664	93574.2362	<0.0001	99.8541
EF	0.956	0.9358	0.9762	0.0103	<0.0001	0.0169	5031.0341	<0.0001	99.8658
HAI	0.8264	0.7227	0.9302	0.0529	<0.0001	0.0562	232.6749	<0.0001	98.8108
**Top serovars**									
Typhi	0.9788	0.968	0.9896	0.0055	<0.0001	0.0031	322.162	<0.0001	99.3455
Enteritidis	0.8212	0.782	0.8603	0.02	<0.0001	0.0402	57888.7327	<0.0001	99.5791
Typhimurium	0.7795	0.7162	0.8429	0.0323	<0.0001	0.0672	1718.7131	<0.0001	99.544
Paratyphi A	0.957	0.9095	1.0045	0.0242	<0.0001	0.0199	2256.5982	<0.0001	99.809
Dublin	0.9365	0.876	0.9971	0.0309	<0.0001	0.0133	62.4583	<0.0001	99.5411
Choleraesuis	0.8214	0.6818	0.9611	0.0712	<<0.0001	0.0522	204.5175	<0.0001	99.248
Paratyphi B	0.5415	0.2739	0.8091	0.1365	<<0.0001	0.1844	5309.0409	<0.0001	99.7284
Weltevreden	0.9075	0.8027	1.0124	0.0535	<<0.0001	0.0218	53.3168	<0.0001	98.9143
Blegdam	0.864	0.7619	0.9661	0.0521	<<0.0001	0.0123	26.6242	<0.0001	84.3913
Agona	0.6479	0.3289	0.9669	0.1628	<<0.0001	0.1561	313.7722	<0.0001	99.7137

Subgroup-analysis

Economic region	Studies	Estimate	Lower bound	Upper bound	Std. error	p-Val	z-Val	Q(df)	Het. p-Val	I^2 / %
Western	202	0.848	0.8144	0.8816	0.0171	<0.0001	49.4983	20648.1476(201)	<0.0001	99.95
Eastern	183	0.8345	0.7975	0.8714	0.0189	<0.0001	44.2163	68593.17(182)	<0.0001	99.65
Central	103	0.8627	0.8166	0.9089	0.0235	<0.0001	36.6611	3272.944(102)	<0.0001	99.86
Northeastern	18	0.8701	0.7885	0.9517	0.0416	<0.0001	20.9028	119.0366(17)	<0.0001	98.37
Overall	506	0.8467	0.8253	0.868	0.0109	<0.0001	77.8452	99807.3721(505)	<0.0001	99.92
**Province**										
Sichuan	41	0.7803	0.7015	0.8592	0.0402	<0.0001	19.3878	1293.6874(40)	<0.0001	99.55
Zhejiang	35	0.873	0.796	0.9499	0.0393	<0.0001	22.2244	746.2727(34)	<0.0001	99.6
Jiangsu	28	0.8816	0.7974	0.9657	0.0429	<0.0001	20.5365	361.7841(27)	<0.0001	99.5
Guangdong	35	0.8066	0.7134	0.8997	0.0475	<0.0001	16.9669	5628.5796(34)	<0.0001	99.56
Hebei	15	0.8618	0.724	0.9997	0.0703	<0.0001	12.2515	24229.123(14)	<0.0001	99.7
Shanghai	6	0.6684	0.3721	0.9647	0.1512	<0.0001	4.4212	195.0724(5)	<0.0001	98.83
Shandong	26	0.8891	0.8206	0.9576	0.035	<0.0001	25.433	260.2511(25)	<0.0001	98.58
Henan	26	0.8968	0.8329	0.9606	0.0326	<0.0001	27.5278	291.2754(25)	<0.0001	99.72
Anhui	16	0.9197	0.8428	0.9965	0.0392	<0.0001	23.4544	94.7531(15)	<0.0001	98.94
Guangxi	38	0.8619	0.7854	0.9385	0.039	<0.0001	22.0735	2793.1296(37)	<0.0001	99.89
Chongqing	13	0.8621	0.7685	0.9557	0.0478	<0.0001	18.0545	128.4652(12)	<0.0001	99.98
Fujian	16	0.7474	0.5893	0.9056	0.0807	<0.0001	9.262	5044.4713(15)	<0.0001	99.63
Macau	1	0.7692	0.5402	0.9983	0.1169	NA	NA	NA	NA	NA
Ningxia	12	0.8426	0.6947	0.9905	0.0755	<0.0001	11.1672	115.9696(11)	<0.0001	99.41
Beijing	8	0.6791	0.4527	0.9056	0.1155	<0.0001	5.8782	377.8078(7)	<0.0001	97.17
Hainan	2	0.8761	0.7538	0.9983	0.0624	<0.0001	14.046	0.0431(1)	0.8355	0.09
Hunan	25	0.9104	0.8388	0.9819	0.0365	<0.0001	24.9449	561.0615(24)	<0.0001	99.82
Jiangxi	11	0.8859	0.7525	1.0194	0.0681	<0.0001	13.0136	97.3416(10)	<0.0001	99.66
Tianjin	5	0.97	0.9377	1.0024	0.0165	<0.0001	58.7933	11.5209(4)	0.0213	81.84
Yunnan	29	0.8155	0.7111	0.9198	0.0532	<0.0001	15.3219	13385.5567(28)	<0.0001	99.95
Liaoning	12	0.8584	0.7474	0.9695	0.0567	<0.0001	15.1503	99.0631(11)	<0.0001	99.1
Gansu	11	0.8312	0.6577	1.0047	0.0885	<0.0001	9.3893	534.7741(10)	<0.0001	99.85
Hubei	21	0.757	0.6101	0.9039	0.075	<0.0001	10.0989	1384.3773(20)	<0.0001	99.75
Shaanxi	4	0.9747	0.9361	1.0133	0.0197	<0.0001	49.5346	0.731(3)	0.8659	12.89
Inner Mongolia	10	0.7302	0.5252	0.9351	0.1046	<0.0001	6.9836	1802.0557(9)	<0.0001	99.84
Guizhou	27	0.972	0.9395	1.0046	0.0166	<0.0001	58.5104	91.9055(26)	<0.0001	99.25
Shanxi	4	0.6237	0.2201	1.0274	0.206	0.0025	3.0285	211.1294(3)	<0.0001	97.68
Jilin	3	0.8497	0.6198	1.0796	0.1173	<0.0001	7.2441	5.5211(2)	0.0633	87.78
Xinjiang	10	0.8673	0.7108	1.0238	0.0799	<0.0001	10.861	171.5636(9)	<0.0001	99.95
Heilongjiang	3	0.9225	0.8278	1.0172	0.0483	<0.0001	19.0943	6.2421(2)	0.0441	60.47
Qinghai	5	0.856	0.6357	1.0762	0.1124	<0.0001	7.6175	48.4164(4)	<0.0001	99.94
Tibet	2	0.9842	0.9444	1.024	0.0203	<0.0001	48.4975	0.2967(1)	0.5859	3.69
Taiwan	5	0.7179	0.5383	0.8974	0.0916	<0.0001	7.8359	199.095(4)	<0.0001	96.18
Hong Kong	1	0.9286	0.7378	1.1194	0.0973	NA	NA	NA	NA	NA
Overall	506	0.8467	0.8253	0.868	0.0109	<0.0001	77.8452	99807.3721(505)	<0.0001	99.92
**Setting**										
School	85	0.898	0.8546	0.9413	0.0221	<0.0001	40.6179	5973.9847(84)	<0.0001	99.93
Residential area	240	0.8519	0.8212	0.8826	0.0157	<0.0001	54.4284	84196.3947(239)	<0.0001	99.92
Nursing home	1	0.913	0.7979	1.0282	0.0588	NA	NA	NA	NA	NA
Restaurant	85	0.7917	0.7304	0.8531	0.0313	<0.0001	25.2907	6964.1561(84)	<0.0001	99.7
Office	21	0.7401	0.6303	0.8498	0.056	<0.0001	13.2154	850.6002(20)	<0.0001	99.7
Factory	14	0.911	0.834	0.9881	0.0393	<0.0001	23.165	187.9398(13)	<0.0001	98.37
Mobile street stall	11	0.8154	0.6757	0.9551	0.0713	<0.0001	11.4389	494.4921(10)	<0.0001	99.23
Military region	12	0.9597	0.9061	1.0134	0.0274	<0.0001	35.0562	19.6612(11)	0.0502	95.5
Public sports venues	1	0.9875	0.9531	1.0219	0.0176	NA	NA	NA	NA	NA
Construction site	14	0.7983	0.6428	0.9538	0.0793	<0.0001	10.0623	450.595(13)	<0.0001	98.18
Hospital	19	0.8123	0.6926	0.932	0.0611	<0.0001	13.2998	223.4168(18)	<0.0001	99.08
Prison	3	0.9788	0.9497	1.008	0.0149	<0.0001	65.8409	2.5152(2)	0.2843	39.37
Overall	506	0.8467	0.8253	0.868	0.0109	<0.0001	77.8452	99807.3721(505)	<0.0001	99.92

Sheet5: Incidence rate of headache

Meta-analysis

Scale	Estimate	Lower bound	Upper bound	Std. error	p-Value	tau^2	Q(df=505)	Het. p-Value	I^2 / %
Overall	0.5353	0.4991	0.5714	0.0185	<0.0001	0.169	1908055.604	<0.0001	99.9649
FP	0.5081	0.466	0.5501	0.0214	<0.0001	0.1594	938518.7479	<0.0001	99.94
EF	0.6376	0.5743	0.7009	0.0323	<0.0001	0.1724	567399.3315	<0.0001	99.9759
HAI	0.1926	0.0445	0.3406	0.0755	0.0108	0.123	2429.8804	<0.0001	99.4434
**Top serovars**									
Typhi	0.6582	0.5829	0.7335	0.0384	<0.0001	0.173	389420.1724	<0.0001	99.9798
Enteritidis	0.4416	0.3661	0.5171	0.0385	<0.0001	0.161	233700.2758	<0.0001	99.9231
Typhimurium	0.4388	0.3439	0.5336	0.0484	<0.0001	0.1589	120906.7819	<0.0001	99.8996
Paratyphi A	0.6935	0.5684	0.8187	0.0639	<0.0001	0.1411	87420.6719	<0.0001	99.9278
Dublin	0.7779	0.6396	0.9163	0.0706	<0.0001	0.08	541.8374	<0.0001	99.9183
Choleraesuis	0.434	0.1948	0.6732	0.122	0.0004	0.1602	1188.5372	<<0.0001	99.7713
Paratyphi B	0.3006	0.0592	0.542	0.1231	0.0147	0.1497	6689.6047	<<0.0001	99.8755
Weltevreden	0.4621	0.2406	0.6837	0.113	<<0.0001	0.1076	4672.0409	<0.0001	99.3846
Blegdam	0.8402	0.6534	1.027	0.0953	<<0.0001	0.0511	48.8316	<0.0001	98.2118
Agona	0.5143	0.1872	0.8414	0.1669	0.0021	0.1643	1751.6838	<<0.0001	99.7053

Subgroup-analysis

Economic region	Studies	Estimate	Lower bound	Upper bound	Std. error	p-Val	z-Val	Q(df)	Het. p-Val	I^2 / %
Western	202	0.586	0.5295	0.6425	0.0288	<0.0001	20.3279	1067369.0362(201)	<0.0001	99.98
Eastern	183	0.4744	0.4144	0.5344	0.0306	<0.0001	15.5028	251788.0841(182)	<0.0001	99.89
Central	103	0.5488	0.4682	0.6293	0.0411	<0.0001	13.3567	445831.0077(102)	<0.0001	99.96
Northeastern	18	0.5021	0.3038	0.7004	0.1012	<0.0001	4.9636	10945.2702(17)	<0.0001	99.75
Overall	506	0.5353	0.4991	0.5714	0.0185	<0.0001	29.0044	1908055.6037(505)	<0.0001	99.96
**Province**										
Sichuan	41	0.4594	0.3321	0.5867	0.0649	<0.0001	7.0738	134244.5026(40)	<0.0001	99.94
Zhejiang	35	0.4081	0.2684	0.5478	0.0713	<0.0001	5.7244	34101.0955(34)	<0.0001	99.89
Jiangsu	28	0.4592	0.2923	0.6261	0.0851	<0.0001	5.3935	19477.5456(27)	<0.0001	99.86
Guangdong	35	0.5595	0.42	0.699	0.0712	<0.0001	7.863	70178.2856(34)	<0.0001	99.92
Hebei	15	0.4878	0.2643	0.7112	0.114	<0.0001	4.2784	18113.6586(14)	<0.0001	99.91
Shanghai	6	0.1335	-0.0484	0.3155	0.0928	0.1503	1.4385	71.7693(5)	<0.0001	97.68
Shandong	26	0.4797	0.3141	0.6452	0.0845	<0.0001	5.6793	38485.2482(25)	<0.0001	99.9
Henan	26	0.4465	0.2862	0.6068	0.0818	<0.0001	5.4589	139609.9105(25)	<0.0001	99.97
Anhui	16	0.5354	0.2996	0.7713	0.1203	<0.0001	4.4497	15982.6267(15)	<0.0001	99.91
Guangxi	38	0.7281	0.6133	0.8429	0.0586	<0.0001	12.4317	39420.6362(37)	<0.0001	99.92
Chongqing	13	0.3969	0.207	0.5868	0.0969	<0.0001	4.0962	15459.2887(12)	<0.0001	99.96
Fujian	16	0.5661	0.3868	0.7455	0.0915	<0.0001	6.1861	7371.2799(15)	<0.0001	99.58
Macau	1	0.6923	0.4414	0.9432	0.128	NA	NA	NA	NA	NA
Ningxia	12	0.4763	0.2302	0.7225	0.1256	<0.0001	3.7927	10174.0435(11)	<0.0001	99.77
Beijing	8	0.6471	0.4126	0.8816	0.1197	<0.0001	5.4077	1522.5063(7)	<0.0001	98.62
Hainan	2	0.3414	0.1106	0.5722	0.1178	0.0037	2.8993	0.974(1)	0.3237	24.19
Hunan	25	0.5932	0.4369	0.7496	0.0798	<0.0001	7.4364	157304.0001(24)	<0.0001	99.96
Jiangxi	11	0.5668	0.2955	0.8381	0.1384	<0.0001	4.0949	22902.297(10)	<0.0001	99.92
Tianjin	5	0.3707	-0.0618	0.8032	0.2207	0.093	1.6798	11861.534(4)	<0.0001	99.95
Yunnan	29	0.6284	0.4821	0.7747	0.0746	<0.0001	8.4195	81272.5825(28)	<0.0001	99.94
Liaoning	12	0.4991	0.2654	0.7327	0.1192	<0.0001	4.1868	8462.9831(11)	<0.0001	99.77
Gansu	11	0.4586	0.1839	0.7333	0.1402	0.0011	3.2717	18775.6059(10)	<0.0001	99.91
Hubei	21	0.638	0.4716	0.8044	0.0849	<0.0001	7.5149	13555.3474(20)	<0.0001	99.77
Shaanxi	4	0.4392	-0.028	0.9063	0.2383	0.0654	1.8426	389.7666(3)	<0.0001	98.89
Inner Mongolia	10	0.526	0.2788	0.7731	0.1261	<0.0001	4.1713	22999.4462(9)	<0.0001	99.9
Guizhou	27	0.712	0.5614	0.8627	0.0768	<0.0001	9.2652	109605.0962(26)	<0.0001	99.96
Shanxi	4	0.4686	0.1035	0.8337	0.1863	0.0119	2.5153	111.0061(3)	<0.0001	96.39
Jilin	3	0.3719	-0.2176	0.9614	0.3008	0.2163	1.2364	333.6368(2)	<0.0001	99.07
Xinjiang	10	0.6691	0.401	0.9373	0.1368	<0.0001	4.8911	25654.0028(9)	<0.0001	99.97
Heilongjiang	3	0.6405	0.0196	1.2614	0.3168	0.0432	2.0217	1056.3618(2)	<0.0001	99.71
Qinghai	5	0.5312	0.1789	0.8836	0.1798	0.0031	2.9549	29521.2889(4)	<0.0001	99.96
Tibet	2	0.9842	0.9444	1.024	0.0203	<0.0001	48.4975	0.2967(1)	0.5859	3.69
Taiwan	5	0.3012	0.0693	0.5332	0.1183	0.0109	2.5457	209.1681(4)	<0.0001	98.33
Hong Kong	1	0.6667	0.2895	1.0439	0.1925	NA	NA	NA	NA	NA
Overall	506	0.5353	0.4991	0.5714	0.0185	<0.0001	29.0044	1908055.6037(505)	<0.0001	99.96
**Setting**										
School	85	0.576	0.4895	0.6624	0.0441	<0.0001	13.061	441050.7538(84)	<0.0001	99.97
Residential area	240	0.5851	0.5332	0.6371	0.0265	<0.0001	22.0763	1135907.0766(239)	<0.0001	99.97
Nursing home	1	0.0208	-0.0363	0.078	0.0292	NA	NA	NA	NA	NA
Restaurant	85	0.4038	0.3209	0.4866	0.0423	<0.0001	9.5535	59495.1242(84)	<0.0001	99.81
Office	21	0.4568	0.2751	0.6385	0.0927	<0.0001	4.9283	11734.0413(20)	<0.0001	99.88
Factory	14	0.5595	0.3254	0.7936	0.1194	<0.0001	4.6846	22796.8219(13)	<0.0001	99.92
Mobile street stall	11	0.5766	0.3544	0.7988	0.1134	<0.0001	5.0857	2306.386(10)	<0.0001	99.53
Military region	12	0.7354	0.4888	0.9819	0.1258	<0.0001	5.8454	6941.9878(11)	<0.0001	99.82
Public sports venues	1	0.9875	0.9531	1.0219	0.0176	NA	NA	NA	NA	NA
Construction site	14	0.7088	0.5196	0.8981	0.0965	<0.0001	7.3422	347.7013(13)	<0.0001	98.7
Hospital	19	0.122	-0.0018	0.2458	0.0632	0.0535	1.9312	1054.8604(18)	<0.0001	99.11
Prison	3	0.4402	-0.0248	0.9053	0.2373	0.0635	1.8554	319.6006(2)	<0.0001	98.93
Overall	506	0.5353	0.4991	0.5714	0.0185	<0.0001	29.0044	1908055.6037(505)	<0.0001	99.96

Sheet6: Incidence rate of nausea

Meta-analysis

Scale	Estimate	Lower bound	Upper bound	Std. error	p-Value	tau^2	Q(df=505)	Het. p-Value	I^2 / %
Overall	0.4711	0.435	0.5072	0.0184	<0.0001	0.168	2116193.221	<0.0001	99.9745
FP	0.6177	0.5788	0.6566	0.0198	<0.0001	0.1356	1103374.505	<0.0001	99.9394
EF	0.1615	0.1141	0.2089	0.0242	<0.0001	0.0963	138583.888	<0.0001	99.9774
HAI	0.1527	0.0242	0.2811	0.0655	0.0198	0.0917	1734.058	<0.0001	99.5218
**Top serovars**									
Typhi	0.144	0.0908	0.1973	0.0272	<0.0001	0.0861	87753.6482	<0.0001	99.9762
Enteritidis	0.6113	0.5423	0.6803	0.0352	<0.0001	0.1338	204574.3493	<0.0001	99.8902
Typhimurium	0.5394	0.4478	0.631	0.0467	<0.0001	0.1467	138745.7752	<0.0001	99.9034
Paratyphi A	0.1004	0.0212	0.1797	0.0404	0.013	0.0561	41402.419	<0.0001	99.9461
Dublin	0.7314	0.5631	0.8997	0.0859	<0.0001	0.1198	13490.7417	<0.0001	99.9493
Choleraesuis	0.7824	0.6067	0.958	0.0896	<<0.0001	0.0851	9828.3596	<0.0001	99.5189
Paratyphi B	0.2145	0.0154	0.4135	0.1015	0.0347	0.1014	328.5713	<<0.0001	99.7429
Weltevreden	0.5827	0.3196	0.8457	0.1342	<<0.0001	0.1562	7016.8392	<0.0001	99.7831
Blegdam	0.8371	0.6348	1.0395	0.1032	<<0.0001	0.0606	61.6346	<0.0001	98.808
Agona	0.5719	0.249	0.8949	0.1648	0.0005	0.1595	3977.8948	<<0.0001	99.7819

Subgroup-analysis

Economic region	Studies	Estimate	Lower bound	Upper bound	Std. error	p-Val	z-Val	Q(df)	Het. p-Val	I^2 / %
Western	202	0.4655	0.4079	0.5231	0.0294	<0.0001	15.8446	1585078.8254(201)	<0.0001	99.99
Eastern	183	0.4868	0.4273	0.5464	0.0304	<0.0001	16.0233	223035.4651(182)	<0.0001	99.91
Central	103	0.4253	0.3456	0.505	0.0407	<0.0001	10.4545	278992.5615(102)	<0.0001	99.96
Northeastern	18	0.6377	0.4551	0.8203	0.0932	<0.0001	6.8441	4111.5723(17)	<0.0001	99.68
Overall	506	0.4711	0.435	0.5072	0.0184	<0.0001	25.5892	2116193.2205(505)	<0.0001	99.97
**Province**										
Sichuan	41	0.4642	0.3509	0.5774	0.0578	<0.0001	8.0347	103249.1049(40)	<0.0001	99.89
Zhejiang	35	0.3406	0.2107	0.4706	0.0663	<0.0001	5.1375	9997.6172(34)	<0.0001	99.88
Jiangsu	28	0.4671	0.3143	0.6198	0.0779	<0.0001	5.9923	17857.0051(27)	<0.0001	99.82
Guangdong	35	0.5199	0.3869	0.6528	0.0678	<0.0001	7.6643	35928.9019(34)	<0.0001	99.86
Hebei	15	0.6595	0.4572	0.8617	0.1032	<0.0001	6.3903	22230.2583(14)	<0.0001	99.91
Shanghai	6	0.125	-0.0166	0.2666	0.0722	0.0835	1.7308	32.4529(5)	<0.0001	95.5
Shandong	26	0.5294	0.3517	0.7071	0.0907	<0.0001	5.8398	38579.3998(25)	<0.0001	99.93
Henan	26	0.3403	0.1911	0.4894	0.0761	<0.0001	4.4717	106549.0308(25)	<0.0001	99.97
Anhui	16	0.5025	0.2751	0.73	0.116	<0.0001	4.331	14932.294(15)	<0.0001	99.89
Guangxi	38	0.5421	0.4034	0.6808	0.0708	<0.0001	7.6611	128952.2964(37)	<0.0001	99.97
Chongqing	13	0.2986	0.0961	0.501	0.1033	0.0038	2.8904	108756.9388(12)	<0.0001	100
Fujian	16	0.6377	0.454	0.8213	0.0937	<0.0001	6.8061	11686.1267(15)	<0.0001	99.73
Macau	1	0.5385	0.2675	0.8095	0.1383	NA	NA	NA	NA	NA
Ningxia	12	0.6788	0.4564	0.9013	0.1135	<0.0001	5.9816	746.6259(11)	<0.0001	99.49
Beijing	8	0.5903	0.3433	0.8373	0.126	<0.0001	4.6842	344.5848(7)	<0.0001	97.58
Hainan	2	0.8393	0.7033	0.9753	0.0694	<0.0001	12.0933	0.0009(1)	0.9766	0
Hunan	25	0.4955	0.3403	0.6507	0.0792	<0.0001	6.2575	121725.1257(24)	<0.0001	99.96
Jiangxi	11	0.3058	0.0515	0.5601	0.1298	0.0184	2.3566	1174.5496(10)	<0.0001	99.91
Tianjin	5	0.5464	0.106	0.9867	0.2247	0.015	2.432	10484.4283(4)	<0.0001	99.95
Yunnan	29	0.4636	0.3033	0.6239	0.0818	<0.0001	5.6669	277268.3465(28)	<0.0001	99.98
Liaoning	12	0.5779	0.3471	0.8087	0.1177	<0.0001	4.9082	3705.7422(11)	<0.0001	99.74
Gansu	11	0.5666	0.2945	0.8387	0.1388	<0.0001	4.0809	18563.4002(10)	<0.0001	99.94
Hubei	21	0.3981	0.2157	0.5805	0.0931	<0.0001	4.277	23574.7497(20)	<0.0001	99.87
Shaanxi	4	0.503	-0.0293	1.0352	0.2716	0.064	1.8521	757.2334(3)	<0.0001	99.54
Inner Mongolia	10	0.7641	0.6031	0.9251	0.0821	<0.0001	9.3031	421.4626(9)	<0.0001	99.73
Guizhou	27	0.2102	0.0732	0.3472	0.0699	0.0026	3.0065	137467.5137(26)	<0.0001	99.96
Shanxi	4	0.7217	0.4658	0.9777	0.1306	<0.0001	5.5261	53.4326(3)	<0.0001	92.08
Jilin	3	0.5445	0.0277	1.0614	0.2637	0.0389	2.0649	102.7671(2)	<0.0001	97.72
Xinjiang	10	0.4467	0.1894	0.7039	0.1312	0.0007	3.4034	28503.2837(9)	<0.0001	99.98
Heilongjiang	3	0.9856	0.9567	1.0144	0.0147	<0.0001	67.0577	0.4091(2)	0.815	8.8
Qinghai	5	0.405	0.0244	0.7855	0.1942	0.037	2.0857	48667.1037(4)	<0.0001	99.98
Tibet	2	0.4757	-0.4341	1.3855	0.4642	0.3055	1.0247	141.0243(1)	<0.0001	99.29
Taiwan	5	0.1106	-0.0228	0.244	0.0681	0.1041	1.6255	39.585(4)	<0.0001	98.62
Hong Kong	1	0.9286	0.7378	1.1194	0.0973	NA	NA	NA	NA	NA
Overall	506	0.4711	0.435	0.5072	0.0184	<0.0001	25.5892	2116193.2205(505)	<0.0001	99.97
**Setting**										
School	85	0.4017	0.3133	0.4901	0.0451	<0.0001	8.904	587912.0204(84)	<0.0001	99.99
Residential area	240	0.4684	0.4156	0.5212	0.027	<0.0001	17.3784	1163543.8686(239)	<0.0001	99.98
Nursing home	1	0.0208	-0.0363	0.078	0.0292	NA	NA	NA	NA	NA
Restaurant	85	0.6434	0.5684	0.7183	0.0383	<0.0001	16.8178	60629.6895(84)	<0.0001	99.73
Office	21	0.5838	0.4184	0.7493	0.0844	<0.0001	6.9156	24997.4141(20)	<0.0001	99.82
Factory	14	0.1533	0.0055	0.301	0.0754	0.0421	2.0329	12697.6046(13)	<0.0001	99.68
Mobile street stall	11	0.5681	0.3181	0.8181	0.1276	<0.0001	4.454	108996.4788(10)	<0.0001	99.98
Military region	12	0.3845	0.1236	0.6453	0.1331	0.0039	2.8883	8233.992(11)	<0.0001	99.84
Public sports venues	1	0.9875	0.9531	1.0219	0.0176	NA	NA	NA	NA	NA
Construction site	14	0.5787	0.364	0.7934	0.1095	<0.0001	5.2829	2080.741(13)	<0.0001	99
Hospital	19	0.1348	-0.0003	0.2698	0.0689	0.0504	1.9563	1600.716(18)	<0.0001	99.55
Prison	3	0.1083	-0.1073	0.3238	0.11	0.3249	0.9845	14.816(2)	0.0006	99.31
Overall	506	0.4711	0.435	0.5072	0.0184	<0.0001	25.5892	2116193.2205(505)	<0.0001	99.97

Sheet7: Incidence rate of abdominal cramps

Meta-analysis

Scale	Estimate	Lower bound	Upper bound	Std. error	p-Value	tau^2	Q(df=505)	Het. p-Value	I^2 / %
Overall	0.6851	0.6528	0.7174	0.0165	<0.0001	0.134	2222181.905	<0.0001	99.9544
FP	0.8091	0.7808	0.8373	0.0144	<0.0001	0.0703	715461.9857	<0.0001	99.854
EF	0.4476	0.3835	0.5116	0.0327	<0.0001	0.1764	689284.7387	<0.0001	99.9806
HAI	0.1951	0.0584	0.3319	0.0698	0.0052	0.1033	1634.8188	<0.0001	99.2674
**Top serovars**									
Typhi	0.4605	0.3849	0.5361	0.0386	<0.0001	0.1739	598257.003	<0.0001	99.9787
Enteritidis	0.8133	0.7663	0.8603	0.024	<0.0001	0.0604	21997.0931	<0.0001	99.6201
Typhimurium	0.675	0.5896	0.7603	0.0435	<0.0001	0.1272	38511.7172	<0.0001	99.7843
Paratyphi A	0.271	0.1459	0.3961	0.0638	<0.0001	0.1412	52759.8727	<0.0001	99.9701
Dublin	0.943	0.8977	0.9883	0.0231	<0.0001	0.0065	52.1652	<0.0001	99.0558
Choleraesuis	0.8564	0.7655	0.9473	0.0464	<<0.0001	0.0206	167.4673	<0.0001	96.4192
Paratyphi B	0.5345	0.2555	0.8135	0.1423	0.0002	0.2007	9628.2281	<<0.0001	99.8531
Weltevreden	0.9032	0.8113	0.9952	0.0469	<<0.0001	0.0164	66.7231	<0.0001	98.3664
Blegdam	0.8105	0.6236	0.9974	0.0953	<<0.0001	0.051	67.8967	<0.0001	97.1744
Agona	0.7363	0.4832	0.9894	0.1291	<<0.0001	0.0971	174.4446	<0.0001	99.5022

Subgroup-analysis

Economic region	Studies	Estimate	Lower bound	Upper bound	Std. error	p-Val	z-Val	Q(df)	Het. p-Val	I^2 / %
Western	202	0.6761	0.6234	0.7288	0.0269	<0.0001	25.1539	1635766.2919(201)	<0.0001	99.98
Eastern	183	0.7235	0.673	0.774	0.0258	<0.0001	28.0615	156063.0534(182)	<0.0001	99.77
Central	103	0.6228	0.5478	0.6978	0.0382	<0.0001	16.2837	339654.8054(102)	<0.0001	99.94
Northeastern	18	0.7557	0.6073	0.904	0.0757	<0.0001	9.9852	1145.3916(17)	<0.0001	99.27
Overall	506	0.6851	0.6528	0.7174	0.0165	<0.0001	41.573	2222181.9047(505)	<0.0001	99.95
**Province**										
Sichuan	41	0.7432	0.6488	0.8376	0.0482	<0.0001	15.4315	7604.0376(40)	<0.0001	99.73
Zhejiang	35	0.5916	0.4506	0.7326	0.0719	<0.0001	8.2254	40979.5399(34)	<0.0001	99.86
Jiangsu	28	0.6796	0.5301	0.8291	0.0763	<0.0001	8.9107	12210.8746(27)	<0.0001	99.68
Guangdong	35	0.7015	0.5903	0.8126	0.0567	<0.0001	12.3737	19485.8892(34)	<0.0001	99.59
Hebei	15	0.8191	0.6837	0.9544	0.069	<0.0001	11.862	2470.5905(14)	<0.0001	99.59
Shanghai	6	0.625	0.2832	0.9668	0.1744	0.0003	3.584	1165.1418(5)	<0.0001	99.2
Shandong	26	0.7788	0.6464	0.9112	0.0675	<0.0001	11.5298	36255.2863(25)	<0.0001	99.8
Henan	26	0.6025	0.456	0.749	0.0748	<0.0001	8.0591	77756.6838(25)	<0.0001	99.93
Anhui	16	0.6383	0.4393	0.8373	0.1015	<0.0001	6.2871	20088.0694(15)	<0.0001	99.85
Guangxi	38	0.7842	0.6764	0.892	0.055	<0.0001	14.2554	160611.8077(37)	<0.0001	99.94
Chongqing	13	0.5957	0.4022	0.7892	0.0987	<0.0001	6.0346	108809.574(12)	<0.0001	99.96
Fujian	16	0.8939	0.7936	0.9941	0.0511	<0.0001	17.4773	488.5829(15)	<0.0001	98.79
Macau	1	0.8462	0.65	1.0423	0.1001	NA	NA	NA	NA	NA
Ningxia	12	0.7529	0.5252	0.9807	0.1162	<0.0001	6.4794	4849.2905(11)	<0.0001	99.82
Beijing	8	0.7254	0.5767	0.874	0.0758	<0.0001	9.5632	106.8891(7)	<0.0001	92.12
Hainan	2	0.8761	0.7538	0.9983	0.0624	<0.0001	14.046	0.0431(1)	0.8355	0.09
Hunan	25	0.5845	0.4285	0.7405	0.0796	<0.0001	7.342	131418.1122(24)	<0.0001	99.95
Jiangxi	11	0.7515	0.5278	0.9751	0.1141	<0.0001	6.5846	1917.1448(10)	<0.0001	99.88
Tianjin	5	0.8946	0.7355	1.0538	0.0812	<0.0001	11.0179	58.4594(4)	<0.0001	98.8
Yunnan	29	0.7474	0.6269	0.8679	0.0615	<0.0001	12.1591	274759.5627(28)	<0.0001	99.96
Liaoning	12	0.782	0.6041	0.9599	0.0908	<0.0001	8.6162	1018.7567(11)	<0.0001	99.43
Gansu	11	0.7151	0.4724	0.9578	0.1238	<0.0001	5.7747	2902.0796(10)	<0.0001	99.9
Hubei	21	0.5845	0.4054	0.7636	0.0914	<0.0001	6.3963	17320.8811(20)	<0.0001	99.84
Shaanxi	4	0.6666	0.2384	1.0947	0.2184	0.0023	3.0514	190.9463(3)	<0.0001	98.67
Inner Mongolia	10	0.854	0.6644	1.0435	0.0967	<0.0001	8.8289	21691.6917(9)	<0.0001	99.84
Guizhou	27	0.3146	0.1583	0.471	0.0798	<0.0001	3.9447	129518.1729(26)	<0.0001	99.96
Shanxi	4	0.8084	0.6278	0.9889	0.0921	<0.0001	8.7763	21.4295(3)	<0.0001	85.02
Jilin	3	0.4935	-0.0024	0.9895	0.253	0.0511	1.9506	76.326(2)	<0.0001	97.3
Xinjiang	10	0.6391	0.4085	0.8697	0.1176	<0.0001	5.4322	36699.274(9)	<0.0001	99.93
Heilongjiang	3	0.8987	0.7666	1.0308	0.0674	<0.0001	13.3357	10.3413(2)	0.0057	76.62
Qinghai	5	0.5085	0.0729	0.944	0.2222	0.0221	2.2882	48754.1575(4)	<0.0001	99.99
Tibet	2	0.7231	0.2983	1.1479	0.2167	0.0008	3.3364	15.9185(1)	<0.0001	93.4
Taiwan	5	0.7992	0.6802	0.9182	0.0607	<0.0001	13.1612	76.0125(4)	<0.0001	91.83
Hong Kong	1	0.8333	0.5351	1.1315	0.1521	NA	NA	NA	NA	NA
Overall	506	0.6851	0.6528	0.7174	0.0165	<0.0001	41.573	2222181.9047(505)	<0.0001	99.95
**Setting**										
School	85	0.6344	0.554	0.7149	0.0411	<0.0001	15.4513	484332.9757(84)	<0.0001	99.97
Residential area	240	0.675	0.6264	0.7236	0.0248	<0.0001	27.2328	1308751.3373(239)	<0.0001	99.97
Nursing home	1	0.087	-0.0282	0.2021	0.0588	NA	NA	NA	NA	NA
Restaurant	85	0.8753	0.8365	0.914	0.0198	<0.0001	44.2713	24465.2144(84)	<0.0001	98.75
Office	21	0.8044	0.7252	0.8835	0.0404	<0.0001	19.918	408.6288(20)	<0.0001	96.29
Factory	14	0.6187	0.4098	0.8277	0.1066	<0.0001	5.8031	9197.7482(13)	<0.0001	99.75
Mobile street stall	11	0.8616	0.772	0.9512	0.0457	<0.0001	18.8444	248.6166(10)	<0.0001	97.34
Military region	12	0.5275	0.2725	0.7826	0.1301	<0.0001	4.0539	8613.1615(11)	<0.0001	99.8
Public sports venues	1	0.9875	0.9531	1.0219	0.0176	NA	NA	NA	NA	NA
Construction site	14	0.7538	0.5536	0.9539	0.1021	<0.0001	7.3824	1537.8323(13)	<0.0001	99.13
Hospital	19	0.1523	0.0174	0.2872	0.0688	0.0269	2.2129	1481.4504(18)	<0.0001	99.23
Prison	3	0.3476	0.0046	0.6907	0.175	0.047	1.9861	107.871(2)	<0.0001	97.13
Overall	506	0.6851	0.6528	0.7174	0.0165	<0.0001	41.573	2222181.9047(505)	<0.0001	99.95

Sheet8: Incidence rate of vomiting

Meta-analysis

Scale	Estimate	Lower bound	Upper bound	Std. error	p-Value	tau^2	Q(df=505)	Het. p-Value	I^2 / %
Overall	0.4591	0.4243	0.4939	0.0177	<0.0001	0.1552	1877574.795	<0.0001	99.9702
FP	0.5953	0.5576	0.633	0.0192	<0.0001	0.1264	905485.1399	<0.0001	99.9203
EF	0.1592	0.1126	0.2058	0.0238	<0.0001	0.0932	145747.5112	<0.0001	99.9765
HAI	0.3525	0.1991	0.506	0.0783	<0.0001	0.1288	3937.9951	<0.0001	99.4785
**Top serovars**									
Typhi	0.1344	0.0835	0.1854	0.026	<0.0001	0.0788	82378.9473	<0.0001	99.9739
Enteritidis	0.5862	0.5212	0.6512	0.0331	<0.0001	0.1171	46859.692	<0.0001	99.7785
Typhimurium	0.5779	0.4927	0.6631	0.0435	<0.0001	0.1253	133069.3188	<0.0001	99.8567
Paratyphi A	0.1176	0.0247	0.2104	0.0474	0.0131	0.0776	54097.953	<0.0001	99.9614
Dublin	0.6431	0.4771	0.8091	0.0847	<0.0001	0.1155	1327.4778	<0.0001	99.7379
Choleraesuis	0.661	0.447	0.875	0.1092	<<0.0001	0.1267	6052.655	<0.0001	99.6237
Paratyphi B	0.2253	0.0584	0.3923	0.0852	0.0082	0.0701	192.0872	<<0.0001	98.7016
Weltevreden	0.5338	0.3064	0.7613	0.1161	<<0.0001	0.113	6710.5666	<0.0001	99.6818
Blegdam	0.7047	0.4388	0.9705	0.1356	<<0.0001	0.1061	132.4426	<0.0001	98.8745
Agona	0.5778	0.2533	0.9023	0.1656	0.0005	0.1612	3976.6462	<<0.0001	99.7844

Subgroup-analysis

Economic region	Studies	Estimate	Lower bound	Upper bound	Std. error	p-Val	z-Val	Q(df)	Het. p-Val	I^2 / %
Western	202	0.4335	0.3775	0.4895	0.0286	<0.0001	15.1706	1355111.949(201)	<0.0001	99.98
Eastern	183	0.4853	0.429	0.5417	0.0288	<0.0001	16.8755	195214.675(182)	<0.0001	99.85
Central	103	0.4261	0.3489	0.5034	0.0394	<0.0001	10.815	264536.8959(102)	<0.0001	99.95
Northeastern	18	0.6723	0.5037	0.841	0.086	<0.0001	7.8131	2450.8117(17)	<0.0001	99.62
Overall	506	0.4591	0.4243	0.4939	0.0177	<0.0001	25.8721	1877574.7954(505)	<0.0001	99.97
**Province**										
Sichuan	41	0.4621	0.3504	0.5739	0.057	<0.0001	8.103	147094.6536(40)	<0.0001	99.91
Zhejiang	35	0.3227	0.1974	0.4481	0.064	<0.0001	5.0453	6394.1495(34)	<0.0001	99.87
Jiangsu	28	0.491	0.334	0.648	0.0801	<0.0001	6.1283	16847.2004(27)	<0.0001	99.69
Guangdong	35	0.546	0.4239	0.6681	0.0623	<0.0001	8.7626	30741.8598(34)	<0.0001	99.77
Hebei	15	0.598	0.3995	0.7965	0.1013	<0.0001	5.904	26966.8709(14)	<0.0001	99.88
Shanghai	6	0.2172	0.0236	0.4108	0.0988	0.0279	2.1984	44.6382(5)	<0.0001	96.82
Shandong	26	0.547	0.3882	0.7058	0.081	<0.0001	6.7511	34624.9886(25)	<0.0001	99.87
Henan	26	0.3755	0.2205	0.5306	0.0791	<0.0001	4.7464	89640.2434(25)	<0.0001	99.96
Anhui	16	0.5345	0.3148	0.7542	0.1121	<0.0001	4.7682	16187.8133(15)	<0.0001	99.9
Guangxi	38	0.4812	0.3448	0.6176	0.0696	<0.0001	6.9153	110475.8687(37)	<0.0001	99.97
Chongqing	13	0.3033	0.1104	0.4961	0.0984	0.0021	3.082	108755.4391(12)	<0.0001	100
Fujian	16	0.5304	0.3444	0.7164	0.0949	<0.0001	5.5894	11324.4579(15)	<0.0001	99.72
Macau	1	0.4615	0.1905	0.7325	0.1383	NA	NA	NA	NA	NA
Ningxia	12	0.5527	0.3141	0.7914	0.1218	<0.0001	4.5388	5124.74(11)	<0.0001	99.71
Beijing	8	0.5401	0.2772	0.803	0.1341	<0.0001	4.0271	860.8057(7)	<0.0001	98.55
Hainan	2	0.6388	0.3784	0.8991	0.1328	<0.0001	4.8094	1.3305(1)	0.2487	34.71
Hunan	25	0.4949	0.3397	0.6502	0.0792	<0.0001	6.25	120298.8013(24)	<0.0001	99.95
Jiangxi	11	0.3574	0.1032	0.6116	0.1297	0.0059	2.7556	1487.1993(10)	<0.0001	99.91
Tianjin	5	0.7706	0.5773	0.9639	0.0986	<0.0001	7.8124	147.6538(4)	<0.0001	97.48
Yunnan	29	0.4386	0.2835	0.5938	0.0792	<0.0001	5.5409	265823.25(28)	<0.0001	99.98
Liaoning	12	0.6552	0.449	0.8614	0.1052	<0.0001	6.2291	1997.0438(11)	<0.0001	99.67
Gansu	11	0.544	0.2643	0.8237	0.1427	<0.0001	3.8119	18682.2713(10)	<0.0001	99.94
Hubei	21	0.3203	0.1724	0.4683	0.0755	<0.0001	4.2444	19068.7057(20)	<0.0001	99.74
Shaanxi	4	0.8241	0.626	1.0222	0.1011	<0.0001	8.1523	17.7805(3)	0.0005	91.14
Inner Mongolia	10	0.6839	0.4788	0.8889	0.1046	<0.0001	6.5371	1403.0687(9)	<0.0001	99.84
Guizhou	27	0.1798	0.0539	0.3056	0.0642	0.0051	2.8	41506.2133(26)	<0.0001	99.93
Shanxi	4	0.6407	0.2992	0.9821	0.1742	0.0002	3.6777	118.6046(3)	<0.0001	96.03
Jilin	3	0.4379	-0.0448	0.9206	0.2463	0.0754	1.778	50.7631(2)	<0.0001	97.1
Xinjiang	10	0.4284	0.1573	0.6994	0.1383	0.002	3.0972	70981.8422(9)	<0.0001	99.98
Heilongjiang	3	0.9856	0.9567	1.0144	0.0147	<0.0001	67.0577	0.4091(2)	0.815	8.8
Qinghai	5	0.1545	-0.0484	0.3573	0.1035	0.1355	1.4926	35.2507(4)	<0.0001	99.92
Tibet	2	0.5765	-0.1364	1.2894	0.3637	0.113	1.5849	52.7882(1)	<0.0001	98.07
Taiwan	5	0.3063	0.0228	0.5898	0.1446	0.0342	2.1178	191.4513(4)	<0.0001	99.08
Hong Kong	1	0.0714	-0.1194	0.2622	0.0973	NA	NA	NA	NA	NA
Overall	506	0.4591	0.4243	0.4939	0.0177	<0.0001	25.8721	1877574.7954(505)	<0.0001	99.97
**Setting**										
School	85	0.3467	0.262	0.4314	0.0432	<0.0001	8.0225	565632.4001(84)	<0.0001	99.99
Residential area	240	0.4566	0.4049	0.5082	0.0263	<0.0001	17.3294	928638.6308(239)	<0.0001	99.97
Nursing home	1	0.9792	0.922	1.0363	0.0292	NA	NA	NA	NA	NA
Restaurant	85	0.6061	0.5356	0.6766	0.036	<0.0001	16.8423	32462.6432(84)	<0.0001	99.63
Office	21	0.5768	0.4155	0.738	0.0823	<0.0001	7.0106	18261.2293(20)	<0.0001	99.72
Factory	14	0.2351	0.0408	0.4294	0.0991	0.0177	2.3717	11182.9754(13)	<0.0001	99.76
Mobile street stall	11	0.6419	0.4318	0.8519	0.1072	<0.0001	5.9892	5897.0809(10)	<0.0001	99.64
Military region	12	0.4122	0.1556	0.6688	0.1309	0.0016	3.1486	8757.6406(11)	<0.0001	99.83
Public sports venues	1	0.0125	-0.0219	0.0469	0.0176	NA	NA	NA	NA	NA
Construction site	14	0.4899	0.2756	0.7041	0.1093	<0.0001	4.4819	1907.4403(13)	<0.0001	98.95
Hospital	19	0.3303	0.1685	0.4921	0.0826	<0.0001	4.0007	2960.8533(18)	<0.0001	99.5
Prison	3	0.1876	-0.0772	0.4524	0.1351	0.1649	1.3887	58.0521(2)	<0.0001	96.64
Overall	506	0.4591	0.4243	0.4939	0.0177	<0.0001	25.8721	1877574.7954(505)	<0.0001	99.97
