# Supplementary material for: Nationwide trends and features of human salmonellosis outbreaks in China
Source: Emerg Microbes Infect. 2024 Jun 26;13(1):2372364. doi: 10.1080/22221751.2024.2372364 (PMC11259058; doi:10.1080/22221751.2024.2372364)
Supplement: Supplemental Material [file TEMI_A_2372364_SM2423.pdf]

Table S6: List of Abbreviations (Arranged Alphabetically)			
**Abbreviation**	**Full name**	**Abbreviation**	**Full name**
AGs	Aminoglycosides	ML	Mean latitude
AMC	Ampicillin and clavulanic acid	MLs	Macrolides
AMK	Amikacin	MS	Mass spectrometry
AMP	Ampicillin	MTR	Metronidazole
AMX	Amoxicillin	MYS	Mean years of schooling
AR	Attack rate	n	Number
ATM	Aztreonam	NAL	Nalidixic acid
AZI	Azithromycin	NC	New case
BAC	Bacitracin	NE	Northeastern
BI	Biochemical identification	NEO	Neomycin
BLs	Beta-lactams	NET	Netilmicin
C	Central	NFs	Nitrofurans
CAZ	Ceftazidime	NIT	Nitrofurantoin
CEC	Cefaclor	NOR	Norfloxacin
CED	Cefradine	NOV	Novobiocin
CEP	Cefalotin	OA	Overall
CFP	Cefoperazone	OFX	Ofloxacin
CHL	Chloramphenicol	OXA	Oxacillin
CIP	Ciprofloxacin	OXY	Oxytetracycline
CLI	Clindamycin	P	Precipitation
CLO	Cloxacillin	PCR	Polymerase chain reaction
CMZ	Cefmetazole	PEN	Penicillin
CPs	Chloramphenicols	PFGE	Pulsed-field gel electrophoresis
CRO	Ceftriaxone	PIP	Piperacillin
CTF	Cefotiam	PIT	Phage infection test
CTT	Cefotetan	PMs	Polymyxins
CTX	Cefotaxime	POL-B	Polymyxin B
CXM	Cefuroxime	PPA	Pipemidic acid
CZO	Cefazolin	PPI	Proportion of primary industry
d	day	PSI	Proportion of secondary industry
DOX	Doxycycline	PTI	Proportion of tertiary industry
DSC	Designated symptom case	Re.	Remove
E	Eastern	QNs	Quinolones
EF	Enteric fever	RCL	Resident consumption level
ERY	Erythromycin	RH	Relative humidity
ETP	Ertapenem	RID	Cefaloridine
F	Female	RIF	Rifampicin
FEP	Cefepime	RNI	Rate of natural increase
FF	Florfenicol	SAM	Ampicillin and sulbactam sodium
FOX	Cefoxitin	SC	Selective culture
FP	Food poisoning	SDI	Sulfadiazine
FRZ	Furazolidone	SM	Streptomycin
GDP	Gross domestic product	SMZ	Sulfamethoxazole
GEN	Gentamicin	SNs	Sulfonamides
GR	Gender ratio	SO	*Salmonella* outbreak
HAI	Healthcare-associated infection	SOX	Sulfisoxazole
HB	Hysteresis bias	SPM	Acetylspiramycin
HDI	Human development index	SRB	Carbenicillin
IMP	Imipenem	SXT	Trimethoprim/sulphamethoxazole
IR	Incidence rate	T	Temperature
IT	Immunological test	TCC	Ticarcillin and clavulnic acid
JOS	Josamycin	TCs	Tetracyclines
KAN	Kanamycin	TET	Tetracycline
KIT	Kitasamycin	TGC	Tigecycline
LEX	Cephalexin	TLC	Ticarcillin
LIN	Lincomycin	TMP	Trimethoprim
LPR	Licensed physician ratio	TOB	Tobramycin
LSs	Lincosamides	TP	Total population
LVX	Levofloxacin	TVI	Total value of imports
M	Male	TZP	Piperacillin and tazobactam sodium
m	month	UR	Urbanization rate
ME	Microscopic examination	VAN	Vancomycin
MEZ	Mezlocillin	W	Western
MID	Midecamycin	WGS	Whole genome sequencing
MIN	Minocycline	y	year
